# Fexinidazole – A New Oral Nitroimidazole Drug Candidate Entering Clinical Development for the Treatment of Sleeping Sickness

**DOI:** 10.1371/journal.pntd.0000923

**Published:** 2010-12-21

**Authors:** Els Torreele, Bernadette Bourdin Trunz, David Tweats, Marcel Kaiser, Reto Brun, Guy Mazué, Michael A. Bray, Bernard Pécoul

**Affiliations:** 1 Drugs for Neglected Diseases *initiative* (DND*i*), Geneva, Switzerland; 2 The Medical School, University of Swansea, Swansea, United Kingdom; 3 Parasite Chemotherapy, Swiss Tropical and Public Health Institute, Basel, Switzerland; 4 University of Basel, Basel, Switzerland; Institute of Tropical Medicine, Belgium

## Abstract

**Background:**

Human African trypanosomiasis (HAT), also known as sleeping sickness, is a fatal parasitic disease caused by trypanosomes. Current treatment options for HAT are scarce, toxic, no longer effective, or very difficult to administer, in particular for the advanced, fatal stage of the disease (stage 2, chronic HAT). New safe, effective and easy-to-use treatments are urgently needed. Here it is shown that fexinidazole, a 2-substituted 5-nitroimidazole rediscovered by the Drugs for Neglected Diseases *initiative* (DND*i*) after extensive compound mining efforts of more than 700 new and existing nitroheterocycles, could be a short-course, safe and effective oral treatment curing both acute and chronic HAT and that could be implemented at the primary health care level. To complete the preclinical development and meet the regulatory requirements before initiating human trials, the anti-parasitic properties and the pharmacokinetic, metabolic and toxicological profile of fexinidazole have been assessed.

**Methods and Findings:**

Standard *in vitro* and *in vivo* anti-parasitic activity assays were conducted to assess drug efficacy in experimental models for HAT. In parallel, a full range of preclinical pharmacology and safety studies, as required by international regulatory guidelines before initiating human studies, have been conducted. Fexinidazole is moderately active *in vitro* against African trypanosomes (IC_50_ against laboratory strains and recent clinical isolates ranged between 0.16 and 0.93 µg/mL) and oral administration of fexinidazole at doses of 100 mg/kg/day for 4 days or 200 mg/kg/day for 5 days cured mice with acute and chronic infection respectively, the latter being a model for the advanced and fatal stage of the disease when parasites have disseminated into the brain. In laboratory animals, fexinidazole is well absorbed after oral administration and readily distributes throughout the body, including the brain. The absolute bioavailability of oral fexinidazole was 41% in mice, 30% in rats, and 10% in dogs. Furthermore, fexinidazole is rapidly metabolised *in vivo* to at least two biologically active metabolites (a sulfoxide and a sulfone derivative) that likely account for a significant portion of the therapeutic effect. Key pharmacokinetic parameter after oral absorption in mice for fexinidazole and its sulfoxide and sulfone metabolites are a C_max_ of 500, 14171 and 13651 ng/mL respectively, and an AUC_0–24_ of 424, 45031 and 96286 h.ng/mL respectively. Essentially similar PK profiles were observed in rats and dogs. Toxicology studies (including safety pharmacology and 4-weeks repeated-dose toxicokinetics in rat and dog) have shown that fexinidazole is well tolerated. The No Observed Adverse Event Levels in the 4-weeks repeated dose toxicity studies in rats and dogs was 200 mg/kg/day in both species, with no issues of concern identified for doses up to 800 mg/kg/day. While fexinidazole, like many nitroheterocycles, is mutagenic in the Ames test due to bacterial specific metabolism, it is not genotoxic to mammalian cells *in vitro* or *in vivo* as assessed in an *in vitro* micronucleus test on human lymphocytes, an *in vivo* mouse bone marrow micronucleus test, and an *ex vivo* unscheduled DNA synthesis test in rats.

**Conclusions:**

The results of the preclinical pharmacological and safety studies indicate that fexinidazole is a safe and effective oral drug candidate with no untoward effects that would preclude evaluation in man. The drug has entered first-in-human phase I studies in September 2009. Fexinidazole is the first new clinical drug candidate with the potential for treating advanced-stage sleeping sickness in thirty years.

## Author Summary

This article describes the preclinical profile of fexinidazole, a new drug candidate with the potential to become a novel, oral, safe and effective short-course treatment for curing both stage 1 and 2 human African trypanosomiasis and replace the old and highly problematic treatment modalities available today. Fexinidazole is orally available and rapidly metabolized in two metabolites having equivalent biological activity to the parent and contributing significantly to the *in vivo* efficacy in animal models of both stage 1 and 2 HAT. Animal toxicology studies indicate that fexinidazole has an excellent safety profile, with no particular issues identified. Fexinidazole is a 5-nitroimidazole and, whilst it is Ames-positive, it is devoid of any genetic toxicity in mammalian cells and therefore does not pose a genotoxic risk for use in man. Fexinidazole, which was rediscovered through a process of compound mining, is the first new drug candidate for stage 2 HAT having entered clinical trials in thirty years, and has the potential to revolutionize therapy of this fatal disease at a cost that is acceptable in the endemic regions.

## Introduction

A major challenge for new drug development is the identification of pharmacologically active compounds with a favourable activity and toxicity profile that can be turned into new drug candidates. The contemporary approach to identifying such compounds is high-throughput screening of large and chemically diverse compound libraries to identify novel pharmacophores, followed by lead optimisation [1], [2]. Sometimes, this screening effort is narrowed down by using more targeted libraries that are thought to be enriched in compounds with a desired type of activity (e.g. kinase inhibitors [3]). However, promising candidates can also be found by revisiting the wealth of past drug discovery research, during which promising lines of research were sometimes not pursued for commercial or other strategic reasons.

In this paper, we report the successful result of a proactive compound mining approach into a well-known class of anti-infectives, the nitroimidazoles, to rediscover fexinidazole, a long forgotten antiparasitic drug candidate. Fexinidazole turned out to be an excellent candidate to cure human African trypanosomiasis (HAT), including the advanced and fatal stage of the disease.

An estimated sixty million people in 36 sub-Saharan African countries are at risk for HAT, especially poor and neglected populations living in remote rural areas [4], [5]. While the number of reported HAT cases has decreased in recent years due to intensified control activities, 50,000 to 70,000 people are estimated to be infected [6]. In west and central Africa, *Trypanosoma brucei gambiense* causes a chronic form of sleeping sickness, whereas in eastern and southern Africa *T. b. rhodesiense* causes an acute form of the disease [7], [8]. Both forms of HAT occur in two stages: stage 1 (early, hemolymphatic) is characterized by non-specific clinical symptoms such as malaise, headache, fever, and peripheral oedema, whereas stage 2 (late, meningoencephalic) is characterized by neurological symptoms including behavioural changes, severe sleeping disturbances, and convulsions, which, if left untreated, lead to coma and death [9], [10].

Available treatments for HAT [8] (Table 1) are few, old, and limited due to toxicity, diminishing efficacy in several geographical regions [11], [12], and complexity of use [13]. Treatment is stage-specific, with the more toxic and difficult-to-use treatments being used for stage 2 HAT.

**Table 1:** Available treatment options for HAT.

Indication	Drug	Associated Problems
Stage 1	Pentamidine (1940)	7–10 daily intramuscular injections; only efficacious for stage 1
	Suramin (1920's)	Used primarily for stage 1 *T. b. rhodesiense* HAT
Stage 2	Melarsoprol (1949)	Ten painful daily intravenous injections; highly toxic, with ∼5 % treatment-related mortality. Increasing number of treatment failures (up to 30% in some regions); used for stage 2 HAT
	Eflornithine (1981)	Administration difficult – 4 slow intravenous infusions per day for 14 days; increasingly used as 1st line for *T. b. gambiense* stage 2 HAT
	NECT(2009)	Simplified regimen combining 7 days eflornithine (two infusions/day) and 10 days oral nifurtimox; expected to replace eflornithine monotherapy and melarsoprol for stage 2 *T. b. gambiense* HAT

NECT, a combination treatment of a simplified course of intravenous eflornithine and oral nifurtimox, has been the only advance in the past 25 years [14], [15], and has been recently accepted into the WHO's Essential Medicines List as treatment for stage 2 HAT [16]. Despite being a clear improvement with reduced toxicity and treatment duration, the requirement for intravenous administration is still a limitation.

It is estimated that less than 20% of currently infected people have access to treatment or are under any HAT surveillance, due to a combination of lack of effective and field-adapted diagnostics and treatments, combined with extreme poverty and remoteness of the affected populations, including in conflict zones [4], [17]. To change the dynamics of HAT control and access more patients while improving their case-management, a safe, effective, affordable, and easy-to-use (short course, preferably oral) treatment is urgently needed.

Nitroimidazoles are a well-known class of pharmacologically active compounds, among which several have shown good activity against trypanosomes [18]. The best-known anti-trypanosomal drug candidate in this class was megazol [19], [20]; its development was abandoned because of toxicity, in particular mutagenicity [21], [22], a known possibility in this chemical family [23], [24]. However, other members of this family including metronidazole [25], are widely used as antibiotics, indicating that it is possible to select compounds with an acceptable activity/toxicity profile in this class.

A systematic review and profiling of more than 700 nitroheterocyclic compounds (mostly nitroimidazoles) from diverse sources was undertaken and included an assessment of antiparasitic activity and mutagenic potential using state-of-the-art scientific methods. From these efforts, fexinidazole, a 2-substituted 5-nitroimidazole, was identified as a promising drug candidate for the treatment of HAT. Fexinidazole (1-methyl-2-((p-(methylthio)phenoxy)methyl)-5-nitroimidazole, CAS registry number 59729-37-2) had been in preclinical development in the 1970s and early 1980s as a broad-spectrum antimicrobial agent by Hoechst AG (now sanofi-aventis), selected within a broader series because of its wider range of action, lower toxicity and comparative ease of chemical synthesis [26], [27]. In 1983, the *in vivo* activity of fexinidazole against African trypanosomes was further substantiated [28]. However, fexinidazole's development was not pursued at the time.

This paper describes the trypanocidal efficacy and preclinical profile of fexinidazole as a novel clinical drug candidate for HAT, devoid of genotoxic risks for patients. The results show fexinidazole's potential to become a safe, efficacious, affordable, short-course (less than 14 days), oral treatment with a suitable shelf life in tropical conditions. Ideally the treatment will be safe and effective in both stages 1 and 2 HAT caused by *T. b. gambiense* and *T. b. rhodesiense*, allowing for significantly simplified diagnosis, treatment and patient-management and ultimately a better control of the disease.

## Methods

### Ethics Statement

All work was conducted in accredited laboratories and according to international guidelines. Specific references to the relevant authorities are provided below as appropriate. Where not stated, details of specific license holders can be obtained from the appropriate laboratories if required.

### Drug preparation

Fexinidazole and metabolites were prepared for *in vitro* studies as a stock solution in DMSO further diluted with water or 0.5% methylcellulose in water to appropriate concentration required for the assay.

For *in vivo* studies, fexinidazole was prepared as an optimized suspension comprising 5% w/v Tween 80/0.5% w/v Methocel in water, unless stated otherwise.

### Anti-trypanosomal activity studies

#### *In vitro* trypanocidal and cytotoxicity assays [29,30,31]

Parasites were cultured with or without the test article at a concentration of 3×10^4^/mL in Minimal Essential Medium, according to Baltz et al. [30], with the following modifications: 0.2 mM 2-mercaptoethanol, 1 mM Na-pyruvate, 0.5 mM hypoxanthine and 15% heat inactivated horse serum at 37°C in 5% CO_2_ in 96-well microtitre plates for 72 h. 10 µL of Alamar Blue was added for the final 3 h to determine viability. The assay was assessed by reading the fluorescence in each well at an excitation wavelength of 536 nm and at an emission wavelength of 588 nm. The IC_50_ values were calculated from the sigmoidal inhibition curves. For cytotoxicity, 4×10^4^/mL L-6 rat skeletal myoblast cells were used, and incubations and assessments carried out as above.

#### *In vivo* trypanocidal assays [32], [33]

Female NMRI mice were infected via intraperitoneal injection with bloodstream forms of *T. b. rhodesiense* STIB900 or *T. b. gambiense* 130R and treated with test drugs daily for 4 consecutive days, starting on day 3 or 7 after infection, respectively, or with bloodstream forms of *T. b brucei* GVR35 and treated daily from day 21 after infection for 5 days. Parasitemia was evaluated by tail blood examination and surviving, aparasitaemic mice at 60, 90, or 180 days, respectively, were considered cured.

The mouse assays were conducted in accordance to relevant national and international guidelines. The studies were approved by the local veterinary office (Kantonales Veterinäramt Basel) under licence No. 739.

### Pharmacokinetic (PK) studies

A range of pharmacokinetic studies have been performed in different species (mouse, rat, dog) in the context of this paper, either as pharmacokinetic studies to establish the PK profile of fexinidazole and its metabolites, or as part of other studies (safety pharmacology and toxicity) to demonstrate the systemic exposure of fexinidazole and its metabolites in the conditions of that particular study.

#### Plasma sampling

Blood samples were taken via into heparinised collection tubes and, following centrifugation, plasma was removed and stored frozen until required for assay.

#### Brain sampling in mice

Following sacrifice, the cranium of mice was opened and the brain removed. Excess blood was washed from the brain with distilled water and any excess fluid blotted on absorbent paper. The brain was then snap frozen using liquid nitrogen and placed into a suitably labelled container and stored at approximately −70°C, pending analysis. Brains were homogenized and extracted samples were prepared appropriately for analysis via liquid chromatography-mass spectrometry (LC-MS).

#### Plasma and brain pharmacokinetic (PK) analytical assessment

All PK evaluations were carried out using validated high performance liquid chromatography (HPLC) and LC-MS assays, as per standard operating procedures of the laboratories involved.

The majority of PK studies were carried out by Accelera, Nerviano Medical Sciences, Italy, following internal Standard Operating Procedures as non-GLP regulated studies, and all procedures for housing and handling of animals were in strict compliance with EEC and Italian Guidelines for Laboratory Animal Welfare.

One mouse PK and brain sampling study was carried out by BioDynamics Ltd., Rushden, UK, according to internal Standard Operating Procedures as a non-GLP regulated study, and animals were maintained as required by the “Code of practice for the housing and care of animals used in scientific procedures” (Home Office, London, 1989).

### ADME (Absorption, Distribution, Metabolism, Excretion) studies

#### *In vitro* hepatocyte metabolism [34]

Fexinidazole was incubated with cryopreserved hepatocytes from male CD-1 mice, male Sprague Dawley rats, male beagle dogs, male cynomolgus monkeys, and from pooled white or African-American human donor samples (purchased commercially), at concentrations of 1 µM and 10 µM. 1 µM samples were used for the cross-species intrinsic clearance determination, whereas 10 µM samples were used for the cross-species metabolite profile determination. The incubated samples were analyzed by LC-MS after extraction into acetonitrile.

#### *In vitro* CYP-450 metabolism [35]

Duplicate samples of human recombinant CYP enzyme isotypes CYP1A2, CYP2B6, CYP2C8, CYP2C9, CYP2C19, CYP2D6, CYP3A4, CYP3A5 (10–30 pmol/mL) with NADPH as cofactor were incubated with fexinidazole, fexinidazole sulfoxide, or fexinidazole sulfone at a concentration of 1.0 µM for up to 60 min (pH 7.4, 37°C). Detection of the compounds was via HPLC-MS.

#### *In vitro* transcellular permeability

Caco-2 [36] or MDR1-MDCK (Madin Darby canine kidney cells transfected with the human multidrug resistance MDR1 gene) [37] cell monolayers were grown to confluence on collagen-coated, microporous, polycarbonate membranes in 12-well microtitre plates. The buffer for the donor chamber was Hanks balanced salt solution with 10 mM HEPES and 15 mM glucose at pH 7.4. The buffer in the receiver chamber contained the same with the addition of 1% bovine serum albumin (BSA). The test article was added at 5 µM in the assay buffer. Cells were dosed on the apical side or baso-lateral side and incubated at 37°C with 5% CO_2_ in a humidified incubator. After 2 h, duplicate aliquots were taken from the donor and receiver chambers and assayed via LC/MS. The apparent permeability and recovery were calculated using standard methods.

#### *In vivo* metabolism and distribution

Whole-body autoradiography in rats was conducted using [^14^C]-radiolabelled fexinidazole. The site of the [^14^C] label in the fexinidazole molecule on the bridging carbon atom ensured that the radioactivity would remain associated with both the sulfoxide and sulfone metabolites. The radiolabelled compound was administered at the dose of 800 mg/kg (approximately 3.7 MBq/kg, 100 µCi/kg) by gastric gavage to eight male albino Sprague Dawley rats. Two animals were sacrificed at each time point 2, 8, 24 or 48 h after administration and the quantitative radioactivity distribution in the organs and tissues was evaluated using quantitative whole-body autoradiography.

Excretion balance in rats was carried out using a single oral dose of radiolabelled fexinidazole administered at the dose of 800 mg/kg (approximately 3.7 MBq/kg, 100 µCi/kg) by gastric gavage to three male albino Sprague Dawley rats.

The animal studies above were carried out by Accelera, Nerviano Medical Sciences, Italy, following internal Standard Operating Procedures as non-GLP regulated studies, and all procedures for housing and handling of animals were in strict compliance with EEC and Italian Guidelines for Laboratory Animal Welfare.

### Safety pharmacology profiling [38], [39]

#### *In vitro* cardiac safety [40]

HEK 293 (human embryonic kidney) cells stably expressing hERG (human ether-a-go-go-related gene) were treated with fexinidazole, fexinidazole sulfoxide, or fexinidazole sulfone at 1, 5 or 30 µM, and the hERG peak tail current was measured.

#### *In vivo* cardiovascular safety assessment [40]

Cardiovascular safety parameters were assessed in beagle dogs. Four adult (>1 year old) beagle dogs were given oral doses of fexinidazole at 100, 300 and 1000 mg/kg in an escalating dose design. During the experimental phase, animals were monitored via a Closed Circuit TeleVision (CCTV) system and physically inspected 24 h after dosing. On the day of dosing, a predefined set of telemetry signals from each animal were collected continuously from at least 1 h before dosing to at least 24 h after treatment.

#### *In vivo* neurobehavioural safety profiling

Neurobehavioural safety was carried out using the Irwin's test of general behaviour and body temperature in male rats [39], [41]. Twenty-four male rats (Crl:CD(SD)BR), aged about 7 weeks on the day of dosing, were divided into four experimental groups of 6 animals per group. Each group of animals was treated orally with fexinidazole at doses of 100, 300, or 1000 mg/kg, or vehicle. General behaviour was assessed before treatment and 2 h and 24 h after vehicle or drug administration. A defined set of behavioural observations using an arbitrary intensity scale of 0 (no behavioural change) to 8 was made before treatment and 2 h and 24 h after treatment. Factors present in normal animals (e.g. alertness, mobility) were scored as 4; potentiation or depression of these factors was indicated as higher or lower integers, respectively. Factors absent in normal animals were scored from 0 (normal) to 8.

#### *In vivo* respiratory safety profiling

Respiratory parameters were assessed in the rat [42]. Groups of eight male rats (Crl:CD(SD)BR), aged about 8 weeks on the day of dosing, were administered doses of 100, 300, or 1000 mg/kg of fexinidazole by oral gavage. A predetermined standard set of respiratory parameters was acquired continuously from individual animals housed in plethysmographic chambers. Basal values were calculated as the mean of values from 30 to 10 min before treatment. After treatment, values were extracted every 30 min up to 4 h. Body weights were recorded on the day of treatment for calculation of dose volumes and for calculation of tidal and minute volumes per kg of body weight.

All safety pharmacology studies in animals were carried out by Accelera, Nerviano Medical Sciences, Italy, following internal Standard Operating Procedures and applicable ICH guidelines (ICH S7A, S7B), were GLP regulated and were conducted in compliance with the DECRETO LEGISLATIVO 2 Marzo 2007, No. 50 and OECD Principles of GLP (January 1998) ENV/MC/CHEM (98) 17.

### Repeated–Dose Study/Toxicokinetics [43], [44]

In the rat repeated-dose toxicokinetic study, fexinidazole was administered orally by gavage once a day for 28 consecutive days to ten or fifteen Crl:CD (SD)IGS BR rats/sex/group at doses of 50, 200, or 800 mg/kg/day. A control group received the vehicle alone (5% Tween 80/0.5% methocel). Ten animals/sex/group were sacrificed at the end of the treatment period on day 29 or 30 of study. The remaining 5 animals/sex/group in the control and high-dose groups were sacrificed on day 43 at the end of a 2-weeks observation period. Systemic exposure to fexinidazole and its sulfoxide and sulfone metabolites was evaluated in three additional animals/sex/group. Samples were taken at predose, and 30 min, 1, 2, 4, 8 and 24 h after dosing on days 1, 14, and 28, and in addition at 48 and 72 h after treatment on day 28.

In the dog repeated-dose toxicokinetic study, fexinidazole was given orally by gavage once a day for 28 days to five (control and high dose) or three (low and mid dose) beagle dogs/sex/dose at the doses of 0 (control group), 50, 200, or 800 mg/kg/day. The control group received the vehicle alone (same as above). Systemic exposure to fexinidazole and of its sulfoxide and sulfone metabolites was evaluated on days 1, 14 and 28 in the same animals used for the toxicological study. Samples were taken at predose, and 30 min, 1, 2, 4, 8 and 24 h after dosing on days 1, 14, and 28, and in addition at 48 and 72 h after treatment on day 28.

For both species, the standard package of toxicological analyses was carried out.

Both rat and dog studies were carried out by Accelera, Nerviano Medical Sciences, Italy, according to their internal Standard Operating Procedures, were GLP regulated and were conducted in compliance with the DECRETO LEGISLATIVO 2 Marzo 2007, No. 50 and OECD Principles of GLP (January 1998) ENV/MC/CHEM (98) 17 and the ICH regulatory guidelines for repeated-dose toxicokinetics studies (ICH M3 and S3A).

### Developmental and reproductive toxicology [45]

Preliminary studies in rat and rabbit have been performed to have an early idea of reproductive and developmental toxicity risks, and to determine dose levels to be used in further pivotal studies.

In the rat study, fexinidazole was administered orally by gavage once a day from day 6 to day 17 of gestation or from day 6 of gestation to day 7 of lactation to 10 mated rat Crl:CD (SD)IGS BR rats/sex/group at doses of 50, 200, or 800 mg/kg/day. A control group received the vehicle alone (5% Tween 80/0.5% methocel).

In the rabbit study, fexinidazole was administered orally by gavage once a day from day 6 to day 20 of gestation to 6 inseminated New Zealand White Rabbit KBL females at the dose of 20, 40 and 80 mg/kg/day. A control group received the vehicle alone (5% Tween 80/0.5% methocel).

Both rat and rabbit studies were carried out by Accelera, Nerviano Medical Sciences, Italy, according to their internal Standard Operating Procedures, were GLP regulated and were conducted in compliance with the DECRETO LEGISLATIVO 2 Marzo 2007, No. 50 and OECD Principles of GLP (January 1998) ENV/MC/CHEM (98) 17 and the appropriate ICH regulatory guidelines for reproductive toxicology (ICH S5A).

### Genotoxicity assessments [46]

#### *In vitro* Ames test

To evaluate bacterial mutagenicity, a full Ames test was carried out on fexinidazole and fexinidazole sulfone using the five strains of *Salmonella typhimurium* recommended by the relevant ICH regulatory guideline, namely TA1535, TA1537, TA98, TA100, and TA102, as well as the corresponding nitroreductase-deficient strains [47], [48]. Standard bacterial plate incorporation assays were carried out, essentially as described by Maron and Ames [49]. Tests on fexinidazole were carried out with and without rat liver post-mitochondrial fraction plus co-factors (S9 mix) to provide a mammalian metabolic activation system. The S9 fraction was prepared from Spague-Dawley rats pretreated with the mixed cytochrome P 450 enzyme inducer Aroclor 1254. Tests on fexinidazole sulfone were carried out only in the absence of rat liver S9 (as this is a metabolite). After incubation at 37°C for three days, plates were scored for mutant colonies using a Colony Counter plate reader.

#### *In vitro* micronucleus test on human lymphocytes [50]

Duplicate human lymphocyte cultures were prepared from the pooled blood of two donors in two independent experiments. Treatments covering a broad range of concentrations, separated by narrow intervals, were done both in the absence and presence of metabolic activation (S9) from Aroclor-1254-induced animals. The highest concentration of fexinidazole was 220 µg/mL. Cells were treated with the drug either 24 h or 48 h after mitogen stimulation by phytohaemagglutinin (PHA). The test concentrations for micronucleus analysis were selected by evaluating the effect of fexinidazole on the replication index (RI). In each experiment, micronuclei were analysed at three concentrations. Similar tests were carried out on fexinidazole sulfone. As per the Ames test, this was carried out in the absence of rat liver S9 only.

#### *In vivo* mouse bone marrow micronucleus test [51]

Groups of six young adult male Crl:CD-1 (ICR) mice were treated with fexinidazole at two oral doses of 0, 500, 1000, or 2000 mg/kg given 24 h apart, and bone marrows were harvested 24 h after the second dose. Slides of bone marrow cells were prepared, Giemsa stained, and 2000 polychromatic erythrocytes per animal were scored for micronuclei.

#### *Ex vivo* unscheduled DNA synthesis (UDS) in rats [52]

Groups of young male Sprague-Dawley rats received fexinidazole at doses of 500, 1000, or 2000 mg/kg orally, and the livers were sampled either 2–4 h or 12–14 h after administration. Hepatocyte suspensions were prepared and incubated in the presence of titrated thymidine. Slides of fixed hepatocytes were coated in photographic emulsion and stored for 14 days at 4°C in the dark. The silver grains above the nuclei or cytoplasm (background) were counted, providing a measure of DNA uptake during DNA repair. The difference between these two counts indicated the extent of DNA repair (nuclear net grain count).

All genotoxicological studies in animals were carried out by Covance Laboratories Ltd, Harrowgate, UK, following internal Standard Operating Procedures, the applicable ICH guidelines (ICH S2) and in compliance with the UK GLP Regulations 1999, Statutory Instrument No. 3106 as amended by the GLP (Codification Amendments Etc.) Regulations 2004 and the OECD Principles on GLP (January 1998) ENV/MC/CHEM (98) 17. Animals were maintained as required by the “Code of practice for the housing and care of animals used in scientific procedures” (Home Office, London, 1989).

#### Redox potential measurement

One-electron reduction potentials were determined by pulse radiolysis following an established procedure [53].

Methods briefly described above are provided in full in the supplementary data sets listed in the results section below.

## Results

### Antiparasitic activity

**Figure 1:**
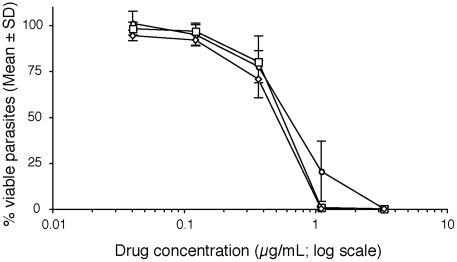
Effect of fexinidazole and its two main metabolites on *T. b. rhodesiense* (STIB 900). Parasite viability was measured *in vitro* after 72-h drug exposure. Fexinidazole - open circles (n = 11). Fexinidazole sulfoxide - open squares (n = 4). Fexinidazole sulfone - open diamonds (n = 4).

The antiparasitic activity of fexinidazole was assessed in experimental models of HAT. *In vitro* fexinidazole and its two main metabolites showed trypanocidal activity against the STIB900 laboratory strain of *T. b. rhodesiense* with very steep dose-response relations when assessed after 72 h of culture (Figure 1). With an IC_50_ of 0.48–0.82 µg/mL, fexinidazole's *in vitro* potency is weaker than that of the reference drug melarsoprol (IC_50_ = 0.003 µg/mL) and other trypanocidal drugs (Table 2) or the abandoned drug candidate megazol (IC_50_ = 0.02 µg/mL), although the two drugs currently used as first line to treat stage 2 HAT have a similarly modest *in vitro* potency (eflornithine: 0.9 µg/mL; nifurtimox: 0.4 µg/mL). Importantly, in contrast to melarsoprol and other drugs, fexinidazole has little or no non-specific cytotoxicity. Fexinidazole has a comparable IC_50_ of 0.16–0.36 µg/mL against a laboratory *T. b. gambiense* strain (STIB930) and against six recent *T. b. gambiense* clinical isolates (IC_50_ values from 0.30 to 0.93 µg/mL) (data not shown).

**Table 2:** *In vitro anti-parasitic* activity of fexinidazole, its metabolites, and reference compounds.

Compound	*T. b. rhodesiense*(STIB 900)	*T. b. gambiense*(STIB 930)	Cytotoxicity L-6 rat myoblast cells
	**IC_50_ in µg/mL (µM)** *		
Fexinidazole	0.48–0.82 (1.71–2.93)	0.16–0.36 (0.58–1.29)	>90 (>322)
Fexinidazole sulfoxide	0.41–0.49 (1.33–1.65)	0.18–0.36 (0.61–1.22)	>90 (>305)
Fexinidazole sulfone	0.35–0.40 (1.14–1.30)	0.16–0.39 (0.48–1.25)	>90 (>289)
***Reference molecules***			
Melarsoprol	0.002–0.004 (0.004–0.009)	0.0015–0.003 (0.004–0.006)	1.3 (3.3)
Megazol	0.02 (0.10)	Not available	57 (254)
Eflornithine	0.90 (3.80)	0.40 (1.67)	12 (51)
Nifurtimox	0.41 (1.44)	0.31 (1.08)	25 (87)
Pentamidine	0.003 (0.009)	0.002 (0.01)	3 (9)
Suramin	0.062 (0.046)	Not available	>90 (>70)

*IC_50_: concentration of drug required to kill 50% of the parasites or skeletal myoblast cells.

*In vivo*, fexinidazole is effective in curing both *T. b. rhodesiense* and *T. b. gambiense* acute models of infection at an oral dose of 100 mg/kg/day (or 50 mg/kg twice a day) for 4 days (Table 3A). Most significantly, in a *T. b. brucei* GVR35 infected mouse model of stage 2 HAT involving brain infection, fexinidazole given orally showed a dose-related increase in efficacy, with a dose of 200 mg/kg/day for 5 days being highly effective (Table 3B). In two other independent experiments, 100% cure was obtained in groups of 5 mice receiving an oral dose of 100 mg/kg, twice per day for 5 days (in these experiments, five daily intraperitoneal injections of 15 mg/kg melarsoprol also cured 100%). Of the drugs currently in clinical use (Table 1), only melarsoprol is effective in this experimental stage 2 HAT model.

**Table 3:** *In vivo* efficacy of fexinidazole experimental infection models for acute and chronic HAT.

	Model	Drug	Dose (mg/kg×No. treatment days)	Route*	Cured/infected	Mean relapse time (days)
**A**	*T. b. rhodesiense* STIB900(acute infection)	No treatment			0/4	7
		Fexinidazole	25×4	po	0/4	12
		Fexinidazole	50×4	po	1/4	>27
		Fexinidazole	100×4	po	4/4	>60
	*T. b. gambiense* 130R(acute infection)	No treatment			0/4	10
		Fexinidazole	100×4	po	3/3	>90
		Melarsoprol	4×4	ip	4/4	>90
**B**	*T. b. brucei*GVR35(chronic infection)	Diminazene+	40×1	ip	0/4	48.6
		Fexinidazole	50×5	po	0/8	41.3
		Fexinidazole	100×5	po	2/8	>82.1
		Fexinidazole	200×5	po	7/8	>163.8
		Melarsoprol	10×5	ip	2/8	>96.6
		Melarsoprol++	15×5	ip	4/5	>180

*ip: intraperitoneal; po: per os. Fexinidazole was formulated as a suspension in 5% Tween 80/95% methyl-cellulose (0.5% w/v in water) and administered via gastric gavage.

**+:** Diminazine diaceturate is used as a control as it is able to eliminate bloodstream parasitaemia but is not effective after CNS infection is established. Single dose on day 21 after infection.

**++:** Data included from a separate experiment for illustration only.

### ADME and pharmacokinetics (PK)

**Figure 2:**
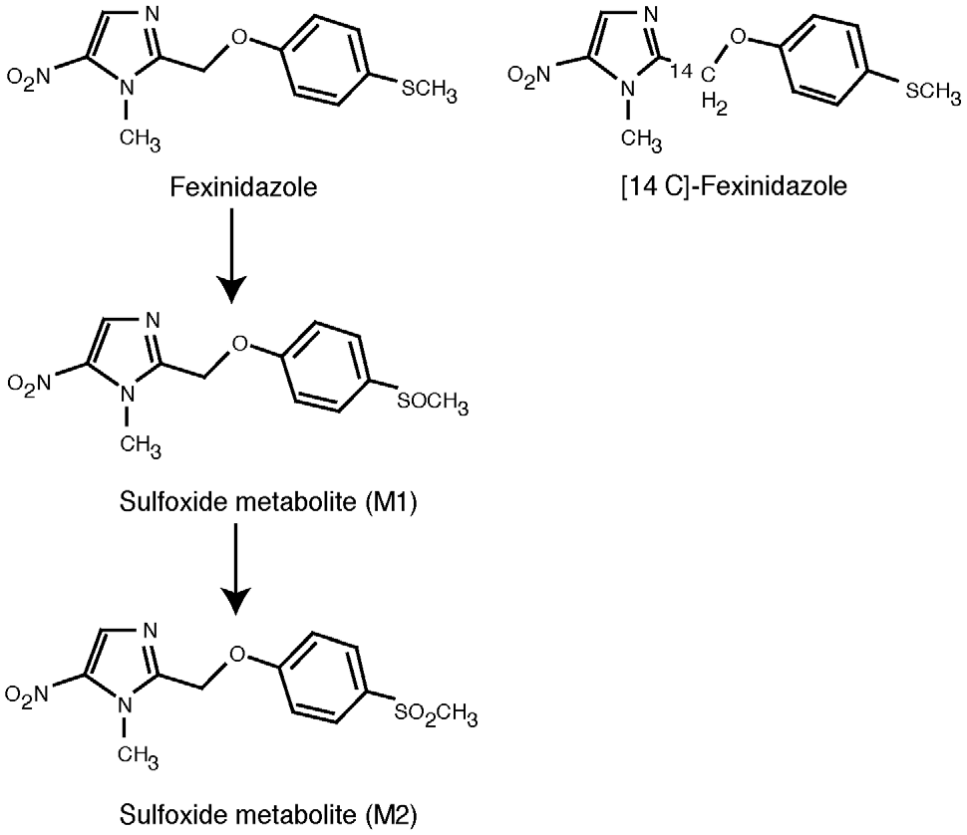
Chemical structure of fexinidazole and its main metabolites [**26**], including ^14^C-labeled fexinidazole indicating which carbon atom was labelled.

Fexinidazole is rapidly metabolised *in vivo*, with the main metabolites being the sulfoxide and sulfone derivatives (Figure 2) [26]. This principle metabolic conversion was confirmed *in vitro* using rat S9 fractions and hepatocytes from the mouse, rat, dog, monkey and human [Dataset S1]. In this comparative hepatocyte metabolism assay, fexinidazole was rapidly metabolised by all species, with *in vitro* intrinsic clearance rates highest in monkey (6500 mL/min/kg) > dog (5000 mL/min/kg) > mouse (4300 mL/min/kg) > rat (2900 mL/min/kg) > human (125 mL/min/kg). No meaningful differences were observed when comparing the *in vitro* metabolism of fexinidazole by hepatocytes from African-American or Caucasian donors [Dataset S2]. The main metabolic pathways of oxidation to the sulfoxide and sulfone derivatives were also confirmed to be the major route of metabolism *in vivo* in mice, rats and dogs (see below). As shown above, both metabolites have *in vitro* anti-trypanosomal activity similar to the parent compound (IC_50_ in µg/mL: 0.41–0.49 for the sulfoxide and 0.35–0.40 for the sulfone versus 0.48–0.82 for the parent compound) (Table 2).

The potential hepatic oxidative pathways involved in fexinidazole metabolism were assessed by testing the clearance of the fexinidazole and its two primary metabolites by a range of cytochrome P450 (CYP450) enzymes [Dataset S3]. The data show that fexinidazole is extensively metabolised by a range of CYP450 enzymes, including 1A2, 2B6, 2C19, 3A4, and 3A5 and, to a lesser extent, 2D6. The 2C8 and 2C9 enzymes were inactive. Interestingly, none of the enzymes tested metabolised either the sulfoxide or the sulfone to any significant degree (their metabolic pathways remain to be established). These data are in agreement with *in vivo* data showing the long systemic half-lives of the sulfoxide and sulfone metabolites in animal studies (see below). Since fexinidazole is metabolised extensively by multiple CYP450 isoforms, its metabolism is unlikely to be significantly affected by other drugs.

The oral absorption potential of fexinidazole was assessed in the well-known Caco-2 cell model for intestinal epithelial permeability [[36], Dataset S4]. In this assay, fexinidazole showed high absorption potential (apparent permeability P_app_ = 57.2 10^−6^ cm/s and no significant efflux). Intestinal permeability of fexinidazole is therefore not expected to be a limiting factor for absorption in humans.

The PK profile of fexinidazole was assessed in single-dose and multiple-dose studies in mice, rats and dogs. The absolute bioavailability of oral fexinidazole was 41% in mice, 30% in rats, and 10% in dogs. In all species tested, fexinidazole was rapidly and extensively metabolised to the sulfoxide and subsequently sulfone derivatives. Key pharmacokinetic parameters after oral absorption in mice for fexinidazole and its sulfoxide and sulfone metabolites are shown in Table 4 [Dataset S5]. Essentially similar PK profiles were observed in rats and dogs [Datasets S6, S7], even if the exact values varied among species (see also below).

**Table 4:** Mouse pharmacokinetics of fexinidazole and its metabolites in plasma and brain after oral administration.

	C_max_(ng/mL)	T_max_(h)	T_1/2_(h)	AUC_0–24_ (ng.h/mL)
Fexinidazole	500	0.25	0.8	424
Sulfoxide metabolite	14171	0.5	1.0	45031
Sulfone metabolite	13651	4.0	1.7	96286

Fexinidazole was formulated as a suspension in 1% DMSO/99% methyl-cellulose (1% w/v in water) and administered to female NMRI mice (n = 3) via gastric gavage at a concentration of 25 mg/kg.

C_max_: maximum plasma concentration. T_max_: time of maximum plasma concentration. T_1/2_: terminal elimination half life. AUC_0–24_: area under curve from time of dosing to the last measurable concentration.

The ability to cross the blood-brain barrier is crucial for drugs intended to treat stage 2 HAT. The ability of fexinidazole to do so was initially assessed *in vitro* in a MDR1-MDCK model [37, Dataset S8]. Fexinidazole showed high predicted brain permeation (apparent permeability P_app_ = 60.6 10^−6^ cm/s and no significant efflux). In mice, the presence of fexinidazole and both metabolites in the brain was confirmed after oral dosing (Table 5, Dataset S5), and is consistent with the data showing efficacy in the murine model of chronic HAT (Table 3B).

**Table 5:** Presence of fexinidazole and metabolites in the brain after oral administration of fexinidazole to mice.

Time point(min)	Fexinidazole(ng/g)	Sulfoxide(ng/g)	Sulfone(ng/g)
15	1136±54.1	ND	ND
30	800±92.6	3315±1611	469±222
60	763±90.7	4873±2335	1183±322

Data are expressed as mean ± SD (n = 3). ND: not determined.

Fexinidazole was formulated as a suspension in 1% DMSO/99% methyl-cellulose (1% w/v in water) and administered to female NMRI mice via gastric gavage at a concentration of 25 mg/kg.

**Figure 3:**
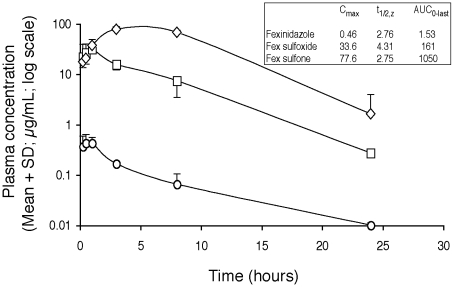
Plasma concentrations of fexinidazole and its two main metabolites after 5 days of oral administration. 200 mg/kg fexinidazole was administered to mice (n = 3). Fexinidazole - open circles. Fexinidazole sulfoxide - open squares. Fexinidazole sulfone - open diamonds.

The PK profile of fexinidazole was further characterised in mice that were administered the same treatment schedule that was curative in the chronic disease model (Table 3B). The plasma profile in mice of fexinidazole and its sulfoxide and sulfone metabolites after 5 days of fexinidazole treatment at the effective dose (200 mg/kg/day) is illustrated in Figure 3. The data show that a high and prolonged systemic bioavailability of biologically active compounds is achieved a few hours after drug administration, seemingly without drug accumulation and associated potential toxicity [Dataset S9].

This pattern of parent and metabolite plasma profiles without significant drug accumulation is further illustrated in Tables 6 and 7, which show plasma PK parameters in Sprague-Dawley rats and beagle dogs after 1 and 14 days of daily oral dosing with fexinidazole (data taken from the 28-day toxicokinetics studies, see below). In both species, it is interesting to note that there is no apparent accumulation in the plasma of either parent drug or metabolites, irrespective of dose, at least during the treatment period of 1–14 days. In the dog, and to some extent in the rat, the only difference seen between the data from day 1 versus day 14 is that the T_max_ for the sulfone metabolite occurs some hours earlier on day 14 compared to day 1, although the overall amount of the metabolite in plasma is similar on both days.

**Table 6:** Rat plasma pharmacokinetic parameters for fexinidazole and its metabolites after oral administration of fexinidazole.

Dose (mg/kg/day)(no. animals)	Sampleday	C_max_(μg/mL)	T_max_(h)	AUC_0-t(last)_(μg⋅h/mL)
**Plasma Fexinidazole**				
50 (6)	1	0.09±0.07	1.42±1.28	0.47±0.34
	14	0.18±0.14	1.92±1.20	0.83±0.54
200 (6)	1	0.38±0.28	1.92±1.20	2.16±1.34
	14	0.52±0.30	2.08±1.11	3.02±1.47
800 (6)	1	1.48±0.71	1.58±0.66	12.8±4.10
	14	1.02±0.87	3.17±2.84	9.29±5.20
**Plasma Fexinidazole sulfoxide**				
50 (6)	1	2.70±1.40	2.50±1.22	15.4±9.28
	14	5.23±3.28	2.17±0.98	29.8±19.4
200 (6)	1	11.4±2.17	2.33±0.82	85.9±14.1
	14	15.8±3.17	3.33±1.03	118±38.9
800 (6)	1	31.7±3.74	4.67±1.63	410±101
	14	25.0±8.99	2.67±1.03	277±160
**Plasma Fexinidazole sulfone**				
50 (6)	1	2.92±2.26	7.33±1.63	36.1±28.6
	14	6.38±2.30	6.00±2.19	89.6±34.1
200 (6)	1	9.29±1.75	8.00±0.00	126±17.5
	14	20.2±2.42	7.33±1.63	287±36.5
800 (6)	1	42.6±13.1	10.7±6.53	574±256
	14	40.5±13.0	6.67±2.07	543±252

Data are expressed as mean ± SD.

C_max_: maximum plasma drug concentration achieved. T_max_: time to reach C_max_. AUC_0-t(last)_: area under the plasma concentration time curve from initial to final data point

**Table 7:** Dog plasma pharmacokinetic parameters for fexinidazole and its metabolites after oral administration of fexinidazole.

Dose (mg/kg/day) (no. animals)	Sampleday	C_max_(μg/mL)	T_max_(h)	AUC_0-t(last)_ (μg⋅h/mL)
**Plasma Fexinidazole**				
50 (6)	1	0.04±0.01	0.75±0.61	0.19±0.13
	14	0.03±0.01	1.08±0.74	0.15±0.13
200 (6)	1	0.07±0.03	1.00±0.55	0.44±0.09
	14	0.08±0.01	1.50±0.77	0.45±0.14
800 (10)	1	0.14±0.07	1.15±0.47	0.84±0.32
	14	0.14±0.05	1.20±0.42	1.05±0.30
**Plasma Fexinidazole sulfoxide**				
50 (6)	1	3.76±0.97	1.17±0.41	19.6±5.68
	14	2.99±0.98	1.33±0.52	13.6±5.99
200 (6)	1	8.12±2.66	1.25±0.61	51.4±14.1
	14	8.99±2.83	2.00±0.00	56.7±16.6
800 (10)	1	14.5±3.95	1.55±0.60	112±31.8
	14	13.6±4.09	1.60±0.97	129±39.8
**Plasma Fexinidazole sulfone**				
50 (6)	1	8.58±2.15	7.33±1.63	146±38.3
	14	7.78±2.89	5.33±2.07	126±59.1
200 (6)	1	17.6±2.46	12.7±8.91	348±63.9
	14	21.8±3.86	5.33±2.07	384±49.9
800 (10)	1	36.1±7.94	14.4±8.26	660±164
	14	36.6±6.24	6.80±1.93	653±118

Data are expressed as mean ± SD.

C_max_: maximum plasma drug concentration achieved. T_max_: time to reach C_max_. AUC_0-t(last)_: area under the plasma concentration time curve from initial to final data point.

Whole-body autoradiography in rats using [^14^C]-radiolabelled fexinidazole (see figure 2 for labelling site) showed that the parent drug and/or its metabolites are broadly distributed to all organs and tissues (the assay did not distinguish between fexinidazole and its metabolites), with peak concentrations in most tissues 2 h after oral dosing. After 48 h, most radioactivity was eliminated from the body and no tissue specific accumulation was noted [Dataset S10]. Furthermore, radioactivity was detected at all times in the brain, with a brain-to-blood concentration ratio of 0.4–0.6.

Excretion balance studies in rats showed that 30% and 59% of fexinidazole-related material was excreted via urine and faeces, respectively, within 96 h [Dataset S11]. Elimination of the radioactivity after oral dosing was rapid, with 84% eliminated within 48 h. About 1.4% of the dose was recovered from the carcass with an overall recovery of the total radioactivity of approximately 93%.

### Safety pharmacology

In regulatory safety pharmacology assessments, *in vitro* exposure of hERG-transfected HEK 293 cells to fexinidazole sulfone, but not fexinidazole or the sulfoxide, showed a statistically significant decrease of 33% on hERG peak tail current at the highest of the three doses tested (30 µM, 9.34 µg/mL; no effect at 1 or 5 µM)[Dataset S12]. However, assessment of cardiovascular parameters in beagle dogs after single oral doses up to 1000 mg/kg showed no meaningful effects on blood pressure, heart rate, and ECG intervals, including the Q-T interval [Dataset S13]. Similarly, no meaningful effects were observed after single oral doses in rats of up to 1000 mg/kg on general behaviour and body temperature (modified Irwin's test) or on respiratory parameters [Dataset S14, S15].

### Repeated-dose toxicity and reproductive toxicity

Because fexinidazole treatment for HAT is expected to be a single regimen of 14 days or less, 28-day regulatory toxicokinetic studies were carried out in rats and dogs.

Once daily oral fexinidazole doses of 50, 200 and 800 mg/kg/day were well tolerated in rats at all doses tested [Dataset S16]. Only a minimal-to-slight decrease in food consumption and in the expected body weight increases (due to normal growth) was observed at 200 and 800 mg/kg, in male animals only. Minimal-to-moderate changes were observed in the liver of all fexinidazole-treated animals (increased liver weight and/or hypertrophy of the centrilobular hepatocytes). However, there was no increase in liver enzymes including AST and ALT, and all other clinical pathology parameters were also normal. Taken together with the observation that these changes were restricted to the dosing period, these were considered of adaptive origin (metabolism) and not indicative for liver toxicity. The No Observed Adverse Event Level (NOAEL) in the rat was set at 200 mg/kg/day.

In Beagle dogs, daily oral fexinidazole doses of 50, 200 and 800 mg/kg/day were also well tolerated [Dataset S17]. Slight-to-moderate body weight loss and reduction in food intake were observed at 800 mg/kg/day during treatment. A minimal-to-slight decrease in the number of lymphocytes was seen at the highest dose. The No Observed Adverse Event Level (NOAEL) in the dog was also set at 200 mg/kg/day.

In both rat and dog studies, plasma levels of fexinidazole and both metabolites were measured and showed that fexinidazole was adequately absorbed, resulting in a significant and prolonged exposure of especially fexinidazole sulfoxide and sulfone (data up to 14 days of treatment in rats and dogs is shown in Tables 6 and 7).

Preliminary studies on the potential effects of fexinidazole on embryo-foetal and early postnatal development were carried out in pregnant rats and no adverse effects on embryos/foetuses, parturition, and neonates were identified in dams. Further standard development and reproductive toxicology (DART) studies are currently ongoing to confirm and extend the preliminary results.

### Genotoxicity

**Figure 4:**
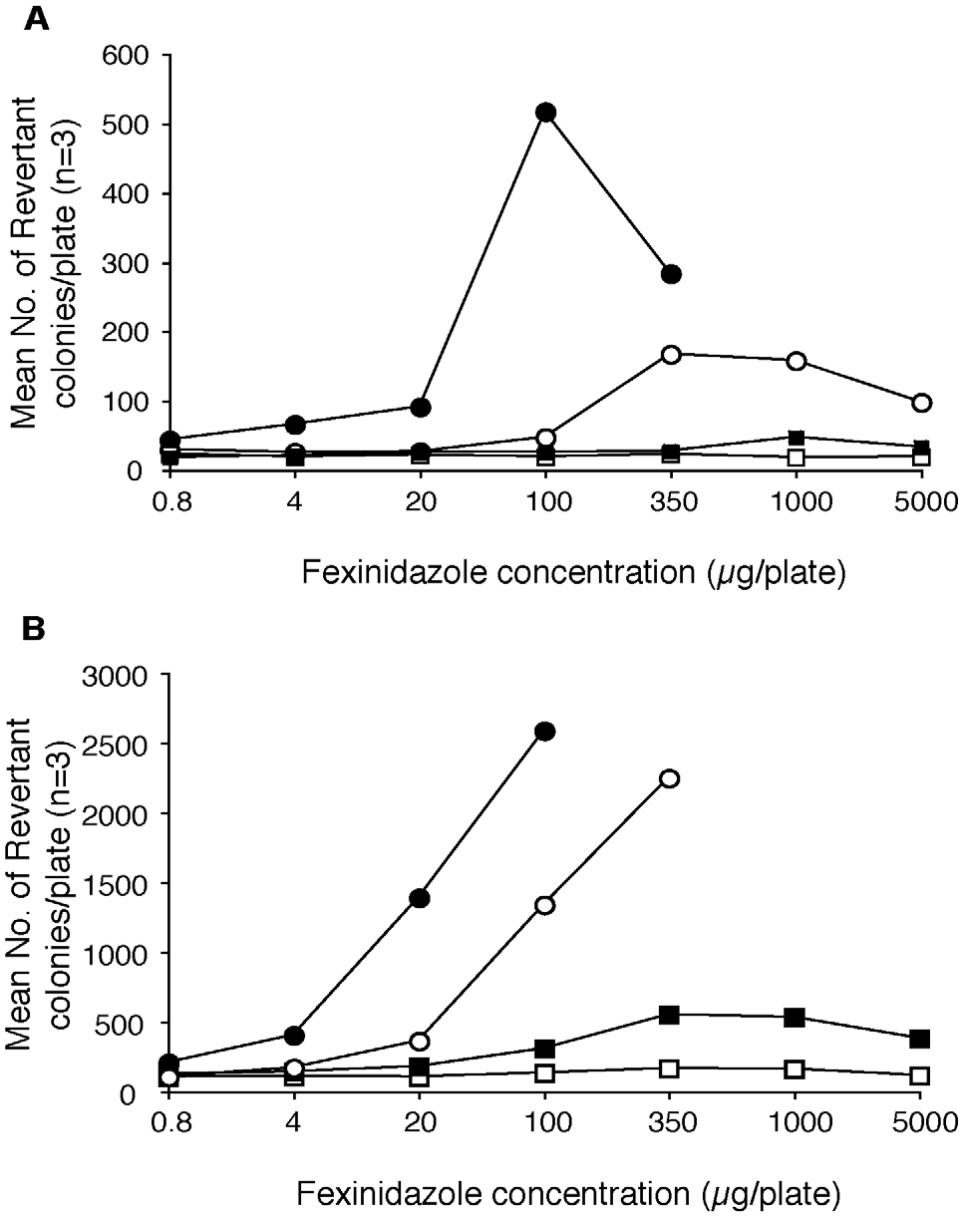
Mutagenic activity of fexinidazole in the Ames test. *Salmonella typhymurium* strains TA98 (A) and TA100 (B) and their nitroreductase-deficient variants TA98NR and TA100NR were used, in the presence and absence of metabolic activation (+/− S9). **A**: Solid circles: TA98 +S9; Open circles: TA98 -S9; Solid squares: TA98NR +S9; Open squares: TA98NR -S9; Negative control: Mean number of revertants per plate were TA98 (−S9): 21; TA98 (+S9): 34; TA98NR (−S9): 29; TA98 (+S9): 18. **B**: Solid circles: TA100 +S9; Open circles: TA100 −S9; Solid squares: TA100NR +S9; Open squares: TA100NR −S9; Negative control: Mean number of revertants per plate were TA100 (−S9): 104; TA100 (+S9): 116; TA100NR (−S9): 90; TA100NR (+S9): 111.

Fexinidazole and its primary metabolites are nitroimidazoles and, like many other nitroheterocyclic compounds, are potentially mutagenic [48]. To evaluate bacterial mutagenicity, a standard full Ames test was carried out on four strains of *Salmonella typhimurium*, with and without rat liver microsomes [Dataset S18]. Fexinidazole elicited both frameshift and base substitution mutations. However, this activity was significantly reduced or abolished when nitroreductase-deficient *Salmonella* strains were used for the assay (representative example shown in Figure 4). Rat liver microsomes metabolise fexinidazole efficiently to the sulfoxide metabolite under these experimental conditions (data not shown), so mutagenicity of this metabolite is covered by the above data. A separate Ames test of the sulfone metabolite gave similar results to fexinidazole (data not shown). These data suggest that the observed mutagenic activity is due to bacterial activation of fexinidazole and its metabolites by nitroreductases, and is not an inherent property of the compounds. A detailed analysis of fexinidazole's genotoxic potential on mammalian systems was undertaken subsequently. First, genotoxicity in mammalian cells was evaluated in an *in vitro* micronucleus test using human peripheral lymphocytes [Dataset S19]. Fexinidazole did not induce the formation of micronuclei, and thus no clastogenic damage, either in the presence or absence of rat liver microsomal enzymes (Table 8A). A separate *in vitro* micronucleus assay of the sulfone metabolite was also negative (data not shown). An *in vivo* bone-marrow micronucleus test in mice administered high oral doses of fexinidazole (up to 2 g/kg) confirmed the lack of clastogenicity (Table 8B, Dataset S20), while plasma analysis of these mice confirmed the exposure to fexinidazole and its two major metabolites (data not shown). Finally, an *ex vivo* rat liver unscheduled DNA synthesis study (Table 8C) confirmed the lack of mammalian genotoxic activity for fexinidazole and its metabolites [Dataset S21]. Taken together, these data support the conclusion that fexinidazole does not pose a genotoxic risk to patients.

**Table 8:** Mutagenicity assessments of fexinidazole on mammalian cells.

A. *In vitro* micronucleus assay on human lymphocytes				
Micronucleated binucleate cells (%)*				
	24-h PHA		48-h PHA	
	−S9	+S9	−S9	+S9
Fexinidazole dose (μg/mL)				
**0**	0.75	0.40	1.1	0.90
**20**	0.50	0.50	1.1	0.60
**40**	0.40	0.55	0.60	1.20
**80**	0.70	0.75	0.90	0.40
**4-nitroquinoline N-oxide, 5.0 µg/mL	10.80		8.95	
**Cyclophosphamide, 6.25 µg/mL		3.60		12.85

B. *In vivo* bone marrow micronucleus assay		
	Micronucleated PCE/1000	PCE/NCE
Fexinidazole dose (mg/kg)		
**0**	1.0	0.75
**500**	1.4	0.82
**1000**	1.1	0.75
**2000**	1.8	0.89
**Cyclophosphamide, 4.0 mg/kg,	19.3	0.89

C *Ex vivo* unscheduled DNA synthesis assay on rat liver cells		
	Net nuclear grain count (2–4 h)	Net nuclear grain count (12–14 h)
Fexinidazole dose (mg/kg)		
**0**	0.2	0.6
**500**	0.0	0.4
**1000**	−0.1	0.5
**2000**	−0.4	0.2
**dimethylnitrosamine, 10 mg/kg	8.9	
**2-acetamidofluorene, 75 mg/kg		9.8

*Relative Replication Index (RI: relative number of nuclei compared to controls) expressed as a %.

**positive control.

PCE: poly-chromatic erythrocyte. NCE: normo-chromatic erythrocyte. PHA: phytohemagglutinin.

No direct studies have been done on the mode of action of fexinidazole. However, fexinidazole might act as a prodrug like other 5-nitroimidazoles that are toxic to the parasites only after bioreductive activation [54]. From studies of trypanosomes resistant to the action of nitroimidazoles, it appears that these parasites have bacterial-like nitroreductases, which can activate nitroimidazole drugs into reactive intermediates that in turn cause cellular damage [55]. Fexinidazole and the sulfoxide and sulfone metabolites were shown to have a low single electron redox potentials being −511 mV, −493 mV, and −488 mV, respectively. In the same study, the single electron redox potential of metronidazole was −516 mV, and of megazole was −422 mV.

## Discussion

This paper provides data showing that fexinidazole, a 2-substituted 5-nitroimidazole identified among a series of existing but long forgotten compounds, is a promising drug candidate for HAT. A full set of preclinical studies have been conducted in accordance with the regulatory requirements for pharmaceuticals for human use, and fexinidazole has now successfully entered phase I clinical trials. Fexinidazole is the first new drug candidate in 30 years that is in clinical development for the advanced and fatal stage of the disease (stage 2). In addition, being an oral drug with the potential to be effective against both stage 1 and stage 2 HAT caused by *T. b. gambiense* and *T. b. rhodesiense*, it could become the much needed breakthrough for HAT control by drastically simplifying case management.

Fexinidazole has been shown to be selectively trypanocidal *in vitro* on *T. b. rhodesiense* and *T. b. gambiense* parasites, both on established laboratory strains and recent clinical isolates. Whilst *in vitro* potency is modest, with IC_50_ values between 0.1 and 0.8 µg/mL, a short course (4 or 5 days) of oral fexinidazole treatment is curative in experimental mouse models of acute and chronic (stage 2) HAT at doses of 100–200 mg/kg/day. This would correspond to a daily human equivalent dose (HED) for adults of 16 mg/kg calculated based on body surface area [56]; however, more detailed mouse pharmacodynamics studies are required together with human PK data to be able to propose an effective therapeutic dose, including duration. The experimental curative capacity of fexinidazole is significant, as among the currently used drugs in the clinic, only the highly toxic drug melarsoprol is curative in the chronic mouse model which involves an established brain infection that mimics stage 2 HAT. The observation that a single high dose of fexinidazole was also partially curative in the acute model (data not shown) underscores the potential for a short course treatment which will be crucial to achieve an easy-to-use treatment for remote and rural areas. While the predictive value of these murine models in terms of the potential for curing stage 2 patients is not fully established (only melarsoprol cures both), the demonstration that a drug candidate can clear systemic trypanosome infections in both the acute and chronic model, as well as clearing the brain infection (no relapse in the chronic model), is widely considered as *the* critical feature for a stage 2 HAT drug candidate.

It has been argued by some that obtaining data from other animal models (rat, monkey) before moving into clinical development is desirable. However, the urgency to find new drugs for HAT combined with the lack of clinical candidates in the pipeline warrants a bolder strategy. Moreover, as fexinidazole's *in vivo* efficacy is likely to depend on the combined exposure profile of the parent drug and its two major metabolites, and knowing that metabolism can vary between species, it is uncertain what can be learned from additional animal disease models. Clearly, the critical studies ahead to determine the curative potential of fexinidazole in humans will be the human safety and PK studies in phase I, and subsequently a proof-of-concept phase II study in patients.

Upon oral administration, fexinidazole is well absorbed and rapidly metabolised into the sulfoxide and sulfone derivatives, both of which have similar *in vitro* trypanocidal activity to the parent compound. The excellent *in vivo* activity of fexinidazole when administered orally is likely to be due to the cumulative exposure to not one but three active compounds which distribute throughout the body with different but overlapping kinetics, thus ensuring effective exposure in both the systemic circulation and the brain. In mice, rats and dogs, the half-life of fexinidazole after oral treatment ranges from 1 h to 3 h, whilst the half-life of the sulfoxide ranges from 2 h to 7 h and that of the sulfone can be up to 24 h after dosing. As the *in vitro* intrinsic clearance rate by human hepatocytes was lower than that of all other species tested, it can be expected that the half-lives in humans will be even longer, which further supports fexinidazole's potential for a once per day short-duration treatment schedule. On the other hand, a non-linear dose-related absorption and consequent exposure was observed in both rats and dogs (not done in mice). It will thus be important to carefully analyse the dose-related PK of fexinidazole and both metabolites after oral dosing in humans to better predict the dose-response relationship.

While fexinidazole and the sulfoxide are metabolised by multiple liver microsomal enzymes, suggesting a low risk for drug-drug interactions, the metabolic route of the sulfone remains to be established. No accumulation of either fexinidazole or the primary metabolites was found in rats, and almost all drug-related material was eliminated from the body within 48 h of oral dosing, excreted mainly through faeces (59%) and urine (30%). The distribution of fexinidazole and metabolites to the brain was confirmed in mice and rats, and considering the lipophilicity of the molecules (logD_pH 7.4_ 2.83 [fexinidazole], 0.74 [sulfone], 0.52 [sulfoxide]), there is no reason to assume that the brain penetration potential, critical for the efficacy in stage 2 HAT, would be different in humans.

A full regulatory toxicology package has been conducted, including safety pharmacology (respiratory, cardiovascular, and general behaviour) and 4-weeks repeated-dose toxicokinetics studies in the rat and the dog. Overall, fexinidazole was well tolerated, with no specific issues of concern or target organs for toxicity identified. Fexinidazole is positive in the classical *in vitro* Ames test, but this effect is highly dependent on the presence of bacterial nitroreductases. A carefully designed set of *in vitro* and *in vivo* assays to detect possible signals of mammalian genotoxicity remained negative.

While a clearly positive Ames test result has long been considered a no-go for drug development (except for terminal diseases) as it would indicate a possible risk for (human) carcinogenicity, bacterial mutagenicity is not necessarily a relevant indication for mammalian genotoxicity, when bacterial specific metabolism is involved, especially with certain compound classes such as nitroimidazoles [57]. In fact several examples exist of nitroaromatic drug candidates currently in development for diseases requiring a much longer treatment than HAT, for instance epilepsy and tuberculosis, in which either the positive Ames test was not considered decisional to indicate a hazard to patients or no bacterial mutagenicity was detected [58], [59], [60]. Instead, a carefully designed series of *in vitro* and *in vivo* mammalian genotoxicity assays can be used to rule out the different possible mechanisms of mutagenicity that would indicate a risk for genotoxic-related carcinogenicity. The observation by us and others that it is possible to select non-mammalian mutagenic compounds within the nitroheterocycles family reopens the potential for the further use of this family of compounds with well-known anti-infective properties.

It is important to emphasize that the observed positive Ames results in nitroreductase-containing tester strains in no way point to a residual risk for carcinogenicity not captured by the detailed *in vitro* and *in vivo* mammalian genotoxicity studies as performed with fexinidazole. In contrast to what is often assumed, the *in vitro* micronucleus test measuring chromosome damage is no less sensitive as a screening test than the Ames test, even if it involves larger scale genetic damage than bacterial point mutations [61]. Although there are a few documented examples of genotoxic carcinogens that can induce chromosome damage but not bacterial point mutations (e.g. arsenic), there are, to our knowledge, no examples of genotoxic carcinogens that induce bacterial point mutation but not chromosome damage.

It has also been argued that gut flora contains bacterial nitroreductases, which could convert nitroaomatics into mutagenic species, much like what is observed in the *in vitro* Ames test and thus still present a genotoxic risk *in vivo*. A recent study of AMP397, a nitroaromatic compound previously in clinical development for epilepsy has attempted to address the issue of potential generation of gut-bacteria derived mutagens [58]. This compound has a similar profile to fexinidazole, with positive Ames test results in standard strains and lack of activity in nitroreductase-deficient bacterial strains and in mammalian cell assays. Suter *et al*. carried out a mutagenicity study of AMP397 *in vivo* in the transgenic MutaMouse model using five daily doses at the maximum tolerated dose and sampling at 3, 7 and 31 days after treatment. No evidence of mutagenicity was seen in the colon or liver. Likewise, a comet assay (measuring DNA strand breakage) did not detect any genetic damage in the jejunum or liver of treated rats after dosing the animals at a dose six times higher than that possible in the mouse study. A radioactive DNA binding study also failed to show any DNA binding in rat liver. Thus, if a mutagenic metabolite was formed by intestinal bacteria, it is unable to exert any genotoxic activity in adjacent intestinal tissue. As the genetic toxicology profile of fexinidazole is the same as AMP397 and the mechanism behind the bacterial specific mutation seen is the same, there is no reason to expect a different assessment regarding gut flora activation.

The mechanism of action of fexinidazole is not yet elucidated, but likely involves bioreductive activation. Fexinidazole and the sulfoxide and sulfone metabolites were shown to have a low single electron redox potentials (ranging between −511 and −488 mV). The nitroreductive enzymes present in mammalian cells can only reduce compounds with relatively high redox potentials under aerobic conditions. In contrast, bacterial nitroreductases such as those in the *Salmonella* assay can act at much lower redox potentials than equivalent mammalian systems. This gives a plausible explanation for the positive results in the standard Ames test and the reduced or abolished activity in nitroreductase-deficient strains. In line with these observations, it is of interest to note that the single electron redox potential of metronidazole was −516 mV, while megazole's is significantly higher at −422 mV.

The rediscovery of fexinidazole as a drug candidate also shows the success of the compound mining approach, during which a careful investigation of existing compounds within a family of known pharmacologically active compounds using state-of-the-art science, has yielded a new drug candidate for clinical development in a relatively short time. Starting the experimental work within this limited set of existing compounds in 2005 (around 700 compounds tested, mainly parasitology and genotoxicity assays), a preclinical candidate could be selected early 2007, and clinical trials initiated in the second half of 2009. Compared to drug discovery “from scratch”, this represents a significant shortcut. It also shows that it is worthwhile to dig into past research efforts to find those potential drug candidates which are lingering in drawers or on shelves. In particular in the context of non-profit drug development such as for neglected diseases where the existence of patents is not considered a prerequisite for development, this compound mining strategy may be worthwhile to pursue more vigorously.

Based on the data presented in this paper, fexinidazole has entered clinical development, and a phase I trial is currently ongoing to establish its PK and tolerability in healthy volunteers from African origin (in a combined single ascending dose and multiple ascending dose study) [62]. If well tolerated, fexinidazole is expected to progress to phase II trials in patients with stage 2 HAT by the end of 2010.

If fexinidazole successfully completes clinical development, it will represent a real breakthrough for the control of HAT in rural Africa for several reasons. Fexinidazole would be the first oral drug for stage 2 HAT, well tolerated and effective upon a short-course treatment. Compared to the current options of either 10 days of daily intravenous melarsoprol with its dreadful toxicity and waning efficacy, the very complicated eflornithine monotherapy (56 infusions over 14 days), or even the recent improvement of NECT (a combination therapy of 10 days oral nifurtimox and 7 days of 12 hourly eflornithine infusions), this would be ground-breaking. Moreover, based on its simple chemistry and short synthesis, fexinidazole is expected to be relatively cheap (certainly not more than US$ 50 per treatment, likely significantly less). Furthermore, stability data to date show that fexinidazole is very stable, which is a good starting point for the development of a stable solid dosage formulation for use in tropical climates. Finally and most significantly, it could be the first treatment to be used for both stage 1 and stage 2 HAT, thereby overturning the long-standing but complicated diagnosis and treatment paradigm which includes systematic lumbar punctures of every diagnosed patient to determine which stage of the disease they are in before deciding which treatment to prescribe (to avoid exposing a stage 1 patient to the risks and burden of the stage 2 treatments).

A safe, effective, cheap and easy to use treatment for both stage 1 and 2 HAT, ideally in combination with an easy field diagnostic, would make HAT control a realistic option for the future. In contrast to the current diagnosis and treatments options which are largely dependent on vertical HAT control approaches, this safe, effective, easy to use stage 1+2 treatment could be integrated into more horizontal approaches which are more likely to reach the extremely poor and remote populations most affected by HAT. Clearly, there are many hurdles to overcome before fexinidazole can reach this target, but it surely is the most promising candidate in many years. A concerted effort to progress fexinidazole efficiently through clinical development and registration is warranted.

## Supporting Information

Alternative Language Abstract S1 Translation of the abstract into French by Christelle Pralong
(0.03 MB DOC)

Dataset S1 (0.36 MB PDF)

Dataset S2 (0.58 MB PDF)

Dataset S3 (0.86 MB PDF)

Dataset S4 (0.15 MB PDF)

Dataset S5 (0.13 MB PDF)

Dataset S6 (0.38 MB PDF)

Dataset S7 (0.36 MB PDF)

Dataset S8 (0.16 MB PDF)

Dataset S9 (0.43 MB PDF)

Dataset S10 (7.51 MB PDF)

Dataset S11 (0.35 MB PDF)

Dataset S12 (0.31 MB PDF)

Dataset S13 (0.66 MB PDF)

Dataset S14 (0.41 MB PDF)

Dataset S15 (0.46 MB PDF)

Dataset S16 (2.17 MB PDF)

Dataset S17 (2.32 MB PDF)

Dataset S18 (0.22 MB PDF)

Dataset S19 (0.41 MB PDF)

Dataset S20 (0.30 MB PDF)

Dataset 21 (0.40 MB PDF)

Text S1 Non-clinical studies list
(0.03 MB DOC)
