# Supplementary material for: Fexinidazole – A New Oral Nitroimidazole Drug Candidate Entering Clinical Development for the Treatment of Sleeping Sickness
Source: PLoS Negl Trop Dis. 2010 Dec 21;4(12):e923. doi: 10.1371/journal.pntd.0000923 (PMC3006138; doi:10.1371/journal.pntd.0000923)
Supplement: Alternative Language Abstract S1 — Translation of the abstract into French by Christelle Pralong (0.03 MB DOC) [file pntd.0000923.s001.doc]

Extrait :

Contexte : La trypanosomiase humaine africaine (THA), connue aussi sous le nom de maladie du sommeil, est une maladie parasitaire mortelle causée par les trypanosomes. Les traitements actuels contre la THA sont rares, toxiques, ont perdu de leur efficacité ou sont très compliqués à administrer, notamment au stade avancé et fatal de la maladie (stade 2, THA chronique). Il est urgent de trouver de nouveaux traitements, efficaces et faciles d’emploi. Il est montré ici que le fexinidazole, un nitroimidazole 2-substitué 5 redécouvert par la *Drugs for Neglected Diseases initiative* (l’initiative Médicaments contre les maladies négligées) (DNDi), après des recherches intensives portant sur plus de 700 nitrohétérocycles nouveaux ou existants, pourrait être un traitement oral rapide, sûr et efficace pour lutter contre la THA aussi bien dans sa phase aigüe que chronique, et qui pourrait être distribué facilement au niveau des centres de santé primaire. Pour finaliser le développement préclinique et respecter les obligations réglementaires pour lancer des essais chez l’homme, les propriétés antiparasitaires du fexinidazole ont été évaluées, son profil pharmacocinétique, métabolique et toxicologique ont été établis.

Méthodes et Résultats : Des essais standards testant l’activité antiparasitaire in vitro et in vivo ont été réalisés pour évaluer l’efficacité du médicament dans des modèles expérimentaux de THA. Parallèlement, une série complète d’études précliniques de pharmacologie et de toxicologie a été réalisée, comme requis par les directives réglementaires internationales, avant de débuter des essais chez l’être humain.
Le fexinidazole est modérément actif in vitro contre les trypanosomes africains (IC50
contre des souches de laboratoire et des isolats cliniques récents situés entre 0.16
et 0.93 μg/mL) ; l’administration orale de fexinidazole à des doses de 100
mg/kg/jour pendant 4 jours ou de 200 mg/kg/jour pendant 5 jours a guéri des souris respectivement atteintes de la maladie aigüe ou de la maladie chronique, cette dernière constituant un modèle du stade avancé et fatal de la maladie lorsque les parasites ont déjà envahi le cerveau. Chez les animaux de laboratoire, le fexinidazole est bien absorbé après administration orale et se diffuse facilement dans tout le corps, y compris dans le cerveau. La biodisponibilité absolue du fexinidazole oral a été de 41% chez les souris, de 30% chez les rats et de 10% chez les chiens. En outre, le fexinidazole est rapidement métabolisé in vivo en au moins deux métabolites biologiquement actifs (dérivés sulfone et sulfoxide), lesquels sont sans doute responsables d’une part significative de l’effet thérapeutique.

Les principaux paramètres pharmacocinétiques après absorption orale chez les souris, pour le fexinidazole et ses métabolites sulfoxide et sulfone, donnent respectivement un Cmax de 500, 14171 et 13651 ng/mL et un AUC_0-24_ de 424, 45031 et 96286 h.ng/mL. Les profils pharmacocinétiques observés chez les rats et les chiens sont fondamentalement similaires.

Les études de toxicologie (y compris celles de pharmacologie d’innocuité et de toxicocinétique avec administration répétée pendant 4 semaines chez les rats et les chiens) ont démontré que le fexinidazole est bien toléré.

La dose sans effet indésirable observé (NOAEL, No Observed Adverse Effect Level) dans les études de toxicité répétée pendant 4 semaines a été fixée à 200 mg/kg/jour chez les rats et les chiens, sans aucun sujet de préoccupation avec des doses s’élevant jusqu’à 800 mg/kg/jour. Bien que le fexinidazole, comme beaucoup de nitrohétérocycles, soit mutagène dans le test d’Ames à cause de son métabolisme bactérien spécifique, il n’est pas génotoxique pour les cellules de mammifères in vitro ou in vivo, comme l’ont démontré un test des micronoyaux réalisé in vitro sur des lymphocytes humains, un test des micronoyaux réalisé in vivo dans la moelle osseuse de souris, et un test de synthèse d’ADN non programmée réalisé ex vivo chez des rats.

Conclusions : Les résultats des études précliniques pharmacologiques et d’innocuité indiquent que le fexinidazole est un bon candidat pour parvenir à un médicament oral sûr et efficace, sans effets indésirables écartant une évaluation chez l’être humain. Le médicament est entré en phase I d’étude clinique (premier essai chez l’être humain) en septembre 2009. Le fexinidazole est le premier candidat médicament ayant le potentiel de traiter le stade avancé de la maladie du sommeil depuis trente ans.
