# Supplementary material for: Fexinidazole – A New Oral Nitroimidazole Drug Candidate Entering Clinical Development for the Treatment of Sleeping Sickness
Source: PLoS Negl Trop Dis. 2010 Dec 21;4(12):e923. doi: 10.1371/journal.pntd.0000923 (PMC3006138; doi:10.1371/journal.pntd.0000923)
Supplement: Dataset S1 — (0.36 MB PDF) [file pntd.0000923.s002.pdf]

Fexinidazole: Evaluation of the *In Vitro* Cross Species Intrinsic Clearance and Metabolism with Mouse, Rat, Dog, Monkey and Human Hepatocytes.

Product Name:	Fexinidazole
Study Number:	0141-2007
Study Director:	
Sponsor Reference Study No.:	NA
Status:	FINAL

SUMMARY

Fexinidazole was incubated with cryopreserved hepatocytes from the male CD-1 mouse, male Sprague Dawley rat, male beagle dog, male cynomolgus monkey and from pooled human samples, at concentrations of 1 and 10 μM. The 1 μM samples were used for the cross species intrinsic clearance determination, while the 10 μM samples were used for the cross species metabolite profile determination. The incubation samples were analyzed by LC-MS/MS.

The intrinsic clearance was calculated using the half-life approach; the half-life and the intrinsic clearance were determined from the concentration of fexinidazole remaining at the sampling time points.

Fexinidazole showed half-life values of less than 2 minutes in hepatocytes of all the animal species and a half-life value of 13 minutes with human hepatocytes. Accordingly, the calculated hepatic intrinsic clearance of fexinidazole was high (≥ 3000 mL/min/kg) in all animal species tested and 124 mL/min/kg in humans.

The metabolite profile of fexinidazole was investigated at t=0 and after 30 and 120 minutes incubation at the concentration of 10 μM.

The main metabolic reactions observed were oxidation of the S atom to fexinidazole sulfoxide and fexinidazole sulfone. Des-methylation on the imidazole ring of fexinidazole sulfoxide was a minor reaction, observed in dog, monkey and humans.

In agreement with the intrinsic clearance results, the metabolism was rapid and, already at t=0, unchanged fexinidazole accounted for 83% of the drug related material with human hepatocytes, while in the animal species it was in the range 42% (mouse) to 68% (rat). The remaining part of drug related material was due to the sulfoxide.

After 30 minutes incubation, in mouse, rat, dog and humans fexinidazole sulfoxide accounted for 90 to 96%. The remaining part of drug related material was due to the sulfone. In the monkey, these metabolites accounted for 60 and 40%, respectively. Small amounts (2%) of unchanged fexinidazole were detected only with human hepatocytes.

After 120 minutes incubation, in mouse, rat, dog and humans fexinidazole sulfoxide ranged from 68% (rat) to 95% (humans), while in the monkey it accounted for 42%. The remaining part of drug related material was due to the sulfone.

The metabolite profile was qualitatively similar between hepatocytes of all species. Quantitatively, the monkey showed faster oxidation than the other species, with the sulfone and not the sulfoxide, being the most abundant metabolite after 120 minutes incubation.

Analysis of the samples on a chiral column showed that the enantiomer of fexinidazole sulfoxide with the shorter retention time was predominant in dog and human hepatocytes at all time points, whilst in the other species it was present to similar extent or with a slight prevalence compared to the enantiomer with the longer retention time.

TABLE OF CONTENTS

SUMMARY 3
1. TEST FACILITY 7
2. STUDY SPONSOR 7
3. ABBREVIATION AND DEFINITIONS OF TERMS 7
4. INTRODUCTION AND OBJECTIVES OF THIS STUDY 7
5. MATERIALS AND METHODS 8
5.1. Test Item 8
5.1.1. Quality Control 9
5.1.2. Metabolites 9
5.2. Chemicals 9
5.3. Hepatocytes 9
5.4. *In vitro* Incubations 9
5.4.1. Intrinsic Clearance Study 9
5.4.2. Metabolite Profile Study 10
5.5. Analysis of Samples for Intrinsic Clearance Determination 10
5.6. Intrinsic Clearance Determination 11
5.7. Analysis of the Samples for Metabolite Profiles 11
5.7.1. MS and MS/MS Conditions for Flow Injection Analysis 11
5.7.2. Analysis of the Incubation Samples 12
5.7.3. Separation of Fexinidazole Sulfoxide Enantiomers with a Chiral Column 14
6. PROTOCOL DEVIATIONS 14
7. ARCHIVING 14
8. RESULTS AND DISCUSSION 14
8.1. Phase I and Phase II Activity of Hepatocytes 14
8.2. Intrinsic Clearance Determination of Fexinidazole 15
8.3. Metabolite Profile and Identification 15
8.3.1. Metabolite Profiles 16
8.3.2. Metabolite Profiles with the Chiral Column 16
9. CONCLUSIONS 17

10. CONTRIBUTORS.....17

TABLES

Table 1. Intrinsic clearance results for 7-ethoxycoumarin incubated at the concentration of 1 µM with mouse, rat, dog, monkey and human hepatocytes.....18

Table 2. Phase II activity of mouse, rat, dog, monkey and human hepatocytes by incubation of 7-hydroxycoumarin at the concentration of 30 µM.....18

Table 3. Intrinsic clearance results for fexinidazole incubated at the concentration of 1 µM with mouse, rat, dog, monkey and human hepatocytes.....18

Table 4. Metabolites found at t=0 incubation of fexinidazole at the concentration of 10 µM with mouse, rat, dog, monkey and human hepatocytes.....19

Table 5. Metabolites found after 30 minutes incubation of fexinidazole at the concentration of 10 µM with mouse, rat, dog, monkey and human hepatocytes.....19

Table 6. Metabolites found after 120 minutes incubation of fexinidazole at the concentration of 10 µM with mouse, rat, dog, monkey and human hepatocytes.....19

Table 7. Percent ratio of the two enantiomers of fexinidazole sulfoxide at t=0 and after 30 and 120 minutes incubation of fexinidazole at the concentration of 10 µM with mouse, rat, dog, monkey and human hepatocytes.....20

FIGURES

Figure 1. ^{1}H-NMR spectrum of fexinidazole.....21

Figure 2. Proposed metabolic pathway of fexinidazole after incubation at the concentration of 10 µM with mouse, rat, dog, monkey and human hepatocytes.....23

Figure 3. Extracted ion chromatograms of fexinidazole sulfoxide in a standard solution and after 120 minutes incubation of fexinidazole at the concentration of 10 µM with mouse, rat, dog, monkey and human hepatocytes.....24

APPENDICES

Appendix 1. Peak area (counts) at different time points of fexinidazole incubated at the concentration of 1 µM with mouse, rat, dog, monkey and human hepatocytes.....25

Appendix 2. Elution gradients tested for chromatographic separation of metabolites (reverse phase column).....26

Appendix 3. Elution gradients tested for chromatographic separation of fexinidazole sulfoxide (chiral column).....27

1. TEST FACILITY

The present study has been conducted at
Accelera
Nerviano Medical Sciences S.r.l.

2. STUDY SPONSOR

Drugs for Neglected Diseases *initiative* (DND*i*)
1, Place St Gervais
CH-1201 Geneva
Switzerland

3. ABBREVIATION AND DEFINITIONS OF TERMS

CL_{int}	Intrinsic clearance
Da	Dalton
DMSO	Dimethyl sulfoxide
7-EC	7-Ethoxycoumarin
ESI	Electrospray ionisation
7-HC	7-Hydroxycoumarin
HPLC	High Performance Liquid Chromatography
LC-MS	Liquid chromatography-Mass spectrometry
LC-MS/MS	Liquid chromatography-Mass spectrometry/Mass spectrometry
m/z	Mass to charge
MRM	Multiple reaction monitoring
MS	Mass spectrometry
MS/MS	Mass spectrometry/Mass spectrometry
PDA	Photodiode Array
$t_{1/2}$	Half life
TEA	Triethylamine
TFA	Trifluoroacetic acid
TIS	Turbo ion spray
TOF	Time of flight

4. INTRODUCTION AND OBJECTIVES OF THIS STUDY

Fexinidazole is a compound under investigation in the laboratories of the Study Sponsor. The structure of the compound is shown below:

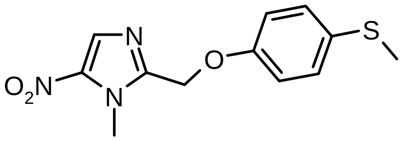

The aim of this study was:

1) to evaluate the intrinsic hepatic clearance of fexinidazole using mouse, rat, dog, monkey and human hepatocytes;

2) to evaluate the *in vitro* metabolite profile of fexinidazole using hepatocytes of the same species as above, and to identify the main metabolites formed.

3) to assess the separation of chiral sulfoxide metabolites.

The intrinsic hepatic clearance of fexinidazole was determined using the half-life approach, by measuring the substrate disappearance during 120 minutes incubation with mouse, rat, dog, monkey and human hepatocytes. LC-MS/MS was used for the detection of the compound during the incubation.

The metabolite profile and metabolite identification of fexinidazole was performed at a concentration of 10 μM of fexinidazole using hepatocytes from the five species above. The incubation samples were also analyzed on a chiral column.

The experimental part of the study was performed in the period 4 June 2007 -5 September 2007.

This study was a non-regulated study and was conducted according to Protocol 0141-2007-P. Relevant Standard Operating Procedures of Accelera, Nerviano Medical Sciences, followed during the study were: MET-P/018/00; PCD-S-027; PCD-S-032; PCD-S-134; PCD-S-166.

5. MATERIALS AND METHODS

5.1. Test Item

Fexinidazole, molecular formula $C_{12}H_{13}N_3O_3S$; formula weight 279.31; batch number not applicable; was provided by the Sponsor. The test compound was stored at room temperature.

5.1.1. Quality Control

A quality control analysis of fexinidazole was performed via ^{1}H-NMR in DMSO and LC-MS on May 21st, 2007, before starting the metabolism work. The purity as per ^{1}H-NMR and LC-MS analyses was greater than 98%. The ^{1}H-NMR of fexinidazole is shown in Figure 1.

5.1.2. Metabolites

Fexinidazole sulfoxide, molecular formula $C_{12}H_{13}N_{3}O_{4}S$; formula weight 295.32; and fexinidazole sulfone, molecular formula $C_{12}H_{13}N_{3}O_{5}S$; formula weight 311.32, were provided by the Swiss Tropical Institute (Basel, Switzerland).

5.2. Chemicals

Reagents and solvents were of analytical grade and obtained from Sigma-Aldrich (St. Louis, Missouri, USA).

5.3. Hepatocytes

Cryopreserved hepatocytes from the male CD1 mouse, lot CSI; male Sprague Dawley rat, lot LPL; male beagle dog, lot BWX; male cynomolgus monkey, lot EHH; and pooled human samples, lot MJI, were purchased from In Vitro Technologies Inc (Baltimore, Maryland, USA).

5.4. *In vitro* Incubations

On the day of the experiment a 10 mM stock solution of fexinidazole in DMSO was prepared and, within 3 hours, aliquots of this freshly prepared solution, after suitable dilution, were added to the incubation matrix, to reach fexinidazole final concentrations of 1 and 10 μM. The final percentage of DMSO was 0.01% in the incubation samples for the intrinsic clearance determination and 0.1% in the incubation samples for metabolite profiling.

On the day of experiment, the cryopreserved hepatocytes were thawed following the detailed instructions provided by the supplier.

The number of viable cells was determined using a Trypan Blue exclusion method. Viabilities were 69% for mouse, 73% for rat, 89% for dog, 84% for monkey and 91% for human hepatocytes.

5.4.1. Intrinsic Clearance Study

For the intrinsic clearance determination, fexinidazole was incubated at a concentration of 1 μM with mouse, rat, dog, monkey and human hepatocytes (300000 cells/mL for the mouse and 1 million cells/mL for the other species) in a final incubation volume of 0.75 mL

Leibovitz L-15 medium, at 37°C. Incubations were performed in duplicate in a 48-well plate under shaking. Sampling was performed using an automatic liquid handling system (Multiprobe II EX, Packard). At 0, 1, 5, 10, 15, 20, 30, 60 and 120 minutes, 50 μL aliquots of the incubates were taken, then 80 μL of ice-cold acetonitrile and 20 μL of 1 μM warfarin in acetonitrile (internal standard) were added, and samples centrifuged at 2000 rpm for 20 min. The supernatant was analyzed by LC-MS/MS.

The chemical stability of fexinidazole was checked by incubating the compound at 37°C in the medium alone at t = 0 and 120 minutes.

For the determination of phase I and phase II activity of hepatocytes, 7-ethoxycoumarin (7-EC) 1 μM, and 7-hydroxycoumarin (7-HC) 30 μM were used as positive controls and incubated under the same conditions as fexinidazole. Aliquots of incubates were taken at 0, 10, 20, 30, 60 and 120 minutes and processed as fexinidazole samples.

5.4.2. Metabolite Profile Study

For metabolite profiling, fexinidazole was incubated at a concentration of 10 μM with mouse, rat, dog, monkey and human hepatocytes (300000 cells/mL for the mouse and 1 million cells/mL for the other species) in a final incubation volume of 2 mL Leibovitz L-15 medium, at 37°C. Aliquots of the incubation samples were taken at t = 0, 30 and 120 minutes; the metabolism was stopped by the addition of an equal volume of cold acetonitrile and the samples centrifuged at 1500 rpm for 20 min. The supernatant was stored at -20°C until analysis. Control incubations were performed with the compound in the medium alone for 120 minutes.

5.5. Analysis of Samples for Intrinsic Clearance Determination

The analysis of the samples for intrinsic clearance determination was performed by an LC-MS/MS method, using the following conditions:

**HPLC Equipment and Conditions**					
HPLC system	Agilent 1100 binary pump (Agilent, Palo Alto, USA)				
Autosampler	CTC Pal (Alfatech, San Jose, USA)				
Analytical column	Guard Column SB-C8 4.6 x 12.5 mm, 5 μm (Zorbax)				
Column temperature	ambient				
Mobile phase A	10 mM ammonium formate pH 4.0: acetonitrile (95:5, v:v)				
Mobile phase B	10 mM ammonium formate pH 4.0: acetonitrile (5:95, v:v)				
Injection volume	20 μL				
Gradient conditions	Step	Total time (min)	flow (μL/min)	%A	% B
	0	0.0	600	100	0
	1	0.0	600	0	100
	2	0.20	600	0	100

	3 1.10 600 0 100<br>4 1.45 600 100 0<br>5 1.55 600 100 0
**MS Equipment and Conditions**	
Mass spectrometer	API 4000 Triple Quadrupole (MDS-Sciex, Toronto, Canada)
Source	Turbo Ion Spray (TIS)
Ion mode	Positive for fexinidazole and 7-EC.<br>Negative for 7-HC, 7-HC sulphate and 7-HC glucuronide.<br>Warfarin was analysed both in positive and negative ion mode.
Scan mode	Multiple Reaction Monitoring (MRM)
MRM transitions	280.0 → 140.2 (fexinidazole)<br>191.0 → 163.0 (7-EC)<br>161.0 → 133.0 (7-HC)<br>241.0 → 161.0 (7-HC sulphate)<br>337.0 → 161.0 (7-HC glucuronide)<br>309.3 → 163.0 (warfarin positive ion mode)<br>307.3 → 57.0 (warfarin negative ion mode)
Software	Analyst 1.2 and Automaton (Sciex)

5.6. Intrinsic Clearance Determination

The intrinsic clearance (CL_{int}) of fexinidazole and of 7-ethoxycoumarin was calculated using the half-life approach. The half-life and the CL_{int} were determined from the concentration remaining at the different sampling points using the LC-MS/MS method. By plotting the natural logarithmic area of the compound remaining against the time, the slope was calculated by exponential regression analysis, and converted into the half-life ($t_{1/2}$) and CL_{int} expressed as µL/min/million cells and mL/min/Kg.

5.7. Analysis of the Samples for Metabolite Profiles

5.7.1. MS and MS/MS Conditions for Flow Injection Analysis

The Q-TOF 2 mass spectrometer was calibrated against the known accurate masses of the fragment ions of [glu[1]]-fibrinopeptide B by continuous infusion at 10 µL/min of a 10 µg/mL solution in a mixture of 1% aqueous formic acid: acetonitrile (1:1, v:v). The tuning parameters were optimized by continuous infusion at 3 µL/min of a 10 µM working solutions of fexinidazole in 10 mM ammonium formate, pH 4.5: acetonitrile (8:2, v:v). In order to improve the accuracy of the mass measurements, during all analyses a 10 µg/mL solution of Met-Arg-Phe-Ala (m/z 524.2655) in 10 mM ammonium formate, pH 4.5: acetonitrile (1:1, v:v) was infused into the reference source of the LockSpray at a rate of 1-2 µL/min and a reference scan obtained every 10 seconds for automatic correction of the

accurate masses. All data were acquired in centroid mode. Afterwards, the main parameters (cone voltage and collision energy) were further optimized by flow injection analysis by switching between 5 cone and 5 collision energy values.

The following apparatus and conditions were used for flow injection analysis:

**MS Equipment and Conditions**	
Mass spectrometer	Q-TOF 2 (Waters, Milford, Massachusetts, USA)
Source	Electrospray Ionization (ESI) (LockSpray)
Acquisition software	Masslynx 4.1 (Waters)
Scan mode	MS Full Scan and MS/MS Full Scan
Acquisition polarity	Positive
Acquisition mode	Centroid
Acquisition time	1.5 min
Scan range	100 to 1000 Da
Scan time	0.5 sec
Interscan time	0.1 sec
Capillary voltage	3 kV
Cone voltage	20, 25, 30, 35, 40 eV
Extractor voltage	2 eV
Rf Lens	1.5 eV
Ion energy	1.5 eV
Source temperature	120°C
Desolvation gas temperature	250°C
Collision energy	20, 25, 30, 35, 40 eV
Mobile phase A	10 mM ammonium formate, pH 4.5
Mobile phase B	Acetonitrile
Elution	Isocratic, 50% A: 50% B
Flow rate	200 µL/min
Injection volume	50 µL
Total run time	1.5 min

5.7.2. Analysis of the Incubation Samples

Before analysis, the incubation samples were evaporated to dryness under a stream of nitrogen (Turbovap), then the residues were reconstituted to the original incubation volumes with 10 mM ammonium formate, pH 4.5: acetonitrile 8:2, v:v.

The analyses were performed using an HPLC system on line with a photodiode array (PDA) detector and a mass spectrometer (MS). Equipment and conditions are given below.

HPLC Equipment and Conditions	
Autosampler	HTC Pal (CTC Analytics, San Jose, USA) equipped with a 100 µL sample loop and a 100 µL syringe
Binary pump	1100 Series (Agilent, Palo Alto, CA, USA)
Degasser	1100 Series (Agilent)
Column oven	1100 Series (Agilent)
Column	XBridge C8, 2.1 x 150 mm, 3.5 µm (Waters)
Guard column	C8, 2 x 4 mm (Phenomenex)
Column temperature	40°C
Mobile phase A	10 mM ammonium formate, pH 4.5
Mobile phase B	Acetonitrile
Flow rate	0.3 mL/min
Run time	35 minutes
Injection volume	75 µL
Gradient conditions	Time (min) / Solvent A (%) / Solvent B (%)<br>0.0 / 95 / 5<br>1.0 / 95 / 5<br>4.0 / 75 / 25<br>21.0 / 40 / 60<br>21.5 / 5 / 95<br>27.0 / 5 / 95<br>27.5 / 95 / 5<br>35.0 / 95 / 5
**PDA Detector and Conditions**	
Diode Array detector	1100 Series (Agilent)
Range	190 to 600 nm
Resolution	2 nm
Width	0.1 min
**MS Acquisition Conditions**	
Acquisition software	Masslynx 4.1 (Waters)
Processing software	Metabolynx 4.1 (Waters)
Scan mode	MS Full Scan and MS/MS Full Scan
Acquisition polarity	Positive
Acquisition mode	Centroid
Acquisition time	27 minutes
Scan range	75 to 750 Da (MS Full Scan); 50 to 750 Da (MS/MS)
Scan time	0.9 sec
Interscan delay	0.1 sec
Capillary voltage	3 kV
Cone voltage	25 eV
Collision energy	10 eV (MS Full Scan); 25 eV (MS/MS)

Extractor voltage	2 eV
Rf Lens	1.5 eV
Ion energy	1.5 eV
Source temperature	120°C
Desolvation gas temperature	250°C

The incubation samples were analyzed in MS positive ion mode and the data searched on the accurate masses of possible metabolites using the Metabolynx software, version 4.1 (Waters), using a mass window of 40 mDa. Samples were then re-run in MS/MS mode by selecting the masses of possible metabolites. The collision energy used to obtain MS/MS data was 25 eV.

5.7.3. Separation of Fexinidazole Sulfoxide Enantiomers with a Chiral Column

The separation was performed using the equipment described in 5.7.2, a chiral HPLC column Chirobiotic TAG 4.6 x 250 mm, 5 µm (Astec) and methanol as mobile phase.

6. PROTOCOL DEVIATIONS

No protocol deviations were observed during the study.

7. ARCHIVING

The original protocol, all raw data and supporting documents produced at the Test Facility, and the original Final Report are filed in the Archives of Accelera, Nerviano Medical Sciences S.r.l., Nerviano (Italy) for the period of time agreed with the Sponsor (at least 3 years) after which the Sponsor will be contacted for instructions regarding dispatch or disposal of the material. A copy of the protocol and a copy of the report with original signatures are filed with the Sponsor.

8. RESULTS AND DISCUSSION

8.1. Phase I and Phase II Activity of Hepatocytes

The hepatocytes of each species used in this study were checked for their activities towards phase I and phase II reactions, using 7-EC and 7-HC. Table 1 shows the disappearance half-life and intrinsic clearance of 7-EC incubated at the concentration of 1 µM with hepatocytes of the five species. Table 2 shows the formation of 7-HC sulphate and 7-HC glucuronide from 7-HC incubated at the concentration of 30 µM with hepatocytes. The results confirmed that the hepatocytes used in this study exhibited active metabolism.

8.2. Intrinsic Clearance Determination of Fexinidazole

The intrinsic clearance of fexinidazole was determined at the concentration of 1 μM in hepatocytes of mouse, rat, dog, monkey and human, using the half-life approach. The starting concentration of 1 μM was assumed to be << of Km.

Fexinidazole showed half-life values of less than 2 minutes in hepatocytes of all animal species and a half-life value of 13 minutes with human hepatocytes. Accordingly, the hepatic intrinsic clearance of fexinidazole was high (≥ 3000 mL/min/kg) in all animal species tested and 124 mL/min/kg in humans (Table 3).

The peak area (counts) of fexinidazole at the different time points are shown in Appendix 1.

Negative controls with fexinidazole incubated for 120 minutes at 37°C in the incubation medium alone show about 30-40% loss of fexinidazole.

8.3. Metabolite Profile and Identification

The metabolite profile of fexinidazole was determined at the concentration of 10 μM at t=0 and after 30 and 120 minutes incubation with mouse, rat, dog, monkey and human hepatocytes.

The chromatographic separation of fexinidazole and its metabolites were obtained with a reverse phase HPLC column XBridge C8, 2.1 x 150 mm, 3.5 μm (Waters). Different gradients were tested to obtain a good separation of the metabolites with suitable retention times for the analysis of the incubation samples. All the gradients tested are shown in Appendix 2.

The identity of metabolites was proposed based on their accurate mass (using LockSpray correction), with an accuracy of +/-5 mDa. MS/MS analyses were performed to verify that peaks were drug-related and to obtain structural information from the fragment ions formed. The identity of fexinidazole sulfoxide and fexinidazole sulfone in the incubation samples was confirmed also by comparing the retention times of the two metabolites with those of the authentic standard compounds.

The approximate relative amounts were determined from the absolute areas taken from a selected ion chromatogram at the metabolite masses with a mass window of 0.04 Da. The absolute areas of fexinidazole, fexinidazole sulfoxide and fexinidazole sulfone were normalized based on their relative MS response determined by injecting a 10 μM solution of the available standards (factors 1.3, 1 and 8 for fexinidazole, fexinidazole sulfoxide and fexinidazole sulfone, respectively). Nevertheless, these results should be regarded as semi-quantitative (approximate relative amounts of fexinidazole and each metabolite in the samples).

The proposed metabolic pathway of fexinidazole is shown in Figure 2.

8.3.1. Metabolite Profiles

The relative amounts of fexinidazole and metabolites detected at t=0 and after 30 and 120 minutes incubation with hepatocytes of different species are reported in Table 4, Table 5 and Table 6, respectively.

Fexinidazole was rapidly metabolized.

At **t=0**, unchanged fexinidazole accounted for 83% of total drug related material with human hepatocytes, while in the animal species it was in the range 42% (mouse) to 68% (rat). The remaining drug related material was due to the sulfoxide **M1**.

After **30 minutes** incubation, fexinidazole was detected in small amounts (2%) only in human hepatocytes. In the mouse, rat, dog and humans, the major part of the drug related material (90 to 96%) was the sulfoxide metabolite M1. The remaining part of drug related material was due to the sulfone **M2**. In the monkey, M1 and M2 accounted for 60 and 40%, respectively. Metabolite **M3** (m/z = 282.06), corresponding to M1 des-methylated on the imidazole ring, was detected in traces (less than 1 to 1%) with dog, monkey and human hepatocytes.

After **120 minutes** incubation, fexinidazole was detected in traces (less than 1%) only in human hepatocytes. In the mouse, rat, dog and humans, the sulfoxide metabolite M1 ranged from 68% (rat) to 95% (humans), while in the monkey M1 accounted for 42%. The remaining part of drug related material was due to the sulfone metabolite M2. Metabolite **M3** was detected in traces only with dog hepatocytes.

As control, fexinidazole was incubated at the concentration of 10 μM with the Leibovitz medium alone. After 2 hours incubation, fexinidazole accounted for about 84% of the total drug related material in the sample.

8.3.2. Metabolite Profiles with the Chiral Column

A chiral HPLC column Chirobiotic TAG 4.6 x 250 mm, 5 μm (Astec) was used for the chromatographic separation of the two enantiomers of fexinidazole sulfoxide. Different gradients and mobile phases were tested. The best results were obtained using methanol as mobile phase with isocratic elution. Under this chromatographic condition, the two enantiomers were eluted with retention times of 8.9 and 9.8 minutes, respectively. All the gradients tested are reported in Appendix 3.

The percent ratio of the two enantiomers of fexinidazole sulfoxide at t=0 and after 30 and 120 minutes incubation of fexinidazole with hepatocytes of the five species is reported in Table 7. The extracted ion chromatograms of fexinidazole sulfoxide in a standard solution and after 120 minutes incubation of fexinidazole with mouse, rat, dog, monkey and human hepatocytes are shown in Figure 3.

In the **mouse**, the enantiomer with the shorter retention time (designated as enantiomer A) is present in higher amounts than the enantiomer with the longer retention time (designated as enantiomer B) at t=0 and 120 min, while the opposite was seen at t=30 min. In the **rat**, A and B are present in similar amounts at t=0; then A increased with time to about 1.5 fold B. In the **dog**, enantiomer A is present at 3 fold greater amounts than enantiomer B at all times. In the **monkey** the two enantiomers are present in a ratio of about 1:1, with a slightly prevalence of A at t=0 and of B at t=120 min. In humans, the two enantiomers are present in a ratio of about 2:1 at all times.

9. CONCLUSIONS

Fexinidazole showed very rapid and high intrinsic clearance with hepatocytes of all animal species and a rapid and high intrinsic clearance with human hepatocytes. These *in vitro* results typically translate into high *in vivo* metabolic clearance values.

In agreement with the above, unchanged fexinidazole was detected in small amounts only (2%) with human hepatocytes after 30 minutes incubation. The metabolic reactions observed were oxidation of the S atom to fexinidazole sulfoxide and fexinidazole sulfone. Small amounts of the sulfoxide des-methylated on the imidazole ring were also detected. The metabolite profiles in hepatocytes are indicative of metabolic pathways qualitatively similar among the different species tested. Fexinidazole sulfoxide is the major metabolite produced by the hepatocytes of all the species, with the exception of the monkey, showing faster oxidation than the other species, with the sulfone, rather than the sulfoxide, being the most abundant metabolite after 120 minutes incubation.

The enantiomer of fexinidazole sulfoxide with the shorter retention time (A) was predominant in dog and human hepatocytes at all times, whilst in the other species it was present to similar extent or with a slight prevalence compared to the enantiomer with longer retention time.

10. CONTRIBUTORS

TABLES AND FIGURES

Table 1. Intrinsic clearance results for 7-ethoxycoumarin incubated at the concentration of 1 μM with mouse, rat, dog, monkey and human hepatocytes.

Species	$t_{1/2}$ (min)	CL_{int} *in vitro* (μL/min/million cells)	CL_{int} *in vitro* (mL/min/kg)
Mouse	2.6	901	2380
Rat	13.2	52.4	251
Dog	1.7	405	3108
Monkey	2.5	272	1479
Human	4.8	144	345

Table 2. Phase II activity of mouse, rat, dog, monkey and human hepatocytes by incubation of 7-hydroxycoumarin at the concentration of 30 μM.

Species	7-HC sulphate (pmol/min/million cells)	7-HC glucuronide (pmol/min/million cells)
Mouse	1384	4390
Rat	595	810
Dog	249	340
Monkey	283	499
Human	7	131

Table 3. Intrinsic clearance results for fexinidazole incubated at the concentration of 1 μM with mouse, rat, dog, monkey and human hepatocytes.

Species	$t_{1/2}$ (min)	CL_{int} *in vitro* (μL/min/million cells)	CL_{int} *in vitro* (mL/min/kg)
Mouse	1.4	1634	4312
Rat	1.2	602	2890
Dog	1.1	656	5042
Monkey	0.6	1189	6467
Human	13.4	51.9	124

Table 4. Metabolites found at t=0 incubation of fexinidazole at the concentration of 10 μM with mouse, rat, dog, monkey and human hepatocytes.

Metabolite	Molecular formula	m/z	RT (min)	% of total drug related material(1)				
				Mouse	Rat	Dog	Monkey	Human
P	$C_{12}H_{13}N_3O_3S$	280.08	16.7	42	68	50	53	83
M1	$C_{12}H_{13}N_3O_4S$	296.07	9.4	58	32	50	47	17

(1) The approximate relative amounts were determined from the absolute areas taken from a selected ion chromatogram at the metabolite masses with a mass window of 0.04 Da. The absolute areas of fexinidazole and M1 were normalized based on their relative MS response determined by injecting a 10 μM solution of the available standards. Nevertheless, these results should be regarded as semi-quantitative.

Table 5. Metabolites found after 30 minutes incubation of fexinidazole at the concentration of 10 μM with mouse, rat, dog, monkey and human hepatocytes.

Metabolite	Molecular formula	m/z	RT (min)	% of total drug related material(1)				
				Mouse	Rat	Dog	Monkey	Human
P	$C_{12}H_{13}N_3O_3S$	280.08	16.7					2
M1	$C_{12}H_{13}N_3O_4S$	296.07	9.4	92	90	94	60	96
M2	$C_{12}H_{13}N_3O_5S$	312.07	10.9	8	10	4	40	2
M3	$C_{11}H_{11}N_3O_4S$	282.06	7.7			1	<1	<1

(1) The approximate relative amounts were determined from the absolute areas taken from a selected ion chromatogram at the metabolite masses with a mass window of 0.04 Da. The absolute areas of fexinidazole, M1 and M2 were normalized based on their relative MS response determined by injecting a 10 μM solution of the available standards. Nevertheless, these results should be regarded as semi-quantitative.

Table 6. Metabolites found after 120 minutes incubation of fexinidazole at the concentration of 10 μM with mouse, rat, dog, monkey and human hepatocytes.

Metabolite	Molecular formula	m/z	RT (min)	% of total drug related material(1)				
				Mouse	Rat	Dog	Monkey	Human
P	$C_{12}H_{13}N_3O_3S$	280.08	16.7					<1
M1	$C_{12}H_{13}N_3O_4S$	296.07	9.4	80	68	84	42	95
M2	$C_{12}H_{13}N_3O_5S$	312.07	10.9	20	32	15	58	4
M3	$C_{11}H_{11}N_3O_4S$	282.06	7.7			1		

(1) The approximate relative amounts were determined from the absolute areas taken from a selected ion chromatogram at the metabolite masses with a mass window of 0.04 Da. The absolute areas of fexinidazole, M1 and M2 were normalized based on their relative MS response determined by injecting a 10 μM solution of the available standards. Nevertheless, these results should be regarded as semi-quantitative.

Table 7. Percent ratio of the two enantiomers of fexinidazole sulfoxide at t=0 and after 30 and 120 minutes incubation of fexinidazole at the concentration of 10 μM with mouse, rat, dog, monkey and human hepatocytes.

Time	% of each enantiomer in the sample									
	Mouse		Rat		Dog		Monkey		Human	
**Rt (min)**	**8.9**	**9.8**	**8.9**	**9.8**	**8.9**	**9.8**	**8.9**	**9.8**	**8.9**	**9.8**
**t=0**	61	39	47	53	81	19	56	44	68	32
**30 min**	39	61	58	42	66	34	50	50	63	37
**120 min**	55	45	62	38	76	24	46	54	68	32

Figure 1. ^{1}H-NMR spectrum of fexinidazole.

05/23/2007 12.41.24
Unknown FexinidazoleHOE239_I

Acquisition Time (sec)	5.0000	*Comment*	Unknown FexinidazoleHOE239_I	*Date*	May 21 2007
Date Stamp	May 21 2007	*File Name*	\\10.206.83.86\archive\400i\spectra\FEXINIDAZOLEHOE239_I	*Frequency (MHz)*	400.50
Nucleus	1H	*Number of Transients*	64	*Origin*	UNITY INOVA-400; HostID 80f8f18d; VNMR 6.1C 2000-08-18 patched all103 / Solaris 7
Original Points Count	39956	*Owner*	Autosampler user 311 (autos); UID/GID: 1003/30	*Points Count*	65536
Pulse Sequence	s2pul	*Receiver Gain*	46.00	*Solvent*	DMSO-d6
Spectrum Offset (Hz)	1798.2625				
Sweep Width (Hz)	7991.21	*Temperature (degree C)*	28.000		

	PREPARATION					RESULTS						
	**MW**	**sol.**	**mg**	**µl t.**	**µl a.**	**ppm**	**int.**	**n°H**	**rel.Int.**	**Avrg.**	**RSD%**	**PURITY**
**FexinidazoleHOE239**	279.310	1	2.119	759	**760**	3.59	294.07	3	98.02	97.98	0.06	**98.3**
						5.28	195.89	2	97.95			
		2	2.146	768	**770**	3.59	295.96	3	98.65	98.66	0.02	
						5.28	197.35	2	98.68			

FEXINIDAZOLEHOE239_I.esp

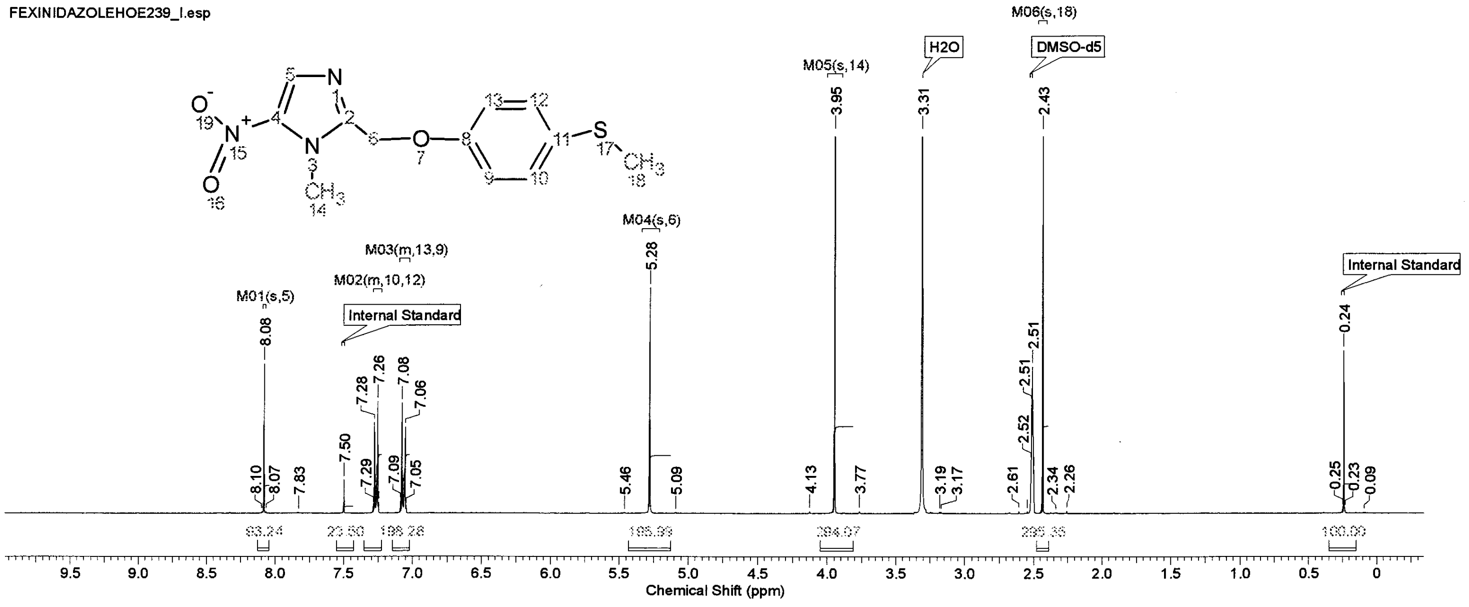

23/05/07

No.	(ppm)	Annotation
1	[0.24 .. 0.26]	Internal Standard
2	[2.51 .. 2.53]	DMSO-d5
3	3.31	H2O
4	[7.50 .. 7.52]	Internal Standard

No.	Atom	Exp. Shift (ppm)	Multiplet
1	18	2.43	M06
2	14	3.95	M05
3	6	5.28	M04
4	13	7.07	M03
5	9	7.07	M03
6	10	7.27	M02
7	12	7.27	M02
8	5	8.08	M01

1H NMR (401 MHz, DMSO-d_6) δ ppm 2.43 (s, 3 H) 3.95 (s, 3 H) 5.28 (s, 2 H) 7.03 - 7.10 (m, 2 H) 7.23 - 7.29 (m, 2 H) 8.08 (s, 1 H)

23/05/07

Figure 2. Proposed metabolic pathway of fexinidazole after incubation at the concentration of 10 μM with mouse, rat, dog, monkey and human hepatocytes.

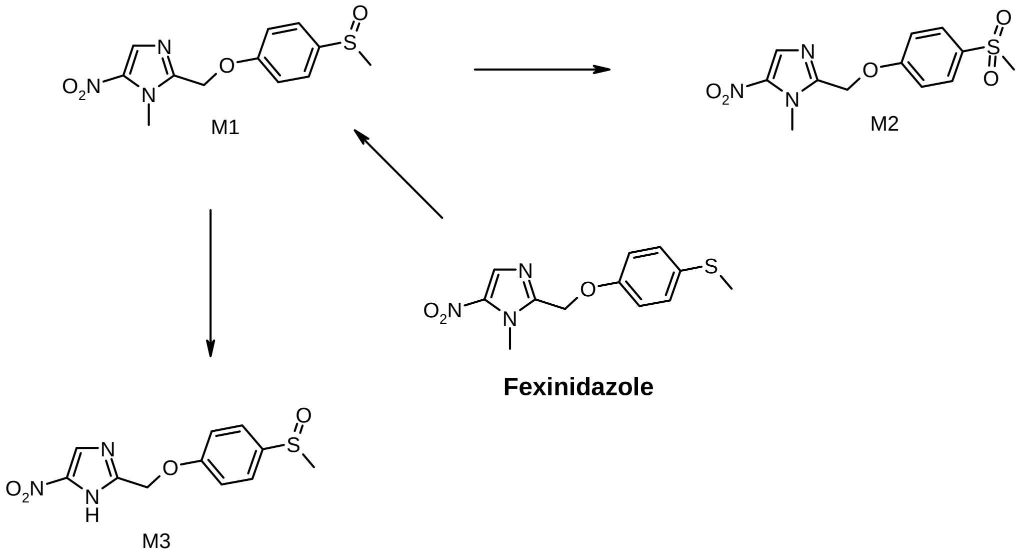

Figure 3. Extracted ion chromatograms of fexinidazole sulfoxide in a standard solution and after 120 minutes incubation of fexinidazole at the concentration of 10 µM with mouse, rat, dog, monkey and human hepatocytes.

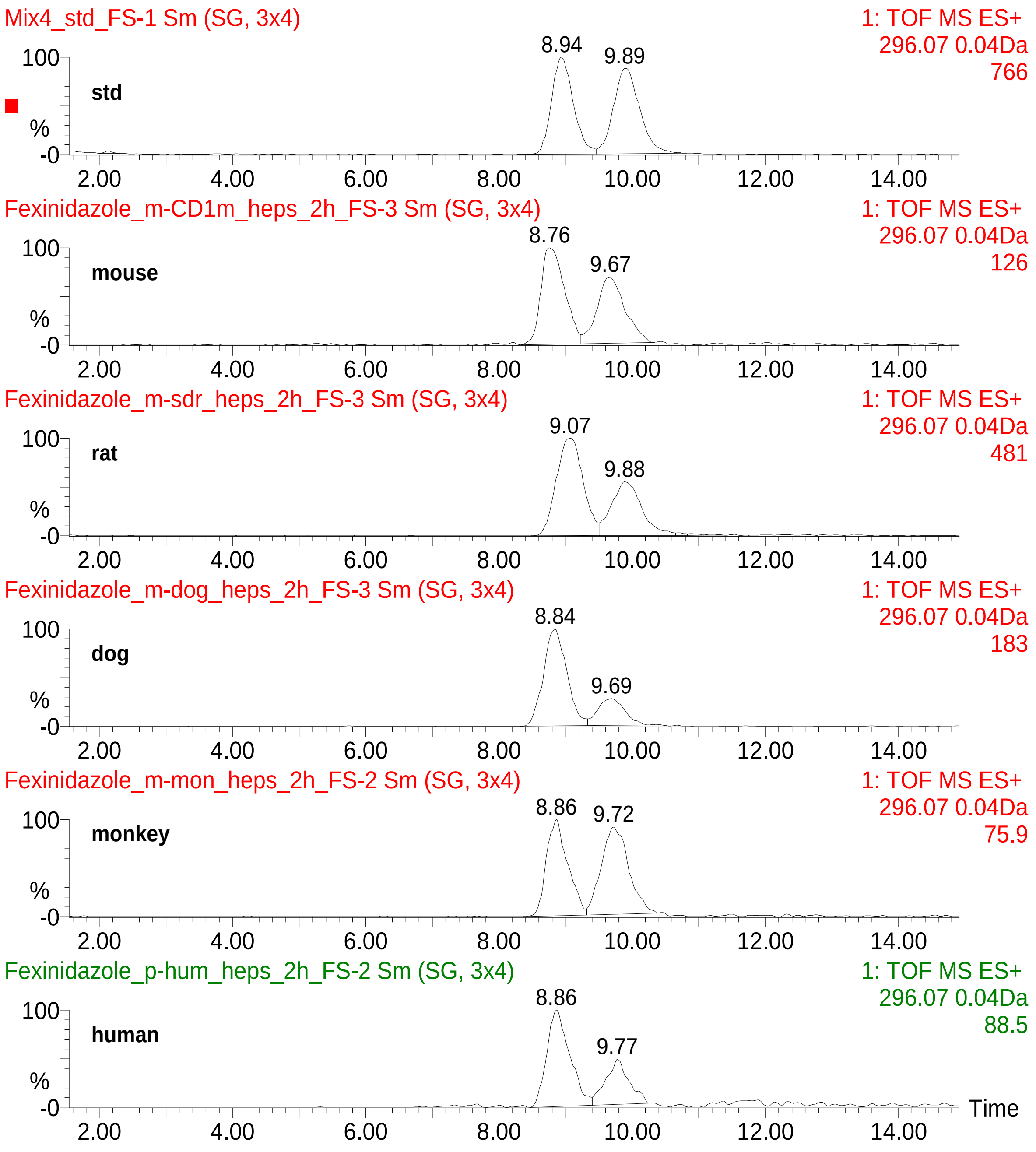

APPENDICES

Appendix 1. Peak area (counts) at different time points of fexinidazole incubated at the concentration of 1 μM with mouse, rat, dog, monkey and human hepatocytes.

Species	Exp	T=0	T=1’	T=5’	T=10’	T=15’	T=20’	T=30’	T=60’	T=120’
Mouse	1	89200	44200	8100	458	216	99	nd	nd	nd
	2	81200	31500	5290	178	nd	nd	nd	nd	nd
Rat	1	39800	22700	2810	nd	nd	nd	nd	nd	nd
	2	30700	17700	970	nd	nd	nd	nd	nd	nd
Dog	1	49200	22400	827	nd	nd	nd	nd	nd	nd
	2	51200	23200	3050	nd	nd	nd	nd	nd	nd
Monkey	1	22300	4260	120	nd	nd	nd	nd	nd	nd
	2	21300	4930	nd	nd	nd	nd	nd	nd	nd
Human	1	53000	21900(1)	32700	25100	20200	19600	7440	1250	nd
	2	50600	24700(1)	42900	29600	21400	18300	8730	734	nd

nd: not detectable
(1) Excluded from the calculation, because outliers.

Appendix 2. Elution gradients tested for chromatographic separation of metabolites (reverse phase column).

Gradient 1			
**Time (min)**	**%A**	**%B**	**Flow (mL/min)**
0	95	5	0.3
1	95	5	0.3
21	5	95	0.3
27	5	95	0.3
27.5	95	5	0.3
35	95	5	0.3
A: 10 mM ammonium formate, pH 4.5<br>B: acetonitrile			

Gradient 2			
**Time (min)**	**%A**	**%B**	**Flow (mL/min)**
0	95	5	0.3
1	95	5	0.3
4	75	25	0.3
21	40	60	0.3
27	5	95	0.3
27.5	95	5	0.3
35	95	5	0.3
A: 10 mM ammonium formate, pH 4.5<br>B: acetonitrile			

Gradient 3			
**Time (min)**	**%A**	**%B**	**Flow (mL/min)**
0	95	5	0.3
1	95	5	0.3
4	75	95	0.3
21	40	60	0.3
21.5	5	95	0.3
27	5	95	0.3
27.5	95	5	0.3
35	95	5	0.3
A: 10 mM ammonium formate, pH 4.5<br>B: acetonitrile			

Appendix 3. Elution gradients tested for chromatographic separation of fexinidazole sulfoxide (chiral column).

Gradient 1			
**Time (min)**	**%A**	**%B**	**Flow (mL/min)**
0	95	5	1
1	95	5	1
25	25	75	1
25.5	10	90	1
33	10	90	1
33.5	95	5	1
45	95	5	1
A: 10 mM ammonium formate, pH 4.5<br>B: acetonitrile			

Gradient 2			
**Time (min)**	**%A**	**%B**	**Flow (mL/min)**
0	50	50	1
1	50	50	1
25	10	90	1
33	10	90	1
33.5	50	50	1
45	50	50	1
A: 10 mM ammonium formate, pH 4.5<br>B: acetonitrile			

Gradient 3			
**Time (min)**	**%A**	**%B**	**Flow (mL/min)**
0	70	30	1
15	70	30	1
20	10	90	1
28	10	90	1
28.5	70	30	1
35	70	30	1
A: 10 mM ammonium formate, pH 4.5<br>B: acetonitrile			

Gradient 4			
**Time (min)**	**%A**	**%B**	**Flow (mL/min)**
0	60	40	1
15	60	40	1
20	10	90	1
28	10	90	1
28.5	60	40	1
35	60	40	1
A: 10 mM ammonium formate, pH 4.5<br>B: acetonitrile			

Gradient 5			
**Time (min)**	**%A**	**%B**	**Flow (mL/min)**
0	65	35	1
15	65	35	1
20	10	90	1
28	10	90	1
28.5	65	35	1
35	65	35	1
A: 10 mM ammonium formate, pH 4.5<br>B: acetonitrile			

Gradient 6			
**Time (min)**	**%A**	**%B**	**Flow (mL/min)**
0	55	45	1
15	55	45	1
20	10	90	1
28	10	90	1
28.5	55	45	1
35	55	45	1
A: 10 mM ammonium formate, pH 4.5<br>B: methanol			

Gradient 7			
Time (min)	%A	%B	Flow (mL/min)
0	40	60	1
15	40	60	1
20	10	90	1
28	10	90	1
28.5	40	60	1
35	40	60	1
A: 10 mM ammonium formate, pH 4.5<br>B: methanol			

Gradient 8			
Time (min)	%A	%B	Flow (mL/min)
0	40	60	1
15	40	60	1
20	10	90	1
28	10	90	1
28.5	40	60	1
35	40	60	1
A: 10 mM ammonium formate, pH 4<br>B: methanol			

Gradient 9			
Time (min)	%A	%B	Flow (mL/min)
0	40	60	1
15	40	60	1
20	10	90	1
28	10	90	1
28.5	40	60	1
35	40	60	1
A: 10 mM ammonium formate, pH 5.5<br>B: methanol			

Gradient 10			
Time (min)	%A	%B	Flow (mL/min)
0	55	45	1
15	55	45	1
20	10	90	1
28	10	90	1
28.5	55	45	1
35	55	45	1
A: H_2O 0.1% TFA<br>B: methanol 0.1% TFA			

Gradient 11			
Time (min)	%A	%B	Flow (mL/min)
0	40	60	1
15	40	60	1
20	10	90	1
28	10	90	1
28.5	40	60	1
35	40	60	1
A: H_2O 0.1% TEA<br>B: methanol			

Gradient 12			
Time (min)	%A	%B	Flow (mL/min)
0	40	60	1
15	40	60	1
20	10	90	1
28	10	90	1
28.5	40	60	1
35	40	60	1
A: H_2O 0.5% TEA<br>B: methanol			

Gradient 13			
Time (min)	%A	%B	Flow (mL/min)
0	0	100	1
15	0	100	1
B: methanol			
