# Supplementary material for: Fexinidazole – A New Oral Nitroimidazole Drug Candidate Entering Clinical Development for the Treatment of Sleeping Sickness
Source: PLoS Negl Trop Dis. 2010 Dec 21;4(12):e923. doi: 10.1371/journal.pntd.0000923 (PMC3006138; doi:10.1371/journal.pntd.0000923)
Supplement: Dataset S2 — (0.58 MB PDF) [file pntd.0000923.s003.pdf]

Fexinidazole: Evaluation of the *In Vitro* Intrinsic Clearance and Metabolism with Hepatocytes from African American Donors

Product Name:	Fexinidazole
Study Number:	0327-2008
Study Director:	
Sponsor Reference Study No.:	N.A.
Status:	FINAL

SUMMARY

Fexinidazole was incubated with cryopreserved hepatocytes from African American donors, at concentrations of 1 and 10 μM. The 1 μM samples were used for the intrinsic clearance determination, while the 10 μM samples were used for metabolite profile determination. The incubation samples were analyzed by LC-MS/MS.

The intrinsic clearance was calculated using the half-life approach; the half-life and the intrinsic clearance were determined from the concentration of fexinidazole remaining at the sampling time points.

The half-life of fexinidazole was 6.5 minutes, and the corresponding intrinsic clearance value was 257 mL/min/kg.

The metabolite profile of fexinidazole was investigated at t=0 and after 30 and 120 minutes incubation at the concentration of 10 μM.

The metabolism was rapid; unchanged fexinidazole accounted for 77% of the drug related material at t=0, and for 1% after 30 minutes incubation.

The main metabolite produced by hepatocytes from African American donors was fexinidazole sulfoxide. This metabolite was present as two enantiomers; the enantiomer with the shorter retention time was detected in a ratio of about 2:1, with respect to the enantiomer with the longer retention time.

Fexinidazole sulfone and fexinidazole sulfoxide des-methylated on the imidazole ring were minor metabolites, accounting for about 1%.

Both the intrinsic clearance and the metabolite profile of fexinidazole after incubation with hepatocytes from African American donors were similar to those previously observed with hepatocytes from Caucasian donors.

TABLE OF CONTENTS

1. ABBREVIATION AND DEFINITIONS OF TERMS 6
2. INTRODUCTION AND OBJECTIVES 6
3. STUDY SPONSOR 7
4. TEST FACILITY 7
5. REGULATORY REQUIREMENTS 7
6. SCHEDULE 7
7. MATERIALS AND METHODS 7
7.1. Test Item 7
7.2. Metabolites 8
7.3. Test System 8
7.4. Chemicals 8
7.5. *In Vitro* Incubations 8
7.5.1. Intrinsic Clearance Study 8
7.5.2. Metabolite Profile Study 9
7.6. Analysis of the Samples of Intrinsic Clearance Study 9
7.7. Intrinsic Clearance Determination 10
7.8. Analysis of the Samples of Metabolite Profile Study 10
7.8.1. Separation of Fexinidazole Sulfoxide Enantiomers with a Chiral Column 12
8. ARCHIVING 12
9. STUDY DEVIATIONS 12
10. STUDY PERSONNEL 12
11. RESULTS AND DISCUSSION 13
11.1. Phase I and Phase II Activities of Hepatocytes 13
11.2. Intrinsic Clearance Determination of Fexinidazole 13
11.3. Metabolite Profile and Identification 13
11.3.1. Metabolite Profiles with the Chiral Column 14
12. CONCLUSIONS 15
13. REFERENCE 15

TABLES

Table 1. Intrinsic clearance results for 7-ethoxycoumarin incubated at the concentration of 1 μM with hepatocytes from African American donors (1). 16

Table 2. Phase II activity of hepatocytes from African American donors by incubation of 7-hydroxycoumarin at the concentration of 30 μM (1)..16

Table 3. Intrinsic clearance results for fexinidazole incubated at the concentration of 1 μM with hepatocytes from African American donors (1). 16

Table 4. Metabolites found after 0, 30 and 120 minutes incubation of fexinidazole at the concentration of 10 μM with hepatocytes from African American donors. 16

FIGURES

Figure 1. Proposed metabolic pathway of fexinidazole after incubation at the concentration of 10 μM with hepatocytes from African American donors. 17

Figure 2. Extracted ion chromatograms of fexinidazole sulfoxide in a standard solution and after 30 and 120 minutes incubation of fexinidazole at the concentration of 10 μM with hepatocytes from African American donors. 18

APPENDICES

Appendix 1. Certificates of analysis of hepatocytes from African American donors used in this study. 19

Appendix 2. Peak area (counts) at different time points of fexinidazole incubated at the concentration of 1 μM with hepatocytes from African American donors. 24

1. ABBREVIATION AND DEFINITIONS OF TERMS

CID	Collision-induced decomposition
CL_{int}	Intrinsic clearance
Da	Dalton
DMSO	Dimethyl sulfoxide
7-ETC	7-Ethoxycoumarin
ESI	Electrospray ionisation
7-HC	7-Hydroxycoumarin
HPLC	High performance liquid chromatography
LC-MS/MS	Liquid chromatography-Mass spectrometry/Mass spectrometry
Km	Michaelis Menten constant
m/z	Mass to charge
MRM	Multiple reaction monitoring
MS	Mass spectrometry
MS/MS	Mass spectrometry/Mass spectrometry
PDA	Photodiode array
$t_{1/2}$	Half life
TIS	Turbo ion spray
TOF	Time of flight

2. INTRODUCTION AND OBJECTIVES

Fexinidazole is a 5-nitroimidazole derivative biologically active against Trypanosoma parasites (*T.b.rhodesiense* and *T.b. brucei*) under investigation in the treatment of the Human African Trypanosomiasis (HAT), known as sleeping sickness.

The intrinsic hepatic clearance and metabolite profile of fexinidazole has been previously investigated *in vitro* with hepatocytes of some animal species (mouse, rat, dog and cynomolgus monkey) and of Caucasian donors [1].

The purpose of this study (0327-2008) was to evaluate the intrinsic hepatic clearance and metabolite profile of fexinidazole with hepatocytes of African American donors.

The intrinsic hepatic clearance of fexinidazole was determined using the half-life approach, by measuring the substrate disappearance during 120 minutes incubation with hepatocytes of African American donors. LC-MS/MS was used for the detection of the compound during the incubation.

The metabolite profile and metabolite identification of fexinidazole was performed at a concentration of 10 μM of fexinidazole. The incubation samples were also analyzed on a chiral column.

3. STUDY SPONSOR

Drugs for Neglected Diseases *initiative* (DND*i*)
15 Chemin Louis-Dunant
CH-1202 Geneva
Switzerland

4. TEST FACILITY

Accelera

5. REGULATORY REQUIREMENTS

This study was conducted for exploratory purposes outside GLP regulations and was not audited by QA. Relevant Standard Operating Procedures of Accelera, Nerviano Medical Sciences, followed during the study were: MET-P/018/00; PCD-S-027; PCD-S-032; PCD-S-134; PCD-S-166.

6. SCHEDULE

Experimental Start Date	21 July 2008
Experimental Completion Date	09 September 2008

7. MATERIALS AND METHODS

7.1. Test Item

Generic Name	Fexinidazole
Chemical name	1H-Imidazole-1-methyl-2-[[4-(methylthio)phenoxy]methyl]-5-nitro
Chemical Structure	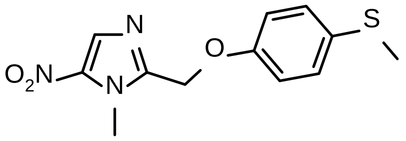
Molecular Formula	$C_{12}H_{13}N_3O_3S$
Molecular weight	279.31
Lot/Batch Number	3168-82-99/C
Purity	98.5% by $HClO_4$ assay
Expiry	September 2008
Storage Conditions	-20°C, light protection
Source Supplier	The compound, manufactured by Centipharm (France), was provided by the Sponsor

7.2. Metabolites

Fexinidazole sulfoxide, lot 1106-I-0170, 99.3% purity, and fexinidazole sulfone, lot 1106-I-0171, 99.9% purity, were provided by the Sponsor.

7.3. Test System

Cryopreserved hepatocytes of five African American single donors, lots EHI, REL, KSE, ZIJ and MRS, were purchased from In Vitro Technologies Inc (Baltimore, Maryland, USA). The certificates of analysis are reported in Appendix 1.

7.4. Chemicals

Reagents and solvents were of analytical grade and obtained from Sigma-Aldrich (St. Louis, Missouri, USA).

7.5. *In Vitro* Incubations

On the day of the experiment a 10 mM stock solution of fexinidazole in DMSO was prepared and, within 3 hours, aliquots of this freshly prepared solution, after suitable dilution, were added to the incubation matrix, to reach fexinidazole final concentrations of 1 and 10 μM. The final percentage of DMSO was 0.01% in the incubation samples for the intrinsic clearance determination and 0.1% in the incubation samples for metabolite profiling.

The cryopreserved hepatocytes were thawed according to the supplier's procedure and the number of viable cells was determined using the Trypan Blue exclusion method. Viabilities were 92% for lot EHI, 89% for lot REL, 82% for lot KSE, 86% for lot ZIJ and 86% for lot MRS in the intrinsic clearance study. In the metabolite profile study viabilities were 91% for lot EHI, 90% for lot REL, 85% for lot KSE, 84% for lot ZIJ and 87% for lot MRS. Afterwards, the same amount of viable cells for each donor were pooled and used for the incubations of fexinidazole.

7.5.1. Intrinsic Clearance Study

For the intrinsic clearance determination, fexinidazole was incubated at a concentration of 1 μM with pooled human hepatocytes (1 million cells/mL) in a final incubation volume of 1 mL Leibovitz L-15 medium, at 37°C. Incubations were performed in duplicate in a 48-well plate under shaking. Sampling was performed using an automatic liquid handling system (Multiprobe II EX, Packard). At 0, 1, 5, 10, 15, 20, 30, 60 and 120 minutes, 50 μL aliquots of the incubates were taken, then 80 μL of ice-cold acetonitrile and 20 μL of 1 μM warfarin in acetonitrile (internal standard) were added, and samples centrifuged at 2000 rpm for 20 min. The supernatant was analyzed by LC-MS/MS.

The chemical stability of fexinidazole was checked by incubating the compound at 37°C in the medium alone at t = 0 and 120 minutes.

For the determination of phase I and phase II activities of hepatocytes, 7-ethoxycoumarin (7-ETC) 1 µM, and 7-hydroxycoumarin (7-HC) 30 µM were used as positive controls and incubated under the same conditions as fexinidazole. Aliquots of incubates were taken at 0, 1, 5, 10, 15, 20, 30, 60 and 120 minutes and processed as fexinidazole samples.

7.5.2. Metabolite Profile Study

For metabolite profiling, fexinidazole was incubated at a concentration of 10 µM with pooled human hepatocytes (1 million cells/mL) in a final incubation volume of 2 mL Leibovitz L-15 medium, at 37°C. Aliquots of the incubation samples were taken at t = 0, 30 and 120 minutes; the metabolism was stopped by the addition of an equal volume of cold acetonitrile and the samples centrifuged at 1100 rpm for 20 min. The supernatant was stored at -20°C until analysis. Control incubations were performed with the compound in the medium alone for 120 minutes.

7.6. Analysis of the Samples of Intrinsic Clearance Study

The analysis of the samples for intrinsic clearance determination was performed by an LC-MS/MS method, using the following conditions:

**HPLC Equipment and Conditions**	
HPLC system	1100 binary pump (Agilent, Palo Alto, USA)
Autosampler	2777 (Waters)
Analytical column	Guard Column SB-C8 4.6 x 12.5 mm, 5 µm (Zorbax)
Column temperature	ambient
Mobile phase A	10 mM ammonium formate pH 4.0: acetonitrile (95:5, v:v)
Mobile phase B	10 mM ammonium formate pH 4.0: acetonitrile (5:95, v:v)
Injection volume	20 µL
Gradient conditions	Step / Total time (min) / flow (µL/min) / %A / % B<br>0 / 0.00 / 1500 / 100 / 0<br>1 / 0.00 / 1500 / 0 / 100<br>2 / 0.15 / 1500 / 0 / 100<br>3 / 0.20 / 600 / 0 / 100<br>4 / 1.00 / 600 / 0 / 100<br>5 / 1.35 / 600 / 100 / 0<br>6 / 1.60 / 1500 / 100 / 0
Valco Divert Valve	Step / Total time (min) / Position<br>1 / 0.30 / Waste<br>2 / 1.50 / Source<br>3 / 1.51 / Waste
**MS Equipment and Conditions**	

Mass spectrometer	API 4000 Triple Quadrupole (Applied Biosystems/MDS Sciex)
Source	Turbo Ion Spray (TIS)
Ion mode	Positive for fexinidazole and 7-ETC.<br>Negative for 7-HC, 7-HC sulphate and 7-HC glucuronide.<br>Warfarin was analysed both in positive and negative ion mode.
Scan mode	Multiple Reaction Monitoring (MRM)
MRM transitions	280.0 → 140.2 (fexinidazole)<br>191.0 → 163.1 (7-ETC)<br>161.0 → 133.1 (7-HC)<br>241.1 → 161.1 (7-HC sulphate)<br>336.9 → 161.1 (7-HC glucuronide)<br>309.3 → 163.0 (warfarin positive ion mode)<br>307.3 → 57.0 (warfarin negative ion mode)
Software	Analyst 1.4.1 (Applied Biosystems)

7.7. Intrinsic Clearance Determination

The intrinsic clearance (CL_{int}) of fexinidazole and of 7-ethoxycoumarin was calculated using the half-life approach. The half-life and the CL_{int} were determined from the concentration remaining at the different sampling points using the LC-MS/MS method. By plotting the natural logarithmic area of the compound remaining against the time, the slope was calculated by exponential regression analysis, and converted into the half-life ($t_{1/2}$) and CL_{int} expressed as μL/min/million cells and mL/min/kg.

7.8. Analysis of the Samples of Metabolite Profile Study

The Q-TOF 2 mass spectrometer was calibrated with a multi-point calibration in the range 70-1000 Da against the known accurate masses of the fragment ions that resulted from the collision-induced decomposition (CID) of [Glu^1]-fibrinopeptide B by continuous infusion at 10 μL/min of a 10 μg/mL solution in a mixture of 1% aqueous formic acid: acetonitrile (1:1, v:v). The tune parameters were those optimized in a previous study with fexinidazole [1]. In order to improve the accuracy of the mass measurements, during all analyses a 10 μg/mL solution of Met-Arg-Phe-Ala (m/z 524.2655) in 10 mM ammonium formate, pH 4.5: acetonitrile (1:1, v:v) was infused into the reference source of the LockSpray at a rate of 1-2 μL/min and a reference scan obtained every 10 seconds for automatic correction of the accurate masses. All data were acquired in centroid mode.

Before analysis, the incubation samples were evaporated to dryness under a stream of nitrogen (Turbovap), then the residues were reconstituted to the original incubation volumes with 10 mM ammonium formate, pH 4.5: acetonitrile 8:2, v:v.

The analyses were performed using an HPLC system on line with a photodiode array (PDA) detector and a mass spectrometer (MS). Equipment and conditions are given below.

**HPLC Equipment and Conditions**			
Autosampler	HTC Pal (CTC Analytics, San Jose, USA) equipped with a 100 µL sample loop and a 100 µL syringe		
Binary pump	1100 Series (Agilent, Palo Alto, CA, USA)		
Degasser	1100 Series (Agilent)		
Column oven	1100 Series (Agilent)		
Column	XBridge C8, 2.1 x 150 mm, 3.5 µm (Waters)		
Guard column	C8, 2 x 4 mm (Phenomenex)		
Column temperature	40°C		
Mobile phase A	10 mM ammonium formate, pH 4.5		
Mobile phase B	Acetonitrile		
Flow rate	0.3 mL/min		
Run time	35 minutes		
Injection volume	75 µL		
Gradient conditions	Time (min)	Solvent A (%)	Solvent B (%)
	0.0	95	5
	1.0	95	5
	4.0	75	25
	21.0	40	60
	21.5	5	95
	27.0	5	95
	27.5	95	5
	35.0	95	5
**PDA Detector and Conditions**			
Diode Array detector	1100 Series (Agilent)		
Range	190 to 600 nm		
Resolution	2 nm		
Width	0.1 min		
**MS Acquisition Conditions**			
Acquisition software	Masslynx 4.1 (Waters)		
Processing software	Metabolynx 4.1 (Waters)		
Scan mode	MS Full Scan and MS/MS Full Scan		
Acquisition polarity	Positive		
Acquisition mode	Centroid		
Acquisition time	27 minutes		
Scan range	75 to 750 Da (MS Full Scan); 50 to 750 Da (MS/MS)		
Scan time	0.9 sec		
Interscan delay	0.1 sec		
Capillary voltage	3 kV		

Cone voltage	25 eV
Collision energy	10 eV (MS Full Scan); 25 eV (MS/MS)
Extractor voltage	2 eV
Rf Lens	1.5 eV
Ion energy	1.5 eV
Source temperature	120°C
Desolvation gas temperature	250°C

The incubation samples were analyzed in MS positive ion mode and the data searched on the accurate masses of possible metabolites using the Metabolynx software, version 4.1 (Waters), using a mass window of 40 mDa. Samples were then re-run in MS/MS mode by selecting the masses of possible metabolites. The collision energy used to obtain MS/MS data was 25 eV.

7.8.1. Separation of Fexinidazole Sulfoxide Enantiomers with a Chiral Column

The separation was performed using the equipment described in 7.8, a chiral HPLC column Chirobiotic TAG 4.6 x 250 mm, 5 μm (Astec) and methanol as mobile phase, with isocratic elution at a flow rate of 1 mL/min.

8. ARCHIVING

The original protocol, all raw data and supporting documents produced at the Test Facility, and the final report with original signatures were filed in the Archives of Accelera, Nerviano Medical Sciences S.r.l., Nerviano (Italy) for the period of time agreed with the Sponsor (at least 3 years) after which the Sponsor will be contacted for instructions regarding dispatch or disposal of the material.

A copy of the protocol, the report with original signatures and all relevant original documentation of the test item were filed by the Sponsor.

9. STUDY DEVIATIONS

No protocol deviations were observed during the study.

10. STUDY PERSONNEL

11. RESULTS AND DISCUSSION

11.1. Phase I and Phase II Activities of Hepatocytes

The hepatocytes used in this study were checked for their activities towards phase I and phase II reactions, using 7-ETC and 7-HC. Table 1 shows the disappearance half-life and intrinsic clearance of 7-ETC incubated at the concentration of 1 μM with hepatocytes. Table 2 shows the formation of 7-HC sulphate and 7-HC glucuronide from 7-HC incubated at the concentration of 30 μM with hepatocytes. The results confirmed that the hepatocytes used in this study exhibited active metabolism.

11.2. Intrinsic Clearance Determination of Fexinidazole

The intrinsic clearance of fexinidazole was determined at the concentration of 1 μM in pooled hepatocytes of five African American donors, using the half-life approach. The starting concentration of 1 μM was assumed to be << of Km.

The half-life of fexinidazole was 6.5 minutes, and the corresponding intrinsic clearance value was 257 mL/min/kg (Table 3).

The peak area (counts) of fexinidazole at the different time points are shown in Appendix 2.

Negative controls with fexinidazole incubated for 120 minutes at 37°C in the incubation medium alone show about 40% loss of fexinidazole.

11.3. Metabolite Profile and Identification

The metabolite profile of fexinidazole was determined at the concentration of 10 μM at t=0 and after 30 and 120 minutes incubation.

The chromatographic separation of fexinidazole and metabolites was obtained using a reverse phase HPLC column XBridge C8, 2.1 x 150 mm, 3.5 μm (Waters), under the same chromatographic conditions as in the previous study, when fexinidazole was incubated with hepatocytes of animal species (mouse, rat, dog and cynomolgus monkey) and of Caucasian donors [1].

The identity of metabolites was proposed based on their accurate mass (using LockSpray correction), with an accuracy of +/-5 mDa. MS/MS analyses were performed to verify that peaks were drug-related and to obtain structural information from the fragment ions formed. The identity of fexinidazole sulfoxide and fexinidazole sulfone in the incubation samples was also confirmed by comparing the retention times of the two metabolites with those of the authentic standard compounds.

The approximate relative amounts were determined from the absolute areas taken from a selected ion chromatogram at the metabolite masses with a mass window of 0.04 Da. The

absolute areas of fexinidazole, fexinidazole sulfoxide and fexinidazole sulfone were normalized based on their relative MS response (factors 1.3, 1 and 8 for fexinidazole, fexinidazole sulfoxide and fexinidazole sulfone, respectively). Nevertheless, these results should be regarded as semi-quantitative (approximate relative amounts of fexinidazole and each metabolite in the samples).

The proposed metabolic pathway of fexinidazole is shown in Figure 1.

The relative amounts of fexinidazole and metabolites detected at t=0, after 30 and 120 minutes incubation with hepatocytes of African American donors are reported in Table 4.

Fexinidazole was rapidly metabolized, as shown by the half-life of the compound.

At **t=0**, unchanged fexinidazole accounted for 77% of total drug related material; the remaining drug related material was due to the sulfoxide **M1**. Virtually t=0 is approximately 30 seconds, just the time to add fexinidazole to hepatocytes, shake, take an aliquot and stop the metabolism by addition of acetonitrile. This explains the amount of M1 present at this incubation time.

After **30 minutes** incubation, fexinidazole was detected in amounts of 1%. The main component (98%) was M1. Metabolite **M3** (m/z = 282.06), corresponding to M1 des-methylated on the imidazole ring, was detected in traces (less 1%).

After **120 minutes** incubation, M1 accounted for 98%. Traces of the sulfone metabolite **M2** (1%) and of metabolite M3 were also detected.

After 120 minutes incubation in the Leibovitz medium alone (**control sample**), fexinidazole accounted for about 82% of the total drug related material.

The lower loss of fexinidazole in the metabolite profile control sample (18%) compared to the clearance control sample (about 40%) could be explained by the different concentration of fexinidazole used: higher (10 μM) in the metabolite profile study and lower (1 μM) in the clearance study.

11.3.1. Metabolite Profiles with the Chiral Column

A standard solution of fexinidazole sulfoxide and the samples after 30 and 120 minutes incubation with hepatocytes were analyzed on a chiral HPLC column for the chromatographic separation of the two enantiomers of fexinidazole sulfoxide. After both 30 and 120 minutes incubation, the enantiomer with the shorter retention time was detected in a ratio of about 2:1, with respect to the enantiomer with the longer retention time, while in the standard solution the ratio was 1:1. The extracted ion chromatograms of fexinidazole sulfoxide are shown in Figure 2.

12. CONCLUSIONS

As previously observed with hepatocytes from Caucasian donors, fexinidazole showed a rapid and high intrinsic clearance with hepatocytes from African American donors. In agreement with the above, unchanged fexinidazole was detected in small amounts (1%) only after 30 minutes incubation. Fexinidazole sulfoxide was the main metabolite produced by human hepatocytes; this metabolite was present as two enantiomers in a ratio of about 2:1. Small amounts of fexinidazole sulfone and of the sulfoxide des-methylated on the imidazole ring were also detected.

13. REFERENCE

1. Fexinidazole: Evaluation of the *in vitro* cross species intrinsic clearance and metabolism with mouse, rat, dog, monkey and human hepatocytes. Accelera Study Report 0141-2007, November 2007.

TABLES AND FIGURES

Table 1. Intrinsic clearance results for 7-ethoxycoumarin incubated at the concentration of 1 μM with hepatocytes from African American donors [(1)].

Species	$t_{1/2}$ (min)		CL_{int} *in vitro* (μL/min/million cells)		CL_{int} *in vitro* (mL/min/kg)	
	clearance study	met profile study	clearance study	met profile study	clearance study	met profile study
Human	6.4	6.2	108	111	258	267

(1): Mean values of two determinations

Table 2. Phase II activity of hepatocytes from African American donors by incubation of 7-hydroxycoumarin at the concentration of 30 μM [(1)].

Species	7-HC sulphate (pmol/min/ million cells)		7-HC glucuronide (pmol/min/ million cells)	
	clearance study	met profile study	clearance study	met profile study
Human	57	38	372	86

(1): Mean values of two determinations

Table 3. Intrinsic clearance results for fexinidazole incubated at the concentration of 1 μM with hepatocytes from African American donors [(1)].

Species	$t_{1/2}$ (min)	CL_{int} *in vitro* (μL/min/million cells)	CL_{int} *in vitro* (mL/min/kg)
Human	6.5	107	257

(1): Mean values of two determinations

Table 4. Metabolites found after 0, 30 and 120 minutes incubation of fexinidazole at the concentration of 10 μM with hepatocytes from African American donors.

Metabolite	Molecular formula	m/z	RT (min)	% of total drug related material[(1)]			
				t=0	30’	120’	BLK
P	$C_{12}H_{13}N_3O_3S$	280.08	16.1	77	1		82
M1	$C_{12}H_{13}N_3O_4S$	296.07	9.0	23	98	98	18
M2	$C_{12}H_{13}N_3O_5S$	312.07	10.5			1	
M3	$C_{11}H_{11}N_3O_4S$	282.06	7.5		<1	<1	

(1) The approximate relative amounts were determined from the absolute areas taken from a selected ion chromatogram at the metabolite masses with a mass window of 0.04 Da. The absolute areas of fexinidazole, M1 and M2 were normalized based on their relative MS response. Nevertheless, these results should be regarded as semi-quantitative.

Figure 1. Proposed metabolic pathway of fexinidazole after incubation at the concentration of 10 μM with hepatocytes from African American donors.

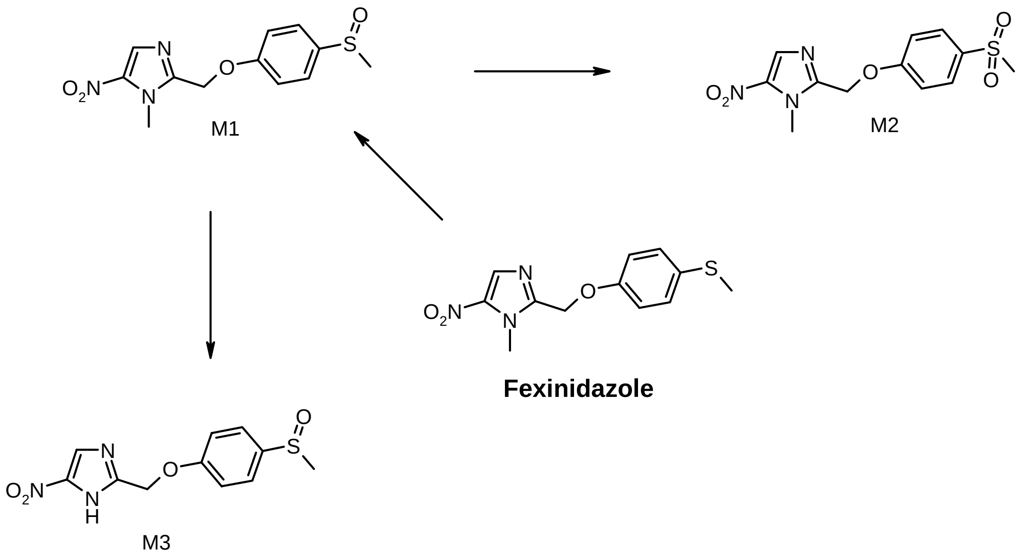

Figure 2. Extracted ion chromatograms of fexinidazole sulfoxide in a standard solution and after 30 and 120 minutes incubation of fexinidazole at the concentration of 10 μM with hepatocytes from African American donors.

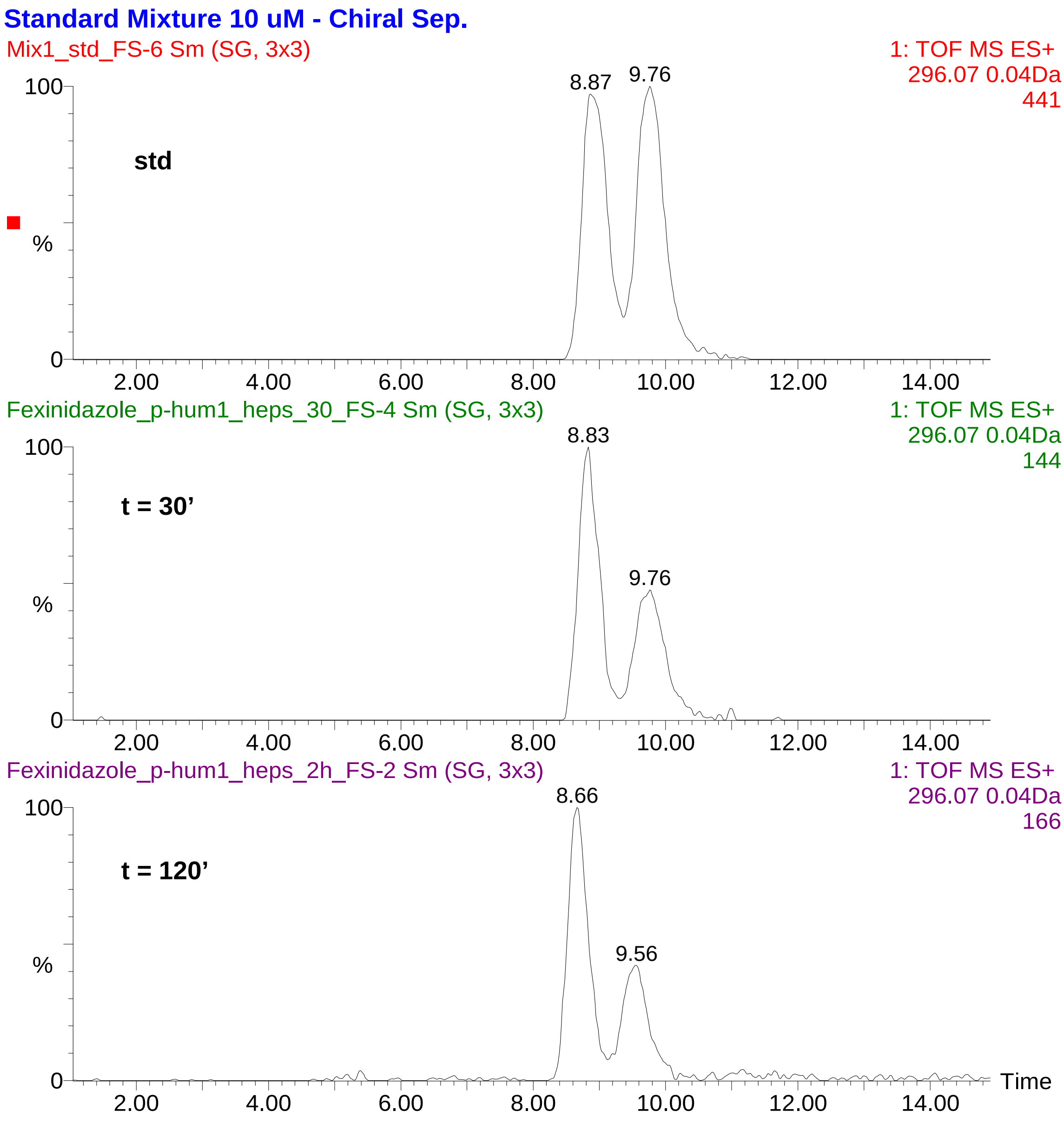

APPENDICES

Appendix 1. Certificates of analysis of hepatocytes from African American donors used in this study.

Certificate of Analysis

Product Number: F00995
Product: Cryopreserved Hepatocytes Human, Female
Quantity: 5 million
Lot Number: EHI
storage condition: below -150° C

IN VITRO TECHNOLOGIES

Relevant Donor Demographics, **as reported to In Vitro Technologies

Age: 56 **Race:** African American
Cause of Death: ICB **BMI:** 35.4

Social History: Alcohol Use: Yes; Narcotic use: (none reported); Cannabinoid use: (none reported); Tobacco use: Yes

Relevant Medical History:

- [x] Diabetes
- [x] Hypertension
- [ ] High cholestrol
- [ ] Cancer (specify below)
- [ ] Depression/Anxiety
- [ ] Asthma
- [ ] Arthritis
- [ ] Other Autoimmune disease
- [ ] Hypothyroidism
- [ ] Kidney disease
- [ ] Gastrointestinal disease
- [ ] Congestive heart failure
- [x] Vascular disease
- [ ] Other heart disease

Relevant Chronic Medications:

- [ ] Hormone replace therapy
- [ ] Anti-hypertensives
- [ ] Lipid-lowering agents
- [ ] Insulin
- [ ] Anti-inflammitories
- [ ] Anti-depressants/Anxiolytic

Specification:		Result:		
1) Serology testing:	CMV	POSITIVE	Hepatitis B	NEGATIVE
	HIV	NEGATIVE	Hepatitis C	NEGATIVE
2) ≥ 5 million cells with at least 70% post-thaw viability as determined by Trypan blue exclusion:		PASS		

Lot Characterization Results

COUM: total rate of formation of 7-hydroxycoumarin	88	pmole/10^6 cells/min
DEX: rate of formation of dextrorphan	3	pmole/10^6 cells/min
ECOD: Total rate of formation of 7-hydroxycoumarin	56	pmole/10^6 cells/min
7-HCG: rate of formation of 7-hydroxycoumarin glucuronide	291	pmole/10^6 cells/min
7-HCS: rate of formation of 7-hydroxycoumarin sulfate	45	pmole/10^6 cells/min
MEPH: rate of formation of 4'-hydroxymephenytoin	1	pmole/10^6 cells/min
TEST: rate of formation of 6β-hydroxytestosterone	248	pmole/10^6 cells/min
TOLB: rate of formation of 4'-methylhydroxytolbutamide	21	pmole/10^6 cells/min
PHEN: rate of formation of acetaminophen	45	pmole/10^6 cells/min
CZX: rate of formation of 6-hydroxychlorzoxazone	43	pmole/10^6 cells/min
NAT1	2.39	nmol/mg/min
NAT2	0.234	nmol/mg/min
NAT1 genotype	*4/*10	
NAT2 genotype	*6/*14	
2C19 genotype		
2D6 genotype		
2C9 genotype		

Biohazard Warning: *These products have been made using human donor tissue and should be considered as potential biohazards. Although the original tissue tested negative for HIV and Hepatitis B and C, caution is advised when handling these products. These products are for research use only and should not be used in animals or humans. These cells have not been approved for any diagnostic or clinical procedures.*

**Donor information is pr[illegible] cannot be confirmed or further substantiated in order to maintain donor confidentiality.

This product has been tested by controlled procedures and conforms to all specifications.

1450 S. Rolling Rd., Baltimore, MD 21227 · Toll Free 888.488.3232 · fax 410.455.1245 · www.invitrotech.com · info@invitrotech.com

CA000 [illegible] date 19 September 2006

Certificate of Analysis

Product Number: F00995
Product: Cryopreserved Hepatocytes Human, Female
Quantity: 5 million
Lot Number: REL
storage condition: below -150° C

VITRO TECHNOLOGIES

Relevant Donor Demographics, **as reported to In Vitro Technologies

Age: 60 **Race:** African American
Cause of Death: ICB **BMI:** 31.6

Social History: Alcohol Use: Yes; Cannabinoid use: (none reported); Narcotic use: (none reported); Tobacco use: Yes

Relevant Medical History:

☐ Diabetes ☐ Depression/Anxiety ☐ Hypothyroidism ☐ Congestive heart failure
☐ Hypertension ☐ Asthma ☐ Kidney disease ☐ Vascular disease
☐ High cholestrol ☐ Arthritis ☐ Gastrointestinal disease ☐ Other heart disease
☐ Cancer (specify below) ☐ Other Autoimmune disease

Relevant Chronic Medications:

☐ Hormone replace therapy ☐ Lipid-lowering agents ☐ Anti-inflammitories
☐ Anti-hypertensives ☐ Insulin ☐ Anti-depressants/Anxiolytic

Specification: **Result:**

1) Serology testing: CMV **NEGATIVE**; HIV **NEGATIVE**; Hepatitis B **NEGATIVE**; Hepatitis C **NEGATIVE**

2) ≥ 5.0 million cells with at least 70% post-thaw viability as determined by Trypan blue exclusion: **PASS**

Lot Characterization Results

COUM: total rate of formation of 7-hydroxycoumarin	40	pmole/10^6 cells/min
DEX: rate of formation of dextrorphan	20	pmole/10^6 cells/min
ECOD: Total rate of formation of 7-hydroxycoumarin	15	pmole/10^6 cells/min
7-HCG: rate of formation of 7-hydroxycoumarin glucuronide	109	pmole/10^6 cells/min
7-HGS: rate of formation of 7-hydroxycoumarin sulfate	9	pmole/10^6 cells/min
MEPH: rate of formation of 4'-hydroxymephenytoin	65	pmole/10^6 cells/min
TEST: rate of formation of 6ß-hydroxytestosterone	100	pmole/10^6 cells/min
TOLB: rate of formation of 4'-methylhydroxytolbutamide	33	pmole/10^6 cells/min
PHEN: rate of formation of acetaminophen	75	pmole/10^6 cells/min
CZX: rate of formation of 6-hydroxychlorzoxazone	10	pmole/10^6 cells/min
NAT1	4.51	nmol/mg/min
NAT2	0.268	nmol/mg/min
NAT1 genotype	*4/*10	
NAT2 genotype	wt/*5	
2C19 genotype	wt/wt	
2D6 genotype	wt/*4	
2C9 genotype	wt/wt	

Biohazard Warning: *These products have been made using human donor tissue and should be considered as potential biohazards. Although the original tissue tested negative for HIV and Hepatitis B and C, caution is advised when handling these products. These products are for research use only and should not be used in animals or humans. These cells have not been approved for any diagnostic or clinical procedures.*

***Donor information is provided as a reference and cannot be confirmed or further substantiated in order to maintain donor confidentiality.*

[signature]

This product has been tested by controlled procedures and conforms to all specifications.

1450 S. Rolling Rd., Baltimore, MD 21227 · Toll Free 888.488.3232 · fax 410.455.1245 · www.invitrotech.com · info@invitrotech.com

CA003 eff. date 06 August 2004

Certificate of Analysis

**Product Number:**	M00995
**Product:**	Cryopreserved Hepatocytes Human, Male
**Quantity:**	5 million
**Lot Number:**	KSE
**storage condition:**	below -150° C

IN VITRO TECHNOLOGIES

Relevant Donor Demographics, **as reported to In Vitro Technologies

**Age:**	79	**Race:**	African American
**Cause of Death:**	CVA	**BMI:**	32.5

Social History:
Alcohol Use: (none reported)
Narcotic use: (none reported)
Cannabinoid use: (none reported)
Tobacco use: Yes

Relevant Medical History:

- ☐ Diabetes
- ☑ Hypertension
- ☐ High cholestrol
- ☑ Cancer (specify below): Breast
- ☐ Depression/Anxiety
- ☐ Asthma
- ☐ Arthritis
- ☐ Other Autoimmune disease
- ☐ Hypothyroidism
- ☐ Kidney disease
- ☐ Gastrointestinal disease
- ☐ Congestive heart failure
- ☑ Vascular disease
- ☐ Other heart disease

Relevant Chronic Medications:

- ☐ Hormone replace therapy
- ☑ Anti-hypertensives
- ☐ Lipid-lowering agents
- ☐ Insulin
- ☐ Anti-inflammitories
- ☐ Anti-depressants/Anxiolytic

**Specification:**		**Result:**		
1) Serology testing:	CMV	**POSITIVE**	Hepatitis B	**NEGATIVE**
	HIV	**NEGATIVE**	Hepatitis C	**NEGATIVE**
2) ≥ 5.0 million cells with at least 70% post-thaw viability as determined by Trypan blue exclusion:		**PASS**		

Lot Characterization Results

COUM: total rate of formation of 7-hydroxycoumarin	65	pmole/10^6 cells/min
DEX: rate of formation of dextrorphan	27	pmole/10^6 cells/min
ECOD: Total rate of formation of 7-hydroxycoumarin	52	pmole/10^6 cells/min
7-HCG: rate of formation of 7-hydroxycoumarin glucuronide	206	pmole/10^6 cells/min
7-HGS: rate of formation of 7-hydroxycoumarin sulfate	74	pmole/10^6 cells/min
MEPH: rate of formation of 4'-hydroxymephenytoin	21	pmole/10^6 cells/min
TEST: rate of formation of 6ß-hydroxytestosterone	123	pmole/10^6 cells/min
TOLB: rate of formation of 4'-methylhydroxytolbutamide	42	pmole/10^6 cells/min
PHEN: rate of formation of acetaminophen	93	pmole/10^6 cells/min
CZX: rate of formation of 6-hydroxychlorzoxazone	16	pmole/10^6 cells/min
NAT1	4.82	nmol/mg/min
NAT2	0.066	nmol/mg/min
NAT1 genotype	*4/*10	
NAT2 genotype	*6/*7	
2C19 genotype	wt/wt	
2D6 genotype	wt/wt	
2C9 genotype	wt/wt	

Biohazard Warning: *These products have been made using human donor tissue and should be considered as potential biohazards. Although the original tissue tested negative for HIV and Hepatitis B and C, caution is advised when handling these products. These products are for research use only and should not be used in animals or humans. These cells have not been approved for any diagnostic or clinical procedures.*

***Donor information is provided as a reference and cannot be confirmed or further substantiated in order to maintain donor confidentiality.*

[signature]

This product has been tested by controlled procedures and conforms to all specifications.

1450 S. Rolling Rd., Baltimore, MD 21227 · Toll Free 888.488.3232 · fax 410.455.1245 · www.invitrotech.com · info@invitrotech.com

CA003 eff. date 06 August 2004

Certificate of Analysis

Product Number: M00995
Product: Cryopreserved Hepatocytes Human, Male
Quantity: 5 million
Lot Number: ZIJ
storage condition: below -150° C

VITRO TECHNOLOGIES

Relevant Donor Demographics, **as reported to In Vitro Technologies

Age: 22 **Race:** African American
Cause of Death: HT **BMI:** 25.1

Social History: Alcohol Use: (none reported) Cannabinoid use: Marijuana
Narcotic use: (none reported) Tobacco use: (none reported)

Relevant Medical History:

☐ Diabetes ☐ Depression/Anxiety ☐ Hypothyroidism ☐ Congestive heart failure
☐ Hypertension ☐ Asthma ☐ Kidney disease ☐ Vascular disease
☐ High cholestrol ☐ Arthritis ☐ Gastrointestinal disease ☐ Other heart disease
☐ Cancer (specify below) ☐ Other Autoimmune disease

Relevant Chronic Medications:

☐ Hormone replace therapy ☐ Lipid-lowering agents ☐ Anti-inflammitories
☐ Anti-hypertensives ☐ Insulin ☐ Anti-depressants/Anxiolytic

Specification: **Result:**

1) Serology testing: CMV **NEGATIVE** Hepatitis B **NEGATIVE**
HIV **NEGATIVE** Hepatitis C **NEGATIVE**

2) ≥ 5.0 million cells with at least 70% post-thaw viability as determined by Trypan blue exclusion: **PASS**

Lot Characterization Results

COUM: total rate of formation of 7-hydroxycoumarin	6	pmole/10^6 cells/min
DEX: rate of formation of dextrorphan	33	pmole/10^6 cells/min
ECOD: Total rate of formation of 7-hydroxycoumarin	31	pmole/10^6 cells/min
7-HCG: rate of formation of 7-hydroxycoumarin glucuronide	320	pmole/10^6 cells/min
7-HGS: rate of formation of 7-hydroxycoumarin sulfate	29	pmole/10^6 cells/min
MEPH: rate of formation of 4'-hydroxymephenytoin	13	pmole/10^6 cells/min
TEST: rate of formation of 6β-hydroxytestosterone	25	pmole/10^6 cells/min
TOLB: rate of formation of 4'-methylhydroxytolbutamide	34	pmole/10^6 cells/min
PHEN: rate of formation of acetaminophen	3	pmole/10^6 cells/min
CZX: rate of formation of 6-hydroxychlorzoxazone	13	pmole/10^6 cells/min
NAT1	7.28	nmol/mg/min
NAT2	0.166	nmol/mg/min
NAT1 genotype	*4/*4	
NAT2 genotype	*5/*6	
2C19 genotype	wt/wt	
2D6 genotype	wt/wt	
2C9 genotype	wt/wt	

Biohazard Warning: *These products have been made using human donor tissue and should be considered as potential biohazards. Although the original tissue tested negative for HIV and Hepatitis B and C, caution is advised when handling these products. These products are for research use only and should not be used in animals or humans. These cells have not been approved for any diagnostic or clinical procedures.*

***Donor information is provided as a reference and cannot be confirmed or further substantiated in order to maintain donor confidentiality.*

[signature]

This product has been tested by controlled procedures and conforms to all specifications.

1450 S. Rolling Rd., Baltimore, MD 21227 · Toll Free 888.488.3232 · fax 410.455.1245 · www.invitrotech.com · info@invitrotech.com

CA003 eff. date 05 August 2004

Certificate of Analysis

Product Number: M00995
Product: Cryopreserved Hepatocytes Human, Male
Quantity: 5 million
Lot Number: MRS
storage condition: below -150° C

VITRO TECHNOLOGIES

Relevant Donor Demographics, **as reported to In Vitro Technologies

Age: 42 **Race:** African American
Cause of Death: ICH **BMI:** 21.3

Social History: Alcohol Use: Yes; Narcotic use: Cocaine; Cannabinoid use: Marijuana; Tobacco use: Yes

Relevant Medical History:

☐ Diabetes ☐ Depression/Anxiety ☐ Hypothyroidism ☐ Congestive heart failure
☐ Hypertension ☐ Asthma ☐ Kidney disease ☐ Vascular disease
☐ High cholestrol ☐ Arthritis ☐ Gastrointestinal disease ☐ Other heart disease
☐ Cancer (specify below) ☐ Other Autoimmune disease

Relevant Chronic Medications:

☐ Hormone replace therapy ☐ Lipid-lowering agents ☐ Anti-inflammitories
☐ Anti-hypertensives ☐ Insulin ☐ Anti-depressants/Anxiolytic

Specification: Result:

1) Serology testing: CMV **NEGATIVE**; HIV **NEGATIVE**; Hepatitis B **NEGATIVE**; Hepatitis C **NEGATIVE**

2) ≥ 5.0 million cells with at least 70% post-thaw viability as determined by Trypan blue exclusion: **PASS**

Lot Characterization Results

COUM: total rate of formation of 7-hydroxycoumarin	119	pmole/10^6 cells/min
DEX: rate of formation of dextrorphan	21	pmole/10^6 cells/min
ECOD: Total rate of formation of 7-hydroxycoumarin	110	pmole/10^6 cells/min
7-HCG: rate of formation of 7-hydroxycoumarin glucuronide	450	pmole/10^6 cells/min
7-HGS: rate of formation of 7-hydroxycoumarin sulfate	50	pmole/10^6 cells/min
MEPH: rate of formation of 4'-hydroxymephenytoin	2	pmole/10^6 cells/min
TEST: rate of formation of 6ß-hydroxytestosterone	675	pmole/10^6 cells/min
TOLB: rate of formation of 4'-methylhydroxytolbutamide	54	pmole/10^6 cells/min
PHEN: rate of formation of acetaminophen	68	pmole/10^6 cells/min
CZX: rate of formation of 6-hydroxychlorzoxazone	28	pmole/10^6 cells/min
NAT1	TBD	nmol/mg/min
NAT2	TBD	nmol/mg/min
NAT1 genotype	TBD	
NAT2 genotype	TBD	
2C19 genotype	TBD	
2D6 genotype	TBD	
2C9 genotype	TBD	

Biohazard Warning: *These products have been made using human donor tissue and should be considered as potential biohazards. Although the original tissue tested negative for HIV and Hepatitis B and C, caution is advised when handling these products. These products are for research use only and should not be used in animals or humans. These cells have not been approved for any diagnostic or clinical procedures.*

***Donor information is provided as a reference and cannot be confirmed or further substantiated in order to maintain donor confidentiality.*

This product has been tested by controlled procedures and conforms to all specifications.

1450 S. Rolling Rd., Baltimore, MD 21227 · Toll Free 888.488.3232 · fax 410.455.1245 · www.invitrotech.com · info@invitrotech.com

CA003 eff. date 05 August 2004

Appendix 2. Peak area (counts) at different time points of fexinidazole incubated at the concentration of 1 μM with hepatocytes from African American donors.

Exp	t=0	t=1’	t=5’	t=10’	t=15’	t=20’	t=30’	t=60’	t=120’
1	814000	685000	457000	255000	158000	98900	24800	1400	nd
2	802000	678000	515000	308000	152000	78500	25100	1380	nd

nd: not detectable
