# Supplementary material for: Fexinidazole – A New Oral Nitroimidazole Drug Candidate Entering Clinical Development for the Treatment of Sleeping Sickness
Source: PLoS Negl Trop Dis. 2010 Dec 21;4(12):e923. doi: 10.1371/journal.pntd.0000923 (PMC3006138; doi:10.1371/journal.pntd.0000923)
Supplement: Dataset S3 — (0.86 MB PDF) [file pntd.0000923.s004.pdf]

STUDY NUMBER 14925
FINAL REPORT

ADME-Tox

- Study of Compounds Fexinidazole, Fexinidazole sulfone and Fexindazole sulfoxyde -

Cerep

Cerep

Ref.: Final Report 14925/ND

STUDY NUMBER 14925

ADME-Tox
- Study of Compounds Fexinidazole, Fexinidazole sulfone and Fexindazole sulfoxyde -

Study Sponsor: DNDI FOUNDATION

Attention: Dr. Els TORREELE
Senior Project Manager

Address: 15 chemin Louis Dunant
CH-1202 GENEVA
SWITZERLAND

Study Director:

Testing Facility: Cerep

Study Period: From August 04, 2008 to August 13, 2008

Report Version: 1

Report Date: August 26, 2008

CONTENTS

1. PURPOSE OF THE STUDY **4**

2. MATERIALS AND METHODS **5**

2.1. ADME-Tox: Bioanalytical 5

2.1.1. General Procedures 5

2.1.2. Experimental Conditions 5

2.1.3. Analysis and Expression of Results 5

2.2. ADME-Tox: *In Vitro* Metabolism 6

2.2.1. General Procedures 6

2.2.2. Experimental Conditions 6

2.2.3. Analysis and Expression of Results 7

3. COMPOUNDS **8**

3.1. Test Compounds 8

3.2. Reference Compounds 8

4. RESULTS **9**

4.1. ADME-Tox: Bioanalytical 9

4.2. ADME-Tox: *In Vitro* Metabolism 11

5. BIBLIOGRAPHY **15**

6. DATA AND RECORDS STORAGE **16**

7. QUALITY ASSURANCE STATEMENT **17**

1. PURPOSE OF THE STUDY

The purpose of this study was to test compounds Fexinidazole, Fexinidazole sulfone and Fexinidazole sulfoxyde in various metabolic stability assays.

2. MATERIALS AND METHODS

2.1. ADME-Tox: Bioanalytical

2.1.1. General Procedures

Assay	Technique	Additional Information
HPLC-MS Screen	HPLC-MS and HPLC-MS/MS	Full scan and product ion spectra; SRM conditions for quantitation, and ionization potential

2.1.2. Experimental Conditions

Assay	Test Compound	Condition	Analytical method
HPLC-MS Screen	200 µM (n=2) acetonitrile/methanol/water (25/25/50, v/v/v)	Full-scan analysis followed by tandem MS	HPLC-MS and HPLC-MS/MS

Abbreviations:
HPLC-MS/MS: HPLC coupled with tandem mass spectrometry (Instrumentation: Thermo Finnigan)
HPLC-MS: HPLC with mass spectrometry detection (Instrumentation: Thermo Finnigan)
HPLC: High performance liquid chromatography
SRM: Selected reaction monitoring

2.1.3. Analysis and Expression of Results

HPLC-MS Screen

Full scan HPLC-MS analysis was conducted on the test compound at 200 µM. Total ion current chromatograms and corresponding mass spectra were generated for each test compound in both positive and negative ionization modes. The precursor ions for MS/MS were selected from either the positive or the negative mass spectrum, as a function of the respective ion abundance. In addition, product ion HPLC-MS/MS analysis was performed in order to determine the appropriate selected fragmentation reaction for use in quantitative analysis. The final reaction monitoring parameters were chosen to maximize the possibility for quantitation of the test compound when present within a complex mixture of components. Finally, each test compound was assigned a rank number of ionization, which directly indicates its ease of quantitation.

2.2. ADME-Tox: *In Vitro* Metabolism

2.2.1. General Procedures

Assay	Source	Reference Compound	Bibliography
Metabolic Stability (CYP1A2)	Human recombinant (40 pmol/mL)	propranolol, ethoxyresorufin	Suzuki et al. (1999)
Metabolic Stability (CYP2B6)	Human recombinant (10 pmol/mL)	propranolol, benzphetamine	Suzuki et al. (1999)
Metabolic Stability (CYP2C8)	Human recombinant (50 pmol/mL)	propranolol, paclitaxel	Suzuki et al. (1999)
Metabolic Stability (CYP2C9)	Human recombinant (10 pmol/mL)	propranolol, diclofenac	Suzuki et al. (1999)
Metabolic Stability (CYP2C19)	Human recombinant (10 pmol/mL)	propranolol, promazine	Suzuki et al. (1999)
Metabolic Stability (CYP2D6)	Human recombinant (10 pmol/mL)	propranolol, terfenadine	Suzuki et al. (1999)
Metabolic Stability (CYP3A4)	Human recombinant (10 pmol/mL)	propranolol, terfenadine	Suzuki et al. (1999)
Metabolic Stability (CYP3A5)	Human recombinant (10 pmol/mL)	propranolol, terfenadine	Suzuki et al. (1999)

Notes:
CYP1A2: from PanVera, catalog number P2792.
CYP2B6: from BD Gentest, catalog number 456255.
CYP2C8: from BD Gentest, catalog number 456252.
CYP2C9: from Panvera, catalog number P2378.
CYP2C19: from PanVera, catalog number P2570.
CYP2D6: from BD Gentest, catalog number 456217.
CYP3A4: from PanVera, catalog number P2377.
CYP3A5: from PanVera, catalog number P2512.

2.2.2. Experimental Conditions

Assay	Substrate / Cofactor	Incubation	Detected Component	Analytical Method
Metabolic Stability (CYP1A2)	Test compound (1 µM), NADP (1.3 mM), G6P (3.3 mM), G6PDHase (0.4 U/ml) (n=2)	0 and 60 min, 37 °C Phosphate buffer, pH 7.4	Product ion corresponding to the test compound via SRM	HPLC-MS/MS
Metabolic Stability (CYP2B6)	Test compound (1 µM), NADP (1.3 mM), G6P (3.3 mM), G6PDHase (0.4 U/mL) (n=2)	0 and 60 min, 37 °C Phosphate buffer, pH 7.4	Product ion corresponding to the test compound via SRM	HPLC-MS/MS

Assay	Substrate / Cofactor	Incubation	Detected Component	Analytical Method
Metabolic Stability (CYP2C8)	Test compound (1 µM), NADP (1.3 mM), G6P (3.3 mM), G6PDHase (0.4 U/ml) (n=2)	0 and 60 min, 37 °C<br>Phosphate buffer, pH 7.4	Product ion corresponding to the test compound via SRM	HPLC-MS/MS
Metabolic Stability (CYP2C9)	Test compound (1 µM), NADP (1.3 mM), G6P (3.3 mM), G6PDHase (0.4 U/ml) (n=2)	0 and 60 min, 37 °C<br>Phosphate buffer, pH 7.4	Product ion corresponding to the test compound via SRM	HPLC-MS/MS
Metabolic Stability (CYP2C19)	Test compound (1 µM), NADP (1.3 mM), G6P (3.3 mM), G6PDHase (0.4 U/ml) (n=2)	0 and 60 min, 37 °C<br>Phosphate buffer, pH 7.4	Product ion corresponding to the test compound via SRM	HPLC-MS/MS
Metabolic Stability (CYP2D6)	Test compound (1 µM), NADP (1.3 mM), G6P (3.3 mM), G6PDHase (0.4 U/mL) (n=2)	0 and 60 min, 37 °C<br>Phosphate buffer, pH 7.4	Product ion corresponding to the test compound via SRM	HPLC-MS/MS
Metabolic Stability (CYP3A4)	Test compound (1 µM), NADP (1.3 mM), G6P (3.3 mM), G6PDHase (0.4 U/mL) (n = 2)	0 and 60 min, 37 °C<br>Phosphate buffer, pH 7.4	Product ion corresponding to the test compound via SRM	HPLC-MS/MS
Metabolic Stability (CYP3A5)	Test compound (1 µM) NADP (1.3 mM), G6P (3.3 mM), G6PDHase (0.4 U/mL) (n=2)	0 and 60 min. 37 °C<br>Phosphate buffer, pH 7.4	Product ion corresponding to the test compound via SRM	HPLC-MS/MS

Abbreviations:
CYP: Cytochrome P450
G6P: D-Glucose-6-phosphate, from Sigma, catalog number G-7772
G6PDHase: Glucose-6-phosphate dehydrogenase, from Sigma, catalog number G-4134
HPLC-MS/MS: HPLC coupled with tandem mass spectrometry (Instrumentation: Thermo Finnigan)
HPLC: High performance liquid chromatography
NADP: β-Nicotinamide adenine dinucleotide phosphate, from Sigma, catalog number N-0505
SRM: Selected reaction monitoring

2.2.3. Analysis and Expression of Results

Metabolic Stability (recombinant CYP, microsomes, S9 or hepatocytes)

At the end of incubation at each of the time points, an equal volume of an organic mixture (acetonitrile/methanol, 50/50, v/v) was added to the incubation mixture followed by centrifugation. Samples were analyzed by HPLC-MS/MS. Peak areas corresponding to the analytes were determined by HPLC-MS/MS. The ratio of precursor compound remaining at the end of the incubation relative to the amount remaining at time zero, expressed as percent, is reported as metabolic stability.

3. COMPOUNDS

3.1. Test Compounds

From: DNDI FOUNDATION

CEREP I.D.	Compound I.D.	Reference Number	Batch Number	Molecular Weight	Submitted F.W.	Stock Solution
14925-1	Fexinidazole	ECH:08179/58	3168-07-01/O	279	279	1.E-02 M DMSO
14925-2	Fexinidazole sulfone	ECH:08179/60	3218-87-56	311	311	1.E-02 M DMSO
14925-3	Fexinidazole sulfoxyde	ECH:08179/59	3217-87-47/B	295	295	1.E-02 M DMSO

F.W.: Formula Weight

3.2. Reference Compounds

In each experiment, the respective reference compounds were tested concurrently with compounds Fexinidazole, Fexinidazole sulfone and Fexinidazole sulfoxyde, and the data were compared with historical values determined at Cerep. The experiment was accepted in accordance with Cerep's validation Standard Operating Procedure.

4. RESULTS

4.1. ADME-Tox: Bioanalytical

The individual data obtained with compounds Fexinidazole, Fexinidazole sulfone and Fexinidazole sulfoxyde are reported in table 1 - 1.

Table 1 - 1

Individual Data

Assay Cerep Compound I.D.	Client Compound I.D.	Molecular Weight	FW	Selected ESI (+) Precursor Ion (m/z)	Product Ion (m/z)	Collision Offset (V)	Ionization Classification
HPLC-MS Screen							
14925-1	Fexinidazole	279	279	280.0	140.0	-20	2.0
14925-2	Fexinidazole sulfone	311	311	312.0	140.0	-20	3.0
14925-3	Fexinidazole sulfoxyde	295	295	296.0	233.0	-20	2.0

Notes:
Ionization Classification:
1 = Highly ionizable compound
2 = Intermediately ionizable compound
3 = Poorly ionizable compound

4.2. ADME-Tox: *In Vitro* Metabolism

Metabolic Stability:

The summary results obtained with compounds Fexinidazole, Fexinidazole sulfone and Fexinidazole sulfoxyde are reported in table 2 - 1.

The individual data obtained with compounds Fexinidazole, Fexinidazole sulfone and Fexinidazole sulfoxyde are reported in table 2 - 2.

The individual data obtained with the reference compounds are reported in table 2 - 3.

Table 2 - 1

Summary Results

Assay Cerep Compound I.D.	Client Compound I.D.	Test Concentration (M)	Mean Parent Remaining (%)
Metabolic Stability (CYP1A2)			
14925-1	Fexinidazole	1.0E-06	28
14925-2	Fexinidazole sulfone	1.0E-06	79
14925-3	Fexinidazole sulfoxyde	1.0E-06	101
Metabolic Stability (CYP2B6)			
14925-1	Fexinidazole	1.0E-06	12
14925-2	Fexinidazole sulfone	1.0E-06	99
14925-3	Fexinidazole sulfoxyde	1.0E-06	98
Metabolic Stability (CYP2C8)			
14925-1	Fexinidazole	1.0E-06	91
14925-2	Fexinidazole sulfone	1.0E-06	96
14925-3	Fexinidazole sulfoxyde	1.0E-06	97
Metabolic Stability (CYP2C9)			
14925-1	Fexinidazole	1.0E-06	97
14925-2	Fexinidazole sulfone	1.0E-06	93
14925-3	Fexinidazole sulfoxyde	1.0E-06	97
Metabolic Stability (CYP2C19)			
14925-1	Fexinidazole	1.0E-06	0
14925-2	Fexinidazole sulfone	1.0E-06	102
14925-3	Fexinidazole sulfoxyde	1.0E-06	101
Metabolic Stability (CYP2D6)			
14925-1	Fexinidazole	1.0E-06	73
14925-2	Fexinidazole sulfone	1.0E-06	103
14925-3	Fexinidazole sulfoxyde	1.0E-06	100
Metabolic Stability (CYP3A4)			
14925-1	Fexinidazole	1.0E-06	13
14925-2	Fexinidazole sulfone	1.0E-06	102
14925-3	Fexinidazole sulfoxyde	1.0E-06	97
Metabolic Stability (CYP3A5)			
14925-1	Fexinidazole	1.0E-06	21
14925-2	Fexinidazole sulfone	1.0E-06	103
14925-3	Fexinidazole sulfoxyde	1.0E-06	105

Table 2 - 2

Individual Data

Assay Cerep Compound I.D.	Client Compound I.D.	Test Concentration (M)	Parent Remaining 1st (%)	2nd (%)	Mean (%)
Metabolic Stability (CYP1A2)					
14925-1	Fexinidazole	1.0E-06	27.8	27.8	**28**
14925-2	Fexinidazole sulfone	1.0E-06	80.1	77.4	**79**
14925-3	Fexinidazole sulfoxyde	1.0E-06	99.7	102.0	**101**
Metabolic Stability (CYP2B6)					
14925-1	Fexinidazole	1.0E-06	12.3	11.9	**12**
14925-2	Fexinidazole sulfone	1.0E-06	99.5	98.7	**99**
14925-3	Fexinidazole sulfoxyde	1.0E-06	99.1	97.9	**98**
Metabolic Stability (CYP2C8)					
14925-1	Fexinidazole	1.0E-06	91.9	89.4	**91**
14925-2	Fexinidazole sulfone	1.0E-06	95.3	96.5	**96**
14925-3	Fexinidazole sulfoxyde	1.0E-06	98.0	95.5	**97**
Metabolic Stability (CYP2C9)					
14925-1	Fexinidazole	1.0E-06	91.5	101.9	**97**
14925-2	Fexinidazole sulfone	1.0E-06	88.5	97.7	**93**
14925-3	Fexinidazole sulfoxyde	1.0E-06	93.3	100.2	**97**
Metabolic Stability (CYP2C19)					
14925-1	Fexinidazole	1.0E-06	0.5	0.4	**0**
14925-2	Fexinidazole sulfone	1.0E-06	103.1	101.0	**102**
14925-3	Fexinidazole sulfoxyde	1.0E-06	101.0	101.3	**101**
Metabolic Stability (CYP2D6)					
14925-1	Fexinidazole	1.0E-06	71.9	73.5	**73**
14925-2	Fexinidazole sulfone	1.0E-06	111.0	94.6	**103**
14925-3	Fexinidazole sulfoxyde	1.0E-06	98.8	100.3	**100**
Metabolic Stability (CYP3A4)					
14925-1	Fexinidazole	1.0E-06	14.4	10.8	**13**
14925-2	Fexinidazole sulfone	1.0E-06	105.8	99.1	**102**
14925-3	Fexinidazole sulfoxyde	1.0E-06	99.6	93.8	**97**
Metabolic Stability (CYP3A5)					
14925-1	Fexinidazole	1.0E-06	20.9	21.5	**21**
14925-2	Fexinidazole sulfone	1.0E-06	104.0	101.1	**103**
14925-3	Fexinidazole sulfoxyde	1.0E-06	106.4	103.9	**105**

Table 2 - 3

Reference Compound Data

Assay<br>Reference Compound	Test Concentration (M)	Parent Remaining 1st (%)	2nd (%)	Mean (%)
Metabolic Stability (CYP1A2)				
Ethoxyresorufin	1.0E-06	0.7	0.7	**1**
Propranolol	1.0E-06	43.4	45.3	**44**
Metabolic Stability (CYP2B6)				
Benaphetamine	1.0E-06	4.3	4.1	**4**
Propranolol	1.0E-06	99.0	101.6	**100**
Metabolic Stability (CYP2C8)				
Paclitaxel	1.0E-06	1.0	3.9	**2**
Propranolol	1.0E-06	96.8	98.6	**98**
Metabolic Stability (CYP2C9)				
Diclofenac	1.0E-06	0.2	0.1	**0**
Propranolol	1.0E-06	102.8	99.4	**101**
Metabolic Stability (CYP2C19)				
Promazine	1.0E-06	56.6	57.4	**57**
Propranolol	1.0E-06	90.5	91.6	**91**
Metabolic Stability (CYP2D6)				
Propranolol	1.0E-06	11.5	11.2	**11**
Terfenadine	1.0E-06	119.3	123.8	**122**
Metabolic Stability (CYP3A4)				
Propranolol	1.0E-06	102.3	101.4	**102**
Terfenadine	1.0E-06	11.2	11.8	**12**
Metabolic Stability (CYP3A5)				
Propranolol	1.0E-06	99.5	104.9	**102**
Terfenadine	1.0E-06	7.6	7.0	**7**

5. BIBLIOGRAPHY

SUZUKI, A., IIDA, I., TANAKA, F., AKIMOTO, M., FUKUSHIMA, K., TANI, M., ISHIZAKI, T., and CHIBA, K. (1999)
Identification of human cytochrome P-450 isoforms involved in metabolism of R(+)- and S(-)- gallopamil: utility of in vitro disappearance rate.
Drug Metab. Dispos., 27: 1254-1259.

6. DATA AND RECORDS STORAGE

All documents generated during the performance of the study (raw data, various records such as QA audit reports, an original copy of the study report, study plan...) will be archived by Cerep for a 10-year period after study completion. The access to the archives is restricted to authorized employees only.

The original final report provided to the sponsor will be kept by the sponsor under its sole responsibility.
