# Supplementary material for: Fexinidazole – A New Oral Nitroimidazole Drug Candidate Entering Clinical Development for the Treatment of Sleeping Sickness
Source: PLoS Negl Trop Dis. 2010 Dec 21;4(12):e923. doi: 10.1371/journal.pntd.0000923 (PMC3006138; doi:10.1371/journal.pntd.0000923)
Supplement: Dataset S4 — (0.15 MB PDF) [file pntd.0000923.s005.pdf]

FINAL REPORT

STUDY NUMBER
6DNDIP2

Caco-2 Permeability

SUMMARY

The bi-directional permeability of three test compounds was examined in a Caco-2 cell system. All test compounds were found to have high permeability and no significant efflux.

DATE OF ISSUE

September 21, 2006

PREPARED FOR

DNDi
1 Place St Gervais
Geneva, CH-1201, Switzerland

PREPARED BY

Absorption Systems, LP

COMPLIANCE

This study followed established practices and standard operating procedures of Absorption Systems, LP. The report is archived in a validated Scientific Data Management System. Electronic signatures comply with the regulation 21 CFR Part 11.

ABSORPTION SYSTEMS *a better way*

1. OBJECTIVE

The objective of this study was to determine the bi-directional Caco-2 permeability and efflux limited absorption potential of three test compounds.

2. PERMEABILITY, CACO-2

Caco-2 monolayers were grown to confluence on collagen-coated, microporous, polycarbonate membranes in 12-well Costar Transwell® plates. Details of the plates and their certification are shown below. The permeability assay buffer for the donor chamber was Hanks Balanced Salt Solution containing 10 mM HEPES and 15 mM glucose at a pH of 7.4. The buffer in the receiver chamber also contained 1% Bovine Serum Albumin (BSA). The test compound dosing concentrations were 5 µM in the assay buffer. The cells were dosed on the apical side (A-to-B) or basolateral side (B-to-A) and incubated at 37°C with 5% CO_2 in a humidified incubator. After two hours, aliquots were taken from the receiver and donor chambers. Each determination was performed in duplicate. The Lucifer Yellow flux was also measured for each monolayer after being subjected to the test compounds to ensure no damage was inflicted to the cell monolayers during the flux period. All samples were assayed by LC/MS/MS using electrospray ionization. Analytical conditions are outlined in Attachment I. The apparent permeability, P_{app}, and percent recovery were calculated as follows:

$$P_{app} = (dC_r/dt) \times V_r/(A \times C) \quad (1)$$

$$\text{Percent Recovery} = 100 \times ((V_r \times C_r^{final}) + (V_d \times C_d^{final}))/(V_d \times C_N) \quad (2)$$

where,

dC_r/dt is the slope of the cumulative concentration in the receiver compartment versus time in µM s^{-1}.
V_r is the volume of the receiver compartment in cm^3.
V_d is the volume of the donor compartment in cm^3.
A is the area of the cell monolayer (1.13 cm^2 for 12-well Transwell®).
C_N is the nominal concentration of the dosing solution in µM.
C is the average of the nominal dosing concentration and the measured concentration in the donor chamber at 2 hours.
C_r^{final} is the cumulative receiver concentration in µM at the end of the incubation period.
C_d^{final} is the concentration of the donor in µM at the end of the incubation period.

Plate:	TW12	
Seed Date:	8/29/06 PSK	
Passage #:	61	
Age (days):	22	
		**Acceptance Criteria**
TEER Value ($\Omega \cdot cm^2$):	512	450-650
Lucifer Yellow P_{app}, x 10^{-6} cm/s:	0.23	<0.40
Atenolol P_{app}, x 10^{-6} cm/s:	0.26	<0.50
Propranolol P_{app}, x 10^{-6} cm/s:	16	15-25
Digoxin (A-B) P_{app}, x 10^{-6} cm/s:	0.53	None
Digoxin (B-A) P_{app}, x 10^{-6} cm/s:	10.4	None
Digoxin (B-A P_{app}) /(A-B P_{app}):	19.6	>3

Table 2.1 Recovery and Apparent Permeability (10^{-6} cm/s) of Test Compounds

Test Compound Identification	Percent Recovery (C)		P_{app}, A→B			P_{app}, B→A			$\frac{P_{app}^{B-A}}{P_{app}^{A-B}}$ Ratio (B)	Absorption Potential (A)	Significant Efflux (B)
	A→B	B→A	Rep. 1	Rep. 2	Avg	Rep. 1	Rep. 2	Avg			
RO-15-0216-001-004	90	94	26.7	25.2	26.0	34.8	34.2*	34.5	1.3	High	No
RO-15-6547-000-001	90	91	24.6	18.6	21.6	28.1	29.0*	28.5	1.3	High	No
Fexinidazole	72	81	53.5	60.9	57.2	50.7	51.1*	50.9	0.9	High	No

(A) Absorption Potential Classification:
P_{app} (A-to-B) ≥ 1.0 x 10^{-6} cm/s: High
P_{app} (A-to-B) < 0.5 x 10^{-6} cm/s: Low

(B) Efflux considered significant if:
P_{app} (B-to-A) ≥ 1.0 x 10^{-6} cm/s and Ratio P_{app} (B-to-A) / P_{app} (A-to-B) ≥ 3.0

(C) Low recoveries caused by non-specific binding, etc. can affect the measured permeability.

COMMENTS

The three test compounds were all classified as having a high absorption potential and having no significant efflux; their intestinal permeability is not expected to be a limiting factor for their absorption in humans.

The values marked with asterisks indicate that the cell monolayers associated with those values failed the post-experiment Lucifer Yellow integrity test (NB# AS521, pages 90-91). However, the values obtained from these monolayers were in agreement with the values obtained from the respective replicate monolayers, so the data was accepted.

ATTACHMENT I

Liquid Chromatography

Column: Keystone Hypersil BDS C18 30x2.0 mm i.d., 3 μm, with guard column

M.P. Buffer: 25 mM Ammonium Formate Buffer, pH 3.5
Aqueous Reservoir (A): 90% water, 10% buffer
Organic Reservoir (B): 90% acetonitrile, 10% buffer

Flow Rate: 300 μL/minute

Gradient Program:

Time (Min)	% A	% B
0.0	100	0
1.5	0	100
2.0	0	100
2.1	100	0
3.5	100	0

Total Run Time: 3.5 min

Autosampler: 10 μL Injection Volume

Autosampler Wash: water/acetonitrile/2-propanol: 1/1/1; with 0.2% formic acid

Mass Spectrometer

Instrument: PE SCIEX API 2000

Interface: Electrospray ("Turbo Ionspray")

Mode: Multiple Reaction Monitoring

Method: 3.5 minute duration

Settings:

Compound	Q1/Q3	DP	FP	EP	CE	CXP	IS	TEM	GS1	GS2	CUR	CAD
RO-15-0216-001-004	+334.1/58.0	66	200	10	43	10	5500	500	40	80	20	4
RO-15-6547-000-001	+360.1/84.1	66	200	10	44	4	5500	500	40	80	20	4
Fexinidazole	+280.3/140.3	87	200	10	24	7	5500	500	40	80	20	4
