# Supplementary material for: Fexinidazole – A New Oral Nitroimidazole Drug Candidate Entering Clinical Development for the Treatment of Sleeping Sickness
Source: PLoS Negl Trop Dis. 2010 Dec 21;4(12):e923. doi: 10.1371/journal.pntd.0000923 (PMC3006138; doi:10.1371/journal.pntd.0000923)
Supplement: Dataset S5 — (0.13 MB PDF) [file pntd.0000923.s006.pdf]

Final Summary Report

Study to investigate the pharmacokinetics and blood brain penetrability of Fexinidazole following intravenous and oral administration in the mouse

Protocol for	*Drugs for Neglected Diseases Initiative (DNDi)*
Address	*1 Place Saint Gervais*<br>*1201 Geneva*<br>*Switzerland*
Tel.	*+ 41 (0) 22 906 9230*
Fax.	*+41 (0) 22 906 9231*
Study Director	
	BioDynamics Research Limited
Tel.	
Fax.	
E-mail.	
BioDynamics study no.	*DND/01*
Date	*10 December 2008*

Contents

	Page
Study personnel	5
Study dates	5
Study Director's statement	6
Summary	7

	Page
1. Materials and methods	9
1.1 Test compound details	9
1.2 Mice: source, specification and husbandry	9
1.3 Experimental procedures	9
1.3.1 Intravenous formulation	9
1.3.2 Oral formulation	9
1.4 Dose regimen	10
1.5 Dose administration	10
1.6 Blood sample collection	10
1.7 Collection of terminal brain samples	10
2. Sample Bioanalysis	10
2.1.1 Preparation of brain samples	11
2.1.2 Preparation of study samples	11
2.2 Chromatography and mass spectroscopy conditions	11
3. Pharmacokinetic analysis	12
3.1.1 Area under the curve (AUC)	12
4. Maintenance of records/data	12
5. Protocol/SOP compliance	12
6. Results	13
6.1 Pharmacokinetic analysis	13
6.2 Plasma results	14
6.2.1 Fexinidazole	14
6.2.2 Fexinidazole sulfoxide	15
6.2.3 Fexinidazole sulfone	16
6.3 Brain results	19

Tables

Table 1	Mean summary pharmacokinetic data of Fexinidazole, sulfone and sulfoxide in plasma following oral and intravenous administration of Fexinidazole to female NMR mice	13
Table 2	Plasma concentrations of Fexinidazole following intravenous administration at a dose level of 1 mg/kg to female NMR1 mice	14
Table 3	Plasma concentrations of Fexinidazole following oral administration at a dose level of 25 mg/kg to female NMR1 mice	14
Table 4	Plasma concentrations of sulfoxide following intravenous administration of Fexinidazole to mice at a dose level 1 mg/kg	15
Table 5	Plasma concentrations of sulfoxide following oral administration of Fexinidazole to mice at 25 mg/kg	16
Table 6	Plasma concentrations of sulfone following intravenous administration of Fexinidazole at a dose level of 1 mg/kg to female NMR mice	17
Table 7	Plasma concentrations of sulfone following oral administration of Fexinidazole at a dose level of 25 mg/kg to female NMR mice	17
Table 8	Mean plasma:brain ratios of Fexinidazole, Sulfone and Sulfoxide	19
Table 9	Mean brain concentrations of Fexinidazole, sulfone and sulfoxide following intravenous and oral administration of Fexinidazole at 1 and 25 mg/kg respectively to female NMR1 mice	19
Table 10	Brain concentrations of Fexinidazole following intravenous and oral administration of Fexinidazole at 1 and 25 mg/kg respectively to female NMR1 mice	20
Table 11	Brain concentrations of sulfoxide following intravenous and oral administration of Fexinidazole at 1 and 25 mg/kg respectively to female NMR1 mice	20
Table 12	Brain concentrations of sulfone following intravenous and oral administration of Fexinidazole at 1 and 25 mg/kg respectively to female NMR1 mice	21

Figures

Figure 1	Linear plot of mean Fexinidazole, sulfone and sulfoxide concentrations in plasma following intravenous administration of Fexinidazole at 1 mg/kg to female NMRI mice	18
Figure 2	Linear plot of mean Fexinidazole, sulfone and sulfoxide concentrations in plasma following oral administration of Fexinidazole at 25 mg/kg to female NMRI mice	18
Figure 3	Brain concentrations of Fexinidazole and its sulfone and sulfoxide metabolites following intravenous administration of Fexinidazole at a dose of 1 mg/kg	22
Figure 4	Brain concentrations of Fexinidazole and its sulfone and sulfoxide metabolites following oral administration of Fexinidazole at a dose of 25 mg/kg.	22

Appendices

Appendix 1	Animal body weights and dose volumes	23

Summary

Two groups of female NMRI mice received an intravenous (n=24) or oral dose (n=27) of the antimicrobial nitroimidazole Fexinidazole at dose levels of 1 and 25 mg/kg respectively.

Following intravenous administration at 1 mg/kg, terminal blood and brain samples were collected from 3 animals per time point at 5, 15, 30, 60, 120, 240, 480 minutes and 24 hours post dose. Similarly, following oral administration terminal blood and brain samples were taken from 3 animals per time point at 15, 30, 60, 120, 240, 360, 480, 720 minutes and 24 hours post dose.

Plasma was prepared from all of the blood samples by centrifugation. All samples were then stored at less than -70°C prior to analysis by LC-MS/MS. Brain samples taken at the 15 minute time-point were also analysed for concentrations of test material. Plasma samples taken at 15, 30 and 60 minutes following the oral dose were screened for the presence of known or potential metabolites (reported separately in DNDi/01: Metabolite ID Summary (Fexinidazole results), 24th July 2007) and the concentrations of the sulfone and sulfoxide metabolites were measured in all plasma samples. In addition selected brain samples (5 and 30 minutes for intravenously dosed animals and 30 and 60 minutes from orally dosed animals) were analysed for concentrations of Fexinidazole and its sulfone and sulfoxide metabolites.

No clinical signs were observed following either intravenous or oral administration of Fexinidazole.

Following intravenous administration of Fexinidazole a C_{max} of 164 ng/mL was achieved with the mean T_{max} being observed at 5 minutes post-dose (first sampling time point). Plasma concentrations of Fexinidazole were measurable in 2 of 3 animals at 60 minutes post dose but all animals were BLQ by 120 minutes post-dose (LLOQ 10 ng/mL). The mean plasma elimination half life was calculated to be 6 minutes. Oral administration of the compound gave a C_{max} of 499.8 ng/mL observed at 15 minutes post dose (first sampling time point), with plasma levels measurable in 2 of 3 animals up to 4 hours post dose. At 6 hours post-dose all animals were BLQ (LLOQ 10 ng/mL). The plasma elimination half life following oral administration was calculated to be 48 minutes and the oral bioavailability of the compound was 41.4 %.

Measurement of Fexinidazole concentrations in brain samples indicated that it does penetrate the blood brain barrier. Following intravenous administration a mean maximum concentration of 92.5 ng/mL was observed at 5 minutes post-dose, compared to a plasma concentration of 164.3 ng/mL. This had declined to 12.1 ng/mL by 30 minutes post-dose (plasma concentration 11.7 ng/mL).
Following oral administration a mean concentration of 378.8 ng/mL was observed at 15 minutes post-dose compared to a plasma concentration of 499.8 ng/mL. At 60 minutes post-dose this had declined to 254.4 ng/mL compared to 207.9 ng/mL in the plasma.

Sulfoxide metabolite concentrations in brain samples were detectable in all animals following both intravenous and oral administration, while sulfone metabolite concentrations were only detectable after oral administration of Fexinidazole. Sulfoxide concentrations were considerably higher after oral administration compared to intravenous administration. The mean maximum concentrations of sulfone and sulfoxide in plasma after oral administration of Fexinidazole were 6599.6 & 14171 ng/mL respectively with intravenous concentrations being 510.7 and 918.3 ng/mL respectively.

These results suggest that both the sulfone and sulfoxide metabolites cross the blood brain barrier following oral administration, while only the sulfoxide metabolite crosses after intravenous administration. The sulfoxide metabolite was found to have a bioavailability of 114% while the sulfone metabolite had a bioavailability of 48.8% following oral administration of Fexinidazole.

After intravenous administration of Fexinidazole, concentrations of the sulfoxide metabolite were found to increase by approximately 1.5 times between 5 and 30 minutes post dose. At 5 minutes post-dose the concentration of sulfoxide metabolite was *ca*. 22 times higher in the plasma than in the brain. By 30 minutes post-dose, sulfoxide levels in the brain had increased such that levels were *ca*.15 times higher in the plasma than in the brain.

Following oral administration of Fexinidazole, both the sulfoxide and sulfone metabolites increased in concentration in the brain between 30 to 60 minutes post dose. The sulfone metabolite increased by approximately 2.5 times while the sulfoxide metabolite increased by approximately 1.5 times. At 30 minutes post-dose the concentration of sulfoxide metabolite was *ca*. 13 times higher in the plasma than in the brain. By 60 minutes post-dose sulfoxide levels had increased in the brain such that levels were *ca*. 9 times higher in the plasma than in the brain. At 30 minutes post-dose the concentration of sulfone metabolite was *ca*. 20 times higher in the plasma than in the brain. By 60 minutes post-dose, concentrations of sulfone in both the brain and plasma had increased similarly, such that sulfone levels were *ca*. 17 times higher in the plasma than in the brain.

1. Materials and methods

1.1 Test compound details

Fexinidazole (S751239-E, Hoechst sample) and its sulfone and sulfoxide metabolites (LPF-sulfone and LPF-sulfoxide) were supplied by DNDi.

Previous studies (not conducted by BioDynamics) reported that Fexinidazole had poor solubility therefore intravenous administration at a dose level of 1 mg/kg was chosen;

- to allow formulation at low concentration, to ensure a suitable dosing formulation was administered (solution)

- as this would not pose a problem in terms of analytical sensitivity using generic mass spectrometry conditions.

1.2 Mice: source, specification and husbandry

Female NMRI mice (Harlan UK, Shaws Farm, Bicester Oxon) were used for this study.

On arrival, all mice were examined for external signs of ill-health and any unhealthy animals excluded from the study. Mice were housed in groups of twenty in polypropylene cages with solid floors, in a thermostatically monitored room (21 ± 2°C) and exposed to 12 hours fluorescent lighting and 12 hours dark per day. The temperature and relative humidity (range 45 - 65%) of the holding room were recorded on a daily basis throughout the study. Animals were equilibrated under standard animal house conditions for a minimum of 2 days prior to use.

Pellet diet (RM1 (E) SQC, Special Diets Services, Witham, Essex, U.K.) and water, from the domestic water supply, was available *ad libitum* throughout the experimental and acclimatisation periods. There were no known contaminants present in the diet or drinking water, which could in the opinion of the Study Director, have interfered with or affected the outcome of the study.

1.3 Experimental procedures

1.3.1 Intravenous formulation

The intravenous formulation was prepared using a standard formulation for poorly soluble compounds, this comprised of 10% dimethyl sulphoxide (DMSO) and 90% hydroxyl-propyl-β-cyclodextrin (HPβCD) 10% w/v in PBS pH7.4. The test material was initially dissolved in DMSO at 1 mg/mL free base, followed by dilution of the co solvent stock to 0.1 mg/mL with the addition of 10% HPβCD. This gave a final intravenous dose comprising of 0.1 mg/mL test material, 10% DMSO and 90% HPβCD (10% w/v in PBS pH7.4).

1.3.2 Oral formulation

The oral dose was formulated by suspending the test material in 1% DMSO/99% methyl-cellulose (1% w/v in water) at a concentration of 2.5 mg/mL free base.

1.4 Dose regimen

Two groups of female mice were dosed as follows:

Group Number	Treatment	Dose (mg/kg)	Route
1	Fexinidazole (S751239-E)	1	i.v.
2	Fexinidazole (S751239-E)	25	p.o.

All doses are expressed as free base

1.5 Dose administration

Intravenous doses were administered into a lateral tail vein using an appropriately sized syringe and 25G needle and the oral formulation by oral gavage using a polypropylene gavage tube. All doses were given as a single bolus dose at a constant dose volume of 10.0 mL/kg.

1.6 Blood sample collection

At each sampling time point, three animals in each treatment group were deeply anaesthetised using isoflurane and blood samples (approximately 1 mL) taken into individual heparinised tubes by cardiac puncture, following which the animals were killed by cervical fracture. Samples were collected at the following times post-dose:

Intravenous 5, 15, 30, 60, 120, 240, 480 minutes and 24 hours

Oral 15, 30, 60, 120, 240, 360, 480, 720 minutes and 24 hours

Following collection, the blood samples were centrifuged (approximately 10000 g, 2 min at 4 °C) and the plasma retained as one aliquot at approximately -70°C prior to analysis of test material concentrations at BioDynamics by LC-MS/MS.

1.7 Collection of terminal brain samples

Following collection of the terminal blood sample the cranium of each mouse was carefully opened and the brain removed. Excess blood was washed from the brain with distilled water and any excess fluid blotted on absorbent paper. The brain was then snap frozen using liquid nitrogen and placed into a suitably labelled container and stored frozen at approximately -70°C pending analysis.

2. Sample Bioanalysis

All of the plasma samples together with the brains from the 15 minute time point were analysed for concentrations of the test material and its sulfone and sulfoxide metabolites. In addition the brains from the 5 and 30 minute time points for the intravenously treated and the 30 and 60 minute time points for the orally treated animals were analysed for concentrations of Fexinidazole and its sulfone and sulfoxide metabolites using an LC-MS/MS method developed at BioDynamics.

All samples were treated as temperature sensitive. Blood samples were collected onto wet ice, followed by centrifugation at 4°C. Plasma and brain samples were snap frozen on dry ice (plasma) or liquid nitrogen (brains) prior to long term storage at less than -70°C pending analysis.

2.1.1 Preparation of brain samples

Prior to analysis for test material concentrations, brain samples were weighed and homogenized with the addition of 2 mL de-ionized water per gram of brain tissue (for example, a brain weighing 0.386 g would be homogenized with 0.772 mL de-ionized water). Homogenates were then placed in an ultrasonic bath and sonicated for 10 minutes.

Due to this homogenization procedure, results expressed as ng/mL of brain homogenate are converted to ng/g brain using a multiplication factor of 3.

2.1.2 Preparation of study samples

All study samples (plasma and brain) were extracted by adding 200 µL of the study sample (plasma) or homogenate (brain), 200 µL methanol and 400 µL internal standard solution in acetonitrile into a 1.5 mL eppendorf tube. This was vortex mixed for ca. 5 minutes to precipitate proteins before being centrifuged for 5 minutes at 12000 rpm. 200 µL of the resulting supernatant was then removed and transferred to an autosampler vial for analysis.

2.2 Chromatography and mass spectroscopy conditions

The chromatographic system used for on-line LC-MS and LC-MS/MS analysis of the reference materials, internal standard and study samples consisted of a Surveyor auto sampler and HPLC pump with ternary gradient unit linked to a Thermo TSQ Quantum triple quadruple mass spectrometer operating in positive ion mode.

Data files were processed using LCQuan software for Windows running on a Dell™ personal workstation.

Liquid chromatography was carried out with a Waters Xterra MS 5µm C18 10cm x 3mm analytical column (Phenomenex™). The mobile phase was a 0.1% formic acid in water: 0.1% formic acid in acetonitrile gradient. The flow rate was 0.2 mL/min and the injection volume was 40 µL.

The MS/MS conditions were:

	Ionisation mode	Electrospray in +ve ion mode
Compound	MRM	Collision Energy (eV)
Fexinidazole	m/z 280.03 → m/z 139.9	20
	m/z 280.06 → m/z 124.9	44
	m/z 280.07 → m/z 140.9	27
Sulfoxide	m/z 296.03 → m/z 233.0	28
	m/z 296.04 → m/z 140.0	24
	m/z 296.05 → m/z 141.0	27
Sulfone	m/z 312.04 → m/z 141.0	23
	m/z 312.05 → m/z 141.0	30
	m/z 312.06 → m/z 111.0	41

Summed transitions were used to enhance sensitivity. No instability of the test materials was noted during the course of analysis or on re-injection of the samples into the mass spectrometer under the conditions documented.

3. Pharmacokinetic analysis

The plasma concentration vs time curves obtained following intravenous and oral administration of the test compounds to female mice were analysed using WinNonLin (Version 4.1, Scientific Consulting Inc. USA). The kinetic data were characterised by a non-compartmental analysis (NCA).

The following pharmacokinetic parameters were derived from the profiles: maximum peak plasma concentration (Cmax); the time of maximum observed concentration (Tmax); the terminal half life (T½), and area under the plasma concentration curve (AUC),

3.1.1 Area under the curve (AUC)

The AUC was determined using the linear/log trapezoidal method. A value of zero was used for any plasma concentrations recorded as below the limits of quantification (LLOQ). The AUC_{inf} (observed) was calculated as the area under the curve from the time of dosing extrapolated to time infinity based on the observed concentrations. The AUC_{last} was also determined. The AUC_{last} parameter is defined as the area under the curve from the point of dosing to the last measurable concentration.

4. Maintenance of records/data

On completion of the study all records for the study were collected and retained by the Study Director. Upon authorisation of the report the records will be transferred to BioDynamics archives and will be retained for a period of three years, the Sponsor will then be contacted with regard to future storage.

5. Protocol/SOP compliance

BioDynamics adopted all reasonable measures to carry out the study in accordance with the protocol and Standard Operating Procedures. Under practical working conditions however, some minor variations may have occurred due to circumstances beyond the control of BioDynamics. Any deviations were documented in the study records, together with the reason for their occurrence.

6. Results

No unusual clinical observations were made following dosing of either of the groups of animals.

6.1 Pharmacokinetic analysis

Table 1 Mean summary pharmacokinetic data of Fexinidazole, sulfone and sulfoxide in plasma following oral and intravenous administration of Fexinidazole to female NMR mice

	C_{max} (ng/mL)	T_{max} (h)	$T_{1/2}$ (h)	AUC_{0-24} (h.ng/mL)	$AUC0_{-inf}$ (h.ng/mL)	Oral Bioavailability (%F)
Fexinidazole (i.v.)	164	0.0833	0.1	41	42	41.4
Fexinidazole (p.o.)	499.8	0.25	0.8	424	439	
Sulfoxide	918.3	0.25	0.6	1586.1	1555.4	114
Sulfoxide	14171	0.5	1.0	45030.5	45002.2	
Sulfone (i.v. administration of Fexinidazole)	790.4	2	n.d.	7990.3	nd	48
Sulfone (p.o. administration of Fexinidazole)	13651.3	4	1.7	96286.4	96307.6	

C_{max} = maximum plasma concentration
T_{max} = time of maximum plasma concentration
AUC_{0-24} = area under curve from time of dosing to the last measurable concentration
AUC_{inf} = area under curve from time of dosing extrapolated to infinity (observed conc.)
$T_{1/2}$ = terminal elimination half life
n.d. = not determined

6.2 Plasma results

6.2.1 Fexinidazole

Following intravenous administration of Fexinidazole at 1mg/kg, the mean C_{max} was 164 ng/mL with T_{max} being observed at the first sampling time point of 5 minutes post-dose. Plasma concentrations of Fexinidazole were measurable up to 30 minutes after injection with the mean AUC_{0-24} being 41 ng/mL*hr and the mean terminal elimination half life 6 minutes.

Following oral administration of Fexinidazole at 25mg/kg, the mean C_{max} was 499.8 ng/mL and T_{max} observed at 15 minutes post-dose which is the first sampling time point. Plasma concentrations of Fexinidazole were measurable up to 4 hours after administration with the mean AUC_{0-24} being 424 ng/mL*hr and the mean terminal elimination half life 48 minutes.

The oral bioavailability was 41.4% based on the i.v. profile.

Table 2 Plasma concentrations of Fexinidazole following intravenous administration at a dose level of 1 mg/kg to female NMR1 mice

	Concentration (ng/mL)							
**Time point (min)**		**Animal**		**Animal**		**Animal**	**Mean**	**SD**
**5**	178.0	103	139.9	104	174.9	105	164.3	21.2
**15**	28.0	106	29.8	107	26.8	108	28.2	1.5
**30**	11.3	109	11.0	110	12.8	111	11.7	1.0
**60**	11.8	112	11.5	113	BLQ	114	BLQ	NC
**120**	BLQ	115	BLQ	116	BLQ	117	BLQ	NC
**240**	BLQ	118	BLQ	119	BLQ	120	BLQ	NC
**480**	BLQ	121	BLQ	122	BLQ	123	BLQ	NC
**1440**	BLQ	124	BLQ	125	BLQ	126	BLQ	NC

LLOQ = 10 ng/mL
BLQ = below limit of quantification
NC = not calculated

Table 3 Plasma concentrations of Fexinidazole following oral administration at a dose level of 25 mg/kg to female NMR1 mice

	Concentration (ng/mL)							
**Time point (min)**		**Animal**		**Animal**		**Animal**	**Mean**	**SD**
**15**	442.7	127	494.3	128	562.5	129	499.8	60.1
**30**	301.1	130	227.1	131	162.1	132	230.1	69.5
**60**	295.2	133	158.4	134	170.1	135	207.9	75.8
**120**	53.9	136	36.8	137	50.2	138	47.0	9.0
**240**	BLQ	139	12.6	140	34.8	141	15.8	17.6
**360**	BLQ	142	BLQ	143	BLQ	144	BLQ	NC
**480**	BLQ	145	BLQ	146	BLQ	147	BLQ	NC
**720**	BLQ	148	BLQ	149	BLQ	150	BLQ	NC
**1440**	BLQ	151	BLQ	152	BLQ	153	BLQ	NC

LLOQ = 10 ng/mL
NC = not calculated
BLQ = below limit of quantification

6.2.2 Fexinidazole sulfoxide

Following intravenous administration of Fexinidazole at 1mg/kg, the mean maximum plasma concentration of the sulfoxide metabolite (C_{max}) was 918.3 ng/mL at 15 minutes (T_{max}) post-dose. Plasma concentrations of Fexinidazole sulfoxide were measurable up to 4 hours after injection. The mean AUC_{0-24} for the sulfoxide metabolite was 1586.1 h*ng/mL and the mean terminal elimination half life ($t_{1/2}$) was 36 minutes. Fexinidazole sulfoxide metabolite was shown to have crossed the blood brain barrier with means of 119.6 ng/g and 172.7 ng/g present at 5 and 30 minutes, respectively.

Following oral administration of Fexinidazole at 25mg/kg, the mean maximum plasma concentration of the sulfoxide metabolite (C_{max}) was 14171 ng/mL at 30 minutes (T_{max}) post-dose. Plasma concentrations of Fexinidazole sulfoxide were measurable up to 8 hours post-dose in all subjects and up to 24 hours in one subject. The mean AUC_{0-24} for the sulfoxide metabolite was 45030.5 h*ng/mL and the mean terminal elimination half life ($t_{1/2}$) was 1 hour. Fexinidazole sulfoxide metabolite was shown to have crossed the blood brain barrier with means of 3314.7 ng/g and 4873.1 ng/g present at 30 and 60 minutes, respectively.

The bioavailability of Fexinidazole sulfoxide following oral administration of Fexinidazole was 114%.

Table 4 Plasma concentrations of sulfoxide following intravenous administration of Fexinidazole to mice at a dose level 1 mg/kg

Time (min)	Fexinidazole Sulfoxide Concentration (ng/mL)							
		Animal		Animal		Animal	Mean	SD
**5**	907.2	103	889.2	104	839.2	105	878.5	35.2
**15**	904.6	106	870.5	107	979.7	108	918.3	55.9
**30**	914.0	109	924.1	110	836.3	111	891.5	48.0
**60**	856.8	112	841.2	113	569.5	114	755.8	161.6
**120**	245.9	115	240.8	116	283.4	117	256.7	23.3
**240**	20.2	118	32.1	119	32.7	120	28.3	7.1
**480**	BLQ	121	BLQ	122	BLQ	123	BLQ	NC
**1440**	BLQ	124	BLQ	125	BLQ	126	BLQ	NC

LLOQ = 25 ng/mL
BLQ = below limit of quantification
NC = not calculated

Table 5 Plasma concentrations of sulfoxide following oral administration of Fexinidazole to mice at 25 mg/kg

	Fexinidazole Sulfoxide Concentration (ng/mL)							
**Time (hrs)**		**Animal**		**Animal**		**Animal**	**Mean**	**SD**
**15**	8093.7	127	8721.5	128	6377.7	129	7731.0	1213.3
**30**	16785.3	130	14730.3	131	10997.5	132	14171.0	2934.2
**60**	17054.0	133	12390.4	134	12371.9	135	13938.8	2697.9
**120**	13725.6	136	11039.6	137	10593.0	138	11786.1	1694.5
**240**	4429.0	139	3600.3	140	6059.0	141	4696.1	1250.9
**360**	743.0	142	809.9	143	741.9	144	764.9	38.9
**480**	437.8	145	961.9	146	47.5	147	482.4	458.8
**720**	BLQ	148	BLQ	149	22.1	150	BLQ	NC
**1440**	BLQ	151	BLQ	152	BLQ	153	BLQ	NC

LLOQ = 25 ng/mL
BLQ = below limit of quantification
NC = not calculated

6.2.3 Fexinidazole sulfone

Following intravenous administration of Fexinidazole at 1mg/kg, the mean maximum plasma concentration of the sulfone metabolite (C_{max}) was 790.4 ng/mL at 2 hours (T_{max}) post-dose. Plasma concentrations of Fexinidazole sulfone were measurable up to 8 hours after injection. The mean AUC_{0-24} for the sulfone metabolite was 7990.3 h*ng/mL. The mean terminal elimination half life ($t_{1/2}$) was not determined. Fexinidazole sulfone metabolite could not be measured in brain at either 5 or 30 minutes.

Following oral administration of Fexinidazole at 25mg/kg, the mean maximum plasma concentration of the sulfone metabolite (C_{max}) was 13651.3 ng/mL at 4 hours (T_{max}) post-dose. Plasma concentrations of Fexinidazole sulfone were measurable up to 24 hours after injection in 2 out of 4 subjects. The mean AUC_{0-24} for the sulfone metabolite was 96286.4 h*ng/mL and the mean terminal elimination half life ($t_{1/2}$) was 1.7 hours. Fexinidazole sulfone metabolite was shown to have crossed the blood brain barrier with means of 467.8 ng/g and 1182.6 ng/g present at 30 and 60 minutes, respectively.

The bioavailability of Fexinidazole sulfone following oral administration of Fexinidazole was 48%.

Table 6 **Plasma concentrations of sulfone following intravenous administration of Fexinidazole at a dose level of 1 mg/kg to female NMR mice**

Time (hrs)	Fexinidazole Sulfone Concentration (ng/mL)							
		Animal		Animal		Animal	Mean	SD
**5**	40.9	103	28.8	104	41.4	105	37.0	7.1
**15**	176.5	106	355.2	107	237.7	108	256.5	90.8
**30**	535.7	109	539.8	110	456.7	111	510.7	46.8
**60**	542.2	112	615.3	113	955.4	114	704.3	220.5
**120**	903.2	115	564.8	116	903.3	117	790.4	195.4
**240**	665.9	118	756.3	119	889.5	120	770.6	112.5
**480**	446.7	121	268.3	122	426.4	123	380.5	97.7
**1440**	BLQ	124	BLQ	125	BLQ	126	BLQ	NC

LLOQ = 25 ng/mL
BLQ = below limit of quantification
NC = not calculated

Table 7 **Plasma concentrations of sulfone following oral administration of Fexinidazole at a dose level of 25 mg/kg to female NMR mice**

	Fexinidazole Sulfone Concentration (ng/mL)							
**Time (hrs)**		Animal		Animal		Animal	Mean	SD
**15**	1505.7	127	1681.2	128	999.9	129	1395.6	353.7
**30**	3843.6	130	3396.1	131	2119.0	132	3119.6	894.9
**60**	7929.0	133	6023.8	134	5845.9	135	6599.6	1154.8
**120**	13439.1	136	11836.1	137	9732.3	138	11669.2	1859.0
**240**	19022.3	139	11734.1	140	10197.6	141	13651.3	4714.4
**360**	10481.7	142	8749.6	143	12434.7	144	10555.3	1843.7
**480**	8519.8	145	9327.3	146	7476.6	147	8441.2	927.8
**720**	659.4	148	753.5	149	1235.0	150	882.6	308.8
**1440**	16.3	151	10.4	152	BLQ	153	BLQ	NC

LLOQ = 25 ng/mL
BLQ = below limit of quantification
NC = not calculated

Figure 1 **Linear plot of mean Fexinidazole, sulfone and sulfoxide concentrations in plasma following intravenous administration of Fexinidazole at 1 mg/kg to female NMRI mice**

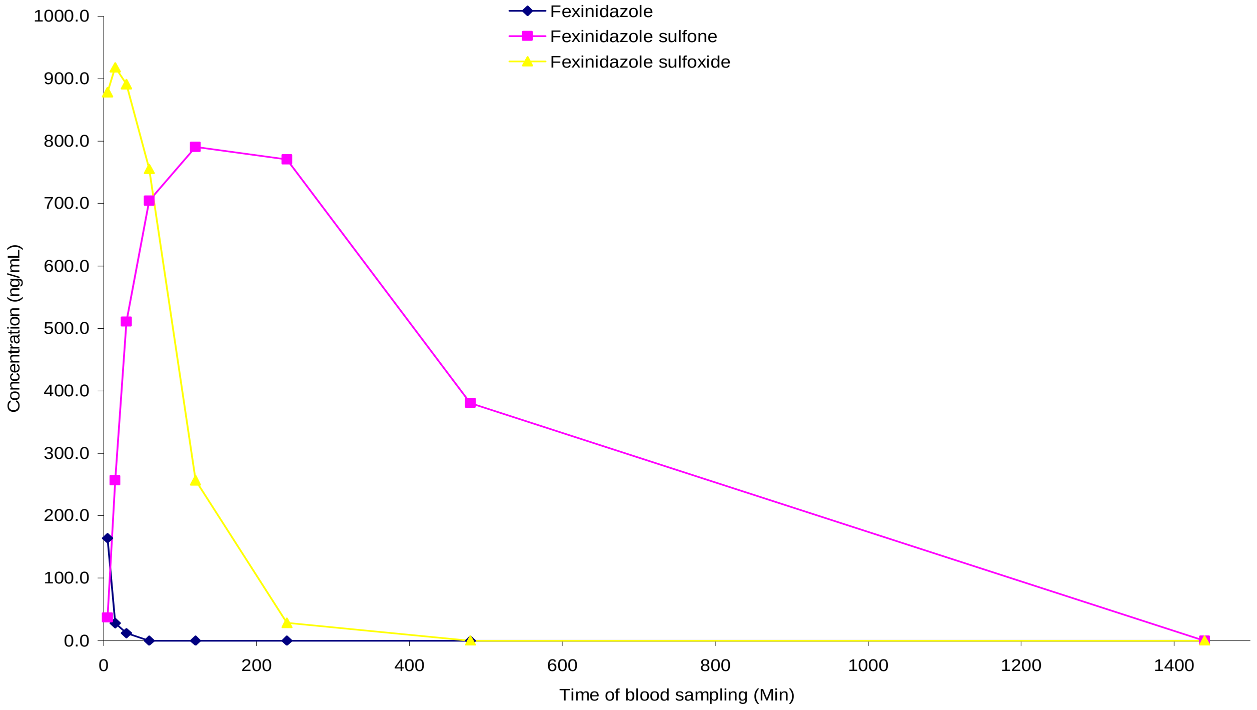

Figure 2 **Linear plot of mean Fexinidazole, sulfone and sulfoxide concentrations in plasma following oral administration of Fexinidazole at 25 mg/kg to female NMRI mice**

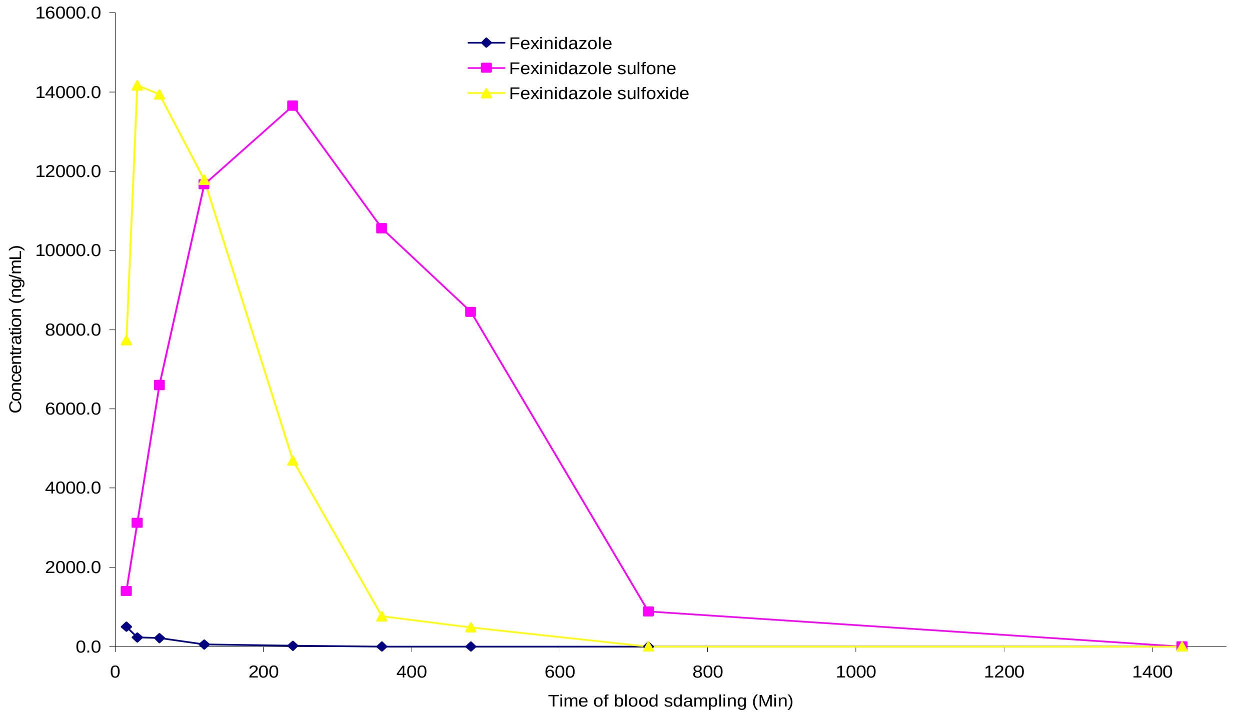

6.3 Brain results

Following intravenous administration of Fexinidazole at 1 mg/kg Fexinidazole was shown to have crossed the blood brain barrier with a mean concentration of 277.4 ng/g (per gram of homogenised brain) at 5 minutes post-dose. This had declined to 36.2 ng/g by 30 minutes post-dose.

Following oral administration of Fexinidazole at 25 mg/kg Fexinidazole was shown to have crossed the blood brain barrier following oral administration with a mean concentration of 1136.4 ng/g at 15 minutes post-dose. By 60 minutes post-dose this had declined to 763.3 ng/g.

Table 8 Mean plasma:brain ratios of Fexinidazole, Sulfone and Sulfoxide

IV

Sample time (mins)	Plasma	Brain Fexinidazole	Brain Sulfone	Brain Sulfoxide
**5**	1.00	0.56	0	0.045
**15**	1.00	0.76	NST	NST
**30**	1.00	1.03	0	0.064

PO

Sample time (mins)	Plasma	Brain Fexinidazole	Brain Sulfone	Brain Sulfoxide
**15**	1.00	0.76	NST	NST
**30**	1.00	1.16	0.05	0.078
**60**	1.00	1.22	0.06	0.12

NST = no sample taken

Table 9 Mean brain concentrations of Fexinidazole, sulfone and sulfoxide following intravenous and oral administration of Fexinidazole at 1 and 25 mg/kg respectively to female NMR1 mice

IV

	Mean Concentration ng/mL (ng/g)		
**Timepoint**	**Fexinidazole**	**Sulfone**	**Sulfoxide**
**5**	92.5 (277.4)	BLQ	39.9 (119.6)
**15**	21.3 (63.8)	NST	NST
**30**	12.1 (36.2)	BLQ	57.6 (172.7)

PO

	Mean Concentration ng/mL (ng/g)		
**Timepoint**	**Fexinidazole**	**Sulfone**	**Sulfoxide**
**15**	378.8 (1136.4)	NST	NST
**30**	266.5 (799.5)	155.9 (467.8)	1104.9 (3314.7)
**60**	254.4 (763.3)	394.2 (1182.6)	1624.4 (4873.1)

NST = no sample taken

Table 10 **Brain concentrations of Fexinidazole following intravenous and oral administration of Fexinidazole at 1 and 25 mg/kg respectively to female NMR1 mice**

IV

	**Concentration of brain homogenate ng/mL (ng/g)**					
**Time (min)**		**Animal**		**Animal**		**Animal**
**5**	81.9 (254.7)	103	75.8 (227.4)	104	119.7 (359..1)	105
**15**	22.2 (66.6)	106	21.4 (64.2)	107	20.2 (60.6)	108
**30**	13.2 (39.6)	109	11.2 (33.6)	110	11.8 (35.4)	111

PO

	**Concentration of brain homogenate ng/mL (ng/g)**					
**Time (min)**		**Animal**		**Animal**		**Animal**
**15**	381.4 (1144.2)	127	359.6 (1078.8)	128	395.4 (1186.2)	129
**30**	286.1 (858.3)	130	282.5 (847.5)	131	230.9 (692.7)	132
**60**	288.0 (864.0)	133	229.4 (688.2)	134	245.9 (737.7)	135

LLOQ = 5 ng/mL or 15 ng/g

Table 11 **Brain concentrations of sulfoxide following intravenous and oral administration of Fexinidazole at 1 and 25 mg/kg respectively to female NMR1 mice**

IV

	**Concentration of brain homogenate ng/mL (ng/g)**					
**Time (min)**		**Animal**		**Animal**		**Animal**
**5**	37.3(111.9)	103	40.2 (120.6)	104	42.1 (126.3)	105
**30**	51.1 (153.3)	109	59.3 (177.9)	110	62.3 (186.9)	111

PO

	**Concentration of brain homogenate ng/mL (ng/g)**					
**Time (min)**		**Animal**		**Animal**		**Animal**
**30**	1351.9 (4055.7)	130	1473.9 (44.21.7)	131	488.9 (1466.7)	132
**60**	2305.8 (6917.4)	133	776.2 (2328.6)	134	1791.1 (5373.3)	135

LLOQ = 5 ng/mL or 15 ng/g

Table 12 Brain concentrations of sulfone following intravenous and oral administration of Fexinidazole at 1 and 25 mg/kg respectively to female NMR1 mice

IV

	**Concentration of brain homogenate ng/mL (ng/g)**					
**Time (min)**		**Animal**		**Animal**		**Animal**
**5**	BLQ (BLQ)	103	BLQ (BLQ)	104	BLQ (BLQ)	105
**30**	BLQ (BLQ)	109	BLQ (BLQ)	110	BLQ (BLQ)	111

PO

	**Concentration of brain homogenate ng/mL (ng/g)**					
**Time (min)**		**Animal**		**Animal**		**Animal**
**30**	207.2 (621.6)	130	189.4 (568.2)	131	71.2 (213.6)	132
**60**	477.7 (1433.1)	133	273.3 (819.9)	134	431.6 (1294.8)	135

LLOQ = 25 ng/mL or 75 ng/g

Figure 3 **Brain concentrations of Fexinidazole and its sulfone and sulfoxide metabolites following intravenous administration of Fexinidazole at a dose of 1 mg/kg**

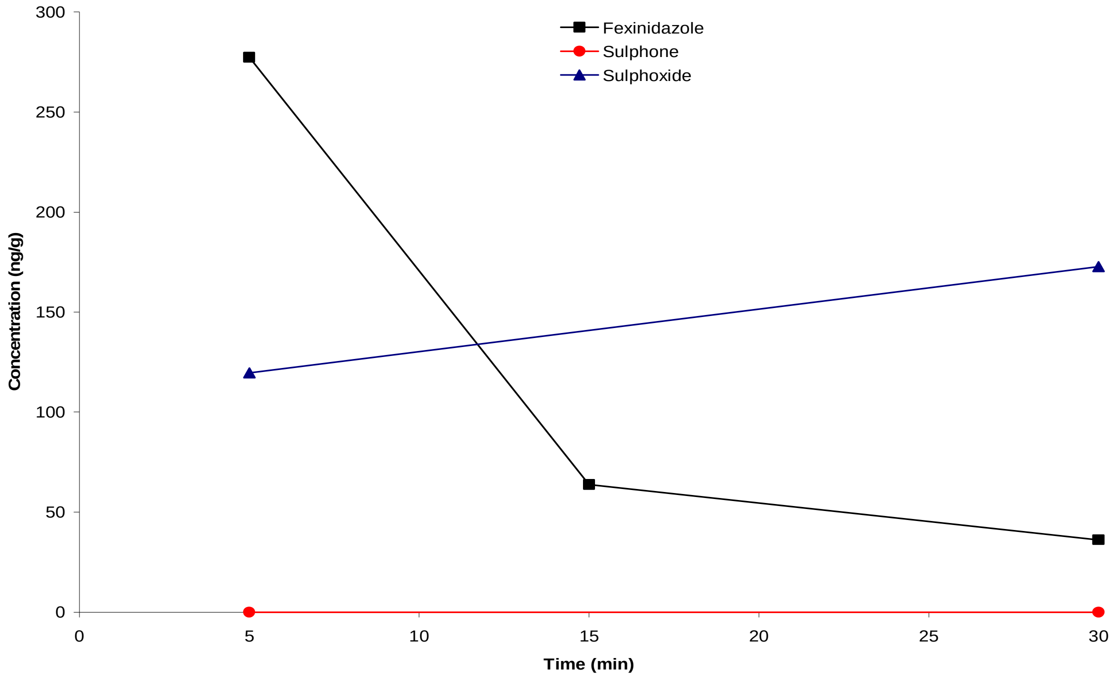

Figure 4 **Brain concentrations of Fexinidazole and its sulfone and sulfoxide metabolites following oral administration of Fexinidazole at a dose of 25 mg/kg.**

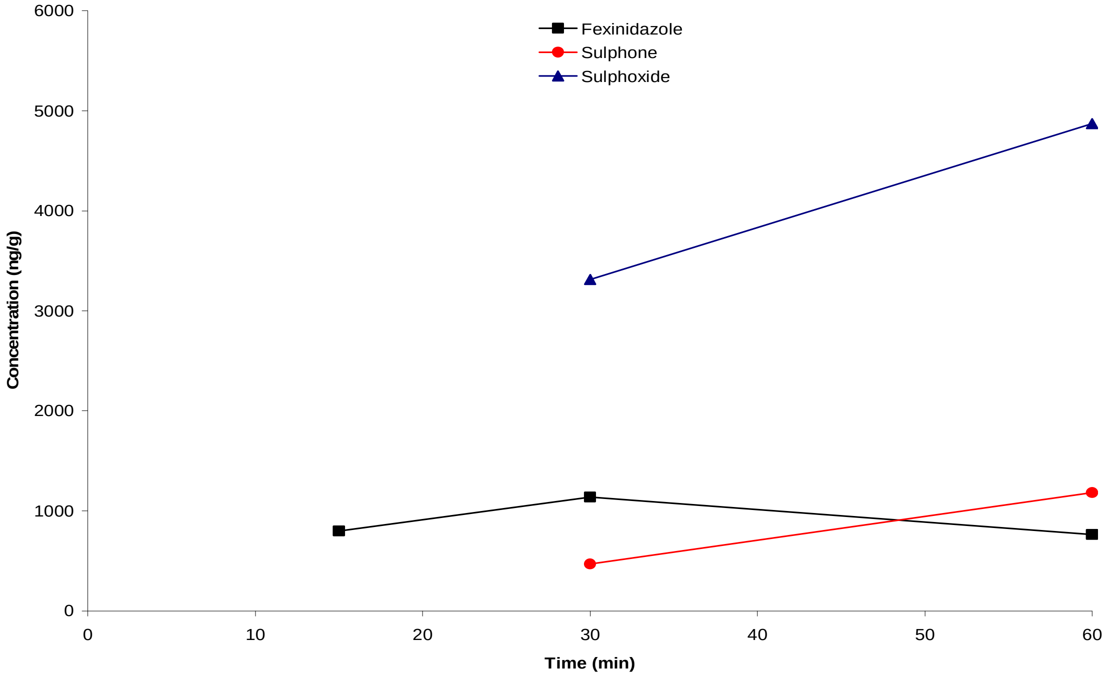

Appendix 1 Animal body weights and dose volumes

Group No.	Treatment	Animal ID	Weight (g)	Dose Vol. (mL)
1	Fexinidazole 1 mg/kg i.v.	103	23	0.23
		104	22	0.22
		105	25	0.25
		106	31	0.31
		107	27	0.27
		108	30	0.30
		109	31	0.31
		110	30	0.30
		111	25	0.25
		112	31	0.31
		113	26	0.26
		114	36	0.36
		115	34	0.34
		116	25	0.25
		117	35	0.35
		118	20	0.20
		119	25	0.25
		120	24	0.24
		121	31	0.31
		122	27	0.27
		123	31	0.31
		124	26	0.26
		125	20	0.20
		126	22	0.22

Appendix 1 cont.

Group No.	Treatment	Animal ID	Weight (g)	Dose Vol. (mL)
2	Fexinidazole 25 mg/kg p.o.	127	29	0.29
		128	31	0.31
		129	28	0.28
		130	34	0.34
		131	26	0.26
		132	31	0.31
		133	33	0.33
		134	31	0.31
		135	29	0.29
		136	28	0.28
		137	30	0.30
		138	32	0.32
		139	27	0.27
		140	31	0.31
		141	38	0.38
		142	31	0.31
		143	28	0.28
		144	30	0.30
		145	25	0.25
		146	23	0.23
		147	23	0.23
		148	29	0.29
		149	31	0.31
		150	25	0.25
