# Supplementary material for: Fexinidazole – A New Oral Nitroimidazole Drug Candidate Entering Clinical Development for the Treatment of Sleeping Sickness
Source: PLoS Negl Trop Dis. 2010 Dec 21;4(12):e923. doi: 10.1371/journal.pntd.0000923 (PMC3006138; doi:10.1371/journal.pntd.0000923)

Fexinidazole: Evaluation of the Pharmacokinetics and of the Bioavailability after Single IV and Oral Administration to Male Sprague Dawley Rats.

Product Name:	Fexinidazole
Study Number:	0327-2007
Study Director:	
Status:	FINAL

TABLE OF CONTENTS

1. STUDY CONDUCT 4
2. OBJECTIVE 4
3. ABBREVIATIONS 4
4. METHODS 5
4.1. Study Design 5
4.2. Sample Collection and Handling 5
4.3. Bioanalytical Method 5
4.4. Pharmacokinetic Calculations 5
5. RESULTS 6
6. CONCLUSIONS 6
7. CONTRIBUTORS 7
8. ARCHIVING 7
9. REFERENCES 7
TABLES AND FIGURES 8
APPENDICES
Appendix 1. Individual plasma concentrations 12
Appendix 2. Bioanalytical method 13
Appendix 3. Analytical performance 14

1. STUDY CONDUCT

The study, sponsored by Drugs for Neglected Diseases *initiative* (DND*i*), was performed within Accelera, Nerviano Medical Sciences, Italy according the internal Standard Operating Procedures as a non-GLP regulated study.

2. OBJECTIVE

The objective of this study was to evaluate the pharmacokinetics and absolute bioavailability of Fexinidazole after single IV 5 mg/kg and oral 10 mg/kg doses of the compound to male Sprague Dawley rats.

3. ABBREVIATIONS

The following abbreviations are used in this document:

$AUC_{0-t(last)}$	Area under the plasma concentration *vs*. time curve up to finite time
$AUC_{0-\infty}$	Area under the plasma concentration *vs*. time curve up to infinite time
$C_{0.033}$	Concentration at 2 minutes after IV dosing, i.e. maximal concentration
C_{max}	Maximal plasma concentration
CV	Coefficient of variation of the mean
F	Absolute bioavailability
h	Hours
ID	Animal identification code
IV	Intravenous
LC	Liquid chromatography
LLOQ	Lower limit of quantification
MS	Mass-spectrometry
Norm	Normalized value
R^2	Correlation coefficient
SD	Standard deviation of the mean
SOP	Standard operating procedure
STD	Standard sample
$t_{1/2,z}$	Terminal half-life
t_{max}	Time to peak plasma concentration
t_{last}	Time of the last detectable concentration
ULOQ	Upper limit of quantification

4. METHODS

4.1. Study Design

The study was conducted according to the study protocol [1].
Fexinidazole was given as IV and oral administrations to male Sprague Dawley rats according to the following scheme

Rat ID	Route	Dose (mg/kg)	Formulation
R4, R5, R6	IV	5	70 % PEG 400 in 5 % dextrose solution
R1, R2, R3	Oral	10	5 % Tween 80 and 0.5% Methocel suspension

IV dose was administered *via* the tail vein, the oral dose was administered by gastric gavage.

4.2. Sample Collection and Handling

About 3 days before dosing and while under anesthesia, animals were fitted with a cannula implanted in the superior vena cava *via* the jugular vein. On the day of dosing, approximately 0.3 mL of blood/sampling time were taken from the cannula using heparinized syringes, transferred immediately into tubes (pre-cooled in an ice/water bath) and centrifuged at 10000g for 3 minutes at 4°C. The separated plasma was split into two aliquots, then stored at -80°C until analysis. Blood was taken at 0.033 (2 minutes), 0.25, 0.5, 1, 2, 4 and 6 h after IV dosing, at 0.25, 0.5, 1, 2, 4, 6 and 8 h after oral dosing.

4.3. Bioanalytical Method

Plasma concentrations of Fexinidazole were determined by a LC-MS-MS method. The lower limit of the bioanalytical method was 5.12 ng/mL. Bioanalytical data were stored in Watson LIMS (v. 6.4.0.04, Thermo Fisher Scientific, Waltham, MA, USA). Details of the bioanalytical method were reported in Appendix 2 and the analytical performance was reported in Appendix 3.

4.4. Pharmacokinetic Calculations

Pharmacokinetic evaluation was carried out using non-compartmental approach with the aid of the Watson package (v. 6.4.0.04, Thermo Fisher Scientific, Waltham, MA, USA).
For the calculations, pre-dose concentrations were set equal to $C_{0.033}$ and equal to zero after IV and oral administrations, respectively.
After IV dosing, $C_{0.033}$, maximal concentration, was read from raw plasma data; after oral dosing, C_{max} and t_{max} were read from raw data as the coordinates of the highest measured concentration. After both doses, the area under plasma concentration *vs.* time curve to finite time, $AUC_{0\text{-}t(last)}$, was determined by the linear trapezoidal rule up to the last detectable concentration. The half-life of the terminal phase, $t_{1/2,z}$, was determined by linear regression analysis of the natural-log concentration *vs.* time curve, where $t_{1/2,z}$ = ln(2)/slope of the regression line. The area under the concentration vs. time curve up to infinite time, $AUC_{0\text{-}\infty}$, was determined as

$$AUC0\text{-}\infty = AUC0 - t(last) + \frac{Ct(last) \cdot t_{1/2,z}}{\ln(2)}$$

After IV dosing, plasma clearance and volume of distribution at steady state were calculated as follows:
CL = Dose/$AUC_{0-\infty}$
V_{ss} = CL·MRT, where MRT is the mean residence time.

After both doses, C_{max} and AUC values were also normalized to a 1 mg/kg dose level.

Absolute bioavailability was calculated from the ratio of the average oral to IV dose-normalized $AUC_{0-\infty}$ values.

Descriptive statistics (mean ± SD, %CV) were reported for plasma concentrations and pharmacokinetic parameters sorted by route of dosing.

Plasma concentrations and pharmacokinetic parameters of Fexinidazole were reported to three significant figures.

5. RESULTS

Mean ±SD systemic exposure parameters of Fexinidazole after IV and oral administrations of the compound are reported in Table 1. Individual and mean pharmacokinetic parameters of Fexinidazole after IV and oral administrations are reported in Tables 2 and 3, respectively. Individual and mean plasma concentrations of the compound are plotted in Figures 1 - 3 and reported in Table 1A1 of Appendix 1.

After IV administration, the rat ID R5 showed red urine half-an hour post dosing. This side effect disappeared at 1 h post dosing.
After IV administration, mean ±SD $C_{0.033}$ value of Fexinidazole was 3660 ±217 ng/mL. The plasma profile of the compound showed a poly-exponential decline (Figure 3) with mean ±SD terminal half-life of 1.71 ±0.3 h. Mean ±SD systemic clearance, 76 ±2 mL/min/kg, was higher than rat hepatic blood flow (69 mL/min/kg, [3]), indicating high rate of elimination of the compound from the systemic circulation. Mean ±SD volume of distribution at steady state, 4690 ±410 mL/kg, was about seven times higher than rat total body water (about 700 mL/kg [4]), suggesting extensive tissue distribution.

After oral administration, detectable concentrations of Fexinidazole were measured at the first sampling time (0.25 h). Mean ±SD maximal plasma concentration of Fexinidazole was 147 ±37 ng/mL, achieved 2 h post dosing. The concentrations of the compound were detectable up to the end of the experiment (8 h). Mean ±SD apparent terminal half-life of the compound was 1.72 ±0.07 h, comparable to that after IV dosing. Mean ±SD $AUC_{0-\infty}$ was 662 ±106 ng·h/mL. Absolute bioavailability was 30 %.

6. CONCLUSIONS

After both doses, low variability of the pharmacokinetic parameters of Fexinidazole was observed.

The results of the experiment indicated that the compound was endowed with a high clearance and volume of distribution. The terminal half-life was short and similar between the two routes of administration. The oral bioavailability of Fexinidazole was 30 %. Notwithstanding the high clearance, Fexinidazole showed a relatively high bioavailability. The reason of this discrepancy can be due to a saturation of hepatic first-pass metabolism or, in addition to the hepatic metabolism, other route(s) of elimination can play a significant role in the extent of plasma clearance of the compound.
After oral dosing, normalized to 1 mg/kg $AUC_{0-\infty}$ (65 ng·h/mL) obtained in the present experiment was slightly higher than the corresponding one (45 ng·h/mL) obtained in a previous study in the rat (report no. 0259-2007-R, [2]).

7. CONTRIBUTORS

8. ARCHIVING

The protocol, raw data, pharmacokinetic analysis and final report were archived within Accelera Archive, Nerviano Medical Sciences, Italy, according the Unit Standard Operating Procedures.

9. REFERENCES

1. Fexinidazole: Evaluation of the Pharmacokinetics and of the Bioavailability after Single IV and Oral Administration to male Sprague Dawley rats. Nerviano Medical Sciences Study Protocol 0327-2007-P, September 25, 2007.

2. Fexinidazole: Evaluation of the Pharmacokinetics and of the Relative Bioavailability after Single Oral Administration of different Formulations of the Compound to male Sprague Dawley rats. Nerviano Medical Sciences Study Report 0259-2007-R, October 17, 2007.

3. Boxenbaum, H. Interspecies variation in liver weight, hepatic blood flow, and antipyrine intrinsic clearance: Extrapolation of data to benzodiazepines and phenytoin. J. Pharmacokin. Biopharm. 8: 165-176, 1980.

4. Spector, W. S. Handbook of Biological Data. Philadelphia, W.B. Saunders Co., 1956.

TABLES AND FIGURES

Table 1. Summary table of mean ±SD systemic exposure values of Fexinidazole after single IV 5 mg/kg and oral 10 mg/kg doses of the compound in male Sprague Dawley rats.

Route	C_{max} (ng/mL)	$AUC_{0-\infty}$ (ng·h/mL)	F (%)
IV	3660 ± 217	1200 ± 26.5	
Oral	147 ± 36.6	662 ± 106	29.7

Table 2. Individual and mean (±SD, %CV) pharmacokinetic parameters of Fexinidazole after single IV 5 mg/kg dose of the compound in male Sprague Dawley rats.

Parameter (Units)	ID			Mean	SD	%CV
	R4	R5	R6			
Weight (g)	265	268	271	268	3	1
Actual Dose (mg/kg)	5.6	5.5	5.4	5.5	0.0651	1
$C_{0.033}$ (ng/mL)	3690	3430	3860	3660	217	6
t_{last} (h)	6	6	6	6	0	0
$AUC_{0-t(last)}$ (ng·h/mL)	1190	1130	1160	1160	30	3
Regression Range (h)	2 - 6	2 - 6	2 - 6	N/A	N/A	N/A
$t_{1/2,z}$ (h)	1.51	1.6	2.03	1.71	0.278	16
$AUC_{0-\infty}$ (ng·h/mL)	1210	1170	1220	1200	26.5	2
CL (mL/min/kg)	76.4	78.2	74	76.2	2.11	3
V_{ss} (mL/kg)	4250	4770	5060	4690	410	9
$C_{0.033}$, norm (1)	665	625	712	667	43.5	7
$AUC_{0-t(last)}$, norm (1)	214	207	215	212	4.36	2
$AUC_{0-\infty}$, norm (1)	218	213	225	219	6.03	3

N/A: not applicable

(1) $C_{0.033}$ (ng/mL) and AUC (ng·h/mL) values normalized to 1 mg/kg dose.

Table 3. Individual and mean (±SD, %CV) pharmacokinetic parameters of Fexinidazole after single oral 10 mg/kg dose of the compound in male Sprague Dawley rats.

Parameter (Units)	ID			Mean	SD	%CV
	R1	R2	R3			
Weight (g)	273	276	277	275	2	1
Actual Dose (mg/kg)	10.3	10.1	10.1	10.2	0.0794	1
C_{max} (ng/mL)	182	109	151	147	36.6	25
t_{max} (h)	2	2	2	2	0	0
t_{last} (h)	8	8	8	8	0	0
$AUC_{0-t(last)}$ (ng·h/mL)	710	524	659	631	96.1	15
Regression Range (h)	2 - 8	2 - 8	2 - 8	N/A	N/A	N/A
$t_{1/2,z}$ (h)	1.78	1.73	1.64	1.72	0.0709	4
$AUC_{0-\infty}$ (ng·h/mL)	754	546	687	662	106	16
C_{max}, norm (1)	17.7	10.7	14.9	14.4	3.52	24
$AUC_{0-t(last)}$, norm (1)	69.2	51.7	65.2	62	9.17	15
$AUC_{0-\infty}$, norm (1)	73.5	53.8	68	65.1	10.2	16
F% $AUC_{0-\infty}$				29.7		

N/A: not applicable

(1) C_{max} (ng/mL) and AUC (ng·h/mL) values normalized to 1 mg/kg dose.

Figure 1. Individual plasma concentrations (ng/mL) of Fexinidazole after single IV 5 mg/kg dose of the compound in male Sprague Dawley rats.

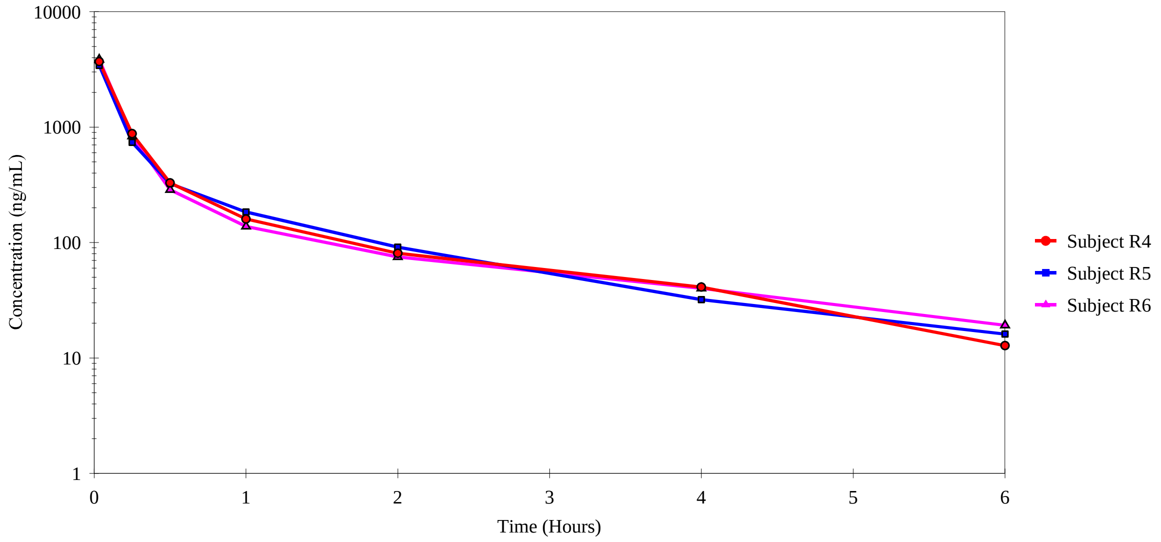

Figure 2. Individual plasma concentrations (ng/mL) of Fexinidazole after single oral 10 mg/kg dose of the compound in male Sprague Dawley rats.

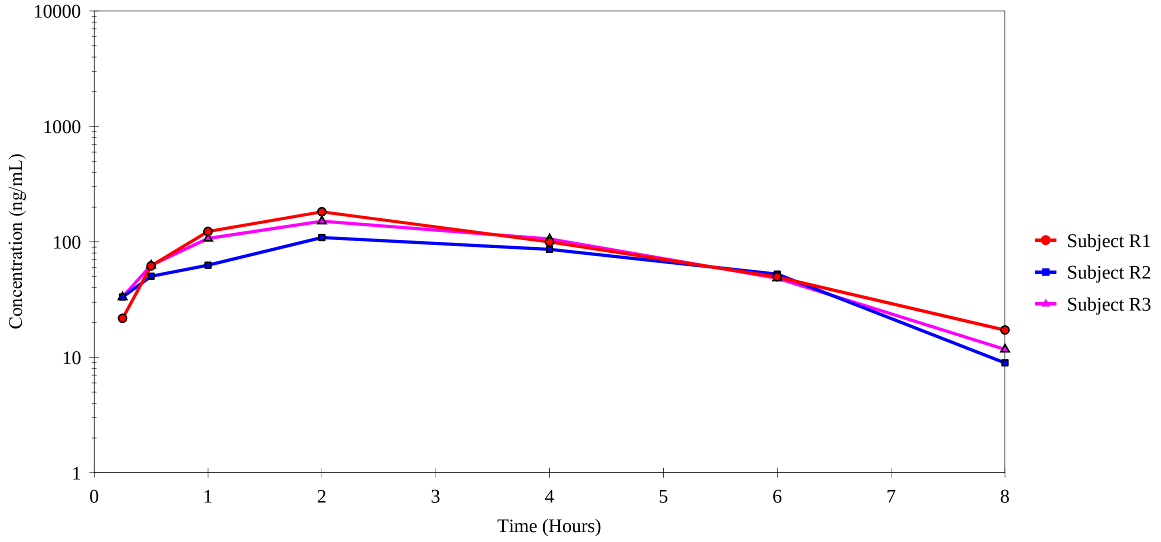

Figure 3. Mean (±SD) plasma concentrations (ng/mL) of Fexinidazole after single IV 5 mg/kg and oral 10 mg/kg doses of the compound in male Sprague Dawley rats.

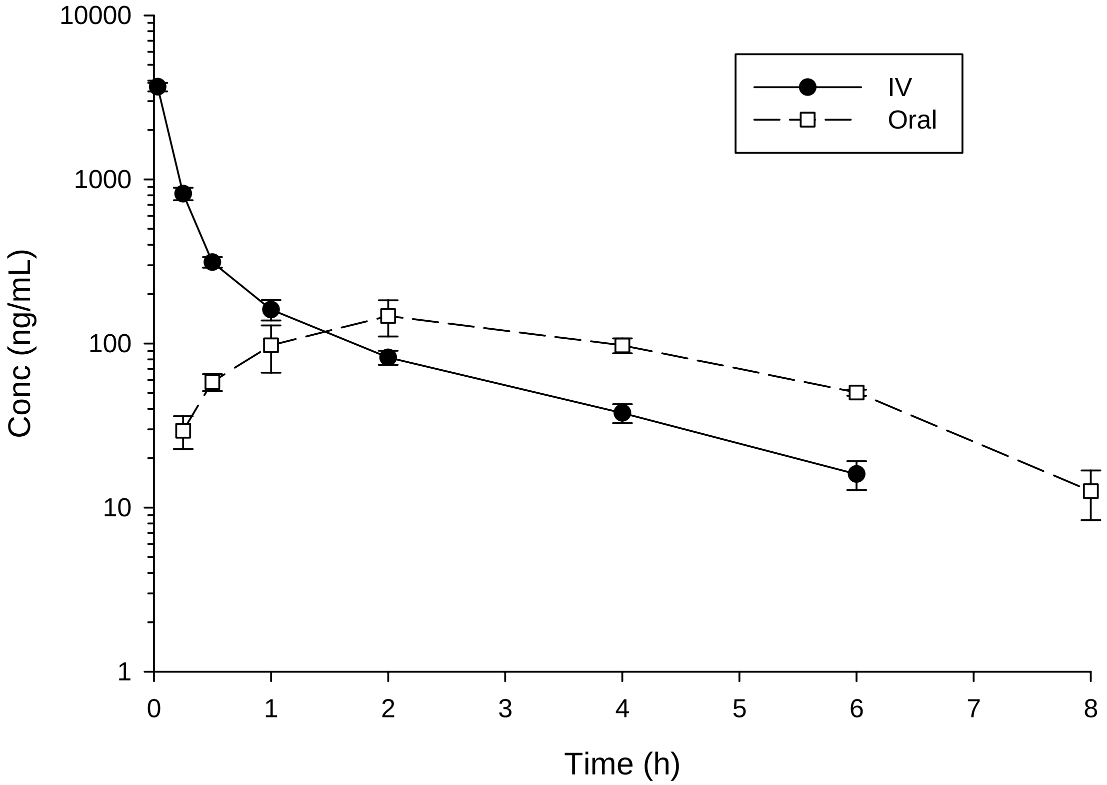

APPENDICES

Appendix 1. Individual plasma concentrations

Table 1A1. Individual and mean (±SD, %CV) plasma concentrations (ng/mL) of Fexinidazole after single IV 5 mg/kg and oral 10 mg/kg doses of the compound in male Sprague Dawley rats.

IV						
Time (h)	ID R4	ID R5	ID R6	Mean	SD	%CV
0.033	3690	3430	3860	3660	217	6
0.25	877	738	836	817	71.4	9
0.5	329	324	287	313	22.9	7
1	160	184	138	161	23	14
2	80.7	91.2	75.1	82.3	8.17	10
4	41.1	32	40.1	37.7	4.99	13
6	12.8	16.1	19.2	16	3.2	20
Oral						
Time (h)	ID R1	ID R2	ID R3	Mean	SD	%CV
0.25	21.7	33.2	33.2	29.4	6.64	23
0.5	61.9	50.3	62.5	58.2	6.88	12
1	123	62.8	107	97.6	31.2	32
2	182	109	151	147	36.6	25
4	99.9	86.1	106	97.3	10.2	11
6	49.6	52.5	48.4	50.2	2.11	4
8	17.2	8.94	11.7	12.6	4.21	33

Appendix 2. Bioanalytical method

Fexinidazole (free base) was assayed using an LC-MS-MS method. Bioanalytical data are stored in Watson LIMS under Watson study 0327-2007 in the 348-Fexinidazole project.

Plasma Sample Preparation
Standards were prepared using rat plasma. Plasma proteins were precipitated by adding 200 μL of methanol containing (2H3)-Fexinidazole as internal standard at a concentration of about 3 ng/mL to 25 μL of plasma in a 96 well plate. After capping and vortex mixing, the plate was centrifuged for 10 minutes at 4000 rpm at 6°C. An aliquot of 100 μL of supernatant was transferred in a 96 well plate and mixed with 100 μL of 10 mM ammonium formate pH 3.5 injected onto the LC-MS-MS system.

LC-MS/MS conditions

HPLC system: Hewlett Packard 1100 series
Mobile phase: Channel A: Ammonium Formate (10 mM pH 3.5)
Channel B: Methanol

Elution mode: ISOCRATIC

Elution conditions

Time (min)	0.0	4.0				
% A	40	40				
% B	60	60				

Total Run Time: 4.0 minutes
Flow rate: 1.0 mL/min
Approximate retention time: Fexinidazole: about 2.3 min.
(2H3)-Fexinidazole: about 2.28 min
Column oven temp.: 30 °C
Analytical column: Chromolith RP-18 50 * 4.6 mm (Merck)

Autosampler type: Perkin Elmer PE 200
Injection volume: 20 μL
Autosampler temperature: RT

MS instrument: Perkin Elmer SCIEX API 4000
Ionisation: TURBO ION SPRAY in positive ion mode
MRM transitions: Fexinidazole: m/z 280.2 m/z 140.2
(2H3)-Fexinidazole m/z 283.2 m/z 143.2
Resolution Q1: Unit
Q3: Unit
LLOQ: 5.12 ng/mL

ULOQ: 955 ng/mL

Batch No. of standard: N/A

Software used

Acquisition and processing: Analyst 1.4

Import data from Analyst file: 0327-2007-Run2.rdb, 0327-2007-Run3.rdb

Data file in Analyst: 0327-2007\Run2.wiff, 0327-2007\Run3.wiff

Appendix 3. Analytical performance

Table 1A3. Analytical Performance: Back-Calculated Concentrations (ng/mL) of Fexinidazole Calibration Standard in Rat Plasma for Study Protocol 0327-2007.

Assay Date	Analytical Run Number	STD.1 5.12 ng/mL	STD.2 50.0 ng/mL	STD.3 95.5 ng/mL	STD.4 500 ng/mL	STD.5 955 ng/mL	STD.6 5100 ng/mL
22-Oct-2007	2	4.81	54.9	97.8	446	936	*2860
		5.34	53.3	*112	490	933	*2860
23-Oct-2007	3	4.88	55.5	97.8	489	922	*2650
		5.29	50.7	*116	477	924	*2800
Mean		5.08	53.6	97.8	476	929	
SD		0.274	2.14	0	20.5	6.8	
%CV		5.4	4	0	4.3	0.7	
%Bias		-0.8	7.2	2.4	-4.8	-2.7	
n		4	4	2	4	4	

* Reason Deactivated : Accuracy more than 15%. Since both high calibration standards have been excluded from regression analysis the ULOQ of the run has been lowered to STD.5 (i.e. 955 ng/mL).

Table 2A3. Calibration Curve Parameters for Fexinidazole Calibration Standards in Rat Plasma for Study Protocol 0327-2007.

Run Date	Curve Number	Slope	Intercept	R^2	LLOQ ng/mL	ULOQ ng/mL
22-Oct-2007	2	0.141682	0.137233	0.9948	5.12	955
23-Oct-2007	3	0.152999	0.045528	0.9967	5.12	955
Mean		0.147341	0.091381	0.9958		
SD		0.0080023	0.064845	0.0013		
%CV		5.4	71	0.1		
n		2	2	2		

Amendment 1

Fexinidazole: Evaluation of the Pharmacokinetics and of the Bioavailability after Single IV and Oral Administration to Male Sprague Dawley Rats.

Study Number	0327-2007
Document Number	0327-2007-R
Amendment Number:	1
Test Article:	Fexinidazole
Study Director:	

1. SPECIFIC CHANGE(S)

1.1. Description(s) of Change(s)

After specific request of the Sponsor, the additional stored plasma aliquots of the blood samples were used to evaluate the pharmacokinetics of the sulphone and sulphoxide metabolites of Fexinidazole.

The following Appendix 4 refers to the evaluation of the pharmacokinetics of the sulphone and sulphoxide metabolites of Fexinidazole and has to be intended as integration of the report 0327-2007-R. The report 0327-2007-R was not changed.

1.1.1. Reason(s) for Change(s)

Analysis was performed on the additional plasma aliquots to evaluate the pharmacokinetics of the sulphone and sulphoxide metabolites of Fexinidazole.

Appendix 4. Evaluation of the pharmacokinetics of sulphone and sulphoxide metabolites of Fexinidazole

TABLE OF CONTENTS

APPROVAL SIGNATURES....2
1. SPECIFIC CHANGE(S)....3
1.1. Description(s) of Change(s)....3
1.1.1. Reason(s) for Change(s)....3
2. ABBREVIATIONS....5
3. METHODS....5
3.1. Bioanalytical Method....5
3.2. Pharmacokinetic Calculations....5
4. RESULTS....6
4.1. IV dosing....6
4.2. Oral dosing....6
5. CONCLUSIONS....7
6. CONTRIBUTORS....7
7. ARCHIVING....7
TABLES AND FIGURES....8

2. ABBREVIATIONS

The following abbreviations are used in this document:

$AUC_{0-t(last)}$	Area under the plasma concentration *vs.* time curve up to finite time
C_{max}	Maximal plasma concentration
CV	Coefficient of variation of the mean
h	Hours
ID	Animal identification code
IV	Intravenous
LC	Liquid chromatography
LLOQ	Lower limit of quantification
MS	Mass-spectrometry
Norm	Normalized value
R^2	Correlation coefficient
SD	Standard deviation of the mean
STD	Standard sample
t_{max}	Time to peak plasma concentration
t_{last}	Time of the last detectable concentration
ULOQ	Upper limit of quantification

3. METHODS

3.1. Bioanalytical Method

Plasma concentrations of the sulphone and sulphoxide Fexinidazole metabolites were determined by a LC-MS-MS method. The lower limit of the bioanalytical method was 5.00 ng/mL for both compounds. Bioanalytical data were stored in Watson LIMS (v. 6.4.0.04, Thermo Fisher Scientific, Waltham, MA, USA). Details of the bioanalytical method are reported in Table 1 and the analytical performances of calibration standards are reported in Tables 2-5.

3.2. Pharmacokinetic Calculations

Pharmacokinetic evaluations of the sulphone and sulphoxide metabolites were carried out using non-compartmental approach with the aid of the Watson package (v. 6.4.0.04, Thermo Fisher Scientific, Waltham, MA, USA).
After both doses, for the calculations, the pre-dose concentrations of the metabolites were set equal to zero.
C_{max} and t_{max} were read from raw data as the coordinates of the highest measured concentration. The area under plasma concentration *vs.* time curve up to finite time, $AUC_{0-t(last)}$, was determined by the linear trapezoidal rule up to the last detectable concentration.

Metabolite to parent ratio was calculated based on C_{max} and $AUC_{0-t(last)}$ values.

C_{max} and $AUC_{0-t(last)}$ values of both compounds were also normalized to a 1 mg/kg dose level.

Descriptive statistics (mean ±SD, %CV) were reported for plasma concentrations and pharmacokinetic parameters of both compounds sorted by route of dosing.

Plasma concentrations and pharmacokinetic parameters of both compounds were reported to three significant figures.

4. RESULTS

Individual and mean plasma concentrations of the sulphone and sulphoxide metabolites of Fexinidazole are reported in Tables 6 and 7, respectively, whilst the corresponding parameters are reported in Tables 8 and 9. Individual plasma concentrations of the sulphone and sulphoxide metabolites are plotted in Figures 1 and 2, respectively, whilst the corresponding mean concentrations with those of the parent compound are reported in Figures 3 and 4.

4.1. IV dosing

After dosing, detectable concentrations of the sulphone metabolite were measured at the first sampling time (2 minutes post dosing). Mean maximal concentration of the compound was 2087 ng/mL, achieved, on average, 5 h post dosing. Detectable concentrations of the compound were measured up to the last sampling time (6 h post dosing). The mean $AUC_{0-t(last)}$ was 9590 ng·h/mL. The metabolite to parent $AUC_{0-t(last)}$ ratio was, on average, 8, indicating that Fexinidazole was extensively metabolised to its sulphone metabolite.

The plasma profile of the sulphoxide metabolite was different from that of the sulphone metabolite (Figure 3). Sulphoxide maximal concentration was, on average, 3067 ng/mL, and was achieved 1 h post dosing. Detectable concentrations of the compound were measured up to the last sampling time. The mean $AUC_{0-t(last)}$ value was 9440 ng·h/mL. The metabolite to parent $AUC_{0-t(last)}$ ratio was, on average, 8, indicating that Fexinidazole was extensively metabolised to its sulphoxide metabolite.

4.2. Oral dosing

After dosing, the maximal concentration of the sulphone metabolite was, on average, 2660 ng/mL, and was achieved 6 h post dosing. Detectable concentrations of the compound were measured up to the last sampling time (8 h post dosing). The $AUC_{0-t(last)}$ value was, on average, 14300 ng·h/mL. The metabolite to parent $AUC_{0-t(last)}$ ratio was, on average, 23.

The plasma profile of the sulphoxide metabolite was different from that of the sulphone metabolite but similar to that of the parent compound (Figure 4). The mean maximal concentration of the sulphoxide was 3520 ng/mL, achieved 2 h post dosing. Detectable concentrations of the compound were measured up to the last sampling time. The mean $AUC_{0-t(last)}$ value was 16200 ng·h/mL. The metabolite to parent $AUC_{0-t(last)}$ ratio was, on average, 26.

5. CONCLUSIONS

After both doses, the coefficient of variation of the mean systemic exposure parameters of both metabolites was low, being at most 25 %.
After both IV and oral dosing, Fexinidazole was extensively metabolized to the sulphone and sulphoxide derivatives. For both metabolites, the metabolite to parent ratio was higher after oral than after IV dosing, suggesting extensive first pass metabolism of Fexinidazole after oral dosing.

6. CONTRIBUTORS

7. ARCHIVING

The protocol, raw data, pharmacokinetic analysis and final report were archived within Accelera Archive, Nerviano Medical Sciences, Italy, according the Unit Standard Operating Procedures.

TABLES AND FIGURES

Table 1. Bio-analytical method.

Plasma Sample Preparation:
Standards were prepared using rat plasma. Plasma proteins were precipitated by adding 200 μL of methanol containing 100 ng/ml of [$^2H_3$] Fexinidazole as Stable Labeled Internal Standard (SLIS) to 25 μL of plasma in a 96 well plate. After capping and vortex mixing, the plate was centrifuged for 10 minutes at 4000 rpm at 6°C. Aliquots of 100 μL of supernatant were transferred in a clean 96 well plate and mixed with 100 μL of 10 mM ammonium formate pH 3.5. Aliquots of 10 μL were then injected onto the LC-MS-MS system.
LC-MS/MS conditions:

HPLC system:	Hewlett Packard 1100 series
Mobile phase	Channel A: Ammonium Formate (10 mM pH 3.5)<br>Channel B: Methanol
Elution mode	Gradient
Elution conditions	(see gradient table below)

Time (min)	0.0	2.10	2.30	5.00	5.20	6.00
% A	65	65	40	40	65	65
% B	35	35	60	60	35	35

Total Run Time:	6.0 minutes
Flow rate:	1.0 mL/min
Approximate retention times:	sulphone: about 2.0 min<br>sulphoxide: about 1.8 min<br>SLIS: about 4.4 min
Column oven temp.	40 °C
Analytical column:	Chromolith RP-18 50 * 4.6 mm (Merck)
Autosampler type:	CTC PAL
Injection volume:	10 μL
Autosampler temperature:	10°C
MS instrument:	Perkin Elmer SCIEX API 4000
Ionisation:	TURBO ION SPRAY in positive ion mode
MRM transitions:	sulphone m/z 312.2 m/z 140.2<br>sulphoxide m/z 296.2 m/z 140.2<br>SLIS m/z 283.2 m/z 143.2
Resolution Q1	Unit
Q3	Unit
LLOQ	5.00 ng/mL
ULOQ	5000 ng/mL
Batch No. of standard	N/A
Software used:	
Acquisition and processing:	Analyst v. 1.4.1
LIMS:	Watson v. 6.4.0.04
Import data from Analyst file:	0327-2007-Run4.rdb
Data file in Analyst:	0327-2007\Run4.wiff

Table 2. Analytical Performance: Back-Calculated Concentrations (ng/mL) of Fexinidazole sulphone Calibration Standard in Rat Plasma for Study Protocol 0327-2007.

Assay Date	Analytical Run Number	STD.1 5.00 ng/mL	STD.2 10.0 ng/mL	STD.3 50.0 ng/mL	STD.4 100 ng/mL	STD.5 500 ng/mL	STD.6 1000 ng/mL	STD.7 4000 ng/mL	STD.8 5000 ng/mL
19-Dec-2007	4	5.17	9.63	48.4	105	443	855	4420	5680
		4.92	*91.5	50.4	101	517	965	*5170	*6280
Mean		5.05	9.63	49.4	103	480	910	4420	5680
SD		0.177		1.41	2.83	52.3	77.8		
%CV		3.5		2.9	2.7	10.9	8.5		
%Bias		1	-3.7	-1.2	3	-4	-9	10.5	13.6
n		2	1	2	2	2	2	1	1
* Reason Deactivated : Accuracy more than 15%.									

Table 3. Calibration Curve Parameters for Fexinidazole sulphone Calibration Standards in Rat Plasma for Study Protocol 0327-2007.

Run Date	Curve Number	Slope	Intercept	R^2	LLOQ ng/mL	ULOQ ng/mL	Regression Footnote(s)
19-Dec-2007	4	0.0024137	-0.0025907	0.9925	5	5000	1
Mean		0.0024137	-0.0025907	0.9925			
n		1	1	1			
Regression Footnote(s):<br>1) Resp. = Slope * Conc. + Intercept							

Table 4. Analytical Performance: Back-Calculated Concentrations (ng/mL) of Fexinidazole sulphoxide Calibration Standard in Rat Plasma for Study Protocol 0327-2007.

Assay Date	Analytical Run Number	STD.1 5.00 ng/mL	STD.2 10.0 ng/mL	STD.3 50.0 ng/mL	STD.4 100 ng/mL	STD.5 500 ng/mL	STD.6 1000 ng/mL	STD.7 4000 ng/mL	STD.8 5000 ng/mL
19-Dec-2007	4	5.02	8.62	45.1	105	448	885	4310	5610
		5.33	*88.3	52.1	104	531	997	*5110	*6180
Mean		5.18	8.62	48.6	105	490	941	4310	5610
SD		0.219		4.95	0.707	58.7	79.2		
%CV		4.2		10.2	0.7	12	8.4		
%Bias		3.6	-13.8	-2.8	5	-2	-5.9	7.8	12.2
n		2	1	2	2	2	2	1	1
* Reason Deactivated : Accuracy more than 15%.									

Table 5. Calibration Curve Parameters for Fexinidazole sulphoxide Calibration Standards in Rat Plasma for Study Protocol 0327-2007.

Run Date	Curve Number	Slope	Intercept	R^2	LLOQ ng/mL	ULOQ ng/mL	Regression Footnote(s)
19-Dec-2007	4	0.0015765	-0.00174	0.9909	5	5000	1
Mean		0.0015765	-0.00174	0.9909			
n		1	1	1			
Regression Footnote(s):<br>1) Resp. = Slope * Conc. + Intercept							

Table 6. Individual and mean (±SD, %CV) plasma concentrations (ng/mL) of sulphone and sulphoxide metabolites of Fexinidazole after single IV 5 mg/kg dose of Fexinidazole in male Sprague Dawley rats.

Sulphone						
Time (h)	ID R4	ID R5	ID R6	Mean	SD	%CV
0.033	16.2	12.8	16.4	15.1	2.02	13
0.25	325	251	272	283	38.1	14
0.5	630	453	574	552	90.5	16
1	1300	851	1040	1060	225	21
2	2120	1210	1600	1640	457	28
4	2490	1360	2180	2010	584	29
6	2370	1480	2290	2050	492	24
Sulphoxide						
Time (h)	ID R4	ID R5	ID R6	Mean	SD	%CV
0.033	444	498	431	458	35.5	8
0.25	2460	2460	2050	2320	237	10
0.5	2780	2620	2650	2680	85	3
1	3380	3060	2760	3070	310	10
2	2320	1980	2130	2140	170	8
4	924	783	1120	942	169	18
6	312	414	579	435	135	31

Table 7. Individual and mean (±SD, %CV) plasma concentrations (ng/mL) of sulphone and sulphoxide metabolites of Fexinidazole after single oral 10 mg/kg dose of Fexinidazole in male Sprague Dawley rats.

Sulphone						
Time (h)	ID R1	ID R2	ID R3	Mean	SD	%CV
0.25	38.1	54.9	58.8	50.6	11	22
0.5	156	162	149	156	6.51	4
1	537	334	496	456	107	24
2	1340	783	1260	1130	301	27
4	2750	1710	2420	2290	531	23
6	2890	2040	3050	2660	543	20
8	2740	1630	2600	2320	605	26
Sulphoxide						
Time (h)	ID R1	ID R2	ID R3	Mean	SD	%CV
0.25	615	843	944	801	169	21
0.5	1730	1490	1370	1530	183	12
1	3330	1780	2710	2610	780	30
2	4360	2650	3550	3520	855	24
4	2930	2440	2530	2630	261	10
6	1310	1260	1370	1310	55.1	4
8	477	241	331	350	119	34

Table 8. Individual and mean (±SD, %CV) pharmacokinetic parameters of sulphone and sulphoxide metabolites of Fexinidazole after single IV 5 mg/kg dose of Fexinidazole in male Sprague Dawley rats.

Sulphone						
Parameter (Units)	Rat ID			Mean	SD	%CV
	ID R4	ID R5	ID R6			
C_{max} (ng/mL)	2490	1480	2290	2087	535	26
t_{max} (h)	4	6	6	5.33	1.15	22
t_{last} (h)	6	6	6	6	0	0
$AUC_{0-t(last)}$ (ng·h/mL)	11800	6880	10100	9590	2500	26
C_{max}, norm (1)	449	270	423	381	96.7	25
$AUC_{0-t(last)}$, norm (1)	2130	1250	1870	1750	452	26
(2)	0.675	0.431	0.593	0.567	0.124	22
(3)	9.92	6.09	8.71	8.24	1.96	24
Sulphoxide						
Parameter (Units)	Rat ID			Mean	SD	%CV
	ID R4	ID R5	ID R6			
C_{max} (ng/mL)	3380	3060	2760	3067	310	10
t_{max} (h)	1	1	1	1	0	0
t_{last} (h)	6	6	6	6	0	0
$AUC_{0-t(last)}$ (ng·h/mL)	9850	8860	9610	9440	516	6
C_{max}, norm (1)	609	557	509	558	50	9
$AUC_{0-t(last)}$, norm (1)	1770	1610	1770	1720	92.4	5
(2)	0.916	0.892	0.715	0.841	0.11	13
(3)	8.28	7.84	8.28	8.13	0.254	3

(1) C_{max} (ng/mL) and $AUC_{0-t(last)}$ (ng·h/mL) values normalized to 1 mg/kg dose.

(2) C_{max}, metabolite / C_{max}, parent

(3) $AUC_{0-t(last)}$, metabolite / $AUC_{0-t(last)}$, parent

Table 9. Individual and mean (±SD, %CV) pharmacokinetic parameters of sulphone and sulphoxide metabolites of Fexinidazole after single oral 10 mg/kg dose of Fexinidazole in male Sprague Dawley rats.

Sulphone						
Parameter (Units)	Rat ID			Mean	SD	%CV
	ID R1	ID R2	ID R3			
C_{max} (ng/mL)	2890	2040	3050	2660	543	20
t_{max} (h)	6	6	6	6	0	0
t_{last} (h)	8	8	8	8	0	0
$AUC_{0-t(last)}$ (ng·h/mL)	16500	10600	15900	14300	3250	23
C_{max}, norm [1]	282	201	302	262	53.5	20
$AUC_{0-t(last)}$, norm [1]	1610	1050	1570	1410	312	22
[2]	15.9	18.7	20.2	18.3	2.19	12
[3]	23.2	20.2	24.1	22.5	2.04	9
Sulphoxide						
Parameter (Units)	Rat ID			Mean	SD	%CV
	ID R1	ID R2	ID R3			
C_{max} (ng/mL)	4360	2650	3550	3520	855	24
t_{max} (h)	2	2	2	2	0	0
t_{last} (h)	8	8	8	8	0	0
$AUC_{0-t(last)}$ (ng·h/mL)	18800	13700	16200	16200	2550	16
C_{max}, norm [1]	425	261	351	346	82.1	24
$AUC_{0-t(last)}$, norm [1]	1830	1350	1610	1600	240	15
[2]	24	24.3	23.5	23.9	0.402	2
[3]	26.5	26.1	24.6	25.7	1.01	4

[1] C_{max} (ng/mL) and $AUC_{0-t(last)}$ (ng·h/mL) values normalized to 1 mg/kg dose.

[2] C_{max}, metabolite / C_{max}, parent

[3] $AUC_{0-t(last)}$, metabolite / $AUC_{0-t(last)}$, parent

Figure 1. Individual plasma concentrations (ng/mL) of sulphone (upper panel) and sulphoxide (lower panel) metabolites after single IV 5 mg/kg dose of Fexinidazole in male Sprague Dawley rats.

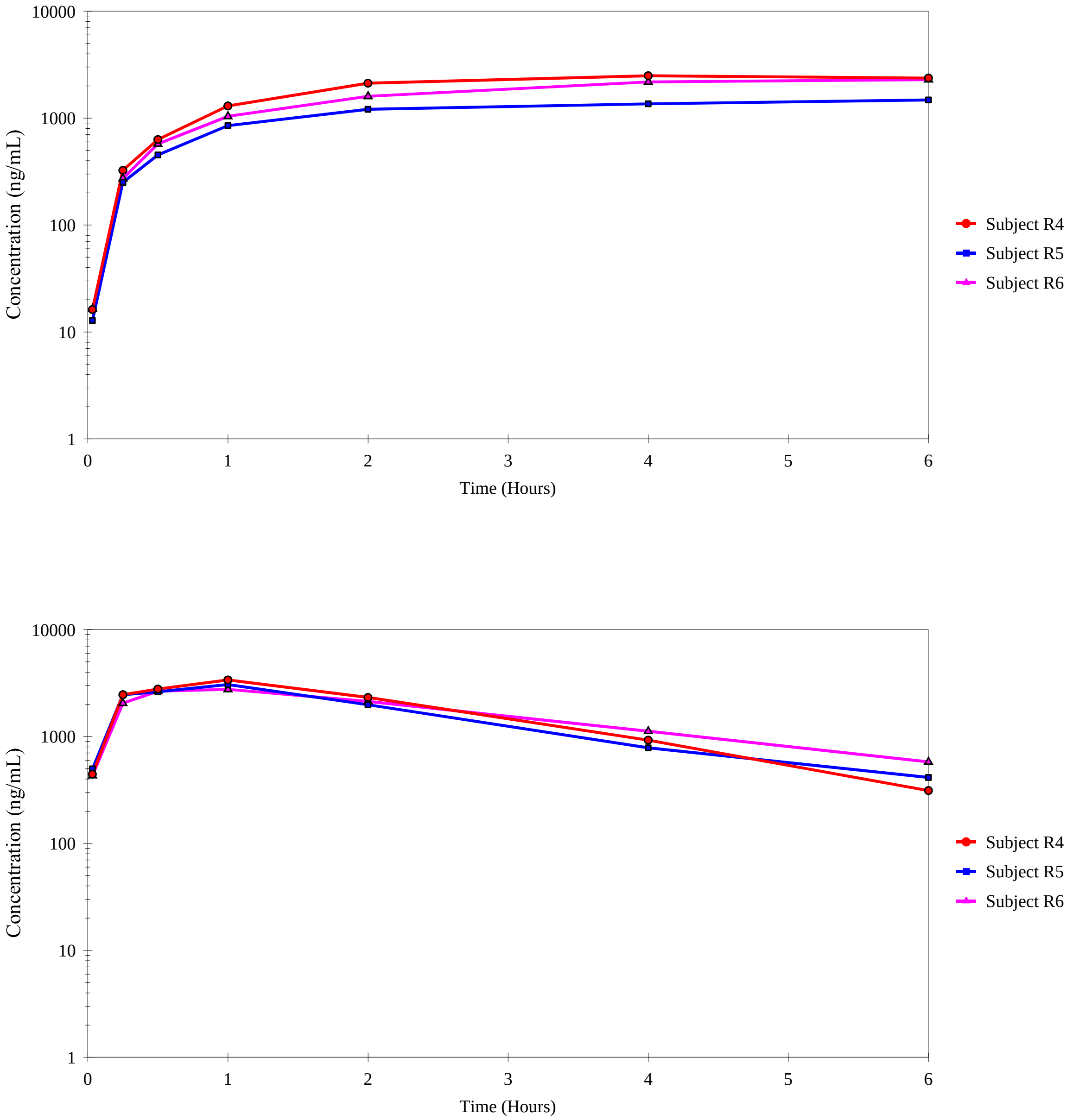

Figure 2. Individual plasma concentrations (ng/mL) of sulphone (upper panel) and sulphoxide (lower panel) metabolites after single oral 10 mg/kg dose of Fexinidazole in male Sprague Dawley rats.

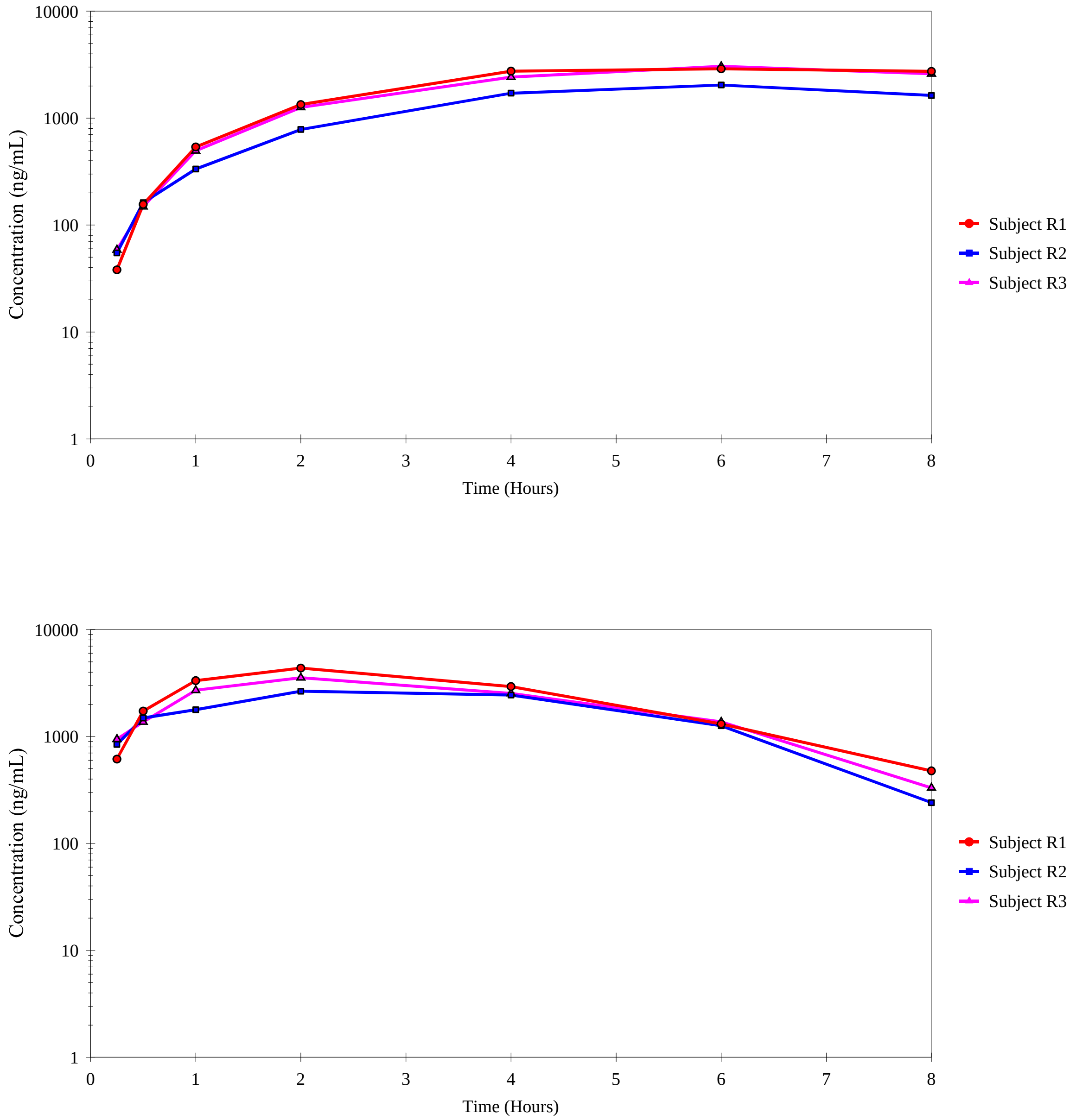

Figure 3. Mean (±SD) plasma concentrations (ng/mL) of Fexinidazole and its sulphone and sulphoxide metabolites after single IV 5 mg/kg of Fexinidazole in male Sprague Dawley rats.

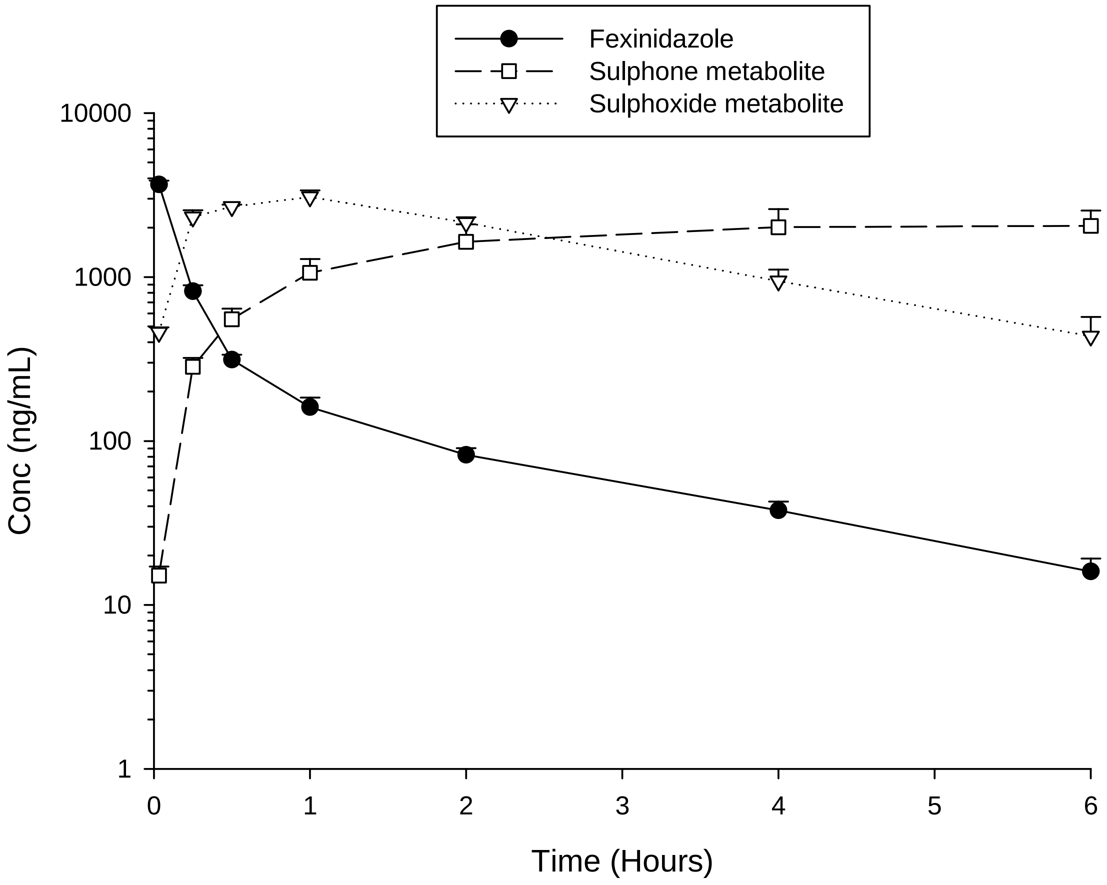

Figure 4. Mean (±SD) plasma concentrations (ng/mL) of Fexinidazole and its sulphone and sulphoxide metabolites after single oral 10 mg/kg dose of Fexinidazole in male Sprague Dawley rats.

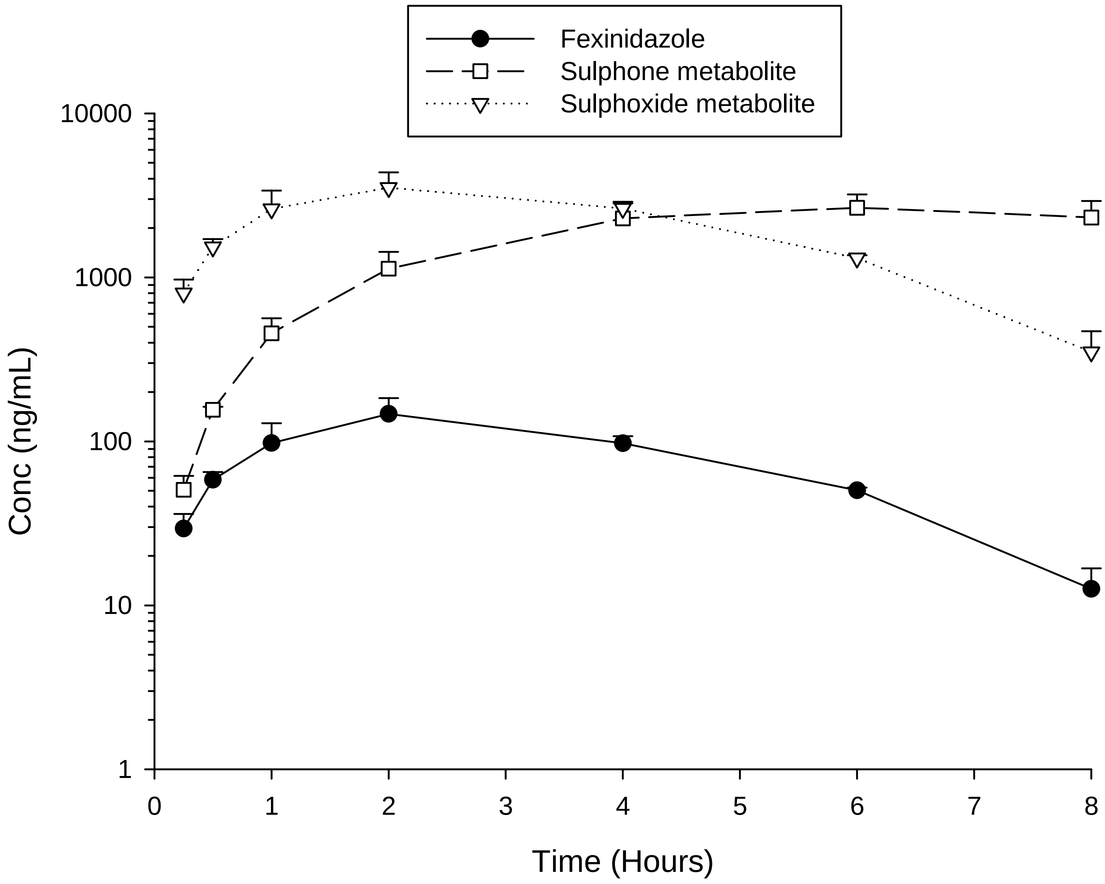

Supplement: Dataset S6 — (0.38 MB PDF) [file pntd.0000923.s007.pdf]
