# Supplementary material for: Fexinidazole – A New Oral Nitroimidazole Drug Candidate Entering Clinical Development for the Treatment of Sleeping Sickness
Source: PLoS Negl Trop Dis. 2010 Dec 21;4(12):e923. doi: 10.1371/journal.pntd.0000923 (PMC3006138; doi:10.1371/journal.pntd.0000923)
Supplement: Dataset S7 — (0.36 MB PDF) [file pntd.0000923.s008.pdf]

# Fexinidazole: Evaluation of Pharmacokinetics Following Single IV and Oral Administration to Beagle Dog

| | |
|---|---|
| Product Name: | Fexinidazole |
| Study Number: | 0320-2007 |
| Study Director | |
| Status: | Final |

# TABLE OF CONTENTS

1. STUDY CONDUCT....4
2. OBJECTIVE....4
3. ABBREVIATIONS....4
4. METHODS....5
4.1. Study Design....5
4.2. Sample Collection and Handling....5
4.3. Bioanalytical Method....5
4.4. Pharmacokinetic Calculations....5
5. RESULTS....6
5.1.1. Clinical and Physical Examinations....6
5.1.1.1. Clinical Signs....6
5.1.1.2. Body Weight....7
5.1.2. Pharmacokinetic analysis....7
5.1.2.1. IV dosing....7
5.1.2.2. Oral dosing....8
6. CONCLUSIONS....8
7. CONTRIBUTORS....8
8. ARCHIVING....9
9. REFERENCES....9
TABLES AND FIGURES....10
APPENDICES
Appendix 1. Individual plasma concentrations....20
Appendix 2. Bioanalytical method....24
Appendix 3. Analytical performance....25
Appendix 4. Clinical signs....28
Appendix 5. Body Weight....28

# 1. STUDY CONDUCT

The study, sponsored by Drugs for Neglected Diseases *initiative* (DND*i*), was performed within Accelera, Nerviano Medical Sciences, Italy according to the internal Standard Operating Procedures as a non-GLP regulated study.

# 2. OBJECTIVE

The objective of this study was to evaluate the pharmacokinetics of Fexinidazole and its sulphone and sulphoxide metabolites and the absolute bioavailability of Fexinidazole after single IV 1 mg/kg and oral 10 mg/kg doses of the compound to male Beagle dogs.

# 3. ABBREVIATIONS

The following abbreviations are used in this document:

| | |
|---|---|
| $AUC_{0-t(last)}$ | Area under the plasma concentration *vs*. time curve up to finite time |
| $AUC_{0-\infty}$ | Area under the plasma concentration *vs*. time curve up to infinite time |
| $C_{0.083}$ | Concentration at 5 minutes after IV dosing |
| $C_{max}$ | Maximal plasma concentration |
| $\%EXT_{t(last)-\infty}$ | Fraction of $AUC_{0-\infty}$ accounted for by extrapolation |
| CV | Coefficient of variation of the mean |
| F | Absolute bioavailability |
| h | Hours |
| ID | Animal identification code |
| IV | Intravenous |
| LC | Liquid chromatography |
| LLOQ | Lower limit of quantification |
| MS | Mass-spectrometry |
| Norm | Normalized value |
| $R^2$ | Correlation coefficient |
| SD | Standard deviation of the mean |
| SOP | Standard operating procedure |
| STD | Standard sample |
| $t_{1/2,z}$ | Terminal half-life |
| $t_{max}$ | Time to peak plasma concentration |
| $t_{last}$ | Time of the last detectable concentration |
| ULOQ | Upper limit of quantification |

# 4. METHODS

## 4.1. Study Design

The study was conducted according to the study protocol [1].
Fexinidazole was given as IV and oral administrations to male Beagle dogs according to the following scheme

| Dog ID | Route | Dose (mg/kg) | Formulation |
|---|---|---|---|
| 1979, 2197, 2205 | IV | 1 | 70 % PEG 400 in 5 % dextrose solution |
| | Oral | 10 | 5 % Tween 80 and 0.5% Methocel suspension |

IV dose was administered *via* the radial vein, the oral dose was administered by gastric gavage. A washout period of 12 days was allowed between the IV (Day 1) and oral (Day 12) doses.

## 4.2. Sample Collection and Handling

Blood was withdrawn from jugular vein and collected in heparinized plastic tubes kept on an ice/water bath, then centrifuged at 1200g for 10 minutes at 4°C. Three aliquots of the separated plasma (about 0.2 mL) was stored at -80°C until analysis for Fexinidazole and its sulphone and sulphoxide metabolites. Blood was taken at pre-dose and 0.083 (5 minutes), 0.25, 0.5, 1, 2, 4, 6 and 10 h after IV dosing; at pre-dose and 0.25, 0.5, 1, 2, 4, 6, 8 and 24 h after oral dosing.

## 4.3. Bioanalytical Method

Plasma concentrations of Fexinidazole and its sulphone and sulphoxide metabolites were determined by LC-MS-MS method following plasma protein precipitation. The lower limit of quantification of the bioanalytical method was 5 ng/mL for all analytes. Bioanalytical data were stored in Watson LIMS (v. 6.4.0.04, Thermo Fisher Scientific, Waltham, MA, USA). Details of the bioanalytical method used are reported in Appendix 2 and analytical performances of calibration standards are reported in Appendix 3.

## 4.4. Pharmacokinetic Calculations

Pharmacokinetic evaluations of Fexinidazole and sulphone and sulphoxide metabolites were carried out using non-compartmental approach with the aid of the Watson package (v. 6.4.0.04, Thermo Fisher Scientific, Waltham, MA, USA).
For the calculations, the pre-dose concentrations of Fexinidazole were set equal to $C_{0.083}$ after IV administration, whilst the pre-dose concentrations of Fexinidazole after oral administration and the pre-dose concentrations of the metabolites after both doses were set equal to zero.

After IV and oral dosing, $C_{max}$ ($C_{0.083}$ for Fexinidazole after IV dosing) and $t_{max}$ of each compound were read from raw data as the coordinates of the highest measured concentration. After both doses, for each compound, the area under plasma concentration *vs.* time curve to finite time, $AUC_{0-t(last)}$, was determined by the linear trapezoidal rule up to the last detectable concentration. The half-life of the terminal phase, $t_{1/2,z}$, was determined by linear regression analysis of the natural-log concentration *vs.* time curve, where $t_{1/2,z}$ = ln(2)/slope of the regression line. The area under the concentration vs. time curve up to infinite time, $AUC_{0-\infty}$, was determined as

$$AUC0-\infty = AUC0-t(last) + \frac{Ct(last)\cdot t_{1/2,z}}{\ln(2)}$$

The fraction of $AUC_{0-\infty}$ accounted for by the extrapolated area under the curve was calculated as follows:

$$\%EXTt(last)-\infty = 100\cdot\frac{AUCt(last)-\infty}{AUC0-\infty}$$

After IV dosing, plasma clearance and volume of distribution at steady state of Fexinidazole were calculated as follows:
CL = Dose/$AUC_{0-\infty}$
$V_{ss}$ = CL·MRT, where MRT is the mean residence time.

Absolute bioavailability of Fexinidazole was calculated from the ratio of individual oral to IV dose-normalized $AUC_{0-t(last)}$ and $AUC_{0-\infty}$ values.

Metabolite to parent ratio was calculated based on $C_{max}$ and $AUC_{0-t(last)}$ values.

After both doses, $C_{max}$ and AUC values of each compound were also normalized to a 1 mg/kg dose level.

Descriptive statistics (mean ± SD, %CV) were reported for plasma concentrations and pharmacokinetic parameters sorted by compound and route of dosing.

Plasma concentrations and pharmacokinetic parameters of each compound were reported to three significant figures.

# 5. RESULTS

## 5.1.1. Clinical and Physical Examinations

### 5.1.1.1. Clinical Signs

Individual clinical signs are reported in Appendix 4.

No significant treatment related clinical signs were observed after intravenous and oral treatments with Fexinidazole. No meaningful clinical signs were recorded during wash out period.

#### 5.1.1.2. Body Weight

Body weights are included in Appendix 5.

No treatment-related changes were observed during the study. Normal fluctuations of the body weight were recorded.

### 5.1.2. Pharmacokinetic analysis

Mean ±SD systemic exposure parameters of Fexinidazole and its metabolites after IV and oral administrations of Fexinidazole are reported in Table 1. Individual and mean pharmacokinetic parameters of Fexinidazole and metabolites after IV and oral administrations are reported in Tables 2 - 5. Individual and mean plasma concentrations of the compounds are plotted in Figures 1 - 6 and reported in Tables 1A1 - 4A1 of Appendix 1.

#### 5.1.2.1. IV dosing

After dosing, mean ±SD $C_{0.083}$ value of Fexinidazole was 320 ±136 ng/mL. The plasma concentrations of the compound declined poly-exponentially with mean terminal half-life of about half an hour (Figure 5). Mean ±SD systemic clearance, 273 ±121 mL/min/kg, was higher than dog hepatic blood flow (41 mL/min/kg, [2]), indicating high rate of elimination of the compound from the systemic circulation. Mean ±SD volume of distribution at steady state, 3260 ±131 mL/kg, was five-six times higher than dog total body water (about 600 mL/kg [3]), indicating extensive tissue distribution.

Sulphone detectable pre-dose concentration was measured in the dog ID 2197. This value, 9.25 ng/mL, close to the LLOQ of the analytical method, was likely due to a contamination and it was excluded from the calculations.
After dosing, detectable concentrations of the sulphone metabolite were measured at the first sampling time (5 minutes post dosing). The concentrations of the compound achieved the maximal concentration, on average 681 ng/mL, 4 - 6 h post dosing, and showed a plateau profile up to the last sampling time (10 h post dosing, Figure 5). This behaviour prevented an unbiased estimate of the pharmacokinetics of the compound. Mean $AUC_{0-t(last)}$ was 5270 ng·h/mL. The metabolite to parent $AUC_{0-t(last)}$ ratio, on average 85, indicated that Fexinidazole was extensively metabolised to its sulphone metabolite.

The plasma profile of the sulphoxide metabolite was different from that of the sulphone metabolite (Figure 5). Sulphoxide maximal concentration, on average 763 ng/mL, was rapidly achieved, within 15 minutes post dosing; then the concentrations of the metabolite declined mono-exponentially with mean half-life of about 1 h. Mean $AUC_{0-t(last)}$ value was 1480 ng·h/mL. The metabolite to parent $AUC_{0-t(last)}$ ratio was, on average, 25, indicating that Fexinidazole was extensively metabolised to its sulphoxide metabolite.

#### 5.1.2.2. Oral dosing

After dosing, mean ±SD maximal plasma concentration of Fexinidazole, 24.2 ±5.9 ng/mL, was achieved within 1 h post dosing. The concentrations of the compound were detectable up to 4 h post dosing. Mean ±SD $AUC_{0\text{-}t(last)}$ was 57.2 ±13.4 ng·h/mL. In the dog ID 2205, the plasma profile did not allow an unbiased estimate of the half-life. Mean (n=2) apparent terminal half-life of the compound was 2 h, longer than that after IV dosing, suggesting that the absorption processes affected the disposition of the compound. Mean ±SD (n=2) $AUC_{0\text{-}\infty}$ was 85.3 ±1.27 ng·h/mL. Absolute bioavailability, based on $AUC_{0\text{-}t(last)}$ and $AUC_{0\text{-}\infty}$ values, was 10 and 14 %, respectively.

After dosing, the maximal concentration of the sulphone metabolite, on average 3260 ng/mL, was achieved 6 - 8 h post dosing. After the achievement of the maximal concentration, the concentrations of the metabolite slowly declined up to the end of the experiment (24 h post dosing). $AUC_{0\text{-}t(last)}$ value was, on average, 44700 ng·h/mL. The metabolite to parent $AUC_{0\text{-}t(last)}$ ratio was very high, on average 810.

The plasma profile of the sulphoxide metabolite was different from that of the sulphone metabolite (Figure 6). Sulphoxide maximal concentration, on average 1903 ng/mL, was achieved 1 h post dosing, then the concentrations of the metabolite declined poly-exponentially with mean terminal half-life of 6 h, longer than that after IV dosing. Mean $AUC_{0\text{-}t(last)}$ value was 8560 ng·h/mL. Metabolite to parent $AUC_{0\text{-}t(last)}$ ratio was, on average, 158.

## 6. CONCLUSIONS

After both doses, the coefficient of variation of the mean parameters of Fexinidazole and its metabolites was lower than 50 %.
Fexinidazole was endowed with high volume of distribution and plasma clearance. After oral dosing, the half-life of Fexinidazole was longer than that after IV dosing, suggesting that the absorption processes affected the disposition of Fexinidazole. The oral bioavailability of Fexinidazole was 10 %. After both doses, Fexinidazole was extensively metabolized to the sulphone and sulphoxide derivatives. For both metabolites, the metabolite to parent ratio was higher after oral than after IV dosing, suggesting an extensive first pass effect metabolism.

## 7. CONTRIBUTORS

## 8. ARCHIVING

The protocol, raw data, pharmacokinetic analysis and final report were archived within Accelera Archive, Nerviano Medical Sciences, Italy, according the Unit Standard Operating Procedures.

## 9. REFERENCES

1. Fexinidazole: Evaluation of Pharmacokinetics Following Single IV and Oral Administration to Beagle dogs. Nerviano Medical Sciences Study Protocol no. 0320-2007-P, October 22, 2007.

2. Boxenbaum, H. Interspecies variation in liver weight, hepatic blood flow, and antipyrine intrinsic clearance: Extrapolation of data to benzodiazepines and phenytoin. J. Pharmacokin. Biopharm. 8: 165-176, 1980.

3. Spector, W. S. Handbook of Biological Data. Philadelphia, W.B. Saunders Co., 1956.

# TABLES AND FIGURES

**Table 1**. Summary table of mean ±SD systemic exposure values of Fexinidazole and its metabolites after single IV 1 mg/kg and oral 10 mg/kg doses of Fexinidazole in male Beagle dogs.

| Dose (mg/kg) | Compound | $C_{max}$ (ng/mL) | $t_{last}$ (h) | $AUC_{0-t(last)}$ (ng·h/mL) | $AUC_{0-\infty}$ (ng·h/mL) | F% $AUC_{0-t(last)}$ |
|---|---|---|---|---|---|---|
| 1 | Fexinidazole | 320 ±136 | 1 | 66.9 ±31.8 | 70.6 ±33.8 | |
| | Sulfone | 681 ±162 | 10 | 5270 ±1230 | NA | |
| | Sulfoxide | 763 ±45.3 | 6 | 1480 ±197 | 1520 ±208 | |
| 10 | Fexinidazole | 24.2 ±5.86 | 4 | 57.2 ±13.4 | 85.3 ±1.27 | 9.60 ±4.32 |
| | Sulfone | 3260 ±762 | 24 | 44700 ±3810 | NA | |
| | Sulfoxide | 1903 ±186 | 24 | 8560 ±767 | 8780 ±895 | |

NA: not assessable

**Table 2**. Individual and mean (±SD, %CV) pharmacokinetic parameters of Fexinidazole after single IV 1 mg/kg dose of the compound in male Beagle dogs.

| Parameter (Units) | Dog ID | | | Mean | SD | %CV |
|---|---|---|---|---|---|---|
| | ID 1979 | ID 2197 | ID 2205 | | | |
| $C_{0.083}$ (ng/mL) | 468 | 200 | 293 | 320 | 136 | 43 |
| $t_{last}$ (h) | 2 | 0.5 | 1 | 1.17 | 0.764 | 65 |
| $AUC_{0-t(last)}$ (ng·h/mL) | 102 | 40.1 | 58.6 | 66.9 | 31.8 | 48 |
| Regression Range (h) | 0.25 - 2 | 0.083 - 0.5 | 0.25 - 1 | N/A | N/A | N/A |
| $t_{1/2,z}$ (h) | 0.704 | 0.111 | 0.403 | 0.406 | 0.297 | 73 |
| $AUC_{0-\infty}$ (ng·h/mL) | 108 | 42.2 | 61.7 | 70.6 | 33.8 | 48 |
| $\%EXT_{t(last)-\infty}$ | 5.15 | 4.87 | 5.08 | 5.03 | 0.146 | 3 |
| CL (mL/min/kg) | 154 | 395 | 270 | 273 | 121 | 44 |
| $V_{ss}$ (mL/kg) | 3380 | 3120 | 3280 | 3260 | 131 | 4 |
| $C_{0.083}$, norm (1) | 468 | 200 | 293 | 320 | 136 | 43 |
| $AUC_{0-t(last)}$, norm (1) | 102 | 40.1 | 58.6 | 66.9 | 31.8 | 48 |
| $AUC_{0-\infty}$, norm (1) | 108 | 42.2 | 61.7 | 70.6 | 33.8 | 48 |

N/A: not applicable

(1) $C_{0.083}$ (ng/mL) and AUC (ng·h/mL) values normalized to 1 mg/kg dose.

**Table 3.** Individual and mean (±SD, %CV) pharmacokinetic parameters of sulphone and sulphoxide metabolites of Fexinidazole after single IV 1 mg/kg dose of Fexinidazole in male Beagle dogs.

| Sulphone | | | | | | |
|---|---|---|---|---|---|---|
| Parameter (Units) | Dog ID | | | Mean | SD | %CV |
| | ID 1979 | ID 2197 | ID 2205 | | | |
| $C_{max}$ (ng/mL) | 763 | 494 | 785 | 681 | 162 | 24 |
| $t_{max}$ (h) | 6 | 4 | 4 | 4.67 | 1.15 | 25 |
| $t_{last}$ (h) | 10 | 10 | 10 | 10 | 0 | 0 |
| $AUC_{0-t(last)}$ (ng·h/mL) | 6040 | 3850 | 5920 | 5270 | 1230 | 23 |
| $C_{max}$, norm (1) | 763 | 494 | 785 | 681 | 162 | 24 |
| $AUC_{0-t(last)}$, norm (1) | 6040 | 3850 | 5920 | 5270 | 1230 | 23 |
| (2) | 1.63 | 2.47 | 2.68 | 2.26 | 0.555 | 25 |
| (3) | 59.2 | 96.0 | 101 | 85.4 | 22.8 | 27 |
| Sulphoxide | | | | | | |
| Parameter (Units) | Dog ID | | | Mean | SD | %CV |
| | ID 1979 | ID 2197 | ID 2205 | | | |
| $C_{max}$ (ng/mL) | 721 | 811 | 757 | 763 | 45.3 | 6 |
| $t_{max}$ (h) | 0.25 | 0.083 | 0.25 | 0.194 | 0.0964 | 50 |
| $t_{last}$ (h) | 6 | 6 | 6 | 6 | 0 | 0 |
| $AUC_{0-t(last)}$ (ng·h/mL) | 1710 | 1380 | 1360 | 1480 | 197 | 13 |
| Regression Range (h) | 0.25 - 6 | 0.083 - 6 | 0.25 - 6 | N/A | N/A | N/A |
| $t_{1/2,z}$ (h) | 1.28 | 1.12 | 1.08 | 1.16 | 0.106 | 9 |
| $AUC_{0-\infty}$ (ng·h/mL) | 1760 | 1410 | 1390 | 1520 | 208 | 14 |
| $C_{max}$, norm (1) | 721 | 811 | 757 | 763 | 45.3 | 6 |
| $AUC_{0-t(last)}$, norm (1) | 1710 | 1380 | 1360 | 1480 | 197 | 13 |
| $AUC_{0-\infty}$, norm (1) | 1760 | 1410 | 1390 | 1520 | 208 | 14 |
| (2) | 1.54 | 4.06 | 2.58 | 2.73 | 1.26 | 46 |
| (3) | 16.8 | 34.4 | 23.2 | 24.8 | 8.93 | 36 |

N/A: not applicable

(1) $C_{max}$ (ng/mL) and AUC (ng·h/mL) values normalized to 1 mg/kg dose.

(2) $C_{max}$, metabolite / $C_{max}$, parent

(3) $AUC_{0-t(last)}$, metabolite / $AUC_{0-t(last)}$, parent

**Table 4.** Individual and mean (±SD, %CV) pharmacokinetic parameters of Fexinidazole after single oral 10 mg/kg dose of the compound in male Beagle dogs.

| Parameter (Units) | Dog ID | | | Mean | SD | %CV |
|---|---|---|---|---|---|---|
| | ID 1979 | ID 2197 | ID 2205 | | | |
| $C_{max}$ (ng/mL) | 28.8 | 26.2 | 17.6 | 24.2 | 5.86 | 24 |
| $t_{max}$ (h) | 0.5 | 0.5 | 1 | 0.667 | 0.289 | 43 |
| $t_{last}$ (h) | 4 | 4 | 4 | 4 | 0 | 0 |
| $AUC_{0\text{-}t(last)}$ (ng·h/mL) | 69.9 | 58.5 | 43.2 | 57.2 | 13.4 | 23 |
| Regression Range (h) | 0.5 - 4 | 0.5 - 4 | (1) | N/A | N/A | N/A |
| $t_{1/2,z}$ (h) | 1.62 | 2.24 | | 1.93 | 0.438 | 23 |
| $AUC_{0\text{-}\infty}$ (ng·h/mL) | 84.4 | 86.2 | | 85.3 | 1.27 | 2 |
| $\%EXT_{t(last)\text{-}\infty}$ | 17.2 | 32.2 | | 24.7 | 10.6 | 43 |
| $C_{max}$, norm (2) | 2.88 | 2.62 | 1.76 | 2.42 | 0.586 | 24 |
| $AUC_{0\text{-}t(last)}$, norm (2) | 6.99 | 5.85 | 4.32 | 5.72 | 1.34 | 23 |
| $AUC_{0\text{-}\infty}$, norm (2) | 8.44 | 8.62 | | 8.53 | 0.127 | 2 |
| F% $AUC_{0\text{-}t(last)}$ | 6.85 | 14.6 | 7.37 | 9.60 | 4.32 | 45 |
| F% $AUC_{0\text{-}\infty}$ | 7.81 | 20.4 | | 14.1 | 8.92 | 63 |

N/A: not applicable

(1) The plasma profile did not allow an unbiased estimate of the half-life

(2) $C_{max}$ (ng/mL) and AUC (ng·h/mL) values normalized to 1 mg/kg dose.

**Table 5.** Individual and mean (±SD, %CV) pharmacokinetic parameters of sulphone and sulphoxide metabolites of Fexinidazole after single oral 10 mg/kg dose of Fexinidazole in male Beagle dogs.

| Sulphone | | | | | | |
|---|---|---|---|---|---|---|
| Parameter (Units) | Dog ID | | | Mean | SD | %CV |
| | ID 1979 | ID 2197 | ID 2205 | | | |
| $C_{max}$ (ng/mL) | 2820 | 4140 | 2820 | 3260 | 762 | 23 |
| $t_{max}$ (h) | 6 | 6 | 8 | 6.67 | 1.15 | 17 |
| $t_{last}$ (h) | 24 | 24 | 24 | 24 | 0 | 0 |
| $AUC_{0-t(last)}$ (ng·h/mL) | 42600 | 49100 | 42400 | 44700 | 3810 | 9 |
| $C_{max}$, norm (1) | 282 | 414 | 282 | 326 | 76.2 | 23 |
| $AUC_{0-t(last)}$, norm (1) | 4260 | 4910 | 4240 | 4470 | 381 | 9 |
| (2) | 97.9 | 158 | 160 | 139 | 35.4 | 25 |
| (3) | 609 | 839 | 981 | 810 | 188 | 23 |
| Sulphoxide | | | | | | |
| Parameter (Units) | Dog ID | | | Mean | SD | %CV |
| | ID 1979 | ID 2197 | ID 2205 | | | |
| $C_{max}$ (ng/mL) | 2030 | 1990 | 1690 | 1903 | 186 | 10 |
| $t_{max}$ (h) | 1 | 0.5 | 2 | 1.17 | 0.764 | 66 |
| $t_{last}$ (h) | 24 | 24 | 24 | 24 | 0 | 0 |
| $AUC_{0-t(last)}$ (ng·h/mL) | 7760 | 8620 | 9290 | 8560 | 767 | 9 |
| Regression Range (h) | 6 - 24 | 6 - 24 | 6 - 24 | N/A | N/A | N/A |
| $t_{1/2,z}$ (h) | 4.83 | 6.3 | 6.03 | 5.72 | 0.782 | 14 |
| $AUC_{0-\infty}$ (ng·h/mL) | 7830 | 8890 | 9610 | 8780 | 895 | 10 |
| $C_{max}$, norm (1) | 203 | 199 | 169 | 190 | 18.6 | 10 |
| $AUC_{0-t(last)}$, norm (1) | 776 | 862 | 929 | 856 | 76.7 | 9 |
| $AUC_{0-\infty}$, norm (1) | 783 | 889 | 961 | 878 | 89.5 | 10 |
| (2) | 70.5 | 76.0 | 96.0 | 80.8 | 13.4 | 17 |
| (3) | 111 | 147 | 215 | 158 | 52.8 | 33 |

N/A: not applicable

(1) $C_{max}$ (ng/mL) and AUC (ng·h/mL) values normalized to 1 mg/kg dose.

(2) $C_{max}$, metabolite / $C_{max}$, parent

(3) $AUC_{0-t(last)}$, metabolite / $AUC_{0-t(last)}$, parent

**Figure 1.** Individual plasma concentrations (ng/mL) of Fexinidazole after single IV 1 mg/kg dose of the compound in male Beagle dogs.

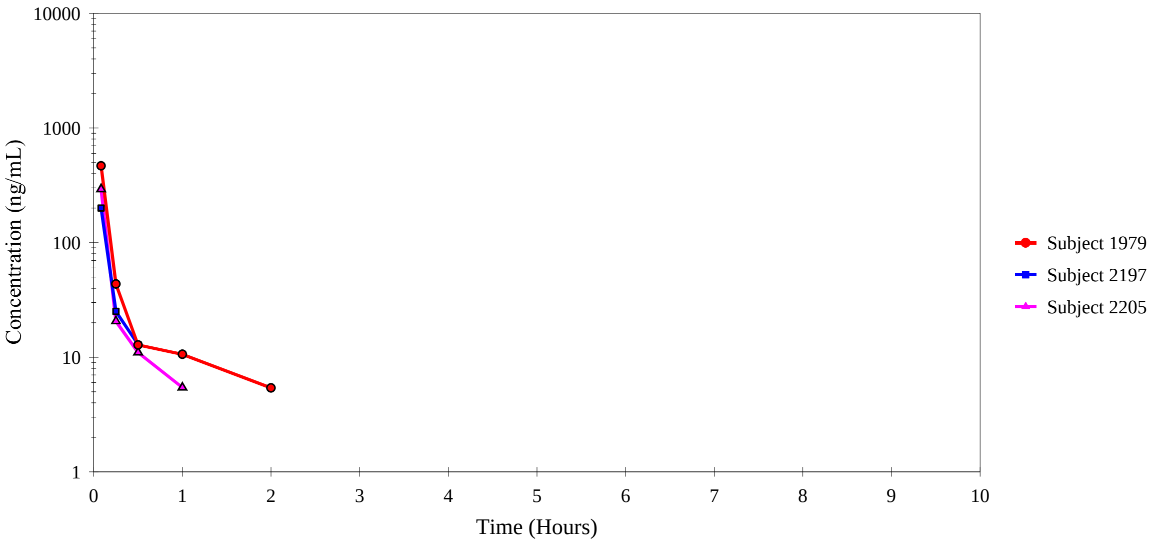

**Figure 2.** Individual plasma concentrations (ng/mL) of sulphone (upper panel) and sulphoxide (lower panel) metabolites of Fexinidazole after single IV 1 mg/kg dose of Fexinidazole in male Beagle dogs.

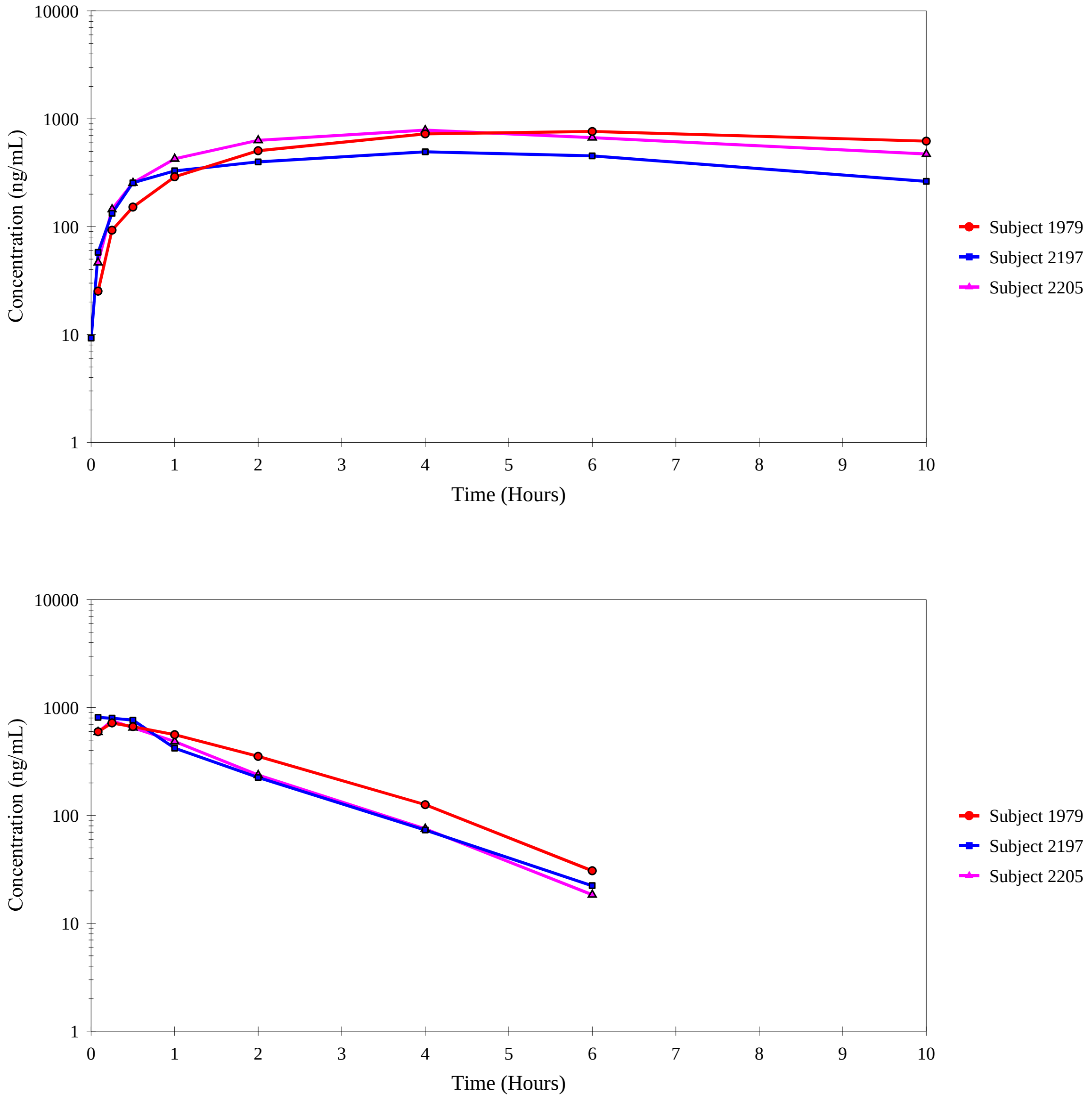

**Figure 3.** Individual plasma concentrations (ng/mL) of Fexinidazole after single oral 10 mg/kg dose of the compound in male Beagle dogs.

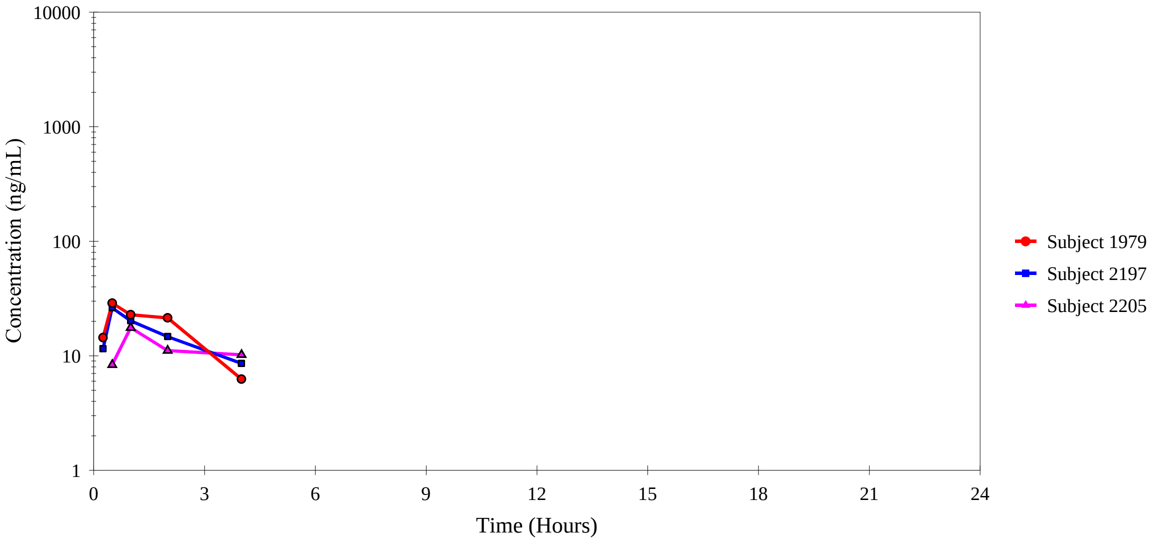

**Figure 4.** Individual plasma concentrations (ng/mL) of sulphone (upper panel) and sulphoxide (lower panel) metabolites of Fexinidazole after single oral 10 mg/kg dose of Fexinidazole in male Beagle dogs.

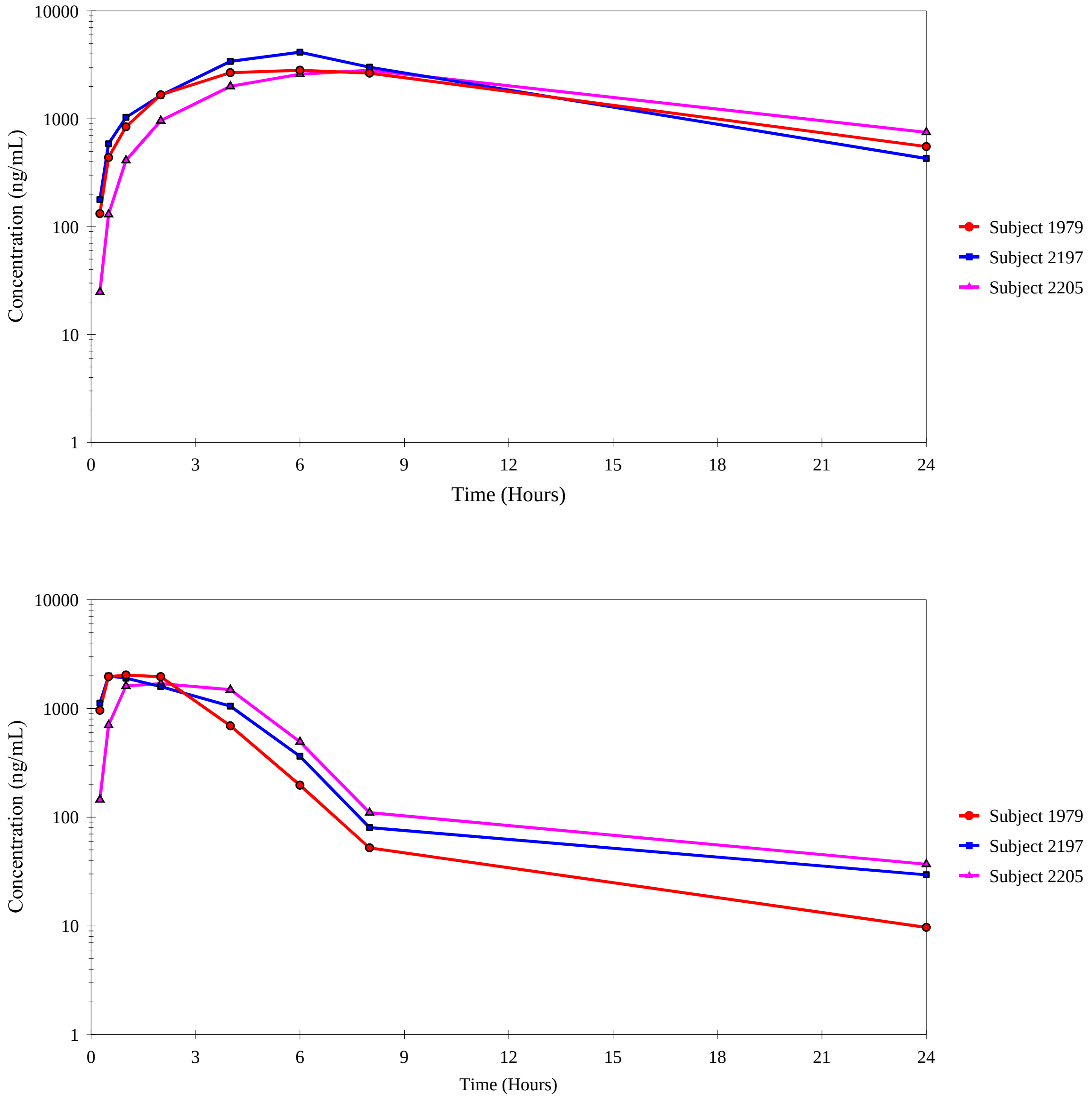

**Figure 5.** Mean (+SD) plasma concentrations (ng/mL) of Fexinidazole and its sulphone and sulphoxide metabolites after single IV 1 mg/kg dose of Fexinidazole in male Beagle dogs.

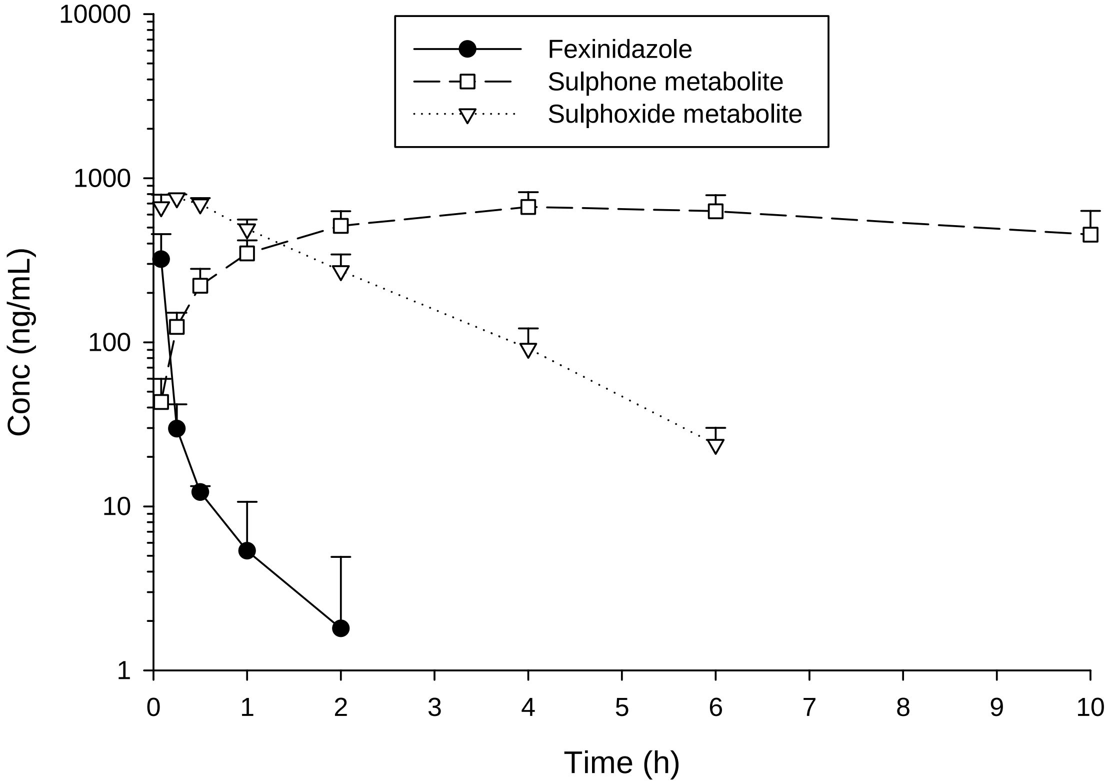

**Figure 6.** Mean (+SD) plasma concentrations (ng/mL) of Fexinidazole and its sulphone and sulphoxide metabolites after single oral 10 mg/kg dose of Fexinidazole in male Beagle dogs.

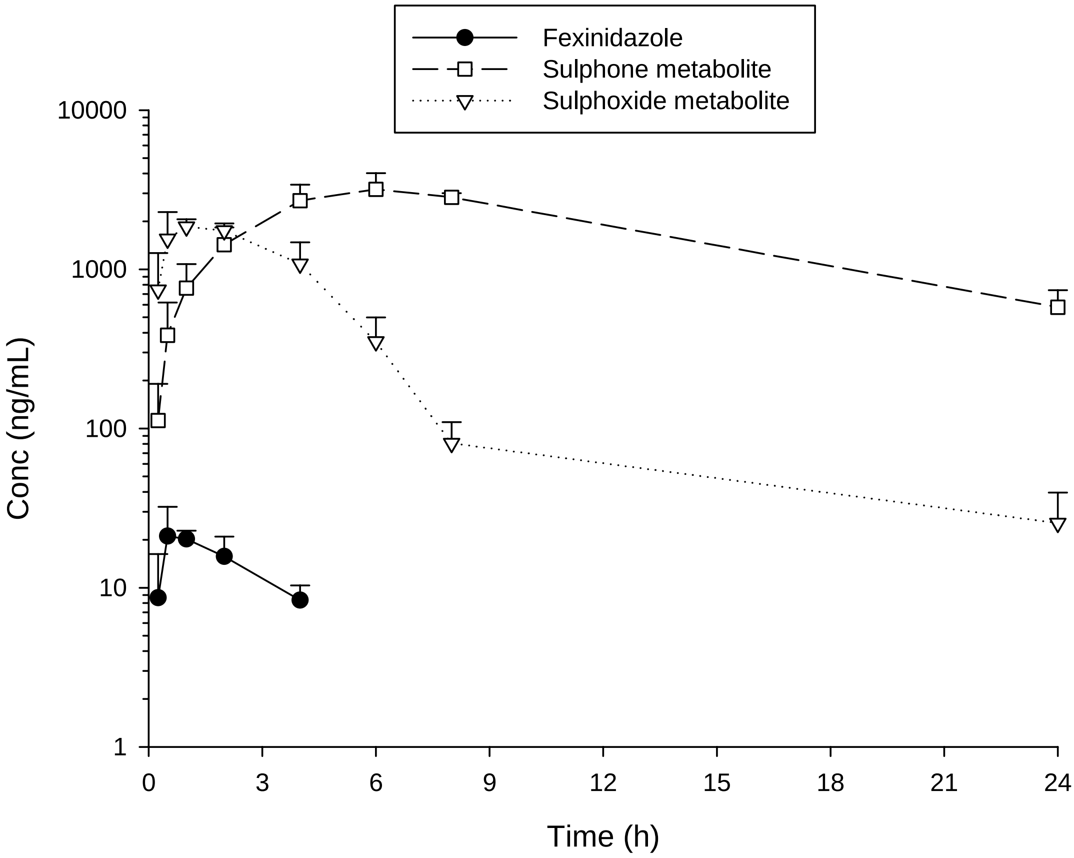

# APPENDICES

## Appendix 1. Individual plasma concentrations

**Table 1A1.** Individual and mean (±SD, %CV) plasma concentrations (ng/mL) of Fexinidazole after single IV 1 mg/kg dose of the compound in male Beagle dogs.

| Time (h) | Dog ID | | | Mean | SD | %CV |
|---|---|---|---|---|---|---|
| | 1979 | 2197 | 2205 | | | |
| Pre-dose | <5.00 | <5.00 | <5.00 | N/A | N/A | N/A |
| 0.083 | 468 | 200 | 293 | 320 | 136 | 43 |
| 0.25 | 43.5 | 25.1 | 20.6 | 29.7 | 12.1 | 41 |
| 0.5 | 12.8 | 12.9 | 11 | 12.2 | 1.07 | 9 |
| 1 | 10.6 | <5.00 | 5.45 | 5.35 | 5.3 | 99 |
| 2 | 5.4 | <5.00 | <5.00 | 1.8* | 3.12 | 173 |
| 4 | <5.00 | <5.00 | <5.00 | N/A | N/A | N/A |
| 6 | <5.00 | <5.00 | <5.00 | N/A | N/A | N/A |
| 10 | <5.00 | <5.00 | <5.00 | N/A | N/A | N/A |

Estimates of mean based on approximation that values below LLOQ are equal to zero. For values marked *, more than half of the individual levels were below LLOQ; descriptive statistics was reported even if strongly biased.

N/A: not applicable.

**Table 2A1.** Individual and mean (±SD, %CV) plasma concentrations (ng/mL) of sulphone and sulphoxide metabolites of Fexinidazole after single IV 1 mg/kg dose of Fexinidazole in male Beagle dogs.

| Sulphone | | | | | | |
|---|---|---|---|---|---|---|
| Time (h) | Dog ID | | | Mean | SD | %CV |
| | 1979 | 2197 | 2205 | | | |
| Pre-dose | <5.00 | 9.25[1] | <5.00 | N/A | N/A | N/A |
| 0.083 | 25.2 | 57.8 | 46.5 | 43.2 | 16.6 | 38 |
| 0.25 | 92.7 | 133 | 145 | 124 | 27.4 | 22 |
| 0.5 | 152 | 255 | 255 | 221 | 59.5 | 27 |
| 1 | 290 | 329 | 426 | 348 | 70 | 20 |
| 2 | 506 | 399 | 632 | 512 | 117 | 23 |
| 4 | 725 | 494 | 785 | 668 | 154 | 23 |
| 6 | 763 | 453 | 670 | 629 | 159 | 25 |
| 10 | 621 | 263 | 472 | 452 | 180 | 40 |
| Sulphoxide | | | | | | |
| Time (h) | Dog ID | | | Mean | SD | %CV |
| | 1979 | 2197 | 2205 | | | |
| Pre-dose | <5.00 | <5.00 | <5.00 | N/A | N/A | N/A |
| 0.083 | 597 | 811 | 593 | 667 | 125 | 19 |
| 0.25 | 721 | 797 | 757 | 758 | 38 | 5 |
| 0.5 | 666 | 766 | 651 | 694 | 62.5 | 9 |
| 1 | 561 | 421 | 485 | 489 | 70.1 | 14 |
| 2 | 353 | 225 | 237 | 272 | 70.7 | 26 |
| 4 | 126 | 73.3 | 75.3 | 91.5 | 29.9 | 33 |
| 6 | 30.7 | 22.3 | 18.4 | 23.8 | 6.29 | 26 |
| 10 | <5.00 | <5.00 | <5.00 | N/A | N/A | N/A |

[1] Value was likely due to contamination

N/A: not applicable.

**Table 3A1.** Individual and mean (±SD, %CV) plasma concentrations (ng/mL) of Fexinidazole after single oral 10 mg/kg dose of the compound in male Beagle dogs.

| Time (h) | Dog ID | | | Mean | SD | %CV |
|---|---|---|---|---|---|---|
| | 1979 | 2197 | 2205 | | | |
| Pre-dose | <5.00 | <5.00 | <5.00 | N/A | N/A | N/A |
| 0.25 | 14.4 | 11.5 | <5.00 | 8.63 | 7.62 | 88 |
| 0.5 | 28.8 | 26.2 | 8.34 | 21.1 | 11.1 | 53 |
| 1 | 22.8 | 20.2 | 17.6 | 20.2 | 2.6 | 13 |
| 2 | 21.4 | 14.7 | 11.1 | 15.7 | 5.23 | 33 |
| 4 | 6.25 | 8.57 | 10.2 | 8.34 | 1.99 | 24 |
| 6 | <5.00 | <5.00 | <5.00 | N/A | N/A | N/A |
| 8 | <5.00 | <5.00 | <5.00 | N/A | N/A | N/A |
| 24 | <5.00 | <5.00 | <5.00 | N/A | N/A | N/A |

Estimates of mean based on approximation that values below LLOQ are equal to zero.

N/A: not applicable.

**Table 4A1.** Individual and mean (±SD, %CV) plasma concentrations (ng/mL) of sulphone and sulphoxide metabolites of Fexinidazole after single oral 10 mg/kg dose of Fexinidazole in male Beagle dogs.

| Sulphone | | | | | | |
|---|---|---|---|---|---|---|
| Time (h) | Dog ID | | | Mean | SD | %CV |
| | 1979 | 2197 | 2205 | | | |
| Pre-dose | <5.00 | <5.00 | <5.00 | N/A | N/A | N/A |
| 0.25 | 132 | 178 | 24.7 | 112 | 78.7 | 70 |
| 0.5 | 438 | 587 | 130 | 385 | 233 | 61 |
| 1 | 843 | 1030 | 411 | 761 | 317 | 42 |
| 2 | 1670 | 1650 | 961 | 1430 | 404 | 28 |
| 4 | 2680 | 3410 | 2000 | 2700 | 705 | 26 |
| 6 | 2820 | 4140 | 2590 | 3180 | 836 | 26 |
| 8 | 2650 | 3010 | 2820 | 2830 | 180 | 6 |
| 24 | 553 | 429 | 750 | 577 | 162 | 28 |
| Sulphoxide | | | | | | |
| Time (h) | Dog ID | | | Mean | SD | %CV |
| | 1979 | 2197 | 2205 | | | |
| Pre-dose | <5.00 | <5.00 | <5.00 | N/A | N/A | N/A |
| 0.25 | 961 | 1120 | 145 | 742 | 523 | 71 |
| 0.5 | 1960 | 1990 | 703 | 1550 | 735 | 47 |
| 1 | 2030 | 1900 | 1610 | 1850 | 215 | 12 |
| 2 | 1960 | 1590 | 1690 | 1750 | 191 | 11 |
| 4 | 693 | 1050 | 1490 | 1080 | 399 | 37 |
| 6 | 197 | 363 | 493 | 351 | 148 | 42 |
| 8 | 52.2 | 80.2 | 110 | 80.8 | 28.9 | 36 |
| 24 | 9.68 | 29.5 | 36.9 | 25.4 | 14.1 | 56 |
| N/A: not applicable. | | | | | | |

# Appendix 2. Bioanalytical method

Fexinidazole, Fexinidazole Sulphoxide (M1) and Fexinidazole Sulphone (M2) (as free base) were assayed using an LC-MS-MS method. Bioanalytical data are stored in Watson LIMS under Watson study 0320-2007 in the 348-Fexinidazole project.

Plasma Sample Preparation
Standards were prepared using dog plasma. Plasma proteins were precipitated by adding 200 μL of methanol to 25 μL of plasma in a 96 well plate. After capping and vortex mixing, the plate was centrifuged for 10 minutes at 4000 rpm at 6°C. An aliquot of 100 μL of supernatant was transferred in a 96 well plate and mixed with 100 μL of 10 mM ammonium formate pH 3.5 injected onto the LC-MS-MS system.

LC-MS/MS conditions

HPLC system: Hewlett Packard 1100 series
Mobile phase: Channel A: Ammonium Formate (10 mM pH 3.5)
Channel B: Methanol

Elution mode: Gradient

Elution conditions:

| Time (min) | 0.0 | 2.10 | 2.30 | 5.00 | 5.20 | 6.00 |
|---|---|---|---|---|---|---|
| % A | 65 | 65 | 40 | 40 | 65 | 65 |
| % B | 35 | 35 | 60 | 60 | 35 | 35 |

Total Run Time: 6.0 minutes
Flow rate: 1.0 mL/min
Approximate retention time: Fexinidazole: about 4.7 min.
M1: about 2.00 min
M2: about 2.15 min
Column oven temp.: 40 °C
Analytical column: Chromolith RP-18 50 * 4.6 mm (Merck)
Autosampler type: Perkin Elmer PE 200
Injection volume: 10 μL
Autosampler temperature: RT

MS instrument: Perkin Elmer SCIEX API 4000
Ionisation: TURBO ION SPRAY in positive ion mode
MRM transitions:

| | | |
|---|---|---|
| Fexinidazole: | m/z 280.2 | m/z 140.2 |
| M1 | m/z 296.2 | m/z 140.2 |
| M2 | m/z 312.2 | m/z 140.2 |

Resolution Q1: Unit
Q3: Unit
LLOQ: 5.00 ng/mL

ULOQ: 5000 ng/mL

Batch No. of standard: N/A

Software used

Acquisition and processing: Analyst 1.4

Import data from Analyst file: 0320-2007-Run1.rdb

Data file in Analyst: 0320-2007\Run1.wiff

# Appendix 3. Analytical performance

**Table 1A3. Analytical Performance: Back-Calculated Concentrations (ng/mL) of Fexinidazole Calibration Standard in Dog Plasma for Study Protocol 0320-2007.**

| Assay Date | Analytical Run Number | STD.1 5.00 ng/mL | STD.2 10.0 ng/mL | STD.3 50.0 ng/mL | STD.4 100 ng/mL | STD.5 500 ng/mL | STD.6 1000 ng/mL | STD.7 4000 ng/mL | STD.8 5000 ng/mL |
|---|---|---|---|---|---|---|---|---|---|
| 07-Nov-2007 | 1 | 4.59 | 9.01 | 46 | 96.6 | 534 | 1000 | 4140 | 5280 |
| | | 5.45 | 11.1 | *41.4 | 88.4 | 481 | 953 | 4340 | 5250 |
| | | | | | | | | | |
| Mean | | 5.02 | 10.1 | 46 | 92.5 | 508 | 977 | 4240 | 5270 |
| SD | | 0.608 | 1.48 | | 5.8 | 37.5 | 33.2 | 141 | 21.2 |
| %CV | | 12.1 | 14.7 | | 6.3 | 7.4 | 3.4 | 3.3 | 0.4 |
| %Bias | | 0.4 | 1 | -8 | -7.5 | 1.6 | -2.3 | 6 | 5.4 |
| n | | 2 | 2 | 1 | 2 | 2 | 2 | 2 | 2 |

* Reason Deactivated : Accuracy more than 15%.

**Table 2A3. Calibration Curve Parameters for Fexinidazole Calibration Standards in Dog Plasma for Study Protocol 0320-2007.**

| Run Date | Curve Number | Slope | Intercept | $R^2$ | LLOQ ng/mL | ULOQ ng/mL | Regression Footnote(s) |
|---|---|---|---|---|---|---|---|
| 07-Nov-2007 | 1 | 2056.37 | -1724.58 | 0.9926 | 5 | 5000 | 1 |
| | | | | | | | |
| Mean | | 2056.37 | -1724.58 | 0.9926 | | | |
| n | | 1 | 1 | 1 | | | |

Regression Footnote(s):
1) Resp. = Slope * Conc. + Intercept

| **Table 3A3.** **Analytical Performance: Back-Calculated Concentrations (ng/mL) of Fexinidazole Sulphoxide Calibration Standard in Dog Plasma for Study Protocol 0320-2007.** | | | | | | | | | |
|---|---|---|---|---|---|---|---|---|---|
| Assay Date | Analytical Run Number | STD.1 5.00 ng/mL | STD.2 10.0 ng/mL | STD.3 50.0 ng/mL | STD.4 100 ng/mL | STD.5 500 ng/mL | STD.6 1000 ng/mL | STD.7 4000 ng/mL | STD.8 5000 ng/mL |
| 07-Nov-2007 | 1 | 4.49 | 8.81 | 43.4 | 95.3 | 556 | 1040 | 4030 | 5080 |
| | | 5.88 | *13.6 | *40.3 | *82.4 | 491 | 967 | 4120 | 5350 |
| | | | | | | | | | |
| Mean | | 5.19 | 8.81 | 43.4 | 95.3 | 524 | 1000 | 4080 | 5220 |
| SD | | 0.983 | | | | 46 | 51.6 | 63.6 | 191 |
| %CV | | 18.9 | | | | 8.8 | 5.2 | 1.6 | 3.7 |
| %Bias | | 3.8 | -11.9 | -13.2 | -4.7 | 4.8 | 0 | 2 | 4.4 |
| n | | 2 | 1 | 1 | 1 | 2 | 2 | 2 | 2 |
| * Reason Deactivated : Accuracy more than 15%. | | | | | | | | | |

| **Table 4A3.** **Calibration Curve Parameters for Fexinidazole Sulphoxide Calibration Standards in Dog Plasma for Study Protocol 0320-2007.** | | | | | | | |
|---|---|---|---|---|---|---|---|
| Run Date | Curve Number | Slope | Intercept | $R^2$ | LLOQ ng/mL | ULOQ ng/mL | Regression Footnote(s) |
| 07-Nov-2007 | 1 | 1616.65 | -2015.67 | 0.9903 | 5 | 5000 | 1 |
| | | | | | | | |
| Mean | | 1616.65 | -2015.67 | 0.9903 | | | |
| n | | 1 | 1 | 1 | | | |
| Regression Footnote(s):<br>1) Resp. = Slope * Conc. + Intercept | | | | | | | |

| **Table 5A3. Analytical Performance: Back-Calculated Concentrations (ng/mL) of Fexinidazole Sulphone Calibration Standard in Dog Plasma for Study Protocol 0320-2007.** | | | | | | | | | |
|---|---|---|---|---|---|---|---|---|---|
| Assay Date | Analytical Run Number | STD.1 5.00 ng/mL | STD.2 10.0 ng/mL | STD.3 50.0 ng/mL | STD.4 100 ng/mL | STD.5 500 ng/mL | STD.6 1000 ng/mL | STD.7 4000 ng/mL | STD.8 5000 ng/mL |
| 07-Nov-2007 | 1 | 4.59 | 8.86 | 44.2 | 99.5 | 549 | 1020 | 4200 | 5320 |
| | | 5.79 | *13.3 | *40.8 | 85.1 | 480 | 951 | 4270 | 5510 |
| | | | | | | | | | |
| Mean | | 5.19 | 8.86 | 44.2 | 92.3 | 515 | 986 | 4240 | 5420 |
| SD | | 0.849 | | | 10.2 | 48.8 | 48.8 | 49.5 | 134 |
| %CV | | 16.4 | | | 11.1 | 9.5 | 4.9 | 1.2 | 2.5 |
| %Bias | | 3.8 | -11.4 | -11.6 | -7.7 | 3 | -1.4 | 6 | 8.4 |
| n | | 2 | 1 | 1 | 2 | 2 | 2 | 2 | 2 |
| * Reason Deactivated : Accuracy more than 15%. | | | | | | | | | |

| **Table 6A3. Calibration Curve Parameters for Fexinidazole Sulphone Calibration Standards in Dog Plasma or Study Protocol 0320-2007.** | | | | | | | |
|---|---|---|---|---|---|---|---|
| Run Date | Curve Number | Slope | Intercept | $R^2$ | LLOQ ng/mL | ULOQ ng/mL | Regression Footnote(s) |
| 07-Nov-2007 | 1 | 2414.79 | -2648.69 | 0.9893 | 5 | 5000 | 1 |
| | | | | | | | |
| Mean | | 2414.79 | -2648.69 | 0.9893 | | | |
| SD | | | | | | | |
| %CV | | | | | | | |
| n | | 1 | 1 | 1 | | | |
| Regression Footnote(s):<br>1) Resp. = Slope * Conc. + Intercept | | | | | | | |

## Appendix 4. Clinical signs

| Individual Animal Clinical Signs for Study Protocol 0320-2007. | | | |
|---|---|---|---|
| Animal ID | Clinical Signs | Day Present | Study Day(s) Noted |
| 1979 | Normal/No significant signs | 21 | Pretest phase 1-10 |
| | | | Test Period 1-7, 10-11 |
| | | | Test Period (2) 1-2 |
| | Diarrhea, Slight | 2 | Test Period 8-9 |
| 2197 | Normal/No significant signs | 23 | Pretest phase 1-10 |
| | | | Test Period 1-11 |
| | | | Test Period (2) 1-2 |
| 2205 | Normal/No significant signs | 23 | Pretest phase 1-10 |
| | | | Test Period 1-11 |
| | | | Test Period (2) 1-2 |
| Test Period: IV Dose<br>Test Period (2): Oral Dose | | | |

## Appendix 5. Body Weight

| Dog ID | Body weight (kg) | | |
|---|---|---|---|
| | Pre-test | IV (Day 1) | Oral (Day 12) |
| 1979 | 9.76 | 9.60 | 9.70 |
| 2197 | 8.54 | 8.42 | 8.79 |
| 2205 | 7.52 | 7.33 | 7.71 |
| Mean | 8.60 | 8.45 | 8.74 |
| SD | 1.12 | 1.14 | 0.997 |
