# Supplementary material for: Fexinidazole – A New Oral Nitroimidazole Drug Candidate Entering Clinical Development for the Treatment of Sleeping Sickness
Source: PLoS Negl Trop Dis. 2010 Dec 21;4(12):e923. doi: 10.1371/journal.pntd.0000923 (PMC3006138; doi:10.1371/journal.pntd.0000923)

Nifurtimox, Fexinidazole: Evaluation of the Pharmacokinetics after 5-day repetitive Oral Administration of the compounds to male NMRI mice.

Products Name:	Nifurtimox, Fexinidazole
Study Number:	0403-2007
Study Director:	
Status:	FINAL

TABLE OF CONTENTS

1. STUDY CONDUCT 4
2. OBJECTIVE 4
3. ABBREVIATIONS 4
4. METHODS 5
4.1. Study Design 5
4.2. Formulation Preparation and Concentration Check 5
4.3. Sample Collection and Handling 5
4.4. Bioanalytical Method 5
4.5. Pharmacokinetic Calculations 5
5. RESULTS 6
6. CONCLUSIONS 7
7. CONTRIBUTORS 7
8. ARCHIVING 7
9. REFERENCES 7
TABLES AND FIGURES 9
APPENDICES
Appendix 1. Individual and mean plasma concentrations 13
Appendix 2. Bioanalytical method for Nifurtimox 14
Appendix 3. Analytical performance for Nifurtimox 15
Appendix 4. Bioanalytical method for Fexinidazole 16
Appendix 5. Analytical performance for Fexinidazole 16

1. STUDY CONDUCT

The study, sponsored by Drugs for Neglected Diseases *initiative* (DND*i*), was performed within Accelera, Nerviano Medical Sciences, Italy according the internal Standard Operating Procedures as a non-GLP regulated study.

2. OBJECTIVE

Aim of this study was to evaluate the pharmacokinetics of Nifurtimox and Fexinidazole after repeated oral doses of the two compounds to male NMRI mice.

3. ABBREVIATIONS

The following abbreviations are used in this document:

$AUC_{0-t(last)}$	Area under the plasma concentration *vs.* time curve up to the last detectable concentration
C_{max}	Maximal plasma concentration
CV	Coefficient of variation of the mean
h	Hours
ID	Animal identification code
LC	Liquid chromatography
LLOQ	Lower limit of quantification
MS	Mass-spectrometry
NMRI	Naval Medical Research Institute
Norm	Normalized value
R^2	Correlation coefficient
SD	Standard deviation of the mean
STD	Standard sample
$t_{1/2,z}$	Apparent terminal half-life
t_{max}	Time to peak plasma concentration
t_{last}	Time of the last detectable concentration
ULOQ	Upper limit of quantification

4. METHODS

4.1. Study Design

The study was conducted according to the study protocol [1].
Nifurtimox and Fexinidazole were given orally to 3 male NMRI mice/compound by gastric gavage daily for 5 consecutive days at a dose of 200 mg/kg in a 5 % tween 80/ 0.5 % methocel vehicle.

4.2. Formulation Preparation and Concentration Check

The Nifurtimox and Fexinidazole suspensions, at the nominal concentration of 20 mg/mL, were prepared according to the procedure already developed [2]; a sufficient volume of each suspension was prepared in order to support the 5-day repeated oral administration. The suspensions were stored at +4°C. The supportive stability data for the Fexinidazole suspension has previously been determined [2] while a stability control on the Nifurtimox suspension after 7 days at +4°C was performed.

The concentration checks on Nifurtimox and Fexinidazole suspensions were performed using a specific, not validated, HPLC-UV method [2]. Three samples were analyzed for each suspension.

4.3. Sample Collection and Handling

About 0.05-0.07 mL of blood/sampling time were taken from the saphenous vein using heparinized tubes (pre-cooled in an ice/water bath) and were centrifuged at 10000g for 3 minutes at 4°C. The separated plasma was stored at -80°C until analysis. Blood was taken at 0.25, 0.5, 1, 3, 8 and 24 h post dosing.

4.4. Bioanalytical Method

Mouse plasma concentrations of Nifurtimox and Fexinidazole were determined using non-validated LC-MS-MS methods. The lower limit of quantification was 9.55 ng/mL for Nifurtimox and 5.00 ng/mL for Fexinidazole. Bioanalytical data are stored in Watson LIMS (v. 6.4.0.04, Thermo Fisher Scientific, Waltham, MA, USA) under Project ID: 348-Fexinidazole and Study ID 0403-2007. Details of the bioanalytical methods are reported in Appendix 2 for Nifurtimox and in Appendix 4 for Fexinidazole. Analytical performance of calibration curves is reported in Appendix 3 for Nifurtimox and in Appendix 5 for Fexinidazole.

4.5. Pharmacokinetic Calculations

Pharmacokinetic evaluation was carried out using a non-compartmental approach with the aid of the Watson package (v. 6.4.0.04, Thermo Fisher Scientific, Waltham, MA, USA).

For the calculations, pre-dose concentrations of both compounds were set equal to zero. After both compounds, on Day 5, C_{max} and t_{max} were read from the raw plasma data as the coordinates of the highest measured concentration. The area under plasma concentration *vs.*

time curve to finite time, $AUC_{0-t(last)}$, was determined by the linear trapezoidal rule up to the last detectable concentration. The half-life of the terminal phase, $t_{1/2,z}$, was determined by linear regression analysis of the natural-log concentration *vs.* time curve, where $t_{1/2,z}$ = ln(2)/slope of the regression line.
C_{max} and $AUC_{0-t(last)}$ values were also normalized to a 1 mg/kg dose level.

Descriptive statistics (mean ± SD, %CV) were reported for plasma concentrations and pharmacokinetic parameters sorted by compound.

Plasma concentrations and pharmacokinetic parameters of Nifurtimox and Fexinidazole were reported to three significant figures.

5. RESULTS

Summary data of Day 5 mean ±SD pharmacokinetic parameters of Nifurtimox and Fexinidazole are reported in Table 1. Individual and mean pharmacokinetic parameters of Nifurtimox and Fexinidazole are reported in Tables 2 and 3, respectively. Individual and mean plasma concentrations of both compounds are plotted in Figures 1 - 3 and reported in Tables 1A1 and 2A1 of Appendix 1.

Dose concentrations of both Nifurtimox and Fexinidazole are reported in the following table

Sample	Nominal Concentration (mg/mL)	HPLC Concentration (mg/mL)	HPLC Assay %
Nifurtimox Suspension	20	19.1	95.5
Fexinidazole Suspension	20	19.3	96.5

Nifurtimox and Fexinidazole suspensions were within the limits of 95-105 % of the nominal concentration. Nifurtimox suspension was stable within the limits of 90-110 % as reported in the following table

Stability point	HPLC Concentration (mg/mL)	HPLC Assay %	Stability %
Initial Time	19.1	95.5	100
7 days at + 4°C	18.2	90.8	95.1

After daily oral administrations of Nifurtimox for five days, detectable concentrations of the compound were measured 15 minutes post dosing on Day 5. Mean ±SD maximal plasma concentration of Nifurtimox was 9250 ±765 ng/mL, achieved within 3 hours post dosing. The concentrations of the compound were detectable up to 8 h post dosing. Mean ±SD $AUC_{0-t(last)}$ was 45700 ±5880 ng·h/mL. The coefficients of variation of C_{max} and $AUC_{0-t(last)}$ were 8 and 13 %, respectively. Mean ±SD apparent terminal half-life of the compound was 3.12 ±1.45 h.

After daily oral administrations of Fexinidazole for five days detectable concentrations of Fexinidazole were measured 15 minutes post dosing on Day 5. Mean ±SD maximal plasma concentration of Fexinidazole was 457 ±179 ng/mL, achieved within 1 hour post dosing. The concentrations of the compound were detectable up to 8 h post dosing. Mean ±SD $AUC_{0-t(last)}$ was 1530 ±348 ng·h/mL. The coefficients of variation of C_{max} and $AUC_{0-t(last)}$ were 39 and 23 %, respectively. Mean ±SD apparent terminal half-life of the compound was 2.76 ±1.26 h.

6. CONCLUSIONS

After both compounds, virtually at steady state assuming invariance of the half-life after single and repeated administrations, Day 5 plasma profiles were similar. The variability of the systemic exposure descriptors was low (CV% < 40%). Systemic exposure parameters of Nifurtimox were markedly higher than those of Fexinidazole.

The comparison between systemic exposure to Fexinidazole after single 50 mg/kg (on average, C_{max}: 154 ng/mL, $AUC_{0-t(last)}$: 416 ng·h/mL [3]) and repeated 200 mg/kg/day doses of Fexinidazole with 5 % tween 80 in 0.5 % methocel indicated that both C_{max} and AUC values increased in direct proportion with the dose, suggesting dose- and time-independent pharmacokinetics of Fexinidazole after oral dosing in this dose range.

7. CONTRIBUTORS

8. ARCHIVING

The protocol, raw data, pharmacokinetic analysis and final report were archived within Accelera Archive, Nerviano Medical Sciences, Italy, according the Unit Standard Operating Procedures.

9. REFERENCES

1. Nifurtimox, Fexinidazole: Evaluation of the Pharmacokinetics after 5-day repetitive Oral Administration of the compounds to male NMRI mice. Nerviano Medical Sciences Study Protocol 0403-2007-P, November 7, 2007.
2. Fexinidazole: Preformulation Study Results. Nerviano Medical Sciences report no. 0221-2007-R.

3. Fexinidazole: Evaluation of the Pharmacokinetics after Single IV and Oral (two formulations) Administration to male NMRI mice. Nerviano Medical Sciences report no. 0275-2007-R.

TABLES AND FIGURES

Table 1. Summary table of Day 5 mean ±SD pharmacokinetic parameters of Nifurtimox and Fexinidazole after repeated oral 200 mg/kg/day doses of the compounds in male NMRI mice.

Parameter (Unit)	Nifurtimox	Fexinidazole
C_{max} (ng/mL)	9250 ±765	457 ±179
t_{max} (h)	1.5 ±1.32	0.833 ±0.289
t_{last} (h)	8 ±0	8 ±0
$AUC_{0-t(last)}$ (ng·h/mL)	45700 ±5880	1530 ±348
$t_{1/2,z}$ (h)	3.12 ±1.45	2.76 ±1.26

Table 2. Day 5 individual and mean (±SD, %CV) pharmacokinetic parameters of Nifurtimox after repeated oral 200 mg/kg/day of the compound in male NMRI mice.

Parameter (Unit)	Mouse ID			Mean	SD	%CV
	M1	M2	M3			
Weight (g)	26.6	28.1	25.1	26.6	1.5	6
Dose (mg/kg)	194	190	190	191	2.07	1
C_{max} (ng/mL)	8380	9820	9550	9250	765	8
t_{max} (h)	3	0.5	1	1.5	1.32	88
t_{last} (h)	8	8	8	8	0	0
$AUC_{0-t(last)}$ (ng·h/mL)	50100	39000	47900	45700	5880	13
Regression Range (h)	1 - 8	0.5 - 8	1 - 8	N/A	N/A	N/A
$t_{1/2,z}$ (h)	4.76	2.59	2.01	3.12	1.45	47
C_{max}, norm (1)	43.2	51.6	50.2	48.3	4.5	9
$AUC_{0-t(last)}$, norm (1)	259	205	252	239	29.4	12

N/A: not applicable

(1) C_{max} (ng/mL) and AUC (ng·h/mL) normalized to 1 mg/kg dose.

Table 3. Day 5 individual and mean (±SD, %CV, n=3) pharmacokinetic parameters of Fexinidazole after repeated oral 200 mg/kg/day of the compound in male NMRI mice.

Parameter (Unit)	Mouse ID			Mean	SD	%CV
	M4	M5	M6			
Weight (g)	31.1	32.2	31.1	31.5	0.635	2
Dose (mg/kg)	192	192	192	192	0.335	0
C_{max} (ng/mL)	318	394	659	457	179	39
t_{max} (h)	1	1	0.5	0.833	0.289	35
t_{last} (h)	8	8	8	8	0	0
$AUC_{0\text{-}t(last)}$ (ng·h/mL)	1300	1360	1930	1530	348	23
Regression Range (h)	1 - 8	1 - 8	0.5 - 8	N/A	N/A	N/A
$t_{1/2,z}$ (h)	4.1	1.59	2.59	2.76	1.26	46
C_{max}, norm (1)	1.65	2.05	3.43	2.38	0.934	39
$AUC_{0\text{-}t(last)}$, norm (1)	6.78	7.1	10	7.96	1.77	22

N/A: not applicable

(1) C_{max} (ng/mL) and AUC (ng·h/mL) normalized to 1 mg/kg dose.

Figure 1. Day 5 individual plasma concentrations (ng/mL) of Nifurtimox after repeated oral 200 mg/kg/day of the compound in male NMRI mice.

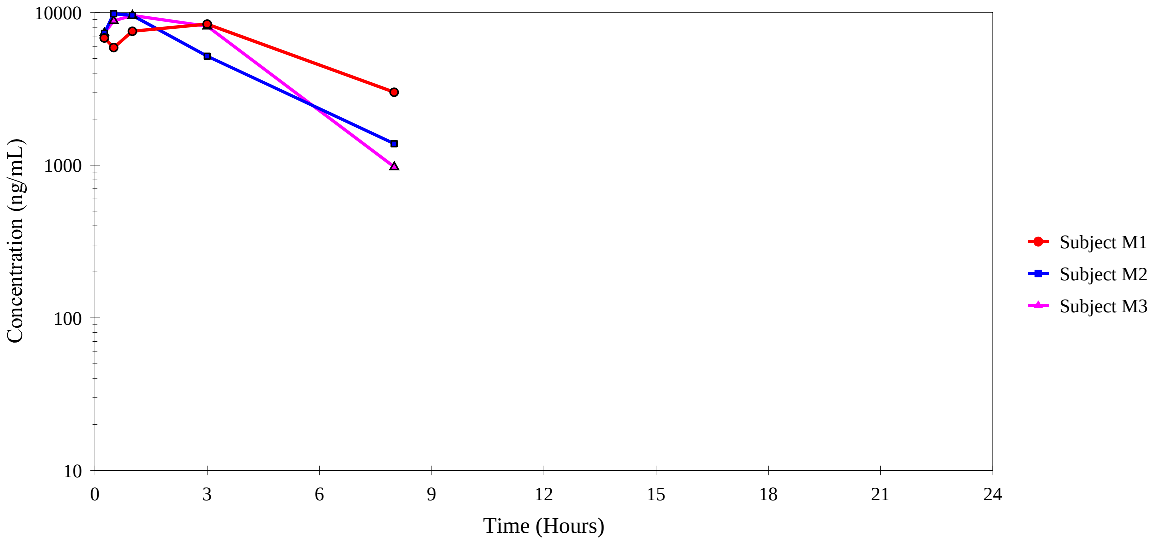

Figure 2. Day 5 individual plasma concentrations (ng/mL) of Fexinidazole after repeated oral 200 mg/kg/day of the compound in male NMRI mice.

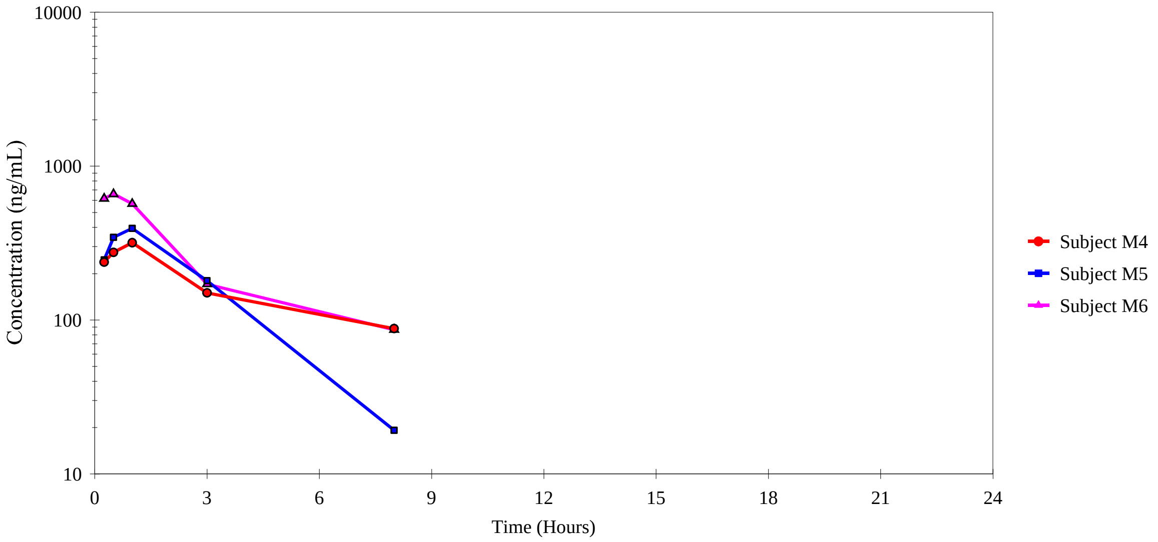

Figure 3. Day 5 mean (±SD) plasma concentrations (ng/mL) of Nifurtimox and Fexinidazole after repeated oral 200 mg/kg/day doses of the compounds in male NMRI mice.

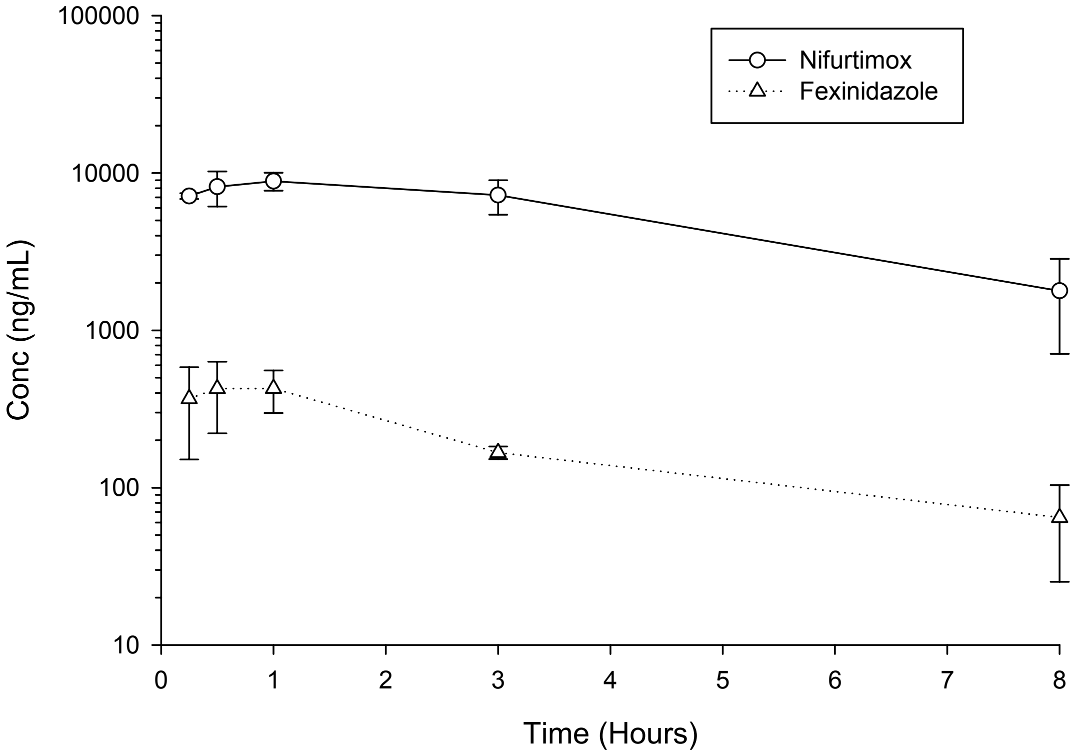

APPENDICES

Appendix 1. Individual and mean plasma concentrations

Table 1A1. Day 5 individual and mean (±SD, %CV) plasma concentrations (ng/mL) of Nifurtimox after repeated oral 200 mg/kg/day of the compound in male NMRI mice.

Time (h)	Mouse ID			Mean	SD	%CV
	M1	M2	M3			
0.25	6800	7290	7330	7140	295	4
0.5	5880	9820	8810	8170	2050	25
1	7530	9560	9550	8880	1170	13
3	8380	5170	8120	7220	1780	25
8	3000	1380	972	1780	1070	60
24	<9.55	<9.55	<9.55	N/A	N/A	N/A

N/A: not applicable

Table 2A1. Day 5 individual and mean (±SD, %CV) plasma concentrations (ng/mL) of Fexinidazole after repeated oral 200 mg/kg/day of the compound in male NMRI mice.

Time (h)	Mouse ID			Mean	SD	%CV
	M4	M5	M6			
0.25	238	246	616	367	216	59
0.5	275	345	659	426	205	48
1	318	394	570	427	129	30
3	150	180	171	167	15.4	9
8	87.9	19.2	86.5	64.5	39.3	61
24	<5	<5	<5	N/A	N/A	N/A

N/A: not applicable

Appendix 2. Bioanalytical method for Nifurtimox

Plasma Sample Preparation:

Standards were prepared using mouse plasma. Plasma proteins were precipitated by adding 150 µL of methanol to 20 µL of plasma in a 96 well plate. After capping and vortex mixing, the plate was centrifuged for 15 minutes at 2060 g at 6°C. An aliquot of 10 µL of the supernatant was injected onto the LC-MS-MS system.

LC-MS/MS conditions:

HPLC system: Hewlett Packard 1100 series
Mobile phase Channel A: Ammonium Formate (10 mM pH 3.5)
Channel B: Acetonitrile
Elution mode Gradient
Elution conditions

Time (min)	0.0	0.2	3.0	3.5	3.6	5.0
% A	90	90	10	10	90	90
% B	10	10	90	90	10	10

Total Run Time: 5.0 minutes
Flow rate: 1.0 mL/min
Approximate retention time Nifurtimox: about 2.8 min.

Column oven temp. 45 °C
Analytical column: Zorbax SB C8 75*4.6 mm, 3.5 µm

Autosampler type: CTC PAL
Injection volume: 10 µL
Autosampler temperature: 10 °C

MS instrument: Perkin Elmer SCIEX API 4000
Ionisation: TURBO ION SPRAY in positive ion mode
MRM transition: Fexinidazole: 288 → 148 m/z

Resolution Q1 Unit
Q3 Low
LLOQ 9.55 ng/mL

ULOQ 9950 ng/mL

Batch No. of standard 110817-0157-001

Software used

Acquisition and processing: Analyst 1.4.1
Import data from Analyst file: 0403-2007-Run2a.rdb
Data file in Analyst: 0403-2007\Run2.wiff

Appendix 3. Analytical performance for Nifurtimox

Table 1A3. Analytical Performance: Back-Calculated Concentrations (ng/mL) of Nifurtimox Calibration Standard in Mouse Plasma for Study Protocol 0403-2007.

Assay Date	Analytical Run Number	STD.1 9.55 ng/mL	STD.2 20.6 ng/mL	STD.3 50.0 ng/mL	STD.4 95.5 ng/mL	STD.5 256 ng/mL	STD.6 500 ng/mL	STD.7 955 ng/mL	STD.8 5100 ng/mL	STD.9 9950 ng/mL
29-Nov-2007	2	8.64	19.8	55	95.2	279	516	1040	6040	9240
		10.6	*11.3	48.9	84.6	209	463	819	5980	9210
Mean		9.62	19.8	52	89.9	244	490	930	6010	9230
SD		1.39		4.31	7.5	49.5	37.5	156	42.4	21.2
%CV		14.4		8.3	8.3	20.3	7.7	16.8	0.7	0.2
%Bias		0.7	-3.9	4	-5.9	-4.7	-2	-2.6	17.8	-7.2
n		2	1	2	2	2	2	2	2	2

* Accuracy more than 20%; excluded from regression analysis.

Table 2A3. Calibration Curve Parameters for Nifurtimox Calibration Standards in Mouse Plasma for Study Protocol 0403-2007.

Run Date	Curve Number	A	B	C	R^2	LLOQ ng/mL	ULOQ ng/mL	Regression Footnote(s)
29-Nov-2007	2	0.0017842	50.6596	63.9823	0.9818	9.55	9950	1
Mean		0.0017842	50.6596	63.9823	0.9818			
SD								
%CV								
n		1	1	1	1			

Regression Footnote(s):
1) Resp. = A * (Conc.**2) + B * Conc. + C

Appendix 4. Bioanalytical method for Fexinidazole

Plasma Sample Preparation:
Standards were prepared using mouse plasma. Plasma proteins were precipitated by adding 200 μL of methanol to 25 μL of plasma in a 96 well plate. After capping and vortex mixing, the plate was centrifuged for 10 minutes at 2060 g at 6°C. An aliquot of 100 μL of supernatant was transferred in a 96 well plate and mixed with 100 μL of 10 mM ammonium formate pH 3.5 injected onto the LC-MS-MS system.

LC-MS/MS conditions:

HPLC system: Hewlett Packard 1100 series
Mobile phase: Channel A: Ammonium Formate (10 mM pH 3.5)
Channel B: Methanol

Elution mode: Gradient
Elution conditions:

Time (min)	0.0	2.10	2.30	5.00	5.20	6.00
% A	65	65	40	40	65	65
% B	35	35	60	60	35	35

Total Run Time: 6.0 minutes
Flow rate: 1.0 mL/min
Approximate retention time: Fexinidazole: about 4.7 min.

Column oven temp.: 40 °C
Analytical column: Chromolith RP-18 50 * 4.6 mm (Merck)

Autosampler type: Perkin Elmer PE 200
Injection volume: 10 μL
Autosampler temperature: RT

MS instrument: Perkin Elmer SCIEX API 4000
Ionisation: TURBO ION SPRAY in positive ion mode
MRM transition: Fexinidazole: 280 → 140 m/z

Resolution Q1: Unit
Q3: Unit
LLOQ: 5.00 ng/mL

ULOQ: 5000 ng/mL

Batch No. of standard: 07285/23

Software used

Acquisition and processing: Analyst 1.4.1
Import data from Analyst file: 0403-2007-Run1.rdb
Data file in Analyst: 0403-2007\Run1.wiff

Appendix 5. Analytical performance for Fexinidazole

Table 1A5. Analytical Performance: Back-Calculated Concentrations (ng/mL) of Fexinidazole Calibration Standard in Mouse Plasma for Study Protocol 0403-2007.

Assay Date	Analytical Run Number	STD.1 5.00 ng/mL	STD.2 10.0 ng/mL	STD.3 50.0 ng/mL	STD.4 100 ng/mL	STD.5 500 ng/mL	STD.6 1000 ng/mL	STD.7 4000 ng/mL	STD.8 5000 ng/mL
26-Nov-2007	1	5.58	9.7	55.6	103	453	965	4240	5480
		4.47	*7.74	47.2	95.5	*411	929	4060	5040
Mean		5.03	9.7	51.4	99.3	453	947	4150	5260
SD		0.785		5.94	5.3		25.5	127	311
%CV		15.6		11.6	5.3		2.7	3.1	5.9
%Bias		0.6	-3	2.8	-0.7	-9.4	-5.3	3.8	5.2
n		2	1	2	2	1	2	2	2

* Accuracy more than 15%; excluded from regression analysis.

Table 2A5. Calibration Curve Parameters for Fexinidazole Calibration Standards in Mouse Plasma for Study Protocol 0403-2007.

Run Date	Curve Number	Slope	Intercept	R^2	LLOQ ng/mL	ULOQ ng/mL	Regression Footnote(s)
26-Nov-2007	1	2103.67	1116.07	0.9931	5	5000	1
Mean		2103.67	1116.07	0.9931			
SD							
%CV							
n		1	1	1			

Regression Footnote(s):
1) Resp. = Slope * Conc. + Intercept

Amendment 1

Nifurtimox, Fexinidazole: Evaluation of the Pharmacokinetics after 5-day repetitive oral administration of the compounds to Male NMRI Mice.

Study Number	0403-2007
Document Number	0403-2007-R
Amendment Number:	1
Test Article:	Fexinidazole
Study Director:	

1. SPECIFIC CHANGE(S)

1.1. Description(s) of Change(s)

After specific request of the Sponsor, the remaining stored plasma aliquots of the blood samples were used to evaluate the pharmacokinetics of the sulphone and sulphoxide metabolites of Fexinidazole.

1.1.1. Reason(s) for Change(s)

Analysis was performed on the remaining plasma aliquots to evaluate the pharmacokinetics of the sulphone and sulphoxide metabolites of Fexinidazole.

1.2. Description(s) of Change(s)

The following Appendix 6 refers to the evaluation of the pharmacokinetics of the sulphone and sulphoxide metabolites of Fexinidazole and has to be intended as integration of the report 0403-2007-R. The report 0403-2007-R was not modified.

1.2.1. Reason(s) for Change(s)

To evaluate the pharmacokinetics of the sulphone and sulphoxide metabolites of Fexinidazole.

Appendix 6. Evaluation of the pharmacokinetics of sulphone and sulphoxide metabolites of Fexinidazole

TABLE OF CONTENTS

APPROVAL SIGNATURES.....2
1. SPECIFIC CHANGE(S).....3
1.1. Description(s) of Change(s).....3
1.1.1. Reason(s) for Change(s).....3
1.2. Description(s) of Change(s).....3
1.2.1. Reason(s) for Change(s).....3
2. ABBREVIATIONS.....5
3. METHODS.....5
3.1. Bioanalytical Method.....5
3.2. Pharmacokinetic Calculations.....5
4. RESULTS.....6
5. CONCLUSIONS.....6
6. CONTRIBUTORS.....7
7. ARCHIVING.....7
8. TABLES AND FIGURES.....8

2. ABBREVIATIONS

The following abbreviations are used in this document:

$AUC_{0-t(last)}$	Area under the plasma concentration *vs.* time curve up to finite time
$AUC_{0-\infty}$	Area under the plasma concentration *vs.* time curve up to infinite time
C_{max}	Maximal plasma concentration
CV	Coefficient of variation of the mean
h	Hours
LC	Liquid chromatography
LLOQ	Lower limit of quantification
MS	Mass-spectrometry
Norm	Normalized value
R^2	Correlation coefficient
SD	Standard deviation of the mean
STD	Standard sample
t_{max}	Time to peak plasma concentration
t_{last}	Time of the last detectable concentration
ULOQ	Upper limit of quantification

3. METHODS

3.1. Bioanalytical Method

Plasma concentrations of sulphoxide and sulphone Fexinidazole metabolites were determined by a LC-MS-MS method. The lower limit of the bioanalytical method was 5 ng/mL (run 1) and 25 ng/mL (run 3) for both compounds. Bioanalytical data were stored in Watson LIMS (v. 6.4.0.04, Thermo Fisher Scientific, Waltham, MA, USA). Details of the bioanalytical method are reported in Table 1. Analytical performance of calibration standards and calibration curve parameters are reported in Tables 2-9.

3.2. Pharmacokinetic Calculations

Pharmacokinetic evaluations of the sulphone and sulphoxide metabolites were carried out using non-compartmental approach with the aid of the Watson package (v. 6.4.0.04, Thermo Fisher Scientific, Waltham, MA, USA).
After both doses, for the calculations, the pre-dose concentrations of the metabolites were set equal to zero.
C_{max} and t_{max} were read from raw data as the coordinates of the highest measured concentration. The area under plasma concentration *vs.* time curve up to finite time, $AUC_{0-t(last)}$, was determined by the linear trapezoidal rule up to the last detectable concentration.

Metabolite to parent ratio was calculated based on C_{max} and $AUC_{0-t(last)}$ values.

C_{max} and $AUC_{0-t(last)}$ values of both metabolites were also normalized to a 1 mg/kg dose level.

The half-life of the terminal phase, $t_{1/2,z}$, was determined by linear regression analysis of the natural-log concentration *vs.* time curve, where $t_{1/2,z}$ = ln(2)/slope of the regression line. The area under the concentration vs. time curve up to infinite time, $AUC_{0-\infty}$, was determined as

$$AUC0-\infty = AUC0 - t(last) + \frac{Ct(last) \cdot t_{1/2,z}}{\ln(2)}$$

Descriptive statistics (mean ±SD, %CV) were reported for plasma concentrations and pharmacokinetic parameters of both metabolites.

Plasma concentrations and pharmacokinetic parameters of both metabolites were reported to three significant figures.

4. RESULTS

Individual and mean plasma concentrations of the sulphone and sulphoxide metabolites of Fexinidazole are reported in Tables 10 and 11, respectively, whilst the corresponding parameters are reported in Tables 12 and 13. Individual plasma concentrations of the sulphone and sulphoxide metabolites are plotted in Figures 1-2 and 3-4, respectively, whilst the corresponding mean concentrations with those of the parent compound are reported in Figure 5.

After repeated oral dosing of Fexinidazole, detectable plasma concentrations of the sulphone metabolite was measured at the first sampling time (mean±SD: 17.6 μg/mL). The maximal concentration of the sulphone metabolite was, on average, 77.6 μg/mL, and was achieved 3 h post dosing. Detectable concentrations of the compound were measured up to the last sampling time (24 h post dosing). The half-life of the compound was on average 2.75 h. The $AUC_{0-t(last)}$ value was, on average, 1050 μg·h/mL with a corresponding $AUC_{0-\infty}$ of 1060 μg·h/mL. The systemic exposure to the metabolite was much higher than that of the parent compound. The metabolite to parent $AUC_{0-t(last)}$ ratio was, on average, 716 with a C_{max} ratio of 192, respectively.

The mean maximal concentration of the sulphoxide was 33.6 μg/mL, achieved earlier than the sulphone metabolite (t_{max} within 1 h post dosing). Detectable concentrations of the compound were measured in two out of three animals up to the last sampling time. The mean $AUC_{0-t(last)}$ value was on average 161 μg·h/mL, with a corresponding $AUC_{0-\infty}$ of 197 μg·h/mL. The half-life of the sulphoxide metabolite was on average 4.31 h. The metabolite to parent $AUC_{0-t(last)}$ ratio was, on average, 101 whereas the corresponding C_{max} ratio was 77.4

5. CONCLUSIONS

After both doses, the coefficient of variation of the mean systemic exposure parameters of both metabolites was low, being at most 25 %.
After repeated oral dosing in NMRI mice, Fexinidazole was extensively metabolized to the sulphone and sulphoxide derivatives.

6. CONTRIBUTORS

7. ARCHIVING

The protocol, raw data, pharmacokinetic analysis and final report were archived within Accelera Archive, Nerviano Medical Sciences, Italy, according the Unit Standard Operating Procedures.

8. TABLES AND FIGURES

Table 1. Bio-analytical method.

Plasma Sample Preparation

Plasma proteins were precipitated by adding 200 µL of methanol containing 50 ng/mL of [$^2H_3$]Fexinidazole as Stable Labelled Internal Standard to 25 µL of mouse plasma in a 96 well plate. After capping and vortex mixing, the plate was centrifuged for 10 minutes at 4000 rpm at 6°C. An aliquot of 100 µL of supernatant was transferred in a 96 well plate and mixed with 200 µL of 10 mM ammonium formate pH 3.5. Aliquots of 10 µL were then injected onto the LC-MS-MS system.

LC-MS/MS conditions

HPLC system:	Hewlett Packard 1100 series
Mobile phase:	Channel A: Ammonium Formate (10 mM pH 3.5)<br>Channel B: Methanol
Elution mode:	Gradient
Elution conditions:	see gradient table below

Time (min)	0.0	2.10	2.30	5.00	5.20	6.00
% A	65	65	40	40	65	65
% B	35	35	60	60	35	35

Total Run Time:	6.0 minutes
Flow rate:	1.0 mL/min
Approximate retention time:	Fexinidazole sulphoxide (M1): about 1.82 min<br>Fexinidazole sulphone (M2): about 1.94 min<br>[$^2H_3$]Fexinidazole: about 4.83
Column oven temperature:	40 °C
Analytical column:	Chromolith RP-18 (50 * 4.6 mm, Merck)
Autosampler type:	CTC PAL
Injection volume:	10 µL
Autosampler temperature:	+4°C
MS instrument:	Perkin Elmer SCIEX API 4000
Ionisation:	TURBO ION SPRAY in positive ion mode
MRM transitions:	M1 m/z 296.2 m/z 140.2<br>M2 m/z 312.2 m/z 140.2<br>[$^2H_3$]Fexinidazole m/z 283.2 m/z 143.2
Resolution: Q1	Unit
Q3	Unit
Software used:	
Acquisition:	Analyst 1.4.1
LIMS database:	Watson v. 6.4.0.04

Table 2. Analytical Performance: Back-Calculated Concentrations (ng/mL) of Fexinidazole Sulphoxide Calibration Standard in Mouse Plasma for Study Protocol 0403-2007.

Assay Date	Analytical Run Number	5.00 ng/mL	10.0 ng/mL	50.0 ng/mL	100 ng/mL	500 ng/mL	1000 ng/mL	4000 ng/mL	5000 ng/mL
26-Nov-2007	1	5.11	10.1	56.6	105	514	980	4050	5200
		4.82	*7.22	46.3	102	466	948	4020	4640
Mean		4.97	10.1	51.5	104	490	964	4040	4920
S.D.		0.205		7.28	2.12	33.9	22.6	21.2	396
%CV		4.1		14.1	2	6.9	2.3	0.5	8
%Bias		-0.6	1	3	4	-2	-3.6	1	-1.6
n		2	1	2	2	2	2	2	2
* Accuracy more than 15%: excluded from regression analysis									

Table 3. Analytical Performance: Back-Calculated Concentrations (ng/mL) of Fexinidazole Sulphoxide Calibration Standard in Mouse Plasma for Study Protocol 0403-2007.

Assay Date	Analytical Run Number	STD.1 25.0 ng/mL	STD.2 50.0 ng/mL	STD.3 250 ng/mL	STD.4 500 ng/mL	STD.5 2500 ng/mL	STD.6 5000 ng/mL	STD.7 22500 ng/mL	STD.8 25000 ng/mL
26-Feb-2008	3	25.9	50.7	*298	536	2780	4820	20200	21400
		22.7	53.8	*299	559	2710	4860	21400	23400
Mean		24.3	52.3		548	2750	4840	20800	22400
S.D.		2.26	2.19		16.3	49.5	28.3	849	1410
%CV		9.3	4.2		3	1.8	0.6	4.1	6.3
%Bias		-2.8	4.6		9.6	10	-3.2	-7.6	-10.4
n		2	2		2	2	2	2	2
* Accuracy more than 15%: excluded from regression analysis									

Table 4. Calibration Curve Parameters for Fexinidazole Sulphoxide Calibration Standards in Mouse Plasma for Study Protocol 0403-2007.

Run Date	Curve Number	Slope	Intercept	R-Squared	LLOQ ng/mL	ULOQ ng/mL	Regression Footnote(s)
26-Nov-2007	1	1574.6	-1167.22	0.9962	5	5000	1
Mean		1574.6	-1167.22	0.9962			
n		1	1	1			
Regression Footnote(s):<br>1) Resp. = Slope * Conc. + Intercept; no Internal Standard was used.							

Table 5. Calibration Curve Parameters for Fexinidazole Sulphoxide Calibration Standards in Mouse Plasma for Study Protocol 0403-2007.

Run Date	Curve Number	Slope	Intercept	R-Squared	LLOQ ng/mL	ULOQ ng/mL	Regression Footnote(s)
26-Feb-2008	3	0.0035315	0.023647	0.9908	25	25000	1
Mean		0.0035315	0.023647	0.9908			
n		1	1	1			
Regression Footnote(s):<br>1) Resp. = Slope * Conc. + Intercept; [$^2H_3$]Fexinidazole as Internal Standard was used.							

Table 6. Analytical Performance: Back-Calculated Concentrations (ng/mL) of Fexinidazole Sulphone Calibration Standard in Mouse Plasma for Study Protocol 0403-2007.

Assay Date	Analytical Run Number	STD.1 5.00 ng/mL	STD.2 10.0 ng/mL	STD.3 50.0 ng/mL	STD.4 100 ng/mL	STD.5 500 ng/mL	STD.6 1000 ng/mL	STD.7 4000 ng/mL	STD.8 5000 ng/mL
26-Nov-2007	1	5.3	10.2	54.9	105	492	970	4160	5450
		4.62	*7.62	47.5	101	439	927	4070	4900
Mean		4.96	10.2	51.2	103	466	949	4120	5180
S.D.		0.481		5.23	2.83	37.5	30.4	63.6	389
%CV		9.7		10.2	2.7	8	3.2	1.5	7.5
%Bias		-0.8	2	2.4	3	-6.8	-5.1	3	3.6
n		2	1	2	2	2	2	2	2
* Accuracy more than 15%: excluded from regression analysis									

Table 7. Analytical Performance: Back-Calculated Concentrations (ng/mL) of Fexinidazole Sulphone Calibration Standard in Mouse Plasma for Study Protocol 0403-2007.

Assay Date	Analytical Run Number	STD.1 25.0 ng/mL	STD.2 50.0 ng/mL	STD.3 250 ng/mL	STD.4 500 ng/mL	STD.5 2500 ng/mL	STD.6 5000 ng/mL	STD.7 22500 ng/mL	STD.8 25000 ng/mL
26-Feb-2008	3	24.6	46.1	268	475	2590	4720	24900	*
		25.8	51.4	261	487	2600	4770	*	*
Mean		25.2	48.8	265	481	2600	4750	24900	
S.D.		0.849	3.75	4.95	8.49	7.07	35.4		
%CV		3.4	7.7	1.9	1.8	0.3	0.7		
%Bias		0.8	-2.4	6	-3.8	4	-5	10.7	
n		2	2	2	2	2	2	1	
* Accuracy more than 15%: excluded from regression analysis									

Table 8. Calibration Curve Parameters for Fexinidazole Sulphone Calibration Standards in Mouse Plasma for Study Protocol 0403-2007.

Run Date	Curve Number	Slope	Intercept	R-Squared	LLOQ ng/mL	ULOQ ng/mL	Regression Footnote(s)
26-Nov-2007	1	2572.52	-1415.72	0.9951	5	5000	1
Mean		2572.52	-1415.72	0.9951			
S.D.							
%CV							
n		1	1	1			

Regression Footnote(s):
1) Resp. = Slope * Conc. + Intercept; no Internal Standard was used.

Table 9. Calibration Curve Parameters for Fexinidazole Sulphone Calibration Standards in Mouse Plasma for Study Protocol 0403-2007.

Run Date	Curve Number	A	B	C	R-Squared	LLOQ ng/mL	ULOQ ng/mL	Regression Footnote(s)
26-Feb-2008	3	-8.63999E-08	0.0045831	-6.37679E-05	0.9968	25	25000	1
Mean		-8.63999E-08	0.0045831	-6.37679E-05	0.9968			
S.D.								
%CV								
n		1	1	1	1			

Regression Footnote(s):
1) Resp. = A * (Conc.**2) + B * Conc. + C; [$^2H_3$]Fexinidazole as Internal Standard was used.

Table 10. Individual and mean (±SD, %CV) plasma concentrations (μg/mL) of sulphone metabolites of Fexinidazole after repeated oral administration at the nominal dose of Fexinidazole of 200 mg/kg to male NMRI mice.

Time Hours	Mouse 4	Mouse 5	Mouse 6	Mean	S.D.	%CV
0.25	42.3	4.19	6.35	17.6	21.4	121.6
0.5	42.3	8.25	11.6	20.7	18.8	90.8
1	47.9	24.8	38.8	37.2	11.6	31.2
3	84.9	81.5	66.5	77.6	9.79	12.6
8	76.1	61.1	65.4	67.5	7.72	11.4
24	0.371	4.26	0.273	1.63	2.27	139.3

Table 11. Individual and mean (±SD, %CV) plasma concentrations (μg/mL) of sulphoxide metabolites of Fexinidazole after repeated oral administration at the nominal dose of Fexinidazole of 200 mg/kg to male NMRI mice.

Time Hours	Mouse 4	Mouse 5	Mouse 6	Mean	S.D.	%CV
0.25	16.8	17.0	31.0	21.6	8.14	37.7
0.5	19.1	21.7	39.4	26.7	11.0	41.2
1	26.7	34.8	33.4	31.6	4.33	13.7
3	13.4	20.0	13.8	15.7	3.70	23.6
8	11.1	3.20	8.05	7.45	3.98	53.4
24	BLQ	0.799	0.0149	0.271	0.457	168.6

Table 12. Individual and mean (±SD, %CV) pharmacokinetic parameters of sulphone metabolite of Fexinidazole after repeated oral 200 mg/kg/day of Fexinidazole in male NMRI mice.

Parameter	Units	Mouse 4	Mouse 5	Mouse 6	Mean	S.D.	%CV
Original Dose	mg/kg/day	192	192	192			
C_{max}	µg/mL	84.9	81.5	66.5	77.6	9.79	12.6
T_{max}	Hours	3	3	3	3		
$AUC_{0\text{-}tlast}$ Interval		(0-24 Hours)	(0-24 Hours)	(0-24 Hours)			
$AUC_{0\text{-}tlast}$	µg*Hours/mL	1190	996	976	1050	118	11.2
$AUC_{0\text{-}\infty}$	µg*Hours/mL	1190	1020	977	1060	113	10.6
$t_{1/2,z}$	Hours	2.08	4.16	2.02	2.75	1.22	44.3
Regression Points	Hours	8, 24	8, 24	8, 24			
C_{max}/Dose	µg/mL	0.441	0.425	0.346	0.404	0.0509	12.6
$AUC_{0\text{-}tlast}$/Dose	µg*Hours/mL	6.16	5.19	5.07	5.47	0.598	10.9
$AUC_{0\text{-}\infty}$/Dose	µg*Hours/mL	6.19	5.32	5.08	5.53	0.584	10.6
C_{max} metabolite/parent		267	207	101	191.8	84.3	43.9
$AUC_{0\text{-}tlast}$ metabolite/parent		909	731	507	716	201	28.1

Table 13. Individual and mean (±SD, %CV) pharmacokinetic parameters of sulphoxide metabolite of Fexinidazole after repeated oral 200 mg/kg/day of Fexinidazole in male NMRI mice.

Parameter	Units	Mouse 4	Mouse 5	Mouse 6	Mean	S.D.	%CV
Original Dose	mg/kg/day	192.38	191.8	192.38			
C_{max}	µg/mL	26.7	34.8	39.4	33.6	6.43	19.1
T_{max}	Hours	1	1	0.5	0.833	0.289	34.6
$AUC_{0\text{-}tlast}$ Interval		(0-8 Hours)	(0-24 Hours)	(0-24 Hours)			
$AUC_{0\text{-}tlast}$	µg*Hours/mL	119	166	197	161	39.3	24.4
$AUC_{0\text{-}\infty}$	µg*Hours/mL	222	171	197	197	25.5	13.0
$t_{1/2,z}$	Hours	6.39	4.44	2.09	4.31	2.15	50.0
Regression Points	Hours	1-8	1-24	0.5-24			
C_{max}/Dose	µg/mL	0.139	0.181	0.205	0.175	0.0334	19.1
$AUC_{0\text{-}tlast}$/Dose	µg*Hours/mL	0.621	0.865	1.03	0.839	0.206	24.5
$AUC_{0\text{-}\infty}$/Dose	µg*Hours/mL	1.15	0.892	1.02	1.02	0.129	12.6
C_{max} metabolite/parent		84.2	88.3	59.8	77.4	15.4	19.9
$AUC_{0\text{-}tlast}$ metabolite/parent		91.6	121.8	103.0	105.5	15.3	14.5

Figure 1: Day 5 Individual plasma concentration (µg/mL) of Fexinidazole Sulphone after 5-day repeated Oral administration of Fexinidazole to male NMRI Mice (Lin-Lin scale)

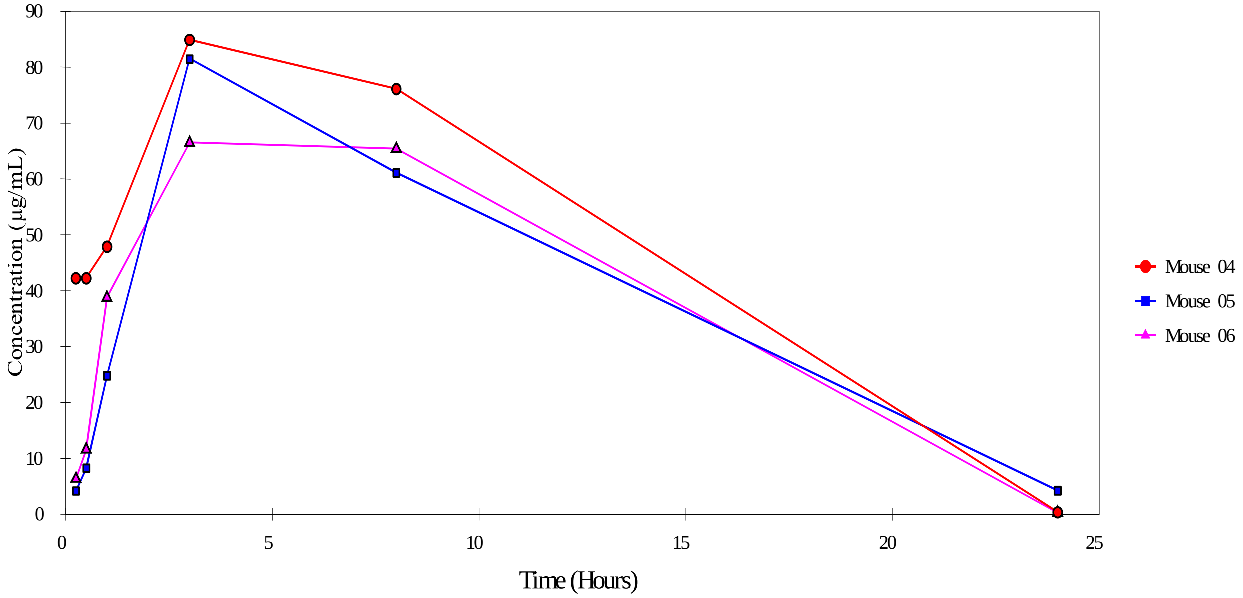

Figure 2: Day 5 Individual plasma concentration (µg/mL) of Fexinidazole Sulphone after 5-day repeated Oral administration of Fexinidazole to male NMRI Mice (Log-Lin scale)

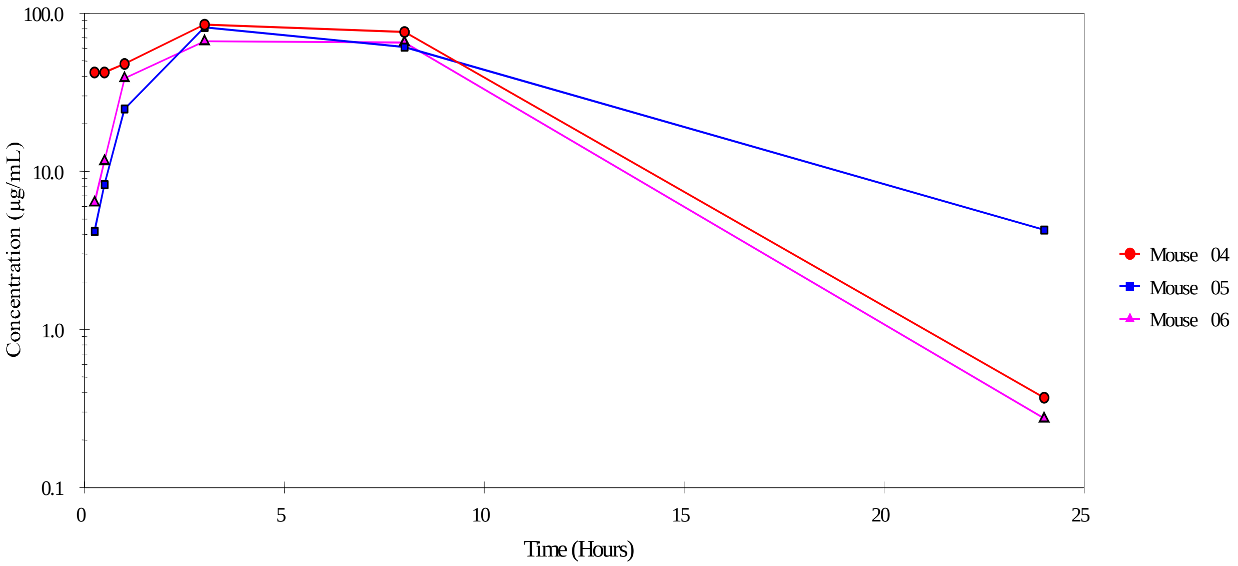

Figure 3: Day 5 Individual plasma concentration (µg/mL) of Fexinidazole Sulphoxide after 5-day repeated Oral administration of Fexinidazole to male NMRI Mice (Lin-Lin scale)

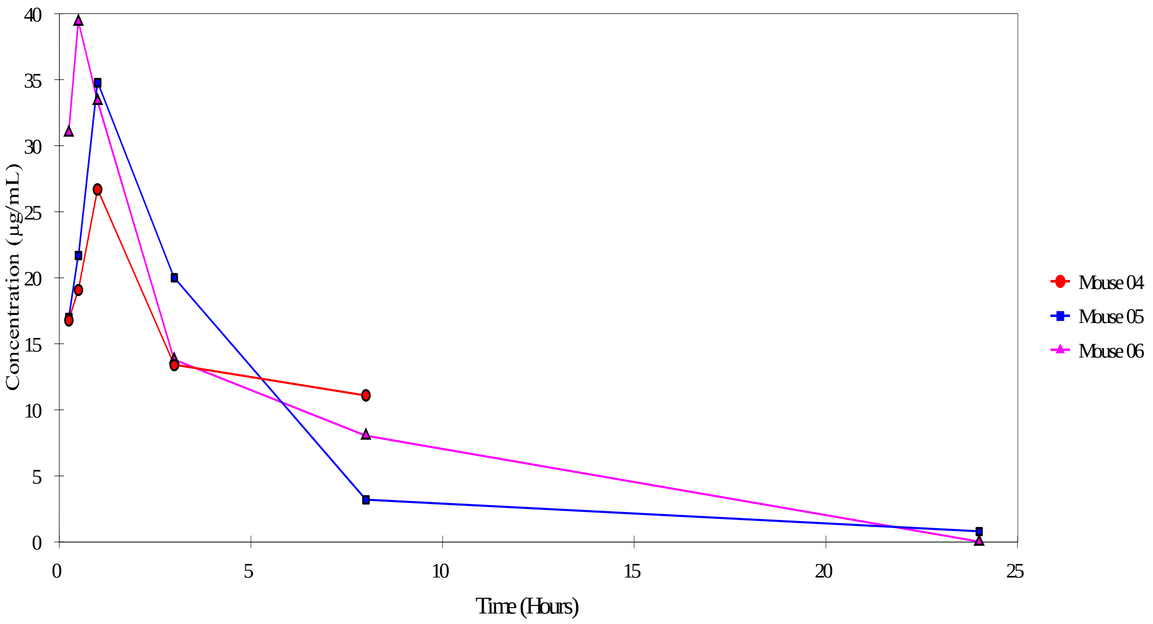

Figure 4: Day 5 Individual plasma concentration (µg/mL) of Fexinidazole Sulphoxide after 5-day repeated Oral administration of Fexinidazole to male NMRI Mice (Log-Lin scale)

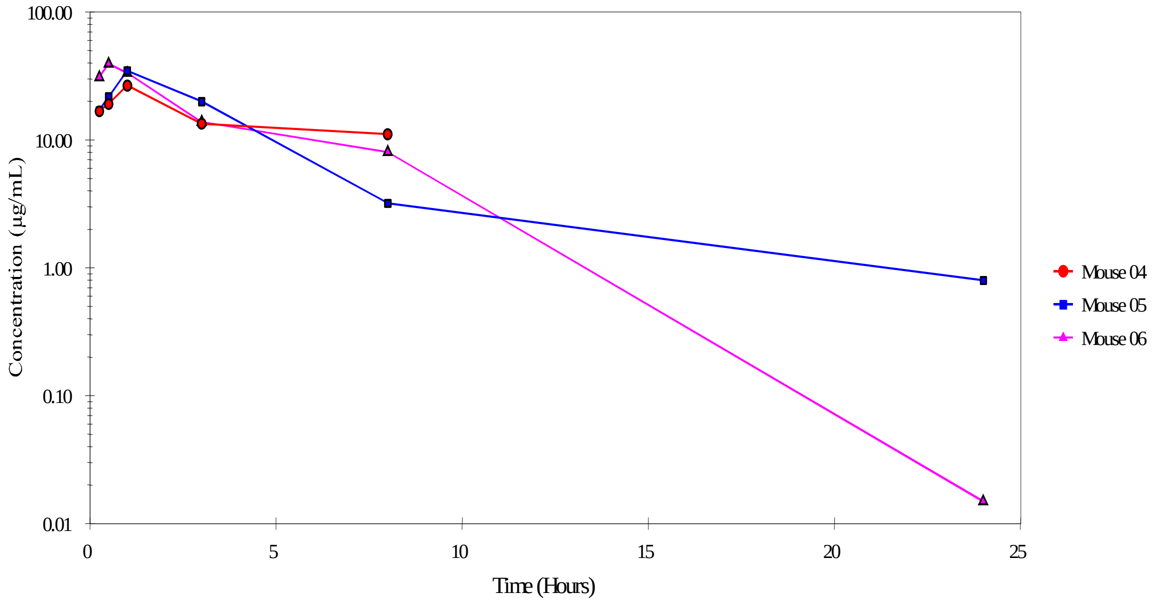

Figure 5. Mean (±SD) plasma concentrations (ng/mL) of Fexinidazole and its sulphone and sulphoxide metabolites after 5-day repeated oral administration of 200 mg/kg of Fexinidazole to male NMRI mice.

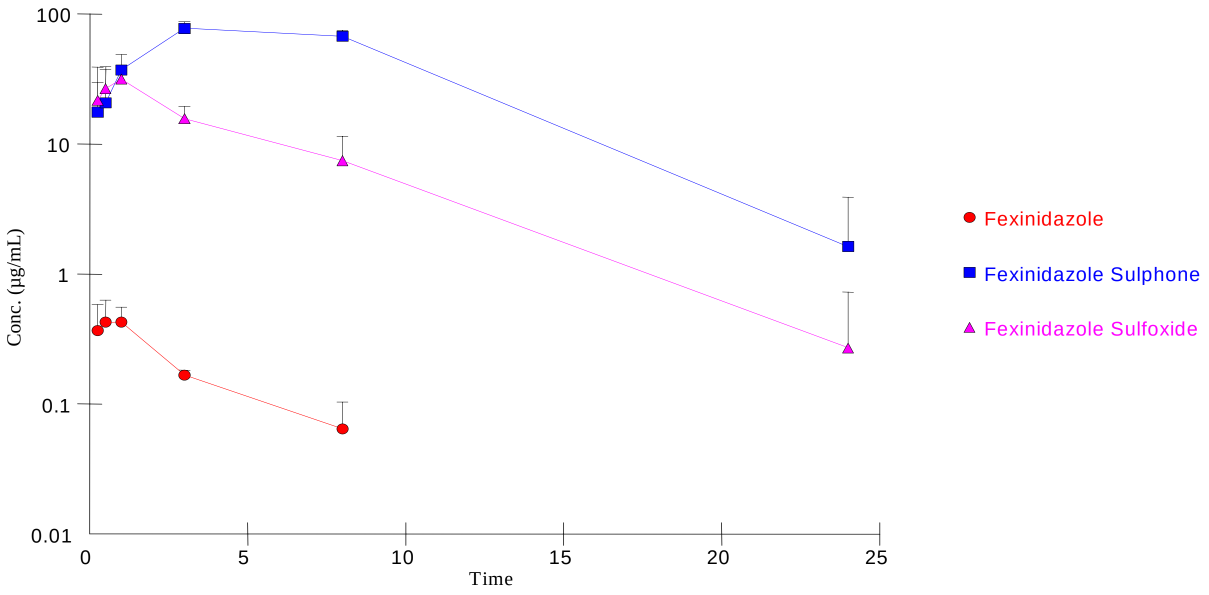

Supplement: Dataset S9 — (0.43 MB PDF) [file pntd.0000923.s010.pdf]
