# Supplementary material for: Fexinidazole – A New Oral Nitroimidazole Drug Candidate Entering Clinical Development for the Treatment of Sleeping Sickness
Source: PLoS Negl Trop Dis. 2010 Dec 21;4(12):e923. doi: 10.1371/journal.pntd.0000923 (PMC3006138; doi:10.1371/journal.pntd.0000923)

Fexinidazole: Determination of Tissue Distribution by Whole Body Autoradiography following Single Oral Administration of [^{14}C]-Fexinidazole to Rats.

Product Name :	FEXINIDAZOLE
Study Number:	0510-2007
Study Director:	
Sponsor Reference Study No.:	Not Applicable
Status:	Final

SUMMARY

Aim of this study was to obtain information on the tissue distribution of radioactive drug-related material following a single oral administration of [^{14}C]-FEXINIDAZOLE to male albino rats.

[^{14}C]-FEXINIDAZOLE was administered at the target dose level of 800 mg/kg (approximately 3.7 MBq/kg, 100 μCi/kg) to 3 male Sprague Dawley rats.

Two animals at each time were sacrificed 2, 8, 24 and 48 hours after administration and the radioactivity distribution in the organs and tissues was evaluated using the Whole Body Autoradioluminography method (WBA).

The obtained results showed that the radioactivity distributed to all the body and was found in all organs and tissues analyzed.

Peak concentrations of radioactivity were generally measured at 2 hours post-dosing; at this time the highest radioactivity levels were measured in the intestinal wall (4900 - 5600 μgeq/g) and in the Stomach, Liver, Adrenal glands, Kidney, Seminal vesicles, Prostate, Pancreas, Urinary bladder, Heart, Muscle, Spleen, Thyroid, Pituitary, Harderian's gland, Salivary glands, Lung and Blood (600 - 300 μgeq/g).

The radioactivity concentration decreased 8 hours after dosing; radioactivity levels were measured at this time in the Intestine wall, Adrenal glands, Liver, Kidney, Stomach and Pancreas (500 - 300 μgeq/g). Radioactivity was found 24 hours after dosing in the Urinary bladder, Intestinal wall and Kidney (300 - 100 μgeq/g).

Radioactivity was detected at all times in the Brain; peak concentrations of radioactivity were measured at 2 - 8 hours after dosing (150 –160 μgeq/g).

Levels of radioactivity < 30 μgeq/g were still measured after 48 hours in most of organs.

TABLE OF CONTENTS

SUMMARY 5
1. INTRODUCTION AND OBJECTIVES 8
2. STUDY SPONSOR 8
3. TEST FACILITY 9
4. REGULATORY REQUIREMENTS 9
5. ABBREVIATION AND DEFINITIONS OF TERMS 9
6. MATERIALS AND METHODS 10
6.1. Test Item 10
6.2. Chemicals 10
6.3. Instrumentation 10
6.4. Test System 11
6.4.1. Species, Specification and Supplier 11
6.4.2. Environment 11
6.4.3. Diet and Drinking Water 11
6.5. Experimental Procedures 11
6.5.1. Animal Observations 11
6.5.2. Body Weights 11
6.6. Preparation and Analysis of Dose Formulation 12
6.6.1. Dose Formulation 12
6.7. Animal Treatment 12
6.7.1. Administered Doses 12
6.8. Sample Collection and Analysis of Radioactivity 12
6.8.1. Animal preparation 12
6.8.2. Embedding 13
6.8.3. Sectioning 13
6.8.4. Whole Body Autoradioluminography 13
6.9. Data Acquisition and Processing 13
6.9.1. Quantification of Total Radioactivity by LSC 13
6.9.2. Radioactivity analysis in autoradioluminograms 13
6.9.3. Radioactivity Data Processing 14
6.10. Sample Storage 15

6.11. Data Presentation....15
7. PROTOCOL DEVIATIONS....15
8. ARCHIVING....15
9. RESULTS AND DISCUSSION....15
9.1. Analysis of Dose Formulation....15
9.2. Animal dosing....16
9.3. Study Observations....16
9.4. Distribution of Total Radioactivity....16
10. CONCLUSIONS....17
11. REFERENCES....18
12. CONTRIBUTORS....18
TABLES AND FIGURES
Table 1. Dosing data for the single oral administration of [^{14}C]-FEXINIDAZOLE to male albino Sprague Dawley rats....19
Table 2. Individual radioactivity concentrations (μgeq/g) in organs and tissues following a single oral administration of [^{14}C]-FEXINIDAZOLE to male albino rats....20
Table 3. Mean radioactivity concentrations (μgeq/g) in organs and tissues following a single oral administration of [^{14}C]-FEXINIDAZOLE to male albino rats....22
Table 4. Tissue to blood ratios (individual and mean values) of total radioactivity following a single oral administration of [^{14}C]-FEXINIDAZOLE to male albino rats....23

Figure 1. Distribution of total radioactivity at 2 hours following a single oral administration of [^{14}C]-FEXINIDAZOLE to male albino rats....25
Figure 2. Distribution of total radioactivity at 8 hours following a single oral administration of [^{14}C]-FEXINIDAZOLE to male albino rats....26
Figure 3. Distribution of total radioactivity at 24 hours following a single oral administration of [^{14}C]-FEXINIDAZOLE to male albino rats....27
Figure 4. Distribution of total radioactivity at 48 hours following a single oral administration of [^{14}C]-FEXINIDAZOLE to male albino rats....27
APPENDICES

Appendix 1. Study Protocol

Appendix 2. Analytical Bulletin

Appendix 3. Individual Raw Data (Autoradioluminograms)

1. INTRODUCTION AND OBJECTIVES

This study was conducted according to Protocol N° 0510-2007. A copy of the experimental protocol is included in Appendix 1.

FEXINIDAZOLE is a 5-nitroimidazole derivative biologically active against Trypanosoma parasites (*T.b. rhodesiense* and *T.b. brucei*) and useful in the treatment of the Human African Trypanosomiasis (HAT), known as sleeping sickness. FEXINIDAZOLE is a compound currently under development by the Study Sponsor.

The objective of this study was to obtain information on the tissue distribution of radioactive drug-related material following oral administration of [^{14}C]-FEXINIDAZOLE (800 mg/kg) to male Sprague Dawley rats.

The albino Sprague Dawley rat was chosen as the species for this study since it was one of the species used in the toxicological evaluation of the test compound. Only male animals were used as no gender differences were expected with respect to the aim of the study. The oral route of administration was chosen as this is the intended therapeutic route. The dose level was selected in agreement with the Study Sponsor and it is within the pharmacological relevant range. No overt toxicity was expected after single dosing at this dose level.

The study was conducted on 8 male animals after single oral administration of [^{14}C]-FEXINIDAZOLE (800 mg/kg, approximately 3.7 MBq/kg, 100 µCi/kg). The animals were sacrificed at 2, 8,24 and 48 hours after administration and each rat was analyzed by a validated Whole Body Autoradioluminography (WBA) method [1], in order to evaluate the quantitative tissue distribution of radioactivity.

A summary of the experimental design is reported below:

Animal no.	Date of Dosing	Administered Dose (oral administration)	Samples collected and analyzed
M1, M2	June 10, 2008	800 mg/kg<br>100 µCi/kg<br>10 mL/kg	Whole body sections
M3, M4			
M5, M6			
M7, M8			

The experimental phase of the study started on June 10, 2008 and was completed on July 25, 2008 (WBA analysis).

2. STUDY SPONSOR

DND*i* – Drugs for Neglected Diseases *Initiative*

3. TEST FACILITY

Accelera S.r.l.

4. REGULATORY REQUIREMENTS

This study was conducted in compliance with the *DECRETO LEGISLATIVO 2 Marzo 2007, No. 50* and with the *Organisation for Economic Co-operation and Development (OECD) Principles of Good Laboratory Practice (GLP) (as revised in 1997).*

This study was conducted according to the methods described in the "Standard Operating Procedures" of the laboratories involved.

5. ABBREVIATION AND DEFINITIONS OF TERMS

BLQ	Below limit of quantification
Bq	Becquerel
C_{max}	Maximal concentration of total radioactivity
CMC	Carboxymethylcellulose
CO_2	Carbon dioxide
Ct	Concentration of total radioactivity at any time
dpm	Disintegration per minute
GI	Gastrointestinal
h	hour
IP	Imaging Plate
LOD	Limit of detection
LOQ	Limit of quantification
LSC	Liquid Scintillation Counting
µgeq/g	microgram equivalent/gram
NA	Not Applicable
Ci	Curie
NC	Not Calculable
ND	Not Detectable
NI	Not Identifiable
PSL	Photo-Stimulated Luminescence
ROI	Region Of Interest
SA	Specific Activity
SD	Standard Deviation of the mean
t_{last}	Time of the last detectable radioactivity concentration
WBA	Whole-Body Autoradioluminography

6. MATERIALS AND METHODS

6.1. Test Item

The test material was prepared in Accelera, mixing appropriate amounts of [^{14}C]-FEXINIDAZOLE (batch No F0129/6, Specific Activity: 57.7 mCi/mmol, radiochemical purity >98%, prepared in Accelera and stored at –20 °C in the dark) and unlabelled FEXINIDAZOLE (Centipharm batch No. 3168-07-01/O, purity 100.2%, expired date October 2008, provided by the Study Sponsor and stored at room temperature, in the dark), in order to obtain [^{14}C]-FEXINIDAZOLE at the final Specific Activity of 4.625 KBq/mg (0.125 μCi/mg).

The analytical bulletins are included in Appendix 3.

6.2. Chemicals

Methylcellulose (400 cP), used for test item formulation, was obtained from Sigma-Aldrich

Tween 80, used for test item formulation, was obtained from Sigma-Aldrich

Water for injection, used for test item formulation, was obtained from Bieffe Medital S.p.A.

Ultima Gold, used as liquid scintillation cocktail, were also obtained from Perkin-Elmer Life Science.

Carboxymethylcellulose was obtained from Sigma.

[^{14}C]-Methyl methacrylate standards were obtained from ARC, American Radiolabeled Chemicals, Inc.

Reagents and solvents (hexane, diethylether, methanol) were of analytical grade (or equivalent), obtained from Carlo Erba Reagents.

6.3. Instrumentation

Balances, mod. AG204, AT201, PB 5001, Mettler.

Liquid scintillation analyzer, mod.1900 TR and 1900 CA, Packard.

Centrifuge Megafuge 1.0R, Heraeus

CryoMicrotome Bright 9400, Bright Instruments,UK.

Phosphor Imaging System (BAS1500 Bio Imaging Analyser, IP Eraser, BAS-IIISR phosphor imaging plates 20x40 cm, BAS 2040 exposition cassettes) was obtained from Fujifilm Imaging & Information, Japan and was connected with a SCSI 2030 to the computer (INTEL Pentium IV, 3 GHz, 74.5 GB, 502 MB RAM) for image data acquisition and processing.

6.4. Test System

6.4.1. Species, Specification and Supplier

Sprague Dawley rats (8 males, adults, age 8 weeks, body weight 280-291 g at the time of dosing) were used. These animals were supplied by Charles River, Calco (CO) Italy. All animals were visually inspected for signs of illness and were deemed fit for use in the study.

6.4.2. Environment

During pretrial holding period the rats were housed in polypropylene and stainless steel cages with wood shavings as bedding. During the experimental period the rats were individually housed in polypropylene and stainless steel cages with raised wire mesh floors.

Holding and study areas had automatic control of light cycle and temperature. The lighting in the study unit was controlled in a 12-hour light-dark cycle throughout the study. Temperature and relative humidity measured during the study were in the range 22.2-23.3 C° and 59-68 %, respectively. The values of temperature recorded during the experimental period, although exceeded the standard range, were considered not relevant and not affecting the experimental results.

6.4.3. Diet and Drinking Water

A complete dry diet (Mucedola 4RF21) was available *ad libitum*. Domestic mains quality water was available *ad libitum* throughout the study. Certificates of analysis from the manufacturer of the diet batches were obtained and were included in the raw data. The water was periodically analyzed for chemical and microbial impurities and the certificates of analysis supplied from the local water authority. There were no contaminants in the diet or water that were considered to have potentially affected the integrity or outcome of the study.

The animals were fasted overnight before administration; food was allowed to the animals from approximately 2 hours after dosing.

6.5. Experimental Procedures

6.5.1. Animal Observations

The animals were observed during the treatment and routinely during the whole course of the experiment to evaluate any evidence of reaction to treatment, change in general appearance, overt signs of suffering or evidence of toxicity.

6.5.2. Body Weights

Animals were weighed before treatment on the morning of dosing. Individual animal weights are reported in Table 1.

6.6. Preparation and Analysis of Dose Formulation

6.6.1. Dose Formulation

The test formulation was prepared on the day of dosing by suspending unlabelled FEXINIDAZOLE and [^{14}C]-FEXINIDAZOLE (see section 6.1) in 5% Tween 80 and 0.5% Methyl cellulose 400 cP (Methocel) in water, in order to obtain [^{14}C]-FEXINIDAZOLE at the final target concentration of 80 mg/mL, corresponding to a radioactivity concentration of about 370 kBq/mL (10 μCi/mL).

The test formulation was prepared by suspending an appropriate amount of unlabelled FEXINIDAZOLE in the dose vehicle. This formulation was homogenised using a Potter-Elvehjem homogenizer apparatus (teflon pestle of approx. 30 mm x 53 mm and glass tube of 40 mL), in order to obtain a fine suspension by up-and-down strokes, gently made by hand. The formulation was then collected and transferred from the homogeniser glass tube to the glass pot containing the [^{14}C]-FEXINIDAZOLE.

During the preparation the test formulation was protected from light as far as possible.

The radioactivity concentration and homogeneity of the test formulation were determined, before administration, by liquid scintillation counting of triplicate weighed aliquots of test formulation. The radiolabelled test formulation was used immediately after formulation.

6.7. Animal Treatment

6.7.1. Administered Doses

The dose of [^{14}C]-FEXINIDAZOLE was orally administered by gastric gavage at the target dose level of 800 mg/kg (radioactivity dose of about 3.7 MBq/kg, 100 μCi/kg) and at a target dose volume of about 10 mL/kg.

Individual doses were prepared for each animal by weighing the appropriate amount of the test formulation in an appropriate syringe for oral administration. The amount of dose administered to each animal was determined by weighing the filled syringe before the treatment and the empty syringe after dose administration. The actual dose received by each animal was calculated using the weight of the administered formulation, the radioactivity concentration and the final specific activity of the test material.

The actual doses received by each animal are documented in Table 1.

6.8. Sample Collection and Analysis of Radioactivity

6.8.1. Animal preparation

Two animals for each time were deeply anaesthetized by diethylether inhalation at 2, 8, 24 and 48 hours after dosing; after sacrifice each animal was immediately frozen in a mixture of hexane and dry ice at about -70 °C. The frozen carcasses were then stored at –20 °C until embedding.

6.8.2. Embedding

Frozen carcasses were embedded in aqueous carboxymethylcellulose gel (CMC, 2 % w/v) using suitable stainless steel box. Before embedding the hair was partially shaved and ears, paws and tail were removed from the carcass. The embedded carcasses were frozen in hexane/dry-ice bath (-70 °C) and stored in freezer at –20 °C until sectioning.

6.8.3. Sectioning

Each frozen cellulose block, containing the carcass of the animal, was mounted as appropriate into a Bright 9400 cryomicrotome. Sagittal sections of 30 μm thickness were collected at different levels through each carcass of animal, to ensure an adequate sampling of all organs and tissues. Sections were collected on an appropriate tape, mounted on wooden frames and dried in the cryochamber at –20 °C for about 48 hours, to ensure dehydration of the sections. Dried sections were then treated with talcum powder.

6.8.4. Whole Body Autoradioluminography

Selected sections were mounted on sheets of paper, labelled and placed in Fuji BAS 2040 exposure cassettes along with calibrated ^{14}C-standard sets (ARC). Sections were then exposed to a phosphor imaging plate (IP BAS IIISR Fuji) for 72 hours. In order to minimize the background radiation, exposure was carried out in a shielding box at room temperature. The remaining collected sections, although retained, were not analyzed.

After exposure the phosphor imaging plates were removed from the cassettes in a darkroom and placed in the Fuji BAS1500 Bio-Imaging Analyzer.

The measured photo-stimulated luminescence (PSL) corresponded to the intensity and to the distribution of the radioactivity in the original sections and was recorded as electronic image. Each electronic image (autoradioluminogram) was stored as a unique electronic file (reading by AIDA as file extension *pcb).

6.9. Data Acquisition and Processing

6.9.1. Quantification of Total Radioactivity by LSC

Radioactivity in test formulation was measured by LSC using Packard liquid scintillation analyzers. Samples were counted up to 1 hour (with the 2 sigma% settled at 0.30 region A and 0.50 region B).

Counting efficiencies were calculated by the external standard method using a series of quenched standards supplied by Packard, in order to generate the calibration curves. The validity of calibration curves was checked before analysis.

6.9.2. Radioactivity analysis in autoradioluminograms

Quantitative evaluation of the autoradioluminograms was performed using the AIDA (Advanced Image Data Analyser) applicative software, version 2.43, RAYTEST GmbH, Germany. The signal, recorded as PSL units, was evaluated in the area corresponding to

region of interest (ROI) and the image density was expressed as PSL/mm^2. Only tissues and/or organs identifiable by visual observation of the latent image were identified as ROIs. Tissues and organs identifiable by visual observation in the original animal sections but not in the corresponding autoradioluminograms due to the very low radioactivity distribution were reported as not detectable (ND in the final tables).

The evaluation of radioactivity in organs and tissues was performed in the ROI corresponding to the whole organ. The distribution of radioactivity in skeletal muscle was measured at the level of muscles of the hind limbs, of the shoulders and of the brachial musculature. The distribution of radioactivity in bone and bone marrow was measured in the femur, ilium and homerus. The distribution of radioactivity in the lymph nodes was measured in the submaxillary region. Radioactivity in blood was measured in the large vessels (vena cava, aorta) and in the cardiac cavity. The distribution of radioactivity in the intestinal wall was measured along the whole intestinal tract without distinction between large and small intestine. Radioactivity distribution was evaluated in the whole eyeball. When possible, the radioactivity distribution in the skin was evaluated in the cutis of the dorsal area. Due to the heterogeneity of the radioactivity concentration in the gastrointestinal tract, the radioactivity levels in gut content were not measured.

The image densities measured in the ^{14}C-standards and the corresponding precalibrated values (nCi/g tissue) were subsequently used to establish calibration curves and convert the image densities, determined in tissues and organs, to radioactivity concentration (µCi/g tissue equivalent) and to µgequivalent/g tissue (µgeq/g) using in this case the appropriate Specific Activity.

The limit of quantification (LOQ) was defined as ten times the standard deviation of background values measured in each analysis [2]. In this study the LOQ accounted for about 0.08 – 0.29 PSL/mm^2. The ROIs with image densities below the LOQ were excluded from the following elaborations.

The autoradiograms were stored as electronic files with extension *ADF after the elaboration and the quantification.

6.9.3. Radioactivity Data Processing

The mean values of radioactivity concentrations in tissues (measured by WBA) were calculated using Excel. Mean values of radioactivity were calculated averaging the tissue concentrations measured in the different slices from replicated analyses obtained from the autoradioluminograms of the same subject. The number of replicate analyses is reported in the relevant tables.

The weights of samples and the radioactivity disintegration rates were directly captured from the output of analytical balances and liquid scintillation counters respectively or manually introduced into a validated Laboratory Information Management System (DEBRA v.5.4, by LabLogic, UK) to be processed for determination of the administered doses to the animals.

6.10. Sample Storage

After preparation the carcasses were stored at ca. –20 ^{o} C. After dehydration the original sections were stored in appropriate folder, protected by an acetate film, until analysis.

6.11. Data Presentation

The radioactivity concentrations in tissues, expressed as μgeq/g, are presented to one decimal place and are quoted to three significant figures. Concentration values below the limit of quantification are denoted as BLQ (below the limit of quantification) and are taken as zero for all subsequent calculations. Values reported as ND (not detectable) are taken as zero for all subsequent calculations. Data presented in Tables are computer generated and appropriately rounded for inclusion in the report. As a consequence, in some instances, calculation of values from data presented may yield minor variations.

7. PROTOCOL DEVIATIONS

Due to technical reasons the animals received a dose of approximately 700 mg/kg instead of 800 mg/kg (about 13 % less than the theoretical dose). Considering the aim of the study this deviation does not affect the results of the study.

No other deviations from the Protocol occurred during this study.

8. ARCHIVING

All raw data, supporting documents produced at the Test Facility, a copy of the documentation of the test article received by the Sponsor, the protocol and the final report as originals were filed in the Archives of Accelera S.r.l., Nerviano, Italy for a period of 3 years, after which the Sponsor will be contacted for instructions regarding dispatch or disposal of the material. Specimens requiring storage deep frozen and the original animal sections are specifically excluded from the above. These will be retained for as long as the quality of the material permits evaluation but for no longer than 6 months after issue of the final report.
The study Sponsor will be notified before specimens are destroyed on their behalf. The copy of the protocol with original signatures and the copy of study report with original signatures were delivered to the Sponsor.

9. RESULTS AND DISCUSSION

9.1. Analysis of Dose Formulation

The radioactivity concentration of [^{14}C]-FEXINIDAZOLE measured in the test formulation used for the administrations was 394.05 kBq/g (10.65 μCi/g) corresponding to a compound concentration of 85.22 mg/g of formulation.

9.2. Animal dosing

The doses received by the animals were in the range of 683.07 - 715.73 mg/kg. The actual doses of radioactive test compound administered to the animals are detailed in Table 1.

9.3. Study Observations

No overt adverse signs were observed in the test animals during the conduct of the study.

9.4. Distribution of Total Radioactivity

In compliance with the aim of the study, the WBA method was used to evaluate the quantitative distribution of total radioactivity in the organs and tissues and therefore no characterization and quantification of radioactive parent and metabolite compounds was possible.

The tissue distribution profiles of total radioactivity in organs and tissues following a single oral administration of [^{14}C]-FEXINIDAZOLE to male albino rats at the dose level of 800 mg/kg, expressed as tissue concentrations of total radioactivity (µgeq/g, individual and mean values) are reported in Table 2 and in Table 3.

The tissue to blood ratios of total radioactivity are reported in Table 4.

After oral dosing the radioactivity distributed to all the body and was found in all organs and tissues analyzed. Peak concentrations of radioactivity were generally measured at 2 hours post-dosing in most of organs and tissues; only in eyes the peak concentration of radioactivity was measured at 8 hours.

The highest levels of radioactivity were measured at 2 hours after dosing in the intestinal wall (4900 - 5600 µgeq/g); mean levels of radioactivity between about 600 and 300 µgeq/g were measured in the Stomach, Liver, Adrenal glands, Kidney, Seminal vesicles, Prostate, Pancreas, Urinary bladder, Heart, Muscle, Spleen, Thyroid, Pituitary, Harderian's gland, Salivary glands, Lung and Blood. Mean levels of radioactivity between 300 and 200 µgeq/g were measured in the Bone marrow, Lymph nodes, Brown fat, Skin and Thymus. The mean radioactivity levels were between 200 and 100 µgeq/g in Testis, Brain and Spinal cord.

The highest radioactivity levels were found at 8 hours after dosing in the Intestinal wall, Adrenal glands, Liver, Kidney, Stomach and Pancreas (500 - 300 µgeq/g, as mean). Mean radioactivity between 300 and 100 µgeq/g was measured in the Heart, Harderian's glands, Seminal vesicles, Urinary bladder, Blood, Prostate, Thyroid, Brown fat, Lung, Salivary glands, Pituitary, Lymph nodes, Muscle, Skin, Spleen, Bone marrow, Thymus, Testis, Brain, Spinal cord and Eyes.

The radioactivity is still detected at 24 hours in all organs and tissues; the highest levels of radioactivity were measured in the Urinary bladder, Intestinal wall and Kidney (300 -

100 µgeq/g, as mean). The radioactivity levels were < 100 µgeq/g at this time in the other organs and tissues.

The radioactivity levels were < 30 µgeq/g after 48 hours in all organs. At this time the radioactivity of some present organs in the original animal sections was not distinguishable in the autoradioluminograms from the radioactivity of the whole body and therefore it was reported as NI in the tables.

Radioactivity was detected at all times in the Brain; peak concentrations of radioactivity were measured at 2 - 8 hours after dosing (150 –160 µgeq/g). Low levels of radioactivity were still measured at 48 hours in the Brain (about 6 µgeq/g). The Brain to Blood ratio of radioactivity concentration ratio was 0.4 – 0.6 at all times.

Due to the low homogeneity of radioactivity distribution in the gastrointestinal contents (GI) and in faeces, the radioactivity was not measured in both the GI contents and faeces. Moreover some regions of gastrointestinal content appeared overexposed with saturation of signal at 2 hours after dosing.

Some images of the autoradioluminograms are reported in Figures 1 - 4. All the original autoradioluminograms obtained in the course of the study are reported in Appendix 3.

10. CONCLUSIONS

After a single oral administration of [^{14}C]-FEXINIDAZOLE to male albino rats at the dose level of 800 mg/kg the radioactivity distributed to all the body and was found in all organs and tissues analyzed.

Peak concentrations of radioactivity were generally measured at 2 hours post-dosing; at this time the highest radioactivity levels were measured in the intestinal wall (4900 - 5600 µgeq/g) and in the Stomach, Liver, Adrenal glands, Kidney, Seminal vesicles, Prostate, Pancreas, Urinary bladder, Heart, Muscle, Spleen, Thyroid, Pituitary, Harderian’s gland, Salivary glands, Lung and Blood (600 - 300 µgeq/g).

The radioactivity concentration decreased 8 hours after dosing; radioactivity levels were measured at this time in the Intestine wall, Adrenal glands, Liver, Kidney, Stomach and Pancreas (500 - 300 µgeq/g). Radioactivity was found 24 hours after dosing in the Urinary bladder, Intestinal wall and Kidney (300 - 100 µgeq/g).

Radioactivity was detected at all times in the Brain; peak concentrations of radioactivity were measured at 2 - 8 hours after dosing (150 –160 µgeq/g).

Levels of radioactivity < 30 µgeq/g were still measured after 48 hours in most of organs.

11. REFERENCES

1. Validation of the Fuji BAS-1500 phosphor imaging system for use in quantitative whole body autoradioluminography. Document b0035455, April 2001.

2. J. Maas, R. Binder, W. Steinke (2000). Quantitative Whole-body Autoradiography: Recommendations for the Standardization of the Method. Regulatory Tox. and Pharm. 31, S15-S21.

12. CONTRIBUTORS

TABLES AND FIGURES

Table 1. Dosing data for the single oral administration of [^{14}C]-FEXINIDAZOLE to male albino Sprague Dawley rats.

Animal number	Animal Weight (g)	Specific Activity µCi/mg	Dose received µCi	mg	mg/kg
M1	285	0.125	25.38	203.06	712.50
M2	282		24.63	197.01	698.63
M3	291		25.61	204.85	703.93
M4	288		25.53	204.25	709.20
M5	285		24.88	199.02	698.33
M6	281		24.58	196.66	699.84
M7	287		24.51	196.04	683.07
M8	280		25.05	200.41	715.73

Table 2. Individual radioactivity concentrations (μgeq/g) in organs and tissues following a single oral administration of [^{14}C]-FEXINIDAZOLE to male albino rats.

	2h						8h						24h						48h					
	M1			M2			M3			M4			M5			M6			M7			M8		
**Sample**	**MEAN**	**S.D.**	**n**	**MEAN**	**S.D.**	**n**	**MEAN**	**S.D.**	**n**	**MEAN**	**S.D.**	**n**	**MEAN**	**S.D.**	**n**	**MEAN**	**S.D.**	**n**	**MEAN**	**S.D.**	**n**	**MEAN**	**S.D.**	**n**
Adrenal gland	565.2	NA	2	514.7	81	3	476.5	37.7	3	453.8	47.6	4	108.8	30.6	3	56.8	NA	2	31.2	NA	2	16.5	3.2	3
Blood	306	43.2	11	300.3	48	14	240.7	42.8	12	305.3	14.2	6	61.8	9.6	5	40.8	5.5	6	15.2	2.8	4	13.3	2.6	3
Bone	38.1	22.4	5	37.5	10.9	6	22.4	9.7	3	24.6	8.1	7	22.6	6.8	4	11.2	7.1	4	NI			NI		
Bone marrow	309.8	95.9	5	268.2	37.6	5	206.4	17	4	216.5	22.2	5	49.8	8.2	4	100.8	55.6	3	20	6.5	3	9.6	0	3
Brain	169.3	19.9	5	150.1	16.2	6	151.6	14.9	6	145.4	7	4	25.1	3.1	5	19.6	2.2	6	5.6	1.5	4	5.9	0.9	3
Brown fat	295.2	18.9	3	257.3	27	3	235.5	27.6	3	267.7	33.8	3	78.4	14	4	54.9	4.6	3	20.3	3.2	3	27.2	5.6	3
Eye	69.3	25.6	4	83.2	26.8	3	100	31.2	3	121.6	NA	2	66.4	NA	2	78.8	NA	2	27.2	5.7	2	32.8	NA	1
Harderian's gl.	281.6	60.4	3	337.8	26	4	292.8	47.5	4	294.4	NA	2	73	9.4	4	41.8	13.4	4	16.5	8.6	3	25.3	9.6	3
Heart	340.8	81	6	343.2	31.4	7	292.8	21.5	5	301	25.9	5	73.9	10.2	6	50.6	3.9	4	15.7	2.5	5	19.6	4.7	4
Intestine wall	4891	2335	8	5672	2041	11	512.5	263.1	6	498.6	183.3	5	90.2	7.2	4	117.1	53.6	3	NI			NI		
Kidney	473.2	71.4	6	443.4	42.5	7	408.3	17.7	5	432.3	50.9	5	120.7	16.7	6	84.8	11.5	3	29.7	5.2	6	28.1	5.7	8
Liver	574.6	81.3	10	534.6	60.1	11	414.2	25.7	7	463.8	47.4	9	116.8	14.4	9	72.3	6.3	9	23.2	4.2	9	20.2	4.3	7
Lung	296	66.7	5	311.3	57.4	6	260.5	13.7	5	238.9	16.6	3	61.6	8.7	4	41.1	5.4	3	17.1	3.9	3	9.3	2.4	3
Lymph node	279.4	27.4	4	283.5	50.4	6	237	10.5	4	230.6	45.5	5	51.6	6.6	4	39.4	7.2	4	NI			NI		
Muscle	346.9	79.4	5	315.0	19.9	4	226.6	10.5	4	239.8	18.8	5	54.4	3.7	3	44.8	4.9	3	20.8	3.5	3	12.2	1.8	4

NI: not identifiable
NA: not applicable

n: number of replications

Table 2 (continued)

	2h						8h						24h						48h					
	M1			M2			M3			M4			M5			M6			M7			M8		
**Sample**	**MEAN**	**S.D.**	**n**	**MEAN**	**S.D.**	**n**	**MEAN**	**S.D.**	**n**	**MEAN**	**S.D.**	**n**	**MEAN**	**S.D.**	**n**	**MEAN**	**S.D.**	**n**	**MEAN**	**S.D.**	**n**	**MEAN**	**S.D.**	**n**
Pancreas	422.1	80.5	3	370.5	49	6	309.6	14	4	302.1	32.2	3	60.3	12.9	3	51.4	5.7	4	12.6	1.4	4	11	3.1	4
Pituitary	312.4	NA	2	318.0	NA	2	244	24.9	3	242	NA	2	53.3	14.8	3	27.7	3.3	3	NI			NI		
Prostate	436.5	76.8	3	371.2	60.9	3	295.2	10.4	3	241.6	NA	2	65.6	7.7	3	35.8	1.8	4	NI			NI		
Salivary glands	303.7	16.7	6	314	37.2	6	254.4	6.8	3	237.3	15.3	5	61.1	9.5	5	36.2	3.2	4	11	1.2	4	11.7	0.5	3
Seminal vesicles	445.9	52.9	3	374.9	26.6	3	293.2	NA	2	291	11.5	4	65.3	5.3	3	40.8	NA	2	NI			NI		
Skin	298.5	71.5	9	239.2	55.7	8	176.9	16	6	279.8	52.8	7	67.7	5.7	3	48.2	16.8	4	20.6	4.4	5	13.1	8	5
Spinal cord	138.1	16.9	3	126.9	14.8	3	142.7	23.9	3	150.8	9.4	4	28.5	3.8	3	17.1	0.9	3	6.4	2.8	3	7.5	2.6	3
Spleen	327.2	63.6	4	319.7	40.1	5	226.4	13.5	4	224.3	16.9	5	55.2	9.7	3	56.8	18.9	5	17.4	4.8	5	13.4	2.6	5
Stomach	826.1	426.4	5	413.3	57.8	3	296.5	67.2	5	316.8	73.1	4	108.2	13.9	4	49.2	NA	2	NI			NI		
Testis	167.7	35.9	9	175.3	14.9	9	139.3	14.9	8	158.1	16.4	6	36.7	3.7	6	25.6	2.5	7	8.8	2.4	4	9.2	2.7	4
Thymus	270.6	26.6	5	255.2	24.1	6	208.3	16.1	5	194.1	4.4	3	50	6.7	4	33.8	3.5	4	9.6	2.8	4	13	1.4	4
Thyroid	324.8	NA	2	309.6	NA	2	236.8	NA	2	290.8	NA	2	NI			NI			NI			NI		
Urinary bladder	411.6	NA	2	333.6	NA	2	212	NA	2	356	NA	1	366.4	NA	1	263.2	NA	1	NI			NI		

NI: not identifiable
NA: not applicable
n: number of replications

Table 3. Mean radioactivity concentrations (µgeq/g) in organs and tissues following a single oral administration of [^{14}C]-FEXINIDAZOLE to male albino rats.

Sample	2h	8h	24h	48h
Adrenal glands	539.9	465.2	82.8	23.9
Blood	303.2	273.0	51.3	14.3
Bone	37.8	23.5	16.9	NI
Bone marrow	289.0	211.4	75.3	14.8
Brain	159.7	148.5	22.4	5.7
Brown fat	276.3	251.6	66.7	23.7
Eyes	76.3	110.8	72.6	30.0
Harderian's glands	309.7	293.6	57.4	20.9
Heart	342.0	296.9	62.2	17.6
Intestine wall	5281.4	505.5	103.6	NI
Kidney	458.3	420.3	102.7	28.9
Liver	554.6	439.0	94.5	21.7
Lung	303.7	249.7	51.3	13.2
Lymph nodes	281.4	233.8	45.5	NI
Muscle	330.9	233.2	49.6	16.5
Pancreas	396.3	305.9	55.8	11.8
Pituitary	315.2	243.0	40.5	NI
Prostate	403.9	268.4	50.7	NI
Salivary glands	308.9	245.8	48.7	11.4
Seminal vesicles	410.4	292.1	53.1	NI
Skin	268.8	228.4	58.0	16.9
Spinal cord	132.5	146.7	22.8	6.9
Spleen	323.4	225.4	56.0	15.4
Stomach	619.7	306.6	78.7	NI
Testis	171.5	148.7	31.1	9.0
Thymus	262.9	201.2	41.9	11.3
Thyroid	317.2	263.8	NI	NI
Urinary bladder	372.6	284.0	314.8	NI

NI: not identifiable

Table 4. Tissue to blood ratios (individual and mean values) of total radioactivity following a single oral administration of [^{14}C]-FEXINIDAZOLE to male albino rats.

	2h			8h			24h			48h		
**Sample**	**M1**	**M2**	**Mean**	**M3**	**M4**	**Mean**	**M5**	**M6**	**Mean**	**M7**	**M8**	**Mean**
Adrenal gland	1.8	1.7	1.8	2.0	1.5	1.7	1.8	1.4	1.6	2.1	1.2	1.6
Bone	0.1	0.1	0.1	0.1	0.1	0.1	0.4	0.3	0.3	NC	NC	NC
Bone marrow	1.0	0.9	1.0	0.9	0.7	0.8	0.8	2.5	1.6	1.3	0.7	1.0
Brain	0.6	0.5	0.5	0.6	0.5	0.6	0.4	0.5	0.4	0.4	0.4	0.4
Brown fat	1.0	0.9	0.9	1.0	0.9	0.9	1.3	1.3	1.3	1.3	2.0	1.7
Eye	0.2	0.3	0.3	0.4	0.4	0.4	1.1	1.9	1.5	1.8	2.5	2.1
Harderian gland	0.9	1.1	1.0	1.2	1.0	1.1	1.2	1.0	1.1	1.1	1.9	1.5
Heart	1.1	1.1	1.1	1.2	1.0	1.1	1.2	1.2	1.2	1.0	1.5	1.3
Intestine wall	16.0	18.9	17.4	2.1	1.6	1.9	1.5	2.9	2.2	NC	NC	NC
Kidney	1.5	1.5	1.5	1.7	1.4	1.6	2.0	2.1	2.0	2.0	2.1	2.0
Liver	1.9	1.8	1.8	1.7	1.5	1.6	1.9	1.8	1.8	1.5	1.5	1.5
Lung	1.0	1.0	1.0	1.1	0.8	0.9	1.0	1.0	1.0	1.1	0.7	0.9
Lymph node	0.9	0.9	0.9	1.0	0.8	0.9	0.8	1.0	0.9	NC	NC	NC
Muscle	1.1	1.0	1.1	0.9	0.8	0.9	0.9	1.1	1.0	1.4	0.9	1.1

NC: not calculable

Table 4 (continued)

	2h			8h			24h			48h		
**Sample**	**M1**	**M2**	**Mean**	**M3**	**M4**	**Mean**	**M5**	**M6**	**Mean**	**M7**	**M8**	**Mean**
Pancreas	1.4	1.2	1.3	1.3	1.0	1.1	1.0	1.3	1.1	0.8	0.8	0.8
Pituitary	1.0	1.1	1.0	1.0	0.8	0.9	0.9	0.7	0.8	NC	NC	NC
Prostate	1.4	1.2	1.3	1.2	0.8	1.0	1.1	0.9	1.0	NC	NC	NC
Salivary glands	1.0	1.0	1.0	1.1	0.8	0.9	1.0	0.9	0.9	0.7	0.9	0.8
Seminal vesicles	1.5	1.2	1.4	1.2	1.0	1.1	1.1	1.0	1.0	NC	NC	NC
Skin	1.0	0.8	0.9	0.7	0.9	0.8	1.1	1.2	1.1	1.4	1.0	1.2
Spinal cord	0.5	0.4	0.4	0.6	0.5	0.5	0.5	0.4	0.4	0.4	0.6	0.5
Spleen	1.1	1.1	1.1	0.9	0.7	0.8	0.9	1.4	1.1	1.1	1.0	1.1
Stomach	2.7	1.4	2.0	1.2	1.0	1.1	1.8	1.2	1.5	NC	NC	NC
Testis	0.5	0.6	0.6	0.6	0.5	0.5	0.6	0.6	0.6	0.6	0.7	0.6
Thymus	0.9	0.8	0.9	0.9	0.6	0.8	0.8	0.8	0.8	0.6	1.0	0.8
Thyroid	1.1	1.0	1.0	1.0	1.0	1.0	NC	NC	NC	NC	NC	NC
Urinary bladder	1.3	1.1	1.2	0.9	1.2	1.0	5.9	6.5	6.2	NC	NC	NC

NC: not calculable

Figure 1. Distribution of total radioactivity at 2 hours following a single oral administration of [^{14}C]-FEXINIDAZOLE to male albino rats.

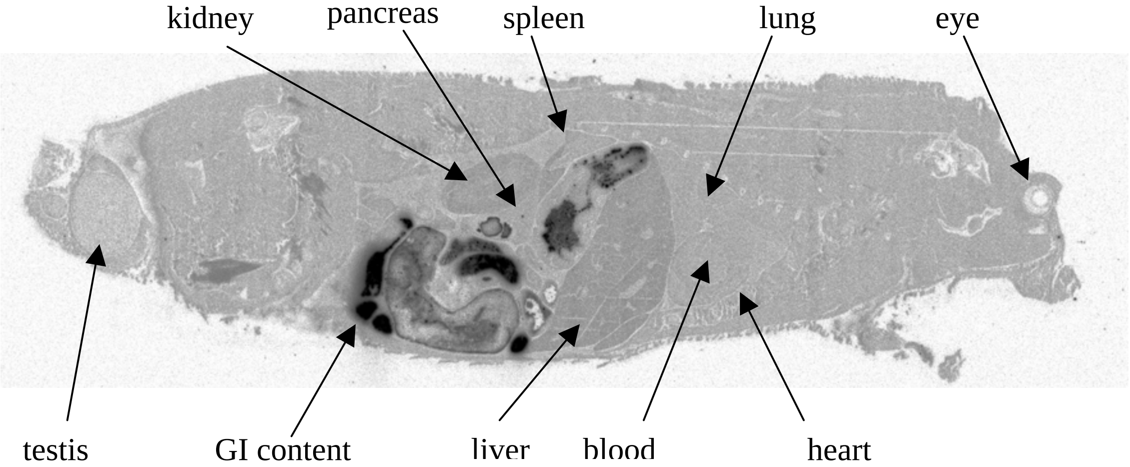

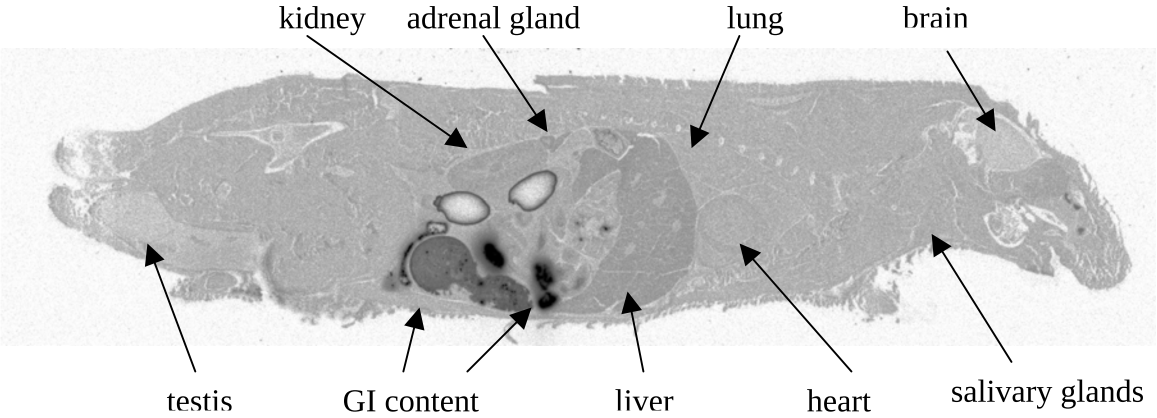

Figure 2. Distribution of total radioactivity at 8 hours following a single oral administration of [^{14}C]-FEXINIDAZOLE to male albino rats.

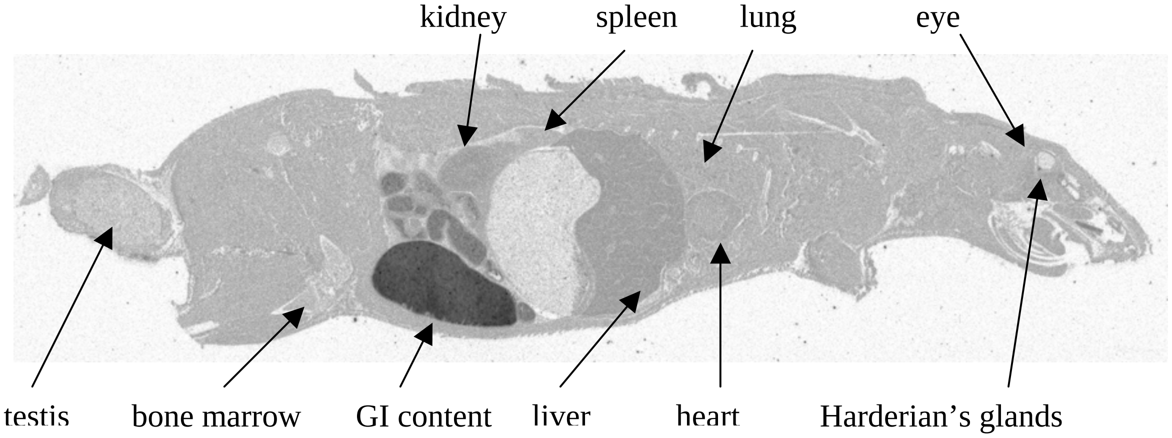

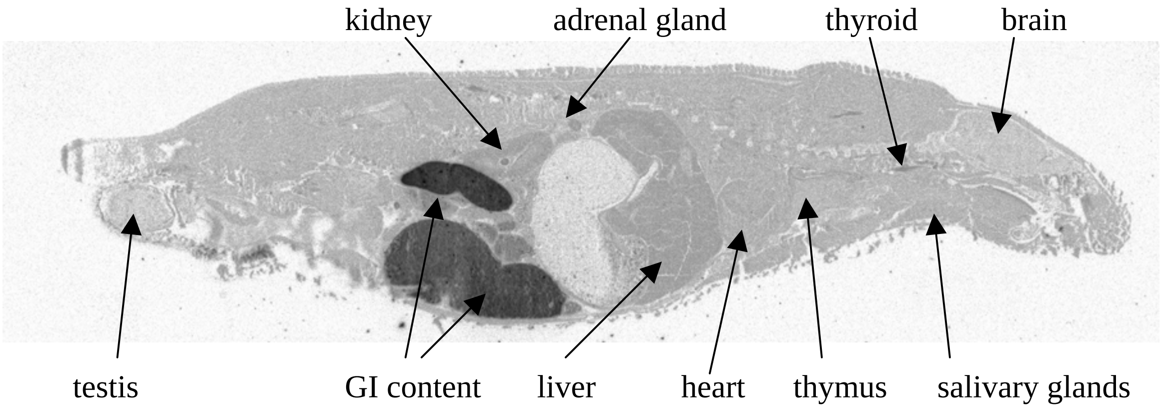

Figure 3. Distribution of total radioactivity at 24 hours following a single oral administration of [^{14}C]-FEXINIDAZOLE to male albino rats.

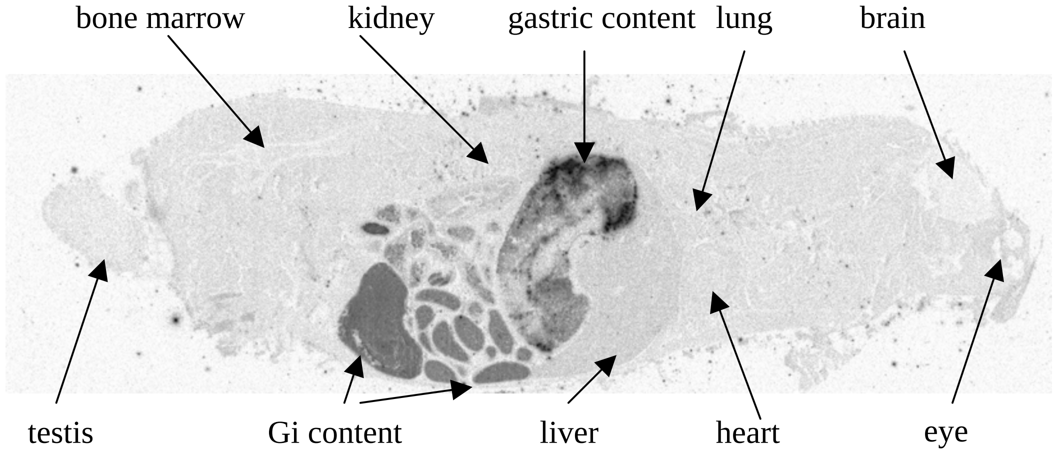

Figure 4. Distribution of total radioactivity at 48 hours following a single oral administration of [^{14}C]-FEXINIDAZOLE to male albino rats.

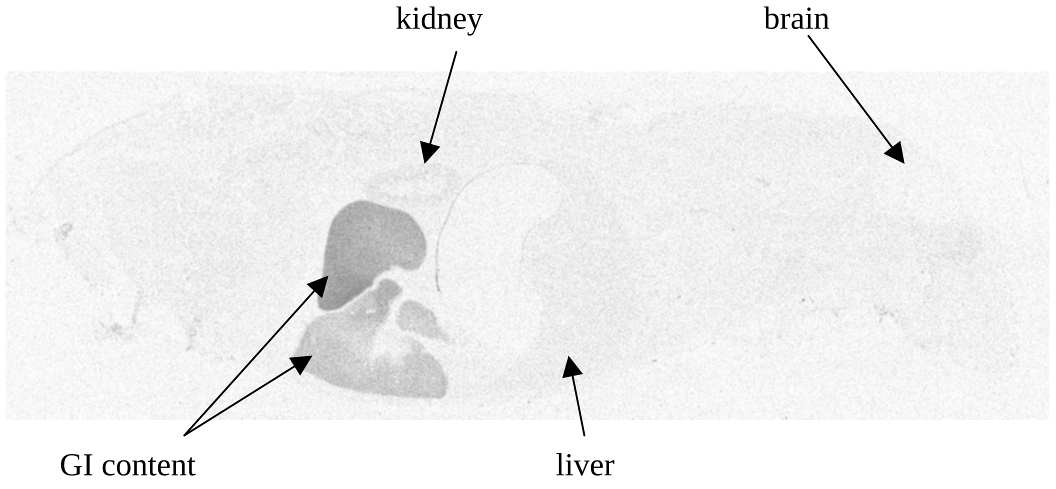

APPENDICES

Appendix 1. Study Protocol

Appendix 2. Analytical Bulletin

NERVIANO MEDICAL SCIENCES

NERVIANO MEDICAL SCIENCE Srl
VIALE PASTEUR, 10
20014 NERVIANO (MI), ITALIA
TEL. +39 0331.581111
FAX +39 0331.581753
WWW.NERVIANOMS.COM

CONFIDENTIAL

Isotope Chemistry

17th April, 2008

RN 03-08

Product	: [^{14}C]Fexinidazole
Labelling Position	:

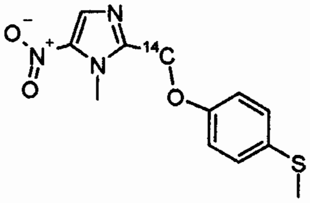

Batch	: F0129/6
Appearance	: yellowish solid
Radiochemical purity (Analysed on 11th April 2008)	: >98% by radio-HPLC (see Enclosure)
Chemical identity	: The material co-chromatographs with authentic material in the above chromatographic system.
Total activity	: 11.1 MBq (301 μCi)
Specific activity	: 2.13 GBq/mmol (57.7 mCi/mmol)
Packing	: 50 ml flask
Storage conditions	: store at –20°C in the dark
Expiry date	: Due to unpredictable radiochemical decompositions, the compound should be tested before use.
Request	: Study Protocol N° 0510-2007 THE PREPARATION WAS USED ALSO FOR THE ADMINISTRATION OF THE ANIMAL M04, STUDY 062-2008
Approval	: Dr. Erminia Fontana Erminia Fontana. 17-April-08

[illegible] 13 oct 2008

THIS PRODUCT HAS BEEN PREPARED FOR LABORATORY USE ONLY AND IS NOT WARRANTED FOR USE IN HUMANS

SEDE LEGALE VIALE PASTEUR, 10 - 20014 NERVIANO (MI) CAPITALE SOCIALE € 32.000,00 REGISTRO DELLE IMPRESE N. 04379750963 TRIB. DI MILANO R.E.A. N. 1743791 C. F. E P. IVA N. 04379750963

[^{14}C]Fexinidazole, batch F0129/6 :
Radio-HPLC profile (upper panel) and UV profile at 254 nm (lower panel)

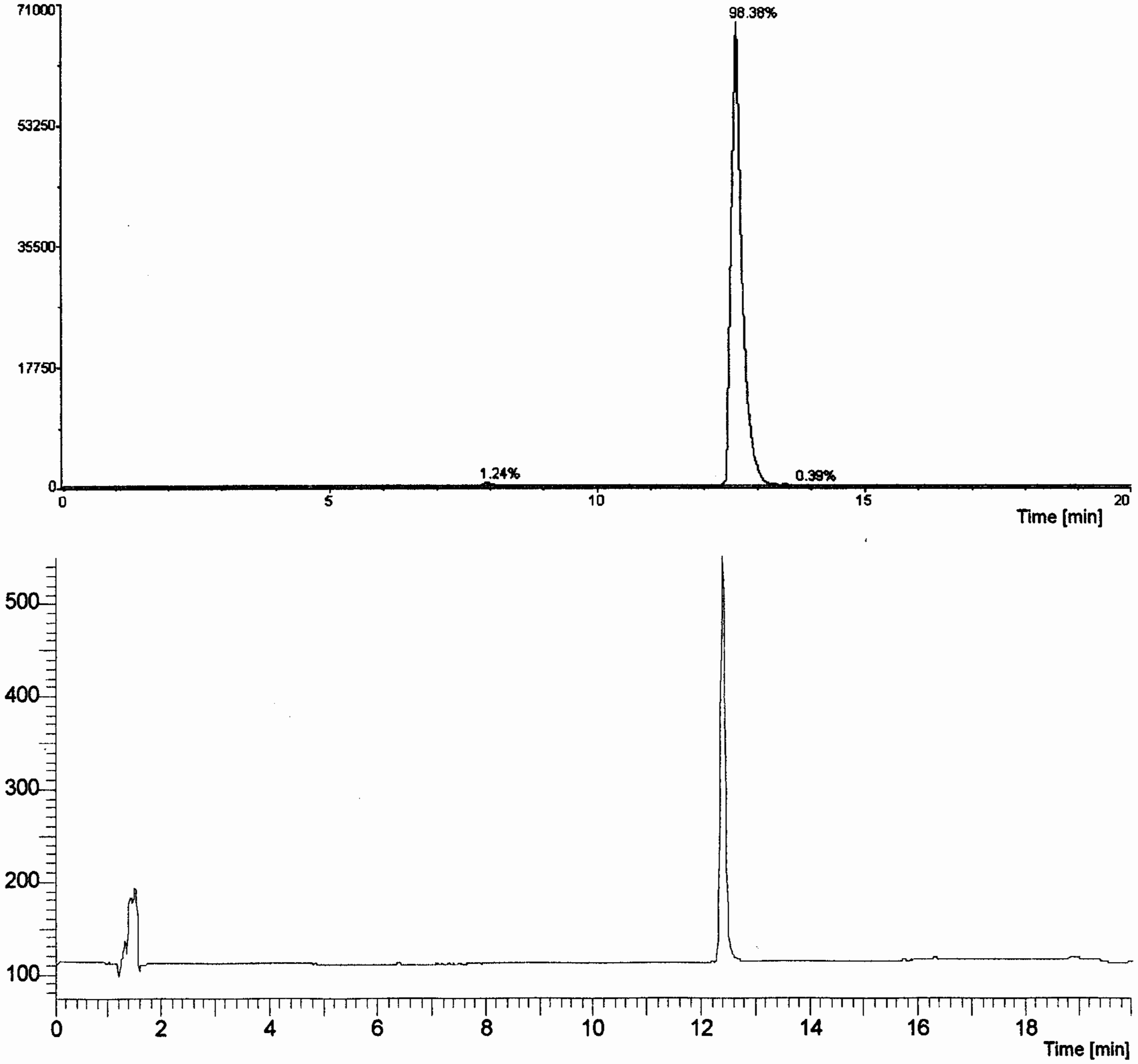

HPLC Method

Column : **XBridge RP18; 100x4.6 mm ID (5µm); supplied by Waters**
Column temperature : **30°C**
Mobile phase A : **Acetonitrile: water: trifluoroacetic acid (10:90:0.1 by volume)**
Mobile phase B : **Acetonitrile: water: trifluoroacetic acid (90:10:0.1 by volume)**
Elution :

time interval (min.)	*pump condition*	*%A*	*%B*
0	ready-to-run	100	0
15	linear gradient	0	100
3	isocratic	0	100
1	re-equilibration, gradient	100	0
4	re-equilibration, isocratic	100	0

Mobile phase flow rate: **1 ml/min.**
UV detection : **analytical wavelength: 254 nm.**
Radiometric detection : **homogeneous (cell volume = 500 µl; scintillation cocktail = Packard Ultima Flo-M; scintillation cocktail to HPLC effluent ratio 2.5:1 by volume)**

ORGASYNTH INDUSTRIES CONFIDENTIAL

ORGASYNTH groupe

ANALYSIS CERTIFICATE

(LEGGI: 2008)

3/10/2008

FEXINIDAZOLE

Manufactur. date	November 2007
Expiry date	October 2000
	Page 1/1

ANALYSIS DATE	November 22 , 2007
ANALYSIS N° CA	07327/01
BATCH N°	3168-07-01/O
WEIGHT (kg)	5
SPECIFICATION	3168 Edition B

DETERMINATIONS	RESULTS	SPECIFICATIONS
IDENTIFICATION	IR Spectrum complies	IR Spectrum complies
APPEARANCE	powder	powder
COLOUR	yellow	yellow
LOSS ON DRYING (%)	0,0	<= 0,5
SULPHATED ASH (%)	0,0	<= 0,1
$HClO_4$ ASSAY (%)	100,2	98,5 à 101,5
RELATED SUBSTANCES - HPLC- Any known impurity (%)	< 0,05	<= 0,15
RELATED SUBSTANCES - HPLC- Any other impurity (%)	0,08	<= 0,10
RELATED SUBSTANCES - HPLC - All impurities sum (%)	0,1	<= 0,5
RESIDUAL SOLVENTS -GC- Acetone (ppm)	740	<= 5000
RESIDUAL SOLVENTS -GC- Methanol (ppm)	20	<= 3000
RESIDUAL SOLVENTS -GC- Toluene (ppm)	4	<= 890

COMMENTS :
NTR

STORAGE REQUIREMENTS : SEE SAFETY DATA SHEET

CONFORMITY	YES / NO

S. SUCHET<br>Quality Assurance Department<br>Date : Visa :<br>December 19, 2007<br>STS	COPIA CONFORME ALL'ORIGINALE<br>RM 12/03/08	M.CONNAN<br>Quality Control Manager<br>Date : Visa :<br>December 18, 2007

AFAQ ISO 9001

Siège social et usine : Chemin de la Madeleine - B.P. 45249 - 06131 GRASSE Cédex - FRANCE
Téléphone : 33 (0)4 93 70 01 32 - Télécopie : 33 (0)4 93 70 05 65 - E-mail : orgasynth-ind.contact@orgasynth.com - www.orgasynth.com

S.A.S. au Capital Social de 1 525 000 € - SIREN : 326 171 378 - R.C.S. 326 171 378 B Grasse - TVA : FR 68 326 171 378 - APE 241 G

Appendix 3. Individual Raw Data (Autoradioluminograms)

0510-2007. Distribution of radioactivity following Single Oral Administration of [14C]-Fexinidazole to male Sprague Dawley rat (animal M1, 2h after administration)

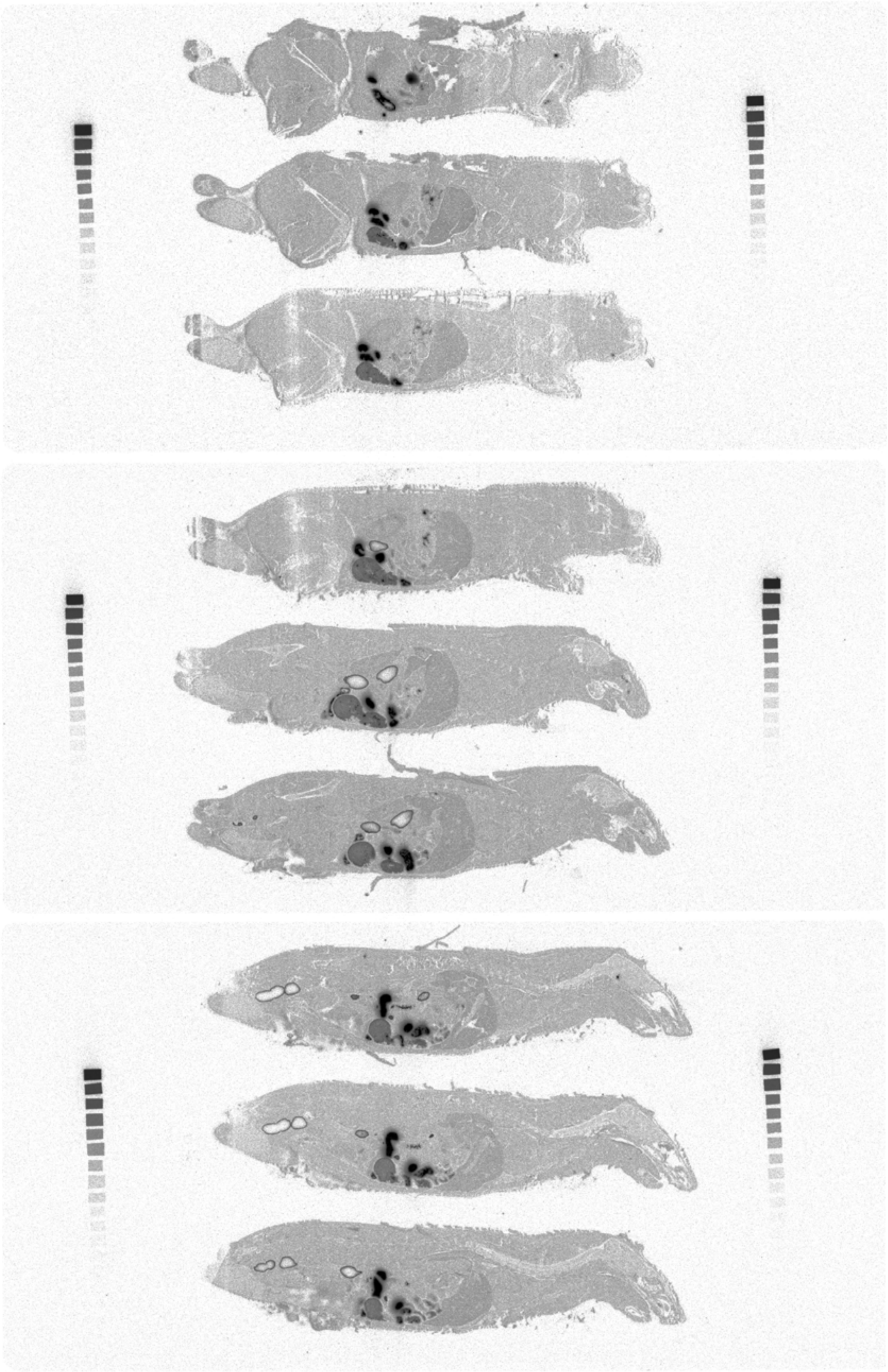

0510-2007. Distribution of radioactivity following Single Oral Administration of [14C]-Fexinidazole to male Sprague Dawley rat (animal M2, 2h after administration)

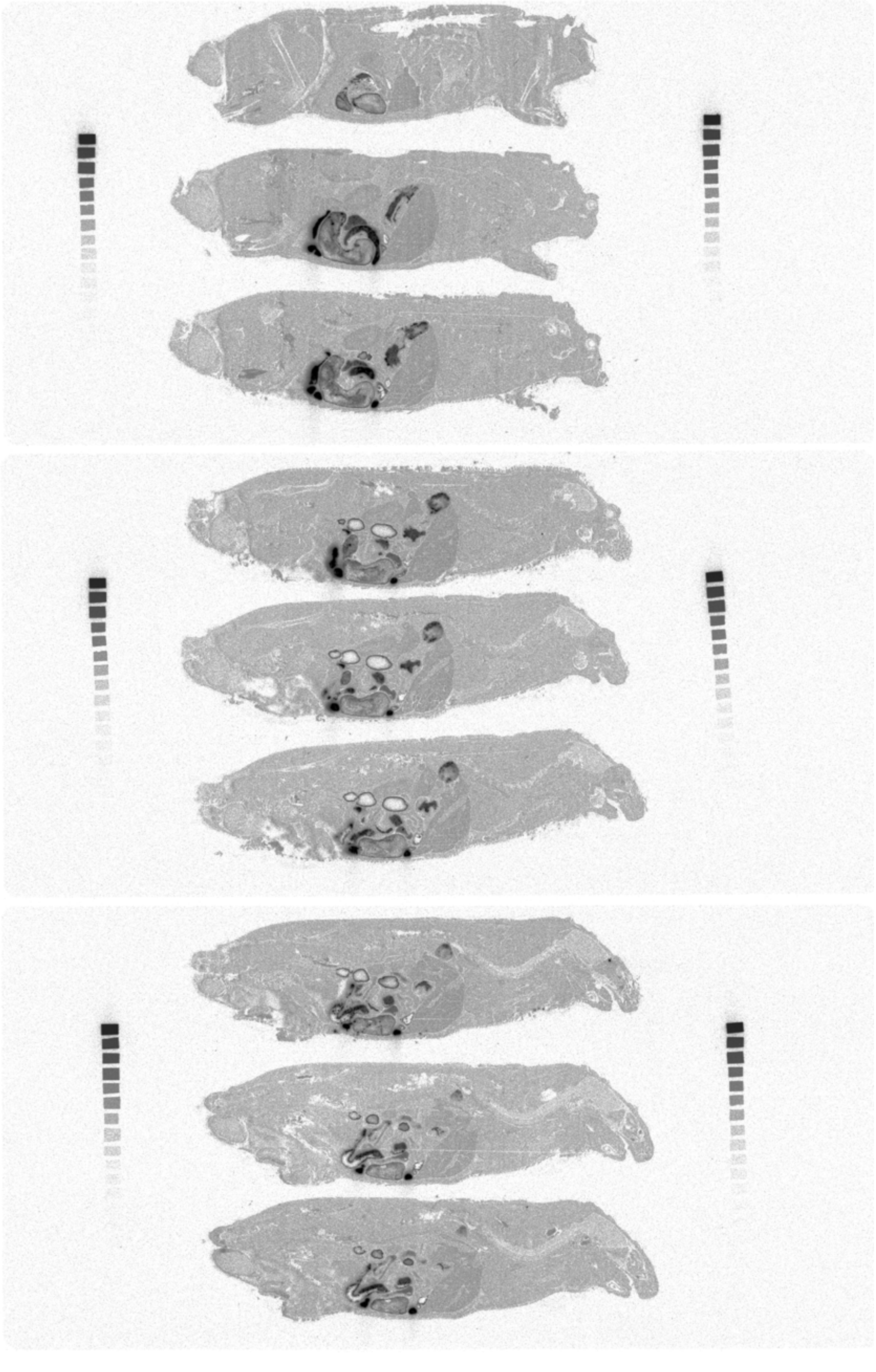

0510-2007. Distribution of radioactivity following Single Oral Administration of [14C]-Fexinidazole to male Sprague Dawley rat (animal M3, 8h after administration)

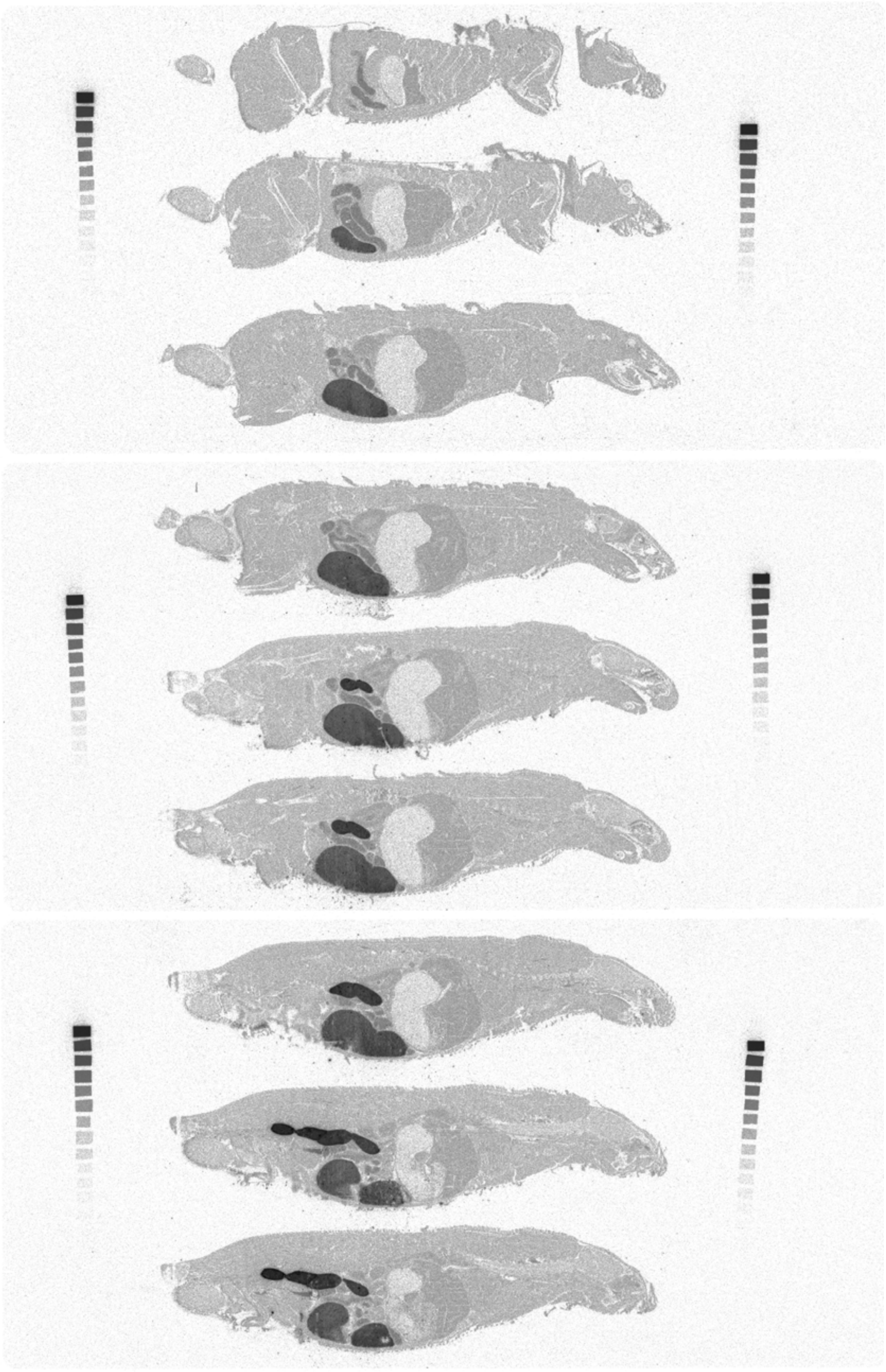

0510-2007. Distribution of radioactivity following Single Oral Administration of [14C]-Fexinidazole to male Sprague Dawley rat (animal M4, 8h after administration)

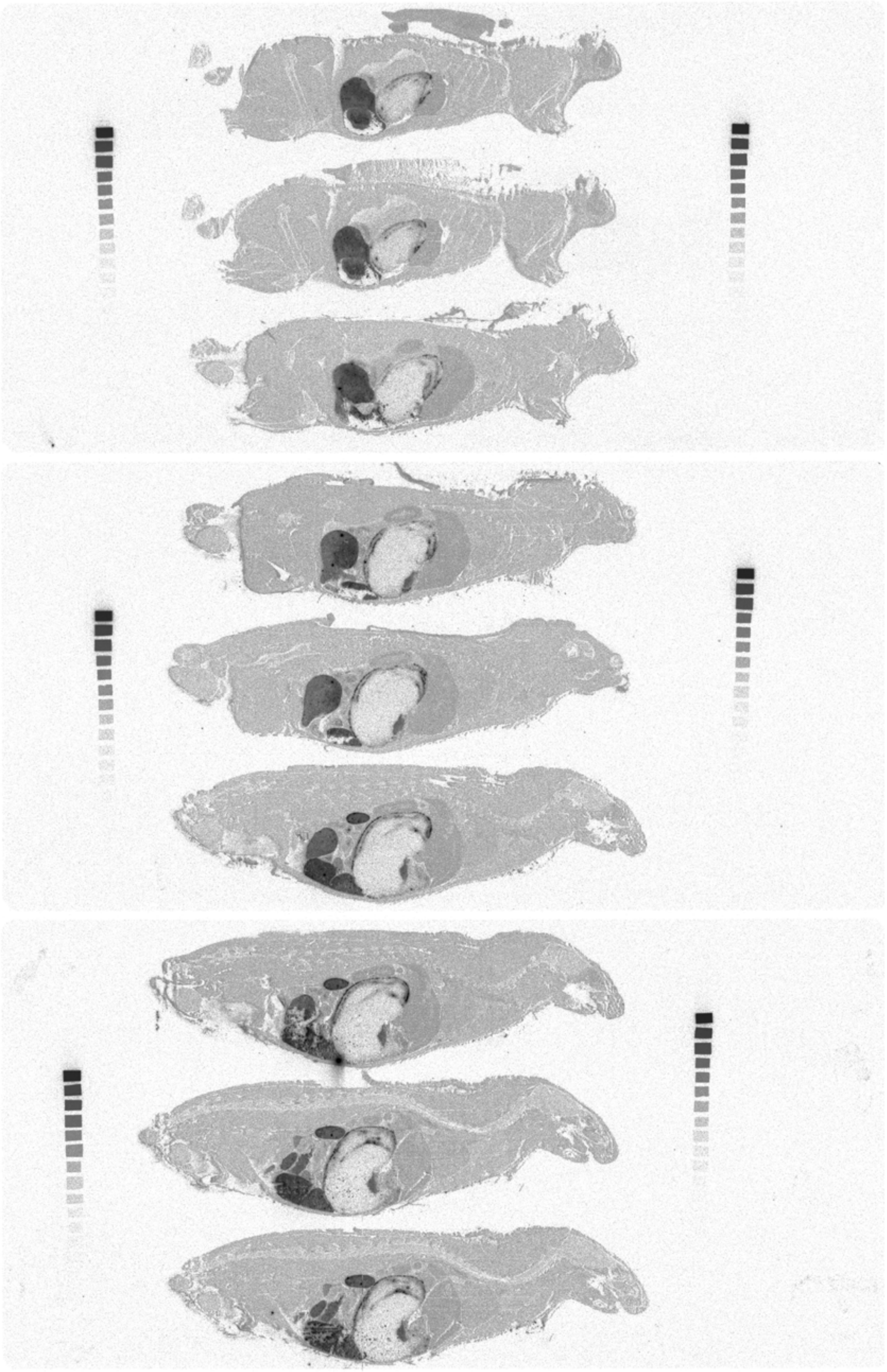

0510-2007. Distribution of radioactivity following Single Oral Administration of [14C]-Fexinidazole to male Sprague Dawley rat (animal M5, 24h after administration))

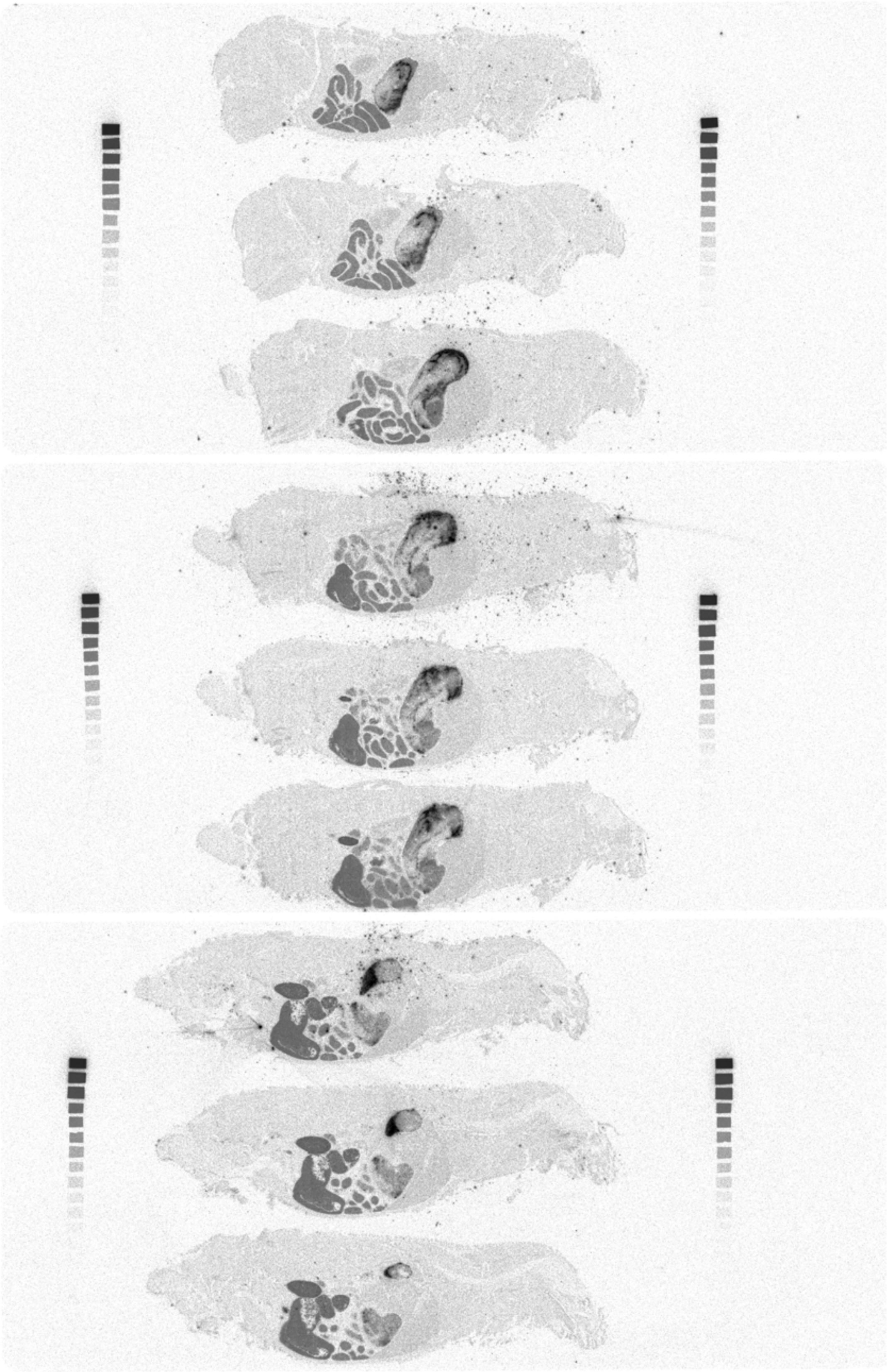

0510-2007. Distribution of radioactivity following Single Oral Administration of [14C]-Fexinidazole to male Sprague Dawley rat (animal M6, 24h after administration)

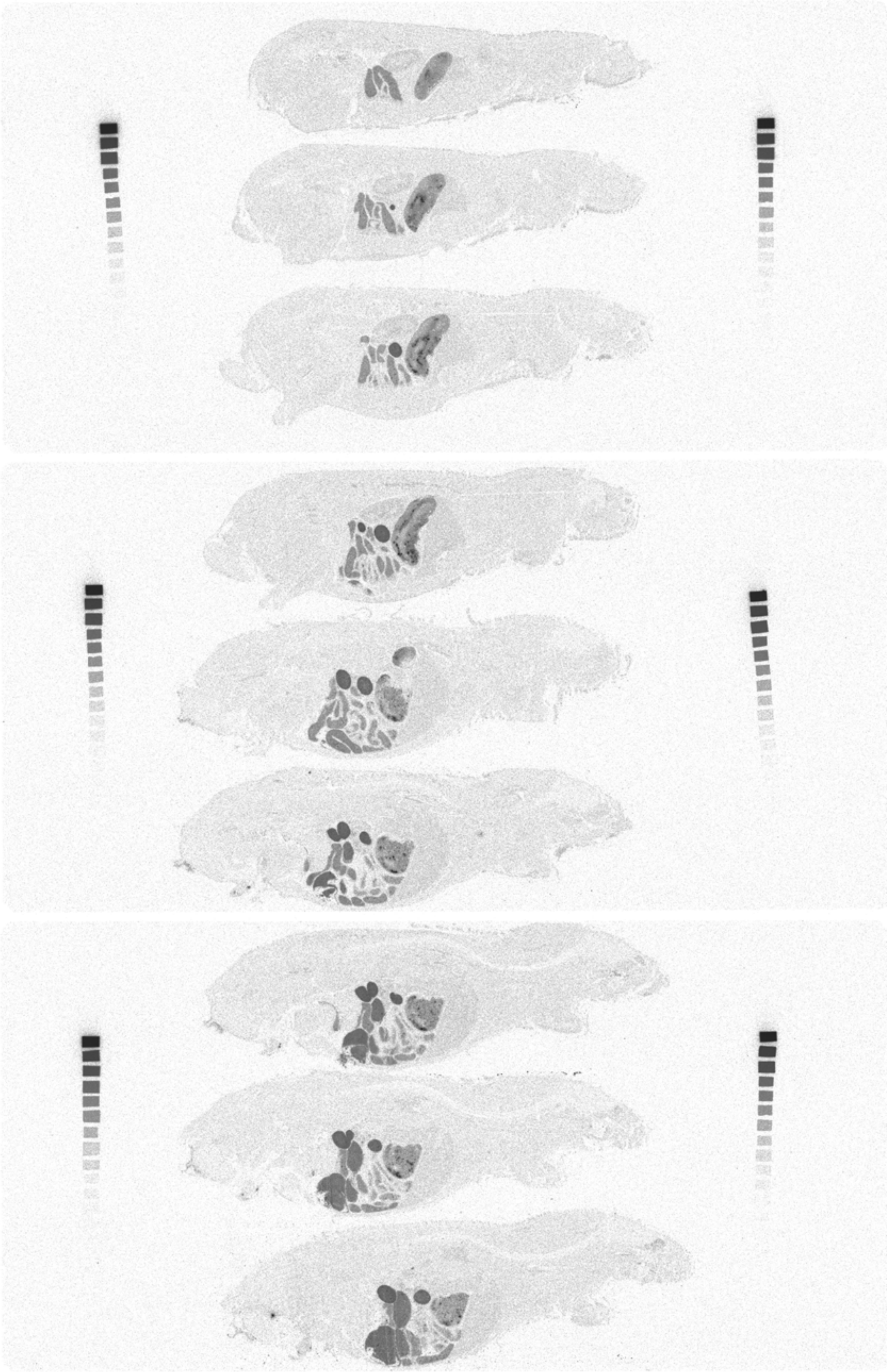

0510-2007. Distribution of radioactivity following Single Oral Administration of [14C]-Fexinidazole to male Sprague Dawley rat (animal M7, 48h after administration)

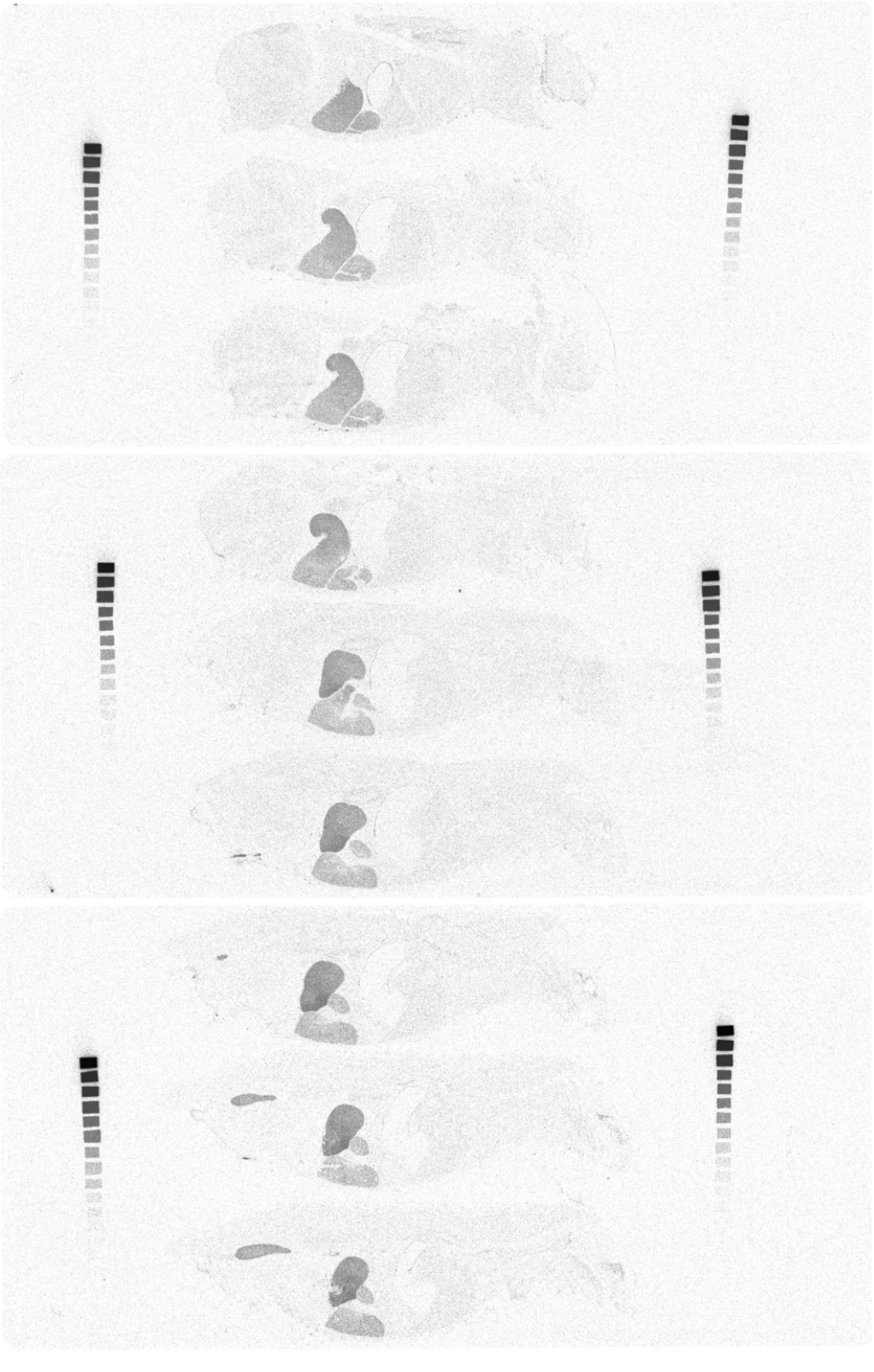

0510-2007. Distribution of radioactivity following Single Oral Administration of [14C]-Fexinidazole to male Sprague Dawley rat (animal M8, 48h after administration)

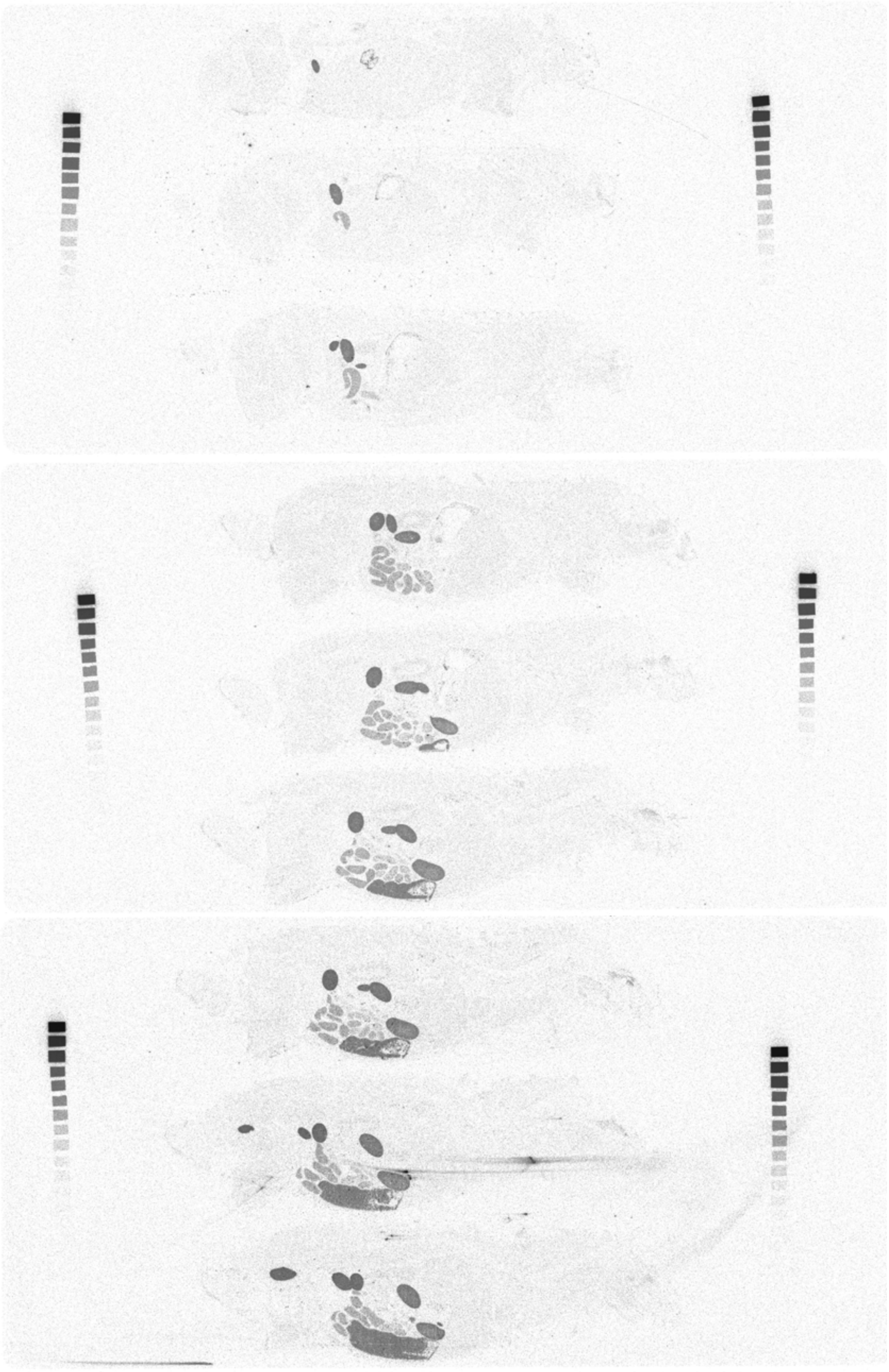

Supplement: Dataset S10 — (7.51 MB PDF) [file pntd.0000923.s011.pdf]
