# Supplementary material for: Fexinidazole – A New Oral Nitroimidazole Drug Candidate Entering Clinical Development for the Treatment of Sleeping Sickness
Source: PLoS Negl Trop Dis. 2010 Dec 21;4(12):e923. doi: 10.1371/journal.pntd.0000923 (PMC3006138; doi:10.1371/journal.pntd.0000923)
Supplement: Dataset S11 — (0.35 MB PDF) [file pntd.0000923.s012.pdf]

Fexinidazole: Determination of Excretion Balance following Single Oral Administration of [^{14}C]-Fexinidazole to Rats.

Product Name :	FEXINIDAZOLE
Study Number:	0162-2008
Study Director/Author:	
Sponsor Reference Study No.:	N.A
Status:	FINAL

SUMMARY

Aim of this study was to obtain information on the elimination of radioactive drug-related material in urine and faeces following a single oral administration of [^{14}C]-FEXINIDAZOLE to male albino rats.

[^{14}C]-FEXINIDAZOLE was administered at the target dose level of 800 mg/kg (approximately 3.7 MBq/kg, 100 μCi/kg) to 3 male Sprague Dawley rats.

The excretion balance of radioactivity was determined up to 96 hours after administration. The radioactivity levels in the excreta and in the carcasses were determined by Liquid Scintillation Counting (LSC).

The radioactive drug related material excreted *via* the urine within 96 hours after administration accounted for about 30 % of the dose; the elimination of radioactivity in faeces was approximately 59 % of the dose. The mean total recovery of radioactivity eliminated in the excreta (including cage washes) in the 0-96 hours after test compound administration accounted for about 91 % of the dose. The elimination of the compound and/or its metabolites after oral dosing was rapid, most of the radioactivity (about 84 % of the dose) being eliminated during the first 48 hours.

At the end of the collection period of excreta about 1.4 % of the dose was recovered from the carcass and the skin. As an overall mean, the recovery of the total radioactivity accounted for approximately 93 % of the dose in the 0-96 hours after administration.

TABLE OF CONTENTS

1. INTRODUCTION AND OBJECTIVES ... 8
2. STUDY SPONSOR ... 9
3. TEST FACILITY ... 9
4. REGULATORY REQUIREMENTS ... 9
5. ABBREVIATION AND DEFINITIONS OF TERMS ... 9
6. MATERIALS AND METHODS ... 10
6.1. Test Item ... 10
6.2. Chemicals ... 10
6.3. Instrumentation ... 10
6.4. Test System ... 11
6.4.1. Environment ... 11
6.4.2. Diet and Drinking Water ... 11
6.5. Experimental Procedures ... 11
6.5.1. Animal Observations ... 11
6.5.2. Body Weights ... 11
6.6. Preparation and Analysis of Dose Formulation ... 11
6.6.1. Dose Formulation ... 11
6.7. Animal Treatment ... 12
6.7.1. Administered Doses ... 12
6.8. Sample Collection and Analysis of Radioactivity ... 12
6.8.1. Sample Collection ... 12
6.8.2. Preparation of Samples for Analysis of Total Radioactivity ... 13
6.8.3. Quantification of Total Radioactivity ... 13
6.8.4. Radioactivity Data Processing ... 14
6.9. Sample Storage ... 14
6.10. Data Presentation ... 14
7. PROTOCOL DEVIATIONS ... 14
8. ARCHIVING ... 14
9. RESULTS AND DISCUSSION ... 15

9.1. Analysis of Dose Formulation 15
9.2. Animal dosing 15
9.3. Study Observations 15
9.4. Elimination of Total Radioactivity 15
10. CONCLUSIONS 16
11. CONTRIBUTORS 16

TABLES

Table 1. Dosing data for the single oral administration of [^{14}C]-FEXINIDAZOLE to male rats. 17
Table 2. Individual and mean (±SD) excreted percent of dose following a single oral administration of [^{14}C]-FEXINIDAZOLE at a target dose level of 800 mg/kg to male rats. 18

Figure 1. Mean of percent of the radioactive dose recovered in excreta (cumulative % of urine and faeces) following a single oral administration of [^{14}C]-FEXINIDAZOLE at a target dose level of 800 mg/kg to male rats. 19

APPENDICES

Appendix 1. Study Protocol and Amendment

Appendix 2. Analytical Bulletin

Appendix 3. Raw Data Report of Individual Samples

1. INTRODUCTION AND OBJECTIVES

This study was conducted according to Protocol N° 0162-2008 and to Amendment 1. A copy of the experimental protocol is included in Appendix 1; a copy of the amendment is included in Appendix 1.

FEXINIDAZOLE is a 5-nitroimidazole derivative biologically active against Trypanosoma parasites (*T.b. rhodesiense* and *T.b. brucei*) and useful in the treatment of the Human African Trypanosomiasis (HAT), known as sleeping sickness. FEXINIDAZOLE is a compound currently under development by the Sponsor.

The objective of this study was to obtain information on the elimination of radioactive drug-related material in urine and faeces following oral administration of [^{14}C]-FEXINIDAZOLE (800 mg/kg) to male Sprague Dawley rats.

The albino Sprague Dawley rat was chosen as the species for this study since it was one of the species used in the toxicological evaluation of the test compound.

Only male animals were used as no gender differences were expected with respect to the aim of the study. The oral route of administration was chosen as this is the intended therapeutic route. The dose level was selected in agreement with the Study Sponsor and it is within the pharmacological relevant range. No overt toxicity was expected after single dosing at this dose level.

The study was conducted on 3 male animals after single oral administration of [^{14}C]-FEXINIDAZOLE (800 mg/kg, approximately 3.7 MBq/kg, 100 µCi/kg).

Urine and faeces were collected up to 96 hours after administration. The radioactivity levels of drug-related material were determined in each collected sample by Liquid Scintillation Counting (LSC).

A summary of the experimental design is reported below:

**Animal no.**	**Date of Dosing**	**Administered Dose (oral administration)**	**Samples collected and analyzed**
M01, M03	April 21, 2008	800 mg/kg<br>100 µCi/kg<br>10 mL/kg	urine, faeces, cage washes, carcass and skin
M04	June 10, 2008		

The experimental phase of the study started on April 21, 2008 and was completed on June 25, 2008 (LSC analysis).

2. STUDY SPONSOR

DND*i* – Drugs for Neglected Diseases *Initiative*

3. TEST FACILITY

Accelera
Nerviano Medical Sciences S.r.l.
Viale Pasteur, 10
20014 Nerviano, Milan, Italy

4. REGULATORY REQUIREMENTS

This study was conducted in compliance with the *DECRETO LEGISLATIVO 2 Marzo 2007, No. 50* and with the *Organisation for Economic Co-operation and Development (OECD) Principles of Good Laboratory Practice (GLP) (as revised in 1997).*

This study was conducted according to the methods described in the "Standard Operating Procedures" of the laboratories involved.

5. ABBREVIATION AND DEFINITIONS OF TERMS

BLQ	Below the limit of quantification
C_{max}	Maximal concentration
dpm	Disintegration per minute
h	hour
ID	Animal Code
LSC	Liquid Scintillation Counting
LOD	Limit of detection
LOQ	Limit of quantification
ngeq/g	nanogram equivalent/gram
NA	Not available
ND	Not detectable
NQ	Not quantifiable
SD	Standard Deviation of the mean

6. MATERIALS AND METHODS

6.1. Test Item

The test material was prepared in Accelera, Nerviano Medical Sciences, mixing appropriate amounts of [^{14}C]-FEXINIDAZOLE (batch No F0129/6 and batch No F0129/7, Specific Activity: 57.7 mCi/mmol, radiochemical purity >98%) and unlabelled FEXINIDAZOLE (Centipharm batch No. 3168-07-01/O, purity 100.2%) provided by the Study Sponsor, in order to obtain [^{14}C]-FEXINIDAZOLE at the final Specific Activity of 4.625 KBq/mg (0.125 μCi/mg). The analytical bulletins are included in Appendix 2.

6.2. Chemicals

Methylcellulose (400 cP), used for test item formulation, was obtained from Sigma-Aldrich

Tween 80, used for test item formulation, was obtained from Sigma-Aldrich

Water for injection, used for test item formulation, was obtained from Bieffe Medital S.p.A.

Sodium Heparin, Vister®, used as anticoagulant, was obtained from Pfizer.

Carbo-Sorb® CO_2 absorbing solution and Permafluor® E+ scintillation fluid were used in conjunction with Packard Automatic Sample Preparation System 387 and were supplied by Perkin-Elmer Life Science. Ultima Gold, used as liquid scintillation cocktail, and Spec-Chec™-^{14}C, used to estimate efficiencies of combustion, were also obtained from Perkin-Elmer Life Science.

Reagents and solvents (methanol) were of analytical grade (or equivalent), obtained from Carlo Erba Reagents.

6.3. Instrumentation

Balances, mod. AG204, AT201, AT250, and PB5001 Mettler.

Liquid scintillation analyzer, mod.1900 TR and 1900 CA, Packard.

Stomacher 80 homogenizer, PBI International.

Automatic Sample Preparation System 387 (Oxidizer 307 and Oximate 80), Packard.

Centrifuge Megafuge 1.0R, Heraeus

Potter-Elvehjem homogeniser AT123, Forlab.

Blixer 4 homogenizer, PBI International.

Lyophilizer Minifast 1000, Edwards.

6.4. Test System

Sprague Dawley rats (3 males, adults, age 8 weeks, body weight 264-277 g at the time of dosing) were used. These animals were supplied by Charles River, Calco (CO) Italy. All animals were visually inspected for signs of illness and were deemed fit for use in the study.

6.4.1. Environment

During pretrial holding period, the rats were housed in polypropylene and stainless steel cages with wood shavings as bedding. During the experimental period, rats were individually housed in glass metabolism cages specially designed for the separate collection of faeces and urine. Holding and study areas had automatic control of light cycle and temperature. The lighting in the study unit was controlled in a 12-hour light-dark cycle throughout the study. Temperature and relative humidity measured during the study were in the range 22.0 –22.5 °C and 45-49 %, respectively.

6.4.2. Diet and Drinking Water

A complete dry diet (Mucedola 4RF21) was available *ad libitum*. Domestic mains quality water was available *ad libitum* throughout the study. Certificates of analysis from the manufacturer of the diet batches were obtained and were included in the raw data. The water was periodically analyzed for chemical and microbial impurities and the certificates of analysis supplied from the local water authority. There were no contaminants in the diet or water that were considered to have potentially affected the integrity or outcome of the study.

The animals were fasted overnight before administration; food was allowed to the animals from approximately 2 hours after dosing.

6.5. Experimental Procedures

6.5.1. Animal Observations

The animals were observed during the treatment and routinely during the whole course of the experiment to evaluate any evidence of reaction to treatment, change in general appearance or overt signs of suffering.

6.5.2. Body Weights

Animals were weighed before treatment on the morning of dosing. Individual animal weights are reported in Table 1.

6.6. Preparation and Analysis of Dose Formulation

6.6.1. Dose Formulation

The test formulations were prepared on the days of dosing.

The test formulations were prepared by suspending unlabelled FEXINIDAZOLE and [^{14}C]-FEXINIDAZOLE (see section 5.1) in 5% Tween 80 and 0.5% Methyl cellulose 400 cP (Methocel) in water, in order to obtain [^{14}C]-FEXINIDAZOLE at the final target concentration of 80 mg/mL corresponding to a radioactivity concentration of about 370 kBq/mL (10 µCi/mL).

The test formulation was prepared by suspending an appropriate amount of unlabelled FEXINIDAZOLE in the dose vehicle. This formulation was homogenised using a Potter-Elvehjem homogenizer apparatus (teflon pestle of approx. 30 mm x 53 mm and glass tube of 40 mL), in order to obtain a fine suspension by up-and-down strokes, gently made by hand. The formulation was then collected and transferred from the homogeniser glass tube to the glass pot containing the [^{14}C]-FEXINIDAZOLE.

During the preparation the test formulation was protected from light as far as possible.

The radioactivity concentration and homogeneity of the test formulation were determined, before administration, by liquid scintillation counting of triplicate weighed aliquots of test formulation. The radiolabelled test formulation was used immediately after formulation.

6.7. Animal Treatment

6.7.1. Administered Doses

The dose of [^{14}C]-FEXINIDAZOLE was orally administered by gastric gavage at the target dose level of 800 mg/kg (radioactivity dose of about 3.7 MBq/kg, 100 µCi/kg) and at a target dose volume of about 10 mL/kg.

Individual doses were prepared for each animal by weighing the appropriate amount of the test formulation in an appropriate syringe for oral administration. The amount of dose administered to each animal was determined by weighing the filled syringe before the treatment and the empty syringe after dose administration. The actual dose received by each animal was calculated using the weight of the administered formulation, the radioactivity concentration and the final specific activity of the test material.

The actual doses received by each animal are documented in Table 1.

6.8. Sample Collection and Analysis of Radioactivity

6.8.1. Sample Collection

Urine and faeces samples were collected from each animal at pre-dose and over 0-8, 8-24, 24-48, 48-72 and 72-96 hours post dose. All urine and faeces samples were collected into pre-weighed, ice-cooled containers protected from light; urine and faeces samples were weighed before freezing.

Each day, at the end of each period of urine and faeces collection, the cages were washed with water (approximately 100 mL) and the washing was retained separately for radioassay. Final washing was performed using about 100 mL of water-methanol solution (1:1 v/v).

Carcasses and skin were collected for analysis.

6.8.2. Preparation of Samples for Analysis of Total Radioactivity

Duplicate aliquots of urine (ca. 0.25 mL) and cage washing (ca. 1 mL) were collected into polypropylene vials and were mixed with 18 mL of Ultima Gold scintillation cocktail before liquid scintillation counting.

Faeces samples were thawed, added with an appropriate amount of water (approximately 1:1 w/v) and re-weighed before homogenization. Samples were homogenized into appropriate plastic bags using the Stomacher 80 system; each faecal sample was processed at medium speed for 2-3 minutes. Duplicate aliquots of each homogenate (ca. 0.5 g) were weighed in a paper cone and combusted using a Packard Automatic Sample Preparation System 387.

The carcass and skin were lyophilized and weighed. After lyophilization carcass and skin were then homogenized using an appropriate blender apparatus. Triplicate aliquots (ca. 0.5 g) of each lyophilized and homogenised sample were weighed in a paper cone and combusted using Packard System 387.

$^{14}CO_2$ generated from the combustion of each sample was collected by absorption in Carbosorb® (8 mL), then Permafluor® E^+ scintillation fluid (12 mL) was added. Combustion of standards (Spec-Chec™-^{14}C) showed that recovery efficiencies were >97.7 %. The results of combusted samples (faeces and carcasses) were corrected for the corresponding efficiency factors.

6.8.3. Quantification of Total Radioactivity

Radioactivity was measured by LSC using Packard liquid scintillation analyzers. Samples were counted up to 1 hour (with the 2 sigma% settled at 0.30 region A and 0.50 region B), together with representative blank and standard vials.

Counting efficiencies were calculated by the external standard method using a series of quenched standards supplied by Packard, in order to generate the calibration curves. The validity of calibration curves was checked before analysis. Samples were allowed to light stabilize before the analysis. Before the calculation of each result, a background disintegration rate was measured and subtracted from each sample count rate. Where appropriate, background disintegration rates were measured in pre-dose samples. The limit of detection (LOD) of radioactivity by LSC was defined as twice the background level.

6.8.4. Radioactivity Data Processing

The weights of samples and the radioactivity disintegration rates were directly captured from the output of analytical balances and liquid scintillation counters respectively or manually introduced into a validated Laboratory Information Management System (DEBRA v.5.4, by LabLogic, UK) to be processed for determination of the administered doses to the animals, the radioactivity concentration in the samples and the percentages of dose recovered.

6.9. Sample Storage

The samples of urine and faeces were frozen and stored at -80° C after collection, except for the aliquots removed for radioanalysis. The whole carcasses and skin were frozen and stored at -20° C after collection, except for the aliquots removed for radioanalysis.

6.10. Data Presentation

Excretion data, expressed as % of administered dose, are presented to two decimal places. Data presented in Tables are computer generated and appropriately rounded for inclusion in the report. As a consequence, in some instances, calculation of values from data presented may yield minor variations.

7. PROTOCOL DEVIATIONS

The animal M02 was excluded from the study and the animal M04 substituted for the excluded subject, as described in the Amendment 1.

Due to technical reasons the animal M04 received a dose of approximately 700 mg/kg instead of 800 mg/kg (about 13 % less than the theoretical dose). Considering the aim of the study this deviation does not affect the results of the study. Moreover for this animal administration a formulation prepared for another study (performed by the same test item) was used; as raw data of this formulation preparation a copy of the related logbook was included in study file.

The carcasses were homogenized after lyophilization and then analyzed.

No other deviations from the Protocol occurred during this study.

8. ARCHIVING

All raw data, supporting documents produced at the Test Facility, a copy of the documentation of the test item received by the Sponsor, the Protocol, the Amendment and the final Report as original were filed in the Archives of Accelera, Nerviano Medical Sciences, Nerviano, Italy for a period of 3 years, after which the Sponsor will be contacted for instructions regarding dispatch or disposal of the material. Specimens requiring storage deep frozen are specifically excluded from the above. These will be retained for as long as the quality of the material permits evaluation but for no longer than 6 months after issue of

the final report. The study Sponsor will be notified before specimens are destroyed on their behalf.

The copy of the Protocol and of the Amendment with original signatures and the copy of study Report with original signatures were delivered to the Sponsor.

9. RESULTS AND DISCUSSION

9.1. Analysis of Dose Formulation

The radioactivity concentrations of [^{14}C]-FEXINIDAZOLE measured in the test formulations used for the administrations were 422.91 kBq/g (11.43 µCi/g) and 394.05 kBq/g (10.65 µCi/g) corresponding to compound concentrations of 91.4 and 85.2 mg/g of formulation, respectively.

9.2. Animal dosing

The doses received by the animals were in the range of 697.7 - 805.4 mg/kg. The actual doses of radioactive test compound administered to the animals are detailed in Table 1.

9.3. Study Observations

No overt adverse signs were observed in the test animals during the conduct of the study.

9.4. Elimination of Total Radioactivity

Following a single oral administration of [^{14}C]-FEXINIDAZOLE to rats, the radioactive dose excreted *via* the urine within 96 hours after administration accounted for about 29.9 % of dose (about 32.6 % including cage washes); the elimination of radioactivity in faeces was approximately 58.8% of the dose. Approximately 91.4 % of the radioactivity was recovered in all the excreta (including cage washes) in the 0-96 hours after test compound administration.

The elimination of the compound and/or its metabolites after oral dosing was rapid, with most of the radioactivity (about 84.3 % of the dose) eliminated during the first 48 hours.

About 1.4 % of the administered dose was recovered from the carcass and the skin of the animals sacrificed following the 96 hrs post administration period.

As an overall mean, the recovery of the total radioactivity accounted for approximately 93 % (+/- 8%) of the administered dose and therefore the recovery was virtually complete.

The elimination profiles of total radioactivity, expressed in terms of percent of dose, are given in Table 2.

10. CONCLUSIONS

After oral administration of [^{14}C]-FEXINIDAZOLE to male rats the radioactive drug related material excreted *via* the urine within 96 hours after administration accounted for about 30 % of the dose; the elimination of radioactivity in faeces was approximately 59 % of the dose. The mean total recovery of radioactivity eliminated in the excreta (including cage washes) in the 0-96 hours after test compound administration accounted for about 91 % of the administered dose. The elimination of the compound and/or its metabolites after oral dosing was rapid, most of the radioactivity (about 84 % of the dose) being eliminated during the first 48 hours.

About 1.4 % of the dose was recovered from the carcass and the skin. As an overall mean, the recovery of the total radioactivity accounted for approximately 93 % of the dose.

11. CONTRIBUTORS

TABLES AND FIGURES

Table 1. Dosing data for the single oral administration of [^{14}C]-FEXINIDAZOLE to male rats.

Animal number	Animal Weight	Specific Activity	Dose received		
	(g)	µCi/mg	µCi	mg of compound	mg/kg
M01	264	0.125	26.58	212.63	805.41
M03	274		27.22	217.77	794.79
M04	277		24.16	193.26	697.67

Table 2. Individual and mean (±SD) excreted percent of dose following a single oral administration of [^{14}C]-FEXINIDAZOLE at a target dose level of 800 mg/kg to male rats.

Sample	Time Interval (h)	Male Rats M01	M03	M04	Mean	SD
URINE	0-8	4.99	4.34	6.24	5.19	0.97
	8-24	15.57	16.94	15.2	15.9	0.92
	24-48	7.68	6.57	7.92	7.39	0.72
	48-72	1.02	1.08	1.38	1.16	0.19
	72-96	0.35	0.27	0.22	0.28	0.07
	0-96	29.61	29.2	30.96	29.92	0.92
FAECES	0-8	NS	NS	NS	NA	NA
	8-24	22.31	40.11	25.53	29.32	9.48
	24-48	22.07	24.00	25.98	24.02	1.96
	48-72	5.77	3.40	5.80	4.99	1.38
	72-96	0.52	0.30	0.52	0.45	0.13
	0-96	50.67	67.81	57.83	58.77	8.61
CAGE	0-24	2.4	2.54	0.61	1.85	1.08
WASHING	24-48	0.74	0.57	0.72	0.68	0.09
	48-72	0.19	0.14	0.07	0.13	0.06
	72-96	0.10	0.07	0.04	0.07	0.03
	0-96	3.43	3.32	1.44	2.73	1.12
CARCASS	96	0.95	0.98	1.11	1.01	0.09
SKIN	96	0.27	0.56	0.46	0.43	0.15
TOTAL	0-96	84.93	101.87	91.80	92.87	8.52

NS: no sample
NA: not available

Figure 1. Mean of percent of the radioactive dose recovered in excreta (cumulative % of urine and faeces) following a single oral administration of [^{14}C]-FEXINIDAZOLE at a target dose level of 800 mg/kg to male rats.

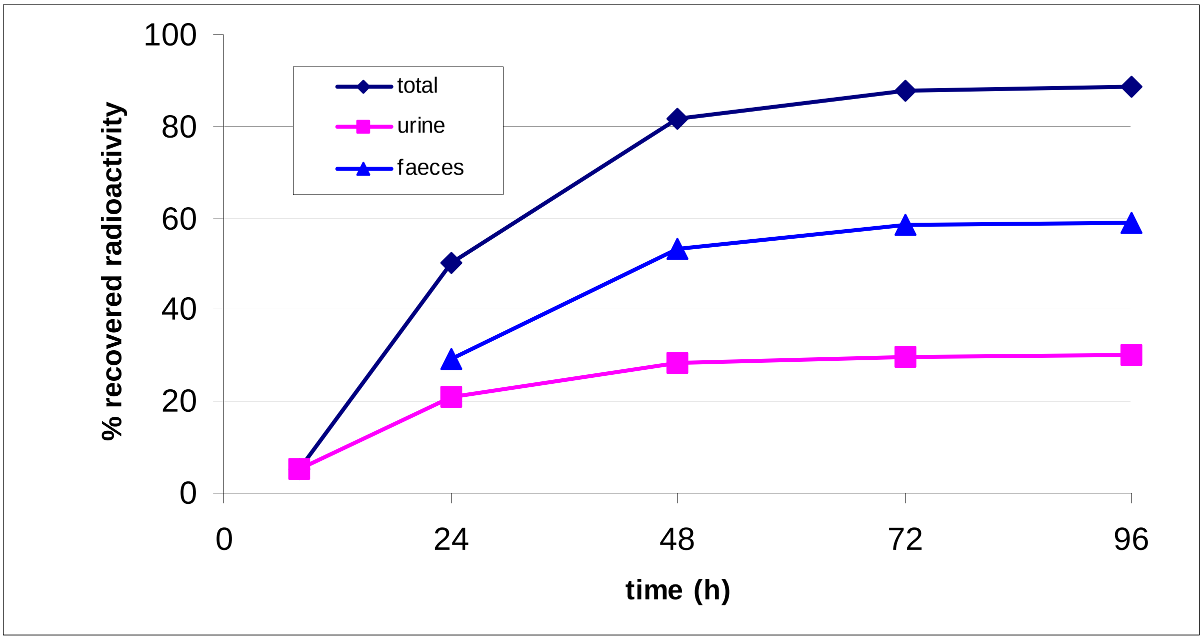

APPENDICES

Appendix 1. Study Protocol and Amendment

Fexinidazole: Determination of Excretion Balance following Single Oral Administration of [^{14}C]-Fexinidazole to Rats.

Product Name:	Fexinidazole
Study Number:	0162-2008
Study Director:	
Sponsor Reference Study No.:	N.A.
Status:	FINAL

TABLE OF CONTENT

1. INTRODUCTION AND OBJECTIVES 5
2. STUDY SPONSOR 5
3. TEST FACILITY 5
4. REGULATORY REQUIREMENTS 5
5. PROPOSED SCHEDULE 5
6. STUDY DESIGN 6
6.1. General Description 6
6.2. Justifications 6
6.3. Dosing Schedule Design 6
7. TEST AND CONTROL ITEM 7
7.1. Test item 7
7.1.1. Radiolabelled Test Item Designation and Specifications 7
7.1.2. Unlabelled Test Item Designation and Specifications 7
7.1.3. Preparation of radiolabelled Test Item 8
7.2. Test Formulation 8
7.2.1. Preparation of dose vehicle 8
7.2.2. Preparation of Radiolabelled dosing formulation 8
7.2.3. Determination of Concentration, Homogeneity and Stability 9
7.2.4. Storage Conditions of Formulated Drug 9
8. TEST SYSTEM 9
8.1. Clinical and physical examinations 9
8.2. Fate of animals at the end of the study 10
9. ENVIRONMENTAL 10
9.1. Location of Study 10
9.2. Environmental Conditions 10
10. EXPERIMENTAL 11
10.1. Method of Dose Administration 11
10.2. Sample Collection, Processing and Schedule 11
10.2.1. Urine 11

10.2.1.1. Method 11
10.2.1.2. Collection times 11
10.2.1.3. Number of samples for analysis 11
10.2.2. Faeces 12
10.2.2.1. Method 12
10.2.2.2. Collection times 12
10.2.2.3. Number of samples for analysis 12
10.2.3. Cage Washing 12
10.2.3.1. Method 12
10.2.3.2. Collection times 12
10.2.3.3. Number of samples for analysis 12
10.2.4. Carcass 12
10.2.4.1. Method 13
10.2.4.2. Collection Time 13
10.2.4.3. Number of samples for analysis 13
10.3. LSC Analyses and Other Measurements 13
10.4. Data Acquisition and Processing 14
10.5. Sample Storage and Handling 14
11. REPORTING 14
11.1. Final Report 14
11.2. Corrections or Additions to the Final Report 14
12. QUALITY ASSURANCE 15
13. ARCHIVING 15
14. STUDY PERSONNEL 15
15. PROTOCOL DISTRIBUTION 16
APPENDICES
Appendix 1. 17

1. INTRODUCTION AND OBJECTIVES

FEXINIDAZOLE is a 5-nitroimidazole derivative biologically active against Trypanosoma parasites (*T.b. rhodesiense* and *T.b. brucei*) and useful in the treatment of the Human African Trypanosomiasis (HAT), known as sleeping sickness. FEXINIDAZOLE is a compound currently under development by the Study Sponsor.

The purpose of this Study is to obtain information on the elimination of radioactive drug-related material in urine and faeces following oral administration of [^{14}C]-FEXINIDAZOLE (800 mg/kg) to male Sprague Dawley rats.

2. STUDY SPONSOR

Sponsor Code at Accelera - Nerviano Medical Sciences S.r.l. is: 348 (see Appendix 1).

3. TEST FACILITY

Accelera

4. REGULATORY REQUIREMENTS

This study will be GLP regulated and will be conducted in compliance with:

- *DECRETO LEGISLATIVO 2 Marzo 2007, No. 50*
- *Organisation for Economic Co-operation and Development (OECD) Principles of Good Laboratory Practice (GLP) (as revised in 1997).*

The methods employed in this study are those described in the "Standard Operating Procedures" of the laboratories involved.

5. PROPOSED SCHEDULE

Experimental Start Date:	April 21, 2008 (animal dosing)
Experimental Completion Date:	April 25, 2008 (*in vivo* phase and sample collection completion)
Preliminary data available:	May 15, 2008
Unaudited Draft Report:	June 15, 2008
Final Report:	Within four weeks after receiving the Study Sponsor’s comments on the draft Report

6. STUDY DESIGN

6.1. General Description

A single Oral dose of [^{14}C]-FEXINIDAZOLE (800 mg/kg) will be administered to Sprague Dawley rats. The radioactivity dose administered will be approximately 3.7 MBq/kg, 100 μCi/kg.

The Excretory Balance will be evaluated in 3 male rats. The elimination of radioactive drug-related material will be determined in urine, faeces and cage washings collected up to 96 hours after administration from the animals. The carcasses will be analysed for residual radioactivity.

The radioactivity levels in the excreta and carcass will be determined by Liquid Scintillation Counting (LSC).

After the completion of all the analyses, the remaining urine and homogenate faeces samples will be retained at -80 °C for future fexinidazole and metabolite determinations. These investigations will be performed in agreement with the Study Sponsor and will be described by a separate Study Protocol.

6.2. Justifications

The albino Sprague Dawley rat has been chosen as the species for this study since it was one of the species used in the toxicological evaluation of the test compound.

Only male rats will be used as no gender differences are expected with respect to the aim of the study. The Oral route is chosen as expected clinical route of administration. The dose level has been chosen by the Study Sponsor and it is within the pharmacological relevant range. No toxicity is expected after single dosing at this dose level.

6.3. Dosing Schedule Design

During the course of the study each animal will receive a single oral dose of [^{14}C]-FEXINIDAZOLE as detailed in the following table:

**Starting Dose Date**	**Animal no.**	**Dose**	**Samples collected**
April 21, 2008	M01, M02, M03	800 mg/kg<br>100 μCi/kg<br>10 mL/kg	Urine, Faeces, Cage wash,<br>Carcass

7. TEST AND CONTROL ITEM

7.1. Test item

7.1.1. Radiolabelled Test Item Designation and Specifications

Compound:	[^{14}C]-FEXINIDAZOLE
Source:	[^{14}C]-FEXINIDAZOLE will be provided by Isotope Chemistry, Accelera, Nerviano Medical Sciences.<br>A Certificate of Analysis will accompany the radiolabelled test compound and will include data, chemical structure and labelling position, the radiochemical purity and the specific activity.
Batch:	Batch number of radiolabelled test compound will be included as raw data and reported in the Study Report.
Specific Activity:	56 mCi/mmol (radiochemical purity >98%).
Storage Conditions:	The radiolabelled test compound will be stored at –20 °C, in the dark.<br>The Study Sponsor will supply written instruction regarding disposal or return of unused test compound on completion of the study.

7.1.2. Unlabelled Test Item Designation and Specifications

Compound:	FEXINIDAZOLE
Chemical name:	1-methyl-2[[4-(methylthio)phenoxy]methyl]-5-nitro-imidazole
Molecular Formula:	$C_{12}H_{13}N_3O_3S$
MW:	279.31
Source:	FEXINIDAZOLE, from Centipharm, will be provided by the Study Sponsor.<br>A Certificate of Analysis, or other suitable documentation, will accompany the supplied test Items.
Batch:	3168-07-01/O, purity 100.2% (by HPCL).
Expire date:	October 2008.
Storage Conditions:	At room temperature.

Conditions:	The Study sponsor will supply written instruction regarding disposal or return of unused test compound on completion of the study.

7.1.3. Preparation of radiolabelled Test Item

The test compound will be prepared mixing in a glass container an appropriate amount of [^{14}C]-FEXINIDAZOLE (see section 7.1.1) and an appropriate amount of unlabelled FEXINIDAZOLE (see section 7.1.2), in order to obtain [^{14}C]-FEXINIDAZOLE at the final Specific Activity of 4.625 KBq/mg, 0.125 µCi/mg. The test compound will be stored at –20 °C, in the dark, until formulation preparation. Further details on the preparation of radiolabelled Test Item will be reported and described in the raw data.

7.2. Test Formulation

7.2.1. Preparation of dose vehicle

The test compound will be formulated in 5% Tween 80 and 0.5% Methyl cellulose 400 cP (Methocel) in water. This vehicle is stable for 1 months at +4 °C.

Details of the constituents used to prepare the vehicle are reported below:

Identification:	5% Tween 80 and 0.5% Methyl cellulose 400 cP (Methocel) in water.	
Source, Lot/Batch number:	Tween 80, Sigma-Aldrich	Lot No. 1324202
	Methyl cellulose 400 cP, Sigma-Aldrich	Lot No. 017K0087
Expiry:	Tween 80	February 2011
	Methyl cellulose 400 cP	January 2010
Storage conditions:	Room Temperature.	
Method of Preparation:	On file at Accelera/Experimental ADMET/Preclinical Formulation.	

7.2.2. Preparation of Radiolabelled dosing formulation

The test formulation will be prepared on the day of dosing.

The test formulation will be prepared dissolving an appropriate amount of [^{14}C]-FEXINIDAZOLE (see section 7.1.3) in the dose vehicle, in order to obtain the target concentration of approximately 80 mg/mL corresponding to a radioactivity concentration of about 370 kBq/mL (10 µCi/mL).
If necessary, after preparation the suspension obtained will be sonicated and then continuously mixed using a magnetic stirrer until the completion of dosing. Further details of formulation preparation will be reported and described in the raw data.

7.2.3. Determination of Concentration, Homogeneity and Stability

The radioactivity concentration and homogeneity of test formulation will be determined, before administration, by liquid scintillation counting of weighed aliquots of test formulation diluted, if appropriate, in a suitable volume of solvent. The test formulation will be also visually inspected before administration for any signs that may indicate a lack of homogeneity. The formulation will be continuously stirred before the phase of the dose administration.

The Study Sponsor will supply information about the stability of the formulated test compound. An aliquot of test formulation will be stored at –20 °C for possible subsequent analysis. Any residual test formulation remaining after dose administration will be retained and stored. The Study Sponsor will supply written instruction regarding disposal or return of unused test formulation on completion of the study.

7.2.4. Storage Conditions of Formulated Drug

The test formulation will be administered within 3 h following preparation. During the animal treatment the test formulation will be protected from light, as far as possible.

8. TEST SYSTEM

Species/Strain or Breed and Source:	Albino Sprague Dawley Rat, Charles River, Calco (CO) Italy.
Age at Dose initiation:	Adults (about 7-11 weeks).
Sex and Number of Animals:	3 Albino Male rats.
Weight at Dose initiation:	About 250-300 g.
Acclimation:	At least 5 days before the administration.
Selection Criteria:	Animals will be selected from a group of health male rats on the basis of their general condition and body weight.
Method of Animal and Cage Identification:	A color-coded cage card will be put to each animal cage, indicating the study number, the compound name, the animal number and gender, the route of administration, the dose level and the date of administration.

8.1. Clinical and physical examinations

Survival and Clinical Sign Observations:	Behavioral changes of the animals will be recorded upon evidence of side effects due to the treatment.
Body Weights:	Before dose administration.
Food Consumption:	Daily during the test period. Only reduced food intake will be recorded.

8.2. Fate of animals at the end of the study

All animals will be sacrificed at the end of the study.

9. ENVIRONMENTAL

9.1. Location of Study

The study will be conducted within the facilities of Accelera, Nerviano Medical Sciences.

9.2. Environmental Conditions

Housing and Caging	Animals will be housed at Accelera, Building 58B.<br>During the predosing period the animals will be housed in polypropylene and stainless steel cages with wood shavings as bedding (JRS, Germany). Certificates of analysis from the manufacturer will be obtained and retained with the study data.<br>During the experimental period the rats will be individually housed in glass metabolism cages specially designed for the separate collection of faeces and urine.
Temperature	21.5 +/- 1.5 °C
Humidity	55% +/- 15%
Air Changes	Approximately 15/hour.
Lighting	Approximately 12-hour light, 12-hour dark cycle.
Diet	The administered diet will be Mucedola 4RF21, available *ad libitum* after test compound administration. Certificates of analysis from the manufacturer of the diet batches will be obtained and retained with the study data.
Fasting	Animals will be fasted overnight before dosing. On the day of dosing the food will be offered about 2-3 hours post dosing.
Water	From municipal mains, available *ad libitum* via water bottles attached to the cage.

Actual conditions are continuously monitored and recorded and the records are retained. If transient major changes occur, additional records will be filed. The release of each lot of feed by the manufacturers is based on analysis of composite samples of each lot, which has met specifications set by the manufacturers. The water is periodically analyzed for chemical and microbial impurities. No contaminants have been identified in the food or water, which are expected to interfere with the results of this study.

All the above environmental conditions, as well as all the procedures adopted throughout the study for housing and handling the animals are in strict compliance with EEC and Italian Guidelines for Laboratory Animal Welfare.

10. EXPERIMENTAL

10.1. Method of Dose Administration

[^{14}C]-FEXINIDAZOLE will be orally administered by gastric gavage at the target dose level of 800 mg/kg and at the target dose volume of about 10 mL/kg. The radioactivity dose will be about 3.7 MBq/kg, 100 μCi/kg.

Individual doses will be prepared for each animal loading the appropriate amount of the test formulation in a syringe.

The weight of the empty apparatus (including gavage tube), of the apparatus containing the dose of test compound before administration (pre-dose) and of the apparatus after administration of the test compound (post-dose) will be determined. The dose administered to each animal will be determined from the weight of dose formulation administered, calculated by the difference of the weights of pre-dose and post-dose apparatus, and from the concentration of test material in the dose formulation, calculated from the measured radioactivity content of the formulation and from the specific activity of the test material.

10.2. Sample Collection, Processing and Schedule

10.2.1. Urine

Urine will be collected from each animal.

10.2.1.1. Method

Urine will be collected into pre-weighed ice cooled containers protected from light. The weight of the samples will be recorded. Two aliquots of urine (about 0.25 mL) will be transferred into a scintillation vial for the determination of the radioactivity content. The remaining amount of urine will be frozen and will be stored at –80 °C.

10.2.1.2. Collection times

Urine will be collected from each animal before administration (pre-dose sample) and over 0-8, 8-24, 24-48, 48-72 and 72-96 hours post dose.

10.2.1.3. Number of samples for analysis

15 urine samples will be collected for the determination of the radioactivity content.

10.2.2. Faeces

Faeces will be collected from each animal.

10.2.2.1. Method

Faeces will be will be collected into pre-weighed ice cooled containers protected from light. At the end of each period of collection the weight of faeces will be recorded. The samples will be stored at –80 °C until analysis. Faeces will be homogenised (see section 10.3) and duplicate aliquots (ca 0.5 g) of each homogenate will be used for the determination of the radioactivity content. The remaining amount of the homogenates will be stored at –80 °C.

10.2.2.2. Collection times

Faeces will be collected from each animal before administration (pre-dose sample) and over 0-8, 8-24, 24-48, 48-72 and 72-96 hours post dose.

10.2.2.3. Number of samples for analysis

15 faeces samples will be collected for the determination of the radioactivity.

10.2.3. Cage Washing

Cage washings will be collected from each metabolism cage.

10.2.3.1. Method

A suitable amount of water (approximately 100 mL) will be used to wash the metabolism cages and will be collected into pre-weighed containers. At the end of each washing the weight of cage washings will be recorded. Two aliquots (about 1 mL) of each washing sample will be used for the determination of the radioactivity content. The remaining amount of cage washing samples will be stored at +4°C and will be discarded when the excretory balance study will be completed, assuming that minimal amounts of radioactivity are contained in each sample.

10.2.3.2. Collection times

Cage washing will be performed daily (time intervals of 24 hours) until 96 hours post dose.

10.2.3.3. Number of samples for analysis

12 cage washing samples will be collected for the determination of the radioactivity.

10.2.4. Carcass

The carcass of each animal will be retained.

10.2.4.1. Method

The animals will be sacrificed after the last collection time and the skin will be separated from the carcass; the skins and the carcasses will be weighed and will be then stored at –20 °C. Skins and the carcasses will be homogenised (see section 10.3).

10.2.4.2. Collection Time

The carcass of each animal will be collected after sacrifice at 96 hours post dose.

10.2.4.3. Number of samples for analysis

3 carcasses of rat will be collected for the determination of the radioactivity content.

10.3. LSC Analyses and Other Measurements

Radioactivity in the formulation and in the biological samples will be determined by liquid scintillation counting (LSC) using Packard equipment (1900CA, 1900TR).

Counting efficiencies will be calculated by the external standard method using a series of quenched standards supplied by Packard, in order to generate the calibration curves. The validity of calibration curves will be checked before analysis. Where appropriate, background disintegration rates will be measured in pre-dose samples or in control samples. The background disintegration rate will be measured and subtracted from the sample disintegration rate. The limit of detection of radioactivity will be defined as twice the background level.

Concentration of radioactivity in urine (duplicate aliquots, about 0.25 mL) and cage washing (duplicate aliquots, about 1 mL) will be measured by mixing the samples with a suitable scintillation cocktail before the LSC. If necessary, a dilution of an aliquot of the sample, using an appropriate solvent, will be performed.

Faeces samples will be homogenised using a Stomacher apparatus after dilution with an appropriate volume of water (approximately 1:1 w/v). The radioactivity concentration in the homogenised faecal samples will be measured by LSC after the combustion of duplicate weighed aliquots (about 0.5 g) of sample.

The carcasses will be homogenized using an appropriate blender apparatus. The radioactivity concentration in samples of homogenised carcass will be measured by LSC after the combustion of triplicate weighed aliquots of sample (about 0.5 g).

The skin will be lyophilized and weighed. The skin will be then homogenized using an appropriate blender apparatus. The radioactivity concentration in samples of lyophilized and homogenised skin will be measured by LSC after the combustion of triplicate weighed aliquots of sample (about 0.5 g).

The combustion of the samples will be performed using a Packard Automatic Sample Preparation System 387 (Oximate 80 and Oxidizer 307). $^{14}CO_2$ generated from the

combustion of each sample will be collected in a suitable absorbent scintillation system. The efficiency of the oxidation system will be determined by combustion of quality control standards.

If the variation between replicated analyses will be inappropriately considerable, if the case, a further analysis will be carried out and the concentration of radioactivity will be calculated as the mean of the values of all the analyses.

10.4. Data Acquisition and Processing

The weights of the animals, the weights of the samples and the radioactivity disintegration rates will be captured, as far as possible, directly from the output of analytical balances and from the output of liquid scintillation counters into a validated Laboratory Information Management System (DEBRA v.5.4, by LabLogic, UK) to be processed.
The collected raw data will be used to determine the administered dose, the excretion balance of radioactivity (total amount of radioactivity in urine, faeces, cage washings and carcasses) and the total recoveries (expressed as % of dose).

10.5. Sample Storage and Handling

The carcasses of the animals and the skin will be stored at -20 °C. All the other biological samples generated in the course of the study will be stored at -80 °C, except the aliquots used for analysis.

After the completion of all the analyses, the remaining urine and homogenate faeces samples will be retained at -80 °C for future fexinidazole and metabolite determinations to be performed in a separate Study Protocol.

11. REPORTING

Any unexpected findings occurring during the course of the study will be immediately reported to the Sponsor's representative. Any changes or revisions of this protocol will be documented as amendment or will be recorded as raw data and documented as deviation (reported in the Study Report).

11.1. Final Report

A draft Report containing all information and data, as required by current internationally recognised regulations, will be submitted to the Sponsor for review. Following receipt of the Sponsor's comments, a final Report will be issued. One copy of the final Report, with original or scanned signatures, will be dispatched to the Sponsor.

11.2. Corrections or Additions to the Final Report

Corrections or additions to the approved (i.e. signed) version of the final Report will be issued as amendment to the Report by the Study Director.

12. QUALITY ASSURANCE

This study will be subjected to the following Quality Assurance procedures:
- protocol inspection;
- study based inspections on experimental phase and/or other routine inspections of procedural nature (Process inspections) on activities not directly related to the study;
- report revision to assure that the methods adopted were in compliance with the Standard Operating Procedures and that the results accurately reflect the raw data.

All raw data and QA documentation pertaining to the study will be available for inspection by the Sponsor's representative and Regulatory Authorities (following authorization from the Sponsor).

13. ARCHIVING

The original Protocol, all Protocol amendments, all raw data and supporting documents produced at the Test Facility, and the original final Report will be filed in the Archives of Accelera, Nerviano Medical Sciences S.r.l., Nerviano (Italy) for the period of time agreed with the Sponsor (at least 3 years) after which the Sponsor will be contacted for instructions regarding dispatch or disposal of the material. A reserve sample of the test item and all the relevant original documentation will be filed by the Sponsor.

14. STUDY PERSONNEL

Other personnel from the laboratories involved will participate in the study as appropriate.

15. PROTOCOL DISTRIBUTION

Two copies of the Protocol with original signatures will be prepared: one will be retained by the Sponsor, the other one will be filed in the Accelera Archive. Copies of the original Protocol will be distributed to the Study Director and to QA. Encrypted copies of the protocol will be distributed to the personnel involved in the study.

Amendment 1

Fexinidazole: Determination of Excretion Balance following Single Oral Administration of [^{14}C]-Fexinidazole to Rats.

Product Name:	FEXINIDAZOLE
Study Number:	0162-2008
Amendment Number:	1
Study Director:	
Sponsor Reference Study No.:	N.A.

1. SPECIFIC CHANGE(S)

1.1. Description(s) of Change(s)

The animal number M02 has been excluded from the study.
The animal number M04 will substitute this subject.

For organizational reasons, the animal M04 will be dosed using the same [^{14}C]-FEXINIDAZOLE formulation that will be prepared for the study 0510-2007 (Fexinidazole: Determination of Tissue Distribution by Whole Body Autoradiography following Single Oral Administration of [^{14}C]-Fexinidazole to Rats) during the animal dosing experimental session. The administered dose will be 800 mg/kg, 100 μCi/kg, dose volume of 10 mL/kg, as per protocol.

All the experimental activities for M04 subject will be performed as described in the Study Protocol.

1.1.1. Effective Date: June 6, 2008

1.1.2. Reason(s) for Change(s)

Because of a low radioactivity levels recovered from faeces samples of the animal M02, due to a loss of part of samples, it has been deemed necessary to replace the rat M2. As a consequence the animal M02 are removed from the study and samples will be discharged. Data collected will be archived, however they won't be included or discussed in the Study Report.

Appendix 2. Analytical Bulletin

Appendix 3. Raw Data Report of Individual Samples

Subject	Sample	Time	Sample (g)	Aliquot weight (g)	DPM	DPM/g	Mean DPM/g	Conc (mg/kg)	Reco (%)	Reco SD
M01	URINE	8 h	3.3111	0.2525	225351.744	892482.155	889411.721	3205.087	4.991	0.024
				0.2548	225839.760	886341.286				
		24 h	4.4348	0.2631	544779.619	2070618.089	2071405.928	7464.526	15.569	0.008
				0.2584	535454.869	2072193.766				
		48 h	10.8161	0.2581	108111.041	418872.689	418804.283	1509.205	7.677	0.002
				0.2615	109499.432	418735.876				
		72 h	13.4486	0.2582	11614.466	44982.440	44964.823	162.035	1.025	0.001
				0.2558	11497.495	44947.206				
		96 h	14.8469	0.2606	3617.774	13882.478	13867.515	49.973	0.349	0.001
				0.2600	3601.664	13852.552				
M03	URINE	8 h	2.1946	0.2582	308305.807	1194058.120	1194608.782	4304.897	4.338	0.003
				0.2575	307753.557	1195159.443				
		24 h	5.6802	0.2562	461577.432	1801629.320	1802201.345	6494.419	16.939	0.008
				0.2573	463853.588	1802773.370				
		48 h	13.6395	0.2568	74703.932	290903.161	291196.692	1049.357	6.572	0.009
				0.2582	75262.776	291490.223				
		72 h	16.5644	0.2598	10241.706	39421.502	39439.564	142.125	1.081	0.001
				0.2579	10176.122	39457.627				
		96 h	15.3394	0.2580	2708.728	10498.945	10472.079	37.737	0.266	0.001
				0.2624	2740.824	10445.213				

Subject	Sample	Time	Sample (g)	Aliquot weight (g)	DPM	DPM/g	Mean DPM/g	Conc (mg/kg)	Reco (%)	Reco SD
M04	URINE	8 h	4.0746	0.2494	204245.149	818946.067	820723.241	2957.561	6.236	0.019
				0.2466	202828.602	822500.415				
		24 h	5.5459	0.2546	374202.884	1469767.807	1469997.177	5297.287	15.202	0.003
				0.2544	374025.634	1470226.547				
		48 h	14.0501	0.2589	78194.985	302027.753	302392.303	1089.702	7.922	0.014
				0.2614	79140.641	302756.853				
		72 h	30.7746	0.2553	6195.966	24269.352	24096.647	86.835	1.383	0.014
				0.2580	6172.377	23923.941				
		96 h	13.8251	0.2513	2103.519	8370.549	8390.439	30.236	0.216	0.001
				0.2525	2123.608	8410.330				

Subject	Sample	Time	Sample weight (g)	Homogenate weight (g)	Aliquot weight (g)	DPM	DPM/g	Mean DPM/g	Conc * (mg/kg)	Reco (%)	Reco SD
M01	FAECES	8 h	NS	NS	NS	N.A.		N.A.	N.A.	N.A.	N.A.
					NS	N.A.					
		24 h	4.8677	10.7745	0.515	613561.555	1191381.660	1221809.279	9745.716	22.311	0.786
					0.477	597066.553	1252236.899				
		48 h	15.0501	29.9856	0.453	198809.977	439067.971	434215.492	3117.566	22.067	0.349
					0.490	210430.812	429363.012				
		72 h	21.9380	42.1067	0.404	30307.694	74963.379	80836.793	559.114	5.769	0.315
					0.431	36875.651	85598.075				
					0.487	39928.017	81920.430				
					0.487	39413.741	80865.288				
		96 h	18.5373	37.7648	0.474	3817.758	8047.550	8152.824	59.853	0.522	0.010
					0.508	4195.940	8258.099				
M03	FAECES	8 h	NS	NS	NS	N.A.		N.A.	N.A.	N.A.	N.A.
					NS	N.A.					
		24 h	6.6098	16.5103	0.5122	692166.042	1351358.925	1468246.098	13216.076	40.113	8.380
					0.4946	574505.530	1161555.864				
					0.4753	896398.862	1885964.364				
					0.4961	731303.608	1474105.238				
		48 h	20.4941	43.5792	0.5168	170899.275	330687.451	332745.477	2549.762	23.995	0.210
					0.4779	160002.594	334803.503				
		72 h	28.1919	58.9835	0.4564	15813.925	34649.265	34798.956	262.367	3.396	0.021
					0.4530	15831.737	34948.646				
		96 h	19.2218	40.7847	0.4692	2144.395	4570.323	4407.144	33.697	0.297	0.016
					0.4671	1982.356	4243.965				

*: concentration in fresh faeces

Subject	Sample	Time	Sample weight (g)	Homogenate weight (g)	Aliquot weight (g)	DPM	DPM/g	Mean DPM/g	Conc * (mg/kg)	Reco (%)	Reco SD
M04	FAECES	8 h	NS	NS	NS	N.A.		N.A.	N.A.	N.A.	N.A.
					NS	N.A.					
		24 h	4.8512	13.4821	0.4840	675276.548	1395199.480	1394279.120	10170.554	25.531	0.024
					0.5437	757569.158	1393358.760				
		48 h	16.9868	36.7190	0.4967	214203.844	431253.964	421328.219	2956.121	25.984	0.866
					0.5317	218742.695	411402.473				
		72 h	28.5504	58.2534	0.4956	27939.908	56375.924	56885.221	392.295	5.796	0.073
					0.4905	28152.011	57394.517				
		96 h	24.4470	52.2874	0.4616	2623.902	5684.363	5725.601	41.081	0.520	0.005
					0.4566	2633.139	5766.839				

*: concentration in fresh faeces

Subject	Sample	Time	Sample weight (g)	Aliquot weight (g)	DPM	DPM/g	Mean DPM/g	Conc (mg/kg)	Reco (%)	Reco SD
M01	CW	24 h	58.9984	0.9917	23784.337	23983.399	23956.999	86.332	2.395	0.004
				0.9809	23473.524	23930.599				
		48 h	72.3113	0.9809	5920.693	6035.980	6030.252	21.731	0.739	0.001
				0.9845	5931.143	6024.523				
		72 h	69.9966	0.9835	1582.845	1609.401	1598.241	5.759	0.190	0.002
				0.9822	1558.831	1587.081				
		96 h	76.0079	0.9866	768.217	778.651	774.630	2.791	0.100	0.001
				0.9984	769.376	770.609				
M03	CW	24 h	62.5742	0.9756	23986.673	24586.585	24546.935	88.457	2.542	0.006
				0.9820	24066.153	24507.284				
		48 h	65.7769	0.9766	5120.972	5243.674	5234.748	18.864	0.570	0.001
				0.9787	5114.512	5225.822				
		72 h	78.1173	0.9809	1024.158	1044.100	1051.425	3.789	0.136	0.001
				0.9850	1042.869	1058.750				
		96 h	79.3987	0.9894	561.210	567.222	565.896	2.039	0.074	0.000
				0.9734	549.552	564.569				
M04	CW	24 h	71.1695	0.9706	4440.085	4574.578	4559.535	16.431	0.605	0.003
				0.9812	4459.056	4544.493				
		48 h	90.8860	0.9742	4121.279	4230.423	4220.761	15.210	0.715	0.002
				0.9875	4158.460	4211.099				
		72 h	95.5720	0.9721	393.016	404.296	398.184	1.435	0.071	0.002
				0.9781	383.486	392.072				
		96 h	71.7092	0.9738	271.044	278.337	280.989	1.013	0.038	0.001
				0.9568	271.388	283.641				

Subject	Sample	Time	Sample weight (g)	Aliquot weight (g)	DPM	DPM/g	Mean DPM/g	Conc (mg/kg)	Reco (%)	Reco SD
M01	CARCASS	96 h	69.8714	0.2615	2088.430	7986.347	8025.352	28.920	0.950	0.034
				0.2689	2239.699	8329.114				
				0.2621	2034.052	7760.595				
M03	CARCASS	96 h	71.9516	0.2701	2204.657	8162.374	8210.191	29.586	0.978	0.016
				0.2620	2124.098	8107.246				
				0.2645	2211.472	8360.954				
M04	CARCASS	96 h	70.8484	0.2560	2192.178	8563.197	8432.319	30.387	1.114	0.015
				0.2625	2196.733	8368.505				
				0.2781	2326.378	8365.255				

Subject	Sample	Time	Homogenate weight (g)	Aliquot weight (g)	DPM	DPM/g	Mean DPM/g	Conc (mg/kg)	Reco (%)	Reco SD
M01	SKIN	96 h	21.6197	0.1865	1346.516	7219.925	7296.518	10.281	0.267	0.004
				0.2279	1692.888	7428.207				
				0.1851	1340.387	7241.421				
M03	SKIN	96 h	24.5742	0.2121	3264.329	15390.521	13755.553	19.460	0.559	0.067
				0.2183	3012.022	13797.625				
				0.2134	2577.555	12078.515				
M04	SKIN	96 h	22.7904	0.2696	3165.773	11742.482	10934.277	15.104	0.465	0.033
				0.2802	3051.339	10889.860				
				0.2752	2798.919	10170.490				
