# Supplementary material for: Fexinidazole – A New Oral Nitroimidazole Drug Candidate Entering Clinical Development for the Treatment of Sleeping Sickness
Source: PLoS Negl Trop Dis. 2010 Dec 21;4(12):e923. doi: 10.1371/journal.pntd.0000923 (PMC3006138; doi:10.1371/journal.pntd.0000923)
Supplement: Dataset S12 — (0.31 MB PDF) [file pntd.0000923.s013.pdf]

Fexinidazole, Fexinidazole Sulfoxide, Fexinidazole Sulfone: Effect on hERG Tail Current Recorded from Stably Transfected HEK 293 Cells

Product Name:	Fexinidazole, Fexinidazole Sulfoxide, Fexinidazole Sulfone
Study Number:	0507-2007
Study Director:	
Sponsor Reference Study No.:	Not Applicable
Status:	Final

SUMMARY

Fexinidazole is a 5-nitroimidazole derivate, biologically active against Trypanosoma parasites (T.b.rhodesiense and T.b. brucei), under investigation for the treatment of the Human African trypanosomiasis (HAT), known as sleeping sickness. The purpose of this study (0507-2007) was to assess the effect of Fexinidazole and two major metabolites (Fexinidazole Sulfoxide and Fexinidazole Sulfone), on hERG tail current.

HEK 293 cells stably transfected with hERG cDNA were used for patch clamp experiments in voltage clamp configuration. Onset and steady state blockade of the hERG channel current (I_{Kr}) due to the test items were monitored using a pulse pattern with fixed amplitudes (first depolarization: -50 mV for 500 ms; second depolarization: +20 mV for 2 seconds; hyperpolarization: -50 mV for 7 seconds). The current amplitude at the onset of the second step to –50 mV ("peak tail current") was monitored until a steady state was obtained in vehicle solution and then the test item was applied to the bath solution at 37°C. At the end the reference item was applied to block 100% of the current for determination of the current "leak" response to the stimulation protocol.

The effect of the test items was ascertained by calculating the residual current as percentage of control (% control). All data were corrected for "leak" current by subtracting the response in presence of 300 nM of the positive control E4031. Each test item was tested at three concentrations: 1, 5, and 30 µM. Exposure to Fexinidazole, Fexinidazole Sulfoxide and Fexinidazole Sulfone did not reduce hERG residual tail current at any of the doses tested. Only the highest dose of Fexinidazole Sulfone (30 µM, 9.34 µg/mL) showed a decrease on hERG peak tail current which was considered significant.

In conclusion, at all concentrations tested exposure to Fexinidazole and Fexinidazole Sulfoxide is judged not to affect hERG peak tail current. Fexinidazole Sulfone showed only at the highest dose (30 µM, 9.34 µg/mL) a significant decrease on hERG peak tail current. This concentration of Fexinidazole Sulfone is ten times higher than the IC_{50} for *in vitro* activity of the compoundagainst Trypanosoma species (2.66 µM, 0.83 µg/mL) [6].

TABLE OF CONTENTS

SUMMARY....................3
1. STUDY SPONSOR....................5
2. TESTING FACILITY....................5
3. INTRODUCTION AND OBJECTIVES....................5
4. MATERIALS AND METHODS....................5
4.1. Treatment Groups....................5
4.1.1. Dose Justification....................6
4.2. Test Items, Reference Item, Vehicle and Internal Solutions....................6
4.2.1. Storage and Formulation of Test Items....................6
4.2.2. Storage and Formulation of Reference Item....................6
4.2.3. Storage and Formulation of Vehicle Solution....................6
4.2.4. Storage and Formulation of Internal Solution....................7
4.3. Test System....................7
4.3.1. Source and Maintenance of hERG Transfected Cells....................7
4.3.2. Recording System....................7
4.4. Experimental Design....................8
4.5. Statistical Analysis....................8
4.6. Archiving....................8
5. RESULTS AND CONCLUSION....................9
6. REFERENCES....................9
7. TABLES AND FIGURES....................11

APPENDICES

Appendix 1. Study Data Listing

Appendix 2. Pharmacy Certification

1. STUDY SPONSOR

DNDi
1, Place St Gervais
CH-1201 Geneva
Switzerland

2. TESTING FACILITY

Accelera

3. INTRODUCTION AND OBJECTIVES

The delayed rectifier potassium current (I_{Kr}) plays a critical role in the control of action potential repolarization in many cell types. When expressed in HEK 293 (human embryonic kidney) cells, hERG (human ether-a-go-go-related gene) encodes the pore forming subunit of a K^+ channel with many characteristics of I_{Kr} [1-2]. During the cardiac action potential, as the cell begins to repolarize, potassium channels recover from inactivation, producing a large outward current, which promotes rapid repolarization. A decrease in I_{Kr} by class III antiarrhythmic drugs or by mutations in hERG can prolong cardiac repolarization, cause long QT syndrome and induce the arrhythmia “torsade de pointes” [3].
Fexinidazole is a 5-nitroimidazole derivate, biologically active against Trypanosoma parasites (T.b.rhodesiense and T.b. brucei), under investigation for the treatment of the Human African trypanosomiasis (HAT), known as sleeping sickness. The purpose of this study (0507-2007) was to assess the effect of Fexinidazole and two major metabolites (Fexinidazole Sulfoxide and Fexinidazole Sulfone), on hERG tail current from HEK 293 cells stably transfected with hERG cDNA.

This study was conducted according to the Standard Operating Procedures of Nerviano Medical Sciences and was conducted as a non-regulated study. The experiment was performed from 28 January 2008 to 04 February 2008 (last recordings).

4. MATERIALS AND METHODS

4.1. Treatment Groups

Groups of cells were treated with vehicle solution, with a single concentration of Fexinidazole, Fexinidazole Sulfoxide or Fexinidazole Sulfone and with the positive control E4031 (300 nM).

Test Group	Treatment	Concentration (µM)	Concentration (µg/mL)	Number of cells
1	Fexinidazole	1	0.28	3
2		5	1.40	4
3		30	8.38	3
4	Fexinidazole Sulfoxide	1	0.30	3
5		5	1.48	3
6		30	8.86	3
7	Fexinidazole Sulfone	1	0.31	3
8		5	1.56	3
9		30	9.34	3

4.1.1. Dose Justification

The concentrations of the test items were selected in agreement with the Sponsor based on the *in vitro* activity of Fexinidazole and its metabolites against Trypanosoma species, where the IC_{50} ranged from 1.14 µM to 4.26 µM. All the test items show similar *in vitro* activity [6].

E-4031 is a selective blocker of the hERG current [4] and was used as the reference item. In a previous study [5], the maximum concentration that completely blocks the hERG current was found to be 300 nM. At this concentration E4031 has sufficiently rapid kinetics to obtain recordings in a reasonable time frame.

4.2. Test Items, Reference Item, Vehicle and Internal Solutions

4.2.1. Storage and Formulation of Test Items

Fexinidazole, Fexinidazole Sulfoxide and Fexinidazole Sulfone (batches n° 3168-82-144, AAO1106-I-0170 and AAO1106-I-0171) were prepared as 30 mM stock solution in DMSO by Accelera/Preclinical Formulation and stored at room temperature until use. On the day of use the stock solution was serially diluted using Tyrode solution to achieve the final perfusion concentrations.

4.2.2. Storage and Formulation of Reference Item

E4031 (batch n° 083k0579, Sigma) was supplied as a white crystalline powder, stored at –20°C. Stock solution of E4031 (100 µM in reverse osmosis water) was stored frozen at –20°C until the day of use. On the day of use the stock solution was added to the vehicle solution to give the final perfusion concentration.

4.2.3. Storage and Formulation of Vehicle Solution

Vehicle was a Tyrode solution plus 0.1% of DMSO (equivalent to the highest test item concentration studied) that was prepared without DMSO by Accelera/Preclinical

Formulation and stored at 2-4°C when not in use. When in use Tyrode solution was stored at room temperature. The composition of the Tyrode solution was (in mM): 154 NaCl, 4 KCl, 2 $CaCl_2$, 1 $MgCl_2$, 5.5 glucose and 5 HEPES. During the patch-clamp experiments the Tyrode solution was pumped into the recording chamber at approximately 2 mL/min at 37°C.

4.2.4. Storage and Formulation of Internal Solution

The internal solution was stored frozen at – 20°C until the day of use. The composition of the internal solution was (in mM): 110 K^+- aspartate, 23 KCl, 3 $MgCl_2$, 1 EGTA, 5 HEPES, 0.4 GTP (Na^+ salt), 5 ATP (Na^+ salt), 5 creatine phosphate, 0.4 $CaCl_2$ (pH 7.3 with KOH).

4.3. Test System

4.3.1. Source and Maintenance of hERG Transfected Cells

HEK 293 cells stably transfected with hERG cDNA, supplied by bSys GmbH Switzerland, were incubated at 37°C in a humidified atmosphere with 5% CO2. The cells were continuously maintained and passaged in sterile culture flasks containing a mixture of Dulbecco’s modified Eagle’s medium and nutrient mixture F-12 supplemented with 10% foetal bovine serum, 0.9% Penicillin/Streptomycin solution, Hygromycin B and Blasticidin. HEK 293 cells were passaged at a confluence of about 80%. For electrophysiology studies, the cells were seeded in Petri dishes (35x10 mm), with culture medium containing tetracycline to activate channel expression, at a density that enables isolated cells to be selected for patch-clamping. The dishes were stored in a humidified, gassed (5% CO2) incubator at 37°C.

4.3.2. Recording System

Petri dishes containing the cells to be used for electrophysiological studies were mounted on the stage of an inverted microscope and were superfused with Tyrode solution. Membrane currents were recorded (Axopatch 200B, Axon Instruments) in the whole cell configuration by using suction pipettes (Harvard apparatus, 1.5 mm o.d. x 0.86 mm i.d.) with tip resistance in the range of 2.5-3.5 MΩ. Gigaohm seals were formed between the patch electrodes and individual cells. If the quality of the seal was judged to be poor, the electrode was replaced and the process repeated with a different cell. Once a stable patch was achieved, recording was commenced in voltage-clamp mode, with the cell initially clamped at –80 mV. Series resistance and capacitance were compensated to about 70% of their initial value. Cells were perfused through a microperfusion system allowing for solution changes to occur within approximately one second. Computer software (pClamp version 8.0; Axon Instruments) was used to generate voltage clamp protocols, acquire data and analyze current traces. The data were stored on a computer hard disk for later analysis. Experiments were performed at a temperature in the recording chamber of 37 ± 1°C, which was maintained with a temperature controller.

4.4. Experimental Design

Cells stably expressing hERG were held at –80 mV. Onset and steady state block of the hERG channel current (I_{Kr}) due to the test items were monitored using a pulse pattern with fixed amplitudes (first depolarization: -50 mV for 500 ms; second depolarization: +20 mV for 2 seconds; hyperpolarization: -50 mV for 7 seconds). This pulse pattern was repeated every 15 seconds, and current amplitude at the onset of the second step to –50 mV ("peak tail current") was monitored until a steady state was obtained. Recordings were acquired at the sampling interval of 2 kHz and low-pass filtered at 1 kHz. The steady state peak tail current was monitored and recorded in vehicle solution and then the test item was applied to the bath solution. At the end the reference item E4031 was applied to block 100% of the current for determination of the current "leak" response to the stimulation protocol. The current amplitude was continually monitored through the onset of the voltage step to –50 mV until a new steady state level of current was achieved. Each cell was exposed to a single test substance concentration.

4.5. Statistical Analysis

For each cell the effect of the test items was ascertained by calculating the residual current as percentage of control (% control). All data were corrected for "leak" current by subtracting the response in presence of 300 nM E4031. Each value represents the average current recorded from 3 sequential voltage pulses. Data from individual cells was collected and the corresponding mean values and standard errors of the mean (SEM) were calculated and entered into an Excel spreadsheet. Statistical comparisons were made by Student's *t* test with $P < 0.05$ considered significant.

4.6. Archiving

The following material was filed in the archives of Accelera, Nerviano Medical Sciences S.r.l.:

- the original protocol
- the original data (study notebook, printouts, etc.)
- the documentation on the test items and raw data concerning the preparation of the test formulations, including notebooks and printouts
- the final report with original signatures

The archived material will be kept for the period of time agreed with the Sponsor (at least 3 years) after which the Sponsor will be contacted for instructions regarding dispatch or disposal of the material.

A copy of the protocol with original signatures were filed by the Sponsor.

5. RESULTS AND CONCLUSION

The results of this experiment are expressed as mean ± standard error and are reported in Table 1 and in Figures 1-6. Individual data are reported in Appendix 1.

Exposure to Fexinidazole, Fexinidazole Sulfoxide and Fexinidazole Sulfone did not reduce hERG residual tail current at any of the concentrations tested. Only the highest of the 3 doses tested of Fexinidazole Sulfone (30 μM, 9.34 μg/mL) showed a statistically significant decrease on hERG peak tail current. The following table shows the residual currents (as % of control ± standard error of the mean) for each test item:

Treatment	Concentration (μM)	Concentration (μg/mL)	Residual Current
Fexinidazole	1	0.28	100.12±4.29
	5	1.40	94.86±1.06
	30	8.38	88.97±8.21
Fexinidazole Sulfoxide	1	0.30	87.06±0.92
	5	1.48	101.39±8.64
	30	8.86	98.04±1.52
Fexinidazole Sulfone	1	0.31	96.16±8.15
	5	1.56	90.76±3.84
	30	9.34	67.41±2.24

In conclusion, at all concentrations tested, exposure to Fexinidazole and Fexinidazole Sulfoxide is judged not to affect hERG peak tail current. Fexinidazole Sulfone, only at the highest dose tested (30 μM, 9.34 μg/mL) significantly decreased the hERG peak tail current. This inhibitory concentration is ten times higher the IC_{50} for the *in vitro* activity of Fexinidazole Sulfone against Trypanosoma species (2.66 μM, 0.83 μg/mL) [6].

6. REFERENCES

1. Sanguinetti MC, Jiang C, Curran ME, Keating MT. A mechanistic link between an inherited and acquired cardiac arrhythmia: HERG encodes the Ikr potassium channel. Cell 1995; 81(2):299-307.

2. Sanguinetti MC, Jurkiewicz NK. Two components of cardiac delayed rectifier K^+ current. Differential sensitivity to block by class III antiarrhythmic agents. J Gen Physiol 1990; 96(1):195-215.

3. Redfen WS, Carlsson L, Davis AS, Lynch WG, MacKenzie I, Palethorpe S, Siegl PKS, Strang I, Sullivan AT, Wallis R, Camm AJ, Hammond TG. Relationships between preclinical cardiac electrophysiology, clinical QT interval prolongation and torsade de

pointes for a broad range of drugs: evidence for a provisional safety margin in drug development. Cardiovasc Res 2003; 58:32-45.

4. Zhou Z, Gong Q, Ye B, Makielski JC, Robertson GA, January CT. Properties of HERG channels stably expressed in HEK 293 cells studied at physiological temperature. Biophys J 1998; 74: 230-241.

5. Study 0247-2004: Effect of E4031 on hERG tail current recorded at 37°C and at room temperature from stably transfected HEK 293 cells.

6. Torreele E, Bourdin B. Fexinidazole Synopsis of experimental and preclinical studies. DNDi, 2007 Apr.

7. TABLES AND FIGURES

Table 1: Effect of the Test Items on hERG Tail Current

EFFECT OF FEXINIDAZOLE ON hERG TAIL CURRENT

Treatment		n	tail current (% cont)	
(μM)	(μg/mL)		MEAN	SEM
1	0.28	3	100.12	4.29
5	1.40	4	94.86	1.06
30	8.38	3	88.97	8.21

EFFECT OF FEXINIDAZOLE SULFOXIDE ON hERG TAIL CURRENT

Treatment		n	tail current (% cont)	
(μM)	(μg/mL)		MEAN	SEM
1	0.30	3	87.06	0.92
5	1.48	3	101.39	8.64
30	8.86	3	98.04	1.52

EFFECT OF FEXINIDAZOLE SULFONE ON hERG TAIL CURRENT

Treatment		n	tail current (% cont)	
(μM)	(μg/mL)		MEAN	SEM
1	0.31	3	96.16	8.15
5	1.56	3	90.76	3.84
30	9.34	3	67.41	2.24

Figure 1: Effect of Fexinidazole on hERG Tail Current

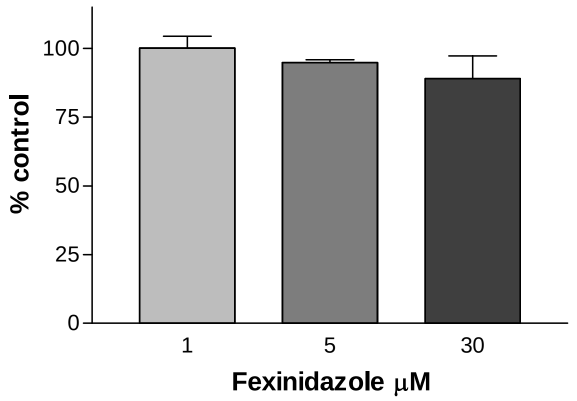

Figure 2: Effect of Fexinidazole Sulfoxide on hERG Tail Current

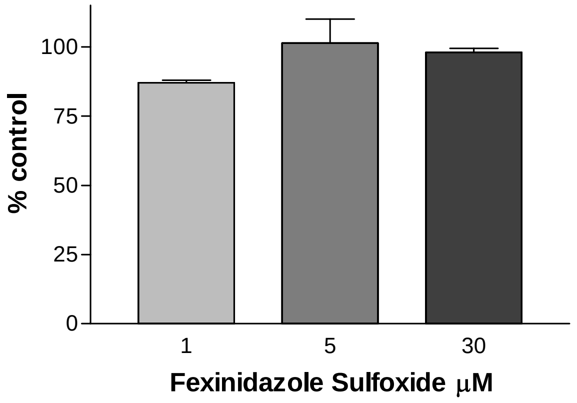

Figure 3: Effect of Fexinidazole Sulfone on hERG Tail Current

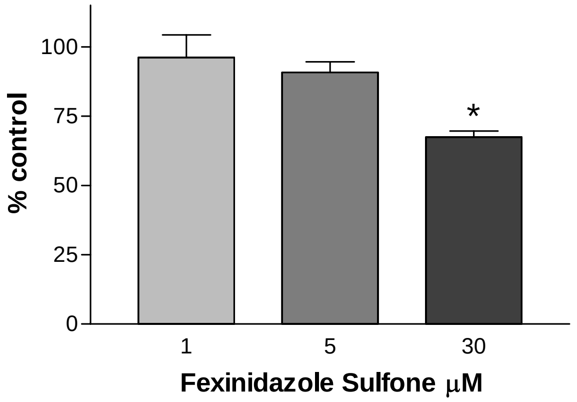

Figure 4: Examples of the Effect of Fexinidazole on hERG Tail Current

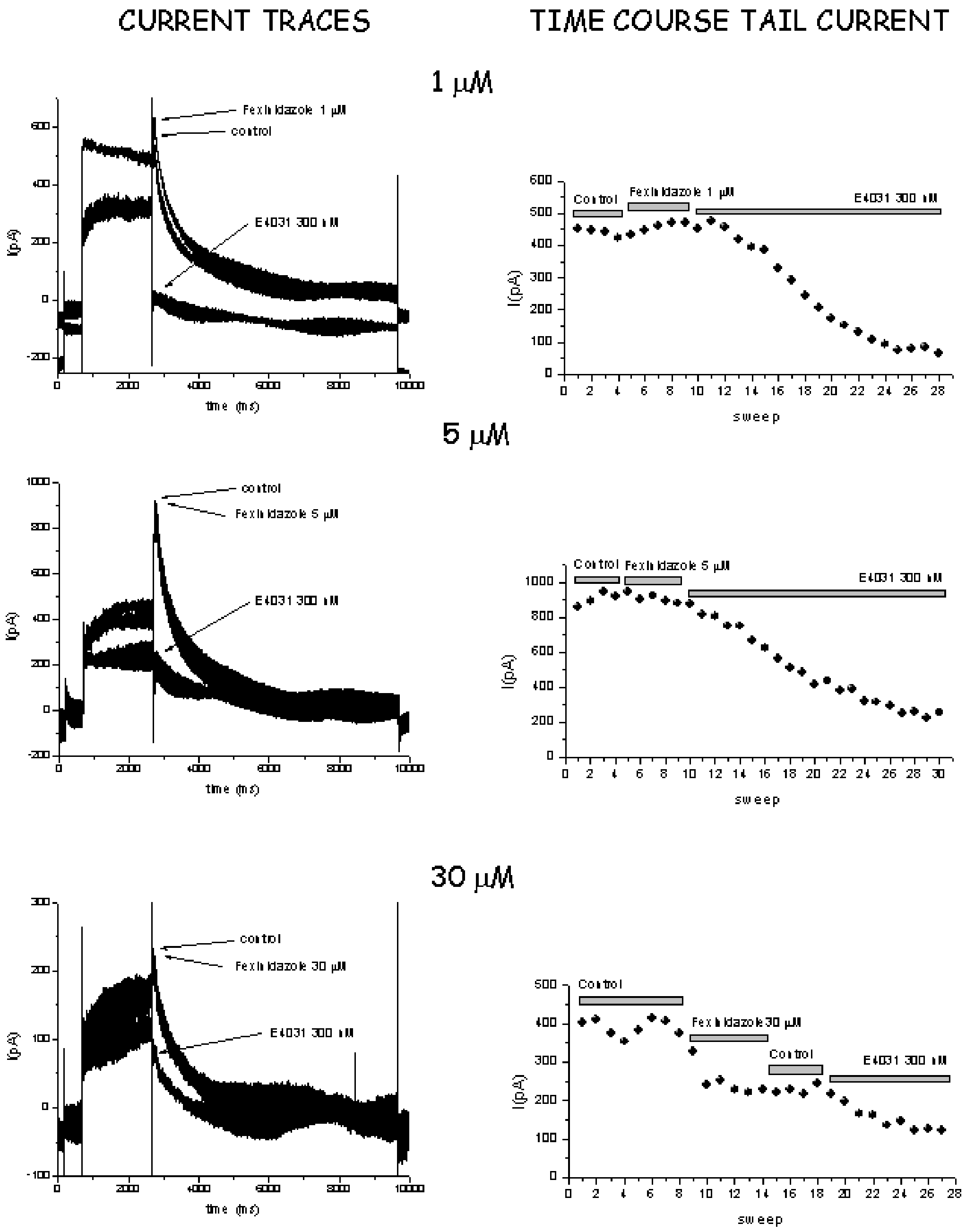

Figure 5: Examples of the Effect of Fexinidazole Sulfoxide on hERG Tail Current

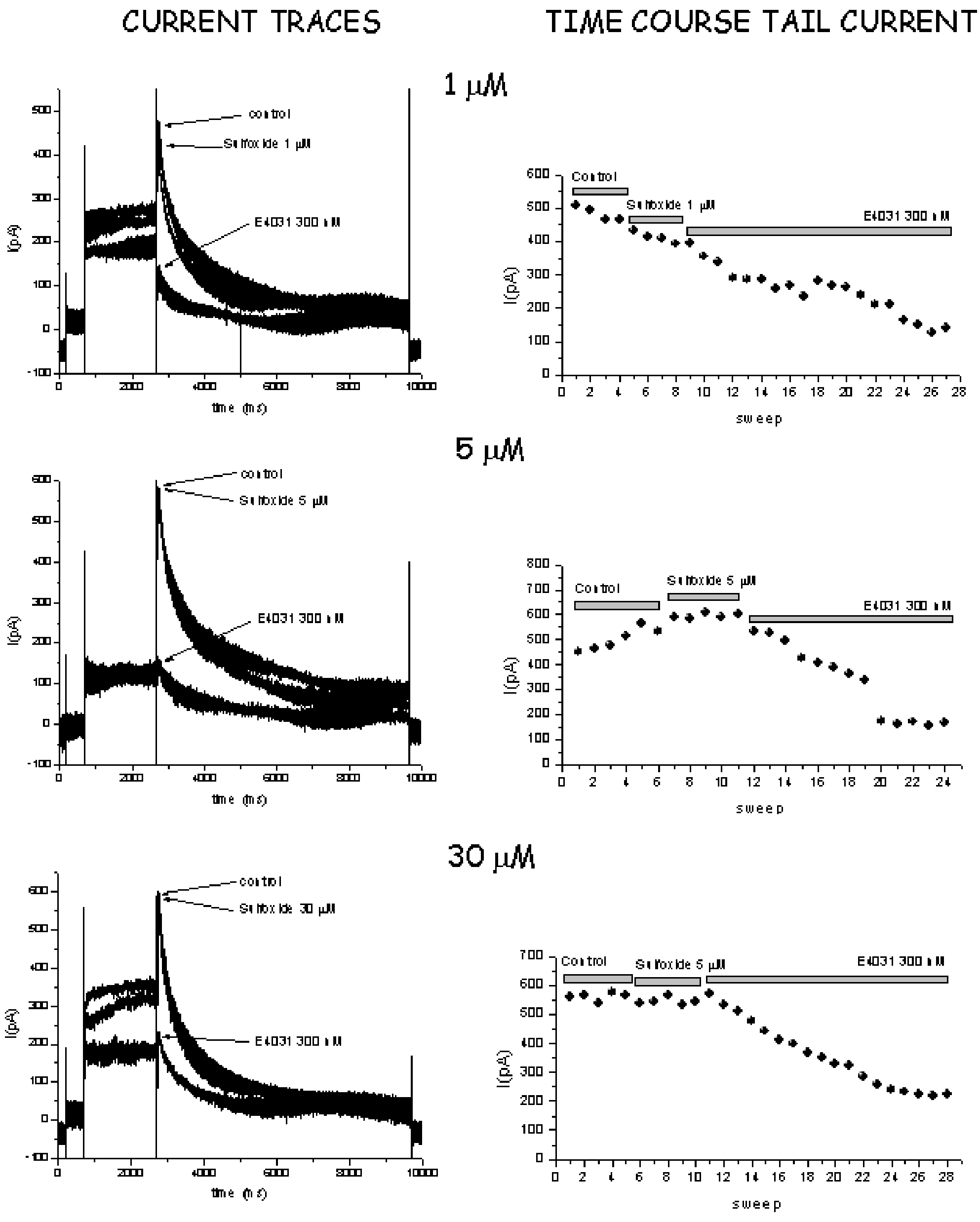

Figure 6: Examples of the Effect of Fexinidazole Sulfone on hERG Tail Current

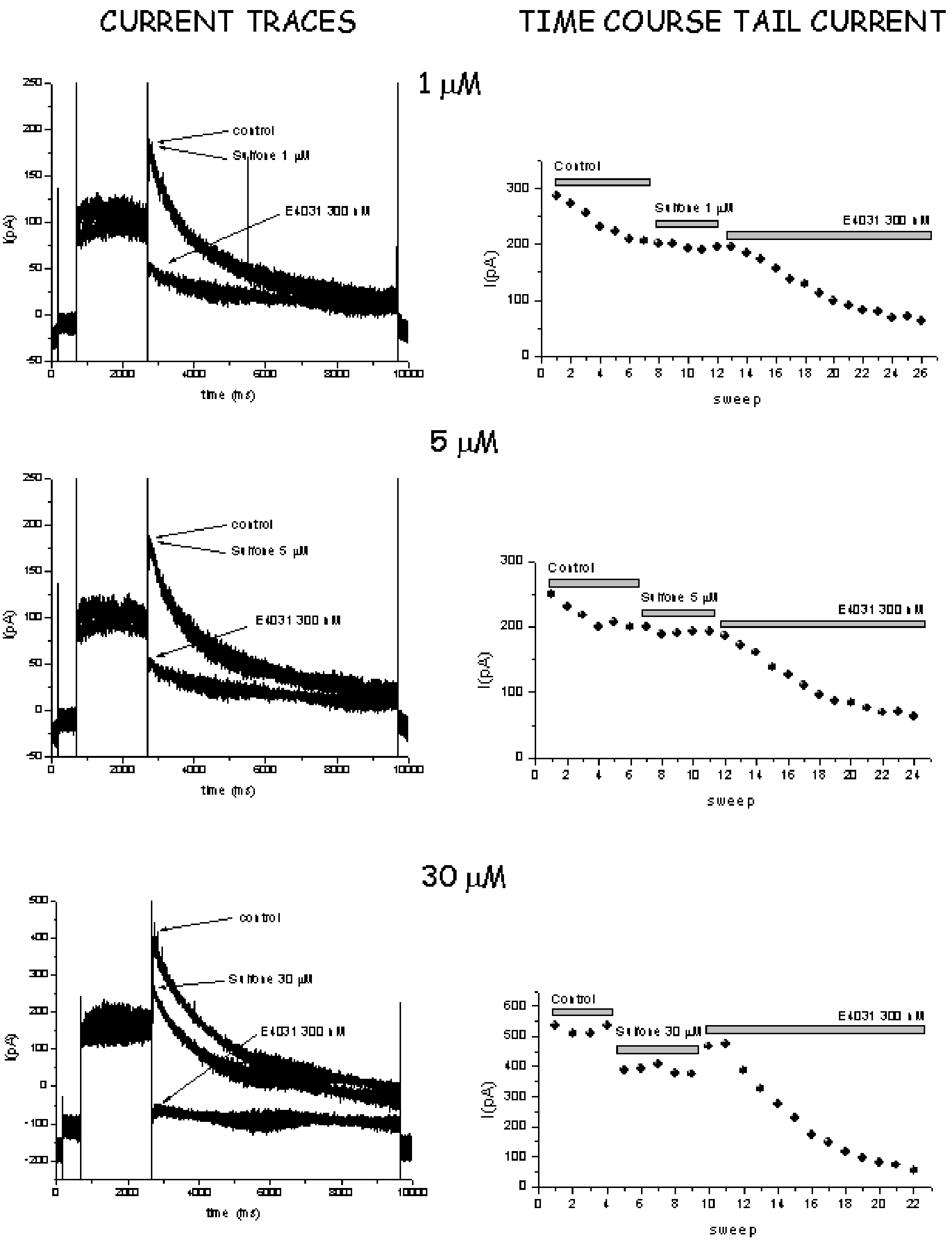

Fexinidazole, Fexinidazole Sulfoxide, Fexinidazole Sulfone: Effect On hERG Tail Current Recorded From Stably Transfected HEK 293 Cells

Study Number 0507-2007

Appendix 1 - Study Data Listing

Individual cell data for Fexinidazole

1 μM 0.28 μg/mL

EXP	CELL	CAP	CONT	E4031	DRUG	ICONT	IDRUG	%CON	**%CON**	I CONT	I DRUG	**%CON**
		pF	pA	pA	pA	pA	pA	DRUG	**E4031**	pA/pF	pA/pF	**DRUG**
28/01/08	2	34	1020.06	167.01	968.50	853.05	801.49	93.96	16.37	25.09	23.57	93.96
	3	53	1648.31	330.30	1622.42	1318.00	1292.11	98.04	20.04	24.87	24.38	98.04
	4	27	437.72	76.19	467.99	361.53	391.80	108.37	17.41	13.39	14.51	108.37
	M	38	1035.36	191.17	1019.64	844.20	828.47	100.12	17.94	21.12	20.82	**100.12**
	DS	13.45	605.44	128.77	578.91	478.30	450.77	7.43	1.89	6.69	5.48	7.43
	SE	7.767	349.55	74.34	334.23	276.15	260.25	4.29	1.09	3.86	3.16	**4.29**
	N	3	3	3	3	3	3	3	3	3	3	3
	P										0.9558	

5 μM 1.40 μg/mL

EXP	CELL	CAP	CONT	E4031	DRUG	ICONT	IDRUG	%CON	**%CON**	I CONT	I DRUG	**%CON**
		pF	pA	pA	pA	pA	pA	DRUG	**E4031**	pA/pF	pA/pF	**DRUG**
29/01/08	5	13	919.09	247.19	899.56	671.90	652.36	97.09	26.90	51.68	50.18	97.09
	6	33	902.15	387.27	867.51	514.88	480.25	93.27	42.93	15.60	14.55	93.27
	7	47	1675.47	505.47	1591.44	1169.99	1085.97	92.82	30.17	24.89	23.11	92.82
	8	44	902.76		868.84	902.76	868.84	96.24		20.52	19.75	96.24
	M	34.25	1099.87	379.98	1056.84	814.88	771.85	94.86	33.33	28.17	26.90	**94.86**
	DS	15.39	383.81	129.29	356.71	285.35	262.92	2.13	8.47	16.13	15.92	2.13
	SE	7.696	191.91	74.65	178.36	142.67	131.46	1.06	4.89	8.06	7.96	**1.06**
	N	4	4	3	4	4	4	4	3	4	4	4
	P										0.9139	

30 μM 8.38 μg/mL

EXP	CELL	CAP	CONT	E4031	DRUG	ICONT	IDRUG	%CON	**%CON**	I CONT	I DRUG	**%CON**
		pF	pA	pA	pA	pA	pA	DRUG	**E4031**	pA/pF	pA/pF	**DRUG**
29/01/08	9	14	229.80	123.70	226.39	106.10	102.69	96.79	53.83	7.58	7.34	96.79
	10	27	786.39		767.21	786.39	767.21	97.56		29.13	28.42	97.56
	11	19	350.63	108.50	284.18	242.13	175.68	72.56	30.94	12.74	9.25	72.56
	M	20	455.60	116.10	425.93	378.20	348.53	88.97	42.39	16.48	15.00	**88.97**
	DS	6.557	292.77	10.75	296.97	359.98	364.42	14.22	16.18	11.25	11.66	14.22
	SE	3.786	169.03	7.60	171.46	207.83	210.40	8.21	11.44	6.49	6.73	**8.21**
	N	3	3	2	3	3	3	3	2	3	3	3
	P										0.8817	

Individual cell data for Fexinidazole Sulfoxide

1 µM 0.30 µg/mL

EXP	CELL	CAP pF	CONT pA	E4031 pA	DRUG pA	ICONT pA	IDRUG pA	%CON DRUG	**%CON E4031**	I CONT pA/pF	I DRUG pA/pF	**%CON DRUG**
30/01/08	12	15	474.96		405.17	474.96	405.17	85.31		31.66	27.01	85.31
	13	33	506.29		447.54	506.29	447.54	88.40		15.34	13.56	88.40
	14	10	543.36		475.26	543.36	475.26	87.47		54.34	47.53	87.47
	M	19.33	508.20		442.66	508.20	442.66	87.06		33.78	29.37	**87.06**
	DS	12.1	34.24		35.30	34.24	35.30	1.59		19.58	17.10	1.59
	SE	6.984	19.77		20.38	19.77	20.38	0.92		11.31	9.88	**0.92**
	N	3	3		3	3	3	3		3	3	3
	P										0.7834	

5 µM 1.48 µg/mL

EXP	CELL	CAP pF	CONT pA	E4031 pA	DRUG pA	ICONT pA	IDRUG pA	%CON DRUG	**%CON E4031**	I CONT pA/pF	I DRUG pA/pF	**%CON DRUG**
30/01/08	15	13	507.81		443.06	507.81	443.06	87.25		39.06	34.08	87.25
	16	20	599.31	167.13	598.65	432.18	431.52	99.85	27.89	21.61	21.58	99.85
	17	22	537.21	164.90	600.74	372.31	435.84	117.06	30.69	16.92	19.81	117.06
	M	18.33	548.11	166.02	547.49	437.44	436.81	101.39	29.29	25.86	25.16	**101.39**
	DS	4.726	46.71	1.58	90.44	67.90	5.83	14.97	1.99	11.67	7.78	14.97
	SE	2.728	26.97	1.12	52.21	39.20	3.37	8.64	1.40	6.74	4.49	**8.64**
	N	3	3	2	3	3	3	3	2	3	3	3
	P										0.7868	

30 µM 8.86 µg/mL

EXP	CELL	CAP pF	CONT pA	E4031 pA	DRUG pA	ICONT pA	IDRUG pA	%CON DRUG	**%CON E4031**	I CONT pA/pF	I DRUG pA/pF	**%CON DRUG**
30/01/08	18	22	612.54	290.73	616.00	321.81	325.27	101.07	47.46	14.63	14.78	101.07
	19	17	560.35	224.15	549.01	336.20	324.86	96.63	40.00	19.78	19.11	96.63
	20	18	560.20	223.44	548.15	336.76	324.71	96.42	39.89	18.71	18.04	96.42
	M	19	577.70	246.11	571.05	331.59	324.94	98.04	42.45	17.70	17.31	**98.04**
	DS	2.646	30.17	38.65	38.93	8.48	0.29	2.63	4.34	2.72	2.25	2.63
	SE	1.528	17.42	22.31	22.47	4.89	0.17	1.52	2.51	1.57	1.30	**1.52**
	N	3	3	3	3	3	3	3	3	3	3	3
	P										0.2893	

Individual cell data for Fexinidazole Sulfone

1 μM 0.31 μg/mL

EXP	CELL	CAP	CONT	E4031	DRUG	ICONT	IDRUG	%CON	**%CON**	I CONT	I DRUG	**%CON**
		pF	pA	pA	pA	pA	pA	DRUG	**E4031**	pA/pF	pA/pF	**DRUG**
04/02/08	21	65	1168.62		1055.60	1168.62	1055.60	90.33		17.98	16.24	90.33
	22	18	213.56	68.22	193.05	145.35	124.84	85.89	31.94	8.07	6.94	85.89
	23	23	221.66	100.93	236.47	120.73	135.54	112.27	45.53	5.25	5.89	112.27
	M	35.33	534.61	84.57	495.04	478.23	438.66	96.16	38.74	10.43	9.69	**96.16**
	DS	25.81	549.08	23.13	485.94	598.02	534.31	14.12	9.61	6.68	5.70	14.12
	SE	14.9	317.01	16.36	280.56	345.27	308.49	8.15	6.80	3.86	3.29	**8.15**
	N	3	3	2	3	3	3	3	2	3	3	3
	P										0.407301	

5 μM 1.56 μg/mL

EXP	CELL	CAP	CONT	E4031	DRUG	ICONT	IDRUG	%CON	**%CON**	I CONT	I DRUG	**%CON**
		pF	pA	pA	pA	pA	pA	DRUG	**E4031**	pA/pF	pA/pF	**DRUG**
04/02/08	24	50	1077.83	258.59	944.21	819.24	685.63	83.69	23.99	16.38	13.71	83.69
	25	57	1066.74		1033.68	1066.74	1033.68	96.90		18.71	18.13	96.90
	26	21	202.96	67.55	191.71	135.42	124.17	91.69	33.28	6.45	5.91	91.69
	M	42.67	782.51	163.07	723.20	673.80	614.49	90.76	28.64	13.85	12.59	**90.76**
	DS	19.09	501.93	135.09	462.45	482.40	458.91	6.65	6.57	6.51	6.19	6.65
	SE	11.02	289.79	95.52	267.00	278.51	264.95	3.84	4.64	3.76	3.57	**3.84**
	N	3	3	2	3	3	3	3	2	3	3	3
	P										0.215134	

30 μM 9.34 μg/mL

EXP	CELL	CAP	CONT	E4031	DRUG	ICONT	IDRUG	%CON	**%CON**	I CONT	I DRUG	**%CON**
		pF	pA	pA	pA	pA	pA	DRUG	**E4031**	pA/pF	pA/pF	**DRUG**
04/02/08	27	18	516.93	69.42	386.13	447.51	316.71	70.77	13.43	24.86	17.60	70.77
	28	28	571.45	149.84	416.20	421.61	266.36	63.18	26.22	15.06	9.51	63.18
	29	20	527.71	75.01	384.11	452.70	309.10	68.28	14.21	22.63	15.46	68.28
	M	22	538.70	98.09	395.48	440.61	297.39	67.41	17.95	20.85	14.19	**67.41**
	DS	5.292	28.88	44.90	17.97	16.65	27.14	3.87	7.17	5.14	4.19	3.87
	SE	3.055	16.67	25.93	10.38	9.62	15.67	2.24	4.14	2.97	2.42	**2.24**
	N	3	3	3	3	3	3	3	3	3	3	3
	P										**0.006989**	

Fexinidazole, Fexinidazole Sulfoxide, Fexinidazole Sulfone: Effect On hERG Tail Current Recorded From Stably Transfected HEK 293 Cells

Study Number 0507-2007

Appendix 2 – Pharmacy Certification

Pharmacy Certification

STUDY NUMBER : **0507-2007**

TEST ITEM : **Fexinidazole – Fexinidazole-sulfoxide – Fexinidazole-sulfone**

NOTEBOOK NUMBER(S) : **19046/10-11-14-18 and 23**

MATERIALS USED FOR THE STUDY:

1. Fexinidazole test item, raw material, Lot No. 3168-82-144
2. Fexinidazole-sulfoxide test item, raw material, Lot No. AAO1106-I-0170
3. Fexinidazole-sulfone test item, raw material, Lot. No. AAO116-I-0171
4. Acqua per preparazioni iniettabili, raw material, Lot No. 07G0201
5. Sodium chloride, raw material, Lot No. 093K0110
6. Potassium chloride, raw material, Lot No. 046K0092
7. Calcium chloride dihydrate, raw material, Lot No. 125K0052
8. Magnesium chloride hexahydrate, raw material, Lot No. 013K0143
9. HEPES, raw material, Lot No. 025K5408
10. D-(+)-Glucose, raw material, Lot No. 044K0147
11. Sodium hydroxide puriss. (Ph.Eur.), raw material, Lot 60600
12. Dimethyl sulfoxide (DMSO), raw material, Lot No. 105K00431

PREPARATIONS:

Prepare solution of Fexinidazole test item, raw material, batch No. 3168-82-144 in DMSO at the concentration of 30 mM.

Prepare solution of Fexinidazole-sulfoxide test item, raw material, batch No. AAO1106-I-0170 in DMSO at the concentration of 30 mM.

Prepare solution of Fexinidazole-sulfone test item, raw material, batch No. AAO1106-I-0171 in DMSO at the concentration of 30 mM.

Prepare Tyrode solution in Sterile water for injection.

Pharm. Cert. 0507-2007

STABILITY:

Fexinidazole test item:

Expiry date October, 2008 for Fexinidazole test item, raw material, batch No. 3168-82-144 if stored at room temperature.

Fexinidazole-sulfoxide test item:

Expiry date for Fexinidazole-sulfoxide test item, raw material, batch No. AAO1106-I-0170 is not applicable therefore it is stored –20°C.

Fexinidazole-sulfone test item:

Expiry date for Fexinidazole-sulfone test item, raw material, batch No. AAO1106-I-0171 is not applicable therefore it is stored –20°C.

Fexinidazole solution:

The solutions are prepared just before dosing.

Fexinidazole-sulfoxide solution:

The solutions are prepared just before dosing.

Fexinidazole-sulfone solution:

The solutions are prepared just before dosing.

Prepared by: Date: February 18, 2008
