# Supplementary material for: Fexinidazole – A New Oral Nitroimidazole Drug Candidate Entering Clinical Development for the Treatment of Sleeping Sickness
Source: PLoS Negl Trop Dis. 2010 Dec 21;4(12):e923. doi: 10.1371/journal.pntd.0000923 (PMC3006138; doi:10.1371/journal.pntd.0000923)
Supplement: Dataset S13 — (0.66 MB PDF) [file pntd.0000923.s014.pdf]

Fexinidazole: Effects on Cardiovascular Parameters after Oral Administration to the Beagle Dog

Product Name:	Fexinidazole
Study Number:	0506-2007
Study Director/Author:	
Sponsor Reference Number:	Not Applicable
Status	Final

SUMMARY

Fexinidazole is a 5-nitroimidazole derivate, biologically active against Trypanosoma parasites (T.b.rhodesiense and T.b. brucei), under investigation for the treatment of the Human African trypanosomiasis (HAT), known as sleeping sickness. The purpose of this study (0506-2007) was to investigate the potential effects of the test item on cardiovascular parameters in the conscious beagle dog following a single oral administration.

Four male animals were used in this study. Each animal received a single oral administration of the vehicle and of fexinidazole at escalating doses of 100, 300 and 1000 mg/kg. The interval between each test item dose was 4 days.

Oral administration of fexinidazole at doses of 100, 300 and 1000 mg/kg to beagle dogs did not have any meaningful effect on blood pressure, heart rate and ECG intervals, including the QT interval. Body temperature was moderately increased in a dose-dependent manner from the dose of 300 mg/kg between 2 and 5 hours after administration.

In the conditions of this study, the no-observed-effect-level (NOEL) for fexinidazole on cardiovascular parameters and ECG intervals was 1000 mg/kg. The NOEL on body temperature was 100 mg/kg.

TABLE OF CONTENTS

QUALITY ASSURANCE STATEMENT 4
SUMMARY 5
1. INTRODUCTION AND OBJECTIVES 8
2. STUDY SPONSOR 8
3. TEST FACILITY 8
4. REGULATORY REQUIREMENTS 8
5. SCHEDULE 8
6. MATERIALS AND METHODS 9
6.1. Test and Control Items 9
6.1.1. Test Item 9
6.1.2. Vehicle/Control Item 9
6.1.3. Test Formulation 9
6.1.4. Test Formulation Analyses 9
6.1.5. Stability 9
6.2. Test System 10
6.2.1. Animals 10
6.2.2. Environmental Conditions 10
6.2.3. Instrumentation 10
6.3. Treatment and Experimental Design 11
6.3.1. Clinical Observation 11
6.3.2. Telemetry recordings 11
7. ARCHIVING 12
8. STUDY PERSONNEL 12
9. RESULTS AND DISCUSSION 12
9.1. Cardiovascular Parameters 12
9.1.1. Arterial Blood Pressure and Heart Rate 12
9.1.2. ECG 13
9.2. Body Temperature 13
10. CONCLUSIONS 13
11. REFERENCES 13
12. FIGURES 14

APPENDICES

Appendix 1. Study data listing

Appendix 1.1. Vehicle

Appendix 1.2. Fexinidazole, 100 mg/kg/day

Appendix 1.3. Fexinidazole, 300 mg/kg/day

Appendix 1.4. Fexinidazole, 1000 mg/kg/day

Appendix 2. Protocol and Amendments

Appendix 3. Pharmacy Certification

1. INTRODUCTION AND OBJECTIVES

Fexinidazole is a 5-nitroimidazole derivate, biologically active against Trypanosoma parasites (T.b.rhodesiense and T.b. brucei), under investigation for the treatment of the Human African trypanosomiasis (HAT), known as sleeping sickness. The purpose of this study (0506-2007) was to determine the potential toxicity of the test item on cardiovascular parameters in the conscious beagle dog following a single oral administration. Cardiovascular parameters were collected from chronically implanted animals (telemetric technique).

2. STUDY SPONSOR

Drugs for Neglected Diseases *initiative* (DND*i*)
1, Place St Gervais
CH-1201 Geneva
Switzerland

3. TEST FACILITY

Accelera

4. REGULATORY REQUIREMENTS

This study was conducted in compliance with:

- Decreto Legislativo 2 Marzo 2007, No. 50;
- Organisation for Economic Co-operation and Development (OECD) Principles of Good Laboratory Practice (GLP) (as revised in 1997).

The methods employed in this study were those described in the "Standard Operating Procedures" of the laboratories involved.

5. SCHEDULE

~~Experimental Start Date (weighing of animals)~~	~~21st March 2008~~
~~First treatment (vehicle)~~	~~25th March 2008~~
~~Last treatment~~	~~4th April 2008~~
~~Experimental Completion Date (end of last telemetry recording)~~	~~5th April 2008~~

6. MATERIALS AND METHODS

6.1. Test and Control Items

6.1.1. Test Item

Identification	Fexinidazole
Lot/Batch Number	3168-07-01/O
Purity	100.2%
Expiry	October 2008
Storage Conditions	Room temperature, protected from light
Source and Manufacturer	Centipharm (formerly Orgasynth, as communicated by the Sponsor)
Special Handling Precautions	Usual protection of all personnel conducting the study (mask, gloves and eyeglasses) or according to MSDS (Material Safety Data Sheet)

6.1.2. Vehicle/Control Item

Identification	5% Tween 80 in 0.5% Methyl cellulose 400 cP (Methocel)
Lot/Batch Number	Tween 80 1324202<br>Methyl cellulose 400 cP 125K0196
Expiry	Tween 80 February 2011<br>Methyl cellulose 400 cP January 2009
Storage Conditions	Room temperature
Source and Manufacturer	Tween 80 Sigma-Aldrich<br>Methyl cellulose 400 cP Sigma-Aldrich
Method of Preparation	On file at ADMET/Preclinical formulation

6.1.3. Test Formulation

Type of Formulation	Suspension in vehicle
Method of Preparation	On file at ADMET/Preclinical formulation
Frequency of Preparation	Suspensions will be prepared according to the stability data
Dose Concentrations	10, 30, 100 mg/mL
Storage Conditions	Room temperature in the dark
Source and Manufacturer	Accelera/Preclinical Formulation

6.1.4. Test Formulation Analyses

Samples (top-middle-bottom, 5 mL each) of each dose suspension were collected under stirring for concentration and homogeneity check test of fexinidazole; 10 mL were taken also from the vehicle. After collection, samples were directly transferred at +4°C to Bioanalysis & Analytical Control for analysis. The analyses were performed using a validated HPLC/UV method.

All values were found to be within acceptable limits.

6.1.5. Stability

Stability data indicate that fexinidazole suspensions in 5% Tween 80 in 0.5% methyl cellulose 400 cP (Methocel) in the range 0.5 - 100 mg/mL are stable up to 7 days at room temperature and 14 days at +4°C (NervianoMS 0293-2007-R).

6.2. Test System

6.2.1. Animals

Species/Strain and Source	Beagle dog, Marshall Europe
Justification of Species	The choice of species and strain (beagle dog) was based on the availability of extensive background data relating to the cardiovascular effects of drugs. Beagle dogs are commonly used as non-rodent species in safety studies. Moreover, the dog was used in the 4-week toxicity study and has a similar metabolism to man, being therefore exposed to the drug and its metabolites (Accelera study No. 0141-2007). Only males were used since no significant gender-related differences in systemic exposure were expected.
Age	Adult (> 1 year)
Weight	7.0 – 13.2 kg at the start of the study
Acclimation	At least 40 days
Selection Criteria	Acceptable ECG and BP telemetry signals (visual inspection).

6.2.2. Environmental Conditions

Caging	Solid floor pen (room No. 010, building 64)
Bedding	Sawdust (L.G. Packing Wood S.r.l., Condove, Turin)
Temperature	18°C to 21°C
Air changes	Approximately 20/hour
Humidity	40-70% UR
Lighting	Approximate 12-hour light, 12-hour dark cycle
Water	Free access to water from municipal main
Diet	300 g/day of a standard GLP pelleted diet (Altromin Rieper S.r.l., Vandoies, Bolzano, Italy)

All the above environmental conditions, as well as all the procedures adopted throughout the study for housing and handling the animals, were in strict compliance with EU and Italian Guidelines for Laboratory Animal Welfare.

6.2.3. Instrumentation

Physiological parameters were measured by means of a telemetry system (Data Science International, St. Paul, MN USA). In this study, telemetry transmitters (model TL11M2-D70-PCT) were implanted in the abdominal cavity and allowed recording of ECG (intracardiac lead), blood pressure (via abdominal aorta) and body temperature. Animals were surgically instrumented at least 3 weeks before the start of the study.

Prior to the start of the study, animals were checked for general health status and for the functionality of the radio-transmitters (readable and normal ECG and blood pressure waveforms).

At the end of the study, the dogs were returned to the pool of instrumented animals.

6.3. Treatment and Experimental Design

Experimental Day	Treatment[a]	Dose (mg/kg)	Volume (mL/kg)	Animal numbers (males)
25th March 2008	Vehicle	0	10	2057 - 2482 - 2552 - 2539
27th March 2008	Fexinidazole	100		
31st March 2008		300		
4th April 2008		1000		
a) Animals were treated following an escalating dose design with an adequate washout (at least 5 half lives of the longer lasting metabolite) between doses				

Doses of 100, 300 and 1000 mg/kg of fexinidazole were selected based on the results of a repeated (7-day) toxicity study in the same species (0339-2007). In this study, the top dose of 2000 mg/kg was well tolerated and did not cause any meaningful toxicological change. On the other hand, plasma levels determined as part of a 3-day toxicity study (study 0513-2007) showed that systemic exposure (both in terms of Cmax and AUC) did not increase when the dose was increased from 500 to 1000 mg/kg/day.

The oral route is that intended for clinical administration.

6.3.1. Clinical Observation

Animals were monitored via a CCTV system and physically inspected 24 hours post-dosing. Body weights are not reported here but they are archived with the study file.

6.3.2. Telemetry recordings

On the day of dosing, telemetry signals from each animal were collected continuously from at least one hour pre-dose to at least 24 hours after treatment.

The following parameters were calculated:

- Systolic blood pressure (SBP, mmHg)
- Diastolic blood pressure (DBP, mmHg)
- Average blood pressure (ABP, mmHg)
- Heart rate (HR, bpm, from the blood pressure signal)
- ECG intervals (RR, PR, QRS and QT, ms)
- Body temperature (BT, °C)

All the above-mentioned parameters were extracted automatically by the Notocord HEM acquisition software (Notocord, Croissy-sur-Seine, France) as the average value of 10-second recordings. Data were extracted every 10 minutes from 1 hour to 10 minutes before treatment, every 15 minutes from treatment to 4 hours later and every 30 minutes from 4 to 7 hours after treatment. Basal values (time “0”) are the mean of pre-treatment values (from 1 hour to 10 minutes before treatment). Data from 7 to 24 hours after treatment were not extracted, but they are archived with the study file.

ECG interval measurements were inspected on the computer screen for the correct placement of markers. QT intervals were corrected for heart rate using the covariate analysis formula {ln (QTca) = ln(QT) -(-0. 229314)*[ln(HR) - ln(HRm)], where HRm=100} [1, 2].

6.3.2.1. Deviations from Protocol

Data for animal No. 2482 at 60 minutes following vehicle administration are not available due to unreadable waveforms (probably caused by environmental interferences on the radio signal). This data point in Appendix 1 is reported as "-".

However, the lack of this single data point was deemed not to have any influence on the interpretation of the results of the study.

7. ARCHIVING

The original protocol and amendments, all raw data, supporting documents, and the final report with original signatures were filed in the Archives of Accelera, Nerviano Medical Sciences S.r.l., Nerviano (Italy), where they will be kept for the period of time agreed with the Sponsor (at least 3 years) after which the Sponsor will be contacted for instructions regarding dispatch or disposal of the material.

A copy of the protocol and amendments, the report with original signatures, a reserve sample and all relevant original documentation on the test item were filed by the Sponsor.

8. STUDY PERSONNEL

9. RESULTS AND DISCUSSION

Mean (± S.E.M.) absolute values and changes from pre-treatment (time 0) for cardiovascular parameters, ECG intervals and body temperature are given in Figures 1-10. Individual data are reported in Appendix 1.1-1.4.

9.1. Cardiovascular Parameters

9.1.1. Arterial Blood Pressure and Heart Rate

No meaningful changes were observed in systolic, diastolic and mean blood pressure following oral administration of fexinidazole up to the dose of 1000 mg/kg.

A moderate, transient increase in heart rate was observed from the dose of 300 mg/kg in the first 30 minutes following administration (peak increase of +40 beats/min at 300 mg/kg and +54 beats/min at 1000 mg/kg). Due to its moderate extent and short duration, this change is not deemed to have an important physiological significance.

9.1.2. ECG

The RR interval showed changes in accordance with the changes observed in heart rate: a moderate decrease at doses of 300 and 1000 mg/kg in the first 30 minutes following treatment.

No changes were observed in PR and QRS intervals.

The QT interval was slightly prolonged (about 40 msec) from 210 to 330 minutes following administration of the dose of 100 mg/kg, but no prolongation was observed following higher doses of 300 and 1000 mg/kg. The same prolongation was still present when the QT was corrected for the heart rate with the covariate formula. Because of the absence of effects at higher doses, the QT prolongation observed at the low dose of 100 mg/kg is deemed not to be a direct consequence of the treatment.

9.2. Body Temperature

Body temperature was moderately increased, in a dose-dependent manner, between 2 and 5 hours after treatment at doses of 300 and 1000 mg/kg (+0.6 and +1.0°C, respectively).

10. CONCLUSIONS

Oral administration of fexinidazole at doses of 100, 300 and 1000 mg/kg to beagle dogs did not have any meaningful effect on blood pressure, heart rate and ECG intervals, including the QT interval. Body temperature was moderately increased in a dose-dependent manner from the dose of 300 mg/kg between 2 and 5 hours after administration.

In the conditions of this study, the no-observed-effect-level (NOEL) for fexinidazole on cardiovascular parameters and ECG intervals is 1000 mg/kg. The NOEL on body temperature is 100 mg/kg.

11. REFERENCES

1. Nerviano Medical Sciences internal report 0081-2005-R “Sotalol: Evaluation of a positive control for QT prolongation in conscious beagle dogs”

2. Cooper M, Branch C, Bastianse R, Flegal M, Lancaster C, Packwood W, et al. Mathematical corrections for the inverse relationship between QT interval and heart rate in conscious beagle dogs and cynomolgus monkeys. Toxicol Sci. 2001; 60: A1251

12. FIGURES

Figure 1. Systolic arterial pressure

A. Absolute values

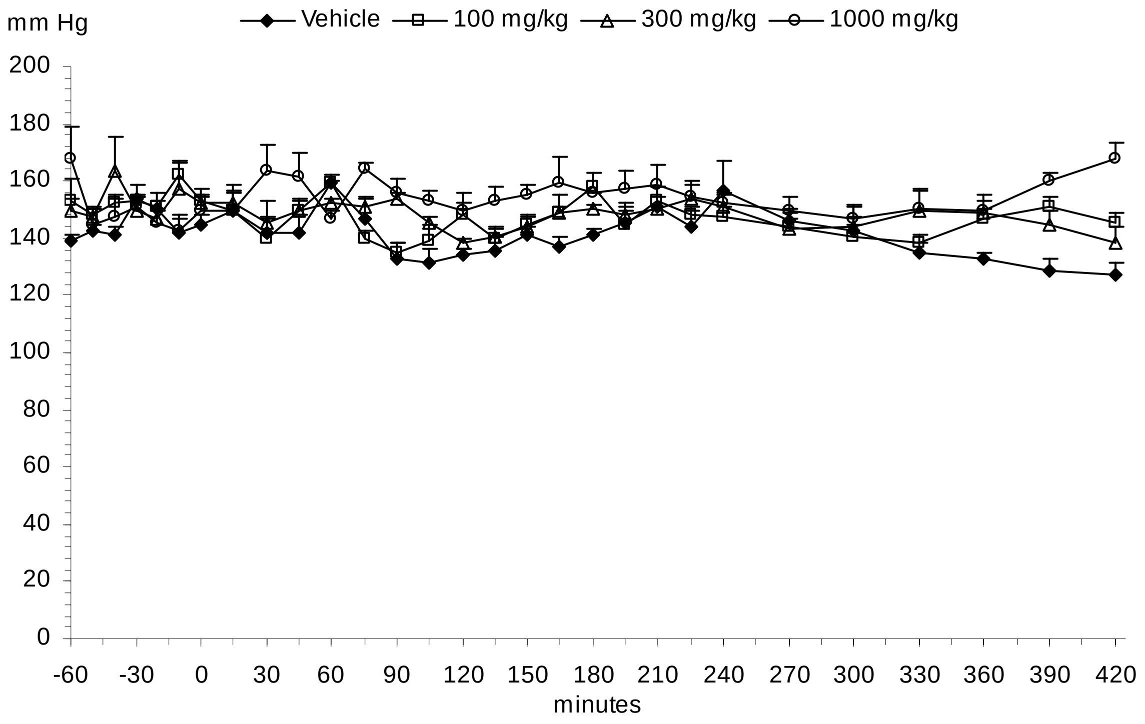

B. Changes from pre-treatment values

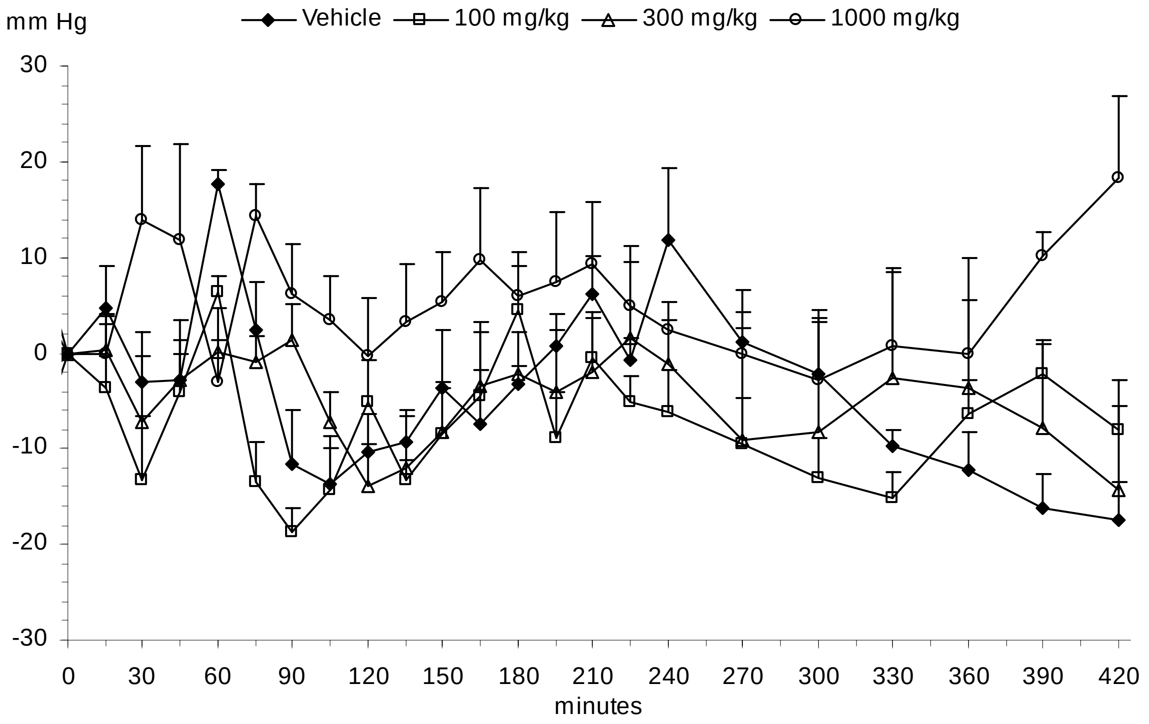

Figure 2. Diastolic arterial pressure

A. Absolute values

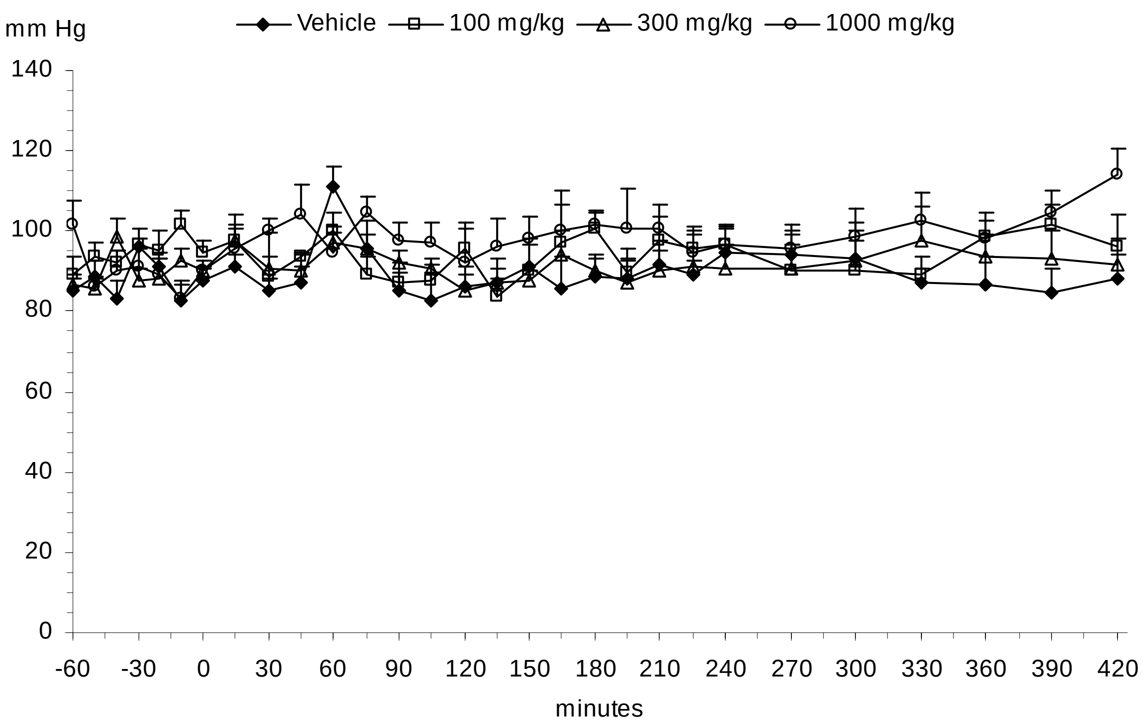

B. Changes from pre-treatment values

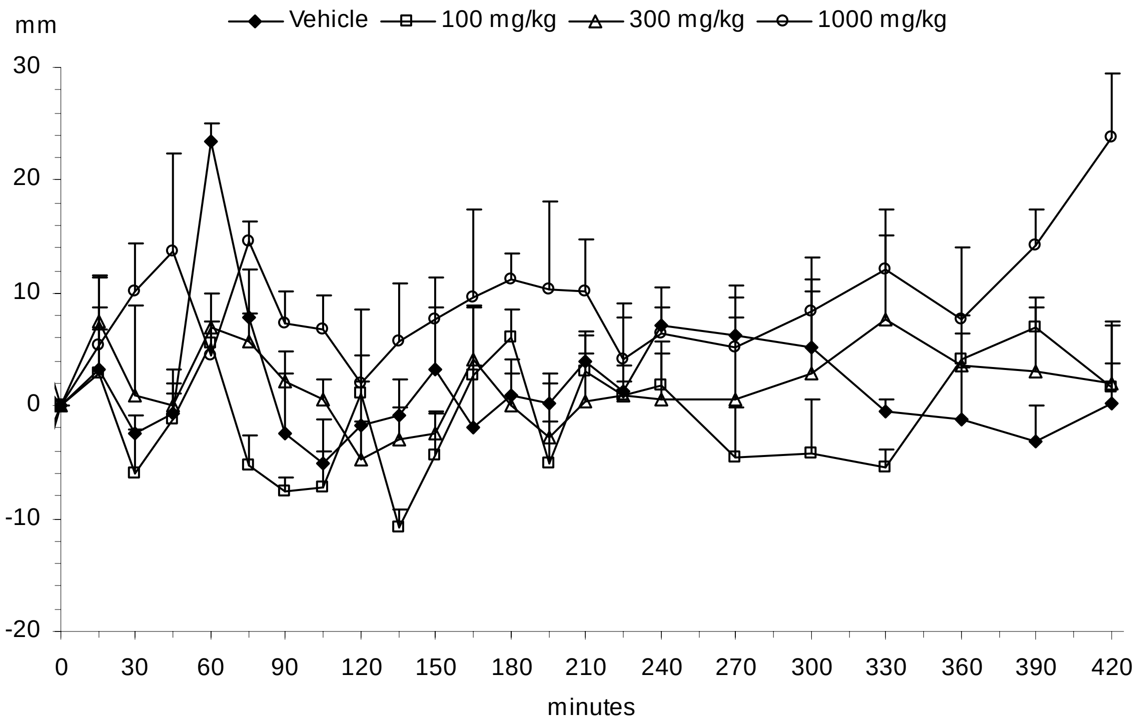

Figure 3. Mean arterial pressure

A. Absolute values

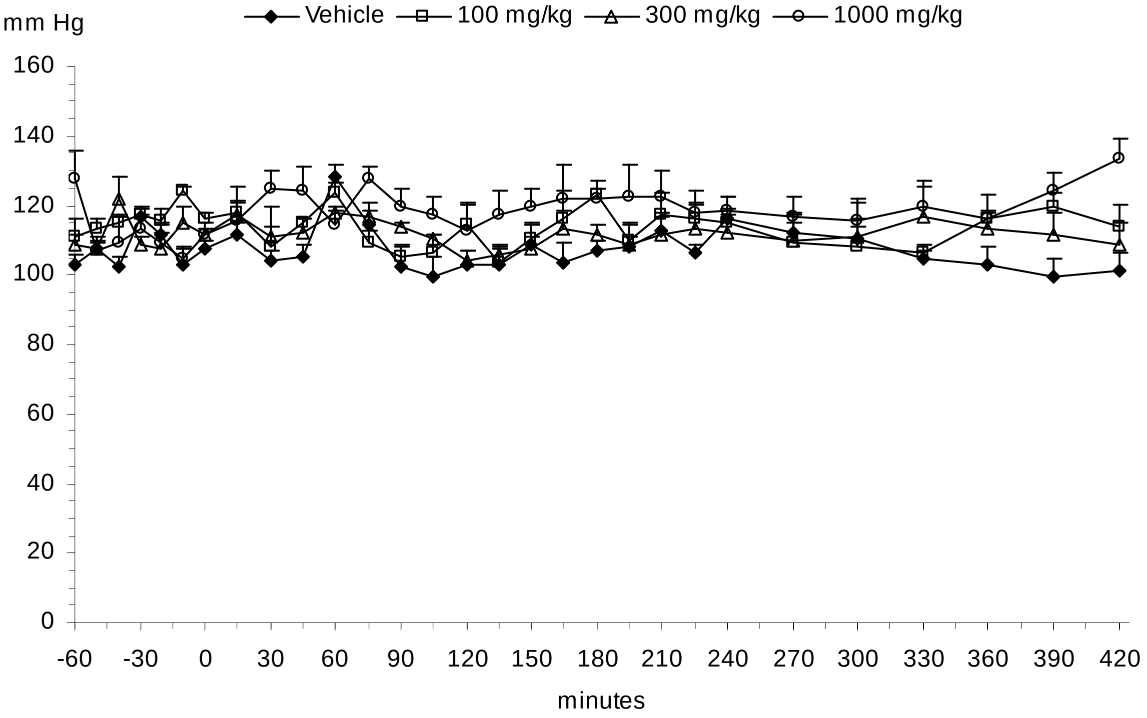

B. Changes from pre-treatment values

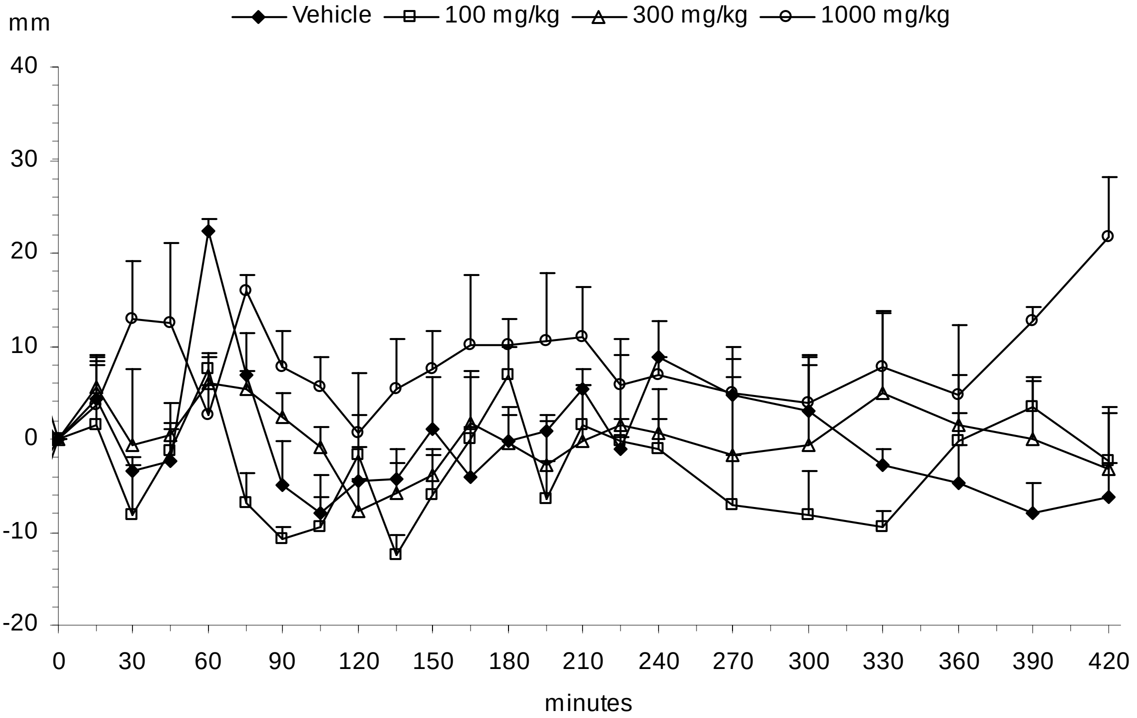

Figure 4. Heart rate

A. Absolute values

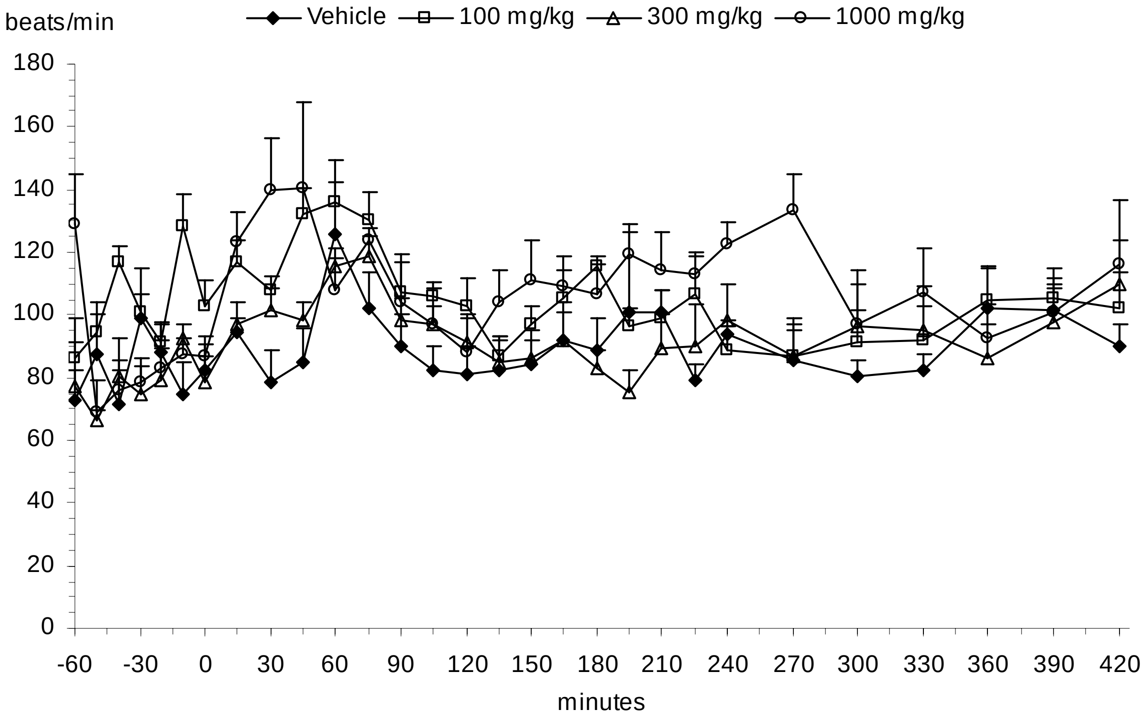

B. Changes from pre-treatment values

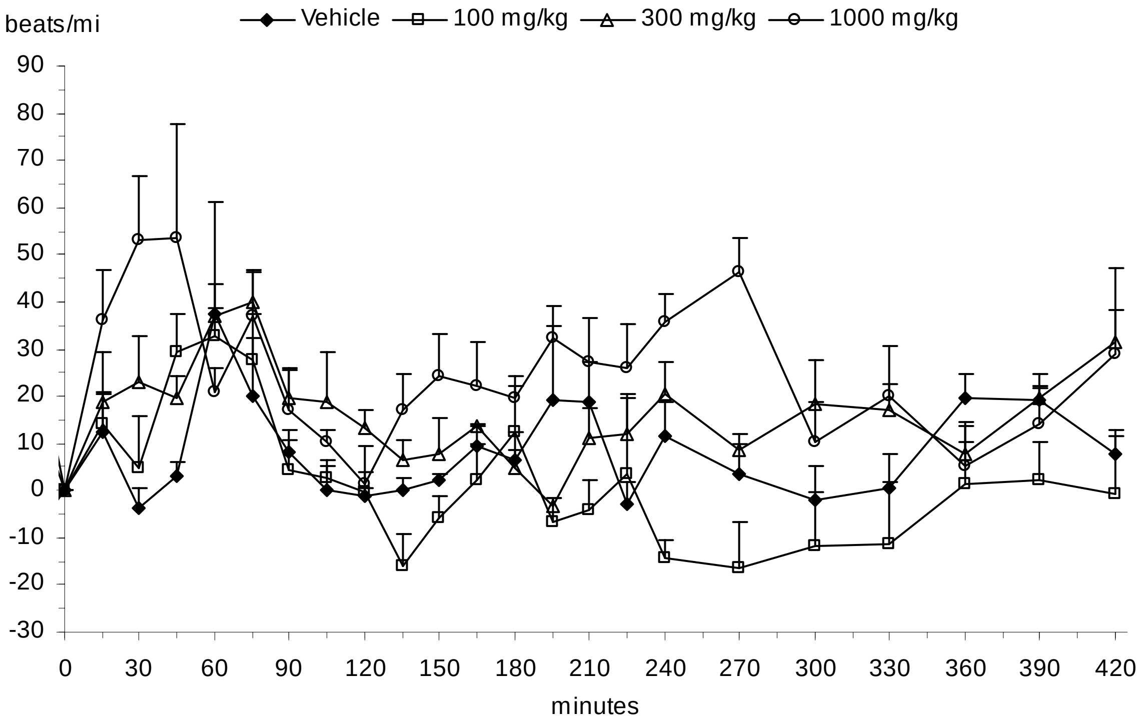

Figure 5. RR interval

A. Absolute values

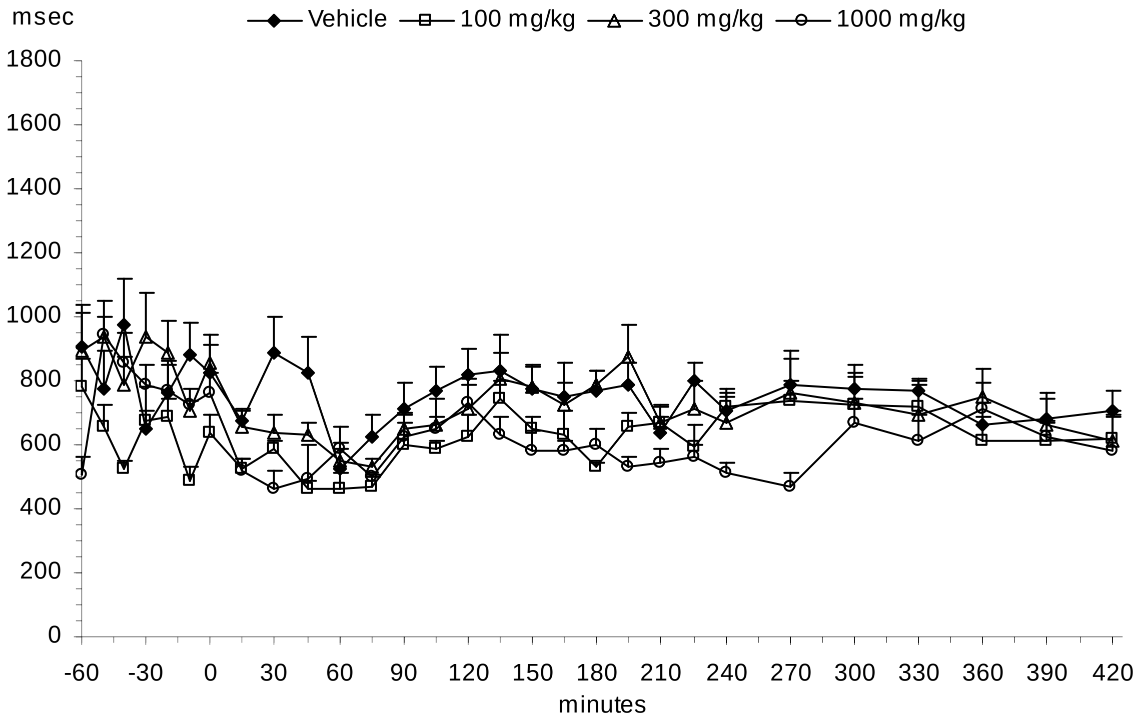

B. changes from pre-treatment values

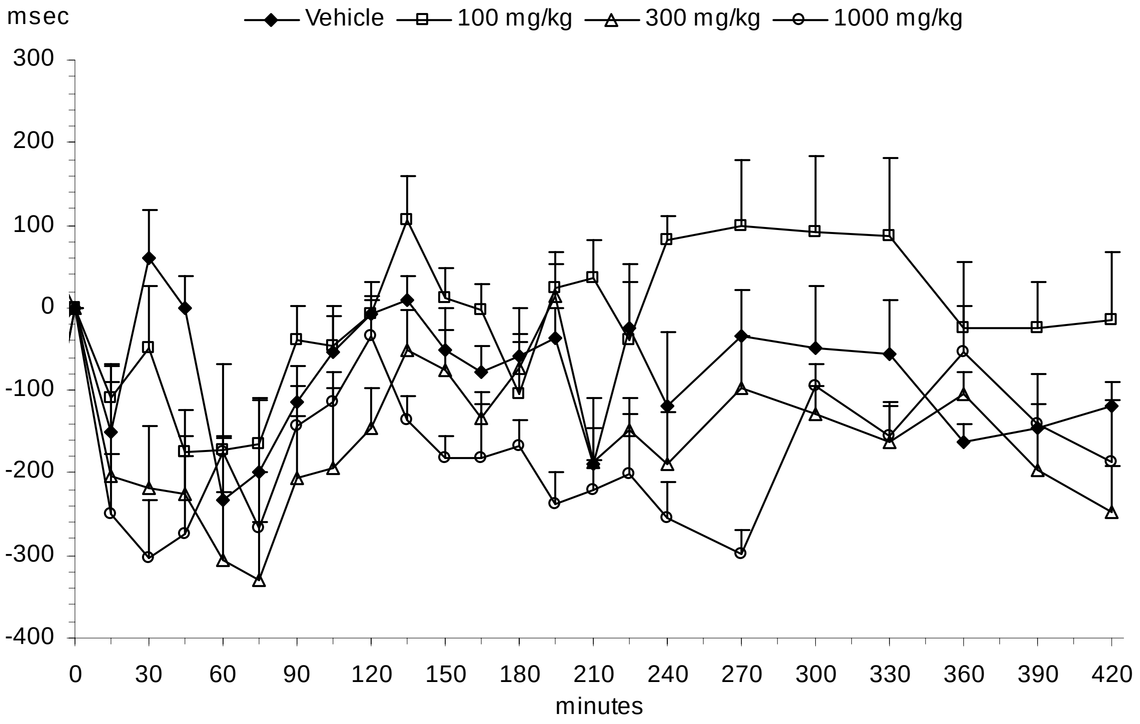

Figure 6. PR interval

A. Absolute values

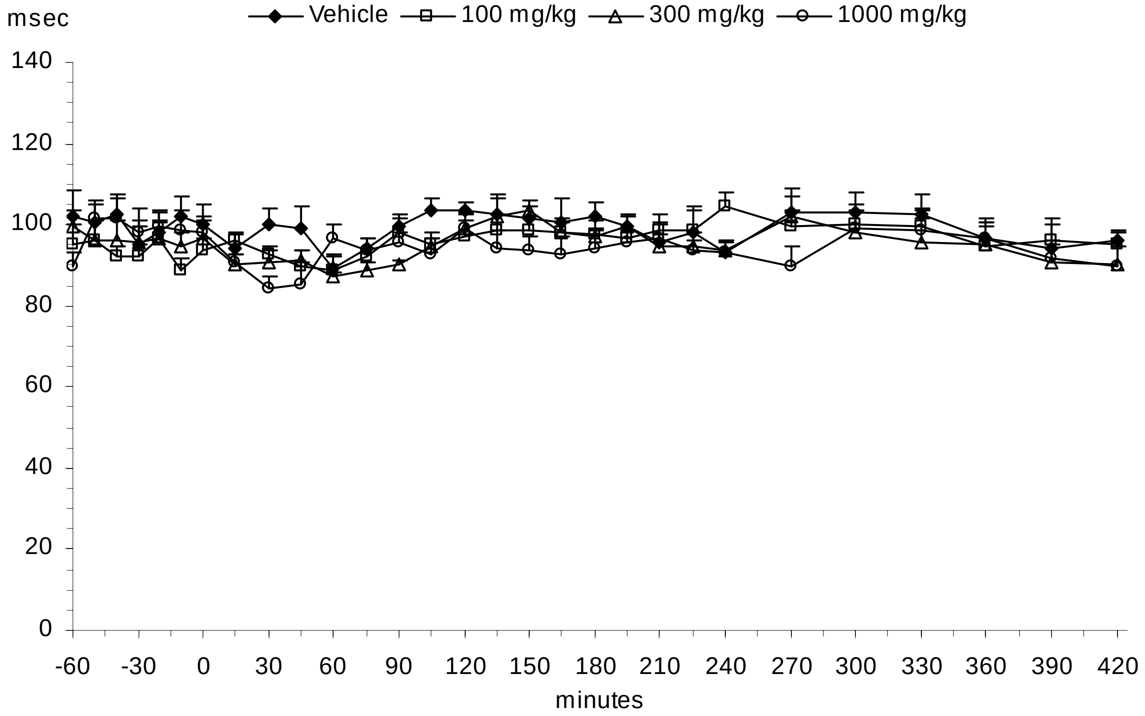

B. Changes from pre-treatment values

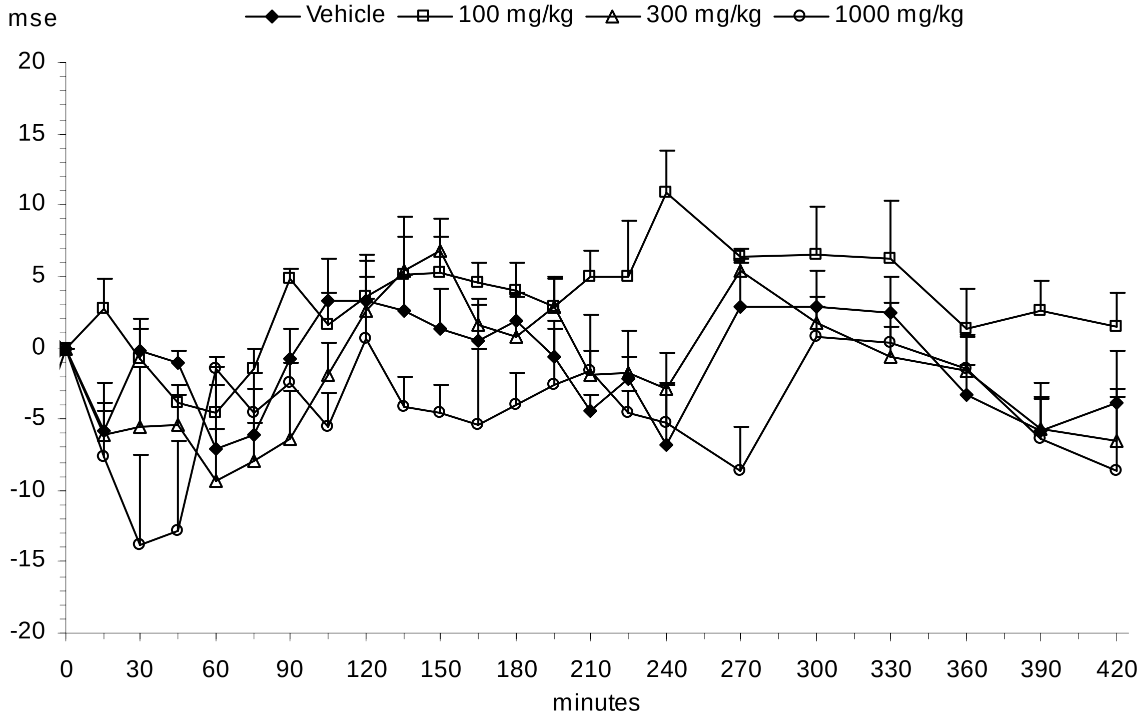

Figure 7. QRS interval

A. Absolute values

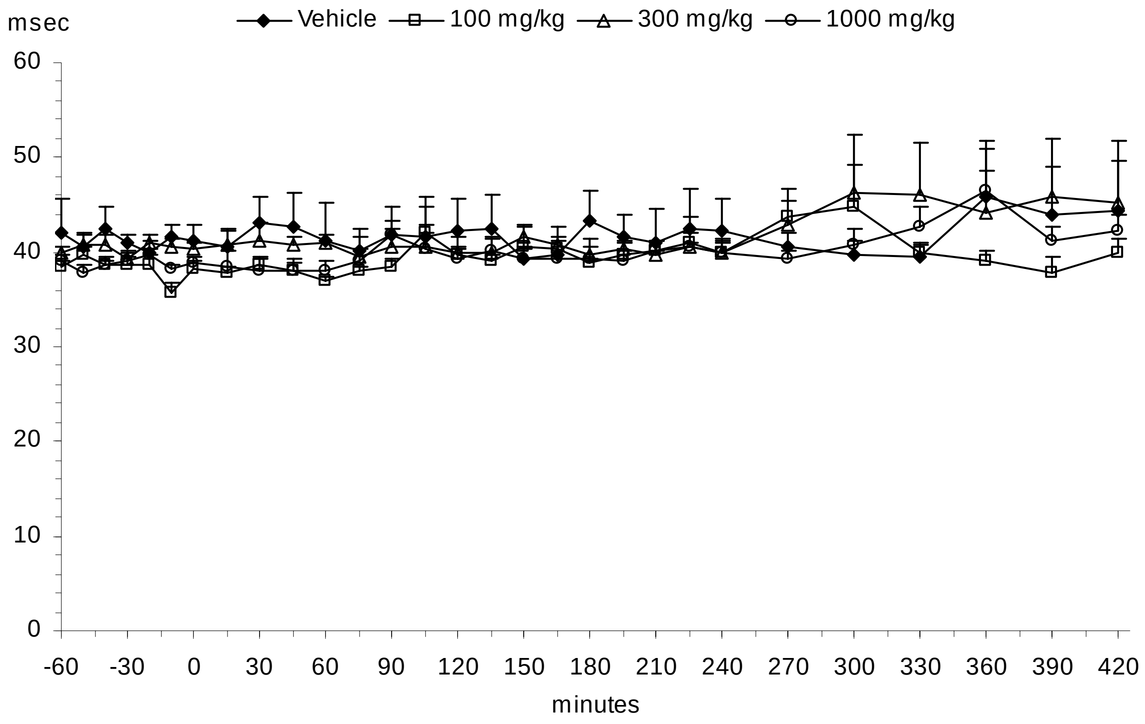

B. Changes from pre-treatment values

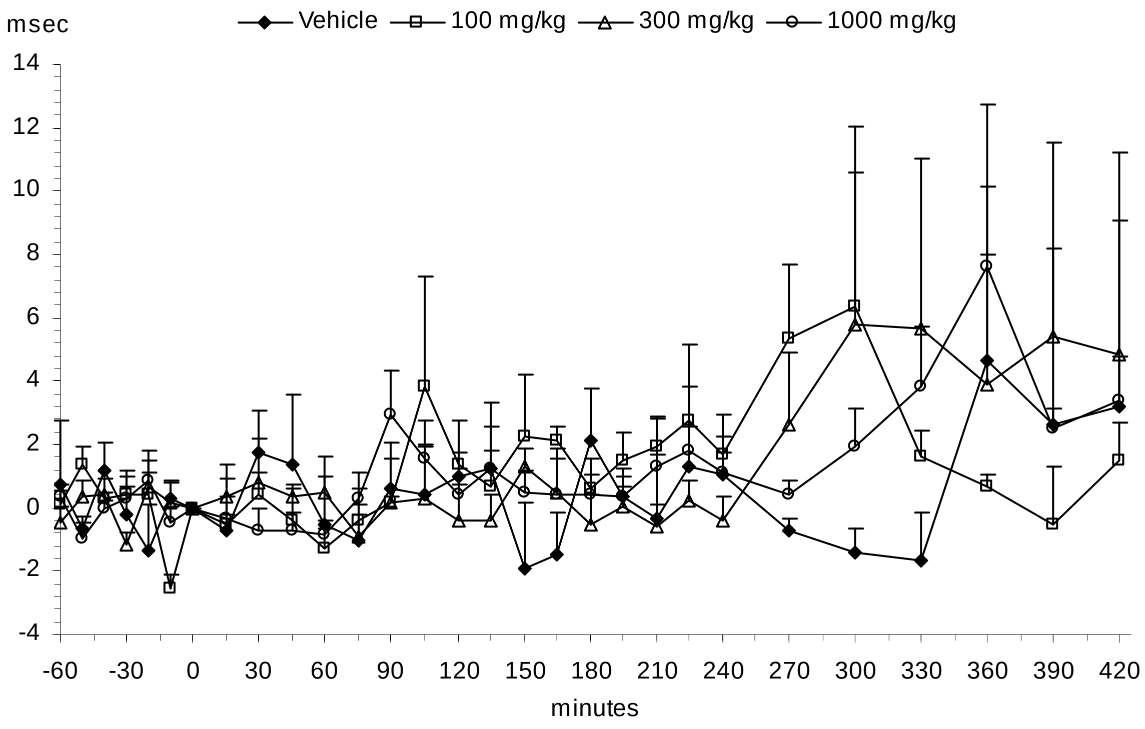

Figure 8. Uncorrected QT interval

A. Absolute values

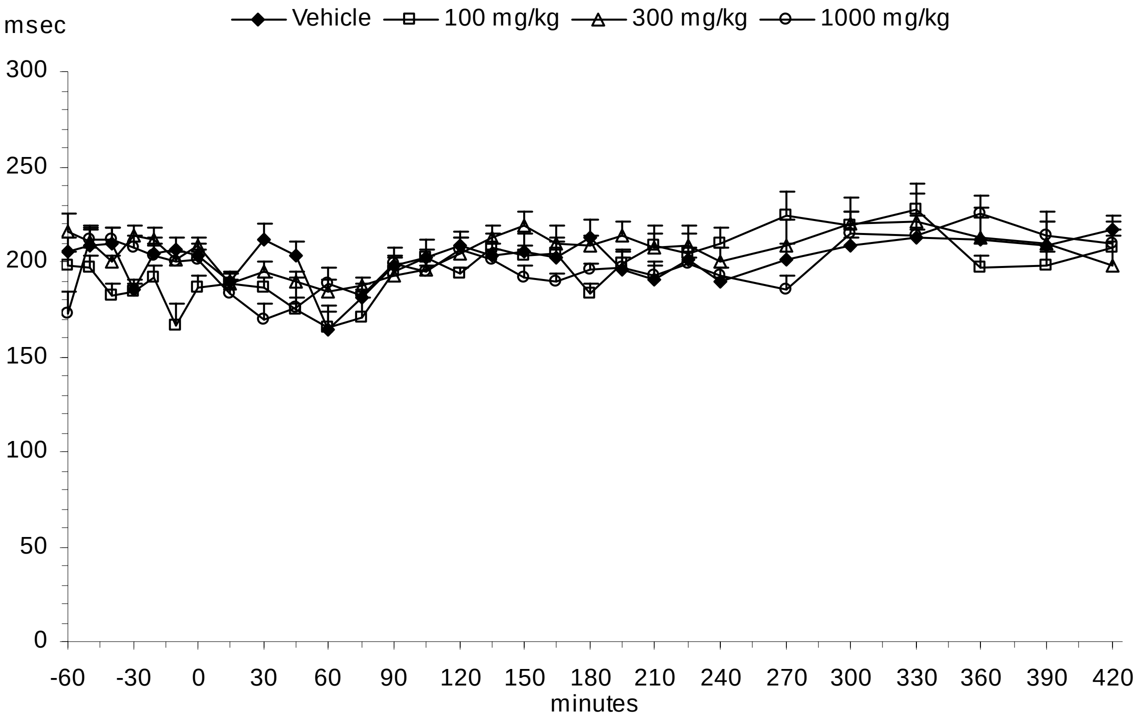

B. Changes from pre-treatment values

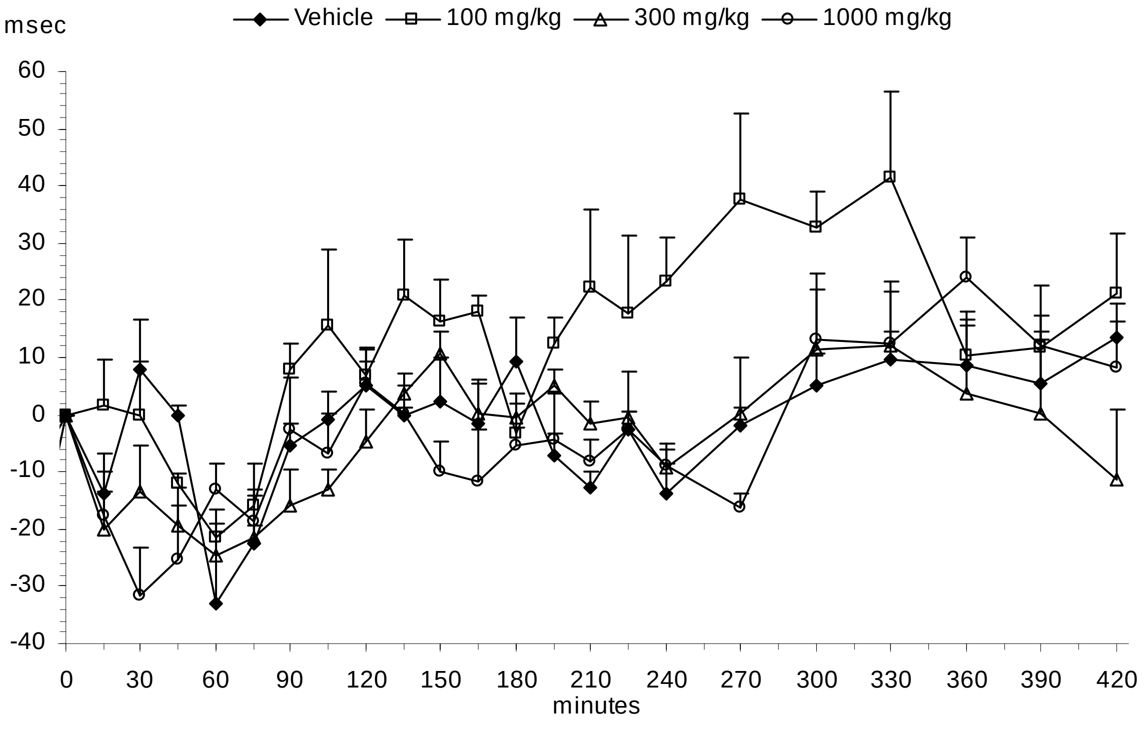

Figure 9. Corrected QT interval (covariate correction, QTca)

A. Absolute values

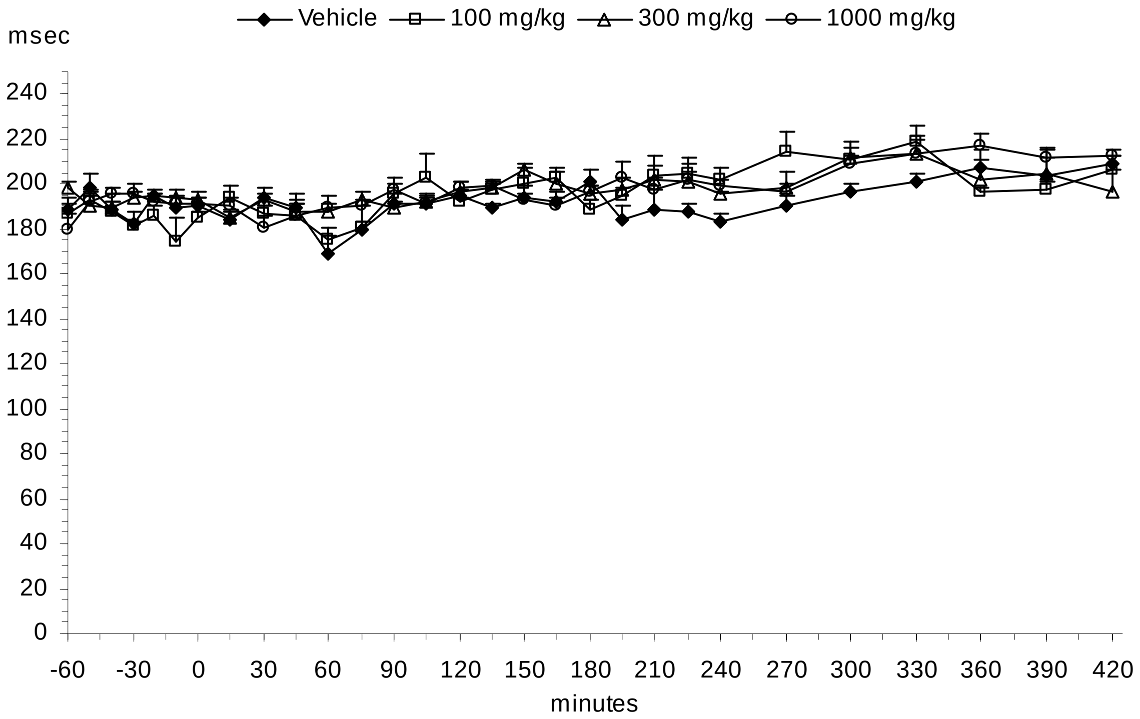

B. Changes from pre-treatment values

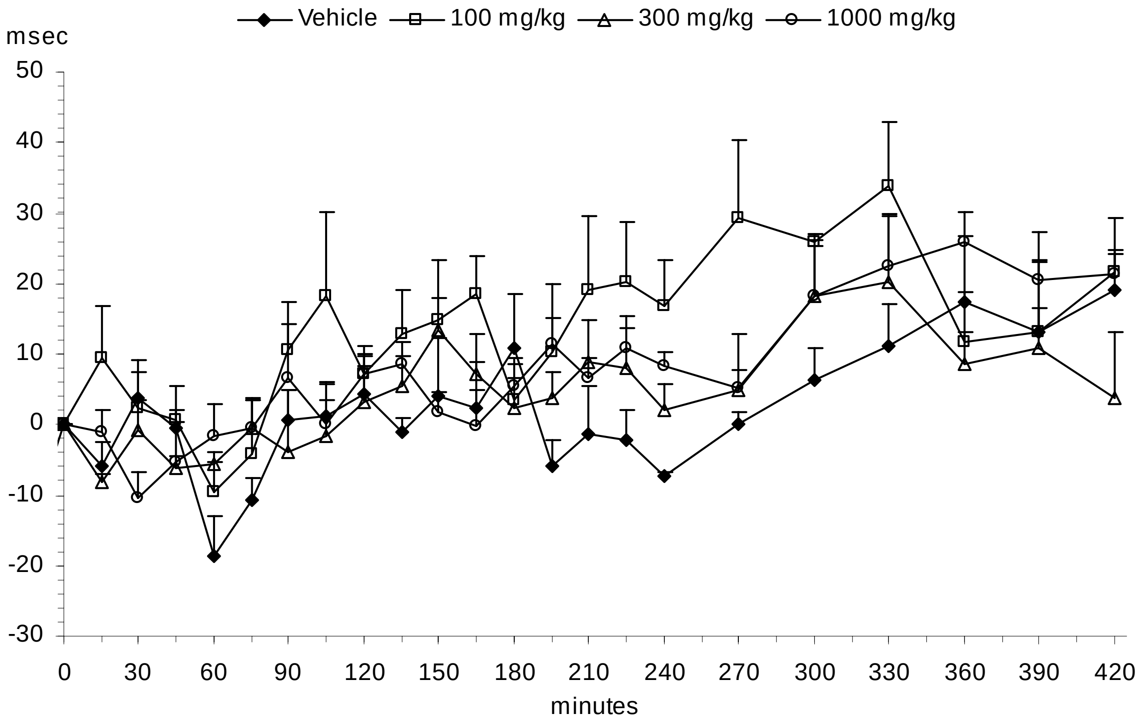

Figure 10. Body temperature

A. Absolute values

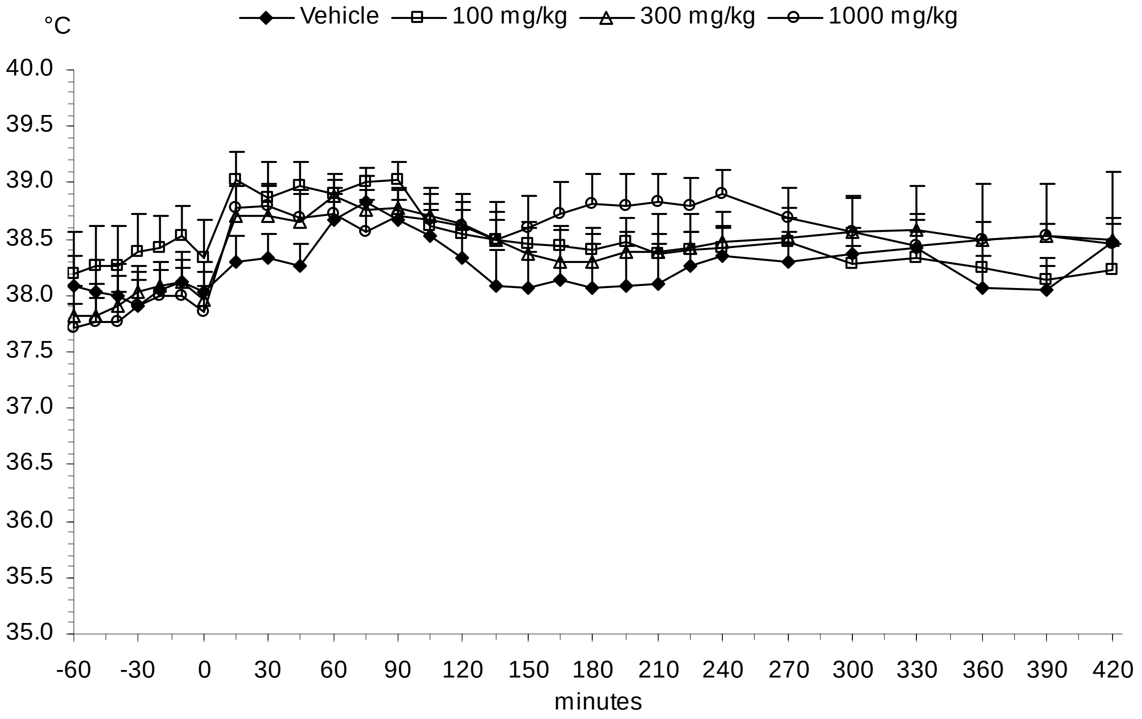

B. Changes from pre-treatment values

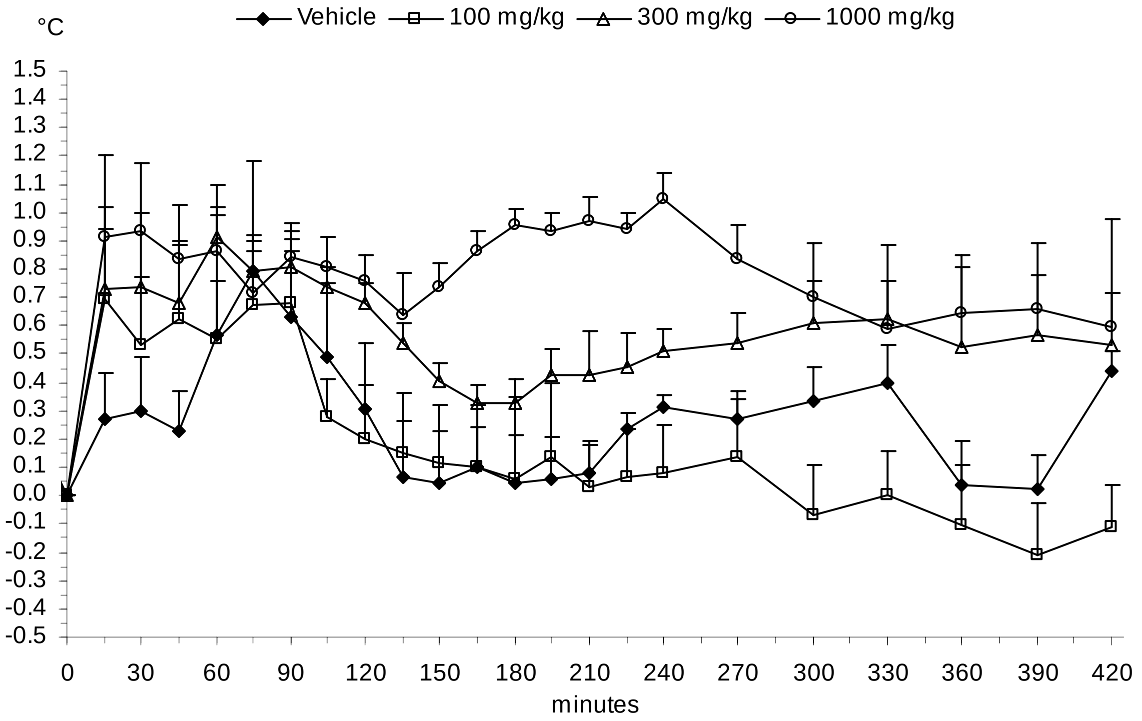

APPENDICES

Appendix 1 Study data listing

Appendix 1.1 – Group 1, Vehicle PO

Systolic blood pressure (mm Hg)

Time from	2482		2539		2552		2057		Absolute	Δ from 0	
dose (min)	Abs.	Δ from 0	Abs.	Δ from 0	Abs.	Δ from 0	Abs.	Δ from 0	Mean ± S.D.	Mean ± S.D.	N
-60	139	-15	144	0	134	-2	139	-6	139 ± 4.0	-6 ± 6.4	4
-50	144	-9	141	-3	138	2	148	3	143 ± 4.2	-2 ± 5.7	4
-40	149	-5	140	-4	136	-1	140	-5	141 ± 5.5	-4 ± 2.0	4
-30	165	11	157	13	141	5	152	7	154 ± 9.9	9 ± 3.7	4
-20	166	12	151	7	138	1	146	1	150 ± 11.9	5 ± 5.5	4
-10	159	5	132	-12	133	-4	144	0	142 ± 12.7	-3 ± 7.4	4
0	154	0	144	0	137	0	145	0	145 ± 6.9	0 ± 0.0	4
15	159	5	158	14	129	-7	152	7	150 ± 13.8	5 ± 9.0	4
30	158	5	141	-3	130	-7	138	-6	142 ± 11.9	-3 ± 5.3	4
45	151	-3	140	-4	124	-13	153	8	142 ± 13.2	-3 ± 8.5	4
60	-	-	159	15	155	18	165	20	159 ± 5.0	18 ± 2.5	3
75	163	9	133	-11	136	-1	157	12	147 ± 14.6	2 ± 10.2	4
90	143	-11	122	-22	141	4	127	-18	133 ± 10.1	-12 ± 11.3	4
105	137	-16	118	-26	128	-9	142	-3	131 ± 10.4	-14 ± 9.8	4
120	136	-18	130	-14	138	1	134	-11	134 ± 3.3	-10 ± 8.1	4
135	143	-11	128	-16	127	-10	144	-1	136 ± 9.2	-9 ± 6.4	4
150	139	-14	135	-9	150	13	140	-5	141 ± 6.4	-4 ± 12.1	4
165	136	-18	129	-15	143	7	141	-4	137 ± 6.3	-8 ± 11.3	4
180	145	-9	142	-2	138	1	142	-3	141 ± 3.1	-3 ± 3.9	4
195	157	3	153	9	132	-4	140	-5	146 ± 11.5	1 ± 6.6	4
210	168	15	147	3	133	-3	155	10	151 ± 14.6	6 ± 7.9	4
225	158	5	140	-4	132	-5	146	1	144 ± 11.1	-1 ± 4.4	4
240	186	33	141	-3	145	8	154	9	157 ± 20.4	12 ± 14.9	4
270	154	0	138	-6	146	9	146	1	146 ± 6.4	1 ± 6.1	4
300	141	-13	137	-7	135	-1	157	12	143 ± 10.0	-2 ± 10.7	4
330	140	-14	136	-8	126	-11	139	-6	135 ± 6.5	-10 ± 3.4	4
360	136	-18	126	-18	136	-1	132	-13	133 ± 4.6	-12 ± 8.2	4
390	135	-19	120	-24	122	-15	138	-7	129 ± 9.1	-16 ± 7.3	4
420	133	-20	117	-27	126	-11	133	-12	127 ± 7.8	-18 ± 7.9	4

"-" Data not available (See Section 6.3.2.1. Deviations from Protocol)

Appendix 1.1 – Group 1, Vehicle PO

Diastolic blood pressure (mm Hg)

Time from	2482		2539		2552		2057		Absolute	Δ from 0	
dose (min)	Abs.	Δ from 0	Abs.	Δ from 0	Abs.	Δ from 0	Abs.	Δ from 0	Mean ± S.D.	Mean ± S.D.	N
-60	85	-4	77	-3	92	1	88	-4	85 ± 6.2	-3 ± 2.1	4
-50	87	-2	78	-2	96	5	94	2	89 ± 8.1	1 ± 3.2	4
-40	79	-9	73	-7	94	3	86	-6	83 ± 8.8	-5 ± 5.1	4
-30	95	7	98	18	92	0	101	9	96 ± 3.9	8 ± 7.2	4
-20	96	8	85	6	90	-1	92	0	91 ± 4.6	3 ± 4.3	4
-10	89	0	67	-12	84	-7	90	-2	83 ± 10.5	-5 ± 5.7	4
0	88	0	80	0	91	0	92	0	88 ± 5.5	0 ± 0.0	4
15	87	-2	90	10	88	-4	100	9	91 ± 6.3	3 ± 6.9	4
30	87	-1	78	-2	91	0	85	-7	85 ± 5.4	-2 ± 3.1	4
45	82	-6	80	0	88	-3	98	6	87 ± 8.2	-1 ± 5.3	4
60	-	-	101	21	117	26	115	24	111 ± 9.1	23 ± 2.7	3
75	103	15	76	-4	97	6	106	14	96 ± 13.6	8 ± 8.7	4
90	90	1	71	-9	102	11	79	-12	85 ± 13.3	-2 ± 10.4	4
105	84	-4	63	-17	90	-2	94	2	83 ± 13.5	-5 ± 8.0	4
120	82	-6	76	-4	101	10	85	-7	86 ± 10.5	-2 ± 7.7	4
135	87	-1	70	-10	95	4	95	4	87 ± 11.9	-1 ± 6.4	4
150	84	-4	76	-4	111	20	93	1	91 ± 14.8	3 ± 11.2	4
165	77	-11	69	-10	104	13	93	1	86 ± 15.4	-2 ± 11.2	4
180	81	-7	81	1	100	9	93	1	89 ± 9.1	1 ± 6.5	4
195	86	-2	75	-4	99	8	91	0	88 ± 9.8	0 ± 5.3	4
210	92	4	82	2	96	5	97	5	92 ± 7.0	4 ± 1.6	4
225	88	-1	80	0	95	3	94	2	89 ± 6.9	1 ± 1.9	4
240	94	5	78	-2	105	14	103	11	95 ± 12.2	7 ± 6.9	4
270	88	-1	83	3	111	20	94	2	94 ± 12.1	6 ± 9.1	4
300	84	-5	80	0	99	8	109	17	93 ± 13.7	5 ± 9.8	4
330	86	-3	79	-1	92	0	93	2	87 ± 6.7	0 ± 1.9	4
360	85	-4	70	-10	103	12	89	-3	87 ± 13.5	-1 ± 9.2	4
390	87	-2	68	-12	88	-3	96	4	85 ± 11.7	-3 ± 6.5	4
420	87	-1	71	-9	99	7	96	4	88 ± 12.5	0 ± 7.2	4

"-" Data not available (See Section 6.3.2.1. Deviations from Protocol)

Appendix 1.1 – Group 1, Vehicle PO

Mean blood pressure (mm Hg)

Time from	2482		2539		2552		2057		Absolute	Δ from 0	
dose (min)	Abs.	Δ from 0	Abs.	Δ from 0	Abs.	Δ from 0	Abs.	Δ from 0	Mean ± S.D.	Mean ± S.D.	N
-60	105	-7	95	-6	108	0	104	-5	103 ± 5.5	-4 ± 3.1	4
-50	107	-5	101	0	111	3	111	2	108 ± 4.6	0 ± 3.4	4
-40	103	-9	95	-6	110	1	102	-7	102 ± 5.9	-5 ± 4.5	4
-30	120	9	119	18	111	2	118	9	117 ± 4.5	10 ± 6.5	4
-20	122	10	108	6	108	-1	110	1	112 ± 6.7	4 ± 4.8	4
-10	114	2	89	-12	103	-6	107	-2	103 ± 10.6	-4 ± 6.3	4
0	112	0	101	0	108	0	109	0	108 ± 4.4	0 ± 0.0	4
15	113	1	114	13	103	-6	117	9	112 ± 6.5	4 ± 8.3	4
30	112	0	98	-3	105	-4	101	-7	104 ± 6.0	-4 ± 3.1	4
45	105	-7	99	-2	101	-8	116	7	105 ± 7.5	-2 ± 6.9	4
60	-	-	121	20	130	22	134	25	128 ± 6.3	22 ± 2.6	3
75	126	14	98	-3	111	3	124	15	114 ± 12.8	7 ± 8.9	4
90	109	-3	90	-11	116	7	96	-13	103 ± 11.8	-5 ± 9.2	4
105	103	-9	82	-19	103	-5	110	1	100 ± 11.8	-8 ± 8.3	4
120	102	-10	94	-7	114	6	102	-7	103 ± 8.3	-4 ± 7.1	4
135	107	-5	89	-12	106	-2	111	3	103 ± 9.6	-4 ± 6.1	4
150	104	-8	97	-4	126	17	108	-1	109 ± 12.0	1 ± 11.0	4
165	98	-14	90	-11	118	9	109	0	103 ± 12.2	-4 ± 10.8	4
180	104	-8	102	1	114	6	109	0	107 ± 5.4	0 ± 5.6	4
195	112	0	101	0	113	4	107	-2	108 ± 5.2	1 ± 2.5	4
210	122	10	106	4	109	0	116	7	113 ± 7.2	5 ± 4.2	4
225	109	-3	99	-2	107	-2	111	3	106 ± 5.1	-1 ± 2.5	4
240	128	16	99	-2	119	10	120	11	116 ± 12.3	9 ± 7.8	4
270	112	0	101	-1	125	16	111	3	112 ± 9.9	5 ± 8.0	4
300	106	-6	100	-2	112	3	125	17	111 ± 10.8	3 ± 9.7	4
330	106	-6	100	-1	104	-5	110	1	105 ± 4.1	-3 ± 3.1	4
360	103	-9	90	-11	116	7	102	-6	103 ± 10.3	-5 ± 8.2	4
390	104	-8	86	-16	100	-9	109	0	100 ± 9.9	-8 ± 6.5	4
420	103	-9	86	-15	108	0	108	-1	101 ± 10.2	-6 ± 7.0	4

"-" Data not available (See Section 6.3.2.1. Deviations from Protocol)

Appendix 1.1 – Group 1, Vehicle PO

Heart rate (beats/min)

Time from	2482		2539		2552		2057		Absolute	Δ from 0	
dose (min)	Abs.	Δ from 0	Abs.	Δ from 0	Abs.	Δ from 0	Abs.	Δ from 0	Mean ± S.D.	Mean ± S.D.	N
-60	61	-2	60	-22	102	-2	67	-13	73 ± 19.8	-10 ± 9.7	4
-50	63	0	105	23	113	9	68	-12	87 ± 25.3	5 ± 14.5	4
-40	48	-14	61	-21	95	-9	82	2	72 ± 20.8	-10 ± 9.8	4
-30	81	19	111	29	114	10	89	9	99 ± 16.2	16 ± 9.2	4
-20	64	2	79	-3	99	-5	109	29	88 ± 20.3	6 ± 16.1	4
-10	58	-5	77	-5	102	-2	64	-16	75 ± 19.5	-7 ± 6.2	4
0	63	0	82	0	104	0	80	0	82 ± 17.0	0 ± 0.0	4
15	82	20	104	22	92	-12	100	20	94 ± 9.6	12 ± 16.5	4
30	62	-1	83	1	106	2	63	-17	78 ± 20.5	-4 ± 8.6	4
45	58	-5	84	2	109	5	90	10	85 ± 21.0	3 ± 6.1	4
60	-	-	149	67	95	-9	135	55	126 ± 28.2	37 ± 41.0	3
75	89	26	134	52	101	-3	85	5	102 ± 22.2	20 ± 24.5	4
90	68	6	103	21	111	7	79	-1	90 ± 20.3	8 ± 9.4	4
105	76	14	82	-1	103	-1	68	-12	82 ± 14.9	0 ± 10.6	4
120	59	-4	85	3	103	-1	76	-4	81 ± 18.4	-1 ± 3.2	4
135	59	-3	77	-5	106	2	87	7	82 ± 19.4	0 ± 5.2	4
150	64	2	85	3	102	-2	86	6	84 ± 15.7	2 ± 3.0	4
165	62	0	86	4	122	18	96	16	92 ± 24.7	9 ± 8.9	4
180	67	5	90	8	116	12	81	1	89 ± 20.3	6 ± 4.4	4
195	63	1	85	2	184	80	73	-7	101 ± 55.6	19 ± 40.6	4
210	92	29	119	37	104	0	89	9	101 ± 13.6	19 ± 17.2	4
225	65	3	84	2	87	-17	81	1	79 ± 9.7	-3 ± 9.3	4
240	96	33	86	4	105	1	87	7	94 ± 8.9	11 ± 14.7	4
270	68	5	76	-6	125	21	74	-6	86 ± 26.4	4 ± 12.7	4
300	79	17	68	-14	94	-10	80	0	80 ± 10.7	-2 ± 13.7	4
330	72	9	79	-3	86	-18	94	14	83 ± 9.5	0 ± 14.2	4
360	78	15	92	10	137	33	100	20	102 ± 25.5	20 ± 10.0	4
390	78	16	94	12	127	23	106	26	101 ± 20.6	19 ± 6.4	4
420	79	16	81	-1	110	6	90	10	90 ± 14.4	8 ± 7.0	4

"-" Data not available (See Section 6.3.2.1. Deviations from Protocol)

Appendix 1.1 – Group 1, Vehicle PO

Uncorrected QT interval (msec)

Time from	2482		2539		2552		2057		Absolute	Δ from 0	
dose (min)	Abs.	Δ from 0	Abs.	Δ from 0	Abs.	Δ from 0	Abs.	Δ from 0	Mean ± S.D.	Mean ± S.D.	N
-60	227	4	205	6	194	-1	196	1	206 ± 14.7	2 ± 3.0	4
-50	231	8	208	8	192	-4	203	8	209 ± 16.5	5 ± 5.9	4
-40	233	10	212	13	198	2	195	0	209 ± 17.4	6 ± 6.3	4
-30	195	-28	172	-28	191	-4	183	-12	185 ± 10.2	-18 ± 11.7	4
-20	226	3	203	3	197	2	192	-3	205 ± 14.9	1 ± 3.0	4
-10	226	3	197	-3	201	5	202	7	206 ± 13.2	3 ± 4.2	4
0	223	0	200	0	195	0	195	0	203 ± 13.2	0 ± 0.0	4
15	197	-26	174	-26	198	3	189	-7	189 ± 11.3	-14 ± 14.4	4
30	222	-1	197	-2	197	2	229	34	211 ± 16.6	8 ± 17.3	4
45	226	3	202	3	193	-2	191	-4	203 ± 15.8	0 ± 3.5	4
60	-	-	144	-55	188	-7	159	-36	164 ± 22.3	-33 ± 24.1	3
75	191	-32	158	-41	187	-9	187	-8	181 ± 15.0	-22 ± 16.5	4
90	211	-11	194	-6	185	-10	201	6	198 ± 11.3	-5 ± 8.0	4
105	209	-14	204	4	192	-3	205	9	202 ± 7.3	-1 ± 10.2	4
120	221	-2	200	0	193	-2	220	24	208 ± 14.0	5 ± 12.8	4
135	222	-1	201	2	192	-4	198	2	203 ± 13.1	0 ± 2.8	4
150	220	-3	198	-2	184	-11	220	24	206 ± 17.5	2 ± 15.3	4
165	227	4	201	1	173	-22	206	10	202 ± 22.0	-2 ± 14.1	4
180	231	8	205	5	189	-7	226	30	213 ± 19.4	9 ± 15.3	4
195	222	-1	182	-18	187	-9	193	-2	196 ± 18.1	-7 ± 7.8	4
210	211	-12	194	-6	182	-13	176	-19	191 ± 15.2	-13 ± 5.6	4
225	217	-6	206	6	189	-7	192	-4	201 ± 13.2	-3 ± 6.0	4
240	194	-29	193	-7	188	-7	184	-11	190 ± 4.6	-14 ± 10.4	4
270	222	-1	204	4	185	-11	195	0	201 ± 15.7	-2 ± 6.2	4
300	215	-8	204	4	199	4	215	19	208 ± 7.8	5 ± 11.2	4
330	221	-2	206	7	206	11	217	22	213 ± 7.7	10 ± 9.8	4
360	232	9	195	-4	189	-6	231	35	212 ± 22.8	8 ± 19.2	4
390	234	11	202	2	177	-18	221	26	209 ± 24.9	5 ± 18.5	4
420	232	9	218	19	196	0	222	26	217 ± 15.3	14 ± 11.4	4

"-" Data not available (See Section 6.3.2.1. Deviations from Protocol)

Appendix 1.1 – Group 1, Vehicle PO

Corrected QT interval (covariate analysis, QTca) (msec)

Time from dose (min)	2482 Abs.	2482 Δ from 0	2539 Abs.	2539 Δ from 0	2552 Abs.	2552 Δ from 0	2057 Abs.	2057 Δ from 0	Absolute Mean ± S.D.	Δ from 0 Mean ± S.D.	N
-60	200	3	180	-7	195	-1	178	-3	188 ± 10.9	-2 ± 4.0	4
-50	207	9	210	23	195	-1	182	1	198 ± 12.6	8 ± 11.0	4
-40	194	-4	188	1	194	-2	178	-3	189 ± 7.4	-2 ± 1.9	4
-30	185	-13	174	-12	196	0	175	-6	182 ± 10.4	-8 ± 6.2	4
-20	201	4	187	0	197	1	193	12	195 ± 5.9	4 ± 5.6	4
-10	198	1	182	-5	199	3	180	-1	190 ± 10.6	-1 ± 3.5	4
0	197	0	187	0	196	0	181	0	190 ± 7.8	0 ± 0.0	4
15	188	-10	174	-13	193	-3	183	2	184 ± 8.4	-6 ± 6.6	4
30	196	-2	183	-4	198	2	200	19	194 ± 7.8	4 ± 10.5	4
45	195	-2	189	2	194	-2	181	0	190 ± 6.6	-1 ± 1.9	4
60	-	-	157	-30	182	-14	169	-12	169 ± 12.8	-19 ± 9.9	3
75	183	-14	169	-17	186	-10	179	-2	180 ± 7.4	-11 ± 6.4	4
90	193	-5	194	8	188	-8	189	8	191 ± 2.9	1 ± 8.3	4
105	192	-6	198	12	190	-6	186	5	192 ± 5.0	1 ± 8.6	4
120	196	-2	189	2	192	-4	202	21	195 ± 5.7	4 ± 11.3	4
135	193	-5	186	-1	193	-3	186	5	189 ± 4.2	-1 ± 4.0	4
150	197	0	188	2	184	-12	208	27	194 ± 10.8	4 ± 16.6	4
165	201	3	190	4	181	-15	198	17	193 ± 8.9	2 ± 13.3	4
180	209	12	195	9	189	-7	211	30	201 ± 10.8	11 ± 15.3	4
195	200	3	175	-12	185	-11	178	-3	184 ± 11.5	-6 ± 7.2	4
210	205	7	200	13	184	-12	167	-14	189 ± 16.9	-1 ± 13.6	4
225	196	-2	190	4	182	-14	184	3	188 ± 6.2	-2 ± 8.4	4
240	190	-7	181	-6	187	-9	174	-7	183 ± 7.3	-7 ± 1.2	4
270	201	4	183	-3	195	-1	182	1	190 ± 9.3	0 ± 3.2	4
300	203	6	187	1	196	0	200	19	197 ± 7.0	6 ± 8.9	4
330	204	6	194	7	198	2	210	29	201 ± 6.7	11 ± 12.0	4
360	217	20	188	2	201	5	224	43	208 ± 15.9	17 ± 18.6	4
390	215	18	193	6	186	-10	219	38	203 ± 16.1	13 ± 20.0	4
420	214	17	209	23	200	4	213	32	209 ± 6.5	19 ± 11.7	4

"-" Data not available (See Section 6.3.2.1. Deviations from Protocol)

Appendix 1.1 – Group 1, Vehicle PO

PR interval (msec)

Time from	2482		2539		2552		2057		Absolute	Δ from 0	
dose (min)	Abs.	Δ from 0	Abs.	Δ from 0	Abs.	Δ from 0	Abs.	Δ from 0	Mean ± S.D.	Mean ± S.D.	N
-60	115	3	111	6	91	-2	92	1	102 ± 12.4	2 ± 3.4	4
-50	113	1	100	-5	94	1	95	4	100 ± 8.6	0 ± 3.8	4
-40	112	1	110	5	98	5	90	-1	103 ± 10.1	2 ± 3.0	4
-30	107	-4	97	-8	90	-3	87	-4	95 ± 9.1	-5 ± 2.4	4
-20	111	-1	101	-4	93	0	88	-3	98 ± 10.0	-2 ± 2.0	4
-10	111	-1	111	6	92	-1	94	3	102 ± 10.5	2 ± 3.6	4
0	111	0	105	0	93	0	91	0	100 ± 9.7	0 ± 0.0	4
15	101	-10	99	-6	92	-1	86	-5	94 ± 6.9	-6 ± 3.8	4
30	110	-1	102	-3	93	0	95	4	100 ± 7.8	0 ± 3.1	4
45	112	0	105	0	90	-3	90	-1	99 ± 11.0	-1 ± 1.7	4
60	-	-	87	-18	95	2	86	-5	89 ± 5.0	-7 ± 9.8	3
75	101	-10	93	-12	90	-3	92	1	94 ± 4.7	-6 ± 6.2	4
90	107	-4	102	-3	93	0	96	5	99 ± 6.3	-1 ± 4.1	4
105	106	-6	112	7	99	6	97	6	103 ± 6.7	3 ± 5.9	4
120	105	-6	109	4	100	7	99	8	103 ± 4.4	3 ± 6.4	4
135	107	-4	110	5	100	7	94	3	103 ± 7.1	3 ± 5.0	4
150	107	-5	106	1	93	0	100	9	101 ± 6.2	1 ± 5.6	4
165	107	-4	114	9	90	-3	92	1	101 ± 11.7	0 ± 5.9	4
180	108	-4	109	4	95	2	97	6	102 ± 7.2	2 ± 4.1	4
195	106	-6	103	-2	93	0	97	6	99 ± 5.8	-1 ± 5.0	4
210	104	-8	102	-3	89	-4	88	-3	96 ± 8.4	-4 ± 2.3	4
225	111	0	102	-3	87	-6	91	0	98 ± 10.9	-2 ± 2.9	4
240	93	-19	99	-6	94	1	87	-3	93 ± 4.7	-7 ± 8.4	4
270	110	-1	116	11	91	-2	94	3	103 ± 12.2	3 ± 6.1	4
300	114	3	107	2	90	-3	100	9	103 ± 10.2	3 ± 5.0	4
330	108	-3	113	8	93	0	97	6	103 ± 9.4	2 ± 5.0	4
360	102	-9	105	0	90	-3	90	-1	97 ± 7.8	-3 ± 4.1	4
390	105	-6	109	4	80	-13	83	-8	94 ± 14.5	-6 ± 6.8	4
420	102	-9	94	-11	97	4	92	1	96 ± 4.4	-4 ± 7.3	4

“-“ Data not available (See Section 6.3.2.1. Deviations from Protocol)

Appendix 1.1 – Group 1, Vehicle PO

QRS interval (msec)

Time from dose (min)	2482 Abs.	2482 Δ from 0	2539 Abs.	2539 Δ from 0	2552 Abs.	2552 Δ from 0	2057 Abs.	2057 Δ from 0	Absolute Mean ± S.D.	Δ from 0 Mean ± S.D.	N
-60	39	-1	53	7	37	-2	40	-1	42 ± 7.2	1 ± 4.0	4
-50	39	0	45	-1	38	0	40	-1	41 ± 2.9	-1 ± 0.3	4
-40	41	1	50	4	38	0	41	0	42 ± 4.9	1 ± 1.8	4
-30	40	0	42	-4	39	0	43	2	41 ± 1.8	0 ± 2.4	4
-20	39	0	40	-6	39	0	41	0	40 ± 0.9	-1 ± 2.9	4
-10	40	0	46	0	41	2	40	-1	42 ± 2.8	0 ± 1.1	4
0	40	0	46	0	39	0	41	0	41 ± 3.2	0 ± 0.0	4
15	38	-1	46	0	37	-2	41	0	41 ± 3.8	-1 ± 0.9	4
30	40	0	52	6	39	1	41	0	43 ± 5.8	2 ± 2.7	4
45	41	1	53	8	38	-1	38	-2	43 ± 7.3	1 ± 4.4	4
60	-	-	49	3	38	-1	37	-4	41 ± 6.9	-1 ± 3.8	3
75	39	-1	47	1	37	-2	38	-3	40 ± 4.6	-1 ± 1.7	4
90	40	0	51	5	38	-1	39	-2	42 ± 5.9	1 ± 2.9	4
105	39	-1	51	5	37	-1	39	-2	42 ± 6.4	0 ± 3.3	4
120	39	-1	52	6	37	-2	41	1	42 ± 6.7	1 ± 3.5	4
135	39	-1	53	7	38	-1	40	-1	43 ± 7.2	1 ± 4.1	4
150	41	2	38	-8	37	-1	41	0	39 ± 2.1	-2 ± 4.2	4
165	41	2	43	-3	34	-4	41	0	40 ± 3.7	-1 ± 2.7	4
180	40	0	53	7	40	1	41	0	43 ± 6.3	2 ± 3.3	4
195	40	0	48	2	37	-2	41	0	42 ± 4.8	0 ± 1.7	4
210	39	-1	51	6	35	-4	39	-2	41 ± 7.2	0 ± 4.1	4
225	40	0	54	9	35	-4	41	0	43 ± 8.2	1 ± 5.1	4
240	37	-2	52	6	39	0	41	0	42 ± 6.8	1 ± 3.7	4
270	40	0	44	-1	37	-1	40	-1	41 ± 2.9	-1 ± 0.7	4
300	40	1	43	-3	36	-2	40	-1	40 ± 2.6	-1 ± 1.6	4
330	40	0	40	-6	36	-3	42	1	40 ± 2.5	-2 ± 3.1	4
360	60	20	43	-2	36	-3	44	3	46 ± 10.1	5 ± 10.9	4
390	58	18	40	-6	35	-4	43	2	44 ± 10.0	3 ± 11.1	4
420	60	20	39	-7	37	-2	43	2	44 ± 10.5	3 ± 11.8	4

“-“ Data not available (See Section 6.3.2.1. Deviations from Protocol)

Appendix 1.1 – Group 1, Vehicle PO

RR interval (msec)

Time from	2482		2539		2552		2057		Absolute	Δ from 0	
dose (min)	Abs.	Δ from 0	Abs.	Δ from 0	Abs.	Δ from 0	Abs.	Δ from 0	Mean ± S.D.	Mean ± S.D.	N
-60	1030	3	1072	246	596	3	917	65	904 ± 215.5	79 ± 115.2	4
-50	982	-45	579	-247	560	-34	978	125	774 ± 237.2	-50 ± 152.6	4
-40	1336	309	1030	204	646	53	883	31	974 ± 288.5	149 ± 131.2	4
-30	760	-267	566	-260	534	-59	733	-119	648 ± 114.8	-176 ± 103.7	4
-20	999	-28	856	31	606	13	589	-263	763 ± 199.4	-62 ± 136.3	4
-10	1056	29	851	25	617	23	1012	160	884 ± 198.8	59 ± 67.0	4
0	1027	0	826	0	593	0	852	0	825 ± 178.3	0 ± 0.0	4
15	738	-289	599	-226	668	75	694	-158	675 ± 58.1	-150 ± 158.9	4
30	1034	7	838	13	588	-5	1082	230	886 ± 224.7	61 ± 112.6	4
45	1132	105	814	-12	582	-11	767	-85	824 ± 228.5	-1 ± 78.3	4
60	-	-	419	-407	689	95	462	-390	523 ± 144.8	-234 ± 285.2	3
75	717	-310	448	-378	601	8	733	-119	625 ± 131.8	-200 ± 176.7	4
90	901	-126	592	-233	557	-36	792	-60	711 ± 163.9	-114 ± 88.3	4
105	875	-152	676	-149	623	30	907	55	770 ± 141.9	-54 ± 112.0	4
120	1019	-8	771	-55	615	22	866	14	818 ± 169.3	-7 ± 34.6	4
135	1106	79	854	28	583	-10	792	-60	834 ± 215.5	9 ± 58.8	4
150	968	-60	749	-77	612	19	762	-90	773 ± 146.7	-52 ± 48.7	4
165	1027	0	754	-72	500	-93	707	-145	747 ± 216.8	-78 ± 60.2	4
180	919	-108	741	-85	601	8	804	-48	766 ± 132.4	-58 ± 50.7	4
195	941	-87	713	-112	633	40	864	12	788 ± 139.7	-37 ± 74.0	4
210	683	-345	527	-299	577	-16	755	-97	635 ± 103.0	-189 ± 157.8	4
225	946	-81	844	18	703	110	709	-143	800 ± 116.7	-24 ± 111.3	4
240	649	-378	797	-28	612	19	765	-87	706 ± 89.1	-119 ± 178.5	4
270	916	-111	951	125	479	-114	810	-42	789 ± 215.2	-35 ± 112.2	4
300	767	-260	873	47	648	54	818	-34	776 ± 96.2	-48 ± 146.9	4
330	867	-161	782	-44	717	124	703	-149	767 ± 74.4	-57 ± 131.9	4
360	804	-224	697	-129	459	-134	686	-166	661 ± 145.1	-163 ± 43.4	4
390	871	-156	730	-95	482	-111	629	-223	678 ± 164.0	-146 ± 57.1	4
420	844	-183	721	-104	544	-49	712	-140	706 ± 123.1	-119 ± 56.8	4

“-“ Data not available (See Section 6.3.2.1. Deviations from Protocol)

Appendix 1.1 – Group 1, Vehicle PO

Body temperature (°C)

Time from dose (min)	2482 Abs.	2482 Δ from 0	2539 Abs.	2539 Δ from 0	2552 Abs.	2552 Δ from 0	2057 Abs.	2057 Δ from 0	Absolute Mean ± S.D.	Δ from 0 Mean ± S.D.	N
-60	38.0	0.1	37.6	-0.1	38.8	0.0	37.9	0.1	38.1 ± 0.52	0.1 ± 0.1	4
-50	38.0	0.2	37.4	-0.2	38.8	0.0	37.8	0.1	38.0 ± 0.58	0.0 ± 0.2	4
-40	37.8	0.0	37.5	-0.2	38.8	0.0	37.8	0.0	38.0 ± 0.56	0.0 ± 0.1	4
-30	37.7	-0.2	37.5	-0.2	38.8	0.0	37.7	-0.1	37.9 ± 0.58	-0.1 ± 0.1	4
-20	37.7	-0.1	37.9	0.2	38.8	0.0	37.7	0.0	38.0 ± 0.52	0.0 ± 0.1	4
-10	37.8	-0.1	38.1	0.4	38.9	0.0	37.8	0.0	38.1 ± 0.52	0.1 ± 0.2	4
0	37.8	0.0	37.7	0.0	38.8	0.0	37.8	0.0	38.0 ± 0.53	0.0 ± 0.0	4
15	38.1	0.3	38.4	0.7	38.9	0.0	37.8	0.0	38.3 ± 0.46	0.3 ± 0.3	4
30	38.2	0.4	38.5	0.8	38.8	0.0	37.8	0.0	38.3 ± 0.43	0.3 ± 0.4	4
45	38.2	0.4	38.2	0.5	38.8	0.0	37.8	0.0	38.3 ± 0.40	0.2 ± 0.3	4
60	-	-	39.0	1.4	38.7	-0.1	38.2	0.4	38.7 ± 0.41	0.6 ± 0.7	3
75	39.3	1.4	39.1	1.4	38.7	-0.1	38.2	0.4	38.8 ± 0.46	0.8 ± 0.8	4
90	39.1	1.2	38.7	1.0	38.7	-0.1	38.1	0.3	38.7 ± 0.39	0.6 ± 0.6	4
105	38.9	1.1	38.5	0.8	38.8	0.0	37.9	0.1	38.5 ± 0.46	0.5 ± 0.5	4
120	38.6	0.7	38.4	0.7	38.8	0.0	37.6	-0.2	38.3 ± 0.53	0.3 ± 0.5	4
135	38.2	0.4	38.1	0.4	38.8	0.0	37.3	-0.5	38.1 ± 0.62	0.1 ± 0.4	4
150	38.0	0.2	38.1	0.4	38.9	0.0	37.3	-0.5	38.1 ± 0.63	0.0 ± 0.4	4
165	38.1	0.2	38.0	0.3	39.0	0.2	37.5	-0.3	38.1 ± 0.62	0.1 ± 0.3	4
180	37.8	0.0	37.9	0.3	39.2	0.3	37.4	-0.4	38.1 ± 0.77	0.0 ± 0.3	4
195	37.8	0.0	38.0	0.3	39.1	0.3	37.5	-0.3	38.1 ± 0.72	0.1 ± 0.3	4
210	37.9	0.1	37.9	0.2	39.1	0.3	37.5	-0.2	38.1 ± 0.69	0.1 ± 0.2	4
225	38.0	0.2	38.0	0.3	39.1	0.3	37.9	0.1	38.3 ± 0.58	0.2 ± 0.1	4
240	38.1	0.3	38.1	0.4	39.2	0.3	38.0	0.2	38.3 ± 0.55	0.3 ± 0.1	4
270	38.3	0.4	38.1	0.4	39.0	0.2	37.8	0.0	38.3 ± 0.52	0.3 ± 0.2	4
300	38.2	0.3	38.4	0.7	39.0	0.2	37.9	0.1	38.4 ± 0.47	0.3 ± 0.2	4
330	38.2	0.4	38.4	0.7	39.2	0.4	37.9	0.1	38.4 ± 0.58	0.4 ± 0.3	4
360	37.9	0.1	38.1	0.4	38.8	0.0	37.5	-0.3	38.1 ± 0.56	0.0 ± 0.3	4
390	37.9	0.0	38.0	0.4	38.7	-0.2	37.7	-0.1	38.1 ± 0.43	0.0 ± 0.2	4
420	38.2	0.4	38.2	0.5	39.1	0.3	38.4	0.6	38.5 ± 0.42	0.4 ± 0.1	4

“-“ Data not available (See Section 6.3.2.1. Deviations from Protocol)

Appendix 1.2 – Group 2, Fexinidazole 100 mg/kg PO

Systolic blood pressure (mm Hg)

Time from	2482		2539		2552		2057		Absolute	Δ from 0	
dose (min)	Abs.	Δ from 0	Abs.	Δ from 0	Abs.	Δ from 0	Abs.	Δ from 0	Mean ± S.D.	Mean ± S.D.	N
-60	150	-8	150	-3	174	20	139	-9	153 ± 14.8	0 ± 13.9	4
-50	155	-3	145	-9	147	-7	146	-2	148 ± 4.5	-5 ± 3.0	4
-40	159	1	153	0	152	-2	145	-3	152 ± 5.7	-1 ± 1.9	4
-30	152	-6	156	3	151	-3	154	6	153 ± 2.4	0 ± 5.3	4
-20	157	-1	150	-4	146	-7	152	3	151 ± 4.4	-2 ± 4.6	4
-10	175	17	167	13	152	-2	154	6	162 ± 10.7	9 ± 8.2	4
0	158	0	154	0	154	0	149	0	153 ± 3.8	0 ± 0.0	4
15	168	10	136	-18	162	8	133	-15	150 ± 18.0	-4 ± 15.1	4
30	157	-1	125	-28	133	-21	145	-4	140 ± 13.9	-13 ± 13.3	4
45	151	-7	161	7	142	-12	143	-5	149 ± 8.6	-4 ± 7.9	4
60	161	3	161	7	158	4	159	11	160 ± 1.2	6 ± 3.6	4
75	132	-25	143	-11	143	-11	142	-6	140 ± 5.1	-13 ± 8.3	4
90	144	-14	128	-25	138	-16	129	-20	135 ± 7.5	-19 ± 5.0	4
105	150	-8	130	-23	148	-6	128	-21	139 ± 11.9	-14 ± 9.1	4
120	150	-8	137	-17	156	2	151	2	148 ± 8.1	-5 ± 9.1	4
135	146	-12	135	-18	146	-8	134	-15	140 ± 6.5	-13 ± 4.3	4
150	137	-21	142	-11	153	0	147	-2	145 ± 6.8	-8 ± 9.4	4
165	144	-14	137	-17	165	12	150	1	149 ± 12.2	-4 ± 13.3	4
180	152	-6	164	10	168	14	148	0	158 ± 9.2	5 ± 9.0	4
195	155	-3	130	-23	151	-3	142	-7	144 ± 10.8	-9 ± 9.6	4
210	156	-1	148	-6	166	12	141	-7	153 ± 10.6	-1 ± 8.8	4
225	151	-7	142	-11	154	1	146	-3	148 ± 5.4	-5 ± 5.2	4
240	142	-16	144	-10	157	3	147	-2	147 ± 6.7	-6 ± 8.6	4
270	140	-18	138	-16	146	-7	152	3	144 ± 6.4	-10 ± 9.7	4
300	149	-9	128	-25	143	-11	141	-7	140 ± 8.7	-13 ± 8.3	4
330	137	-21	146	-8	137	-17	133	-15	138 ± 5.5	-15 ± 5.6	4
360	153	-5	150	-3	137	-17	148	0	147 ± 7.0	-6 ± 7.3	4
390	149	-9	150	-4	160	6	146	-2	151 ± 5.9	-2 ± 6.2	4
420	136	-22	143	-11	154	0	149	1	145 ± 7.8	-8 ± 10.7	4

Appendix 1.2 – Group 2, Fexinidazole 100 mg/kg PO

Diastolic blood pressure (mm Hg)

Time from	2482		2539		2552		2057		Absolute	Δ from 0	
dose (min)	Abs.	Δ from 0	Abs.	Δ from 0	Abs.	Δ from 0	Abs.	Δ from 0	Mean ± S.D.	Mean ± S.D.	N
-60	88	-3	86	-2	101	0	81	-17	89 ± 8.8	-5 ± 7.6	4
-50	94	3	84	-5	99	-2	98	0	94 ± 7.1	-1 ± 3.5	4
-40	89	-2	85	-3	101	-1	93	-5	92 ± 6.5	-3 ± 1.6	4
-30	88	-3	91	3	103	1	104	7	97 ± 8.0	2 ± 3.9	4
-20	95	4	82	-7	99	-2	104	7	95 ± 9.7	0 ± 6.2	4
-10	91	0	104	15	106	4	105	8	102 ± 6.9	7 ± 6.4	4
0	91	0	89	0	102	0	97	0	95 ± 5.9	0 ± 0.0	4
15	107	16	83	-6	111	10	89	-8	98 ± 13.6	3 ± 11.7	4
30	92	1	75	-14	90	-11	98	0	89 ± 9.8	-6 ± 7.8	4
45	90	-1	93	5	95	-7	96	-2	93 ± 2.7	-1 ± 4.5	4
60	94	3	90	1	108	7	108	11	100 ± 9.4	5 ± 4.2	4
75	80	-11	85	-4	94	-8	99	1	89 ± 8.6	-5 ± 5.4	4
90	87	-4	79	-9	93	-9	89	-9	87 ± 5.5	-8 ± 2.7	4
105	91	0	80	-9	97	-5	83	-15	88 ± 7.9	-7 ± 6.4	4
120	88	-3	82	-7	109	8	104	6	96 ± 12.7	1 ± 6.9	4
135	83	-8	74	-15	93	-9	86	-11	84 ± 8.1	-11 ± 3.2	4
150	76	-15	84	-5	101	-1	100	3	90 ± 12.3	-4 ± 7.6	4
165	82	-9	84	-5	120	19	103	6	97 ± 18.1	3 ± 12.4	4
180	93	2	99	10	111	10	100	3	101 ± 7.9	6 ± 4.7	4
195	87	-4	73	-16	102	0	96	-1	90 ± 12.6	-5 ± 7.3	4
210	91	0	89	0	115	14	96	-2	98 ± 12.1	3 ± 7.3	4
225	97	7	84	-5	105	4	96	-1	96 ± 8.9	1 ± 5.2	4
240	88	-3	88	-1	104	2	107	10	97 ± 10.4	2 ± 5.8	4
270	78	-13	79	-10	98	-4	105	8	90 ± 13.6	-5 ± 9.1	4
300	86	-4	72	-16	99	-3	104	7	90 ± 14.1	-4 ± 9.5	4
330	82	-9	82	-7	97	-4	96	-2	89 ± 8.4	-5 ± 3.2	4
360	93	2	91	3	102	0	108	11	99 ± 7.9	4 ± 4.7	4
390	91	0	98	9	113	12	105	8	102 ± 9.7	7 ± 5.1	4
420	82	-9	85	-4	103	2	116	18	96 ± 16.0	2 ± 11.9	4

Appendix 1.2 – Group 2, Fexinidazole 100 mg/kg PO

Mean blood pressure (mm Hg)

Time from	2482		2539		2552		2057		Absolute	Δ from 0	
dose (min)	Abs.	Δ from 0	Abs.	Δ from 0	Abs.	Δ from 0	Abs.	Δ from 0	Mean ± S.D.	Mean ± S.D.	N
-60	111	-6	109	-3	124	3	100	-15	111 ± 10.2	-5 ± 7.6	4
-50	117	1	106	-6	118	-4	113	-2	114 ± 5.2	-3 ± 2.9	4
-40	117	1	111	-2	121	0	112	-3	115 ± 4.9	-1 ± 1.8	4
-30	111	-6	116	3	123	1	120	6	117 ± 5.4	1 ± 4.8	4
-20	118	2	105	-7	118	-4	120	6	115 ± 6.9	-1 ± 5.7	4
-10	123	7	127	15	124	3	124	9	125 ± 1.8	8 ± 5.0	4
0	116	0	112	0	121	0	115	0	116 ± 3.8	0 ± 0.0	4
15	131	15	103	-10	132	11	106	-9	118 ± 15.9	2 ± 12.9	4
30	118	2	92	-20	107	-14	115	0	108 ± 11.3	-8 ± 10.5	4
45	114	-2	119	6	113	-8	114	-1	115 ± 2.6	-1 ± 6.0	4
60	121	5	117	5	129	8	127	13	124 ± 5.4	8 ± 3.6	4
75	101	-15	107	-5	114	-7	115	0	109 ± 6.7	-7 ± 6.5	4
90	109	-7	98	-14	111	-10	103	-12	105 ± 5.8	-11 ± 2.8	4
105	113	-3	98	-15	117	-4	99	-16	107 ± 9.8	-10 ± 6.7	4
120	110	-6	100	-12	127	6	120	5	114 ± 11.7	-2 ± 8.8	4
135	105	-12	94	-18	113	-8	102	-13	104 ± 7.9	-13 ± 4.2	4
150	99	-17	105	-8	121	0	116	1	110 ± 10.3	-6 ± 8.6	4
165	104	-12	103	-9	138	17	119	4	116 ± 16.3	0 ± 13.2	4
180	116	0	123	10	135	14	118	3	123 ± 8.4	7 ± 6.3	4
195	112	-4	94	-18	121	-1	112	-3	110 ± 11.2	-6 ± 8.0	4
210	115	-2	108	-4	136	15	112	-3	118 ± 12.6	1 ± 9.0	4
225	119	2	105	-7	125	4	115	0	116 ± 8.2	0 ± 4.7	4
240	107	-9	108	-4	124	3	120	5	115 ± 8.6	-1 ± 6.6	4
270	99	-17	99	-13	117	-5	121	7	109 ± 11.6	-7 ± 10.5	4
300	109	-7	91	-21	116	-6	116	1	108 ± 11.5	-8 ± 9.3	4
330	102	-14	104	-8	112	-9	109	-6	107 ± 4.6	-9 ± 3.5	4
360	115	-2	112	0	115	-6	122	8	116 ± 4.5	0 ± 5.7	4
390	111	-5	115	3	132	10	120	5	120 ± 8.8	3 ± 6.4	4
420	100	-16	105	-7	123	1	127	12	114 ± 12.8	-2 ± 11.8	4

Appendix 1.2 – Group 2, Fexinidazole 100 mg/kg PO

Heart rate (beats/min)

Time from	2482		2539		2552		2057		Absolute	Δ from 0	
dose (min)	Abs.	Δ from 0	Abs.	Δ from 0	Abs.	Δ from 0	Abs.	Δ from 0	Mean ± S.D.	Mean ± S.D.	N
-60	68	-19	83	-32	123	5	70	-22	86 ± 25.8	-17 ± 15.8	4
-50	73	-14	109	-7	112	-6	84	-8	94 ± 19.2	-9 ± 3.4	4
-40	108	22	125	9	126	7	107	15	117 ± 10.2	14 ± 6.6	4
-30	76	-10	134	18	115	-4	79	-12	101 ± 27.8	-2 ± 13.8	4
-20	92	6	95	-21	103	-15	77	-15	92 ± 11.2	-11 ± 11.7	4
-10	100	14	149	33	131	12	134	42	128 ± 20.3	25 ± 14.4	4
0	86	0	116	0	118	0	92	0	103 ± 16.4	0 ± 0.0	4
15	99	13	115	0	129	11	124	32	117 ± 13.2	14 ± 13.6	4
30	121	35	107	-9	104	-15	99	8	108 ± 9.4	5 ± 22.3	4
45	113	27	151	35	127	9	139	47	132 ± 16.2	29 ± 16.1	4
60	106	20	164	48	152	34	121	29	136 ± 26.9	33 ± 11.9	4
75	109	23	141	25	125	7	147	55	131 ± 16.9	28 ± 20.2	4
90	76	-10	132	16	117	-1	105	13	107 ± 23.9	5 ± 12.3	4
105	95	9	112	-4	115	-4	101	10	106 ± 9.2	3 ± 7.5	4
120	77	-9	114	-1	117	-1	103	11	103 ± 18.1	0 ± 8.0	4
135	74	-12	81	-35	103	-15	90	-2	87 ± 12.8	-16 ± 13.9	4
150	91	5	113	-3	99	-19	86	-6	97 ± 11.6	-6 ± 9.9	4
165	77	-10	139	24	112	-6	93	1	105 ± 27.0	2 ± 14.8	4
180	111	25	119	3	107	-11	124	32	115 ± 7.3	12 ± 19.7	4
195	84	-3	111	-5	97	-21	94	2	96 ± 11.3	-7 ± 9.9	4
210	78	-8	94	-22	123	5	99	7	99 ± 18.7	-4 ± 13.5	4
225	83	-3	86	-30	114	-4	142	50	106 ± 27.8	3 ± 33.8	4
240	69	-17	94	-22	105	-13	87	-5	89 ± 15.1	-14 ± 7.2	4
270	67	-19	79	-37	100	-19	102	10	87 ± 16.9	-16 ± 19.4	4
300	74	-13	73	-42	113	-5	104	12	91 ± 20.6	-12 ± 22.9	4
330	73	-13	73	-42	107	-11	113	22	92 ± 21.6	-11 ± 26.1	4
360	78	-9	104	-12	106	-12	130	38	104 ± 21.5	1 ± 24.6	4
390	80	-6	104	-12	122	4	115	23	105 ± 18.7	2 ± 15.6	4
420	75	-11	101	-14	101	-17	131	39	102 ± 23.0	-1 ± 26.8	4

Appendix 1.2 – Group 2, Fexinidazole 100 mg/kg PO

Uncorrected QT interval (msec)

Time from	2482		2539		2552		2057		Absolute	Δ from 0	
dose (min)	Abs.	Δ from 0	Abs.	Δ from 0	Abs.	Δ from 0	Abs.	Δ from 0	Mean ± S.D.	Mean ± S.D.	N
-60	214	14	203	27	178	1	197	4	198 ± 14.9	11 ± 11.5	4
-50	212	12	195	19	179	1	200	7	196 ± 13.9	10 ± 7.5	4
-40	196	-4	168	-7	172	-5	191	-2	182 ± 13.6	-5 ± 2.4	4
-30	192	-8	174	-2	180	2	191	-2	184 ± 8.9	-2 ± 4.1	4
-20	201	1	184	8	181	4	202	9	192 ± 11.1	6 ± 3.6	4
-10	184	-16	132	-44	174	-3	176	-17	166 ± 23.6	-20 ± 17.3	4
0	200	0	176	0	177	0	193	0	186 ± 11.8	0 ± 0.0	4
15	186	-14	200	24	175	-2	191	-2	188 ± 10.4	2 ± 16.1	4
30	173	-27	192	16	185	7	196	3	186 ± 10.2	0 ± 18.8	4
45	185	-15	162	-14	166	-12	186	-7	175 ± 12.6	-12 ± 3.4	4
60	186	-14	146	-30	165	-12	163	-30	165 ± 16.5	-21 ± 9.8	4
75	190	-10	141	-35	175	-2	177	-16	171 ± 20.9	-16 ± 14.1	4
90	203	3	198	22	181	4	195	3	194 ± 9.3	8 ± 9.5	4
105	198	-1	231	55	188	11	191	-2	202 ± 19.4	16 ± 26.7	4
120	204	5	193	17	189	12	187	-6	193 ± 7.8	7 ± 10.0	4
135	214	14	225	50	190	13	200	7	207 ± 15.5	21 ± 19.4	4
150	212	13	212	36	192	15	195	2	203 ± 10.9	16 ± 14.4	4
165	220	20	201	25	192	15	206	13	205 ± 11.7	18 ± 5.5	4
180	195	-4	170	-5	188	11	179	-14	183 ± 10.9	-3 ± 10.4	4
195	206	6	200	24	192	15	199	6	199 ± 5.6	13 ± 8.8	4
210	211	11	236	60	199	22	189	-3	209 ± 20.0	22 ± 27.1	4
225	210	10	232	56	190	13	184	-9	204 ± 21.7	18 ± 27.4	4
240	218	18	221	45	187	10	213	20	210 ± 15.4	23 ± 15.3	4
270	230	30	258	82	202	25	206	13	224 ± 25.8	37 ± 30.6	4
300	236	36	225	49	204	27	213	20	219 ± 14.0	33 ± 12.6	4
330	235	35	261	85	207	29	208	15	228 ± 25.8	41 ± 30.5	4
360	216	16	193	17	194	17	184	-9	197 ± 13.5	10 ± 12.7	4
390	216	17	201	26	182	4	194	1	198 ± 14.6	12 ± 11.3	4
420	221	21	225	49	193	16	191	-2	208 ± 17.9	21 ± 21.1	4

Appendix 1.2 – Group 2, Fexinidazole 100 mg/kg PO

Corrected QT interval (covariate analysis, QTca) (msec)

Time from	2482		2539		2552		2057		Absolute	Δ from 0	
dose (min)	Abs.	Δ from 0	Abs.	Δ from 0	Abs.	Δ from 0	Abs.	Δ from 0	Mean ± S.D.	Mean ± S.D.	N
-60	194	3	193	13	182	-1	178	-7	187 ± 8.0	2 ± 8.7	4
-50	196	5	198	18	182	0	195	9	193 ± 7.1	8 ± 7.8	4
-40	199	8	177	-2	181	-2	192	6	187 ± 10.3	3 ± 5.4	4
-30	178	-14	186	6	185	3	176	-10	181 ± 5.1	-4 ± 9.6	4
-20	197	5	180	1	182	-1	187	1	186 ± 7.3	1 ± 2.5	4
-10	184	-8	144	-36	183	1	187	1	174 ± 20.6	-10 ± 17.4	4
0	191	0	180	0	183	0	186	0	185 ± 5.1	0 ± 0.0	4
15	185	-6	207	28	185	3	199	13	194 ± 10.9	9 ± 14.6	4
30	180	-11	190	10	185	2	195	9	187 ± 6.4	2 ± 9.9	4
45	190	-1	177	-2	175	-8	200	15	186 ± 12.0	1 ± 9.7	4
60	188	-4	163	-17	182	-1	169	-17	175 ± 11.3	-9 ± 8.4	4
75	193	2	152	-27	184	2	192	7	181 ± 19.3	-4 ± 15.5	4
90	190	-2	209	30	187	5	195	9	195 ± 9.9	11 ± 13.6	4
105	194	3	233	54	193	10	192	6	203 ± 20.2	18 ± 23.9	4
120	191	0	195	15	196	13	186	0	192 ± 4.3	7 ± 8.3	4
135	197	5	211	32	190	7	192	6	198 ± 9.6	13 ± 12.8	4
150	206	15	217	38	189	7	186	0	200 ± 14.8	15 ± 16.5	4
165	207	15	214	34	196	13	197	11	203 ± 8.6	18 ± 10.8	4
180	200	9	176	-4	191	8	186	0	188 ± 10.1	4 ± 6.1	4
195	197	5	204	25	187	4	193	7	195 ± 7.3	10 ± 9.7	4
210	199	7	227	48	203	21	186	0	204 ± 17.2	19 ± 20.9	4
225	201	10	225	46	194	11	199	13	205 ± 13.8	20 ± 17.0	4
240	200	9	215	35	189	6	203	17	202 ± 10.5	17 ± 12.9	4
270	210	18	242	63	198	16	206	20	214 ± 19.3	29 ± 22.3	4
300	216	25	206	27	208	25	212	26	211 ± 4.5	26 ± 0.9	4
330	217	26	240	61	207	25	210	24	219 ± 15.0	34 ± 17.9	4
360	201	10	194	15	196	13	195	9	197 ± 3.1	12 ± 2.7	4
390	203	11	202	22	189	7	198	12	198 ± 6.2	13 ± 6.7	4
420	206	15	224	44	193	10	203	17	206 ± 12.9	22 ± 15.4	4

Appendix 1.2 – Group 2, Fexinidazole 100 mg/kg PO

PR interval (msec)

Time from	2482		2539		2552		2057		Absolute	Δ from 0	
dose (min)	Abs.	Δ from 0	Abs.	Δ from 0	Abs.	Δ from 0	Abs.	Δ from 0	Mean ± S.D.	Mean ± S.D.	N
-60	108	5	97	6	89	-1	86	-3	95 ± 9.9	2 ± 4.3	4
-50	108	5	98	7	89	-2	90	1	96 ± 9.2	3 ± 3.8	4
-40	98	-5	89	-3	93	2	89	1	92 ± 4.2	-1 ± 3.3	4
-30	100	-3	87	-5	94	4	87	-2	92 ± 6.3	-1 ± 3.8	4
-20	108	4	96	4	88	-2	94	5	96 ± 8.1	3 ± 3.5	4
-10	97	-6	82	-10	89	-2	86	-2	89 ± 6.2	-5 ± 3.8	4
0	103	0	92	0	90	0	89	0	93 ± 6.6	0 ± 0.0	4
15	101	-2	93	2	98	8	92	4	96 ± 4.1	3 ± 4.1	4
30	95	-8	95	4	93	3	87	-2	93 ± 3.8	-1 ± 5.5	4
45	101	-3	85	-7	89	-2	85	-4	90 ± 7.5	-4 ± 2.3	4
60	99	-4	83	-9	91	1	82	-7	89 ± 8.1	-5 ± 4.0	4
75	98	-5	89	-2	92	2	88	-1	92 ± 4.4	-2 ± 2.9	4
90	107	4	99	7	95	4	93	4	98 ± 6.4	5 ± 1.5	4
105	101	-2	100	8	90	0	89	1	95 ± 6.0	2 ± 4.4	4
120	106	2	96	4	100	10	86	-2	97 ± 8.2	4 ± 5.1	4
135	106	3	105	13	92	1	92	3	99 ± 8.0	5 ± 5.4	4
150	104	1	104	13	96	5	91	2	99 ± 6.7	5 ± 5.1	4
165	105	2	100	8	93	2	95	6	98 ± 5.4	5 ± 2.9	4
180	105	1	101	9	96	5	89	0	97 ± 6.8	4 ± 3.9	4
195	101	-2	99	7	93	2	93	4	96 ± 4.3	3 ± 3.8	4
210	108	5	100	8	98	7	89	0	99 ± 7.9	5 ± 3.6	4
225	112	9	103	12	96	6	83	-6	98 ± 12.3	5 ± 7.7	4
240	111	8	111	19	96	5	100	12	104 ± 7.6	11 ± 5.9	4
270	111	8	97	5	96	6	95	6	100 ± 7.6	6 ± 1.2	4
300	104	1	108	16	95	5	92	4	100 ± 7.5	6 ± 6.7	4
330	104	0	110	18	94	4	91	3	100 ± 8.5	6 ± 8.1	4
360	105	1	101	9	90	0	84	-5	95 ± 9.6	1 ± 5.8	4
390	106	2	100	9	90	0	88	0	96 ± 8.2	3 ± 4.2	4
420	101	-2	99	7	95	4	85	-3	95 ± 6.8	1 ± 4.9	4

Appendix 1.2 – Group 2, Fexinidazole 100 mg/kg PO

QRS interval (msec)

Time from	2482		2539		2552		2057		Absolute	Δ from 0	
dose (min)	Abs.	Δ from 0	Abs.	Δ from 0	Abs.	Δ from 0	Abs.	Δ from 0	Mean ± S.D.	Mean ± S.D.	N
-60	40	1	38	-1	37	1	39	-1	38 ± 1.4	0 ± 0.8	4
-50	40	1	42	3	37	1	40	1	40 ± 1.9	1 ± 1.2	4
-40	40	0	39	0	37	1	39	0	39 ± 1.2	0 ± 0.5	4
-30	40	1	37	-2	37	1	41	2	39 ± 2.2	0 ± 1.6	4
-20	39	0	41	3	36	0	39	0	39 ± 2.2	0 ± 1.5	4
-10	37	-3	35	-3	33	-3	38	-1	36 ± 1.9	-3 ± 0.9	4
0	39	0	39	0	36	0	39	0	38 ± 1.4	0 ± 0.0	4
15	38	-1	36	-2	38	2	39	-1	38 ± 1.2	-1 ± 1.8	4
30	38	-1	41	2	37	1	39	0	39 ± 1.6	0 ± 1.4	4
45	38	-1	36	-3	38	1	40	1	38 ± 1.9	0 ± 2.0	4
60	38	-2	37	-1	37	0	37	-3	37 ± 0.6	-1 ± 1.3	4
75	38	-1	39	1	36	0	38	-1	38 ± 1.2	0 ± 1.0	4
90	39	0	38	0	36	0	40	1	38 ± 1.7	0 ± 0.4	4
105	39	0	53	14	37	1	39	0	42 ± 7.3	4 ± 7.0	4
120	40	0	41	2	38	1	41	2	40 ± 1.5	1 ± 0.8	4
135	37	-2	41	2	39	3	39	0	39 ± 1.6	1 ± 2.3	4
150	39	-1	47	8	38	2	39	0	41 ± 4.0	2 ± 4.0	4
165	40	1	40	2	39	3	42	3	40 ± 1.4	2 ± 1.0	4
180	38	-1	39	0	40	3	39	0	39 ± 0.6	1 ± 1.9	4
195	39	0	41	3	37	0	42	3	40 ± 2.6	1 ± 1.8	4
210	39	0	43	4	39	3	40	1	40 ± 1.8	2 ± 2.0	4
225	39	0	48	10	37	0	40	1	41 ± 5.1	3 ± 4.8	4
240	40	1	40	2	37	1	42	3	40 ± 2.0	2 ± 1.1	4
270	50	10	47	8	36	0	42	3	44 ± 5.8	5 ± 4.7	4
300	58	19	40	2	38	1	43	4	45 ± 9.2	6 ± 8.4	4
330	39	0	42	4	37	1	41	2	40 ± 2.3	2 ± 1.6	4
360	41	2	39	1	36	0	40	1	39 ± 2.1	1 ± 0.7	4
390	34	-6	40	1	36	0	41	2	38 ± 3.6	-1 ± 3.6	4
420	40	1	43	5	35	-1	41	1	40 ± 3.3	2 ± 2.4	4

Appendix 1.2 – Group 2, Fexinidazole 100 mg/kg PO

RR interval (msec)

Time from	2482		2539		2552		2057		Absolute	Δ from 0	
dose (min)	Abs.	Δ from 0	Abs.	Δ from 0	Abs.	Δ from 0	Abs.	Δ from 0	Mean ± S.D.	Mean ± S.D.	N
-60	909	173	741	190	548	19	930	204	782 ± 177.7	146 ± 85.7	4
-50	840	104	564	13	549	20	673	-54	656 ± 134.5	21 ± 64.5	4
-40	553	-183	482	-68	481	-48	586	-141	525 ± 52.6	-110 ± 62.9	4
-30	846	110	454	-97	524	-4	866	140	673 ± 214.0	37 ± 108.6	4
-20	665	-71	655	104	591	63	846	119	689 ± 109.4	54 ± 86.8	4
-10	605	-132	408	-143	478	-50	457	-269	487 ± 83.7	-148 ± 90.4	4
0	736	0	551	0	528	0	726	0	635 ± 111.2	0 ± 0.0	4
15	615	-121	517	-34	469	-59	502	-224	526 ± 62.9	-110 ± 84.6	4
30	502	-234	627	76	599	70	618	-109	586 ± 57.2	-49 ± 150.2	4
45	534	-203	404	-147	477	-51	430	-296	461 ± 57.1	-174 ± 102.7	4
60	580	-157	370	-181	394	-134	513	-213	464 ± 99.1	-171 ± 33.7	4
75	555	-181	427	-123	483	-45	415	-311	470 ± 63.9	-165 ± 112.3	4
90	797	61	470	-81	518	-10	604	-122	597 ± 144.4	-38 ± 80.5	4
105	657	-79	568	18	537	9	594	-133	589 ± 51.1	-46 ± 72.2	4
120	809	73	574	24	517	-12	609	-117	627 ± 127.2	-8 ± 80.7	4
135	861	124	794	243	600	71	715	-11	742 ± 112.1	107 ± 106.3	4
150	680	-56	539	-12	636	108	737	11	648 ± 83.9	13 ± 69.4	4
165	791	55	455	-96	556	28	726	0	632 ± 154.1	-3 ± 65.6	4
180	541	-196	522	-28	562	33	503	-223	532 ± 25.0	-104 ± 125.4	4
195	730	-6	547	-3	680	152	677	-49	659 ± 78.1	23 ± 88.2	4
210	780	44	705	154	546	18	650	-76	670 ± 98.5	35 ± 94.7	4
225	723	-13	684	133	549	21	424	-302	595 ± 136.1	-40 ± 185.3	4
240	863	126	686	135	576	47	743	17	717 ± 119.5	81 ± 58.4	4
270	898	161	793	242	648	120	600	-126	735 ± 136.0	99 ± 158.5	4
300	877	140	870	320	552	23	607	-119	726 ± 171.3	91 ± 185.7	4
330	848	111	866	315	590	62	583	-144	721 ± 156.2	86 ± 188.3	4
360	817	81	581	30	578	50	468	-259	611 ± 147.3	-25 ± 157.5	4
390	800	63	594	43	503	-25	545	-182	610 ± 131.7	-25 ± 111.1	4
420	807	71	614	63	597	69	467	-259	621 ± 140.1	-14 ± 163.5	4

Appendix 1.2 – Group 2, Fexinidazole 100 mg/kg PO

Body temperature (°C)

Time from	2482		2539		2552		2057		Absolute	Δ from 0	
dose (min)	Abs.	Δ from 0	Abs.	Δ from 0	Abs.	Δ from 0	Abs.	Δ from 0	Mean ± S.D.	Mean ± S.D.	N
-60	38.1	-0.2	38.2	-0.1	39.1	0.0	37.3	-0.3	38.2 ± 0.75	-0.2 ± 0.1	4
-50	38.1	-0.1	38.2	-0.1	39.2	0.0	37.5	-0.1	38.3 ± 0.70	-0.1 ± 0.1	4
-40	38.1	-0.2	38.3	-0.1	39.2	0.0	37.5	-0.1	38.3 ± 0.71	-0.1 ± 0.1	4
-30	38.4	0.1	38.4	0.0	39.2	0.0	37.6	0.0	38.4 ± 0.67	0.0 ± 0.1	4
-20	38.4	0.2	38.4	0.0	39.2	0.0	37.8	0.2	38.4 ± 0.58	0.1 ± 0.1	4
-10	38.4	0.2	38.6	0.3	39.2	0.0	37.9	0.3	38.5 ± 0.53	0.2 ± 0.1	4
0	38.2	0.0	38.3	0.0	39.2	0.0	37.6	0.0	38.3 ± 0.65	0.0 ± 0.0	4
15	39.4	1.2	39.2	0.8	39.2	0.0	38.4	0.8	39.0 ± 0.47	0.7 ± 0.5	4
30	39.5	1.2	38.8	0.4	39.2	0.0	38.0	0.5	38.9 ± 0.62	0.5 ± 0.5	4
45	39.4	1.1	39.1	0.8	39.1	-0.1	38.3	0.8	39.0 ± 0.44	0.6 ± 0.5	4
60	39.1	0.8	39.0	0.7	39.1	0.0	38.4	0.8	38.9 ± 0.37	0.6 ± 0.4	4
75	39.0	0.8	39.1	0.8	39.2	0.0	38.7	1.1	39.0 ± 0.24	0.7 ± 0.5	4
90	39.0	0.7	39.2	0.8	39.4	0.2	38.6	1.0	39.0 ± 0.32	0.7 ± 0.4	4
105	38.5	0.3	38.6	0.2	39.2	0.0	38.2	0.6	38.6 ± 0.39	0.3 ± 0.3	4
120	38.7	0.5	38.3	-0.1	39.0	-0.2	38.1	0.5	38.5 ± 0.41	0.2 ± 0.4	4
135	38.7	0.4	38.2	-0.1	38.9	-0.3	38.2	0.6	38.5 ± 0.35	0.2 ± 0.4	4
150	38.5	0.2	38.2	-0.1	38.9	-0.3	38.2	0.6	38.5 ± 0.30	0.1 ± 0.4	4
165	38.3	0.1	38.2	-0.1	38.9	-0.2	38.3	0.7	38.4 ± 0.33	0.1 ± 0.4	4
180	38.2	0.0	38.2	-0.2	38.8	-0.4	38.5	0.9	38.4 ± 0.27	0.1 ± 0.6	4
195	38.3	0.0	38.5	0.2	38.7	-0.5	38.4	0.8	38.5 ± 0.18	0.1 ± 0.5	4
210	38.3	0.0	38.4	0.0	38.8	-0.3	38.0	0.4	38.4 ± 0.35	0.0 ± 0.3	4
225	38.3	0.1	38.4	0.1	38.8	-0.4	38.1	0.5	38.4 ± 0.31	0.1 ± 0.4	4
240	38.3	0.1	38.3	-0.1	39.0	-0.2	38.1	0.6	38.4 ± 0.37	0.1 ± 0.3	4
270	38.3	0.1	38.2	-0.2	39.1	-0.1	38.3	0.7	38.5 ± 0.42	0.1 ± 0.4	4
300	38.2	-0.1	38.3	0.0	38.7	-0.5	37.9	0.3	38.3 ± 0.32	-0.1 ± 0.3	4
330	38.5	0.2	38.1	-0.3	38.9	-0.3	37.9	0.3	38.3 ± 0.44	0.0 ± 0.3	4
360	37.8	-0.4	38.0	-0.3	39.0	-0.2	38.1	0.5	38.2 ± 0.52	-0.1 ± 0.4	4
390	37.9	-0.3	38.0	-0.4	38.7	-0.5	37.9	0.3	38.1 ± 0.40	-0.2 ± 0.4	4
420	38.4	0.1	38.0	-0.3	38.8	-0.4	37.7	0.1	38.2 ± 0.46	-0.1 ± 0.3	4

Appendix 1.3 – Group 3, Fexinidazole 300 mg/kg PO

Systolic blood pressure (mm Hg)

Time from	2482		2539		2552		2057		Absolute	Δ from 0	
dose (min)	Abs.	Δ from 0	Abs.	Δ from 0	Abs.	Δ from 0	Abs.	Δ from 0	Mean ± S.D.	Mean ± S.D.	N
-60	159	-4	151	-8	149	5	141	-3	150 ± 7.4	-3 ± 5.8	4
-50	154	-8	148	-12	139	-5	149	6	148 ± 6.6	-5 ± 7.5	4
-40	170	7	194	34	148	4	142	-1	164 ± 23.6	11 ± 15.9	4
-30	161	-2	153	-7	138	-6	146	2	149 ± 9.9	-3 ± 4.2	4
-20	157	-6	142	-18	144	1	145	1	147 ± 6.8	-5 ± 9.1	4
-10	175	12	171	12	143	0	140	-4	157 ± 18.3	5 ± 8.2	4
0	163	0	160	0	143	0	144	0	152 ± 10.2	0 ± 0.0	4
15	163	0	154	-6	143	-1	151	7	153 ± 8.4	0 ± 5.4	4
30	163	0	124	-35	144	1	149	5	145 ± 16.0	-7 ± 18.9	4
45	155	-8	143	-17	156	13	144	1	150 ± 6.8	-3 ± 12.4	4
60	151	-11	156	-4	151	7	152	8	153 ± 2.4	0 ± 9.3	4
75	156	-7	156	-4	146	2	149	5	151 ± 5.0	-1 ± 5.5	4
90	153	-9	160	0	151	8	151	7	154 ± 4.4	1 ± 7.8	4
105	149	-14	149	-11	140	-3	143	-1	145 ± 4.2	-7 ± 6.3	4
120	143	-19	137	-23	137	-7	138	-6	139 ± 3.1	-14 ± 8.5	4
135	146	-17	137	-23	132	-11	147	3	140 ± 6.9	-12 ± 10.9	4
150	147	-15	144	-16	150	7	135	-9	144 ± 6.4	-8 ± 10.6	4
165	148	-15	145	-15	155	12	148	4	149 ± 4.4	-3 ± 13.4	4
180	154	-9	149	-11	149	5	150	6	150 ± 2.4	-2 ± 8.9	4
195	154	-9	141	-18	156	13	141	-2	148 ± 7.9	-4 ± 13.0	4
210	157	-6	144	-16	158	14	143	-1	150 ± 7.9	-2 ± 12.5	4
225	161	-1	141	-19	162	19	152	8	154 ± 9.8	2 ± 15.8	4
240	154	-8	155	-5	156	12	140	-4	151 ± 7.5	-1 ± 9.1	4
270	136	-27	128	-32	155	12	154	11	143 ± 13.5	-9 ± 23.4	4
300	145	-18	124	-36	161	18	148	4	144 ± 15.4	-8 ± 23.7	4
330	143	-20	140	-20	172	29	144	0	150 ± 15.2	-3 ± 23.2	4
360	145	-17	138	-22	157	14	154	10	149 ± 8.9	-4 ± 18.6	4
390	146	-17	132	-28	158	15	142	-2	145 ± 10.9	-8 ± 18.7	4
420	146	-17	122	-38	145	2	139	-5	138 ± 11.1	-14 ± 17.4	4

Appendix 1.3 – Group 3, Fexinidazole 300 mg/kg PO

Diastolic blood pressure (mm Hg)

Time from	2482		2539		2552		2057		Absolute	Δ from 0	
dose (min)	Abs.	Δ from 0	Abs.	Δ from 0	Abs.	Δ from 0	Abs.	Δ from 0	Mean ± S.D.	Mean ± S.D.	N
-60	85	-3	81	-6	92	-1	89	-2	87 ± 4.7	-3 ± 2.2	4
-50	83	-5	81	-6	86	-7	93	2	86 ± 5.2	-4 ± 3.9	4
-40	91	3	111	24	102	9	90	-1	98 ± 9.7	8 ± 11.0	4
-30	87	-2	84	-3	85	-8	96	4	88 ± 5.5	-2 ± 5.0	4
-20	86	-3	75	-12	98	5	94	2	88 ± 10.0	-2 ± 7.5	4
-10	99	11	91	4	94	2	87	-4	93 ± 5.2	3 ± 6.2	4
0	89	0	87	0	93	0	91	0	90 ± 2.6	0 ± 0.0	4
15	106	17	87	0	96	3	101	10	98 ± 8.1	8 ± 7.7	4
30	105	17	67	-21	93	1	99	7	91 ± 17.0	1 ± 15.8	4
45	93	5	77	-10	95	2	94	2	90 ± 8.5	0 ± 6.6	4
60	94	6	87	0	101	8	106	14	97 ± 8.2	7 ± 6.0	4
75	95	6	86	-1	100	7	102	11	96 ± 6.9	6 ± 4.7	4
90	89	0	91	3	89	-4	100	9	92 ± 5.4	2 ± 5.2	4
105	87	-2	87	-1	91	-2	98	6	90 ± 5.1	0 ± 3.7	4
120	84	-5	76	-11	85	-8	96	5	85 ± 8.2	-5 ± 6.7	4
135	85	-4	78	-9	90	-2	95	4	87 ± 7.7	-3 ± 5.5	4
150	84	-5	84	-3	95	3	87	-4	88 ± 5.5	-2 ± 3.5	4
165	86	-3	83	-4	108	15	100	9	94 ± 11.7	4 ± 9.2	4
180	83	-5	83	-4	96	3	98	7	90 ± 8.2	0 ± 5.9	4
195	87	-1	71	-16	99	7	91	0	87 ± 11.8	-3 ± 9.4	4
210	90	1	73	-14	107	14	92	1	90 ± 14.0	0 ± 11.7	4
225	85	-3	72	-15	110	18	96	4	91 ± 16.1	1 ± 13.6	4
240	83	-6	82	-5	108	16	89	-2	91 ± 12.2	1 ± 10.1	4
270	82	-6	73	-14	96	3	111	20	91 ± 16.7	1 ± 14.6	4
300	89	1	68	-19	113	20	101	10	93 ± 19.0	3 ± 16.5	4
330	87	-2	84	-3	122	29	98	7	98 ± 17.2	8 ± 14.9	4
360	87	-1	81	-6	105	12	101	10	94 ± 11.2	4 ± 8.6	4
390	86	-3	78	-9	109	16	99	8	93 ± 13.7	3 ± 11.2	4
420	95	7	74	-14	101	8	97	6	92 ± 12.5	2 ± 10.4	4

Appendix 1.3 – Group 3, Fexinidazole 300 mg/kg PO

Mean blood pressure (mm Hg)

Time from	2482		2539		2552		2057		Absolute	Δ from 0	
dose (min)	Abs.	Δ from 0	Abs.	Δ from 0	Abs.	Δ from 0	Abs.	Δ from 0	Mean ± S.D.	Mean ± S.D.	N
-60	113	-2	104	-7	114	1	105	-3	109 ± 5.1	-3 ± 3.1	4
-50	110	-6	102	-9	107	-6	112	3	107 ± 4.3	-4 ± 5.3	4
-40	119	4	139	28	122	9	108	-1	122 ± 12.8	10 ± 12.5	4
-30	113	-2	105	-6	106	-7	112	4	109 ± 4.4	-3 ± 5.1	4
-20	111	-4	96	-14	116	3	108	0	108 ± 8.2	-4 ± 7.4	4
-10	125	10	118	8	113	0	105	-4	115 ± 8.5	3 ± 6.2	4
0	115	0	111	0	113	0	108	0	112 ± 3.0	0 ± 0.0	4
15	128	13	111	0	114	1	117	9	117 ± 7.5	6 ± 6.2	4
30	127	12	86	-25	114	1	116	8	111 ± 17.6	-1 ± 16.5	4
45	116	1	101	-9	119	7	111	3	112 ± 7.9	0 ± 6.8	4
60	117	2	112	2	121	8	121	13	118 ± 4.2	6 ± 5.6	4
75	119	4	112	1	119	6	119	10	117 ± 3.5	5 ± 4.0	4
90	112	-3	114	4	112	0	117	9	114 ± 2.2	2 ± 5.1	4
105	109	-6	110	0	111	-2	113	5	111 ± 1.5	-1 ± 4.3	4
120	104	-11	97	-14	105	-8	111	2	104 ± 5.6	-8 ± 7.1	4
135	106	-9	99	-12	107	-6	112	3	106 ± 5.4	-6 ± 6.6	4
150	107	-8	103	-7	118	5	103	-6	108 ± 6.9	-4 ± 5.9	4
165	107	-8	104	-7	127	14	116	8	113 ± 10.5	2 ± 11.1	4
180	108	-7	104	-7	117	5	116	8	111 ± 6.4	0 ± 7.5	4
195	111	-5	94	-16	123	10	108	0	109 ± 11.6	-3 ± 10.7	4
210	115	0	95	-16	126	13	110	2	112 ± 12.9	0 ± 11.9	4
225	112	-3	94	-17	132	19	115	7	113 ± 15.6	2 ± 15.1	4
240	108	-8	107	-4	127	14	108	0	112 ± 9.9	1 ± 9.6	4
270	102	-13	93	-18	119	6	127	18	110 ± 15.5	-2 ± 16.9	4
300	109	-6	86	-24	133	20	116	8	111 ± 19.2	-1 ± 18.9	4
330	108	-7	103	-8	142	29	113	5	117 ± 17.7	5 ± 17.4	4
360	109	-6	101	-10	124	11	118	10	113 ± 10.2	1 ± 10.8	4
390	109	-6	97	-13	127	14	114	5	112 ± 12.5	0 ± 12.4	4
420	113	-2	90	-21	119	6	112	4	109 ± 12.7	-3 ± 12.1	4

Appendix 1.3 – Group 3, Fexinidazole 300 mg/kg PO

Heart rate (beats/min)

Time from	2482		2539		2552		2057		Absolute	Δ from 0	
dose (min)	Abs.	Δ from 0	Abs.	Δ from 0	Abs.	Δ from 0	Abs.	Δ from 0	Mean ± S.D.	Mean ± S.D.	N
-60	52	-10	56	-13	115	13	84	5	77 ± 29.1	-1 ± 12.4	4
-50	62	0	65	-5	76	-26	63	-16	67 ± 6.4	-12 ± 11.8	4
-40	68	6	72	2	117	15	63	-15	80 ± 25.1	2 ± 12.7	4
-30	55	-7	54	-15	103	0	86	7	75 ± 23.8	-4 ± 9.6	4
-20	55	-7	76	6	105	3	79	0	79 ± 20.6	1 ± 5.7	4
-10	81	18	95	25	98	-4	97	18	93 ± 8.2	14 ± 12.9	4
0	62	0	70	0	102	0	79	0	78 ± 17.4	0 ± 0.0	4
15	103	40	104	34	105	3	76	-3	97 ± 14.1	19 ± 21.9	4
30	115	52	82	12	111	9	97	19	101 ± 15.0	23 ± 20.1	4
45	87	25	99	30	114	11	92	13	98 ± 11.5	20 ± 9.0	4
60	100	38	124	55	125	23	112	33	115 ± 11.7	37 ± 13.3	4
75	98	36	122	52	125	23	128	50	118 ± 13.7	40 ± 13.5	4
90	89	26	103	33	116	14	84	6	98 ± 14.4	20 ± 12.4	4
105	80	17	117	47	116	13	76	-3	97 ± 22.3	19 ± 21.0	4
120	69	7	88	18	109	6	100	21	92 ± 17.0	13 ± 7.7	4
135	63	1	80	10	101	-2	95	16	85 ± 16.8	6 ± 8.4	4
150	68	5	99	29	105	2	73	-6	86 ± 18.4	8 ± 14.9	4
165	76	14	82	13	117	14	92	13	92 ± 17.8	14 ± 0.7	4
180	79	17	89	19	96	-7	69	-10	83 ± 11.7	5 ± 15.2	4
195	62	-1	66	-4	95	-8	78	-1	75 ± 14.9	-3 ± 3.3	4
210	87	25	64	-6	117	15	90	11	90 ± 21.8	11 ± 12.9	4
225	82	20	59	-10	122	19	98	19	90 ± 26.3	12 ± 15.0	4
240	66	4	99	29	116	13	113	34	99 ± 23.0	20 ± 14.2	4
270	62	0	86	16	111	9	89	10	87 ± 20.2	9 ± 6.8	4
300	71	8	70	0	147	44	98	20	96 ± 36.1	18 ± 19.3	4
330	68	5	88	19	134	31	91	12	95 ± 27.7	17 ± 11.0	4
360	64	2	73	4	114	12	92	13	86 ± 21.9	8 ± 5.5	4
390	66	4	96	27	124	21	105	26	98 ± 24.0	19 ± 10.7	4
420	74	12	104	35	139	37	121	43	110 ± 27.7	31 ± 13.5	4

Appendix 1.3 – Group 3, Fexinidazole 300 mg/kg PO

Uncorrected QT interval (msec)

Time from	2482		2539		2552		2057		Absolute	Δ from 0	
dose (min)	Abs.	Δ from 0	Abs.	Δ from 0	Abs.	Δ from 0	Abs.	Δ from 0	Mean ± S.D.	Mean ± S.D.	N
-60	238	18	222	17	195	-6	209	1	216 ± 18.3	7 ± 11.9	4
-50	230	10	198	-8	206	4	209	1	211 ± 13.9	2 ± 7.2	4
-40	198	-23	195	-11	202	1	206	-2	200 ± 5.2	-9 ± 10.7	4
-30	228	7	219	14	204	2	203	-5	213 ± 12.1	5 ± 8.0	4
-20	224	4	210	5	197	-5	217	9	212 ± 11.7	3 ± 5.6	4
-10	205	-16	189	-17	205	4	205	-3	201 ± 8.2	-8 ± 9.9	4
0	220	0	205	0	202	0	208	0	209 ± 8.2	0 ± 0.0	4
15	186	-35	177	-29	192	-9	200	-8	189 ± 9.9	-20 ± 13.5	4
30	184	-37	200	-5	191	-11	207	-1	195 ± 10.2	-14 ± 16.1	4
45	201	-19	176	-29	186	-16	195	-13	190 ± 11.0	-19 ± 7.1	4
60	197	-24	168	-37	182	-20	189	-19	184 ± 12.1	-25 ± 8.3	4
75	197	-23	179	-26	179	-22	193	-15	187 ± 9.3	-22 ± 4.7	4
90	210	-11	199	-7	167	-34	196	-12	193 ± 18.0	-16 ± 12.3	4
105	215	-6	187	-18	181	-20	200	-8	196 ± 14.8	-13 ± 7.1	4
120	226	6	210	5	186	-15	194	-14	204 ± 17.7	-5 ± 11.4	4
135	232	11	213	8	200	-2	205	-3	212 ± 14.0	4 ± 6.9	4
150	234	13	213	8	203	1	228	20	219 ± 14.1	11 ± 8.1	4
165	233	13	209	4	187	-14	207	-1	209 ± 18.9	0 ± 11.4	4
180	216	-4	214	9	191	-10	211	3	208 ± 11.3	-1 ± 8.4	4
195	232	12	210	5	199	-3	214	6	214 ± 13.9	5 ± 6.0	4
210	224	3	206	1	189	-13	211	3	207 ± 14.7	-2 ± 7.7	4
225	235	14	217	12	192	-10	190	-18	208 ± 21.6	-1 ± 16.1	4
240	221	1	200	-5	188	-14	190	-18	200 ± 15.2	-9 ± 8.3	4
270	245	24	213	8	189	-13	189	-19	209 ± 26.4	0 ± 19.7	4
300	248	27	223	18	182	-20	228	20	220 ± 27.6	11 ± 21.0	4
330	247	27	225	20	180	-22	232	24	221 ± 29.0	12 ± 22.8	4
360	253	33	216	11	180	-21	202	-6	213 ± 30.7	4 ± 23.2	4
390	247	27	203	-2	167	-35	219	11	209 ± 33.6	0 ± 26.2	4
420	242	22	194	-12	166	-35	187	-21	198 ± 32.2	-11 ± 24.2	4

Appendix 1.3 – Group 3, Fexinidazole 300 mg/kg PO

Corrected QT interval (covariate analysis, QTca) (msec)

Time from	2482		2539		2552		2057		Absolute	Δ from 0	
dose (min)	Abs.	Δ from 0	Abs.	Δ from 0	Abs.	Δ from 0	Abs.	Δ from 0	Mean ± S.D.	Mean ± S.D.	N
-60	205	8	193	9	200	1	197	3	199 ± 5.1	5 ± 4.0	4
-50	206	9	173	-11	194	-5	188	-5	190 ± 13.6	-3 ± 8.6	4
-40	183	-13	177	-7	208	9	188	-5	189 ± 13.5	-4 ± 9.3	4
-30	198	1	185	2	197	-3	196	2	194 ± 5.8	1 ± 2.2	4
-20	195	-1	191	7	196	-3	196	3	195 ± 2.8	1 ± 4.4	4
-10	192	-4	183	0	201	2	197	3	194 ± 7.6	0 ± 3.2	4
0	197	0	184	0	199	0	194	0	193 ± 6.8	0 ± 0.0	4
15	186	-11	178	-6	191	-8	186	-8	185 ± 5.4	-8 ± 2.1	4
30	186	-10	189	5	194	-6	202	8	193 ± 6.9	-1 ± 8.6	4
45	194	-2	176	-8	190	-10	189	-5	187 ± 7.9	-6 ± 3.3	4
60	195	-1	177	-7	189	-10	189	-4	188 ± 7.6	-6 ± 3.8	4
75	196	-1	187	3	188	-12	201	7	193 ± 7.0	-1 ± 8.3	4
90	202	6	197	14	172	-27	185	-8	189 ± 13.5	-4 ± 18.0	4
105	202	5	193	9	186	-13	186	-7	192 ± 7.4	-2 ± 10.5	4
120	205	8	202	18	188	-11	191	-3	197 ± 8.2	3 ± 12.9	4
135	205	9	200	16	197	-2	193	-1	199 ± 5.1	5 ± 8.5	4
150	211	15	206	23	200	0	210	16	207 ± 5.2	13 ± 9.5	4
165	213	17	198	14	190	-9	200	6	200 ± 9.7	7 ± 11.8	4
180	203	6	200	17	187	-13	193	-1	196 ± 7.4	2 ± 12.4	4
195	207	10	183	-1	195	-4	203	9	197 ± 10.6	4 ± 7.3	4
210	216	19	192	8	192	-7	209	15	202 ± 11.9	9 ± 11.6	4
225	223	27	196	12	201	1	186	-8	201 ± 15.9	8 ± 15.0	4
240	200	3	195	11	193	-6	195	1	196 ± 2.7	2 ± 7.0	4
270	215	18	203	19	192	-7	183	-11	198 ± 14.0	5 ± 16.3	4
300	225	29	202	19	198	-2	221	27	212 ± 13.6	18 ± 14.0	4
330	225	28	217	33	191	-9	222	28	214 ± 15.5	20 ± 19.3	4
360	228	31	201	17	183	-16	195	2	202 ± 18.7	8 ± 20.4	4
390	226	29	199	15	174	-26	218	25	204 ± 23.2	11 ± 25.0	4
420	222	25	192	8	180	-20	194	1	197 ± 17.9	4 ± 18.8	4

Appendix 1.3 – Group 3, Fexinidazole 300 mg/kg PO

PR interval (msec)

Time from	2482		2539		2552		2057		Absolute	Δ from 0	
dose (min)	Abs.	Δ from 0	Abs.	Δ from 0	Abs.	Δ from 0	Abs.	Δ from 0	Mean ± S.D.	Mean ± S.D.	N
-60	109	2	104	3	92	3	94	4	100 ± 8.0	3 ± 0.8	4
-50	108	1	101	1	86	-3	90	-1	96 ± 10.3	0 ± 1.7	4
-40	108	1	98	-2	89	1	90	-1	96 ± 8.9	0 ± 1.3	4
-30	108	1	100	0	89	0	87	-4	96 ± 10.2	-1 ± 2.3	4
-20	107	0	101	1	88	-1	91	1	97 ± 8.8	0 ± 0.9	4
-10	103	-4	97	-3	87	-1	92	1	95 ± 6.7	-2 ± 2.4	4
0	107	0	100	0	88	0	91	0	97 ± 8.7	0 ± 0.0	4
15	91	-16	96	-5	89	1	86	-5	90 ± 4.0	-6 ± 7.3	4
30	89	-18	98	-2	90	2	86	-4	91 ± 5.2	-6 ± 8.4	4
45	95	-12	96	-4	86	-3	87	-3	91 ± 5.4	-5 ± 4.3	4
60	89	-18	89	-12	87	-1	84	-7	87 ± 2.4	-9 ± 7.2	4
75	94	-13	89	-11	87	-1	84	-6	89 ± 4.0	-8 ± 5.4	4
90	92	-15	91	-9	88	0	89	-1	90 ± 2.1	-6 ± 6.7	4
105	99	-8	97	-3	90	2	92	2	95 ± 4.3	-2 ± 4.6	4
120	103	-4	107	7	92	3	95	4	99 ± 7.4	3 ± 4.7	4
135	107	0	115	14	97	9	89	-2	102 ± 11.2	5 ± 7.6	4
150	107	0	108	8	97	8	101	11	103 ± 5.4	7 ± 4.6	4
165	108	1	98	-2	92	4	94	4	98 ± 7.0	2 ± 3.0	4
180	101	-6	99	-1	96	7	93	3	97 ± 3.6	1 ± 5.6	4
195	105	-2	102	1	96	8	95	4	99 ± 4.8	3 ± 4.1	4
210	102	-5	96	-4	91	3	89	-2	95 ± 6.1	-2 ± 3.4	4
225	100	-7	99	-2	95	6	86	-5	95 ± 6.5	-2 ± 5.8	4
240	101	-6	93	-7	92	4	89	-2	94 ± 5.1	-3 ± 5.1	4
270	114	7	107	7	92	4	95	4	102 ± 10.5	5 ± 1.8	4
300	109	2	104	4	87	-1	94	3	98 ± 9.8	2 ± 2.2	4
330	109	2	101	0	90	2	83	-7	96 ± 11.4	-1 ± 4.4	4
360	113	6	98	-3	84	-5	86	-5	95 ± 13.3	-2 ± 4.9	4
390	106	-1	91	-9	86	-3	81	-10	91 ± 10.8	-6 ± 4.3	4
420	103	-4	88	-12	89	1	80	-11	90 ± 9.7	-7 ± 6.2	4

Appendix 1.3 – Group 3, Fexinidazole 300 mg/kg PO

QRS interval (msec)

Time from	2482		2539		2552		2057		Absolute	Δ from 0	
dose (min)	Abs.	Δ from 0	Abs.	Δ from 0	Abs.	Δ from 0	Abs.	Δ from 0	Mean ± S.D.	Mean ± S.D.	N
-60	40	0	40	-2	38	-1	41	1	40 ± 1.2	0 ± 1.0	4
-50	40	0	44	2	39	0	40	0	41 ± 2.0	0 ± 1.0	4
-40	40	-1	44	2	39	0	41	1	41 ± 2.2	0 ± 1.1	4
-30	39	-1	39	-2	38	0	40	-1	39 ± 0.5	-1 ± 0.8	4
-20	43	3	41	-1	39	0	41	0	41 ± 1.7	1 ± 1.7	4
-10	39	-2	43	1	40	1	41	0	41 ± 1.8	0 ± 1.3	4
0	40	0	42	0	39	0	41	0	40 ± 1.2	0 ± 0.0	4
15	38	-2	45	3	38	-1	42	1	41 ± 3.1	0 ± 2.1	4
30	39	-1	47	5	39	0	40	0	41 ± 3.8	1 ± 2.8	4
45	41	0	43	1	38	-1	41	0	41 ± 1.9	0 ± 0.8	4
60	40	0	43	1	39	0	42	1	41 ± 2.0	1 ± 1.0	4
75	40	-1	43	1	34	-5	41	1	39 ± 4.1	-1 ± 2.9	4
90	40	0	46	4	36	-3	40	-1	41 ± 4.1	0 ± 2.9	4
105	41	0	46	5	36	-3	40	-1	41 ± 4.4	0 ± 3.3	4
120	40	-1	45	3	36	-2	39	-1	40 ± 3.4	0 ± 2.2	4
135	41	0	42	0	36	-3	41	1	40 ± 2.7	0 ± 1.7	4
150	42	2	44	2	39	0	41	1	42 ± 2.3	1 ± 1.2	4
165	42	2	44	2	35	-4	42	1	41 ± 3.8	0 ± 2.7	4
180	39	-1	44	2	36	-3	40	0	40 ± 3.1	-1 ± 1.9	4
195	41	1	39	-3	39	0	43	2	40 ± 1.6	0 ± 1.9	4
210	39	-1	40	-2	40	1	41	0	40 ± 0.6	-1 ± 1.4	4
225	41	1	42	0	41	2	39	-1	41 ± 1.0	0 ± 1.3	4
240	39	-1	43	1	37	-2	41	0	40 ± 2.7	0 ± 1.6	4
270	49	9	41	0	38	-1	44	4	43 ± 4.8	3 ± 4.5	4
300	64	24	41	-1	36	-3	44	3	46 ± 12.6	6 ± 12.5	4
330	62	21	42	0	36	-3	44	4	46 ± 10.9	6 ± 10.7	4
360	56	15	42	0	35	-4	44	3	44 ± 8.5	4 ± 8.2	4
390	63	23	43	2	34	-5	43	2	46 ± 12.4	5 ± 12.2	4
420	64	24	43	1	34	-5	40	0	45 ± 12.9	5 ± 12.7	4

Appendix 1.3 – Group 3, Fexinidazole 300 mg/kg PO

RR interval (msec)

Time from	2482		2539		2552		2057		Absolute	Δ from 0	
dose (min)	Abs.	Δ from 0	Abs.	Δ from 0	Abs.	Δ from 0	Abs.	Δ from 0	Mean ± S.D.	Mean ± S.D.	N
-60	1157	163	1112	126	541	-92	766	-56	894 ± 292.9	35 ± 128.1	4
-50	973	-21	1065	80	767	134	940	118	937 ± 124.6	78 ± 69.5	4
-40	836	-157	901	-84	528	-105	892	70	789 ± 176.5	-69 ± 97.7	4
-30	1104	110	1242	257	703	69	704	-119	938 ± 276.9	79 ± 154.5	4
-20	1102	108	914	-72	607	-27	922	100	886 ± 205.5	27 ± 90.3	4
-10	790	-203	677	-308	655	21	709	-114	708 ± 59.3	-151 ± 139.5	4
0	994	0	985	0	633	0	822	0	859 ± 169.6	0 ± 0.0	4
15	600	-394	579	-406	617	-17	823	1	655 ± 113.4	-204 ± 226.7	4
30	562	-432	773	-212	559	-74	665	-157	640 ± 101.7	-219 ± 153.0	4
45	698	-295	599	-386	546	-88	688	-134	633 ± 73.0	-226 ± 138.8	4
60	624	-370	481	-504	506	-127	598	-224	552 ± 69.1	-306 ± 165.2	4
75	618	-376	506	-479	491	-143	501	-321	529 ± 59.7	-330 ± 140.8	4
90	699	-295	616	-369	531	-102	764	-58	652 ± 100.9	-206 ± 149.7	4
105	784	-210	531	-454	535	-99	812	-10	665 ± 153.5	-193 ± 192.2	4
120	922	-72	707	-278	569	-64	650	-172	712 ± 150.7	-146 ± 100.3	4
135	1020	27	795	-191	632	-1	786	-36	808 ± 159.8	-50 ± 97.0	4
150	928	-66	691	-294	640	7	871	49	783 ± 138.7	-76 ± 153.0	4
165	883	-110	764	-221	559	-74	695	-127	725 ± 135.4	-133 ± 62.4	4
180	786	-208	803	-182	670	37	887	65	787 ± 89.3	-72 ± 142.7	4
195	994	0	1097	112	650	17	749	-73	872 ± 208.0	14 ± 76.1	4
210	707	-287	811	-174	550	-84	619	-203	671 ± 113.1	-187 ± 83.8	4
225	748	-245	939	-46	492	-142	659	-163	709 ± 186.5	-149 ± 82.0	4
240	936	-58	677	-308	528	-106	537	-285	669 ± 190.4	-189 ± 125.9	4
270	1053	59	740	-246	557	-76	698	-124	762 ± 209.0	-97 ± 126.0	4
300	905	-89	920	-65	416	-218	678	-144	730 ± 236.8	-129 ± 67.8	4
330	903	-91	706	-280	459	-175	719	-103	697 ± 182.4	-162 ± 86.7	4
360	951	-43	817	-169	555	-78	689	-133	753 ± 169.8	-105 ± 56.0	4
390	892	-102	658	-327	500	-134	603	-219	663 ± 165.8	-196 ± 100.7	4
420	882	-112	620	-366	430	-203	513	-310	611 ± 196.3	-248 ± 112.6	4

Appendix 1.3 – Group 3, Fexinidazole 300 mg/kg PO

Body temperature (°C)

Time from	2482		2539		2552		2057		Absolute	Δ from 0	
dose (min)	Abs.	Δ from 0	Abs.	Δ from 0	Abs.	Δ from 0	Abs.	Δ from 0	Mean ± S.D.	Mean ± S.D.	N
-60	37.6	-0.3	37.8	-0.1	38.6	-0.1	37.4	-0.1	37.8 ± 0.51	-0.1 ± 0.1	4
-50	37.7	-0.2	37.8	0.0	38.5	-0.1	37.2	-0.3	37.8 ± 0.56	-0.1 ± 0.1	4
-40	37.8	-0.1	37.9	0.0	38.6	0.0	37.4	-0.1	37.9 ± 0.52	-0.1 ± 0.1	4
-30	37.9	0.1	37.9	0.0	38.7	0.1	37.6	0.1	38.0 ± 0.46	0.1 ± 0.0	4
-20	38.0	0.2	37.9	0.1	38.7	0.1	37.7	0.2	38.1 ± 0.45	0.1 ± 0.1	4
-10	38.2	0.3	37.9	0.0	38.6	0.0	37.8	0.3	38.1 ± 0.39	0.2 ± 0.2	4
0	37.9	0.0	37.9	0.0	38.6	0.0	37.5	0.0	38.0 ± 0.48	0.0 ± 0.0	4
15	39.3	1.4	38.8	0.9	38.7	0.1	38.0	0.5	38.7 ± 0.54	0.7 ± 0.6	4
30	39.4	1.5	38.5	0.7	38.9	0.2	38.0	0.5	38.7 ± 0.56	0.7 ± 0.5	4
45	39.2	1.3	38.4	0.5	38.9	0.3	38.1	0.6	38.6 ± 0.49	0.7 ± 0.4	4
60	39.1	1.2	38.9	1.1	39.0	0.4	38.5	1.0	38.9 ± 0.29	0.9 ± 0.4	4
75	38.9	1.1	38.7	0.8	39.1	0.5	38.3	0.8	38.8 ± 0.34	0.8 ± 0.2	4
90	38.9	1.0	38.8	0.9	39.1	0.5	38.3	0.8	38.8 ± 0.33	0.8 ± 0.2	4
105	38.8	0.9	38.5	0.7	39.2	0.6	38.3	0.8	38.7 ± 0.40	0.7 ± 0.1	4
120	38.6	0.7	38.4	0.6	39.2	0.6	38.4	0.9	38.6 ± 0.39	0.7 ± 0.1	4
135	38.4	0.5	38.2	0.4	39.2	0.6	38.2	0.7	38.5 ± 0.46	0.5 ± 0.2	4
150	38.3	0.4	38.1	0.3	39.2	0.5	37.9	0.5	38.4 ± 0.55	0.4 ± 0.1	4
165	38.2	0.3	38.2	0.3	39.1	0.5	37.7	0.2	38.3 ± 0.60	0.3 ± 0.1	4
180	38.1	0.2	38.1	0.3	39.2	0.6	37.8	0.3	38.3 ± 0.62	0.3 ± 0.2	4
195	38.1	0.2	38.3	0.4	39.3	0.6	37.9	0.4	38.4 ± 0.60	0.4 ± 0.2	4
210	37.9	0.0	38.5	0.6	39.3	0.7	37.8	0.4	38.4 ± 0.69	0.4 ± 0.3	4
225	38.0	0.1	38.4	0.5	39.3	0.7	38.0	0.5	38.4 ± 0.62	0.5 ± 0.2	4
240	38.2	0.3	38.4	0.5	39.3	0.6	38.1	0.6	38.5 ± 0.54	0.5 ± 0.2	4
270	38.1	0.3	38.4	0.5	39.3	0.7	38.2	0.7	38.5 ± 0.54	0.5 ± 0.2	4
300	38.1	0.2	38.4	0.5	39.4	0.8	38.4	0.9	38.6 ± 0.57	0.6 ± 0.3	4
330	38.1	0.2	38.0	0.1	39.7	1.0	38.6	1.1	38.6 ± 0.77	0.6 ± 0.5	4
360	37.8	0.0	37.9	0.0	40.0	1.3	38.2	0.7	38.5 ± 1.01	0.5 ± 0.7	4
390	37.9	0.1	37.8	0.0	39.8	1.2	38.5	1.0	38.5 ± 0.93	0.6 ± 0.6	4
420	38.0	0.1	37.3	-0.5	40.2	1.5	38.4	1.0	38.5 ± 1.20	0.5 ± 0.9	4

Appendix 1.4 – Group 4, Fexinidazole 1000 mg/kg PO

Systolic blood pressure (mm Hg)

Time from dose (min)	2482		2539		2552		2057		Absolute	Δ from 0	
	Abs.	Δ from 0	Abs.	Δ from 0	Abs.	Δ from 0	Abs.	Δ from 0	Mean ± S.D.	Mean ± S.D.	N
-60	185	23	184	37	165	15	138	-2	168 ± 22.1	18 ± 16.2	4
-50	160	-2	140	-7	146	-4	132	-8	145 ± 11.7	-5 ± 2.7	4
-40	152	-10	139	-8	163	13	135	-4	147 ± 12.5	-2 ± 10.3	4
-30	164	2	144	-4	149	-1	147	8	151 ± 8.8	1 ± 4.8	4
-20	156	-6	135	-12	145	-5	146	7	145 ± 8.5	-4 ± 7.9	4
-10	155	-6	142	-6	133	-17	140	0	142 ± 9.5	-7 ± 7.3	4
0	162	0	147	0	150	0	140	0	150 ± 9.2	0 ± 0.0	4
15	161	-1	159	12	145	-5	133	-7	150 ± 13.2	0 ± 8.3	4
30	173	12	183	36	156	6	142	2	164 ± 18.5	14 ± 15.4	4
45	154	-8	187	39	155	5	150	10	161 ± 17.0	12 ± 20.1	4
60	147	-15	144	-3	155	5	141	2	147 ± 5.8	-3 ± 8.8	4
75	169	7	159	12	168	18	161	21	164 ± 5.0	14 ± 6.5	4
90	155	-7	152	5	169	19	147	7	156 ± 9.6	6 ± 10.6	4
105	155	-7	146	-1	163	13	149	9	153 ± 7.4	4 ± 8.9	4
120	151	-11	146	-1	167	17	134	-6	149 ± 13.4	0 ± 11.9	4
135	153	-9	141	-6	166	16	152	12	153 ± 9.9	3 ± 12.3	4
150	157	-5	145	-3	163	13	155	15	155 ± 7.8	5 ± 10.4	4
165	181	19	136	-12	162	12	160	20	160 ± 18.5	10 ± 14.8	4
180	155	-6	155	8	156	6	156	16	156 ± 0.4	6 ± 9.3	4
195	155	-7	145	-3	175	25	154	14	157 ± 12.8	7 ± 14.9	4
210	157	-5	157	9	177	27	146	6	159 ± 12.9	9 ± 13.0	4
225	151	-10	153	6	170	20	145	5	155 ± 10.9	5 ± 12.4	4
240	156	-6	153	6	157	7	142	2	152 ± 7.2	2 ± 5.9	4
270	144	-18	145	-2	164	14	146	6	150 ± 9.5	0 ± 13.6	4
300	145	-17	138	-9	148	-2	157	17	147 ± 8.0	-3 ± 14.8	4
330	150	-12	134	-13	162	12	156	16	150 ± 12.0	1 ± 15.5	4
360	133	-29	153	6	156	6	156	17	150 ± 11.4	0 ± 20.0	4
390	165	3	162	15	162	12	151	11	160 ± 6.2	10 ± 5.2	4
420	155	-7	181	33	172	22	164	24	168 ± 10.9	18 ± 17.3	4

Appendix 1.4 – Group 4, Fexinidazole 1000 mg/kg PO

Diastolic blood pressure (mm Hg)

Time from	2482		2539		2552		2057		Absolute	Δ from 0	
dose (min)	Abs.	Δ from 0	Abs.	Δ from 0	Abs.	Δ from 0	Abs.	Δ from 0	Mean ± S.D.	Mean ± S.D.	N
-60	102	11	110	26	110	15	85	-6	102 ± 11.8	11 ± 13.0	4
-50	87	-4	81	-3	91	-5	85	-5	86 ± 4.0	-4 ± 1.0	4
-40	86	-5	85	1	106	10	84	-7	90 ± 10.4	0 ± 7.6	4
-30	95	4	80	-4	89	-6	101	10	91 ± 8.7	1 ± 7.4	4
-20	89	-2	74	-10	94	-1	100	9	89 ± 10.9	-1 ± 7.8	4
-10	87	-4	74	-10	82	-13	89	-2	83 ± 6.5	-7 ± 5.2	4
0	91	0	84	0	95	0	91	0	90 ± 4.7	0 ± 0.0	4
15	109	18	98	14	90	-6	86	-5	96 ± 10.3	5 ± 12.3	4
30	96	5	107	23	103	7	96	5	100 ± 5.5	10 ± 8.6	4
45	88	-3	121	37	111	16	96	5	104 ± 14.8	14 ± 17.3	4
60	90	-1	84	0	107	11	98	8	95 ± 9.9	4 ± 6.0	4
75	104	12	95	11	113	18	108	17	105 ± 7.8	14 ± 3.6	4
90	94	3	89	5	111	16	96	5	98 ± 9.4	7 ± 5.7	4
105	92	1	87	3	110	15	99	8	97 ± 10.0	7 ± 6.1	4
120	88	-4	79	-5	117	22	86	-5	92 ± 16.9	2 ± 13.1	4
135	90	-1	81	-3	115	20	98	8	96 ± 14.5	6 ± 10.4	4
150	96	5	83	-1	108	13	105	15	98 ± 11.3	8 ± 7.3	4
165	102	11	71	-13	116	21	111	20	100 ± 19.9	10 ± 15.6	4
180	97	6	94	10	108	13	107	16	102 ± 7.2	11 ± 4.5	4
195	92	1	80	-4	126	31	105	14	101 ± 19.9	10 ± 15.7	4
210	92	1	92	8	118	23	99	8	100 ± 12.3	10 ± 9.1	4
225	88	-3	84	-1	114	19	92	1	94 ± 13.5	4 ± 9.8	4
240	94	2	89	5	108	13	96	5	97 ± 8.1	6 ± 4.4	4
270	89	-3	83	-1	112	17	97	7	95 ± 12.7	5 ± 8.9	4
300	95	4	81	-3	112	17	106	15	99 ± 13.6	8 ± 9.4	4
330	99	8	83	-1	116	21	112	21	102 ± 14.7	12 ± 10.6	4
360	83	-8	91	7	105	9	113	23	98 ± 13.5	8 ± 12.6	4
390	111	20	91	7	114	19	102	12	105 ± 10.6	14 ± 6.4	4
420	99	8	106	22	127	31	124	34	114 ± 13.3	24 ± 11.4	4

Appendix 1.4 – Group 4, Fexinidazole 1000 mg/kg PO

Mean blood pressure (mm Hg)

Time from	2482		2539		2552		2057		Absolute	Δ from 0	
dose (min)	Abs.	Δ from 0	Abs.	Δ from 0	Abs.	Δ from 0	Abs.	Δ from 0	Mean ± S.D.	Mean ± S.D.	N
-60	133	16	140	35	132	15	104	-3	128 ± 16.0	16 ± 15.8	4
-50	114	-4	100	-5	114	-4	101	-6	107 ± 7.5	-5 ± 1.0	4
-40	110	-7	96	-9	130	12	100	-7	109 ± 14.9	-3 ± 9.8	4
-30	122	4	102	-3	113	-4	117	9	114 ± 8.2	2 ± 6.4	4
-20	113	-4	94	-11	115	-3	116	8	109 ± 10.4	-2 ± 8.0	4
-10	111	-6	98	-7	102	-15	106	-1	105 ± 5.7	-7 ± 6.0	4
0	117	0	105	0	118	0	107	0	112 ± 6.6	0 ± 0.0	4
15	127	10	120	15	113	-5	102	-6	116 ± 10.9	4 ± 10.5	4
30	126	9	136	31	124	7	112	5	125 ± 10.0	13 ± 12.4	4
45	113	-4	141	36	129	12	113	6	124 ± 13.8	12 ± 17.3	4
60	111	-6	106	1	127	9	114	6	114 ± 8.9	2 ± 6.7	4
75	130	13	118	13	136	18	127	20	128 ± 7.3	16 ± 3.5	4
90	117	0	114	8	136	18	112	4	120 ± 11.0	8 ± 7.8	4
105	116	-1	107	2	131	13	116	8	117 ± 10.1	6 ± 6.6	4
120	109	-8	102	-4	138	20	102	-6	113 ± 17.0	1 ± 12.8	4
135	114	-3	102	-3	136	18	117	10	117 ± 14.2	5 ± 10.7	4
150	119	2	105	-1	131	13	123	16	119 ± 11.1	8 ± 8.1	4
165	132	15	93	-12	135	17	128	21	122 ± 19.4	10 ± 14.9	4
180	120	3	116	11	130	12	123	16	122 ± 5.9	10 ± 5.6	4
195	117	0	104	-2	148	30	122	14	122 ± 18.5	11 ± 14.7	4
210	117	0	115	10	144	26	115	8	123 ± 14.1	11 ± 10.9	4
225	113	-4	110	5	137	19	110	3	118 ± 12.8	6 ± 9.6	4
240	119	1	113	8	129	11	114	6	119 ± 7.3	7 ± 4.2	4
270	109	-8	108	3	134	17	116	8	117 ± 12.2	5 ± 10.2	4
300	113	-4	100	-5	127	9	123	16	116 ± 12.2	4 ± 10.4	4
330	116	-1	100	-5	135	18	126	19	120 ± 15.0	8 ± 12.4	4
360	101	-16	111	6	126	8	128	20	117 ± 12.8	5 ± 15.3	4
390	131	14	115	10	134	16	118	10	125 ± 9.6	13 ± 3.1	4
420	119	2	131	26	146	28	138	31	134 ± 11.2	22 ± 13.1	4

Appendix 1.4 – Group 4, Fexinidazole 1000 mg/kg PO

Heart rate (beats/min)

Time from	2482		2539		2552		2057		Absolute	Δ from 0	
dose (min)	Abs.	Δ from 0	Abs.	Δ from 0	Abs.	Δ from 0	Abs.	Δ from 0	Mean ± S.D.	Mean ± S.D.	N
-60	100	30	175	86	123	24	116	28	129 ± 32.5	42 ± 29.4	4
-50	54	-17	60	-30	100	1	62	-27	69 ± 21.0	-18 ± 13.7	4
-40	56	-15	78	-11	101	2	67	-21	76 ± 19.1	-11 ± 9.6	4
-30	79	8	64	-26	81	-18	90	2	78 ± 11.0	-9 ± 16.3	4
-20	63	-8	72	-18	99	0	97	9	83 ± 18.3	-4 ± 11.4	4
-10	73	2	89	-1	91	-9	98	9	87 ± 10.6	0 ± 7.4	4
0	71	0	90	0	99	0	88	0	87 ± 11.8	0 ± 0.0	4
15	120	50	148	59	123	24	100	12	123 ± 19.7	36 ± 21.8	4
30	120	49	172	82	164	64	105	17	140 ± 32.6	53 ± 27.8	4
45	93	22	199	109	176	77	94	6	141 ± 54.9	54 ± 48.0	4
60	80	9	107	17	129	30	116	28	108 ± 20.8	21 ± 9.7	4
75	132	62	121	31	118	19	126	37	124 ± 6.4	37 ± 18.1	4
90	84	13	99	9	141	42	94	5	104 ± 25.1	17 ± 16.5	4
105	85	14	99	9	113	14	92	3	97 ± 11.8	10 ± 5.1	4
120	78	7	77	-13	120	21	78	-10	88 ± 21.4	1 ± 16.0	4
135	82	12	90	0	118	19	126	37	104 ± 21.1	17 ± 15.7	4
150	91	20	94	5	146	46	115	26	111 ± 25.1	24 ± 17.2	4
165	119	48	95	5	114	15	110	21	109 ± 10.3	22 ± 18.2	4
180	87	16	105	16	132	33	101	13	107 ± 18.9	20 ± 9.2	4
195	103	33	118	28	138	39	119	30	119 ± 14.2	32 ± 4.6	4
210	103	32	101	12	150	51	103	15	114 ± 23.6	27 ± 18.0	4
225	123	52	113	23	119	20	97	9	113 ± 11.2	26 ± 18.3	4
240	104	33	124	34	122	23	140	52	123 ± 14.7	36 ± 11.9	4
270	101	30	134	45	144	45	154	65	133 ± 22.8	46 ± 14.3	4
300	79	8	83	-6	133	34	93	4	97 ± 24.7	10 ± 17.1	4
330	87	16	83	-7	143	44	116	27	107 ± 27.9	20 ± 21.2	4
360	77	7	70	-20	111	12	111	23	92 ± 21.8	5 ± 18.0	4
390	101	30	91	1	123	24	90	2	101 ± 15.2	14 ± 14.9	4
420	71	0	107	18	114	15	171	83	116 ± 41.4	29 ± 36.7	4

Appendix 1.4 – Group 4, Fexinidazole 1000 mg/kg PO

Uncorrected QT interval (msec)

Time from	2482		2539		2552		2057		Absolute	Δ from 0	
dose (min)	Abs.	Δ from 0	Abs.	Δ from 0	Abs.	Δ from 0	Abs.	Δ from 0	Mean ± S.D.	Mean ± S.D.	N
-60	187	-30	141	-54	179	-15	185	-14	173 ± 21.7	-28 ± 18.5	4
-50	231	14	212	17	198	3	207	9	212 ± 14.2	11 ± 6.2	4
-40	228	11	204	10	198	3	216	18	212 ± 13.6	10 ± 6.0	4
-30	221	3	213	18	193	-1	205	6	208 ± 11.7	7 ± 8.4	4
-20	222	5	201	7	198	4	192	-7	203 ± 13.1	2 ± 6.1	4
-10	214	-4	196	2	201	6	187	-11	200 ± 11.1	-2 ± 7.6	4
0	217	0	194	0	195	0	198	0	201 ± 10.9	0 ± 0.0	4
15	186	-32	165	-29	185	-10	198	0	184 ± 13.6	-18 ± 15.2	4
30	175	-42	148	-47	167	-28	189	-9	170 ± 17.2	-32 ± 16.8	4
45	202	-15	132	-62	176	-19	193	-5	176 ± 31.0	-25 ± 25.2	4
60	207	-10	175	-20	174	-20	196	-2	188 ± 16.4	-13 ± 8.6	4
75	183	-34	186	-8	176	-19	185	-13	183 ± 4.6	-19 ± 11.1	4
90	205	-13	213	19	173	-21	204	5	199 ± 17.5	-3 ± 17.9	4
105	194	-23	205	11	188	-7	191	-8	195 ± 7.7	-7 ± 13.9	4
120	219	1	209	15	192	-3	207	9	207 ± 11.1	6 ± 7.8	4
135	210	-7	209	14	193	-1	193	-5	201 ± 9.3	0 ± 9.6	4
150	205	-12	197	2	171	-23	191	-7	191 ± 14.3	-10 ± 10.6	4
165	178	-39	195	0	195	1	189	-9	189 ± 7.8	-12 ± 18.6	4
180	205	-12	198	3	189	-6	191	-7	196 ± 7.4	-5 ± 6.5	4
195	212	-5	205	11	168	-26	202	3	197 ± 19.5	-4 ± 16.1	4
210	209	-9	187	-8	177	-18	200	1	193 ± 14.1	-8 ± 7.9	4
225	206	-11	196	1	191	-4	202	4	199 ± 6.9	-2 ± 6.3	4
240	206	-11	190	-4	190	-5	182	-16	192 ± 10.3	-9 ± 5.8	4
270	208	-10	179	-16	175	-19	178	-20	185 ± 15.2	-16 ± 4.9	4
300	227	10	238	43	182	-12	210	12	214 ± 24.1	13 ± 22.8	4
330	225	8	223	29	183	-12	223	25	214 ± 20.6	12 ± 18.5	4
360	249	32	234	40	208	14	209	11	225 ± 20.0	24 ± 14.1	4
390	206	-11	224	30	195	0	228	30	213 ± 15.6	12 ± 20.6	4
420	241	24	209	15	203	9	184	-15	209 ± 23.7	8 ± 16.4	4

Appendix 1.4 – Group 4, Fexinidazole 1000 mg/kg PO

Corrected QT interval (covariate analysis, QTca) (msec)

Time from	2482		2539		2552		2057		Absolute	Δ from 0	
dose (min)	Abs.	Δ from 0	Abs.	Δ from 0	Abs.	Δ from 0	Abs.	Δ from 0	Mean ± S.D.	Mean ± S.D.	N
-60	186	-12	159	-24	186	-6	189	-2	180 ± 14.2	-11 ± 9.9	4
-50	200	2	187	4	195	2	186	-5	192 ± 6.6	1 ± 3.6	4
-40	199	1	189	5	196	3	198	8	196 ± 4.8	5 ± 2.9	4
-30	207	9	191	8	186	-6	197	7	195 ± 8.8	4 ± 7.0	4
-20	199	1	185	2	197	4	188	-2	192 ± 6.7	1 ± 2.7	4
-10	196	-2	189	6	195	3	184	-6	191 ± 5.6	0 ± 5.2	4
0	198	0	183	0	192	0	190	0	191 ± 6.0	0 ± 0.0	4
15	191	-7	179	-5	193	1	197	7	190 ± 7.9	-1 ± 6.2	4
30	181	-17	167	-16	185	-7	189	-1	181 ± 9.7	-10 ± 7.5	4
45	197	-1	158	-26	201	8	188	-3	186 ± 19.6	-5 ± 14.5	4
60	195	-3	176	-7	185	-8	202	11	189 ± 11.4	-2 ± 8.9	4
75	194	-4	193	10	183	-10	192	2	191 ± 5.4	-1 ± 8.6	4
90	196	-2	213	29	187	-6	195	4	198 ± 10.8	7 ± 15.7	4
105	187	-11	201	17	192	0	184	-6	191 ± 7.1	0 ± 12.3	4
120	205	7	194	10	199	6	195	5	198 ± 4.9	7 ± 2.2	4
135	200	2	200	17	199	7	199	9	200 ± 0.4	9 ± 6.2	4
150	200	2	191	8	187	-6	194	4	193 ± 5.5	2 ± 5.7	4
165	183	-15	191	7	199	7	190	-1	191 ± 6.5	0 ± 10.2	4
180	199	1	198	15	201	9	187	-3	196 ± 6.3	5 ± 8.2	4
195	213	15	213	29	180	-12	204	14	202 ± 15.4	11 ± 17.3	4
210	210	12	186	3	193	0	201	11	198 ± 10.1	7 ± 5.7	4
225	213	15	200	16	197	5	197	7	202 ± 7.6	11 ± 5.8	4
240	207	9	197	13	198	5	196	6	199 ± 5.1	8 ± 3.7	4
270	207	9	191	7	190	-2	196	6	196 ± 8.0	5 ± 5.0	4
300	213	15	226	43	193	1	205	15	209 ± 13.6	18 ± 17.5	4
330	215	17	211	28	198	5	229	39	213 ± 13.0	22 ± 14.4	4
360	234	36	213	30	212	20	209	19	217 ± 11.4	26 ± 8.3	4
390	206	8	219	35	203	10	219	28	211 ± 8.6	20 ± 13.7	4
420	222	24	212	28	209	17	208	17	213 ± 6.2	21 ± 5.5	4

Appendix 1.4 – Group 4, Fexinidazole 1000 mg/kg PO

PR interval (msec)

Time from	2482		2539		2552		2057		Absolute	Δ from 0	
dose (min)	Abs.	Δ from 0	Abs.	Δ from 0	Abs.	Δ from 0	Abs.	Δ from 0	Mean ± S.D.	Mean ± S.D.	N
-60	95	-13	81	-21	95	3	88	-2	90 ± 6.6	-8 ± 10.4	4
-50	108	1	110	8	92	-1	95	4	101 ± 9.2	3 ± 4.1	4
-40	111	3	110	8	94	1	90	0	101 ± 10.7	3 ± 3.8	4
-30	113	6	102	0	89	-4	89	-1	98 ± 11.6	0 ± 4.2	4
-20	108	1	104	2	96	4	90	0	100 ± 7.9	1 ± 1.7	4
-10	110	2	104	2	91	-2	91	1	99 ± 9.3	1 ± 1.9	4
0	107	0	102	0	93	0	91	0	98 ± 7.8	0 ± 0.0	4
15	97	-11	87	-15	89	-4	90	-1	91 ± 4.1	-8 ± 6.3	4
30	77	-31	86	-16	86	-7	89	-2	84 ± 5.3	-14 ± 12.6	4
45	96	-11	71	-31	87	-6	88	-3	85 ± 10.6	-13 ± 12.5	4
60	103	-4	102	0	92	-1	90	-1	97 ± 6.7	-2 ± 1.8	4
75	96	-11	99	-3	95	2	85	-6	94 ± 6.0	-5 ± 5.5	4
90	101	-6	99	-3	92	-1	91	0	96 ± 4.9	-2 ± 2.9	4
105	96	-11	101	0	86	-7	88	-3	93 ± 7.4	-6 ± 4.7	4
120	102	-6	107	5	91	-2	96	5	99 ± 7.2	1 ± 5.6	4
135	100	-7	103	1	90	-3	83	-8	94 ± 9.4	-4 ± 4.3	4
150	98	-10	101	-1	90	-2	86	-5	94 ± 6.7	-4 ± 3.7	4
165	86	-21	105	3	91	-2	89	-2	93 ± 8.2	-5 ± 10.7	4
180	101	-7	101	-1	93	0	82	-9	94 ± 8.8	-4 ± 4.4	4
195	96	-11	98	-4	101	8	87	-3	96 ± 5.9	-3 ± 8.0	4
210	95	-13	108	6	95	2	89	-2	97 ± 8.0	-2 ± 7.9	4
225	99	-8	96	-6	92	-1	88	-3	94 ± 5.1	-5 ± 3.0	4
240	100	-8	92	-10	96	3	85	-6	93 ± 6.1	-5 ± 5.5	4
270	99	-9	91	-11	93	0	76	-15	90 ± 9.7	-9 ± 6.0	4
300	109	1	109	7	94	1	84	-7	99 ± 12.1	1 ± 5.6	4
330	102	-5	110	8	92	0	90	-1	99 ± 9.4	0 ± 5.6	4
360	100	-7	106	4	92	-1	88	-3	97 ± 8.0	-2 ± 4.6	4
390	95	-13	98	-4	93	1	82	-9	92 ± 6.8	-6 ± 5.8	4
420	103	-4	96	-6	94	1	65	-25	90 ± 16.7	-9 ± 11.6	4

Appendix 1.4 – Group 4, Fexinidazole 1000 mg/kg PO

QRS interval (msec)

Time from	2482		2539		2552		2057		Absolute	Δ from 0	
dose (min)	Abs.	Δ from 0	Abs.	Δ from 0	Abs.	Δ from 0	Abs.	Δ from 0	Mean ± S.D.	Mean ± S.D.	N
-60	39	0	40	1	38	1	40	0	39 ± 1.1	0 ± 0.9	4
-50	39	0	36	-3	36	0	40	0	38 ± 1.9	-1 ± 1.5	4
-40	39	0	39	-1	37	0	40	0	39 ± 1.6	0 ± 0.5	4
-30	39	0	40	1	36	-1	41	1	39 ± 2.2	0 ± 0.7	4
-20	39	0	43	4	36	-1	40	0	40 ± 2.8	1 ± 1.9	4
-10	38	-1	38	-1	38	1	39	-1	38 ± 0.8	0 ± 1.0	4
0	39	0	39	0	37	0	40	0	39 ± 1.4	0 ± 0.0	4
15	37	-2	42	3	35	-2	40	0	38 ± 3.4	0 ± 2.4	4
30	37	-2	40	0	35	-1	41	0	38 ± 2.5	-1 ± 1.4	4
45	38	-1	40	0	35	-2	40	0	38 ± 2.5	-1 ± 1.1	4
60	39	0	38	-1	35	-2	40	0	38 ± 2.1	-1 ± 0.8	4
75	38	-1	42	2	36	-1	41	1	39 ± 2.6	0 ± 1.6	4
90	40	1	46	7	39	3	42	1	42 ± 3.2	3 ± 2.8	4
105	40	2	44	5	37	0	40	0	40 ± 3.1	2 ± 2.4	4
120	39	0	42	2	35	-1	41	1	39 ± 2.9	0 ± 1.6	4
135	40	1	44	5	37	0	39	-1	40 ± 3.2	1 ± 2.6	4
150	39	0	42	2	36	0	40	0	39 ± 2.4	1 ± 1.3	4
165	37	-2	43	3	37	0	40	0	39 ± 2.7	0 ± 2.2	4
180	39	0	40	1	36	-1	42	2	39 ± 2.7	0 ± 1.3	4
195	38	-1	40	1	37	0	41	1	39 ± 1.8	0 ± 0.7	4
210	38	-1	38	-1	42	6	42	2	40 ± 2.3	1 ± 3.1	4
225	39	0	42	3	38	1	44	3	41 ± 2.6	2 ± 1.5	4
240	39	0	42	3	37	0	42	2	40 ± 2.3	1 ± 1.3	4
270	40	1	41	1	37	0	40	0	39 ± 1.9	0 ± 0.8	4
300	43	4	39	0	37	0	45	4	41 ± 3.5	2 ± 2.4	4
330	48	9	41	2	37	1	43	3	43 ± 4.5	4 ± 3.8	4
360	62	23	41	2	38	1	45	5	46 ± 10.5	8 ± 10.2	4
390	43	4	42	2	38	1	43	3	41 ± 2.6	2 ± 1.2	4
420	46	7	42	3	38	1	43	3	42 ± 3.6	3 ± 2.7	4

Appendix 1.4 – Group 4, Fexinidazole 1000 mg/kg PO

RR interval (msec)

Time from	2482		2539		2552		2057		Absolute	Δ from 0	
dose (min)	Abs.	Δ from 0	Abs.	Δ from 0	Abs.	Δ from 0	Abs.	Δ from 0	Mean ± S.D.	Mean ± S.D.	N
-60	616	-294	355	-437	510	-120	543	-186	506 ± 110.1	-259 ± 138.2	4
-50	1134	224	1032	241	644	13	960	231	943 ± 211.3	177 ± 109.5	4
-40	1086	176	847	55	624	-7	871	142	857 ± 188.9	92 ± 83.0	4
-30	793	-117	953	161	701	70	708	-21	789 ± 117.3	23 ± 119.6	4
-20	959	48	857	66	621	-9	647	-82	771 ± 163.7	6 ± 66.7	4
-10	873	-37	705	-87	684	53	645	-84	727 ± 100.5	-39 ± 65.3	4
0	910	0	791	0	631	0	729	0	765 ± 117.0	0 ± 0.0	4
15	531	-379	425	-366	499	-132	612	-117	517 ± 77.3	-249 ± 143.4	4
30	513	-397	352	-440	383	-248	597	-132	461 ± 114.5	-304 ± 141.2	4
45	667	-243	280	-511	335	-296	681	-48	491 ± 213.0	-275 ± 190.7	4
60	776	-134	583	-209	468	-163	535	-194	590 ± 132.4	-175 ± 33.2	4
75	469	-441	507	-285	510	-121	507	-222	498 ± 19.5	-267 ± 134.2	4
90	721	-190	606	-186	433	-198	730	0	622 ± 138.4	-143 ± 96.0	4
105	704	-206	665	-127	546	-84	693	-36	652 ± 72.4	-113 ± 72.1	4
120	797	-113	838	47	512	-119	779	50	731 ± 148.6	-34 ± 95.1	4
135	748	-162	720	-72	525	-106	524	-205	629 ± 121.6	-136 ± 59.1	4
150	674	-236	680	-111	415	-216	561	-168	583 ± 124.4	-183 ± 55.9	4
165	532	-378	660	-131	548	-83	593	-136	583 ± 57.5	-182 ± 132.9	4
180	692	-218	593	-198	451	-179	656	-73	598 ± 106.0	-167 ± 64.6	4
195	591	-319	514	-278	444	-187	567	-162	529 ± 65.2	-237 ± 74.2	4
210	588	-323	605	-187	408	-223	579	-150	545 ± 91.8	-221 ± 74.2	4
225	523	-387	545	-246	521	-110	666	-63	564 ± 68.8	-202 ± 145.8	4
240	592	-319	520	-271	500	-130	431	-298	511 ± 65.9	-254 ± 84.9	4
270	604	-306	455	-336	418	-213	393	-336	468 ± 94.2	-298 ± 58.4	4
300	798	-112	747	-45	462	-168	671	-58	670 ± 147.6	-96 ± 56.4	4
330	731	-179	758	-34	423	-208	532	-197	611 ± 160.7	-154 ± 81.2	4
360	790	-120	903	111	554	-77	600	-129	712 ± 163.0	-54 ± 112.2	4
390	612	-298	666	-125	504	-126	718	-11	625 ± 91.5	-140 ± 118.6	4
420	863	-47	571	-221	532	-98	351	-378	579 ± 212.2	-186 ± 147.3	4

Appendix 1.4 – Group 4, Fexinidazole 1000 mg/kg PO

Body temperature (°C)

Time from	2482		2539		2552		2057		Absolute	Δ from 0	
dose (min)	Abs.	Δ from 0	Abs.	Δ from 0	Abs.	Δ from 0	Abs.	Δ from 0	Mean ± S.D.	Mean ± S.D.	N
-60	37.5	-0.2	37.5	0.0	38.4	-0.2	37.4	-0.2	37.7 ± 0.44	-0.1 ± 0.1	4
-50	37.7	-0.1	37.5	-0.1	38.4	-0.1	37.5	-0.1	37.8 ± 0.44	-0.1 ± 0.0	4
-40	37.7	0.0	37.2	-0.3	38.5	-0.1	37.7	0.0	37.8 ± 0.51	-0.1 ± 0.2	4
-30	37.8	0.0	37.5	0.0	38.6	0.0	37.8	0.2	37.9 ± 0.44	0.1 ± 0.1	4
-20	37.9	0.1	37.7	0.1	38.7	0.1	37.8	0.1	38.0 ± 0.45	0.1 ± 0.0	4
-10	37.9	0.2	37.8	0.3	38.7	0.2	37.5	-0.1	38.0 ± 0.49	0.1 ± 0.1	4
0	37.7	0.0	37.6	0.0	38.5	0.0	37.6	0.0	37.9 ± 0.45	0.0 ± 0.0	4
15	39.2	1.4	38.9	1.4	38.8	0.2	38.2	0.6	38.8 ± 0.40	0.9 ± 0.6	4
30	39.2	1.5	38.8	1.2	38.9	0.4	38.2	0.6	38.8 ± 0.41	0.9 ± 0.5	4
45	39.0	1.2	38.7	1.1	39.1	0.6	38.0	0.4	38.7 ± 0.49	0.8 ± 0.4	4
60	38.9	1.1	38.7	1.1	39.2	0.7	38.1	0.5	38.7 ± 0.48	0.9 ± 0.3	4
75	38.6	0.8	38.5	1.0	39.3	0.7	37.9	0.3	38.6 ± 0.56	0.7 ± 0.3	4
90	38.8	1.0	38.6	1.0	39.3	0.8	38.2	0.5	38.7 ± 0.49	0.8 ± 0.2	4
105	38.7	1.0	38.4	0.8	39.4	0.9	38.1	0.5	38.7 ± 0.56	0.8 ± 0.2	4
120	38.7	0.9	38.2	0.7	39.4	0.9	38.2	0.5	38.6 ± 0.59	0.8 ± 0.2	4
135	38.5	0.7	38.3	0.7	39.4	0.9	37.8	0.2	38.5 ± 0.67	0.6 ± 0.3	4
150	38.6	0.8	38.3	0.7	39.4	0.9	38.1	0.5	38.6 ± 0.58	0.7 ± 0.2	4
165	38.7	1.0	38.3	0.7	39.5	1.0	38.4	0.8	38.7 ± 0.56	0.9 ± 0.1	4
180	38.8	1.0	38.4	0.8	39.6	1.0	38.5	0.9	38.8 ± 0.52	1.0 ± 0.1	4
195	38.7	1.0	38.5	0.9	39.6	1.1	38.4	0.8	38.8 ± 0.56	0.9 ± 0.1	4
210	38.7	0.9	38.7	1.2	39.6	1.0	38.4	0.8	38.8 ± 0.51	1.0 ± 0.2	4
225	38.6	0.9	38.6	1.1	39.5	1.0	38.4	0.8	38.8 ± 0.50	0.9 ± 0.1	4
240	38.8	1.0	38.9	1.3	39.5	1.0	38.5	0.9	38.9 ± 0.41	1.0 ± 0.2	4
270	38.5	0.8	38.7	1.1	39.4	0.9	38.2	0.5	38.7 ± 0.53	0.8 ± 0.2	4
300	38.1	0.4	38.7	1.2	39.4	0.9	38.0	0.4	38.6 ± 0.65	0.7 ± 0.4	4
330	37.9	0.2	38.5	0.9	39.0	0.5	38.4	0.8	38.4 ± 0.44	0.6 ± 0.3	4
360	38.3	0.6	38.2	0.6	38.8	0.3	38.7	1.1	38.5 ± 0.29	0.6 ± 0.3	4
390	38.3	0.6	38.4	0.9	38.9	0.4	38.4	0.8	38.5 ± 0.24	0.7 ± 0.2	4
420	38.1	0.3	38.4	0.9	38.9	0.4	38.4	0.8	38.5 ± 0.35	0.6 ± 0.3	4

Appendix 2 Protocol and Amendments

Appendix 3 Pharmacy Certification

PHARMACY CERTIFICATION

Pharm. Cert. 0506-2007

TEST ARTICLE: **Fexinidazole**
STUDY NUMBER: **0506-2007**
NOTEBOOK NUMBERS: **F0094 and F0115**

DOCUMENTATION ENCLOSED IN THE NOTEBOOK:

A. Analysis certificate dated December 18, 2007 issued by Orgasynth Industries for Fexinidazole test item, raw material, Batch No. 3168-07-01/O
B. Material safety data sheet issued by Orgasynth Industries for Fexinidazole
C. Certificate of Analysis issued by Sigma-Aldrich for Methylcellulose 400 cP, raw material, Lot No. 125K0196
D. Certificate of Analysis issued by Sigma-Aldrich for Tween® 80, raw material, Lot No. 1324202
E. Label's photocopy of Acqua per preparazioni iniettabili (Bieffe Medital S.p.A.), raw material, Lot No. 07K1503
F. Copy of request cards of test item

ANALYTICAL DOCUMENTATION:

Requests and Analytical Results issued by Accelera/ADMET/Preclinical Formulation and Accelera/DMPK&ART/Bioanalysis and Analytical Control

MATERIALS USED FOR THE STUDY:

1. Fexinidazole test item, raw material, Batch No. 3168-07-01/O
2. Methylcellulose 400 cP, raw material, Lot No. 125K0196
3. Tween® 80, raw material, Lot No. 1324202
4. Acqua per preparazioni iniettabili, raw material, Lot No. 07K1503

PREPARATIONS:

Prepare suspension of Fexinidazole test item, raw material, Lot No. 3168-07-01/O in the vehicle (5% Tween® 80 in 0.5% Methylcellulose 400 cP solution) at the concentration of 10 mg/mL, 30 mg/mL and 100 mg/mL

CONCENTRATION CHECKS: (ACCURANCY LIMITS: ± 10% OF LABELED AMOUNT)

Fexinidazole suspensions	Preparation date	Request No.	% of L.A.
10 mg/mL - TOP	26th March 2008	200800092	101.33
10 mg/mL - MIDDLE	26th March 2008	200800093	102.67
10 mg/mL - BOTTOM	26th March 2008	200800094	100.16
30 mg/mL - TOP	28th March 2008	200800095	102.03
30 mg/mL - MIDDLE	28th March 2008	200800096	100.86
30 mg/mL - BOTTOM	28th March 2008	200800097	100.41
100 mg/mL - TOP	3rd April 2008	200800122	96.90
100 mg/mL - MIDDLE	3rd April 2008	200800123	101.06
100 mg/mL - BOTTOM	3rd April 2008	200800124	99.95

STABILITY:

Fexinidazole test article:
Expire date October 2008 for Fexinidazole, test item, raw material, Lot No. 3168-07-01/O if stored at room temperature protected from light.

Fexinidazole solutions:
Stability data indicate that Fexinidazole suspensions in the vehicle (5% Tween® 80 in 0.5% Methylcellulose 400 cP solution) in the range 0.5-100 mg/mL are stable up to 7 days at room temperature and 14 days at +4°C (Nerviano MS 0293-2007-R)

20th May 2008
