# Supplementary material for: Fexinidazole – A New Oral Nitroimidazole Drug Candidate Entering Clinical Development for the Treatment of Sleeping Sickness
Source: PLoS Negl Trop Dis. 2010 Dec 21;4(12):e923. doi: 10.1371/journal.pntd.0000923 (PMC3006138; doi:10.1371/journal.pntd.0000923)
Supplement: Dataset S14 — (0.41 MB PDF) [file pntd.0000923.s015.pdf]

Fexinidazole: Effects on General Behavior (Irwin's test) and Body Temperature in the Male Rat after Oral Administration

Product Name :	Fexinidazole
Study Number:	0508-2007
Study Director/Author:	
Sponsor Reference Study No.:	Not Applicable
Status:	Final

SUMMARY

The purpose of this study (0508-2007) was to investigate the potential effects of fexinidazole, an agent under investigation for the treatment of the Human African trypanosomiasis (HAT), on general behavior (Irwin's test) and on body temperature in conscious rats following a single oral administration.

Twenty-four male animals, divided into four experimental groups, were used in this study. Each group of animals was treated orally with the vehicle or the test item at doses of 100, 300 or 1000 mg/kg. General behavior was assessed before treatment and 2 and 24 hours after vehicle or test item administration.

No effects were observed on general behavior and body temperature at any of the doses tested. We can therefore conclude that the No-Observed-Effect-Level for fexinidazole on general behavior and body temperature in rats is ≥ 1000 mg/kg.

TABLE OF CONTENTS

1. INTRODUCTION AND OBJECTIVES 8
2. STUDY SPONSOR 8
3. TEST FACILITY 8
4. REGULATORY REQUIREMENTS 8
5. SCHEDULE 8
6. MATERIALS AND METHODS 9
6.1. Test and Control Items 9
6.1.1. Test Item 9
6.1.2. Vehicle/Control Item 9
6.1.3. Test Formulation 9
6.1.4. Test Formulation Analyses 9
6.2. Test System 10
6.2.1. Identification 10
6.3. Experimental Design 10
6.3.1. Experimental Groups 10
6.3.2. Allocation/Randomization 11
6.3.3. Dose Administration 11
6.3.4. Dose Justification 11
6.4. Experimental procedure 11
6.4.1. Behavioral observations 11
7. ARCHIVING 12
8. STUDY PERSONNEL 12
9. RESULTS AND DISCUSSION 13
10. CONCLUSIONS 13
11. TABLE 14

APPENDICES

Appendix 1. Study Data Listings

- Appendix 1.1. General behavior, Vehicle
- Appendix 1.2. General behavior, Fexinidazole, 100 mg/kg
- Appendix 1.3. General behavior, Fexinidazole, 300 mg/kg
- Appendix 1.4. General behavior, Fexinidazole, 1000 mg/kg
- Appendix 1.5. Body temperature

Appendix 2. Protocol

Appendix 3. Pharmacy Certification

1. INTRODUCTION AND OBJECTIVES

Fexinidazole is a 5-nitroimidazole derivative, biologically active against Trypanosoma parasites (T. b. rhodesiense and T. b. brucei), under investigation for the treatment of the Human African trypanosomiasis (HAT), known as sleeping sickness. The purpose of this study (0508-2007) was to determine the potential toxicity of the test article on general behavior (Irwin's test) and on body temperature in conscious rats following a single oral administration.

2. STUDY SPONSOR

Drugs for Neglected Diseases *initiative* (DND*i*)

1, Place St Gervais
CH-1201 Geneva
Switzerland

3. TEST FACILITY

Accelera

4. REGULATORY REQUIREMENTS

This study was conducted in compliance with:

- Decreto Legislativo 2 Marzo 2007, No. 50;
- Organisation for Economic Co-operation and Development (OECD) Principles of Good Laboratory Practice (GLP) (as revised in 1997).

The methods employed in this study were those described in the "Standard Operating Procedures" of the laboratories involved.

5. SCHEDULE

Experimental Start Date (randomization of animals)	4th Febraury 2008
Pre-treatment behavioral observation (all rats)	5th February 2008
First day of treatment (first 3 rats/group)	6th February 2008
Last day of treatment (last 3 rats/group)	7th February 2008
Experimental Completion Date (last behavioral observation)	8th February 2008

6. MATERIALS AND METHODS

6.1. Test and Control Items

6.1.1. Test Item

Identification	Fexinidazole
Lot/Batch Number	3168-07-01/O
Purity and Expiry	100.2%, October 2008
Storage Conditions	Room temperature, protected from light
Source and Manufacturer	Orgasynth Industries
Special Handling Precautions	Usual protection of all personnel conducting the study (mask, gloves and eyeglasses), according to MSDS (Material Safety Data Sheet)

6.1.1.1. Deviations from Protocol

In the protocol, the specification “protected from light” was inadvertently omitted. The test item has, however, been stored protected from light as requested.

6.1.2. Vehicle/Control Item

Identification	5% Tween 80 in 0.5% Methyl cellulose 400 cP (Methocel)	
Lot/Batch Number	Tween 80<br>Methyl cellulose 400 cP	1239316<br>105K0074
Expiry	Tween 80<br>Methyl cellulose 400 cP	November 2010<br>October 2008
Storage Conditions	Room temperature	
Source and Manufacturer	Tween 80<br>Methyl cellulose 400 cP	Sigma-Aldrich<br>Sigma-Aldrich
Method of Preparation	On file at Accelera/ADMET/Preclinical Formulation	

6.1.3. Test Formulation

Type of Formulation	Suspension in vehicle
Method of Preparation	On file at Accelera/ADMET/Preclinical formulation
Frequency of Preparation	Suspensions were prepared according to the stability data
Dose Concentrations	5, 15, 50 mg/mL
Storage Conditions	Room temperature in the dark
Source and Manufacturer	Accelera/ADMET/Preclinical Formulation

6.1.4. Test Formulation Analyses

6.1.4.1. Concentration and Homogeneity

Samples (top-middle-bottom, 5 mL each) of each dose suspension were collected under stirring for concentration and homogeneity check test of fexinidazole; 10 mL were taken also from the vehicle. After collection, samples were directly transferred at +4°C to Bioanalysis

& Analytical Control for analysis. The analyses were performed using a validated HPLC/UV method.

All values were found to be within acceptable limits.

6.1.4.2. Stability

Stability data indicate that fexinidazole suspensions in 5% Tween 80 in 0.5% methyl cellulose 400 cP (Methocel) in the range 0.5 - 100 mg/mL are stable up to 7 days at room temperature and 14 days at +4°C (NervianoMS 0293-2007-R).

6.2. Test System

Species/Strain and Source	Rat/Crl:CD(SD)BR, Charles River Labs Italia S.p.A. (Calco, Lecco)
Justification of Species and Sex	The rat has been used extensively in safety studies and a large amount of biological data is available. Only males were used since no significant gender-related differences in systemic exposure are expected.
Age	About 7 weeks on the day of dosing
Weight	217-262 g on the day of dosing
Acclimation	At least 6 days
Selection Criteria	Body weight and physical examination

6.2.1. Identification

Test Group	Color Code	Animal Identification No.
1	White	2876 - 2877 - 2878 - 2879 - 2880 - 2881
2	Yellow	2882 - 2883 - 2884 - 2885 - 2886 - 2887
3	Green	2888 - 2889 - 2890 - 2891 - 2892 - 2893
4	Red	2894 - 2895 - 2896 - 2897 - 2898 - 2899

Each animal was identified with a metal ear tag bearing a unique serial number. A color-coded cage card was affixed to each study animal’s cage, indicating the study number, animal number, and dose level.

6.3. Experimental Design

6.3.1. Experimental Groups

Exp. Session	Treatment[(a)]	Dose (mg/kg)	Number of Animals/Group (Males only)
1	Vehicle	0 (vehicle)	6
2	Fexinidazole	100	6
3	Fexinidazole	300	6

Exp. Session	Treatment[(a)]	Dose (mg/kg)	Number of Animals/Group (Males only)
4	Fexinidazole	1000	6
(a) Three animals/group were dosed each day of study.			

6.3.2. Allocation/Randomization

The animals were selected and distributed into experimental groups using random number tables (Fisher and Yates).

6.3.3. Dose Administration

Route and Method	Oral, gastric gavage
Duration	Single dose
Frequency	Once
Volume	20 mL/kg
Rate	Bolus

Dosage volumes were adjusted to the individual animal body weights recorded on the day of dosing.

6.3.4. Dose Justification

Doses of 100, 300 and 1000 mg/kg of fexinidazole were selected based on the results of a repeated (7-day) toxicity study in the same species (0339-2007). In this study, the top dose of 2000 mg/kg was well tolerated and did not cause any meaningful toxicological change. On the other hand, plasma levels determined as part of a 3-day toxicity study (study 0513-2007) showed that systemic exposure (both in terms of Cmax and AUC) did not increase when the dose went from 500 to 1000 mg/kg/day.

The oral route is that intended for clinical administration.

6.4. Experimental procedure

6.4.1. Behavioral observations

Behavior of the animals was assessed before treatment, 2 hours and 24 hours after treatment. After treatment, animals were put in their home cages. Observation was performed in 5 steps:

- Home cage observations: piloerection, bizarre behavior, exophthalmus, respiration, tremors, twitches, convulsions

- Open field observations: mobility, alertness, body posture, Straub tail, abnormal gait, palpebral closure, startle response, touch response

- Manipulation observations: positional passivity, visual placing, grip strength, body sag, pinna reflex, corneal reflex, flexor reflex

- Close observations: skin color, limb tone, abdomen tone, pupil size, lacrimation, salivation, provoked biting

- Final observations: pain response (tail pinch), righting reflex, body temperature, ease of handling, fear, aggressiveness, vocalization, stools, urines, other symptoms

The evaluation of the signs was made in individual animals according to an arbitrary scale from 0 to 8. Factors present in normal animals (e.g. alertness, mobility, etc.) were scored as 4; potentiation or depression of these factors was indicated as higher or lower integers, respectively. Factors absent in normal animals were scored from 0 (normal) to 8.

Body temperature (°C) was measured by means of a digital thermometer with a rectal probe.

Pre-treatment observations (including body temperature) were performed the day before treatment approximately at the same time foreseen for test article or vehicle administration.

7. ARCHIVING

The original protocol, all protocol amendments, all raw data, supporting documents, and specimens produced at the Test Facility, and the final report with original signatures were filed in the Archives of Accelera, Nerviano Medical Sciences S.r.l., Nerviano (Italy), where they will be kept for the period of time agreed with the Sponsor (at least 3 years) after which the Sponsor will be contacted for instructions regarding dispatch or disposal of the material.

A copy of the protocol, the report with original signatures, a reserve sample and all relevant original documentation of the test item were filed by the Sponsor.

8. STUDY PERSONNEL

o	

9. RESULTS AND DISCUSSION

Mean values (± S.D.) of body temperature is reported in Table 1. Individual data for behavioral observation and body temperature are reported in Appendix 1-4.

Following administration of fexinidazole at the dose of 100 and 300 mg/kg, no behavioral changes were observed. At 1000 mg/kg, two animals showed a minimal reduction in mobility and three animals presented with slight to moderate palpebral closure. At 24 hours after treatment, the same signs with the same grading were still present in 1 animal only. These minimal changes are not deemed to be drug-related. However, even in the unlikely case that they are treatment-related, due to the absence of changes in other behavioral signs, such as alertness, gait, passivity, reflexes, and to the minimal severity of the observations, they are of no toxicological or pharmacological significance.

10. CONCLUSIONS

Following oral administration of fexinidazole to male rats at doses of 100, 300 or 1000 mg/kg, no effects were observed on general behavior and body temperature at any of the doses tested. We can therefore conclude that the No-Observed-Effect-Level for fexinidazole on general behavior and body temperature in rats is ≥ 1000 mg/kg.

11. TABLE

Table 1. Body temperature, °C (mean ± S.D.)

Time from treatment	Treatment			
	Vehicle	Fexinidazole		
		100 mg/kg	300 mg/kg	1000 mg/kg
Basal	38.3 ± 0.2	38.1 ± 0.3	38.1 ± 0.4	37.7 ± 0.4
2 hours	37.9 ± 0.4	38.3 ± 0.3	38.1 ± 0.5	38.3 ± 0.3
24 hours	38.3 ± 0.3	38.2 ± 0.3	37.9 ± 0.6	38.1 ± 0.3

APPENDICES

Appendix 1 Study Data Listings

APPENDIX 1.1 - GENERAL BEHAVIOR, VEHICLE

Basal

Symptoms	Reference values	Animal No						Mean ± S.D.	
		2876	2877	2878	2879	2880	2881		
Piloerection	0	0	0	0	0	0	0	0.0	± 0.0
Eye prominence	0	0	0	0	0	0	0	0.0	± 0.0
Respiration	4	4	4	4	4	4	4	4.0	± 0.0
Tremors	0	0	0	0	0	0	0	0.0	± 0.0
Twitches	0	0	0	0	0	0	0	0.0	± 0.0
Convulsions	0	0	0	0	0	0	0	0.0	± 0.0
Bizarre behaviors	0	0	0	0	0	0	0	0.0	± 0.0
Mobility	4	4	4	4	4	4	4	4.0	± 0.0
Alertness	4	4	4	4	4	4	4	4.0	± 0.0
Body position	4	4	4	4	4	4	4	4.0	± 0.0
Straub tail	0	0	0	0	0	0	0	0.0	± 0.0
Abnormal gait	0	0	0	0	0	0	0	0.0	± 0.0
Palpebral closure	0	0	0	0	0	0	0	0.0	± 0.0
Startle response	4	4	4	4	4	4	4	4.0	± 0.0
Touch response	4	4	4	4	4	4	4	4.0	± 0.0
Positional passivity	4	4	4	4	4	2	4	3.7	± 0.8
Visual placing	0	0	0	0	0	0	0	0.0	± 0.0
Grip strength	4	4	4	4	4	4	4	4.0	± 0.0
Body sag	0	0	0	0	0	0	0	0.0	± 0.0
Pinna reflex	4	4	4	4	4	4	4	4.0	± 0.0
Corneal reflex	4	4	4	4	4	4	4	4.0	± 0.0
Flexor reflex	4	4	4	4	4	4	4	4.0	± 0.0
Skin colour	4	4	4	4	4	4	4	4.0	± 0.0
Limb tone	4	4	4	4	4	4	4	4.0	± 0.0
Abdomen tone	4	4	4	4	4	4	4	4.0	± 0.0
Pupil response	0	0	0	0	0	0	0	0.0	± 0.0
Lacrimation	0	0	0	0	0	0	0	0.0	± 0.0
Salivation	0	0	0	0	0	0	0	0.0	± 0.0
Biting	0	0	0	0	0	0	0	0.0	± 0.0
Tail pinch response	4	4	4	4	4	4	4	4.0	± 0.0
Righting reflex	0	0	0	0	0	0	0	0.0	± 0.0
Ease of handling	4	4	4	4	4	4	4	4.0	± 0.0
Fear	0	0	0	0	0	0	0	0.0	± 0.0
Aggressivity	0	0	0	0	0	0	0	0.0	± 0.0
Vocalization	0	0	0	0	0	0	0	0.0	± 0.0
Stools	0	0	0	0	0	0	0	0.0	± 0.0
Urines	0	0	0	0	0	0	0	0.0	± 0.0
Other symptoms	0	0	0	0	0	0	0	0.0	± 0.0

APPENDIX 1.1 - GENERAL BEHAVIOR, VEHICLE

Vehicle, 2 hours

Symptoms	Reference values	Animal No						Mean ± S.D.	
		2876	2877	2878	2879	2880	2881		
Piloerection	0	0	0	0	0	0	0	0.0	± 0.0
Eye prominence	0	0	0	0	0	0	0	0.0	± 0.0
Respiration	4	4	4	4	4	4	4	4.0	± 0.0
Tremors	0	0	0	0	0	0	0	0.0	± 0.0
Twitches	0	0	0	0	0	0	0	0.0	± 0.0
Convulsions	0	0	0	0	0	0	0	0.0	± 0.0
Bizarre behaviors	0	0	0	0	0	0	0	0.0	± 0.0
Mobility	4	4	4	4	4	4	4	4.0	± 0.0
Alertness	4	4	4	4	4	4	4	4.0	± 0.0
Body position	4	4	4	4	4	4	4	4.0	± 0.0
Straub tail	0	0	0	0	0	0	0	0.0	± 0.0
Abnormal gait	0	0	0	0	0	0	0	0.0	± 0.0
Palpebral closure	0	0	0	0	0	0	0	0.0	± 0.0
Startle response	4	4	4	4	4	4	4	4.0	± 0.0
Touch response	4	4	4	4	4	4	4	4.0	± 0.0
Positional passivity	4	4	4	4	4	4	4	4.0	± 0.0
Visual placing	0	0	0	0	0	0	0	0.0	± 0.0
Grip strength	4	4	4	4	4	4	4	4.0	± 0.0
Body sag	0	0	0	0	0	0	0	0.0	± 0.0
Pinna reflex	4	4	4	4	4	4	4	4.0	± 0.0
Corneal reflex	4	4	4	4	4	4	4	4.0	± 0.0
Flexor reflex	4	4	4	4	4	4	4	4.0	± 0.0
Skin colour	4	4	4	4	4	4	4	4.0	± 0.0
Limb tone	4	4	4	4	4	4	4	4.0	± 0.0
Abdomen tone	4	4	4	4	4	4	4	4.0	± 0.0
Pupil response	0	0	0	0	0	0	0	0.0	± 0.0
Lacrimation	0	0	0	0	0	0	0	0.0	± 0.0
Salivation	0	0	0	0	0	0	0	0.0	± 0.0
Biting	0	0	0	0	0	0	0	0.0	± 0.0
Tail pinch response	4	4	4	4	4	4	4	4.0	± 0.0
Righting reflex	0	0	0	0	0	0	0	0.0	± 0.0
Ease of handling	4	4	4	4	4	4	4	4.0	± 0.0
Fear	0	0	0	0	0	0	0	0.0	± 0.0
Aggressivity	0	0	0	0	0	0	0	0.0	± 0.0
Vocalization	0	0	0	0	0	0	0	0.0	± 0.0
Stools	0	0	0	0	0	0	0	0.0	± 0.0
Urines	0	0	0	0	0	0	0	0.0	± 0.0
Other symptoms	0	0	0	0	0	0	0	0.0	± 0.0

APPENDIX 1.1 - GENERAL BEHAVIOR, VEHICLE

24 hours

Symptoms	Reference values	Animal No						Mean ± S.D.	
		2876	2877	2878	2879	2880	2881		
Piloerection	0	0	0	0	0	0	0	0.0	± 0.0
Eye prominence	0	0	0	0	0	0	0	0.0	± 0.0
Respiration	4	4	4	4	4	4	4	4.0	± 0.0
Tremors	0	0	0	0	0	0	0	0.0	± 0.0
Twitches	0	0	0	0	0	0	0	0.0	± 0.0
Convulsions	0	0	0	0	0	0	0	0.0	± 0.0
Bizarre behaviors	0	0	0	0	0	0	0	0.0	± 0.0
Mobility	4	4	4	4	4	4	4	4.0	± 0.0
Alertness	4	4	4	4	4	4	4	4.0	± 0.0
Body position	4	4	4	4	4	4	4	4.0	± 0.0
Straub tail	0	0	0	0	0	0	0	0.0	± 0.0
Abnormal gait	0	0	0	0	0	0	0	0.0	± 0.0
Palpebral closure	0	0	0	0	0	0	0	0.0	± 0.0
Startle response	4	4	4	4	4	4	4	4.0	± 0.0
Touch response	4	4	4	4	4	4	4	4.0	± 0.0
Positional passivity	4	4	4	4	4	4	4	4.0	± 0.0
Visual placing	0	0	0	0	0	0	2	0.3	± 0.8
Grip strength	4	4	4	4	4	4	4	4.0	± 0.0
Body sag	0	0	0	0	0	0	0	0.0	± 0.0
Pinna reflex	4	4	4	4	4	4	4	4.0	± 0.0
Corneal reflex	4	4	4	4	4	4	4	4.0	± 0.0
Flexor reflex	4	4	4	4	4	4	4	4.0	± 0.0
Skin colour	4	4	4	4	4	4	4	4.0	± 0.0
Limb tone	4	4	4	4	4	4	4	4.0	± 0.0
Abdomen tone	4	4	4	4	4	4	4	4.0	± 0.0
Pupil response	0	0	0	0	0	0	0	0.0	± 0.0
Lacrimation	0	0	0	0	0	0	0	0.0	± 0.0
Salivation	0	0	0	0	0	0	0	0.0	± 0.0
Biting	0	0	0	0	0	0	0	0.0	± 0.0
Tail pinch response	4	4	4	4	4	4	4	4.0	± 0.0
Righting reflex	0	0	0	0	0	0	0	0.0	± 0.0
Ease of handling	4	4	4	4	4	4	4	4.0	± 0.0
Fear	0	0	0	0	0	0	0	0.0	± 0.0
Aggressivity	0	0	0	0	0	0	0	0.0	± 0.0
Vocalization	0	0	0	0	0	0	0	0.0	± 0.0
Stools	0	0	0	0	0	0	0	0.0	± 0.0
Urines	0	0	0	0	0	0	0	0.0	± 0.0
Other symptoms	0	0	0	0	0	0	0	0.0	± 0.0

APPENDIX 1.2 - GENERAL BEHAVIOR, FEXINIDAZOLE 100 mg/kg

Basal

Symptoms	Reference values	Animal No						Mean ± S.D.
		2882	2883	2884	2885	2886	2887	
Piloerection	0	0	0	0	0	0	0	0.0 ± 0.0
Eye prominence	0	0	0	0	0	0	0	0.0 ± 0.0
Respiration	4	4	4	4	4	4	4	4.0 ± 0.0
Tremors	0	0	0	0	0	0	0	0.0 ± 0.0
Twitches	0	0	0	0	0	0	0	0.0 ± 0.0
Convulsions	0	0	0	0	0	0	0	0.0 ± 0.0
Bizarre behaviors	0	0	0	0	0	0	0	0.0 ± 0.0
Mobility	4	4	4	4	4	4	4	4.0 ± 0.0
Alertness	4	4	4	4	4	4	4	4.0 ± 0.0
Body position	4	4	4	4	4	4	4	4.0 ± 0.0
Straub tail	0	0	0	0	0	0	0	0.0 ± 0.0
Abnormal gait	0	0	0	0	0	0	0	0.0 ± 0.0
Palpebral closure	0	0	0	0	0	0	0	0.0 ± 0.0
Startle response	4	4	4	4	4	4	4	4.0 ± 0.0
Touch response	4	4	4	4	4	4	4	4.0 ± 0.0
Positional passivity	4	4	4	4	4	4	4	4.0 ± 0.0
Visual placing	0	0	0	0	0	0	0	0.0 ± 0.0
Grip strength	4	4	4	4	4	4	4	4.0 ± 0.0
Body sag	0	0	0	0	0	0	0	0.0 ± 0.0
Pinna reflex	4	4	4	4	4	4	4	4.0 ± 0.0
Corneal reflex	4	4	4	4	4	4	4	4.0 ± 0.0
Flexor reflex	4	4	4	4	4	4	4	4.0 ± 0.0
Skin colour	4	4	4	4	4	4	4	4.0 ± 0.0
Limb tone	4	4	4	4	4	4	4	4.0 ± 0.0
Abdomen tone	4	4	4	4	4	4	4	4.0 ± 0.0
Pupil response	0	0	0	0	0	0	0	0.0 ± 0.0
Lacrimation	0	0	0	0	0	0	0	0.0 ± 0.0
Salivation	0	0	0	0	0	0	0	0.0 ± 0.0
Biting	0	0	0	0	0	0	0	0.0 ± 0.0
Tail pinch response	4	4	4	4	4	4	4	4.0 ± 0.0
Righting reflex	0	0	0	0	0	0	0	0.0 ± 0.0
Ease of handling	4	4	4	4	4	4	4	4.0 ± 0.0
Fear	0	0	0	0	0	0	0	0.0 ± 0.0
Aggressivity	0	0	0	0	0	0	0	0.0 ± 0.0
Vocalization	0	0	0	0	0	0	0	0.0 ± 0.0
Stools	0	0	0	0	0	0	0	0.0 ± 0.0
Urines	0	0	0	0	0	0	0	0.0 ± 0.0
Other symptoms	0	0	0	0	0	0	0	0.0 ± 0.0

APPENDIX 1.2 - GENERAL BEHAVIOR, FEXINIDAZOLE 100 mg/kg

2 hours

Symptoms	Reference values	Animal No						Mean ± S.D.	
		2882	2883	2884	2885	2886	2887		
Piloerection	0	0	0	0	0	0	0	0.0	± 0.0
Eye prominence	0	0	0	0	0	0	0	0.0	± 0.0
Respiration	4	4	4	4	4	4	4	4.0	± 0.0
Tremors	0	0	0	0	0	0	0	0.0	± 0.0
Twitches	0	0	0	0	0	0	0	0.0	± 0.0
Convulsions	0	0	0	0	0	0	0	0.0	± 0.0
Bizarre behaviors	0	0	0	0	0	0	0	0.0	± 0.0
Mobility	4	4	4	4	4	4	4	4.0	± 0.0
Alertness	4	4	4	4	4	4	4	4.0	± 0.0
Body position	4	4	4	4	4	4	4	4.0	± 0.0
Straub tail	0	0	0	0	0	0	0	0.0	± 0.0
Abnormal gait	0	0	0	0	0	0	0	0.0	± 0.0
Palpebral closure	0	0	0	0	0	0	0	0.0	± 0.0
Startle response	4	4	4	4	4	4	4	4.0	± 0.0
Touch response	4	4	4	4	4	4	4	4.0	± 0.0
Positional passivity	4	4	4	4	4	4	4	4.0	± 0.0
Visual placing	0	0	0	0	0	0	0	0.0	± 0.0
Grip strength	4	4	4	4	4	4	4	4.0	± 0.0
Body sag	0	0	0	0	0	0	0	0.0	± 0.0
Pinna reflex	4	4	4	4	4	4	4	4.0	± 0.0
Corneal reflex	4	4	4	4	4	4	4	4.0	± 0.0
Flexor reflex	4	4	4	4	4	4	4	4.0	± 0.0
Skin colour	4	4	4	4	4	4	4	4.0	± 0.0
Limb tone	4	4	4	4	4	4	4	4.0	± 0.0
Abdomen tone	4	4	4	4	4	4	4	4.0	± 0.0
Pupil response	0	0	0	0	0	0	0	0.0	± 0.0
Lacrimation	0	0	0	0	0	0	0	0.0	± 0.0
Salivation	0	0	0	0	0	0	0	0.0	± 0.0
Biting	0	0	0	0	0	0	0	0.0	± 0.0
Tail pinch response	4	4	4	4	4	4	4	4.0	± 0.0
Righting reflex	0	0	0	0	0	0	0	0.0	± 0.0
Ease of handling	4	4	4	4	4	4	4	4.0	± 0.0
Fear	0	0	0	0	0	0	0	0.0	± 0.0
Aggressivity	0	0	0	0	0	0	0	0.0	± 0.0
Vocalization	0	0	0	0	0	0	0	0.0	± 0.0
Stools	0	0	0	0	0	0	0	0.0	± 0.0
Urines	0	0	0	0	0	0	0	0.0	± 0.0
Other symptoms	0	0	0	0	0	0	0	0.0	± 0.0

APPENDIX 1.2 - GENERAL BEHAVIOR, FEXINIDAZOLE 100 mg/kg

24 hours

Symptoms	Reference values	Animal No						Mean ± S.D.	
		2882	2883	2884	2885	2886	2887		
Piloerection	0	0	0	0	0	0	0	0.0	± 0.0
Eye prominence	0	0	0	0	0	0	0	0.0	± 0.0
Respiration	4	4	4	4	4	4	4	4.0	± 0.0
Tremors	0	0	0	0	0	0	0	0.0	± 0.0
Twitches	0	0	0	0	0	0	0	0.0	± 0.0
Convulsions	0	0	0	0	0	0	0	0.0	± 0.0
Bizarre behaviors	0	0	0	0	0	0	0	0.0	± 0.0
Mobility	4	4	4	4	4	4	4	4.0	± 0.0
Alertness	4	4	4	4	4	4	4	4.0	± 0.0
Body position	4	4	4	4	4	4	4	4.0	± 0.0
Straub tail	0	0	0	0	0	0	0	0.0	± 0.0
Abnormal gait	0	0	0	0	0	0	0	0.0	± 0.0
Palpebral closure	0	0	0	0	0	0	0	0.0	± 0.0
Startle response	4	4	4	4	4	4	4	4.0	± 0.0
Touch response	4	4	4	4	4	4	4	4.0	± 0.0
Positional passivity	4	4	4	4	4	4	4	4.0	± 0.0
Visual placing	0	0	0	0	0	0	0	0.0	± 0.0
Grip strength	4	4	4	4	4	4	4	4.0	± 0.0
Body sag	0	0	0	0	0	0	0	0.0	± 0.0
Pinna reflex	4	4	4	4	4	4	4	4.0	± 0.0
Corneal reflex	4	4	4	4	4	4	4	4.0	± 0.0
Flexor reflex	4	4	4	4	4	4	4	4.0	± 0.0
Skin colour	4	4	4	4	4	4	4	4.0	± 0.0
Limb tone	4	4	4	4	4	4	4	4.0	± 0.0
Abdomen tone	4	4	4	4	4	4	4	4.0	± 0.0
Pupil response	0	0	0	0	0	0	0	0.0	± 0.0
Lacrimation	0	0	0	0	0	0	0	0.0	± 0.0
Salivation	0	0	0	0	0	0	0	0.0	± 0.0
Biting	0	0	0	0	0	0	0	0.0	± 0.0
Tail pinch response	4	4	4	4	4	4	4	4.0	± 0.0
Righting reflex	0	0	0	0	0	0	0	0.0	± 0.0
Ease of handling	4	4	4	4	4	4	4	4.0	± 0.0
Fear	0	0	0	0	0	0	0	0.0	± 0.0
Aggressivity	0	0	0	0	0	0	0	0.0	± 0.0
Vocalization	0	0	0	0	0	0	0	0.0	± 0.0
Stools	0	0	0	0	0	0	0	0.0	± 0.0
Urines	0	0	0	0	0	0	0	0.0	± 0.0
Other symptoms	0	0	0	0	0	0	0	0.0	± 0.0

APPENDIX 1.3 - GENERAL BEHAVIOR, FEXINIDAZOLE 300 mg/kg

Basal

Symptoms	Reference values	Animal No						Mean ± S.D.	
		2888	2889	2890	2891	2892	2893		
Piloerection	0	0	0	0	0	0	0	0.0	± 0.0
Eye prominence	0	0	0	0	0	0	0	0.0	± 0.0
Respiration	4	4	4	4	4	4	4	4.0	± 0.0
Tremors	0	0	0	0	0	0	0	0.0	± 0.0
Twitches	0	0	0	0	0	0	0	0.0	± 0.0
Convulsions	0	0	0	0	0	0	0	0.0	± 0.0
Bizarre behaviors	0	0	0	0	0	0	0	0.0	± 0.0
Mobility	4	4	4	4	4	4	4	4.0	± 0.0
Alertness	4	4	4	4	4	4	4	4.0	± 0.0
Body position	4	4	4	4	4	4	4	4.0	± 0.0
Straub tail	0	0	0	0	0	0	0	0.0	± 0.0
Abnormal gait	0	0	0	0	0	0	0	0.0	± 0.0
Palpebral closure	0	0	0	0	0	0	0	0.0	± 0.0
Startle response	4	4	4	4	4	4	4	4.0	± 0.0
Touch response	4	4	4	4	4	4	4	4.0	± 0.0
Positional passivity	4	4	4	4	4	4	4	4.0	± 0.0
Visual placing	0	0	0	0	0	0	0	0.0	± 0.0
Grip strength	4	4	4	4	4	4	4	4.0	± 0.0
Body sag	0	0	0	0	0	0	0	0.0	± 0.0
Pinna reflex	4	4	4	4	4	4	4	4.0	± 0.0
Corneal reflex	4	4	4	4	4	4	4	4.0	± 0.0
Flexor reflex	4	4	4	4	4	4	4	4.0	± 0.0
Skin colour	4	4	4	4	4	4	4	4.0	± 0.0
Limb tone	4	4	4	4	4	4	4	4.0	± 0.0
Abdomen tone	4	4	4	4	4	4	4	4.0	± 0.0
Pupil response	0	0	0	0	0	0	0	0.0	± 0.0
Lacrimation	0	0	0	0	0	0	0	0.0	± 0.0
Salivation	0	0	0	0	0	0	0	0.0	± 0.0
Biting	0	0	0	0	0	0	0	0.0	± 0.0
Tail pinch response	4	4	4	4	4	4	4	4.0	± 0.0
Righting reflex	0	0	0	0	0	0	0	0.0	± 0.0
Ease of handling	4	4	4	4	4	4	4	4.0	± 0.0
Fear	0	0	0	0	0	0	0	0.0	± 0.0
Aggressivity	0	0	0	0	0	0	0	0.0	± 0.0
Vocalization	0	0	0	0	0	0	0	0.0	± 0.0
Stools	0	0	0	0	0	0	0	0.0	± 0.0
Urines	0	0	0	0	0	0	0	0.0	± 0.0
Other symptoms	0	0	0	0	0	0	0	0.0	± 0.0

APPENDIX 1.3 - GENERAL BEHAVIOR, FEXINIDAZOLE 300 mg/kg

2 hours

Symptoms	Reference values	Animal No						Mean ± S.D.	
		2888	2889	2890	2891	2892	2893		
Piloerection	0	0	0	0	0	0	0	0.0	± 0.0
Eye prominence	0	0	0	0	0	0	0	0.0	± 0.0
Respiration	4	4	4	4	4	4	4	4.0	± 0.0
Tremors	0	0	0	0	0	0	0	0.0	± 0.0
Twitches	0	0	0	0	0	0	0	0.0	± 0.0
Convulsions	0	0	0	0	0	0	0	0.0	± 0.0
Bizarre behaviors	0	0	0	0	0	0	0	0.0	± 0.0
Mobility	4	4	4	4	4	4	4	4.0	± 0.0
Alertness	4	4	4	4	4	4	4	4.0	± 0.0
Body position	4	4	4	4	4	4	4	4.0	± 0.0
Straub tail	0	0	0	0	0	0	0	0.0	± 0.0
Abnormal gait	0	0	0	0	0	0	0	0.0	± 0.0
Palpebral closure	0	0	0	0	0	2	0	0.3	± 0.8
Startle response	4	4	4	4	4	4	4	4.0	± 0.0
Touch response	4	4	4	4	4	4	4	4.0	± 0.0
Positional passivity	4	4	4	4	4	4	4	4.0	± 0.0
Visual placing	0	0	0	0	0	0	0	0.0	± 0.0
Grip strength	4	4	4	4	4	4	4	4.0	± 0.0
Body sag	0	0	0	0	0	0	0	0.0	± 0.0
Pinna reflex	4	4	4	4	4	4	4	4.0	± 0.0
Corneal reflex	4	4	4	4	4	4	4	4.0	± 0.0
Flexor reflex	4	4	4	4	4	4	4	4.0	± 0.0
Skin colour	4	4	4	4	4	4	4	4.0	± 0.0
Limb tone	4	4	4	4	4	4	4	4.0	± 0.0
Abdomen tone	4	4	4	4	4	4	4	4.0	± 0.0
Pupil response	0	0	0	0	0	0	0	0.0	± 0.0
Lacrimation	0	0	0	0	0	0	0	0.0	± 0.0
Salivation	0	0	0	0	0	0	0	0.0	± 0.0
Biting	0	0	0	0	0	0	0	0.0	± 0.0
Tail pinch response	4	4	4	4	4	4	4	4.0	± 0.0
Righting reflex	0	0	0	0	0	0	0	0.0	± 0.0
Ease of handling	4	4	4	4	4	4	4	4.0	± 0.0
Fear	0	0	0	0	0	0	0	0.0	± 0.0
Aggressivity	0	0	0	0	0	0	0	0.0	± 0.0
Vocalization	0	0	0	0	0	0	0	0.0	± 0.0
Stools	0	0	0	0	0	0	0	0.0	± 0.0
Urines	0	0	0	0	0	0	0	0.0	± 0.0
Other symptoms	0	0	0	0	0	0	0	0.0	± 0.0

APPENDIX 1.3 - GENERAL BEHAVIOR, FEXINIDAZOLE 300 mg/kg

24 hours

Symptoms	Reference values	Animal No						Mean ± S.D.	
		2888	2889	2890	2891	2892	2893		
Piloerection	0	0	0	0	0	0	0	0.0	± 0.0
Eye prominence	0	0	0	0	0	0	0	0.0	± 0.0
Respiration	4	4	4	4	4	4	4	4.0	± 0.0
Tremors	0	0	0	0	0	0	0	0.0	± 0.0
Twitches	0	0	0	0	0	0	0	0.0	± 0.0
Convulsions	0	0	0	0	0	0	0	0.0	± 0.0
Bizarre behaviors	0	0	0	0	0	0	0	0.0	± 0.0
Mobility	4	4	4	4	4	4	4	4.0	± 0.0
Alertness	4	4	4	4	4	4	4	4.0	± 0.0
Body position	4	4	4	4	4	4	4	4.0	± 0.0
Straub tail	0	0	0	0	0	0	0	0.0	± 0.0
Abnormal gait	0	0	0	0	0	0	0	0.0	± 0.0
Palpebral closure	0	0	0	0	0	0	0	0.0	± 0.0
Startle response	4	4	4	4	4	4	4	4.0	± 0.0
Touch response	4	4	4	4	4	4	4	4.0	± 0.0
Positional passivity	4	4	4	4	4	4	4	4.0	± 0.0
Visual placing	0	0	0	0	0	0	0	0.0	± 0.0
Grip strength	4	4	4	4	4	4	4	4.0	± 0.0
Body sag	0	0	0	0	0	0	0	0.0	± 0.0
Pinna reflex	4	4	4	4	4	4	4	4.0	± 0.0
Corneal reflex	4	4	4	4	4	4	4	4.0	± 0.0
Flexor reflex	4	4	4	4	4	4	4	4.0	± 0.0
Skin colour	4	4	4	4	4	4	4	4.0	± 0.0
Limb tone	4	4	4	4	4	4	4	4.0	± 0.0
Abdomen tone	4	4	4	4	4	4	4	4.0	± 0.0
Pupil response	0	0	0	0	0	0	0	0.0	± 0.0
Lacrimation	0	0	0	0	0	0	0	0.0	± 0.0
Salivation	0	0	0	0	0	0	0	0.0	± 0.0
Biting	0	0	0	0	0	0	0	0.0	± 0.0
Tail pinch response	4	4	4	4	4	4	4	4.0	± 0.0
Righting reflex	0	0	0	0	0	0	0	0.0	± 0.0
Ease of handling	4	4	4	4	4	4	4	4.0	± 0.0
Fear	0	0	0	0	0	0	0	0.0	± 0.0
Aggressivity	0	0	0	0	0	0	0	0.0	± 0.0
Vocalization	0	0	0	0	0	0	0	0.0	± 0.0
Stools	0	0	0	0	0	0	0	0.0	± 0.0
Urines	0	0	0	0	0	0	0	0.0	± 0.0
Other symptoms	0	0	0	0	0	0	0	0.0	± 0.0

APPENDIX 1.4 - GENERAL BEHAVIOR, FEXINIDAZOLE 1000 mg/kg

Basal

Symptoms	Reference values	Animal No						Mean ± S.D.	
		2894	2895	2896	2897	2898	2899		
Piloerection	0	0	0	0	0	0	0	0.0	± 0.0
Eye prominence	0	0	0	0	0	0	0	0.0	± 0.0
Respiration	4	4	4	4	4	4	4	4.0	± 0.0
Tremors	0	0	0	0	0	0	0	0.0	± 0.0
Twitches	0	0	0	0	0	0	0	0.0	± 0.0
Convulsions	0	0	0	0	0	0	0	0.0	± 0.0
Bizarre behaviors	0	0	0	0	0	0	0	0.0	± 0.0
Mobility	4	4	4	4	4	4	4	4.0	± 0.0
Alertness	4	4	4	4	4	4	4	4.0	± 0.0
Body position	4	4	4	4	4	4	4	4.0	± 0.0
Straub tail	0	0	0	0	0	0	0	0.0	± 0.0
Abnormal gait	0	0	0	0	0	0	0	0.0	± 0.0
Palpebral closure	0	0	0	0	0	0	0	0.0	± 0.0
Startle response	4	4	4	4	4	4	4	4.0	± 0.0
Touch response	4	4	4	4	4	4	4	4.0	± 0.0
Positional passivity	4	4	4	4	4	2	4	3.7	± 0.8
Visual placing	0	0	0	0	0	0	0	0.0	± 0.0
Grip strength	4	4	4	4	4	4	4	4.0	± 0.0
Body sag	0	0	0	0	0	0	0	0.0	± 0.0
Pinna reflex	4	4	4	4	4	4	4	4.0	± 0.0
Corneal reflex	4	4	4	4	4	4	4	4.0	± 0.0
Flexor reflex	4	4	4	4	4	4	4	4.0	± 0.0
Skin colour	4	4	4	4	4	4	4	4.0	± 0.0
Limb tone	4	4	4	4	4	4	4	4.0	± 0.0
Abdomen tone	4	4	4	4	4	4	4	4.0	± 0.0
Pupil response	0	0	0	0	0	0	0	0.0	± 0.0
Lacrimation	0	0	0	0	0	0	0	0.0	± 0.0
Salivation	0	0	0	0	0	0	0	0.0	± 0.0
Biting	0	0	0	0	0	0	0	0.0	± 0.0
Tail pinch response	4	4	4	4	4	4	4	4.0	± 0.0
Righting reflex	0	0	0	0	0	0	0	0.0	± 0.0
Ease of handling	4	4	4	4	4	4	4	4.0	± 0.0
Fear	0	0	0	0	0	0	0	0.0	± 0.0
Aggressivity	0	0	0	0	0	0	0	0.0	± 0.0
Vocalization	0	0	0	0	0	0	0	0.0	± 0.0
Stools	0	0	0	0	0	0	0	0.0	± 0.0
Urines	0	0	0	0	0	0	0	0.0	± 0.0
Other symptoms	0	0	0	0	0	0	0	0.0	± 0.0

APPENDIX 1.4 - GENERAL BEHAVIOR, FEXINIDAZOLE 1000 mg/kg

2 hours

Symptoms	Reference values	Animal No						Mean ± S.D.	
		2894	2895	2896	2897	2898	2899		
Piloerection	0	0	0	0	0	0	0	0.0	± 0.0
Eye prominence	0	0	0	0	0	0	0	0.0	± 0.0
Respiration	4	4	4	4	4	4	4	4.0	± 0.0
Tremors	0	0	0	0	0	0	0	0.0	± 0.0
Twitches	0	0	0	0	0	0	0	0.0	± 0.0
Convulsions	0	0	0	0	0	0	0	0.0	± 0.0
Bizarre behaviors	0	0	0	0	0	0	0	0.0	± 0.0
Mobility	4	3	3	4	4	4	4	3.7	± 0.5
Alertness	4	4	4	4	4	4	4	4.0	± 0.0
Body position	4	4	4	4	4	4	4	4.0	± 0.0
Straub tail	0	0	0	0	0	0	0	0.0	± 0.0
Abnormal gait	0	0	0	0	0	0	0	0.0	± 0.0
Palpebral closure	0	2	2	2	0	0	0	1.0	± 1.1
Startle response	4	4	4	4	4	4	4	4.0	± 0.0
Touch response	4	4	4	4	4	4	4	4.0	± 0.0
Positional passivity	4	4	4	4	4	4	4	4.0	± 0.0
Visual placing	0	0	0	0	0	0	0	0.0	± 0.0
Grip strength	4	4	4	4	4	4	4	4.0	± 0.0
Body sag	0	0	0	0	0	0	0	0.0	± 0.0
Pinna reflex	4	4	4	4	4	4	4	4.0	± 0.0
Corneal reflex	4	4	4	4	4	4	4	4.0	± 0.0
Flexor reflex	4	4	4	4	4	4	4	4.0	± 0.0
Skin colour	4	4	4	4	4	4	4	4.0	± 0.0
Limb tone	4	4	4	4	4	4	4	4.0	± 0.0
Abdomen tone	4	4	4	4	4	4	4	4.0	± 0.0
Pupil response	0	0	0	0	0	0	0	0.0	± 0.0
Lacrimation	0	0	0	0	0	0	0	0.0	± 0.0
Salivation	0	0	0	0	0	0	0	0.0	± 0.0
Biting	0	0	0	0	0	0	0	0.0	± 0.0
Tail pinch response	4	4	4	4	4	4	4	4.0	± 0.0
Righting reflex	0	0	0	0	0	0	0	0.0	± 0.0
Ease of handling	4	4	4	4	4	4	4	4.0	± 0.0
Fear	0	0	0	0	0	0	0	0.0	± 0.0
Aggressivity	0	0	0	0	0	0	0	0.0	± 0.0
Vocalization	0	0	0	0	0	0	0	0.0	± 0.0
Stools	0	0	0	0	0	0	0	0.0	± 0.0
Urines	0	0	0	0	0	0	0	0.0	± 0.0
Other symptoms	0	0	0	0	0	0	0	0.0	± 0.0

APPENDIX 1.4 - GENERAL BEHAVIOR, FEXINIDAZOLE 1000 mg/kg

24 hours

Symptoms	Reference values	Animal No						Mean ± S.D.	
		2894	2895	2896	2897	2898	2899		
Piloerection	0	0	0	0	0	0	0	0.0	± 0.0
Eye prominence	0	0	0	0	0	0	0	0.0	± 0.0
Respiration	4	4	4	4	4	4	4	4.0	± 0.0
Tremors	0	0	0	0	0	0	0	0.0	± 0.0
Twitches	0	0	0	0	0	0	0	0.0	± 0.0
Convulsions	0	0	0	0	0	0	0	0.0	± 0.0
Bizarre behaviors	0	0	0	0	0	0	0	0.0	± 0.0
Mobility	4	3	4	4	4	4	4	3.8	± 0.4
Alertness	4	4	4	4	4	4	4	4.0	± 0.0
Body position	4	4	4	4	4	4	4	4.0	± 0.0
Straub tail	0	0	0	0	0	0	0	0.0	± 0.0
Abnormal gait	0	0	0	0	0	0	0	0.0	± 0.0
Palpebral closure	0	2	0	0	0	0	0	0.3	± 0.8
Startle response	4	4	4	4	4	4	4	4.0	± 0.0
Touch response	4	4	4	4	4	4	4	4.0	± 0.0
Positional passivity	4	4	4	4	4	4	4	4.0	± 0.0
Visual placing	0	0	0	0	0	0	0	0.0	± 0.0
Grip strength	4	4	4	4	4	4	4	4.0	± 0.0
Body sag	0	0	0	0	0	0	0	0.0	± 0.0
Pinna reflex	4	4	4	4	4	4	4	4.0	± 0.0
Corneal reflex	4	4	4	4	4	4	4	4.0	± 0.0
Flexor reflex	4	4	4	4	4	4	4	4.0	± 0.0
Skin colour	4	4	4	4	4	4	4	4.0	± 0.0
Limb tone	4	4	4	4	4	4	4	4.0	± 0.0
Abdomen tone	4	4	4	4	4	4	4	4.0	± 0.0
Pupil response	0	0	0	0	0	0	0	0.0	± 0.0
Lacrimation	0	0	0	0	0	0	0	0.0	± 0.0
Salivation	0	0	0	0	0	0	0	0.0	± 0.0
Biting	0	0	0	0	0	0	0	0.0	± 0.0
Tail pinch response	4	4	4	4	4	4	4	4.0	± 0.0
Righting reflex	0	0	0	0	0	0	0	0.0	± 0.0
Ease of handling	4	4	4	4	4	4	4	4.0	± 0.0
Fear	0	0	0	0	0	0	0	0.0	± 0.0
Aggressivity	0	0	0	0	0	0	0	0.0	± 0.0
Vocalization	0	0	0	0	0	0	0	0.0	± 0.0
Stools	0	0	0	0	0	0	0	0.0	± 0.0
Urines	0	0	0	0	0	0	0	0.0	± 0.0
Other symptoms	0	0	0	0	0	0	0	0.0	± 0.0

APPENDIX 1.5 - BODY TEMPERATURE

Dose	Time from Treatment	Animal No						Mean ± S.D.
		2876	2877	2878	2879	2880	2881	
	Basal	38.2	38.4	38.6	38.3	38.2	38.3	38.3 ± 0.2
**Vehicle**	2 hours	37.5	38.4	38.4	37.7	37.7	37.7	37.9 ± 0.4
	24 hours	37.9	38.5	38.7	38.0	38.2	38.2	38.3 ± 0.3

Dose	Time from Treatment	Animal No						Mean ± S.D.
		2882	2883	2884	2885	2886	2887	
**Fexinidazole**	Basal	37.8	37.9	38.2	38.4	37.8	38.2	38.1 ± 0.3
**100 mg/kg**	2 hours	37.9	38.2	38.5	38.3	38.7	38.4	38.3 ± 0.3
	24 hours	37.9	38.1	38.2	38.1	38.1	38.7	38.2 ± 0.3

Dose	Time from Treatment	Animal No						Mean ± S.D.
		2888	2889	2890	2891	2892	2893	
**Fexinidazole**	Basal	38.3	37.8	38.7	38.2	37.6	38.0	38.1 ± 0.4
**300 mg/kg**	2 hours	38.4	37.5	38.9	37.8	37.8	38.3	38.1 ± 0.5
	24 hours	37.5	37.5	38.7	37.9	37.4	38.6	37.9 ± 0.6

Dose	Time from Treatment	Animal No						Mean ± S.D.
		2894	2895	2896	2897	2898	2899	
**Fexinidazole**	Basal	37.1	37.7	37.8	38.0	38.1	37.6	37.7 ± 0.4
**1000 mg/kg**	2 hours	38.7	37.8	38.1	38.2	38.5	38.4	38.3 ± 0.3
	24 hours	38.6	37.7	38.4	37.8	38.0	38.1	38.1 ± 0.3

Appendix 2 Protocol and Amendment

Appendix 3 Pharmacy Certification

PHARMACY CERTIFICATION

Pharm. Cert. 0508-2007

TEST ARTICLE: **Fexinidazole**
STUDY NUMBER: **0508-2007**
NOTEBOOK NUMBERS: **G0088**

DOCUMENTATION ENCLOSED IN THE NOTEBOOK:

A. Analysis certificate dated December 18, 2007 issued by Orgasynth Industries for Fexinidazole test item, raw material, Batch No. 3168-07-01/O
B. Material safety data sheet issued by Orgasynth Industries for Fexinidazole
C. Certificate of Analysis issued by Sigma-Aldrich for Tween® 80, raw material, Lot No. 1239316
D. Certificate of Analysis issued by Sigma-Aldrich for Methylcellulose 400 cP, raw material, Lot No. 105K0074
E. Label's photocopy of Acqua per preparazioni iniettabili (Bieffe Medital S.p.A.), raw material, Lot No. 07G0201

ANALYTICAL DOCUMENTATION:

Requests and Analytical Results issued by Accelera/ADMET/Preclinical Formulation and Accelera/DMPK&ART/Bioanalysis and Analytical Control

MATERIALS USED FOR THE STUDY:

1. Fexinidazole test item, raw material, Batch No. 3168-07-01/O
2. Tween® 80, raw material, Lot No. 1239316
3. Methylcellulose 400 cP, raw material, Lot No. 105K0074
4. Acqua per preparazioni iniettabili, raw material, Lot No. 07G0201

PREPARATIONS:

Prepare suspension of Fexinidazole test item, raw material, Lot No. 3168-07-01/O in the vehicle (5% Tween® 80 in 0.5% Methylcellulose 400 cP solution) at the concentration of 5 mg/mL, 15 mg/mL and 50 mg/mL

CONCENTRATION CHECKS: (ACCURANCY LIMITS: ± 10% OF LABELED AMOUNT)

Fexinidazole suspensions	Preparation date	Request No.	% of L.A.
5 mg/mL - TOP	5th February 2008	Request No. 200800042	103.52
5 mg/mL - MIDDLE	5th February 2008	Request No. 200800043	108.01
5 mg/mL - BOTTOM	5th February 2008	Request No. 200800044	104.44
15 mg/mL - TOP	5th February 2008	Request No. 200800039	99.54
15 mg/mL - MIDDLE	5th February 2008	Request No. 200800040	102.48
15 mg/mL - BOTTOM	5th February 2008	Request No. 200800041	102.84
50 mg/mL - TOP	5th February 2008	Request No. 200800036	102.65
50 mg/mL - MIDDLE	5th February 2008	Request No.200800037	100.55
50 mg/mL - BOTTOM	5th February 2008	Request No.200800038	105.10

STABILITY:

Fexinidazole test item:

Expire date October 2008 for Fexinidazole, test item, raw material, Lot No. 3168-07-01/O if stored at room temperature protected from light

Fexinidazole suspensions:

Stability data indicate that Fexinidazole suspensions in the vehicle (5% Tween® 80 in 0.5% Methylcellulose 400 cP solution) in the range 0.5-100 mg/mL are stable up to 7 days at room temperature and 14 days at +4°C (Nerviano MS 0293-2007-R)

Prepared by: 11th March2008
