# Supplementary material for: Fexinidazole – A New Oral Nitroimidazole Drug Candidate Entering Clinical Development for the Treatment of Sleeping Sickness
Source: PLoS Negl Trop Dis. 2010 Dec 21;4(12):e923. doi: 10.1371/journal.pntd.0000923 (PMC3006138; doi:10.1371/journal.pntd.0000923)
Supplement: Dataset S15 — (0.46 MB PDF) [file pntd.0000923.s016.pdf]

Fexinidazole: Effects on Respiratory Parameters in the Unrestrained Conscious Male Rat after Oral Administration

Product Name :	Fexinidazole
Study Number:	0509-2007
Study Director/Author:	
Sponsor Reference Study No.:	Not Applicable
Status:	Final

SUMMARY

The purpose of this study (0509-2007) was to investigate the potential effects of fexinidazole, an agent under investigation for the treatment of the Human African trypanosomiasis (HAT), on the respiratory function in the conscious unrestrained rats following a single oral administration.

Groups of eight male rats were given a single dose of fexinidazole at dosages of 0 (vehicle), 100, 300 or 1000 mg/kg.

The effect of treatment on respiratory function was assessed by placing the animals in individual plethysmographic chambers and recording respiratory parameters for 4 hours after treatment.

Respiratory parameters were not affected by oral administration of fexinidazole at any of the doses tested and were relatively stable throughout the 4-hour recording period following treatment, with the exception of time point changes in a few parameters which were scattered among dose groups and were mainly due to changes in individual animals.

In conclusion, oral administration of fexinidazole to male rats at doses of 100, 300 and 1000 mg/kg did not have any effect on respiratory parameters. The No-Observed Effect Level (NOEL) for the test item can therefore be defined at 1000 mg/kg.

TABLE OF CONTENTS

1. INTRODUCTION AND OBJECTIVES ... 8
2. STUDY SPONSOR ... 8
3. TEST FACILITY ... 8
4. REGULATORY REQUIREMENTS ... 8
5. SCHEDULE ... 8
6. MATERIALS AND METHODS ... 9
6.1. Test and Control Items ... 9
6.1.1. Test Item ... 9
6.1.2. Vehicle/Control Item ... 9
6.1.3. Test Formulation ... 9
6.1.4. Test Formulation Analyses ... 9
6.2. Test System ... 10
6.2.1. Identification ... 10
6.2.2. Husbandry and Environmental Conditions ... 10
6.3. Experimental Design ... 11
6.3.1. Experimental Groups ... 11
6.3.2. Allocation/Randomization ... 11
6.3.3. Dose Administration ... 11
6.3.4. Dose Justification ... 11
6.4. Experimental procedure ... 12
6.4.1. Deviations from Protocol ... 12
7. ARCHIVING ... 13
8. STUDY PERSONNEL ... 13
9. RESULTS ... 13
10. CONCLUSIONS ... 14
11. FIGURES ... 15

APPENDICES

Appendix 1. Study Data Listings

- Appendix 1.1. Vehicle
- Appendix 1.2. Fexinidazole, 100 mg/kg
- Appendix 1.3. Fexinidazole, 300 mg/kg
- Appendix 1.4. Fexinidazole, 1000 mg/kg

Appendix 2. Protocol and Amendment

Appendix 3. Pharmacy Certification

1. INTRODUCTION AND OBJECTIVES

Fexinidazole is a 5-nitroimidazole derivative, biologically active against Trypanosoma parasites (T.b.rhodesiense and T.b. brucei), under investigation for the treatment of the Human African trypanosomiasis (HAT), known as sleeping sickness. The purpose of this study (0509-2007) was to determine the potential adverse effects of the test item on the respiratory function in the conscious unrestrained rats following a single oral administration.

2. STUDY SPONSOR

Drugs for Neglected Diseases *initiative* (DND*i*)
1, Place St Gervais
CH-1201 Geneva
Switzerland

3. TEST FACILITY

Accelera

4. REGULATORY REQUIREMENTS

This study was conducted in compliance with:

- Decreto Legislativo 2 Marzo 2007, No. 50;
- Organisation for Economic Co-operation and Development (OECD) Principles of Good Laboratory Practice (GLP) (as revised in 1997).

The methods employed in this study were those described in the "Standard Operating Procedures" of the laboratories involved.

5. SCHEDULE

Experimental Start Date (randomization of animals)	4th February 2008
First day of treatment (first 2 rats/group)	5th February 2008
Last day of treatment (last 2 rats/group)	8th February 2008
Experimental Completion Date (end of experimental recording)	8th February 2008

6. MATERIALS AND METHODS

6.1. Test and Control Items

6.1.1. Test Item

Identification	Fexinidazole
Lot/Batch Number	3168-07-01/O
Purity and Expiry	100.2%, October 2008
Storage Conditions	Room temperature, protected from light
Source and Manufacturer	Centipharm (formerly Orgasynth, as communicated by the Sponsor)
Special Handling Precautions	Usual protection of all personnel conducting the study (mask, gloves and eyeglasses), according to MSDS (Material Safety Data Sheet)

6.1.1.1. Deviations from Protocol

In the protocol, the specification "protected from light" (reported on the MSDS) was inadvertently omitted. The test item has, however, been stored protected from light as requested.

6.1.2. Vehicle/Control Item

Identification	5% Tween 80 in 0.5% Methyl cellulose 400 cP (Methocel)	
Lot/Batch Number	Tween 80<br>Methyl cellulose 400 cP	1239316<br>105K0074
Expiry	Tween 80<br>Methyl cellulose 400 cP	November 2010<br>October 2008
Storage Conditions	Room temperature	
Source and Manufacturer	Tween 80<br>Methyl cellulose 400 cP	Sigma-Aldrich<br>Sigma-Aldrich
Method of Preparation	On file at Accelera/ADMET/Preclinical Formulation	

6.1.3. Test Formulation

Type of Formulation	Suspension in vehicle
Method of Preparation	On file at Accelera/ADMET/Preclinical formulation
Frequency of Preparation	Suspensions were prepared according to the stability data
Dose Concentrations	5, 15, 50 mg/mL
Storage Conditions	Room temperature in the dark
Source and Manufacturer	Accelera/ADMET/Preclinical Formulation

6.1.4. Test Formulation Analyses

6.1.4.1. Concentration and Homogeneity

Samples (top-middle-bottom, 5 mL each) of each dose suspension were collected under

stirring for concentration and homogeneity check test of fexinidazole; 10 mL were taken also from the vehicle. After collection, samples were directly transferred at +4°C to Bioanalysis & Analytical Control for analysis. The analyses were performed using a validated HPLC-UV method.

All values were found to be within acceptable limits (see Appendix 3).

6.1.4.2. Stability

Stability data indicate that fexinidazole suspensions in 5% Tween 80 in 0.5% methyl cellulose 400 cP (Methocel) in the range 0.5 - 100 mg/mL are stable up to 7 days at room temperature and 14 days at +4°C (NervianoMS 0293-2007-R).

6.2. Test System

Species/Strain and Source	Rat/Crl:CD(SD)BR, Charles River Labs Italia S.p.A. (Calco, Lecco)
Justification of Species and Sex	The rat has been used extensively in safety studies and a large amount of biological data is available. Only males were used since no significant gender-related differences in systemic exposure are expected.
Age	About 8 weeks on the day of dosing
Weight	255-313 g on the day of dosing
Acclimation	At least 6 days
Selection Criteria	Body weight and physical examination

6.2.1. Identification

Test Group	Color Code	Animal Identification No.
1	White	2833 - 2834 - 2835 - 2836 - 2837 - 2838 - 2839 - 2840
2	Yellow	2841 - 2842 - 2843 - 2844 - 2845 - 2846 - 2847 - 2848
3	Green	2849 - 2850 - 2851 - 2852 - 2853 - 2854 - 2855 - 2856
4	Red	2857 - 2858 - 2859 - 2860 - 2861 - 2862 - 2863 - 2864

Each animal was identified with a metal ear tag bearing a unique serial number. A color-coded cage card was affixed to each study animal's cage, indicating the study number, animal number, and dose level.

6.2.2. Husbandry and Environmental Conditions

Room	#120 (acclimatization), #114 (experiments) Blg 64 rodent facility
Caging	Makrolon cages (59x38.5x20 cm, Tecniplast Gazzada, Buguggiate, Varese)
Bedding	700/2000 sawdust bedding (L.G. Packing Wood S.r.l., Condove, Turin).
Temperature	21.5 ± 1.5°C
Humidity	55 ± 15%
Lighting	Approximate 12-hour light, 12-hour dark cycle.
Ventilation	At least 20 room air changes per hour
Water	Municipal main water in bottles
Diet	4RF21GLP pellets, (Mucedola s.r.l., Settimo Milanese, Milan)

Environmental conditions were continuously monitored and recordings were retained. Release of each lot of feed (and bedding) by the manufacturers is based on analysis of composite samples of each lot, which has met specifications set by the manufacturers. Water is periodically analyzed for chemical and microbial impurities. No contaminants have been identified in the food or water (or bedding), which are expected to interfere with the results or conclusions of this study.

All the above environmental conditions, as well as all the procedures adopted throughout the study for housing and handling the animals, were in strict compliance with EU and Italian Guidelines for Laboratory Animal Welfare.

6.3. Experimental Design

6.3.1. Experimental Groups

Exp. Session	Treatment[(a)]	Dose (mg/kg)	Number of Animals/Group (Males only)
1	Vehicle	0 (vehicle)	8
2	Fexinidazole	100	8
3	Fexinidazole	300	8
4	Fexinidazole	1000	8
(a) Two animals/group were dosed each day of study.			

6.3.2. Allocation/Randomization

The animals were selected and distributed into experimental groups using random number tables (Fisher and Yates).

6.3.3. Dose Administration

Route and Method	Oral, gastric gavage
Duration	Single dose
Frequency	Once
Volume	20 mL/kg
Rate	Bolus

Dosage volumes were adjusted to the individual animal body weights recorded on the day of dosing.

6.3.4. Dose Justification

Doses of 100, 300 and 1000 mg/kg of fexinidazole were selected based on the results of a repeated (7-day) toxicity study in the same species (0339-2007). In this study, the top dose of 2000 mg/kg was well tolerated and did not cause any meaningful toxicological change. On the other hand, plasma levels determined as part of a 3-day toxicity study (study 0513-2007) showed that systemic exposure (both in terms of Cmax and AUC) did not increase when the dose went from 500 to 1000 mg/kg/day.

The oral route is that intended for clinical administration.

6.4. Experimental procedure

On the day of dosing, animals were placed in individual plethysmographic chambers. After an acclimation period of at least 1 hour, and once respiratory parameters had stabilized, recording of respiratory signals was started and basal data were recorded for at least 30 minutes. Animals were taken from the chambers, orally dosed and placed again in the chambers for at least 4 hours.

Respiratory parameters were acquired continuously at the sampling frequency of 500 Hz by Notocord HEM 3.4 software and were extracted, following visual inspection, as the average value of 6-second recordings every 5 minutes. If the signal in the 6-second recording to be extracted was deemed not to be of good quality, the extraction zone was moved forward or backward for a maximum of 150 seconds. In case the quality of the signal did not improve, the time point was not extracted. Basal values were calculated as the mean of values extracted every 5 minutes from 30 to 10 minutes before treatment. After treatment, values were extracted every 30 minutes up to 4 hours. The following parameters were calculated from each respiratory cycle:

- Respiratory rate (f, breath per minutes)
- Peak inspiration flow (PIF, mL/s)
- Peak expiration flow (PEF, mL/s)
- Inspiration time (IT, msec)
- Expiration time (ET, msec)
- Relaxation time (RT, msec)
- Tidal volume (TV, mL/kg)
- Minute volume (MV, mL/kg/min calculated as TV*f)
- Enhanced Pause (Penh, calculated as ((ET/RT)-1)*(PEF/PIF))

Body weight (grams) was recorded on the day of treatment for calculation of dose volumes and for calculation of TV and MV per kg of body weight. It is not reported here but it is archived with the study file.

At the end of the experiment animals were euthanized by exposure to CO_2/O_2.

6.4.1. Deviations from Protocol

Some data points are not available due to unreadable waveforms (probably caused by movements or sniffing of the animals). In such cases, data in Appendix 1 are reported as "-". The following table summarizes all instances in which data are not available:

Treatment	Animal No.	Time from dosing
Vehicle	2833	210 min
	2837	30 min
Fexinidazole, 100 mg/kg	2842	60 and 210 min
Fexinidazole, 300 mg/kg	2852	90 min
	2856	210 min
Fexinidazole, 1000 mg/kg	2858	120, 150 and 180 min
	2861	-25, -20 and –10 min

However, the lack of these data is deemed not to have any influence on the interpretation of the results of the study.

7. ARCHIVING

The original protocol, all protocol amendments, all raw data, supporting documents, and specimens produced at the Test Facility, and the final report with original signatures were filed in the Archives of Accelera, Nerviano Medical Sciences S.r.l., Nerviano (Italy), where they will be kept for the period of time agreed with the Sponsor (at least 3 years) after which the Sponsor will be contacted for instructions regarding dispatch or disposal of the material.

A copy of the protocol, the report with original signatures, a reserve sample and all relevant original documentation of the test item were filed by the Sponsor.

8. STUDY PERSONNEL

9. RESULTS

Respiratory parameters were not affected by oral administration of fexinidazole at any of the doses tested and were relatively stable throughout the 4-hour recording period following treatment. Some decreases in peak inspiratory flow (PIF) were observed from 120 minutes after treatment, but they were scattered among dose groups and were mainly due to time

point changes observed in individual animals (in group 3, fexinidazole 300 mg/kg, animals no. 2850 and 2852 at 180 minutes; in group 4, fexinidazole 1000 mg/kg, animal no. 2863 at 120 minutes and animal no. 2861 at several time points). Enhanced pause (Penh) values also showed some changes, but these were again scattered throughout the experimental period and the dose groups, therefore they are not considered related to the treatment.

10. CONCLUSIONS

Oral administration of fexinidazole to male rats at doses of 100, 300 and 1000 mg/kg did not have any effect on respiratory parameters. The No-Observed-Effect-Level (NOEL) for the test item can therefore be defined at 1000 mg/kg.

11. FIGURES

Figure 1 – Tidal volume

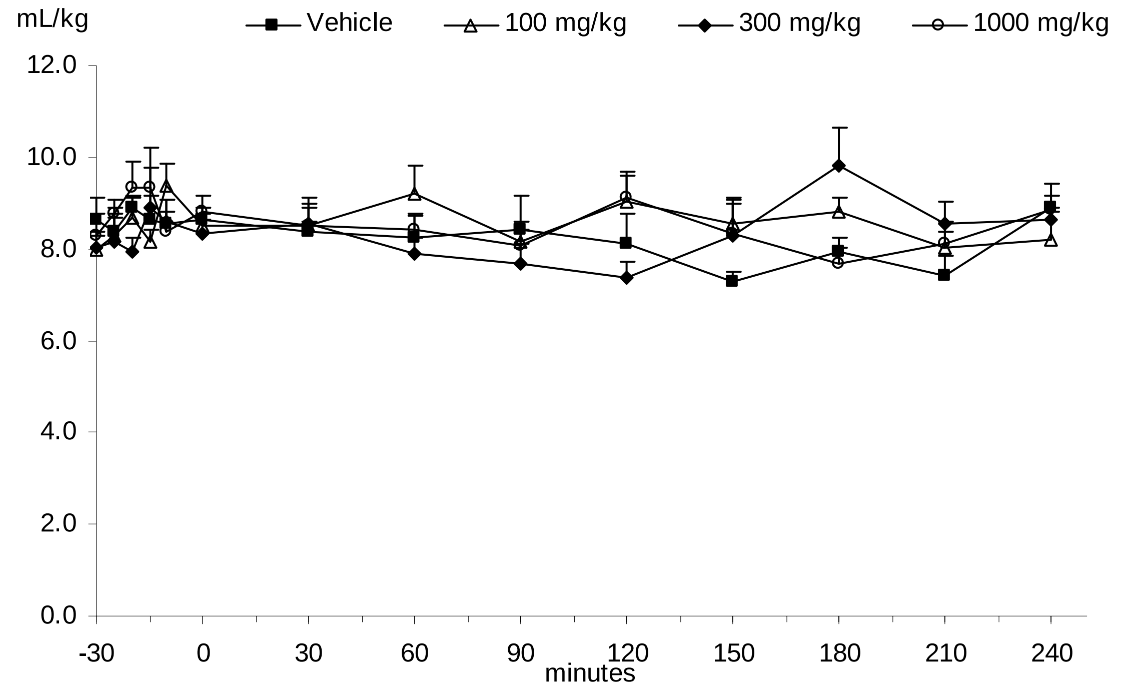

Figure 2 – Minute volume

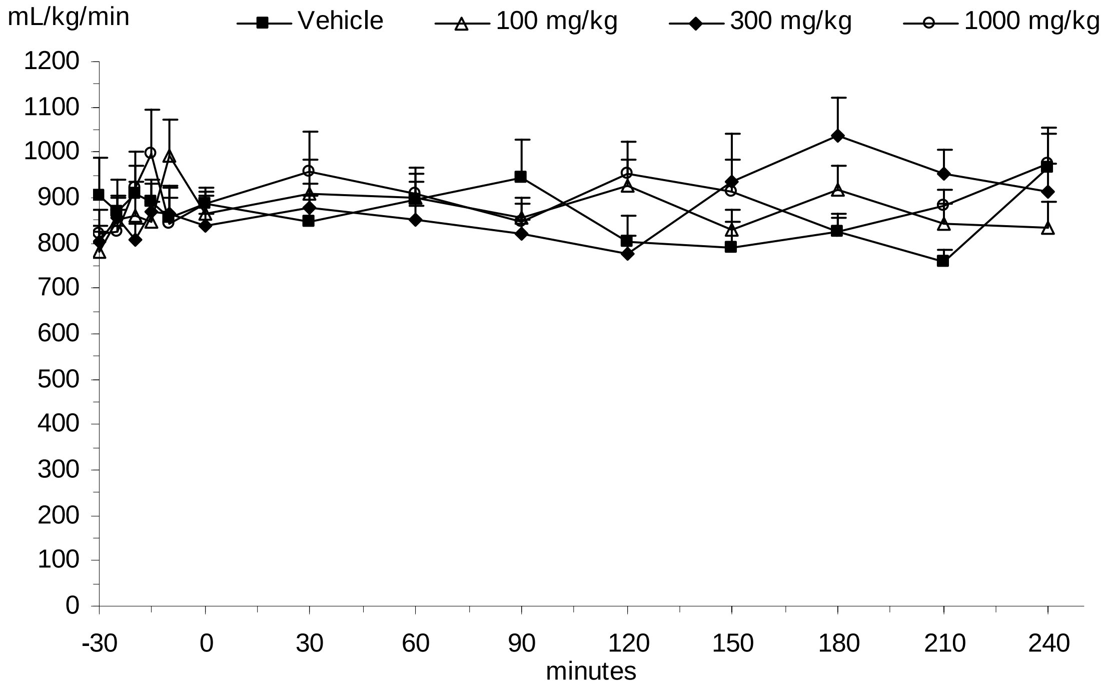

Figure 3 – Peak inspiration flow

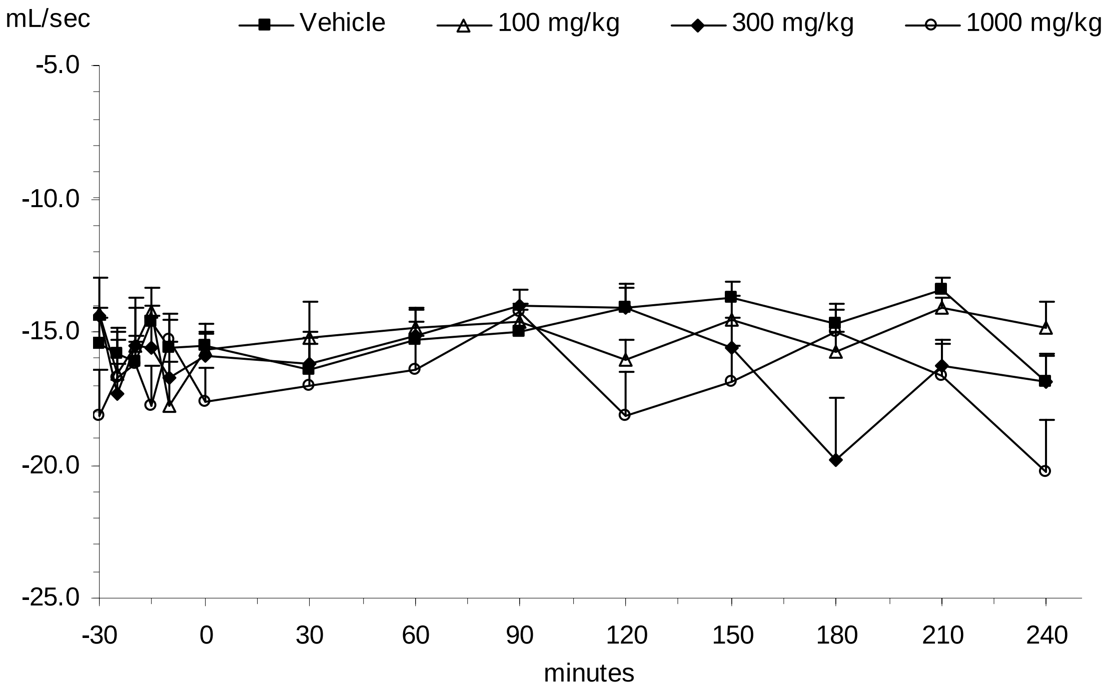

Figure 4 – Peak expiration flow

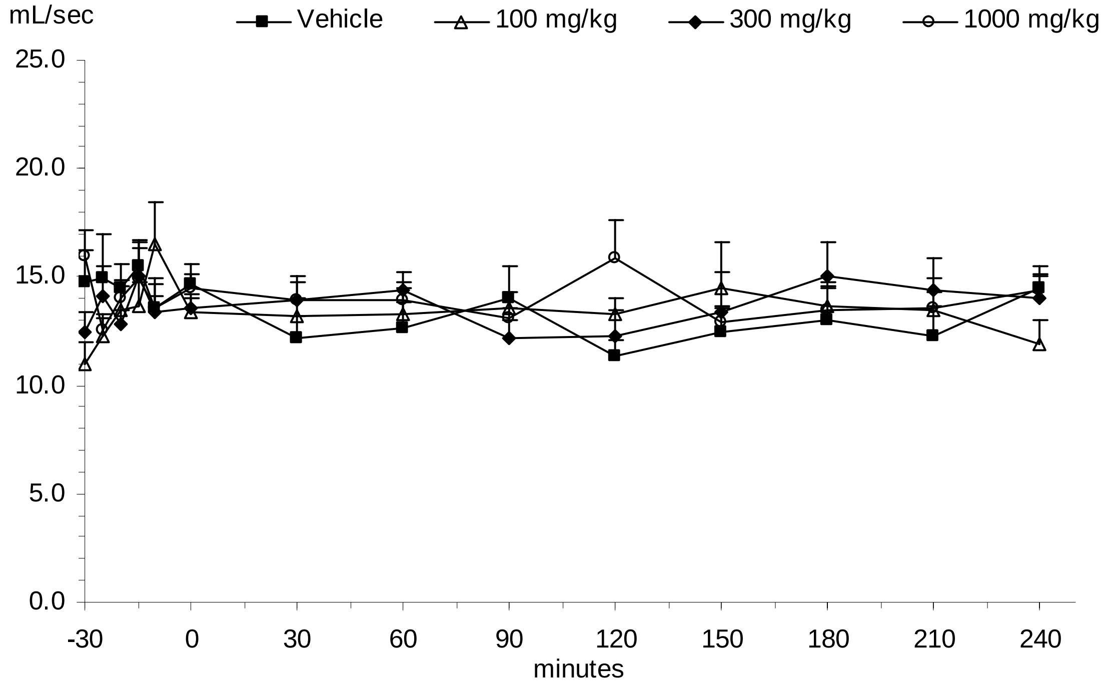

Figure 5 – Inspiration time

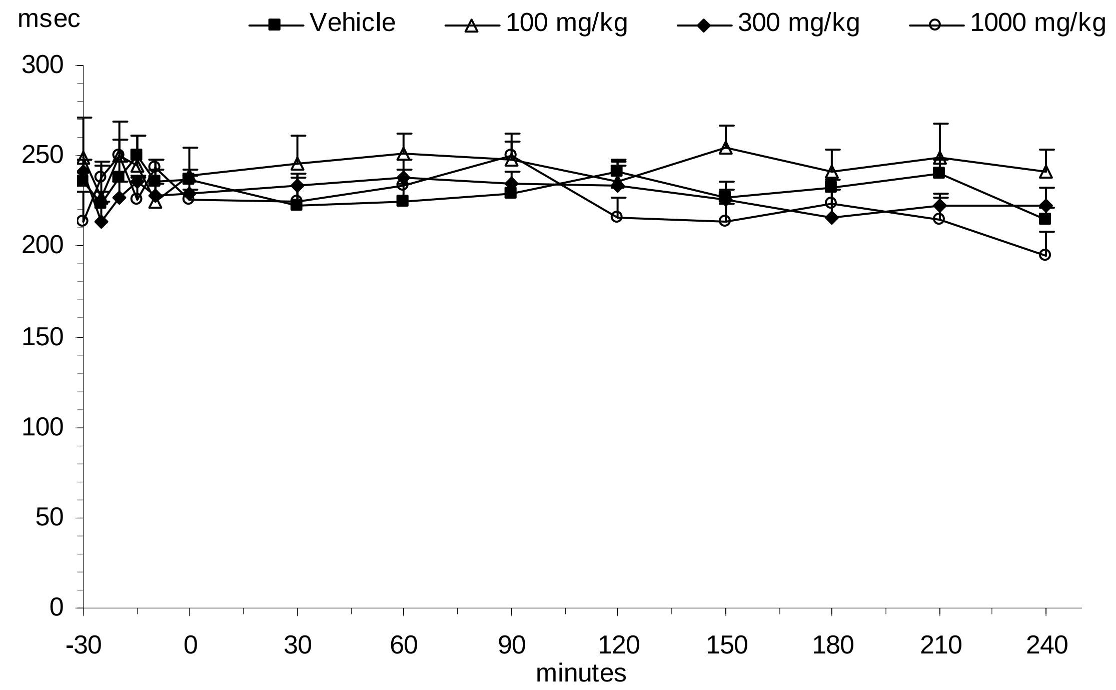

Figure 6 - Expiration time

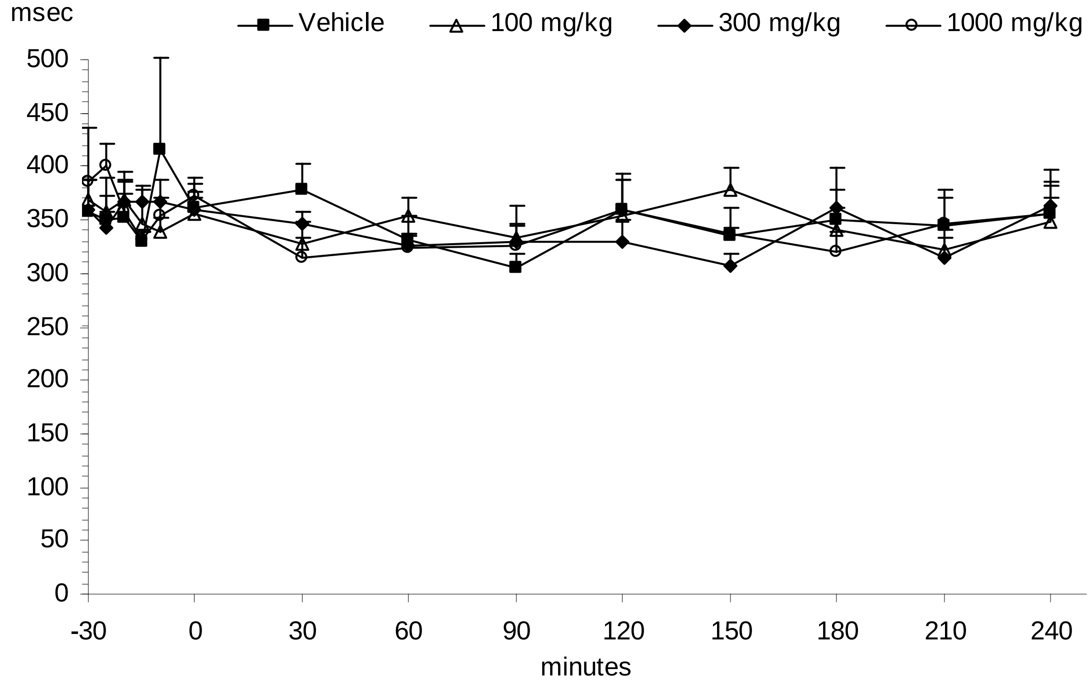

Figure 7 – Relaxation time

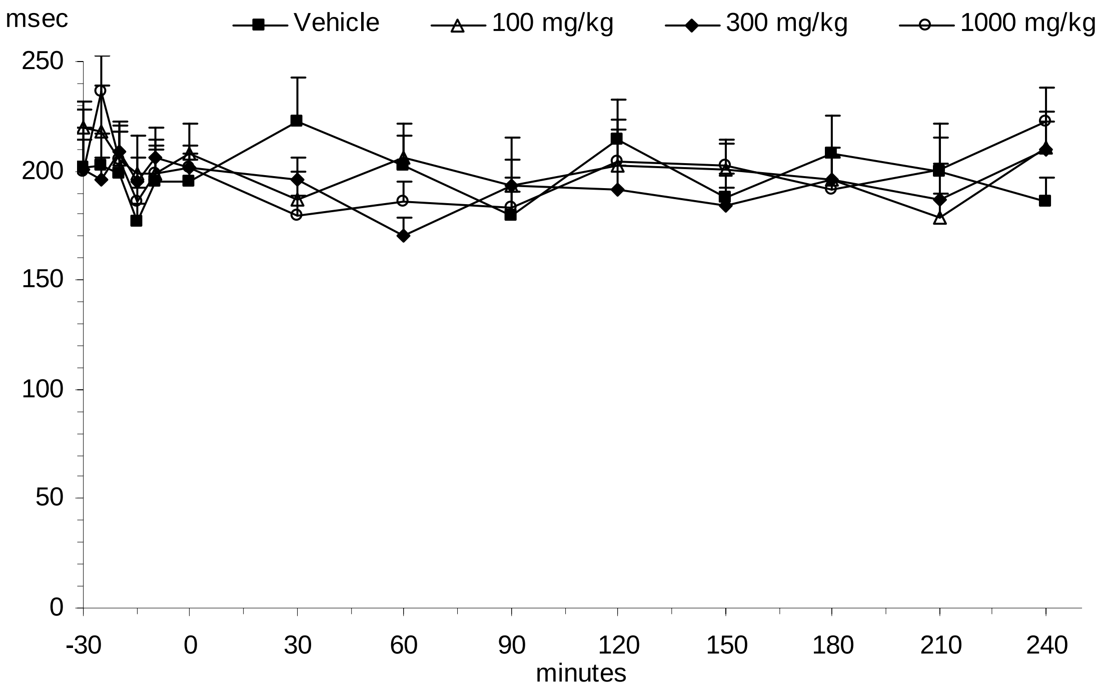

Figure 8 – Respiratory rate

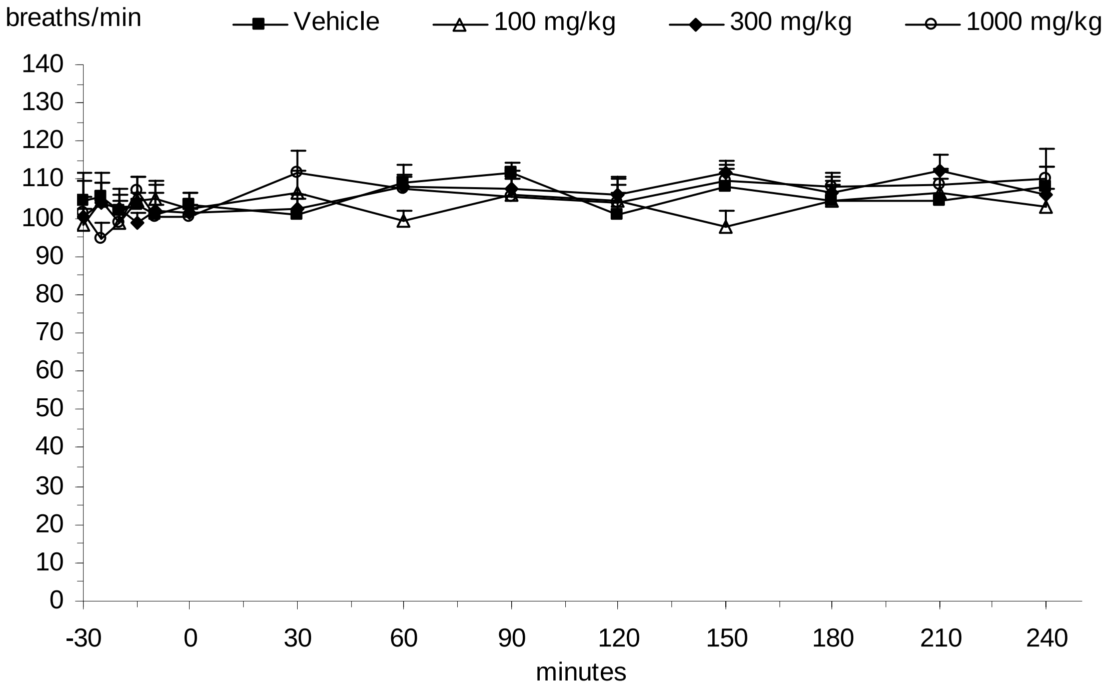

Figure 9 – Penh

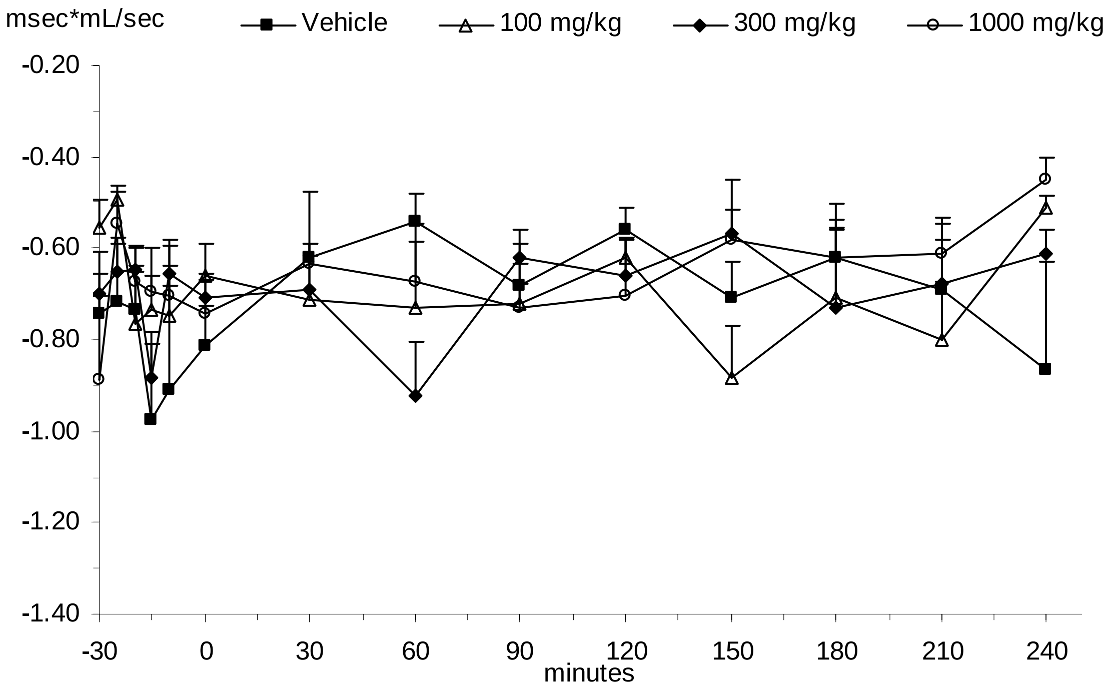

APPENDICES

Appendix 1 Study Data Listings

Appendix 1.1 – Vehicle PO

Tidal volume (mL/kg)

Time from treatment (min)	Animal number								Mean ± S.D.	N
	2833	2834	2835	2836	2837	2838	2839	2840		
-30	10.7	9.3	7.8	8.1	8.8	6.7	7.5	10.2	**8.6 ± 1.4**	**8**
-25	10.3	7.1	7.6	10.4	9.5	6.5	6.8	8.6	**8.4 ± 1.6**	**8**
-20	8.5	9.3	9.2	9.4	9.4	9.2	7.5	8.7	**8.9 ± 0.7**	**8**
-15	10.6	10.5	9.1	8.5	8.4	7.0	8.4	6.8	**8.6 ± 1.4**	**8**
-10	9.6	6.8	9.3	11.0	8.3	7.8	9.1	6.6	**8.6 ± 1.5**	**8**
0	9.9	8.6	8.6	9.5	8.9	7.4	7.9	8.2	**8.6 ± 0.8**	**8**
30	9.2	7.8	9.0	8.5	-	8.0	8.2	7.9	**8.4 ± 0.6**	**7**
60	9.8	6.8	8.5	8.2	6.6	7.5	7.9	10.7	**8.2 ± 1.4**	**8**
90	12.2	6.3	7.3	8.4	10.3	6.6	7.6	8.9	**8.4 ± 2.0**	**8**
120	10.9	6.2	7.4	7.9	7.6	6.2	8.2	10.6	**8.1 ± 1.8**	**8**
150	7.6	6.0	7.9	7.9	7.7	7.1	7.4	6.8	**7.3 ± 0.6**	**8**
180	8.5	7.9	8.3	8.0	5.8	7.8	9.1	8.1	**7.9 ± 0.9**	**8**
210	-	7.2	8.9	8.0	7.1	6.7	5.4	8.4	**7.4 ± 1.2**	**7**
240	9.3	7.2	9.1	10.6	7.5	8.4	7.9	11.2	**8.9 ± 1.4**	**8**

Minute volume (mL/kg/min)

Time from treatment (min)	Animal number								Mean ± S.D.	N
	2833	2834	2835	2836	2837	2838	2839	2840		
-30	978	1241	600	752	1183	793	646	1040	**904 ± 242**	**8**
-25	963	858	751	976	908	708	823	949	**867 ± 100**	**8**
-20	1040	1143	911	761	1037	897	621	869	**910 ± 167**	**8**
-15	1160	893	921	900	936	727	888	717	**893 ± 138**	**8**
-10	628	849	944	1199	1075	796	721	632	**855 ± 206**	**8**
0	954	997	825	918	1028	784	740	841	**886 ± 104**	**8**
30	1088	910	909	832	-	855	690	659	**849 ± 145**	**7**
60	1154	790	818	1036	767	819	640	1157	**898 ± 193**	**8**
90	1453	742	849	884	1120	726	898	890	**945 ± 238**	**8**
120	1194	796	703	771	758	697	721	793	**804 ± 162**	**8**
150	671	726	893	906	1062	815	692	557	**790 ± 161**	**8**
180	1030	877	704	779	666	858	741	930	**823 ± 123**	**8**
210	-	816	683	752	770	779	664	854	**760 ± 68**	**7**
240	1254	816	1002	1014	762	989	681	1221	**967 ± 206**	**8**

Respiratory rate (breaths/min)

Time from treatment (min)	Animal number								Mean ± S.D.	N
	2833	2834	2835	2836	2837	2838	2839	2840		
-30	91	133	77	93	134	118	86	102	**104 ± 22**	**8**
-25	94	121	99	94	95	110	121	110	**105 ± 12**	**8**
-20	123	123	99	81	110	98	83	100	**102 ± 16**	**8**
-15	110	85	101	106	112	104	105	106	**104 ± 8**	**8**
-10	66	125	101	109	129	102	80	96	**101 ± 21**	**8**
0	97	117	95	97	116	106	95	103	**103 ± 9**	**8**
30	118	117	101	98	-	107	85	83	**101 ± 14**	**7**
60	117	117	97	127	116	109	81	109	**109 ± 14**	**8**
90	119	118	116	105	109	109	119	100	**112 ± 7**	**8**
120	110	129	94	97	100	113	88	75	**101 ± 17**	**8**
150	88	120	114	114	138	115	93	82	**108 ± 19**	**8**
180	122	112	85	98	115	110	82	114	**105 ± 15**	**8**
210	-	113	77	94	108	116	123	102	**105 ± 15**	**7**
240	134	113	110	95	101	118	86	109	**108 ± 15**	**8**

“-“ see text (point 6.4.1 Deviations from Protocol)

Appendix 1.1 – Vehicle PO

Penh (enhanced pause, ((ET/RT)-1)*(PEF/PIF))

Time from treatment (min)	Animal number								Mean ± S.D.	N
	2833	2834	2835	2836	2837	2838	2839	2840		
-30	-1.14	-0.51	-0.45	-0.91	-0.77	-0.57	-0.65	-0.93	**-0.74 ± 0.24**	**8**
-25	-0.55	-0.67	-0.42	-0.62	-0.56	-0.58	-0.78	-1.56	**-0.72 ± 0.36**	**8**
-20	-0.74	-0.64	-0.52	-0.68	-1.05	-0.44	-0.58	-1.24	**-0.73 ± 0.27**	**8**
-15	-0.85	-1.88	-0.45	-0.57	-1.07	-0.69	-0.91	-1.37	**-0.97 ± 0.47**	**8**
-10	-2.42	-0.71	-0.49	-1.00	-0.72	-0.76	-0.38	-0.78	**-0.91 ± 0.64**	**8**
0	-1.14	-0.88	-0.47	-0.76	-0.83	-0.61	-0.66	-1.17	**-0.81 ± 0.25**	**8**
30	-0.30	-1.40	-0.24	-0.59	-	-0.74	-0.65	-0.43	**-0.62 ± 0.39**	**7**
60	-0.59	-0.42	-0.30	-0.86	-0.41	-0.53	-0.66	-0.58	**-0.54 ± 0.17**	**8**
90	-0.49	-0.70	-0.50	-0.80	-1.16	-0.59	-0.87	-0.34	**-0.68 ± 0.26**	**8**
120	-0.52	-0.56	-0.38	-0.59	-0.55	-0.60	-0.83	-0.45	**-0.56 ± 0.13**	**8**
150	-0.46	-0.64	-0.92	-0.51	-0.71	-0.55	-0.76	-1.10	**-0.71 ± 0.22**	**8**
180	-0.53	-0.73	-0.34	-0.71	-0.59	-0.46	-0.49	-1.12	**-0.62 ± 0.24**	**8**
210	-	-0.76	-0.38	-0.77	-0.67	-0.53	-0.49	-1.24	**-0.69 ± 0.28**	**7**
240	-0.74	-0.75	-0.42	-0.69	-2.46	-0.38	-0.91	-0.58	**-0.87 ± 0.67**	**8**

Peak inspiration flow (mL/sec)

Time from treatment (min)	Animal number								Mean ± S.D.	N
	2833	2834	2835	2836	2837	2838	2839	2840		
-30	-14.1	-18.9	-11.7	-14.2	-19.3	-14.1	-14.2	-17.2	**-15.5 ± 2.7**	**8**
-25	-18.3	-12.7	-12.3	-19.7	-16.9	-13.0	-16.5	-17.2	**-15.8 ± 2.8**	**8**
-20	-13.3	-17.6	-13.8	-17.1	-19.4	-20.1	-13.5	-14.1	**-16.1 ± 2.8**	**8**
-15	-15.2	-14.4	-14.6	-16.2	-14.4	-13.4	-17.5	-11.6	**-14.7 ± 1.8**	**8**
-10	-21.2	-12.4	-14.0	-18.4	-15.7	-13.9	-18.5	-10.6	**-15.6 ± 3.6**	**8**
0	-16.4	-15.2	-13.3	-17.1	-17.1	-14.9	-16.0	-14.1	**-15.5 ± 1.4**	**8**
30	-19.8	-12.9	-21.7	-16.5	-	-16.4	-14.4	-13.4	**-16.5 ± 3.3**	**7**
60	-14.7	-13.3	-16.7	-17.3	-13.2	-15.8	-13.3	-18.2	**-15.3 ± 2.0**	**8**
90	-19.4	-9.9	-13.0	-15.6	-17.8	-13.6	-14.1	-16.5	**-15.0 ± 3.0**	**8**
120	-15.7	-11.9	-12.2	-13.1	-14.0	-12.6	-15.4	-17.6	**-14.1 ± 2.0**	**8**
150	-14.0	-10.8	-14.7	-14.6	-15.8	-15.2	-13.4	-11.5	**-13.7 ± 1.8**	**8**
180	-13.0	-12.9	-14.6	-13.7	-12.4	-16.0	-16.9	-17.8	**-14.7 ± 2.0**	**8**
210	-	-11.7	-14.0	-13.8	-14.1	-15.3	-12.0	-13.3	**-13.5 ± 1.3**	**7**
240	-15.7	-13.6	-14.9	-18.8	-14.0	-18.9	-16.9	-22.3	**-16.9 ± 3.0**	**8**

Peak expiration flow (mL/sec)

Time from treatment (min)	Animal number								Mean ± S.D.	N
	2833	2834	2835	2836	2837	2838	2839	2840		
-30	16.8	17.3	8.6	12.1	19.9	11.8	12.1	19.6	**14.8 ± 4.2**	**8**
-25	14.5	12.0	8.6	16.6	14.3	10.5	15.4	27.5	**14.9 ± 5.7**	**8**
-20	14.0	14.6	11.2	13.2	19.2	13.0	11.4	19.4	**14.5 ± 3.2**	**8**
-15	17.1	20.9	11.0	12.2	17.0	11.3	18.1	16.1	**15.5 ± 3.6**	**8**
-10	12.6	12.5	11.4	20.2	15.6	13.5	11.5	11.3	**13.6 ± 3.0**	**8**
0	15.0	15.5	10.2	14.8	17.2	12.0	13.7	18.8	**14.6 ± 2.7**	**8**
30	10.7	16.3	9.0	11.9	-	14.5	12.9	10.3	**12.2 ± 2.5**	**7**
60	13.5	11.0	9.0	17.6	9.3	12.6	11.4	17.0	**12.7 ± 3.2**	**8**
90	15.1	11.1	10.2	13.7	22.8	11.5	16.2	11.3	**14.0 ± 4.1**	**8**
120	13.6	10.0	8.1	10.3	10.7	11.0	13.1	14.3	**11.4 ± 2.1**	**8**
150	8.0	9.8	16.7	10.6	17.1	12.0	11.8	13.3	**12.4 ± 3.2**	**8**
180	11.3	13.2	8.4	11.3	10.1	12.0	12.8	24.8	**13.0 ± 5.0**	**8**
210	-	11.9	8.9	12.1	12.2	11.4	9.7	19.8	**12.3 ± 3.5**	**7**
240	17.2	11.8	10.9	14.7	14.9	12.6	13.4	20.0	**14.5 ± 3.0**	**8**

“-“ see text (point 6.4.1 Deviations from Protocol)

Appendix 1.1 – Vehicle PO

Inspiration time (msec)

Time from treatment (min)	Animal number								Mean ± S.D.	N
	2833	2834	2835	2836	2837	2838	2839	2840		
-30	259	186	274	241	198	218	267	245	**236 ± 32**	**8**
-25	211	211	257	213	234	245	196	221	**223 ± 20**	**8**
-20	234	217	265	237	217	207	271	257	**238 ± 24**	**8**
-15	245	323	248	212	232	247	228	264	**250 ± 34**	**8**
-10	169	216	270	237	214	253	248	278	**236 ± 35**	**8**
0	223	231	263	228	219	234	242	253	**237 ± 15**	**8**
30	167	244	161	218	-	212	277	280	**223 ± 48**	**7**
60	223	203	210	198	214	230	291	228	**225 ± 29**	**8**
90	204	246	233	218	231	230	250	221	**229 ± 15**	**8**
120	240	212	258	243	243	226	257	256	**242 ± 16**	**8**
150	207	232	227	210	195	217	247	279	**227 ± 27**	**8**
180	232	233	246	233	223	220	258	211	**232 ± 15**	**8**
210	-	258	264	240	217	212	226	261	**240 ± 22**	**7**
240	199	223	244	228	216	197	215	201	**215 ± 16**	**8**

Expiration time (msec)

Time from treatment (min)	Animal number								Mean ± S.D.	N
	2833	2834	2835	2836	2837	2838	2839	2840		
-30	395	263	496	399	248	294	422	341	**357 ± 86**	**8**
-25	435	284	349	426	395	300	277	337	**350 ± 63**	**8**
-20	255	272	338	503	256	407	450	336	**352 ± 93**	**8**
-15	302	374	346	351	302	325	341	288	**329 ± 30**	**8**
-10	989	261	322	314	249	331	512	343	**415 ± 245**	**8**
0	475	291	370	399	290	331	400	329	**361 ± 63**	**8**
30	343	267	433	396	-	343	429	440	**379 ± 64**	**7**
60	287	310	411	258	301	319	448	321	**332 ± 64**	**8**
90	299	259	282	321	319	316	260	378	**304 ± 39**	**8**
120	306	251	374	373	356	302	413	507	**360 ± 78**	**8**
150	434	265	302	312	237	305	396	426	**335 ± 74**	**8**
180	259	304	455	380	297	323	470	319	**351 ± 77**	**8**
210	-	270	517	397	336	304	262	325	**344 ± 88**	**7**
240	246	307	301	396	463	311	479	347	**356 ± 82**	**8**

Relaxation time (msec)

Time from treatment (min)	Animal number								Mean ± S.D.	N
	2833	2834	2835	2836	2837	2838	2839	2840		
-30	202	169	307	193	142	175	240	188	**202 ± 51**	**8**
-25	257	167	218	245	236	175	151	171	**203 ± 41**	**8**
-20	149	153	206	268	124	242	268	177	**198 ± 56**	**8**
-15	172	163	216	200	159	179	182	145	**177 ± 23**	**8**
-10	196	153	201	164	144	186	318	199	**195 ± 54**	**8**
0	195	161	230	214	161	191	232	176	**195 ± 28**	**8**
30	220	127	275	218	-	187	248	283	**222 ± 54**	**7**
60	175	205	264	140	190	190	254	198	**202 ± 40**	**8**
90	184	160	173	168	168	186	148	252	**180 ± 32**	**8**
120	192	150	239	213	208	178	209	326	**214 ± 52**	**8**
150	240	156	167	183	143	180	213	218	**187 ± 33**	**8**
180	160	177	285	205	173	200	285	177	**208 ± 50**	**8**
210	-	155	324	212	190	178	163	177	**200 ± 58**	**7**
240	147	165	191	210	140	198	223	212	**186 ± 31**	**8**

“-“ see text (point 6.4.1 Deviations from Protocol)

Appendix 1.2 – Fexinidazole, 100 mg/kg PO

Tidal volume (mL/kg)

Time from treatment (min)	Animal number								Mean ± S.D.	N
	2841	2842	2843	2844	2845	2846	2847	2848		
-30	9.8	7.6	7.6	8.5	8.7	7.1	8.4	6.3	**8.0 ± 1.1**	**8**
-25	8.6	7.5	8.6	8.2	8.9	5.6	10.8	7.9	**8.3 ± 1.4**	**8**
-20	10.7	10.3	7.2	8.8	8.2	6.7	8.9	8.8	**8.7 ± 1.4**	**8**
-15	8.9	7.9	8.0	8.8	7.7	8.4	8.8	7.0	**8.2 ± 0.7**	**8**
-10	8.2	8.9	7.6	9.2	11.0	8.5	11.0	10.7	**9.4 ± 1.4**	**8**
0	9.2	8.5	7.8	8.7	8.9	7.3	9.6	8.1	**8.5 ± 0.8**	**8**
30	7.8	7.7	6.5	11.0	9.6	8.1	8.7	8.5	**8.5 ± 1.4**	**8**
60	8.4	-	8.8	8.8	8.7	7.9	12.8	9.1	**9.2 ± 1.6**	**7**
90	8.2	6.2	7.7	8.4	9.4	8.8	9.7	6.9	**8.2 ± 1.2**	**8**
120	8.5	7.6	8.1	10.7	7.6	7.8	12.2	9.7	**9.0 ± 1.7**	**8**
150	8.5	8.1	11.0	7.6	7.3	6.8	9.7	9.4	**8.6 ± 1.4**	**8**
180	9.1	8.0	9.1	10.2	8.6	8.3	9.7	7.5	**8.8 ± 0.9**	**8**
210	8.1	-	5.4	8.3	7.2	8.7	8.5	10.1	**8.0 ± 1.5**	**7**
240	11.8	8.2	7.3	7.3	6.2	7.9	8.5	8.4	**8.2 ± 1.7**	**8**

Minute volume (mL/kg/min)

Time from treatment (min)	Animal number								Mean ± S.D.	N
	2841	2842	2843	2844	2845	2846	2847	2848		
-30	921	840	759	816	923	789	636	569	**782 ± 126**	**8**
-25	1137	884	784	795	949	728	827	691	**849 ± 142**	**8**
-20	1197	1162	744	818	877	740	608	750	**862 ± 211**	**8**
-15	914	823	1030	809	878	931	622	784	**849 ± 121**	**8**
-10	972	964	607	904	1364	873	1091	1157	**991 ± 222**	**8**
0	1028	934	785	828	998	812	757	790	**867 ± 105**	**8**
30	867	882	574	1070	1290	965	857	764	**909 ± 211**	**8**
60	849	-	888	820	906	854	1082	910	**901 ± 86**	**7**
90	960	727	995	769	1031	858	708	791	**855 ± 126**	**8**
120	954	776	927	1324	898	856	847	815	**925 ± 171**	**8**
150	847	845	1010	841	614	769	959	747	**829 ± 124**	**8**
180	1026	835	1068	1140	960	802	744	766	**918 ± 151**	**8**
210	793	-	708	867	862	977	698	985	**841 ± 116**	**7**
240	1207	758	897	758	746	823	705	785	**835 ± 161**	**8**

Respiratory rate (breaths/min)

Time from treatment (min)	Animal number								Mean ± S.D.	N
	2841	2842	2843	2844	2845	2846	2847	2848		
-30	94	110	100	96	106	111	76	91	**98 ± 12**	**8**
-25	133	118	91	97	106	129	77	87	**105 ± 20**	**8**
-20	112	113	103	93	107	110	68	86	**99 ± 16**	**8**
-15	103	104	129	92	114	111	71	112	**105 ± 17**	**8**
-10	119	109	80	98	124	103	99	108	**105 ± 14**	**8**
0	112	110	101	95	111	113	78	97	**102 ± 12**	**8**
30	111	115	88	97	134	119	99	90	**107 ± 16**	**8**
60	101	-	101	93	104	109	85	101	**99 ± 8**	**7**
90	117	117	128	92	110	97	73	114	**106 ± 18**	**8**
120	112	102	114	124	118	110	70	84	**104 ± 18**	**8**
150	100	104	91	111	84	112	99	79	**98 ± 12**	**8**
180	113	104	118	112	112	97	77	102	**104 ± 13**	**8**
210	98	-	132	105	119	112	82	97	**106 ± 16**	**7**
240	102	92	122	104	121	104	83	93	**103 ± 14**	**8**

“-“ see text (point 6.4.1 Deviations from Protocol)

Appendix 1.2 – Fexinidazole, 100 mg/kg PO

Inspiration time (msec)

Time from treatment (min)	Animal number								Mean ± S.D.	N
	2841	2842	2843	2844	2845	2846	2847	2848		
-30	196	220	280	240	225	168	336	331	**249 ± 61**	**8**
-25	167	206	267	229	209	179	241	321	**227 ± 50**	**8**
-20	165	213	269	237	233	232	337	312	**250 ± 55**	**8**
-15	202	223	226	227	219	221	347	292	**244 ± 49**	**8**
-10	190	211	328	228	189	239	169	247	**225 ± 50**	**8**
0	184	214	274	232	215	208	286	301	**239 ± 42**	**8**
30	219	225	300	207	194	239	271	311	**246 ± 43**	**8**
60	235	-	235	234	248	222	290	297	**251 ± 30**	**7**
90	244	195	231	239	235	244	340	253	**248 ± 41**	**8**
120	219	221	219	201	234	234	304	253	**236 ± 31**	**8**
150	251	232	266	217	310	234	231	294	**255 ± 33**	**8**
180	235	223	228	212	204	257	309	265	**242 ± 34**	**8**
210	251	-	188	215	223	240	340	284	**249 ± 50**	**7**
240	211	230	222	221	232	223	295	299	**242 ± 35**	**8**

Expiration time (msec)

Time from treatment (min)	Animal number								Mean ± S.D.	N
	2841	2842	2843	2844	2845	2846	2847	2848		
-30	439	325	319	382	344	373	447	324	**369 ± 51**	**8**
-25	271	302	381	387	356	268	540	365	**359 ± 87**	**8**
-20	371	320	309	404	326	325	535	362	**369 ± 74**	**8**
-15	373	354	238	427	306	343	496	240	**347 ± 88**	**8**
-10	313	320	366	382	295	340	388	308	**339 ± 36**	**8**
0	353	324	323	396	325	330	481	320	**357 ± 56**	**8**
30	321	294	378	410	251	265	351	353	**328 ± 55**	**8**
60	360	-	355	407	325	313	416	302	**354 ± 45**	**7**
90	268	319	234	417	311	370	481	270	**334 ± 84**	**8**
120	317	360	307	281	277	306	553	434	**354 ± 95**	**8**
150	352	343	403	321	395	298	458	453	**378 ± 59**	**8**
180	295	351	280	323	330	360	465	322	**341 ± 57**	**8**
210	352	-	265	356	268	296	401	320	**322 ± 50**	**7**
240	386	419	265	350	251	354	419	342	**348 ± 63**	**8**

Relaxation time (msec)

Time from treatment (min)	Animal number								Mean ± S.D.	N
	2841	2842	2843	2844	2845	2846	2847	2848		
-30	259	173	184	240	191	249	256	208	**220 ± 35**	**8**
-25	166	174	249	239	209	157	335	219	**218 ± 58**	**8**
-20	231	156	179	231	192	179	304	171	**205 ± 48**	**8**
-15	236	188	135	226	172	205	281	145	**198 ± 49**	**8**
-10	208	166	185	208	184	199	278	162	**199 ± 36**	**8**
0	220	172	187	229	189	198	291	181	**208 ± 38**	**8**
30	184	175	196	255	135	153	175	217	**186 ± 37**	**8**
60	217	-	195	270	195	190	236	139	**206 ± 41**	**7**
90	147	138	137	275	170	244	279	158	**194 ± 62**	**8**
120	179	196	177	166	165	192	307	236	**202 ± 48**	**8**
150	176	182	214	185	204	178	198	272	**201 ± 31**	**8**
180	183	205	174	180	180	205	289	152	**196 ± 41**	**8**
210	194	-	132	208	159	187	204	167	**179 ± 27**	**7**
240	238	233	170	209	159	210	253	213	**211 ± 32**	**8**

“-“ see text (point 6.4.1 Deviations from Protocol)

Appendix 1.3 – Fexinidazole, 300 mg/kg PO

Tidal volume (mL/kg)

Time from treatment (min)	Animal number 2849	2850	2851	2852	2853	2854	2855	2856	Mean ± S.D.	N
-30	8.4	8.6	7.3	7.4	8.1	7.1	7.9	9.4	**8.0 ± 0.8**	**8**
-25	8.6	6.0	7.1	9.7	10.3	7.9	8.6	7.3	**8.2 ± 1.4**	**8**
-20	9.0	6.5	8.9	7.6	8.8	8.2	6.9	7.7	**7.9 ± 0.9**	**8**
-15	12.1	6.0	7.1	9.7	12.2	7.8	6.5	10.0	**8.9 ± 2.4**	**8**
-10	9.0	7.5	8.5	9.8	8.1	8.4	11.1	6.2	**8.6 ± 1.5**	**8**
0	9.4	6.9	7.8	8.8	9.5	7.9	8.2	8.1	**8.3 ± 0.9**	**8**
30	8.2	7.7	7.7	10.6	8.0	9.3	8.0	8.9	**8.6 ± 1.0**	**8**
60	9.0	8.4	7.7	9.5	7.9	7.0	6.5	7.2	**7.9 ± 1.0**	**8**
90	9.0	6.2	7.0	-	8.9	7.7	8.3	6.9	**7.7 ± 1.1**	**7**
120	6.6	9.2	7.1	7.2	8.3	6.0	7.3	7.3	**7.4 ± 1.0**	**8**
150	6.5	5.9	8.3	7.8	13.4	8.0	8.4	8.2	**8.3 ± 2.2**	**8**
180	8.9	13.1	10.5	13.4	8.3	8.3	8.2	7.7	**9.8 ± 2.3**	**8**
210	8.6	6.8	9.2	8.2	7.2	10.8	9.0	-	**8.5 ± 1.4**	**7**
240	8.7	9.3	9.5	7.0	8.2	8.6	8.4	9.2	**8.6 ± 0.8**	**8**

Minute volume (mL/kg/min)

Time from treatment (min)	Animal number 2849	2850	2851	2852	2853	2854	2855	2856	Mean ± S.D.	N
-30	816	818	833	764	605	869	738	975	**802 ± 107**	**8**
-25	671	628	673	1068	1318	905	774	796	**854 ± 236**	**8**
-20	801	662	974	907	783	743	732	842	**806 ± 100**	**8**
-15	1066	611	731	907	1105	796	705	1021	**868 ± 184**	**8**
-10	874	798	860	818	767	791	1230	797	**867 ± 151**	**8**
0	846	703	814	893	915	821	836	886	**839 ± 66**	**8**
30	777	749	848	1138	765	999	784	973	**879 ± 142**	**8**
60	922	920	778	1006	766	839	769	804	**851 ± 89**	**8**
90	829	693	767	-	885	855	946	773	**821 ± 84**	**7**
120	644	939	654	741	883	773	716	859	**776 ± 108**	**8**
150	707	685	880	857	1653	938	918	843	**935 ± 304**	**8**
180	861	1205	1192	1451	978	921	765	938	**1039 ± 225**	**8**
210	947	740	1056	802	958	1139	1027	-	**953 ± 141**	**7**
240	742	740	1117	859	836	1178	743	1077	**912 ± 183**	**8**

Respiratory rate (breaths/min)

Time from treatment (min)	Animal number 2849	2850	2851	2852	2853	2854	2855	2856	Mean ± S.D.	N
-30	97	95	113	103	75	123	94	103	**101 ± 14**	**8**
-25	78	105	95	110	128	114	91	108	**104 ± 15**	**8**
-20	89	102	110	120	89	90	106	110	**102 ± 11**	**8**
-15	88	102	103	93	91	102	109	102	**99 ± 7**	**8**
-10	97	106	101	84	94	95	111	128	**102 ± 13**	**8**
0	90	102	105	102	96	105	102	110	**101 ± 6**	**8**
30	94	97	110	107	95	108	97	110	**102 ± 7**	**8**
60	103	110	101	106	97	119	119	112	**108 ± 8**	**8**
90	93	112	110	-	99	112	114	112	**107 ± 8**	**7**
120	98	103	92	103	107	129	98	117	**106 ± 12**	**8**
150	109	116	106	111	124	117	109	103	**112 ± 7**	**8**
180	96	92	113	108	118	111	93	121	**107 ± 11**	**8**
210	110	109	115	98	133	105	114	-	**112 ± 11**	**7**
240	85	80	118	123	102	137	88	117	**106 ± 20**	**8**

“-“ see text (point 6.4.1 Deviations from Protocol)

Appendix 1.3 – Fexinidazole, 300 mg/kg PO

Penh (enhanced pause, ((ET/RT)-1)*(PEF/PIF))

Time from treatment (min)	Animal number 2849	2850	2851	2852	2853	2854	2855	2856	Mean ± S.D.	N
-30	-0.64	-0.66	-0.48	-0.52	-0.72	-0.51	-0.79	-1.28	**-0.70 ± 0.26**	**8**
-25	-0.91	-0.80	-0.52	-0.48	-0.46	-0.98	-0.42	-0.63	**-0.65 ± 0.22**	**8**
-20	-0.60	-0.66	-0.38	-0.80	-0.85	-0.60	-0.59	-0.69	**-0.64 ± 0.14**	**8**
-15	-0.65	-1.20	-0.67	-1.17	-0.62	-0.81	-0.65	-1.29	**-0.88 ± 0.29**	**8**
-10	-0.69	-0.72	-0.46	-0.45	-0.53	-1.02	-0.47	-0.91	**-0.66 ± 0.22**	**8**
0	-0.70	-0.81	-0.50	-0.68	-0.64	-0.78	-0.58	-0.96	**-0.71 ± 0.14**	**8**
30	-0.43	-1.08	-0.34	-0.48	-0.74	-0.81	-0.59	-1.05	**-0.69 ± 0.28**	**8**
60	-0.56	-1.05	-0.53	-0.89	-0.97	-0.72	-1.56	-1.10	**-0.92 ± 0.33**	**8**
90	-0.38	-0.67	-0.43	-	-0.74	-0.60	-0.82	-0.70	**-0.62 ± 0.16**	**7**
120	-0.49	-1.12	-0.58	-0.36	-0.49	-0.78	-0.83	-0.63	**-0.66 ± 0.24**	**8**
150	-0.48	-0.64	-0.38	-0.44	-0.78	-0.65	-0.48	-0.67	**-0.57 ± 0.14**	**8**
180	-0.58	-2.27	-0.22	-0.29	-0.50	-0.71	-0.66	-0.64	**-0.73 ± 0.64**	**8**
210	-0.42	-0.59	-0.54	-0.40	-1.22	-0.36	-1.21	-	**-0.68 ± 0.38**	**7**
240	-0.61	-0.83	-0.57	-0.40	-0.70	-0.51	-0.49	-0.78	**-0.61 ± 0.15**	**8**

Peak inspiration flow (mL/sec)

Time from treatment (min)	Animal number 2849	2850	2851	2852	2853	2854	2855	2856	Mean ± S.D.	N
-30	-14.0	-14.8	-13.7	-14.3	-15.8	-13.2	-14.1	-15.0	**-14.4 ± 0.8**	**8**
-25	-20.7	-11.3	-13.1	-25.6	-25.3	-13.8	-15.2	-13.3	**-17.3 ± 5.7**	**8**
-20	-14.3	-12.7	-24.5	-13.7	-17.2	-15.3	-12.5	-13.9	**-15.5 ± 3.9**	**8**
-15	-19.6	-11.5	-13.5	-17.0	-21.5	-13.6	-12.8	-15.5	**-15.6 ± 3.5**	**8**
-10	-17.3	-14.5	-15.4	-17.5	-18.8	-14.5	-24.0	-11.6	**-16.7 ± 3.7**	**8**
0	-17.2	-13.0	-16.0	-17.6	-19.7	-14.1	-15.7	-13.9	**-15.9 ± 2.2**	**8**
30	-14.9	-14.4	-14.7	-21.4	-15.9	-16.3	-15.6	-16.6	**-16.2 ± 2.2**	**8**
60	-16.8	-15.8	-13.8	-21.1	-13.1	-12.7	-14.2	-13.4	**-15.1 ± 2.8**	**8**
90	-16.4	-12.5	-12.4	-	-15.0	-13.8	-15.4	-12.7	**-14.0 ± 1.6**	**7**
120	-11.1	-15.2	-13.0	-19.3	-15.0	-11.7	-12.8	-14.5	**-14.1 ± 2.6**	**8**
150	-13.2	-12.1	-15.3	-18.1	-22.4	-13.1	-16.3	-14.5	**-15.6 ± 3.3**	**8**
180	-16.5	-30.3	-22.9	-28.4	-17.0	-14.0	-14.0	-15.4	**-19.8 ± 6.5**	**8**
210	-15.9	-13.1	-15.4	-16.3	-15.5	-19.7	-17.8	-	**-16.2 ± 2.0**	**7**
240	-14.9	-17.8	-19.1	-14.2	-15.5	-17.5	-14.2	-21.5	**-16.9 ± 2.6**	**8**

Peak expiration flow (mL/sec)

Time from treatment (min)	Animal number 2849	2850	2851	2852	2853	2854	2855	2856	Mean ± S.D.	N
-30	12.0	11.4	10.8	10.6	12.9	11.2	12.3	18.5	**12.5 ± 2.6**	**8**
-25	18.6	10.5	9.7	13.8	18.7	18.8	10.8	12.3	**14.1 ± 4.0**	**8**
-20	11.9	10.6	13.5	15.3	14.8	12.9	10.3	12.9	**12.8 ± 1.8**	**8**
-15	17.2	12.1	10.6	22.0	17.1	12.1	10.2	19.1	**15.0 ± 4.4**	**8**
-10	14.7	12.4	11.1	11.5	12.1	14.4	16.7	14.3	**13.4 ± 1.9**	**8**
0	14.9	11.4	11.1	14.6	15.1	13.9	12.0	15.4	**13.6 ± 1.8**	**8**
30	10.3	12.9	10.2	15.6	14.6	18.1	11.4	18.0	**13.9 ± 3.2**	**8**
60	12.6	15.4	10.4	17.6	15.1	12.9	15.9	15.2	**14.4 ± 2.3**	**8**
90	11.6	9.8	9.5	-	12.4	12.1	16.0	13.7	**12.2 ± 2.2**	**7**
120	9.5	19.8	9.4	10.2	12.2	12.4	11.9	13.0	**12.3 ± 3.3**	**8**
150	9.8	9.7	10.9	11.2	24.8	12.9	12.2	15.9	**13.4 ± 5.0**	**8**
180	12.6	25.5	13.8	15.9	13.3	13.9	11.7	13.9	**15.1 ± 4.4**	**8**
210	11.7	10.5	13.2	10.8	19.2	14.1	20.9	-	**14.4 ± 4.1**	**7**
240	12.2	17.5	13.2	11.3	14.0	14.6	11.2	18.6	**14.1 ± 2.7**	**8**

"-" see text (point 6.4.1 Deviations from Protocol)

Appendix 1.3 – Fexinidazole, 300 mg/kg PO

Inspiration time (msec)

Time from treatment (min)	Animal number								Mean ± S.D.	N
	2849	2850	2851	2852	2853	2854	2855	2856		
-30	252	238	230	219	254	222	248	271	**242 ± 18**	**8**
-25	189	226	239	170	170	228	256	229	**213 ± 33**	**8**
-20	260	223	169	225	238	224	241	237	**227 ± 27**	**8**
-15	241	229	242	232	235	230	224	254	**236 ± 10**	**8**
-10	229	217	235	252	220	253	200	217	**228 ± 18**	**8**
0	234	226	223	220	223	231	234	241	**229 ± 7**	**8**
30	251	240	227	219	224	237	247	224	**234 ± 12**	**8**
60	237	231	237	230	270	228	231	239	**238 ± 13**	**8**
90	244	212	240	-	263	223	223	237	**235 ± 17**	**7**
120	272	236	244	180	239	203	258	239	**234 ± 29**	**8**
150	229	211	235	200	227	241	224	244	**226 ± 15**	**8**
180	248	137	183	207	227	232	277	219	**216 ± 42**	**8**
210	239	213	237	232	213	213	208	-	**222 ± 13**	**7**
240	261	224	204	224	230	193	258	189	**223 ± 27**	**8**

Expiration time (msec)

Time from treatment (min)	Animal number								Mean ± S.D.	N
	2849	2850	2851	2852	2853	2854	2855	2856		
-30	364	390	293	359	521	266	384	304	**360 ± 79**	**8**
-25	310	341	387	392	302	294	396	322	**343 ± 43**	**8**
-20	405	362	387	274	426	448	322	306	**366 ± 61**	**8**
-15	438	352	334	414	387	356	323	330	**367 ± 42**	**8**
-10	386	345	354	460	414	379	343	249	**366 ± 62**	**8**
0	381	358	351	380	410	348	353	302	**360 ± 31**	**8**
30	380	350	316	340	404	320	358	300	**346 ± 35**	**8**
60	346	314	352	339	342	273	339	297	**325 ± 28**	**8**
90	402	318	299	-	366	314	307	297	**329 ± 39**	**7**
120	332	347	375	410	322	236	348	270	**330 ± 55**	**8**
150	320	304	299	342	256	270	324	337	**307 ± 31**	**8**
180	372	609	344	349	282	307	349	274	**361 ± 106**	**8**
210	302	333	282	379	232	356	318	-	**315 ± 49**	**7**
240	438	526	331	267	353	244	421	327	**363 ± 94**	**8**

Relaxation time (msec)

Time from treatment (min)	Animal number								Mean ± S.D.	N
	2849	2850	2851	2852	2853	2854	2855	2856		
-30	208	210	183	211	277	166	202	149	**201 ± 38**	**8**
-25	154	183	226	207	185	171	248	192	**196 ± 30**	**8**
-20	236	203	230	159	215	263	188	175	**208 ± 34**	**8**
-15	252	165	181	217	218	187	177	162	**195 ± 31**	**8**
-10	212	188	216	273	227	187	205	143	**206 ± 37**	**8**
0	212	190	207	213	224	195	204	164	**201 ± 18**	**8**
30	233	158	211	205	224	185	198	153	**196 ± 29**	**8**
60	199	151	206	164	186	160	142	151	**170 ± 24**	**8**
90	260	171	192	-	193	186	171	181	**193 ± 31**	**7**
120	211	186	209	243	200	136	184	159	**191 ± 33**	**8**
150	195	169	195	199	150	163	197	209	**185 ± 21**	**8**
180	212	165	252	229	172	179	195	161	**196 ± 33**	**8**
210	192	193	174	237	117	237	157	-	**187 ± 43**	**7**
240	251	285	181	178	199	151	259	172	**209 ± 49**	**8**

“-“ see text (point 6.4.1 Deviations from Protocol)

Appendix 1.4 – Fexinidazole, 1000 mg/kg PO

Tidal volume (mL/kg)

Time from treatment (min)	Animal number								Mean ± S.D.	N
	2857	2858	2859	2860	2861	2862	2863	2864		
-30	10.4	8.1	7.3	7.7	8.9	8.4	9.5	6.1	**8.3 ± 1.3**	**8**
-25	9.2	9.1	7.7	9.0	-	8.2	8.0	10.1	**8.8 ± 0.8**	**7**
-20	12.3	9.0	10.3	9.2	-	7.8	9.0	7.7	**9.3 ± 1.6**	**7**
-15	12.6	13.1	8.7	9.1	9.1	5.8	7.1	9.1	**9.3 ± 2.5**	**8**
-10	8.7	9.0	9.6	8.8	-	7.1	9.1	6.5	**8.4 ± 1.1**	**7**
0	10.6	9.6	8.7	8.8	9.0	7.5	8.5	7.9	**8.8 ± 1.0**	**8**
30	8.2	11.7	9.3	9.1	7.7	5.8	8.5	7.7	**8.5 ± 1.7**	**8**
60	9.9	7.7	8.2	8.9	9.3	8.2	8.4	6.7	**8.4 ± 1.0**	**8**
90	9.3	8.9	8.6	8.9	6.6	7.3	7.5	7.5	**8.1 ± 1.0**	**8**
120	10.4	-	10.1	7.4	9.0	6.8	10.1	10.1	**9.1 ± 1.5**	**7**
150	11.7	-	8.7	6.4	8.9	7.5	7.6	7.6	**8.3 ± 1.7**	**7**
180	8.6	-	7.8	6.5	8.0	6.4	8.2	8.4	**7.7 ± 0.9**	**7**
210	9.2	9.0	8.1	8.1	7.9	8.1	7.8	6.7	**8.1 ± 0.7**	**8**
240	9.7	9.3	8.6	9.8	9.6	6.9	8.4	8.3	**8.8 ± 1.0**	**8**

Minute volume (mL/kg/min)

Time from treatment (min)	Animal number								Mean ± S.D.	N
	2857	2858	2859	2860	2861	2862	2863	2864		
-30	871	1008	845	957	595	903	676	727	**823 ± 144**	**8**
-25	697	807	770	886	-	874	713	1043	**827 ± 120**	**7**
-20	1234	804	1115	868	-	878	951	614	**923 ± 204**	**7**
-15	1398	1319	882	928	1193	656	707	892	**997 ± 276**	**8**
-10	848	789	1111	865	-	721	935	630	**843 ± 155**	**7**
0	1009	945	944	901	894	806	796	781	**885 ± 83**	**8**
30	766	1328	1299	1110	852	680	871	746	**956 ± 255**	**8**
60	997	747	937	1095	1000	900	839	741	**907 ± 126**	**8**
90	1063	773	840	933	878	798	708	774	**846 ± 112**	**8**
120	1083	-	1075	668	1118	728	893	1103	**953 ± 190**	**7**
150	1257	-	1029	782	964	788	739	831	**913 ± 185**	**7**
180	890	-	935	783	860	689	783	841	**826 ± 82**	**7**
210	894	1058	923	826	909	902	750	799	**883 ± 94**	**8**
240	866	842	1298	1249	1172	773	837	747	**973 ± 227**	**8**

Respiratory rate (breaths/min)

Time from treatment (min)	Animal number								Mean ± S.D.	N
	2857	2858	2859	2860	2861	2862	2863	2864		
-30	83	124	115	124	67	108	71	119	**101 ± 24**	**8**
-25	76	89	100	99	-	106	89	104	**95 ± 11**	**7**
-20	100	89	108	95	-	113	105	80	**99 ± 12**	**7**
-15	111	101	101	102	131	113	99	99	**107 ± 11**	**8**
-10	97	88	116	98	-	102	103	96	**100 ± 8**	**7**
0	94	98	108	103	99	108	94	99	**100 ± 6**	**8**
30	93	113	140	122	110	118	102	96	**112 ± 15**	**8**
60	100	97	115	123	108	109	99	111	**108 ± 9**	**8**
90	114	87	98	104	132	110	94	103	**105 ± 14**	**8**
120	104	-	107	90	124	107	89	109	**104 ± 12**	**7**
150	108	-	118	122	109	105	97	110	**110 ± 8**	**7**
180	104	-	120	121	108	108	96	100	**108 ± 9**	**7**
210	97	118	114	102	114	111	96	119	**109 ± 9**	**8**
240	89	90	152	127	122	111	99	90	**110 ± 22**	**8**

"-" see text (point 6.4.1 Deviations from Protocol)

Appendix 1.4 – Fexinidazole, 1000 mg/kg PO

Penh (enhanced pause, ((ET/RT)-1)*(PEF/PIF))

Time from treatment (min)	Animal number								Mean ± S.D.	N
	2857	2858	2859	2860	2861	2862	2863	2864		
-30	-0.62	-1.40	-0.72	-0.93	-1.90	-0.44	-0.44	-0.64	**-0.89 ± 0.51**	**8**
-25	-0.66	-0.54	-0.83	-0.29	-	-0.55	-0.33	-0.65	**-0.55 ± 0.19**	**7**
-20	-0.72	-0.81	-0.53	-0.42	-	-1.00	-0.66	-0.55	**-0.67 ± 0.19**	**7**
-15	-0.54	-0.68	-1.19	-0.56	-0.51	-0.57	-0.46	-1.07	**-0.70 ± 0.27**	**8**
-10	-0.67	-0.62	-0.68	-0.43	-	-0.90	-0.97	-0.66	**-0.70 ± 0.18**	**7**
0	-0.64	-0.81	-0.79	-0.53	-1.20	-0.69	-0.57	-0.71	**-0.74 ± 0.21**	**8**
30	-0.68	-0.44	-0.73	-0.79	-0.68	-0.50	-0.53	-0.72	**-0.63 ± 0.12**	**8**
60	-1.20	-0.55	-0.56	-0.59	-0.44	-0.86	-0.62	-0.56	**-0.67 ± 0.24**	**8**
90	-0.67	-0.42	-0.79	-0.81	-0.68	-0.78	-0.90	-0.80	**-0.73 ± 0.15**	**8**
120	-0.36	-	-1.20	-0.61	-1.14	-0.53	-0.48	-0.60	**-0.70 ± 0.33**	**7**
150	-0.29	-	-0.44	-1.30	-0.68	-0.41	-0.55	-0.37	**-0.58 ± 0.34**	**7**
180	-0.61	-	-0.98	-0.62	-0.57	-0.52	-0.65	-0.40	**-0.62 ± 0.18**	**7**
210	-0.49	-0.79	-0.51	-0.54	-0.95	-0.68	-0.39	-0.54	**-0.61 ± 0.18**	**8**
240	-0.65	-0.56	-0.52	-0.28	-0.30	-0.46	-0.29	-0.53	**-0.45 ± 0.14**	**8**

Peak inspiration flow (mL/sec)

Time from treatment (min)	Animal number								Mean ± S.D.	N
	2857	2858	2859	2860	2861	2862	2863	2864		
-30	-16.1	-15.6	-13.5	-18.2	-28.8	-22.2	-17.6	-13.6	**-18.2 ± 5.1**	**8**
-25	-15.4	-16.3	-15.0	-16.8	-	-16.8	-17.7	-19.1	**-16.7 ± 1.4**	**7**
-20	-18.7	-14.0	-18.8	-15.5	-	-14.5	-18.2	-13.7	**-16.2 ± 2.3**	**7**
-15	-21.6	-23.1	-13.9	-15.4	-23.6	-12.0	-16.6	-16.2	**-17.8 ± 4.4**	**8**
-10	-13.7	-16.1	-17.5	-15.4	-	-14.1	-17.6	-12.4	**-15.3 ± 2.0**	**7**
0	-17.1	-17.0	-15.8	-16.3	-26.2	-15.9	-17.6	-15.0	**-17.6 ± 3.6**	**8**
30	-12.2	-29.6	-18.7	-17.9	-13.4	-11.9	-17.4	-15.2	**-17.0 ± 5.7**	**8**
60	-13.0	-12.5	-21.2	-18.5	-21.1	-15.3	-17.6	-12.3	**-16.4 ± 3.7**	**8**
90	-14.0	-14.1	-13.9	-15.9	-13.0	-15.4	-14.1	-13.6	**-14.3 ± 1.0**	**8**
120	-20.7	-	-16.1	-16.0	-15.7	-12.9	-26.3	-19.7	**-18.2 ± 4.4**	**7**
150	-22.7	-	-18.8	-12.5	-18.8	-15.7	-14.7	-14.7	**-16.9 ± 3.5**	**7**
180	-12.9	-	-16.2	-12.3	-14.0	-14.4	-17.4	-17.5	**-15.0 ± 2.1**	**7**
210	-12.9	-24.9	-16.6	-16.2	-14.3	-18.1	-18.0	-12.4	**-16.7 ± 3.9**	**8**
240	-14.3	-16.8	-21.2	-24.3	-31.8	-17.6	-19.9	-16.3	**-20.3 ± 5.6**	**8**

Peak expiration flow (mL/sec)

Time from treatment (min)	Animal number								Mean ± S.D.	N
	2857	2858	2859	2860	2861	2862	2863	2864		
-30	11.9	19.6	12.7	20.1	19.7	15.1	14.1	14.4	**16.0 ± 3.3**	**8**
-25	10.8	12.4	13.2	10.1	-	13.2	12.3	16.1	**12.6 ± 1.9**	**7**
-20	17.4	13.5	14.1	10.2	-	14.8	15.8	12.2	**14.0 ± 2.4**	**7**
-15	17.7	20.2	17.9	11.4	15.6	10.0	10.7	16.3	**15.0 ± 3.8**	**8**
-10	11.3	12.5	15.4	10.8	-	13.3	21.0	10.6	**13.6 ± 3.7**	**7**
0	13.8	15.6	14.7	12.5	17.6	13.3	14.8	13.9	**14.5 ± 1.6**	**8**
30	12.3	14.4	17.9	16.8	13.0	9.7	13.3	13.7	**13.9 ± 2.6**	**8**
60	15.6	10.0	13.9	16.0	14.3	15.8	15.5	10.1	**13.9 ± 2.5**	**8**
90	14.1	10.0	12.4	14.1	13.5	14.2	14.2	12.3	**13.1 ± 1.5**	**8**
120	13.5	-	22.3	10.7	21.0	10.7	16.2	16.4	**15.8 ± 4.6**	**7**
150	13.4	-	13.1	13.0	16.5	11.2	12.2	11.1	**12.9 ± 1.8**	**7**
180	10.9	-	18.8	11.2	13.2	10.8	15.7	13.4	**13.4 ± 3.0**	**7**
210	11.8	15.1	13.2	12.9	16.8	12.8	15.2	10.7	**13.6 ± 2.0**	**8**
240	14.5	13.8	17.4	13.4	17.6	12.2	14.5	11.3	**14.4 ± 2.2**	**8**

“-“ see text (point 6.4.1 Deviations from Protocol)

Appendix 1.4 – Fexinidazole, 1000 mg/kg PO

Inspiration time (msec)

Time from treatment (min)	Animal number								Mean ± S.D.	N
	2857	2858	2859	2860	2861	2862	2863	2864		
-30	261	209	225	195	124	186	270	241	**214 ± 47**	**8**
-25	284	235	230	214	-	223	230	251	**238 ± 23**	**7**
-20	259	250	220	241	-	241	239	299	**250 ± 25**	**7**
-15	206	227	270	241	155	223	231	257	**226 ± 35**	**8**
-10	255	239	220	237	-	254	242	255	**243 ± 13**	**7**
0	253	232	233	225	139	225	242	261	**226 ± 37**	**8**
30	291	152	187	202	251	227	250	237	**225 ± 43**	**8**
60	298	255	190	188	191	264	234	246	**233 ± 41**	**8**
90	243	255	257	250	225	224	292	257	**250 ± 22**	**8**
120	192	-	245	194	238	248	183	214	**216 ± 27**	**7**
150	185	-	185	219	202	227	261	218	**214 ± 27**	**7**
180	253	-	189	226	240	224	221	213	**224 ± 20**	**7**
210	286	139	194	214	229	217	211	225	**214 ± 41**	**8**
240	246	226	158	159	134	197	208	228	**194 ± 40**	**8**

Expiration time (msec)

Time from treatment (min)	Animal number								Mean ± S.D.	N
	2857	2858	2859	2860	2861	2862	2863	2864		
-30	457	248	294	277	614	369	567	262	**386 ± 144**	**8**
-25	484	438	369	395	-	342	447	324	**400 ± 59**	**7**
-20	339	410	334	389	-	297	329	412	**359 ± 45**	**7**
-15	335	326	321	348	300	308	371	339	**331 ± 23**	**8**
-10	356	439	297	373	-	330	334	349	**354 ± 44**	**7**
0	394	372	323	357	457	329	410	337	**372 ± 46**	**8**
30	331	383	241	275	290	279	333	382	**314 ± 52**	**8**
60	303	363	319	299	364	287	367	295	**325 ± 34**	**8**
90	283	429	352	323	228	330	337	326	**326 ± 57**	**8**
120	352	-	297	471	250	307	499	336	**359 ± 92**	**7**
150	347	-	323	312	349	345	350	333	**337 ± 15**	**7**
180	320	-	254	266	314	331	375	383	**320 ± 49**	**7**
210	329	455	331	375	249	343	412	280	**347 ± 67**	**8**
240	423	437	239	295	307	332	392	424	**356 ± 73**	**8**

Relaxation time (msec)

Time from treatment (min)	Animal number								Mean ± S.D.	N
	2857	2858	2859	2860	2861	2862	2863	2864		
-30	249	118	167	151	163	224	367	163	**200 ± 79**	**8**
-25	249	257	190	267	-	201	304	183	**236 ± 45**	**7**
-20	191	222	195	236	-	150	187	254	**205 ± 35**	**7**
-15	201	183	167	198	169	184	218	164	**186 ± 19**	**8**
-10	197	244	168	232	-	168	184	197	**198 ± 29**	**7**
0	217	205	177	217	166	186	252	192	**201 ± 27**	**8**
30	198	200	137	150	171	173	197	212	**180 ± 27**	**8**
60	151	215	173	178	220	156	216	175	**185 ± 28**	**8**
90	170	271	187	169	137	178	178	173	**183 ± 38**	**8**
120	226	-	159	247	135	187	280	196	**204 ± 50**	**7**
150	233	-	197	139	197	219	210	223	**203 ± 31**	**7**
180	187	-	137	158	195	195	217	252	**192 ± 37**	**7**
210	214	198	202	223	138	175	282	172	**200 ± 43**	**8**
240	257	260	146	196	199	200	279	241	**222 ± 44**	**8**

“-“ see text (point 6.4.1 Deviations from Protocol)

Appendix 2 Protocol and Amendment

Appendix 3 Pharmacy Certification

PHARMACY CERTIFICATION

Pharm. Cert. 0509-2007

TEST ARTICLE: **Fexinidazole**
STUDY NUMBER: **0509-2007**
NOTEBOOK NUMBERS: **G0095**

DOCUMENTATION ENCLOSED IN THE NOTEBOOK:

A. Analysis certificate dated December 18, 2007 issued by Orgasynth Industries for Fexinidazole test item, raw material, Batch No. 3168-07-01/O
B. Material safety data sheet issued by Orgasynth Industries for Fexinidazole
C. Certificate of Analysis issued by Sigma-Aldrich for Tween® 80, raw material, Lot No. 1239316
D. Certificate of Analysis issued by Sigma-Aldrich for Methylcellulose 400 cP, raw material, Lot No. 105K0074
E. Label's photocopy of Acqua per preparazioni iniettabili (Bieffe Medital S.p.A.), raw material, Lot No. 07G0201

ANALYTICAL DOCUMENTATION:

Requests and Analytical Results issued by Accelera/ADMET/Preclinical Formulation and Bioanalysis & Analytical Control

MATERIALS USED FOR THE STUDY:

1. Fexinidazole test item, raw material, Batch No. 3168-07-01/O
2. Tween® 80, raw material, Lot No. 1239316
3. Methylcellulose 400 cP, raw material, Lot No. 105K0074
4. Acqua per preparazioni iniettabili, raw material, Lot No. 07G0201

PREPARATIONS:

Prepare suspension of Fexinidazole test item, raw material, Lot No. 3168-07-01/O in the vehicle (5% Tween® 80 in 0.5% Methylcellulose 400 cP solution) at the concentration of 5 mg/mL, 15 mg/mL and 50 mg/mL

CONCENTRATION CHECKS: (ACCURANCY LIMITS: ± 10% OF LABELED AMOUNT)

Fexinidazole suspensions	Preparation date	Request No.	% of L.A.
5 mg/mL - TOP	2nd February 2008	Request No. 200800024	104.66
5 mg/mL - MIDDLE	2nd February 2008	Request No. 200800025	98.20
5 mg/mL - BOTTOM	2nd February 2008	Request No. 200800026	105.70
15 mg/mL - TOP	2nd February 2008	Request No. 200800021	101.80
15 mg/mL - MIDDLE	2nd February 2008	Request No. 200800022	101.09
15 mg/mL - BOTTOM	2nd February 2008	Request No. 200800023	100.60
50 mg/mL - TOP	2nd February 2008	Request No. 200800018	94.52
50 mg/mL - MIDDLE	2nd February 2008	Request No. 200800019	102.76
50 mg/mL - BOTTOM	2nd February 2008	Request No. 200800020	97.49

STABILITY:

Fexinidazole test item:

Expire date October 2008 for Fexinidazole, test item, raw material, Lot No. 3168-07-01/O if stored at room temperature protected from light

Fexinidazole suspensions:

Stability data indicate that Fexinidazole suspensions in the vehicle (5% Tween® 80 in 0.5% Methylcellulose 400 cP solution) in the range 0.5-100 mg/mL are stable up to 7 days at room temperature and 14 days at +4°C (Nerviano MS 0293-2007-R)

Prepared by: 11th March 2008
