# Supplementary material for: Fexinidazole – A New Oral Nitroimidazole Drug Candidate Entering Clinical Development for the Treatment of Sleeping Sickness
Source: PLoS Negl Trop Dis. 2010 Dec 21;4(12):e923. doi: 10.1371/journal.pntd.0000923 (PMC3006138; doi:10.1371/journal.pntd.0000923)
Supplement: Dataset S16 — (2.17 MB PDF) [file pntd.0000923.s017.pdf]

Fexinidazole: 28-Day Oral Toxicity Study in the Rat

Product Name :	Fexinidazole
Study Number:	0504-2007
Study Director/Author:	
Sponsor Reference Study No.:	Not applicable
Status:	Final

SUMMARY

Methods

Fexinidaxole, a 5-nitroimidazole derivative biologically active against Trypanosoma parasites (*T.b. rhodesiense* and *T.b. brucei*), was administered orally by gavage once daily for 28 consecutive days to ten or fifteen Crl:CD (SD)IGS BR rats/sex/group at doses of 50, 200 and 800 mg/kg/day in a volume of 10 mL/kg.

A control group received the vehicle alone (5% Tween 80 in 0.5% Methyl cellulose 400 cP (Methocel) suspension).

Ten animals/sex/group were sacrificed at the end of the treatment period on Day 29 or 30 of study. The remaining 5 animals/sex in the control and high-dose groups were sacrificed on Day 43 at the end of a two-week observation period.

Mortality, behavior and general condition were observed daily. Body weight was recorded pretest, and on Days 1, 8, 14, 21, 28, 35 and 42. Food consumption was recorded from Days 1 to 8, 8 to 14, 14 to 21, 21 to 28, 29 to 35 and 35 to 42. Ophthalmoscopy was performed pre-test, and on Days 28 and 42. Hematological and clinical chemistry examinations and urine analysis were performed on Days 29 and 43.

Post-mortem examinations performed at the end of the treatment period (Day 29 or 30) or the recovery period (Day 43) included necropsy, organ weights and histological examination.

Systemic exposure information was obtained from three additional animals/sex/group on Days 1 and 14 at different time-points up to 24 hours after treatment and on Day 28 at different time-points up to 72 hours after treatment.

Results

No compound-related mortality occurred in any group.

No meaningful clinical signs or changes in ophthalmoscopic examinations were seen.

A minimal to slight, dose-dependent reduction in mean body weight was seen in male animals at 200 and 800 mg/kg for the entire treatment period. When treatment was discontinued a tendency to recover was seen, but values did not reach those of the controls. No meaningful changes in body weight were seen in female animals during the treatment period. During the recovery period high-dose females showed a minimally reduced body weight gain in comparison with controls.

A dose-dependent, minimal to slight decrease in food consumption was seen in male animals at 200 and 800 mg/kg. An almost complete recovery occurred on Day 42 of study. No meaningful changes in food consumption were seen in female animals.

At hematology, clinical chemistry and urinalysis no clear treatment-related changes were recorded during the study.

At gross and histopathological examination, the most significant changes, which were considered a direct effect of the treatment indicative of adaptation, were observed in the liver at all doses with some dose-relationship. On gross examination the liver from females showed increases in mean absolute and relative weights, which were minimal at the doses of 50 and 200 mg/kg/day and more prominent and statistically significant in animals receiving 800 mg/kg/day. Minimal to slight hypertrophy (increase in cell size) of the centrilobular hepatocytes was observed histologically in almost all animals of both sexes, at all doses with scant dose-relationship, correlating in females with the liver weight increases. After recovery the weight increases regressed completely at the dose of 800 mg/kg/day and the hepatocellular hypertrophy showed partial significant regression in terms of both incidence and severity in both sexes.

After the first and repeated dosing Fexinidazole was extensively metabolized to the sulfone and sulfoxide derivatives.
After the first and repeated administrations of the three dose levels, Cmax and AUCs of Fexinidazole were two to three times higher in females than in males. No relevant gender difference was detected for metabolites on Day 1, except at the low dose of 50 mg/kg. On Days 14 and 28 no relevant gender difference was detected for the sulfone metabolite while Cmax and AUCs of the sulfoxide metabolite were two to three times higher in females than in males.
On Days 1, 14 and 28, in both genders, AUCs of Fexinidazole and its metabolites increased with the dose administered.
Day 1 and Day 28 mean ±SD systemic exposure to Fexinidazole is reported in the following table.

Doses of Fexinidazole (mg/kg/day)	Day 1 AUC0-t(last) (ng·h/mL)		Day 28 AUC0-t(last) (ng·h/mL)	
	M	F	M	F
50	178±99	759±153	889±287	1447±335 (1)
				1630±570
200	1120±708	3200±856	2326±1757 (1)	4437±514 (1)
			4410±3980	4750±508
800	10000±2160	15700±3610	3173±734 (1)	7640±1782 (1,2)
			11900±5360	7940±2210 (2)

(1) AUC within 24 hours post dosing
(2) n=2

Day 1 and Day 28 mean ±SD systemic exposure to the sulfoxide metabolite is reported in the following table.

Doses of Fexinidazole (mg/kg/day)	Day 1 AUC0-t(last) (ng·h/mL)		Day 28 AUC0-t(last) (ng·h/mL)	
	M	F	M	F
50	7660±2380	23200±5330	29467±8919 (1)	53833±15716 (1)
			30600±10900	78000±23600
200	80500±4310	91400±19700	122100±29340 (1)	138333±14189 (1)
			138000±45000	153000±36900
800	366000±113000	453000±84700	129733±56812 (1)	291500±40305 (1,2)
			268000±64100	331000±57300 (2)

(1) AUC within 24 hours post dosing
(2) n=2

Day 1 and Day 28 mean ±SD systemic exposure to the sulfone metabolite is reported in the following table.

Doses of Fexinidazole (mg/kg/day)	Day 1 AUC0-t(last) (ng·h/mL)		Day 28 AUC0-t(last) (ng·h/mL)	
	M	F	M	F
50	16500±9760	55800±28100	106267±9530 (1)	174667±40501 (1)
			113000±13700	197000±50600
200	119000±21900	134000±10800	341333±39068 (1)	341333±66124 (1)
			387000±34000	448000±135000
800	465000±264000	683000±241000	459000±60605 (1)	571500±54447 (1,2)
			509000±94900	738000±135000 (2)

(1) AUC within 24 hours post dosing
(2) n=2

Conclusions

Fexinidaxole, given orally once daily for 28 consecutive days at doses of 50, 200 or 800 mg/kg/day to Crl:CD (SD)IGS BR rats, was well tolerated at all doses tested, inducing only minimal to slight decreases in body weight and food consumption in male animals at 200 and 800 mg/kg. The minimal to moderate changes observed in the liver of all fexinidaxole-treated animals (increased liver weight and/or hypertrophy of the centrilobular hepatocytes) were restricted to dosing period and were considered of adaptative origin. Based on these results the dose of 200 mg/kg was considered the NOAEL. This dose corresponds on Day 28 to a mean AUC_{0-24} of fexinidazole of 3173 or 7640 ng•h/mL in males and females respectively, a mean AUC_{0-24} of sulfoxide metabolite of 129733 or 291500 ng•h/mL in males and females respectively, and a mean AUC_{0-24} of sulfone metabolite of 459000 or 571500 ng•h/mL in males and females respectively.

TABLE OF CONTENTS

1. INTRODUCTION AND OBJECTIVES 11
2. STUDY SPONSOR 11
3. TEST FACILITY 11
4. REGULATORY REQUIREMENTS 11
5. SCHEDULE 11
6. MATERIALS AND METHODS 12
6.1. Test and Control Items 12
6.1.1. Test Item 12
6.1.2. Vehicle/Control Item 12
6.1.3. Test Formulation 13
6.1.4. Test Formulation Analyses 13
6.2. Test System 13
6.2.1. Environmental Conditions 13
6.2.2. Allocation/Randomization 14
6.3. Experimental Design 14
6.3.1. Identification 15
6.3.2. Dose Administration 15
6.3.3. Dose Justification 15
6.4. Clinical and physical examinations 16
6.5. Clinical Pathology 16
6.5.1. Hematology 16
6.5.2. Clinical Chemistry 17
6.5.3. Urine Analysis 17
6.6. Postmortem Examinations 18
6.6.1. Unscheduled Deaths 18
6.6.2. Scheduled Necropsy, Tissue Collection, and Tissue Examination 18
6.6.3. Tissue Preparation 20
6.6.4. Pathology Peer Review 20

6.7. Systemic Exposure ... 20
7. DATA ACQUISITION ... 21
8. STATISTICAL ANALYSIS ... 21
8.1. Variables ... 21
8.2. Methods ... 21
9. ARCHIVING ... 22
10. STUDY DEVIATIONS ... 22
11. STUDY PERSONNEL ... 22
12. RESULTS ... 22
12.1. Clinical and Physical Examinations ... 22
12.1.1. Mortality ... 22
12.1.2. Clinical Signs ... 23
12.1.3. Opthalmoscopic examination ... 23
12.1.4. Body Weight ... 23
12.1.5. Food Consumption ... 23
12.2. Clinical Pathology ... 23
12.2.1. Hematology ... 23
12.2.2. Clinical Chemistry ... 24
12.2.3. Urinalysis ... 24
12.3. Postmortem Examinations ... 24
12.3.1. Organ Weights ... 24
12.3.2. Gross Pathology ... 25
12.3.3. Histopathology ... 25
12.4. Systemic Exposure ... 26
13. DISCUSSION ... 29
14. CONCLUSIONS ... 30
15. REFERENCES ... 31
FIGURES
Figure 1: Mean Body Weight
Figure 2: Mean Food Consumption

TABLES

Table 1 Clinical Signs
Table 2 Body Weights
Table 3 Food Consumption
Table 4 Ophthalmoscopic Findings
Table 5 Hematology
Table 6 Clinical Chemistry
Table 7 Urine Analysis - Quantitative
Table 8 Urine Analysis - Macroscopic
Table 9 Absolute Organ Weights
Table 10 Relative Organ Weights
Table 11 Gross Necropsy Observations
Table 12 Microscopic Observations

APPENDICES

Appendix 1 Clinical Signs
Appendix 2 Body Weights
Appendix 3 Food Consumption
Appendix 4 Ophthalmoscopic Findings
Appendix 5 Hematology
Appendix 6 Clinical Chemistry
Appendix 7 Urine Analysis
Appendix 8 Absolute Organ Weights
Appendix 9 Relative Organ Weights
Appendix 10 Gross Necropsy and Microscopic Observations
Appendix 11 Toxicokinetic Report
Appendix 12 Pharmacy Documentation
Appendix 13 Clinical Pathology Methods
Appendix 14 Protocol and Amendment

1. INTRODUCTION AND OBJECTIVES

The purpose of this study (0504-2007) was to determine the potential toxicity of and systemic exposure to Fexinidazole when administered orally, by gavage, once a day for 28 consecutive days to Crl:CD (SD)IGS BR rats. Reversal of possible toxic effects was determined for control and high dose animals over a 2-week observation period. Systemic exposure information was obtained from animals that are similarly dosed for 28 consecutive days and were designated for the systemic exposure phase of the study.

Fexinidazole is a 5-nitroimidazole derivate, biologically active against Trypanosoma parasites (*T.b.rhodesiense* and *T.b. brucei*) and useful in the treatment of the Human African Trypanosomiasis (HAT), known as sleeping sickness.

2. STUDY SPONSOR

DND*i* – Drugs for Neglected Diseases *Initiative*
1 Place St Gervais
CH – 1201 Geneva, Switzerland

3. TEST FACILITY

Accelera

4. REGULATORY REQUIREMENTS

This study was conducted in compliance with:

- Decreto Legislativo 2 Marzo 2007, No. 50;
- Organisation for Economic Co-operation and Development (OECD) Principles of Good Laboratory Practice (GLP) (as revised in 1997).

The methods employed in this study were those described in the "Standard Operating Procedures" of the laboratories involved.

5. SCHEDULE

	Males	Females
Experimental Start Date	1 February 2008 (Randomization date)	
Dose (Day 1)		
Main study	5 February 2008	7 February 2008
Toxicokinetics	5 February 2008	7 February 2008

Last Dose (Day 28 or Day 29)	3 or 4 March 2008	5 or 6 March 2008
Start of recovery (Day 29)	4 March 2008	6 March 2008
Necropsy Dosing Phase (Dosing Necropsy) (Day 29 or Day 30)	4 or 5 March 2008	6 or 7 March 2008
Necropsy Recovery Phase (Recovery Necropsy) (Day 43)	18 March 2008	20 March 2008
In vivo Experimental Completion Date	Last day of necropsy; 20 March 2008	

6. MATERIALS AND METHODS

6.1. Test and Control Items

6.1.1. Test Item

Identification	Fexinidazole
Lot/Batch Number	3168-07-01/O
Expiry	October 2008
HPCL Purity (%),	100.2%
Storage Conditions	Room Temperature, protected from light
Source and Manufacturer	Centipharm (formerly Orgasynth Industries)
Special Handling Precautions	According to MSDS (Material Safety Data Sheet)

6.1.2. Vehicle/Control Item

Identification	5% Tween 80 in 0.5% Methyl cellulose 400 cP (Methocel) suspension, in water for injection	
Lot/Batch Number	Tween 80	1324202
	Methyl cellulose 400 cP	-1st: 125K0196 in use up to Feb. 19, 2008<br>-2nd: 017K0087
	Water for injection	-1st: 07G0201 in use up to Feb. 19, 2008<br>-2nd: 07K1503
Storage Conditions	Room Temperature	
Expiry	Tween 80	February 2011
	Methyl cellulose 400 cP	-1st: January 2009<br>-2nd: January 2010
	Water for injection	-1st: July 2010<br>-2nd: November 2010
Source and Manufacturer	Tween 80	Sigma-Aldrich
	Methyl cellulose 400 cP	Sigma-Aldrich
	Water for injection	Bieffe Medical
Method of Preparation	On file at Accelera/Experimental ADMET/Preclinical Formulation	

6.1.3. Test Formulation

Type of Formulation	5% Tween 80 in 0.5% Methyl cellulose 400 cP (Methocel) suspension
Method of Preparation	On file at Accelera/Experimental AMDET/Preclinical Formulation
Frequency of Preparation	The solution was prepared according to the stability data
Dose Concentrations	5, 20 and 80 mg/mL
Storage Conditions	Room temperature
Source and Manufacturer	Accelera/ Experimental AMDET/Preclinical Formulation

6.1.4. Test Formulation Analyses

6.1.4.1. Homogeneity and Concentration

Samples (top-middle-bottom, 5 mL each) of each dose suspension prepared for the first and the last week of treatment (week 1 and 4) were collected under stirring for fexinidazole concentration and homogeneity checks; 10 mL were taken also from the vehicle. After collection, samples were directly transferred at room temperature to Accelera/DMPK & ART/Bioanalysis and Analytical Control for analysis. The analyses were performed using a validated HPLC-UV method (NervianoMS report 0293-2007-R).

Values were found to be within acceptable limits (± 10%) according to the internal SOPs of Accelera - Nerviano Medical Sciences.

6.1.4.2. Stability

Stability data indicated that Fexinidazole in 5% Tween 80 in 0.5% Methyl cellulose 400 cP (Methocel) suspension in the range 0.5-100 mg/mL is stable for 7 days at room temperature or for 14 days at +4°C (NervianoMS report 0293-2007-R).

6.2. Test System

Species/Strain or Breed, Source, Sex	Sprague Dawley rats supplied by Charles River Laboratories, Italia S.p.A., Via Indipendenza 11, Calco (Italy).
Justification of Species/Sex	The rat has been used extensively in safety studies and a large amount of biological data is available.
Age	Approximately 6 weeks at the start of treatment
Weight	Males: range from 169.7 to 203.5 grams at pre-test<br>Females: range from 160.9 to 189.2 grams at pre-test
Acclimation	At least 5 days
Selection Criteria	General condition and body weight

6.2.1. Environmental Conditions

Caging	Makrolon, (42x26x15 cm). Animals were caged two or three/cage
Room	108/B
Bedding	Saw dust 700/2000 supplied by L.G. Packing Wood, s.n.c., Condove (Italy)

Temperature	21.5° C +/- 1.5° C; monitored
Humidity	55% +/- 15; monitored
Air	Approximately twenty air changes per hour; monitored
Lighting	Approximate 12-hour light, 12-hour dark cycle. The lighting cycle could be interrupted for performance of protocol-defined activities.
Water	Municipal main, water available ad libitum
Diet	4RF21 GLP pellets ad libitum, supplied by Mucedola

Actual conditions were continuously monitored and recorded, and records were retained. Release of each lot of feed by the manufacturers was based on analysis of composite samples of each lot, which met specifications set by the manufacturers. Water was periodically analyzed for chemical and microbial impurities. No contaminants were identified in the food or water which were expected to interfere with the results or conclusions of this study.

All the above environmental conditions, as well as all the procedures adopted throughout the study for housing and handling the animals, were in strict compliance with EEC and Italian Guidelines for Laboratory Animal Welfare.

6.2.2. Allocation/Randomization

The animals were selected and distributed into experimental groups using random number tables (Fisher and Yates). The randomization was performed during the pretest period on February 1, 2008 for all animals.

One male animal (No. 2846) assigned to satellite study group for toxicokinetic purposes, found dead on Day 2 of study, was replaced with another one taken from the same arrival batch of animals, in order to maintain the number of three animals/sex/group for subsequent blood samplings. The animal maintained the number 2846.

6.3. Experimental Design

The study was performed according to the following experimental scheme:

Test Group	Dose (mg/kg/day)	Volume (mL/kg/day)	Number of Animals/Group					
			Toxicology				Toxicokinetic	
			Main Study		Recovery			
			Males	Females	Males	Females	Males	Females
1	0 (vehicle)[a]	10	10	10	5	5	-	-
2	50	10	10	10	-	-	-	-
3	200	10	10	10	-	-	-	-
4	800	10	10	10	5	5	-	-
5	0 (vehicle)[a]	10	-	-	-	-	3	3
6	50	10	-	-	-	-	3	3
7	200	10	-	-	-	-	3	3
8	800	10	-	-	-	-	3	3
a: 5% Tween 80 in 0.5% Methyl cellulose 400 cP (Methocel) suspension								

Animals were treated orally, by gavage, daily for 28 consecutive days. At the end of treatment period all animals from groups 2 and 3 and the first ten animals in numerical order (survival permitting) from groups 1 and 4 were sacrificed; the remaining animals were allowed a two-week recovery period.

6.3.1. Identification

The animals were identified by numbered metal tags on the right ear and different nicks on the left ear. A color-coded cage card was affixed to each study animal's cage and indicated the study number, cage number, treatment start date, test article, animal number, sex and dose level.

Test Group	Color Code	Animal Identification					
		Toxicology				Toxicokinetic[a]	
		Main Study		Recovery[a]			
		Males	Females	Males	Females	Males	Females
1	White	2735-2744	2785-2794	2745-2749	2795-2799	-	-
2	Yellow	2750-2759	2800-2809	-	-	-	-
3	Green	2760-2769	2810-2819	-	-	-	-
4	Red	2770-2779	2820-2829	2780-2784	2830-2834	-	-
5	White - dashed	-	-	-	-	2835-2837	2847-2849
6	Yellow - dashed	-	-	-	-	2838-2840	2850-2852
7	Green - dashed	-	-	-	-	2841-2843	2853-2855
8	Red - dashed	-	-	-	-	2844-2846	2856-2858
a Survival permitting							

6.3.2. Dose Administration

Method and Route	Os, gavage
Duration	28 days
Frequency	Once daily, at approximately the same time
Volume	10 mL/kg/day; Individual dose volumes were calculated based on the most recent body weight recorded
Rate	Not applicable

6.3.3. Dose Justification

Doses were selected taking into account the results of previous repeated oral toxicity studies performed in the same species.

In a 7-day repeated toxicity study (NervianoMS Study 0339-2007) Fexinidaxole given orally once daily at doses of 500, 1000 or 2000 mg/kg/day to Crl:CD (SD)IGS BR rats, was well

tolerated and did not cause meaningful toxicological changes at any dose tested, apart from a minimal to slight decrease in food consumption and minimal to slight changes in a few hematological parameters seen in both sexes at all doses tested.

In a 90-day repeated toxicity study (Hoechst Report 77.1238) Fexinidaxole given orally once daily at the doses of 50, 200 and 800 mg/kg/day induced toxicological effects, albeit reversible, at the two highest doses of 200 and 800 mg/kg/day. At 800 mg/kg/day protracted impairment of body weight was seen even if associated with an increase in food consumption. At 200 and 800 mg/kg/day decreased erythrocyte counts and increases in bilirubin levels were seen. In addition, at 800 mg/kg/day an increase in reticulocyte count was also detected.

Evaluation of the pharmacokinetics after a 3-day oral administration to male Sprague Dawley rats (NMS Study No. 0513-2007) demonstrated that exposure did not increased significantly from 500 to 1000 mg/kg/day.

The oral route and daily administration schedule are those envisaged for clinical setting.

6.4. Clinical and physical examinations

Survival and Moribundity Observations	At least once a day during the pre-test and test periods
Clinical Signs	Every day, at least one session/day
Unscheduled Observations	Upon evidence of changes in general appearance, overt signs of toxicity or evidence of moribundity
Body Weights	Once pretest and on Days 1, 8, 14, 21, 28, 35 and 42
Food Consumption	For Day-intervals 1 to 8, 8 to 14, 14 to 21, 21 to 28, 29 to 35 and 35 to 42.
Ophthalmoscopy	Pre-test, then on Day 28 (end of treatment period) for groups 1, 2, 3 and 4 and Day 42 (end of recovery period) for groups 1 and 4

6.5. Clinical Pathology

6.5.1. Hematology

Collection Intervals	Day 29 (end of treatment period) for groups 1, 2, 3 and 4 and Day 43 (end of recovery period) for groups 1 and 4
Number of Animals	The last five animals in numerical order, i.e., the third five of groups 1 and 4 and the second five of groups 2 and 3
Collection Site	Retroorbital sinus plexus
Anesthesia	Isofluorane
Fasting Requirements	Overnight (about 16 hours)
Unscheduled Samples	No further determinations and/or sampling times were added
Target Blood Volume	0.5 mL
Anticoagulant	8% EDTA solution

Hematology Parameters Evaluated

Red Blood Cells	Mean Corpuscular Volume reticulocytes
Hemoglobin	Mean Corpuscular Hemoglobin Concentration reticulocytes
Hematocrit	Mean Corpuscular Hemoglobin reticulocytes
Mean Corpuscular Volume	Platelets
Mean Corpuscular Hemoglobin	Mean Platelet Volume
Mean Corpuscular Hemoglobin Concentration	Platelet Distribution Width
Red Cell Distribution Width	Platelet Hematocrit
Hemoglobin Distribution Width	White Blood Cells
Reticulocyte Count (absolute and percent)	Differential White Blood Cells (absolute and percent)

6.5.2. Clinical Chemistry

Collection Intervals	Day 29 (end of treatment period) for groups 1, 2, 3 and 4 and Day 43 (end of recovery period) for groups 1 and 4
Number of Animals	The last five animals in numerical order, i.e., the third five of groups 1 and 4 and the second five of groups 2 and 3
Collection Site	Retroorbital sinus plexus
Anesthesia	Isofluorane
Fasting Requirements	Overnight (about 16 hours)
Unscheduled Samples	No further determinations and/or sampling times were added
Target Blood Volume	1 mL in tube for serum separation
Anticoagulant	None

Clinical Chemistry Parameters Evaluated

Urea	Albumin/Globulin Ratio (calculated)
Creatinine	Glucose
Alanine Aminotransferase	Triglycerides
Aspartate Aminotransferase	Total Cholesterol
Alkaline Phosphatase	Calcium
Gamma-glutamyltransferase	Phosphorus
Total Bilirubin	Sodium
Total Proteins	Potassium
Albumin	Chloride
Globulin	

6.5.3. Urine Analysis

Collection Intervals	Day 29 (end of treatment period) for groups 1, 2, 3 and 4 and Day 43 (end of recovery period) for groups 1 and 4
Number of Animals	The last five animals in numerical order, i.e., the third five of groups 1 and 4 and the second five of groups 2 and 3
Method of Collection	Metabolism cages. Before urine collection about 10 mL/kg of water were administered by gavage to each animal.
Sample Requirements	1% Thimerosal (0,2 ml) in each bottle, before collection
Fasting Requirements	Overnight (about 16 hours)
Unscheduled Samplings	No further determinations and/or sampling times were added

Urine Parameters Evaluated

**Urinalysis Parameters**	Macroscopic appearance (description)
pH	Ketones
White Blood Cells	Urobilinogen
Nitrites	Bilirubin
Proteins	Hemoglobin/Red Blood Cells
Glucose	Specific gravity

6.6. Postmortem Examinations

6.6.1. Unscheduled Deaths

Female No 2824 treated with 800 mg/kg/day, found dead on Day 14 of study, was necropsied at the earliest possible time. Tissues listed in Section 6.6.2 were collected from this animal and examined microscopically.

6.6.2. Scheduled Necropsy, Tissue Collection, and Tissue Examination

Sacrifice Schedule	Day 29 or 30 (end of treatment period) and Day 43 (end of recovery period). Animals with scheduled sacrifice on Day 30 were treated for an additional day to allow histopathological investigations about 24 hours after the last treatment.
Number of Animals (survival permitting)	Day 29: the second 5 animals/sex group in numerical order<br>Day 30: the first 5 animals/sex group in numerical order<br>Day 43: the last five animals/sex for groups 1 and 4.
Method of Euthanasia	Sodium thiopental i.p. and abdominal exsanguination
Fasting Requirements	Overnight (about 16 hours)
Terminal Body Weight	All animals
Macroscopic Examination	All animals

Organs/tissues from animals surviving at the end of the treatment period or recovery period were collected and examined microscopically (E) according to the following table. Paired organs were weighed together. Relative organ weights were calculated using the terminal fasted body weight of each animal.

Organ/Tissue	Main study and Recovery animals		Examined (mg/kg/day) Main study animals			
	Collected	Weighed	0	50	200	800
Adrenal glands (both)	X	X	E			E
Aorta	X		E			E
Bone marrow smear[a]	X		E			E
Bone, sternum (with marrow)	X		E			E
Brain[c]	X	X	E			E
Cecum	X		E			E
Colon	X		E			E
Duodenum	X		E			E
Diaphragm	X		E			E

Organ/Tissue	Main study and Recovery animals		Examined (mg/kg/day) Main study animals			
	Collected	Weighed	0	50	200	800
Epididymides (both)	X		E			E
Esophagus	X		E			E
Eyes, optic nerve (both)	X		E			E
Femur	X		E			E
Harderian gland	X		E	E	E	E
Heart	X	X	E			E
Ileum	X		E			E
Jejunum	X		E			E
Kidneys (both)	X	X	E			E
Liver[c]	X	X	E	E	E	E
Lungs	X		E			E
Lymph node, mandibular	X		E			E
Lymph node, mesenteric	X		E			E
Mammary gland	X		E			E
Ovaries	X	X	E			E
Pancreas	X		E			E
Pituitary	X		E			E
Prostate	X	X	E			E
Salivary glands (mandibular, parotids)	X		E			E
Sciatic nerve	X		E			E
Seminal vesicles	X		E			E
Skeletal muscle	X		E			E
Skin	X		E			E
Spinal cord (cervical, thoracic)	X		E			E
Spleen	X	X	E			E
Stifle joint	X		E			E
Stomach	X		E			E
Testes (both)	X	X	E			E
Thymus	X	X	E			E
Thyroid glands (with parathyroid glands)[b]	X		E			E
Tongue	X		E			E
Trachea	X		E			E
Urinary bladder	X		E			E
Uterus	X		E			E
Vagina	X		E			E
Lesions	X		E	E	E	E

Organ/Tissue	Main study and Recovery animals		Examined (mg/kg/day) Main study animals			
	Collected	Weighed	0	50	200	800
Histological examination was performed on all tissues/organs from the high-dose female found dead and from rats of the control and high dose group killed at the end of the treatment period. Examination of the liver and Harderian glands was extended to the other dose-groups for terminally killed animals and to all groups for animals killed after recovery.<br><br>a Except from animals found dead<br>b Parathyroid glands examined microscopically if included in the section of thyroid glands<br>c Samples from brain and liver were frozen in liquid nitrogen and kept at –80°C for further possible analysis<br><br>Fixatives:<br>Bone Marrow Smears: Methanol-ether<br>All Other Tissues: 10% neutral buffered formalin						

Toxicokinetic study animals found dead were subjected to necropsy for the purpose of determining a possible cause of death; however, tissues were not collected from these animals.

6.6.3. Tissue Preparation

Histological sections of all tissues listed in 6.6.2 were trimmed, embedded, sectioned, and stained with hematoxylin and erythrosine (further stains if needed). Bone marrow smears were prepared and stained with May Grünwald-Giemsa.

All organs/tissues were kept for any further test necessary.

6.6.4. Pathology Peer Review

A pathology peer review was conducted by Paola Della Torre. The signed Peer Review Report was archived with study raw data.

6.7. Systemic Exposure

The toxicokinetics of Fexinidazole and its metabolites M1 (sulfoxide) and M2 (sulfone) were evaluated in accordance with the collection schedule and procedures tabulated below. The bioanalysis was conducted by Accelera/DMPK & ART/Bioanalysis and Analytical Control.

Dose Levels (Groups)	Groups 5, 6, 7 and 8
Collection Intervals	Days 1, 14 and 28
Collection Time Points	Days 1 and 14: Pre-dose, 30 minutes, 1, 2, 4, 8 and 24h after dosing<br><br>Day 28: Pre-dose, 1, 2, 4, 8, 24, 48 and 72h after dosing

	For controls, samples were taken pre-dose and at a representative time-point (2 hours).
Animals/Time Point	All
Anesthesia	Isofluorane
Collection Volume per Sample	About 0.25 mL of blood/point.
Collection Site	Retroorbital sinus plexus
Sample Requirements	Blood was put in heparinized plastic tubes kept on an ice-water bath, then centrifuged (10 min, 1200g, +4°C). Two aliquots of about 50 μL of plasma were stored in a freezer at –80°C until analysis.
Disposition of Animals	Euthanized after final blood collection, without necropsy

After blood collection, frozen plasma specimens were transferred in plastic boxes to Accelera/DMPK & ART/Bioanalysis and Analytical Control, Nerviano Medical Sciences. The samples were loaded according to Watson 6.4.0.04 (Thermo Fisher Scientific Waltham, MA, USA).

Plasma samples were analyzed for the quantitation of Fexinidazole and its two metabolites using a validated LC-MS-MS method by Accelera/DMPK & ART/Bioanalysis and Analytical Control group.

Toxicokinetic calculations were carried out by Accelera/Pharmacokinetics & Modeling/Pharmacokinetics group. Details of the analytical method and of calculation methods were included in the toxicokinetic final report (Appendix 11).

7. DATA ACQUISITION

Clinical observations, body weights, organ weights, gross necropsy observations, histopathologic findings, and dose administration documentation were directly entered into the Xybion Path/Tox System or were recorded on appropriate paper forms, and, if appropriate, later entered into the Xybion Path/Tox System. Clinical pathology data were processed according to Clinical Pathology Laboratories procedures.

8. STATISTICAL ANALYSIS

8.1. Variables

The following parameters were evaluated: body weights, clinical chemistry, hematology, urinalysis, organ weights, organ/terminal body weight ratios.

8.2. Methods

Dunnett’s test, included in a customized Xybion package, was performed.

9. ARCHIVING

The original protocol, the protocol amendment, all raw data, supporting documents, and specimens produced at the Test Facility, and the final report with original signatures were filed in the Archives of Accelera, Nerviano Medical Sciences S.r.l., Nerviano (Italy) for the period of time agreed with the Sponsor (at least 3 years) after which the Sponsor will be contacted for instructions regarding dispatch or disposal of the material.

A copy of the protocol, the report with original signatures, a reserve sample and all relevant original documentation of the test item were filed by the Sponsor.

10. STUDY DEVIATIONS

The following deviations from protocol were recorded:

Paragraph 5.3 Test System
Animal body weight range at pre-test was 169.7-203.5 grams for males and 160.9-189.2 grams for females instead of 180-200 grams at the beginning of the study as stated in the protocol.

Paragraph 6.6.2 Scheduled Necropsy
On Day 29 of study the second five animals/sex/group in numerical order were sacrificed instead of the first five as stated in the protocol. On Day 30 the first 5 animals/sex/group in numerical order were sacrificed instead of the second 5 as stated in the protocol.

11. STUDY PERSONNEL

12. RESULTS

12.1. Clinical and Physical Examinations

12.1.1. Mortality

No treatment-related deaths occurred during the study.

One accidental death occurred in one female (No. 2824) treated with Fexinidaxole 800 mg/kg on Day 14 of study as a consequence of a faulty gavage during the last treatment performed on Day 13 (see also gross pathology and histopathology sections).

12.1.2. Clinical Signs

No treatment-related symptoms were observed in male or female animals at any dose tested.

12.1.3. Opthalmoscopic examination

Ophthalmoscopic examinations performed at the end of the treatment and recovery periods did not reveal toxicological findings.

12.1.4. Body Weight

A dose-dependent reduction in mean body weight gain related to the first day of treatment was seen in male animals at 200 and 800 mg/kg for the entire treatment period. The reduction on Day 28 of study was considered minimal at 200 mg/kg (-11.3%) and slight at 800 mg/kg (-22.1%). Mean body weight showed the same trend, reaching statistical significance at the high dose from Day 8 up to the end of treatment. During the recovery period (control and high dose group) a tendency to recover was seen in high-dose males, but values did not reach those of the controls. On Day 42 mean body weight gain of high dose males was slightly reduced in comparison with controls (-17%).

No meaningful toxicological changes in body weight were seen in female animals during the treatment period. During the recovery period (control and high dose group) high dose females showed a minimally reduced body weight gain in comparison with controls (-8% on Day 42).

12.1.5. Food Consumption

A dose-dependent minimal to slight decrease in food consumption was seen in male animals of all Fexinidazole-treated groups. At 50 mg/kg/day the decrease was statistically significant only on Day 28 of study while statistical significance was attained from Day 8 to Day 28 at 200 and 800 mg/kg. An almost complete recovery occurred on Day 42 of study.

No meaningful changes in food consumption were seen in female animals.

12.2. Clinical Pathology

12.2.1. Hematology

A few alterations were observed in some hematological parameters throughout the study, but no clear treatment-related changes were recorded.

Minimal (8%) decreases in hemoglobin and moderate (about 46%) decreases in neutrophils were recorded in high-dose females on Day 29. Recovery occurred on Day 43. At this last sampling time a minimal (1.2- to 1.5-fold) increase in lymphocytes and monocytes was observed in females.

12.2.2. Clinical Chemistry

A few alterations in clinical chemistry values were observed, but these were considered secondary and not directly related to the compound.

A minimal to slight decrease in alkaline phosphatase was detected in all treated groups, with dose-relationship on Day 29 (-23 to -36% of control values in males, -11 to –34% of control values in females). In addition, minimal (1.1 to 1.5) increases in globulins (+13%) and total cholesterol (+51%) were recorded in high-dose females, along with a moderate decrease (-50%) in triglycerides in high-dosed males. At the same sampling time, phosphorus was slightly to moderately (1.3- to 1.7-fold) increased in high-dose males Nos. 2781, 2782, 2783.

On Day 43 a complete recovery occurred in all values apart from alkaline phosphatase, which recovered only partially in high-dose males.

12.2.3. Urinalysis

No clear treatment-related changes were recorded throughout the study.

A slight increase in white blood cells was recorded in high-dose males on Day 29. Recovery occurred on Day 43.

12.3. Postmortem Examinations

12.3.1. Organ Weights

End of treatment (Days 29-30)

The liver of females showed treatment-related increases in mean absolute and relative weights which were minimal at the doses of 50 and 200 mg/kg/day and more prominent and statistically significant in animals receiving 800 mg/kg/day. The percentage variations in comparison with controls are set out in the following table**.**

Doses (mg/kg/day)	LIVER			
	Males		Females	
	Abs.	Rel.	Abs.	Rel.
50	+ 5	+ 10	+ 10	+ 8
200	+ 3	+ 13	+ 13	+10
800	+ 6	+ 21	+ 36**	+38

(**) = Statistical significance at P = 0.01

<u>End of recovery (Day 43)</u>

The increases in liver weights regressed completely in females receiving 800 mg/kg/day.

Other variations in mean weights sometimes noted in treated animals killed at termination or after recovery were considered to reflect either the individual variability observed also in controls or, only in males, some body weight decreases.

12.3.2. Gross Pathology

<u>Unscheduled death</u>

No treatment-related changes were seen in the high-dose female No 2824 found dead on Day 14 as a consequence of a faulty gavage during the last treatment performed on Day 13 (see also histopathology sections).

The main findings noted in this animal were represented by fairly good general condition, instead of good as in controls, and by the presence of abnormal content in the pleural cavity, consisting of clear liquid and soft yellow material (probably residual compound) adherent to the diaphragm and lungs.

<u>End of treatment (Day 29 or 30) and of recovery (Day 43)</u>

No treatment-related changes were noted in male and female animals killed either at termination at all doses or after recovery at the dose of 800 mg/kg/day.

Some findings noted in single instances, mainly in the group killed at termination after receiving 200 mg/kg/day, were considered unrelated to the treatment as they are known to occur, also on the basis of our experience, in untreated rats of the same strain and similar age. These changes, which are recorded in the “individual animal report”, were mainly represented by encrusted or alopecic areas in the skin of both sexes and by clear content in the pleural cavity from a single female.

12.3.3. Histopathology

<u>Unscheduled death</u>

No treatment-related changes were noted in the high-dose female No 2824 that died on Day 14 as a consequence of a faulty gavage performed on Day 13. The main findings in this animal, graded as slight to marked, were characterized by: acute pleuritis, with abundant exudate in the pleural cavity, involving the lungs, the diaphragm, the thymus, the pericardium and the tissues adjacent to the aorta; lymphoid depletion of the thymus, spleen, mesenteric and mandibular lymph nodes; porphyrin deposits in the Harderian glands (graded

as marked); acinar hypertrophy of both salivary glands. The pleuritis, which was considered indicative of faulty gavage, correlated with the clear liquid noted in the pleural cavity on gross examination.

The other changes were considered mainly due to stress.

End of treatment (Days 29 and 30)

Changes considered as a direct effect of the treatment occurred in the liver as follows.

Liver. Minimal to slight hypertrophy of the centrilobular hepatocytes in almost all animals of both sexes at all doses, with scant dose-relationship, correlating for females with some weight increases noted on gross examination.

End of recovery (Day 43)

The liver changes showed some regression in high-dose animals as follows.

Liver. Minimal hypertrophy of centrilobular hepatocytes in two out of five males and in one female.

The slightly more prominent porphyrin deposits noted in the Harderian gland from treated animals when compared with controls, either at termination at all doses without dose-relationship or after recovery, were considered minor indirect effects of the treatment probably mediated by stress. As well as by the lack of dose-relationship, this hypothesis is supported by the presence of more prominent porphyrin deposits than those noted in animals killed at termination, in the high-dose female that died accidentally as a result of faulty gavage on Day 14.

The unilateral changes (hemorrhage/adenitis) noted in the Harderian glands from treated animals receiving 50 and 200 mg/kg/day killed at termination and from control and high dose animals killed after recovery were considered related to the blood sampling from the retroorbital sinus. All other changes noted in treated animals killed at termination were considered related to spontaneous pathology because they occurred with similar incidence and severity as in controls or because they are known to occur in our experience in untreated animals of the same stain and similar age.

12.4. Systemic Exposure

Day 1

Mean ±SD systemic exposure to Fexinidazole is reported in the following table

Dose	Male			Female		
mg/kg	Cmax	tmax	AUC0-t(last)	Cmax	tmax	AUC0-t(last)
	ng/mL	hour	ng·hour/mL	ng/mL	hour	ng·hour/mL

50	39±17	1±0	178±99	149±46	2±2	759±153
200	165±97	2±1	1120±708	604±211	2±2	3200±856
800	1478±1111	2±1	10000±2160	1483±200	2±1	15700±3610

At each dose, C_{max} and $AUC_{0\text{-}t(last)}$ values were about three times higher in female than in male rats. In both genders, the maximal plasma concentrations of Fexinidazole were promptly achieved, on average within 2 hours post dosing. In both genders, $AUC_{0\text{-}t(last)}$ values of Fexinidazole increased with the dose administered.

Mean ±SD systemic exposure to the sulfoxide metabolite is reported in the following table

Dose	Male			Female		
mg/kg	Cmax	tmax	AUC0-t(last)	Cmax	tmax	AUC0-t(last)
	ng/mL	hour	ng·hour/mL	ng/mL	hour	ng·hour/mL
50	1503±413	2±1	7660±2380	3893 ±663	3±1	23200±5330
200	9987±2131	2±0	80500±4310	12800±1127	3±1	91400±19700
800	32833±4952	5±2	366000±113000	30633±2616	4±0	453000±84700

Mean ±SD systemic exposure to the sulfone metabolite is reported in the following table

Dose	Male			Female		
mg/kg	Cmax	tmax	AUC0-t(last)	Cmax	tmax	AUC0-t(last)
	ng/mL	hour	ng·hour/mL	ng/mL	hour	ng·hour/mL
50	1417±393	7±2	16500±9760	4417±2410	8±0	55800±28100
200	8550±2309	8±0	119000±21900	10023±815	8±0	134000±10800
800	40300±1562	8±0	465000±264000	44833±20128	13±9	683000±241000

Apart from 50 mg/kg dose, no relevant gender difference in C_{max} and $AUC_{0\text{-}t(last)}$ values of metabolites was observed. In both genders, t_{max} values of the sulfoxide metabolite were similar to the corresponding ones of the parent compound while t_{max} values of the sulfone metabolite were achieved at later times.
In both genders, the systemic exposure to the metabolites increased with the dose administered. The systemic exposure to the metabolites was much higher than that to the parent compound.

In male rats, the sulfoxide metabolite to parent $AUC_{0\text{-}t(last)}$ ratios were, on average, 46, 107 and 37 after 50, 200 and 800 mg/kg, respectively; the corresponding values in females were 29, 30 and 28.

In male rats, the sulfone metabolite to parent $AUC_{0\text{-}t(last)}$ ratios were, on average, 81, 172 and 46 after 50, 200 and 800 mg/kg, respectively; the corresponding values in females were 64, 40 and 39.

<u>Repeated dosing</u>
Day 14 and Day 28 mean ±SD systemic exposure to Fexinidazole is reported in the following table

Dose	Male			Female		
mg/kg/day	Cmax	tmax	AUC0-t(last)	Cmax	tmax	AUC0-t(last)
	ng/mL	hour	ng·hour/mL	ng/mL	hour	ng·hour/mL
	Day 14					
50	62±19	2±2	345±125	300±91	2±1	1310±161
200	274±163	3±1	2010±1520	773±107	2±1	4030±215

800	565±106	3±1	5120±1540	1478±1115	3±4	13500±3620
	Day 28					
50	133±24	4±0	889±287	422±358	1±0	1447±335 (1)
						1630±570
200	255±136	2±2	2326±1757 (1)	903±87	2±1	4437±514 (1)
			4410±3980			4750±508
800	523±127	2±2	3173±734 (1)	749±90 (2)	2±1 (2)	7640±1782 (1,2)
			11900±5360			7940±2210 (2)
(1) AUC within 24 hours post dosing<br>(2) n=2						

At each dose, C_{max} and $AUC_{0-t(last)}$ values were two to three times higher in female than in male rats. No relevant difference in the Fexinidazole levels was observed on Day 28 compared to Day 14. On Days 14 and 28, in both genders, the maximal plasma concentrations of Fexinidazole were achieved, on average, within 4 hours post dosing. On Day 28, in males, mean ±SD apparent terminal half-lives were 4.7 ±1.2, 8.8 ±4.8 and 11 ±2.7 hours after 50, 200 and 800 mg/kg/day, respectively. The corresponding female half-lives were 5.1 ±5.1, 6.3 ±4.5 and 6.7 ±2.3 (n=2) hours. In both genders, $AUC_{0-t(last)}$ values of Fexinidazole increased with the dose.
Day 14 and Day 28 male $AUC_{0-t(last)}$ (AUC within 24 hours post dosing on Day 28) accumulation ratios were, on average, 2.2 and 5.7 after 50 mg/kg/day, 1.6 and 2 after 200 mg/kg/day and 0.5 and 0.3 after 800 mg/kg/day, respectively. The corresponding values in females were 1.7 and 2, 1.3 and 1.5, 0.9 and 0.4.

Day 14 and Day 28 mean ±SD systemic exposure to the sulfoxide metabolite is reported in the following table

Dose	Male			Female		
mg/kg/day	Cmax	tmax	AUC0-t(last)	Cmax	tmax	AUC0-t(last)
	ng/mL	hour	ng·hour/mL	ng/mL	hour	ng·hour/mL
	Day 14					
50	2267±505	2±2	12500±4050	8197±519	2±0	47100±4920
200	13100±1044	3±1	93600±27300	18567±1266	3±1	143000±34900
800	17733±1701	3±1	165000±29100	32233±6435	2±0	390000±159000
	Day 28					
50	4530±664	3±1	29467±8919 (1)	11413±6603	1±1	53833±15716 (1)
			30600±10900			78000±23600
200	11633±2403	3±1	122100±29340 (1)	22333±1474	2±1	138333±14189 (1)
			138000±45000			153000±36900
800	16567±2079	2±1	129733±56812 (1)	25850±2051(2)	3±1(2)	291500±40305(1,2)
			268000±64100			331000±57300(2)
(1) AUC within 24 hours post dosing - (2) n=2						

At each dose, the levels of the metabolite were about two to three times higher in the female than in the male rat. No relevant difference in the levels of the metabolite were observed on Day 28 compared to Day 14. In both genders, the maximal plasma concentrations of the sulfoxide metabolite were achieved, on average, 2 - 3 hours post dosing. On Day 28, mean ±SD male apparent terminal half-lives were 5 ±2, 6.3 ±2.9 and 6.6 ±4.1 hours after 50, 200 and 800 mg/kg/day, respectively, whilst the corresponding female half-lives were 6.1 ±3.8,

8.1 ±3.1 and 8.6 ±2.3 (n=2) hours. In both genders, the systemic exposure to the metabolite increased with the dose .
Day 14 and Day 28 male $AUC_{0\text{-}t(last)}$ (AUC within 24 hours post dosing on Day 28) accumulation ratios were, on average, 1.6 and 3.9 after 50 mg/kg/day, 1.2 and 1.5 after 200 mg/kg/day and 0.5 and 0.4 after 800 mg/kg/day, respectively. The corresponding values in females were 2.1 and 2.5, 1.6 and 1.5, 0.8 and 0.6.
The systemic exposure to the metabolite was much higher than that to the parent compound.

Day 14 and Day 28 mean ±SD systemic exposure to the sulfone metabolite is reported in the following table

Dose	Male			Female		
mg/kg/day	Cmax	tmax	AUC0-t(last)	Cmax	tmax	AUC0-t(last)
	ng/mL	hour	ng·hour/mL	ng/mL	hour	ng·hour/mL
	Day 14					
50	4773±566	4±0	62800±11300	7980±2285	8±0	116000±24900
200	18800±1735	7±2	268000±35800	21533±2458	8±0	306000±31200
800	30833±1724	5±2	423000±70500	50233±11651	8±0	664000±332000
	Day 28					
50	7750±831	7±2	106267±9530 (1)	10700±700	7±2	174667±40501 (1)
			113000±13700			197000±50600
200	24200±3041	8±0	341333±39068 (1)	21433±4179	8±0	341333±66124 (1)
			387000±34000			448000±135000
800	33000±6655	5±2	459000±60605 (1)	41900±2970(2)	6±3(2)	571500±54447(1,2)
			509000±94900			738000±135000(2)
(1) AUC within 24 hours post dosing - (2) n=2						

At each dose, no relevant gender difference in C_{max} and $AUC_{0\text{-}t(last)}$ values was observed. No relevant difference in the levels of the metabolite were observed on Day 28 compared to Day 14. T_{max} values of the metabolite were achieved at later times in comparison with the corresponding ones of the parent compound. On Day 28, mean male apparent terminal half-lives were 6.1 ±1.2, 7.5 ±2.4 and 10 ±3 hours after 50, 200 and 800 mg/kg/day, respectively, the corresponding half-lives in females were 13 ±9.8, 7.3 ±2.6 and 11 ±5.6 (n=2) hours. The systemic exposure to the metabolite increased with the dose.
Day 14 and Day 28 male $AUC_{0\text{-}t(last)}$ (AUC within 24 hours post dosing on Day 28) accumulation ratios were, on average, 5.2 and 9.3 after 50 mg/kg/day, 2.3 and 2.9 after 200 mg/kg/day and 1.3 and 1.6 after 800 mg/kg/day, respectively. The corresponding values in females were 2.3 and 3.8, 2.3 and 2.6, 1 and 0.8.
The systemic exposure to the metabolite was much higher than that to the parent compound.

13. DISCUSSION

Fexinidazole was administered orally by gavage once daily for 28 consecutive days to ten or fifteen Crl:CD (SD)IGS BR rats/sex/group at doses of 50, 200 and 800 mg/kg/day in a volume of 10 mL/kg.

Fexinidazole was well tolerated at all doses tested. No drug-related deaths occurred and no meaningful clinical signs were seen during the study. In addition, at hematology, clinical chemistry and urinalysis no clear treatment-related changes were recorded during the study.

Minimal to slight signs of toxicity were restricted to a dose-dependent, minimal to slight, reduction in body weight seen in male animals at 200 and 800 mg/kg for the entire treatment period which was concomitant with a dose-dependent minimal to slight decrease in food consumption. When treatment was discontinued a tendency to recover was seen both for body weight and food consumption although values on Day 42 remained below the control values. No meaningful toxicological changes in body weight and food consumption were seen in female animals during treatment period.

A direct effect of the treatment, indicative of adaptation, was observed in the liver at all doses with some dose-relationship. On gross examination the liver from females showed increases in mean absolute and relative weights, which were minimal at the doses of 50 and 200 mg/kg/day and more prominent and statistically significant in animals receiving 800 mg/kg/day. Minimal to slight hypertrophy (increase in cell size) of the centrilobular hepatocytes was observed histologically in almost all animals of both sexes, at all doses with scant dose-relationship. This finding correlated with the liver weight increases in females. After recovery the weight increases regressed completely at the dose of 800 mg/kg/day and the hepatocellular hypertrophy showed partial significant regression in terms of both incidence and severity in both sexes. Hepatocellular hypertrophy, may be indicative of adaptation, which, in itself, is not necessarily adverse unless associated with other more severe changes (e.g. degeneration, necrosis and hyperplasia) [(1)].

After the first and repeated dosing in male and female rats Fexinidazole was extensively metabolized to the sulfone and sulfoxide derivatives.
On Days 1, 14 and 28, in both genders, AUCs of Fexinidazole and its metabolites increased with the dose administered.
After the first and repeated administrations of the three dose levels, Cmax and AUCs of Fexinidazole were two to three times higher in females than in males. No relevant gender differences were detected for metabolites on Day 1, except at the low dose of 50 mg/kg. On Days 14 and 28 no relevant gender differences were detected for the sulfone metabolite, while Cmax and AUCs of the sulfoxide metabolite were two to three times higher in females than in males. This may be indicative of a higher metabolic activity in females, with a consequent more marked increase in liver weight.

14. CONCLUSIONS

Fexinidaxole, given orally once daily for 28 consecutive days at doses of 50, 200 or 800 mg/kg/day to Crl:CD (SD)IGS BR rats, was well tolerated at all doses tested inducing only minimal to slight decrease in body weight and food consumption in male animals at 200 and 800 mg/kg. The minimal to moderate changes observed in the liver of all fexinidaxole-

treated animals (increased liver weight and/or hypertrophy of the centrilobular hepatocytes) were restricted to the dosing period and were considered of adaptative origin. Based on these results the dose of 200 mg/kg was considered the NOAEL. This dose on Day 28 corresponds to a mean AUC_{0-24} of fexinidazole of 3173 or 7640 ng•h/mL in males and females, respectively, a mean AUC_{0-24} of sulfoxide metabolite of 129733 or 291500 ng•h/mL in males and females, respectively, and a mean AUC_{0-24} of sulfone metabolite of 459000 or 571500 ng•h/mL in males and females, respectively.

15. REFERENCES

(1) USEPA (2002) Hepatocellular hypertrophy. HED Guidance Document # G2002.01, October 21 (p.21-24)

FIGURES

Figure 1 Mean Body Weight

Fig. 1: Mean Body Weight (g)

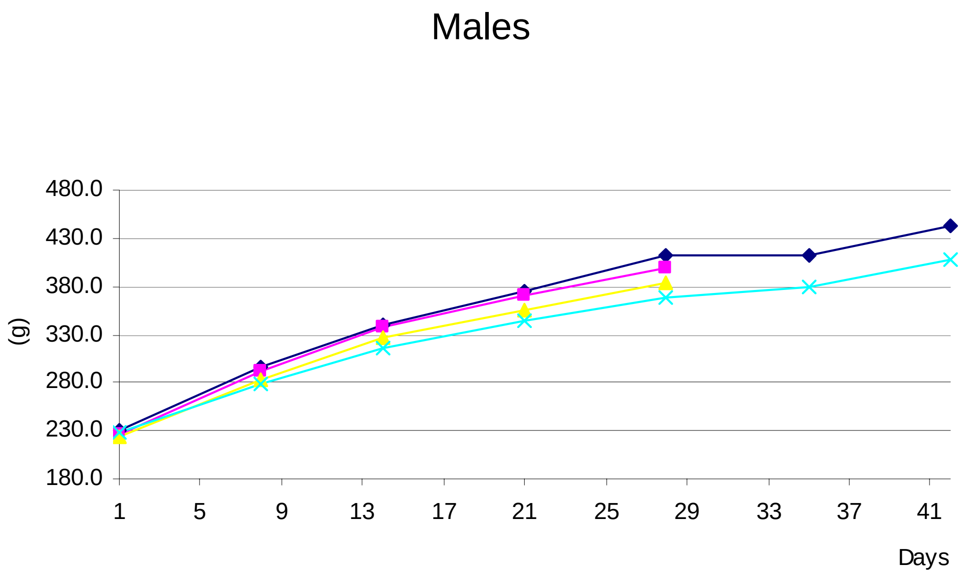

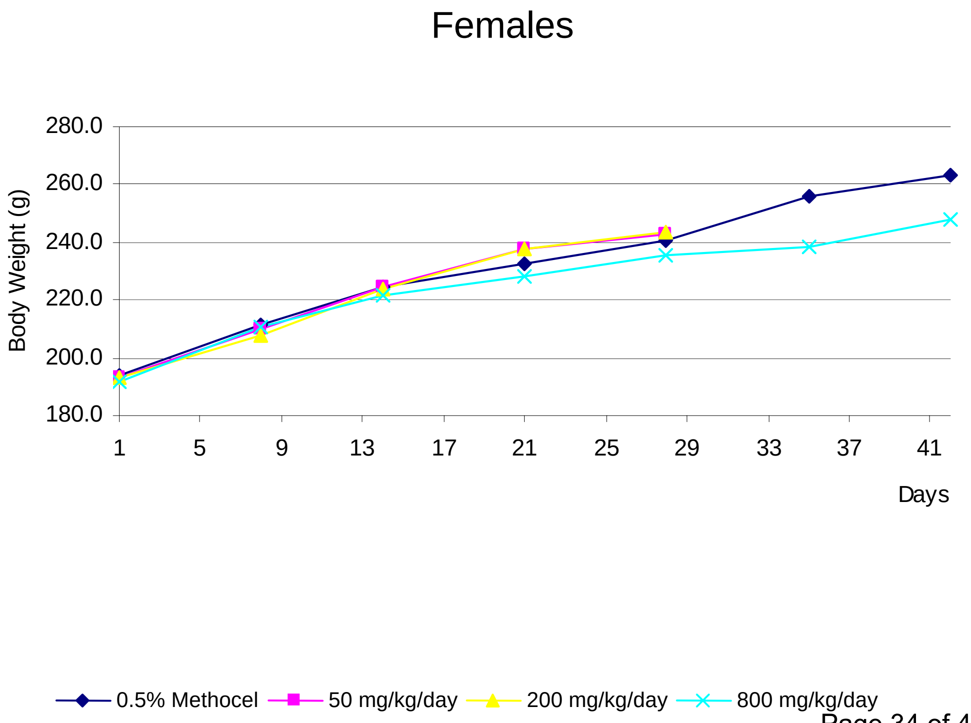

Figure 2 Mean Food Consumption

Fig. 2: Mean Food Consumption (g/rat/day)

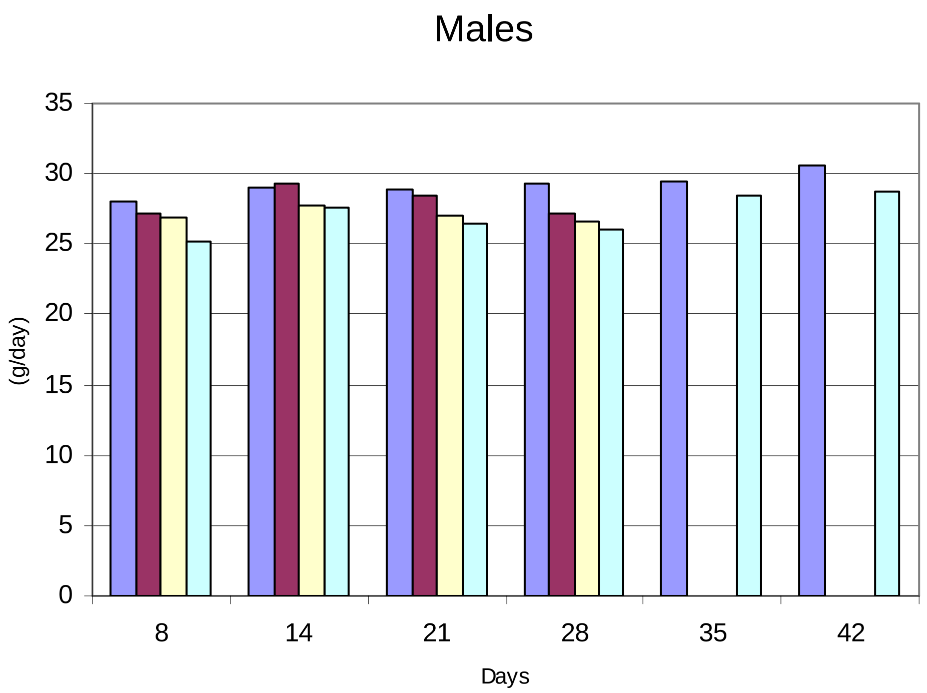

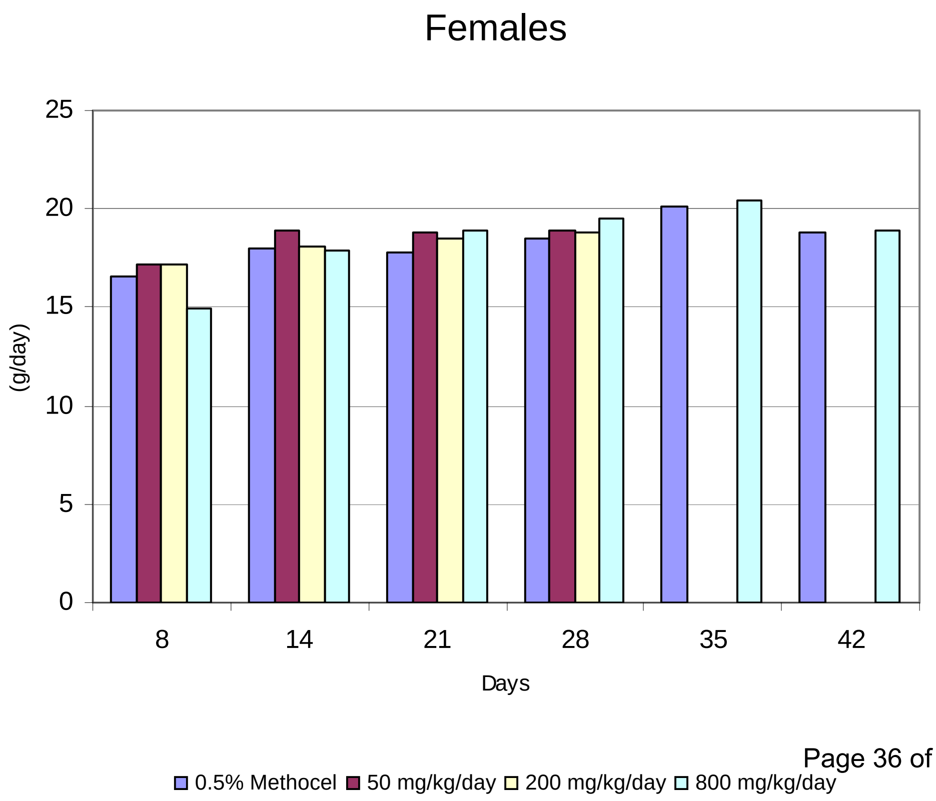

TABLES

Table 1 Clinical Signs

Table 1
Group Incidence of Clinical Signs

Fexinidazole — Study Number: 0504-2007

Study Days 1-43	Group Number	1		2		3		4	
		Males							
	Number of Animals+	15		10		10		15	
		a	(b)	a	(b)	a	(b)	a	(b)
Normal									
Normal/no visible abnormalities		15	(34.0)	10	(29.5)	10	(28.3)	15	(34.0)
Body surface									
Accidental wound		0	(0.0)	0	(0.0)	0	(0.0)	0	(0.0)
Focal alopecia		0	(0.0)	0	(0.0)	2	(2.0)	0	(0.0)
Fur thinning		0	(0.0)	0	(0.0)	0	(0.0)	0	(0.0)
Scabbed area		0	(0.0)	0	(0.0)	1	(7.0)	0	(0.0)
Ulceration		0	(0.0)	0	(0.0)	1	(1.0)	0	(0.0)

Group 1:vehicle   Group 2:50 mg/kg   Group 3:200 mg/kg   Group 4:800 mg/kg

Key: + = Number of animals alive at start of interval
a = Number of animals affected
(b) = Mean number of animal days that the group displayed the sign

Table 1
Group Incidence of Clinical Signs

Fexinidazole | Study Number: 0504-2007

Study Days 1-43	Females							
Group Number	1		2		3		4	
Number of Animals+	15		10		10		15	
	a	(b)	a	(b)	a	(b)	a	(b)
**Normal**								
Normal/no visible abnormalities	15	(33.4)	10	(28.2)	10	(28.6)	15	(31.5)
**Body surface**								
Accidental wound	0	(0.0)	2	(6.5)	0	(0.0)	0	(0.0)
Focal alopecia	0	(0.0)	0	(0.0)	0	(0.0)	1	(21.0)
Fur thinning	1	(9.0)	0	(0.0)	0	(0.0)	0	(0.0)
Scabbed area	0	(0.0)	0	(0.0)	1	(9.0)	0	(0.0)
Ulceration	0	(0.0)	0	(0.0)	0	(0.0)	0	(0.0)

Group 1:vehicle   Group 2:50 mg/kg   Group 3:200 mg/kg   Group 4:800 mg/kg

Key: + = Number of animals alive at start of interval
a = Number of animals affected
(b) = Mean number of animal days that the group displayed the sign

Table 2 Body Weights

Table 2
Body Weights (g)

Fexinidazole Study Number: 0504-2007

Group Number	Dose Level	Study Day	-4"	1#	8	14	21	28	35	42
			**M a l e s**							
1	vehicle	N	15	15	15	15	15	15	5	5
		Mean	184.65	230.51	295.95	339.69	375.81	411.43	411.48	443.70
		Sdev	7.555	9.488	14.740	17.261	22.066	26.150	35.483	43.977
2	50 mg/kg	N	10	10	10	10	10	10	0	0
		Mean	180.41	226.01	292.00	338.43	371.37	398.72	-	-
		Sdev	8.305	12.514	20.140	26.098	33.719	37.044	-	-
3	200 mg/kg	N	10	10	10	10	10	10	0	0
		Mean	176.41	222.75	283.82	326.04	355.11	383.27	-	-
		Sdev	12.286	9.177	16.202	25.391	33.557	43.635	-	-
4	800 mg/kg	N	15	15	15	15	15	15	5	5
		Mean	184.53	227.38	279.83+	316.42+	343.38+	368.37*	379.10	407.84
		Sdev	9.253	11.692	15.294	24.025	30.076	33.944	26.169	30.210

Note: " = Pretest phase (groups); # = Test period
+ The group mean was significantly different from the control at p=0.05 with Dunnett's test of significance
* The group mean was significantly different from the control at p=0.01 with Dunnett's test of significance

Table 2
Body Weights (g)

Fexinidazole | Study Number: 0504-2007

Group Number	Dose Level	Study Day	-4"	1#	8	14	21	28	35	42
			Females							
1	vehicle	N	15	15	15	15	15	15	5	5
		Mean	174.38	193.63	211.34	224.88	232.91	240.77	255.74	263.14
		Sdev	6.636	9.427	9.771	10.257	13.184	13.302	10.702	12.416
2	50 mg/kg	N	10	10	10	10	10	10	0	0
		Mean	172.13	193.40	210.07	224.83	237.95	242.96	-	-
		Sdev	7.049	8.686	11.221	15.835	13.784	15.632	-	-
3	200 mg/kg	N	10	10	10	10	10	10	0	0
		Mean	170.59	193.06	207.44	223.70	237.52	243.66	-	-
		Sdev	7.623	7.484	6.935	8.361	10.225	12.139	-	-
4	800 mg/kg	N	15	15	15	14	14	14	5	5
		Mean	173.72	191.44	210.95	221.57	228.37	235.75	238.74	248.12
		Sdev	6.485	11.039	9.748	12.295	12.410	14.788	13.715	15.860

Note: " = Pretest phase (groups); # = Test period

Table 3 Food Consumption

Table 3
Feed Consumed per Day (g)

Fexinidazole Study Number: 0504-2007

Group Number	Dose Level	Study Day	Males 8	14	21	28	35	42
1	vehicle	N	15	15	15	15	5	5
		Mean	28.05	29.04	28.95	29.37	29.51	30.53
		Sdev	1.90	1.25	1.28	1.15	1.47	1.27
2	50 mg/kg	N	10	10	10	10	0	0
		Mean	27.14	29.25	28.48	27.12*	-	-
		Sdev	1.71	1.29	2.02	0.84	-	-
3	200 mg/kg	N	10	10	10	10	0	0
		Mean	26.84+	27.72+	27.09+	26.65*	-	-
		Sdev	0.62	1.55	1.89	1.66	-	-
4	800 mg/kg	N	15	15	15	15	5	5
		Mean	25.18*	27.67	26.50+	25.97*	28.50	28.78
		Sdev	1.54	3.51	3.45	2.87	3.08	2.50

Note: Data for Test period
+ The group mean was significantly different from the control at p=0.05 with Dunnett's test of significance
* The group mean was significantly different from the control at p=0.01 with Dunnett's test of significance

Table 3
Feed Consumed per Day (g)

Fexinidazole | Study Number: 0504-2007

Group Number	Dose Level	Study Day	Females 8	14	21	28	35	42
1	vehicle	N	15	15	15	15	5	5
		Mean	16.55	18.01	17.74	18.48	20.12	18.84
		Sdev	2.90	0.93	0.98	1.17	0.77	0.28
2	50 mg/kg	N	10	10	10	10	0	0
		Mean	17.14	18.91	18.78	18.86	-	-
		Sdev	0.84	1.39	1.28	1.45	-	-
3	200 mg/kg	N	10	10	10	10	0	0
		Mean	17.16	18.14	18.48	18.78	-	-
		Sdev	1.02	0.72	1.81	2.13	-	-
4	800 mg/kg	N	15	15	14	14	5	5
		Mean	14.89	17.87	18.90	19.55	20.38	18.91
		Sdev	0.79	1.19	1.40	1.78	0.30	1.14

Note: Data for Test period

Table 4 Ophthalmoscopic Findings

Table 4
Incidence of Ophthalmoscopic Findings

Fexinidazole Study Number: 0504-2007

Study Day	Group:	Males 1	2	3	4
-2					
NORMAL	/Normal (No Abnormal Findings)	15	10	10	15
28					
NORMAL	/Normal (No Abnormal Findings)	15	10	10	15
42					
NORMAL	/Normal (No Abnormal Findings)	5	0	0	5

Group 1:vehicle Group 2:50 mg/kg Group 3:200 mg/kg Group 4:800 mg/kg

Table 4
Incidence of Ophthalmoscopic Findings

Fexinidazole

Study Number: 0504-2007

Study Day	Group:	Females 1	2	3	4
-2					
NORMAL	/Normal (No Abnormal Findings)	15	10	10	15
28					
NORMAL	/Normal (No Abnormal Findings)	15	10	10	14
42					
NORMAL	/Normal (No Abnormal Findings)	5	0	0	5

Group 1:vehicle   Group 2:50 mg/kg   Group 3:200 mg/kg   Group 4:800 mg/kg

Table 5 Hematology

Table 5
Day 29 Hematology Data
Test period

Session 1 (Scheduled)
Fexinidazole

Study Number: 0504-2007

Parameter	Group Number:	Males 1	Males 2	Males 3	Males 4	Females 1	Females 2	Females 3	Females 4
RED BLOOD CELLS	N	5	5	5	5	5	5	5	5
10^6/mcL	Mean	8.70	8.35	8.49	8.30	8.30	8.11	8.11	7.92
	Sdev	0.499	0.261	0.354	0.342	0.331	0.190	0.097	0.327
HEMOGLOBIN	N	5	5	5	5	5	5	5	5
g/dL	Mean	15.7	15.2	15.6	15.3	15.1	14.9	14.7	13.9*
	Sdev	0.63	0.36	0.37	0.31	0.63	0.46	0.52	0.50
HEMATOCRIT	N	5	5	5	5	5	5	5	5
%	Mean	47.9	47.0	48.0	46.8	44.2	44.1	43.7	41.8
	Sdev	1.62	1.08	1.11	1.65	1.96	1.11	1.16	1.68
MEAN CORPUSCULAR VOLUME	N	5	5	5	5	5	5	5	5
fL	Mean	55.1	56.4	56.7	56.4	53.3	54.5	53.9	52.8
	Sdev	1.83	1.67	2.28	0.38	1.44	1.18	1.11	1.06
MEAN CORPUSCULAR HEMOGLOBIN	N	5	5	5	5	5	5	5	5
pg	Mean	18.0	18.3	18.3	18.5	18.2	18.3	18.1	17.6
	Sdev	0.75	0.66	0.78	0.48	0.57	0.36	0.51	0.39
MEAN CORPUSCULAR HGB CONC.	N	5	5	5	5	5	5	5	5
g/dL	Mean	32.7	32.4	32.4	32.7	34.2	33.7	33.6	33.4+
	Sdev	0.52	0.28	0.49	0.68	0.36	0.36	0.60	0.51
RED CELL DISTRIBUTION WIDTH	N	5	5	5	5	5	5	5	5
%	Mean	10.9	11.0	10.7	10.9	10.4	10.6	11.1+	10.9
	Sdev	0.29	0.44	0.47	0.50	0.22	0.38	0.53	0.49

Group 1:vehicle Group 2:50 mg/kg Group 3:200 mg/kg Group 4:800 mg/kg

Group means without footnotes were not statistically different from the control at p=0.05 or p=0.01.
+ The group mean was significantly different from the control at p=0.05 with Dunnett's test of significance
* The group mean was significantly different from the control at p=0.01 with Dunnett's test of significance

Table 5
Day 29 Hematology Data
Test period

Session 1 (Scheduled)
Fexinidazole

Study Number: 0504-2007

Parameter	Group Number:	Males 1	Males 2	Males 3	Males 4	Females 1	Females 2	Females 3	Females 4
HEMOGLOBIN DISTRIB. WIDTH	N	5	5	5	5	5	5	5	5
g/dL	Mean	2.60	2.63	2.64	2.88+	2.30	2.45	2.56+	2.53
	Sdev	0.060	0.048	0.093	0.212	0.150	0.151	0.089	0.179
RETICULOCYTES	N	5	5	5	5	5	5	5	5
%	Mean	2.6	2.5	2.3	2.8	2.2	2.5	2.3	2.8
	Sdev	0.31	0.39	0.89	0.39	0.46	0.74	0.60	0.31
RETICULOCYTES ABS	N	5	5	5	5	5	5	5	5
10^9/L	Mean	223.4	205.0	189.3	233.4	179.4	202.2	189.9	218.1
	Sdev	17.46	29.01	66.54	31.22	31.60	55.68	47.06	23.88
MEAN CORPUSCOLAR VOL. RETIC.	N	5	5	5	5	5	5	5	5
fL	Mean	62.2	63.9	64.1	63.9	61.2	63.7+	63.1	62.0
	Sdev	1.70	1.05	1.99	0.46	1.63	1.02	0.39	1.22
MEAN HEMOGLOBIN CONC. RETIC.	N	5	5	5	5	5	5	5	5
g/dL	Mean	30.0	29.7	30.1	30.0	31.6	31.4	31.5	30.6*
	Sdev	0.27	0.28	0.40	0.23	0.38	0.32	0.49	0.12
CELLULAR HEMOGLOBIN RETIC.	N	5	5	5	5	5	5	5	5
pg	Mean	18.6	18.9	19.3	19.1	19.3	20.0	19.8	18.9
	Sdev	0.60	0.48	0.85	0.27	0.66	0.44	0.39	0.34
PLATELETS	N	5	5	5	5	5	5	5	5
10^3/mcL	Mean	1234.	1411.	1245.	1300.	1299.	1108.	1252.	1258.
	Sdev	89.9	129.4	275.2	94.3	133.7	95.6	207.7	89.5

Group 1:vehicle   Group 2:50 mg/kg   Group 3:200 mg/kg   Group 4:800 mg/kg

Group means without footnotes were not statistically different from the control at p=0.05 or p=0.01.
+ The group mean was significantly different from the control at p=0.05 with Dunnett’s test of significance
* The group mean was significantly different from the control at p=0.01 with Dunnett’s test of significance

Table 5
Day 29 Hematology Data
Test period

Session 1 (Scheduled)
Fexinidazole

Study Number: 0504-2007

Parameter	Group Number:	Males 1	Males 2	Males 3	Males 4	Females 1	Females 2	Females 3	Females 4
MEAN PLATELET VOLUME	N	5	5	5	5	5	5	5	5
fL	Mean	6.7	6.7	6.5	6.6	5.8	6.0	6.1	6.0
	Sdev	0.16	0.49	0.13	0.22	0.12	0.17	0.39	0.13
PLATELET DISTRIBUTION WIDTH	N	5	5	5	5	5	5	5	5
%	Mean	54.9	52.7	54.6	54.9	53.2	52.7	53.4	53.1
	Sdev	2.71	1.10	2.07	0.91	2.26	2.29	2.44	1.85
PLATELET HEMATOCRIT	N	5	5	5	5	5	5	5	5
%	Mean	0.83	0.95	0.81	0.86	0.75	0.66	0.77	0.75
	Sdev	0.064	0.145	0.169	0.079	0.081	0.042	0.145	0.068
WHITE BLOOD CELLS	N	5	5	5	5	5	5	5	5
10^3/mcL	Mean	11.19	11.95	11.99	10.25	10.87	9.51	10.23	10.98
	Sdev	1.479	1.804	2.726	1.996	2.777	2.322	2.901	2.124
NEUTROPHILS ABS	N	5	5	5	5	5	5	5	5
10^3/mcL	Mean	1.78	2.08	1.92	1.83	1.93	1.70	1.55	1.03
	Sdev	0.386	0.279	0.277	0.644	0.872	0.601	0.903	0.258
LYMPHOCYTES ABS	N	5	5	5	5	5	5	5	5
10^3/mcL	Mean	8.83	9.20	9.46	7.86	8.35	7.21	8.12	9.43
	Sdev	1.153	1.670	2.609	1.475	1.888	2.246	2.280	2.064
MONOCYTES ABS	N	5	5	5	5	5	5	5	5
10^3/mcL	Mean	0.32	0.32	0.30	0.28	0.29	0.30	0.28	0.26
	Sdev	0.157	0.054	0.007	0.021	0.094	0.064	0.038	0.077

Group 1:vehicle   Group 2:50 mg/kg   Group 3:200 mg/kg   Group 4:800 mg/kg

Group means without footnotes were not statistically different from the control at p=0.05 or p=0.01.

Table 5
Day 29 Hematology Data
Test period

Session 1 (Scheduled)
Fexinidazole

Study Number: 0504-2007

Parameter	Group Number:	Males 1	Males 2	Males 3	Males 4	Females 1	Females 2	Females 3	Females 4
EOSINOPHILS ABS	N	5	5	5	5	5	5	5	5
10^3/mcL	Mean	0.10	0.13	0.13+	0.12	0.16	0.17	0.11	0.10
	Sdev	0.011	0.026	0.027	0.055	0.027	0.062	0.021	0.031
BASOPHILS ABS	N	5	5	5	5	5	5	5	5
10^3/mcL	Mean	0.03	0.03	0.02	0.02	0.03	0.02*	0.02	0.03
	Sdev	0.008	0.005	0.011	0.011	0.000	0.005	0.011	0.004
LARGE UNSTAINED CELLS ABS	N	5	5	5	5	5	5	5	5
10^3/mcL	Mean	0.14	0.19	0.16	0.13	0.12	0.11	0.15	0.14
	Sdev	0.023	0.030	0.048	0.031	0.043	0.047	0.065	0.054
NEUTROPHILS %	N	5	5	5	5	5	5	5	5
%	Mean	15.8	17.7	16.5	17.7	17.1	18.7	15.0	9.6
	Sdev	2.11	2.87	3.65	4.13	3.88	9.17	6.39	2.95
LYMPHOCITES %	N	5	5	5	5	5	5	5	5
%	Mean	79.0	76.6	78.2	76.8	77.4	74.9	79.4	85.6
	Sdev	3.24	3.34	4.31	3.74	3.88	9.18	5.93	2.72
MONOCYTES %	N	5	5	5	5	5	5	5	5
%	Mean	2.8	2.7	2.6	2.8	2.7	3.3	2.8	2.4
	Sdev	1.13	0.60	0.51	0.55	0.70	0.77	0.51	0.61
EOSINOPHILS %	N	5	5	5	5	5	5	5	5
%	Mean	0.8	1.1	1.1+	1.1	1.5	1.8	1.1	0.9
	Sdev	0.11	0.40	0.08	0.31	0.31	0.52	0.11	0.39

Group 1:vehicle Group 2:50 mg/kg Group 3:200 mg/kg Group 4:800 mg/kg

Group means without footnotes were not statistically different from the control at p=0.05 or p=0.01.
+ The group mean was significantly different from the control at p=0.05 with Dunnett's test of significance
* The group mean was significantly different from the control at p=0.01 with Dunnett's test of significance

Table 5
Day 29 Hematology Data
Test period

Session 1 (Scheduled)
Fexinidazole
Study Number: 0504-2007

Parameter	Group Number:	Males 1	Males 2	Males 3	Males 4	Females 1	Females 2	Females 3	Females 4
BASOPHILS %	N	5	5	5	5	5	5	5	5
%	Mean	0.3	0.3	0.2	0.2	0.3	0.2	0.2	0.3
	Sdev	0.05	0.05	0.04	0.04	0.08	0.04	0.08	0.05
LARGE UNSTAINED CELLS %	N	5	5	5	5	5	5	5	5
%	Mean	1.2	1.6	1.3	1.3	1.1	1.1	1.4	1.3
	Sdev	0.36	0.23	0.43	0.40	0.26	0.23	0.37	0.30

Group 1:vehicle Group 2:50 mg/kg Group 3:200 mg/kg Group 4:800 mg/kg

Group means without footnotes were not statistically different from the control at p=0.05 or p=0.01.

Table 5
Day 43 Hematology Data
Test period

Session 1 (Scheduled)
Fexinidazole
Study Number: 0504-2007

Parameter	Group Number:	Males 1	Males 2	Males 3	Males 4	Females 1	Females 2	Females 3	Females 4
RED BLOOD CELLS	N	5	0	0	5	5	0	0	5
10^6/mcL	Mean	8.84	-	-	8.50	8.25	-	-	8.16
	Sdev	0.410	-	-	0.503	0.188	-	-	0.314
HEMOGLOBIN	N	5	0	0	5	5	0	0	5
g/dL	Mean	15.6	-	-	15.6	14.8	-	-	14.6
	Sdev	0.53	-	-	0.60	0.54	-	-	0.60
HEMATOCRIT	N	5	0	0	5	5	0	0	5
%	Mean	47.2	-	-	47.4	45.4	-	-	45.0
	Sdev	1.03	-	-	2.47	1.67	-	-	1.48
MEAN CORPUSCULAR VOLUME	N	5	0	0	5	5	0	0	5
fL	Mean	53.4	-	-	55.8+	55.1	-	-	55.1
	Sdev	1.69	-	-	0.59	1.77	-	-	1.00
MEAN CORPUSCULAR HEMOGLOBIN	N	5	0	0	5	5	0	0	5
pg	Mean	17.7	-	-	18.4	17.9	-	-	17.9
	Sdev	0.72	-	-	0.62	0.55	-	-	0.15
MEAN CORPUSCULAR HGB CONC.	N	5	0	0	5	5	0	0	5
g/dL	Mean	33.1	-	-	32.9	32.5	-	-	32.4
	Sdev	0.56	-	-	0.74	0.24	-	-	0.44
RED CELL DISTRIBUTION WIDTH	N	5	0	0	5	5	0	0	5
%	Mean	11.7	-	-	12.0	11.4	-	-	12.2*
	Sdev	0.41	-	-	0.46	0.29	-	-	0.44

Group 1:vehicle   Group 2:50 mg/kg   Group 3:200 mg/kg   Group 4:800 mg/kg

Group means without footnotes were not statistically different from the control at p=0.05 or p=0.01.
+ The group mean was significantly different from the control at p=0.05 with Dunnett’s test of significance
* The group mean was significantly different from the control at p=0.01 with Dunnett’s test of significance

Table 5
Day 43 Hematology Data
Test period

Session 1 (Scheduled)
Fexinidazole

Study Number: 0504-2007

Parameter	Group Number:	Males 1	Males 2	Males 3	Males 4	Females 1	Females 2	Females 3	Females 4
HEMOGLOBIN DISTRIB. WIDTH	N	5	0	0	5	5	0	0	5
g/dL	Mean	2.67	-	-	2.80	2.31	-	-	2.34
	Sdev	0.101	-	-	0.125	0.143	-	-	0.080
RETICULOCYTES	N	5	0	0	5	5	0	0	5
%	Mean	2.5	-	-	3.3+	2.7	-	-	2.3
	Sdev	0.39	-	-	0.44	0.31	-	-	0.55
RETICULOCYTES ABS	N	5	0	0	5	5	0	0	5
10^9/L	Mean	223.2	-	-	277.3+	223.5	-	-	186.5
	Sdev	27.65	-	-	26.55	27.21	-	-	42.74
MEAN CORPUSCOLAR VOL. RETIC.	N	5	0	0	5	5	0	0	5
fL	Mean	62.0	-	-	64.7+	64.8	-	-	64.5
	Sdev	1.78	-	-	0.95	1.75	-	-	1.06
MEAN HEMOGLOBIN CONC. RETIC.	N	5	0	0	5	5	0	0	5
g/dL	Mean	30.3	-	-	30.6	30.5	-	-	30.0+
	Sdev	0.24	-	-	0.44	0.42	-	-	0.26
CELLULAR HEMOGLOBIN RETIC.	N	5	0	0	5	5	0	0	5
pg	Mean	18.8	-	-	19.8+	19.7	-	-	19.3
	Sdev	0.59	-	-	0.51	0.73	-	-	0.40
PLATELETS	N	5	0	0	5	5	0	0	5
10^3/mcL	Mean	1179.	-	-	1206.	1346.	-	-	1421.
	Sdev	147.1	-	-	102.0	82.9	-	-	128.7

Group 1:vehicle Group 2:50 mg/kg Group 3:200 mg/kg Group 4:800 mg/kg

Group means without footnotes were not statistically different from the control at p=0.05 or p=0.01.
\+ The group mean was significantly different from the control at p=0.05 with Dunnett’s test of significance

Table 5
Day 43 Hematology Data
Test period

Session 1 (Scheduled)
Fexinidazole
Study Number: 0504-2007

Parameter	Group Number:	Males 1	Males 2	Males 3	Males 4	Females 1	Females 2	Females 3	Females 4
MEAN PLATELET VOLUME	N	5	0	0	5	5	0	0	5
fL	Mean	6.3	-	-	6.4	6.7	-	-	6.8
	Sdev	0.27	-	-	0.11	0.04	-	-	0.18
PLATELET DISTRIBUTION WIDTH	N	5	0	0	5	5	0	0	5
%	Mean	55.6	-	-	54.2	51.8	-	-	52.2
	Sdev	2.63	-	-	1.93	1.25	-	-	1.94
PLATELET HEMATOCRIT	N	5	0	0	5	5	0	0	5
%	Mean	0.75	-	-	0.77	0.90	-	-	0.96
	Sdev	0.115	-	-	0.061	0.054	-	-	0.097
WHITE BLOOD CELLS	N	5	0	0	5	5	0	0	5
10^3/mcL	Mean	10.50	-	-	10.59	10.61	-	-	13.42
	Sdev	1.292	-	-	1.235	1.907	-	-	2.374
NEUTROPHILS ABS	N	5	0	0	5	5	0	0	5
10^3/mcL	Mean	1.81	-	-	1.89	1.49	-	-	1.65
	Sdev	0.421	-	-	0.308	0.390	-	-	0.507
LYMPHOCYTES ABS	N	5	0	0	5	5	0	0	5
10^3/mcL	Mean	8.13	-	-	8.09	8.57	-	-	11.00
	Sdev	1.038	-	-	1.077	1.895	-	-	1.925
MONOCYTES ABS	N	5	0	0	5	5	0	0	5
10^3/mcL	Mean	0.32	-	-	0.34	0.24	-	-	0.40*
	Sdev	0.141	-	-	0.107	0.070	-	-	0.066

Group 1:vehicle   Group 2:50 mg/kg   Group 3:200 mg/kg   Group 4:800 mg/kg

Group means without footnotes were not statistically different from the control at p=0.05 or p=0.01.
* The group mean was significantly different from the control at p=0.01 with Dunnett’s test of significance

CONFIDENTIAL

Table 5
Day 43 Hematology Data
Test period

Session 1 (Scheduled)
Fexinidazole

Study Number: 0504-2007

Parameter	Group Number:	Males 1	Males 2	Males 3	Males 4	Females 1	Females 2	Females 3	Females 4
EOSINOPHILS ABS	N	5	0	0	5	5	0	0	5
10^3/mcL	Mean	0.14	-	-	0.14	0.13	-	-	0.11
	Sdev	0.043	-	-	0.045	0.019	-	-	0.050
BASOPHILS ABS	N	5	0	0	5	5	0	0	5
10^3/mcL	Mean	0.03	-	-	0.03	0.02	-	-	0.04
	Sdev	0.013	-	-	0.009	0.005	-	-	0.015
LARGE UNSTAINED CELLS ABS	N	5	0	0	5	5	0	0	5
10^3/mcL	Mean	0.08	-	-	0.11	0.16	-	-	0.23
	Sdev	0.049	-	-	0.051	0.082	-	-	0.085
NEUTROPHILS %	N	5	0	0	5	5	0	0	5
%	Mean	17.3	-	-	17.9	14.4	-	-	12.1
	Sdev	3.55	-	-	2.19	5.07	-	-	2.32
LYMPHOCITES %	N	5	0	0	5	5	0	0	5
%	Mean	77.5	-	-	76.3	80.3	-	-	82.0
	Sdev	4.25	-	-	3.15	6.01	-	-	1.70
MONOCYTES %	N	5	0	0	5	5	0	0	5
%	Mean	3.0	-	-	3.3	2.3	-	-	3.0
	Sdev	1.07	-	-	1.02	0.65	-	-	0.41
EOSINOPHILS %	N	5	0	0	5	5	0	0	5
%	Mean	1.3	-	-	1.3	1.2	-	-	0.9
	Sdev	0.31	-	-	0.33	0.23	-	-	0.44

Group 1:vehicle   Group 2:50 mg/kg   Group 3:200 mg/kg   Group 4:800 mg/kg

Group means without footnotes were not statistically different from the control at p=0.05 or p=0.01.

Table 5
Day 43 Hematology Data
Test period

Session 1 (Scheduled)
Fexinidazole

Study Number: 0504-2007

Parameter	Group Number:	Males 1	Males 2	Males 3	Males 4	Females 1	Females 2	Females 3	Females 4
BASOPHILS %	N	5	0	0	5	5	0	0	5
%	Mean	0.3	-	-	0.3	0.2	-	-	0.3
	Sdev	0.12	-	-	0.09	0.05	-	-	0.09
LARGE UNSTAINED CELLS %	N	5	0	0	5	5	0	0	5
%	Mean	0.7	-	-	1.1	1.5	-	-	1.8
	Sdev	0.35	-	-	0.48	0.62	-	-	0.65

Group 1:vehicle Group 2:50 mg/kg Group 3:200 mg/kg Group 4:800 mg/kg

Group means without footnotes were not statistically different from the control at p=0.05 or p=0.01.

Table 6 Clinical Chemistry

Table 6
Day 29 Clinical Chemistry Data
Test period

Session 1 (Scheduled)
Fexinidazole

Study Number: 0504-2007

Parameter	Group Number:	Males 1	Males 2	Males 3	Males 4	Females 1	Females 2	Females 3	Females 4
UREA	N	5	5	5	5	5	5	5	5
mg/dL	Mean	28.	27.	30.	27.	34.	33.	31.	37.
	Sdev	4.6	2.5	1.1	2.3	4.0	3.6	3.0	4.2
CREATININE	N	5	5	5	5	5	5	5	5
mg/dL	Mean	0.55	0.56	0.56	0.58	0.62	0.65	0.73*	0.79*
	Sdev	0.030	0.040	0.029	0.047	0.029	0.041	0.043	0.046
ASPARTATE AMINO TRANSFERASE	N	5	5	5	5	5	5	5	5
IU/L	Mean	147.	132.	131.	124.	122.	126.	130.	114.
	Sdev	24.5	22.1	19.1	21.6	25.9	18.5	21.3	19.4
ALANINE AMINO TRANSFERASE	N	5	5	5	5	5	5	5	5
IU/L	Mean	28.	28.	29.	25.	29.	27.	26.	23.
	Sdev	6.6	5.3	4.2	3.3	5.9	3.8	2.9	1.2
ALKALINE PHOSPHATASE	N	5	5	5	5	5	5	5	5
IU/L	Mean	188.	144.	132.+	120.+	92.	82.	75.	61.
	Sdev	30.5	47.6	10.6	25.5	20.6	12.9	28.6	9.9
GAMMA GLUTAMYL TRANSFERASE	N	5	2	0	1	5	2	2	1
IU/L	Mean	5.	5.	-	5.	5.	5.	5.	4.
	Sdev	2.5	0.0	-	-	0.5	0.7	0.0	-
TOTAL BILIRUBIN	N	5	5	5	5	5	5	5	5
mg/dL	Mean	0.09	0.06*	0.07	0.07	0.12	0.11	0.11	0.11
	Sdev	0.018	0.011	0.015	0.008	0.034	0.010	0.021	0.009

Group 1:vehicle   Group 2:50 mg/kg   Group 3:200 mg/kg   Group 4:800 mg/kg

Group means without footnotes were not statistically different from the control at p=0.05 or p=0.01.
+ The group mean was significantly different from the control at p=0.05 with Dunnett’s test of significance
* The group mean was significantly different from the control at p=0.01 with Dunnett’s test of significance

Table 6
Day 29 Clinical Chemistry Data
Test period

Session 1 (Scheduled)
Fexinidazole

Study Number: 0504-2007

Parameter	Group Number:	Males 1	Males 2	Males 3	Males 4	Females 1	Females 2	Females 3	Females 4
TOTAL PROTEIN	N	5	5	5	5	5	5	5	5
g/dL	Mean	6.7	6.8	6.8	7.0	7.3	7.7	7.6	8.0+
	Sdev	0.23	0.21	0.20	0.23	0.30	0.46	0.36	0.30
ALBUMIN	N	5	5	5	5	5	5	5	5
g/dL	Mean	3.09	3.10	3.16	3.17	3.46	3.63	3.69	3.78
	Sdev	0.129	0.103	0.032	0.134	0.187	0.309	0.249	0.148
GLOBULIN	N	5	5	5	5	5	5	5	5
g/dL	Mean	3.6	3.7	3.7	3.8	3.8	4.1	3.9	4.3+
	Sdev	0.11	0.19	0.21	0.11	0.17	0.20	0.33	0.16
GLUCOSE	N	5	5	5	5	5	5	5	5
mg/dL	Mean	80.	81.	84.	78.	91.	91.	95.	105.+
	Sdev	6.5	6.8	8.2	3.1	10.4	4.9	6.1	8.0
TRIGLYCERIDES	N	5	5	5	5	5	5	5	5
mg/dL	Mean	57.	56.	43.	28.	34.	48.	52.	44.
	Sdev	19.7	17.1	25.3	10.8	5.3	7.2	13.9	16.3
TOTAL CHOLESTEROL	N	5	5	5	5	5	5	5	5
mg/dL	Mean	55.	54.	54.	64.	70.	88.	90.	106.+
	Sdev	4.2	12.7	14.2	17.2	16.6	14.6	17.4	19.2
CALCIUM	N	5	5	5	5	5	5	5	5
mg/dL	Mean	10.2	10.2	10.0	10.3	10.6	10.7	10.7	11.3
	Sdev	0.22	0.07	0.40	1.06	0.21	0.63	0.56	0.35

Group 1:vehicle   Group 2:50 mg/kg   Group 3:200 mg/kg   Group 4:800 mg/kg

Group means without footnotes were not statistically different from the control at p=0.05 or p=0.01.
\+ The group mean was significantly different from the control at p=0.05 with Dunnett’s test of significance

Table 6
Day 29 Clinical Chemistry Data
Test period

Session 1 (Scheduled)
Fexinidazole

Study Number: 0504-2007

Parameter	Group Number:	Males 1	Males 2	Males 3	Males 4	Females 1	Females 2	Females 3	Females 4
PHOSPHOROUS	N	5	5	5	5	5	5	5	5
mg/dL	Mean	8.8	8.7	8.1+	11.4	6.7	6.7	7.3	6.6
	Sdev	0.18	0.61	0.33	2.90	0.42	0.44	0.53	0.52
ALBUMIN/GLOBULIN	N	5	5	5	5	5	5	5	5
	Mean	0.9	0.9	0.9	0.8	0.9	0.9	1.0	0.9
	Sdev	0.02	0.06	0.05	0.02	0.05	0.06	0.11	0.02
SODIUM	N	5	5	5	5	5	5	5	5
mEq/L	Mean	139.6	141.0+	141.6*	143.0*	139.2	140.8	142.4*	143.2*
	Sdev	0.55	0.71	1.14	0.71	0.84	2.05	0.55	1.30
POTASSIUM	N	5	5	5	5	5	5	5	5
mEq/L	Mean	4.8	5.0	4.8	5.1	4.7	4.8	4.8	4.9
	Sdev	0.17	0.18	0.36	0.33	0.14	0.32	0.19	0.20
CHLORIDE	N	5	5	5	5	5	5	5	5
mEq/L	Mean	102.6	102.2	103.4	103.4	102.2	103.0	103.6	104.6*
	Sdev	0.55	0.84	0.89	0.55	0.45	1.22	1.34	0.89

Group 1:vehicle   Group 2:50 mg/kg   Group 3:200 mg/kg   Group 4:800 mg/kg

Group means without footnotes were not statistically different from the control at p=0.05 or p=0.01.
+ The group mean was significantly different from the control at p=0.05 with Dunnett’s test of significance
* The group mean was significantly different from the control at p=0.01 with Dunnett’s test of significance

Table 6
Day 43 Clinical Chemistry Data
Test period

Session 1 (Scheduled)
Fexinidazole
Study Number: 0504-2007

Parameter	Group Number:	Males 1	Males 2	Males 3	Males 4	Females 1	Females 2	Females 3	Females 4
UREA	N	5	0	0	5	5	0	0	5
mg/dL	Mean	30.	-	-	32.	37.	-	-	39.
	Sdev	2.5	-	-	2.3	3.6	-	-	6.5
CREATININE	N	5	0	0	5	5	0	0	5
mg/dL	Mean	0.68	-	-	0.67	0.66	-	-	0.70
	Sdev	0.018	-	-	0.031	0.045	-	-	0.045
ASPARTATE AMINO TRANSFERASE	N	5	0	0	5	5	0	0	5
IU/L	Mean	149.	-	-	136.	136.	-	-	142.
	Sdev	20.1	-	-	23.8	27.1	-	-	23.7
ALANINE AMINO TRANSFERASE	N	5	0	0	5	5	0	0	5
IU/L	Mean	33.	-	-	32.	32.	-	-	28.
	Sdev	4.1	-	-	4.6	7.2	-	-	2.9
ALKALINE PHOSPHATASE	N	5	0	0	5	5	0	0	5
IU/L	Mean	145.	-	-	118.	87.	-	-	61.+
	Sdev	21.9	-	-	17.2	15.6	-	-	12.5
GAMMA GLUTAMYL TRANSFERASE	N	3	0	0	0	2	0	0	1
IU/L	Mean	6.	-	-	-	5.	-	-	7.
	Sdev	1.5	-	-	-	1.4	-	-	-
TOTAL BILIRUBIN	N	5	0	0	4	5	0	0	5
mg/dL	Mean	0.08	-	-	0.09	0.09	-	-	0.10
	Sdev	0.015	-	-	0.014	0.013	-	-	0.020

Group 1:vehicle   Group 2:50 mg/kg   Group 3:200 mg/kg   Group 4:800 mg/kg

Group means without footnotes were not statistically different from the control at p=0.05 or p=0.01.
\+ The group mean was significantly different from the control at p=0.05 with Dunnett’s test of significance

Table 6
Day 43 Clinical Chemistry Data
Test period

Session 1 (Scheduled)
Fexinidazole
Study Number: 0504-2007

Parameter	Group Number:	Males 1	Males 2	Males 3	Males 4	Females 1	Females 2	Females 3	Females 4
TOTAL PROTEIN	N	5	0	0	5	5	0	0	5
g/dL	Mean	6.9	-	-	7.0	7.5	-	-	7.7
	Sdev	0.24	-	-	0.19	0.25	-	-	0.23
ALBUMIN	N	5	0	0	5	5	0	0	5
g/dL	Mean	3.07	-	-	3.11	3.56	-	-	3.67
	Sdev	0.059	-	-	0.035	0.187	-	-	0.224
GLOBULIN	N	5	0	0	5	5	0	0	5
g/dL	Mean	3.9	-	-	3.9	3.9	-	-	4.0
	Sdev	0.21	-	-	0.19	0.15	-	-	0.12
GLUCOSE	N	5	0	0	5	5	0	0	5
mg/dL	Mean	91.	-	-	83.+	105.	-	-	104.
	Sdev	5.0	-	-	4.5	7.9	-	-	6.8
TRIGLYCERIDES	N	5	0	0	5	5	0	0	5
mg/dL	Mean	53.	-	-	60.	39.	-	-	42.
	Sdev	16.3	-	-	18.0	8.9	-	-	4.3
TOTAL CHOLESTEROL	N	5	0	0	5	5	0	0	5
mg/dL	Mean	51.	-	-	60.	72.	-	-	87.
	Sdev	5.8	-	-	13.8	14.3	-	-	12.3
CALCIUM	N	5	0	0	5	5	0	0	5
mg/dL	Mean	10.3	-	-	10.4	10.7	-	-	11.1
	Sdev	0.15	-	-	0.24	0.15	-	-	0.36

Group 1:vehicle   Group 2:50 mg/kg   Group 3:200 mg/kg   Group 4:800 mg/kg

Group means without footnotes were not statistically different from the control at p=0.05 or p=0.01.
\+ The group mean was significantly different from the control at p=0.05 with Dunnett’s test of significance

Table 6
Day 43 Clinical Chemistry Data
Test period

Session 1 (Scheduled)
Fexinidazole

Study Number: 0504-2007

Parameter	Group Number:	Males 1	Males 2	Males 3	Males 4	Females 1	Females 2	Females 3	Females 4
PHOSPHOROUS	N	5	0	0	5	5	0	0	5
mg/dL	Mean	8.1	-	-	8.5	6.5	-	-	6.6
	Sdev	0.49	-	-	0.85	0.47	-	-	0.58
ALBUMIN/GLOBULIN	N	5	0	0	5	5	0	0	5
	Mean	0.8	-	-	0.8	0.9	-	-	0.9
	Sdev	0.04	-	-	0.04	0.05	-	-	0.07
SODIUM	N	5	0	0	5	5	0	0	5
mEq/L	Mean	139.0	-	-	140.8*	143.0	-	-	143.2
	Sdev	0.71	-	-	0.84	1.00	-	-	1.64
POTASSIUM	N	5	0	0	5	5	0	0	5
mEq/L	Mean	5.0	-	-	5.0	4.8	-	-	4.9
	Sdev	0.31	-	-	0.10	0.24	-	-	0.24
CHLORIDE	N	5	0	0	5	5	0	0	5
mEq/L	Mean	101.8	-	-	102.6	104.4	-	-	104.6
	Sdev	0.84	-	-	0.89	0.89	-	-	0.89

Group 1:vehicle   Group 2:50 mg/kg   Group 3:200 mg/kg   Group 4:800 mg/kg

Group means without footnotes were not statistically different from the control at p=0.05 or p=0.01.
* The group mean was significantly different from the control at p=0.01 with Dunnett’s test of significance

Table 7 Urine Analysis - Quantitative

Table 7
Day 29 Urine Data
Test period

Session 1 (Scheduled)
Fexinidazole

Study Number: 0504-2007

Parameter	Group Number:	Males 1	Males 2	Males 3	Males 4	Females 1	Females 2	Females 3	Females 4
PH	N	5	5	5	5	5	5	5	5
UNITS	Mean	7.0	7.0	7.0	7.0	6.9	7.0	7.0	7.0
	Sdev	0.00	0.00	0.00	0.00	0.22	0.00	0.00	0.00
SPECIFIC GRAVITY	N	5	5	5	5	5	5	5	5
	Mean	1.020	1.020	1.020	1.021	1.020	1.019	1.021	1.017
	Sdev	0.0036	0.0020	0.0011	0.0013	0.0036	0.0030	0.0011	0.0015
URINARY VOLUME	N	5	5	5	5	5	5	5	5
mL	Mean	10.6	12.4	12.2	11.4	8.6	8.0	7.6	9.2
	Sdev	1.67	1.14	2.95	2.19	1.95	1.41	1.52	2.17

Group 1:vehicle Group 2:50 mg/kg Group 3:200 mg/kg Group 4:800 mg/kg

Group means without footnotes were not statistically different from the control at p=0.05 or p=0.01.

Table 7
Day 43 Urine Data
Test period

Session 1 (Scheduled)
Fexinidazole
Study Number: 0504-2007

Parameter	Group Number:	Males 1	Males 2	Males 3	Males 4	Females 1	Females 2	Females 3	Females 4
PH	N	5	0	0	5	5	0	0	5
UNITS	Mean	7.0	-	-	7.0	7.0	-	-	7.0
	Sdev	0.00	-	-	0.00	0.00	-	-	0.00
SPECIFIC GRAVITY	N	5	0	0	5	5	0	0	5
	Mean	1.019	-	-	1.019	1.017	-	-	1.018
	Sdev	0.0015	-	-	0.0023	0.0020	-	-	0.0021
URINARY VOLUME	N	5	0	0	5	5	0	0	5
mL	Mean	11.4	-	-	10.2	9.8	-	-	6.6
	Sdev	0.89	-	-	1.10	1.10	-	-	3.21

Group 1:vehicle Group 2:50 mg/kg Group 3:200 mg/kg Group 4:800 mg/kg

Group means without footnotes were not statistically different from the control at p=0.05 or p=0.01.

Table 8 Urine Analysis - Macroscopic

Table 8
Day 29 Urine Data
Test period

Session 1 (Scheduled)
Fexinidazole

Study Number: 0504-2007

Parameter	Group Number:	Males 1	Males 2	Males 3	Males 4	Females 1	Females 2	Females 3	Females 4
WHITE BLOOD CELLS	N	5	5	5	5	5	5	5	5
SCORE	Mean	0.	0.	0.	1.	0.	0.	0.	0.
	Sdev	0.0	0.4	0.0	0.4	0.0	0.0	0.0	0.0
NITRITES	N	5	5	5	5	5	5	5	5
SCORE	Mean	0.	0.	0.	1.	0.	0.	0.	0.
	Sdev	0.0	0.4	0.4	0.5	0.0	0.0	0.0	0.5
PROTEINS	N	5	5	5	5	5	5	5	5
SCORE	Mean	1.	1.	1.	1.	0.	0.	1.	0.
	Sdev	0.5	0.0	0.0	0.0	0.4	0.4	0.4	0.4
GLUCOSE	N	5	5	5	5	5	5	5	5
SCORE	Mean	0.	0.	0.	0.	0.	0.	0.	0.
	Sdev	0.0	0.0	0.0	0.0	0.0	0.0	0.0	0.0
KETONE BODIES	N	5	5	5	5	5	5	5	5
SCORE	Mean	0.	0.	1.	1.	0.	0.	0.	0.
	Sdev	0.4	0.5	0.5	0.5	0.0	0.0	0.0	0.0
UROBILINOGEN	N	5	5	5	5	5	5	5	5
SCORE	Mean	0.	0.	0.	0.	0.	0.	0.	0.
	Sdev	0.0	0.0	0.0	0.0	0.0	0.0	0.0	0.0
BILIRUBIN	N	5	5	5	5	5	5	5	5
SCORE	Mean	0.	0.	0.	0.	0.	0.	0.	0.
	Sdev	0.0	0.0	0.0	0.0	0.0	0.0	0.0	0.0
HEMOGLOBIN/RED BLOOD CE/+	N	5	5	5	5	5	5	5	5
SCORE	Mean	1.	0.	0.	0.	0.	0.	0.	0.
	Sdev	0.5	0.0	0.4	0.4	0.4	0.4	0.0	0.0

Group 1:vehicle   Group 2:50 mg/kg   Group 3:200 mg/kg   Group 4:800 mg/kg

Table 8
Day 43 Urine Data
Session 1 (Scheduled) Test period
Fexinidazole Study Number: 0504-2007

Parameter	Group Number:	Males 1	Males 2	Males 3	Males 4	Females 1	Females 2	Females 3	Females 4
WHITE BLOOD CELLS	N	5	0	0	5	5	0	0	5
SCORE	Mean	0.	-	-	0.	0.	-	-	0.
	Sdev	0.0	-	-	0.0	0.0	-	-	0.0
NITRITES	N	5	0	0	5	5	0	0	5
SCORE	Mean	0.	-	-	0.	0.	-	-	0.
	Sdev	0.0	-	-	0.0	0.0	-	-	0.0
PROTEINS	N	5	0	0	5	5	0	0	5
SCORE	Mean	1.	-	-	1.	0.	-	-	0.
	Sdev	0.4	-	-	0.0	0.0	-	-	0.0
GLUCOSE	N	5	0	0	5	5	0	0	5
SCORE	Mean	0.	-	-	0.	0.	-	-	0.
	Sdev	0.0	-	-	0.0	0.0	-	-	0.0
KETONE BODIES	N	5	0	0	5	5	0	0	5
SCORE	Mean	0.	-	-	1.	0.	-	-	0.
	Sdev	0.0	-	-	0.5	0.0	-	-	0.0
UROBILINOGEN	N	5	0	0	5	5	0	0	5
SCORE	Mean	0.	-	-	0.	0.	-	-	0.
	Sdev	0.0	-	-	0.0	0.0	-	-	0.0
BILIRUBIN	N	5	0	0	5	5	0	0	5
SCORE	Mean	0.	-	-	0.	0.	-	-	0.
	Sdev	0.0	-	-	0.0	0.0	-	-	0.0
HEMOGLOBIN/RED BLOOD CE/+	N	5	0	0	5	5	0	0	5
SCORE	Mean	0.	-	-	0.	0.	-	-	0.
	Sdev	0.4	-	-	0.0	0.0	-	-	0.5

Group 1:vehicle Group 2:50 mg/kg Group 3:200 mg/kg Group 4:800 mg/kg

Table 9 Absolute Organ Weights

Table 9
Absolute Organ Weights (g)
Test period
Days 29 Interim Sacrifice

Fexinidazole

Study Number: 0504-2007

Group Number	Dose Level		Terminal Body Wt (g)	SPLEEN	KIDNEYS	LIVER	THYMUS	HEART
				Males				
1	vehicle							
		N	10	10	10	10	10	10
		Mean	391.53	0.87	2.92	10.73	0.542	1.44
		Sdev	22.597	0.104	0.203	1.360	0.1066	0.159
2	50 mg/kg							
		N	10	10	10	10	10	10
		Mean	374.45	0.78	2.75	11.26	0.575	1.27+
		Sdev	37.492	0.122	0.225	1.122	0.0868	0.109
3	200 mg/kg							
		N	10	10	10	10	10	10
		Mean	356.36	0.78	2.74	11.01	0.495	1.23*
		Sdev	40.429	0.143	0.274	1.464	0.1062	0.153
4	800 mg/kg							
		N	10	10	10	10	10	10
		Mean	343.74	0.70+	2.57*	11.35	0.436+	1.16*
		Sdev	35.627	0.163	0.283	1.045	0.0824	0.162

Note: Data collected using grace days.
+ The group mean was significantly different from the control at p=0.05.
* The group mean was significantly different from the control at p=0.01.

Table 9
Absolute Organ Weights (g)
Test period
Days 29 Interim Sacrifice

Fexinidazole | Study Number: 0504-2007

Group Number	Dose Level		Terminal Body Wt (g)	BRAIN	TESTES	PROSTATE	ADRENALS
			M a l e s				
1	vehicle						
		N	10	10	10	10	10
		Mean	391.53	2.08	3.49	0.62	0.062
		Sdev	22.597	0.099	0.188	0.114	0.0102
2	50 mg/kg						
		N	10	10	10	10	10
		Mean	374.45	2.02	3.38	0.54	0.061
		Sdev	37.492	0.085	0.209	0.118	0.0083
3	200 mg/kg						
		N	10	10	10	10	10
		Mean	356.36	1.98	3.32	0.55	0.064
		Sdev	40.429	0.074	0.287	0.120	0.0095
4	800 mg/kg						
		N	10	10	10	10	10
		Mean	343.74	2.04	3.45	0.58	0.063
		Sdev	35.627	0.133	0.331	0.107	0.0106

Note: Data collected using grace days.

Table 9
Absolute Organ Weights (g)
Test period
Days 29 Interim Sacrifice

Fexinidazole | Study Number: 0504-2007

Group Number	Dose Level		Terminal Body Wt (g)	SPLEEN	KIDNEYS	LIVER	THYMUS
			**Females**				
1	vehicle						
		N	10	10	10	10	10
		Mean	222.80	0.57	1.61	6.02	0.336
		Sdev	12.387	0.075	0.170	0.604	0.0615
2	50 mg/kg						
		N	10	10	10	10	10
		Mean	227.81	0.68*	1.66	6.65	0.377
		Sdev	11.403	0.104	0.130	0.715	0.0696
3	200 mg/kg						
		N	10	10	10	10	10
		Mean	228.82	0.57	1.70	6.80	0.346
		Sdev	9.799	0.067	0.119	0.505	0.0558
4	800 mg/kg						
		N	9	9	9	9	9
		Mean	218.81	0.53	1.69	8.17*	0.317
		Sdev	14.739	0.058	0.175	1.221	0.0696

Note: Data collected using grace days.
* The group mean was significantly different from the control at p=0.01.

Table 9
Absolute Organ Weights (g)
Test period
Days 29 Interim Sacrifice

Fexinidazole Study Number: 0504-2007

Group Number	Dose Level		Terminal Body Wt (g)	HEART	BRAIN	OVARIES	ADRENALS
			**Females**				
1	vehicle						
		N	10	10	10	10	10
		Mean	222.80	0.82	1.91	0.120	0.074
		Sdev	12.387	0.050	0.111	0.0161	0.0085
2	50 mg/kg						
		N	10	10	10	10	10
		Mean	227.81	0.86	1.92	0.124	0.074
		Sdev	11.403	0.044	0.084	0.0182	0.0074
3	200 mg/kg						
		N	10	10	10	10	10
		Mean	228.82	0.81	1.87	0.112	0.071
		Sdev	9.799	0.064	0.077	0.0169	0.0119
4	800 mg/kg						
		N	9	9	9	9	9
		Mean	218.81	0.80	1.87	0.119	0.069
		Sdev	14.739	0.071	0.059	0.0154	0.0059

Note: Data collected using grace days.

Table 9
Absolute Organ Weights (g)
Test period
Days 43 Final Sacrifice

Fexinidazole

Study Number: 0504-2007

Group Number	Dose Level		Terminal Body Wt (g)	SPLEEN	KIDNEYS	LIVER	THYMUS	HEART
				Males				
1	vehicle							
		N	5	5	5	5	5	5
		Mean	406.98	0.63	2.88	10.44	0.470	1.40
		Sdev	41.035	0.101	0.387	1.586	0.0255	0.108
4	800 mg/kg							
		N	5	5	5	5	5	5
		Mean	372.50	0.70	2.68	10.46	0.410	1.33
		Sdev	25.037	0.085	0.215	1.135	0.0941	0.081

Note: Data collected using grace days.

Table 9
Absolute Organ Weights (g)
Test period
Days 43 Final Sacrifice

Fexinidazole | Study Number: 0504-2007

Group Number	Dose Level		Terminal Body Wt (g)	BRAIN	TESTES	PROSTATE	ADRENALS
			Males				
1	vehicle						
		N	5	5	5	5	5
		Mean	406.98	2.02	3.40	0.59	0.057
		Sdev	41.035	0.077	0.264	0.158	0.0123
4	800 mg/kg						
		N	5	5	5	5	5
		Mean	372.50	2.08	3.54	0.64	0.062
		Sdev	25.037	0.020	0.333	0.071	0.0078

Note: Data collected using grace days.

Table 9
Absolute Organ Weights (g)
Test period
Days 43 Final Sacrifice

Fexinidazole   Study Number: 0504-2007

Group Number	Dose Level		Terminal Body Wt (g)	SPLEEN	KIDNEYS	LIVER	THYMUS
				Females			
1	vehicle						
		N	5	5	5	5	5
		Mean	239.42	0.52	1.76	6.38	0.302
		Sdev	12.405	0.018	0.150	0.220	0.0626
4	800 mg/kg						
		N	5	5	5	5	5
		Mean	224.54	0.52	1.55+	6.51	0.300
		Sdev	15.046	0.156	0.114	0.437	0.0693

Note: Data collected using grace days.
+ The group mean was significantly different from the control at p=0.05.

Table 9
Absolute Organ Weights (g)
Test period
Days 43 Final Sacrifice

Fexinidazole | Study Number: 0504-2007

Group Number	Dose Level		Terminal Body Wt (g)	HEART	BRAIN	OVARIES	ADRENALS
				Females			
1	vehicle						
		N	5	5	5	5	5
		Mean	239.42	0.89	1.98	0.103	0.074
		Sdev	12.405	0.044	0.100	0.0148	0.0075
4	800 mg/kg						
		N	5	5	5	5	5
		Mean	224.54	0.84	1.85+	0.092	0.060*
		Sdev	15.046	0.063	0.063	0.0048	0.0040

Note: Data collected using grace days.
+ The group mean was significantly different from the control at p=0.05.
* The group mean was significantly different from the control at p=0.01.

Table 10 Relative Organ Weights

Table 10
Relative Organ Weights (% Body Weight)
Test period
Days 29 Interim Sacrifice

Fexinidazole

Study Number: 0504-2007

Group Number	Dose Level		Terminal Body Wt (g)	SPLEEN	KIDNEYS	LIVER	THYMUS	HEART
					Males			
1	vehicle							
		N	10	10	10	10	10	10
		Mean	391.53	0.22	0.75	2.74	0.139	0.37
		Sdev	22.597	0.028	0.059	0.263	0.0304	0.045
2	50 mg/kg							
		N	10	10	10	10	10	10
		Mean	374.45	0.21	0.74	3.01	0.154	0.34
		Sdev	37.492	0.034	0.065	0.180	0.0242	0.033
3	200 mg/kg							
		N	10	10	10	10	10	10
		Mean	356.36	0.22	0.77	3.09	0.139	0.35
		Sdev	40.429	0.037	0.054	0.188	0.0270	0.023
4	800 mg/kg							
		N	10	10	10	10	10	10
		Mean	343.74	0.20	0.75	3.32	0.127	0.34
		Sdev	35.627	0.042	0.053	0.336	0.0202	0.029

Note: Data collected using grace days.

Table 10
Relative Organ Weights (% Body Weight)
Test period
Days 29 Interim Sacrifice

Fexinidazole

Study Number: 0504-2007

Group Number	Dose Level		Terminal Body Wt (g)	BRAIN	TESTES	PROSTATE	ADRENALS
				Males			
1	vehicle						
		N	10	10	10	10	10
		Mean	391.53	0.53	0.89	0.16	0.016
		Sdev	22.597	0.037	0.066	0.035	0.0022
2	50 mg/kg						
		N	10	10	10	10	10
		Mean	374.45	0.54	0.91	0.14	0.016
		Sdev	37.492	0.044	0.082	0.027	0.0022
3	200 mg/kg						
		N	10	10	10	10	10
		Mean	356.36	0.56	0.94	0.15	0.018
		Sdev	40.429	0.075	0.070	0.035	0.0023
4	800 mg/kg						
		N	10	10	10	10	10
		Mean	343.74	0.60	1.01	0.17	0.019
		Sdev	35.627	0.037	0.113	0.031	0.0033

Note: Data collected using grace days.

Table 10
Relative Organ Weights (% Body Weight)
Test period
Days 29 Interim Sacrifice

Fexinidazole — Study Number: 0504-2007

Group Number	Dose Level		Terminal Body Wt (g)	SPLEEN	KIDNEYS	LIVER	THYMUS
				Females			
1	vehicle						
		N	10	10	10	10	10
		Mean	222.80	0.26	0.72	2.70	0.151
		Sdev	12.387	0.035	0.059	0.168	0.0298
2	50 mg/kg						
		N	10	10	10	10	10
		Mean	227.81	0.30	0.73	2.91	0.166
		Sdev	11.403	0.043	0.055	0.230	0.0335
3	200 mg/kg						
		N	10	10	10	10	10
		Mean	228.82	0.25	0.74	2.97	0.152
		Sdev	9.799	0.029	0.033	0.172	0.0287
4	800 mg/kg						
		N	9	9	9	9	9
		Mean	218.81	0.24	0.77	3.73	0.144
		Sdev	14.739	0.026	0.098	0.522	0.0249

Note: Data collected using grace days.

Table 10
Relative Organ Weights (% Body Weight)
Test period
Days 29 Interim Sacrifice

Fexinidazole — Study Number: 0504-2007

Group Number	Dose Level		Terminal Body Wt (g)	HEART	BRAIN	OVARIES	ADRENALS
				Females			
1	vehicle						
		N	10	10	10	10	10
		Mean	222.80	0.37	0.86	0.054	0.033
		Sdev	12.387	0.019	0.056	0.0069	0.0036
2	50 mg/kg						
		N	10	10	10	10	10
		Mean	227.81	0.38	0.84	0.055	0.032
		Sdev	11.403	0.024	0.050	0.0083	0.0030
3	200 mg/kg						
		N	10	10	10	10	10
		Mean	228.82	0.35	0.82	0.049	0.031
		Sdev	9.799	0.028	0.038	0.0062	0.0047
4	800 mg/kg						
		N	9	9	9	9	9
		Mean	218.81	0.36	0.86	0.055	0.031
		Sdev	14.739	0.024	0.066	0.0059	0.0012

Note: Data collected using grace days.

Table 10
Relative Organ Weights (% Body Weight)
Test period
Days 43 Final Sacrifice

Fexinidazole

Study Number: 0504-2007

Group Number	Dose Level		Terminal Body Wt (g)	SPLEEN	KIDNEYS	LIVER	THYMUS	HEART
				Males				
1	vehicle							
		N	5	5	5	5	5	5
		Mean	406.98	0.16	0.72	2.59	0.117	0.35
		Sdev	41.035	0.030	0.140	0.526	0.0168	0.046
4	800 mg/kg							
		N	5	5	5	5	5	5
		Mean	372.50	0.19	0.72	2.81	0.110	0.36
		Sdev	25.037	0.029	0.016	0.310	0.0246	0.026

Note: Data collected using grace days.

Table 10
Relative Organ Weights (% Body Weight)
Test period
Days 43 Final Sacrifice

Fexinidazole

Study Number: 0504-2007

Group Number	Dose Level		Terminal Body Wt (g)	BRAIN	TESTES	PROSTATE	ADRENALS
				**Males**			
1	vehicle						
		N	5	5	5	5	5
		Mean	406.98	0.50	0.84	0.15	0.014
		Sdev	41.035	0.065	0.099	0.046	0.0040
4	800 mg/kg						
		N	5	5	5	5	5
		Mean	372.50	0.56	0.95	0.17	0.017
		Sdev	25.037	0.042	0.107	0.023	0.0024

Note: Data collected using grace days.

Table 10
Relative Organ Weights (% Body Weight)
Test period
Days 43 Final Sacrifice

Fexinidazole | Study Number: 0504-2007

Group Number	Dose Level		Terminal Body Wt (g)	SPLEEN	KIDNEYS	LIVER	THYMUS
				Females			
1	vehicle						
		N	5	5	5	5	5
		Mean	239.42	0.22	0.74	2.67	0.126
		Sdev	12.405	0.007	0.061	0.164	0.0261
4	800 mg/kg						
		N	5	5	5	5	5
		Mean	224.54	0.23	0.69	2.90	0.134
		Sdev	15.046	0.054	0.052	0.120	0.0303

Note: Data collected using grace days.

Table 10
Relative Organ Weights (% Body Weight)
Test period
Days 43 Final Sacrifice

Fexinidazole | Study Number: 0504-2007

Group Number	Dose Level		Terminal Body Wt (g)	HEART	BRAIN	OVARIES	ADRENALS
				Females			
1	vehicle						
		N	5	5	5	5	5
		Mean	239.42	0.37	0.83	0.043	0.031
		Sdev	12.405	0.014	0.067	0.0073	0.0044
4	800 mg/kg						
		N	5	5	5	5	5
		Mean	224.54	0.37	0.83	0.041	0.027
		Sdev	15.046	0.028	0.070	0.0022	0.0025

Note: Data collected using grace days.

Table 11 Gross Necropsy Observations

Table 11
Incidence Summary for Gross Necropsy Observations
Test period
Days 29 Interim Sacrifice

Fexinidazole | Study Number: 0504-2007

	Males				Females			
Group Number:	1	2	3	4	1	2	3	4
Number in Group:	10	10	10	10	10	10	10	9
GENER. CONDITION								
GOOD	10	10	10	10	10	10	10	9
PLEURAL CAVITY								
ABNORMAL CONTENTS	0	0	0	0	0	0	1	0
SKIN								
ALOPECIA	0	0	0	0	0	0	0	1
ENCRUSTED AREA(S)	0	0	2	0	0	0	1	0

Note: The necropsy was conducted over multiple days.
Group 1:vehicle Group 2:50 mg/kg Group 3:200 mg/kg Group 4:800 mg/kg

Table 11
Incidence Summary for Gross Necropsy Observations
Test period
Days 43 Final Sacrifice

Fexinidazole Study Number: 0504-2007

	Males				Females			
Group Number:	1	2	3	4	1	2	3	4
Number in Group:	5	0	0	5	5	0	0	5
GENER. CONDITION								
GOOD	5	0	0	5	5	0	0	5
SPLEEN								
ENLARGED	0	0	0	0	0	0	0	1
STOMACH								
DARK GLANDULAR MUCOSA	0	0	0	0	1	0	0	0

Note: The necropsy was conducted over multiple days.
Group 1:vehicle Group 2:50 mg/kg Group 3:200 mg/kg Group 4:800 mg/kg

Table 11
Incidence Summary for Gross Necropsy Observations
Test period
Unscheduled Sacrifices

Fexinidazole | Study Number: 0504-2007

	Males				Females			
Group Number:	1	2	3	4	1	2	3	4
Number in Group:	0	0	0	0	0	0	0	1
GENER. CONDITION								
FAIRLY GOOD	0	0	0	0	0	0	0	1
AUTOLYTIC CHANGES	0	0	0	0	0	0	0	1
PLEURAL CAVITY								
ABNORMAL CONTENTS	0	0	0	0	0	0	0	1

Note: The necropsy was conducted over multiple days.
Group 1:vehicle Group 2:50 mg/kg Group 3:200 mg/kg Group 4:800 mg/kg

Table 12 Microscopic Observations

Table 12
Expanded Incidence Summary for Microscopic Observations
Test period
Days 29 Interim Sacrifice

Fexinidazole   Study Number: 0504-2007

CONTROLS FROM GROUP(S): 1	ANIMAL SEX:	Males				Females			
	DOSAGE GROUP:	1	2	3	4	1	2	3	4
T I S S U E S W I T H D I A G N O S E S	NO. IN GROUP:	10	10	10	10	10	10	10	9
ADRENALS	NUMBER EXAMINED:	10	0	0	10	10	0	0	9
CORTICAL VACUOLATION									
	Nad>	8	0	0	8	9	0	0	9
	Minimal>	1	0	0	1	0	0	0	0
	Slight>	1	0	0	1	1	0	0	0
AORTA	NUMBER EXAMINED:	10	0	0	10	10	0	0	9
INFLAMMATION OF ADJACENT TISSUES									
	Nad>	9	0	0	10	10	0	0	9
	Minimal>	1	0	0	0	0	0	0	0
BONE MARROW	NUMBER EXAMINED:	10	0	0	10	10	0	0	9
SMEAR NOT SAMPLED									
	Nad>	10	0	0	10	10	0	0	9
BRAIN	NUMBER EXAMINED:	10	0	0	10	10	0	0	9
CECUM	NUMBER EXAMINED:	10	0	0	10	10	0	0	9
COLON	NUMBER EXAMINED:	10	0	0	10	10	0	0	9
DIAPHRAGM	NUMBER EXAMINED:	10	0	0	10	10	0	0	9
MYOSITIS									
	Nad>	10	0	0	10	10	0	0	9
PLEURITIS									
	Nad>	10	0	0	10	10	0	0	9
DUODENUM	NUMBER EXAMINED:	10	0	0	10	10	0	0	9
EPIDIDYMIDES	NUMBER EXAMINED:	10	0	0	10	0	0	0	0

Nad = No abnormalities detected   NOS = Not otherwise specified
Group 1:vehicle   Group 2:50 mg/kg   Group 3:200 mg/kg   Group 4:800 mg/kg

Table 12
Expanded Incidence Summary for Microscopic Observations
Test period
Days 29 Interim Sacrifice

Fexinidazole — Study Number: 0504-2007

CONTROLS FROM GROUP(S): 1	ANIMAL SEX:	Males				Females			
	DOSAGE GROUP:	1	2	3	4	1	2	3	4
T I S S U E S W I T H D I A G N O S E S	NO. IN GROUP:	10	10	10	10	10	10	10	9
EPIDIDYMIDES (Continued)	NUMBER EXAMINED:	10	0	0	10	0	0	0	0
LYMPHOCYTIC INFILTRATION									
	Nad>	9	0	0	10	0	0	0	0
	Minimal>	1	0	0	0	0	0	0	0
ESOPHAGUS	NUMBER EXAMINED:	10	0	0	10	10	0	0	9
EYES	NUMBER EXAMINED:	10	0	0	10	10	0	0	9
FEMUR	NUMBER EXAMINED:	10	0	0	10	10	0	0	9
HEART	NUMBER EXAMINED:	10	0	0	10	10	0	0	9
MYOCARDIAL INFLAMMATION									
	Nad>	7	0	0	8	9	0	0	8
	Minimal>	3	0	0	2	1	0	0	1
PERICARDIAL INFLAMMATION									
	Nad>	10	0	0	10	10	0	0	9
ILEUM	NUMBER EXAMINED:	10	0	0	10	10	0	0	9
JEJUNUM	NUMBER EXAMINED:	10	0	0	10	10	0	0	9
KIDNEYS	NUMBER EXAMINED:	10	0	0	10	10	0	0	9
CHRONIC INFLAMMATION									
	Nad>	7	0	0	8	8	0	0	6
	Minimal>	3	0	0	2	2	0	0	3
CORTICAL TUBULAR REGENERATIVE BASOPHILIA									
	Nad>	7	0	0	7	10	0	0	8
	Minimal>	3	0	0	3	0	0	0	1
MEDULLARY TUBULAR DILATATION									
	Nad>	10	0	0	10	9	0	0	8
	Moderate>	0	0	0	0	1	0	0	1

Nad = No abnormalities detected NOS = Not otherwise specified
Group 1:vehicle Group 2:50 mg/kg Group 3:200 mg/kg Group 4:800 mg/kg

Table 12
Expanded Incidence Summary for Microscopic Observations
Test period
Days 29 Interim Sacrifice

Fexinidazole — Study Number: 0504-2007

CONTROLS FROM GROUP(S): 1	ANIMAL SEX:	Males				Females			
	DOSAGE GROUP:	1	2	3	4	1	2	3	4
TISSUES WITH DIAGNOSES	NO. IN GROUP:	10	10	10	10	10	10	10	9
KIDNEYS (Continued)	NUMBER EXAMINED:	10	0	0	10	10	0	0	9
PELVIC DILATATION									
	Nad>	10	0	0	9	10	0	0	9
	Slight>	0	0	0	1	0	0	0	0
PYELITIS									
	Nad>	9	0	0	10	10	0	0	9
	Slight>	1	0	0	0	0	0	0	0
HARDERIAN GLANDS	NUMBER EXAMINED:	10	10	10	10	10	10	10	9
PORPHYRIN DEPOSITS									
	Nad>	4	1	0	0	1	0	0	0
	Minimal>	6	4	1	4	9	2	0	0
	Slight>	0	4	6	5	0	6	5	6
	Moderate>	0	1	3	1	0	2	5	3
HEMORRHAGE,UNILATERAL									
	Nad>	10	9	8	10	10	6	6	9
	Slight>	0	1	2	0	0	4	4	0
ADENITIS,UNILATERAL									
	Nad>	10	8	8	10	10	7	9	9
	Minimal>	0	1	0	0	0	0	0	0
	Slight>	0	1	2	0	0	2	1	0
	Moderate>	0	0	0	0	0	1	0	0
LYMPHOCYTIC INFILTRATION									
	Nad>	8	10	9	8	9	8	9	8
	Minimal>	2	0	1	2	1	2	1	1
LIVER	NUMBER EXAMINED:	10	10	10	10	10	10	10	9
CHRONIC INFLAMMATION									
	Minimal>	9	8	8	8	10	10	10	9
	Slight>	1	2	2	2	0	0	0	0

Nad = No abnormalities detected   NOS = Not otherwise specified
Group 1:vehicle   Group 2:50 mg/kg   Group 3:200 mg/kg   Group 4:800 mg/kg

Table 12
Expanded Incidence Summary for Microscopic Observations
Test period
Days 29 Interim Sacrifice

Fexinidazole — Study Number: 0504-2007

CONTROLS FROM GROUP(S): 1	ANIMAL SEX:	Males				Females			
	DOSAGE GROUP:	1	2	3	4	1	2	3	4
TISSUES WITH DIAGNOSES	NO. IN GROUP:	10	10	10	10	10	10	10	9
LIVER (Continued)	NUMBER EXAMINED:	10	10	10	10	10	10	10	9
HEPATOCELLULAR HYPERTROPHY,CENTRILOBULAR									
	Nad>	10	0	0	0	10	3	0	0
	Minimal>	0	7	7	0	0	7	9	0
	Slight>	0	3	3	10	0	0	1	9
HEPATOCELLULAR VACUOLATION,PERIPORTAL									
	Nad>	5	7	6	6	2	0	1	2
	Minimal>	5	3	4	4	3	7	4	3
	Slight>	0	0	0	0	5	3	5	4
HEPATOCELLULAR VACUOLATION,DIFFUSE									
	Nad>	9	8	10	9	10	10	10	9
	Minimal>	1	2	0	0	0	0	0	0
	Slight>	0	0	0	1	0	0	0	0
FOCAL NECROSIS,SUBCAPSULAR									
	Nad>	10	9	10	9	10	10	9	9
	Minimal>	0	1	0	1	0	0	1	0
MANDIBULAR L.N.	NUMBER EXAMINED:	10	0	0	10	10	0	0	9
LYMPHOID DEPLETION									
	Nad>	10	0	0	10	10	0	0	9
PLASMACYTOSIS									
	Nad>	2	0	0	0	0	0	0	1
	Slight>	3	0	0	4	3	0	0	1
	Moderate>	4	0	0	5	7	0	0	6
	Marked>	1	0	0	1	0	0	0	1
MESENTERIC L.N.	NUMBER EXAMINED:	10	0	0	10	10	0	0	9
LYMPHOID DEPLETION									
	Nad>	10	0	0	10	10	0	0	9
LUNG	NUMBER EXAMINED:	10	0	0	10	10	0	0	9

Nad = No abnormalities detected   NOS = Not otherwise specified
Group 1:vehicle   Group 2:50 mg/kg   Group 3:200 mg/kg   Group 4:800 mg/kg

Table 12
Expanded Incidence Summary for Microscopic Observations
Test period
Days 29 Interim Sacrifice

Fexinidazole | Study Number: 0504-2007

CONTROLS FROM GROUP(S): 1	ANIMAL SEX:	Males				Females			
	DOSAGE GROUP:	1	2	3	4	1	2	3	4
TISSUES WITH DIAGNOSES	NO. IN GROUP:	10	10	10	10	10	10	10	9
LUNG (Continued)	NUMBER EXAMINED:	10	0	0	10	10	0	0	9
ALVEOLAR HEMORRHAGE									
	Nad>	9	0	0	8	9	0	0	9
	Minimal>	1	0	0	0	1	0	0	0
	Slight>	0	0	0	2	0	0	0	0
ACUTE INFLAMMATION									
	Nad>	8	0	0	10	10	0	0	9
	Minimal>	1	0	0	0	0	0	0	0
	Slight>	1	0	0	0	0	0	0	0
ALVEOLAR MACROPHAGE INFILTRATION									
	Nad>	5	0	0	6	6	0	0	6
	Minimal>	4	0	0	4	4	0	0	3
	Slight>	1	0	0	0	0	0	0	0
PLEURITIS									
	Nad>	10	0	0	10	10	0	0	9
MAMMARY GLAND	NUMBER EXAMINED:	10	0	0	10	10	0	0	9
CERVICAL MAMMARY GLAND EXAMINED									
	Nad>	10	0	0	9	9	0	0	9
	Present>	0	0	0	1	1	0	0	0
NO MAMMARY TISSUE IN THE SECTION									
	Nad>	9	0	0	10	10	0	0	9
	Present>	1	0	0	0	0	0	0	0
SKELETAL MUSCLE	NUMBER EXAMINED:	10	0	0	10	10	0	0	9
CHRONIC INFLAMMATION									
	Nad>	9	0	0	10	9	0	0	9
	Minimal>	1	0	0	0	1	0	0	0
SCIATIC NERVE	NUMBER EXAMINED:	10	0	0	10	10	0	0	9
OPTIC NERVES	NUMBER EXAMINED:	9	0	0	10	10	0	0	9
ONLY ONE NERVE IS AVAILABLE FOR EXAMINATION									
	Nad>	8	0	0	8	7	0	0	4
	Present>	1	0	0	2	3	0	0	5

Nad = No abnormalities detected NOS = Not otherwise specified
Group 1:vehicle Group 2:50 mg/kg Group 3:200 mg/kg Group 4:800 mg/kg

Table 12
Expanded Incidence Summary for Microscopic Observations
Test period
Days 29 Interim Sacrifice

Fexinidazole — Study Number: 0504-2007

CONTROLS FROM GROUP(S): 1	ANIMAL SEX:	Males				Females			
	DOSAGE GROUP:	1	2	3	4	1	2	3	4
TISSUES WITH DIAGNOSES	NO. IN GROUP:	10	10	10	10	10	10	10	9
OVARIES	NUMBER EXAMINED:	0	0	0	0	10	0	0	9
PANCREAS	NUMBER EXAMINED:	10	0	0	10	10	0	0	9
CHRONIC INFLAMMATION									
	Nad>	9	0	0	10	9	0	0	9
	Minimal>	1	0	0	0	1	0	0	0
PITUITARY	NUMBER EXAMINED:	10	0	0	10	10	0	0	9
PROSTATE	NUMBER EXAMINED:	10	0	0	10	0	0	0	0
LYMPHOCYTIC INFILTRATION									
	Nad>	6	0	0	8	0	0	0	0
	Minimal>	3	0	0	2	0	0	0	0
	Slight>	1	0	0	0	0	0	0	0
PARATHYROIDS	NUMBER EXAMINED:	10	0	0	10	10	0	0	9
ONLY ONE PARATHYROID AVAILABLE FOR EXAMINATION									
	Nad>	5	0	0	3	4	0	0	5
	Present>	5	0	0	7	6	0	0	4
SPINAL CORD CERV.	NUMBER EXAMINED:	10	0	0	10	10	0	0	9
SPINAL CORD THOR.	NUMBER EXAMINED:	10	0	0	10	10	0	0	9
MANDIBULAR S.G.	NUMBER EXAMINED:	10	0	0	10	10	0	0	9
ACINAR HYPERTROPHY									
	Nad>	10	0	0	10	10	0	0	9
PAROTIDS	NUMBER EXAMINED:	10	0	0	10	10	0	0	9

Nad = No abnormalities detected NOS = Not otherwise specified
Group 1:vehicle Group 2:50 mg/kg Group 3:200 mg/kg Group 4:800 mg/kg

Table 12
Expanded Incidence Summary for Microscopic Observations
Test period
Days 29 Interim Sacrifice

Fexinidazole — Study Number: 0504-2007

CONTROLS FROM GROUP(S): 1	ANIMAL SEX:	Males				Females			
	DOSAGE GROUP:	1	2	3	4	1	2	3	4
TISSUES WITH DIAGNOSES	NO. IN GROUP:	10	10	10	10	10	10	10	9
PAROTIDS (Continued)	NUMBER EXAMINED:	10	0	0	10	10	0	0	9
ACINAR HYPERTROPHY									
	Nad>	10	0	0	10	10	0	0	9
STIFLE JOINT	NUMBER EXAMINED:	10	0	0	10	10	0	0	9
SKIN	NUMBER EXAMINED:	10	0	2	10	10	0	1	9
SCAB FORMATION									
	Nad>	10	0	0	10	10	0	1	9
	Slight>	0	0	1	0	0	0	0	0
	Moderate>	0	0	1	0	0	0	0	0
ACANTHOSIS									
	Nad>	10	0	0	10	10	0	1	9
	Slight>	0	0	2	0	0	0	0	0
EPIDERMAL/DERMAL INFLAMMATION									
	Nad>	10	0	0	10	10	0	1	9
	Slight>	0	0	2	0	0	0	0	0
SPLEEN	NUMBER EXAMINED:	10	0	0	10	10	0	0	9
LYMPHOID DEPLETION									
	Nad>	10	0	0	10	10	0	0	9
EXTRAMEDULLARY HEMOPOIESIS									
	Nad>	7	0	0	8	9	0	0	8
	Minimal>	3	0	0	2	1	0	0	1
INFLAMMATION OF THE CAPSULE									
	Nad>	9	0	0	9	10	0	0	9
	Minimal>	1	0	0	1	0	0	0	0
STOMACH	NUMBER EXAMINED:	10	0	0	10	10	0	0	9
EROSION OF GLANDULAR STOMACH									
	Nad>	10	0	0	10	10	0	0	9
STERNUM	NUMBER EXAMINED:	10	0	0	10	10	0	0	9
SEMINAL VESICLES	NUMBER EXAMINED:	10	0	0	10	0	0	0	0

Nad = No abnormalities detected   NOS = Not otherwise specified
Group 1:vehicle   Group 2:50 mg/kg   Group 3:200 mg/kg   Group 4:800 mg/kg

Table 12
Expanded Incidence Summary for Microscopic Observations
Test period
Days 29 Interim Sacrifice

Fexinidazole   Study Number: 0504-2007

CONTROLS FROM GROUP(S): 1	ANIMAL SEX:	Males				Females			
	DOSAGE GROUP:	1	2	3	4	1	2	3	4
TISSUES WITH DIAGNOSES	NO. IN GROUP:	10	10	10	10	10	10	10	9
TESTES	NUMBER EXAMINED:	10	0	0	10	0	0	0	0
THYROIDS	NUMBER EXAMINED:	10	0	0	10	10	0	0	9
ECTOPIC THYMUS									
	Nad>	9	0	0	10	9	0	0	9
	Present>	1	0	0	0	1	0	0	0
LYMPHOCYTIC INFILTRATION									
	Nad>	8	0	0	10	10	0	0	7
	Minimal>	2	0	0	0	0	0	0	2
COLLOID DEPLETION									
	Nad>	10	0	0	10	10	0	0	9
THYMUS	NUMBER EXAMINED:	10	0	0	10	10	0	0	9
LYMPHOID DEPLETION									
	Nad>	10	0	0	10	10	0	0	9
PLEURITIS									
	Nad>	10	0	0	10	10	0	0	9
TONGUE	NUMBER EXAMINED:	10	0	0	10	10	0	0	9
TRACHEA	NUMBER EXAMINED:	10	0	0	10	10	0	0	9
URINARY BLADDER	NUMBER EXAMINED:	10	0	0	10	10	0	0	9
PROTEINACEOUS PLUG									
	Nad>	9	0	0	10	10	0	0	9
	Present>	1	0	0	0	0	0	0	0
UTERUS	NUMBER EXAMINED:	0	0	0	0	10	0	0	9
VAGINA	NUMBER EXAMINED:	0	0	0	0	10	0	0	9

Nad = No abnormalities detected   NOS = Not otherwise specified
Group 1:vehicle   Group 2:50 mg/kg   Group 3:200 mg/kg   Group 4:800 mg/kg

Table 12
Expanded Incidence Summary for Microscopic Observations
Test period
Days 43 Final Sacrifice

Fexinidazole | Study Number: 0504-2007

CONTROLS FROM GROUP(S): 1	ANIMAL SEX:	Males				Females			
	DOSAGE GROUP:	1	2	3	4	1	2	3	4
TISSUES WITH DIAGNOSES	NO. IN GROUP:	5	0	0	5	5	0	0	5
HARDERIAN GLANDS.	NUMBER EXAMINED:	5	0	0	5	5	0	0	5
PORPHYRIN DEPOSITS									
	Minimal>	4	0	0	0	3	0	0	0
	Slight>	0	0	0	2	2	0	0	3
	Moderate>	1	0	0	3	0	0	0	2
HEMORRHAGE,UNILATERAL									
	Nad>	0	0	0	2	2	0	0	1
	Minimal>	0	0	0	1	1	0	0	2
	Slight>	4	0	0	1	2	0	0	1
	Moderate>	1	0	0	1	0	0	0	1
ADENITIS,UNILATERAL									
	Nad>	2	0	0	3	5	0	0	2
	Minimal>	1	0	0	0	0	0	0	2
	Slight>	2	0	0	2	0	0	0	1
LYMPHOCYTIC INFILTRATION									
	Nad>	3	0	0	5	4	0	0	5
	Minimal>	2	0	0	0	1	0	0	0
LIVER	NUMBER EXAMINED:	5	0	0	5	5	0	0	5
CHRONIC INFLAMMATION									
	Minimal>	5	0	0	5	5	0	0	5
HEPATOCELLULAR HYPERTROPHY,CENTRILOBULAR									
	Nad>	5	0	0	3	5	0	0	4
	Minimal>	0	0	0	2	0	0	0	1
HEPATOCELLULAR VACUOLATION,PERIPORTAL									
	Nad>	3	0	0	4	2	0	0	3
	Minimal>	2	0	0	1	2	0	0	2
	Slight>	0	0	0	0	1	0	0	0
HEPATOCELLULAR VACUOLATION,DIFFUSE									
	Nad>	4	0	0	5	5	0	0	5
	Minimal>	1	0	0	0	0	0	0	0

Nad = No abnormalities detected NOS = Not otherwise specified
Group 1:vehicle Group 2:50 mg/kg Group 3:200 mg/kg Group 4:800 mg/kg

Table 12
Expanded Incidence Summary for Microscopic Observations
Test period
Days 43 Final Sacrifice

Fexinidazole Study Number: 0504-2007

CONTROLS FROM GROUP(S): 1	ANIMAL SEX:	Males				Females			
	DOSAGE GROUP:	1	2	3	4	1	2	3	4
TISSUES WITH DIAGNOSES	NO. IN GROUP:	5	0	0	5	5	0	0	5
LIVER (Continued)	NUMBER EXAMINED:	5	0	0	5	5	0	0	5
FOCAL NECROSIS,SUBCAPSULAR									
	Nad>	5	0	0	5	4	0	0	4
	Minimal>	0	0	0	0	1	0	0	1
SPLEEN	NUMBER EXAMINED:	0	0	0	0	0	0	0	1
LYMPHOID DEPLETION									
	Nad>	0	0	0	0	0	0	0	1
EXTRAMEDULLARY HEMOPOIESIS									
	Nad>	0	0	0	0	0	0	0	1
INFLAMMATION OF THE CAPSULE									
	Nad>	0	0	0	0	0	0	0	1
STOMACH	NUMBER EXAMINED:	0	0	0	0	1	0	0	0
EROSION OF GLANDULAR STOMACH									
	Minimal>	0	0	0	0	1	0	0	0

Nad = No abnormalities detected NOS = Not otherwise specified
Group 1:vehicle Group 2:50 mg/kg Group 3:200 mg/kg Group 4:800 mg/kg

Table 12
Expanded Incidence Summary for Microscopic Observations
Test period
Unscheduled Sacrifices

Fexinidazole | Study Number: 0504-2007

CONTROLS FROM GROUP(S): 1	ANIMAL SEX:	Males				Females			
	DOSAGE GROUP:	1	2	3	4	1	2	3	4
TISSUES WITH DIAGNOSES	NO. IN GROUP:	0	0	0	0	0	0	0	1
ADRENALS	NUMBER EXAMINED:	0	0	0	0	0	0	0	1
CORTICAL VACUOLATION	Nad>	0	0	0	0	0	0	0	1
AORTA	NUMBER EXAMINED:	0	0	0	0	0	0	0	1
INFLAMMATION OF ADJACENT TISSUES	Moderate>	0	0	0	0	0	0	0	1
BONE MARROW	NUMBER EXAMINED:	0	0	0	0	0	0	0	1
SMEAR NOT SAMPLED	Present>	0	0	0	0	0	0	0	1
BRAIN	NUMBER EXAMINED:	0	0	0	0	0	0	0	1
CECUM	NUMBER EXAMINED:	0	0	0	0	0	0	0	1
COLON	NUMBER EXAMINED:	0	0	0	0	0	0	0	1
DIAPHRAGM	NUMBER EXAMINED:	0	0	0	0	0	0	0	1
MYOSITIS	Slight>	0	0	0	0	0	0	0	1
PLEURITIS	Slight>	0	0	0	0	0	0	0	1
DUODENUM	NUMBER EXAMINED:	0	0	0	0	0	0	0	1
EPIDIDYMIDES	NUMBER EXAMINED:	0	0	0	0	0	0	0	0
ESOPHAGUS	NUMBER EXAMINED:	0	0	0	0	0	0	0	1
EYES	NUMBER EXAMINED:	0	0	0	0	0	0	0	1
FEMUR	NUMBER EXAMINED:	0	0	0	0	0	0	0	1

Nad = No abnormalities detected NOS = Not otherwise specified
Group 1:vehicle Group 2:50 mg/kg Group 3:200 mg/kg Group 4:800 mg/kg

Table 12
Expanded Incidence Summary for Microscopic Observations
Test period
Unscheduled Sacrifices

Fexinidazole | Study Number: 0504-2007

CONTROLS FROM GROUP(S): 1	ANIMAL SEX:	Males				Females			
	DOSAGE GROUP:	1	2	3	4	1	2	3	4
TISSUES WITH DIAGNOSES	NO. IN GROUP:	0	0	0	0	0	0	0	1
HEART	NUMBER EXAMINED:	0	0	0	0	0	0	0	1
MYOCARDIAL INFLAMMATION	Nad>	0	0	0	0	0	0	0	1
PERICARDIAL INFLAMMATION	Slight>	0	0	0	0	0	0	0	1
ILEUM	NUMBER EXAMINED:	0	0	0	0	0	0	0	1
JEJUNUM	NUMBER EXAMINED:	0	0	0	0	0	0	0	0
KIDNEYS	NUMBER EXAMINED:	0	0	0	0	0	0	0	1
CHRONIC INFLAMMATION	Nad>	0	0	0	0	0	0	0	1
CORTICAL TUBULAR REGENERATIVE BASOPHILIA	Nad>	0	0	0	0	0	0	0	1
MEDULLARY TUBULAR DILATATION	Nad>	0	0	0	0	0	0	0	1
PELVIC DILATATION	Nad>	0	0	0	0	0	0	0	1
PYELITIS	Nad>	0	0	0	0	0	0	0	1
HARDERIAN GLANDS	NUMBER EXAMINED:	0	0	0	0	0	0	0	1
PORPHYRIN DEPOSITS	Marked>	0	0	0	0	0	0	0	1
HEMORRHAGE,UNILATERAL	Nad>	0	0	0	0	0	0	0	1
ADENITIS,UNILATERAL	Nad>	0	0	0	0	0	0	0	1

Nad = No abnormalities detected   NOS = Not otherwise specified
Group 1:vehicle   Group 2:50 mg/kg   Group 3:200 mg/kg   Group 4:800 mg/kg

Table 12
Expanded Incidence Summary for Microscopic Observations
Test period
Unscheduled Sacrifices

Fexinidazole — Study Number: 0504-2007

CONTROLS FROM GROUP(S): 1	ANIMAL SEX:	Males				Females			
	DOSAGE GROUP:	1	2	3	4	1	2	3	4
TISSUES WITH DIAGNOSES	NO. IN GROUP:	0	0	0	0	0	0	0	1
HARDERIAN GLANDS(Continued)	NUMBER EXAMINED:	0	0	0	0	0	0	0	1
LYMPHOCYTIC INFILTRATION									
	Nad>	0	0	0	0	0	0	0	1
LIVER	NUMBER EXAMINED:	0	0	0	0	0	0	0	1
CHRONIC INFLAMMATION									
	Nad>	0	0	0	0	0	0	0	1
HEPATOCELLULAR HYPERTROPHY,CENTRILOBULAR									
	Nad>	0	0	0	0	0	0	0	1
HEPATOCELLULAR VACUOLATION,PERIPORTAL									
	Nad>	0	0	0	0	0	0	0	1
HEPATOCELLULAR VACUOLATION,DIFFUSE									
	Nad>	0	0	0	0	0	0	0	1
FOCAL NECROSIS,SUBCAPSULAR									
	Nad>	0	0	0	0	0	0	0	1
MANDIBULAR L.N.	NUMBER EXAMINED:	0	0	0	0	0	0	0	1
LYMPHOID DEPLETION									
	Moderate>	0	0	0	0	0	0	0	1
PLASMACYTOSIS									
	Nad>	0	0	0	0	0	0	0	1
MESENTERIC L.N.	NUMBER EXAMINED:	0	0	0	0	0	0	0	1
LYMPHOID DEPLETION									
	Slight>	0	0	0	0	0	0	0	1
LUNG	NUMBER EXAMINED:	0	0	0	0	0	0	0	1
ALVEOLAR HEMORRHAGE									
	Nad>	0	0	0	0	0	0	0	1
ACUTE INFLAMMATION									
	Nad>	0	0	0	0	0	0	0	1

Nad = No abnormalities detected   NOS = Not otherwise specified
Group 1:vehicle   Group 2:50 mg/kg   Group 3:200 mg/kg   Group 4:800 mg/kg

Table 12
Expanded Incidence Summary for Microscopic Observations
Test period
Unscheduled Sacrifices

Fexinidazole — Study Number: 0504-2007

CONTROLS FROM GROUP(S): 1	ANIMAL SEX:	Males				Females			
	DOSAGE GROUP:	1	2	3	4	1	2	3	4
TISSUES WITH DIAGNOSES	NO. IN GROUP:	0	0	0	0	0	0	0	1
LUNG (Continued)	NUMBER EXAMINED:	0	0	0	0	0	0	0	1
ALVEOLAR MACROPHAGE INFILTRATION	Nad>	0	0	0	0	0	0	0	1
PLEURITIS	Moderate>	0	0	0	0	0	0	0	1
MAMMARY GLAND	NUMBER EXAMINED:	0	0	0	0	0	0	0	1
CERVICAL MAMMARY GLAND EXAMINED	Nad>	0	0	0	0	0	0	0	1
NO MAMMARY TISSUE IN THE SECTION	Nad>	0	0	0	0	0	0	0	1
SKELETAL MUSCLE	NUMBER EXAMINED:	0	0	0	0	0	0	0	1
CHRONIC INFLAMMATION	Nad>	0	0	0	0	0	0	0	1
SCIATIC NERVE	NUMBER EXAMINED:	0	0	0	0	0	0	0	1
OPTIC NERVES	NUMBER EXAMINED:	0	0	0	0	0	0	0	1
ONLY ONE NERVE IS AVAILABLE FOR EXAMINATION	Present>	0	0	0	0	0	0	0	1
OVARIES	NUMBER EXAMINED:	0	0	0	0	0	0	0	1
PANCREAS	NUMBER EXAMINED:	0	0	0	0	0	0	0	1
CHRONIC INFLAMMATION	Nad>	0	0	0	0	0	0	0	1
PITUITARY	NUMBER EXAMINED:	0	0	0	0	0	0	0	1
PROSTATE	NUMBER EXAMINED:	0	0	0	0	0	0	0	0
PARATHYROIDS	NUMBER EXAMINED:	0	0	0	0	0	0	0	1
ONLY ONE PARATHYROID AVAILABLE FOR EXAMINATION	Present>	0	0	0	0	0	0	0	1

Nad = No abnormalities detected   NOS = Not otherwise specified
Group 1:vehicle   Group 2:50 mg/kg   Group 3:200 mg/kg   Group 4:800 mg/kg

Table 12
Expanded Incidence Summary for Microscopic Observations
Test period
Unscheduled Sacrifices

Fexinidazole — Study Number: 0504-2007

CONTROLS FROM GROUP(S): 1

TISSUES WITH DIAGNOSES	ANIMAL SEX:	Males				Females			
	DOSAGE GROUP:	1	2	3	4	1	2	3	4
	NO. IN GROUP:	0	0	0	0	0	0	0	1
SPINAL CORD CERV.	NUMBER EXAMINED:	0	0	0	0	0	0	0	1
SPINAL CORD THOR.	NUMBER EXAMINED:	0	0	0	0	0	0	0	1
MANDIBULAR S.G.	NUMBER EXAMINED:	0	0	0	0	0	0	0	1
ACINAR HYPERTROPHY	Slight>	0	0	0	0	0	0	0	1
PAROTIDS	NUMBER EXAMINED:	0	0	0	0	0	0	0	1
ACINAR HYPERTROPHY	Slight>	0	0	0	0	0	0	0	1
STIFLE JOINT	NUMBER EXAMINED:	0	0	0	0	0	0	0	1
SKIN	NUMBER EXAMINED:	0	0	0	0	0	0	0	1
SCAB FORMATION	Nad>	0	0	0	0	0	0	0	1
ACANTHOSIS	Nad>	0	0	0	0	0	0	0	1
EPIDERMAL/DERMAL INFLAMMATION	Nad>	0	0	0	0	0	0	0	1
SPLEEN	NUMBER EXAMINED:	0	0	0	0	0	0	0	1
LYMPHOID DEPLETION	Moderate>	0	0	0	0	0	0	0	1
EXTRAMEDULLARY HEMOPOIESIS	Nad>	0	0	0	0	0	0	0	1
INFLAMMATION OF THE CAPSULE	Nad>	0	0	0	0	0	0	0	1
STOMACH	NUMBER EXAMINED:	0	0	0	0	0	0	0	1

Nad = No abnormalities detected   NOS = Not otherwise specified
Group 1:vehicle   Group 2:50 mg/kg   Group 3:200 mg/kg   Group 4:800 mg/kg

Table 12
Expanded Incidence Summary for Microscopic Observations
Test period
Unscheduled Sacrifices

Fexinidazole | Study Number: 0504-2007

CONTROLS FROM GROUP(S): 1	ANIMAL SEX:	Males				Females			
	DOSAGE GROUP:	1	2	3	4	1	2	3	4
T I S S U E S W I T H D I A G N O S E S	NO. IN GROUP:	0	0	0	0	0	0	0	1
STOMACH (Continued)	NUMBER EXAMINED:	0	0	0	0	0	0	0	1
EROSION OF GLANDULAR STOMACH	Nad>	0	0	0	0	0	0	0	1
STERNUM	NUMBER EXAMINED:	0	0	0	0	0	0	0	1
SEMINAL VESICLES	NUMBER EXAMINED:	0	0	0	0	0	0	0	0
TESTES	NUMBER EXAMINED:	0	0	0	0	0	0	0	0
THYROIDS	NUMBER EXAMINED:	0	0	0	0	0	0	0	1
ECTOPIC THYMUS	Nad>	0	0	0	0	0	0	0	1
LYMPHOCYTIC INFILTRATION	Nad>	0	0	0	0	0	0	0	1
COLLOID DEPLETION	Moderate>	0	0	0	0	0	0	0	1
THYMUS	NUMBER EXAMINED:	0	0	0	0	0	0	0	1
LYMPHOID DEPLETION	Moderate>	0	0	0	0	0	0	0	1
PLEURITIS	Moderate>	0	0	0	0	0	0	0	1
TONGUE	NUMBER EXAMINED:	0	0	0	0	0	0	0	1
TRACHEA	NUMBER EXAMINED:	0	0	0	0	0	0	0	1
URINARY BLADDER	NUMBER EXAMINED:	0	0	0	0	0	0	0	1
PROTEINACEOUS PLUG	Nad>	0	0	0	0	0	0	0	1
UTERUS	NUMBER EXAMINED:	0	0	0	0	0	0	0	1
VAGINA	NUMBER EXAMINED:	0	0	0	0	0	0	0	1

Nad = No abnormalities detected   NOS = Not otherwise specified
Group 1:vehicle   Group 2:50 mg/kg   Group 3:200 mg/kg   Group 4:800 mg/kg

APPENDICES

Appendix 1 Clinical Signs

Appendix 1
Individual Animal Clinical Signs

Fexinidazole Study Number: 0504-2007

M a l e s

Animal Number	Dose	Clinical Signs	Days Present	Study Day(s) Noted
2735	vehicle	Normal/no visible abnormalities	30	Test period 1-30
2736	vehicle	Normal/no visible abnormalities	30	Test period 1-30
2737	vehicle	Normal/no visible abnormalities	30	Test period 1-30
2738	vehicle	Normal/no visible abnormalities	30	Test period 1-30
2739	vehicle	Normal/no visible abnormalities	30	Test period 1-30
2740	vehicle	Normal/no visible abnormalities	29	Test period 1-29
2741	vehicle	Normal/no visible abnormalities	29	Test period 1-29
2742	vehicle	Normal/no visible abnormalities	29	Test period 1-29
2743	vehicle	Normal/no visible abnormalities	29	Test period 1-29
2744	vehicle	Normal/no visible abnormalities	29	Test period 1-29
2745	vehicle	Normal/no visible abnormalities	43	Test period 1-43
2746	vehicle	Normal/no visible abnormalities	43	Test period 1-43
2747	vehicle	Normal/no visible abnormalities	43	Test period 1-43
2748	vehicle	Normal/no visible abnormalities	43	Test period 1-43
2749	vehicle	Normal/no visible abnormalities	43	Test period 1-43
2750	50 mg/kg	Normal/no visible abnormalities	30	Test period 1-30
2751	50 mg/kg	Normal/no visible abnormalities	30	Test period 1-30

Appendix 1
Individual Animal Clinical Signs

Fexinidazole | Study Number: 0504-2007

M a l e s

Animal Number	Dose	Clinical Signs	Days Present	Study Day(s) Noted
2752	50 mg/kg	Normal/no visible abnormalities	30	Test period 1-30
2753	50 mg/kg	Normal/no visible abnormalities	30	Test period 1-30
2754	50 mg/kg	Normal/no visible abnormalities	30	Test period 1-30
2755	50 mg/kg	Normal/no visible abnormalities	29	Test period 1-29
2756	50 mg/kg	Normal/no visible abnormalities	29	Test period 1-29
2757	50 mg/kg	Normal/no visible abnormalities	29	Test period 1-29
2758	50 mg/kg	Normal/no visible abnormalities	29	Test period 1-29
2759	50 mg/kg	Normal/no visible abnormalities	29	Test period 1-29
2760	200 mg/kg	Normal/no visible abnormalities	30	Test period 1-30
2761	200 mg/kg	Normal/no visible abnormalities	30	Test period 1-30
2762	200 mg/kg	Normal/no visible abnormalities	30	Test period 1-30
2763	200 mg/kg	Normal/no visible abnormalities	20	Test period 1-20
		Focal alopecia, Neck	2	Test period 29-30
		Scabbed area, Neck	7	Test period 22-28
		Ulceration, Neck	1	Test period 21
2764	200 mg/kg	Normal/no visible abnormalities	28	Test period 1-28
		Focal alopecia, Head	2	Test period 29-30
2765	200 mg/kg	Normal/no visible abnormalities	29	Test period 1-29
2766	200 mg/kg	Normal/no visible abnormalities	29	Test period 1-29

Appendix 1
Individual Animal Clinical Signs

Fexinidazole

Study Number: 0504-2007

M a l e s

Animal Number	Dose	Clinical Signs	Days Present	Study Day(s) Noted
2767	200 mg/kg	Normal/no visible abnormalities	29	Test period 1-29
2768	200 mg/kg	Normal/no visible abnormalities	29	Test period 1-29
2769	200 mg/kg	Normal/no visible abnormalities	29	Test period 1-29
2770	800 mg/kg	Normal/no visible abnormalities	30	Test period 1-30
2771	800 mg/kg	Normal/no visible abnormalities	30	Test period 1-30
2772	800 mg/kg	Normal/no visible abnormalities	30	Test period 1-30
2773	800 mg/kg	Normal/no visible abnormalities	30	Test period 1-30
2774	800 mg/kg	Normal/no visible abnormalities	30	Test period 1-30
2775	800 mg/kg	Normal/no visible abnormalities	29	Test period 1-29
2776	800 mg/kg	Normal/no visible abnormalities	29	Test period 1-29
2777	800 mg/kg	Normal/no visible abnormalities	29	Test period 1-29
2778	800 mg/kg	Normal/no visible abnormalities	29	Test period 1-29
2779	800 mg/kg	Normal/no visible abnormalities	29	Test period 1-29
2780	800 mg/kg	Normal/no visible abnormalities	43	Test period 1-43
2781	800 mg/kg	Normal/no visible abnormalities	43	Test period 1-43
2782	800 mg/kg	Normal/no visible abnormalities	43	Test period 1-43
2783	800 mg/kg	Normal/no visible abnormalities	43	Test period 1-43

Appendix 1
Individual Animal Clinical Signs

Fexinidazole

Study Number: 0504-2007

M a l e s

Animal Number	Dose	Clinical Signs	Days Present	Study Day(s) Noted
2784	800 mg/kg	Normal/no visible abnormalities	43	Test period 1-43

Appendix 1
Individual Animal Clinical Signs

Fexinidazole Study Number: 0504-2007

F e m a l e

Animal Number	Dose	Clinical Signs	Days Present	Study Day(s) Noted
2785	vehicle	Normal/no visible abnormalities	30	Test period 1-30
2786	vehicle	Normal/no visible abnormalities	30	Test period 1-30
2787	vehicle	Normal/no visible abnormalities	30	Test period 1-30
2788	vehicle	Normal/no visible abnormalities	21	Test period 1-21
		Fur thinning, Back	9	Test period 22-30
2789	vehicle	Normal/no visible abnormalities	30	Test period 1-30
2790	vehicle	Normal/no visible abnormalities	29	Test period 1-29
2791	vehicle	Normal/no visible abnormalities	29	Test period 1-29
2792	vehicle	Normal/no visible abnormalities	29	Test period 1-29
2793	vehicle	Normal/no visible abnormalities	29	Test period 1-29
2794	vehicle	Normal/no visible abnormalities	29	Test period 1-29
2795	vehicle	Normal/no visible abnormalities	43	Test period 1-43
2796	vehicle	Normal/no visible abnormalities	43	Test period 1-43
2797	vehicle	Normal/no visible abnormalities	43	Test period 1-43
2798	vehicle	Normal/no visible abnormalities	43	Test period 1-43
2799	vehicle	Normal/no visible abnormalities	43	Test period 1-43
2800	50 mg/kg	Normal/no visible abnormalities	20	Test period 1-5,16-30

Appendix 1
Individual Animal Clinical Signs

Fexinidazole | Study Number: 0504-2007

F e m a l e

Animal Number	Dose	Clinical Signs	Days Present	Study Day(s) Noted
2800	50 mg/kg	Accidental wound, Forelimb/s	10	Test period 6-15
2801	50 mg/kg	Normal/no visible abnormalities	30	Test period 1-30
2802	50 mg/kg	Normal/no visible abnormalities	30	Test period 1-30
2803	50 mg/kg	Normal/no visible abnormalities	30	Test period 1-30
2804	50 mg/kg	Normal/no visible abnormalities	30	Test period 1-30
2805	50 mg/kg	Normal/no visible abnormalities	29	Test period 1-29
2806	50 mg/kg	Normal/no visible abnormalities	26	Test period 1-12,16-29
		Accidental wound, Forelimb/s	3	Test period 13-15
2807	50 mg/kg	Normal/no visible abnormalities	29	Test period 1-29
2808	50 mg/kg	Normal/no visible abnormalities	29	Test period 1-29
2809	50 mg/kg	Normal/no visible abnormalities	29	Test period 1-29
2810	200 mg/kg	Normal/no visible abnormalities	30	Test period 1-30
2811	200 mg/kg	Normal/no visible abnormalities	30	Test period 1-30
2812	200 mg/kg	Normal/no visible abnormalities	30	Test period 1-30
2813	200 mg/kg	Normal/no visible abnormalities	30	Test period 1-30
2814	200 mg/kg	Normal/no visible abnormalities	21	Test period 1-21
		Scabbed area, Cheek/s	9	Test period 22-30
2815	200 mg/kg	Normal/no visible abnormalities	29	Test period 1-29

Appendix 1
Individual Animal Clinical Signs

Fexinidazole | Study Number: 0504-2007

Female

Animal Number	Dose	Clinical Signs	Days Present	Study Day(s) Noted
2816	200 mg/kg	Normal/no visible abnormalities	29	Test period 1-29
2817	200 mg/kg	Normal/no visible abnormalities	29	Test period 1-29
2818	200 mg/kg	Normal/no visible abnormalities	29	Test period 1-29
2819	200 mg/kg	Normal/no visible abnormalities	29	Test period 1-29
2820	800 mg/kg	Normal/no visible abnormalities	30	Test period 1-30
2821	800 mg/kg	Normal/no visible abnormalities	30	Test period 1-30
2822	800 mg/kg	Normal/no visible abnormalities	9	Test period 1-9
		Focal alopecia, Forelimb/s	21	Test period 10-30
2823	800 mg/kg	Normal/no visible abnormalities	30	Test period 1-30
2824	800 mg/kg	Normal/no visible abnormalities	13	Test period 1-13
2825	800 mg/kg	Normal/no visible abnormalities	29	Test period 1-29
2826	800 mg/kg	Normal/no visible abnormalities	29	Test period 1-29
2827	800 mg/kg	Normal/no visible abnormalities	29	Test period 1-29
2828	800 mg/kg	Normal/no visible abnormalities	29	Test period 1-29
2829	800 mg/kg	Normal/no visible abnormalities	29	Test period 1-29
2830	800 mg/kg	Normal/no visible abnormalities	43	Test period 1-43
2831	800 mg/kg	Normal/no visible abnormalities	43	Test period 1-43

Appendix 1
Individual Animal Clinical Signs

Fexinidazole | Study Number: 0504-2007

F e m a l e

Animal Number	Dose	Clinical Signs	Days Present	Study Day(s) Noted
2832	800 mg/kg	Normal/no visible abnormalities	43	Test period 1-43
2833	800 mg/kg	Normal/no visible abnormalities	43	Test period 1-43
2834	800 mg/kg	Normal/no visible abnormalities	43	Test period 1-43

Appendix 2 Body Weights

Appendix 2
Body Weights (g)

Fexinidazole | Study Number: 0504-2007

Males

Dose Level	Animal Number	Group/ Subgroup	Study Day -4"	1#	8	14	21	28	35	42
vehicle										
	2735	1/1	189.50	237.20	295.80	330.10	364.30	409.00	Dead	
	2736	1/1	193.30	237.10	303.80	350.50	390.50	426.90	Dead	
	2737	1/1	178.50	232.80	317.30	368.60	403.20	444.50	Dead	
	2738	1/1	185.20	227.90	291.80	337.90	371.30	404.60	Dead	
	2739	1/1	188.00	238.70	311.00	356.20	400.20	440.80	Dead	
	2740	1/1	183.40	232.60	300.60	357.90	402.30	438.90	Dead	
	2741	1/1	195.00	241.30	308.20	345.90	382.90	408.50	Dead	
	2742	1/1	183.40	224.70	277.70	315.10	347.80	386.60	Dead	
	2743	1/1	178.90	227.90	293.50	334.20	366.40	406.50	Dead	
	2744	1/1	185.70	234.00	304.20	347.90	393.20	429.50	Dead	
	2745	1/1	184.80	226.80	289.10	337.40	368.80	395.80	412.10	440.40
	2746	1/1	169.70	208.80	268.10	307.30	334.70	363.40	377.50	404.40
	2747	1/1	172.70	216.00	282.40	335.50	380.60	419.70	434.30	471.60
	2748	1/1	196.50	245.00	316.40	353.70	390.00	434.70	457.40	502.50
	2749	1/1	185.20	226.80	279.30	317.10	341.00	362.10	376.10	399.60
		N	15	15	15	15	15	15	5	5
		Mean	184.65	230.51	295.95	339.69	375.81	411.43	411.48	443.70
		Sdev	7.555	9.488	14.740	17.261	22.066	26.150	35.483	43.977
50 mg/kg										
	2750	2/1	192.20	243.60	324.00	379.80	427.80	443.40	Dead	
	2751	2/1	175.60	224.80	286.50	333.30	370.20	389.70	Dead	
	2752	2/1	173.10	219.40	286.70	343.30	389.90	423.20	Dead	
	2753	2/1	185.40	230.80	294.60	335.30	358.70	382.40	Dead	
	2754	2/1	187.70	233.20	292.00	323.70	351.00	370.20	Dead	
	2755	2/1	172.00	247.60	330.70	390.30	430.30	476.70	Dead	
	2756	2/1	193.30	211.60	274.20	320.00	353.30	381.80	Dead	
	2757	2/1	174.50	218.40	275.20	317.30	337.90	368.50	Dead	
	2758	2/1	175.00	212.10	271.90	316.60	342.30	364.50	Dead	
	2759	2/1	175.30	218.60	284.20	324.70	352.30	386.80	Dead	
		N	10	10	10	10	10	10	0	0
		Mean	180.41	226.01	292.00	338.43	371.37	398.72	-	-
		Sdev	8.305	12.514	20.140	26.098	33.719	37.044	-	-

Note: " = Pretest phase (groups); # = Test period

Appendix 2
Body Weights (g)

Fexinidazole Study Number: 0504-2007

Males

Dose Level	Animal Number	Group/ Subgroup	Study Day -4"	1#	8	14	21	28	35	42
200 mg/kg										
	2760	3/1	182.50	229.30	293.80	335.70	358.70	381.20	Dead	
	2761	3/1	149.10	220.10	283.80	333.30	371.60	410.50	Dead	
	2762	3/1	177.10	228.20	294.60	348.60	380.00	419.90	Dead	
	2763	3/1	174.70	211.80	270.10	307.00	326.20	345.50	Dead	
	2764	3/1	196.30	239.10	308.10	350.90	384.00	414.20	Dead	
	2765	3/1	188.40	227.20	295.40	339.20	370.00	393.80	Dead	
	2766	3/1	175.90	227.50	278.60	321.60	342.00	371.00	Dead	
	2767	3/1	174.40	216.30	257.20	270.30	288.80	297.90	Dead	
	2768	3/1	174.10	219.20	293.10	347.70	400.80	447.30	Dead	
	2769	3/1	171.60	208.80	263.50	306.10	329.00	351.40	Dead	
		N	10	10	10	10	10	10	0	0
		Mean	176.41	222.75	283.82	326.04	355.11	383.27	-	-
		Sdev	12.286	9.177	16.202	25.391	33.557	43.635	-	-
800 mg/kg										
	2770	4/1	175.20	220.70	280.20	321.50	346.70	368.50	Dead	
	2771	4/1	192.00	238.40	292.50	323.60	337.70	366.30	Dead	
	2772	4/1	190.00	234.40	301.00	356.80	400.00	437.80	Dead	
	2773	4/1	193.10	235.90	272.70	286.70	300.10	314.70	Dead	
	2774	4/1	173.20	206.40	240.90	264.30	284.50	307.30	Dead	
	2775	4/1	177.70	222.90	283.90	341.70	381.50	409.60	Dead	
	2776	4/1	194.90	240.50	297.60	330.40	350.10	368.30	Dead	
	2777	4/1	175.20	222.00	281.70	325.70	353.40	382.90	Dead	
	2778	4/1	174.10	210.60	270.00	311.00	337.50	370.70	Dead	
	2779	4/1	203.50	244.90	288.20	310.80	331.40	350.50	Dead	
	2780	4/1	175.10	210.70	262.40	301.20	331.60	358.30	370.50	406.20
	2781	4/1	185.00	225.10	271.70	297.70	319.20	339.70	348.00	368.50
	2782	4/1	185.00	232.40	280.10	308.60	339.10	365.00	369.60	394.80
	2783	4/1	189.50	234.70	294.90	350.30	380.10	405.50	417.80	450.20
	2784	4/1	184.40	231.10	279.70	316.00	357.80	380.50	389.60	419.50
		N	15	15	15	15	15	15	5	5
		Mean	184.53	227.38	279.83	316.42	343.38	368.37	379.10	407.84
		Sdev	9.253	11.692	15.294	24.025	30.076	33.944	26.169	30.210

Note: " = Pretest phase (groups); # = Test period

Appendix 2
Body Weights (g)

Fexinidazole   Study Number: 0504-2007

Dose Level	Animal Number	Group/ Subgroup	Study Day -4"	1#	8	14	21	28	35	42
			**F e m a l e s**							
vehicle										
	2785	1/1	172.60	185.70	197.50	200.70	212.00	229.60	Dead	
	2786	1/1	177.50	199.30	197.50	237.40	247.20	256.40	Dead	
	2787	1/1	168.50	191.70	207.30	219.70	226.60	223.20	Dead	
	2788	1/1	186.30	192.40	213.80	218.10	220.80	235.50	Dead	
	2789	1/1	171.00	184.30	205.00	214.80	216.70	222.40	Dead	
	2790	1/1	174.30	197.00	209.20	226.40	228.80	243.20	Dead	
	2791	1/1	182.90	188.90	209.20	215.60	212.40	222.90	Dead	
	2792	1/1	175.80	197.20	202.30	226.50	243.90	249.70	Dead	
	2793	1/1	175.10	189.20	216.60	226.30	230.40	240.40	Dead	
	2794	1/1	171.50	193.60	226.10	233.30	250.20	261.20	Dead	
	2795	1/1	163.30	188.80	205.40	226.80	239.80	236.90	251.20	256.20
	2796	1/1	163.30	179.80	211.00	223.30	232.20	232.60	249.50	256.80
	2797	1/1	182.00	202.30	217.80	229.20	241.30	244.00	244.00	249.90
	2798	1/1	179.30	220.10	231.40	242.40	249.80	258.50	268.90	275.60
	2799	1/1	172.30	194.10	220.00	232.70	241.60	255.10	265.10	277.20
		N	15	15	15	15	15	15	5	5
		Mean	174.38	193.63	211.34	224.88	232.91	240.77	255.74	263.14
		Sdev	6.636	9.427	9.771	10.257	13.184	13.302	10.702	12.416
50 mg/kg										
	2800	2/1	166.80	185.70	194.70	196.00	214.80	222.20	Dead	
	2801	2/1	178.10	196.90	220.00	238.50	243.70	259.40	Dead	
	2802	2/1	170.90	190.60	215.90	231.20	245.00	248.00	Dead	
	2803	2/1	162.20	187.70	199.40	217.10	230.80	229.90	Dead	
	2804	2/1	164.00	177.50	190.60	209.10	219.50	215.90	Dead	
	2805	2/1	180.40	202.70	208.10	230.80	243.60	250.40	Dead	
	2806	2/1	168.10	195.30	217.20	214.90	233.50	245.00	Dead	
	2807	2/1	171.10	190.40	217.20	222.60	236.90	241.60	Dead	
	2808	2/1	177.40	201.70	216.20	241.50	254.10	255.50	Dead	
	2809	2/1	182.30	205.50	221.40	246.60	257.60	261.70	Dead	
		N	10	10	10	10	10	10	0	0
		Mean	172.13	193.40	210.07	224.83	237.95	242.96	-	-
		Sdev	7.049	8.686	11.221	15.835	13.784	15.632	-	-

Note: " = Pretest phase (groups); # = Test period

Appendix 2
Body Weights (g)

Fexinidazole Study Number: 0504-2007

Dose Level	Animal Number	Group/ Subgroup	Study Day -4"	1#	8	14	21	28	35	42
			**F e m a l e s**							
200 mg/kg										
	2810	3/1	160.90	195.60	203.10	220.70	224.10	229.40	Dead	
	2811	3/1	174.20	183.40	204.30	227.50	234.10	245.30	Dead	
	2812	3/1	172.10	195.80	209.80	223.00	235.80	244.30	Dead	
	2813	3/1	160.90	184.10	210.20	224.60	242.20	251.80	Dead	
	2814	3/1	165.40	189.80	204.10	207.20	222.40	223.30	Dead	
	2815	3/1	170.80	188.40	197.90	220.50	233.60	233.80	Dead	
	2816	3/1	178.70	199.90	214.60	227.60	245.70	251.80	Dead	
	2817	3/1	185.20	208.40	219.20	240.90	257.50	260.40	Dead	
	2818	3/1	170.80	191.70	212.40	220.50	241.10	257.00	Dead	
	2819	3/1	166.90	193.50	198.80	224.50	238.70	239.50	Dead	
		N	10	10	10	10	10	10	0	0
		Mean	170.59	193.06	207.44	223.70	237.52	243.66	-	-
		Sdev	7.623	7.484	6.935	8.361	10.225	12.139	-	-
800 mg/kg										
	2820	4/1	179.70	195.20	203.80	206.00	217.50	224.70	Dead	
	2821	4/1	176.80	191.20	199.80	198.80	210.10	213.50	Dead	
	2822	4/1	183.60	212.40	215.30	230.40	237.00	236.60	Dead	
	2823	4/1	178.70	192.70	228.10	242.90	251.00	270.20	Dead	
	2824	4/1	178.80	204.60	216.40	Dead				
	2825	4/1	166.20	178.80	203.30	213.60	217.50	229.80	Dead	
	2826	4/1	177.80	200.30	218.10	220.60	229.00	229.80	Dead	
	2827	4/1	171.80	184.80	220.80	230.20	227.10	241.70	Dead	
	2828	4/1	167.40	182.10	211.70	224.80	237.30	245.20	Dead	
	2829	4/1	170.00	192.10	209.10	222.60	233.70	229.60	Dead	
	2830	4/1	182.80	201.50	220.80	235.70	234.00	248.40	258.40	264.60
	2831	4/1	162.00	167.40	197.10	207.00	206.80	226.00	226.90	228.70
	2832	4/1	171.10	188.00	194.30	215.00	220.60	215.90	224.70	233.90
	2833	4/1	168.30	192.90	209.10	226.10	237.60	243.90	239.70	258.70
	2834	4/1	170.80	187.60	216.60	228.30	238.00	245.20	244.00	254.70
		N	15	15	15	14	14	14	5	5
		Mean	173.72	191.44	210.95	221.57	228.37	235.75	238.74	248.12
		Sdev	6.485	11.039	9.748	12.295	12.410	14.788	13.715	15.860

Note: " = Pretest phase (groups); # = Test period

Appendix 3 Food Consumption

Appendix 3
Feed Consumed per Day (g)

Fexinidazole

Study Number: 0504-2007

Males

Dose Level	Animal Number	Group/ Subgroup	Study Day 8	14	21	28	35	42
vehicle								
	2735-2737	1/1	31.12	30.64	30.39	30.90	-	-
	2738-2739	1/1	28.25	29.52	29.75	30.09	-	-
	2740-2742	1/1	27.59	28.84	29.75	29.14	-	-
	2743-2744	1/1	27.99	29.85	28.72	29.95	-	-
	2745-2747	1/1	25.18	28.25	28.22	28.06	28.47	29.64
	2748-2749	1/1	28.16	27.13	26.88	28.07	30.55	31.43
		N	15	15	15	15	5	5
		Mean	28.05	29.04	28.95	29.37	29.51	30.53
		Sdev	1.90	1.25	1.28	1.15	1.47	1.27
50 mg/kg								
	2750-2752	2/1	25.13	31.17	31.48	26.07	-	-
	2753-2754	2/1	29.29	28.64	27.10	26.82	-	-
	2755-2757	2/1	26.86	28.48	27.65	27.78	-	-
	2758-2759	2/1	27.29	28.71	27.69	27.82	-	-
		N	10	10	10	10	0	0
		Mean	27.14	29.25	28.48	27.12	-	-
		Sdev	1.71	1.29	2.02	0.84	-	-
200 mg/kg								
	2760-2762	3/1	27.64	29.79	28.41	27.35	-	-
	2763-2764	3/1	26.39	26.80	25.95	26.14	-	-
	2765-2767	3/1	26.30	26.31	25.04	24.60	-	-
	2768-2769	3/1	27.01	27.97	28.95	28.49	-	-
		N	10	10	10	10	0	0
		Mean	26.84	27.72	27.09	26.65	-	-
		Sdev	0.62	1.55	1.89	1.66	-	-

Note: Data for Test period
- Missing data

Appendix 3
Feed Consumed per Day (g)

Fexinidazole | Study Number: 0504-2007

Males

Dose Level	Animal Number	Group/ Subgroup	Study Day 8	14	21	28	35	42
800 mg/kg								
	2770-2772	4/1	27.43	30.17	29.12	28.16	-	-
	2773-2774	4/1	22.99	21.64	20.86	21.66	-	-
	2775-2777	4/1	25.70	30.04	28.45	27.70	-	-
	2778-2779	4/1	24.89	27.55	26.27	25.09	-	-
	2780-2782	4/1	24.16	25.79	24.30	24.02	26.32	27.01
	2783-2784	4/1	25.89	30.83	30.00	29.18	30.68	30.54
		N	15	15	15	15	5	5
		Mean	25.18	27.67	26.50	25.97	28.50	28.78
		Sdev	1.54	3.51	3.45	2.87	3.08	2.50

Note: Data for Test period
- Missing data

Appendix 3
Feed Consumed per Day (g)

Fexinidazole Study Number: 0504-2007

Females

Dose Level	Animal Number	Group/ Subgroup	Study Day 8	14	21	28	35	42
vehicle								
	2785-2787	1/1	11.04	17.74	17.43	17.47	-	-
	2788-2789	1/1	17.45	16.75	16.47	18.29	-	-
	2790-2792	1/1	16.06	17.37	17.08	17.64	-	-
	2793-2794	1/1	18.98	19.39	19.29	20.61	-	-
	2795-2797	1/1	17.15	18.49	18.08	17.91	19.58	18.64
	2798-2799	1/1	18.62	18.33	18.09	18.96	20.67	19.04
		N	15	15	15	15	5	5
		Mean	16.55	18.01	17.74	18.48	20.12	18.84
		Sdev	2.90	0.93	0.98	1.17	0.77	0.28
50 mg/kg								
	2800-2802	2/1	16.64	18.30	18.19	18.52	-	-
	2803-2804	2/1	16.99	18.65	17.71	17.42	-	-
	2805-2807	2/1	16.55	17.76	18.61	18.62	-	-
	2808-2809	2/1	18.37	20.92	20.63	20.89	-	-
		N	10	10	10	10	0	0
		Mean	17.14	18.91	18.78	18.86	-	-
		Sdev	0.84	1.39	1.28	1.45	-	-
200 mg/kg								
	2810-2812	3/1	16.35	18.11	17.53	18.28	-	-
	2813-2814	3/1	16.77	17.23	17.30	16.70	-	-
	2815-2817	3/1	16.89	18.26	17.90	18.38	-	-
	2818-2819	3/1	18.65	18.98	21.17	21.76	-	-
		N	10	10	10	10	0	0
		Mean	17.16	18.14	18.48	18.78	-	-
		Sdev	1.02	0.72	1.81	2.13	-	-

Note: Data for Test period
- Missing data

Appendix 3
Feed Consumed per Day (g)

Fexinidazole | Study Number: 0504-2007

Females

Dose Level	Animal Number	Group/ Subgroup	Study Day 8	14	21	28	35	42
800 mg/kg								
	2820-2822	4/1	13.68	17.27	18.27	17.79	-	-
	2823-2824	4/1	15.29	16.13	21.11	22.27	-	-
	2825-2827	4/1	14.50	18.09	17.67	17.92	-	-
	2828-2829	4/1	15.79	19.38	19.92	19.89	-	-
	2830-2832	4/1	14.52	17.42	17.50	18.61	20.17	18.10
	2833-2834	4/1	15.55	18.95	18.93	20.81	20.59	19.71
		N	15	15	14	14	5	5
		Mean	14.89	17.87	18.90	19.55	20.38	18.91
		Sdev	0.79	1.19	1.40	1.78	0.30	1.14

Note: Data for Test period
- Missing data

Appendix 4 Ophthalmoscopic Findings

Appendix 4
Individual Animal Ophthalmoscopic Observations

Fexinidazole | Study Number: 0504-2007

Males

Animal Number	Dose	Clinical Signs	Study Day(s) Noted
2735	vehicle	NORMAL	Pretest phase (groups) -4<br>Test period 28
2736	vehicle	NORMAL	Pretest phase (groups) -4<br>Test period 28
2737	vehicle	NORMAL	Pretest phase (groups) -4<br>Test period 28
2738	vehicle	NORMAL	Pretest phase (groups) -4<br>Test period 28
2739	vehicle	NORMAL	Pretest phase (groups) -4<br>Test period 28
2740	vehicle	NORMAL	Pretest phase (groups) -4<br>Test period 28
2741	vehicle	NORMAL	Pretest phase (groups) -4<br>Test period 28
2742	vehicle	NORMAL	Pretest phase (groups) -4<br>Test period 28
2743	vehicle	NORMAL	Pretest phase (groups) -4<br>Test period 28
2744	vehicle	NORMAL	Pretest phase (groups) -4<br>Test period 28
2745	vehicle	NORMAL	Pretest phase (groups) -4<br>Test period 28,42

Appendix 4
Individual Animal Ophthalmoscopic Observations

Fexinidazole

Study Number: 0504-2007

M a l e s

Animal Number	Dose	Clinical Signs	Study Day(s) Noted
2746	vehicle	NORMAL	Pretest phase (groups) -4<br>Test period 28,42
2747	vehicle	NORMAL	Pretest phase (groups) -4<br>Test period 28,42
2748	vehicle	NORMAL	Pretest phase (groups) -4<br>Test period 28,42
2749	vehicle	NORMAL	Pretest phase (groups) -4<br>Test period 28,42
2750	50mg/kg	NORMAL	Pretest phase (groups) -4<br>Test period 28
2751	50mg/kg	NORMAL	Pretest phase (groups) -4<br>Test period 28
2752	50mg/kg	NORMAL	Pretest phase (groups) -4<br>Test period 28
2753	50mg/kg	NORMAL	Pretest phase (groups) -4<br>Test period 28
2754	50mg/kg	NORMAL	Pretest phase (groups) -4<br>Test period 28
2755	50mg/kg	NORMAL	Pretest phase (groups) -4<br>Test period 28
2756	50mg/kg	NORMAL	Pretest phase (groups) -4<br>Test period 28

Appendix 4
Individual Animal Ophthalmoscopic Observations

Fexinidazole Study Number: 0504-2007

M a l e s

Animal Number	Dose	Clinical Signs	Study Day(s) Noted
2757	50mg/kg	NORMAL	Pretest phase (groups) -4<br>Test period 28
2758	50mg/kg	NORMAL	Pretest phase (groups) -4<br>Test period 28
2759	50mg/kg	NORMAL	Pretest phase (groups) -4<br>Test period 28
2760	200mg/kg	NORMAL	Pretest phase (groups) -4<br>Test period 28
2761	200mg/kg	NORMAL	Pretest phase (groups) -4<br>Test period 28
2762	200mg/kg	NORMAL	Pretest phase (groups) -4<br>Test period 28
2763	200mg/kg	NORMAL	Pretest phase (groups) -4<br>Test period 28
2764	200mg/kg	NORMAL	Pretest phase (groups) -4<br>Test period 28
2765	200mg/kg	NORMAL	Pretest phase (groups) -4<br>Test period 28
2766	200mg/kg	NORMAL	Pretest phase (groups) -4<br>Test period 28
2767	200mg/kg	NORMAL	Pretest phase (groups) -4<br>Test period 28

Appendix 4
Individual Animal Ophthalmoscopic Observations

Fexinidazole | Study Number: 0504-2007

M a l e s

Animal Number	Dose	Clinical Signs	Study Day(s) Noted
2768	200mg/kg	NORMAL	Pretest phase (groups) -4<br>Test period 28
2769	200mg/kg	NORMAL	Pretest phase (groups) -4<br>Test period 28
2770	800mg/kg	NORMAL	Pretest phase (groups) -4<br>Test period 28
2771	800mg/kg	NORMAL	Pretest phase (groups) -4<br>Test period 28
2772	800mg/kg	NORMAL	Pretest phase (groups) -4<br>Test period 28
2773	800mg/kg	NORMAL	Pretest phase (groups) -4<br>Test period 28
2774	800mg/kg	NORMAL	Pretest phase (groups) -4<br>Test period 28
2775	800mg/kg	NORMAL	Pretest phase (groups) -4<br>Test period 28
2776	800mg/kg	NORMAL	Pretest phase (groups) -4<br>Test period 28
2777	800mg/kg	NORMAL	Pretest phase (groups) -4<br>Test period 28
2778	800mg/kg	NORMAL	Pretest phase (groups) -4<br>Test period 28

Appendix 4
Individual Animal Ophthalmoscopic Observations

Fexinidazole | Study Number: 0504-2007

M a l e s

Animal Number	Dose	Clinical Signs	Study Day(s) Noted
2779	800mg/kg	NORMAL	Pretest phase (groups) -4<br>Test period 28
2780	800mg/kg	NORMAL	Pretest phase (groups) -4<br>Test period 28,42
2781	800mg/kg	NORMAL	Pretest phase (groups) -4<br>Test period 28,42
2782	800mg/kg	NORMAL	Pretest phase (groups) -4<br>Test period 28,42
2783	800mg/kg	NORMAL	Pretest phase (groups) -4<br>Test period 28,42
2784	800mg/kg	NORMAL	Pretest phase (groups) -4<br>Test period 28,42

Appendix 4
Individual Animal Ophthalmoscopic Observations

Fexinidazole — Study Number: 0504-2007

F e m a l e

Animal Number	Dose	Clinical Signs	Study Day(s) Noted
2785	vehicle	NORMAL	Pretest phase (groups) -3<br>Test period 28
2786	vehicle	NORMAL	Pretest phase (groups) -3<br>Test period 28
2787	vehicle	NORMAL	Pretest phase (groups) -3<br>Test period 28
2788	vehicle	NORMAL	Pretest phase (groups) -3<br>Test period 28
2789	vehicle	NORMAL	Pretest phase (groups) -3<br>Test period 28
2790	vehicle	NORMAL	Pretest phase (groups) -3<br>Test period 28
2791	vehicle	NORMAL	Pretest phase (groups) -3<br>Test period 28
2792	vehicle	NORMAL	Pretest phase (groups) -3<br>Test period 28
2793	vehicle	NORMAL	Pretest phase (groups) -3<br>Test period 28
2794	vehicle	NORMAL	Pretest phase (groups) -3<br>Test period 28
2795	vehicle	NORMAL	Pretest phase (groups) -3<br>Test period 28,42

Appendix 4
Individual Animal Ophthalmoscopic Observations

Fexinidazole Study Number: 0504-2007

F e m a l e

Animal Number	Dose	Clinical Signs	Study Day(s) Noted
2796	vehicle	NORMAL	Pretest phase (groups) -3<br>Test period 28,42
2797	vehicle	NORMAL	Pretest phase (groups) -3<br>Test period 28,42
2798	vehicle	NORMAL	Pretest phase (groups) -3<br>Test period 28,42
2799	vehicle	NORMAL	Pretest phase (groups) -3<br>Test period 28,42
2800	50mg/kg	NORMAL	Pretest phase (groups) -3<br>Test period 28
2801	50mg/kg	NORMAL	Pretest phase (groups) -3<br>Test period 28
2802	50mg/kg	NORMAL	Pretest phase (groups) -3<br>Test period 28
2803	50mg/kg	NORMAL	Pretest phase (groups) -3<br>Test period 28
2804	50mg/kg	NORMAL	Pretest phase (groups) -3<br>Test period 28
2805	50mg/kg	NORMAL	Pretest phase (groups) -3<br>Test period 28
2806	50mg/kg	NORMAL	Pretest phase (groups) -3<br>Test period 28

Appendix 4
Individual Animal Ophthalmoscopic Observations

Fexinidazole — Study Number: 0504-2007

F e m a l e

Animal Number	Dose	Clinical Signs	Study Day(s) Noted
2807	50mg/kg	NORMAL	Pretest phase (groups) -3<br>Test period 28
2808	50mg/kg	NORMAL	Pretest phase (groups) -3<br>Test period 28
2809	50mg/kg	NORMAL	Pretest phase (groups) -3<br>Test period 28
2810	200mg/kg	NORMAL	Pretest phase (groups) -3<br>Test period 28
2811	200mg/kg	NORMAL	Pretest phase (groups) -3<br>Test period 28
2812	200mg/kg	NORMAL	Pretest phase (groups) -3<br>Test period 28
2813	200mg/kg	NORMAL	Pretest phase (groups) -3<br>Test period 28
2814	200mg/kg	NORMAL	Pretest phase (groups) -3<br>Test period 28
2815	200mg/kg	NORMAL	Pretest phase (groups) -3<br>Test period 28
2816	200mg/kg	NORMAL	Pretest phase (groups) -3<br>Test period 28
2817	200mg/kg	NORMAL	Pretest phase (groups) -3<br>Test period 28

Appendix 4
Individual Animal Ophthalmoscopic Observations

Fexinidazole | Study Number: 0504-2007

F e m a l e

Animal Number	Dose	Clinical Signs	Study Day(s) Noted
2818	200mg/kg	NORMAL	Pretest phase (groups) -3<br>Test period 28
2819	200mg/kg	NORMAL	Pretest phase (groups) -3<br>Test period 28
2820	800mg/kg	NORMAL	Pretest phase (groups) -3<br>Test period 28
2821	800mg/kg	NORMAL	Pretest phase (groups) -3<br>Test period 28
2822	800mg/kg	NORMAL	Pretest phase (groups) -3<br>Test period 28
2823	800mg/kg	NORMAL	Pretest phase (groups) -3<br>Test period 28
2824	800mg/kg	NORMAL	Pretest phase (groups) -3
2825	800mg/kg	NORMAL	Pretest phase (groups) -3<br>Test period 28
2826	800mg/kg	NORMAL	Pretest phase (groups) -3<br>Test period 28
2827	800mg/kg	NORMAL	Pretest phase (groups) -3<br>Test period 28
2828	800mg/kg	NORMAL	Pretest phase (groups) -3<br>Test period 28
2829	800mg/kg	NORMAL	Pretest phase (groups) -3

Appendix 4
Individual Animal Ophthalmoscopic Observations

Fexinidazole Study Number: 0504-2007

F e m a l e

Animal Number	Dose	Clinical Signs	Study Day(s) Noted
2829	800mg/kg	NORMAL	Test period 28
2830	800mg/kg	NORMAL	Pretest phase (groups) -3<br>Test period 28,42
2831	800mg/kg	NORMAL	Pretest phase (groups) -3<br>Test period 28,42
2832	800mg/kg	NORMAL	Pretest phase (groups) -3<br>Test period 28,42
2833	800mg/kg	NORMAL	Pretest phase (groups) -3<br>Test period 28,42
2834	800mg/kg	NORMAL	Pretest phase (groups) -3<br>Test period 28,42

Appendix 5 Hematology

Appendix 5
Day 29 Hematology Data
Test period

Session 1 (Scheduled)
Fexinidazole

Study Number: 0504-2007

M a l e s

Dose Level	Animal Number	Group/ Subgroup	Study Day	RBC 10^6/mcL	HGB g/dL	HCT %	MCV fL	MCH pg	MCHC g/dL	RDW %	HDW g/dL
vehicle											
	2745	1/1	29	8.42	15.8	47.3	56.1	18.7	33.4	10.5	2.66
	2746	1/1	29	9.02	15.4	47.8	53.0	17.1	32.2	11.1	2.57
	2747	1/1	29	8.09	15.2	46.6	57.7	18.9	32.7	11.0	2.64
	2748	1/1	29	8.61	15.2	47.1	54.7	17.6	32.2	11.2	2.51
	2749	1/1	29	9.36	16.7	50.7	54.1	17.9	33.0	10.7	2.62
			N	5	5	5	5	5	5	5	5
			Mean	8.70	15.7	47.9	55.1	18.0	32.7	10.9	2.60
			Sdev	0.499	0.63	1.62	1.83	0.75	0.52	0.29	0.060
50 mg/kg											
	2755	2/1	29	8.16	15.8	48.2	59.1	19.3	32.7	11.2	2.61
	2756	2/1	29	8.65	15.3	47.6	55.0	17.7	32.2	10.3	2.58
	2757	2/1	29	8.52	15.2	47.0	55.1	17.8	32.3	10.9	2.61
	2758	2/1	29	8.01	14.8	45.3	56.6	18.5	32.7	10.9	2.70
	2759	2/1	29	8.39	15.1	47.0	56.0	18.0	32.1	11.5	2.66
			N	5	5	5	5	5	5	5	5
			Mean	8.35	15.2	47.0	56.4	18.3	32.4	11.0	2.63
			Sdev	0.261	0.36	1.08	1.67	0.66	0.28	0.44	0.048
200 mg/kg											
	2765	3/1	29	8.40	15.7	47.4	56.5	18.7	33.1	10.4	2.53
	2766	3/1	29	8.30	15.5	47.6	57.4	18.7	32.6	10.5	2.64
	2767	3/1	29	8.80	15.1	47.1	53.5	17.1	32.0	11.3	2.79
	2768	3/1	29	8.06	15.4	48.2	59.8	19.1	31.9	11.2	2.64
	2769	3/1	29	8.91	16.1	49.9	56.1	18.1	32.3	10.3	2.62
			N	5	5	5	5	5	5	5	5
			Mean	8.49	15.6	48.0	56.7	18.3	32.4	10.7	2.64
			Sdev	0.354	0.37	1.11	2.28	0.78	0.49	0.47	0.093

RBC - RED BLOOD CELLS
HGB - HEMOGLOBIN
HCT - HEMATOCRIT
MCV - MEAN CORPUSCULAR VOLUME
MCH - MEAN CORPUSCULAR HEMOGLOBIN
MCHC - MEAN CORPUSCULAR HGB CONC.
RDW - RED CELL DISTRIBUTION WIDTH
HDW - HEMOGLOBIN DISTRIB. WIDTH

Appendix 5
Day 29 Hematology Data
Test period

Session 1 (Scheduled)
Fexinidazole

Study Number: 0504-2007

Males

Dose Level	Animal Number	Group/ Subgroup	Study Day	RBC 10^6/mcL	HGB g/dL	HCT %	MCV fL	MCH pg	MCHC g/dL	RDW %	HDW g/dL
800 mg/kg											
	2780	4/1	29	8.07	15.1	45.8	56.8	18.8	33.0	10.4	2.79
	2781	4/1	29	8.15	15.4	46.0	56.4	18.9	33.5	11.0	3.25
	2782	4/1	29	8.90	15.7	49.7	55.8	17.7	31.7	10.6	2.83
	2783	4/1	29	8.24	15.4	46.6	56.6	18.7	33.0	11.7	2.77
	2784	4/1	29	8.13	14.9	45.9	56.5	18.4	32.5	11.0	2.74
			N	5	5	5	5	5	5	5	5
			Mean	8.30	15.3	46.8	56.4	18.5	32.7	10.9	2.88
			Sdev	0.342	0.31	1.65	0.38	0.48	0.68	0.50	0.212

RBC - RED BLOOD CELLS
HGB - HEMOGLOBIN
HCT - HEMATOCRIT
MCV - MEAN CORPUSCULAR VOLUME
MCH - MEAN CORPUSCULAR HEMOGLOBIN
MCHC - MEAN CORPUSCULAR HGB CONC.
RDW - RED CELL DISTRIBUTION WIDTH
HDW - HEMOGLOBIN DISTRIB. WIDTH

Appendix 5
Day 29 Hematology Data
Test period

Session 1 (Scheduled)
Fexinidazole
Study Number: 0504-2007

Males

Dose Level	Animal Number	Group/ Subgroup	Study Day	R %	RAB 10^9/L	MCVr fL	CHCM g/dL	CHr pg	PLT 10^3/mcL	MPV fL	PDW %
vehicle											
	2745	1/1	29	2.4	201.5	63.2	29.8	18.8	1341.	6.8	52.1
	2746	1/1	29	2.5	223.5	60.3	29.6	17.8	1223.	6.6	56.3
	2747	1/1	29	3.1	249.7	64.6	30.1	19.4	1266.	6.5	55.2
	2748	1/1	29	2.6	225.4	61.2	30.0	18.3	1094.	6.8	58.6
	2749	1/1	29	2.3	216.8	61.7	30.3	18.7	1247.	6.9	52.5
			N	5	5	5	5	5	5	5	5
			Mean	2.6	223.4	62.2	30.0	18.6	1234.	6.7	54.9
			Sdev	0.31	17.46	1.70	0.27	0.60	89.9	0.16	2.71
50 mg/kg											
	2755	2/1	29	2.9	233.1	64.7	30.0	19.4	1535.	7.4	52.9
	2756	2/1	29	1.9	165.5	62.7	29.4	18.4	1232.	6.6	53.6
	2757	2/1	29	2.2	190.7	62.8	29.4	18.4	1539.	6.9	53.2
	2758	2/1	29	2.5	202.5	64.9	29.9	19.3	1360.	6.3	50.8
	2759	2/1	29	2.8	233.4	64.2	29.6	19.0	1387.	6.2	53.0
			N	5	5	5	5	5	5	5	5
			Mean	2.5	205.0	63.9	29.7	18.9	1411.	6.7	52.7
			Sdev	0.39	29.01	1.05	0.28	0.48	129.4	0.49	1.10
200 mg/kg											
	2765	3/1	29	1.9	158.9	65.0	30.4	19.7	1255.	6.4	53.4
	2766	3/1	29	2.4	194.6	64.2	29.9	19.2	960.	6.7	57.2
	2767	3/1	29	1.8	158.2	61.4	29.5	18.1	993.	6.6	55.5
	2768	3/1	29	3.7	301.7	66.7	30.5	20.4	1414.	6.6	55.1
	2769	3/1	29	1.5	133.1	63.1	30.1	19.0	1605.	6.4	51.8
			N	5	5	5	5	5	5	5	5
			Mean	2.3	189.3	64.1	30.1	19.3	1245.	6.5	54.6
			Sdev	0.89	66.54	1.99	0.40	0.85	275.2	0.13	2.07

R - RETICULOCYTES
RAB - RETICULOCYTES ABS
MCVr - MEAN CORPUSCOLAR VOL. RETIC.
CHCM - MEAN HEMOGLOBIN CONC. RETIC.
CHr - CELLULAR HEMOGLOBIN RETIC.
PLT - PLATELETS
MPV - MEAN PLATELET VOLUME
PDW - PLATELET DISTRIBUTION WIDTH

Appendix 5
Day 29 Hematology Data
Test period

Session 1 (Scheduled)
Fexinidazole

Study Number: 0504-2007

Males

Dose Level	Animal Number	Group/ Subgroup	Study Day	R %	RAB 10^9/L	MCVr fL	CHCM g/dL	CHr pg	PLT 10^3/mcL	MPV fL	PDW %
800 mg/kg											
	2780	4/1	29	2.6	212.2	64.2	30.1	19.3	1279.	6.6	56.2
	2781	4/1	29	3.4	276.3	64.2	30.2	19.4	1256.	6.7	54.8
	2782	4/1	29	2.7	242.3	63.1	29.7	18.7	1227.	6.2	55.3
	2783	4/1	29	2.4	195.1	64.0	29.8	19.1	1274.	6.7	53.8
	2784	4/1	29	3.0	241.3	63.8	30.2	19.2	1465.	6.7	54.4
			N	5	5	5	5	5	5	5	5
			Mean	2.8	233.4	63.9	30.0	19.1	1300.	6.6	54.9
			Sdev	0.39	31.22	0.46	0.23	0.27	94.3	0.22	0.91

R - RETICULOCYTES
RAB - RETICULOCYTES ABS
MCVr - MEAN CORPUSCOLAR VOL. RETIC.
CHCM - MEAN HEMOGLOBIN CONC. RETIC.
CHr - CELLULAR HEMOGLOBIN RETIC.
PLT - PLATELETS
MPV - MEAN PLATELET VOLUME
PDW - PLATELET DISTRIBUTION WIDTH

Appendix 5
Day 29 Hematology Data
Test period

Session 1 (Scheduled)
Fexinidazole

Study Number: 0504-2007

M a l e s

Dose Level	Animal Number	Group/ Subgroup	Study Day	PCT %	WBC 10^3/mcL	NAB 10^3/mcL	LYAB 10^3/mcL	MAB 10^3/mcL	EAB 10^3/mcL	BAB 10^3/mcL
vehicle										
	2745	1/1	29	0.91	11.00	1.66	8.81	0.30	0.08	0.03
	2746	1/1	29	0.80	9.56	1.60	7.46	0.22	0.10	0.02
	2747	1/1	29	0.82	10.20	1.66	7.94	0.34	0.09	0.03
	2748	1/1	29	0.74	13.35	2.46	10.04	0.57	0.11	0.04
	2749	1/1	29	0.86	11.83	1.51	9.92	0.16	0.10	0.02
			N	5	5	5	5	5	5	5
			Mean	0.83	11.19	1.78	8.83	0.32	0.10	0.03
			Sdev	0.064	1.479	0.386	1.153	0.157	0.011	0.008
50 mg/kg										
	2755	2/1	29	1.14	13.13	2.07	10.31	0.37	0.12	0.04
	2756	2/1	29	0.81	9.28	2.09	6.56	0.30	0.16	0.03
	2757	2/1	29	1.06	11.31	1.75	8.88	0.31	0.14	0.03
	2758	2/1	29	0.86	12.04	1.99	9.35	0.37	0.09	0.03
	2759	2/1	29	0.86	13.97	2.52	10.88	0.24	0.12	0.04
			N	5	5	5	5	5	5	5
			Mean	0.95	11.95	2.08	9.20	0.32	0.13	0.03
			Sdev	0.145	1.804	0.279	1.670	0.054	0.026	0.005
200 mg/kg										
	2765	3/1	29	0.80	14.17	2.11	11.43	0.30	0.15	0.03
	2766	3/1	29	0.65	10.11	2.17	7.36	0.30	0.12	0.01
	2767	3/1	29	0.65	15.63	1.88	13.05	0.31	0.17	0.04
	2768	3/1	29	0.93	10.53	1.98	7.90	0.29	0.13	0.02
	2769	3/1	29	1.03	9.53	1.47	7.56	0.30	0.10	0.02
			N	5	5	5	5	5	5	5
			Mean	0.81	11.99	1.92	9.46	0.30	0.13	0.02
			Sdev	0.169	2.726	0.277	2.609	0.007	0.027	0.011

PCT - PLATELET HEMATOCRIT
WBC - WHITE BLOOD CELLS
NAB - NEUTROPHILS ABS
LYAB - LYMPHOCYTES ABS
MAB - MONOCYTES ABS
EAB - EOSINOPHILS ABS
BAB - BASOPHILS ABS

Appendix 5
Day 29 Hematology Data
Test period

Session 1 (Scheduled)
Fexinidazole

Study Number: 0504-2007

Males

Dose Level	Animal Number	Group/ Subgroup	Study Day	PCT %	WBC 10^3/mcL	NAB 10^3/mcL	LYAB 10^3/mcL	MAB 10^3/mcL	EAB 10^3/mcL	BAB 10^3/mcL
800 mg/kg										
	2780	4/1	29	0.84	8.92	1.22	7.15	0.27	0.10	0.02
	2781	4/1	29	0.84	12.87	2.45	9.78	0.28	0.21	0.04
	2782	4/1	29	0.76	7.82	1.55	5.82	0.28	0.06	0.01
	2783	4/1	29	0.86	10.23	1.35	8.29	0.27	0.13	0.02
	2784	4/1	29	0.98	11.43	2.60	8.27	0.32	0.11	0.02
			N	5	5	5	5	5	5	5
			Mean	0.86	10.25	1.83	7.86	0.28	0.12	0.02
			Sdev	0.079	1.996	0.644	1.475	0.021	0.055	0.011

PCT - PLATELET HEMATOCRIT
WBC - WHITE BLOOD CELLS
NAB - NEUTROPHILS ABS
LYAB - LYMPHOCYTES ABS
MAB - MONOCYTES ABS
EAB - EOSINOPHILS ABS
BAB - BASOPHILS ABS

Appendix 5
Day 29 Hematology Data
Test period

Session 1 (Scheduled)
Fexinidazole

Study Number: 0504-2007

Males

Dose Level	Animal Number	Group/ Subgroup	Study Day	LUAB 10^3/mcL	N %	LY %	M %	E %	B %	LU %
vehicle										
	2745	1/1	29	0.11	15.1	80.1	2.7	0.7	0.3	1.0
	2746	1/1	29	0.17	16.7	78.0	2.3	1.0	0.2	1.8
	2747	1/1	29	0.14	16.2	77.8	3.4	0.9	0.3	1.4
	2748	1/1	29	0.14	18.4	75.2	4.3	0.8	0.3	1.0
	2749	1/1	29	0.12	12.7	83.9	1.3	0.8	0.2	1.0
			N	5	5	5	5	5	5	5
			Mean	0.14	15.8	79.0	2.8	0.8	0.3	1.2
			Sdev	0.023	2.11	3.24	1.13	0.11	0.05	0.36
50 mg/kg										
	2755	2/1	29	0.22	15.8	78.5	2.8	0.9	0.3	1.7
	2756	2/1	29	0.15	22.5	70.7	3.2	1.7	0.3	1.6
	2757	2/1	29	0.21	15.5	78.5	2.7	1.3	0.2	1.8
	2758	2/1	29	0.21	16.5	77.7	3.1	0.7	0.2	1.7
	2759	2/1	29	0.17	18.0	77.8	1.7	0.9	0.3	1.2
			N	5	5	5	5	5	5	5
			Mean	0.19	17.7	76.6	2.7	1.1	0.3	1.6
			Sdev	0.030	2.87	3.34	0.60	0.40	0.05	0.23
200 mg/kg										
	2765	3/1	29	0.16	14.9	80.6	2.1	1.1	0.2	1.1
	2766	3/1	29	0.15	21.4	72.8	3.0	1.2	0.1	1.5
	2767	3/1	29	0.18	12.0	83.5	2.0	1.1	0.2	1.2
	2768	3/1	29	0.21	18.8	75.0	2.7	1.2	0.2	2.0
	2769	3/1	29	0.08	15.4	79.3	3.1	1.0	0.2	0.9
			N	5	5	5	5	5	5	5
			Mean	0.16	16.5	78.2	2.6	1.1	0.2	1.3
			Sdev	0.048	3.65	4.31	0.51	0.08	0.04	0.43

LUAB - LARGE UNSTAINED CELLS ABS
N - NEUTROPHILS %
LY - LYMPHOCITES %
M - MONOCYTES %
E - EOSINOPHILS %
B - BASOPHILS %
LU - LARGE UNSTAINED CELLS %

Appendix 5
Day 29 Hematology Data
Test period

Session 1 (Scheduled)
Fexinidazole
Study Number: 0504-2007

Males

Dose Level	Animal Number	Group/ Subgroup	Study Day	LUAB 10^3/mcL	N %	LY %	M %	E %	B %	LU %
800 mg/kg										
	2780	4/1	29	0.15	13.7	80.2	3.0	1.1	0.2	1.7
	2781	4/1	29	0.12	19.0	76.0	2.1	1.6	0.3	0.9
	2782	4/1	29	0.10	19.8	74.4	3.6	0.8	0.2	1.3
	2783	4/1	29	0.17	13.2	81.1	2.6	1.2	0.2	1.7
	2784	4/1	29	0.10	22.8	72.4	2.8	0.9	0.2	0.9
			N	5	5	5	5	5	5	5
			Mean	0.13	17.7	76.8	2.8	1.1	0.2	1.3
			Sdev	0.031	4.13	3.74	0.55	0.31	0.04	0.40

LUAB - LARGE UNSTAINED CELLS ABS
N - NEUTROPHILS %
LY - LYMPHOCITES %
M - MONOCYTES %
E - EOSINOPHILS %
B - BASOPHILS %
LU - LARGE UNSTAINED CELLS %

Appendix 5
Day 29 Hematology Data
Test period

Session 1 (Scheduled)
Fexinidazole
Study Number: 0504-2007

Females

Dose Level	Animal Number	Group/ Subgroup	Study Day	RBC 10^6/mcL	HGB g/dL	HCT %	MCV fL	MCH pg	MCHC g/dL	RDW %	HDW g/dL
vehicle											
	2795	1/1	29	8.34	14.4	42.6	51.0	17.3	33.8	10.4	2.19
	2796	1/1	29	8.55	15.7	45.9	53.7	18.3	34.2	10.5	2.36
	2797	1/1	29	8.10	15.3	44.5	55.0	18.8	34.3	10.3	2.10
	2798	1/1	29	8.65	15.7	46.2	53.4	18.1	33.9	10.1	2.45
	2799	1/1	29	7.84	14.5	41.8	53.3	18.5	34.7	10.7	2.41
			N	5	5	5	5	5	5	5	5
			Mean	8.30	15.1	44.2	53.3	18.2	34.2	10.4	2.30
			Sdev	0.331	0.63	1.96	1.44	0.57	0.36	0.22	0.150
50 mg/kg											
	2805	2/1	29	7.91	14.2	42.3	53.5	18.0	33.7	11.2	2.68
	2806	2/1	29	8.28	15.5	45.3	54.7	18.7	34.2	10.2	2.51
	2807	2/1	29	8.34	14.9	44.2	53.0	17.9	33.8	10.8	2.42
	2808	2/1	29	7.99	14.9	44.3	55.4	18.6	33.6	10.6	2.32
	2809	2/1	29	8.02	14.8	44.6	55.7	18.5	33.2	10.4	2.32
			N	5	5	5	5	5	5	5	5
			Mean	8.11	14.9	44.1	54.5	18.3	33.7	10.6	2.45
			Sdev	0.190	0.46	1.11	1.18	0.36	0.36	0.38	0.151
200 mg/kg											
	2815	3/1	29	8.12	15.2	44.6	55.0	18.7	34.0	10.8	2.68
	2816	3/1	29	8.16	15.1	43.8	53.7	18.5	34.4	11.3	2.55
	2817	3/1	29	8.05	14.5	44.1	54.8	18.0	32.9	10.7	2.47
	2818	3/1	29	7.98	13.9	41.7	52.2	17.4	33.3	12.0	2.61
	2819	3/1	29	8.23	14.8	44.3	53.8	17.9	33.4	10.9	2.48
			N	5	5	5	5	5	5	5	5
			Mean	8.11	14.7	43.7	53.9	18.1	33.6	11.1	2.56
			Sdev	0.097	0.52	1.16	1.11	0.51	0.60	0.53	0.089

RBC - RED BLOOD CELLS
HGB - HEMOGLOBIN
HCT - HEMATOCRIT
MCV - MEAN CORPUSCULAR VOLUME
MCH - MEAN CORPUSCULAR HEMOGLOBIN
MCHC - MEAN CORPUSCULAR HGB CONC.
RDW - RED CELL DISTRIBUTION WIDTH
HDW - HEMOGLOBIN DISTRIB. WIDTH

Appendix 5
Day 29 Hematology Data
Test period

Session 1 (Scheduled)
Fexinidazole

Study Number: 0504-2007

Females

Dose Level	Animal Number	Group/ Subgroup	Study Day	RBC 10^6/mcL	HGB g/dL	HCT %	MCV fL	MCH pg	MCHC g/dL	RDW %	HDW g/dL
800 mg/kg											
	2830	4/1	29	7.72	13.4	40.3	52.2	17.3	33.3	10.8	2.53
	2831	4/1	29	7.87	13.6	41.0	52.1	17.3	33.3	10.6	2.33
	2832	4/1	29	7.69	13.9	40.7	52.9	18.1	34.2	11.3	2.73
	2833	4/1	29	8.49	14.7	44.3	52.2	17.3	33.3	11.5	2.69
	2834	4/1	29	7.83	14.1	42.8	54.6	17.9	32.8	10.3	2.38
			N	5	5	5	5	5	5	5	5
			Mean	7.92	13.9	41.8	52.8	17.6	33.4	10.9	2.53
			Sdev	0.327	0.50	1.68	1.06	0.39	0.51	0.49	0.179

RBC - RED BLOOD CELLS
HGB - HEMOGLOBIN
HCT - HEMATOCRIT
MCV - MEAN CORPUSCULAR VOLUME
MCH - MEAN CORPUSCULAR HEMOGLOBIN
MCHC - MEAN CORPUSCULAR HGB CONC.
RDW - RED CELL DISTRIBUTION WIDTH
HDW - HEMOGLOBIN DISTRIB. WIDTH

Appendix 5
Day 29 Hematology Data
Test period

Session 1 (Scheduled)
Fexinidazole

Study Number: 0504-2007

Females

Dose Level	Animal Number	Group/ Subgroup	Study Day	R %	RAB 10^9/L	MCVr fL	CHCM g/dL	CHr pg	PLT 10^3/mcL	MPV fL	PDW %
vehicle											
	2795	1/1	29	2.0	168.5	58.4	31.2	18.2	1173.	5.7	53.3
	2796	1/1	29	2.2	189.8	61.6	31.9	19.6	1224.	6.0	56.6
	2797	1/1	29	2.3	184.9	61.9	32.1	19.9	1284.	5.8	53.6
	2798	1/1	29	1.6	133.8	61.7	31.7	19.5	1522.	5.8	52.3
	2799	1/1	29	2.8	220.1	62.6	31.3	19.5	1294.	5.7	50.4
			N	5	5	5	5	5	5	5	5
			Mean	2.2	179.4	61.2	31.6	19.3	1299.	5.8	53.2
			Sdev	0.46	31.60	1.63	0.38	0.66	133.7	0.12	2.26
50 mg/kg											
	2805	2/1	29	3.7	290.1	63.8	31.6	20.2	1212.	5.8	54.5
	2806	2/1	29	1.8	150.6	64.5	31.5	20.2	1076.	6.0	49.3
	2807	2/1	29	2.2	184.8	62.0	31.0	19.2	1038.	6.2	54.9
	2808	2/1	29	2.1	165.1	63.6	31.8	20.2	1207.	5.8	51.7
	2809	2/1	29	2.8	220.5	64.5	31.2	20.1	1008.	6.0	53.2
			N	5	5	5	5	5	5	5	5
			Mean	2.5	202.2	63.7	31.4	20.0	1108.	6.0	52.7
			Sdev	0.74	55.68	1.02	0.32	0.44	95.6	0.17	2.29
200 mg/kg											
	2815	3/1	29	2.5	199.8	63.5	31.6	20.0	1085.	5.7	50.7
	2816	3/1	29	2.6	213.4	63.5	31.8	20.1	1140.	6.6	57.3
	2817	3/1	29	1.7	135.4	62.9	31.6	19.8	1556.	6.1	53.5
	2818	3/1	29	3.1	250.6	62.7	30.6	19.1	1381.	6.3	52.9
	2819	3/1	29	1.8	150.5	62.8	31.7	19.8	1100.	5.7	52.4
			N	5	5	5	5	5	5	5	5
			Mean	2.3	189.9	63.1	31.5	19.8	1252.	6.1	53.4
			Sdev	0.60	47.06	0.39	0.49	0.39	207.7	0.39	2.44

R - RETICULOCYTES
RAB - RETICULOCYTES ABS
MCVr - MEAN CORPUSCOLAR VOL. RETIC.
CHCM - MEAN HEMOGLOBIN CONC. RETIC.
CHr - CELLULAR HEMOGLOBIN RETIC.
PLT - PLATELETS
MPV - MEAN PLATELET VOLUME
PDW - PLATELET DISTRIBUTION WIDTH

Appendix 5
Day 29 Hematology Data
Test period

Session 1 (Scheduled)
Fexinidazole

Study Number: 0504-2007

Females

Dose Level	Animal Number	Group/ Subgroup	Study Day	R %	RAB 10^9/L	MCVr fL	CHCM g/dL	CHr pg	PLT 10^3/mcL	MPV fL	PDW %
800 mg/kg											
	2830	4/1	29	3.1	239.0	63.3	30.4	19.2	1136.	5.8	53.2
	2831	4/1	29	3.1	243.2	60.8	30.6	18.6	1376.	6.1	53.4
	2832	4/1	29	2.5	192.3	61.3	30.7	18.7	1216.	5.9	52.9
	2833	4/1	29	2.6	221.2	61.2	30.7	18.8	1270.	6.1	55.6
	2834	4/1	29	2.5	194.9	63.3	30.6	19.4	1294.	5.9	50.4
			N	5	5	5	5	5	5	5	5
			Mean	2.8	218.1	62.0	30.6	18.9	1258.	6.0	53.1
			Sdev	0.31	23.88	1.22	0.12	0.34	89.5	0.13	1.85

R - RETICULOCYTES
RAB - RETICULOCYTES ABS
MCVr - MEAN CORPUSCOLAR VOL. RETIC.
CHCM - MEAN HEMOGLOBIN CONC. RETIC.
CHr - CELLULAR HEMOGLOBIN RETIC.
PLT - PLATELETS
MPV - MEAN PLATELET VOLUME
PDW - PLATELET DISTRIBUTION WIDTH

Appendix 5
Day 29 Hematology Data
Test period

Session 1 (Scheduled)
Fexinidazole

Study Number: 0504-2007

Females

Dose Level	Animal Number	Group/ Subgroup	Study Day	PCT %	WBC 10^3/mcL	NAB 10^3/mcL	LYAB 10^3/mcL	MAB 10^3/mcL	EAB 10^3/mcL	BAB 10^3/mcL
vehicle										
	2795	1/1	29	0.67	13.03	2.40	9.86	0.36	0.18	0.03
	2796	1/1	29	0.73	8.28	1.28	6.45	0.27	0.17	0.03
	2797	1/1	29	0.74	7.55	0.98	6.12	0.23	0.11	0.03
	2798	1/1	29	0.89	13.60	3.15	9.74	0.41	0.16	0.03
	2799	1/1	29	0.74	11.87	1.83	9.56	0.18	0.16	0.03
			N	5	5	5	5	5	5	5
			Mean	0.75	10.87	1.93	8.35	0.29	0.16	0.03
			Sdev	0.081	2.777	0.872	1.888	0.094	0.027	0.000
50 mg/kg										
	2805	2/1	29	0.71	7.60	0.74	6.35	0.24	0.17	0.01
	2806	2/1	29	0.65	12.82	1.81	10.34	0.25	0.21	0.02
	2807	2/1	29	0.64	7.05	2.38	4.24	0.28	0.07	0.01
	2808	2/1	29	0.70	9.64	1.92	7.03	0.37	0.23	0.02
	2809	2/1	29	0.61	10.46	1.67	8.10	0.37	0.17	0.02
			N	5	5	5	5	5	5	5
			Mean	0.66	9.51	1.70	7.21	0.30	0.17	0.02
			Sdev	0.042	2.322	0.601	2.246	0.064	0.062	0.005
200 mg/kg										
	2815	3/1	29	0.62	10.53	0.45	9.40	0.30	0.13	0.03
	2816	3/1	29	0.76	9.87	1.95	7.40	0.26	0.11	0.03
	2817	3/1	29	0.95	14.88	2.86	11.32	0.33	0.13	0.03
	2818	3/1	29	0.87	8.72	1.23	7.04	0.24	0.10	0.01
	2819	3/1	29	0.63	7.14	1.28	5.43	0.25	0.08	0.01
			N	5	5	5	5	5	5	5
			Mean	0.77	10.23	1.55	8.12	0.28	0.11	0.02
			Sdev	0.145	2.901	0.903	2.280	0.038	0.021	0.011

PCT - PLATELET HEMATOCRIT
WBC - WHITE BLOOD CELLS
NAB - NEUTROPHILS ABS
LYAB - LYMPHOCYTES ABS
MAB - MONOCYTES ABS
EAB - EOSINOPHILS ABS
BAB - BASOPHILS ABS

Appendix 5
Day 29 Hematology Data
Test period

Session 1 (Scheduled)
Fexinidazole

Study Number: 0504-2007

Females

Dose Level	Animal Number	Group/ Subgroup	Study Day	PCT %	WBC 10^3/mcL	NAB 10^3/mcL	LYAB 10^3/mcL	MAB 10^3/mcL	EAB 10^3/mcL	BAB 10^3/mcL
800 mg/kg										
	2830	4/1	29	0.66	9.45	1.09	7.90	0.19	0.12	0.03
	2831	4/1	29	0.84	9.86	1.06	8.29	0.25	0.10	0.03
	2832	4/1	29	0.71	11.24	0.58	10.03	0.38	0.13	0.02
	2833	4/1	29	0.77	9.76	1.22	8.13	0.20	0.09	0.03
	2834	4/1	29	0.77	14.57	1.18	12.80	0.29	0.05	0.03
			N	5	5	5	5	5	5	5
			Mean	0.75	10.98	1.03	9.43	0.26	0.10	0.03
			Sdev	0.068	2.124	0.258	2.064	0.077	0.031	0.004

PCT - PLATELET HEMATOCRIT
WBC - WHITE BLOOD CELLS
NAB - NEUTROPHILS ABS
LYAB - LYMPHOCYTES ABS
MAB - MONOCYTES ABS
EAB - EOSINOPHILS ABS
BAB - BASOPHILS ABS

Appendix 5
Day 29 Hematology Data
Test period

Session 1 (Scheduled)
Fexinidazole

Study Number: 0504-2007

Females

Dose Level	Animal Number	Group/ Subgroup	Study Day	LUAB 10^3/mcL	N %	LY %	M %	E %	B %	LU %
vehicle										
	2795	1/1	29	0.19	18.4	75.7	2.7	1.4	0.3	1.5
	2796	1/1	29	0.09	15.4	77.9	3.2	2.0	0.3	1.1
	2797	1/1	29	0.08	13.0	81.1	3.1	1.4	0.4	1.0
	2798	1/1	29	0.11	23.1	71.6	3.0	1.2	0.2	0.8
	2799	1/1	29	0.11	15.4	80.5	1.5	1.3	0.2	1.0
			N	5	5	5	5	5	5	5
			Mean	0.12	17.1	77.4	2.7	1.5	0.3	1.1
			Sdev	0.043	3.88	3.88	0.70	0.31	0.08	0.26
50 mg/kg										
	2805	2/1	29	0.09	9.8	83.5	3.2	2.2	0.1	1.1
	2806	2/1	29	0.18	14.2	80.7	2.0	1.6	0.2	1.4
	2807	2/1	29	0.06	33.8	60.1	3.9	1.1	0.2	0.9
	2808	2/1	29	0.08	19.9	72.9	3.8	2.4	0.2	0.8
	2809	2/1	29	0.12	16.0	77.5	3.5	1.6	0.2	1.1
			N	5	5	5	5	5	5	5
			Mean	0.11	18.7	74.9	3.3	1.8	0.2	1.1
			Sdev	0.047	9.17	9.18	0.77	0.52	0.04	0.23
200 mg/kg										
	2815	3/1	29	0.21	4.3	89.3	2.9	1.2	0.3	2.0
	2816	3/1	29	0.12	19.7	75.0	2.7	1.1	0.3	1.2
	2817	3/1	29	0.22	19.2	76.1	2.2	0.9	0.2	1.5
	2818	3/1	29	0.10	14.1	80.7	2.7	1.1	0.2	1.1
	2819	3/1	29	0.08	17.9	76.1	3.6	1.1	0.1	1.2
			N	5	5	5	5	5	5	5
			Mean	0.15	15.0	79.4	2.8	1.1	0.2	1.4
			Sdev	0.065	6.39	5.93	0.51	0.11	0.08	0.37

LUAB - LARGE UNSTAINED CELLS ABS
N - NEUTROPHILS %
LY - LYMPHOCITES %
M - MONOCYTES %
E - EOSINOPHILS %
B - BASOPHILS %
LU - LARGE UNSTAINED CELLS %

Appendix 5
Day 29 Hematology Data
Test period

Session 1 (Scheduled)
Fexinidazole

Study Number: 0504-2007

Females

Dose Level	Animal Number	Group/ Subgroup	Study Day	LUAB 10^3/mcL	N %	LY %	M %	E %	B %	LU %
800 mg/kg										
	2830	4/1	29	0.12	11.5	83.6	2.0	1.3	0.3	1.3
	2831	4/1	29	0.14	10.7	84.0	2.5	1.0	0.3	1.5
	2832	4/1	29	0.10	5.2	89.2	3.4	1.2	0.2	0.9
	2833	4/1	29	0.10	12.5	83.3	2.0	0.9	0.3	1.0
	2834	4/1	29	0.23	8.1	87.8	2.0	0.3	0.2	1.6
			N	5	5	5	5	5	5	5
			Mean	0.14	9.6	85.6	2.4	0.9	0.3	1.3
			Sdev	0.054	2.95	2.72	0.61	0.39	0.05	0.30

LUAB - LARGE UNSTAINED CELLS ABS
N - NEUTROPHILS %
LY - LYMPHOCITES %
M - MONOCYTES %
E - EOSINOPHILS %
B - BASOPHILS %
LU - LARGE UNSTAINED CELLS %

Appendix 5
Day 43 Hematology Data
Test period

Session 1 (Scheduled)
Fexinidazole

Study Number: 0504-2007

Males

Dose Level	Animal Number	Group/ Subgroup	Study Day	RBC 10^6/mcL	HGB g/dL	HCT %	MCV fL	MCH pg	MCHC g/dL	RDW %	HDW g/dL
vehicle											
	2745	1/1	43	8.66	15.6	46.8	54.1	18.0	33.2	11.0	2.65
	2746	1/1	43	9.18	15.2	47.1	51.2	16.5	32.3	11.7	2.60
	2747	1/1	43	8.21	15.1	45.8	55.8	18.4	32.9	12.0	2.85
	2748	1/1	43	9.00	15.9	47.6	52.9	17.7	33.4	12.0	2.65
	2749	1/1	43	9.16	16.4	48.6	53.1	17.9	33.8	11.8	2.62
			N	5	5	5	5	5	5	5	5
			Mean	8.84	15.6	47.2	53.4	17.7	33.1	11.7	2.67
			Sdev	0.410	0.53	1.03	1.69	0.72	0.56	0.41	0.101
800 mg/kg											
	2780	4/1	43	8.19	15.3	45.8	55.9	18.6	33.3	11.5	2.74
	2781	4/1	43	8.29	15.7	46.8	56.4	19.0	33.6	12.2	2.98
	2782	4/1	43	9.34	16.6	51.7	55.4	17.8	32.2	11.6	2.87
	2783	4/1	43	8.57	15.1	47.2	55.1	17.6	32.0	12.4	2.75
	2784	4/1	43	8.10	15.3	45.7	56.4	18.8	33.4	12.5	2.66
			N	5	5	5	5	5	5	5	5
			Mean	8.50	15.6	47.4	55.8	18.4	32.9	12.0	2.80
			Sdev	0.503	0.60	2.47	0.59	0.62	0.74	0.46	0.125

RBC - RED BLOOD CELLS
HGB - HEMOGLOBIN
HCT - HEMATOCRIT
MCV - MEAN CORPUSCULAR VOLUME
MCH - MEAN CORPUSCULAR HEMOGLOBIN
MCHC - MEAN CORPUSCULAR HGB CONC.
RDW - RED CELL DISTRIBUTION WIDTH
HDW - HEMOGLOBIN DISTRIB. WIDTH

Appendix 5
Day 43 Hematology Data
Test period

Session 1 (Scheduled)
Fexinidazole

Study Number: 0504-2007

Males

Dose Level	Animal Number	Group/ Subgroup	Study Day	R %	RAB 10^9/L	MCVr fL	CHCM g/dL	CHr pg	PLT 10^3/mcL	MPV fL	PDW %
vehicle											
	2745	1/1	43	2.1	183.9	62.9	30.3	19.0	1343.	6.4	51.8
	2746	1/1	43	2.4	215.3	59.4	30.0	17.8	1110.	6.1	57.7
	2747	1/1	43	3.2	258.4	64.1	30.2	19.4	1261.	6.3	58.0
	2748	1/1	43	2.5	221.0	61.2	30.6	18.7	963.	6.0	54.0
	2749	1/1	43	2.6	237.6	62.3	30.5	18.9	1218.	6.7	56.3
			N	5	5	5	5	5	5	5	5
			Mean	2.5	223.2	62.0	30.3	18.8	1179.	6.3	55.6
			Sdev	0.39	27.65	1.78	0.24	0.59	147.1	0.27	2.63
800 mg/kg											
	2780	4/1	43	3.2	262.7	65.7	30.9	20.3	1201.	6.4	52.2
	2781	4/1	43	3.6	297.0	64.4	31.0	20.0	1144.	6.5	54.4
	2782	4/1	43	2.9	266.3	63.6	30.5	19.3	1082.	6.3	56.7
	2783	4/1	43	2.9	247.9	64.0	29.9	19.2	1253.	6.4	55.4
	2784	4/1	43	3.9	312.4	65.6	30.8	20.2	1348.	6.2	52.4
			N	5	5	5	5	5	5	5	5
			Mean	3.3	277.3	64.7	30.6	19.8	1206.	6.4	54.2
			Sdev	0.44	26.55	0.95	0.44	0.51	102.0	0.11	1.93

R - RETICULOCYTES
RAB - RETICULOCYTES ABS
MCVr - MEAN CORPUSCOLAR VOL. RETIC.
CHCM - MEAN HEMOGLOBIN CONC. RETIC.
CHr - CELLULAR HEMOGLOBIN RETIC.
PLT - PLATELETS
MPV - MEAN PLATELET VOLUME
PDW - PLATELET DISTRIBUTION WIDTH

Appendix 5
Day 43 Hematology Data
Test period

Session 1 (Scheduled)
Fexinidazole

Study Number: 0504-2007

Males

Dose Level	Animal Number	Group/ Subgroup	Study Day	PCT %	WBC 10^3/mcL	NAB 10^3/mcL	LYAB 10^3/mcL	MAB 10^3/mcL	EAB 10^3/mcL	BAB 10^3/mcL
vehicle										
	2745	1/1	43	0.86	9.63	1.69	7.44	0.33	0.07	0.05
	2746	1/1	43	0.68	9.06	1.50	7.15	0.20	0.15	0.02
	2747	1/1	43	0.80	10.73	2.48	7.63	0.38	0.14	0.03
	2748	1/1	43	0.58	12.44	1.95	9.59	0.51	0.19	0.04
	2749	1/1	43	0.82	10.66	1.45	8.83	0.16	0.13	0.02
			N	5	5	5	5	5	5	5
			Mean	0.75	10.50	1.81	8.13	0.32	0.14	0.03
			Sdev	0.115	1.292	0.421	1.038	0.141	0.043	0.013
800 mg/kg										
	2780	4/1	43	0.76	9.94	1.78	7.45	0.38	0.13	0.02
	2781	4/1	43	0.75	12.22	1.92	9.77	0.20	0.18	0.03
	2782	4/1	43	0.68	8.92	1.51	6.96	0.26	0.09	0.04
	2783	4/1	43	0.81	10.98	2.36	7.87	0.46	0.19	0.02
	2784	4/1	43	0.84	10.88	1.88	8.38	0.40	0.10	0.02
			N	5	5	5	5	5	5	5
			Mean	0.77	10.59	1.89	8.09	0.34	0.14	0.03
			Sdev	0.061	1.235	0.308	1.077	0.107	0.045	0.009

PCT - PLATELET HEMATOCRIT
WBC - WHITE BLOOD CELLS
NAB - NEUTROPHILS ABS
LYAB - LYMPHOCYTES ABS
MAB - MONOCYTES ABS
EAB - EOSINOPHILS ABS
BAB - BASOPHILS ABS

Appendix 5
Day 43 Hematology Data
Test period

Session 1 (Scheduled)
Fexinidazole

Study Number: 0504-2007

Males

Dose Level	Animal Number	Group/ Subgroup	Study Day	LUAB 10^3/mcL	N %	LY %	M %	E %	B %	LU %
vehicle										
	2745	1/1	43	0.04	17.6	77.3	3.5	0.8	0.5	0.4
	2746	1/1	43	0.04	16.6	78.9	2.2	1.6	0.2	0.5
	2747	1/1	43	0.08	23.1	71.1	3.5	1.3	0.3	0.7
	2748	1/1	43	0.16	15.7	77.1	4.1	1.5	0.3	1.3
	2749	1/1	43	0.07	13.6	82.9	1.5	1.2	0.2	0.7
			N	5	5	5	5	5	5	5
			Mean	0.08	17.3	77.5	3.0	1.3	0.3	0.7
			Sdev	0.049	3.55	4.25	1.07	0.31	0.12	0.35
800 mg/kg										
	2780	4/1	43	0.19	17.9	74.9	3.8	1.3	0.2	1.9
	2781	4/1	43	0.13	15.7	79.9	1.6	1.5	0.3	1.0
	2782	4/1	43	0.06	16.9	78.1	3.0	1.0	0.4	0.7
	2783	4/1	43	0.08	21.5	71.7	4.2	1.7	0.2	0.8
	2784	4/1	43	0.09	17.3	77.0	3.7	0.9	0.2	0.9
			N	5	5	5	5	5	5	5
			Mean	0.11	17.9	76.3	3.3	1.3	0.3	1.1
			Sdev	0.051	2.19	3.15	1.02	0.33	0.09	0.48

LUAB - LARGE UNSTAINED CELLS ABS
N - NEUTROPHILS %
LY - LYMPHOCITES %
M - MONOCYTES %
E - EOSINOPHILS %
B - BASOPHILS %
LU - LARGE UNSTAINED CELLS %

Appendix 5
Day 43 Hematology Data
Session 1 (Scheduled)
Test period
Fexinidazole
Study Number: 0504-2007

Females

Dose Level	Animal Number	Group/ Subgroup	Study Day	RBC 10^6/mcL	HGB g/dL	HCT %	MCV fL	MCH pg	MCHC g/dL	RDW %	HDW g/dL
vehicle											
	2795	1/1	43	8.33	14.4	43.7	52.5	17.2	32.8	11.4	2.16
	2796	1/1	43	8.53	15.6	47.7	55.9	18.3	32.7	11.4	2.36
	2797	1/1	43	8.10	15.1	46.5	57.3	18.6	32.4	10.9	2.16
	2798	1/1	43	8.20	14.6	45.2	55.1	17.7	32.2	11.6	2.48
	2799	1/1	43	8.07	14.3	44.1	54.6	17.7	32.4	11.6	2.38
			N	5	5	5	5	5	5	5	5
			Mean	8.25	14.8	45.4	55.1	17.9	32.5	11.4	2.31
			Sdev	0.188	0.54	1.67	1.77	0.55	0.24	0.29	0.143
800 mg/kg											
	2830	4/1	43	8.18	14.4	44.3	54.1	17.6	32.5	11.6	2.26
	2831	4/1	43	8.47	15.2	46.7	55.2	17.9	32.5	12.5	2.34
	2832	4/1	43	7.75	13.8	42.9	55.4	17.9	32.2	12.2	2.38
	2833	4/1	43	8.46	15.2	46.0	54.3	17.9	33.0	12.7	2.46
	2834	4/1	43	7.96	14.4	45.1	56.6	18.0	31.8	11.9	2.28
			N	5	5	5	5	5	5	5	5
			Mean	8.16	14.6	45.0	55.1	17.9	32.4	12.2	2.34
			Sdev	0.314	0.60	1.48	1.00	0.15	0.44	0.44	0.080

RBC - RED BLOOD CELLS
HGB - HEMOGLOBIN
HCT - HEMATOCRIT
MCV - MEAN CORPUSCULAR VOLUME
MCH - MEAN CORPUSCULAR HEMOGLOBIN
MCHC - MEAN CORPUSCULAR HGB CONC.
RDW - RED CELL DISTRIBUTION WIDTH
HDW - HEMOGLOBIN DISTRIB. WIDTH

Appendix 5
Day 43 Hematology Data
Test period

Session 1 (Scheduled)
Fexinidazole

Study Number: 0504-2007

Females

Dose Level	Animal Number	Group/ Subgroup	Study Day	R %	RAB 10^9/L	MCVr fL	CHCM g/dL	CHr pg	PLT 10^3/mcL	MPV fL	PDW %
vehicle											
	2795	1/1	43	2.7	221.0	62.0	30.1	18.6	1224.	6.7	50.3
	2796	1/1	43	2.8	235.9	65.9	30.7	20.2	1364.	6.8	53.7
	2797	1/1	43	2.4	196.3	64.9	31.1	20.2	1322.	6.7	52.0
	2798	1/1	43	3.2	263.1	66.6	30.5	20.3	1452.	6.7	51.3
	2799	1/1	43	2.5	201.3	64.7	30.1	19.4	1367.	6.7	51.5
			N	5	5	5	5	5	5	5	5
			Mean	2.7	223.5	64.8	30.5	19.7	1346.	6.7	51.8
			Sdev	0.31	27.21	1.75	0.42	0.73	82.9	0.04	1.25
800 mg/kg											
	2830	4/1	43	2.2	180.0	65.3	29.6	19.2	1438.	6.8	50.3
	2831	4/1	43	1.5	126.9	62.9	29.9	18.7	1602.	7.0	54.1
	2832	4/1	43	2.9	224.5	65.6	30.3	19.8	1268.	6.7	53.1
	2833	4/1	43	2.7	231.2	64.2	29.9	19.2	1333.	6.8	53.7
	2834	4/1	43	2.1	170.1	64.6	30.1	19.4	1466.	6.5	50.0
			N	5	5	5	5	5	5	5	5
			Mean	2.3	186.5	64.5	30.0	19.3	1421.	6.8	52.2
			Sdev	0.55	42.74	1.06	0.26	0.40	128.7	0.18	1.94

R - RETICULOCYTES
RAB - RETICULOCYTES ABS
MCVr - MEAN CORPUSCOLAR VOL. RETIC.
CHCM - MEAN HEMOGLOBIN CONC. RETIC.
CHr - CELLULAR HEMOGLOBIN RETIC.
PLT - PLATELETS
MPV - MEAN PLATELET VOLUME
PDW - PLATELET DISTRIBUTION WIDTH

Appendix 5
Day 43 Hematology Data
Test period

Session 1 (Scheduled)
Fexinidazole

Study Number: 0504-2007

Females

Dose Level	Animal Number	Group/ Subgroup	Study Day	PCT %	WBC 10^3/mcL	NAB 10^3/mcL	LYAB 10^3/mcL	MAB 10^3/mcL	EAB 10^3/mcL	BAB 10^3/mcL
vehicle										
	2795	1/1	43	0.82	13.56	1.72	11.10	0.29	0.14	0.02
	2796	1/1	43	0.92	9.10	1.39	7.23	0.20	0.14	0.02
	2797	1/1	43	0.89	8.88	2.03	6.26	0.27	0.10	0.02
	2798	1/1	43	0.97	11.28	1.26	9.45	0.31	0.14	0.03
	2799	1/1	43	0.91	10.25	1.04	8.82	0.14	0.11	0.03
			N	5	5	5	5	5	5	5
			Mean	0.90	10.61	1.49	8.57	0.24	0.13	0.02
			Sdev	0.054	1.907	0.390	1.895	0.070	0.019	0.005
800 mg/kg										
	2830	4/1	43	0.98	11.37	1.63	9.18	0.31	0.12	0.02
	2831	4/1	43	1.11	12.38	1.49	10.00	0.36	0.16	0.05
	2832	4/1	43	0.85	11.99	1.01	10.16	0.40	0.14	0.02
	2833	4/1	43	0.91	14.11	1.68	11.61	0.48	0.11	0.05
	2834	4/1	43	0.95	17.26	2.42	14.07	0.44	0.03	0.04
			N	5	5	5	5	5	5	5
			Mean	0.96	13.42	1.65	11.00	0.40	0.11	0.04
			Sdev	0.097	2.374	0.507	1.925	0.066	0.050	0.015

PCT - PLATELET HEMATOCRIT
WBC - WHITE BLOOD CELLS
NAB - NEUTROPHILS ABS
LYAB - LYMPHOCYTES ABS
MAB - MONOCYTES ABS
EAB - EOSINOPHILS ABS
BAB - BASOPHILS ABS

Appendix 5
Day 43 Hematology Data
Test period

Session 1 (Scheduled)
Fexinidazole
Study Number: 0504-2007

Females

Dose Level	Animal Number	Group/ Subgroup	Study Day	LUAB 10^3/mcL	N %	LY %	M %	E %	B %	LU %
vehicle										
	2795	1/1	43	0.29	12.7	81.9	2.1	1.0	0.2	2.1
	2796	1/1	43	0.12	15.3	79.5	2.2	1.6	0.2	1.3
	2797	1/1	43	0.19	22.8	70.5	3.0	1.2	0.3	2.2
	2798	1/1	43	0.09	11.2	83.7	2.7	1.2	0.2	0.8
	2799	1/1	43	0.11	10.1	86.1	1.3	1.1	0.3	1.1
			N	5	5	5	5	5	5	5
			Mean	0.16	14.4	80.3	2.3	1.2	0.2	1.5
			Sdev	0.082	5.07	6.01	0.65	0.23	0.05	0.62
800 mg/kg										
	2830	4/1	43	0.12	14.3	80.7	2.7	1.0	0.2	1.1
	2831	4/1	43	0.34	12.0	80.7	2.9	1.3	0.4	2.7
	2832	4/1	43	0.25	8.5	84.8	3.4	1.2	0.2	2.1
	2833	4/1	43	0.18	11.9	82.3	3.4	0.8	0.3	1.3
	2834	4/1	43	0.27	14.0	81.5	2.5	0.2	0.2	1.6
			N	5	5	5	5	5	5	5
			Mean	0.23	12.1	82.0	3.0	0.9	0.3	1.8
			Sdev	0.085	2.32	1.70	0.41	0.44	0.09	0.65

LUAB - LARGE UNSTAINED CELLS ABS
N - NEUTROPHILS %
LY - LYMPHOCITES %
M - MONOCYTES %
E - EOSINOPHILS %
B - BASOPHILS %
LU - LARGE UNSTAINED CELLS %

Appendix 6 Clinical Chemistry

Appendix 6
Day 29 Clinical Chemistry Data
Test period

Session 1 (Scheduled)
Fexinidazole
Study Number: 0504-2007

Males

Dose Level	Animal Number	Group/ Subgroup	Study Day	UREA mg/dL	CREA mg/dL	AST IU/L	ALT IU/L	AP IU/L	GGT IU/L	TBIL mg/dL
vehicle										
	2745	1/1	29	24.	0.59	132.	24.	187.	6.	0.11
	2746	1/1	29	35.	0.56	128.	28.	203.	7.	0.09
	2747	1/1	29	25.	0.54	129.	26.	139.	5.	0.08
	2748	1/1	29	31.	0.51	170.	39.	188.	7.	0.06
	2749	1/1	29	27.	0.53	178.	22.	221.	1.	0.09
			N	5	5	5	5	5	5	5
			Mean	28.	0.55	147.	28.	188.	5.	0.09
			Sdev	4.6	0.030	24.5	6.6	30.5	2.5	0.018
50 mg/kg										
	2755	2/1	29	28.	0.58	128.	25.	131.	NT	0.04
	2756	2/1	29	28.	0.56	150.	36.	213.	5.	0.05
	2757	2/1	29	29.	0.57	160.	25.	115.	5.	0.06
	2758	2/1	29	23.	0.49	116.	32.	93.	NT	0.06
	2759	2/1	29	29.	0.59	108.	24.	170.	NT	0.07
			N	5	5	5	5	5	2	5
			Mean	27.	0.56	132.	28.	144.	5.	0.06
			Sdev	2.5	0.040	22.1	5.3	47.6	0.0	0.011
200 mg/kg										
	2765	3/1	29	28.	0.54	143.	30.	120.	NT	0.05
	2766	3/1	29	30.	0.56	125.	25.	128.	NT	0.08
	2767	3/1	29	31.	0.56	150.	34.	128.	NT	0.08
	2768	3/1	29	29.	0.61	101.	24.	139.	NT	0.07
	2769	3/1	29	30.	0.54	135.	31.	147.	NT	0.05
			N	5	5	5	5	5	0	5
			Mean	30.	0.56	131.	29.	132.	-	0.07
			Sdev	1.1	0.029	19.1	4.2	10.6	-	0.015

UREA - UREA
CREA - CREATININE
AST - ASPARTATE AMINO TRANSFERASE
ALT - ALANINE AMINO TRANSFERASE
AP - ALKALINE PHOSPHATASE
GGT - GAMMA GLUTAMYL TRANSFERASE
TBIL - TOTAL BILIRUBIN

Appendix 6
Day 29 Clinical Chemistry Data
Test period

Session 1 (Scheduled)
Fexinidazole

Study Number: 0504-2007

M a l e s

Dose Level	Animal Number	Group/ Subgroup	Study Day	UREA mg/dL	CREA mg/dL	AST IU/L	ALT IU/L	AP IU/L	GGT IU/L	TBIL mg/dL
800 mg/kg										
	2780	4/1	29	27.	0.60	122.	22.	150.	NT	0.07
	2781	4/1	29	27.	0.55	159.	27.	96.	NT	0.07
	2782	4/1	29	30.	0.54	101.	25.	122.	NT	0.06
	2783	4/1	29	24.	0.65	123.	21.	93.	NT	0.08
	2784	4/1	29	29.	0.55	114.	29.	140.	5.	0.06
			N	5	5	5	5	5	1	5
			Mean	27.	0.58	124.	25.	120.	5.	0.07
			Sdev	2.3	0.047	21.6	3.3	25.5	-	0.008

UREA - UREA
CREA - CREATININE
AST - ASPARTATE AMINO TRANSFERASE
ALT - ALANINE AMINO TRANSFERASE
AP - ALKALINE PHOSPHATASE
GGT - GAMMA GLUTAMYL TRANSFERASE
TBIL - TOTAL BILIRUBIN

Appendix 6
Day 29 Clinical Chemistry Data
Test period

Session 1 (Scheduled)
Fexinidazole
Study Number: 0504-2007

Males

Dose Level	Animal Number	Group/ Subgroup	Study Day	TPRO g/dL	ALB g/dL	GLOB g/dL	GLUC mg/dL	TG mg/dL	TCHO mg/dL
vehicle									
	2745	1/1	29	6.9	3.20	3.7	85.	74.	62.
	2746	1/1	29	6.6	3.10	3.5	73.	34.	52.
	2747	1/1	29	6.8	3.15	3.7	87.	78.	53.
	2748	1/1	29	6.3	2.87	3.4	74.	58.	56.
	2749	1/1	29	6.7	3.14	3.6	83.	40.	52.
			N	5	5	5	5	5	5
			Mean	6.7	3.09	3.6	80.	57.	55.
			Sdev	0.23	0.129	0.11	6.5	19.7	4.2
50 mg/kg									
	2755	2/1	29	6.8	3.01	3.8	82.	67.	55.
	2756	2/1	29	6.7	3.10	3.6	88.	59.	74.
	2757	2/1	29	7.1	3.21	3.9	75.	45.	47.
	2758	2/1	29	6.6	2.99	3.6	74.	34.	40.
	2759	2/1	29	6.6	3.20	3.4	88.	77.	55.
			N	5	5	5	5	5	5
			Mean	6.8	3.10	3.7	81.	56.	54.
			Sdev	0.21	0.103	0.19	6.8	17.1	12.7
200 mg/kg									
	2765	3/1	29	6.8	3.11	3.7	74.	42.	57.
	2766	3/1	29	7.0	3.15	3.9	87.	33.	54.
	2767	3/1	29	6.5	3.17	3.3	89.	28.	40.
	2768	3/1	29	6.8	3.18	3.6	94.	87.	76.
	2769	3/1	29	7.0	3.19	3.8	78.	26.	43.
			N	5	5	5	5	5	5
			Mean	6.8	3.16	3.7	84.	43.	54.
			Sdev	0.20	0.032	0.21	8.2	25.3	14.2

TPRO - TOTAL PROTEIN
ALB - ALBUMIN
GLOB - GLOBULIN
GLUC - GLUCOSE
TG - TRIGLYCERIDES
TCHO - TOTAL CHOLESTEROL

Appendix 6
Day 29 Clinical Chemistry Data
Test period

Session 1 (Scheduled)
Fexinidazole

Study Number: 0504-2007

Males

Dose Level	Animal Number	Group/ Subgroup	Study Day	TPRO g/dL	ALB g/dL	GLOB g/dL	GLUC mg/dL	TG mg/dL	TCHO mg/dL
800 mg/kg									
	2780	4/1	29	7.0	3.24	3.8	79.	42.	93.
	2781	4/1	29	7.2	3.34	3.9	79.	21.	56.
	2782	4/1	29	6.9	3.08	3.8	76.	14.	59.
	2783	4/1	29	7.1	3.21	3.9	81.	31.	63.
	2784	4/1	29	6.6	3.00	3.6	73.	32.	48.
			N	5	5	5	5	5	5
			Mean	7.0	3.17	3.8	78.	28.	64.
			Sdev	0.23	0.134	0.11	3.1	10.8	17.2

TPRO - TOTAL PROTEIN
ALB - ALBUMIN
GLOB - GLOBULIN
GLUC - GLUCOSE
TG - TRIGLYCERIDES
TCHO - TOTAL CHOLESTEROL

Appendix 6
Day 29 Clinical Chemistry Data
Test period

Session 1 (Scheduled)
Fexinidazole

Study Number: 0504-2007

Males

Dose Level	Animal Number	Group/ Subgroup	Study Day	CA mg/dL	PHOS mg/dL	AG	NA mEq/L	K mEq/L	CL mEq/L
vehicle									
	2745	1/1	29	10.5	8.6	0.9	140.0	4.9	102.0
	2746	1/1	29	9.9	8.6	0.9	139.0	4.5	103.0
	2747	1/1	29	10.3	8.7	0.9	140.0	4.8	103.0
	2748	1/1	29	10.2	8.9	0.8	139.0	4.9	102.0
	2749	1/1	29	10.2	9.0	0.9	140.0	5.0	103.0
			N	5	5	5	5	5	5
			Mean	10.2	8.8	0.9	139.6	4.8	102.6
			Sdev	0.22	0.18	0.02	0.55	0.17	0.55
50 mg/kg									
	2755	2/1	29	10.3	9.1	0.8	140.0	4.9	101.0
	2756	2/1	29	10.2	7.9	0.9	141.0	4.7	103.0
	2757	2/1	29	10.1	8.7	0.8	141.0	5.1	102.0
	2758	2/1	29	10.2	8.5	0.8	141.0	5.0	102.0
	2759	2/1	29	10.2	9.5	0.9	142.0	5.2	103.0
			N	5	5	5	5	5	5
			Mean	10.2	8.7	0.9	141.0	5.0	102.2
			Sdev	0.07	0.61	0.06	0.71	0.18	0.84
200 mg/kg									
	2765	3/1	29	9.5	8.3	0.8	140.0	4.8	103.0
	2766	3/1	29	10.1	7.8	0.8	142.0	4.4	102.0
	2767	3/1	29	9.8	7.9	1.0	142.0	4.4	104.0
	2768	3/1	29	10.3	8.0	0.9	141.0	4.9	104.0
	2769	3/1	29	10.5	8.6	0.8	143.0	5.3	104.0
			N	5	5	5	5	5	5
			Mean	10.0	8.1	0.9	141.6	4.8	103.4
			Sdev	0.40	0.33	0.05	1.14	0.36	0.89

CA - CALCIUM
PHOS - PHOSPHOROUS
AG - ALBUMIN/GLOBULIN
NA - SODIUM
K - POTASSIUM
CL - CHLORIDE

Appendix 6
Day 29 Clinical Chemistry Data
Test period

Session 1 (Scheduled)
Fexinidazole

Study Number: 0504-2007

Males

Dose Level	Animal Number	Group/ Subgroup	Study Day	CA mg/dL	PHOS mg/dL	AG	NA mEq/L	K mEq/L	CL mEq/L
800 mg/kg									
	2780	4/1	29	10.1	8.0	0.9	143.0	5.0	103.0
	2781	4/1	29	12.1	15.3	0.9	143.0	5.6	103.0
	2782	4/1	29	10.0	12.8	0.8	143.0	5.1	104.0
	2783	4/1	29	9.6	11.8	0.8	142.0	4.9	104.0
	2784	4/1	29	9.5	9.2	0.8	144.0	4.7	103.0
			N	5	5	5	5	5	5
			Mean	10.3	11.4	0.8	143.0	5.1	103.4
			Sdev	1.06	2.90	0.02	0.71	0.33	0.55

CA - CALCIUM
PHOS - PHOSPHOROUS
AG - ALBUMIN/GLOBULIN
NA - SODIUM
K - POTASSIUM
CL - CHLORIDE

Appendix 6
Day 29 Clinical Chemistry Data
Test period

Session 1 (Scheduled)
Fexinidazole

Study Number: 0504-2007

Females

Dose Level	Animal Number	Group/ Subgroup	Study Day	UREA mg/dL	CREA mg/dL	AST IU/L	ALT IU/L	AP IU/L	GGT IU/L	TBIL mg/dL
vehicle										
	2795	1/1	29	41.	0.61	118.	29.	120.	6.	0.12
	2796	1/1	29	35.	0.62	129.	32.	89.	5.	0.14
	2797	1/1	29	33.	0.57	87.	25.	64.	5.	0.16
	2798	1/1	29	31.	0.64	159.	36.	101.	6.	0.07
	2799	1/1	29	32.	0.64	118.	21.	86.	5.	0.11
			N	5	5	5	5	5	5	5
			Mean	34.	0.62	122.	29.	92.	5.	0.12
			Sdev	4.0	0.029	25.9	5.9	20.6	0.5	0.034
50 mg/kg										
	2805	2/1	29	28.	0.60	129.	23.	80.	NT	0.10
	2806	2/1	29	34.	0.63	149.	28.	97.	5.	0.10
	2807	2/1	29	38.	0.67	116.	33.	93.	4.	0.12
	2808	2/1	29	34.	0.71	101.	25.	68.	NT	0.12
	2809	2/1	29	32.	0.65	136.	27.	71.	NT	0.11
			N	5	5	5	5	5	2	5
			Mean	33.	0.65	126.	27.	82.	5.	0.11
			Sdev	3.6	0.041	18.5	3.8	12.9	0.7	0.010
200 mg/kg										
	2815	3/1	29	35.	0.79	118.	30.	59.	NT	0.13
	2816	3/1	29	32.	0.74	150.	26.	95.	5.	0.10
	2817	3/1	29	27.	0.73	102.	23.	58.	NT	0.14
	2818	3/1	29	31.	0.74	152.	27.	48.	NT	0.11
	2819	3/1	29	29.	0.67	128.	23.	115.	5.	0.09
			N	5	5	5	5	5	2	5
			Mean	31.	0.73	130.	26.	75.	5.	0.11
			Sdev	3.0	0.043	21.3	2.9	28.6	0.0	0.021

UREA - UREA
CREA - CREATININE
AST - ASPARTATE AMINO TRANSFERASE
ALT - ALANINE AMINO TRANSFERASE
AP - ALKALINE PHOSPHATASE
GGT - GAMMA GLUTAMYL TRANSFERASE
TBIL - TOTAL BILIRUBIN

Appendix 6
Day 29 Clinical Chemistry Data
Test period

Session 1 (Scheduled)
Fexinidazole
Study Number: 0504-2007

Females

Dose Level	Animal Number	Group/ Subgroup	Study Day	UREA mg/dL	CREA mg/dL	AST IU/L	ALT IU/L	AP IU/L	GGT IU/L	TBIL mg/dL
800 mg/kg										
	2830	4/1	29	34.	0.71	113.	22.	61.	NT	0.10
	2831	4/1	29	43.	0.81	93.	23.	62.	4.	0.10
	2832	4/1	29	34.	0.83	138.	23.	60.	NT	0.12
	2833	4/1	29	34.	0.78	129.	22.	75.	NT	0.10
	2834	4/1	29	40.	0.80	98.	25.	47.	NT	0.11
			N	5	5	5	5	5	1	5
			Mean	37.	0.79	114.	23.	61.	4.	0.11
			Sdev	4.2	0.046	19.4	1.2	9.9	-	0.009

UREA - UREA
CREA - CREATININE
AST - ASPARTATE AMINO TRANSFERASE
ALT - ALANINE AMINO TRANSFERASE
AP - ALKALINE PHOSPHATASE
GGT - GAMMA GLUTAMYL TRANSFERASE
TBIL - TOTAL BILIRUBIN

Appendix 6
Day 29 Clinical Chemistry Data
Test period

Session 1 (Scheduled)
Fexinidazole

Study Number: 0504-2007

Females

Dose Level	Animal Number	Group/ Subgroup	Study Day	TPRO g/dL	ALB g/dL	GLOB g/dL	GLUC mg/dL	TG mg/dL	TCHO mg/dL
vehicle									
	2795	1/1	29	7.6	3.50	4.1	107.	31.	61.
	2796	1/1	29	7.2	3.48	3.7	84.	28.	87.
	2797	1/1	29	7.6	3.68	3.9	93.	41.	57.
	2798	1/1	29	6.9	3.16	3.7	80.	32.	57.
	2799	1/1	29	7.2	3.47	3.7	89.	38.	90.
			N	5	5	5	5	5	5
			Mean	7.3	3.46	3.8	91.	34.	70.
			Sdev	0.30	0.187	0.17	10.4	5.3	16.6
50 mg/kg									
	2805	2/1	29	7.2	3.43	3.8	99.	41.	75.
	2806	2/1	29	7.3	3.26	4.0	87.	47.	73.
	2807	2/1	29	7.7	3.67	4.0	90.	42.	85.
	2808	2/1	29	8.3	4.07	4.2	87.	59.	106.
	2809	2/1	29	8.0	3.73	4.3	90.	49.	99.
			N	5	5	5	5	5	5
			Mean	7.7	3.63	4.1	91.	48.	88.
			Sdev	0.46	0.309	0.20	4.9	7.2	14.6
200 mg/kg									
	2815	3/1	29	7.5	3.93	3.6	97.	54.	117.
	2816	3/1	29	7.7	3.39	4.3	90.	36.	81.
	2817	3/1	29	8.1	3.96	4.1	103.	49.	97.
	2818	3/1	29	7.6	3.65	4.0	98.	74.	79.
	2819	3/1	29	7.1	3.53	3.6	88.	48.	75.
			N	5	5	5	5	5	5
			Mean	7.6	3.69	3.9	95.	52.	90.
			Sdev	0.36	0.249	0.33	6.1	13.9	17.4

TPRO - TOTAL PROTEIN
ALB - ALBUMIN
GLOB - GLOBULIN
GLUC - GLUCOSE
TG - TRIGLYCERIDES
TCHO - TOTAL CHOLESTEROL

Appendix 6
Day 29 Clinical Chemistry Data
Test period

Session 1 (Scheduled)
Fexinidazole
Study Number: 0504-2007

Females

Dose Level	Animal Number	Group/ Subgroup	Study Day	TPRO g/dL	ALB g/dL	GLOB g/dL	GLUC mg/dL	TG mg/dL	TCHO mg/dL
800 mg/kg									
	2830	4/1	29	7.7	3.61	4.1	102.	47.	99.
	2831	4/1	29	8.5	3.98	4.5	98.	49.	134.
	2832	4/1	29	8.0	3.84	4.2	104.	68.	117.
	2833	4/1	29	7.9	3.66	4.2	119.	29.	97.
	2834	4/1	29	8.1	3.80	4.3	103.	29.	85.
			N	5	5	5	5	5	5
			Mean	8.0	3.78	4.3	105.	44.	106.
			Sdev	0.30	0.148	0.16	8.0	16.3	19.2

TPRO - TOTAL PROTEIN
ALB - ALBUMIN
GLOB - GLOBULIN
GLUC - GLUCOSE
TG - TRIGLYCERIDES
TCHO - TOTAL CHOLESTEROL

Appendix 6
Day 29 Clinical Chemistry Data
Test period

Session 1 (Scheduled)
Fexinidazole

Study Number: 0504-2007

Females

Dose Level	Animal Number	Group/ Subgroup	Study Day	CA mg/dL	PHOS mg/dL	AG	NA mEq/L	K mEq/L	CL mEq/L
vehicle									
	2795	1/1	29	10.7	6.3	0.9	138.0	4.4	102.0
	2796	1/1	29	10.4	6.7	0.9	139.0	4.8	102.0
	2797	1/1	29	10.8	6.6	0.9	140.0	4.6	102.0
	2798	1/1	29	10.3	6.5	0.8	139.0	4.7	102.0
	2799	1/1	29	10.6	7.4	0.9	140.0	4.7	103.0
			N	5	5	5	5	5	5
			Mean	10.6	6.7	0.9	139.2	4.7	102.2
			Sdev	0.21	0.42	0.05	0.84	0.14	0.45
50 mg/kg									
	2805	2/1	29	10.4	6.6	0.9	139.0	4.8	101.0
	2806	2/1	29	10.0	6.3	0.8	140.0	4.5	103.0
	2807	2/1	29	10.4	6.3	0.9	139.0	4.6	103.0
	2808	2/1	29	11.6	7.3	1.0	143.0	5.3	104.0
	2809	2/1	29	11.0	7.0	0.9	143.0	4.6	104.0
			N	5	5	5	5	5	5
			Mean	10.7	6.7	0.9	140.8	4.8	103.0
			Sdev	0.63	0.44	0.06	2.05	0.32	1.22
200 mg/kg									
	2815	3/1	29	11.1	6.7	1.1	143.0	4.6	105.0
	2816	3/1	29	10.4	6.9	0.8	142.0	5.1	105.0
	2817	3/1	29	11.4	7.6	1.0	143.0	4.7	103.0
	2818	3/1	29	10.3	7.1	0.9	142.0	4.9	102.0
	2819	3/1	29	10.1	8.0	1.0	142.0	4.8	103.0
			N	5	5	5	5	5	5
			Mean	10.7	7.3	1.0	142.4	4.8	103.6
			Sdev	0.56	0.53	0.11	0.55	0.19	1.34

CA - CALCIUM
PHOS - PHOSPHOROUS
AG - ALBUMIN/GLOBULIN
NA - SODIUM
K - POTASSIUM
CL - CHLORIDE

Appendix 6
Day 29 Clinical Chemistry Data
Test period

Session 1 (Scheduled)
Fexinidazole

Study Number: 0504-2007

Females

Dose Level	Animal Number	Group/ Subgroup	Study Day	CA mg/dL	PHOS mg/dL	AG	NA mEq/L	K mEq/L	CL mEq/L
800 mg/kg									
	2830	4/1	29	11.2	6.0	0.9	142.0	4.7	105.0
	2831	4/1	29	11.8	6.7	0.9	145.0	5.0	104.0
	2832	4/1	29	11.5	7.2	0.9	143.0	5.2	104.0
	2833	4/1	29	11.1	6.9	0.9	142.0	5.0	104.0
	2834	4/1	29	10.9	6.1	0.9	144.0	4.8	106.0
			N	5	5	5	5	5	5
			Mean	11.3	6.6	0.9	143.2	4.9	104.6
			Sdev	0.35	0.52	0.02	1.30	0.20	0.89

CA - CALCIUM
NA - SODIUM
PHOS - PHOSPHOROUS
K - POTASSIUM
AG - ALBUMIN/GLOBULIN
CL - CHLORIDE

Appendix 6
Day 43 Clinical Chemistry Data
Test period

Session 1 (Scheduled)
Fexinidazole

Study Number: 0504-2007

M a l e s

Dose Level	Animal Number	Group/ Subgroup	Study Day	UREA mg/dL	CREA mg/dL	AST IU/L	ALT IU/L	AP IU/L	GGT IU/L	TBIL mg/dL
vehicle										
	2745	1/1	43	28.	0.70	134.	28.	137.	NT	0.08
	2746	1/1	43	33.	0.65	140.	36.	148.	6.	0.09
	2747	1/1	43	27.	0.68	141.	34.	115.	NT	0.08
	2748	1/1	43	32.	0.67	145.	37.	152.	4.	0.05
	2749	1/1	43	30.	0.68	184.	29.	175.	7.	0.08
			N	5	5	5	5	5	3	5
			Mean	30.	0.68	149.	33.	145.	6.	0.08
			Sdev	2.5	0.018	20.1	4.1	21.9	1.5	0.015
800 mg/kg										
	2780	4/1	43	30.	0.69	128.	29.	133.	NT	0.08
	2781	4/1	43	32.	0.70	176.	35.	101.	NT	0.08
	2782	4/1	43	34.	0.64	116.	35.	122.	NT	NT
	2783	4/1	43	30.	0.68	136.	25.	99.	NT	0.11
	2784	4/1	43	35.	0.63	122.	35.	135.	NT	0.09
			N	5	5	5	5	5	0	4
			Mean	32.	0.67	136.	32.	118.	-	0.09
			Sdev	2.3	0.031	23.8	4.6	17.2	-	0.014

UREA - UREA
CREA - CREATININE
AST - ASPARTATE AMINO TRANSFERASE
ALT - ALANINE AMINO TRANSFERASE
AP - ALKALINE PHOSPHATASE
GGT - GAMMA GLUTAMYL TRANSFERASE
TBIL - TOTAL BILIRUBIN

Appendix 6
Day 43 Clinical Chemistry Data
Test period

Session 1 (Scheduled)
Fexinidazole
Study Number: 0504-2007

Males

Dose Level	Animal Number	Group/ Subgroup	Study Day	TPRO g/dL	ALB g/dL	GLOB g/dL	GLUC mg/dL	TG mg/dL	TCHO mg/dL
vehicle									
	2745	1/1	43	7.1	3.06	4.0	95.	45.	52.
	2746	1/1	43	6.6	3.03	3.6	85.	33.	49.
	2747	1/1	43	7.2	3.12	4.1	95.	57.	52.
	2748	1/1	43	6.8	2.99	3.8	87.	77.	58.
	2749	1/1	43	6.9	3.13	3.8	95.	51.	42.
			N	5	5	5	5	5	5
			Mean	6.9	3.07	3.9	91.	53.	51.
			Sdev	0.24	0.059	0.21	5.0	16.3	5.8
800 mg/kg									
	2780	4/1	43	6.9	3.11	3.8	89.	53.	79.
	2781	4/1	43	6.9	3.05	3.9	79.	53.	54.
	2782	4/1	43	6.9	3.14	3.8	80.	43.	50.
	2783	4/1	43	7.2	3.11	4.1	87.	90.	70.
	2784	4/1	43	7.3	3.13	4.2	81.	62.	47.
			N	5	5	5	5	5	5
			Mean	7.0	3.11	3.9	83.	60.	60.
			Sdev	0.19	0.035	0.19	4.5	18.0	13.8

TPRO - TOTAL PROTEIN
ALB - ALBUMIN
GLOB - GLOBULIN
GLUC - GLUCOSE
TG - TRIGLYCERIDES
TCHO - TOTAL CHOLESTEROL

Appendix 6
Day 43 Clinical Chemistry Data
Test period

Session 1 (Scheduled)
Fexinidazole

Study Number: 0504-2007

Males

Dose Level	Animal Number	Group/ Subgroup	Study Day	CA mg/dL	PHOS mg/dL	AG	NA mEq/L	K mEq/L	CL mEq/L
vehicle									
	2745	1/1	43	10.3	7.5	0.8	138.0	4.6	102.0
	2746	1/1	43	10.1	8.5	0.8	139.0	4.8	101.0
	2747	1/1	43	10.4	7.8	0.8	139.0	5.1	102.0
	2748	1/1	43	10.5	8.7	0.8	139.0	5.2	101.0
	2749	1/1	43	10.4	8.2	0.8	140.0	5.3	103.0
			N	5	5	5	5	5	5
			Mean	10.3	8.1	0.8	139.0	5.0	101.8
			Sdev	0.15	0.49	0.04	0.71	0.31	0.84
800 mg/kg									
	2780	4/1	43	10.2	7.8	0.8	141.0	5.1	102.0
	2781	4/1	43	10.3	7.9	0.8	140.0	5.1	102.0
	2782	4/1	43	10.3	8.2	0.8	140.0	5.0	104.0
	2783	4/1	43	10.8	9.8	0.8	142.0	5.0	103.0
	2784	4/1	43	10.3	9.0	0.8	141.0	4.9	102.0
			N	5	5	5	5	5	5
			Mean	10.4	8.5	0.8	140.8	5.0	102.6
			Sdev	0.24	0.85	0.04	0.84	0.10	0.89

CA - CALCIUM
PHOS - PHOSPHOROUS
AG - ALBUMIN/GLOBULIN
NA - SODIUM
K - POTASSIUM
CL - CHLORIDE

Appendix 6
Day 43 Clinical Chemistry Data
Test period

Session 1 (Scheduled)
Fexinidazole
Study Number: 0504-2007

Females

Dose Level	Animal Number	Group/ Subgroup	Study Day	UREA mg/dL	CREA mg/dL	AST IU/L	ALT IU/L	AP IU/L	GGT IU/L	TBIL mg/dL
vehicle										
	2795	1/1	43	36.	0.62	128.	41.	106.	6.	0.10
	2796	1/1	43	35.	0.64	140.	30.	89.	NT	0.08
	2797	1/1	43	35.	0.70	110.	30.	65.	4.	0.11
	2798	1/1	43	34.	0.63	180.	36.	95.	NT	0.08
	2799	1/1	43	43.	0.72	120.	22.	79.	NT	0.10
			N	5	5	5	5	5	2	5
			Mean	37.	0.66	136.	32.	87.	5.	0.09
			Sdev	3.6	0.045	27.1	7.2	15.6	1.4	0.013
800 mg/kg										
	2830	4/1	43	30.	0.64	135.	33.	56.	NT	0.09
	2831	4/1	43	40.	0.69	123.	28.	56.	NT	0.09
	2832	4/1	43	35.	0.76	168.	25.	66.	7.	0.08
	2833	4/1	43	42.	0.73	166.	28.	79.	NT	0.13
	2834	4/1	43	47.	0.69	118.	27.	46.	NT	0.11
			N	5	5	5	5	5	1	5
			Mean	39.	0.70	142.	28.	61.	7.	0.10
			Sdev	6.5	0.045	23.7	2.9	12.5	-	0.020

UREA - UREA
CREA - CREATININE
AST - ASPARTATE AMINO TRANSFERASE
ALT - ALANINE AMINO TRANSFERASE
AP - ALKALINE PHOSPHATASE
GGT - GAMMA GLUTAMYL TRANSFERASE
TBIL - TOTAL BILIRUBIN

Appendix 6
Day 43 Clinical Chemistry Data
Test period

Session 1 (Scheduled)
Fexinidazole

Study Number: 0504-2007

Females

Dose Level	Animal Number	Group/ Subgroup	Study Day	TPRO g/dL	ALB g/dL	GLOB g/dL	GLUC mg/dL	TG mg/dL	TCHO mg/dL
vehicle									
	2795	1/1	43	7.6	3.64	4.0	115.	29.	63.
	2796	1/1	43	7.1	3.45	3.7	100.	33.	80.
	2797	1/1	43	7.7	3.81	3.9	105.	49.	64.
	2798	1/1	43	7.3	3.32	4.0	110.	35.	59.
	2799	1/1	43	7.6	3.59	4.0	95.	47.	93.
			N	5	5	5	5	5	5
			Mean	7.5	3.56	3.9	105.	39.	72.
			Sdev	0.25	0.187	0.15	7.9	8.9	14.3
800 mg/kg									
	2830	4/1	43	7.3	3.28	4.0	99.	40.	68.
	2831	4/1	43	7.8	3.80	4.0	95.	41.	90.
	2832	4/1	43	7.6	3.69	3.9	108.	41.	98.
	2833	4/1	43	7.9	3.72	4.2	112.	50.	97.
	2834	4/1	43	7.7	3.84	3.9	105.	40.	83.
			N	5	5	5	5	5	5
			Mean	7.7	3.67	4.0	104.	42.	87.
			Sdev	0.23	0.224	0.12	6.8	4.3	12.3

TPRO - TOTAL PROTEIN
ALB - ALBUMIN
GLOB - GLOBULIN
GLUC - GLUCOSE
TG - TRIGLYCERIDES
TCHO - TOTAL CHOLESTEROL

Appendix 6
Day 43 Clinical Chemistry Data
Test period

Session 1 (Scheduled)
Fexinidazole
Study Number: 0504-2007

Females

Dose Level	Animal Number	Group/ Subgroup	Study Day	CA mg/dL	PHOS mg/dL	AG	NA mEq/L	K mEq/L	CL mEq/L
vehicle									
	2795	1/1	43	10.8	6.2	0.9	142.0	4.7	103.0
	2796	1/1	43	10.5	6.5	0.9	144.0	5.0	105.0
	2797	1/1	43	10.8	6.0	1.0	143.0	4.6	104.0
	2798	1/1	43	10.7	6.7	0.8	142.0	5.1	105.0
	2799	1/1	43	10.9	7.2	0.9	144.0	4.7	105.0
			N	5	5	5	5	5	5
			Mean	10.7	6.5	0.9	143.0	4.8	104.4
			Sdev	0.15	0.47	0.05	1.00	0.24	0.89
800 mg/kg									
	2830	4/1	43	10.5	6.5	0.8	141.0	4.8	104.0
	2831	4/1	43	11.1	7.0	1.0	144.0	4.8	105.0
	2832	4/1	43	11.1	5.6	0.9	142.0	5.0	104.0
	2833	4/1	43	11.5	6.9	0.9	144.0	5.3	104.0
	2834	4/1	43	11.2	6.9	1.0	145.0	4.7	106.0
			N	5	5	5	5	5	5
			Mean	11.1	6.6	0.9	143.2	4.9	104.6
			Sdev	0.36	0.58	0.07	1.64	0.24	0.89

CA - CALCIUM
PHOS - PHOSPHOROUS
AG - ALBUMIN/GLOBULIN
NA - SODIUM
K - POTASSIUM
CL - CHLORIDE

Appendix 7 Urine Analysis

Appendix 7
Day 29 Urine Data
Test period

Session 1 (Scheduled)
Fexinidazole

Study Number: 0504-2007

Males

Dose Level	Animal Number	Group/ Subgroup	Study Day	PH UNITS	WBC SCORE	NIT SCORE	PRO SCORE	GLU SCORE	KET SCORE	UBG SCORE
vehicle										
	2745	1/1	29	7.0	0.	0.	1.	0.	1.	0.
	2746	1/1	29	7.0	0.	0.	0.	0.	0.	0.
	2747	1/1	29	7.0	0.	0.	1.	0.	0.	0.
	2748	1/1	29	7.0	0.	0.	1.	0.	0.	0.
	2749	1/1	29	7.0	0.	0.	0.	0.	0.	0.
			N	5	5	5	5	5	5	5
			Mean	7.0	0.	0.	1.	0.	0.	0.
			Sdev	0.00	0.0	0.0	0.5	0.0	0.4	0.0
50 mg/kg										
	2755	2/1	29	7.0	0.	0.	1.	0.	1.	0.
	2756	2/1	29	7.0	0.	1.	1.	0.	1.	0.
	2757	2/1	29	7.0	1.	0.	1.	0.	0.	0.
	2758	2/1	29	7.0	0.	0.	1.	0.	0.	0.
	2759	2/1	29	7.0	0.	0.	1.	0.	0.	0.
			N	5	5	5	5	5	5	5
			Mean	7.0	0.	0.	1.	0.	0.	0.
			Sdev	0.00	0.4	0.4	0.0	0.0	0.5	0.0
200 mg/kg										
	2765	3/1	29	7.0	0.	0.	1.	0.	1.	0.
	2766	3/1	29	7.0	0.	0.	1.	0.	0.	0.
	2767	3/1	29	7.0	0.	0.	1.	0.	1.	0.
	2768	3/1	29	7.0	0.	0.	1.	0.	0.	0.
	2769	3/1	29	7.0	0.	1.	1.	0.	1.	0.
			N	5	5	5	5	5	5	5
			Mean	7.0	0.	0.	1.	0.	1.	0.
			Sdev	0.00	0.0	0.4	0.0	0.0	0.5	0.0

PH - PH
WBC - WHITE BLOOD CELLS
NIT - NITRITES
PRO - PROTEINS
GLU - GLUCOSE
KET - KETONE BODIES
UBG - UROBILINOGEN

Appendix 7
Day 29 Urine Data
Test period

Session 1 (Scheduled)
Fexinidazole

Study Number: 0504-2007

M a l e s

Dose Level	Animal Number	Group/ Subgroup	Study Day	PH UNITS	WBC SCORE	NIT SCORE	PRO SCORE	GLU SCORE	KET SCORE	UBG SCORE
800 mg/kg										
	2780	4/1	29	7.0	1.	1.	1.	0.	1.	0.
	2781	4/1	29	7.0	1.	1.	1.	0.	0.	0.
	2782	4/1	29	7.0	1.	0.	1.	0.	1.	0.
	2783	4/1	29	7.0	1.	0.	1.	0.	1.	0.
	2784	4/1	29	7.0	0.	1.	1.	0.	0.	0.
			N	5	5	5	5	5	5	5
			Mean	7.0	1.	1.	1.	0.	1.	0.
			Sdev	0.00	0.4	0.5	0.0	0.0	0.5	0.0

PH - PH
PRO - PROTEINS
UBG - UROBILINOGEN
WBC - WHITE BLOOD CELLS
GLU - GLUCOSE
NIT - NITRITES
KET - KETONE BODIES

Appendix 7
Day 29 Urine Data
Test period

Session 1 (Scheduled)
Fexinidazole

Study Number: 0504-2007

Males

Dose Level	Animal Number	Group/ Subgroup	Study Day	BIL SCORE	RBC SCORE	SG	COL	APP	VOL mL
vehicle									
	2745	1/1	29	0.	1.	1.026	Y	LT	10.0
	2746	1/1	29	0.	1.	1.018	Y	LT	8.0
	2747	1/1	29	0.	1.	1.018	Y	L	12.0
	2748	1/1	29	0.	0.	1.018	Y	L	12.0
	2749	1/1	29	0.	0.	1.018	Y	L	11.0
			N	5	5	5	0	0	5
			Mean	0.	1.	1.020	-	-	10.6
			Sdev	0.0	0.5	0.0036	-	-	1.67
50 mg/kg									
	2755	2/1	29	0.	0.	1.022	W	L	11.0
	2756	2/1	29	0.	0.	1.022	Y	L	12.0
	2757	2/1	29	0.	0.	1.020	Y	L	12.0
	2758	2/1	29	0.	0.	1.020	Y	L	13.0
	2759	2/1	29	0.	0.	1.017	W	L	14.0
			N	5	5	5	0	0	5
			Mean	0.	0.	1.020	-	-	12.4
			Sdev	0.0	0.0	0.0020	-	-	1.14
200 mg/kg									
	2765	3/1	29	0.	0.	1.020	Y	L	11.0
	2766	3/1	29	0.	0.	1.020	Y	L	12.0
	2767	3/1	29	0.	1.	1.021	Y	LT	9.0
	2768	3/1	29	0.	0.	1.019	Y	L	17.0
	2769	3/1	29	0.	0.	1.018	Y	L	12.0
			N	5	5	5	0	0	5
			Mean	0.	0.	1.020	-	-	12.2
			Sdev	0.0	0.4	0.0011	-	-	2.95

BIL - BILIRUBIN
RBC - HEMOGLOBIN/RED BLOOD CELLS
SG - SPECIFIC GRAVITY
COL - COLOUR
APP - APPEARANCE
VOL - URINARY VOLUME

Appendix 7
Day 29 Urine Data
Test period

Session 1 (Scheduled)
Fexinidazole

Study Number: 0504-2007

Males

Dose Level	Animal Number	Group/ Subgroup	Study Day	BIL SCORE	RBC SCORE	SG	COL	APP	VOL mL
800 mg/kg									
	2780	4/1	29	0.	0.	1.021	Y	L	8.0
	2781	4/1	29	0.	0.	1.023	Y	L	12.0
	2782	4/1	29	0.	1.	1.020	Y	L	11.0
	2783	4/1	29	0.	0.	1.020	Y	L	14.0
	2784	4/1	29	0.	0.	1.020	Y	L	12.0
			N	5	5	5	0	0	5
			Mean	0.	0.	1.021	-	-	11.4
			Sdev	0.0	0.4	0.0013	-	-	2.19

BIL - BILIRUBIN
RBC - HEMOGLOBIN/RED BLOOD CELLS
SG - SPECIFIC GRAVITY
COL - COLOUR
APP - APPEARANCE
VOL - URINARY VOLUME

Appendix 7
Day 29 Urine Data
Test period

Session 1 (Scheduled)
Fexinidazole

Study Number: 0504-2007

Females

Dose Level	Animal Number	Group/ Subgroup	Study Day	PH UNITS	WBC SCORE	NIT SCORE	PRO SCORE	GLU SCORE	KET SCORE	UBG SCORE
vehicle										
	2795	1/1	29	6.5	0.	0.	0.	0.	0.	0.
	2796	1/1	29	7.0	0.	0.	1.	0.	0.	0.
	2797	1/1	29	7.0	0.	0.	0.	0.	0.	0.
	2798	1/1	29	7.0	0.	0.	0.	0.	0.	0.
	2799	1/1	29	7.0	0.	0.	0.	0.	0.	0.
			N	5	5	5	5	5	5	5
			Mean	6.9	0.	0.	0.	0.	0.	0.
			Sdev	0.22	0.0	0.0	0.4	0.0	0.0	0.0
50 mg/kg										
	2805	2/1	29	7.0	0.	0.	0.	0.	0.	0.
	2806	2/1	29	7.0	0.	0.	0.	0.	0.	0.
	2807	2/1	29	7.0	0.	0.	0.	0.	0.	0.
	2808	2/1	29	7.0	0.	0.	0.	0.	0.	0.
	2809	2/1	29	7.0	0.	0.	1.	0.	0.	0.
			N	5	5	5	5	5	5	5
			Mean	7.0	0.	0.	0.	0.	0.	0.
			Sdev	0.00	0.0	0.0	0.4	0.0	0.0	0.0
200 mg/kg										
	2815	3/1	29	7.0	0.	0.	1.	0.	0.	0.
	2816	3/1	29	7.0	0.	0.	1.	0.	0.	0.
	2817	3/1	29	7.0	0.	0.	1.	0.	0.	0.
	2818	3/1	29	7.0	0.	0.	0.	0.	0.	0.
	2819	3/1	29	7.0	0.	0.	1.	0.	0.	0.
			N	5	5	5	5	5	5	5
			Mean	7.0	0.	0.	1.	0.	0.	0.
			Sdev	0.00	0.0	0.0	0.4	0.0	0.0	0.0

PH - PH
WBC - WHITE BLOOD CELLS
NIT - NITRITES
PRO - PROTEINS
GLU - GLUCOSE
KET - KETONE BODIES
UBG - UROBILINOGEN

Appendix 7
Day 29 Urine Data
Test period

Session 1 (Scheduled)
Fexinidazole

Study Number: 0504-2007

Females

Dose Level	Animal Number	Group/ Subgroup	Study Day	PH UNITS	WBC SCORE	NIT SCORE	PRO SCORE	GLU SCORE	KET SCORE	UBG SCORE
800 mg/kg										
	2830	4/1	29	7.0	0.	0.	0.	0.	0.	0.
	2831	4/1	29	7.0	0.	1.	1.	0.	0.	0.
	2832	4/1	29	7.0	0.	1.	0.	0.	0.	0.
	2833	4/1	29	7.0	0.	0.	0.	0.	0.	0.
	2834	4/1	29	7.0	0.	0.	0.	0.	0.	0.
			N	5	5	5	5	5	5	5
			Mean	7.0	0.	0.	0.	0.	0.	0.
			Sdev	0.00	0.0	0.5	0.4	0.0	0.0	0.0

PH - PH
WBC - WHITE BLOOD CELLS
NIT - NITRITES
PRO - PROTEINS
GLU - GLUCOSE
KET - KETONE BODIES
UBG - UROBILINOGEN

Appendix 7
Day 29 Urine Data
Test period

Session 1 (Scheduled)
Fexinidazole

Study Number: 0504-2007

Females

Dose Level	Animal Number	Group/ Subgroup	Study Day	BIL SCORE	RBC SCORE	SG	COL	APP	VOL mL
vehicle									
	2795	1/1	29	0.	0.	1.020	Y	L	8.0
	2796	1/1	29	0.	0.	1.025	Y	L	6.0
	2797	1/1	29	0.	0.	1.020	Y	L	10.0
	2798	1/1	29	0.	1.	1.019	W	L	11.0
	2799	1/1	29	0.	0.	1.015	Y	L	8.0
			N	5	5	5	0	0	5
			Mean	0.	0.	1.020	-	-	8.6
			Sdev	0.0	0.4	0.0036	-	-	1.95
50 mg/kg									
	2805	2/1	29	0.	0.	1.019	Y	L	9.0
	2806	2/1	29	0.	0.	1.016	Y	L	9.0
	2807	2/1	29	0.	0.	1.021	Y	L	9.0
	2808	2/1	29	0.	1.	1.015	Y	L	6.0
	2809	2/1	29	0.	0.	1.022	Y	L	7.0
			N	5	5	5	0	0	5
			Mean	0.	0.	1.019	-	-	8.0
			Sdev	0.0	0.4	0.0030	-	-	1.41
200 mg/kg									
	2815	3/1	29	0.	0.	1.020	Y	L	8.0
	2816	3/1	29	0.	0.	1.022	Y	L	6.0
	2817	3/1	29	0.	0.	1.022	Y	L	7.0
	2818	3/1	29	0.	0.	1.022	Y	L	10.0
	2819	3/1	29	0.	0.	1.020	Y	L	7.0
			N	5	5	5	0	0	5
			Mean	0.	0.	1.021	-	-	7.6
			Sdev	0.0	0.0	0.0011	-	-	1.52

BIL - BILIRUBIN
RBC - HEMOGLOBIN/RED BLOOD CELLS
SG - SPECIFIC GRAVITY
COL - COLOUR
APP - APPEARANCE
VOL - URINARY VOLUME

Appendix 7
Day 29 Urine Data
Test period

Session 1 (Scheduled)
Fexinidazole

Study Number: 0504-2007

Females

Dose Level	Animal Number	Group/ Subgroup	Study Day	BIL SCORE	RBC SCORE	SG	COL	APP	VOL mL
800 mg/kg									
	2830	4/1	29	0.	0.	1.017	Y	L	11.0
	2831	4/1	29	0.	0.	1.019	Y	L	11.0
	2832	4/1	29	0.	0.	1.016	Y	L	6.0
	2833	4/1	29	0.	0.	1.015	Y	L	8.0
	2834	4/1	29	0.	0.	1.016	Y	L	10.0
			N	5	5	5	0	0	5
			Mean	0.	0.	1.017	-	-	9.2
			Sdev	0.0	0.0	0.0015	-	-	2.17

BIL - BILIRUBIN
RBC - HEMOGLOBIN/RED BLOOD CELLS
SG - SPECIFIC GRAVITY
COL - COLOUR
APP - APPEARANCE
VOL - URINARY VOLUME

Appendix 7
Day 43 Urine Data
Test period

Session 1 (Scheduled)
Fexinidazole

Study Number: 0504-2007

Males

Dose Level	Animal Number	Group/ Subgroup	Study Day	PH UNITS	WBC SCORE	NIT SCORE	PRO SCORE	GLU SCORE	KET SCORE	UBG SCORE
vehicle										
	2745	1/1	43	7.0	0.	0.	1.	0.	0.	0.
	2746	1/1	43	7.0	0.	0.	1.	0.	0.	0.
	2747	1/1	43	7.0	0.	0.	1.	0.	0.	0.
	2748	1/1	43	7.0	0.	0.	1.	0.	0.	0.
	2749	1/1	43	7.0	0.	0.	0.	0.	0.	0.
			N	5	5	5	5	5	5	5
			Mean	7.0	0.	0.	1.	0.	0.	0.
			Sdev	0.00	0.0	0.0	0.4	0.0	0.0	0.0
800 mg/kg										
	2780	4/1	43	7.0	0.	0.	1.	0.	1.	0.
	2781	4/1	43	7.0	0.	0.	1.	0.	1.	0.
	2782	4/1	43	7.0	0.	0.	1.	0.	0.	0.
	2783	4/1	43	7.0	0.	0.	1.	0.	1.	0.
	2784	4/1	43	7.0	0.	0.	1.	0.	0.	0.
			N	5	5	5	5	5	5	5
			Mean	7.0	0.	0.	1.	0.	1.	0.
			Sdev	0.00	0.0	0.0	0.0	0.0	0.5	0.0

PH - PH
WBC - WHITE BLOOD CELLS
NIT - NITRITES
PRO - PROTEINS
GLU - GLUCOSE
KET - KETONE BODIES
UBG - UROBILINOGEN

Appendix 7
Day 43 Urine Data
Test period

Session 1 (Scheduled)
Fexinidazole

Study Number: 0504-2007

Males

Dose Level	Animal Number	Group/ Subgroup	Study Day	BIL SCORE	RBC SCORE	SG	COL	APP	VOL mL
vehicle									
	2745	1/1	43	0.	0.	1.021	Y	L	12.0
	2746	1/1	43	0.	0.	1.018	Y	L	10.0
	2747	1/1	43	0.	0.	1.018	Y	L	12.0
	2748	1/1	43	0.	1.	1.021	Y	L	12.0
	2749	1/1	43	0.	0.	1.019	Y	L	11.0
			N	5	5	5	0	0	5
			Mean	0.	0.	1.019	-	-	11.4
			Sdev	0.0	0.4	0.0015	-	-	0.89
800 mg/kg									
	2780	4/1	43	0.	0.	1.020	W	L	9.0
	2781	4/1	43	0.	0.	1.022	Y	L	10.0
	2782	4/1	43	0.	0.	1.017	Y	L	10.0
	2783	4/1	43	0.	0.	1.021	Y	L	10.0
	2784	4/1	43	0.	0.	1.017	Y	L	12.0
			N	5	5	5	0	0	5
			Mean	0.	0.	1.019	-	-	10.2
			Sdev	0.0	0.0	0.0023	-	-	1.10

BIL - BILIRUBIN
RBC - HEMOGLOBIN/RED BLOOD CELLS
SG - SPECIFIC GRAVITY
COL - COLOUR
APP - APPEARANCE
VOL - URINARY VOLUME

Appendix 7
Day 43 Urine Data
Test period

Session 1 (Scheduled)
Fexinidazole

Study Number: 0504-2007

Females

Dose Level	Animal Number	Group/ Subgroup	Study Day	PH UNITS	WBC SCORE	NIT SCORE	PRO SCORE	GLU SCORE	KET SCORE	UBG SCORE
vehicle										
	2795	1/1	43	7.0	0.	0.	0.	0.	0.	0.
	2796	1/1	43	7.0	0.	0.	0.	0.	0.	0.
	2797	1/1	43	7.0	0.	0.	0.	0.	0.	0.
	2798	1/1	43	7.0	0.	0.	0.	0.	0.	0.
	2799	1/1	43	7.0	0.	0.	0.	0.	0.	0.
			N	5	5	5	5	5	5	5
			Mean	7.0	0.	0.	0.	0.	0.	0.
			Sdev	0.00	0.0	0.0	0.0	0.0	0.0	0.0
800 mg/kg										
	2830	4/1	43	7.0	0.	0.	0.	0.	0.	0.
	2831	4/1	43	7.0	0.	0.	0.	0.	0.	0.
	2832	4/1	43	7.0	0.	0.	0.	0.	0.	0.
	2833	4/1	43	7.0	0.	0.	0.	0.	0.	0.
	2834	4/1	43	7.0	0.	0.	0.	0.	0.	0.
			N	5	5	5	5	5	5	5
			Mean	7.0	0.	0.	0.	0.	0.	0.
			Sdev	0.00	0.0	0.0	0.0	0.0	0.0	0.0

PH - PH
WBC - WHITE BLOOD CELLS
NIT - NITRITES
PRO - PROTEINS
GLU - GLUCOSE
KET - KETONE BODIES
UBG - UROBILINOGEN

Appendix 7
Day 43 Urine Data
Test period

Session 1 (Scheduled)
Fexinidazole

Study Number: 0504-2007

Females

Dose Level	Animal Number	Group/ Subgroup	Study Day	BIL SCORE	RBC SCORE	SG	COL	APP	VOL mL
vehicle									
	2795	1/1	43	0.	0.	1.018	Y	L	10.0
	2796	1/1	43	0.	0.	1.019	Y	L	8.0
	2797	1/1	43	0.	0.	1.016	Y	L	10.0
	2798	1/1	43	0.	0.	1.018	Y	L	11.0
	2799	1/1	43	0.	0.	1.014	Y	L	10.0
			N	5	5	5	0	0	5
			Mean	0.	0.	1.017	-	-	9.8
			Sdev	0.0	0.0	0.0020	-	-	1.10
800 mg/kg									
	2830	4/1	43	0.	0.	1.020	Y	L	9.0
	2831	4/1	43	0.	1.	1.017	W	L	1.0
	2832	4/1	43	0.	1.	1.020	Y	L	7.0
	2833	4/1	43	0.	0.	1.015	Y	L	8.0
	2834	4/1	43	0.	0.	1.018	Y	L	8.0
			N	5	5	5	0	0	5
			Mean	0.	0.	1.018	-	-	6.6
			Sdev	0.0	0.5	0.0021	-	-	3.21

BIL - BILIRUBIN
RBC - HEMOGLOBIN/RED BLOOD CELLS
SG - SPECIFIC GRAVITY
COL - COLOUR
APP - APPEARANCE
VOL - URINARY VOLUME

Appendix 8 Absolute Organ Weights

Appendix 8
Absolute Organ Weights (g)
Test period
Days 29 Interim Sacrifice

Fexinidazole — Study Number: 0504-2007

Dose Level	Animal Number	Group/ Subgroup	Terminal Body Wt(g)	SPLEEN	KIDNEYS	LIVER	THYMUS	HEART
					M a l e s			
vehicle								
	2735	1/1	380.90	0.76	3.05	10.72	0.480	1.58
	2736	1/1	396.80	0.95	3.00	11.81	0.620	1.42
	2737	1/1	422.80	0.88	2.84	12.86	0.520	1.38
	2738	1/1	383.80	0.79	2.75	8.95	0.360	1.28
	2739	1/1	419.00	0.88	2.85	11.80	0.490	1.45
	2740	1/1	410.50	0.81	3.10	10.80	0.470	1.48
	2741	1/1	381.10	0.75	2.79	10.24	0.760	1.78
	2742	1/1	348.70	0.88	2.90	10.08	0.570	1.35
	2743	1/1	373.70	0.91	2.61	8.45	0.560	1.21
	2744	1/1	398.00	1.10	3.32	11.61	0.590	1.44
		N	10	10	10	10	10	10
		Mean	391.53	0.87	2.92	10.73	0.542	1.44
		Sdev	22.597	0.104	0.203	1.360	0.1066	0.159
50 mg/kg								
	2750	2/1	433.20	0.86	3.12	12.70	0.540	1.35
	2751	2/1	374.70	0.60	2.62	10.82	0.580	1.31
	2752	2/1	401.40	0.63	2.60	11.95	0.580	1.25
	2753	2/1	364.60	0.91	2.80	9.88	0.450	1.19
	2754	2/1	358.50	0.82	3.03	11.67	0.660	1.40
	2755	2/1	438.50	0.92	2.92	13.20	0.710	1.38
	2756	2/1	343.40	0.81	2.40	11.17	0.450	1.21
	2757	2/1	340.10	0.66	2.55	9.89	0.520	1.06
	2758	2/1	336.50	0.72	2.79	10.85	0.650	1.34
	2759	2/1	353.60	0.91	2.68	10.43	0.610	1.17
		N	10	10	10	10	10	10
		Mean	374.45	0.78	2.75	11.26	0.575	1.27
		Sdev	37.492	0.122	0.225	1.122	0.0868	0.109
200 mg/kg								
	2760	3/1	359.30	0.73	2.64	10.11	0.440	1.30
	2761	3/1	385.00	0.82	3.00	11.88	0.540	1.37
	2762	3/1	393.60	0.96	2.98	11.62	0.520	1.42
	2763	3/1	330.50	0.72	2.56	10.80	0.360	1.07
	2764	3/1	396.50	0.95	2.66	12.07	0.410	1.35
	2765	3/1	362.80	0.87	2.85	11.59	0.670	1.13
	2766	3/1	340.60	0.91	3.01	11.53	0.590	1.32
	2767	3/1	276.70	0.71	2.18	7.80	0.430	0.99
	2768	3/1	401.50	0.63	2.99	12.94	0.610	1.30
	2769	3/1	317.10	0.53	2.54	9.77	0.380	1.06

Note: Data collected using grace days.

Appendix 8
Absolute Organ Weights (g)
Test period
Days 29 Interim Sacrifice

Fexinidazole | Study Number: 0504-2007

Dose Level	Animal Number	Group/ Subgroup	Terminal Body Wt(g)	SPLEEN	KIDNEYS	LIVER	THYMUS	HEART
			Males					
200 mg/kg								
		N	10	10	10	10	10	10
		Mean	356.36	0.78	2.74	11.01	0.495	1.23
		Sdev	40.429	0.143	0.274	1.464	0.1062	0.153
800 mg/kg								
	2770	4/1	353.30	0.67	2.55	11.65	0.390	1.25
	2771	4/1	344.70	0.81	2.80	13.35	0.460	1.37
	2772	4/1	411.00	0.68	2.62	10.73	0.470	1.27
	2773	4/1	298.50	0.57	2.19	9.54	0.460	0.95
	2774	4/1	293.60	0.54	2.23	10.63	0.320	0.98
	2775	4/1	385.00	0.79	3.09	11.98	0.570	1.40
	2776	4/1	338.80	0.63	2.56	11.41	0.360	1.11
	2777	4/1	351.00	1.05	2.77	11.62	0.500	1.20
	2778	4/1	338.80	0.76	2.63	12.03	0.500	1.01
	2779	4/1	322.70	0.49	2.27	10.56	0.330	1.06
		N	10	10	10	10	10	10
		Mean	343.74	0.70	2.57	11.35	0.436	1.16
		Sdev	35.627	0.163	0.283	1.045	0.0824	0.162

Note: Data collected using grace days.

Appendix 8
Absolute Organ Weights (g)
Test period
Days 29 Interim Sacrifice

Fexinidazole | Study Number: 0504-2007

Dose Level	Animal Number	Group/ Subgroup	Terminal Body Wt(g)	BRAIN	TESTES	PROSTATE	ADRENALS
			M a l e s				
vehicle							
	2735	1/1	380.90	2.15	3.48	0.78	0.069
	2736	1/1	396.80	2.20	3.63	0.76	0.066
	2737	1/1	422.80	1.94	3.22	0.43	0.064
	2738	1/1	383.80	2.17	3.85	0.64	0.056
	2739	1/1	419.00	2.02	3.45	0.48	0.071
	2740	1/1	410.50	2.16	3.63	0.62	0.073
	2741	1/1	381.10	2.07	3.53	0.62	0.045
	2742	1/1	348.70	1.98	3.24	0.70	0.054
	2743	1/1	373.70	1.94	3.43	0.59	0.051
	2744	1/1	398.00	2.12	3.41	0.53	0.076
		N	10	10	10	10	10
		Mean	391.53	2.08	3.49	0.62	0.062
		Sdev	22.597	0.099	0.188	0.114	0.0102
50 mg/kg							
	2750	2/1	433.20	2.13	3.52	0.79	0.074
	2751	2/1	374.70	2.07	3.29	0.61	0.071
	2752	2/1	401.40	2.01	3.07	0.47	0.060
	2753	2/1	364.60	2.01	3.52	0.46	0.062
	2754	2/1	358.50	2.07	3.52	0.68	0.054
	2755	2/1	438.50	2.05	3.72	0.51	0.057
	2756	2/1	343.40	1.87	3.51	0.48	0.067
	2757	2/1	340.10	2.09	3.11	0.51	0.046
	2758	2/1	336.50	1.89	3.27	0.45	0.060
	2759	2/1	353.60	1.97	3.26	0.42	0.059
		N	10	10	10	10	10
		Mean	374.45	2.02	3.38	0.54	0.061
		Sdev	37.492	0.085	0.209	0.118	0.0083
200 mg/kg							
	2760	3/1	359.30	1.99	3.16	0.60	0.050
	2761	3/1	385.00	1.99	3.33	0.72	0.072
	2762	3/1	393.60	1.88	3.53	0.57	0.062
	2763	3/1	330.50	1.91	3.02	0.50	0.074
	2764	3/1	396.50	2.14	3.82	0.41	0.075
	2765	3/1	362.80	1.96	3.32	0.68	0.065
	2766	3/1	340.60	2.04	3.59	0.67	0.069
	2767	3/1	276.70	2.02	2.86	0.50	0.048
	2768	3/1	401.50	1.93	3.41	0.42	0.066
	2769	3/1	317.10	1.97	3.12	0.39	0.057

Note: Data collected using grace days.

Appendix 8
Absolute Organ Weights (g)
Test period
Days 29 Interim Sacrifice

Fexinidazole | Study Number: 0504-2007

Dose Level	Animal Number	Group/ Subgroup	Terminal Body Wt(g)	BRAIN	TESTES	PROSTATE	ADRENALS
				M a l e s			
200 mg/kg							
		N	10	10	10	10	10
		Mean	356.36	1.98	3.32	0.55	0.064
		Sdev	40.429	0.074	0.287	0.120	0.0095
800 mg/kg							
	2770	4/1	353.30	2.14	3.74	0.57	0.075
	2771	4/1	344.70	2.14	3.40	0.42	0.078
	2772	4/1	411.00	2.19	3.54	0.58	0.057
	2773	4/1	298.50	1.77	2.98	0.47	0.046
	2774	4/1	293.60	1.96	3.71	0.61	0.063
	2775	4/1	385.00	2.19	3.92	0.72	0.058
	2776	4/1	338.80	1.93	3.67	0.75	0.077
	2777	4/1	351.00	2.03	3.17	0.59	0.063
	2778	4/1	338.80	2.06	3.37	0.64	0.062
	2779	4/1	322.70	2.00	2.95	0.47	0.054
		N	10	10	10	10	10
		Mean	343.74	2.04	3.45	0.58	0.063
		Sdev	35.627	0.133	0.331	0.107	0.0106

Note: Data collected using grace days.

Appendix 8
Absolute Organ Weights (g)
Test period
Days 29 Interim Sacrifice

Fexinidazole | Study Number: 0504-2007

Dose Level	Animal Number	Group/ Subgroup	Terminal Body Wt(g)	SPLEEN	KIDNEYS	LIVER	THYMUS
			Females				
vehicle							
	2785	1/1	212.40	0.57	1.52	6.06	0.350
	2786	1/1	243.20	0.60	1.62	6.35	0.340
	2787	1/1	219.60	0.65	1.59	5.77	0.230
	2788	1/1	222.60	0.64	1.63	5.69	0.420
	2789	1/1	208.30	0.63	1.39	5.28	0.350
	2790	1/1	224.50	0.54	1.86	6.40	0.360
	2791	1/1	207.60	0.40	1.29	5.17	0.290
	2792	1/1	234.00	0.50	1.76	6.45	0.280
	2793	1/1	217.30	0.59	1.68	5.83	0.430
	2794	1/1	238.50	0.59	1.71	7.19	0.310
		N	10	10	10	10	10
		Mean	222.80	0.57	1.61	6.02	0.336
		Sdev	12.387	0.075	0.170	0.604	0.0615
50 mg/kg							
	2800	2/1	216.80	0.57	1.67	6.02	0.320
	2801	2/1	247.90	0.63	1.60	6.93	0.310
	2802	2/1	235.20	0.80	1.66	6.15	0.370
	2803	2/1	225.20	0.75	1.67	6.74	0.310
	2804	2/1	209.90	0.68	1.61	5.73	0.420
	2805	2/1	233.00	0.79	1.93	7.10	0.450
	2806	2/1	220.30	0.74	1.55	6.31	0.510
	2807	2/1	220.50	0.51	1.51	6.20	0.300
	2808	2/1	230.60	0.59	1.56	7.12	0.400
	2809	2/1	238.70	0.78	1.83	8.16	0.380
		N	10	10	10	10	10
		Mean	227.81	0.68	1.66	6.65	0.377
		Sdev	11.403	0.104	0.130	0.715	0.0696
200 mg/kg							
	2810	3/1	218.20	0.55	1.68	6.52	0.400
	2811	3/1	237.90	0.54	1.76	6.27	0.250
	2812	3/1	235.20	0.63	1.64	6.71	0.360
	2813	3/1	240.00	0.58	1.88	7.66	0.320
	2814	3/1	213.00	0.58	1.50	6.05	0.390
	2815	3/1	219.20	0.60	1.53	6.64	0.390
	2816	3/1	231.50	0.57	1.77	6.61	0.280
	2817	3/1	238.10	0.68	1.82	7.36	0.310
	2818	3/1	233.20	0.48	1.68	7.29	0.420
	2819	3/1	221.90	0.45	1.70	6.90	0.340

Note: Data collected using grace days.

Appendix 8
Absolute Organ Weights (g)
Test period
Days 29 Interim Sacrifice

Fexinidazole | Study Number: 0504-2007

Dose Level	Animal Number	Group/ Subgroup	Terminal Body Wt(g)	SPLEEN	KIDNEYS	LIVER	THYMUS
			F e m a l e s				
200 mg/kg							
		N	10	10	10	10	10
		Mean	228.82	0.57	1.70	6.80	0.346
		Sdev	9.799	0.067	0.119	0.505	0.0558
800 mg/kg							
	2820	4/1	207.90	0.52	1.78	7.77	0.330
	2821	4/1	195.40	0.47	1.52	6.54	0.240
	2822	4/1	228.20	0.49	1.67	6.99	0.260
	2823	4/1	248.60	0.54	1.61	9.30	0.460
	2825	4/1	213.50	0.54	2.11	10.43	0.350
	2826	4/1	212.30	0.54	1.58	7.69	0.230
	2827	4/1	223.50	0.65	1.58	8.02	0.330
	2828	4/1	219.90	0.59	1.68	9.09	0.330
	2829	4/1	220.00	0.47	1.66	7.68	0.320
		N	9	9	9	9	9
		Mean	218.81	0.53	1.69	8.17	0.317
		Sdev	14.739	0.058	0.175	1.221	0.0696

Note: Data collected using grace days.

Appendix 8
Absolute Organ Weights (g)
Test period
Days 29 Interim Sacrifice

Fexinidazole

Study Number: 0504-2007

Dose Level	Animal Number	Group/ Subgroup	Terminal Body Wt(g)	HEART	BRAIN	OVARIES	ADRENALS
				F e m a l e s			
vehicle							
	2785	1/1	212.40	0.82	1.91	0.133	0.072
	2786	1/1	243.20	0.84	2.10	0.149	0.068
	2787	1/1	219.60	0.76	2.00	0.105	0.067
	2788	1/1	222.60	0.85	1.86	0.116	0.076
	2789	1/1	208.30	0.79	1.88	0.120	0.074
	2790	1/1	224.50	0.86	2.05	0.135	0.067
	2791	1/1	207.60	0.71	1.85	0.118	0.075
	2792	1/1	234.00	0.84	1.89	0.101	0.072
	2793	1/1	217.30	0.85	1.76	0.098	0.073
	2794	1/1	238.50	0.86	1.78	0.122	0.096
		N	10	10	10	10	10
		Mean	222.80	0.82	1.91	0.120	0.074
		Sdev	12.387	0.050	0.111	0.0161	0.0085
50 mg/kg							
	2800	2/1	216.80	0.79	2.03	0.130	0.070
	2801	2/1	247.90	0.87	1.95	0.123	0.080
	2802	2/1	235.20	0.82	2.09	0.121	0.069
	2803	2/1	225.20	0.91	1.85	0.171	0.073
	2804	2/1	209.90	0.83	1.86	0.108	0.070
	2805	2/1	233.00	0.91	1.91	0.131	0.072
	2806	2/1	220.30	0.84	1.89	0.107	0.082
	2807	2/1	220.50	0.92	1.87	0.117	0.059
	2808	2/1	230.60	0.88	1.82	0.119	0.077
	2809	2/1	238.70	0.83	1.90	0.115	0.084
		N	10	10	10	10	10
		Mean	227.81	0.86	1.92	0.124	0.074
		Sdev	11.403	0.044	0.084	0.0182	0.0074
200 mg/kg							
	2810	3/1	218.20	0.80	1.86	0.094	0.074
	2811	3/1	237.90	0.72	1.85	0.112	0.085
	2812	3/1	235.20	0.83	2.03	0.100	0.079
	2813	3/1	240.00	0.85	1.88	0.120	0.071
	2814	3/1	213.00	0.68	1.85	0.088	0.056
	2815	3/1	219.20	0.82	1.76	0.112	0.049
	2816	3/1	231.50	0.81	1.94	0.115	0.086
	2817	3/1	238.10	0.85	1.91	0.149	0.069
	2818	3/1	233.20	0.81	1.78	0.116	0.065
	2819	3/1	221.90	0.90	1.87	0.113	0.077

Note: Data collected using grace days.

Appendix 8
Absolute Organ Weights (g)
Test period
Days 29 Interim Sacrifice

Fexinidazole | Study Number: 0504-2007

Dose Level	Animal Number	Group/ Subgroup	Terminal Body Wt(g)	HEART	BRAIN	OVARIES	ADRENALS
				Females			
200 mg/kg							
		N	10	10	10	10	10
		Mean	228.82	0.81	1.87	0.112	0.071
		Sdev	9.799	0.064	0.077	0.0169	0.0119
800 mg/kg							
	2820	4/1	207.90	0.84	1.90	0.123	0.061
	2821	4/1	195.40	0.67	1.85	0.090	0.063
	2822	4/1	228.20	0.80	1.79	0.135	0.073
	2823	4/1	248.60	0.88	1.86	0.127	0.080
	2825	4/1	213.50	0.77	1.99	0.127	0.069
	2826	4/1	212.30	0.74	1.82	0.110	0.067
	2827	4/1	223.50	0.84	1.90	0.112	0.070
	2828	4/1	219.90	0.88	1.82	0.140	0.070
	2829	4/1	220.00	0.75	1.87	0.112	0.065
		N	9	9	9	9	9
		Mean	218.81	0.80	1.87	0.119	0.069
		Sdev	14.739	0.071	0.059	0.0154	0.0059

Note: Data collected using grace days.

Appendix 8
Absolute Organ Weights (g)
Test period
Days 43 Final Sacrifice

Fexinidazole — Study Number: 0504-2007

Dose Level	Animal Number	Group/ Subgroup	Terminal Body Wt(g)	SPLEEN	KIDNEYS	LIVER	THYMUS	HEART
				M a l e s				
vehicle								
	2745	1/1	405.30	0.63	2.90	9.74	0.470	1.34
	2746	1/1	370.80	0.51	2.56	9.01	0.460	1.26
	2747	1/1	436.60	0.74	3.13	11.85	0.470	1.42
	2748	1/1	458.60	0.55	2.44	9.17	0.440	1.43
	2749	1/1	363.60	0.72	3.37	12.42	0.510	1.55
		N	5	5	5	5	5	5
		Mean	406.98	0.63	2.88	10.44	0.470	1.40
		Sdev	41.035	0.101	0.387	1.586	0.0255	0.108
800 mg/kg								
	2780	4/1	369.00	0.64	2.60	10.24	0.530	1.20
	2781	4/1	341.60	0.81	2.47	9.13	0.390	1.35
	2782	4/1	359.30	0.59	2.58	12.00	0.270	1.33
	2783	4/1	407.10	0.71	3.03	11.15	0.420	1.42
	2784	4/1	385.50	0.73	2.72	9.76	0.440	1.36
		N	5	5	5	5	5	5
		Mean	372.50	0.70	2.68	10.46	0.410	1.33
		Sdev	25.037	0.085	0.215	1.135	0.0941	0.081

Note: Data collected using grace days.

Appendix 8
Absolute Organ Weights (g)
Test period
Days 43 Final Sacrifice

Fexinidazole

Study Number: 0504-2007

Dose Level	Animal Number	Group/ Subgroup	Terminal Body Wt(g)	BRAIN	TESTES	PROSTATE	ADRENALS
			**M a l e s**				
vehicle							
	2745	1/1	405.30	2.08	3.13	0.75	0.055
	2746	1/1	370.80	2.10	3.10	0.41	0.045
	2747	1/1	436.60	1.98	3.58	0.60	0.062
	2748	1/1	458.60	1.91	3.50	0.44	0.049
	2749	1/1	363.60	2.02	3.67	0.73	0.076
		N	5	5	5	5	5
		Mean	406.98	2.02	3.40	0.59	0.057
		Sdev	41.035	0.077	0.264	0.158	0.0123
800 mg/kg							
	2780	4/1	369.00	2.06	3.42	0.53	0.065
	2781	4/1	341.60	2.10	3.79	0.65	0.069
	2782	4/1	359.30	2.10	3.39	0.67	0.051
	2783	4/1	407.10	2.06	3.96	0.61	0.068
	2784	4/1	385.50	2.08	3.13	0.72	0.057
		N	5	5	5	5	5
		Mean	372.50	2.08	3.54	0.64	0.062
		Sdev	25.037	0.020	0.333	0.071	0.0078

Note: Data collected using grace days.

Appendix 8
Absolute Organ Weights (g)
Test period
Days 43 Final Sacrifice

Fexinidazole | Study Number: 0504-2007

Dose Level	Animal Number	Group/ Subgroup	Terminal Body Wt(g)	SPLEEN	KIDNEYS	LIVER	THYMUS
			F e m a l e s				
vehicle							
	2795	1/1	231.10	0.52	1.61	6.25	0.250
	2796	1/1	233.10	0.52	1.75	6.11	0.380
	2797	1/1	227.50	0.50	1.77	6.67	0.260
	2798	1/1	255.10	0.55	1.66	6.36	0.260
	2799	1/1	250.30	0.52	2.00	6.52	0.360
		N	5	5	5	5	5
		Mean	239.42	0.52	1.76	6.38	0.302
		Sdev	12.405	0.018	0.150	0.220	0.0626
800 mg/kg							
	2830	4/1	245.00	0.80	1.72	7.19	0.270
	2831	4/1	206.80	0.45	1.53	6.24	0.230
	2832	4/1	213.00	0.45	1.56	6.22	0.320
	2833	4/1	229.20	0.47	1.40	6.18	0.270
	2834	4/1	228.70	0.44	1.55	6.70	0.410
		N	5	5	5	5	5
		Mean	224.54	0.52	1.55	6.51	0.300
		Sdev	15.046	0.156	0.114	0.437	0.0693

Note: Data collected using grace days.

Appendix 8
Absolute Organ Weights (g)
Test period
Days 43 Final Sacrifice

Fexinidazole

Study Number: 0504-2007

Dose Level	Animal Number	Group/ Subgroup	Terminal Body Wt(g)	HEART	BRAIN	OVARIES	ADRENALS
			Females				
vehicle							
	2795	1/1	231.10	0.82	1.89	0.094	0.071
	2796	1/1	233.10	0.90	1.92	0.105	0.077
	2797	1/1	227.50	0.88	2.14	0.121	0.085
	2798	1/1	255.10	0.93	2.00	0.110	0.072
	2799	1/1	250.30	0.92	1.93	0.082	0.065
		N	5	5	5	5	5
		Mean	239.42	0.89	1.98	0.103	0.074
		Sdev	12.405	0.044	0.100	0.0148	0.0075
800 mg/kg							
	2830	4/1	245.00	0.92	1.78	0.098	0.063
	2831	4/1	206.80	0.81	1.91	0.093	0.058
	2832	4/1	213.00	0.85	1.79	0.085	0.065
	2833	4/1	229.20	0.75	1.91	0.091	0.056
	2834	4/1	228.70	0.86	1.87	0.095	0.057
		N	5	5	5	5	5
		Mean	224.54	0.84	1.85	0.092	0.060
		Sdev	15.046	0.063	0.063	0.0048	0.0040

Note: Data collected using grace days.

Appendix 9 Relative Organ Weights

Appendix 9
Relative Organ Weights (% Body Weight)
Test period
Days 29 Interim Sacrifice

Fexinidazole — Study Number: 0504-2007

Dose Level	Animal Number	Group/ Subgroup	Terminal Body Wt(g)	SPLEEN	KIDNEYS	LIVER	THYMUS	HEART
					Males			
vehicle								
	2735	1/1	380.90	0.20	0.80	2.81	0.126	0.41
	2736	1/1	396.80	0.24	0.76	2.98	0.156	0.36
	2737	1/1	422.80	0.21	0.67	3.04	0.123	0.33
	2738	1/1	383.80	0.21	0.72	2.33	0.094	0.33
	2739	1/1	419.00	0.21	0.68	2.82	0.117	0.35
	2740	1/1	410.50	0.20	0.76	2.63	0.114	0.36
	2741	1/1	381.10	0.20	0.73	2.69	0.199	0.47
	2742	1/1	348.70	0.25	0.83	2.89	0.163	0.39
	2743	1/1	373.70	0.24	0.70	2.26	0.150	0.32
	2744	1/1	398.00	0.28	0.83	2.92	0.148	0.36
		N	10	10	10	10	10	10
		Mean	391.53	0.22	0.75	2.74	0.139	0.37
		Sdev	22.597	0.028	0.059	0.263	0.0304	0.045
50 mg/kg								
	2750	2/1	433.20	0.20	0.72	2.93	0.125	0.31
	2751	2/1	374.70	0.16	0.70	2.89	0.155	0.35
	2752	2/1	401.40	0.16	0.65	2.98	0.144	0.31
	2753	2/1	364.60	0.25	0.77	2.71	0.123	0.33
	2754	2/1	358.50	0.23	0.85	3.26	0.184	0.39
	2755	2/1	438.50	0.21	0.67	3.01	0.162	0.31
	2756	2/1	343.40	0.24	0.70	3.25	0.131	0.35
	2757	2/1	340.10	0.19	0.75	2.91	0.153	0.31
	2758	2/1	336.50	0.21	0.83	3.22	0.193	0.40
	2759	2/1	353.60	0.26	0.76	2.95	0.173	0.33
		N	10	10	10	10	10	10
		Mean	374.45	0.21	0.74	3.01	0.154	0.34
		Sdev	37.492	0.034	0.065	0.180	0.0242	0.033
200 mg/kg								
	2760	3/1	359.30	0.20	0.73	2.81	0.122	0.36
	2761	3/1	385.00	0.21	0.78	3.09	0.140	0.36
	2762	3/1	393.60	0.24	0.76	2.95	0.132	0.36
	2763	3/1	330.50	0.22	0.77	3.27	0.109	0.32
	2764	3/1	396.50	0.24	0.67	3.04	0.103	0.34
	2765	3/1	362.80	0.24	0.79	3.19	0.185	0.31
	2766	3/1	340.60	0.27	0.88	3.39	0.173	0.39
	2767	3/1	276.70	0.26	0.79	2.82	0.155	0.36
	2768	3/1	401.50	0.16	0.74	3.22	0.152	0.32
	2769	3/1	317.10	0.17	0.80	3.08	0.120	0.33

Note: Data collected using grace days.

Appendix 9
Relative Organ Weights (% Body Weight)
Test period
Days 29 Interim Sacrifice

Fexinidazole

Study Number: 0504-2007

Dose Level	Animal Number	Group/ Subgroup	Terminal Body Wt(g)	SPLEEN	KIDNEYS	LIVER	THYMUS	HEART
			Males					
200 mg/kg								
		N	10	10	10	10	10	10
		Mean	356.36	0.22	0.77	3.09	0.139	0.35
		Sdev	40.429	0.037	0.054	0.188	0.0270	0.023
800 mg/kg								
	2770	4/1	353.30	0.19	0.72	3.30	0.110	0.35
	2771	4/1	344.70	0.23	0.81	3.87	0.133	0.40
	2772	4/1	411.00	0.17	0.64	2.61	0.114	0.31
	2773	4/1	298.50	0.19	0.73	3.20	0.154	0.32
	2774	4/1	293.60	0.18	0.76	3.62	0.109	0.33
	2775	4/1	385.00	0.21	0.80	3.11	0.148	0.36
	2776	4/1	338.80	0.19	0.76	3.37	0.106	0.33
	2777	4/1	351.00	0.30	0.79	3.31	0.142	0.34
	2778	4/1	338.80	0.22	0.78	3.55	0.148	0.30
	2779	4/1	322.70	0.15	0.70	3.27	0.102	0.33
		N	10	10	10	10	10	10
		Mean	343.74	0.20	0.75	3.32	0.127	0.34
		Sdev	35.627	0.042	0.053	0.336	0.0202	0.029

Note: Data collected using grace days.

Appendix 9
Relative Organ Weights (% Body Weight)
Test period
Days 29 Interim Sacrifice

Fexinidazole Study Number: 0504-2007

Dose Level	Animal Number	Group/ Subgroup	Terminal Body Wt(g)	BRAIN	TESTES	PROSTATE	ADRENALS
				Males			
vehicle							
	2735	1/1	380.90	0.56	0.91	0.20	0.018
	2736	1/1	396.80	0.55	0.91	0.19	0.017
	2737	1/1	422.80	0.46	0.76	0.10	0.015
	2738	1/1	383.80	0.57	1.00	0.17	0.015
	2739	1/1	419.00	0.48	0.82	0.11	0.017
	2740	1/1	410.50	0.53	0.88	0.15	0.018
	2741	1/1	381.10	0.54	0.93	0.16	0.012
	2742	1/1	348.70	0.57	0.93	0.20	0.016
	2743	1/1	373.70	0.52	0.92	0.16	0.014
	2744	1/1	398.00	0.53	0.86	0.13	0.019
		N	10	10	10	10	10
		Mean	391.53	0.53	0.89	0.16	0.016
		Sdev	22.597	0.037	0.066	0.035	0.0022
50 mg/kg							
	2750	2/1	433.20	0.49	0.81	0.18	0.017
	2751	2/1	374.70	0.55	0.88	0.16	0.019
	2752	2/1	401.40	0.50	0.76	0.12	0.015
	2753	2/1	364.60	0.55	0.97	0.13	0.017
	2754	2/1	358.50	0.58	0.98	0.19	0.015
	2755	2/1	438.50	0.47	0.85	0.12	0.013
	2756	2/1	343.40	0.54	1.02	0.14	0.020
	2757	2/1	340.10	0.61	0.91	0.15	0.013
	2758	2/1	336.50	0.56	0.97	0.13	0.018
	2759	2/1	353.60	0.56	0.92	0.12	0.017
		N	10	10	10	10	10
		Mean	374.45	0.54	0.91	0.14	0.016
		Sdev	37.492	0.044	0.082	0.027	0.0022
200 mg/kg							
	2760	3/1	359.30	0.55	0.88	0.17	0.014
	2761	3/1	385.00	0.52	0.86	0.19	0.019
	2762	3/1	393.60	0.48	0.90	0.14	0.016
	2763	3/1	330.50	0.58	0.91	0.15	0.022
	2764	3/1	396.50	0.54	0.96	0.10	0.019
	2765	3/1	362.80	0.54	0.92	0.19	0.018
	2766	3/1	340.60	0.60	1.05	0.20	0.020
	2767	3/1	276.70	0.73	1.03	0.18	0.017
	2768	3/1	401.50	0.48	0.85	0.10	0.017
	2769	3/1	317.10	0.62	0.98	0.12	0.018

Note: Data collected using grace days.

Appendix 9
Relative Organ Weights (% Body Weight)
Test period
Days 29 Interim Sacrifice

Fexinidazole Study Number: 0504-2007

Dose Level	Animal Number	Group/ Subgroup	Terminal Body Wt(g)	BRAIN	TESTES	PROSTATE	ADRENALS
			Males				
200 mg/kg							
		N	10	10	10	10	10
		Mean	356.36	0.56	0.94	0.15	0.018
		Sdev	40.429	0.075	0.070	0.035	0.0023
800 mg/kg							
	2770	4/1	353.30	0.61	1.06	0.16	0.021
	2771	4/1	344.70	0.62	0.99	0.12	0.023
	2772	4/1	411.00	0.53	0.86	0.14	0.014
	2773	4/1	298.50	0.59	1.00	0.16	0.015
	2774	4/1	293.60	0.67	1.26	0.21	0.021
	2775	4/1	385.00	0.57	1.02	0.19	0.015
	2776	4/1	338.80	0.57	1.08	0.22	0.023
	2777	4/1	351.00	0.58	0.90	0.17	0.018
	2778	4/1	338.80	0.61	0.99	0.19	0.018
	2779	4/1	322.70	0.62	0.91	0.15	0.017
		N	10	10	10	10	10
		Mean	343.74	0.60	1.01	0.17	0.019
		Sdev	35.627	0.037	0.113	0.031	0.0033

Note: Data collected using grace days.

Appendix 9
Relative Organ Weights (% Body Weight)
Test period
Days 29 Interim Sacrifice

Fexinidazole — Study Number: 0504-2007

Dose Level	Animal Number	Group/ Subgroup	Terminal Body Wt(g)	SPLEEN	KIDNEYS	LIVER	THYMUS
			Females				
vehicle							
	2785	1/1	212.40	0.27	0.72	2.85	0.165
	2786	1/1	243.20	0.25	0.67	2.61	0.140
	2787	1/1	219.60	0.30	0.72	2.63	0.105
	2788	1/1	222.60	0.29	0.73	2.56	0.189
	2789	1/1	208.30	0.30	0.67	2.53	0.168
	2790	1/1	224.50	0.24	0.83	2.85	0.160
	2791	1/1	207.60	0.19	0.62	2.49	0.140
	2792	1/1	234.00	0.21	0.75	2.76	0.120
	2793	1/1	217.30	0.27	0.77	2.68	0.198
	2794	1/1	238.50	0.25	0.72	3.01	0.130
		N	10	10	10	10	10
		Mean	222.80	0.26	0.72	2.70	0.151
		Sdev	12.387	0.035	0.059	0.168	0.0298
50 mg/kg							
	2800	2/1	216.80	0.26	0.77	2.78	0.148
	2801	2/1	247.90	0.25	0.65	2.80	0.125
	2802	2/1	235.20	0.34	0.71	2.61	0.157
	2803	2/1	225.20	0.33	0.74	2.99	0.138
	2804	2/1	209.90	0.32	0.77	2.73	0.200
	2805	2/1	233.00	0.34	0.83	3.05	0.193
	2806	2/1	220.30	0.34	0.70	2.86	0.232
	2807	2/1	220.50	0.23	0.68	2.81	0.136
	2808	2/1	230.60	0.26	0.68	3.09	0.173
	2809	2/1	238.70	0.33	0.77	3.42	0.159
		N	10	10	10	10	10
		Mean	227.81	0.30	0.73	2.91	0.166
		Sdev	11.403	0.043	0.055	0.230	0.0335
200 mg/kg							
	2810	3/1	218.20	0.25	0.77	2.99	0.183
	2811	3/1	237.90	0.23	0.74	2.64	0.105
	2812	3/1	235.20	0.27	0.70	2.85	0.153
	2813	3/1	240.00	0.24	0.78	3.19	0.133
	2814	3/1	213.00	0.27	0.70	2.84	0.183
	2815	3/1	219.20	0.27	0.70	3.03	0.178
	2816	3/1	231.50	0.25	0.76	2.86	0.121
	2817	3/1	238.10	0.29	0.76	3.09	0.130
	2818	3/1	233.20	0.21	0.72	3.13	0.180
	2819	3/1	221.90	0.20	0.77	3.11	0.153

Note: Data collected using grace days.

Appendix 9
Relative Organ Weights (% Body Weight)
Test period
Days 29 Interim Sacrifice

Fexinidazole | Study Number: 0504-2007

Dose Level	Animal Number	Group/ Subgroup	Terminal Body Wt(g)	SPLEEN	KIDNEYS	LIVER	THYMUS
			Females				
200 mg/kg							
		N	10	10	10	10	10
		Mean	228.82	0.25	0.74	2.97	0.152
		Sdev	9.799	0.029	0.033	0.172	0.0287
800 mg/kg							
	2820	4/1	207.90	0.25	0.86	3.74	0.159
	2821	4/1	195.40	0.24	0.78	3.35	0.123
	2822	4/1	228.20	0.21	0.73	3.06	0.114
	2823	4/1	248.60	0.22	0.65	3.74	0.185
	2825	4/1	213.50	0.25	0.99	4.89	0.164
	2826	4/1	212.30	0.25	0.74	3.62	0.108
	2827	4/1	223.50	0.29	0.71	3.59	0.148
	2828	4/1	219.90	0.27	0.76	4.13	0.150
	2829	4/1	220.00	0.21	0.75	3.49	0.145
		N	9	9	9	9	9
		Mean	218.81	0.24	0.77	3.73	0.144
		Sdev	14.739	0.026	0.098	0.522	0.0249

Note: Data collected using grace days.

Appendix 9
Relative Organ Weights (% Body Weight)
Test period
Days 29 Interim Sacrifice

Fexinidazole | Study Number: 0504-2007

Dose Level	Animal Number	Group/ Subgroup	Terminal Body Wt(g)	HEART	BRAIN	OVARIES	ADRENALS
Females							
vehicle							
	2785	1/1	212.40	0.39	0.90	0.062	0.034
	2786	1/1	243.20	0.35	0.86	0.061	0.028
	2787	1/1	219.60	0.35	0.91	0.048	0.031
	2788	1/1	222.60	0.38	0.84	0.052	0.034
	2789	1/1	208.30	0.38	0.90	0.058	0.036
	2790	1/1	224.50	0.38	0.91	0.060	0.030
	2791	1/1	207.60	0.34	0.89	0.057	0.036
	2792	1/1	234.00	0.36	0.81	0.043	0.031
	2793	1/1	217.30	0.39	0.81	0.045	0.033
	2794	1/1	238.50	0.36	0.75	0.051	0.040
		N	10	10	10	10	10
		Mean	222.80	0.37	0.86	0.054	0.033
		Sdev	12.387	0.019	0.056	0.0069	0.0036
50 mg/kg							
	2800	2/1	216.80	0.36	0.94	0.060	0.032
	2801	2/1	247.90	0.35	0.79	0.049	0.032
	2802	2/1	235.20	0.35	0.89	0.051	0.029
	2803	2/1	225.20	0.40	0.82	0.076	0.032
	2804	2/1	209.90	0.40	0.89	0.052	0.033
	2805	2/1	233.00	0.39	0.82	0.056	0.031
	2806	2/1	220.30	0.38	0.86	0.049	0.037
	2807	2/1	220.50	0.42	0.85	0.053	0.027
	2808	2/1	230.60	0.38	0.79	0.051	0.033
	2809	2/1	238.70	0.35	0.80	0.048	0.035
		N	10	10	10	10	10
		Mean	227.81	0.38	0.84	0.055	0.032
		Sdev	11.403	0.024	0.050	0.0083	0.0030
200 mg/kg							
	2810	3/1	218.20	0.37	0.85	0.043	0.034
	2811	3/1	237.90	0.30	0.78	0.047	0.036
	2812	3/1	235.20	0.35	0.86	0.043	0.033
	2813	3/1	240.00	0.35	0.78	0.050	0.030
	2814	3/1	213.00	0.32	0.87	0.041	0.026
	2815	3/1	219.20	0.37	0.80	0.051	0.022
	2816	3/1	231.50	0.35	0.84	0.050	0.037
	2817	3/1	238.10	0.36	0.80	0.063	0.029
	2818	3/1	233.20	0.35	0.76	0.050	0.028
	2819	3/1	221.90	0.41	0.84	0.051	0.035

Note: Data collected using grace days.

Appendix 9
Relative Organ Weights (% Body Weight)
Test period
Days 29 Interim Sacrifice

Fexinidazole | Study Number: 0504-2007

Dose Level	Animal Number	Group/ Subgroup	Terminal Body Wt(g)	HEART	BRAIN	OVARIES	ADRENALS
			Females				
200 mg/kg							
		N	10	10	10	10	10
		Mean	228.82	0.35	0.82	0.049	0.031
		Sdev	9.799	0.028	0.038	0.0062	0.0047
800 mg/kg							
	2820	4/1	207.90	0.40	0.91	0.059	0.029
	2821	4/1	195.40	0.34	0.95	0.046	0.032
	2822	4/1	228.20	0.35	0.78	0.059	0.032
	2823	4/1	248.60	0.35	0.75	0.051	0.032
	2825	4/1	213.50	0.36	0.93	0.060	0.032
	2826	4/1	212.30	0.35	0.86	0.052	0.032
	2827	4/1	223.50	0.38	0.85	0.050	0.031
	2828	4/1	219.90	0.40	0.83	0.064	0.032
	2829	4/1	220.00	0.34	0.85	0.051	0.029
		N	9	9	9	9	9
		Mean	218.81	0.36	0.86	0.055	0.031
		Sdev	14.739	0.024	0.066	0.0059	0.0012

Note: Data collected using grace days.

Appendix 9
Relative Organ Weights (% Body Weight)
Test period
Days 43 Final Sacrifice

Fexinidazole
Study Number: 0504-2007

Dose Level	Animal Number	Group/ Subgroup	Terminal Body Wt(g)	SPLEEN	KIDNEYS	LIVER	THYMUS	HEART
				Males				
vehicle								
	2745	1/1	405.30	0.16	0.72	2.40	0.116	0.33
	2746	1/1	370.80	0.14	0.69	2.43	0.124	0.34
	2747	1/1	436.60	0.17	0.72	2.71	0.108	0.33
	2748	1/1	458.60	0.12	0.53	2.00	0.096	0.31
	2749	1/1	363.60	0.20	0.93	3.42	0.140	0.43
		N	5	5	5	5	5	5
		Mean	406.98	0.16	0.72	2.59	0.117	0.35
		Sdev	41.035	0.030	0.140	0.526	0.0168	0.046
800 mg/kg								
	2780	4/1	369.00	0.17	0.70	2.78	0.144	0.33
	2781	4/1	341.60	0.24	0.72	2.67	0.114	0.40
	2782	4/1	359.30	0.16	0.72	3.34	0.075	0.37
	2783	4/1	407.10	0.17	0.74	2.74	0.103	0.35
	2784	4/1	385.50	0.19	0.71	2.53	0.114	0.35
		N	5	5	5	5	5	5
		Mean	372.50	0.19	0.72	2.81	0.110	0.36
		Sdev	25.037	0.029	0.016	0.310	0.0246	0.026

Note: Data collected using grace days.

Appendix 9
Relative Organ Weights (% Body Weight)
Test period
Days 43 Final Sacrifice

Fexinidazole

Study Number: 0504-2007

Dose Level	Animal Number	Group/ Subgroup	Terminal Body Wt(g)	BRAIN	TESTES	PROSTATE	ADRENALS
Males							
vehicle							
	2745	1/1	405.30	0.51	0.77	0.19	0.013
	2746	1/1	370.80	0.57	0.84	0.11	0.012
	2747	1/1	436.60	0.45	0.82	0.14	0.014
	2748	1/1	458.60	0.42	0.76	0.10	0.011
	2749	1/1	363.60	0.56	1.01	0.20	0.021
		N	5	5	5	5	5
		Mean	406.98	0.50	0.84	0.15	0.014
		Sdev	41.035	0.065	0.099	0.046	0.0040
800 mg/kg							
	2780	4/1	369.00	0.56	0.93	0.14	0.018
	2781	4/1	341.60	0.61	1.11	0.19	0.020
	2782	4/1	359.30	0.58	0.94	0.19	0.014
	2783	4/1	407.10	0.51	0.97	0.15	0.017
	2784	4/1	385.50	0.54	0.81	0.19	0.015
		N	5	5	5	5	5
		Mean	372.50	0.56	0.95	0.17	0.017
		Sdev	25.037	0.042	0.107	0.023	0.0024

Note: Data collected using grace days.

Appendix 9
Relative Organ Weights (% Body Weight)
Test period
Days 43 Final Sacrifice

Fexinidazole — Study Number: 0504-2007

Dose Level	Animal Number	Group/ Subgroup	Terminal Body Wt(g)	SPLEEN	KIDNEYS	LIVER	THYMUS
Females							
vehicle							
	2795	1/1	231.10	0.23	0.70	2.70	0.108
	2796	1/1	233.10	0.22	0.75	2.62	0.163
	2797	1/1	227.50	0.22	0.78	2.93	0.114
	2798	1/1	255.10	0.22	0.65	2.49	0.102
	2799	1/1	250.30	0.21	0.80	2.60	0.144
		N	5	5	5	5	5
		Mean	239.42	0.22	0.74	2.67	0.126
		Sdev	12.405	0.007	0.061	0.164	0.0261
800 mg/kg							
	2830	4/1	245.00	0.33	0.70	2.93	0.110
	2831	4/1	206.80	0.22	0.74	3.02	0.111
	2832	4/1	213.00	0.21	0.73	2.92	0.150
	2833	4/1	229.20	0.21	0.61	2.70	0.118
	2834	4/1	228.70	0.19	0.68	2.93	0.179
		N	5	5	5	5	5
		Mean	224.54	0.23	0.69	2.90	0.134
		Sdev	15.046	0.054	0.052	0.120	0.0303

Note: Data collected using grace days.

Appendix 9
Relative Organ Weights (% Body Weight)
Test period
Days 43 Final Sacrifice

Fexinidazole

Study Number: 0504-2007

Dose Level	Animal Number	Group/ Subgroup	Terminal Body Wt(g)	HEART	BRAIN	OVARIES	ADRENALS
**F e m a l e s**							
vehicle							
	2795	1/1	231.10	0.35	0.82	0.041	0.031
	2796	1/1	233.10	0.39	0.82	0.045	0.033
	2797	1/1	227.50	0.39	0.94	0.053	0.038
	2798	1/1	255.10	0.36	0.78	0.043	0.028
	2799	1/1	250.30	0.37	0.77	0.033	0.026
		N	5	5	5	5	5
		Mean	239.42	0.37	0.83	0.043	0.031
		Sdev	12.405	0.014	0.067	0.0073	0.0044
800 mg/kg							
	2830	4/1	245.00	0.38	0.73	0.040	0.026
	2831	4/1	206.80	0.39	0.92	0.045	0.028
	2832	4/1	213.00	0.40	0.84	0.040	0.030
	2833	4/1	229.20	0.33	0.83	0.040	0.024
	2834	4/1	228.70	0.38	0.82	0.042	0.025
		N	5	5	5	5	5
		Mean	224.54	0.37	0.83	0.041	0.027
		Sdev	15.046	0.028	0.070	0.0022	0.0025

Note: Data collected using grace days.

Appendix 10 Gross Necropsy and Microscopic Observations

Appendix 10
Individual Animal Microscopic vs. Gross Observations

Fexinidazole — Study Number: 0504-2007

ANIMAL: 2735 SEX: Male GROUP: 1 DOSE LEVEL: vehicle
DAY OF DEATH: 30 Test period STATUS: Scheduled phase sacrifice # 1 TERMINAL BODY WEIGHT (g) : 380.9

Tissue	Gross observations/comments	Correlated Microscopic Observations
GENER. CONDITION . . .	GOOD	No micropathology observations on tissue.
HARDERIAN GLANDS . . .	No gross observations on tissue	PORPHYRIN DEPOSITS, Minimal, Multifocal.
LIVER	No gross observations on tissue	CHRONIC INFLAMMATION, Minimal, Multifocal.<br>HEPATOCELLULAR VACUOLATION,PERIPORTAL, Minimal.
MANDIBULAR L.N.	No gross observations on tissue	PLASMACYTOSIS, Moderate, Diffuse, Unilateral..
LUNG	No gross observations on tissue	ALVEOLAR MACROPHAGE INFILTRATION, Minimal, Multifocal.
OPTIC NERVES	No gross observations on tissue	Tissue is unremarkable.<br>sections of optic nerves are included in block 15.
PARATHYROIDS	No gross observations on tissue	ONLY ONE PARATHYROID AVAILABLE FOR EXAMINATION, Present.
SPLEEN	No gross observations on tissue	EXTRAMEDULLARY HEMOPOIESIS, Minimal, Multifocal.

The following tissues have no gross observations and were not examined microscopically:
No tissue to list.

Appendix 10
Individual Animal Microscopic vs. Gross Observations

Fexinidazole Study Number: 0504-2007

ANIMAL: 2735 SEX: Male GROUP: 1 DOSE LEVEL: vehicle
DAY OF DEATH: 30 Test period STATUS: Scheduled phase sacrifice # 1 TERMINAL BODY WEIGHT (g) : 380.9

Tissue	Gross observations/comments	Correlated Microscopic Observations

The following tissues have no gross observations and were marked as unremarkable microscopically:

ADRENALS	AORTA	BONE MARROW	BRAIN	CECUM
COLON	DIAPHRAGM	DUODENUM	EPIDIDYMIDES	ESOPHAGUS
EYES	FEMUR	HEART	ILEUM	JEJUNUM
KIDNEYS	MESENTERIC L.N.	MAMMARY GLAND	SKELETAL MUSCLE	SCIATIC NERVE
PANCREAS	PITUITARY	PROSTATE	SPINAL CORD CERV	SPINAL CORD THOR
MANDIBULAR S.G.	PAROTIDS	STIFLE JOINT	SKIN	STOMACH
STERNUM	SEMINAL VESICLES	TESTES	THYROIDS	THYMUS
TONGUE	TRACHEA	URINARY BLADDER		

Appendix 10
Individual Animal Microscopic vs. Gross Observations

Fexinidazole   Study Number: 0504-2007

ANIMAL: 2736 | SEX: Male | GROUP: 1 | DOSE LEVEL: vehicle
DAY OF DEATH: 30 Test period | STATUS: Scheduled phase sacrifice # 1 | TERMINAL BODY WEIGHT (g) : 396.8

Tissue	Gross observations/comments	Correlated Microscopic Observations
GENER. CONDITION . . .	GOOD	No micropathology observations on tissue.
KIDNEYS	No gross observations on tissue	CHRONIC INFLAMMATION, Minimal, Multifocal.<br>CORTICAL TUBULAR REGENERATIVE BASOPHILIA, Minimal, Multifocal.
HARDERIAN GLANDS . . .	No gross observations on tissue	PORPHYRIN DEPOSITS, Minimal, Multifocal.
LIVER	No gross observations on tissue	CHRONIC INFLAMMATION, Minimal, Multifocal.
MANDIBULAR L.N.	No gross observations on tissue	PLASMACYTOSIS, Moderate, Diffuse.
PROSTATE	No gross observations on tissue	LYMPHOCYTIC INFILTRATION, Minimal, Multifocal.
PARATHYROIDS	No gross observations on tissue	ONLY ONE PARATHYROID AVAILABLE FOR EXAMINATION, Present.
SPLEEN	No gross observations on tissue	EXTRAMEDULLARY HEMOPOIESIS, Minimal, Multifocal.

The following tissues have no gross observations and were not examined microscopically:
No tissue to list.

Appendix 10
Individual Animal Microscopic vs. Gross Observations

Fexinidazole | Study Number: 0504-2007

ANIMAL: 2736 | SEX: Male | GROUP: 1 | DOSE LEVEL: vehicle
DAY OF DEATH: 30 Test period | STATUS: Scheduled phase sacrifice # 1 | TERMINAL BODY WEIGHT (g) : 396.8

Tissue	Gross observations/comments	Correlated Microscopic Observations

The following tissues have no gross observations and were marked as unremarkable microscopically:

ADRENALS	AORTA	BONE MARROW	BRAIN	CECUM
COLON	DIAPHRAGM	DUODENUM	EPIDIDYMIDES	ESOPHAGUS
EYES	FEMUR	HEART	ILEUM	JEJUNUM
MESENTERIC L.N.	LUNG	MAMMARY GLAND	SKELETAL MUSCLE	SCIATIC NERVE
OPTIC NERVES	PANCREAS	PITUITARY	SPINAL CORD CERV	SPINAL CORD THOR
MANDIBULAR S.G.	PAROTIDS	STIFLE JOINT	SKIN	STOMACH
STERNUM	SEMINAL VESICLES	TESTES	THYROIDS	THYMUS
TONGUE	TRACHEA	URINARY BLADDER		

Appendix 10
Individual Animal Microscopic vs. Gross Observations

Fexinidazole   Study Number: 0504-2007

ANIMAL: 2737   SEX: Male   GROUP: 1   DOSE LEVEL: vehicle
DAY OF DEATH: 30 Test period   STATUS: Scheduled phase sacrifice # 1   TERMINAL BODY WEIGHT (g) : 422.8

Tissue	Gross observations/comments	Correlated Microscopic Observations
GENER. CONDITION . . .	GOOD	No micropathology observations on tissue.
HEART	No gross observations on tissue	MYOCARDIAL INFLAMMATION, Minimal, Focal. / of septum.
LIVER	No gross observations on tissue	CHRONIC INFLAMMATION, Minimal, Multifocal.
MANDIBULAR L.N.	No gross observations on tissue	PLASMACYTOSIS, Marked, Diffuse, Unilateral..
LUNG	No gross observations on tissue	ALVEOLAR HEMORRHAGE, Minimal, Multifocal. / with associated hematoidin crystals.
		ACUTE INFLAMMATION, Slight, Multifocal.
		ALVEOLAR MACROPHAGE INFILTRATION, Slight, Multifocal.
OPTIC NERVES	No gross observations on tissue	ONLY ONE NERVE IS AVAILABLE FOR EXAMINATION, Present.
PANCREAS	No gross observations on tissue	CHRONIC INFLAMMATION, Minimal, Focal.

The following tissues have no gross observations and were not examined microscopically:
No tissue to list.

Appendix 10
Individual Animal Microscopic vs. Gross Observations

Fexinidazole   Study Number: 0504-2007

ANIMAL: 2737   SEX: Male   GROUP: 1   DOSE LEVEL: vehicle
DAY OF DEATH: 30 Test period   STATUS: Scheduled phase sacrifice # 1   TERMINAL BODY WEIGHT (g) : 422.8

Tissue	Gross observations/comments	Correlated Microscopic Observations

The following tissues have no gross observations and were marked as unremarkable microscopically:

ADRENALS	AORTA	BONE MARROW	BRAIN	CECUM
COLON	DIAPHRAGM	DUODENUM	EPIDIDYMIDES	ESOPHAGUS
EYES	FEMUR	ILEUM	JEJUNUM	KIDNEYS
HARDERIAN GLANDS	MESENTERIC L.N.	MAMMARY GLAND	SKELETAL MUSCLE	SCIATIC NERVE
PITUITARY	PROSTATE	PARATHYROIDS	SPINAL CORD CERV	SPINAL CORD THOR
MANDIBULAR S.G.	PAROTIDS	STIFLE JOINT	SKIN	SPLEEN
STOMACH	STERNUM	SEMINAL VESICLES	TESTES	THYROIDS
THYMUS	TONGUE	TRACHEA	URINARY BLADDER	

Appendix 10
Individual Animal Microscopic vs. Gross Observations

Fexinidazole | Study Number: 0504-2007

ANIMAL: 2738 | SEX: Male | GROUP: 1 | DOSE LEVEL: vehicle
DAY OF DEATH: 30 Test period | STATUS: Scheduled phase sacrifice # 1 | TERMINAL BODY WEIGHT (g) : 383.8

Tissue	Gross observations/comments	Correlated Microscopic Observations
ADRENALS	No gross observations on tissue	CORTICAL VACUOLATION, Slight, Diffuse.
GENER. CONDITION	GOOD	No micropathology observations on tissue.
HARDERIAN GLANDS	No gross observations on tissue	PORPHYRIN DEPOSITS, Minimal, Multifocal.<br>LYMPHOCYTIC INFILTRATION, Minimal, Multifocal.
LIVER	No gross observations on tissue	CHRONIC INFLAMMATION, Minimal, Multifocal.<br>HEPATOCELLULAR VACUOLATION,DIFFUSE, Minimal.
MANDIBULAR L.N.	No gross observations on tissue	PLASMACYTOSIS, Slight, Diffuse.
LUNG	No gross observations on tissue	ALVEOLAR MACROPHAGE INFILTRATION, Minimal, Multifocal.
SKELETAL MUSCLE	No gross observations on tissue	CHRONIC INFLAMMATION, Minimal, Multifocal.

The following tissues have no gross observations and were not examined microscopically:
No tissue to list.

The following tissues have no gross observations and were marked as unremarkable microscopically:
AORTA BONE MARROW BRAIN CECUM COLON

Appendix 10
Individual Animal Microscopic vs. Gross Observations

Fexinidazole Study Number: 0504-2007

ANIMAL: 2738 SEX: Male GROUP: 1 DOSE LEVEL: vehicle
DAY OF DEATH: 30 Test period STATUS: Scheduled phase sacrifice # 1 TERMINAL BODY WEIGHT (g) : 383.8

Tissue	Gross observations/comments	Correlated Microscopic Observations

The following tissues have no gross observations and were marked as unremarkable microscopically:

DIAPHRAGM	DUODENUM	EPIDIDYMIDES	ESOPHAGUS	EYES
FEMUR	HEART	ILEUM	JEJUNUM	KIDNEYS
MESENTERIC L.N.	MAMMARY GLAND	SCIATIC NERVE	OPTIC NERVES	PANCREAS
PITUITARY	PROSTATE	PARATHYROIDS	SPINAL CORD CERV	SPINAL CORD THOR
MANDIBULAR S.G.	PAROTIDS	STIFLE JOINT	SKIN	SPLEEN
STOMACH	STERNUM	SEMINAL VESICLES	TESTES	THYROIDS
THYMUS	TONGUE	TRACHEA	URINARY BLADDER	

Appendix 10
Individual Animal Microscopic vs. Gross Observations

Fexinidazole | Study Number: 0504-2007

ANIMAL: 2739 | SEX: Male | GROUP: 1 | DOSE LEVEL: vehicle
DAY OF DEATH: 30 Test period | STATUS: Scheduled phase sacrifice # 1 | TERMINAL BODY WEIGHT (g) : 419.0

Tissue	Gross observations/comments	Correlated Microscopic Observations
AORTA	No gross observations on tissue	INFLAMMATION OF ADJACENT TISSUES, Minimal, Multifocal.
GENER. CONDITION . . .	GOOD	No micropathology observations on tissue.
HEART	No gross observations on tissue	MYOCARDIAL INFLAMMATION, Minimal, Multifocal.
HARDERIAN GLANDS . . .	No gross observations on tissue	PORPHYRIN DEPOSITS, Minimal, Multifocal.
LIVER	No gross observations on tissue	CHRONIC INFLAMMATION, Minimal, Multifocal.
		HEPATOCELLULAR VACUOLATION,PERIPORTAL, Minimal.
MANDIBULAR L.N.	No gross observations on tissue	PLASMACYTOSIS, Slight, Diffuse.
THYROIDS	No gross observations on tissue	LYMPHOCYTIC INFILTRATION, Minimal, Focal, Unilateral..

The following tissues have no gross observations and were not examined microscopically:
No tissue to list.

The following tissues have no gross observations and were marked as unremarkable microscopically:
ADRENALS BONE MARROW BRAIN CECUM COLON

Appendix 10
Individual Animal Microscopic vs. Gross Observations

Fexinidazole | Study Number: 0504-2007

ANIMAL: 2739 | SEX: Male | GROUP: 1 | DOSE LEVEL: vehicle
DAY OF DEATH: 30 Test period | STATUS: Scheduled phase sacrifice # 1 | TERMINAL BODY WEIGHT (g) : 419.0

Tissue	Gross observations/comments	Correlated Microscopic Observations

The following tissues have no gross observations and were marked as unremarkable microscopically:

DIAPHRAGM	DUODENUM	EPIDIDYMIDES	ESOPHAGUS	EYES
FEMUR	ILEUM	JEJUNUM	KIDNEYS	MESENTERIC L.N.
LUNG	MAMMARY GLAND	SKELETAL MUSCLE	SCIATIC NERVE	OPTIC NERVES
PANCREAS	PITUITARY	PROSTATE	PARATHYROIDS	SPINAL CORD CERV
SPINAL CORD THOR	MANDIBULAR S.G.	PAROTIDS	STIFLE JOINT	SKIN
SPLEEN	STOMACH	STERNUM	SEMINAL VESICLES	TESTES
THYMUS	TONGUE	TRACHEA	URINARY BLADDER	

Appendix 10
Individual Animal Microscopic vs. Gross Observations

Fexinidazole | Study Number: 0504-2007

ANIMAL: 2740 | SEX: Male | GROUP: 1 | DOSE LEVEL: vehicle
DAY OF DEATH: 29 Test period | STATUS: Scheduled phase sacrifice # 1 | TERMINAL BODY WEIGHT (g) : 410.5

Tissue	Gross observations/comments	Correlated Microscopic Observations
GENER. CONDITION . . .	GOOD	No micropathology observations on tissue.
KIDNEYS	No gross observations on tissue	CHRONIC INFLAMMATION, Minimal, Multifocal, Unilateral right..
LIVER	No gross observations on tissue	CHRONIC INFLAMMATION, Minimal, Multifocal.
MANDIBULAR L.N.	No gross observations on tissue	PLASMACYTOSIS, Moderate, Diffuse, Unilateral..
PARATHYROIDS	No gross observations on tissue	ONLY ONE PARATHYROID AVAILABLE FOR EXAMINATION, Present.
URINARY BLADDER	No gross observations on tissue	PROTEINACEOUS PLUG, Present.

The following tissues have no gross observations and were not examined microscopically:
No tissue to list.

The following tissues have no gross observations and were marked as unremarkable microscopically:

ADRENALS	AORTA	BONE MARROW	BRAIN	CECUM
COLON	DIAPHRAGM	DUODENUM	EPIDIDYMIDES	ESOPHAGUS
EYES	FEMUR	HEART	ILEUM	JEJUNUM
HARDERIAN GLANDS	MESENTERIC L.N.	LUNG	MAMMARY GLAND	SKELETAL MUSCLE
SCIATIC NERVE	OPTIC NERVES	PANCREAS	PITUITARY	PROSTATE
SPINAL CORD CERV	SPINAL CORD THOR	MANDIBULAR S.G.	PAROTIDS	STIFLE JOINT
SKIN	SPLEEN	STOMACH	STERNUM	SEMINAL VESICLES
TESTES	THYROIDS	THYMUS	TONGUE	TRACHEA

Appendix 10
Individual Animal Microscopic vs. Gross Observations

Fexinidazole | Study Number: 0504-2007

ANIMAL: 2741 | SEX: Male | GROUP: 1 | DOSE LEVEL: vehicle
DAY OF DEATH: 29 Test period | STATUS: Scheduled phase sacrifice # 1 | TERMINAL BODY WEIGHT (g) : 381.1

Tissue	Gross observations/comments	Correlated Microscopic Observations
EPIDIDYMIDES	No gross observations on tissue	LYMPHOCYTIC INFILTRATION, Minimal, Multifocal.
GENER. CONDITION . . .	GOOD	No micropathology observations on tissue.
HEART	No gross observations on tissue	MYOCARDIAL INFLAMMATION, Minimal, Multifocal.
KIDNEYS	No gross observations on tissue	CORTICAL TUBULAR REGENERATIVE BASOPHILIA, Minimal, Multifocal.
HARDERIAN GLANDS . . .	No gross observations on tissue	PORPHYRIN DEPOSITS, Minimal, Multifocal.
LIVER	No gross observations on tissue	CHRONIC INFLAMMATION, Slight, Multifocal.<br>HEPATOCELLULAR VACUOLATION,PERIPORTAL, Minimal.
OPTIC NERVES	No gross observations on tissue	Tissue is missing.
PROSTATE	No gross observations on tissue	LYMPHOCYTIC INFILTRATION, Minimal, Multifocal.

The following tissues have no gross observations and were not examined microscopically:
No tissue to list.

Appendix 10
Individual Animal Microscopic vs. Gross Observations

Fexinidazole | Study Number: 0504-2007

ANIMAL: 2741 | SEX: Male | GROUP: 1 | DOSE LEVEL: vehicle
DAY OF DEATH: 29 Test period | STATUS: Scheduled phase sacrifice # 1 | TERMINAL BODY WEIGHT (g) : 381.1

Tissue	Gross observations/comments	Correlated Microscopic Observations

The following tissues have no gross observations and were marked as unremarkable microscopically:

ADRENALS	AORTA	BONE MARROW	BRAIN	CECUM
COLON	DIAPHRAGM	DUODENUM	ESOPHAGUS	EYES
FEMUR	ILEUM	JEJUNUM	MANDIBULAR L.N.	MESENTERIC L.N.
LUNG	MAMMARY GLAND	SKELETAL MUSCLE	SCIATIC NERVE	PANCREAS
PITUITARY	PARATHYROIDS	SPINAL CORD CERV	SPINAL CORD THOR	MANDIBULAR S.G.
PAROTIDS	STIFLE JOINT	SKIN	SPLEEN	STOMACH
STERNUM	SEMINAL VESICLES	TESTES	THYROIDS	THYMUS
TONGUE	TRACHEA	URINARY BLADDER		

Appendix 10
Individual Animal Microscopic vs. Gross Observations

Fexinidazole | Study Number: 0504-2007

ANIMAL: 2742 | SEX: Male | GROUP: 1 | DOSE LEVEL: vehicle
DAY OF DEATH: 29 Test period | STATUS: Scheduled phase sacrifice # 1 | TERMINAL BODY WEIGHT (g) : 348.7

Tissue	Gross observations/comments	Correlated Microscopic Observations
GENER. CONDITION . . .	GOOD	No micropathology observations on tissue.
HARDERIAN GLANDS . . .	No gross observations on tissue	LYMPHOCYTIC INFILTRATION, Minimal, Multifocal.
LIVER	No gross observations on tissue	CHRONIC INFLAMMATION, Minimal, Multifocal.
		HEPATOCELLULAR VACUOLATION,PERIPORTAL, Minimal.
LUNG	No gross observations on tissue	ACUTE INFLAMMATION, Minimal, Multifocal.
		ALVEOLAR MACROPHAGE INFILTRATION, Minimal, Multifocal.
PROSTATE	No gross observations on tissue	LYMPHOCYTIC INFILTRATION, Minimal, Multifocal.
THYROIDS	No gross observations on tissue	LYMPHOCYTIC INFILTRATION, Minimal, Multifocal, Unilateral..

The following tissues have no gross observations and were not examined microscopically:
No tissue to list.

The following tissues have no gross observations and were marked as unremarkable microscopically:
ADRENALS AORTA BONE MARROW BRAIN CECUM

Appendix 10
Individual Animal Microscopic vs. Gross Observations

Fexinidazole Study Number: 0504-2007

ANIMAL: 2742 SEX: Male GROUP: 1 DOSE LEVEL: vehicle
DAY OF DEATH: 29 Test period STATUS: Scheduled phase sacrifice # 1 TERMINAL BODY WEIGHT (g) : 348.7

Tissue	Gross observations/comments	Correlated Microscopic Observations

The following tissues have no gross observations and were marked as unremarkable microscopically:

COLON	DIAPHRAGM	DUODENUM	EPIDIDYMIDES	ESOPHAGUS
EYES	FEMUR	HEART	ILEUM	JEJUNUM
KIDNEYS	MANDIBULAR L.N.	MESENTERIC L.N.	MAMMARY GLAND	SKELETAL MUSCLE
SCIATIC NERVE	OPTIC NERVES	PANCREAS	PITUITARY	PARATHYROIDS
SPINAL CORD CERV	SPINAL CORD THOR	MANDIBULAR S.G.	PAROTIDS	STIFLE JOINT
SKIN	SPLEEN	STOMACH	STERNUM	SEMINAL VESICLES
TESTES	THYMUS	TONGUE	TRACHEA	URINARY BLADDER

Appendix 10
Individual Animal Microscopic vs. Gross Observations

Fexinidazole   Study Number: 0504-2007

ANIMAL: 2743   SEX: Male   GROUP: 1   DOSE LEVEL: vehicle
DAY OF DEATH: 29 Test period   STATUS: Scheduled phase sacrifice # 1   TERMINAL BODY WEIGHT (g) : 373.7

Tissue	Gross observations/comments	Correlated Microscopic Observations
GENER. CONDITION . . .	GOOD	No micropathology observations on tissue.
KIDNEYS	No gross observations on tissue	CORTICAL TUBULAR REGENERATIVE BASOPHILIA, Minimal, Multifocal.
LIVER	No gross observations on tissue	CHRONIC INFLAMMATION, Minimal, Multifocal.
MANDIBULAR L.N.	No gross observations on tissue	PLASMACYTOSIS, Slight, Diffuse.
PARATHYROIDS	No gross observations on tissue	ONLY ONE PARATHYROID AVAILABLE FOR EXAMINATION, Present.
SPLEEN	No gross observations on tissue	INFLAMMATION OF THE CAPSULE, Minimal, Focal, Chronic.

The following tissues have no gross observations and were not examined microscopically:
No tissue to list.

The following tissues have no gross observations and were marked as unremarkable microscopically:

ADRENALS	AORTA	BONE MARROW	BRAIN	CECUM
COLON	DIAPHRAGM	DUODENUM	EPIDIDYMIDES	ESOPHAGUS
EYES	FEMUR	HEART	ILEUM	JEJUNUM
HARDERIAN GLANDS	MESENTERIC L.N.	LUNG	MAMMARY GLAND	SKELETAL MUSCLE
SCIATIC NERVE	OPTIC NERVES	PANCREAS	PITUITARY	PROSTATE
SPINAL CORD CERV	SPINAL CORD THOR	MANDIBULAR S.G.	PAROTIDS	STIFLE JOINT
SKIN	STOMACH	STERNUM	SEMINAL VESICLES	TESTES
THYROIDS	THYMUS	TONGUE	TRACHEA	URINARY BLADDER

Appendix 10
Individual Animal Microscopic vs. Gross Observations

Fexinidazole | Study Number: 0504-2007

ANIMAL: 2744 | SEX: Male | GROUP: 1 | DOSE LEVEL: vehicle
DAY OF DEATH: 29 Test period | STATUS: Scheduled phase sacrifice # 1 | TERMINAL BODY WEIGHT (g) : 398.0

Tissue	Gross observations/comments	Correlated Microscopic Observations
ADRENALS	No gross observations on tissue	CORTICAL VACUOLATION, Minimal, Multifocal.
GENER. CONDITION . . .	GOOD	No micropathology observations on tissue.
KIDNEYS	No gross observations on tissue	CHRONIC INFLAMMATION, Minimal, Multifocal.
		PYELITIS, Slight, Multifocal. / acute to chronic.
HARDERIAN GLANDS . . .	No gross observations on tissue	PORPHYRIN DEPOSITS, Minimal, Multifocal.
LIVER	No gross observations on tissue	CHRONIC INFLAMMATION, Minimal, Multifocal.
		HEPATOCELLULAR VACUOLATION,PERIPORTAL, Minimal.
MANDIBULAR L.N.	No gross observations on tissue	PLASMACYTOSIS, Moderate, Diffuse.
LUNG	No gross observations on tissue	ALVEOLAR MACROPHAGE INFILTRATION, Minimal, Multifocal.
MAMMARY GLAND	No gross observations on tissue	NO MAMMARY TISSUE IN THE SECTION, Present.
OPTIC NERVES	No gross observations on tissue	Tissue is unremarkable.
		sections of optic nerves are included in

Appendix 10
Individual Animal Microscopic vs. Gross Observations

Fexinidazole | Study Number: 0504-2007

ANIMAL: 2744 | SEX: Male | GROUP: 1 | DOSE LEVEL: vehicle
DAY OF DEATH: 29 Test period | STATUS: Scheduled phase sacrifice # 1 | TERMINAL BODY WEIGHT (g) : 398.0

Tissue	Gross observations/comments	Correlated Microscopic Observations
		block 15.
PROSTATE	No gross observations on tissue	LYMPHOCYTIC INFILTRATION, Slight, Multifocal.
PARATHYROIDS	No gross observations on tissue	ONLY ONE PARATHYROID AVAILABLE FOR EXAMINATION, Present.
SPLEEN	No gross observations on tissue	EXTRAMEDULLARY HEMOPOIESIS, Minimal, Multifocal.
THYROIDS	No gross observations on tissue	ECTOPIC THYMUS, Present, Unilateral..

The following tissues have no gross observations and were not examined microscopically:
No tissue to list.

The following tissues have no gross observations and were marked as unremarkable microscopically:

AORTA	BONE MARROW	BRAIN	CECUM	COLON
DIAPHRAGM	DUODENUM	EPIDIDYMIDES	ESOPHAGUS	EYES
FEMUR	HEART	ILEUM	JEJUNUM	MESENTERIC L.N.
SKELETAL MUSCLE	SCIATIC NERVE	PANCREAS	PITUITARY	SPINAL CORD CERV
SPINAL CORD THOR	MANDIBULAR S.G.	PAROTIDS	STIFLE JOINT	SKIN
STOMACH	STERNUM	SEMINAL VESICLES	TESTES	THYMUS
TONGUE	TRACHEA	URINARY BLADDER		

Appendix 10
Individual Animal Microscopic vs. Gross Observations

Fexinidazole — Study Number: 0504-2007

ANIMAL: 2745 SEX: Male GROUP: 1 DOSE LEVEL: vehicle
DAY OF DEATH: 43 Test period STATUS: Final phase sacrifice TERMINAL BODY WEIGHT (g) : 405.3

Tissue	Gross observations/comments	Correlated Microscopic Observations
GENER. CONDITION . . .	GOOD	No micropathology observations on tissue.
HARDERIAN GLANDS . . .	No gross observations on tissue	PORPHYRIN DEPOSITS, Minimal, Multifocal.
		HEMORRHAGE,UNILATERAL, Slight, Multifocal.
		ADENITIS,UNILATERAL, Minimal, Multifocal, Chronic.
		LYMPHOCYTIC INFILTRATION, Minimal, Multifocal, Unilateral..
LIVER	No gross observations on tissue	CHRONIC INFLAMMATION, Minimal, Multifocal.
		HEPATOCELLULAR VACUOLATION,PERIPORTAL, Minimal.

The following tissues have no gross observations and were not examined microscopically:

ADRENALS	AORTA	BONE MARROW	BRAIN	CECUM
COLON	DIAPHRAGM	DUODENUM	EPIDIDYMIDES	ESOPHAGUS
EYES	FEMUR	HEART	ILEUM	JEJUNUM
KIDNEYS	MANDIBULAR L.N.	MESENTERIC L.N.	LUNG	MAMMARY GLAND
SKELETAL MUSCLE	SCIATIC NERVE	OPTIC NERVES	PANCREAS	PITUITARY
PROSTATE	PARATHYROIDS	SPINAL CORD CERV	SPINAL CORD THOR	MANDIBULAR S.G.
PAROTIDS	STIFLE JOINT	SKIN	SPLEEN	STOMACH
STERNUM	SEMINAL VESICLES	TESTES	THYROIDS	THYMUS
TONGUE	TRACHEA	URINARY BLADDER		

The following tissues have no gross observations and were marked as unremarkable microscopically:
No tissue to list.

Appendix 10
Individual Animal Microscopic vs. Gross Observations

Fexinidazole | Study Number: 0504-2007

ANIMAL: 2746 | SEX: Male | GROUP: 1 | DOSE LEVEL: vehicle
DAY OF DEATH: 43 Test period | STATUS: Final phase sacrifice | TERMINAL BODY WEIGHT (g) : 370.8

Tissue	Gross observations/comments	Correlated Microscopic Observations
GENER. CONDITION . . .	GOOD	No micropathology observations on tissue.
HARDERIAN GLANDS . . .	No gross observations on tissue	PORPHYRIN DEPOSITS, Moderate, Multifocal.<br>HEMORRHAGE,UNILATERAL, Slight, Multifocal.<br>ADENITIS,UNILATERAL, Slight, Multifocal.<br>LYMPHOCYTIC INFILTRATION, Minimal, Focal, Unilateral..
LIVER	No gross observations on tissue	CHRONIC INFLAMMATION, Minimal, Multifocal.<br>HEPATOCELLULAR VACUOLATION,PERIPORTAL, Minimal.

The following tissues have no gross observations and were not examined microscopically:

ADRENALS	AORTA	BONE MARROW	BRAIN	CECUM
COLON	DIAPHRAGM	DUODENUM	EPIDIDYMIDES	ESOPHAGUS
EYES	FEMUR	HEART	ILEUM	JEJUNUM
KIDNEYS	MANDIBULAR L.N.	MESENTERIC L.N.	LUNG	MAMMARY GLAND
SKELETAL MUSCLE	SCIATIC NERVE	OPTIC NERVES	PANCREAS	PITUITARY
PROSTATE	PARATHYROIDS	SPINAL CORD CERV	SPINAL CORD THOR	MANDIBULAR S.G.
PAROTIDS	STIFLE JOINT	SKIN	SPLEEN	STOMACH
STERNUM	SEMINAL VESICLES	TESTES	THYROIDS	THYMUS
TONGUE	TRACHEA	URINARY BLADDER		

The following tissues have no gross observations and were marked as unremarkable microscopically:
No tissue to list.

Appendix 10
Individual Animal Microscopic vs. Gross Observations

Fexinidazole | Study Number: 0504-2007

ANIMAL: 2747 | SEX: Male | GROUP: 1 | DOSE LEVEL: vehicle
DAY OF DEATH: 43 Test period | STATUS: Final phase sacrifice | TERMINAL BODY WEIGHT (g) : 436.6

Tissue	Gross observations/comments	Correlated Microscopic Observations
GENER. CONDITION	GOOD	No micropathology observations on tissue.
HARDERIAN GLANDS	No gross observations on tissue	PORPHYRIN DEPOSITS, Minimal, Multifocal.
		HEMORRHAGE,UNILATERAL, Slight, Focal.
LIVER	No gross observations on tissue	CHRONIC INFLAMMATION, Minimal, Multifocal.

The following tissues have no gross observations and were not examined microscopically:

ADRENALS	AORTA	BONE MARROW	BRAIN	CECUM
COLON	DIAPHRAGM	DUODENUM	EPIDIDYMIDES	ESOPHAGUS
EYES	FEMUR	HEART	ILEUM	JEJUNUM
KIDNEYS	MANDIBULAR L.N.	MESENTERIC L.N.	LUNG	MAMMARY GLAND
SKELETAL MUSCLE	SCIATIC NERVE	OPTIC NERVES	PANCREAS	PITUITARY
PROSTATE	PARATHYROIDS	SPINAL CORD CERV	SPINAL CORD THOR	MANDIBULAR S.G.
PAROTIDS	STIFLE JOINT	SKIN	SPLEEN	STOMACH
STERNUM	SEMINAL VESICLES	TESTES	THYROIDS	THYMUS
TONGUE	TRACHEA	URINARY BLADDER		

The following tissues have no gross observations and were marked as unremarkable microscopically:
No tissue to list.

Appendix 10
Individual Animal Microscopic vs. Gross Observations

Fexinidazole | Study Number: 0504-2007

ANIMAL: 2748 | SEX: Male | GROUP: 1 | DOSE LEVEL: vehicle
DAY OF DEATH: 43 Test period | STATUS: Final phase sacrifice | TERMINAL BODY WEIGHT (g) : 458.6

Tissue	Gross observations/comments	Correlated Microscopic Observations
GENER. CONDITION . . .	GOOD	No micropathology observations on tissue.
HARDERIAN GLANDS . . .	No gross observations on tissue	PORPHYRIN DEPOSITS, Minimal, Multifocal.<br>HEMORRHAGE,UNILATERAL, Moderate, Multifocal.
LIVER	No gross observations on tissue	CHRONIC INFLAMMATION, Minimal, Multifocal.

The following tissues have no gross observations and were not examined microscopically:

ADRENALS	AORTA	BONE MARROW	BRAIN	CECUM
COLON	DIAPHRAGM	DUODENUM	EPIDIDYMIDES	ESOPHAGUS
EYES	FEMUR	HEART	ILEUM	JEJUNUM
KIDNEYS	MANDIBULAR L.N.	MESENTERIC L.N.	LUNG	MAMMARY GLAND
SKELETAL MUSCLE	SCIATIC NERVE	OPTIC NERVES	PANCREAS	PITUITARY
PROSTATE	PARATHYROIDS	SPINAL CORD CERV	SPINAL CORD THOR	MANDIBULAR S.G.
PAROTIDS	STIFLE JOINT	SKIN	SPLEEN	STOMACH
STERNUM	SEMINAL VESICLES	TESTES	THYROIDS	THYMUS
TONGUE	TRACHEA	URINARY BLADDER		

The following tissues have no gross observations and were marked as unremarkable microscopically:
No tissue to list.

Appendix 10
Individual Animal Microscopic vs. Gross Observations

Fexinidazole   Study Number: 0504-2007

ANIMAL: 2749   SEX: Male   GROUP: 1   DOSE LEVEL: vehicle
DAY OF DEATH: 43 Test period   STATUS: Final phase sacrifice   TERMINAL BODY WEIGHT (g) : 363.6

Tissue	Gross observations/comments	Correlated Microscopic Observations
GENER. CONDITION . . .	GOOD	No micropathology observations on tissue.
HARDERIAN GLANDS . . .	No gross observations on tissue	PORPHYRIN DEPOSITS, Minimal, Multifocal, Unilateral..
		HEMORRHAGE,UNILATERAL, Slight, Multifocal.
		ADENITIS,UNILATERAL, Slight, Multifocal, Chronic.
LIVER	No gross observations on tissue	CHRONIC INFLAMMATION, Minimal, Multifocal.
		HEPATOCELLULAR VACUOLATION,DIFFUSE, Minimal.

The following tissues have no gross observations and were not examined microscopically:

ADRENALS	AORTA	BONE MARROW	BRAIN	CECUM
COLON	DIAPHRAGM	DUODENUM	EPIDIDYMIDES	ESOPHAGUS
EYES	FEMUR	HEART	ILEUM	JEJUNUM
KIDNEYS	MANDIBULAR L.N.	MESENTERIC L.N.	LUNG	MAMMARY GLAND
SKELETAL MUSCLE	SCIATIC NERVE	OPTIC NERVES	PANCREAS	PITUITARY
PROSTATE	PARATHYROIDS	SPINAL CORD CERV	SPINAL CORD THOR	MANDIBULAR S.G.
PAROTIDS	STIFLE JOINT	SKIN	SPLEEN	STOMACH
STERNUM	SEMINAL VESICLES	TESTES	THYROIDS	THYMUS
TONGUE	TRACHEA	URINARY BLADDER		

The following tissues have no gross observations and were marked as unremarkable microscopically:
No tissue to list.

Appendix 10
Individual Animal Microscopic vs. Gross Observations

Fexinidazole

Study Number: 0504-2007

ANIMAL: 2750 SEX: Male GROUP: 2 DOSE LEVEL: 50 mg/kg
DAY OF DEATH: 30 Test period STATUS: Scheduled phase sacrifice # 1 TERMINAL BODY WEIGHT (g) : 433.2

Tissue	Gross observations/comments	Correlated Microscopic Observations
GENER. CONDITION . . .	GOOD	No micropathology observations on tissue.
LIVER	No gross observations on tissue	CHRONIC INFLAMMATION, Minimal, Multifocal.<br>HEPATOCELLULAR HYPERTROPHY,CENTRILOBULAR, Minimal.

The following tissues have no gross observations and were not examined microscopically:

ADRENALS	AORTA	BONE MARROW	BRAIN	CECUM
COLON	DIAPHRAGM	DUODENUM	EPIDIDYMIDES	ESOPHAGUS
EYES	FEMUR	HEART	ILEUM	JEJUNUM
KIDNEYS	MANDIBULAR L.N.	MESENTERIC L.N.	LUNG	MAMMARY GLAND
SKELETAL MUSCLE	SCIATIC NERVE	OPTIC NERVES	PANCREAS	PITUITARY
PROSTATE	PARATHYROIDS	SPINAL CORD CERV	SPINAL CORD THOR	MANDIBULAR S.G.
PAROTIDS	STIFLE JOINT	SKIN	SPLEEN	STOMACH
STERNUM	SEMINAL VESICLES	TESTES	THYROIDS	THYMUS
TONGUE	TRACHEA	URINARY BLADDER		

The following tissues have no gross observations and were marked as unremarkable microscopically:
HARDERIAN GLANDS

Appendix 10
Individual Animal Microscopic vs. Gross Observations

Fexinidazole | Study Number: 0504-2007

ANIMAL: 2751 | SEX: Male | GROUP: 2 | DOSE LEVEL: 50 mg/kg
DAY OF DEATH: 30 Test period | STATUS: Scheduled phase sacrifice # 1 | TERMINAL BODY WEIGHT (g) : 374.7

Tissue	Gross observations/comments	Correlated Microscopic Observations
GENER. CONDITION . . .	GOOD	No micropathology observations on tissue.
HARDERIAN GLANDS . . .	No gross observations on tissue	PORPHYRIN DEPOSITS, Minimal, Multifocal.
LIVER	No gross observations on tissue	CHRONIC INFLAMMATION, Minimal, Multifocal.<br>HEPATOCELLULAR HYPERTROPHY,CENTRILOBULAR, Minimal.<br>FOCAL NECROSIS,SUBCAPSULAR, Minimal. / with associated chronic inflammation.

The following tissues have no gross observations and were not examined microscopically:

ADRENALS	AORTA	BONE MARROW	BRAIN	CECUM
COLON	DIAPHRAGM	DUODENUM	EPIDIDYMIDES	ESOPHAGUS
EYES	FEMUR	HEART	ILEUM	JEJUNUM
KIDNEYS	MANDIBULAR L.N.	MESENTERIC L.N.	LUNG	MAMMARY GLAND
SKELETAL MUSCLE	SCIATIC NERVE	OPTIC NERVES	PANCREAS	PITUITARY
PROSTATE	PARATHYROIDS	SPINAL CORD CERV	SPINAL CORD THOR	MANDIBULAR S.G.
PAROTIDS	STIFLE JOINT	SKIN	SPLEEN	STOMACH
STERNUM	SEMINAL VESICLES	TESTES	THYROIDS	THYMUS
TONGUE	TRACHEA	URINARY BLADDER		

The following tissues have no gross observations and were marked as unremarkable microscopically:
No tissue to list.

Appendix 10
Individual Animal Microscopic vs. Gross Observations

Fexinidazole Study Number: 0504-2007

ANIMAL: 2752 SEX: Male GROUP: 2 DOSE LEVEL: 50 mg/kg
DAY OF DEATH: 30 Test period STATUS: Scheduled phase sacrifice # 1 TERMINAL BODY WEIGHT (g) : 401.4

Tissue	Gross observations/comments	Correlated Microscopic Observations
GENER. CONDITION . . .	GOOD	No micropathology observations on tissue.
HARDERIAN GLANDS . . .	No gross observations on tissue	PORPHYRIN DEPOSITS, Minimal, Multifocal.
LIVER	No gross observations on tissue	CHRONIC INFLAMMATION, Minimal, Multifocal.<br>HEPATOCELLULAR HYPERTROPHY,CENTRILOBULAR, Slight.<br>HEPATOCELLULAR VACUOLATION,PERIPORTAL, Minimal.

The following tissues have no gross observations and were not examined microscopically:

ADRENALS	AORTA	BONE MARROW	BRAIN	CECUM
COLON	DIAPHRAGM	DUODENUM	EPIDIDYMIDES	ESOPHAGUS
EYES	FEMUR	HEART	ILEUM	JEJUNUM
KIDNEYS	MANDIBULAR L.N.	MESENTERIC L.N.	LUNG	MAMMARY GLAND
SKELETAL MUSCLE	SCIATIC NERVE	OPTIC NERVES	PANCREAS	PITUITARY
PROSTATE	PARATHYROIDS	SPINAL CORD CERV	SPINAL CORD THOR	MANDIBULAR S.G.
PAROTIDS	STIFLE JOINT	SKIN	SPLEEN	STOMACH
STERNUM	SEMINAL VESICLES	TESTES	THYROIDS	THYMUS
TONGUE	TRACHEA	URINARY BLADDER		

The following tissues have no gross observations and were marked as unremarkable microscopically:
No tissue to list.

Appendix 10
Individual Animal Microscopic vs. Gross Observations

Fexinidazole Study Number: 0504-2007

ANIMAL: 2753 SEX: Male GROUP: 2 DOSE LEVEL: 50 mg/kg
DAY OF DEATH: 30 Test period STATUS: Scheduled phase sacrifice # 1 TERMINAL BODY WEIGHT (g) : 364.6

Tissue	Gross observations/comments	Correlated Microscopic Observations
GENER. CONDITION . . .	GOOD	No micropathology observations on tissue.
HARDERIAN GLANDS . . .	No gross observations on tissue	PORPHYRIN DEPOSITS, Minimal, Multifocal.
LIVER	No gross observations on tissue	CHRONIC INFLAMMATION, Slight, Multifocal.
		HEPATOCELLULAR HYPERTROPHY,CENTRILOBULAR, Minimal.

The following tissues have no gross observations and were not examined microscopically:

ADRENALS	AORTA	BONE MARROW	BRAIN	CECUM
COLON	DIAPHRAGM	DUODENUM	EPIDIDYMIDES	ESOPHAGUS
EYES	FEMUR	HEART	ILEUM	JEJUNUM
KIDNEYS	MANDIBULAR L.N.	MESENTERIC L.N.	LUNG	MAMMARY GLAND
SKELETAL MUSCLE	SCIATIC NERVE	OPTIC NERVES	PANCREAS	PITUITARY
PROSTATE	PARATHYROIDS	SPINAL CORD CERV	SPINAL CORD THOR	MANDIBULAR S.G.
PAROTIDS	STIFLE JOINT	SKIN	SPLEEN	STOMACH
STERNUM	SEMINAL VESICLES	TESTES	THYROIDS	THYMUS
TONGUE	TRACHEA	URINARY BLADDER		

The following tissues have no gross observations and were marked as unremarkable microscopically:
No tissue to list.

Appendix 10
Individual Animal Microscopic vs. Gross Observations

Fexinidazole　　Study Number: 0504-2007

ANIMAL: 2754　SEX: Male　GROUP: 2　DOSE LEVEL: 50 mg/kg
DAY OF DEATH: 30 Test period　STATUS: Scheduled phase sacrifice # 1　TERMINAL BODY WEIGHT (g) : 358.5

Tissue	Gross observations/comments	Correlated Microscopic Observations
GENER. CONDITION . . .	GOOD	No micropathology observations on tissue.
HARDERIAN GLANDS . . .	No gross observations on tissue	PORPHYRIN DEPOSITS, Minimal, Multifocal.
LIVER	No gross observations on tissue	CHRONIC INFLAMMATION, Slight, Multifocal.<br>HEPATOCELLULAR HYPERTROPHY,CENTRILOBULAR, Minimal.

The following tissues have no gross observations and were not examined microscopically:

ADRENALS	AORTA	BONE MARROW	BRAIN	CECUM
COLON	DIAPHRAGM	DUODENUM	EPIDIDYMIDES	ESOPHAGUS
EYES	FEMUR	HEART	ILEUM	JEJUNUM
KIDNEYS	MANDIBULAR L.N.	MESENTERIC L.N.	LUNG	MAMMARY GLAND
SKELETAL MUSCLE	SCIATIC NERVE	OPTIC NERVES	PANCREAS	PITUITARY
PROSTATE	PARATHYROIDS	SPINAL CORD CERV	SPINAL CORD THOR	MANDIBULAR S.G.
PAROTIDS	STIFLE JOINT	SKIN	SPLEEN	STOMACH
STERNUM	SEMINAL VESICLES	TESTES	THYROIDS	THYMUS
TONGUE	TRACHEA	URINARY BLADDER		

The following tissues have no gross observations and were marked as unremarkable microscopically:
No tissue to list.

Appendix 10
Individual Animal Microscopic vs. Gross Observations

Fexinidazole — Study Number: 0504-2007

ANIMAL: 2755 | SEX: Male | GROUP: 2 | DOSE LEVEL: 50 mg/kg
DAY OF DEATH: 29 Test period | STATUS: Scheduled phase sacrifice # 1 | TERMINAL BODY WEIGHT (g) : 438.5

Tissue	Gross observations/comments	Correlated Microscopic Observations
GENER. CONDITION . . .	GOOD	No micropathology observations on tissue.
HARDERIAN GLANDS . . .	No gross observations on tissue	PORPHYRIN DEPOSITS, Slight, Multifocal.
LIVER	No gross observations on tissue	CHRONIC INFLAMMATION, Minimal, Multifocal.<br>HEPATOCELLULAR HYPERTROPHY,CENTRILOBULAR, Minimal.<br>HEPATOCELLULAR VACUOLATION,PERIPORTAL, Minimal.

The following tissues have no gross observations and were not examined microscopically:

ADRENALS	AORTA	BONE MARROW	BRAIN	CECUM
COLON	DIAPHRAGM	DUODENUM	EPIDIDYMIDES	ESOPHAGUS
EYES	FEMUR	HEART	ILEUM	JEJUNUM
KIDNEYS	MANDIBULAR L.N.	MESENTERIC L.N.	LUNG	MAMMARY GLAND
SKELETAL MUSCLE	SCIATIC NERVE	OPTIC NERVES	PANCREAS	PITUITARY
PROSTATE	PARATHYROIDS	SPINAL CORD CERV	SPINAL CORD THOR	MANDIBULAR S.G.
PAROTIDS	STIFLE JOINT	SKIN	SPLEEN	STOMACH
STERNUM	SEMINAL VESICLES	TESTES	THYROIDS	THYMUS
TONGUE	TRACHEA	URINARY BLADDER		

The following tissues have no gross observations and were marked as unremarkable microscopically:
No tissue to list.

Appendix 10
Individual Animal Microscopic vs. Gross Observations

Fexinidazole — Study Number: 0504-2007

ANIMAL: 2756 | SEX: Male | GROUP: 2 | DOSE LEVEL: 50 mg/kg
DAY OF DEATH: 29 Test period | STATUS: Scheduled phase sacrifice # 1 | TERMINAL BODY WEIGHT (g) : 343.4

Tissue	Gross observations/comments	Correlated Microscopic Observations
GENER. CONDITION . . .	GOOD	No micropathology observations on tissue.
HARDERIAN GLANDS . . .	No gross observations on tissue	PORPHYRIN DEPOSITS, Moderate, Multifocal.<br>HEMORRHAGE,UNILATERAL, Slight, Multifocal.
LIVER	No gross observations on tissue	CHRONIC INFLAMMATION, Minimal, Multifocal.<br>HEPATOCELLULAR HYPERTROPHY,CENTRILOBULAR, Slight.<br>HEPATOCELLULAR VACUOLATION,DIFFUSE, Minimal.

The following tissues have no gross observations and were not examined microscopically:

ADRENALS	AORTA	BONE MARROW	BRAIN	CECUM
COLON	DIAPHRAGM	DUODENUM	EPIDIDYMIDES	ESOPHAGUS
EYES	FEMUR	HEART	ILEUM	JEJUNUM
KIDNEYS	MANDIBULAR L.N.	MESENTERIC L.N.	LUNG	MAMMARY GLAND
SKELETAL MUSCLE	SCIATIC NERVE	OPTIC NERVES	PANCREAS	PITUITARY
PROSTATE	PARATHYROIDS	SPINAL CORD CERV	SPINAL CORD THOR	MANDIBULAR S.G.
PAROTIDS	STIFLE JOINT	SKIN	SPLEEN	STOMACH
STERNUM	SEMINAL VESICLES	TESTES	THYROIDS	THYMUS
TONGUE	TRACHEA	URINARY BLADDER		

The following tissues have no gross observations and were marked as unremarkable microscopically:
No tissue to list.

Appendix 10
Individual Animal Microscopic vs. Gross Observations

Fexinidazole | Study Number: 0504-2007

ANIMAL: 2757 | SEX: Male | GROUP: 2 | DOSE LEVEL: 50 mg/kg
DAY OF DEATH: 29 Test period | STATUS: Scheduled phase sacrifice # 1 | TERMINAL BODY WEIGHT (g) : 340.1

Tissue	Gross observations/comments	Correlated Microscopic Observations
GENER. CONDITION . . .	GOOD	No micropathology observations on tissue.
HARDERIAN GLANDS . . .	No gross observations on tissue	PORPHYRIN DEPOSITS, Slight, Multifocal.
LIVER	No gross observations on tissue	CHRONIC INFLAMMATION, Minimal, Multifocal.<br>HEPATOCELLULAR HYPERTROPHY,CENTRILOBULAR, Minimal.

The following tissues have no gross observations and were not examined microscopically:

ADRENALS	AORTA	BONE MARROW	BRAIN	CECUM
COLON	DIAPHRAGM	DUODENUM	EPIDIDYMIDES	ESOPHAGUS
EYES	FEMUR	HEART	ILEUM	JEJUNUM
KIDNEYS	MANDIBULAR L.N.	MESENTERIC L.N.	LUNG	MAMMARY GLAND
SKELETAL MUSCLE	SCIATIC NERVE	OPTIC NERVES	PANCREAS	PITUITARY
PROSTATE	PARATHYROIDS	SPINAL CORD CERV	SPINAL CORD THOR	MANDIBULAR S.G.
PAROTIDS	STIFLE JOINT	SKIN	SPLEEN	STOMACH
STERNUM	SEMINAL VESICLES	TESTES	THYROIDS	THYMUS
TONGUE	TRACHEA	URINARY BLADDER		

The following tissues have no gross observations and were marked as unremarkable microscopically:
No tissue to list.

Appendix 10
Individual Animal Microscopic vs. Gross Observations

Fexinidazole | Study Number: 0504-2007

ANIMAL: 2758 | SEX: Male | GROUP: 2 | DOSE LEVEL: 50 mg/kg
DAY OF DEATH: 29 Test period | STATUS: Scheduled phase sacrifice # 1 | TERMINAL BODY WEIGHT (g) : 336.5

Tissue	Gross observations/comments	Correlated Microscopic Observations
GENER. CONDITION . . .	GOOD	No micropathology observations on tissue.
HARDERIAN GLANDS . . .	No gross observations on tissue	PORPHYRIN DEPOSITS, Slight, Multifocal.<br>ADENITIS,UNILATERAL, Slight, Multifocal, Acute.
LIVER	No gross observations on tissue	CHRONIC INFLAMMATION, Minimal, Multifocal.<br>HEPATOCELLULAR HYPERTROPHY,CENTRILOBULAR, Slight.<br>HEPATOCELLULAR VACUOLATION,DIFFUSE, Minimal.

The following tissues have no gross observations and were not examined microscopically:

ADRENALS	AORTA	BONE MARROW	BRAIN	CECUM
COLON	DIAPHRAGM	DUODENUM	EPIDIDYMIDES	ESOPHAGUS
EYES	FEMUR	HEART	ILEUM	JEJUNUM
KIDNEYS	MANDIBULAR L.N.	MESENTERIC L.N.	LUNG	MAMMARY GLAND
SKELETAL MUSCLE	SCIATIC NERVE	OPTIC NERVES	PANCREAS	PITUITARY
PROSTATE	PARATHYROIDS	SPINAL CORD CERV	SPINAL CORD THOR	MANDIBULAR S.G.
PAROTIDS	STIFLE JOINT	SKIN	SPLEEN	STOMACH
STERNUM	SEMINAL VESICLES	TESTES	THYROIDS	THYMUS
TONGUE	TRACHEA	URINARY BLADDER		

The following tissues have no gross observations and were marked as unremarkable microscopically:
No tissue to list.

Appendix 10
Individual Animal Microscopic vs. Gross Observations

Fexinidazole | Study Number: 0504-2007

ANIMAL: 2759 | SEX: Male | GROUP: 2 | DOSE LEVEL: 50 mg/kg
DAY OF DEATH: 29 Test period | STATUS: Scheduled phase sacrifice # 1 | TERMINAL BODY WEIGHT (g) : 353.6

Tissue	Gross observations/comments	Correlated Microscopic Observations
GENER. CONDITION . . .	GOOD	No micropathology observations on tissue.
HARDERIAN GLANDS . . .	No gross observations on tissue	PORPHYRIN DEPOSITS, Slight, Multifocal.<br>ADENITIS,UNILATERAL, Minimal, Multifocal, Acute.
LIVER	No gross observations on tissue	CHRONIC INFLAMMATION, Minimal, Multifocal.<br>HEPATOCELLULAR HYPERTROPHY,CENTRILOBULAR, Minimal.<br>HEPATOCELLULAR VACUOLATION,PERIPORTAL, Minimal.

The following tissues have no gross observations and were not examined microscopically:

ADRENALS	AORTA	BONE MARROW	BRAIN	CECUM
COLON	DIAPHRAGM	DUODENUM	EPIDIDYMIDES	ESOPHAGUS
EYES	FEMUR	HEART	ILEUM	JEJUNUM
KIDNEYS	MANDIBULAR L.N.	MESENTERIC L.N.	LUNG	MAMMARY GLAND
SKELETAL MUSCLE	SCIATIC NERVE	OPTIC NERVES	PANCREAS	PITUITARY
PROSTATE	PARATHYROIDS	SPINAL CORD CERV	SPINAL CORD THOR	MANDIBULAR S.G.
PAROTIDS	STIFLE JOINT	SKIN	SPLEEN	STOMACH
STERNUM	SEMINAL VESICLES	TESTES	THYROIDS	THYMUS
TONGUE	TRACHEA	URINARY BLADDER		

The following tissues have no gross observations and were marked as unremarkable microscopically:
No tissue to list.

Appendix 10
Individual Animal Microscopic vs. Gross Observations

Fexinidazole   Study Number: 0504-2007

ANIMAL: 2760   SEX: Male   GROUP: 3   DOSE LEVEL: 200 mg/kg
DAY OF DEATH: 30 Test period   STATUS: Scheduled phase sacrifice # 1   TERMINAL BODY WEIGHT (g) : 359.3

Tissue	Gross observations/comments	Correlated Microscopic Observations
GENER. CONDITION . . .	GOOD	No micropathology observations on tissue.
HARDERIAN GLANDS . . .	No gross observations on tissue	PORPHYRIN DEPOSITS, Minimal, Multifocal.
LIVER	No gross observations on tissue	CHRONIC INFLAMMATION, Slight, Multifocal.<br>HEPATOCELLULAR HYPERTROPHY,CENTRILOBULAR, Minimal.

The following tissues have no gross observations and were not examined microscopically:

ADRENALS	AORTA	BONE MARROW	BRAIN	CECUM
COLON	DIAPHRAGM	DUODENUM	EPIDIDYMIDES	ESOPHAGUS
EYES	FEMUR	HEART	ILEUM	JEJUNUM
KIDNEYS	MANDIBULAR L.N.	MESENTERIC L.N.	LUNG	MAMMARY GLAND
SKELETAL MUSCLE	SCIATIC NERVE	OPTIC NERVES	PANCREAS	PITUITARY
PROSTATE	PARATHYROIDS	SPINAL CORD CERV	SPINAL CORD THOR	MANDIBULAR S.G.
PAROTIDS	STIFLE JOINT	SKIN	SPLEEN	STOMACH
STERNUM	SEMINAL VESICLES	TESTES	THYROIDS	THYMUS
TONGUE	TRACHEA	URINARY BLADDER		

The following tissues have no gross observations and were marked as unremarkable microscopically:
No tissue to list.

Appendix 10
Individual Animal Microscopic vs. Gross Observations

Fexinidazole | Study Number: 0504-2007

ANIMAL: 2761 | SEX: Male | GROUP: 3 | DOSE LEVEL: 200 mg/kg
DAY OF DEATH: 30 Test period | STATUS: Scheduled phase sacrifice # 1 | TERMINAL BODY WEIGHT (g) : 385.0

Tissue	Gross observations/comments	Correlated Microscopic Observations
GENER. CONDITION . . .	GOOD	No micropathology observations on tissue.
HARDERIAN GLANDS . . .	No gross observations on tissue	PORPHYRIN DEPOSITS, Slight, Multifocal.
LIVER	No gross observations on tissue	CHRONIC INFLAMMATION, Minimal, Multifocal.<br>HEPATOCELLULAR HYPERTROPHY,CENTRILOBULAR, Minimal.

The following tissues have no gross observations and were not examined microscopically:

ADRENALS	AORTA	BONE MARROW	BRAIN	CECUM
COLON	DIAPHRAGM	DUODENUM	EPIDIDYMIDES	ESOPHAGUS
EYES	FEMUR	HEART	ILEUM	JEJUNUM
KIDNEYS	MANDIBULAR L.N.	MESENTERIC L.N.	LUNG	MAMMARY GLAND
SKELETAL MUSCLE	SCIATIC NERVE	OPTIC NERVES	PANCREAS	PITUITARY
PROSTATE	PARATHYROIDS	SPINAL CORD CERV	SPINAL CORD THOR	MANDIBULAR S.G.
PAROTIDS	STIFLE JOINT	SKIN	SPLEEN	STOMACH
STERNUM	SEMINAL VESICLES	TESTES	THYROIDS	THYMUS
TONGUE	TRACHEA	URINARY BLADDER		

The following tissues have no gross observations and were marked as unremarkable microscopically:
No tissue to list.

Appendix 10
Individual Animal Microscopic vs. Gross Observations

Fexinidazole Study Number: 0504-2007

ANIMAL: 2762 SEX: Male GROUP: 3 DOSE LEVEL: 200 mg/kg
DAY OF DEATH: 30 Test period STATUS: Scheduled phase sacrifice # 1 TERMINAL BODY WEIGHT (g) : 393.6

Tissue	Gross observations/comments	Correlated Microscopic Observations
GENER. CONDITION . . .	GOOD	No micropathology observations on tissue.
HARDERIAN GLANDS . . .	No gross observations on tissue	PORPHYRIN DEPOSITS, Slight, Multifocal.
LIVER	No gross observations on tissue	CHRONIC INFLAMMATION, Minimal, Multifocal.
		HEPATOCELLULAR HYPERTROPHY,CENTRILOBULAR, Minimal.
		HEPATOCELLULAR VACUOLATION,PERIPORTAL, Minimal.

The following tissues have no gross observations and were not examined microscopically:

ADRENALS	AORTA	BONE MARROW	BRAIN	CECUM
COLON	DIAPHRAGM	DUODENUM	EPIDIDYMIDES	ESOPHAGUS
EYES	FEMUR	HEART	ILEUM	JEJUNUM
KIDNEYS	MANDIBULAR L.N.	MESENTERIC L.N.	LUNG	MAMMARY GLAND
SKELETAL MUSCLE	SCIATIC NERVE	OPTIC NERVES	PANCREAS	PITUITARY
PROSTATE	PARATHYROIDS	SPINAL CORD CERV	SPINAL CORD THOR	MANDIBULAR S.G.
PAROTIDS	STIFLE JOINT	SKIN	SPLEEN	STOMACH
STERNUM	SEMINAL VESICLES	TESTES	THYROIDS	THYMUS
TONGUE	TRACHEA	URINARY BLADDER		

The following tissues have no gross observations and were marked as unremarkable microscopically:
No tissue to list.

Appendix 10
Individual Animal Microscopic vs. Gross Observations

Fexinidazole Study Number: 0504-2007

ANIMAL: 2763 SEX: Male GROUP: 3 DOSE LEVEL: 200 mg/kg
DAY OF DEATH: 30 Test period STATUS: Scheduled phase sacrifice # 1 TERMINAL BODY WEIGHT (g) : 330.5

Tissue	Gross observations/comments	Correlated Microscopic Observations
GENER. CONDITION . . .	GOOD	No micropathology observations on tissue.
HARDERIAN GLANDS . . .	No gross observations on tissue	PORPHYRIN DEPOSITS, Moderate, Multifocal.
LIVER	No gross observations on tissue	CHRONIC INFLAMMATION, Minimal, Multifocal.<br>HEPATOCELLULAR HYPERTROPHY,CENTRILOBULAR, Slight.
SKIN	ENCRUSTED AREA(S), single/ in the head region, 10 mm in diameter.	Examined 1 correlation found:<br>SCAB FORMATION, Slight, Multifocal.<br>ACANTHOSIS, Slight, Multifocal.<br>EPIDERMAL/DERMAL INFLAMMATION, Slight, Multifocal. / acute to chronic.

The following tissues have no gross observations and were not examined microscopically:

ADRENALS	AORTA	BONE MARROW	BRAIN	CECUM
COLON	DIAPHRAGM	DUODENUM	EPIDIDYMIDES	ESOPHAGUS
EYES	FEMUR	HEART	ILEUM	JEJUNUM
KIDNEYS	MANDIBULAR L.N.	MESENTERIC L.N.	LUNG	MAMMARY GLAND
SKELETAL MUSCLE	SCIATIC NERVE	OPTIC NERVES	PANCREAS	PITUITARY
PROSTATE	PARATHYROIDS	SPINAL CORD CERV	SPINAL CORD THOR	MANDIBULAR S.G.
PAROTIDS	STIFLE JOINT	SPLEEN	STOMACH	STERNUM
SEMINAL VESICLES	TESTES	THYROIDS	THYMUS	TONGUE
TRACHEA	URINARY BLADDER			

The following tissues have no gross observations and were marked as unremarkable microscopically:
No tissue to list.

Appendix 10
Individual Animal Microscopic vs. Gross Observations

Fexinidazole — Study Number: 0504-2007

ANIMAL: 2764 — SEX: Male — GROUP: 3 — DOSE LEVEL: 200 mg/kg
DAY OF DEATH: 30 Test period — STATUS: Scheduled phase sacrifice # 1 — TERMINAL BODY WEIGHT (g) : 396.5

Tissue	Gross observations/comments	Correlated Microscopic Observations
GENER. CONDITION . . .	GOOD	No micropathology observations on tissue.
HARDERIAN GLANDS . . .	No gross observations on tissue	PORPHYRIN DEPOSITS, Slight, Multifocal.
LIVER	No gross observations on tissue	CHRONIC INFLAMMATION, Minimal, Multifocal.<br>HEPATOCELLULAR HYPERTROPHY,CENTRILOBULAR, Slight.<br>HEPATOCELLULAR VACUOLATION,PERIPORTAL, Minimal.
SKIN	ENCRUSTED AREA(S), single/ in the head region, 10 mm in diameter.	Examined 1 correlation found: SCAB FORMATION, Moderate, Focal.<br>ACANTHOSIS, Slight, Multifocal.<br>EPIDERMAL/DERMAL INFLAMMATION, Slight, Multifocal. / acute to chronic.

The following tissues have no gross observations and were not examined microscopically:

ADRENALS	AORTA	BONE MARROW	BRAIN	CECUM
COLON	DIAPHRAGM	DUODENUM	EPIDIDYMIDES	ESOPHAGUS
EYES	FEMUR	HEART	ILEUM	JEJUNUM
KIDNEYS	MANDIBULAR L.N.	MESENTERIC L.N.	LUNG	MAMMARY GLAND
SKELETAL MUSCLE	SCIATIC NERVE	OPTIC NERVES	PANCREAS	PITUITARY
PROSTATE	PARATHYROIDS	SPINAL CORD CERV	SPINAL CORD THOR	MANDIBULAR S.G.
PAROTIDS	STIFLE JOINT	SPLEEN	STOMACH	STERNUM
SEMINAL VESICLES	TESTES	THYROIDS	THYMUS	TONGUE
TRACHEA	URINARY BLADDER			

Appendix 10
Individual Animal Microscopic vs. Gross Observations

Fexinidazole Study Number: 0504-2007

ANIMAL: 2764 SEX: Male GROUP: 3 DOSE LEVEL: 200 mg/kg
DAY OF DEATH: 30 Test period STATUS: Scheduled phase sacrifice # 1 TERMINAL BODY WEIGHT (g) : 396.5

Tissue	Gross observations/comments	Correlated Microscopic Observations

The following tissues have no gross observations and were marked as unremarkable microscopically:
No tissue to list.

Appendix 10
Individual Animal Microscopic vs. Gross Observations

Fexinidazole Study Number: 0504-2007

ANIMAL: 2765 SEX: Male GROUP: 3 DOSE LEVEL: 200 mg/kg
DAY OF DEATH: 29 Test period STATUS: Scheduled phase sacrifice # 1 TERMINAL BODY WEIGHT (g) : 362.8

Tissue	Gross observations/comments	Correlated Microscopic Observations
GENER. CONDITION . . .	GOOD	No micropathology observations on tissue.
HARDERIAN GLANDS . . .	No gross observations on tissue	PORPHYRIN DEPOSITS, Slight, Multifocal.
LIVER	No gross observations on tissue	CHRONIC INFLAMMATION, Minimal, Multifocal.<br>HEPATOCELLULAR HYPERTROPHY,CENTRILOBULAR, Minimal.

The following tissues have no gross observations and were not examined microscopically:

ADRENALS	AORTA	BONE MARROW	BRAIN	CECUM
COLON	DIAPHRAGM	DUODENUM	EPIDIDYMIDES	ESOPHAGUS
EYES	FEMUR	HEART	ILEUM	JEJUNUM
KIDNEYS	MANDIBULAR L.N.	MESENTERIC L.N.	LUNG	MAMMARY GLAND
SKELETAL MUSCLE	SCIATIC NERVE	OPTIC NERVES	PANCREAS	PITUITARY
PROSTATE	PARATHYROIDS	SPINAL CORD CERV	SPINAL CORD THOR	MANDIBULAR S.G.
PAROTIDS	STIFLE JOINT	SKIN	SPLEEN	STOMACH
STERNUM	SEMINAL VESICLES	TESTES	THYROIDS	THYMUS
TONGUE	TRACHEA	URINARY BLADDER		

The following tissues have no gross observations and were marked as unremarkable microscopically:
No tissue to list.

Appendix 10
Individual Animal Microscopic vs. Gross Observations

Fexinidazole | Study Number: 0504-2007

ANIMAL: 2766 | SEX: Male | GROUP: 3 | DOSE LEVEL: 200 mg/kg
DAY OF DEATH: 29 Test period | STATUS: Scheduled phase sacrifice # 1 | TERMINAL BODY WEIGHT (g) : 340.6

Tissue	Gross observations/comments	Correlated Microscopic Observations
GENER. CONDITION . . .	GOOD	No micropathology observations on tissue.
HARDERIAN GLANDS . . .	No gross observations on tissue	PORPHYRIN DEPOSITS, Moderate, Multifocal.<br>HEMORRHAGE,UNILATERAL, Slight, Multifocal.
LIVER	No gross observations on tissue	CHRONIC INFLAMMATION, Minimal, Multifocal.<br>HEPATOCELLULAR HYPERTROPHY,CENTRILOBULAR, Slight.

The following tissues have no gross observations and were not examined microscopically:

ADRENALS	AORTA	BONE MARROW	BRAIN	CECUM
COLON	DIAPHRAGM	DUODENUM	EPIDIDYMIDES	ESOPHAGUS
EYES	FEMUR	HEART	ILEUM	JEJUNUM
KIDNEYS	MANDIBULAR L.N.	MESENTERIC L.N.	LUNG	MAMMARY GLAND
SKELETAL MUSCLE	SCIATIC NERVE	OPTIC NERVES	PANCREAS	PITUITARY
PROSTATE	PARATHYROIDS	SPINAL CORD CERV	SPINAL CORD THOR	MANDIBULAR S.G.
PAROTIDS	STIFLE JOINT	SKIN	SPLEEN	STOMACH
STERNUM	SEMINAL VESICLES	TESTES	THYROIDS	THYMUS
TONGUE	TRACHEA	URINARY BLADDER		

The following tissues have no gross observations and were marked as unremarkable microscopically:
No tissue to list.

Appendix 10
Individual Animal Microscopic vs. Gross Observations

Fexinidazole Study Number: 0504-2007

ANIMAL: 2767 SEX: Male GROUP: 3 DOSE LEVEL: 200 mg/kg
DAY OF DEATH: 29 Test period STATUS: Scheduled phase sacrifice # 1 TERMINAL BODY WEIGHT (g) : 276.7

Tissue	Gross observations/comments	Correlated Microscopic Observations
GENER. CONDITION . . .	GOOD	No micropathology observations on tissue.
HARDERIAN GLANDS . . .	No gross observations on tissue	PORPHYRIN DEPOSITS, Slight, Multifocal.<br>HEMORRHAGE,UNILATERAL, Slight, Multifocal.<br>ADENITIS,UNILATERAL, Slight, Multifocal, Acute.
LIVER	No gross observations on tissue	CHRONIC INFLAMMATION, Slight, Multifocal.<br>HEPATOCELLULAR HYPERTROPHY,CENTRILOBULAR, Minimal.

The following tissues have no gross observations and were not examined microscopically:

ADRENALS	AORTA	BONE MARROW	BRAIN	CECUM
COLON	DIAPHRAGM	DUODENUM	EPIDIDYMIDES	ESOPHAGUS
EYES	FEMUR	HEART	ILEUM	JEJUNUM
KIDNEYS	MANDIBULAR L.N.	MESENTERIC L.N.	LUNG	MAMMARY GLAND
SKELETAL MUSCLE	SCIATIC NERVE	OPTIC NERVES	PANCREAS	PITUITARY
PROSTATE	PARATHYROIDS	SPINAL CORD CERV	SPINAL CORD THOR	MANDIBULAR S.G.
PAROTIDS	STIFLE JOINT	SKIN	SPLEEN	STOMACH
STERNUM	SEMINAL VESICLES	TESTES	THYROIDS	THYMUS
TONGUE	TRACHEA	URINARY BLADDER		

The following tissues have no gross observations and were marked as unremarkable microscopically:
No tissue to list.

Appendix 10
Individual Animal Microscopic vs. Gross Observations

Fexinidazole — Study Number: 0504-2007

ANIMAL: 2768 — SEX: Male — GROUP: 3 — DOSE LEVEL: 200 mg/kg
DAY OF DEATH: 29 Test period — STATUS: Scheduled phase sacrifice # 1 — TERMINAL BODY WEIGHT (g) : 401.5

Tissue	Gross observations/comments	Correlated Microscopic Observations
GENER. CONDITION . . .	GOOD	No micropathology observations on tissue.
HARDERIAN GLANDS . . .	No gross observations on tissue	PORPHYRIN DEPOSITS, Slight, Multifocal.
LIVER	No gross observations on tissue	CHRONIC INFLAMMATION, Minimal, Multifocal.<br>HEPATOCELLULAR HYPERTROPHY,CENTRILOBULAR, Minimal.<br>HEPATOCELLULAR VACUOLATION,PERIPORTAL, Minimal.

The following tissues have no gross observations and were not examined microscopically:

ADRENALS	AORTA	BONE MARROW	BRAIN	CECUM
COLON	DIAPHRAGM	DUODENUM	EPIDIDYMIDES	ESOPHAGUS
EYES	FEMUR	HEART	ILEUM	JEJUNUM
KIDNEYS	MANDIBULAR L.N.	MESENTERIC L.N.	LUNG	MAMMARY GLAND
SKELETAL MUSCLE	SCIATIC NERVE	OPTIC NERVES	PANCREAS	PITUITARY
PROSTATE	PARATHYROIDS	SPINAL CORD CERV	SPINAL CORD THOR	MANDIBULAR S.G.
PAROTIDS	STIFLE JOINT	SKIN	SPLEEN	STOMACH
STERNUM	SEMINAL VESICLES	TESTES	THYROIDS	THYMUS
TONGUE	TRACHEA	URINARY BLADDER		

The following tissues have no gross observations and were marked as unremarkable microscopically:
No tissue to list.

Appendix 10
Individual Animal Microscopic vs. Gross Observations

Fexinidazole | Study Number: 0504-2007

ANIMAL: 2769 | SEX: Male | GROUP: 3 | DOSE LEVEL: 200 mg/kg
DAY OF DEATH: 29 Test period | STATUS: Scheduled phase sacrifice # 1 | TERMINAL BODY WEIGHT (g) : 317.1

Tissue	Gross observations/comments	Correlated Microscopic Observations
GENER. CONDITION . . .	GOOD	No micropathology observations on tissue.
HARDERIAN GLANDS . . .	No gross observations on tissue	PORPHYRIN DEPOSITS, Moderate, Multifocal.<br>ADENITIS,UNILATERAL, Slight, Multifocal, Acute.<br>LYMPHOCYTIC INFILTRATION, Minimal, Multifocal, Unilateral..
LIVER	No gross observations on tissue	CHRONIC INFLAMMATION, Minimal, Multifocal.<br>HEPATOCELLULAR HYPERTROPHY,CENTRILOBULAR, Minimal.<br>HEPATOCELLULAR VACUOLATION,PERIPORTAL, Minimal.

The following tissues have no gross observations and were not examined microscopically:

ADRENALS	AORTA	BONE MARROW	BRAIN	CECUM
COLON	DIAPHRAGM	DUODENUM	EPIDIDYMIDES	ESOPHAGUS
EYES	FEMUR	HEART	ILEUM	JEJUNUM
KIDNEYS	MANDIBULAR L.N.	MESENTERIC L.N.	LUNG	MAMMARY GLAND
SKELETAL MUSCLE	SCIATIC NERVE	OPTIC NERVES	PANCREAS	PITUITARY
PROSTATE	PARATHYROIDS	SPINAL CORD CERV	SPINAL CORD THOR	MANDIBULAR S.G.
PAROTIDS	STIFLE JOINT	SKIN	SPLEEN	STOMACH
STERNUM	SEMINAL VESICLES	TESTES	THYROIDS	THYMUS
TONGUE	TRACHEA	URINARY BLADDER		

The following tissues have no gross observations and were marked as unremarkable microscopically:
No tissue to list.

Appendix 10
Individual Animal Microscopic vs. Gross Observations

Fexinidazole Study Number: 0504-2007

ANIMAL: 2770 SEX: Male GROUP: 4 DOSE LEVEL: 800 mg/kg
DAY OF DEATH: 30 Test period STATUS: Scheduled phase sacrifice # 1 TERMINAL BODY WEIGHT (g) : 353.3

Tissue	Gross observations/comments	Correlated Microscopic Observations
GENER. CONDITION . . .	GOOD	No micropathology observations on tissue.
HARDERIAN GLANDS . . .	No gross observations on tissue	PORPHYRIN DEPOSITS, Slight, Multifocal.
LIVER	No gross observations on tissue	CHRONIC INFLAMMATION, Minimal, Multifocal.<br>HEPATOCELLULAR HYPERTROPHY,CENTRILOBULAR, Slight.<br>HEPATOCELLULAR VACUOLATION,PERIPORTAL, Minimal.<br>FOCAL NECROSIS,SUBCAPSULAR, Minimal. / with associated acute inflammation.
MANDIBULAR L.N.	No gross observations on tissue	PLASMACYTOSIS, Moderate, Diffuse, Unilateral..
PROSTATE	No gross observations on tissue	LYMPHOCYTIC INFILTRATION, Minimal, Multifocal.
PARATHYROIDS	No gross observations on tissue	ONLY ONE PARATHYROID AVAILABLE FOR EXAMINATION, Present.

The following tissues have no gross observations and were not examined microscopically:
No tissue to list.

Appendix 10
Individual Animal Microscopic vs. Gross Observations

Fexinidazole Study Number: 0504-2007

ANIMAL: 2770 SEX: Male GROUP: 4 DOSE LEVEL: 800 mg/kg
DAY OF DEATH: 30 Test period STATUS: Scheduled phase sacrifice # 1 TERMINAL BODY WEIGHT (g) : 353.3

Tissue	Gross observations/comments	Correlated Microscopic Observations

The following tissues have no gross observations and were marked as unremarkable microscopically:

ADRENALS	AORTA	BONE MARROW	BRAIN	CECUM
COLON	DIAPHRAGM	DUODENUM	EPIDIDYMIDES	ESOPHAGUS
EYES	FEMUR	HEART	ILEUM	JEJUNUM
KIDNEYS	MESENTERIC L.N.	LUNG	MAMMARY GLAND	SKELETAL MUSCLE
SCIATIC NERVE	OPTIC NERVES	PANCREAS	PITUITARY	SPINAL CORD CERV
SPINAL CORD THOR	MANDIBULAR S.G.	PAROTIDS	STIFLE JOINT	SKIN
SPLEEN	STOMACH	STERNUM	SEMINAL VESICLES	TESTES
THYROIDS	THYMUS	TONGUE	TRACHEA	URINARY BLADDER

Appendix 10
Individual Animal Microscopic vs. Gross Observations

Fexinidazole Study Number: 0504-2007

ANIMAL: 2771 SEX: Male GROUP: 4 DOSE LEVEL: 800 mg/kg
DAY OF DEATH: 30 Test period STATUS: Scheduled phase sacrifice # 1 TERMINAL BODY WEIGHT (g) : 344.7

Tissue	Gross observations/comments	Correlated Microscopic Observations
GENER. CONDITION . . .	GOOD	No micropathology observations on tissue.
KIDNEYS	No gross observations on tissue	CORTICAL TUBULAR REGENERATIVE BASOPHILIA, Minimal, Multifocal.
HARDERIAN GLANDS . . .	No gross observations on tissue	PORPHYRIN DEPOSITS, Slight, Multifocal.<br>LYMPHOCYTIC INFILTRATION, Minimal, Multifocal.
LIVER	No gross observations on tissue	CHRONIC INFLAMMATION, Minimal, Multifocal.<br>HEPATOCELLULAR HYPERTROPHY,CENTRILOBULAR, Slight.<br>HEPATOCELLULAR VACUOLATION,PERIPORTAL, Minimal.
MANDIBULAR L.N.	No gross observations on tissue	PLASMACYTOSIS, Slight, Diffuse.
PARATHYROIDS	No gross observations on tissue	ONLY ONE PARATHYROID AVAILABLE FOR EXAMINATION, Present.

The following tissues have no gross observations and were not examined microscopically:
No tissue to list.

The following tissues have no gross observations and were marked as unremarkable microscopically:
ADRENALS AORTA BONE MARROW BRAIN CECUM

Appendix 10
Individual Animal Microscopic vs. Gross Observations

Fexinidazole Study Number: 0504-2007

ANIMAL: 2771 SEX: Male GROUP: 4 DOSE LEVEL: 800 mg/kg
DAY OF DEATH: 30 Test period STATUS: Scheduled phase sacrifice # 1 TERMINAL BODY WEIGHT (g) : 344.7

Tissue	Gross observations/comments	Correlated Microscopic Observations

The following tissues have no gross observations and were marked as unremarkable microscopically:

COLON	DIAPHRAGM	DUODENUM	EPIDIDYMIDES	ESOPHAGUS
EYES	FEMUR	HEART	ILEUM	JEJUNUM
MESENTERIC L.N.	LUNG	MAMMARY GLAND	SKELETAL MUSCLE	SCIATIC NERVE
OPTIC NERVES	PANCREAS	PITUITARY	PROSTATE	SPINAL CORD CERV
SPINAL CORD THOR	MANDIBULAR S.G.	PAROTIDS	STIFLE JOINT	SKIN
SPLEEN	STOMACH	STERNUM	SEMINAL VESICLES	TESTES
THYROIDS	THYMUS	TONGUE	TRACHEA	URINARY BLADDER

Appendix 10
Individual Animal Microscopic vs. Gross Observations

Fexinidazole | Study Number: 0504-2007

ANIMAL: 2772 | SEX: Male | GROUP: 4 | DOSE LEVEL: 800 mg/kg
DAY OF DEATH: 30 Test period | STATUS: Scheduled phase sacrifice # 1 | TERMINAL BODY WEIGHT (g) : 411.0

Tissue	Gross observations/comments	Correlated Microscopic Observations
ADRENALS	No gross observations on tissue	CORTICAL VACUOLATION, Slight, Diffuse.
GENER. CONDITION . . .	GOOD	No micropathology observations on tissue.
KIDNEYS	No gross observations on tissue	PELVIC DILATATION, Slight.
HARDERIAN GLANDS . . .	No gross observations on tissue	PORPHYRIN DEPOSITS, Minimal, Multifocal.
LIVER	No gross observations on tissue	CHRONIC INFLAMMATION, Minimal, Multifocal.
		HEPATOCELLULAR HYPERTROPHY,CENTRILOBULAR, Slight.
		HEPATOCELLULAR VACUOLATION,PERIPORTAL, Minimal.
MANDIBULAR L.N.	No gross observations on tissue	PLASMACYTOSIS, Moderate, Diffuse, Unilateral..
LUNG	No gross observations on tissue	ALVEOLAR MACROPHAGE INFILTRATION, Minimal, Multifocal.
PARATHYROIDS	No gross observations on tissue	ONLY ONE PARATHYROID AVAILABLE FOR EXAMINATION, Present.
SPLEEN	No gross observations on tissue	INFLAMMATION OF THE CAPSULE, Minimal, Multifocal, Chronic.

Appendix 10
Individual Animal Microscopic vs. Gross Observations

Fexinidazole Study Number: 0504-2007

ANIMAL: 2772 SEX: Male GROUP: 4 DOSE LEVEL: 800 mg/kg
DAY OF DEATH: 30 Test period STATUS: Scheduled phase sacrifice # 1 TERMINAL BODY WEIGHT (g) : 411.0

Tissue	Gross observations/comments	Correlated Microscopic Observations

The following tissues have no gross observations and were not examined microscopically:
No tissue to list.

The following tissues have no gross observations and were marked as unremarkable microscopically:

AORTA	BONE MARROW	BRAIN	CECUM	COLON
DIAPHRAGM	DUODENUM	EPIDIDYMIDES	ESOPHAGUS	EYES
FEMUR	HEART	ILEUM	JEJUNUM	MESENTERIC L.N.
MAMMARY GLAND	SKELETAL MUSCLE	SCIATIC NERVE	OPTIC NERVES	PANCREAS
PITUITARY	PROSTATE	SPINAL CORD CERV	SPINAL CORD THOR	MANDIBULAR S.G.
PAROTIDS	STIFLE JOINT	SKIN	STOMACH	STERNUM
SEMINAL VESICLES	TESTES	THYROIDS	THYMUS	TONGUE
TRACHEA	URINARY BLADDER			

Appendix 10
Individual Animal Microscopic vs. Gross Observations

Fexinidazole | Study Number: 0504-2007

ANIMAL: 2773 | SEX: Male | GROUP: 4 | DOSE LEVEL: 800 mg/kg
DAY OF DEATH: 30 Test period | STATUS: Scheduled phase sacrifice # 1 | TERMINAL BODY WEIGHT (g) : 298.5

Tissue	Gross observations/comments	Correlated Microscopic Observations
GENER. CONDITION . . .	GOOD	No micropathology observations on tissue.
KIDNEYS	No gross observations on tissue	CORTICAL TUBULAR REGENERATIVE BASOPHILIA, Minimal, Multifocal, Unilateral left..
HARDERIAN GLANDS . . .	No gross observations on tissue	PORPHYRIN DEPOSITS, Slight, Multifocal.
LIVER	No gross observations on tissue	CHRONIC INFLAMMATION, Minimal, Multifocal.
		HEPATOCELLULAR HYPERTROPHY,CENTRILOBULAR, Slight.
MANDIBULAR L.N.	No gross observations on tissue	PLASMACYTOSIS, Moderate, Diffuse.
LUNG	No gross observations on tissue	ALVEOLAR MACROPHAGE INFILTRATION, Minimal, Multifocal.
OPTIC NERVES	No gross observations on tissue	ONLY ONE NERVE IS AVAILABLE FOR EXAMINATION, Present.
PARATHYROIDS	No gross observations on tissue	ONLY ONE PARATHYROID AVAILABLE FOR EXAMINATION, Present.

The following tissues have no gross observations and were not examined microscopically:
No tissue to list.

Appendix 10
Individual Animal Microscopic vs. Gross Observations

Fexinidazole Study Number: 0504-2007

ANIMAL: 2773 SEX: Male GROUP: 4 DOSE LEVEL: 800 mg/kg
DAY OF DEATH: 30 Test period STATUS: Scheduled phase sacrifice # 1 TERMINAL BODY WEIGHT (g) : 298.5

Tissue	Gross observations/comments	Correlated Microscopic Observations

The following tissues have no gross observations and were marked as unremarkable microscopically:

ADRENALS	AORTA	BONE MARROW	BRAIN	CECUM
COLON	DIAPHRAGM	DUODENUM	EPIDIDYMIDES	ESOPHAGUS
EYES	FEMUR	HEART	ILEUM	JEJUNUM
MESENTERIC L.N.	MAMMARY GLAND	SKELETAL MUSCLE	SCIATIC NERVE	PANCREAS
PITUITARY	PROSTATE	SPINAL CORD CERV	SPINAL CORD THOR	MANDIBULAR S.G.
PAROTIDS	STIFLE JOINT	SKIN	SPLEEN	STOMACH
STERNUM	SEMINAL VESICLES	TESTES	THYROIDS	THYMUS
TONGUE	TRACHEA	URINARY BLADDER		

Appendix 10
Individual Animal Microscopic vs. Gross Observations

Fexinidazole Study Number: 0504-2007

ANIMAL: 2774 SEX: Male GROUP: 4 DOSE LEVEL: 800 mg/kg
DAY OF DEATH: 30 Test period STATUS: Scheduled phase sacrifice # 1 TERMINAL BODY WEIGHT (g) : 293.6

Tissue	Gross observations/comments	Correlated Microscopic Observations
ADRENALS	No gross observations on tissue	CORTICAL VACUOLATION, Minimal, Multifocal.
GENER. CONDITION	GOOD	No micropathology observations on tissue.
HARDERIAN GLANDS	No gross observations on tissue	PORPHYRIN DEPOSITS, Slight, Multifocal.
LIVER	No gross observations on tissue	CHRONIC INFLAMMATION, Minimal, Multifocal.<br>HEPATOCELLULAR HYPERTROPHY,CENTRILOBULAR, Slight.
MANDIBULAR L.N.	No gross observations on tissue	PLASMACYTOSIS, Moderate, Diffuse.
PARATHYROIDS	No gross observations on tissue	ONLY ONE PARATHYROID AVAILABLE FOR EXAMINATION, Present.

The following tissues have no gross observations and were not examined microscopically:
No tissue to list.

The following tissues have no gross observations and were marked as unremarkable microscopically:

AORTA	BONE MARROW	BRAIN	CECUM	COLON
DIAPHRAGM	DUODENUM	EPIDIDYMIDES	ESOPHAGUS	EYES
FEMUR	HEART	ILEUM	JEJUNUM	KIDNEYS
MESENTERIC L.N.	LUNG	MAMMARY GLAND	SKELETAL MUSCLE	SCIATIC NERVE
OPTIC NERVES	PANCREAS	PITUITARY	PROSTATE	SPINAL CORD CERV
SPINAL CORD THOR	MANDIBULAR S.G.	PAROTIDS	STIFLE JOINT	SKIN
SPLEEN	STOMACH	STERNUM	SEMINAL VESICLES	TESTES
THYROIDS	THYMUS	TONGUE	TRACHEA	URINARY BLADDER

Appendix 10
Individual Animal Microscopic vs. Gross Observations

Fexinidazole | Study Number: 0504-2007

ANIMAL: 2775 | SEX: Male | GROUP: 4 | DOSE LEVEL: 800 mg/kg
DAY OF DEATH: 29 Test period | STATUS: Scheduled phase sacrifice # 1 | TERMINAL BODY WEIGHT (g) : 385.0

Tissue	Gross observations/comments	Correlated Microscopic Observations
GENER. CONDITION . . .	GOOD	No micropathology observations on tissue.
HEART	No gross observations on tissue	MYOCARDIAL INFLAMMATION, Minimal, Multifocal.
HARDERIAN GLANDS . . .	No gross observations on tissue	PORPHYRIN DEPOSITS, Minimal, Multifocal.
		LYMPHOCYTIC INFILTRATION, Minimal, Focal, Unilateral..
LIVER	No gross observations on tissue	CHRONIC INFLAMMATION, Slight, Multifocal.
		HEPATOCELLULAR HYPERTROPHY,CENTRILOBULAR, Slight.
		HEPATOCELLULAR VACUOLATION,PERIPORTAL, Minimal.
MANDIBULAR L.N.	No gross observations on tissue	PLASMACYTOSIS, Slight, Diffuse.
OPTIC NERVES	No gross observations on tissue	ONLY ONE NERVE IS AVAILABLE FOR EXAMINATION, Present.
SPLEEN	No gross observations on tissue	EXTRAMEDULLARY HEMOPOIESIS, Minimal, Multifocal.

The following tissues have no gross observations and were not examined microscopically:
No tissue to list.

Appendix 10
Individual Animal Microscopic vs. Gross Observations

Fexinidazole Study Number: 0504-2007

ANIMAL: 2775 SEX: Male GROUP: 4 DOSE LEVEL: 800 mg/kg
DAY OF DEATH: 29 Test period STATUS: Scheduled phase sacrifice # 1 TERMINAL BODY WEIGHT (g) : 385.0

Tissue	Gross observations/comments	Correlated Microscopic Observations

The following tissues have no gross observations and were marked as unremarkable microscopically:

ADRENALS	AORTA	BONE MARROW	BRAIN	CECUM
COLON	DIAPHRAGM	DUODENUM	EPIDIDYMIDES	ESOPHAGUS
EYES	FEMUR	ILEUM	JEJUNUM	KIDNEYS
MESENTERIC L.N.	LUNG	MAMMARY GLAND	SKELETAL MUSCLE	SCIATIC NERVE
PANCREAS	PITUITARY	PROSTATE	PARATHYROIDS	SPINAL CORD CERV
SPINAL CORD THOR	MANDIBULAR S.G.	PAROTIDS	STIFLE JOINT	SKIN
STOMACH	STERNUM	SEMINAL VESICLES	TESTES	THYROIDS
THYMUS	TONGUE	TRACHEA	URINARY BLADDER	

Appendix 10
Individual Animal Microscopic vs. Gross Observations

Fexinidazole — Study Number: 0504-2007

ANIMAL: 2776 | SEX: Male | GROUP: 4 | DOSE LEVEL: 800 mg/kg
DAY OF DEATH: 29 Test period | STATUS: Scheduled phase sacrifice # 1 | TERMINAL BODY WEIGHT (g) : 338.8

Tissue	Gross observations/comments	Correlated Microscopic Observations
GENER. CONDITION . . .	GOOD	No micropathology observations on tissue.
KIDNEYS	No gross observations on tissue	CHRONIC INFLAMMATION, Minimal, Multifocal.<br>CORTICAL TUBULAR REGENERATIVE BASOPHILIA, Minimal, Multifocal.
HARDERIAN GLANDS . . .	No gross observations on tissue	PORPHYRIN DEPOSITS, Slight, Multifocal.
LIVER	No gross observations on tissue	CHRONIC INFLAMMATION, Minimal, Multifocal.<br>HEPATOCELLULAR HYPERTROPHY,CENTRILOBULAR, Slight.
MANDIBULAR L.N.	No gross observations on tissue	PLASMACYTOSIS, Moderate, Diffuse.
LUNG	No gross observations on tissue	ALVEOLAR HEMORRHAGE, Slight, Focal.
MAMMARY GLAND	No gross observations on tissue	CERVICAL MAMMARY GLAND EXAMINED, Present.
PARATHYROIDS	No gross observations on tissue	ONLY ONE PARATHYROID AVAILABLE FOR EXAMINATION, Present.

The following tissues have no gross observations and were not examined microscopically:
No tissue to list.

Appendix 10
Individual Animal Microscopic vs. Gross Observations

Fexinidazole Study Number: 0504-2007

ANIMAL: 2776 SEX: Male GROUP: 4 DOSE LEVEL: 800 mg/kg
DAY OF DEATH: 29 Test period STATUS: Scheduled phase sacrifice # 1 TERMINAL BODY WEIGHT (g) : 338.8

Tissue	Gross observations/comments	Correlated Microscopic Observations

The following tissues have no gross observations and were marked as unremarkable microscopically:

ADRENALS	AORTA	BONE MARROW	BRAIN	CECUM
COLON	DIAPHRAGM	DUODENUM	EPIDIDYMIDES	ESOPHAGUS
EYES	FEMUR	HEART	ILEUM	JEJUNUM
MESENTERIC L.N.	SKELETAL MUSCLE	SCIATIC NERVE	OPTIC NERVES	PANCREAS
PITUITARY	PROSTATE	SPINAL CORD CERV	SPINAL CORD THOR	MANDIBULAR S.G.
PAROTIDS	STIFLE JOINT	SKIN	SPLEEN	STOMACH
STERNUM	SEMINAL VESICLES	TESTES	THYROIDS	THYMUS
TONGUE	TRACHEA	URINARY BLADDER		

Appendix 10
Individual Animal Microscopic vs. Gross Observations

Fexinidazole — Study Number: 0504-2007

ANIMAL: 2777 | SEX: Male | GROUP: 4 | DOSE LEVEL: 800 mg/kg
DAY OF DEATH: 29 Test period | STATUS: Scheduled phase sacrifice # 1 | TERMINAL BODY WEIGHT (g) : 351.0

Tissue	Gross observations/comments	Correlated Microscopic Observations
GENER. CONDITION . . .	GOOD	No micropathology observations on tissue.
HEART	No gross observations on tissue	MYOCARDIAL INFLAMMATION, Minimal, Focal, Chronic.
KIDNEYS	No gross observations on tissue	CHRONIC INFLAMMATION, Minimal, Multifocal.
HARDERIAN GLANDS . . .	No gross observations on tissue	PORPHYRIN DEPOSITS, Minimal, Multifocal.
LIVER	No gross observations on tissue	CHRONIC INFLAMMATION, Minimal, Multifocal.
		HEPATOCELLULAR HYPERTROPHY,CENTRILOBULAR, Slight.
		HEPATOCELLULAR VACUOLATION,DIFFUSE, Slight.
MANDIBULAR L.N.	No gross observations on tissue	PLASMACYTOSIS, Slight, Diffuse.
LUNG	No gross observations on tissue	ALVEOLAR HEMORRHAGE, Slight, Focal.
		ALVEOLAR MACROPHAGE INFILTRATION, Minimal, Multifocal.

The following tissues have no gross observations and were not examined microscopically:
No tissue to list.

Appendix 10
Individual Animal Microscopic vs. Gross Observations

Fexinidazole

Study Number: 0504-2007

ANIMAL: 2777 SEX: Male GROUP: 4 DOSE LEVEL: 800 mg/kg
DAY OF DEATH: 29 Test period STATUS: Scheduled phase sacrifice # 1 TERMINAL BODY WEIGHT (g) : 351.0

Tissue	Gross observations/comments	Correlated Microscopic Observations

The following tissues have no gross observations and were marked as unremarkable microscopically:

ADRENALS	AORTA	BONE MARROW	BRAIN	CECUM
COLON	DIAPHRAGM	DUODENUM	EPIDIDYMIDES	ESOPHAGUS
EYES	FEMUR	ILEUM	JEJUNUM	MESENTERIC L.N.
MAMMARY GLAND	SKELETAL MUSCLE	SCIATIC NERVE	OPTIC NERVES	PANCREAS
PITUITARY	PROSTATE	PARATHYROIDS	SPINAL CORD CERV	SPINAL CORD THOR
MANDIBULAR S.G.	PAROTIDS	STIFLE JOINT	SKIN	SPLEEN
STOMACH	STERNUM	SEMINAL VESICLES	TESTES	THYROIDS
THYMUS	TONGUE	TRACHEA	URINARY BLADDER	

Appendix 10
Individual Animal Microscopic vs. Gross Observations

Fexinidazole   Study Number: 0504-2007

ANIMAL: 2778   SEX: Male   GROUP: 4   DOSE LEVEL: 800 mg/kg
DAY OF DEATH: 29 Test period   STATUS: Scheduled phase sacrifice # 1   TERMINAL BODY WEIGHT (g) : 338.8

Tissue	Gross observations/comments	Correlated Microscopic Observations
GENER. CONDITION . . .	GOOD	No micropathology observations on tissue.
HARDERIAN GLANDS . . .	No gross observations on tissue	PORPHYRIN DEPOSITS, Minimal, Multifocal.
LIVER	No gross observations on tissue	CHRONIC INFLAMMATION, Minimal, Multifocal.<br>HEPATOCELLULAR HYPERTROPHY,CENTRILOBULAR, Slight.
MANDIBULAR L.N.	No gross observations on tissue	PLASMACYTOSIS, Slight, Diffuse.
LUNG	No gross observations on tissue	ALVEOLAR MACROPHAGE INFILTRATION, Minimal, Multifocal.
SPLEEN	No gross observations on tissue	EXTRAMEDULLARY HEMOPOIESIS, Minimal, Multifocal.

The following tissues have no gross observations and were not examined microscopically:
No tissue to list.

The following tissues have no gross observations and were marked as unremarkable microscopically:

ADRENALS	AORTA	BONE MARROW	BRAIN	CECUM
COLON	DIAPHRAGM	DUODENUM	EPIDIDYMIDES	ESOPHAGUS
EYES	FEMUR	HEART	ILEUM	JEJUNUM
KIDNEYS	MESENTERIC L.N.	MAMMARY GLAND	SKELETAL MUSCLE	SCIATIC NERVE
OPTIC NERVES	PANCREAS	PITUITARY	PROSTATE	PARATHYROIDS
SPINAL CORD CERV	SPINAL CORD THOR	MANDIBULAR S.G.	PAROTIDS	STIFLE JOINT
SKIN	STOMACH	STERNUM	SEMINAL VESICLES	TESTES

Appendix 10
Individual Animal Microscopic vs. Gross Observations

Fexinidazole | Study Number: 0504-2007

ANIMAL: 2778 | SEX: Male | GROUP: 4 | DOSE LEVEL: 800 mg/kg
DAY OF DEATH: 29 Test period | STATUS: Scheduled phase sacrifice # 1 | TERMINAL BODY WEIGHT (g) : 338.8

Tissue	Gross observations/comments	Correlated Microscopic Observations

THYROIDS THYMUS TONGUE TRACHEA URINARY BLADDER

Appendix 10
Individual Animal Microscopic vs. Gross Observations

Fexinidazole   Study Number: 0504-2007

ANIMAL: 2779   SEX: Male   GROUP: 4   DOSE LEVEL: 800 mg/kg
DAY OF DEATH: 29 Test period   STATUS: Scheduled phase sacrifice # 1   TERMINAL BODY WEIGHT (g) : 322.7

Tissue	Gross observations/comments	Correlated Microscopic Observations
GENER. CONDITION . . .	GOOD	No micropathology observations on tissue.
HARDERIAN GLANDS . . .	No gross observations on tissue	PORPHYRIN DEPOSITS, Moderate, Multifocal.
LIVER	No gross observations on tissue	CHRONIC INFLAMMATION, Slight, Multifocal.<br>HEPATOCELLULAR HYPERTROPHY,CENTRILOBULAR, Slight.
MANDIBULAR L.N.	No gross observations on tissue	PLASMACYTOSIS, Marked, Diffuse, Unilateral..
PROSTATE	No gross observations on tissue	LYMPHOCYTIC INFILTRATION, Minimal, Multifocal.
PARATHYROIDS	No gross observations on tissue	ONLY ONE PARATHYROID AVAILABLE FOR EXAMINATION, Present.

The following tissues have no gross observations and were not examined microscopically:
No tissue to list.

The following tissues have no gross observations and were marked as unremarkable microscopically:

ADRENALS	AORTA	BONE MARROW	BRAIN	CECUM
COLON	DIAPHRAGM	DUODENUM	EPIDIDYMIDES	ESOPHAGUS
EYES	FEMUR	HEART	ILEUM	JEJUNUM
KIDNEYS	MESENTERIC L.N.	LUNG	MAMMARY GLAND	SKELETAL MUSCLE
SCIATIC NERVE	OPTIC NERVES	PANCREAS	PITUITARY	SPINAL CORD CERV
SPINAL CORD THOR	MANDIBULAR S.G.	PAROTIDS	STIFLE JOINT	SKIN

Appendix 10
Individual Animal Microscopic vs. Gross Observations

Fexinidazole Study Number: 0504-2007

ANIMAL: 2779 SEX: Male GROUP: 4 DOSE LEVEL: 800 mg/kg
DAY OF DEATH: 29 Test period STATUS: Scheduled phase sacrifice # 1 TERMINAL BODY WEIGHT (g) : 322.7

Tissue	Gross observations/comments	Correlated Microscopic Observations

SPLEEN	STOMACH	STERNUM	SEMINAL VESICLES	TESTES
THYROIDS	THYMUS	TONGUE	TRACHEA	URINARY BLADDER

Appendix 10
Individual Animal Microscopic vs. Gross Observations

Fexinidazole | Study Number: 0504-2007

ANIMAL: 2780 | SEX: Male | GROUP: 4 | DOSE LEVEL: 800 mg/kg
DAY OF DEATH: 43 Test period | STATUS: Final phase sacrifice | TERMINAL BODY WEIGHT (g) : 369.0

Tissue	Gross observations/comments	Correlated Microscopic Observations
GENER. CONDITION . . .	GOOD	No micropathology observations on tissue.
HARDERIAN GLANDS . . .	No gross observations on tissue	PORPHYRIN DEPOSITS, Slight, Multifocal.<br>HEMORRHAGE,UNILATERAL, Moderate, Focal.
LIVER	No gross observations on tissue	CHRONIC INFLAMMATION, Minimal, Multifocal.<br>HEPATOCELLULAR VACUOLATION,PERIPORTAL, Minimal.

The following tissues have no gross observations and were not examined microscopically:

ADRENALS	AORTA	BONE MARROW	BRAIN	CECUM
COLON	DIAPHRAGM	DUODENUM	EPIDIDYMIDES	ESOPHAGUS
EYES	FEMUR	HEART	ILEUM	JEJUNUM
KIDNEYS	MANDIBULAR L.N.	MESENTERIC L.N.	LUNG	MAMMARY GLAND
SKELETAL MUSCLE	SCIATIC NERVE	OPTIC NERVES	PANCREAS	PITUITARY
PROSTATE	PARATHYROIDS	SPINAL CORD CERV	SPINAL CORD THOR	MANDIBULAR S.G.
PAROTIDS	STIFLE JOINT	SKIN	SPLEEN	STOMACH
STERNUM	SEMINAL VESICLES	TESTES	THYROIDS	THYMUS
TONGUE	TRACHEA	URINARY BLADDER		

The following tissues have no gross observations and were marked as unremarkable microscopically:
No tissue to list.

Appendix 10
Individual Animal Microscopic vs. Gross Observations

Fexinidazole | Study Number: 0504-2007

ANIMAL: 2781 | SEX: Male | GROUP: 4 | DOSE LEVEL: 800 mg/kg
DAY OF DEATH: 43 Test period | STATUS: Final phase sacrifice | TERMINAL BODY WEIGHT (g) : 341.6

Tissue	Gross observations/comments	Correlated Microscopic Observations
GENER. CONDITION . . .	GOOD	No micropathology observations on tissue.
HARDERIAN GLANDS . . .	No gross observations on tissue	PORPHYRIN DEPOSITS, Moderate, Multifocal.<br>HEMORRHAGE,UNILATERAL, Minimal, Multifocal.
LIVER	No gross observations on tissue	CHRONIC INFLAMMATION, Minimal, Multifocal.

The following tissues have no gross observations and were not examined microscopically:

ADRENALS	AORTA	BONE MARROW	BRAIN	CECUM
COLON	DIAPHRAGM	DUODENUM	EPIDIDYMIDES	ESOPHAGUS
EYES	FEMUR	HEART	ILEUM	JEJUNUM
KIDNEYS	MANDIBULAR L.N.	MESENTERIC L.N.	LUNG	MAMMARY GLAND
SKELETAL MUSCLE	SCIATIC NERVE	OPTIC NERVES	PANCREAS	PITUITARY
PROSTATE	PARATHYROIDS	SPINAL CORD CERV	SPINAL CORD THOR	MANDIBULAR S.G.
PAROTIDS	STIFLE JOINT	SKIN	SPLEEN	STOMACH
STERNUM	SEMINAL VESICLES	TESTES	THYROIDS	THYMUS
TONGUE	TRACHEA	URINARY BLADDER		

The following tissues have no gross observations and were marked as unremarkable microscopically:
No tissue to list.

Appendix 10
Individual Animal Microscopic vs. Gross Observations

Fexinidazole | Study Number: 0504-2007

ANIMAL: 2782 | SEX: Male | GROUP: 4 | DOSE LEVEL: 800 mg/kg
DAY OF DEATH: 43 Test period | STATUS: Final phase sacrifice | TERMINAL BODY WEIGHT (g) : 359.3

Tissue	Gross observations/comments	Correlated Microscopic Observations
GENER. CONDITION . . .	GOOD	No micropathology observations on tissue.
HARDERIAN GLANDS . . .	No gross observations on tissue	PORPHYRIN DEPOSITS, Moderate, Multifocal.<br>HEMORRHAGE,UNILATERAL, Slight, Multifocal.<br>ADENITIS,UNILATERAL, Slight, Multifocal, Acute.
LIVER	No gross observations on tissue	CHRONIC INFLAMMATION, Minimal, Multifocal.<br>HEPATOCELLULAR HYPERTROPHY,CENTRILOBULAR, Minimal.

The following tissues have no gross observations and were not examined microscopically:

ADRENALS	AORTA	BONE MARROW	BRAIN	CECUM
COLON	DIAPHRAGM	DUODENUM	EPIDIDYMIDES	ESOPHAGUS
EYES	FEMUR	HEART	ILEUM	JEJUNUM
KIDNEYS	MANDIBULAR L.N.	MESENTERIC L.N.	LUNG	MAMMARY GLAND
SKELETAL MUSCLE	SCIATIC NERVE	OPTIC NERVES	PANCREAS	PITUITARY
PROSTATE	PARATHYROIDS	SPINAL CORD CERV	SPINAL CORD THOR	MANDIBULAR S.G.
PAROTIDS	STIFLE JOINT	SKIN	SPLEEN	STOMACH
STERNUM	SEMINAL VESICLES	TESTES	THYROIDS	THYMUS
TONGUE	TRACHEA	URINARY BLADDER		

The following tissues have no gross observations and were marked as unremarkable microscopically:
No tissue to list.

Appendix 10
Individual Animal Microscopic vs. Gross Observations

Fexinidazole — Study Number: 0504-2007

ANIMAL: 2783 SEX: Male GROUP: 4 DOSE LEVEL: 800 mg/kg
DAY OF DEATH: 43 Test period STATUS: Final phase sacrifice TERMINAL BODY WEIGHT (g) : 407.1

Tissue	Gross observations/comments	Correlated Microscopic Observations
GENER. CONDITION . . .	GOOD	No micropathology observations on tissue.
HARDERIAN GLANDS . . .	No gross observations on tissue	PORPHYRIN DEPOSITS, Slight, Multifocal.
LIVER	No gross observations on tissue	CHRONIC INFLAMMATION, Minimal, Multifocal.

The following tissues have no gross observations and were not examined microscopically:

ADRENALS	AORTA	BONE MARROW	BRAIN	CECUM
COLON	DIAPHRAGM	DUODENUM	EPIDIDYMIDES	ESOPHAGUS
EYES	FEMUR	HEART	ILEUM	JEJUNUM
KIDNEYS	MANDIBULAR L.N.	MESENTERIC L.N.	LUNG	MAMMARY GLAND
SKELETAL MUSCLE	SCIATIC NERVE	OPTIC NERVES	PANCREAS	PITUITARY
PROSTATE	PARATHYROIDS	SPINAL CORD CERV	SPINAL CORD THOR	MANDIBULAR S.G.
PAROTIDS	STIFLE JOINT	SKIN	SPLEEN	STOMACH
STERNUM	SEMINAL VESICLES	TESTES	THYROIDS	THYMUS
TONGUE	TRACHEA	URINARY BLADDER		

The following tissues have no gross observations and were marked as unremarkable microscopically:
No tissue to list.

Appendix 10
Individual Animal Microscopic vs. Gross Observations

Fexinidazole Study Number: 0504-2007

ANIMAL: 2784 SEX: Male GROUP: 4 DOSE LEVEL: 800 mg/kg
DAY OF DEATH: 43 Test period STATUS: Final phase sacrifice TERMINAL BODY WEIGHT (g) : 385.5

Tissue	Gross observations/comments	Correlated Microscopic Observations
GENER. CONDITION . . .	GOOD	No micropathology observations on tissue.
HARDERIAN GLANDS . . .	No gross observations on tissue	PORPHYRIN DEPOSITS, Moderate, Multifocal.<br>ADENITIS,UNILATERAL, Slight, Focal, Chronic.
LIVER	No gross observations on tissue	CHRONIC INFLAMMATION, Minimal, Multifocal.<br>HEPATOCELLULAR HYPERTROPHY,CENTRILOBULAR, Minimal.

The following tissues have no gross observations and were not examined microscopically:

ADRENALS	AORTA	BONE MARROW	BRAIN	CECUM
COLON	DIAPHRAGM	DUODENUM	EPIDIDYMIDES	ESOPHAGUS
EYES	FEMUR	HEART	ILEUM	JEJUNUM
KIDNEYS	MANDIBULAR L.N.	MESENTERIC L.N.	LUNG	MAMMARY GLAND
SKELETAL MUSCLE	SCIATIC NERVE	OPTIC NERVES	PANCREAS	PITUITARY
PROSTATE	PARATHYROIDS	SPINAL CORD CERV	SPINAL CORD THOR	MANDIBULAR S.G.
PAROTIDS	STIFLE JOINT	SKIN	SPLEEN	STOMACH
STERNUM	SEMINAL VESICLES	TESTES	THYROIDS	THYMUS
TONGUE	TRACHEA	URINARY BLADDER		

The following tissues have no gross observations and were marked as unremarkable microscopically:
No tissue to list.

Appendix 10
Individual Animal Microscopic vs. Gross Observations

Fexinidazole | Study Number: 0504-2007

ANIMAL: 2785 | SEX: Female | GROUP: 1 | DOSE LEVEL: vehicle
DAY OF DEATH: 30 Test period | STATUS: Scheduled phase sacrifice # 1 | TERMINAL BODY WEIGHT (g) : 212.4

Tissue	Gross observations/comments	Correlated Microscopic Observations
GENER. CONDITION . . .	GOOD	No micropathology observations on tissue.
HARDERIAN GLANDS . . .	No gross observations on tissue	PORPHYRIN DEPOSITS, Minimal, Multifocal, Unilateral..
LIVER	No gross observations on tissue	CHRONIC INFLAMMATION, Minimal, Multifocal.
		HEPATOCELLULAR VACUOLATION,PERIPORTAL, Minimal.
MANDIBULAR L.N.	No gross observations on tissue	PLASMACYTOSIS, Slight, Diffuse.
SPLEEN	No gross observations on tissue	EXTRAMEDULLARY HEMOPOIESIS, Minimal, Multifocal.

The following tissues have no gross observations and were not examined microscopically:
No tissue to list.

The following tissues have no gross observations and were marked as unremarkable microscopically:

ADRENALS	AORTA	BONE MARROW	BRAIN	CECUM
COLON	DIAPHRAGM	DUODENUM	ESOPHAGUS	EYES
FEMUR	HEART	ILEUM	JEJUNUM	KIDNEYS
MESENTERIC L.N.	LUNG	MAMMARY GLAND	SKELETAL MUSCLE	SCIATIC NERVE
OPTIC NERVES	OVARIES	PANCREAS	PITUITARY	PARATHYROIDS
SPINAL CORD CERV	SPINAL CORD THOR	MANDIBULAR S.G.	PAROTIDS	STIFLE JOINT
SKIN	STOMACH	STERNUM	THYROIDS	THYMUS
TONGUE	TRACHEA	URINARY BLADDER	UTERUS	VAGINA

Appendix 10
Individual Animal Microscopic vs. Gross Observations

Fexinidazole   Study Number: 0504-2007

ANIMAL: 2786   SEX: Female   GROUP: 1   DOSE LEVEL: vehicle
DAY OF DEATH: 30 Test period   STATUS: Scheduled phase sacrifice # 1   TERMINAL BODY WEIGHT (g) : 243.2

Tissue	Gross observations/comments	Correlated Microscopic Observations
GENER. CONDITION . . .	GOOD	No micropathology observations on tissue.
HEART	No gross observations on tissue	MYOCARDIAL INFLAMMATION, Minimal, Multifocal, Chronic.
HARDERIAN GLANDS . . .	No gross observations on tissue	PORPHYRIN DEPOSITS, Minimal, Multifocal.
LIVER	No gross observations on tissue	CHRONIC INFLAMMATION, Minimal, Multifocal.
		HEPATOCELLULAR VACUOLATION,PERIPORTAL, Slight.
MANDIBULAR L.N.	No gross observations on tissue	PLASMACYTOSIS, Slight, Diffuse, Unilateral..
SKELETAL MUSCLE	No gross observations on tissue	CHRONIC INFLAMMATION, Minimal, Multifocal.
PANCREAS	No gross observations on tissue	CHRONIC INFLAMMATION, Minimal, Multifocal.

The following tissues have no gross observations and were not examined microscopically:
No tissue to list.

The following tissues have no gross observations and were marked as unremarkable microscopically:
ADRENALS   AORTA   BONE MARROW   BRAIN   CECUM

Appendix 10
Individual Animal Microscopic vs. Gross Observations

Fexinidazole Study Number: 0504-2007

ANIMAL: 2786 SEX: Female GROUP: 1 DOSE LEVEL: vehicle
DAY OF DEATH: 30 Test period STATUS: Scheduled phase sacrifice # 1 TERMINAL BODY WEIGHT (g) : 243.2

Tissue	Gross observations/comments	Correlated Microscopic Observations

The following tissues have no gross observations and were marked as unremarkable microscopically:

COLON	DIAPHRAGM	DUODENUM	ESOPHAGUS	EYES
FEMUR	ILEUM	JEJUNUM	KIDNEYS	MESENTERIC L.N.
LUNG	MAMMARY GLAND	SCIATIC NERVE	OPTIC NERVES	OVARIES
PITUITARY	PARATHYROIDS	SPINAL CORD CERV	SPINAL CORD THOR	MANDIBULAR S.G.
PAROTIDS	STIFLE JOINT	SKIN	SPLEEN	STOMACH
STERNUM	THYROIDS	THYMUS	TONGUE	TRACHEA
URINARY BLADDER	UTERUS	VAGINA		

Appendix 10
Individual Animal Microscopic vs. Gross Observations

Fexinidazole   Study Number: 0504-2007

ANIMAL: 2787   SEX: Female   GROUP: 1   DOSE LEVEL: vehicle
DAY OF DEATH: 30 Test period   STATUS: Scheduled phase sacrifice # 1   TERMINAL BODY WEIGHT (g) : 219.6

Tissue	Gross observations/comments	Correlated Microscopic Observations
ADRENALS	No gross observations on tissue	CORTICAL VACUOLATION, Slight, Focal, Unilateral..
GENER. CONDITION . . .	GOOD	No micropathology observations on tissue.
HARDERIAN GLANDS . . .	No gross observations on tissue	PORPHYRIN DEPOSITS, Minimal, Multifocal.
LIVER	No gross observations on tissue	CHRONIC INFLAMMATION, Minimal, Multifocal.
MANDIBULAR L.N.	No gross observations on tissue	PLASMACYTOSIS, Moderate, Diffuse.
PARATHYROIDS	No gross observations on tissue	ONLY ONE PARATHYROID AVAILABLE FOR EXAMINATION, Present.

The following tissues have no gross observations and were not examined microscopically:
No tissue to list.

The following tissues have no gross observations and were marked as unremarkable microscopically:

AORTA	BONE MARROW	BRAIN	CECUM	COLON
DIAPHRAGM	DUODENUM	ESOPHAGUS	EYES	FEMUR
HEART	ILEUM	JEJUNUM	KIDNEYS	MESENTERIC L.N.
LUNG	MAMMARY GLAND	SKELETAL MUSCLE	SCIATIC NERVE	OPTIC NERVES
OVARIES	PANCREAS	PITUITARY	SPINAL CORD CERV	SPINAL CORD THOR
MANDIBULAR S.G.	PAROTIDS	STIFLE JOINT	SKIN	SPLEEN
STOMACH	STERNUM	THYROIDS	THYMUS	TONGUE
TRACHEA	URINARY BLADDER	UTERUS	VAGINA	

Appendix 10
Individual Animal Microscopic vs. Gross Observations

Fexinidazole — Study Number: 0504-2007

ANIMAL: 2788 — SEX: Female — GROUP: 1 — DOSE LEVEL: vehicle
DAY OF DEATH: 30 Test period — STATUS: Scheduled phase sacrifice # 1 — TERMINAL BODY WEIGHT (g) : 222.6

Tissue	Gross observations/comments	Correlated Microscopic Observations
GENER. CONDITION . . .	GOOD	No micropathology observations on tissue.
HARDERIAN GLANDS . . .	No gross observations on tissue	PORPHYRIN DEPOSITS, Minimal, Multifocal.
LIVER	No gross observations on tissue	CHRONIC INFLAMMATION, Minimal, Multifocal.<br>HEPATOCELLULAR VACUOLATION,PERIPORTAL, Slight.
MANDIBULAR L.N.	No gross observations on tissue	PLASMACYTOSIS, Moderate, Diffuse.
LUNG	No gross observations on tissue	ALVEOLAR MACROPHAGE INFILTRATION, Minimal, Multifocal.
OPTIC NERVES	No gross observations on tissue	ONLY ONE NERVE IS AVAILABLE FOR EXAMINATION, Present.
PARATHYROIDS	No gross observations on tissue	ONLY ONE PARATHYROID AVAILABLE FOR EXAMINATION, Present.

The following tissues have no gross observations and were not examined microscopically:
No tissue to list.

The following tissues have no gross observations and were marked as unremarkable microscopically:
ADRENALS AORTA BONE MARROW BRAIN CECUM

Appendix 10
Individual Animal Microscopic vs. Gross Observations

Fexinidazole Study Number: 0504-2007

ANIMAL: 2788 SEX: Female GROUP: 1 DOSE LEVEL: vehicle
DAY OF DEATH: 30 Test period STATUS: Scheduled phase sacrifice # 1 TERMINAL BODY WEIGHT (g) : 222.6

Tissue	Gross observations/comments	Correlated Microscopic Observations

The following tissues have no gross observations and were marked as unremarkable microscopically:

COLON	DIAPHRAGM	DUODENUM	ESOPHAGUS	EYES
FEMUR	HEART	ILEUM	JEJUNUM	KIDNEYS
MESENTERIC L.N.	MAMMARY GLAND	SKELETAL MUSCLE	SCIATIC NERVE	OVARIES
PANCREAS	PITUITARY	SPINAL CORD CERV	SPINAL CORD THOR	MANDIBULAR S.G.
PAROTIDS	STIFLE JOINT	SKIN	SPLEEN	STOMACH
STERNUM	THYROIDS	THYMUS	TONGUE	TRACHEA
URINARY BLADDER	UTERUS	VAGINA		

Appendix 10
Individual Animal Microscopic vs. Gross Observations

Fexinidazole | Study Number: 0504-2007

ANIMAL: 2789 | SEX: Female | GROUP: 1 | DOSE LEVEL: vehicle
DAY OF DEATH: 30 Test period | STATUS: Scheduled phase sacrifice # 1 | TERMINAL BODY WEIGHT (g) : 208.3

Tissue	Gross observations/comments	Correlated Microscopic Observations
GENER. CONDITION . . .	GOOD	No micropathology observations on tissue.
KIDNEYS	No gross observations on tissue	CHRONIC INFLAMMATION, Minimal, Focal, Unilateral left..
		MEDULLARY TUBULAR DILATATION, Moderate, Focal, Unilateral right..
HARDERIAN GLANDS . . .	No gross observations on tissue	PORPHYRIN DEPOSITS, Minimal, Multifocal.
		LYMPHOCYTIC INFILTRATION, Minimal, Multifocal.
LIVER	No gross observations on tissue	CHRONIC INFLAMMATION, Minimal, Multifocal.
MANDIBULAR L.N.	No gross observations on tissue	PLASMACYTOSIS, Moderate, Diffuse, Unilateral..
LUNG	No gross observations on tissue	ALVEOLAR MACROPHAGE INFILTRATION, Minimal, Multifocal.
OPTIC NERVES	No gross observations on tissue	ONLY ONE NERVE IS AVAILABLE FOR EXAMINATION, Present.
PARATHYROIDS	No gross observations on tissue	ONLY ONE PARATHYROID AVAILABLE FOR EXAMINATION, Present.

Appendix 10
Individual Animal Microscopic vs. Gross Observations

Fexinidazole Study Number: 0504-2007

ANIMAL: 2789 SEX: Female GROUP: 1 DOSE LEVEL: vehicle
DAY OF DEATH: 30 Test period STATUS: Scheduled phase sacrifice # 1 TERMINAL BODY WEIGHT (g) : 208.3

Tissue	Gross observations/comments	Correlated Microscopic Observations

The following tissues have no gross observations and were not examined microscopically:
No tissue to list.

The following tissues have no gross observations and were marked as unremarkable microscopically:

ADRENALS	AORTA	BONE MARROW	BRAIN	CECUM
COLON	DIAPHRAGM	DUODENUM	ESOPHAGUS	EYES
FEMUR	HEART	ILEUM	JEJUNUM	MESENTERIC L.N.
MAMMARY GLAND	SKELETAL MUSCLE	SCIATIC NERVE	OVARIES	PANCREAS
PITUITARY	SPINAL CORD CERV	SPINAL CORD THOR	MANDIBULAR S.G.	PAROTIDS
STIFLE JOINT	SKIN	SPLEEN	STOMACH	STERNUM
THYROIDS	THYMUS	TONGUE	TRACHEA	URINARY BLADDER
UTERUS	VAGINA			

Appendix 10
Individual Animal Microscopic vs. Gross Observations

Fexinidazole | Study Number: 0504-2007

ANIMAL: 2790 | SEX: Female | GROUP: 1 | DOSE LEVEL: vehicle
DAY OF DEATH: 29 Test period | STATUS: Scheduled phase sacrifice # 1 | TERMINAL BODY WEIGHT (g) : 224.5

Tissue	Gross observations/comments	Correlated Microscopic Observations
GENER. CONDITION . . .	GOOD	No micropathology observations on tissue.
HARDERIAN GLANDS . . .	No gross observations on tissue	PORPHYRIN DEPOSITS, Minimal, Multifocal.
LIVER	No gross observations on tissue	CHRONIC INFLAMMATION, Minimal, Multifocal.<br>HEPATOCELLULAR VACUOLATION,PERIPORTAL, Minimal.
MANDIBULAR L.N.	No gross observations on tissue	PLASMACYTOSIS, Slight, Diffuse.
PARATHYROIDS	No gross observations on tissue	ONLY ONE PARATHYROID AVAILABLE FOR EXAMINATION, Present.

The following tissues have no gross observations and were not examined microscopically:
No tissue to list.

The following tissues have no gross observations and were marked as unremarkable microscopically:

ADRENALS	AORTA	BONE MARROW	BRAIN	CECUM
COLON	DIAPHRAGM	DUODENUM	ESOPHAGUS	EYES
FEMUR	HEART	ILEUM	JEJUNUM	KIDNEYS
MESENTERIC L.N.	LUNG	MAMMARY GLAND	SKELETAL MUSCLE	SCIATIC NERVE
OPTIC NERVES	OVARIES	PANCREAS	PITUITARY	SPINAL CORD CERV
SPINAL CORD THOR	MANDIBULAR S.G.	PAROTIDS	STIFLE JOINT	SKIN
SPLEEN	STOMACH	STERNUM	THYROIDS	THYMUS
TONGUE	TRACHEA	URINARY BLADDER	UTERUS	VAGINA

Appendix 10
Individual Animal Microscopic vs. Gross Observations

Fexinidazole Study Number: 0504-2007

ANIMAL: 2791 SEX: Female GROUP: 1 DOSE LEVEL: vehicle
DAY OF DEATH: 29 Test period STATUS: Scheduled phase sacrifice # 1 TERMINAL BODY WEIGHT (g) : 207.6

Tissue	Gross observations/comments	Correlated Microscopic Observations
GENER. CONDITION . . .	GOOD	No micropathology observations on tissue.
KIDNEYS	No gross observations on tissue	CHRONIC INFLAMMATION, Minimal, Focal, Unilateral left..
HARDERIAN GLANDS . . .	No gross observations on tissue	PORPHYRIN DEPOSITS, Minimal, Multifocal.
LIVER	No gross observations on tissue	CHRONIC INFLAMMATION, Minimal, Multifocal.
		HEPATOCELLULAR VACUOLATION,PERIPORTAL, Slight.
MANDIBULAR L.N.	No gross observations on tissue	PLASMACYTOSIS, Moderate, Diffuse.
THYROIDS	No gross observations on tissue	ECTOPIC THYMUS, Present, Unilateral..

The following tissues have no gross observations and were not examined microscopically:
No tissue to list.

The following tissues have no gross observations and were marked as unremarkable microscopically:

ADRENALS	AORTA	BONE MARROW	BRAIN	CECUM
COLON	DIAPHRAGM	DUODENUM	ESOPHAGUS	EYES
FEMUR	HEART	ILEUM	JEJUNUM	MESENTERIC L.N.
LUNG	MAMMARY GLAND	SKELETAL MUSCLE	SCIATIC NERVE	OPTIC NERVES
OVARIES	PANCREAS	PITUITARY	PARATHYROIDS	SPINAL CORD CERV
SPINAL CORD THOR	MANDIBULAR S.G.	PAROTIDS	STIFLE JOINT	SKIN
SPLEEN	STOMACH	STERNUM	THYMUS	TONGUE
TRACHEA	URINARY BLADDER	UTERUS	VAGINA	

Appendix 10
Individual Animal Microscopic vs. Gross Observations

Fexinidazole — Study Number: 0504-2007

ANIMAL: 2792 — SEX: Female — GROUP: 1 — DOSE LEVEL: vehicle
DAY OF DEATH: 29 Test period — STATUS: Scheduled phase sacrifice # 1 — TERMINAL BODY WEIGHT (g) : 234.0

Tissue	Gross observations/comments	Correlated Microscopic Observations
GENER. CONDITION . . .	GOOD	No micropathology observations on tissue.
HARDERIAN GLANDS . . .	No gross observations on tissue	PORPHYRIN DEPOSITS, Minimal, Multifocal, Unilateral..
LIVER	No gross observations on tissue	CHRONIC INFLAMMATION, Minimal, Multifocal.<br>HEPATOCELLULAR VACUOLATION,PERIPORTAL, Slight.
MANDIBULAR L.N.	No gross observations on tissue	PLASMACYTOSIS, Moderate, Diffuse.
LUNG	No gross observations on tissue	ALVEOLAR MACROPHAGE INFILTRATION, Minimal, Multifocal.

The following tissues have no gross observations and were not examined microscopically:
No tissue to list.

The following tissues have no gross observations and were marked as unremarkable microscopically:

ADRENALS	AORTA	BONE MARROW	BRAIN	CECUM
COLON	DIAPHRAGM	DUODENUM	ESOPHAGUS	EYES
FEMUR	HEART	ILEUM	JEJUNUM	KIDNEYS
MESENTERIC L.N.	MAMMARY GLAND	SKELETAL MUSCLE	SCIATIC NERVE	OPTIC NERVES
OVARIES	PANCREAS	PITUITARY	PARATHYROIDS	SPINAL CORD CERV
SPINAL CORD THOR	MANDIBULAR S.G.	PAROTIDS	STIFLE JOINT	SKIN
SPLEEN	STOMACH	STERNUM	THYROIDS	THYMUS
TONGUE	TRACHEA	URINARY BLADDER	UTERUS	VAGINA

Appendix 10
Individual Animal Microscopic vs. Gross Observations

Fexinidazole — Study Number: 0504-2007

ANIMAL: 2793 — SEX: Female — GROUP: 1 — DOSE LEVEL: vehicle
DAY OF DEATH: 29 Test period — STATUS: Scheduled phase sacrifice # 1 — TERMINAL BODY WEIGHT (g) : 217.3

Tissue	Gross observations/comments	Correlated Microscopic Observations
GENER. CONDITION . . .	GOOD	No micropathology observations on tissue.
LIVER	No gross observations on tissue	CHRONIC INFLAMMATION, Minimal, Multifocal.<br>HEPATOCELLULAR VACUOLATION,PERIPORTAL, Slight.
MANDIBULAR L.N.	No gross observations on tissue	PLASMACYTOSIS, Moderate, Diffuse.
LUNG	No gross observations on tissue	ALVEOLAR HEMORRHAGE, Minimal, Focal.<br>ALVEOLAR MACROPHAGE INFILTRATION, Minimal, Multifocal.
PARATHYROIDS	No gross observations on tissue	ONLY ONE PARATHYROID AVAILABLE FOR EXAMINATION, Present.

The following tissues have no gross observations and were not examined microscopically:
No tissue to list.

The following tissues have no gross observations and were marked as unremarkable microscopically:

ADRENALS	AORTA	BONE MARROW	BRAIN	CECUM
COLON	DIAPHRAGM	DUODENUM	ESOPHAGUS	EYES
FEMUR	HEART	ILEUM	JEJUNUM	KIDNEYS
HARDERIAN GLANDS	MESENTERIC L.N.	MAMMARY GLAND	SKELETAL MUSCLE	SCIATIC NERVE
OPTIC NERVES	OVARIES	PANCREAS	PITUITARY	SPINAL CORD CERV
SPINAL CORD THOR	MANDIBULAR S.G.	PAROTIDS	STIFLE JOINT	SKIN
SPLEEN	STOMACH	STERNUM	THYROIDS	THYMUS
TONGUE	TRACHEA	URINARY BLADDER	UTERUS	VAGINA

Appendix 10
Individual Animal Microscopic vs. Gross Observations

Fexinidazole | Study Number: 0504-2007

ANIMAL: 2794 | SEX: Female | GROUP: 1 | DOSE LEVEL: vehicle
DAY OF DEATH: 29 Test period | STATUS: Scheduled phase sacrifice # 1 | TERMINAL BODY WEIGHT (g) : 238.5

Tissue	Gross observations/comments	Correlated Microscopic Observations
GENER. CONDITION . . .	GOOD	No micropathology observations on tissue.
HARDERIAN GLANDS . . .	No gross observations on tissue	PORPHYRIN DEPOSITS, Minimal, Multifocal.
LIVER	No gross observations on tissue	CHRONIC INFLAMMATION, Minimal, Multifocal.<br>HEPATOCELLULAR VACUOLATION,PERIPORTAL, Minimal.
MANDIBULAR L.N.	No gross observations on tissue	PLASMACYTOSIS, Moderate, Diffuse.
MAMMARY GLAND	No gross observations on tissue	CERVICAL MAMMARY GLAND EXAMINED, Present.
OPTIC NERVES	No gross observations on tissue	ONLY ONE NERVE IS AVAILABLE FOR EXAMINATION, Present.
PARATHYROIDS	No gross observations on tissue	ONLY ONE PARATHYROID AVAILABLE FOR EXAMINATION, Present.

The following tissues have no gross observations and were not examined microscopically:
No tissue to list.

The following tissues have no gross observations and were marked as unremarkable microscopically:
ADRENALS AORTA BONE MARROW BRAIN CECUM

Appendix 10
Individual Animal Microscopic vs. Gross Observations

Fexinidazole Study Number: 0504-2007

ANIMAL: 2794 SEX: Female GROUP: 1 DOSE LEVEL: vehicle
DAY OF DEATH: 29 Test period STATUS: Scheduled phase sacrifice # 1 TERMINAL BODY WEIGHT (g) : 238.5

Tissue	Gross observations/comments	Correlated Microscopic Observations

The following tissues have no gross observations and were marked as unremarkable microscopically:

COLON	DIAPHRAGM	DUODENUM	ESOPHAGUS	EYES
FEMUR	HEART	ILEUM	JEJUNUM	KIDNEYS
MESENTERIC L.N.	LUNG	SKELETAL MUSCLE	SCIATIC NERVE	OVARIES
PANCREAS	PITUITARY	SPINAL CORD CERV	SPINAL CORD THOR	MANDIBULAR S.G.
PAROTIDS	STIFLE JOINT	SKIN	SPLEEN	STOMACH
STERNUM	THYROIDS	THYMUS	TONGUE	TRACHEA
URINARY BLADDER	UTERUS	VAGINA		

Appendix 10
Individual Animal Microscopic vs. Gross Observations

Fexinidazole   Study Number: 0504-2007

ANIMAL: 2795   SEX: Female   GROUP: 1   DOSE LEVEL: vehicle
DAY OF DEATH: 43 Test period   STATUS: Final phase sacrifice   TERMINAL BODY WEIGHT (g) : 231.1

Tissue	Gross observations/comments	Correlated Microscopic Observations
GENER. CONDITION . . .	GOOD	No micropathology observations on tissue.
HARDERIAN GLANDS . . .	No gross observations on tissue	PORPHYRIN DEPOSITS, Slight, Multifocal.<br>HEMORRHAGE,UNILATERAL, Minimal, Multifocal.
LIVER	No gross observations on tissue	CHRONIC INFLAMMATION, Minimal, Multifocal.<br>HEPATOCELLULAR VACUOLATION,PERIPORTAL, Minimal.

The following tissues have no gross observations and were not examined microscopically:

ADRENALS	AORTA	BONE MARROW	BRAIN	CECUM
COLON	DIAPHRAGM	DUODENUM	ESOPHAGUS	EYES
FEMUR	HEART	ILEUM	JEJUNUM	KIDNEYS
MANDIBULAR L.N.	MESENTERIC L.N.	LUNG	MAMMARY GLAND	SKELETAL MUSCLE
SCIATIC NERVE	OPTIC NERVES	OVARIES	PANCREAS	PITUITARY
PARATHYROIDS	SPINAL CORD CERV	SPINAL CORD THOR	MANDIBULAR S.G.	PAROTIDS
STIFLE JOINT	SKIN	SPLEEN	STOMACH	STERNUM
THYROIDS	THYMUS	TONGUE	TRACHEA	URINARY BLADDER
UTERUS	VAGINA			

The following tissues have no gross observations and were marked as unremarkable microscopically:
No tissue to list.

Appendix 10
Individual Animal Microscopic vs. Gross Observations

Fexinidazole | Study Number: 0504-2007

ANIMAL: 2796 | SEX: Female | GROUP: 1 | DOSE LEVEL: vehicle
DAY OF DEATH: 43 Test period | STATUS: Final phase sacrifice | TERMINAL BODY WEIGHT (g) : 233.1

Tissue	Gross observations/comments	Correlated Microscopic Observations
GENER. CONDITION . . .	GOOD	No micropathology observations on tissue.
HARDERIAN GLANDS . . .	No gross observations on tissue	PORPHYRIN DEPOSITS, Minimal, Multifocal, Unilateral..
LIVER	No gross observations on tissue	CHRONIC INFLAMMATION, Minimal, Multifocal.<br>HEPATOCELLULAR VACUOLATION,PERIPORTAL, Slight.<br>FOCAL NECROSIS,SUBCAPSULAR, Minimal. / with associated chronic inflammation.

The following tissues have no gross observations and were not examined microscopically:

ADRENALS	AORTA	BONE MARROW	BRAIN	CECUM
COLON	DIAPHRAGM	DUODENUM	ESOPHAGUS	EYES
FEMUR	HEART	ILEUM	JEJUNUM	KIDNEYS
MANDIBULAR L.N.	MESENTERIC L.N.	LUNG	MAMMARY GLAND	SKELETAL MUSCLE
SCIATIC NERVE	OPTIC NERVES	OVARIES	PANCREAS	PITUITARY
PARATHYROIDS	SPINAL CORD CERV	SPINAL CORD THOR	MANDIBULAR S.G.	PAROTIDS
STIFLE JOINT	SKIN	SPLEEN	STOMACH	STERNUM
THYROIDS	THYMUS	TONGUE	TRACHEA	URINARY BLADDER
UTERUS	VAGINA			

The following tissues have no gross observations and were marked as unremarkable microscopically:
No tissue to list.

Appendix 10
Individual Animal Microscopic vs. Gross Observations

Fexinidazole | Study Number: 0504-2007

ANIMAL: 2797 | SEX: Female | GROUP: 1 | DOSE LEVEL: vehicle
DAY OF DEATH: 43 Test period | STATUS: Final phase sacrifice | TERMINAL BODY WEIGHT (g) : 227.5

Tissue	Gross observations/comments	Correlated Microscopic Observations
GENER. CONDITION . . .	GOOD	No micropathology observations on tissue.
HARDERIAN GLANDS . . .	No gross observations on tissue	PORPHYRIN DEPOSITS, Minimal, Multifocal.<br>HEMORRHAGE,UNILATERAL, Slight, Focal.<br>LYMPHOCYTIC INFILTRATION, Minimal, Focal, Unilateral..
LIVER	No gross observations on tissue	CHRONIC INFLAMMATION, Minimal, Multifocal.
STOMACH	DARK GLANDULAR MUCOSA/ one area, 1 mm in diameter.	Examined 1 correlation found:<br>EROSION OF GLANDULAR STOMACH, Minimal, Multifocal.

The following tissues have no gross observations and were not examined microscopically:

ADRENALS	AORTA	BONE MARROW	BRAIN	CECUM
COLON	DIAPHRAGM	DUODENUM	ESOPHAGUS	EYES
FEMUR	HEART	ILEUM	JEJUNUM	KIDNEYS
MANDIBULAR L.N.	MESENTERIC L.N.	LUNG	MAMMARY GLAND	SKELETAL MUSCLE
SCIATIC NERVE	OPTIC NERVES	OVARIES	PANCREAS	PITUITARY
PARATHYROIDS	SPINAL CORD CERV	SPINAL CORD THOR	MANDIBULAR S.G.	PAROTIDS
STIFLE JOINT	SKIN	SPLEEN	STERNUM	THYROIDS
THYMUS	TONGUE	TRACHEA	URINARY BLADDER	UTERUS
VAGINA				

The following tissues have no gross observations and were marked as unremarkable microscopically:
No tissue to list.

Appendix 10
Individual Animal Microscopic vs. Gross Observations

Fexinidazole — Study Number: 0504-2007

ANIMAL: 2798 — SEX: Female — GROUP: 1 — DOSE LEVEL: vehicle
DAY OF DEATH: 43 Test period — STATUS: Final phase sacrifice — TERMINAL BODY WEIGHT (g) : 255.1

Tissue	Gross observations/comments	Correlated Microscopic Observations
GENER. CONDITION . . .	GOOD	No micropathology observations on tissue.
HARDERIAN GLANDS . . .	No gross observations on tissue	PORPHYRIN DEPOSITS, Minimal, Multifocal.
LIVER	No gross observations on tissue	CHRONIC INFLAMMATION, Minimal, Multifocal.<br>HEPATOCELLULAR VACUOLATION,PERIPORTAL, Minimal.

The following tissues have no gross observations and were not examined microscopically:

ADRENALS	AORTA	BONE MARROW	BRAIN	CECUM
COLON	DIAPHRAGM	DUODENUM	ESOPHAGUS	EYES
FEMUR	HEART	ILEUM	JEJUNUM	KIDNEYS
MANDIBULAR L.N.	MESENTERIC L.N.	LUNG	MAMMARY GLAND	SKELETAL MUSCLE
SCIATIC NERVE	OPTIC NERVES	OVARIES	PANCREAS	PITUITARY
PARATHYROIDS	SPINAL CORD CERV	SPINAL CORD THOR	MANDIBULAR S.G.	PAROTIDS
STIFLE JOINT	SKIN	SPLEEN	STOMACH	STERNUM
THYROIDS	THYMUS	TONGUE	TRACHEA	URINARY BLADDER
UTERUS	VAGINA			

The following tissues have no gross observations and were marked as unremarkable microscopically:
No tissue to list.

Appendix 10
Individual Animal Microscopic vs. Gross Observations

Fexinidazole | Study Number: 0504-2007

ANIMAL: 2799 | SEX: Female | GROUP: 1 | DOSE LEVEL: vehicle
DAY OF DEATH: 43 Test period | STATUS: Final phase sacrifice | TERMINAL BODY WEIGHT (g) : 250.3

Tissue	Gross observations/comments	Correlated Microscopic Observations
GENER. CONDITION . . .	GOOD	No micropathology observations on tissue.
HARDERIAN GLANDS . . .	No gross observations on tissue	PORPHYRIN DEPOSITS, Slight, Multifocal.
		HEMORRHAGE,UNILATERAL, Slight, Multifocal.
LIVER	No gross observations on tissue	CHRONIC INFLAMMATION, Minimal, Multifocal.

The following tissues have no gross observations and were not examined microscopically:

ADRENALS	AORTA	BONE MARROW	BRAIN	CECUM
COLON	DIAPHRAGM	DUODENUM	ESOPHAGUS	EYES
FEMUR	HEART	ILEUM	JEJUNUM	KIDNEYS
MANDIBULAR L.N.	MESENTERIC L.N.	LUNG	MAMMARY GLAND	SKELETAL MUSCLE
SCIATIC NERVE	OPTIC NERVES	OVARIES	PANCREAS	PITUITARY
PARATHYROIDS	SPINAL CORD CERV	SPINAL CORD THOR	MANDIBULAR S.G.	PAROTIDS
STIFLE JOINT	SKIN	SPLEEN	STOMACH	STERNUM
THYROIDS	THYMUS	TONGUE	TRACHEA	URINARY BLADDER
UTERUS	VAGINA			

The following tissues have no gross observations and were marked as unremarkable microscopically:
No tissue to list.

Appendix 10
Individual Animal Microscopic vs. Gross Observations

Fexinidazole — Study Number: 0504-2007

ANIMAL: 2800 | SEX: Female | GROUP: 2 | DOSE LEVEL: 50 mg/kg
DAY OF DEATH: 30 Test period | STATUS: Scheduled phase sacrifice # 1 | TERMINAL BODY WEIGHT (g) : 216.8

Tissue	Gross observations/comments	Correlated Microscopic Observations
GENER. CONDITION . . .	GOOD	No micropathology observations on tissue.
HARDERIAN GLANDS . . .	No gross observations on tissue	PORPHYRIN DEPOSITS, Minimal, Multifocal.<br>LYMPHOCYTIC INFILTRATION, Minimal, Focal, Unilateral..
LIVER	No gross observations on tissue	CHRONIC INFLAMMATION, Minimal, Multifocal.<br>HEPATOCELLULAR HYPERTROPHY,CENTRILOBULAR, Minimal.<br>HEPATOCELLULAR VACUOLATION,PERIPORTAL, Minimal.

The following tissues have no gross observations and were not examined microscopically:

ADRENALS	AORTA	BONE MARROW	BRAIN	CECUM
COLON	DIAPHRAGM	DUODENUM	ESOPHAGUS	EYES
FEMUR	HEART	ILEUM	JEJUNUM	KIDNEYS
MANDIBULAR L.N.	MESENTERIC L.N.	LUNG	MAMMARY GLAND	SKELETAL MUSCLE
SCIATIC NERVE	OPTIC NERVES	OVARIES	PANCREAS	PITUITARY
PARATHYROIDS	SPINAL CORD CERV	SPINAL CORD THOR	MANDIBULAR S.G.	PAROTIDS
STIFLE JOINT	SKIN	SPLEEN	STOMACH	STERNUM
THYROIDS	THYMUS	TONGUE	TRACHEA	URINARY BLADDER
UTERUS	VAGINA			

The following tissues have no gross observations and were marked as unremarkable microscopically:
No tissue to list.

Appendix 10
Individual Animal Microscopic vs. Gross Observations

Fexinidazole Study Number: 0504-2007

ANIMAL: 2801 SEX: Female GROUP: 2 DOSE LEVEL: 50 mg/kg
DAY OF DEATH: 30 Test period STATUS: Scheduled phase sacrifice # 1 TERMINAL BODY WEIGHT (g) : 247.9

Tissue	Gross observations/comments	Correlated Microscopic Observations
GENER. CONDITION . . .	GOOD	No micropathology observations on tissue.
HARDERIAN GLANDS . . .	No gross observations on tissue	PORPHYRIN DEPOSITS, Slight, Multifocal.
LIVER	No gross observations on tissue	CHRONIC INFLAMMATION, Minimal, Multifocal.
		HEPATOCELLULAR HYPERTROPHY,CENTRILOBULAR, Minimal.
		HEPATOCELLULAR VACUOLATION,PERIPORTAL, Minimal.

The following tissues have no gross observations and were not examined microscopically:

ADRENALS	AORTA	BONE MARROW	BRAIN	CECUM
COLON	DIAPHRAGM	DUODENUM	ESOPHAGUS	EYES
FEMUR	HEART	ILEUM	JEJUNUM	KIDNEYS
MANDIBULAR L.N.	MESENTERIC L.N.	LUNG	MAMMARY GLAND	SKELETAL MUSCLE
SCIATIC NERVE	OPTIC NERVES	OVARIES	PANCREAS	PITUITARY
PARATHYROIDS	SPINAL CORD CERV	SPINAL CORD THOR	MANDIBULAR S.G.	PAROTIDS
STIFLE JOINT	SKIN	SPLEEN	STOMACH	STERNUM
THYROIDS	THYMUS	TONGUE	TRACHEA	URINARY BLADDER
UTERUS	VAGINA			

The following tissues have no gross observations and were marked as unremarkable microscopically:
No tissue to list.

Appendix 10
Individual Animal Microscopic vs. Gross Observations

Fexinidazole | Study Number: 0504-2007

ANIMAL: 2802 | SEX: Female | GROUP: 2 | DOSE LEVEL: 50 mg/kg
DAY OF DEATH: 30 Test period | STATUS: Scheduled phase sacrifice # 1 | TERMINAL BODY WEIGHT (g) : 235.2

Tissue	Gross observations/comments	Correlated Microscopic Observations
GENER. CONDITION . . .	GOOD	No micropathology observations on tissue.
HARDERIAN GLANDS . . .	No gross observations on tissue	PORPHYRIN DEPOSITS, Moderate, Multifocal.
LIVER	No gross observations on tissue	CHRONIC INFLAMMATION, Minimal, Multifocal.<br>HEPATOCELLULAR HYPERTROPHY,CENTRILOBULAR, Minimal.<br>HEPATOCELLULAR VACUOLATION,PERIPORTAL, Minimal.

The following tissues have no gross observations and were not examined microscopically:

ADRENALS	AORTA	BONE MARROW	BRAIN	CECUM
COLON	DIAPHRAGM	DUODENUM	ESOPHAGUS	EYES
FEMUR	HEART	ILEUM	JEJUNUM	KIDNEYS
MANDIBULAR L.N.	MESENTERIC L.N.	LUNG	MAMMARY GLAND	SKELETAL MUSCLE
SCIATIC NERVE	OPTIC NERVES	OVARIES	PANCREAS	PITUITARY
PARATHYROIDS	SPINAL CORD CERV	SPINAL CORD THOR	MANDIBULAR S.G.	PAROTIDS
STIFLE JOINT	SKIN	SPLEEN	STOMACH	STERNUM
THYROIDS	THYMUS	TONGUE	TRACHEA	URINARY BLADDER
UTERUS	VAGINA			

The following tissues have no gross observations and were marked as unremarkable microscopically:
No tissue to list.

Appendix 10
Individual Animal Microscopic vs. Gross Observations

Fexinidazole | Study Number: 0504-2007

ANIMAL: 2803 | SEX: Female | GROUP: 2 | DOSE LEVEL: 50 mg/kg
DAY OF DEATH: 30 Test period | STATUS: Scheduled phase sacrifice # 1 | TERMINAL BODY WEIGHT (g) : 225.2

Tissue	Gross observations/comments	Correlated Microscopic Observations
GENER. CONDITION . . .	GOOD	No micropathology observations on tissue.
HARDERIAN GLANDS . . .	No gross observations on tissue	PORPHYRIN DEPOSITS, Slight, Multifocal.
LIVER	No gross observations on tissue	CHRONIC INFLAMMATION, Minimal, Multifocal.<br>HEPATOCELLULAR HYPERTROPHY,CENTRILOBULAR, Minimal.<br>HEPATOCELLULAR VACUOLATION,PERIPORTAL, Minimal.

The following tissues have no gross observations and were not examined microscopically:

ADRENALS	AORTA	BONE MARROW	BRAIN	CECUM
COLON	DIAPHRAGM	DUODENUM	ESOPHAGUS	EYES
FEMUR	HEART	ILEUM	JEJUNUM	KIDNEYS
MANDIBULAR L.N.	MESENTERIC L.N.	LUNG	MAMMARY GLAND	SKELETAL MUSCLE
SCIATIC NERVE	OPTIC NERVES	OVARIES	PANCREAS	PITUITARY
PARATHYROIDS	SPINAL CORD CERV	SPINAL CORD THOR	MANDIBULAR S.G.	PAROTIDS
STIFLE JOINT	SKIN	SPLEEN	STOMACH	STERNUM
THYROIDS	THYMUS	TONGUE	TRACHEA	URINARY BLADDER
UTERUS	VAGINA			

The following tissues have no gross observations and were marked as unremarkable microscopically:
No tissue to list.

Appendix 10
Individual Animal Microscopic vs. Gross Observations

Fexinidazole — Study Number: 0504-2007

ANIMAL: 2804 — SEX: Female — GROUP: 2 — DOSE LEVEL: 50 mg/kg
DAY OF DEATH: 30 Test period — STATUS: Scheduled phase sacrifice # 1 — TERMINAL BODY WEIGHT (g) : 209.9

Tissue	Gross observations/comments	Correlated Microscopic Observations
GENER. CONDITION . . .	GOOD	No micropathology observations on tissue.
HARDERIAN GLANDS . . .	No gross observations on tissue	PORPHYRIN DEPOSITS, Slight, Multifocal.
LIVER	No gross observations on tissue	CHRONIC INFLAMMATION, Minimal, Multifocal.<br>HEPATOCELLULAR VACUOLATION,PERIPORTAL, Slight.

The following tissues have no gross observations and were not examined microscopically:

ADRENALS	AORTA	BONE MARROW	BRAIN	CECUM
COLON	DIAPHRAGM	DUODENUM	ESOPHAGUS	EYES
FEMUR	HEART	ILEUM	JEJUNUM	KIDNEYS
MANDIBULAR L.N.	MESENTERIC L.N.	LUNG	MAMMARY GLAND	SKELETAL MUSCLE
SCIATIC NERVE	OPTIC NERVES	OVARIES	PANCREAS	PITUITARY
PARATHYROIDS	SPINAL CORD CERV	SPINAL CORD THOR	MANDIBULAR S.G.	PAROTIDS
STIFLE JOINT	SKIN	SPLEEN	STOMACH	STERNUM
THYROIDS	THYMUS	TONGUE	TRACHEA	URINARY BLADDER
UTERUS	VAGINA			

The following tissues have no gross observations and were marked as unremarkable microscopically:
No tissue to list.

Appendix 10
Individual Animal Microscopic vs. Gross Observations

Fexinidazole | Study Number: 0504-2007

ANIMAL: 2805 | SEX: Female | GROUP: 2 | DOSE LEVEL: 50 mg/kg
DAY OF DEATH: 29 Test period | STATUS: Scheduled phase sacrifice # 1 | TERMINAL BODY WEIGHT (g) : 233.0

Tissue	Gross observations/comments	Correlated Microscopic Observations
GENER. CONDITION . . .	GOOD	No micropathology observations on tissue.
HARDERIAN GLANDS . . .	No gross observations on tissue	PORPHYRIN DEPOSITS, Slight, Multifocal.<br>HEMORRHAGE,UNILATERAL, Slight, Multifocal.<br>LYMPHOCYTIC INFILTRATION, Minimal, Focal, Unilateral..
LIVER	No gross observations on tissue	CHRONIC INFLAMMATION, Minimal, Multifocal.<br>HEPATOCELLULAR HYPERTROPHY,CENTRILOBULAR, Minimal.<br>HEPATOCELLULAR VACUOLATION,PERIPORTAL, Minimal.

The following tissues have no gross observations and were not examined microscopically:

ADRENALS	AORTA	BONE MARROW	BRAIN	CECUM
COLON	DIAPHRAGM	DUODENUM	ESOPHAGUS	EYES
FEMUR	HEART	ILEUM	JEJUNUM	KIDNEYS
MANDIBULAR L.N.	MESENTERIC L.N.	LUNG	MAMMARY GLAND	SKELETAL MUSCLE
SCIATIC NERVE	OPTIC NERVES	OVARIES	PANCREAS	PITUITARY
PARATHYROIDS	SPINAL CORD CERV	SPINAL CORD THOR	MANDIBULAR S.G.	PAROTIDS
STIFLE JOINT	SKIN	SPLEEN	STOMACH	STERNUM
THYROIDS	THYMUS	TONGUE	TRACHEA	URINARY BLADDER
UTERUS	VAGINA			

The following tissues have no gross observations and were marked as unremarkable microscopically:
No tissue to list.

Appendix 10
Individual Animal Microscopic vs. Gross Observations

Fexinidazole — Study Number: 0504-2007

ANIMAL: 2806 — SEX: Female — GROUP: 2 — DOSE LEVEL: 50 mg/kg
DAY OF DEATH: 29 Test period — STATUS: Scheduled phase sacrifice # 1 — TERMINAL BODY WEIGHT (g) : 220.3

Tissue	Gross observations/comments	Correlated Microscopic Observations
GENER. CONDITION . . .	GOOD	No micropathology observations on tissue.
HARDERIAN GLANDS . . .	No gross observations on tissue	PORPHYRIN DEPOSITS, Minimal, Multifocal.<br>HEMORRHAGE,UNILATERAL, Slight, Multifocal.<br>ADENITIS,UNILATERAL, Slight, Multifocal, Acute.
LIVER	No gross observations on tissue	CHRONIC INFLAMMATION, Minimal, Multifocal.<br>HEPATOCELLULAR HYPERTROPHY,CENTRILOBULAR, Minimal.<br>HEPATOCELLULAR VACUOLATION,PERIPORTAL, Minimal.

The following tissues have no gross observations and were not examined microscopically:

ADRENALS	AORTA	BONE MARROW	BRAIN	CECUM
COLON	DIAPHRAGM	DUODENUM	ESOPHAGUS	EYES
FEMUR	HEART	ILEUM	JEJUNUM	KIDNEYS
MANDIBULAR L.N.	MESENTERIC L.N.	LUNG	MAMMARY GLAND	SKELETAL MUSCLE
SCIATIC NERVE	OPTIC NERVES	OVARIES	PANCREAS	PITUITARY
PARATHYROIDS	SPINAL CORD CERV	SPINAL CORD THOR	MANDIBULAR S.G.	PAROTIDS
STIFLE JOINT	SKIN	SPLEEN	STOMACH	STERNUM
THYROIDS	THYMUS	TONGUE	TRACHEA	URINARY BLADDER
UTERUS	VAGINA			

The following tissues have no gross observations and were marked as unremarkable microscopically:

No tissue to list.

Appendix 10
Individual Animal Microscopic vs. Gross Observations

Fexinidazole | Study Number: 0504-2007

ANIMAL: 2807 | SEX: Female | GROUP: 2 | DOSE LEVEL: 50 mg/kg
DAY OF DEATH: 29 Test period | STATUS: Scheduled phase sacrifice # 1 | TERMINAL BODY WEIGHT (g) : 220.5

Tissue	Gross observations/comments	Correlated Microscopic Observations
GENER. CONDITION . . .	GOOD	No micropathology observations on tissue.
HARDERIAN GLANDS . . .	No gross observations on tissue	PORPHYRIN DEPOSITS, Slight, Multifocal.
LIVER	No gross observations on tissue	CHRONIC INFLAMMATION, Minimal, Multifocal.<br>HEPATOCELLULAR VACUOLATION,PERIPORTAL, Slight.

The following tissues have no gross observations and were not examined microscopically:

ADRENALS	AORTA	BONE MARROW	BRAIN	CECUM
COLON	DIAPHRAGM	DUODENUM	ESOPHAGUS	EYES
FEMUR	HEART	ILEUM	JEJUNUM	KIDNEYS
MANDIBULAR L.N.	MESENTERIC L.N.	LUNG	MAMMARY GLAND	SKELETAL MUSCLE
SCIATIC NERVE	OPTIC NERVES	OVARIES	PANCREAS	PITUITARY
PARATHYROIDS	SPINAL CORD CERV	SPINAL CORD THOR	MANDIBULAR S.G.	PAROTIDS
STIFLE JOINT	SKIN	SPLEEN	STOMACH	STERNUM
THYROIDS	THYMUS	TONGUE	TRACHEA	URINARY BLADDER
UTERUS	VAGINA			

The following tissues have no gross observations and were marked as unremarkable microscopically:
No tissue to list.

Appendix 10
Individual Animal Microscopic vs. Gross Observations

Fexinidazole | Study Number: 0504-2007

ANIMAL: 2808 | SEX: Female | GROUP: 2 | DOSE LEVEL: 50 mg/kg
DAY OF DEATH: 29 Test period | STATUS: Scheduled phase sacrifice # 1 | TERMINAL BODY WEIGHT (g) : 230.6

Tissue	Gross observations/comments	Correlated Microscopic Observations
GENER. CONDITION . . .	GOOD	No micropathology observations on tissue.
HARDERIAN GLANDS . . .	No gross observations on tissue	PORPHYRIN DEPOSITS, Moderate, Multifocal.<br>HEMORRHAGE,UNILATERAL, Slight, Multifocal.<br>ADENITIS,UNILATERAL, Slight, Focal, Acute.
LIVER	No gross observations on tissue	CHRONIC INFLAMMATION, Minimal, Multifocal.<br>HEPATOCELLULAR HYPERTROPHY,CENTRILOBULAR, Minimal.<br>HEPATOCELLULAR VACUOLATION,PERIPORTAL, Slight.

The following tissues have no gross observations and were not examined microscopically:

ADRENALS	AORTA	BONE MARROW	BRAIN	CECUM
COLON	DIAPHRAGM	DUODENUM	ESOPHAGUS	EYES
FEMUR	HEART	ILEUM	JEJUNUM	KIDNEYS
MANDIBULAR L.N.	MESENTERIC L.N.	LUNG	MAMMARY GLAND	SKELETAL MUSCLE
SCIATIC NERVE	OPTIC NERVES	OVARIES	PANCREAS	PITUITARY
PARATHYROIDS	SPINAL CORD CERV	SPINAL CORD THOR	MANDIBULAR S.G.	PAROTIDS
STIFLE JOINT	SKIN	SPLEEN	STOMACH	STERNUM
THYROIDS	THYMUS	TONGUE	TRACHEA	URINARY BLADDER
UTERUS	VAGINA			

The following tissues have no gross observations and were marked as unremarkable microscopically:
No tissue to list.

Appendix 10
Individual Animal Microscopic vs. Gross Observations

Fexinidazole — Study Number: 0504-2007

ANIMAL: 2809 — SEX: Female — GROUP: 2 — DOSE LEVEL: 50 mg/kg
DAY OF DEATH: 29 Test period — STATUS: Scheduled phase sacrifice # 1 — TERMINAL BODY WEIGHT (g) : 238.7

Tissue	Gross observations/comments	Correlated Microscopic Observations
GENER. CONDITION . . .	GOOD	No micropathology observations on tissue.
HARDERIAN GLANDS . . .	No gross observations on tissue	PORPHYRIN DEPOSITS, Slight, Multifocal.<br>HEMORRHAGE,UNILATERAL, Slight, Multifocal.<br>ADENITIS,UNILATERAL, Moderate, Focal, Acute.
LIVER	No gross observations on tissue	CHRONIC INFLAMMATION, Minimal, Multifocal.<br>HEPATOCELLULAR VACUOLATION,PERIPORTAL, Minimal.

The following tissues have no gross observations and were not examined microscopically:

ADRENALS	AORTA	BONE MARROW	BRAIN	CECUM
COLON	DIAPHRAGM	DUODENUM	ESOPHAGUS	EYES
FEMUR	HEART	ILEUM	JEJUNUM	KIDNEYS
MANDIBULAR L.N.	MESENTERIC L.N.	LUNG	MAMMARY GLAND	SKELETAL MUSCLE
SCIATIC NERVE	OPTIC NERVES	OVARIES	PANCREAS	PITUITARY
PARATHYROIDS	SPINAL CORD CERV	SPINAL CORD THOR	MANDIBULAR S.G.	PAROTIDS
STIFLE JOINT	SKIN	SPLEEN	STOMACH	STERNUM
THYROIDS	THYMUS	TONGUE	TRACHEA	URINARY BLADDER
UTERUS	VAGINA			

The following tissues have no gross observations and were marked as unremarkable microscopically:
No tissue to list.

Appendix 10
Individual Animal Microscopic vs. Gross Observations

Fexinidazole | Study Number: 0504-2007

ANIMAL: 2810 | SEX: Female | GROUP: 3 | DOSE LEVEL: 200 mg/kg
DAY OF DEATH: 30 Test period | STATUS: Scheduled phase sacrifice # 1 | TERMINAL BODY WEIGHT (g) : 218.2

Tissue	Gross observations/comments	Correlated Microscopic Observations
GENER. CONDITION . . .	GOOD	No micropathology observations on tissue.
HARDERIAN GLANDS . . .	No gross observations on tissue	PORPHYRIN DEPOSITS, Moderate, Multifocal.
LIVER	No gross observations on tissue	CHRONIC INFLAMMATION, Minimal, Multifocal.
		HEPATOCELLULAR HYPERTROPHY,CENTRILOBULAR, Minimal.
		HEPATOCELLULAR VACUOLATION,PERIPORTAL, Minimal.

The following tissues have no gross observations and were not examined microscopically:

ADRENALS	AORTA	BONE MARROW	BRAIN	CECUM
COLON	DIAPHRAGM	DUODENUM	ESOPHAGUS	EYES
FEMUR	HEART	ILEUM	JEJUNUM	KIDNEYS
MANDIBULAR L.N.	MESENTERIC L.N.	LUNG	MAMMARY GLAND	SKELETAL MUSCLE
SCIATIC NERVE	OPTIC NERVES	OVARIES	PANCREAS	PITUITARY
PARATHYROIDS	SPINAL CORD CERV	SPINAL CORD THOR	MANDIBULAR S.G.	PAROTIDS
STIFLE JOINT	SKIN	SPLEEN	STOMACH	STERNUM
THYROIDS	THYMUS	TONGUE	TRACHEA	URINARY BLADDER
UTERUS	VAGINA			

The following tissues have no gross observations and were marked as unremarkable microscopically:
No tissue to list.

Appendix 10
Individual Animal Microscopic vs. Gross Observations

Fexinidazole — Study Number: 0504-2007

ANIMAL: 2811 — SEX: Female — GROUP: 3 — DOSE LEVEL: 200 mg/kg
DAY OF DEATH: 30 Test period — STATUS: Scheduled phase sacrifice # 1 — TERMINAL BODY WEIGHT (g) : 237.9

Tissue	Gross observations/comments	Correlated Microscopic Observations
GENER. CONDITION . . .	GOOD	No micropathology observations on tissue.
HARDERIAN GLANDS . . .	No gross observations on tissue	PORPHYRIN DEPOSITS, Slight, Multifocal.
LIVER	No gross observations on tissue	CHRONIC INFLAMMATION, Minimal, Multifocal.<br>HEPATOCELLULAR HYPERTROPHY,CENTRILOBULAR, Minimal.<br>HEPATOCELLULAR VACUOLATION,PERIPORTAL, Slight.
PLEURAL CAVITY	ABNORMAL CONTENTS/ clear liquid.	No micropathology observations on tissue.

The following tissues have no gross observations and were not examined microscopically:

ADRENALS	AORTA	BONE MARROW	BRAIN	CECUM
COLON	DIAPHRAGM	DUODENUM	ESOPHAGUS	EYES
FEMUR	HEART	ILEUM	JEJUNUM	KIDNEYS
MANDIBULAR L.N.	MESENTERIC L.N.	LUNG	MAMMARY GLAND	SKELETAL MUSCLE
SCIATIC NERVE	OPTIC NERVES	OVARIES	PANCREAS	PITUITARY
PARATHYROIDS	SPINAL CORD CERV	SPINAL CORD THOR	MANDIBULAR S.G.	PAROTIDS
STIFLE JOINT	SKIN	SPLEEN	STOMACH	STERNUM
THYROIDS	THYMUS	TONGUE	TRACHEA	URINARY BLADDER
UTERUS	VAGINA			

The following tissues have no gross observations and were marked as unremarkable microscopically:
No tissue to list.

Appendix 10
Individual Animal Microscopic vs. Gross Observations

Fexinidazole   Study Number: 0504-2007

ANIMAL: 2812   SEX: Female   GROUP: 3   DOSE LEVEL: 200 mg/kg
DAY OF DEATH: 30 Test period   STATUS: Scheduled phase sacrifice # 1   TERMINAL BODY WEIGHT (g) : 235.2

Tissue	Gross observations/comments	Correlated Microscopic Observations
GENER. CONDITION . . .	GOOD	No micropathology observations on tissue.
HARDERIAN GLANDS . . .	No gross observations on tissue	PORPHYRIN DEPOSITS, Slight, Multifocal.
LIVER	No gross observations on tissue	CHRONIC INFLAMMATION, Minimal, Multifocal.<br>HEPATOCELLULAR HYPERTROPHY,CENTRILOBULAR, Minimal.

The following tissues have no gross observations and were not examined microscopically:

ADRENALS	AORTA	BONE MARROW	BRAIN	CECUM
COLON	DIAPHRAGM	DUODENUM	ESOPHAGUS	EYES
FEMUR	HEART	ILEUM	JEJUNUM	KIDNEYS
MANDIBULAR L.N.	MESENTERIC L.N.	LUNG	MAMMARY GLAND	SKELETAL MUSCLE
SCIATIC NERVE	OPTIC NERVES	OVARIES	PANCREAS	PITUITARY
PARATHYROIDS	SPINAL CORD CERV	SPINAL CORD THOR	MANDIBULAR S.G.	PAROTIDS
STIFLE JOINT	SKIN	SPLEEN	STOMACH	STERNUM
THYROIDS	THYMUS	TONGUE	TRACHEA	URINARY BLADDER
UTERUS	VAGINA			

The following tissues have no gross observations and were marked as unremarkable microscopically:
No tissue to list.

Appendix 10
Individual Animal Microscopic vs. Gross Observations

Fexinidazole — Study Number: 0504-2007

ANIMAL: 2813 — SEX: Female — GROUP: 3 — DOSE LEVEL: 200 mg/kg
DAY OF DEATH: 30 Test period — STATUS: Scheduled phase sacrifice # 1 — TERMINAL BODY WEIGHT (g) : 240.0

Tissue	Gross observations/comments	Correlated Microscopic Observations
GENER. CONDITION . . .	GOOD	No micropathology observations on tissue.
HARDERIAN GLANDS . . .	No gross observations on tissue	PORPHYRIN DEPOSITS, Moderate, Multifocal.
LIVER	No gross observations on tissue	CHRONIC INFLAMMATION, Minimal, Multifocal.<br>HEPATOCELLULAR HYPERTROPHY,CENTRILOBULAR, Minimal.<br>HEPATOCELLULAR VACUOLATION,PERIPORTAL, Slight.

The following tissues have no gross observations and were not examined microscopically:

ADRENALS	AORTA	BONE MARROW	BRAIN	CECUM
COLON	DIAPHRAGM	DUODENUM	ESOPHAGUS	EYES
FEMUR	HEART	ILEUM	JEJUNUM	KIDNEYS
MANDIBULAR L.N.	MESENTERIC L.N.	LUNG	MAMMARY GLAND	SKELETAL MUSCLE
SCIATIC NERVE	OPTIC NERVES	OVARIES	PANCREAS	PITUITARY
PARATHYROIDS	SPINAL CORD CERV	SPINAL CORD THOR	MANDIBULAR S.G.	PAROTIDS
STIFLE JOINT	SKIN	SPLEEN	STOMACH	STERNUM
THYROIDS	THYMUS	TONGUE	TRACHEA	URINARY BLADDER
UTERUS	VAGINA			

The following tissues have no gross observations and were marked as unremarkable microscopically:
No tissue to list.

Appendix 10
Individual Animal Microscopic vs. Gross Observations

Fexinidazole | Study Number: 0504-2007

ANIMAL: 2814 | SEX: Female | GROUP: 3 | DOSE LEVEL: 200 mg/kg
DAY OF DEATH: 30 Test period | STATUS: Scheduled phase sacrifice # 1 | TERMINAL BODY WEIGHT (g) : 213.0

Tissue	Gross observations/comments	Correlated Microscopic Observations
GENER. CONDITION . . .	GOOD	No micropathology observations on tissue.
HARDERIAN GLANDS . . .	No gross observations on tissue	PORPHYRIN DEPOSITS, Slight, Multifocal.
LIVER	No gross observations on tissue	CHRONIC INFLAMMATION, Minimal, Multifocal.<br>HEPATOCELLULAR HYPERTROPHY,CENTRILOBULAR, Minimal.<br>HEPATOCELLULAR VACUOLATION,PERIPORTAL, Minimal.
SKIN	ENCRUSTED AREA(S), single/ in the left cheek, 3 mm in diameter.	Tissue is unremarkable.

The following tissues have no gross observations and were not examined microscopically:

ADRENALS	AORTA	BONE MARROW	BRAIN	CECUM
COLON	DIAPHRAGM	DUODENUM	ESOPHAGUS	EYES
FEMUR	HEART	ILEUM	JEJUNUM	KIDNEYS
MANDIBULAR L.N.	MESENTERIC L.N.	LUNG	MAMMARY GLAND	SKELETAL MUSCLE
SCIATIC NERVE	OPTIC NERVES	OVARIES	PANCREAS	PITUITARY
PARATHYROIDS	SPINAL CORD CERV	SPINAL CORD THOR	MANDIBULAR S.G.	PAROTIDS
STIFLE JOINT	SPLEEN	STOMACH	STERNUM	THYROIDS
THYMUS	TONGUE	TRACHEA	URINARY BLADDER	UTERUS
VAGINA				

The following tissues have no gross observations and were marked as unremarkable microscopically:
No tissue to list.

Appendix 10
Individual Animal Microscopic vs. Gross Observations

Fexinidazole   Study Number: 0504-2007

ANIMAL: 2815   SEX: Female   GROUP: 3   DOSE LEVEL: 200 mg/kg
DAY OF DEATH: 29 Test period   STATUS: Scheduled phase sacrifice # 1   TERMINAL BODY WEIGHT (g) : 219.2

Tissue	Gross observations/comments	Correlated Microscopic Observations
GENER. CONDITION . . .	GOOD	No micropathology observations on tissue.
HARDERIAN GLANDS . . .	No gross observations on tissue	PORPHYRIN DEPOSITS, Moderate, Multifocal.
		HEMORRHAGE,UNILATERAL, Slight, Focal.
LIVER	No gross observations on tissue	CHRONIC INFLAMMATION, Minimal, Multifocal.
		HEPATOCELLULAR HYPERTROPHY,CENTRILOBULAR, Slight.
		HEPATOCELLULAR VACUOLATION,PERIPORTAL, Slight.

The following tissues have no gross observations and were not examined microscopically:

ADRENALS	AORTA	BONE MARROW	BRAIN	CECUM
COLON	DIAPHRAGM	DUODENUM	ESOPHAGUS	EYES
FEMUR	HEART	ILEUM	JEJUNUM	KIDNEYS
MANDIBULAR L.N.	MESENTERIC L.N.	LUNG	MAMMARY GLAND	SKELETAL MUSCLE
SCIATIC NERVE	OPTIC NERVES	OVARIES	PANCREAS	PITUITARY
PARATHYROIDS	SPINAL CORD CERV	SPINAL CORD THOR	MANDIBULAR S.G.	PAROTIDS
STIFLE JOINT	SKIN	SPLEEN	STOMACH	STERNUM
THYROIDS	THYMUS	TONGUE	TRACHEA	URINARY BLADDER
UTERUS	VAGINA			

The following tissues have no gross observations and were marked as unremarkable microscopically:
No tissue to list.

Appendix 10
Individual Animal Microscopic vs. Gross Observations

Fexinidazole | Study Number: 0504-2007

ANIMAL: 2816 | SEX: Female | GROUP: 3 | DOSE LEVEL: 200 mg/kg
DAY OF DEATH: 29 Test period | STATUS: Scheduled phase sacrifice # 1 | TERMINAL BODY WEIGHT (g) : 231.5

Tissue	Gross observations/comments	Correlated Microscopic Observations
GENER. CONDITION . . .	GOOD	No micropathology observations on tissue.
HARDERIAN GLANDS . . .	No gross observations on tissue	PORPHYRIN DEPOSITS, Moderate, Multifocal.<br>HEMORRHAGE,UNILATERAL, Slight, Focal.<br>LYMPHOCYTIC INFILTRATION, Minimal, Multifocal, Unilateral..
LIVER	No gross observations on tissue	CHRONIC INFLAMMATION, Minimal, Multifocal.<br>HEPATOCELLULAR HYPERTROPHY,CENTRILOBULAR, Minimal.<br>HEPATOCELLULAR VACUOLATION,PERIPORTAL, Minimal.

The following tissues have no gross observations and were not examined microscopically:

ADRENALS	AORTA	BONE MARROW	BRAIN	CECUM
COLON	DIAPHRAGM	DUODENUM	ESOPHAGUS	EYES
FEMUR	HEART	ILEUM	JEJUNUM	KIDNEYS
MANDIBULAR L.N.	MESENTERIC L.N.	LUNG	MAMMARY GLAND	SKELETAL MUSCLE
SCIATIC NERVE	OPTIC NERVES	OVARIES	PANCREAS	PITUITARY
PARATHYROIDS	SPINAL CORD CERV	SPINAL CORD THOR	MANDIBULAR S.G.	PAROTIDS
STIFLE JOINT	SKIN	SPLEEN	STOMACH	STERNUM
THYROIDS	THYMUS	TONGUE	TRACHEA	URINARY BLADDER
UTERUS	VAGINA			

The following tissues have no gross observations and were marked as unremarkable microscopically:
No tissue to list.

Appendix 10
Individual Animal Microscopic vs. Gross Observations

Fexinidazole Study Number: 0504-2007

ANIMAL: 2817 SEX: Female GROUP: 3 DOSE LEVEL: 200 mg/kg
DAY OF DEATH: 29 Test period STATUS: Scheduled phase sacrifice # 1 TERMINAL BODY WEIGHT (g) : 238.1

Tissue	Gross observations/comments	Correlated Microscopic Observations
GENER. CONDITION . . .	GOOD	No micropathology observations on tissue.
HARDERIAN GLANDS . . .	No gross observations on tissue	PORPHYRIN DEPOSITS, Slight, Multifocal.<br>ADENITIS,UNILATERAL, Slight, Focal, Acute.
LIVER	No gross observations on tissue	CHRONIC INFLAMMATION, Minimal, Multifocal.<br>HEPATOCELLULAR HYPERTROPHY,CENTRILOBULAR, Minimal.<br>HEPATOCELLULAR VACUOLATION,PERIPORTAL, Minimal.<br>FOCAL NECROSIS,SUBCAPSULAR, Minimal. / with associated acute inflammation

The following tissues have no gross observations and were not examined microscopically:

ADRENALS	AORTA	BONE MARROW	BRAIN	CECUM
COLON	DIAPHRAGM	DUODENUM	ESOPHAGUS	EYES
FEMUR	HEART	ILEUM	JEJUNUM	KIDNEYS
MANDIBULAR L.N.	MESENTERIC L.N.	LUNG	MAMMARY GLAND	SKELETAL MUSCLE
SCIATIC NERVE	OPTIC NERVES	OVARIES	PANCREAS	PITUITARY
PARATHYROIDS	SPINAL CORD CERV	SPINAL CORD THOR	MANDIBULAR S.G.	PAROTIDS
STIFLE JOINT	SKIN	SPLEEN	STOMACH	STERNUM
THYROIDS	THYMUS	TONGUE	TRACHEA	URINARY BLADDER
UTERUS	VAGINA			

The following tissues have no gross observations and were marked as unremarkable microscopically:
No tissue to list.

Appendix 10
Individual Animal Microscopic vs. Gross Observations

Fexinidazole Study Number: 0504-2007

ANIMAL: 2818 SEX: Female GROUP: 3 DOSE LEVEL: 200 mg/kg
DAY OF DEATH: 29 Test period STATUS: Scheduled phase sacrifice # 1 TERMINAL BODY WEIGHT (g) : 233.2

Tissue	Gross observations/comments	Correlated Microscopic Observations
GENER. CONDITION . . .	GOOD	No micropathology observations on tissue.
HARDERIAN GLANDS . . .	No gross observations on tissue	PORPHYRIN DEPOSITS, Moderate, Multifocal.<br>HEMORRHAGE,UNILATERAL, Slight, Multifocal.
LIVER	No gross observations on tissue	CHRONIC INFLAMMATION, Minimal, Multifocal.<br>HEPATOCELLULAR HYPERTROPHY,CENTRILOBULAR, Minimal.<br>HEPATOCELLULAR VACUOLATION,PERIPORTAL, Slight.

The following tissues have no gross observations and were not examined microscopically:

ADRENALS	AORTA	BONE MARROW	BRAIN	CECUM
COLON	DIAPHRAGM	DUODENUM	ESOPHAGUS	EYES
FEMUR	HEART	ILEUM	JEJUNUM	KIDNEYS
MANDIBULAR L.N.	MESENTERIC L.N.	LUNG	MAMMARY GLAND	SKELETAL MUSCLE
SCIATIC NERVE	OPTIC NERVES	OVARIES	PANCREAS	PITUITARY
PARATHYROIDS	SPINAL CORD CERV	SPINAL CORD THOR	MANDIBULAR S.G.	PAROTIDS
STIFLE JOINT	SKIN	SPLEEN	STOMACH	STERNUM
THYROIDS	THYMUS	TONGUE	TRACHEA	URINARY BLADDER
UTERUS	VAGINA			

The following tissues have no gross observations and were marked as unremarkable microscopically:
No tissue to list.

Appendix 10
Individual Animal Microscopic vs. Gross Observations

Fexinidazole | Study Number: 0504-2007

ANIMAL: 2819 | SEX: Female | GROUP: 3 | DOSE LEVEL: 200 mg/kg
DAY OF DEATH: 29 Test period | STATUS: Scheduled phase sacrifice # 1 | TERMINAL BODY WEIGHT (g) : 221.9

Tissue	Gross observations/comments	Correlated Microscopic Observations
GENER. CONDITION . . .	GOOD	No micropathology observations on tissue.
HARDERIAN GLANDS . . .	No gross observations on tissue	PORPHYRIN DEPOSITS, Slight, Multifocal.<br>HEMORRHAGE,UNILATERAL, Slight, Multifocal.
LIVER	No gross observations on tissue	CHRONIC INFLAMMATION, Minimal, Multifocal.<br>HEPATOCELLULAR HYPERTROPHY,CENTRILOBULAR, Minimal.<br>HEPATOCELLULAR VACUOLATION,PERIPORTAL, Slight.

The following tissues have no gross observations and were not examined microscopically:

ADRENALS	AORTA	BONE MARROW	BRAIN	CECUM
COLON	DIAPHRAGM	DUODENUM	ESOPHAGUS	EYES
FEMUR	HEART	ILEUM	JEJUNUM	KIDNEYS
MANDIBULAR L.N.	MESENTERIC L.N.	LUNG	MAMMARY GLAND	SKELETAL MUSCLE
SCIATIC NERVE	OPTIC NERVES	OVARIES	PANCREAS	PITUITARY
PARATHYROIDS	SPINAL CORD CERV	SPINAL CORD THOR	MANDIBULAR S.G.	PAROTIDS
STIFLE JOINT	SKIN	SPLEEN	STOMACH	STERNUM
THYROIDS	THYMUS	TONGUE	TRACHEA	URINARY BLADDER
UTERUS	VAGINA			

The following tissues have no gross observations and were marked as unremarkable microscopically:
No tissue to list.

Appendix 10
Individual Animal Microscopic vs. Gross Observations

Fexinidazole | Study Number: 0504-2007

ANIMAL: 2820 | SEX: Female | GROUP: 4 | DOSE LEVEL: 800 mg/kg
DAY OF DEATH: 30 Test period | STATUS: Scheduled phase sacrifice # 1 | TERMINAL BODY WEIGHT (g) : 207.9

Tissue	Gross observations/comments	Correlated Microscopic Observations
GENER. CONDITION . . .	GOOD	No micropathology observations on tissue.
HARDERIAN GLANDS . . .	No gross observations on tissue	PORPHYRIN DEPOSITS, Slight, Multifocal.
LIVER	No gross observations on tissue	CHRONIC INFLAMMATION, Minimal, Multifocal.<br>HEPATOCELLULAR HYPERTROPHY,CENTRILOBULAR, Slight.
MANDIBULAR L.N.	No gross observations on tissue	PLASMACYTOSIS, Moderate, Diffuse.
PARATHYROIDS	No gross observations on tissue	ONLY ONE PARATHYROID AVAILABLE FOR EXAMINATION, Present.

The following tissues have no gross observations and were not examined microscopically:
No tissue to list.

The following tissues have no gross observations and were marked as unremarkable microscopically:

ADRENALS	AORTA	BONE MARROW	BRAIN	CECUM
COLON	DIAPHRAGM	DUODENUM	ESOPHAGUS	EYES
FEMUR	HEART	ILEUM	JEJUNUM	KIDNEYS
MESENTERIC L.N.	LUNG	MAMMARY GLAND	SKELETAL MUSCLE	SCIATIC NERVE
OPTIC NERVES	OVARIES	PANCREAS	PITUITARY	SPINAL CORD CERV
SPINAL CORD THOR	MANDIBULAR S.G.	PAROTIDS	STIFLE JOINT	SKIN
SPLEEN	STOMACH	STERNUM	THYROIDS	THYMUS
TONGUE	TRACHEA	URINARY BLADDER	UTERUS	VAGINA

Appendix 10
Individual Animal Microscopic vs. Gross Observations

Fexinidazole | Study Number: 0504-2007

ANIMAL: 2821 | SEX: Female | GROUP: 4 | DOSE LEVEL: 800 mg/kg
DAY OF DEATH: 30 Test period | STATUS: Scheduled phase sacrifice # 1 | TERMINAL BODY WEIGHT (g) : 195.4

Tissue	Gross observations/comments	Correlated Microscopic Observations
GENER. CONDITION . . .	GOOD	No micropathology observations on tissue.
HARDERIAN GLANDS . . .	No gross observations on tissue	PORPHYRIN DEPOSITS, Slight, Multifocal.
LIVER	No gross observations on tissue	CHRONIC INFLAMMATION, Minimal, Multifocal.
		HEPATOCELLULAR HYPERTROPHY,CENTRILOBULAR, Slight.
		HEPATOCELLULAR VACUOLATION,PERIPORTAL, Minimal.
MANDIBULAR L.N.	No gross observations on tissue	PLASMACYTOSIS, Moderate, Diffuse.
THYROIDS	No gross observations on tissue	LYMPHOCYTIC INFILTRATION, Minimal, Focal, Unilateral..

The following tissues have no gross observations and were not examined microscopically:
No tissue to list.

The following tissues have no gross observations and were marked as unremarkable microscopically:

ADRENALS	AORTA	BONE MARROW	BRAIN	CECUM
COLON	DIAPHRAGM	DUODENUM	ESOPHAGUS	EYES
FEMUR	HEART	ILEUM	JEJUNUM	KIDNEYS
MESENTERIC L.N.	LUNG	MAMMARY GLAND	SKELETAL MUSCLE	SCIATIC NERVE
OPTIC NERVES	OVARIES	PANCREAS	PITUITARY	PARATHYROIDS
SPINAL CORD CERV	SPINAL CORD THOR	MANDIBULAR S.G.	PAROTIDS	STIFLE JOINT
SKIN	SPLEEN	STOMACH	STERNUM	THYMUS
TONGUE	TRACHEA	URINARY BLADDER	UTERUS	VAGINA

Appendix 10
Individual Animal Microscopic vs. Gross Observations

Fexinidazole — Study Number: 0504-2007

ANIMAL: 2822 SEX: Female GROUP: 4 DOSE LEVEL: 800 mg/kg
DAY OF DEATH: 30 Test period STATUS: Scheduled phase sacrifice # 1 TERMINAL BODY WEIGHT (g) : 228.2

Tissue	Gross observations/comments	Correlated Microscopic Observations
GENER. CONDITION . . .	GOOD	No micropathology observations on tissue.
HARDERIAN GLANDS . . .	No gross observations on tissue	PORPHYRIN DEPOSITS, Moderate, Multifocal.
LIVER	No gross observations on tissue	CHRONIC INFLAMMATION, Minimal, Multifocal.<br>HEPATOCELLULAR HYPERTROPHY,CENTRILOBULAR, Slight.<br>HEPATOCELLULAR VACUOLATION,PERIPORTAL, Minimal.
LUNG	No gross observations on tissue	ALVEOLAR MACROPHAGE INFILTRATION, Minimal, Multifocal.
OPTIC NERVES	No gross observations on tissue	ONLY ONE NERVE IS AVAILABLE FOR EXAMINATION, Present.
SKIN	ALOPECIA/ one area in the right shoulder region, 30x20 mm.	Tissue is unremarkable.
THYROIDS	No gross observations on tissue	LYMPHOCYTIC INFILTRATION, Minimal, Focal, Unilateral..

The following tissues have no gross observations and were not examined microscopically:
No tissue to list.

Appendix 10
Individual Animal Microscopic vs. Gross Observations

Fexinidazole | Study Number: 0504-2007

ANIMAL: 2822 | SEX: Female | GROUP: 4 | DOSE LEVEL: 800 mg/kg
DAY OF DEATH: 30 Test period | STATUS: Scheduled phase sacrifice # 1 | TERMINAL BODY WEIGHT (g) : 228.2

Tissue	Gross observations/comments	Correlated Microscopic Observations

The following tissues have no gross observations and were marked as unremarkable microscopically:

ADRENALS	AORTA	BONE MARROW	BRAIN	CECUM
COLON	DIAPHRAGM	DUODENUM	ESOPHAGUS	EYES
FEMUR	HEART	ILEUM	JEJUNUM	KIDNEYS
MANDIBULAR L.N.	MESENTERIC L.N.	MAMMARY GLAND	SKELETAL MUSCLE	SCIATIC NERVE
OVARIES	PANCREAS	PITUITARY	PARATHYROIDS	SPINAL CORD CERV
SPINAL CORD THOR	MANDIBULAR S.G.	PAROTIDS	STIFLE JOINT	SPLEEN
STOMACH	STERNUM	THYMUS	TONGUE	TRACHEA
URINARY BLADDER	UTERUS	VAGINA		

Appendix 10
Individual Animal Microscopic vs. Gross Observations

Fexinidazole | Study Number: 0504-2007

ANIMAL: 2823 | SEX: Female | GROUP: 4 | DOSE LEVEL: 800 mg/kg
DAY OF DEATH: 30 Test period | STATUS: Scheduled phase sacrifice # 1 | TERMINAL BODY WEIGHT (g) : 248.6

Tissue	Gross observations/comments	Correlated Microscopic Observations
GENER. CONDITION . . .	GOOD	No micropathology observations on tissue.
HEART	No gross observations on tissue	MYOCARDIAL INFLAMMATION, Minimal, Multifocal.
HARDERIAN GLANDS . . .	No gross observations on tissue	PORPHYRIN DEPOSITS, Moderate, Multifocal.
		LYMPHOCYTIC INFILTRATION, Minimal, Focal, Unilateral..
LIVER	No gross observations on tissue	CHRONIC INFLAMMATION, Minimal, Multifocal.
		HEPATOCELLULAR HYPERTROPHY,CENTRILOBULAR, Slight.
		HEPATOCELLULAR VACUOLATION,PERIPORTAL, Slight.
MANDIBULAR L.N.	No gross observations on tissue	PLASMACYTOSIS, Moderate, Diffuse, Unilateral..
PARATHYROIDS	No gross observations on tissue	ONLY ONE PARATHYROID AVAILABLE FOR EXAMINATION, Present.

The following tissues have no gross observations and were not examined microscopically:
No tissue to list.

Appendix 10
Individual Animal Microscopic vs. Gross Observations

Fexinidazole Study Number: 0504-2007

ANIMAL: 2823 SEX: Female GROUP: 4 DOSE LEVEL: 800 mg/kg
DAY OF DEATH: 30 Test period STATUS: Scheduled phase sacrifice # 1 TERMINAL BODY WEIGHT (g) : 248.6

Tissue	Gross observations/comments	Correlated Microscopic Observations

The following tissues have no gross observations and were marked as unremarkable microscopically:

ADRENALS	AORTA	BONE MARROW	BRAIN	CECUM
COLON	DIAPHRAGM	DUODENUM	ESOPHAGUS	EYES
FEMUR	ILEUM	JEJUNUM	KIDNEYS	MESENTERIC L.N.
LUNG	MAMMARY GLAND	SKELETAL MUSCLE	SCIATIC NERVE	OPTIC NERVES
OVARIES	PANCREAS	PITUITARY	SPINAL CORD CERV	SPINAL CORD THOR
MANDIBULAR S.G.	PAROTIDS	STIFLE JOINT	SKIN	SPLEEN
STOMACH	STERNUM	THYROIDS	THYMUS	TONGUE
TRACHEA	URINARY BLADDER	UTERUS	VAGINA	

Appendix 10
Individual Animal Microscopic vs. Gross Observations

Fexinidazole | Study Number: 0504-2007

ANIMAL: 2824 | SEX: Female | GROUP: 4 | DOSE LEVEL: 800 mg/kg
DAY OF DEATH: 14 Test period | STATUS: FOUND DEAD | TERMINAL BODY WEIGHT (g) : 195.0

Tissue	Gross observations/comments	Correlated Microscopic Observations
AORTA	No gross observations on tissue	INFLAMMATION OF ADJACENT TISSUES, Moderate, Multifocal, Acute.
BONE MARROW	No gross observations on tissue	SMEAR NOT SAMPLED, Present.
DIAPHRAGM	No gross observations on tissue	MYOSITIS, Slight, Multifocal, Acute.<br>PLEURITIS, Slight, Diffuse, Acute.
GENER. CONDITION . . .	FAIRLY GOOD<br>AUTOLYTIC CHANGES	No micropathology observations on tissue.
HEART	No gross observations on tissue	PERICARDIAL INFLAMMATION, Slight, Multifocal, Acute.
JEJUNUM	No gross observations on tissue	Tissue is autolytic and unreadable.
HARDERIAN GLANDS . . .	No gross observations on tissue	PORPHYRIN DEPOSITS, Marked, Diffuse.
MANDIBULAR L.N.	No gross observations on tissue	LYMPHOID DEPLETION, Moderate, Diffuse.
MESENTERIC L.N.	No gross observations on tissue	LYMPHOID DEPLETION, Slight, Diffuse.
LUNG	No gross observations on tissue	PLEURITIS, Moderate, Multifocal, Acute.

Appendix 10
Individual Animal Microscopic vs. Gross Observations

Fexinidazole Study Number: 0504-2007

ANIMAL: 2824 SEX: Female GROUP: 4 DOSE LEVEL: 800 mg/kg
DAY OF DEATH: 14 Test period STATUS: FOUND DEAD TERMINAL BODY WEIGHT (g) : 195.0

Tissue	Gross observations/comments	Correlated Microscopic Observations
LUNG		pleuritis is mainly characterized by the presence of abundant inflammatory exudate in the pleural cavity,focally adherent to the pleura and with scant involvement of the lung parenchyma. These findings correlated with the abnormal contents,in particular with the clear liquid, noted on gross examination in the pleural cavity.
OPTIC NERVES	No gross observations on tissue	ONLY ONE NERVE IS AVAILABLE FOR EXAMINATION, Present.
PLEURAL CAVITY		Tissue is unremarkable.
	ABNORMAL CONTENTS/ clear liquid and soft yellowish material adherent to the diaphragm and lungs.	for abnormal contents noted on gross examination see "lungs".
PARATHYROIDS	No gross observations on tissue	ONLY ONE PARATHYROID AVAILABLE FOR EXAMINATION, Present.
MANDIBULAR S.G.	No gross observations on tissue	ACINAR HYPERTROPHY, Slight, Diffuse.
PAROTIDS	No gross observations on tissue	ACINAR HYPERTROPHY, Slight, Diffuse.
SPLEEN	No gross observations on tissue	LYMPHOID DEPLETION, Moderate, Diffuse.

Appendix 10
Individual Animal Microscopic vs. Gross Observations

Fexinidazole Study Number: 0504-2007

ANIMAL: 2824 SEX: Female GROUP: 4 DOSE LEVEL: 800 mg/kg
DAY OF DEATH: 14 Test period STATUS: FOUND DEAD TERMINAL BODY WEIGHT (g) : 195.0

Tissue	Gross observations/comments	Correlated Microscopic Observations
THYROIDS	No gross observations on tissue	COLLOID DEPLETION, Moderate.
THYMUS	No gross observations on tissue	LYMPHOID DEPLETION, Moderate, Diffuse.
		PLEURITIS, Moderate, Multifocal, Acute.

The following tissues have no gross observations and were not examined microscopically:
No tissue to list.

The following tissues have no gross observations and were marked as unremarkable microscopically:

ADRENALS	BRAIN	CECUM	COLON	DUODENUM
ESOPHAGUS	EYES	FEMUR	ILEUM	KIDNEYS
LIVER	MAMMARY GLAND	SKELETAL MUSCLE	SCIATIC NERVE	OVARIES
PANCREAS	PITUITARY	SPINAL CORD CERV	SPINAL CORD THOR	STIFLE JOINT
SKIN	STOMACH	STERNUM	TONGUE	TRACHEA
URINARY BLADDER	UTERUS	VAGINA		

Appendix 10
Individual Animal Microscopic vs. Gross Observations

Fexinidazole　　　　Study Number: 0504-2007

ANIMAL: 2825　　SEX: Female　　GROUP: 4　　DOSE LEVEL: 800 mg/kg
DAY OF DEATH: 29 Test period　　STATUS: Scheduled phase sacrifice # 1　　TERMINAL BODY WEIGHT (g) : 213.5

Tissue	Gross observations/comments	Correlated Microscopic Observations
GENER. CONDITION . . .	GOOD	No micropathology observations on tissue.
KIDNEYS	No gross observations on tissue	CHRONIC INFLAMMATION, Minimal, Focal, Unilateral left..
HARDERIAN GLANDS . . .	No gross observations on tissue	PORPHYRIN DEPOSITS, Moderate, Multifocal.
LIVER	No gross observations on tissue	CHRONIC INFLAMMATION, Minimal, Multifocal.
		HEPATOCELLULAR HYPERTROPHY,CENTRILOBULAR, Slight.
MANDIBULAR L.N.	No gross observations on tissue	PLASMACYTOSIS, Moderate, Diffuse, Unilateral..
OPTIC NERVES	No gross observations on tissue	ONLY ONE NERVE IS AVAILABLE FOR EXAMINATION, Present.

The following tissues have no gross observations and were not examined microscopically:
No tissue to list.

The following tissues have no gross observations and were marked as unremarkable microscopically:

ADRENALS	AORTA	BONE MARROW	BRAIN	CECUM
COLON	DIAPHRAGM	DUODENUM	ESOPHAGUS	EYES
FEMUR	HEART	ILEUM	JEJUNUM	MESENTERIC L.N.
LUNG	MAMMARY GLAND	SKELETAL MUSCLE	SCIATIC NERVE	OVARIES
PANCREAS	PITUITARY	PARATHYROIDS	SPINAL CORD CERV	SPINAL CORD THOR
MANDIBULAR S.G.	PAROTIDS	STIFLE JOINT	SKIN	SPLEEN

Appendix 10
Individual Animal Microscopic vs. Gross Observations

Fexinidazole Study Number: 0504-2007

ANIMAL: 2825 SEX: Female GROUP: 4 DOSE LEVEL: 800 mg/kg
DAY OF DEATH: 29 Test period STATUS: Scheduled phase sacrifice # 1 TERMINAL BODY WEIGHT (g) : 213.5

Tissue	Gross observations/comments	Correlated Microscopic Observations

STOMACH	STERNUM	THYROIDS	THYMUS	TONGUE
TRACHEA	URINARY BLADDER	UTERUS	VAGINA	

Appendix 10
Individual Animal Microscopic vs. Gross Observations

Fexinidazole — Study Number: 0504-2007

ANIMAL: 2826 — SEX: Female — GROUP: 4 — DOSE LEVEL: 800 mg/kg
DAY OF DEATH: 29 Test period — STATUS: Scheduled phase sacrifice # 1 — TERMINAL BODY WEIGHT (g) : 212.3

Tissue	Gross observations/comments	Correlated Microscopic Observations
GENER. CONDITION . . .	GOOD	No micropathology observations on tissue.
KIDNEYS	No gross observations on tissue	CHRONIC INFLAMMATION, Minimal, Focal, Unilateral right..<br><br>MEDULLARY TUBULAR DILATATION, Moderate, Focal, Unilateral right..
HARDERIAN GLANDS . . .	No gross observations on tissue	PORPHYRIN DEPOSITS, Slight, Multifocal.
LIVER	No gross observations on tissue	CHRONIC INFLAMMATION, Minimal, Multifocal.<br><br>HEPATOCELLULAR HYPERTROPHY,CENTRILOBULAR, Slight.<br><br>HEPATOCELLULAR VACUOLATION,PERIPORTAL, Minimal.
MANDIBULAR L.N.	No gross observations on tissue	PLASMACYTOSIS, Marked, Diffuse, Unilateral..
OPTIC NERVES	No gross observations on tissue	ONLY ONE NERVE IS AVAILABLE FOR EXAMINATION, Present.
PARATHYROIDS	No gross observations on tissue	ONLY ONE PARATHYROID AVAILABLE FOR EXAMINATION, Present.

The following tissues have no gross observations and were not examined microscopically:
No tissue to list.

Appendix 10
Individual Animal Microscopic vs. Gross Observations

Fexinidazole   Study Number: 0504-2007

ANIMAL: 2826   SEX: Female   GROUP: 4   DOSE LEVEL: 800 mg/kg
DAY OF DEATH: 29 Test period   STATUS: Scheduled phase sacrifice # 1   TERMINAL BODY WEIGHT (g) : 212.3

Tissue	Gross observations/comments	Correlated Microscopic Observations

The following tissues have no gross observations and were marked as unremarkable microscopically:

ADRENALS	AORTA	BONE MARROW	BRAIN	CECUM
COLON	DIAPHRAGM	DUODENUM	ESOPHAGUS	EYES
FEMUR	HEART	ILEUM	JEJUNUM	MESENTERIC L.N.
LUNG	MAMMARY GLAND	SKELETAL MUSCLE	SCIATIC NERVE	OVARIES
PANCREAS	PITUITARY	SPINAL CORD CERV	SPINAL CORD THOR	MANDIBULAR S.G.
PAROTIDS	STIFLE JOINT	SKIN	SPLEEN	STOMACH
STERNUM	THYROIDS	THYMUS	TONGUE	TRACHEA
URINARY BLADDER	UTERUS	VAGINA		

Appendix 10
Individual Animal Microscopic vs. Gross Observations

Fexinidazole | Study Number: 0504-2007

ANIMAL: 2827 | SEX: Female | GROUP: 4 | DOSE LEVEL: 800 mg/kg
DAY OF DEATH: 29 Test period | STATUS: Scheduled phase sacrifice # 1 | TERMINAL BODY WEIGHT (g) : 223.5

Tissue	Gross observations/comments	Correlated Microscopic Observations
GENER. CONDITION . . .	GOOD	No micropathology observations on tissue.
HARDERIAN GLANDS . . .	No gross observations on tissue	PORPHYRIN DEPOSITS, Slight, Multifocal.
LIVER	No gross observations on tissue	CHRONIC INFLAMMATION, Minimal, Multifocal.<br>HEPATOCELLULAR HYPERTROPHY,CENTRILOBULAR, Slight.<br>HEPATOCELLULAR VACUOLATION,PERIPORTAL, Slight.
MANDIBULAR L.N.	No gross observations on tissue	PLASMACYTOSIS, Slight, Diffuse.
OPTIC NERVES	No gross observations on tissue	ONLY ONE NERVE IS AVAILABLE FOR EXAMINATION, Present.
PARATHYROIDS	No gross observations on tissue	ONLY ONE PARATHYROID AVAILABLE FOR EXAMINATION, Present.
SPLEEN	No gross observations on tissue	EXTRAMEDULLARY HEMOPOIESIS, Minimal, Multifocal.

The following tissues have no gross observations and were not examined microscopically:
No tissue to list.

Appendix 10
Individual Animal Microscopic vs. Gross Observations

Fexinidazole Study Number: 0504-2007

ANIMAL: 2827 SEX: Female GROUP: 4 DOSE LEVEL: 800 mg/kg
DAY OF DEATH: 29 Test period STATUS: Scheduled phase sacrifice # 1 TERMINAL BODY WEIGHT (g) : 223.5

Tissue	Gross observations/comments	Correlated Microscopic Observations

The following tissues have no gross observations and were marked as unremarkable microscopically:

ADRENALS	AORTA	BONE MARROW	BRAIN	CECUM
COLON	DIAPHRAGM	DUODENUM	ESOPHAGUS	EYES
FEMUR	HEART	ILEUM	JEJUNUM	KIDNEYS
MESENTERIC L.N.	LUNG	MAMMARY GLAND	SKELETAL MUSCLE	SCIATIC NERVE
OVARIES	PANCREAS	PITUITARY	SPINAL CORD CERV	SPINAL CORD THOR
MANDIBULAR S.G.	PAROTIDS	STIFLE JOINT	SKIN	STOMACH
STERNUM	THYROIDS	THYMUS	TONGUE	TRACHEA
URINARY BLADDER	UTERUS	VAGINA		

Appendix 10
Individual Animal Microscopic vs. Gross Observations

Fexinidazole Study Number: 0504-2007

ANIMAL: 2828 SEX: Female GROUP: 4 DOSE LEVEL: 800 mg/kg
DAY OF DEATH: 29 Test period STATUS: Scheduled phase sacrifice # 1 TERMINAL BODY WEIGHT (g) : 219.9

Tissue	Gross observations/comments	Correlated Microscopic Observations
GENER. CONDITION . . .	GOOD	No micropathology observations on tissue.
HARDERIAN GLANDS . . .	No gross observations on tissue	PORPHYRIN DEPOSITS, Slight, Multifocal.
LIVER	No gross observations on tissue	CHRONIC INFLAMMATION, Minimal, Multifocal.
		HEPATOCELLULAR HYPERTROPHY,CENTRILOBULAR, Slight.
		HEPATOCELLULAR VACUOLATION,PERIPORTAL, Slight.
MANDIBULAR L.N.	No gross observations on tissue	PLASMACYTOSIS, Moderate, Diffuse.
LUNG	No gross observations on tissue	ALVEOLAR MACROPHAGE INFILTRATION, Minimal, Multifocal.

The following tissues have no gross observations and were not examined microscopically:
No tissue to list.

The following tissues have no gross observations and were marked as unremarkable microscopically:

ADRENALS	AORTA	BONE MARROW	BRAIN	CECUM
COLON	DIAPHRAGM	DUODENUM	ESOPHAGUS	EYES
FEMUR	HEART	ILEUM	JEJUNUM	KIDNEYS
MESENTERIC L.N.	MAMMARY GLAND	SKELETAL MUSCLE	SCIATIC NERVE	OPTIC NERVES
OVARIES	PANCREAS	PITUITARY	PARATHYROIDS	SPINAL CORD CERV
SPINAL CORD THOR	MANDIBULAR S.G.	PAROTIDS	STIFLE JOINT	SKIN
SPLEEN	STOMACH	STERNUM	THYROIDS	THYMUS
TONGUE	TRACHEA	URINARY BLADDER	UTERUS	VAGINA

Appendix 10
Individual Animal Microscopic vs. Gross Observations

Fexinidazole — Study Number: 0504-2007

ANIMAL: 2829 SEX: Female GROUP: 4 DOSE LEVEL: 800 mg/kg
DAY OF DEATH: 29 Test period STATUS: Scheduled phase sacrifice # 1 TERMINAL BODY WEIGHT (g) : 220.0

Tissue	Gross observations/comments	Correlated Microscopic Observations
GENER. CONDITION . . .	GOOD	No micropathology observations on tissue.
KIDNEYS	No gross observations on tissue	CHRONIC INFLAMMATION, Minimal, Multifocal, Unilateral right..<br>CORTICAL TUBULAR REGENERATIVE BASOPHILIA, Minimal, Multifocal.
HARDERIAN GLANDS . . .	No gross observations on tissue	PORPHYRIN DEPOSITS, Slight, Multifocal.
LIVER	No gross observations on tissue	CHRONIC INFLAMMATION, Minimal, Multifocal.<br>HEPATOCELLULAR HYPERTROPHY,CENTRILOBULAR, Slight.<br>HEPATOCELLULAR VACUOLATION,PERIPORTAL, Slight.
MANDIBULAR L.N.	No gross observations on tissue	PLASMACYTOSIS, Moderate, Diffuse.
LUNG	No gross observations on tissue	ALVEOLAR MACROPHAGE INFILTRATION, Minimal, Multifocal.
OPTIC NERVES	No gross observations on tissue	ONLY ONE NERVE IS AVAILABLE FOR EXAMINATION, Present.

The following tissues have no gross observations and were not examined microscopically:
No tissue to list.

Appendix 10
Individual Animal Microscopic vs. Gross Observations

Fexinidazole Study Number: 0504-2007

ANIMAL: 2829 SEX: Female GROUP: 4 DOSE LEVEL: 800 mg/kg
DAY OF DEATH: 29 Test period STATUS: Scheduled phase sacrifice # 1 TERMINAL BODY WEIGHT (g) : 220.0

Tissue	Gross observations/comments	Correlated Microscopic Observations

The following tissues have no gross observations and were marked as unremarkable microscopically:

ADRENALS	AORTA	BONE MARROW	BRAIN	CECUM
COLON	DIAPHRAGM	DUODENUM	ESOPHAGUS	EYES
FEMUR	HEART	ILEUM	JEJUNUM	MESENTERIC L.N.
MAMMARY GLAND	SKELETAL MUSCLE	SCIATIC NERVE	OVARIES	PANCREAS
PITUITARY	PARATHYROIDS	SPINAL CORD CERV	SPINAL CORD THOR	MANDIBULAR S.G.
PAROTIDS	STIFLE JOINT	SKIN	SPLEEN	STOMACH
STERNUM	THYROIDS	THYMUS	TONGUE	TRACHEA
URINARY BLADDER	UTERUS	VAGINA		

Appendix 10
Individual Animal Microscopic vs. Gross Observations

Fexinidazole | Study Number: 0504-2007

ANIMAL: 2830 | SEX: Female | GROUP: 4 | DOSE LEVEL: 800 mg/kg
DAY OF DEATH: 43 Test period | STATUS: Final phase sacrifice | TERMINAL BODY WEIGHT (g) : 245.0

Tissue	Gross observations/comments	Correlated Microscopic Observations
GENER. CONDITION . . .	GOOD	No micropathology observations on tissue.
HARDERIAN GLANDS . . .	No gross observations on tissue	PORPHYRIN DEPOSITS, Slight, Multifocal.<br>HEMORRHAGE,UNILATERAL, Minimal, Multifocal.<br>ADENITIS,UNILATERAL, Minimal, Multifocal, Acute.
LIVER	No gross observations on tissue	CHRONIC INFLAMMATION, Minimal, Multifocal.<br>HEPATOCELLULAR VACUOLATION,PERIPORTAL, Minimal.<br>FOCAL NECROSIS,SUBCAPSULAR, Minimal. / with associated chronic inflammation.
SPLEEN	ENLARGED	Tissue is unremarkable.

The following tissues have no gross observations and were not examined microscopically:

ADRENALS	AORTA	BONE MARROW	BRAIN	CECUM
COLON	DIAPHRAGM	DUODENUM	ESOPHAGUS	EYES
FEMUR	HEART	ILEUM	JEJUNUM	KIDNEYS
MANDIBULAR L.N.	MESENTERIC L.N.	LUNG	MAMMARY GLAND	SKELETAL MUSCLE
SCIATIC NERVE	OPTIC NERVES	OVARIES	PANCREAS	PITUITARY
PARATHYROIDS	SPINAL CORD CERV	SPINAL CORD THOR	MANDIBULAR S.G.	PAROTIDS
STIFLE JOINT	SKIN	STOMACH	STERNUM	THYROIDS
THYMUS	TONGUE	TRACHEA	URINARY BLADDER	UTERUS
VAGINA				

The following tissues have no gross observations and were marked as unremarkable microscopically:
No tissue to list.

Appendix 10
Individual Animal Microscopic vs. Gross Observations

Fexinidazole   Study Number: 0504-2007

ANIMAL: 2831   SEX: Female   GROUP: 4   DOSE LEVEL: 800 mg/kg
DAY OF DEATH: 43 Test period   STATUS: Final phase sacrifice   TERMINAL BODY WEIGHT (g) : 206.8

Tissue	Gross observations/comments	Correlated Microscopic Observations
GENER. CONDITION . . .	GOOD	No micropathology observations on tissue.
HARDERIAN GLANDS . . .	No gross observations on tissue	PORPHYRIN DEPOSITS, Moderate, Multifocal.
		HEMORRHAGE,UNILATERAL, Minimal, Multifocal.
LIVER	No gross observations on tissue	CHRONIC INFLAMMATION, Minimal, Multifocal.

The following tissues have no gross observations and were not examined microscopically:

ADRENALS	AORTA	BONE MARROW	BRAIN	CECUM
COLON	DIAPHRAGM	DUODENUM	ESOPHAGUS	EYES
FEMUR	HEART	ILEUM	JEJUNUM	KIDNEYS
MANDIBULAR L.N.	MESENTERIC L.N.	LUNG	MAMMARY GLAND	SKELETAL MUSCLE
SCIATIC NERVE	OPTIC NERVES	OVARIES	PANCREAS	PITUITARY
PARATHYROIDS	SPINAL CORD CERV	SPINAL CORD THOR	MANDIBULAR S.G.	PAROTIDS
STIFLE JOINT	SKIN	SPLEEN	STOMACH	STERNUM
THYROIDS	THYMUS	TONGUE	TRACHEA	URINARY BLADDER
UTERUS	VAGINA			

The following tissues have no gross observations and were marked as unremarkable microscopically:
No tissue to list.

Appendix 10
Individual Animal Microscopic vs. Gross Observations

Fexinidazole | Study Number: 0504-2007

ANIMAL: 2832 | SEX: Female | GROUP: 4 | DOSE LEVEL: 800 mg/kg
DAY OF DEATH: 43 Test period | STATUS: Final phase sacrifice | TERMINAL BODY WEIGHT (g) : 213.0

Tissue	Gross observations/comments	Correlated Microscopic Observations
GENER. CONDITION . . .	GOOD	No micropathology observations on tissue.
HARDERIAN GLANDS . . .	No gross observations on tissue	PORPHYRIN DEPOSITS, Slight, Multifocal.<br>HEMORRHAGE,UNILATERAL, Slight, Multifocal.
LIVER	No gross observations on tissue	CHRONIC INFLAMMATION, Minimal, Multifocal.<br>HEPATOCELLULAR HYPERTROPHY,CENTRILOBULAR, Minimal.

The following tissues have no gross observations and were not examined microscopically:

ADRENALS	AORTA	BONE MARROW	BRAIN	CECUM
COLON	DIAPHRAGM	DUODENUM	ESOPHAGUS	EYES
FEMUR	HEART	ILEUM	JEJUNUM	KIDNEYS
MANDIBULAR L.N.	MESENTERIC L.N.	LUNG	MAMMARY GLAND	SKELETAL MUSCLE
SCIATIC NERVE	OPTIC NERVES	OVARIES	PANCREAS	PITUITARY
PARATHYROIDS	SPINAL CORD CERV	SPINAL CORD THOR	MANDIBULAR S.G.	PAROTIDS
STIFLE JOINT	SKIN	SPLEEN	STOMACH	STERNUM
THYROIDS	THYMUS	TONGUE	TRACHEA	URINARY BLADDER
UTERUS	VAGINA			

The following tissues have no gross observations and were marked as unremarkable microscopically:
No tissue to list.

Appendix 10
Individual Animal Microscopic vs. Gross Observations

Fexinidazole | Study Number: 0504-2007

ANIMAL: 2833 | SEX: Female | GROUP: 4 | DOSE LEVEL: 800 mg/kg
DAY OF DEATH: 43 Test period | STATUS: Final phase sacrifice | TERMINAL BODY WEIGHT (g) : 229.2

Tissue	Gross observations/comments	Correlated Microscopic Observations
GENER. CONDITION . . .	GOOD	No micropathology observations on tissue.
HARDERIAN GLANDS . . .	No gross observations on tissue	PORPHYRIN DEPOSITS, Slight, Multifocal.<br><br>ADENITIS,UNILATERAL, Minimal, Multifocal, Acute.
LIVER	No gross observations on tissue	CHRONIC INFLAMMATION, Minimal, Multifocal.<br><br>HEPATOCELLULAR VACUOLATION,PERIPORTAL, Minimal.

The following tissues have no gross observations and were not examined microscopically:

ADRENALS	AORTA	BONE MARROW	BRAIN	CECUM
COLON	DIAPHRAGM	DUODENUM	ESOPHAGUS	EYES
FEMUR	HEART	ILEUM	JEJUNUM	KIDNEYS
MANDIBULAR L.N.	MESENTERIC L.N.	LUNG	MAMMARY GLAND	SKELETAL MUSCLE
SCIATIC NERVE	OPTIC NERVES	OVARIES	PANCREAS	PITUITARY
PARATHYROIDS	SPINAL CORD CERV	SPINAL CORD THOR	MANDIBULAR S.G.	PAROTIDS
STIFLE JOINT	SKIN	SPLEEN	STOMACH	STERNUM
THYROIDS	THYMUS	TONGUE	TRACHEA	URINARY BLADDER
UTERUS	VAGINA			

The following tissues have no gross observations and were marked as unremarkable microscopically:
No tissue to list.

Appendix 10
Individual Animal Microscopic vs. Gross Observations

Fexinidazole Study Number: 0504-2007

ANIMAL: 2834 SEX: Female GROUP: 4 DOSE LEVEL: 800 mg/kg
DAY OF DEATH: 43 Test period STATUS: Final phase sacrifice TERMINAL BODY WEIGHT (g) : 228.7

Tissue	Gross observations/comments	Correlated Microscopic Observations
GENER. CONDITION . . .	GOOD	No micropathology observations on tissue.
HARDERIAN GLANDS . . .	No gross observations on tissue	PORPHYRIN DEPOSITS, Moderate, Multifocal.<br>HEMORRHAGE,UNILATERAL, Moderate, Focal.<br>ADENITIS,UNILATERAL, Slight, Focal, Chronic.
LIVER	No gross observations on tissue	CHRONIC INFLAMMATION, Minimal, Multifocal.

The following tissues have no gross observations and were not examined microscopically:

ADRENALS	AORTA	BONE MARROW	BRAIN	CECUM
COLON	DIAPHRAGM	DUODENUM	ESOPHAGUS	EYES
FEMUR	HEART	ILEUM	JEJUNUM	KIDNEYS
MANDIBULAR L.N.	MESENTERIC L.N.	LUNG	MAMMARY GLAND	SKELETAL MUSCLE
SCIATIC NERVE	OPTIC NERVES	OVARIES	PANCREAS	PITUITARY
PARATHYROIDS	SPINAL CORD CERV	SPINAL CORD THOR	MANDIBULAR S.G.	PAROTIDS
STIFLE JOINT	SKIN	SPLEEN	STOMACH	STERNUM
THYROIDS	THYMUS	TONGUE	TRACHEA	URINARY BLADDER
UTERUS	VAGINA			

The following tissues have no gross observations and were marked as unremarkable microscopically:
No tissue to list.

Appendix 11 Toxicokinetic Report

TOXICOKINETIC REPORT FOR THE STUDY

Fexinidazole: 28-Day Oral Toxicity Study in the Rat

Product Name:	Fexinidazole
Study Number:	0504-2007
Study Director:	
Status:	Final

TABLE OF CONTENTS

1. INTRODUCTION AND OBJECTIVE 3
2. STUDY SPONSOR 3
3. TEST FACILITY....................................3
4. REGULATORY REQUIREMENTS....................................3
5. ABBREVIATIONS 3
6. METHODS 4
6.1. Study Design 4
6.2. Sample Collection and Handling....................................4
6.3. Bioanalytical Method 4
6.4. Pharmacokinetic Calculations 4
7. RESULTS 5
7.1. Tables and Figures....................................5
7.2. Control Sample Analysis....................................5
7.3. Pharmacokinetic results....................................6
8. CONCLUSIONS....................................9
9. CONTRIBUTORS....................................10
10. ARCHIVING 10
11. REFERENCES 10
TABLES AND FIGURES 11
APPENDICES
Appendix 1. Individual plasma concentrations 59
Appendix 2. In-Study Bioanalytical Validation Data 79
Appendix 3. Certificates of Analysis....................................88

1. INTRODUCTION AND OBJECTIVE

Fexinidazole is a 5-nitroimidazole derivate under investigation for the treatment of the Human African Trypanosomiasis (HAT), known as sleeping sickness.

As part of a GLP toxicity study, the toxicokinetics of Fexinidazole and its sulfoxide and sulfone metabolites were evaluated after the first and repeated oral administrations of Fexinidazole to male and female Crl:CD (SD)IGS BR rats.

2. STUDY SPONSOR

DND*i* – Drugs for Neglected Diseases *Initiative*
Place St Gervais 1, 1201 Geneva, Switzerland.

3. TEST FACILITY

Accelera

4. REGULATORY REQUIREMENTS

This study will be GLP regulated and will be conducted in compliance with:

- DECRETO LEGISLATIVO 2 Marzo 2007, No. 50 and
- Organization for Economic Co-operation and Development (OECD) Principles of Good Laboratory Practice (GLP) (as revised in 1997).

5. ABBREVIATIONS

The following abbreviations are used in this document:

$AUC_{0-t(last)}$	Area under the plasma concentration *vs.* time curve up to finite time
C_{max}	Maximal plasma concentration
CV	Coefficient of variation of the mean
F	Female
ID	Animal identification code
LC	Liquid chromatography
LLOQ	Lower limit of quantification
M	Male
MS	Mass-spectrometry
Norm	Normalized value
QC	Quality control sample
R^2	Correlation coefficient
R_A	Accumulation ratio

SD	Standard deviation of the mean
STD	Standard sample
$t_{1/2,z}$	Terminal half-life
t_{max}	Time to peak plasma concentration
ULOQ	Upper limit of quantification

6. METHODS

6.1. Study Design

The study was conducted according to the study protocol and related amendment [1, 2]. Fexinidazole was orally given by gavage once a day for 28 days to male and female Sprague Dawley rats according to the following scheme

Dose (mg/kg/day)	Volume (mL/kg/day)	Rat ID
0	10	M: 2835-2837; F: 2847-2849
50	10	M: 2838-2840; F: 2850-2852
200	10	M: 2841-2843; F: 2853-2855
800	10	M: 2844-2846; F: 2856-2858

Fexinidazole was suspended with 5% Tween 80 in 0.5% Methyl cellulose 400 cP.

6.2. Sample Collection and Handling

Blood samples (about 0.25 mL/sampling time) were withdrawn from retro-orbital sinus plexus under isofluorane anesthesia and put in heparinized plastic tubes kept on a ice-water bath, then centrifuged for 10 min at 1200g at +4°C and two aliquots of about 50 µL of plasma were stored in a freezer at -80°C until analysis. Blood was taken from three rats/gender/dose on Days 1 and 14 at pre-dose and 0.5, 1, 2, 4, 8 and 24 hours after dosing, on Day 28 at pre-dose and 1, 2, 4, 8, 24, 48 and 72 hours after dosing. After the administration of the vehicle, the samples were taken from three rats/gender at pre-dose and 2 hours post dosing.

6.3. Bioanalytical Method

Rat plasma concentrations of Fexinidazole and its sulfoxide (M1) and sulfone (M2) metabolites were determined by a validated LC/MS/MS method [3,4]. The calibration range for the assay was 5-1000 ng/mL for Fexinidazole and 25-25000 ng/mL for M1 and M2. Study samples containing analytes concentrations above the upper limit of quantitation were diluted with blank matrix prior to analysis.

6.4. Pharmacokinetic Calculations

Pharmacokinetic evaluation was carried out using non-compartmental approach with the aid of the Watson package (v. 6.4.0.04, Thermo Fisher Scientific, Waltham, MA, USA) and Excel spreadsheet (Microsoft Inc., Seattle, USA).

In the calculations, the undetectable concentrations between detectable ones were ignored.

After each dose level, C_{max} and t_{max} of Fexinidazole and sulfoxide and sulfone metabolites were read from raw data as the coordinates of the highest measured concentration. The area under plasma concentration *vs.* time curve to finite time, $AUC_{0-t(last)}$, was determined by the linear trapezoidal rule up to the last detectable concentration. On Day 28, since the blood sampling was performed up to 72 hours post dosing, in addition to $AUC_{0-t(last)}$, AUC within 24 hours post dosing was calculated and denoted AUC_{0-24}. This AUC was calculated in order to compare AUC values on Day 28 to those on Day 1 and Day 14. On Day 28, in addition, the half-life of the terminal phase, $t_{1/2,z}$, was determined by linear regression analysis of the natural-log concentration *vs.* time curve, where $t_{1/2,z}$ = ln(2)/slope of the regression line.

After each dose, C_{max} and AUC values of each compound were also normalized to 1 mg/kg/day dose level.

Metabolite to parent ratio was calculated based on C_{max} and $AUC_{0-t(last)}$ values corrected for the molecular weight.

Fexinidazole and metabolites accumulation ratios, based on C_{max} (R_A,C_{max}), AUC_{0-24} (R_A,AUC_{0-24}) and $AUC_{0-t(last)}$ (R_A,$AUC_{0-t(last)}$), were calculated as the ratio between the parameters obtained on Day 14 and Day 28 to the corresponding one at Day 1.

Descriptive statistics (mean ± SD, %CV) were reported for plasma concentrations and pharmacokinetic parameters of each compound sorted by dose, gender and day of dosing.

7. RESULTS

7.1. Tables and Figures

Mean C_{max}, t_{max}, AUC within 24 hours post dosing and $AUC_{0-t(last)}$ parameters of each compound are reported in Tables 1 and 2, whilst individual and mean parameters of each compound are reported in Tables 3 - 20. Individual and mean (±SD, CV%) plasma concentrations of Fexinidazole and sulfoxide and sulfone metabolites are reported in Tables 2A1 - 19A1 of Appendix 1. Individual plasma concentrations of Fexinidazole and both metabolites are plot in Figures 1 - 9, whilst the mean concentrations are plot in Figures 10 - 15. Mean normalized C_{max} and $AUC_{0-t(last)}$ values of Fexinidazole and metabolites vs. dose are plot in Figures 16 - 21.

In-study bioanalytical validation data are reported in Appendix 2. All analytical batches met acceptance criteria as described in PCD-M-BPK-001-01 SOP [5]. Bioanalytical data are stored in Watson LIMS (v. 6.4.0.04, Thermo Fisher Scientific, Waltham, MA, USA) under Project ID: 348-Fexinidazole and Study ID 0504-2007. Certificates of analysis are reported in Appendix 3.

7.2. Control Sample Analysis

One detectable concentrations of each compound was measured in the control samples. Analyses were rerun in duplicate and the results confirmed. The detectable concentration of Fexinidazole was 9.61 ng/mL, whilst those of sulfoxide and sulfone metabolites were 29.4

and 32.7 ng/mL, respectively (see Table 1A1 of Appendix 1). These values were close to the lower limit of quantification of the method for each compound. For this reason, the observed contaminations do not affect the conclusions drawn from the toxicokinetic analysis for this study.

7.3. Pharmacokinetic results

Day 1

Mean ±SD systemic exposure to Fexinidazole is reported in the following table

Dose	Male			Female		
mg/kg	C_{max}	t_{max}	$AUC_{0\text{-}t(last)}$	C_{max}	t_{max}	$AUC_{0\text{-}t(last)}$
	ng/mL	hour	ng·hour/mL	ng/mL	hour	ng·hour/mL
50	39±17	1±0	178±99	149±46	2±2	759±153
200	165±97	2±1	1120±708	604±211	2±2	3200±856
800	1478±1111	2±1	10000±2160	1483±200	2±1	15700±3610

The male rat ID 2843 at 200 mg/kg showed levels of Fexinidazole about five times lower than those measured in the other two rats.
At each dose, C_{max} and $AUC_{0\text{-}t(last)}$ values were about three times higher in female than in male rats. In both genders, the maximal plasma concentrations of Fexinidazole were promptly achieved, on average within 2 hours post dosing. In both genders, $AUC_{0\text{-}t(last)}$ values of Fexinidazole increased with the dose administered (Figures 16 - 17).

Mean ±SD systemic exposure to the sulfoxide metabolite is reported in the following table

Dose	Male			Female		
mg/kg	C_{max}	t_{max}	$AUC_{0\text{-}t(last)}$	C_{max}	t_{max}	$AUC_{0\text{-}t(last)}$
	ng/mL	hour	ng·hour/mL	ng/mL	hour	ng·hour/mL
50	1503±413	2±1	7660±2380	3893 ±663	3±1	23200±5330
200	9987±2131	2±0	80500±4310	12800±1127	3±1	91400±19700
800	32833±4952	5±2	366000±113000	30633±2616	4±0	453000±84700

Apart from 50 mg/kg dose, no relevant gender difference in C_{max} and $AUC_{0\text{-}t(last)}$ values was observed. In both genders, t_{max} values of the metabolite were similar to the corresponding ones of the parent compound. In both genders, the systemic exposure to the metabolite increased roughly in direct proportion with the dose (Figures 18 - 19).
In the male rat, the metabolite to parent $AUC_{0\text{-}t(last)}$ ratios were, on average, 46, 107 and 37 after 50, 200 and 800 mg/kg, respectively; the corresponding values in females were 29, 30 and 28. The systemic exposure to the metabolite was much higher than that to the parent compound.

Mean ±SD systemic exposure to the sulfone metabolite is reported in the following table

Dose	Male			Female		
mg/kg	C_{max}	t_{max}	$AUC_{0-t(last)}$	C_{max}	t_{max}	$AUC_{0-t(last)}$
	ng/mL	hour	ng·hour/mL	ng/mL	hour	ng·hour/mL
50	1417±393	7±2	16500±9760	4417±2410	8±0	55800±28100
200	8550±2309	8±0	119000±21900	10023±815	8±0	134000±10800
800	40300±1562	8±0	465000±264000	44833±20128	13±9	683000±241000

Apart from 50 mg/kg dose, no relevant gender difference in C_{max} and $AUC_{0-t(last)}$ values was observed. In both genders, t_{max} values of the metabolite were achieved at later times in comparison with the corresponding ones of the parent compound. The systemic exposure to the metabolite increased with the dose administered (Figures 20 - 21).
In the male rats, the metabolite to parent $AUC_{0-t(last)}$ ratios were, on average, 81, 172 and 46 after 50, 200 and 800 mg/kg, respectively; the corresponding values in females were 64, 40 and 39. The systemic exposure to the metabolite was much higher than that to the parent compound.

Repeated dosing

Day 14 and Day 28 mean ±SD systemic exposure to Fexinidazole is reported in the following table

Dose	Male			Female		
mg/kg/day	C_{max}	t_{max}	$AUC_{0-t(last)}$	C_{max}	t_{max}	$AUC_{0-t(last)}$
	ng/mL	hour	ng·hour/mL	ng/mL	hour	ng·hour/mL
	Day 14					
50	62±19	2±2	345±125	300±91	2±1	1310±161
200	274±163	3±1	2010±1520	773±107	2±1	4030±215
800	565±106	3±1	5120±1540	1478±1115	3±4	13500±3620
	Day 28					
50	133±24	4±0	889±287	422±358	1±0	1447±335 (1)
						1630±570
200	255±136	2±2	2326±1757 (1)	903±87	2±1	4437±514 (1)
			4410±3980			4750±508
800	523±127	2±2	3173±734 (1)	749±90 (2)	2±1 (2)	7640±1782 (1,2)
			11900±5360			7940±2210 (2)
(1) AUC within 24 hours post dosing<br>(2) n=2						

As for Day 1, the male rat ID 2843 treated at 200 mg/kg/day showed levels of Fexinidazole remarkably lower than those measured in the other two rats.
At each dose, C_{max} and $AUC_{0-t(last)}$ values were two - three times higher in female than in male rats. No relevant difference in the Fexinidazole levels were observed on Day 28 compared to Day 14. On Days 14 and 28, in both genders, the maximal plasma concentrations of Fexinidazole were achieved, on average, within 4 hours post dosing. On

Day 28, in males, mean ±SD apparent terminal half-lives were 4.7 ±1.2, 8.8 ±4.8 and 11 ±2.7 hours after 50, 200 and 800 mg/kg/day, respectively. The corresponding female half-lives were 5.1 ±5.1, 6.3 ±4.5 and 6.7 ±2.3 (n=2) hours. In both genders, $AUC_{0\text{-}t(last)}$ values of Fexinidazole increased with the dose (Figures 16 - 17).
Day 14 and Day 28 male $AUC_{0\text{-}t(last)}$ (AUC within 24 hours post dosing on Day 28) accumulation ratios were, on average, 2.2 and 5.7 after 50 mg/kg/day, 1.6 and 2 after 200 mg/kg/day and 0.5 and 0.3 after 800 mg/kg/day, respectively. The corresponding values in females were 1.7 and 2, 1.3 and 1.5, 0.9 and 0.4.

Day 14 and Day 28 mean ±SD systemic exposure to the sulfoxide metabolite is reported in the following table

Dose	Male			Female		
mg/kg/day	C_{max}	t_{max}	$AUC_{0\text{-}t(last)}$	C_{max}	t_{max}	$AUC_{0\text{-}t(last)}$
	ng/mL	hour	ng·hour/mL	ng/mL	hour	ng·hour/mL
	Day 14					
50	2267±505	2±2	12500±4050	8197±519	2±0	47100±4920
200	13100±1044	3±1	93600±27300	18567±1266	3±1	143000±34900
800	17733±1701	3±1	165000±29100	32233±6435	2±0	390000±159000
	Day 28					
50	4530±664	3±1	29467±8919 (1)	11413±6603	1±1	53833±15716 (1)
			30600±10900			78000±23600
200	11633±2403	3±1	122100±29340 (1)	22333±1474	2±1	138333±14189 (1)
			138000±45000			153000±36900
800	16567±2079	2±1	129733±56812 (1)	25850±2051(2)	3±1(2)	291500±40305(1,2)
			268000±64100			331000±57300(2)

(1) AUC within 24 hours post dosing

(2) n=2

At each dose, the levels of the metabolite were about two - three times higher in the female than in the male rat. No relevant difference in the levels of the metabolite were observed on Day 28 compared to Day 14. In both genders, the maximal plasma concentrations of the sulfoxide metabolite were achieved, on average, 2 - 3 hours post dosing. On Day 28, mean ±SD male apparent terminal half-lives were 5 ±2, 6.3 ±2.9 and 6.6 ±4.1 hours after 50, 200 and 800 mg/kg/day, respectively, whilst the corresponding female half-lives were 6.1 ±3.8, 8.1 ±3.1 and 8.6 ±2.3 (n=2) hours. In both genders, the systemic exposure to the metabolite increased with the dose (Figures 18 - 19).
Day 14 and Day 28 male $AUC_{0\text{-}t(last)}$ (AUC within 24 hours post dosing on Day 28) accumulation ratios were, on average, 1.6 and 3.9 after 50 mg/kg/day, 1.2 and 1.5 after 200 mg/kg/day and 0.5 and 0.4 after 800 mg/kg/day, respectively. The corresponding values in females were 2.1 and 2.5, 1.6 and 1.5, 0.8 and 0.6.
The systemic exposure to the metabolite was much higher than that to the parent compound.

Day 14 and Day 28 mean ±SD systemic exposure to the sulfone metabolite is reported in the following table

Dose	Male			Female		
mg/kg/day	C_{max}	t_{max}	$AUC_{0-t(last)}$	C_{max}	t_{max}	$AUC_{0-t(last)}$
	ng/mL	hour	ng·hour/mL	ng/mL	hour	ng·hour/mL
	Day 14					
50	4773±566	4±0	62800±11300	7980±2285	8±0	116000±24900
200	18800±1735	7±2	268000±35800	21533±2458	8±0	306000±31200
800	30833±1724	5±2	423000±70500	50233±11651	8±0	664000±332000
	Day 28					
50	7750±831	7±2	106267±9530 (1)	10700±700	7±2	174667±40501 (1)
			113000±13700			197000±50600
200	24200±3041	8±0	341333±39068 (1)	21433±4179	8±0	341333±66124 (1)
			387000±34000			448000±135000
800	33000±6655	5±2	459000±60605 (1)	41900±2970 (2)	6±3 (2)	571500±54447 (1,2)
			509000±94900			738000±135000 (2)

(1) AUC within 24 hours post dosing

(2) n=2

At each dose, no relevant gender difference in C_{max} and $AUC_{0-t(last)}$ values was observed. No relevant difference in the levels of the metabolite were observed on Day 28 compared to Day 14. T_{max} values of the metabolite were achieved at later times in comparison with the corresponding ones of the parent compound. On Day 28, mean male apparent terminal half-lives were 6.1 ±1.2, 7.5 ±2.4 and 10 ±3 hours after 50, 200 and 800 mg/kg/day, respectively, the corresponding half-lives in females were 13 ±9.8, 7.3 ±2.6 and 11 ±5.6 (n=2) hours. The systemic exposure to the metabolite increased with the dose (Figures 20 - 21).
Day 14 and Day 28 male $AUC_{0-t(last)}$ (AUC within 24 hours post dosing on Day 28) accumulation ratios were, on average, 5.2 and 9.3 after 50 mg/kg/day, 2.3 and 2.9 after 200 mg/kg/day and 1.3 and 1.6 after 800 mg/kg/day, respectively. The corresponding values in females were 2.3 and 3.8, 2.3 and 2.6, 1 and 0.8.
The systemic exposure to the metabolite was much higher than that to the parent compound.

8. CONCLUSIONS

After the first and repeated administrations of the three dose levels, AUCs of Fexinidazole were two - three times higher in females than in males. On Days 1, 14 and 28, in both genders, AUCs of Fexinidazole increased with the dose administered. Whilst no accumulation was observed after the highest dose, in both genders, an accumulation ratio of Fexinidazole of about 2 was observed after 50 and 200 mg/kg/day.
After the first and repeated dosing to male and female rats, Fexinidazole was extensively metabolized to the sulfone and sulfoxide derivatives. The half-lives of both metabolites were similar to those of the parent compound.

9. CONTRIBUTORS

10. ARCHIVING

The protocol, the protocol amendment as original, raw data, pharmacokinetic analysis and final report as original were archived within Accelera Archive, Nerviano Medical Sciences, Italy, according the Unit Standard Operating Procedures.

11. REFERENCES

1. Fexinidazole: 28-Day Oral Toxicity Study in the Rat. Nerviano Medical Sciences Study Protocol 0504-2007-P, January 29, 2008.

2. Amendment no. 1 to Fexinidazole: 28-Day Oral Toxicity Study in the Rat. February 21, 2008.

3. Analytical Procedure for the Determination of Fexinidazole and its Metabolites Fexinidazole Sulphoxide (M1) and Fexinidazole Sulphone (M2) in Rat Plasma by LC-MS-MS Following Plasma Protein Precipitation. Analytical Procedure Number: NMS/FEXINIDAZOLE/03.0. NervianoMS Reference Number: 0290-2007-AP. February 29, 2008.

4. Validation of an Analytical Method for the Determination of Fexinidazole and its Metabolites M1 and M2 in Rat Plasma by LC-MS-MS. Document Number: 0290-2007-R.

5. SOP: PCD-M-BPK-001-01: “Bioanalytical Run Acceptance”. 13 December 2006.

TABLES AND FIGURES

Table 1. Summary table of mean ±SD systemic exposure values of Fexinidazole and metabolites after oral 50, 200 and 800 mg/kg/day of Fexinidazole in male Sprague Dawley rats.

Day	Fexinidazole			Sulfoxide			Sulfone		
	C_{max}	t_{max}	$AUC_{0-t(last)}$	C_{max}	t_{max}	$AUC_{0-t(last)}$	C_{max}	t_{max}	$AUC_{0-t(last)}$
	ng/mL	hour	ng·hour/mL	ng/mL	hour	ng·hour/mL	ng/mL	hour	ng·hour/mL
	50 mg/kg/day								
1	39±17	1±0	178±99	1503±413	2±1	7660±2380	1417±393	7±2	16500±9760
14	62±19	2±2	345±125	2267±505	2±2	12500±4050	4773±566	4±0	62800±11300
28	133±24	4±0	889±287	4530±664	3±1	29467±8919 (1)	7750±831	7±2	106267±9530 (1)
						30600±10900			113000±13700
	200 mg/kg/day								
1	165±97	2±1	1120±708	9987±2131	2±0	80500±4310	8550±2309	8±0	119000±21900
14	274±163	3±1	2010±1520	13100±1044	3±1	93600±27300	18800±1735	7±2	268000±35800
28	255±136	2±2	2326±1757 (1)	11633±2403	3±1	122100±29340 (1)	24200±3041	8±0	341333±39068 (1)
			4410±3980			138000±45000			387000±34000
	800 mg/kg/day								
1	1478±1111	2±1	10000±2160	32833±4952	5±2	366000±113000	40300±1562	8±0	465000±264000
14	565±106	3±1	5120±1540	17733±1701	3±1	165000±29100	30833±1724	5±2	423000±70500
28	523±127	2±2	3173±734 (1)	16567±2079	2±1	129733±56812 (1)	33000±6655	5±2	459000±60605 (1)
			11900±5360			268000±64100			509000±94900

(1) AUC within 24 hours post dosing (AUC_{0-24})

Table 2. Summary table of mean ±SD systemic exposure values of Fexinidazole and metabolites after oral 50, 200 and 800 mg/kg/day of Fexinidazole in female Sprague Dawley rats.

Day	Fexinidazole			Sulfoxide			Sulfone		
	C_{max}	t_{max}	$AUC_{0-t(last)}$	C_{max}	t_{max}	$AUC_{0-t(last)}$	C_{max}	t_{max}	$AUC_{0-t(last)}$
	ng/mL	hour	ng·hour/mL	ng/mL	hour	ng·hour/mL	ng/mL	hour	ng·hour/mL
	50 mg/kg/day								
1	149±46	2±2	759±153	3893±663	3±1	23200±5330	4417±2410	8±0	55800±28100
14	300±91	2±1	1310±161	8197±519	2±0	47100±4920	7980±2285	8±0	116000±24900
28	422±358	1±0	1447±335 (1)	11413±6603	1±1	53833±15716 (1)	10700±700	7±2	174667±40501 (1)
			1630±570			78000±23600			197000±50600
	200 mg/kg/day								
1	604±211	2±2	3200±856	12800±1127	3±1	91400±19700	10023±815	8±0	134000±10800
14	773±107	2±1	4030±215	18567±1266	3±1	143000±34900	21533±2458	8±0	306000±31200
28	903±87	2±1	4437±514 (1)	22333±1474	2±1	138333±14189 (1)	21433±4179	8±0	341333±66124 (1)
			4750±508			153000±36900			448000±135000
	800 mg/kg/day								
1	1483±200	2±1	15700±3610	30633±2616	4±0	453000±84700	44833±20128	13±9	683000±241000
14	1478±1115	3±4	13500±3620	32233±6435	2±0	390000±159000	50233±11651	8±0	664000±332000
28(2)	749±90	2±1	7640±1782 (1)	25850±2051	3±1	291500±40305 (1)	41900±2970	6±3	571500±54447 (1)
			7940±2210			331000±57300			738000±135000

(1) AUC within 24 hours post dosing (AUC_{0-24})
(2) n=2

Table 3. Day 1 individual and mean (±SD, %CV) pharmacokinetic parameters of Fexinidazole and metabolites after oral 50 mg/kg/day of Fexinidazole in male Sprague Dawley rats.

Parameter (Units)	ID 2838	ID 2839	ID 2840	Mean	SD	%CV
	Fexinidazole					
C_{max} (ng/mL)	35.5	24.3	56.9	38.9	16.6	43
t_{max} (hour)	1	1	1	1	0	0
$AUC_{0-t(last)}$ Interval (hour)	0-8	0-4	0-8	N/A	N/A	N/A
$AUC_{0-t(last)}$ (ng·hour/mL)	184	75.8	274	178	99.2	56
C_{max}, norm (1)	0.71	0.486	1.14	0.779	0.332	43
$AUC_{0-t(last)}$, norm (1)	3.68	1.52	5.48	3.56	1.98	56
	Sulfoxide					
C_{max} (ng/mL)	1450	1120	1940	1503	413	27
t_{max} (hour)	2	1	2	1.67	0.577	35
$AUC_{0-t(last)}$ Interval (hour)	0-8	0-8	0-8	N/A	N/A	N/A
$AUC_{0-t(last)}$ (ng·hour/mL)	7940	5150	9880	7660	2380	31
C_{max}, norm (1)	29	22.4	38.8	30.1	8.25	27
$AUC_{0-t(last)}$, norm (1)	159	103	198	153	47.8	31
(2)	38.6	43.6	32.2	38.2	5.69	15
(3)	40.8	64.3	34.1	46.4	15.8	34
	Sulfone					
C_{max} (ng/mL)	1470	1000	1780	1417	393	28
t_{max} (hour)	8	4	8	6.67	2.31	35
$AUC_{0-t(last)}$ Interval (hour)	0-24	0-8	0-24	N/A	N/A	N/A
$AUC_{0-t(last)}$ (ng·hour/mL)	19700	5580	24300	16500	9760	59
C_{max}, norm (1)	29.4	20	35.6	28.3	7.85	28
$AUC_{0-t(last)}$, norm (1)	394	112	486	331	195	59
(2)	37.2	36.9	28.1	34	5.18	15
(3)	96.1	66	79.6	80.6	15	19

N/A: not applicable
(1) C_{max} (ng/mL) and AUC (ng·hour/mL) values normalized to 1 mg/kg dose.
(2) C_{max}, metabolite / C_{max}, parent; (3) $AUC_{0-t(last)}$, metabolite / $AUC_{0-t(last)}$, parent

Table 4. Day 1 individual and mean (±SD, %CV) pharmacokinetic parameters of Fexinidazole and metabolites after oral 50 mg/kg/day of Fexinidazole in female Sprague Dawley rats.

Parameter (Units)	ID 2850	ID 2851	ID 2852	Mean	SD	%CV
	Fexinidazole					
C_{max} (ng/mL)	182	168	95.6	149	46.4	31
t_{max} (hour)	1	0.5	4	1.83	1.89	103
$AUC_{0\text{-}t(last)}$ Interval (hour)	0-8	0-8	0-8	N/A	N/A	N/A
$AUC_{0\text{-}t(last)}$ (ng·hour/mL)	922	737	619	759	153	20
C_{max}, norm (1)	3.64	3.36	1.91	2.97	0.929	31
$AUC_{0\text{-}t(last)}$, norm (1)	18.4	14.7	12.4	15.2	3.03	20
	Sulfoxide					
C_{max} (ng/mL)	4520	3960	3200	3893	663	17
t_{max} (hour)	4	2	4	3.33	1.15	35
$AUC_{0\text{-}t(last)}$ Interval (hour)	0-8	0-8	0-8	N/A	N/A	N/A
$AUC_{0\text{-}t(last)}$ (ng·hour/mL)	29200	21400	19000	23200	5330	23
C_{max}, norm (1)	90.4	79.2	64	77.9	13.3	17
$AUC_{0\text{-}t(last)}$, norm (1)	583	428	379	463	106	23
(2)	23.5	22.3	31.7	25.8	5.1	20
(3)	30	27.5	29	28.8	1.26	4
	Sulfone					
C_{max} (ng/mL)	7190	2830	3230	4417	2410	55
t_{max} (hour)	8	8	8	8	0	0
$AUC_{0\text{-}t(last)}$ Interval (hour)	0-24	0-24	0-24	N/A	N/A	N/A
$AUC_{0\text{-}t(last)}$ (ng·hour/mL)	88100	37500	41700	55800	28100	50
C_{max}, norm (1)	144	56.6	64.6	88.4	48.3	55
$AUC_{0\text{-}t(last)}$, norm (1)	1760	750	834	1110	560	50
(2)	35.4	15.1	30.3	27	10.6	39
(3)	85.7	45.7	60.4	63.9	20.3	32

N/A: not applicable
(1) C_{max} (ng/mL) and AUC (ng·hour/mL) values normalized to 1 mg/kg dose.
(2) C_{max}, metabolite / C_{max}, parent; (3) $AUC_{0\text{-}t(last)}$, metabolite / $AUC_{0\text{-}t(last)}$, parent

Table 5. Day 14 individual and mean (±SD, %CV) pharmacokinetic parameters of Fexinidazole and metabolites after oral 50 mg/kg/day of Fexinidazole in male Sprague Dawley rats.

Parameter (Units)	ID 2838	ID 2839	ID 2840	Mean	SD	%CV
	Fexinidazole					
C_{max} (ng/mL)	79	42.2	65.8	62.3	18.6	30
t_{max} (hour)	4	2	1	2.33	1.53	66
$AUC_{0-t(last)}$ Interval (hour)	0-8	0-8	0-8	N/A	N/A	N/A
$AUC_{0-t(last)}$ (ng·hour/mL)	441	204	389	345	125	36
C_{max}, norm (1)	1.58	0.844	1.32	1.25	0.373	30
$AUC_{0-t(last)}$, norm (1)	8.83	4.07	7.78	6.89	2.5	36
R_A,C_{max}	2.23	1.74	1.16	1.71	0.535	31
R_A,$AUC_{0-t(last)}$	2.4	2.69	1.42	2.17	0.666	31
	Sulfoxide					
C_{max} (ng/mL)	2820	1830	2150	2267	505	22
t_{max} (hour)	4	2	1	2.33	1.53	66
$AUC_{0-t(last)}$ Interval (hour)	0-8	0-8	0-24	N/A	N/A	N/A
$AUC_{0-t(last)}$ (ng·hour/mL)	15300	7870	14400	12500	4050	32
C_{max}, norm (1)	56.4	36.6	43	45.3	10.1	22
$AUC_{0-t(last)}$, norm (1)	306	157	287	250	81.1	32
(2)	33.8	41	30.9	35.2	5.21	15
(3)	32.8	36.5	35	34.8	1.85	5
R_A,C_{max}	1.94	1.63	1.11	1.56	0.423	27
R_A,$AUC_{0-t(last)}$	1.93	1.53	1.46	1.64	0.253	15
	Sulfone					
C_{max} (ng/mL)	5110	4120	5090	4773	566	12
t_{max} (hour)	4	4	4	4	0	0
$AUC_{0-t(last)}$ Interval (hour)	0-24	0-24	0-24	N/A	N/A	N/A
$AUC_{0-t(last)}$ (ng·hour/mL)	73900	51400	63100	62800	11300	18
C_{max}, norm (1)	102	82.4	102	95.5	11.3	12
$AUC_{0-t(last)}$, norm (1)	1480	1030	1260	1260	225	18
(2)	58	87.6	69.4	71.7	14.9	21
(3)	150	226	146	174	45.2	26
R_A,C_{max}	3.48	4.12	2.86	3.49	0.63	18
R_A,$AUC_{0-t(last)}$	3.75	9.21	2.6	5.19	3.53	68

N/A: not applicable
(1) C_{max} (ng/mL) and AUC (ng·hour/mL) values normalized to 1 mg/kg dose.
(2) C_{max}, metabolite / C_{max}, parent; (3) $AUC_{0-t(last)}$, metabolite / $AUC_{0-t(last)}$, parent

Table 6. Day 14 individual and mean (±SD, %CV) pharmacokinetic parameters of Fexinidazole and metabolites after oral 50 mg/kg/day of Fexinidazole in female Sprague Dawley rats.

Parameter (Units)	ID 2850	ID 2851	ID 2852	Mean	SD	%CV
	Fexinidazole					
C_{max} (ng/mL)	292	395	213	300	91.3	30
t_{max} (hour)	2	0.5	2	1.5	0.866	58
$AUC_{0\text{-}t(last)}$ Interval (hour)	0-8	0-8	0-8	N/A	N/A	N/A
$AUC_{0\text{-}t(last)}$ (ng·hour/mL)	1480	1290	1160	1310	161	12
C_{max}, norm [(1)]	5.84	7.9	4.26	6	1.83	30
$AUC_{0\text{-}t(last)}$, norm [(1)]	29.7	25.8	23.3	26.3	3.23	12
R_A,C_{max}	1.6	2.35	2.23	2.06	0.4	19
R_A,$AUC_{0\text{-}t(last)}$	1.61	1.75	1.87	1.74	0.135	8
	Sulfoxide					
C_{max} (ng/mL)	8750	7720	8120	8197	519	6
t_{max} (hour)	2	2	2	2	0	0
$AUC_{0\text{-}t(last)}$ Interval (hour)	0-8	0-8	0-8	N/A	N/A	N/A
$AUC_{0\text{-}t(last)}$ (ng·hour/mL)	52600	43100	45600	47100	4920	11
C_{max}, norm [(1)]	175	154	162	164	10.6	7
$AUC_{0\text{-}t(last)}$, norm [(1)]	1050	863	912	942	97	10
[(2)]	28.3	18.5	36.1	27.6	8.81	32
[(3)]	33.6	31.6	37.2	34.1	2.83	8
R_A,C_{max}	1.94	1.95	2.54	2.14	0.343	16
R_A,$AUC_{0\text{-}t(last)}$	1.8	2.01	2.4	2.07	0.303	15
	Sulfone					
C_{max} (ng/mL)	10000	5500	8440	7980	2285	29
t_{max} (hour)	8	8	8	8	0	0
$AUC_{0\text{-}t(last)}$ Interval (hour)	0-24	0-24	0-24	N/A	N/A	N/A
$AUC_{0\text{-}t(last)}$ (ng·hour/mL)	138000	89200	122000	116000	24900	21
C_{max}, norm [(1)]	200	110	169	160	45.7	29
$AUC_{0\text{-}t(last)}$, norm [(1)]	2770	1780	2440	2330	504	22
[(2)]	30.7	12.5	35.6	26.3	12.2	46
[(3)]	83.7	62	94.4	80	16.5	21
R_A,C_{max}	1.39	1.94	2.61	1.98	0.612	31
R_A,$AUC_{0\text{-}t(last)}$	1.57	2.38	2.93	2.29	0.684	30

N/A: not applicable
[(1)] C_{max} (ng/mL) and AUC (ng·hour/mL) values normalized to 1 mg/kg dose.
[(2)] C_{max}, metabolite / C_{max}, parent; [(3)] $AUC_{0\text{-}t(last)}$, metabolite / $AUC_{0\text{-}t(last)}$, parent

Table 7. Day 28 individual and mean (±SD, %CV) pharmacokinetic parameters of Fexinidazole and metabolites after oral 50 mg/kg/day of Fexinidazole in male Sprague Dawley rats.

Parameter (Units)	ID 2838	ID 2839	ID 2840	Mean	SD	%CV
	Fexinidazole					
C_{max} (ng/mL)	148	105	145	133	24	18
t_{max} (hour)	4	4	4	4	0	0
$AUC_{0-t(last)}$ (ng·hour/mL)	859	619	1190	889	287	32
Regr. Range (hour)	4-8	4-8	4-24	N/A	N/A	N/A
$t_{1/2,z}$ (hour)	3.36	5.49	5.24	4.7	1.16	25
C_{max}, norm (1)	2.96	2.1	2.9	2.65	0.48	18
$AUC_{0-t(last)}$, norm (1)	17.2	12.4	23.8	17.8	5.72	32
R_A,C_{max}	4.17	4.32	2.55	3.68	0.983	27
R_A,$AUC_{0-t(last)}$	4.67	8.17	4.34	5.73	2.12	37
	Sulfoxide					
C_{max} (ng/mL)	4820	3770	5000	4530	664	15
t_{max} (hour)	4	2	4	3.33	1.15	35
AUC_{0-24} (ng·hour/mL)	27200	21900	39300	29467	8919	30
$AUC_{0-t(last)}$ (ng·hour/mL)	27200	21900	42800	30600	10900	36
Regr. Range (hour)	4-8	2-8	4-48	N/A	N/A	N/A
$t_{1/2,z}$ (hour)	2.63	6.11	6.23	4.99	2.04	41
C_{max}, norm (1)	96.4	75.4	100	90.6	13.3	15
AUC_{0-24}, norm (1)	545	438	786	590	178	30
$AUC_{0-t(last)}$, norm (1)	545	438	855	613	217	35
(2)	30.8	34	32.6	32.5	1.58	5
(3)	29.9	33.5	34	32.5	2.21	7
R_A,C_{max}	3.32	3.37	2.58	3.09	0.444	14
R_A,AUC_{0-24}	3.43	4.25	3.98	3.89	0.421	11
R_A,$AUC_{0-t(last)}$	3.43	4.25	4.33	4	0.502	13

Table 7. (cont'd). Day 28 individual and mean (±SD, %CV) pharmacokinetic parameters of Fexinidazole and metabolites after oral 50 mg/kg/day of Fexinidazole in male Sprague Dawley rats.

Parameter (Units)	ID 2838	ID 2839	ID 2840	Mean	SD	%CV
	Sulfone					
C_{max} (ng/mL)	7260	7280	8710	7750	831	11
t_{max} (hour)	8	8	4	6.67	2.31	35
AUC_{0-24} (ng·hour/mL)	103000	98800	117000	106267	9530	9
$AUC_{0-t(last)}$ (ng·hour/mL)	110000	101000	128000	113000	13700	12
Regr. Range (hour)	8-48	8-48	4-48	N/A	N/A	N/A
$t_{1/2,z}$ (hour)	5.97	5.08	7.36	6.14	1.15	19
C_{max}, norm (1)	145	146	174	155	16.5	11
AUC_{0-24}, norm (1)	2060	1976	2340	2125	191	9
$AUC_{0-t(last)}$, norm (1)	2200	2020	2550	2260	270	12
(2)	44	62.2	53.9	53.4	9.11	17
(3)	115	146	96.5	119	25.2	21
R_A,C_{max}	4.94	7.28	4.89	5.7	1.37	24
R_A,AUC_{0-24}	5.23	17.7	4.81	9.25	7.33	79
R_A,$AUC_{0-t(last)}$	5.58	18.1	5.27	9.65	7.32	76

N/A: not applicable;
(1) C_{max} (ng/mL) and AUC (ng·hour/mL) values normalized to 1 mg/kg dose.
(2) C_{max}, metabolite / C_{max}, parent;
(3) $AUC_{0-t(last)}$, metabolite / $AUC_{0-t(last)}$, parent

Table 8. Day 28 individual and mean (±SD, %CV) pharmacokinetic parameters of Fexinidazole and metabolites after oral 50 mg/kg/day of Fexinidazole in female Sprague Dawley rats.

Parameter (Units)	ID 2850	ID 2851	ID 2852	Mean	SD	%CV
	Fexinidazole					
C_{max} (ng/mL)	216	214	835	422	358	85
t_{max} (hour)	1	1	1	1	0	0
AUC_{0-24} (ng·hour/mL)	1060	1640	1640	1447	335	23
$AUC_{0-t(last)}$ (ng·hour/mL)	1060	2200	1640	1630	570	35
Regr. Range (hour)	4-8	4-48	1-8	N/A	N/A	N/A
$t_{1/2,z}$ (hour)	2.44	11	1.78	5.07	5.14	101
C_{max}, norm (1)	4.32	4.28	16.7	8.43	7.16	85
AUC_{0-24}, norm (1)	21.2	32.8	32.8	28.9	6.7	23
$AUC_{0-t(last)}$, norm (1)	21.2	44	32.7	32.6	11.4	35
R_A,C_{max}	1.19	1.27	8.73	3.73	4.33	116
R_A,AUC_{0-24}	1.15	2.23	2.65	2.01	0.773	38
R_A,$AUC_{0-t(last)}$	1.15	2.99	2.65	2.26	0.977	43
	Sulfoxide					
C_{max} (ng/mL)	6420	8920	18900	11413	6603	58
t_{max} (hour)	2	1	1	1.33	0.577	43
AUC_{0-24} (ng·hour/mL)	39600	70700	51200	53833	15716	29
$AUC_{0-t(last)}$ (ng·hour/mL)	87100	95700	51200	78000	23600	30
Regr. Range (hour)	2-48	8-72	1-8	N/A	N/A	N/A
$t_{1/2,z}$ (hour)	6.43	9.77	2.18	6.13	3.8	62
C_{max}, norm (1)	128	178	378	228	132	58
AUC_{0-24}, norm (1)	792	1414	1020	1075	315	29
$AUC_{0-t(last)}$, norm (1)	1740	1910	1020	1560	472	30
(2)	28.1	39.4	21.4	29.6	9.11	31
(3)	77.7	41.1	29.5	49.5	25.1	51
R_A,C_{max}	1.42	2.25	5.91	3.19	2.39	75
R_A,AUC_{0-24}	1.36	3.3	2.69	2.45	0.996	41
R_A,$AUC_{0-t(last)}$	2.98	4.47	2.69	3.38	0.954	28

Table 8. (cont'd). Day 28 individual and mean (±SD, %CV) pharmacokinetic parameters of Fexinidazole and metabolites after oral 50 mg/kg/day of Fexinidazole in female Sprague Dawley rats.

Parameter (Units)	ID 2850	ID 2851	ID 2852	Mean	SD	%CV
	Sulfone					
C_{max} (ng/mL)	10700	11400	10000	10700	700	7
t_{max} (hour)	8	8	4	6.67	2.31	35
AUC_{0-24} (ng·hour/mL)	146000	221000	157000	174667	40501	23
$AUC_{0-t(last)}$ (ng·hour/mL)	158000	254000	178000	197000	50600	26
Regr. Range (hour)	8-48	8-72	4-48	N/A	N/A	N/A
$t_{1/2,z}$ (hour)	6.23	23.8	7.45	12.5	9.81	79
C_{max}, norm (1)	214	228	200	214	14	7
AUC_{0-24}, norm (1)	2920	4420	3140	3493	810	23
$AUC_{0-t(last)}$, norm (1)	3150	5070	3560	3930	1010	26
(2)	44.4	47.8	10.7	34.3	20.5	60
(3)	134	104	97.4	112	19.4	17
R_A, C_{max}	1.49	4.03	3.1	2.87	1.28	45
R_A, AUC_{0-24}	1.66	5.89	3.76	3.77	2.12	56
$R_A, AUC_{0-t(last)}$	1.79	6.77	4.27	4.28	2.49	58

N/A: not applicable;
(1) C_{max} (ng/mL) and AUC (ng·hour/mL) values normalized to 1 mg/kg dose;
(2) C_{max}, metabolite / C_{max}, parent;
(3) $AUC_{0-t(last)}$, metabolite / $AUC_{0-t(last)}$, parent

Table 9. Day 1 individual and mean (±SD, %CV) pharmacokinetic parameters of Fexinidazole and metabolites after oral 200 mg/kg/day of Fexinidazole in male Sprague Dawley rats.

Parameter (Units)	ID 2841	ID 2842	ID 2843	Mean	SD	%CV
	Fexinidazole					
C_{max} (ng/mL)	176	256	63	165	97	59
t_{max} (hour)	2	2	1	1.67	0.577	35
$AUC_{0-t(last)}$ Interval (hour)	0-24	0-8	0-8	N/A	N/A	N/A
$AUC_{0-t(last)}$ (ng·hour/mL)	1700	1330	332	1120	708	63
C_{max}, norm (1)	0.88	1.28	0.315	0.825	0.485	59
$AUC_{0-t(last)}$, norm (1)	8.52	6.65	1.66	5.61	3.55	63
	Sulfoxide					
C_{max} (ng/mL)	7950	9810	12200	9987	2131	21
t_{max} (hour)	2	2	2	2	0	0
$AUC_{0-t(last)}$ Interval (hour)	0-24	0-24	0-8	N/A	N/A	N/A
$AUC_{0-t(last)}$ (ng·hour/mL)	85000	80100	76400	80500	4310	5
C_{max}, norm (1)	39.8	49.1	61	50	10.6	21
$AUC_{0-t(last)}$, norm (1)	425	400	382	402	21.6	5
(2)	42.7	36.2	183	87.4	83	95
(3)	47.3	57	218	107	95.7	89
	Sulfone					
C_{max} (ng/mL)	6600	7950	11100	8550	2309	27
t_{max} (hour)	8	8	8	8	0	0
$AUC_{0-t(last)}$ Interval (hour)	0-24	0-24	0-24	N/A	N/A	N/A
$AUC_{0-t(last)}$ (ng·hour/mL)	106000	106000	144000	119000	21900	19
C_{max}, norm (1)	33	39.8	55.5	42.8	11.5	27
$AUC_{0-t(last)}$, norm (1)	529	531	720	593	110	19
(2)	33.6	27.9	158	73.2	73.6	101
(3)	55.9	71.5	389	172	188	109

N/A: not applicable
(1) C_{max} (ng/mL) and AUC (ng·hour/mL) values normalized to 1 mg/kg dose.
(2) C_{max}, metabolite / C_{max}, parent; (3) $AUC_{0-t(last)}$, metabolite / $AUC_{0-t(last)}$, parent

Table 10. Day 1 individual and mean (±SD, %CV) pharmacokinetic parameters of Fexinidazole and metabolites after oral 200 mg/kg/day of Fexinidazole in female Sprague Dawley rats.

Parameter (Units)	ID 2853	ID 2854	ID 2855	Mean	SD	%CV
	Fexinidazole					
C_{max} (ng/mL)	830	569	412	604	211	35
t_{max} (hour)	2	4	0.5	2.17	1.76	81
$AUC_{0-t(last)}$ Interval (hour)	0-8	0-8	0-8	N/A	N/A	N/A
$AUC_{0-t(last)}$ (ng·hour/mL)	3850	3520	2230	3200	856	27
C_{max}, norm (1)	4.15	2.85	2.06	3.02	1.06	35
$AUC_{0-t(last)}$, norm (1)	19.2	17.6	11.2	16	4.23	27
	Sulfoxide					
C_{max} (ng/mL)	13400	13500	11500	12800	1127	9
t_{max} (hour)	2	4	2	2.67	1.15	43
$AUC_{0-t(last)}$ Interval (hour)	0-8	0-8	0-24	N/A	N/A	N/A
$AUC_{0-t(last)}$ (ng·hour/mL)	82000	78100	114000	91400	19700	22
C_{max}, norm (1)	67	67.5	57.5	64	5.63	9
$AUC_{0-t(last)}$, norm (1)	410	390	572	457	99.8	22
(2)	15.3	22.4	26.4	21.4	5.64	26
(3)	20.1	21	48.4	29.8	16	54
	Sulfone					
C_{max} (ng/mL)	9290	9880	10900	10023	815	8
t_{max} (hour)	8	8	8	8	0	0
$AUC_{0-t(last)}$ Interval (hour)	0-24	0-24	0-24	N/A	N/A	N/A
$AUC_{0-t(last)}$ (ng·hour/mL)	126000	129000	146000	134000	10800	8
C_{max}, norm (1)	46.5	49.4	54.5	50.1	4.05	8
$AUC_{0-t(last)}$, norm (1)	628	643	728	666	53.9	8
(2)	10	15.6	23.7	16.5	6.89	42
(3)	29.4	32.9	58.7	40.3	16	40

N/A: not applicable
(1) C_{max} (ng/mL) and AUC (ng·hour/mL) values normalized to 1 mg/kg dose.
(2) C_{max}, metabolite / C_{max}, parent; (3) $AUC_{0-t(last)}$, metabolite / $AUC_{0-t(last)}$, parent

Table 11. Day 14 individual and mean (±SD, %CV) pharmacokinetic parameters of Fexinidazole and metabolites after oral 200 mg/kg/day of Fexinidazole in male Sprague Dawley rats.

Parameter (Units)	ID 2841	ID 2842	ID 2843	Mean	SD	%CV
	Fexinidazole					
C_{max} (ng/mL)	355	381	87.2	274	163	59
t_{max} (hour)	2	4	2	2.67	1.15	43
$AUC_{0-t(last)}$ Interval (hour)	0-24	0-8	0-8	N/A	N/A	N/A
$AUC_{0-t(last)}$ (ng·hour/mL)	3410	2240	388	2010	1520	76
C_{max}, norm (1)	1.78	1.91	0.436	1.38	0.816	59
$AUC_{0-t(last)}$, norm (1)	17.1	11.2	1.94	10.1	7.64	76
R_A,C_{max}	2.02	1.49	1.38	1.63	0.339	21
R_A,$AUC_{0-t(last)}$	2.01	1.68	1.17	1.62	0.422	26
	Sulfoxide					
C_{max} (ng/mL)	12600	14300	12400	13100	1044	8
t_{max} (hour)	2	4	4	3.33	1.15	35
$AUC_{0-t(last)}$ Interval (hour)	0-24	0-8	0-8	N/A	N/A	N/A
$AUC_{0-t(last)}$ (ng·hour/mL)	125000	80800	75100	93600	27300	29
C_{max}, norm (1)	63	71.5	62	65.5	5.22	8
$AUC_{0-t(last)}$, norm (1)	627	404	376	469	138	29
(2)	33.6	35.5	134	67.9	57.7	85
(3)	34.7	34.1	183	84	85.8	102
R_A,C_{max}	1.58	1.46	1.02	1.35	0.298	22
R_A,$AUC_{0-t(last)}$	1.47	1.01	0.983	1.15	0.274	24
	Sulfone					
C_{max} (ng/mL)	19700	19900	16800	18800	1735	9
t_{max} (hour)	8	8	4	6.67	2.31	35
$AUC_{0-t(last)}$ Interval (hour)	0-24	0-24	0-24	N/A	N/A	N/A
$AUC_{0-t(last)}$ (ng·hour/mL)	301000	273000	230000	268000	35800	13
C_{max}, norm (1)	98.5	99.5	84	94	8.67	9
$AUC_{0-t(last)}$, norm (1)	1510	1370	1150	1340	181	14
(2)	49.8	46.9	173	89.8	71.9	80
(3)	79.2	109	532	240	253	105
R_A,C_{max}	2.98	2.5	1.51	2.33	0.75	32
R_A,$AUC_{0-t(last)}$	2.84	2.58	1.6	2.34	0.655	28

N/A: not applicable
(1) C_{max} (ng/mL) and AUC (ng·hour/mL) values normalized to 1 mg/kg dose.
(2) C_{max}, metabolite / C_{max}, parent; (3) $AUC_{0-t(last)}$, metabolite / $AUC_{0-t(last)}$, parent

Table 12. Day 14 individual and mean (±SD, %CV) pharmacokinetic parameters of Fexinidazole and metabolites after oral 200 mg/kg/day of Fexinidazole in female Sprague Dawley rats.

Parameter (Units)	ID 2853	ID 2854	ID 2855	Mean	SD	%CV
	Fexinidazole					
C_{max} (ng/mL)	891	746	683	773	107	14
t_{max} (hour)	2	0.5	2	1.5	0.866	58
$AUC_{0-t(last)}$ Interval (hour)	0-8	0-8	0-8	N/A	N/A	N/A
$AUC_{0-t(last)}$ (ng·hour/mL)	4240	4050	3810	4030	215	5
C_{max}, norm (1)	4.46	3.73	3.42	3.87	0.534	14
$AUC_{0-t(last)}$, norm (1)	21.2	20.3	19.1	20.2	1.05	5
R_A,C_{max}	1.07	1.31	1.66	1.35	0.294	22
R_A,$AUC_{0-t(last)}$	1.1	1.15	1.71	1.32	0.337	26
	Sulfoxide					
C_{max} (ng/mL)	20000	17600	18100	18567	1266	7
t_{max} (hour)	2	4	4	3.33	1.15	35
$AUC_{0-t(last)}$ Interval (hour)	0-8	0-8	0-24	N/A	N/A	N/A
$AUC_{0-t(last)}$ (ng·hour/mL)	127000	119000	183000	143000	34900	24
C_{max}, norm (1)	100	88	90.5	92.8	6.33	7
$AUC_{0-t(last)}$, norm (1)	635	595	917	716	176	25
(2)	21.2	22.3	25.1	22.9	1.98	9
(3)	28.3	27.8	45.4	33.8	10	30
R_A,C_{max}	1.49	1.3	1.57	1.46	0.139	10
R_A,$AUC_{0-t(last)}$	1.55	1.52	1.61	1.56	0.0418	3
	Sulfone					
C_{max} (ng/mL)	20700	19600	24300	21533	2458	11
t_{max} (hour)	8	8	8	8	0	0
$AUC_{0-t(last)}$ Interval (hour)	0-24	0-24	0-24	N/A	N/A	N/A
$AUC_{0-t(last)}$ (ng·hour/mL)	277000	302000	339000	306000	31200	10
C_{max}, norm (1)	104	98	122	108	12.5	12
$AUC_{0-t(last)}$, norm (1)	1390	1510	1690	1530	151	10
(2)	20.8	23.6	31.9	25.4	5.77	23
(3)	58.6	66.9	79.8	68.4	10.7	16
R_A,C_{max}	2.23	1.98	2.23	2.15	0.141	7
R_A,$AUC_{0-t(last)}$	2.2	2.34	2.32	2.29	0.0774	3

N/A: not applicable
(1) C_{max} (ng/mL) and AUC (ng·hour/mL) values normalized to 1 mg/kg dose.
(2) C_{max}, metabolite / C_{max}, parent; (3) $AUC_{0-t(last)}$, metabolite / $AUC_{0-t(last)}$, parent

Table 13. Day 28 individual and mean (±SD, %CV) pharmacokinetic parameters of Fexinidazole and metabolites after oral 200 mg/kg/day of Fexinidazole in male Sprague Dawley rats.

Parameter (Units)	ID 2841	ID 2842	ID 2843	Mean	SD	%CV
	Fexinidazole					
C_{max} (ng/mL)	311	353	100	255	136	53
t_{max} (hour)	1	4	2	2.33	1.53	66
AUC_{0-24} (ng·hour/mL)	4100	2290	587	2326	1757	76
$AUC_{0-t(last)}$ (ng·hour/mL)	4100	8530	587	4410	3980	90
Regr. Range (hour)	1-24	4-48	2-8	N/A	N/A	N/A
$t_{1/2,z}$ (hour)	12.6	10.3	3.34	8.75	4.82	55
C_{max}, norm (1)	1.56	1.77	0.5	1.28	0.681	53
AUC_{0-24}, norm (1)	20.5	11.5	2.94	11.6	8.78	76
$AUC_{0-t(last)}$, norm (1)	20.5	42.6	2.94	22	19.9	90
$R_{A,Cmax}$	1.77	1.38	1.59	1.58	0.194	12
$R_{A,AUC0-24}$	2.41	1.72	1.77	1.97	0.386	20
$R_{A,AUC0-t(last)}$	2.41	6.41	1.77	3.53	2.52	71
	Sulfoxide					
C_{max} (ng/mL)	9300	11500	14100	11633	2403	21
t_{max} (hour)	2	4	4	3.33	1.15	35
AUC_{0-24} (ng·hour/mL)	137000	141000	88300	122100	29340	24
$AUC_{0-t(last)}$ (ng·hour/mL)	176000	150000	88300	138000	45000	33
Regr. Range (hour)	4-48	4-48	4-8	N/A	N/A	N/A
$t_{1/2,z}$ (hour)	5.86	9.31	3.57	6.25	2.89	46
C_{max}, norm (1)	46.5	57.5	70.5	58.2	12	21
AUC_{0-24}, norm (1)	685	705	441	610	147	24
$AUC_{0-t(last)}$, norm (1)	880	749	441	690	225	33
(2)	28.3	30.8	133	64.2	59.9	93
(3)	40.6	16.6	142	66.5	66.7	100
$R_{A,Cmax}$	1.17	1.17	1.16	1.17	0.00892	1
$R_{A,AUC0-24}$	1.61	1.76	1.16	1.51	0.315	21
$R_{A,AUC0-t(last)}$	2.07	1.87	1.16	1.7	0.481	28

Table 13. (cont'd). Day 28 individual and mean (±SD, %CV) pharmacokinetic parameters of Fexinidazole and metabolites after oral 200 mg/kg/day of Fexinidazole in male Sprague Dawley rats.

Parameter (Units)	ID 2841	ID 2842	ID 2843	Mean	SD	%CV
	Sulfone					
C_{max} (ng/mL)	22200	22700	27700	24200	3041	13
t_{max} (hour)	8	8	8	8	0	0
AUC_{0-24} (ng·hour/mL)	344000	301000	379000	341333	39068	11
$AUC_{0-t(last)}$ (ng·hour/mL)	420000	352000	388000	387000	34000	9
Regr. Range (hour)	8-72	8-72	8-48	N/A	N/A	N/A
$t_{1/2,z}$ (hour)	7.58	9.93	5.06	7.52	2.44	32
C_{max}, norm (1)	111	114	139	121	15.4	13
AUC_{0-24}, norm (1)	1720	1505	1895	1707	195	11
$AUC_{0-t(last)}$, norm (1)	2100	1760	1940	1930	170	9
(2)	64	57.7	249	123	108	88
(3)	91.9	37	593	241	306	127
R_A,C_{max}	3.36	2.86	2.5	2.9	0.436	15
R_A,AUC_{0-24}	3.25	2.84	2.63	2.91	0.312	11
R_A,$AUC_{0-t(last)}$	3.96	3.32	2.69	3.33	0.634	19

N/A: not applicable;
(1) C_{max} (ng/mL) and AUC (ng·hour/mL) values normalized to 1 mg/kg dose;
(2) C_{max}, metabolite / C_{max}, parent;
(3) $AUC_{0-t(last)}$, metabolite / $AUC_{0-t(last)}$, parent

Table 14. Day 28 individual and mean (±SD, %CV) pharmacokinetic parameters of Fexinidazole and metabolites after oral 200 mg/kg/day of Fexinidazole in female Sprague Dawley rats.

Parameter (Units)	ID 2853	ID 2854	ID 2855	Mean	SD	%CV
	Fexinidazole					
C_{max} (ng/mL)	943	962	803	903	86.8	10
t_{max} (hour)	2	2	1	1.67	0.577	35
AUC_{0-24} (ng·hour/mL)	5030	4160	4120	4437	514	12
$AUC_{0-t(last)}$ (ng·hour/mL)	5030	4160	5050	4750	508	11
Regr. Range (hour)	2-8	2-8	8-48	N/A	N/A	N/A
$t_{1/2,z}$ (hour)	4.22	3.24	11.5	6.32	4.51	71
C_{max}, norm (1)	4.72	4.81	4.02	4.52	0.432	10
AUC_{0-24}, norm (1)	25.1	20.8	20.6	22.2	2.54	11
$AUC_{0-t(last)}$, norm (1)	25.1	20.8	25.3	23.7	2.54	11
R_A,C_{max}	1.14	1.69	1.95	1.59	0.415	26
R_A,AUC_{0-24}	1.31	1.18	1.85	1.45	0.354	24
R_A,$AUC_{0-t(last)}$	1.31	1.18	2.26	1.58	0.592	37
	Sulfoxide					
C_{max} (ng/mL)	21200	21800	24000	22333	1474	7
t_{max} (hour)	2	2	1	1.67	0.577	35
AUC_{0-24} (ng·hour/mL)	141000	123000	151000	138333	14189	10
$AUC_{0-t(last)}$ (ng·hour/mL)	141000	123000	194000	153000	36900	24
Regr. Range (hour)	4-8	2-8	4-48	N/A	N/A	N/A
$t_{1/2,z}$ (hour)	6.88	5.74	11.6	8.07	3.11	39
C_{max}, norm (1)	106	109	120	112	7.37	7
AUC_{0-24}, norm (1)	703	613	755	690	71.8	10
$AUC_{0-t(last)}$, norm (1)	703	613	968	761	185	24
(2)	21.3	21.4	28.3	23.7	4	17
(3)	26.5	28	36.3	30.3	5.3	18
R_A,C_{max}	1.58	1.61	2.09	1.76	0.283	16
R_A,AUC_{0-24}	1.72	1.57	1.32	1.54	0.2	13
R_A,$AUC_{0-t(last)}$	1.72	1.57	1.7	1.67	0.0789	5

Table 14. (cont'd). Day 28 individual and mean (±SD, %CV) pharmacokinetic parameters of Fexinidazole and metabolites after oral 200 mg/kg/day of Fexinidazole in female Sprague Dawley rats.

Parameter (Units)	ID 2853	ID 2854	ID 2855	Mean	SD	%CV
	Sulfone					
C_{max} (ng/mL)	26200	19700	18400	21433	4179	20
t_{max} (hour)	8	8	8	8	0	0
AUC_{0-24} (ng·hour/mL)	405000	273000	346000	341333	66124	19
$AUC_{0-t(last)}$ (ng·hour/mL)	507000	294000	544000	448000	135000	30
Regr. Range (hour)	8-72	8-48	8-72	N/A	N/A	N/A
$t_{1/2,z}$ (hour)	6.48	5.15	10.2	7.28	2.62	36
C_{max}, norm (1)	131	98.5	92	107	20.9	20
AUC_{0-24}, norm (1)	2025	1365	1730	1707	331	19
$AUC_{0-t(last)}$, norm (1)	2530	1470	2720	2240	674	30
(2)	24.9	18.4	20.6	21.3	3.34	16
(3)	90.4	63.4	96.6	83.5	17.7	21
R_A,C_{max}	2.82	1.99	1.69	2.17	0.586	27
R_A,AUC_{0-24}	3.21	2.12	2.37	2.57	0.575	22
R_A,$AUC_{0-t(last)}$	4.02	2.28	3.73	3.34	0.933	28

N/A: not applicable;
(1) C_{max} (ng/mL) and AUC (ng·hour/mL) values normalized to 1 mg/kg dose;
(2) C_{max}, metabolite / C_{max}, parent;
(3) $AUC_{0-t(last)}$, metabolite / $AUC_{0-t(last)}$, parent.

Table 15. Day 1 individual and mean (±SD, %CV) pharmacokinetic parameters of Fexinidazole and metabolites after oral 800 mg/kg/day of Fexinidazole in male Sprague Dawley rats.

Parameter (Units)	ID 2844	ID 2845	ID 2846	Mean	SD	%CV
	Fexinidazole					
C_{max} (ng/mL)	805	868	2760	1478	1111	75
t_{max} (hour)	2	2	0.5	1.5	0.866	58
$AUC_{0\text{-}t(last)}$ Interval (hour)	0-24	0-24	0-8	N/A	N/A	N/A
$AUC_{0\text{-}t(last)}$ (ng·hour/mL)	8770	8750	12500	10000	2160	22
C_{max}, norm (1)	1.01	1.09	3.45	1.85	1.39	75
$AUC_{0\text{-}t(last)}$, norm (1)	11	10.9	15.6	12.5	2.69	22
	Sulfoxide					
C_{max} (ng/mL)	36500	27200	34800	32833	4952	15
t_{max} (hour)	4	4	8	5.33	2.31	43
$AUC_{0\text{-}t(last)}$ Interval (hour)	0-24	0-24	0-8	N/A	N/A	N/A
$AUC_{0\text{-}t(last)}$ (ng·hour/mL)	462000	394000	242000	366000	113000	31
C_{max}, norm (1)	45.6	34	43.5	41	6.18	15
$AUC_{0\text{-}t(last)}$, norm (1)	577	493	302	457	141	31
(2)	42.9	29.6	11.9	28.1	15.5	55
(3)	49.8	42.6	18.3	36.9	16.5	45
	Sulfone					
C_{max} (ng/mL)	39500	42100	39300	40300	1562	4
t_{max} (hour)	8	8	8	8	0	0
$AUC_{0\text{-}t(last)}$ Interval (hour)	0-24	0-24	0-8	N/A	N/A	N/A
$AUC_{0\text{-}t(last)}$ (ng·hour/mL)	613000	621000	160000	465000	264000	57
C_{max}, norm (1)	49.4	52.6	49.1	50.4	1.94	4
$AUC_{0\text{-}t(last)}$, norm (1)	766	777	200	581	330	57
(2)	44	43.5	12.8	33.4	17.9	54
(3)	62.7	63.7	11.5	46	29.9	65

N/A: not applicable
(1) C_{max} (ng/mL) and AUC (ng·hour/mL) values normalized to 1 mg/kg dose.
(2) C_{max}, metabolite / C_{max}, parent; (3) $AUC_{0\text{-}t(last)}$, metabolite / $AUC_{0\text{-}t(last)}$, parent

Table 16. Day 1 individual and mean (±SD, %CV) pharmacokinetic parameters of Fexinidazole and metabolites after oral 800 mg/kg/day of Fexinidazole in female Sprague Dawley rats.

Parameter (Units)	ID 2856	ID 2857	ID 2858	Mean	SD	%CV
	Fexinidazole					
C_{max} (ng/mL)	1690	1290	1470	1483	200	14
t_{max} (hour)	2	2	1	1.67	0.577	35
$AUC_{0-t(last)}$ Interval (hour)	0-24	0-24	0-24	N/A	N/A	N/A
$AUC_{0-t(last)}$ (ng·hour/mL)	16700	18700	11700	15700	3610	23
C_{max}, norm (1)	2.11	1.61	1.84	1.85	0.25	14
$AUC_{0-t(last)}$, norm (1)	20.9	23.4	14.6	19.6	4.53	23
	Sulfoxide					
C_{max} (ng/mL)	28200	30300	33400	30633	2616	9
t_{max} (hour)	4	4	4	4	0	0
$AUC_{0-t(last)}$ Interval (hour)	0-24	0-24	0-24	N/A	N/A	N/A
$AUC_{0-t(last)}$ (ng·hour/mL)	408000	551000	401000	453000	84700	19
C_{max}, norm (1)	35.3	37.9	41.8	38.3	3.27	9
$AUC_{0-t(last)}$, norm (1)	510	689	501	567	106	19
(2)	15.8	22.2	21.5	19.8	3.52	18
(3)	23.1	27.9	32.4	27.8	4.65	17
	Sulfone					
C_{max} (ng/mL)	29400	67600	37500	44833	20128	45
t_{max} (hour)	8	24	8	13.3	9.24	69
$AUC_{0-t(last)}$ Interval (hour)	0-24	0-24	0-24	N/A	N/A	N/A
$AUC_{0-t(last)}$ (ng·hour/mL)	542000	961000	545000	683000	241000	35
C_{max}, norm (1)	36.8	84.5	46.9	56.1	25.1	45
$AUC_{0-t(last)}$, norm (1)	678	1200	681	853	301	35
(2)	15.6	47	22.9	28.5	16.4	58
(3)	29.1	46.1	41.8	39	8.83	23

N/A: not applicable
(1) C_{max} (ng/mL) and AUC (ng·hour/mL) values normalized to 1 mg/kg dose.
(2) C_{max}, metabolite / C_{max}, parent; (3) $AUC_{0-t(last)}$, metabolite / $AUC_{0-t(last)}$, parent

Table 17. Day 14 individual and mean (±SD, %CV) pharmacokinetic parameters of Fexinidazole and metabolites after oral 800 mg/kg/day of Fexinidazole in male Sprague Dawley rats.

Parameter (Units)	ID 2844	ID 2845	ID 2846	Mean	SD	%CV
	Fexinidazole					
C_{max} (ng/mL)	678	551	467	565	106	19
t_{max} (hour)	4	2	4	3.33	1.15	35
$AUC_{0-t(last)}$ Interval (hour)	0-24	0-24	0-24	N/A	N/A	N/A
$AUC_{0-t(last)}$ (ng·hour/mL)	6890	4240	4220	5120	1540	30
C_{max}, norm (1)	0.848	0.689	0.584	0.707	0.133	19
$AUC_{0-t(last)}$, norm (1)	8.61	5.3	5.27	6.39	1.92	30
R_A,C_{max}	0.842	0.635	0.169	0.549	0.345	63
R_A,$AUC_{0-t(last)}$	0.786	0.485	0.338	0.536	0.228	43
	Sulfoxide					
C_{max} (ng/mL)	17800	19400	16000	17733	1701	10
t_{max} (hour)	4	2	4	3.33	1.15	35
$AUC_{0-t(last)}$ Interval (hour)	0-24	0-24	0-24	N/A	N/A	N/A
$AUC_{0-t(last)}$ (ng·hour/mL)	195000	137000	163000	165000	29100	18
C_{max}, norm (1)	22.3	24.3	20	22.2	2.15	10
$AUC_{0-t(last)}$, norm (1)	244	171	203	206	36.6	18
(2)	24.8	33.3	32.4	30.2	4.65	15
(3)	26.8	30.6	36.5	31.3	4.92	16
R_A,C_{max}	0.488	0.713	0.46	0.554	0.139	25
R_A,$AUC_{0-t(last)}$	0.422	0.348	0.674	0.481	0.171	35
	Sulfone					
C_{max} (ng/mL)	32700	29300	30500	30833	1724	6
t_{max} (hour)	8	4	4	5.33	2.31	43
$AUC_{0-t(last)}$ Interval (hour)	0-24	0-24	0-24	N/A	N/A	N/A
$AUC_{0-t(last)}$ (ng·hour/mL)	493000	352000	423000	423000	70500	17
C_{max}, norm (1)	40.9	36.6	38.1	38.5	2.18	6
$AUC_{0-t(last)}$, norm (1)	616	440	529	528	88	17
(2)	43.3	47.7	58.6	49.9	7.89	16
(3)	64.2	74.5	89.9	76.2	13	17
R_A,C_{max}	0.828	0.696	0.776	0.767	0.0664	9
R_A,$AUC_{0-t(last)}$	0.804	0.567	2.64	1.34	1.14	85

N/A: not applicable
(1) C_{max} (ng/mL) and AUC (ng·hour/mL) values normalized to 1 mg/kg dose.
(2) C_{max}, metabolite / C_{max}, parent; (3) $AUC_{0-t(last)}$, metabolite / $AUC_{0-t(last)}$, parent

Table 18. Day 14 individual and mean (±SD, %CV) pharmacokinetic parameters of Fexinidazole and metabolites after oral 800 mg/kg/day of Fexinidazole in female Sprague Dawley rats.

Parameter (Units)	ID 2856	ID 2857	ID 2858	Mean	SD	%CV
	Fexinidazole					
C_{max} (ng/mL)	624	1070	2740	1478	1115	76
t_{max} (hour)	0.5	8	0.5	3	4.33	144
$AUC_{0\text{-}t(last)}$ Interval (hour)	0-24	0-24	0-8	N/A	N/A	N/A
$AUC_{0\text{-}t(last)}$ (ng·hour/mL)	9380	16300	14700	13500	3620	27
C_{max}, norm (1)	0.78	1.34	3.43	1.85	1.4	76
$AUC_{0\text{-}t(last)}$, norm (1)	11.7	20.4	18.4	16.8	4.56	27
R_A,C_{max}	0.369	0.829	1.86	1.02	0.766	75
R_A,$AUC_{0\text{-}t(last)}$	0.562	0.872	1.26	0.897	0.348	39
	Sulfoxide					
C_{max} (ng/mL)	25100	34000	37600	32233	6435	20
t_{max} (hour)	2	2	2	2	0	0
$AUC_{0\text{-}t(last)}$ Interval (hour)	0-24	0-24	0-8	N/A	N/A	N/A
$AUC_{0\text{-}t(last)}$ (ng·hour/mL)	365000	560000	244000	390000	159000	41
C_{max}, norm (1)	31.4	42.5	47	40.3	8.03	20
$AUC_{0\text{-}t(last)}$, norm (1)	456	700	305	487	199	41
(2)	38	30.1	13	27	12.8	47
(3)	36.8	32.5	15.7	28.3	11.2	39
R_A,C_{max}	0.89	1.12	1.13	1.05	0.135	13
R_A,$AUC_{0\text{-}t(last)}$	0.895	1.02	0.608	0.84	0.209	25
	Sulfone					
C_{max} (ng/mL)	38500	61800	50400	50233	11651	23
t_{max} (hour)	8	8	8	8	0	0
$AUC_{0\text{-}t(last)}$ Interval (hour)	0-24	0-24	0-8	N/A	N/A	N/A
$AUC_{0\text{-}t(last)}$ (ng·hour/mL)	674000	990000	327000	664000	332000	50
C_{max}, norm (1)	48.1	77.3	63	62.8	14.6	23
$AUC_{0\text{-}t(last)}$, norm (1)	842	1240	409	830	416	50
(2)	55.4	51.8	16.5	41.2	21.5	52
(3)	64.5	54.5	20	46.3	23.4	50
R_A,C_{max}	1.31	0.914	1.34	1.19	0.239	20
R_A,$AUC_{0\text{-}t(last)}$	1.24	1.03	0.6	0.958	0.328	34

N/A: not applicable
(1) C_{max} (ng/mL) and AUC (ng·hour/mL) values normalized to 1 mg/kg dose.
(2) C_{max}, metabolite / C_{max}, parent; (3) $AUC_{0\text{-}t(last)}$, metabolite / $AUC_{0\text{-}t(last)}$, parent

Table 19. Day 28 individual and mean (±SD, %CV) pharmacokinetic parameters of Fexinidazole and metabolites after oral 800 mg/kg/day of Fexinidazole in male Sprague Dawley rats.

Parameter (Units)	ID 2844	ID 2845	ID 2846	Mean	SD	%CV
	Fexinidazole					
C_{max} (ng/mL)	670	448	451	523	127	24
t_{max} (hour)	2	1	4	2.33	1.53	66
AUC_{0-24} (ng·hour/mL)	4000	2600	2920	3173	734	23
$AUC_{0-t(last)}$ (ng·hour/mL)	18100	8880	8770	11900	5360	45
Regr. Range (hour)	8-48	1-48	1-48	N/A	N/A	N/A
$t_{1/2,z}$ (hour)	14	8.63	11.2	11.3	2.69	24
C_{max}, norm (1)	0.838	0.56	0.564	0.654	0.159	24
AUC_{0-24}, norm (1)	5	3.25	3.65	3.97	0.917	23
$AUC_{0-t(last)}$, norm (1)	22.6	11.1	11	14.9	6.67	45
R_A,C_{max}	0.832	0.516	0.163	0.504	0.335	66
R_A,AUC_{0-24}	0.456	0.297	0.234	0.329	0.115	35
R_A,$AUC_{0-t(last)}$	2.06	1.01	0.702	1.26	0.713	57
	Sulfoxide					
C_{max} (ng/mL)	18100	14200	17400	16567	2079	13
t_{max} (hour)	2	1	2	1.67	0.577	35
AUC_{0-24} (ng·hour/mL)	194000	86200	109000	129733	56812	44
$AUC_{0-t(last)}$ (ng·hour/mL)	194000	303000	307000	268000	64100	24
Regr. Range (hour)	8-24	1-72	4-72	N/A	N/A	N/A
$t_{1/2,z}$ (hour)	1.9	9.59	8.31	6.6	4.12	62
C_{max}, norm (1)	22.6	17.8	21.8	20.7	2.57	12
AUC_{0-24}, norm (1)	243	108	136	162	71	44
$AUC_{0-t(last)}$, norm (1)	243	379	384	335	80	24
(2)	25.6	30	36.5	30.7	5.5	18
(3)	10.1	32.3	33.1	25.2	13	52
R_A,C_{max}	0.496	0.522	0.5	0.506	0.0141	3
R_A,AUC_{0-24}	0.42	0.219	0.45	0.363	0.126	35
R_A,$AUC_{0-t(last)}$	0.42	0.769	1.27	0.819	0.427	52

Table 19. (cont'd). Day 28 individual and mean (±SD, %CV) pharmacokinetic parameters of Fexinidazole and metabolites after oral 800 mg/kg/day of Fexinidazole in male Sprague Dawley rats.

Parameter (Units)	ID 2844	ID 2845	ID 2846	Mean	SD	%CV
	Sulfone					
C_{max} (ng/mL)	30700	27800	40500	33000	6655	20
t_{max} (hour)	8	4	4	5.33	2.31	43
AUC_{0-24} (ng·hour/mL)	443000	408000	526000	459000	60605	13
$AUC_{0-t(last)}$ (ng·hour/mL)	460000	448000	618000	509000	94900	19
Regr. Range (hour)	8-72	8-72	4-72	N/A	N/A	N/A
$t_{1/2,z}$ (hour)	7.21	13.2	9.74	10.1	3.01	30
C_{max}, norm (1)	38.4	34.8	50.6	41.3	8.28	20
AUC_{0-24}, norm (1)	554	510	658	574	75.8	13
$AUC_{0-t(last)}$, norm (1)	575	561	773	636	119	19
(2)	41.1	55.7	80.6	59.1	20	34
(3)	22.8	45.3	63.2	43.8	20.3	46
R_A,C_{max}	0.777	0.66	1.03	0.823	0.189	23
R_A,AUC_{0-24}	0.723	0.657	3.29	1.56	1.5	96
R_A,$AUC_{0-t(last)}$	0.75	0.721	3.86	1.78	1.81	102

N/A: not applicable;
(1) C_{max} (ng/mL) and AUC (ng·hour/mL) values normalized to 1 mg/kg dose
(2) C_{max}, metabolite / C_{max}, parent;
(3) $AUC_{0-t(last)}$, metabolite / $AUC_{0-t(last)}$, parent

Table 20. Day 28 individual and mean (±SD, %CV) pharmacokinetic parameters of Fexinidazole and metabolites after oral 800 mg/kg/day of Fexinidazole in female Sprague Dawley rats.

Parameter (Units)	ID 2856	ID 2857	Mean	SD	%CV
	Fexinidazole				
C_{max} (ng/mL)	685	812	749	89.8	12
t_{max} (hour)	2	1	1.5	0.707	47
AUC_{0-24} (ng·hour/mL)	6380	8900	7640	1782	23
$AUC_{0-t(last)}$ (ng·hour/mL)	6380	9500	7940	2210	28
Regr. Range (hour)	2-24	1-48	N/A	N/A	N/A
$t_{1/2,z}$ (hour)	5.13	8.35	6.74	2.28	34
C_{max}, norm (1)	0.856	1.02	0.938	0.116	12
AUC_{0-24}, norm (1)	7.97	11.1	9.55	2.23	23
$AUC_{0-t(last)}$, norm (1)	7.97	11.9	9.94	2.78	28
R_A,C_{max}	0.405	0.629	0.517	0.158	31
R_A,AUC_{0-24}	0.382	0.476	0.429	0.0664	15
R_A,$AUC_{0-t(last)}$	0.382	0.508	0.445	0.0891	20
	Sulfoxide				
C_{max} (ng/mL)	24400	27300	25850	2051	8
t_{max} (hour)	2	4	3	1.41	47
AUC_{0-24} (ng·hour/mL)	263000	320000	291500	40305	14
$AUC_{0-t(last)}$ (ng·hour/mL)	290000	371000	331000	57300	17
Regr. Range (hour)	4-48	4-72	N/A	N/A	N/A
$t_{1/2,z}$ (hour)	6.99	10.2	8.6	2.27	26
C_{max}, norm (1)	30.5	34.1	32.3	2.55	8
AUC_{0-24}, norm (1)	329	400	364	50.4	14
$AUC_{0-t(last)}$, norm (1)	362	463	413	71.4	17
(2)	33.7	31.8	32.7	1.34	4
(3)	43	36.9	40	4.28	11
R_A,C_{max}	0.865	0.901	0.883	0.0253	3
R_A,AUC_{0-24}	0.645	0.581	0.613	0.0451	7
R_A,$AUC_{0-t(last)}$	0.711	0.673	0.692	0.0265	4

Table 20. (cont'd). Day 28 individual and mean (±SD, %CV) pharmacokinetic parameters of Fexinidazole and metabolites after oral 800 mg/kg/day of Fexinidazole in female Sprague Dawley rats.

Parameter (Units)	ID 2856	ID 2857	Mean	SD	%CV
	Sulfone				
C_{max} (ng/mL)	44000	39800	41900	2970	7
t_{max} (hour)	4	8	6	2.83	47
AUC_{0-24} (ng·hour/mL)	533000	610000	571500	54447	10
$AUC_{0-t(last)}$ (ng·hour/mL)	642000	833000	738000	135000	18
Regr. Range (hour)	4-72	8-72	N/A	N/A	N/A
$t_{1/2,z}$ (hour)	6.83	14.7	10.8	5.56	52
C_{max}, norm (1)	55	49.8	52.4	3.68	7
AUC_{0-24}, norm (1)	666	763	714	68.1	10
$AUC_{0-t(last)}$, norm (1)	803	1040	922	168	18
(2)	57.6	44	50.8	9.66	19
(3)	90.3	78.7	84.5	8.21	10
R_A,C_{max}	1.5	0.589	1.04	0.642	62
R_A,AUC_{0-24}	0.983	0.635	0.809	0.247	30
R_A,$AUC_{0-t(last)}$	1.18	0.867	1.03	0.225	22

N/A: not applicable;
(1) C_{max} (ng/mL) and AUC (ng·hour/mL) values normalized to 1 mg/kg dose
(2) C_{max}, metabolite / C_{max}, parent;
(3) $AUC_{0-t(last)}$, metabolite / $AUC_{0-t(last)}$, parent.

Figure 1. Day 1 individual plasma concentrations (ng/mL) of Fexinidazole and metabolites after oral 50 mg/kg/day dose of Fexinidazole in male (upper panel) and female (lower panel) Sprague Dawley rats.

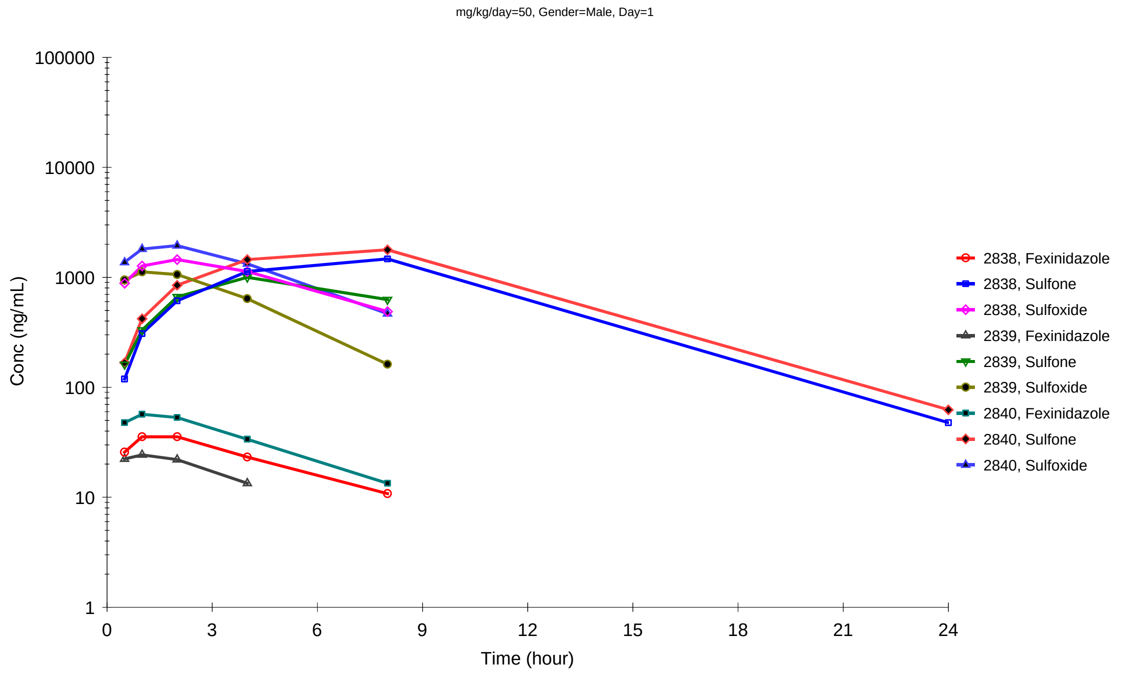

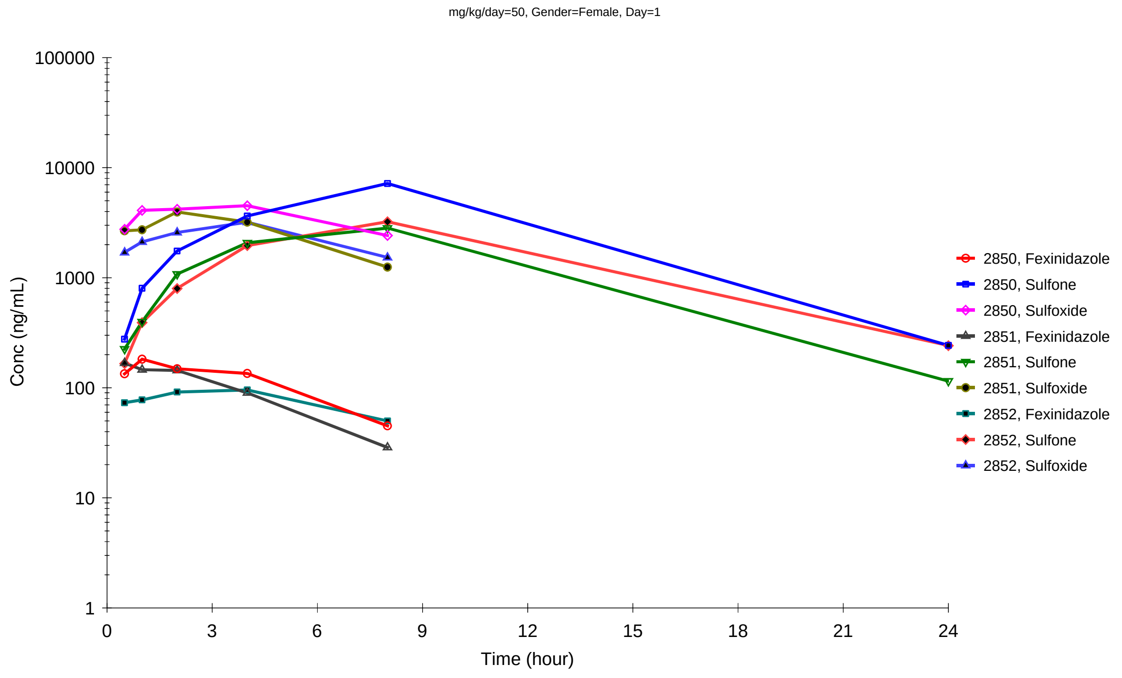

Figure 2. Day 14 individual plasma concentrations (ng/mL) of Fexinidazole and metabolites after oral 50 mg/kg/day dose of Fexinidazole in male (upper panel) and female (lower panel) Sprague Dawley rats.

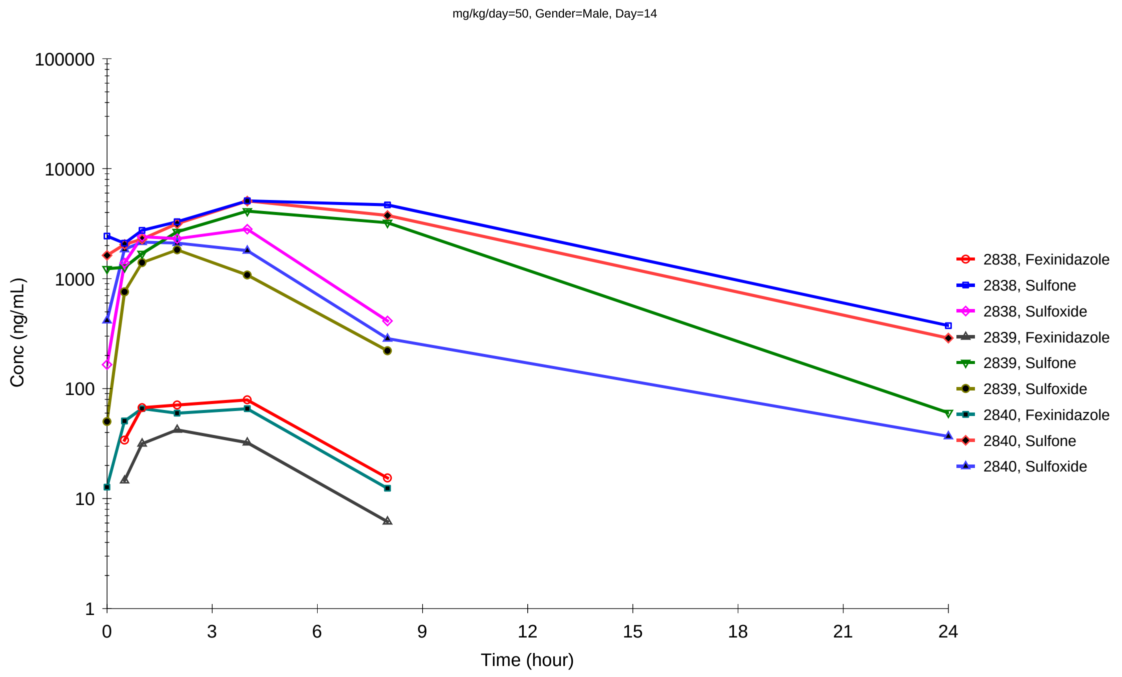

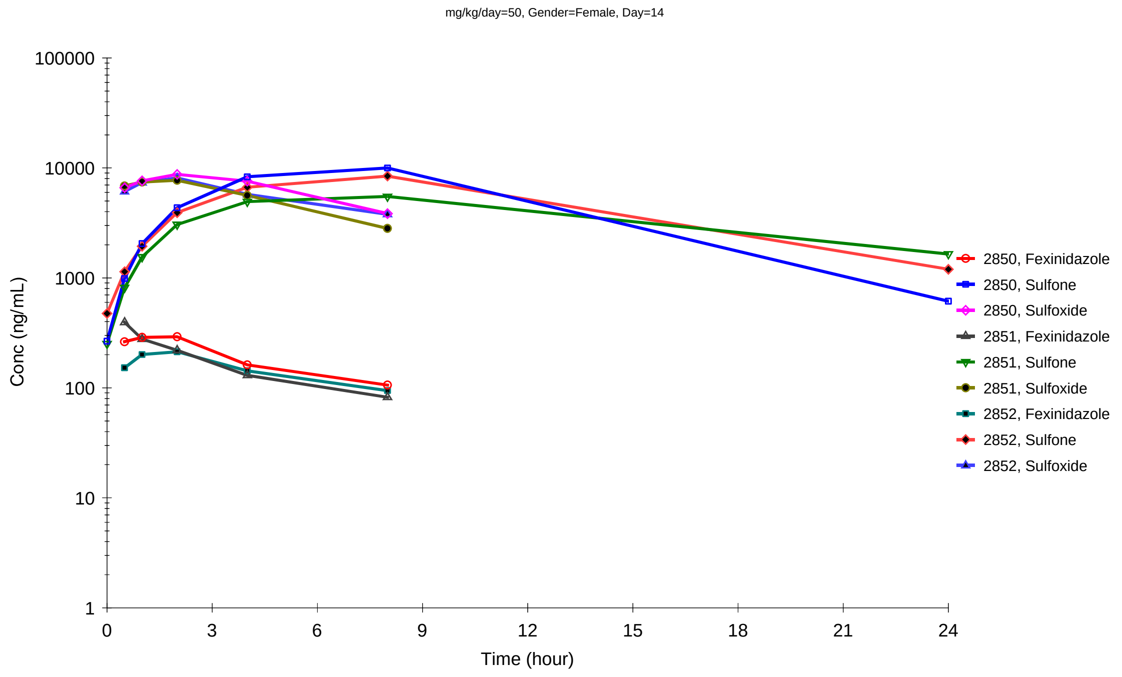

Figure 3. Day 28 individual plasma concentrations (ng/mL) of Fexinidazole and metabolites after oral 50 mg/kg/day dose of Fexinidazole in male (upper panel) and female (lower panel) Sprague Dawley rats.

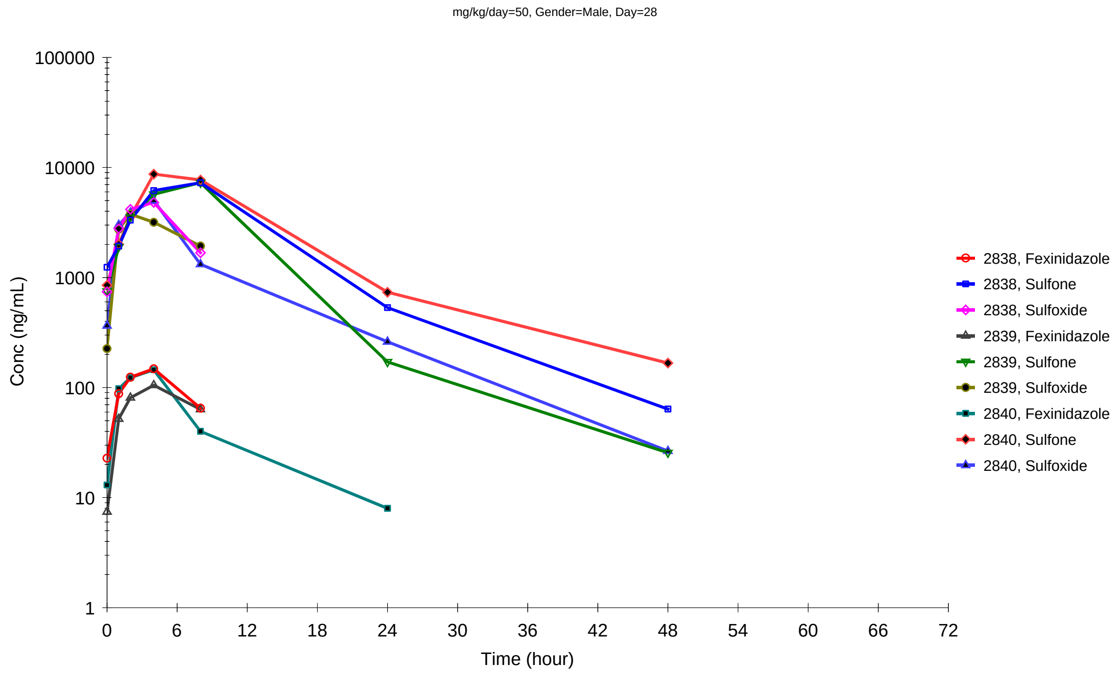

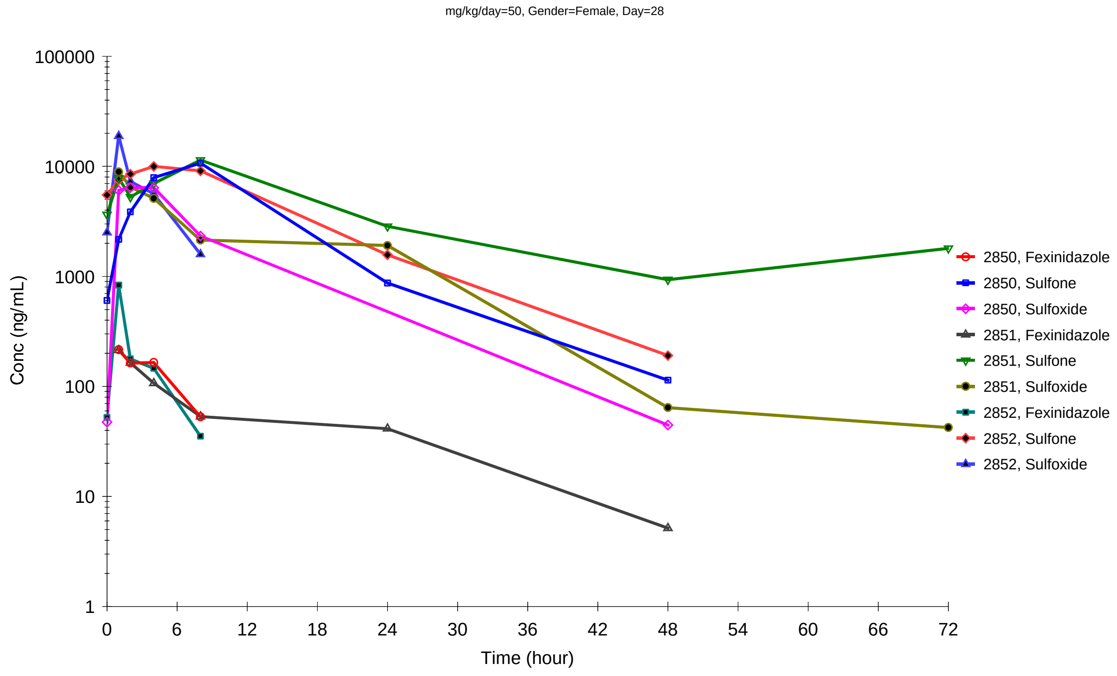

Figure 4. Day 1 individual plasma concentrations (ng/mL) of Fexinidazole and metabolites after oral 200 mg/kg/day dose of Fexinidazole in male (upper panel) and female (lower panel) Sprague Dawley rats.

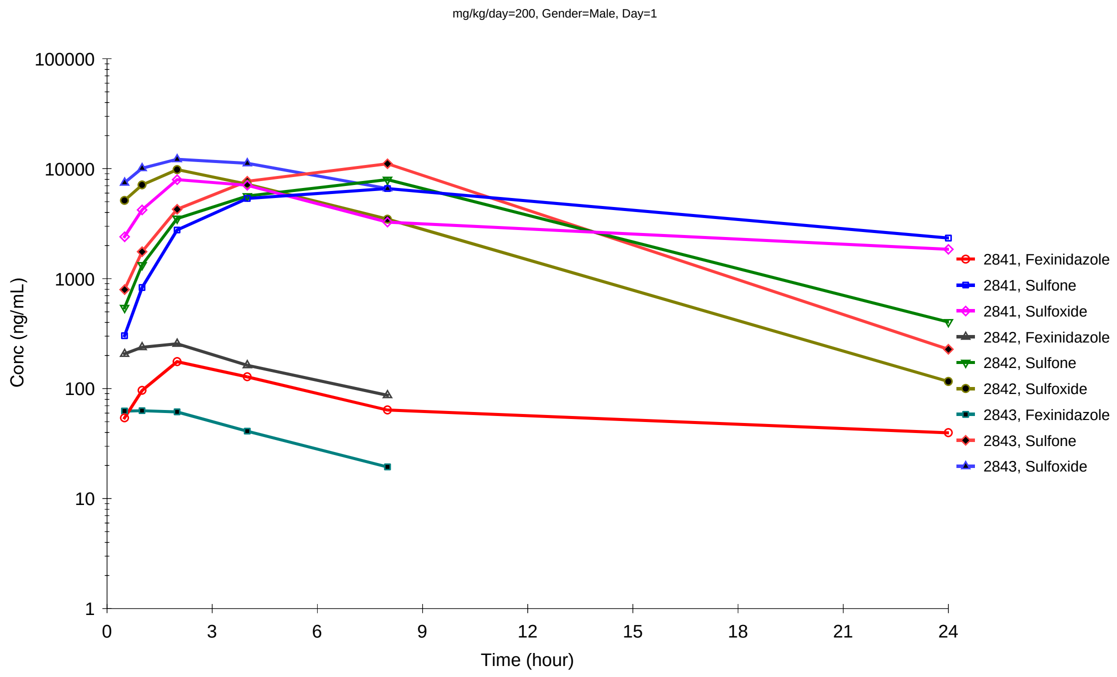

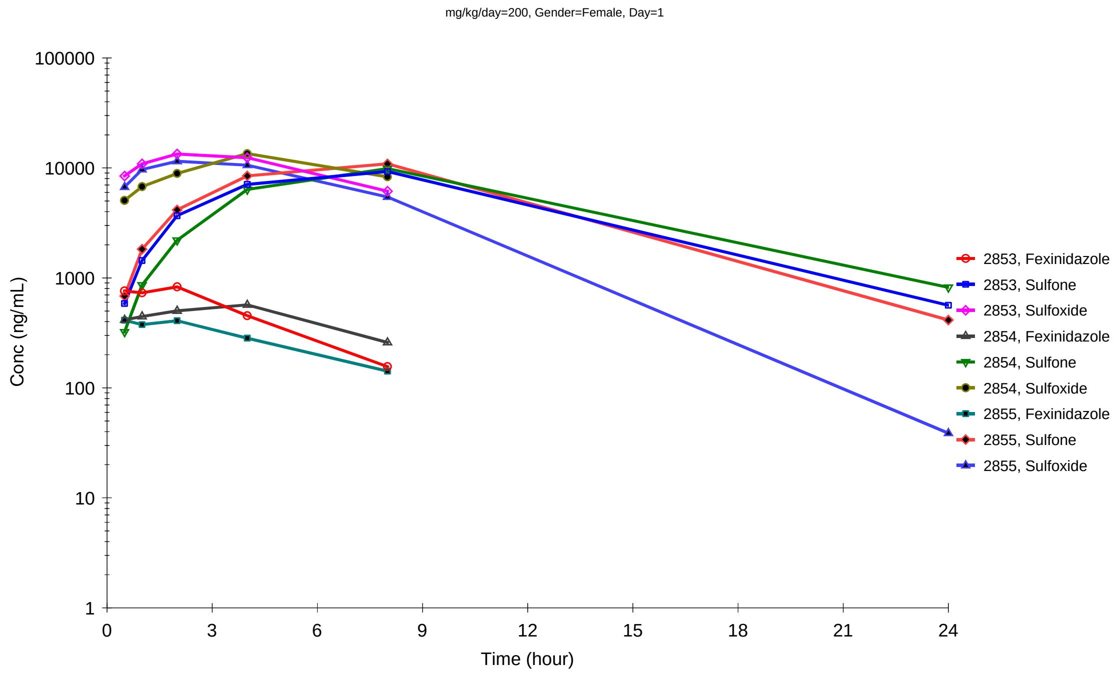

Figure 5. Day 14 individual plasma concentrations (ng/mL) of Fexinidazole and metabolites after oral 200 mg/kg/day dose of Fexinidazole in male (upper panel) and female (lower panel) Sprague Dawley rats.

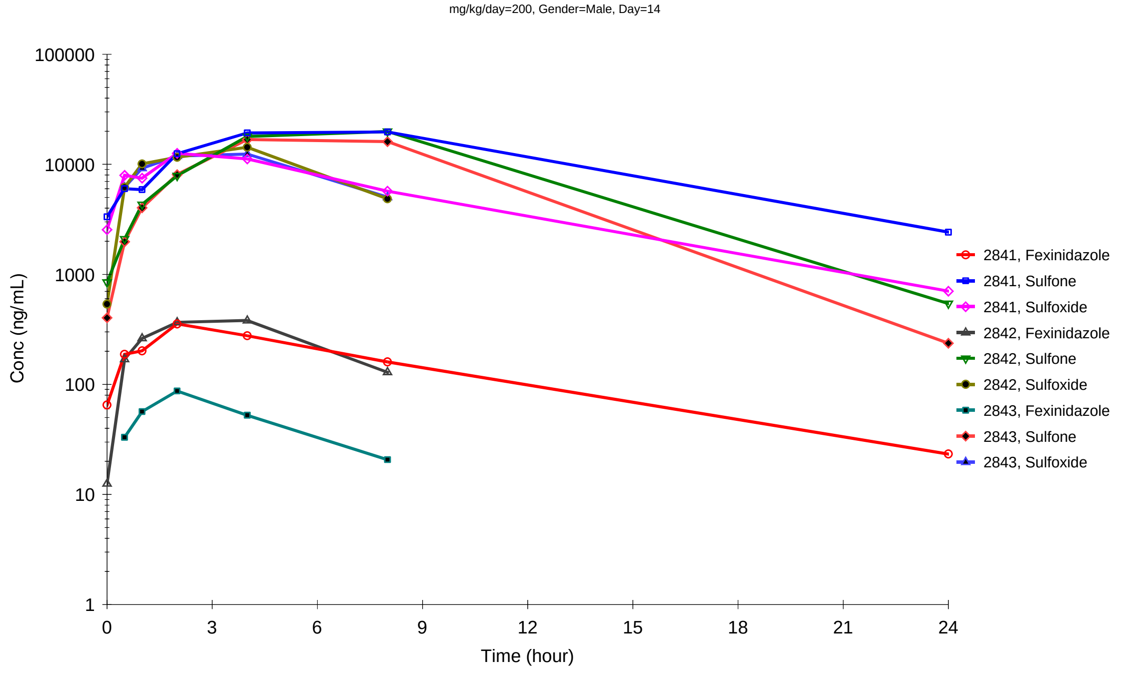

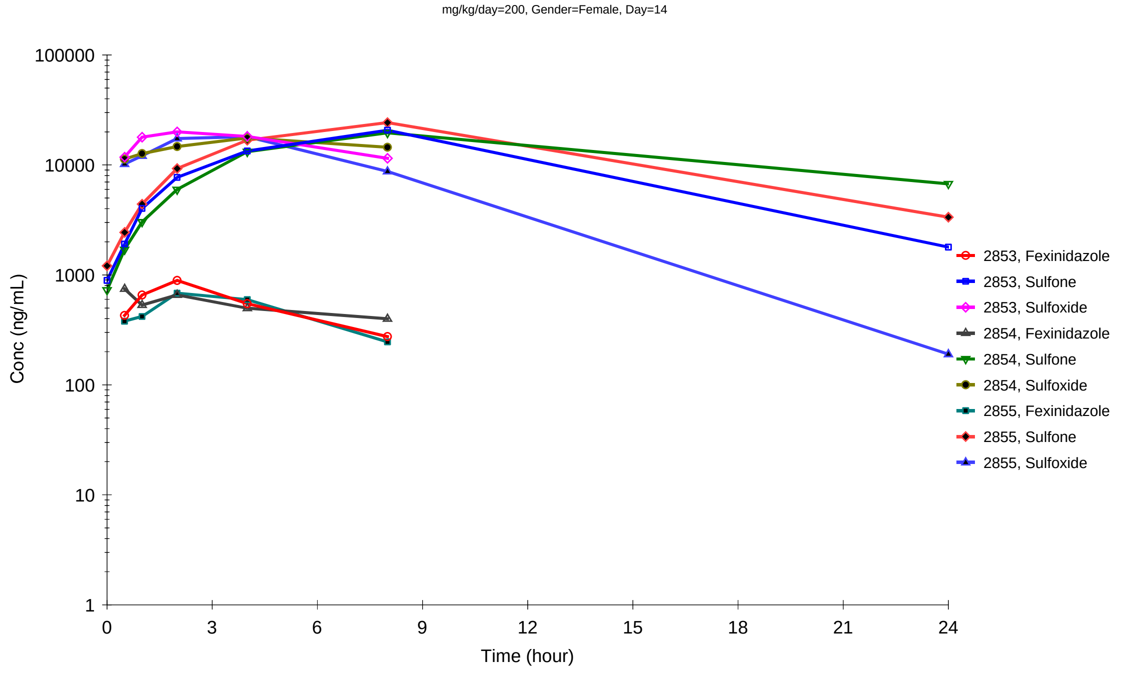

Figure 6. Day 28 individual plasma concentrations (ng/mL) of Fexinidazole and metabolites after oral 200 mg/kg/day dose of Fexinidazole in male (upper panel) and female (lower panel) Sprague Dawley rats.

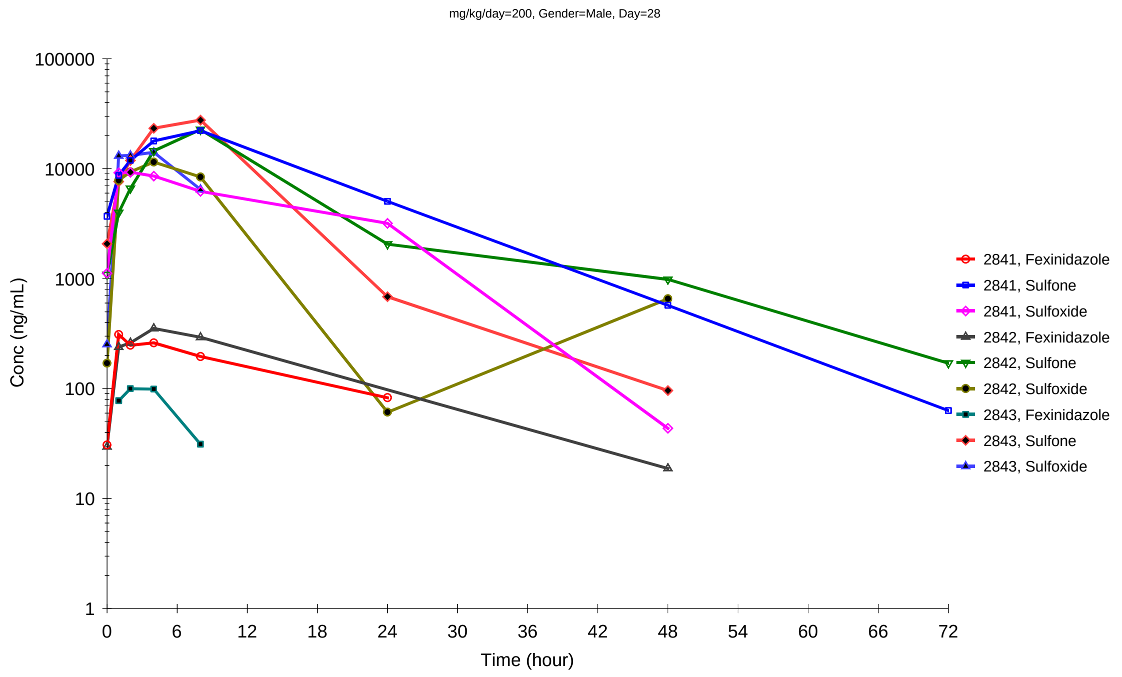

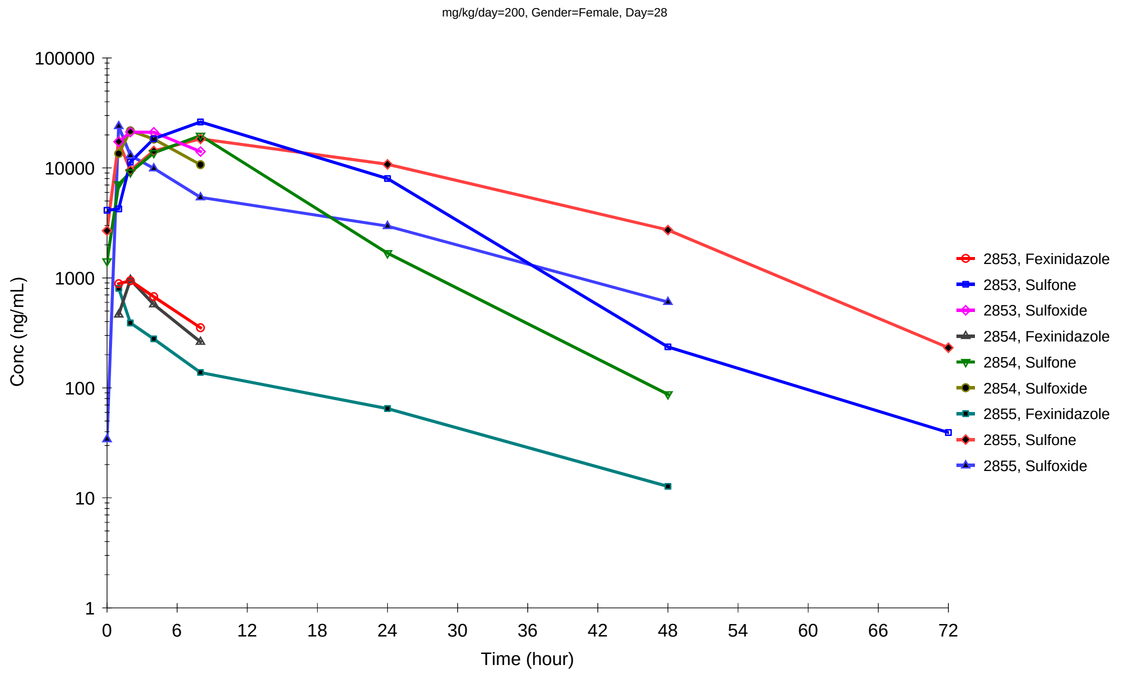

Figure 7. Day 1 individual plasma concentrations (ng/mL) of Fexinidazole and metabolites after oral 800 mg/kg/day dose of Fexinidazole in male (upper panel) and female (lower panel) Sprague Dawley rats.

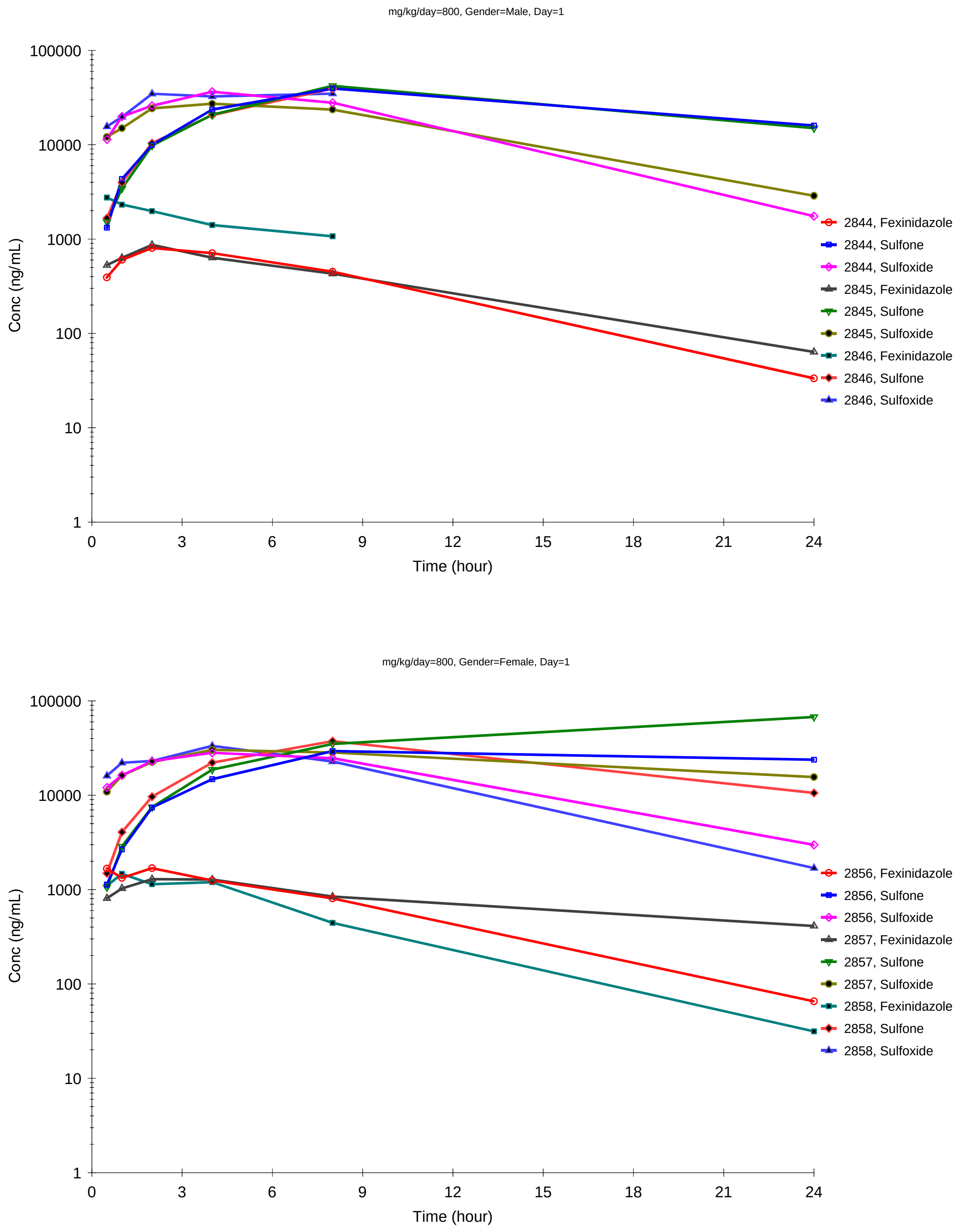

Figure 8. Day 14 individual plasma concentrations (ng/mL) of Fexinidazole and metabolites after oral 800 mg/kg/day dose of Fexinidazole in male (upper panel) and female (lower panel) Sprague Dawley rats.

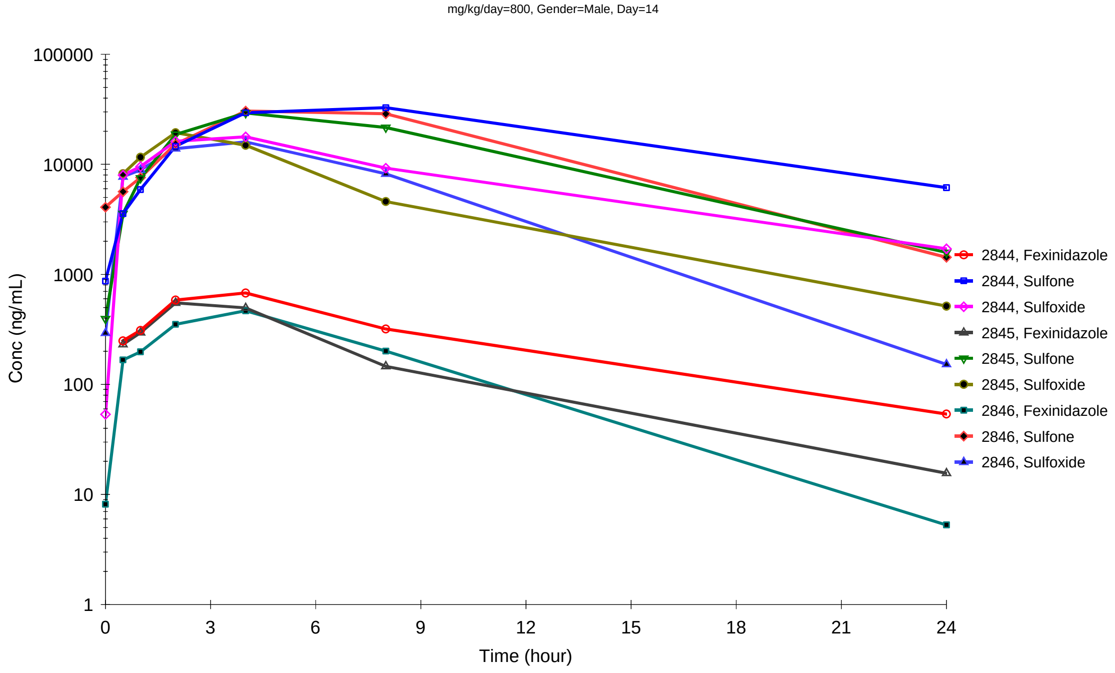

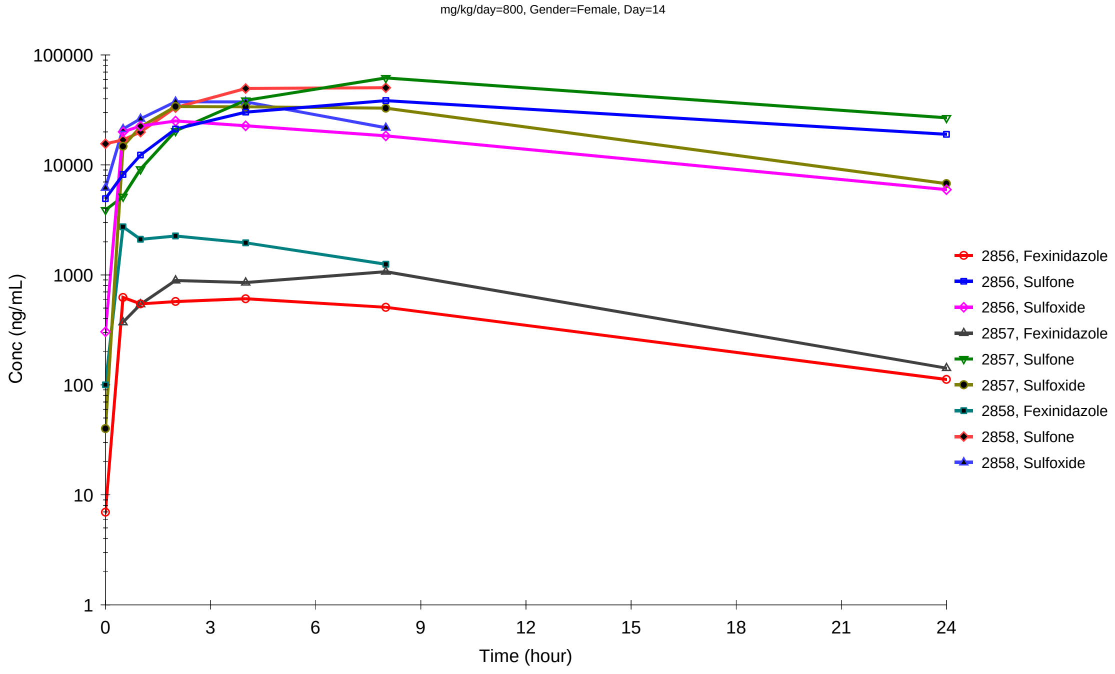

Figure 9. Day 28 individual plasma concentrations (ng/mL) of Fexinidazole and metabolites after oral 800 mg/kg/day dose of Fexinidazole in male (upper panel) and female (lower panel) Sprague Dawley rats.

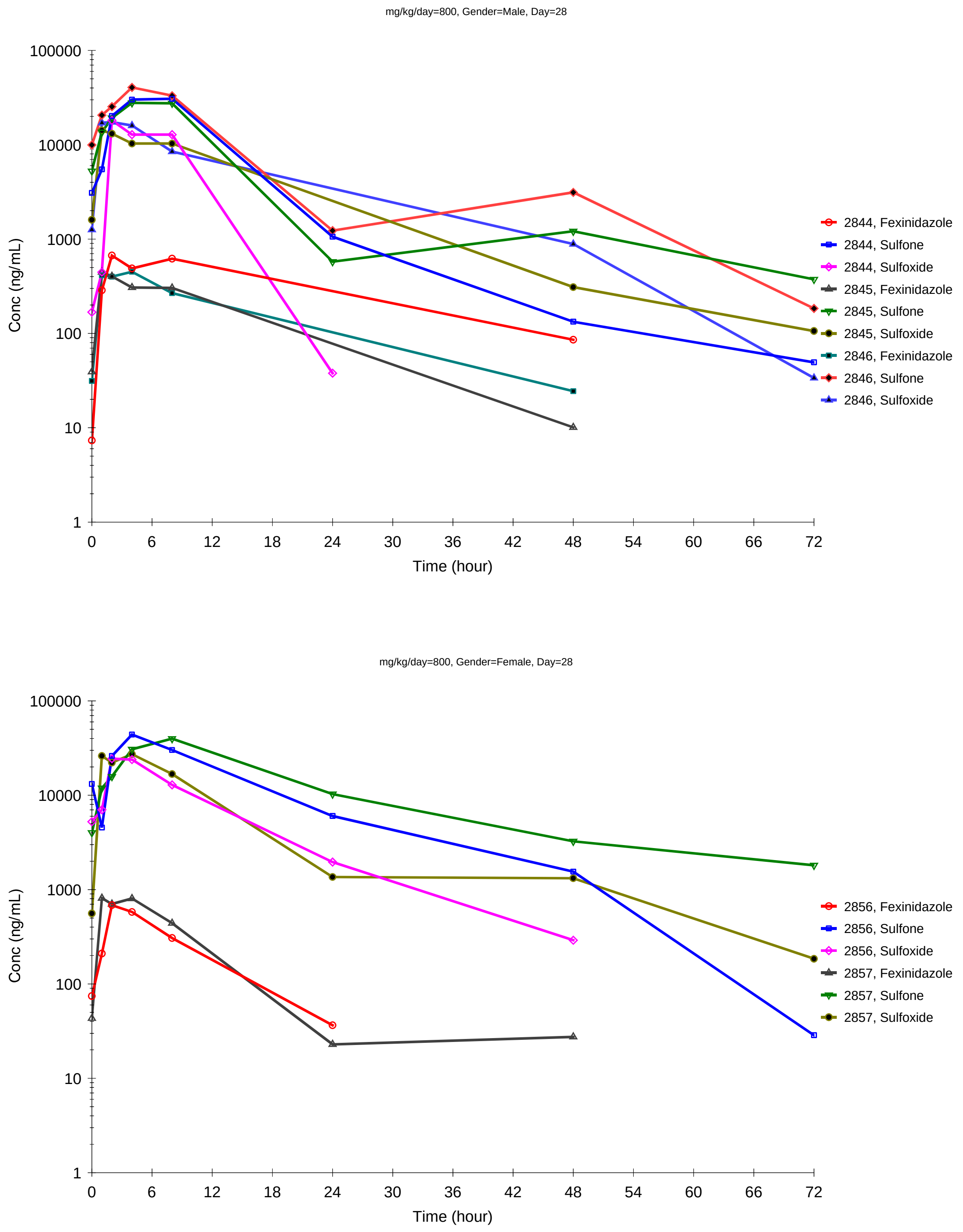

Figure 10. Day 1 mean (+SD) plasma concentrations (ng/mL) of Fexinidazole and metabolites after single and repeated oral 50, 200 and 800 mg/kg/day of Fexinidazole in male Sprague Dawley rats.

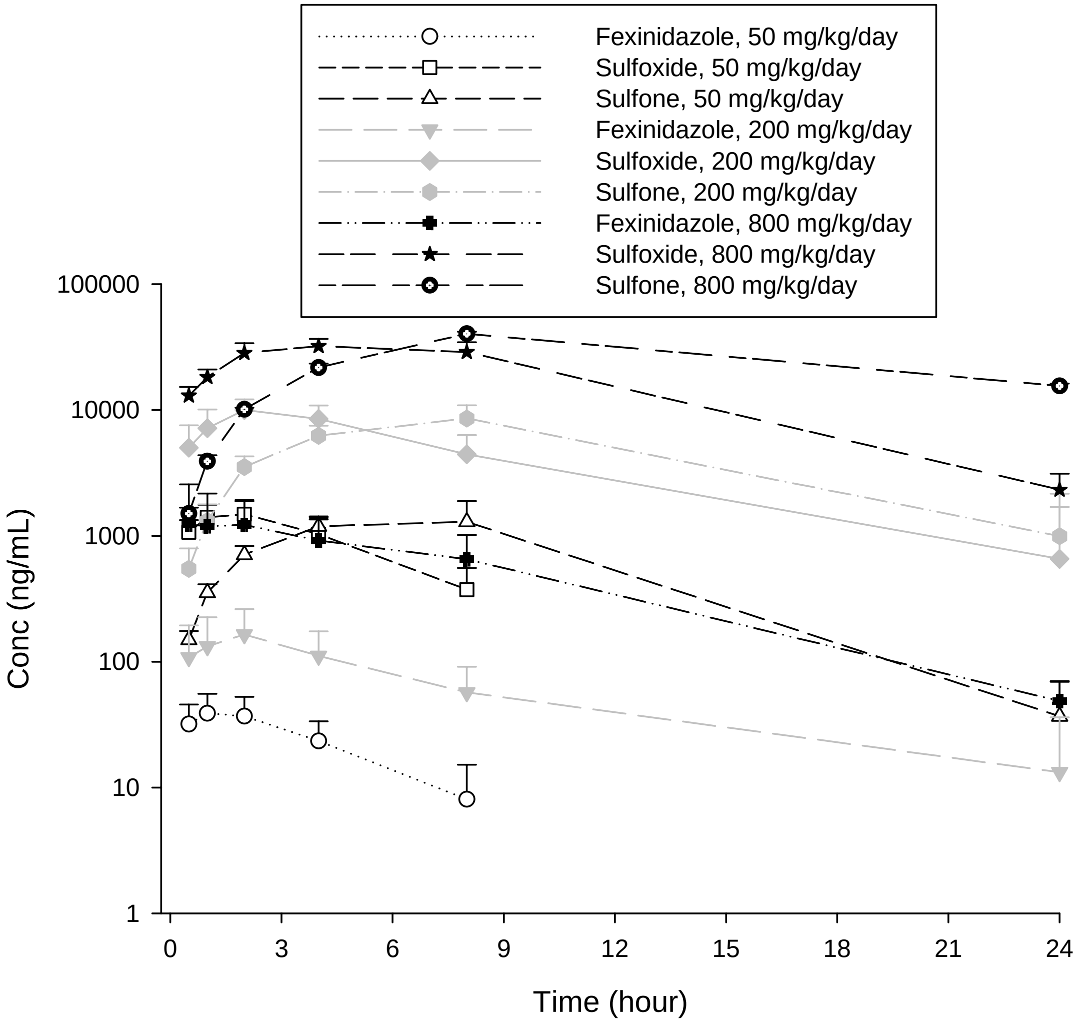

Figure 11. Day 1 mean (+SD) plasma concentrations (ng/mL) of Fexinidazole and metabolites after single and repeated oral 50, 200 and 800 mg/kg/day of Fexinidazole in female Sprague Dawley rats.

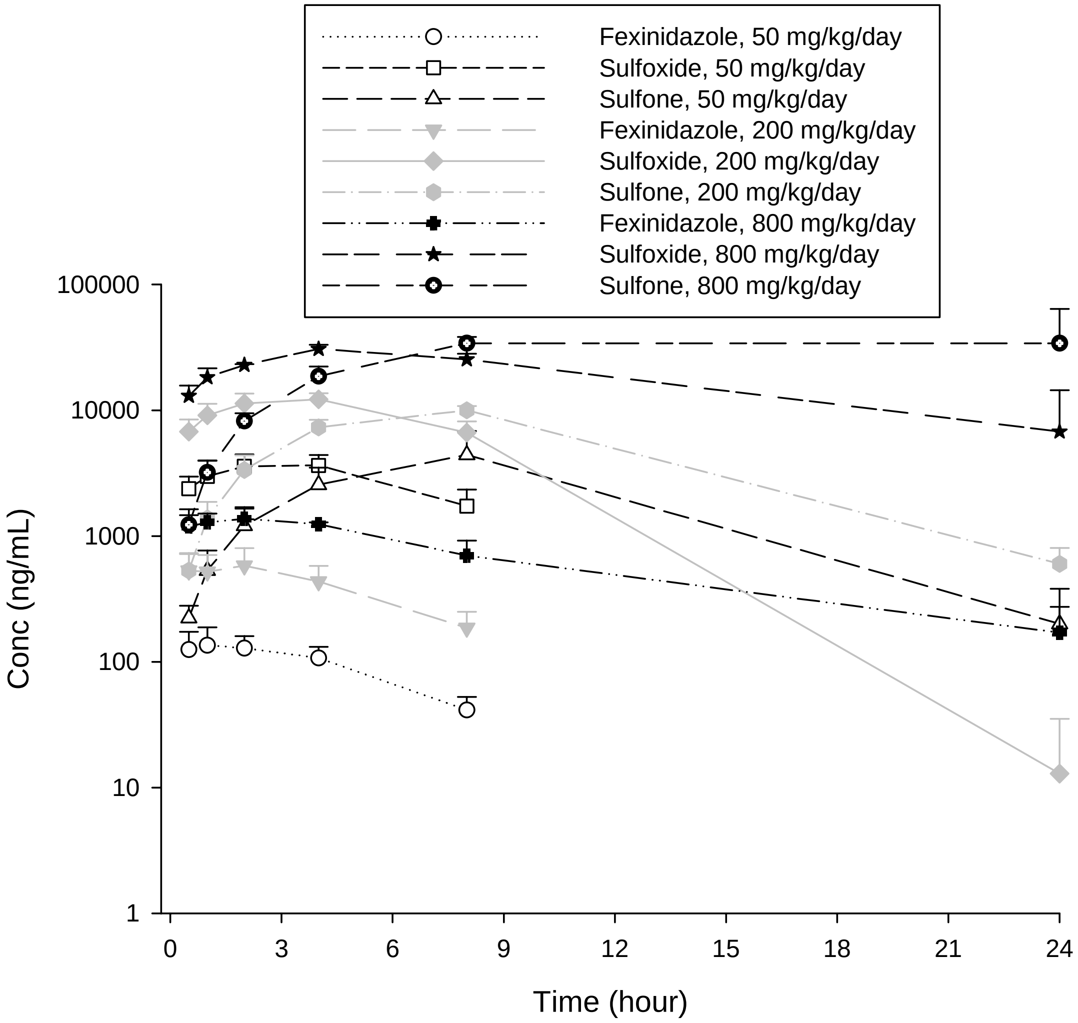

Figure 12. Day 14 mean (+SD) plasma concentrations (ng/mL) of Fexinidazole and metabolites after single and repeated oral 50, 200 and 800 mg/kg/day of Fexinidazole in male Sprague Dawley rats.

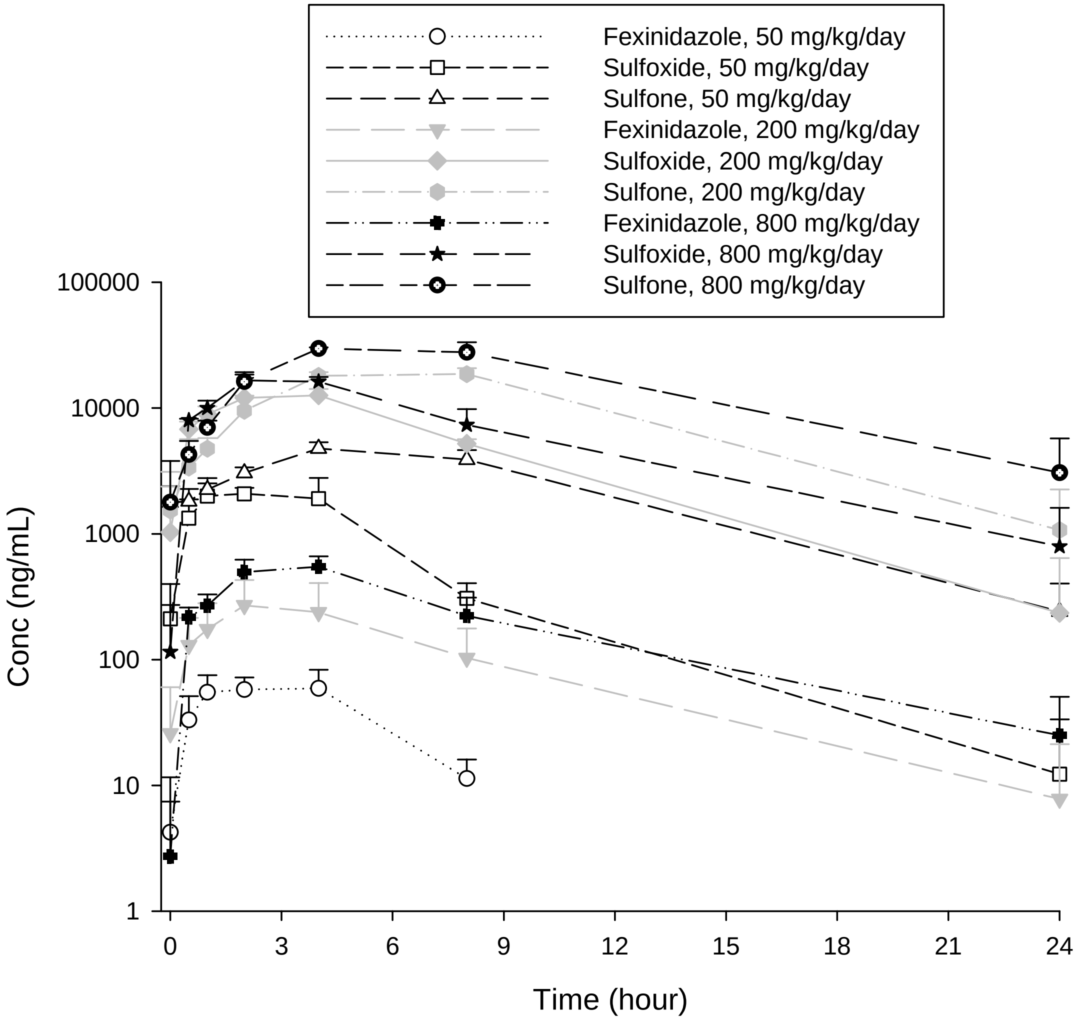

Figure 13. Day 14 mean (+SD) plasma concentrations (ng/mL) of Fexinidazole and metabolites after single and repeated oral 50, 200 and 800 mg/kg/day of Fexinidazole in female Sprague Dawley rats.

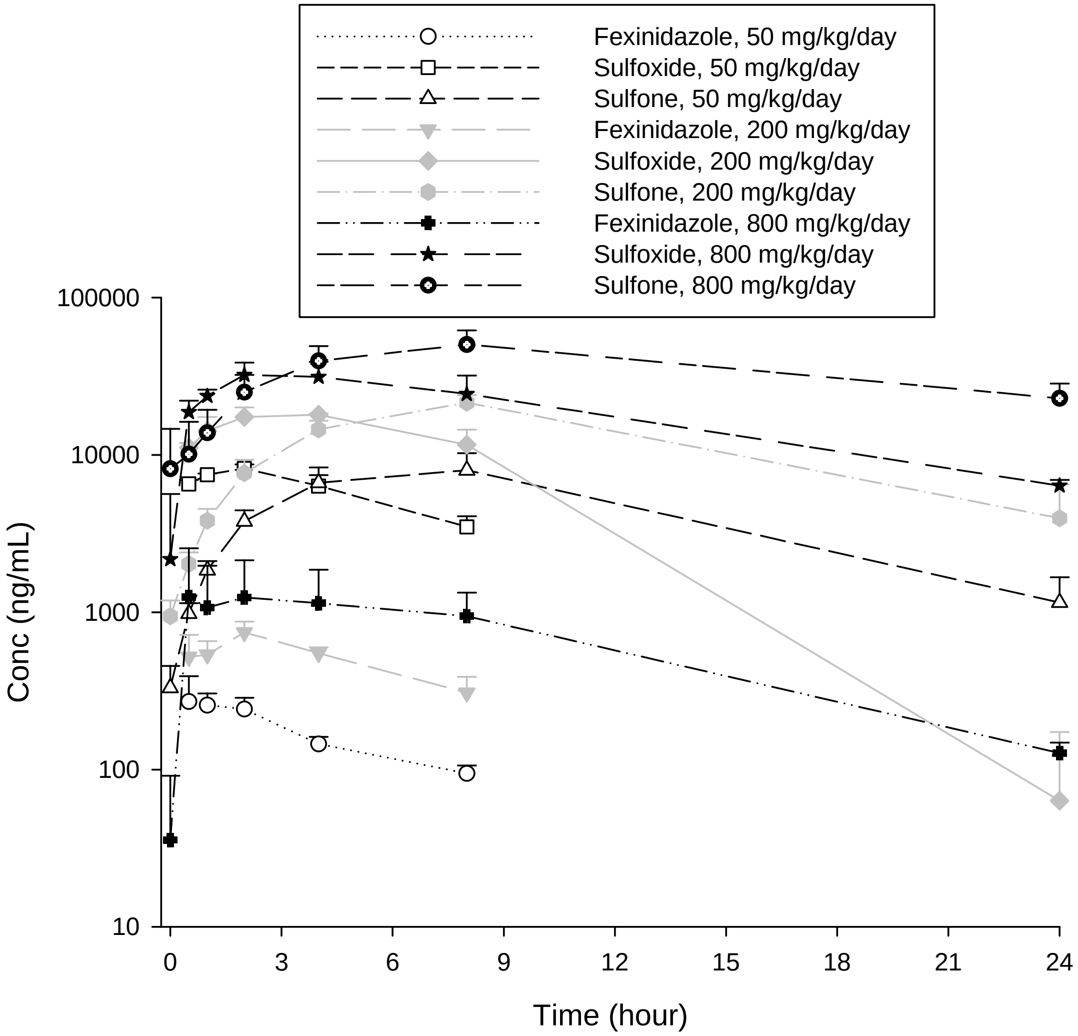

Figure 14. Day 28 mean (+SD) plasma concentrations (ng/mL) of Fexinidazole and metabolites after single and repeated oral 50, 200 and 800 mg/kg/day of Fexinidazole in male Sprague Dawley rats.

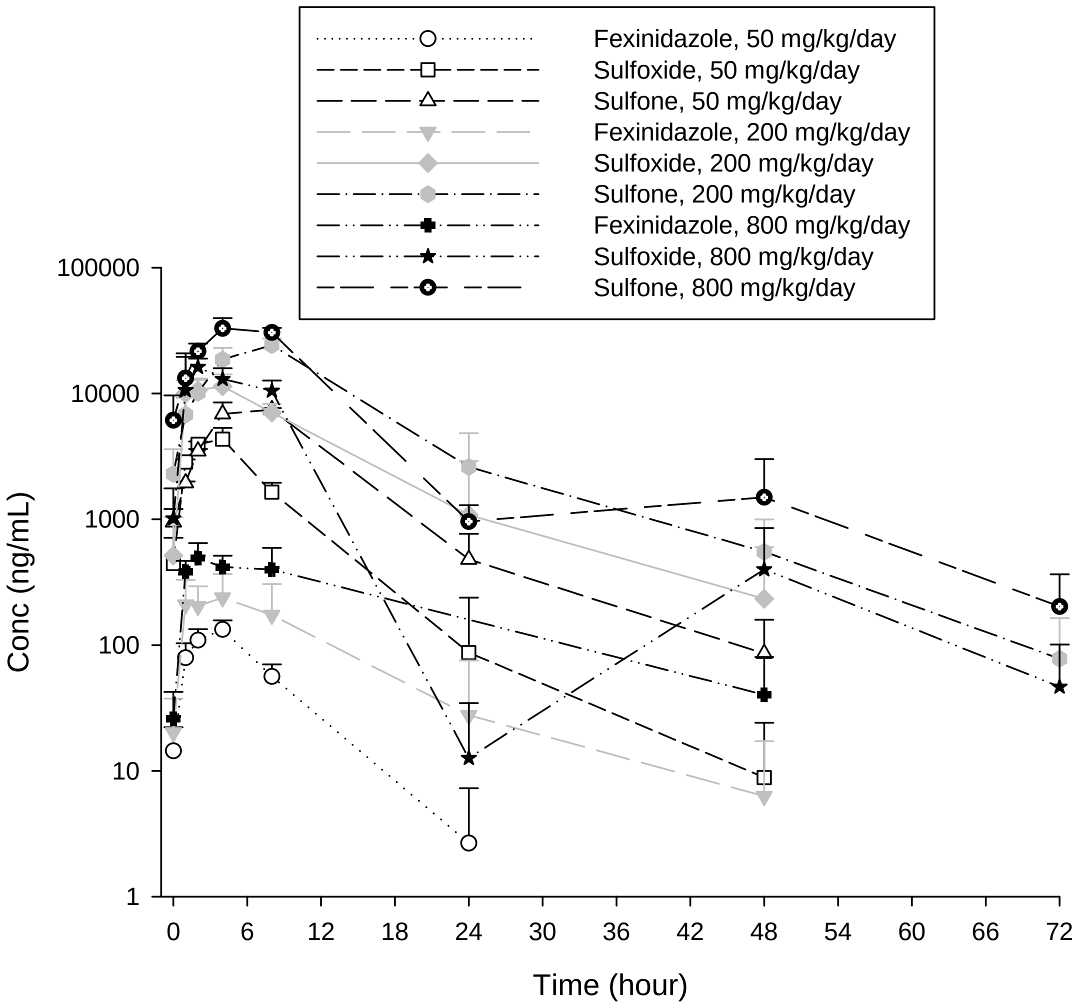

Figure 15. Day 28 mean (+SD) plasma concentrations (ng/mL) of Fexinidazole and metabolites after single and repeated oral 50, 200 and 800 mg/kg/day of Fexinidazole in female Sprague Dawley rats.

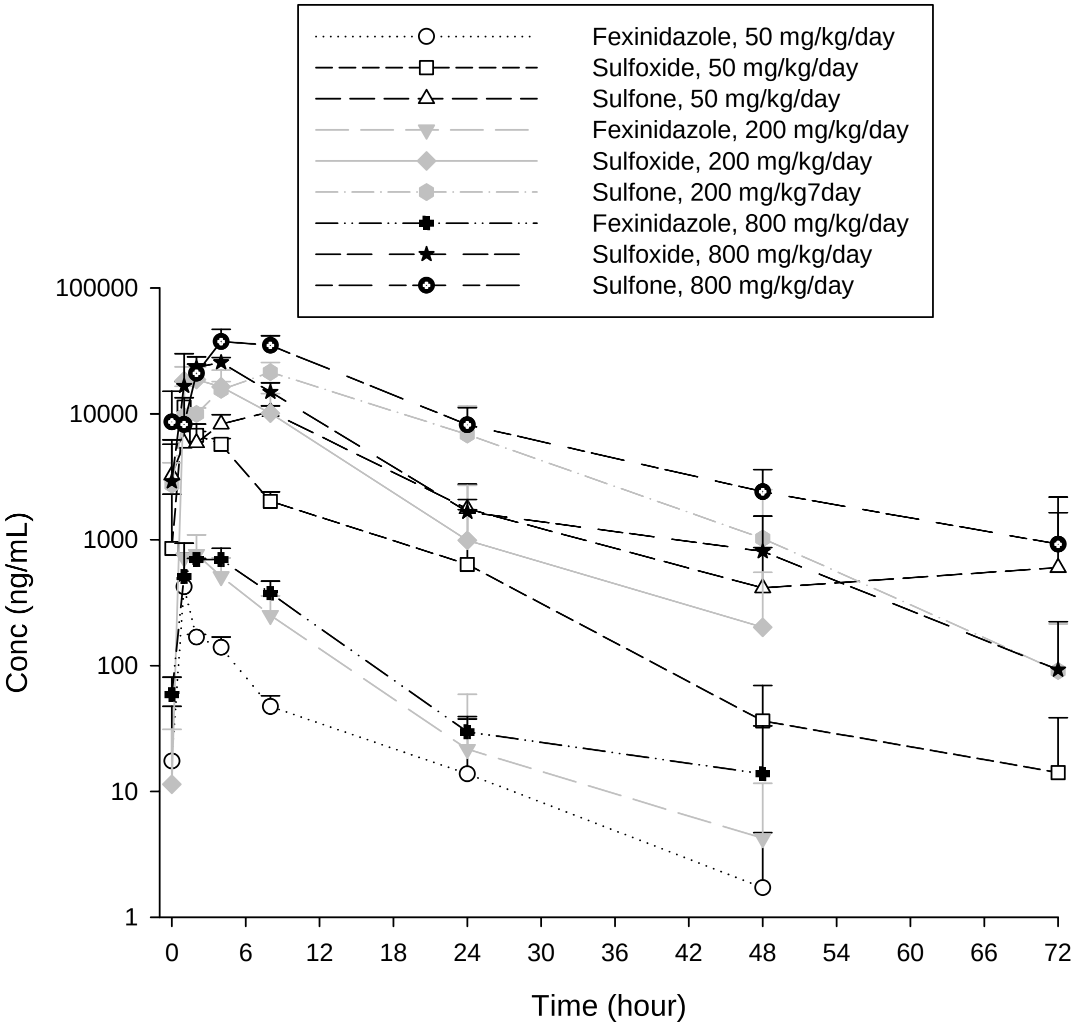

Figure 16. Mean (±SD) normalized C_{max} (upper panel) and $AUC_{0\text{-}t(last)}$ (on Day 28 AUC within 24 hours, lower panel) of Fexinidazole after single and repeated oral 50, 200 and 800 mg/kg/day of Fexinidazole in male Sprague Dawley rats.

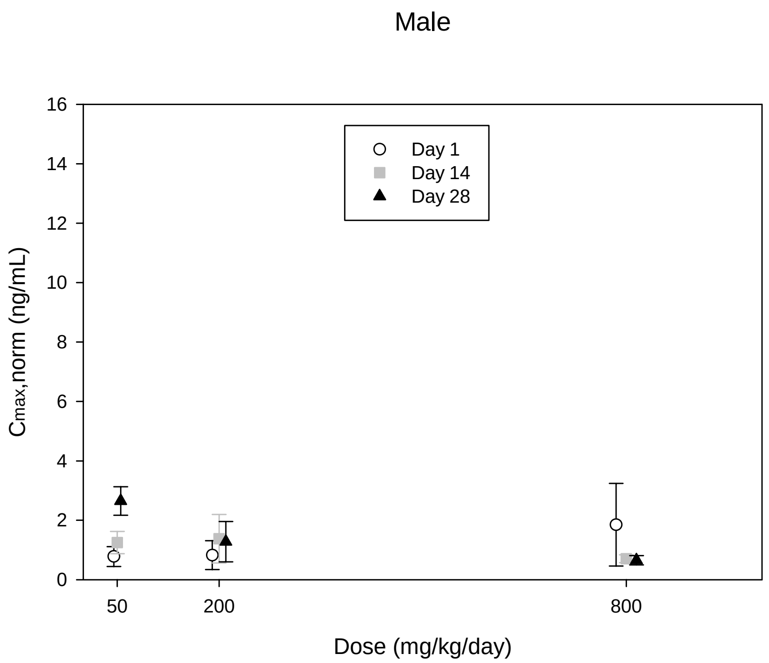

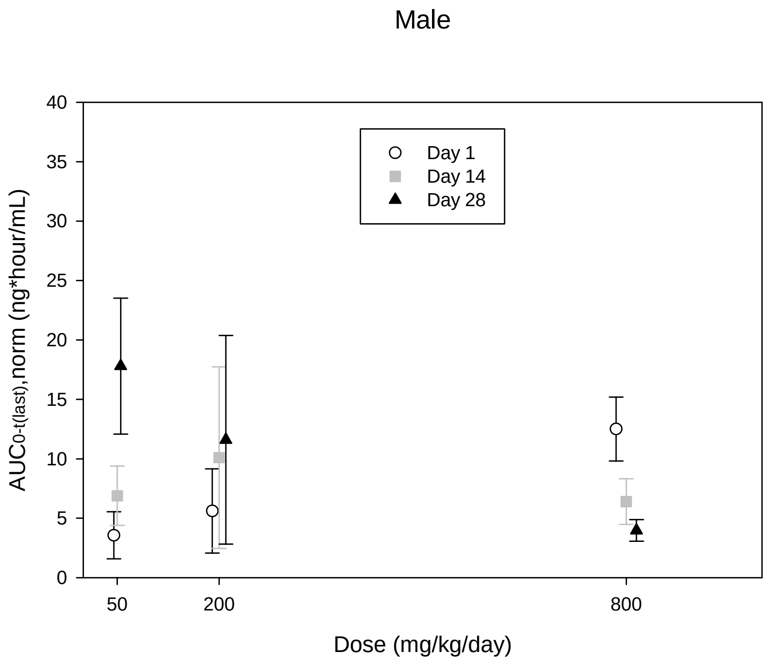

Figure 17. Mean (±SD) normalized C_{max} (upper panel) and $AUC_{0-t(last)}$ (on Day 28 AUC within 24 hours, lower panel) of Fexinidazole after single and repeated oral 50, 200 and 800 mg/kg/day of Fexinidazole in female Sprague Dawley rats.

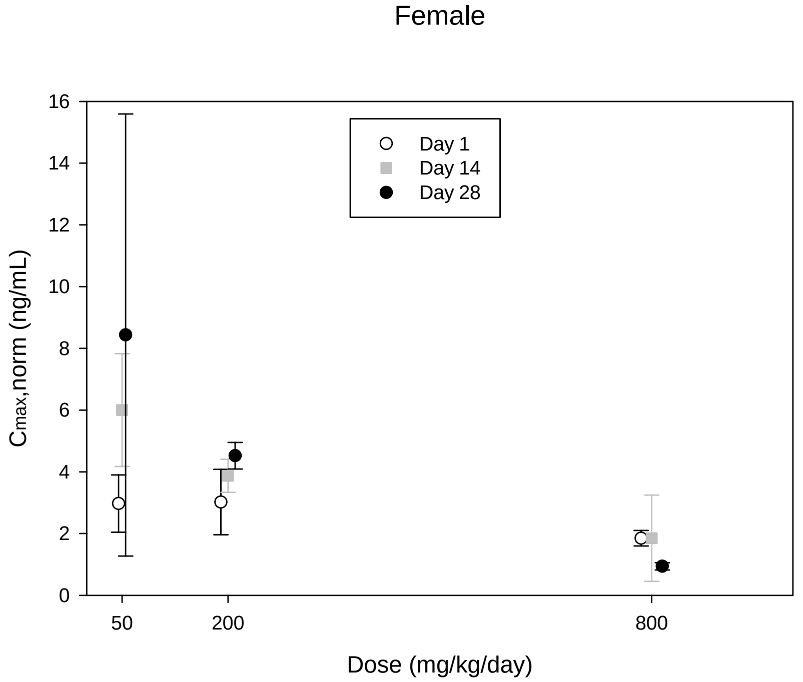

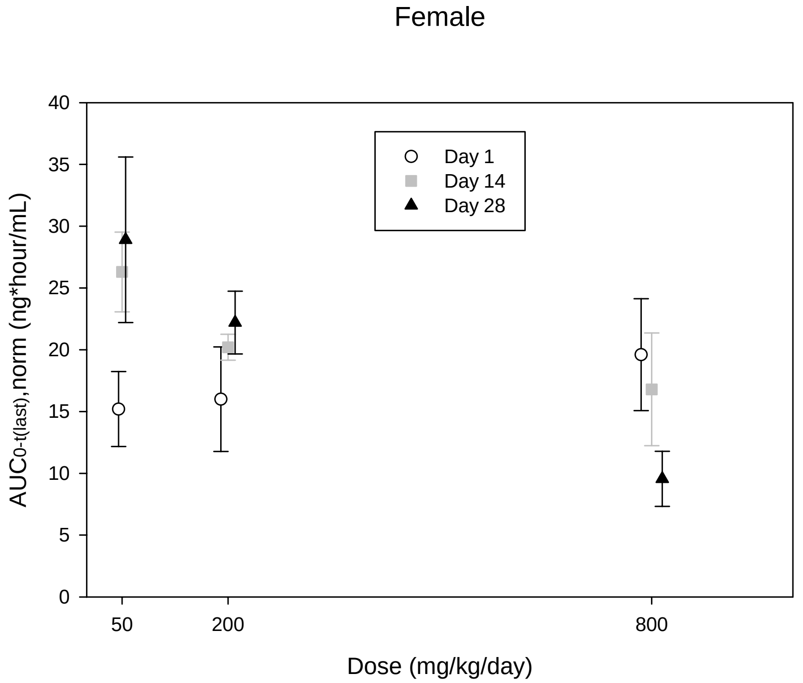

Figure 18. Mean (±SD) normalized C_{max} (upper panel) and $AUC_{0-t(last)}$ (on Day 28 AUC within 24 hours, lower panel) of the sulfoxide derivative after single and repeated oral 50, 200 and 800 mg/kg/day of Fexinidazole in male Sprague Dawley rats.

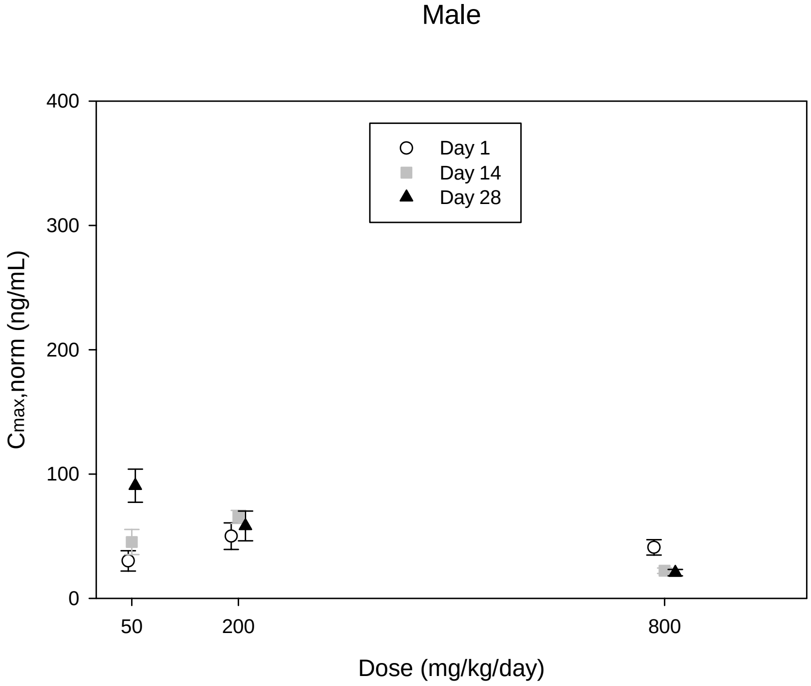

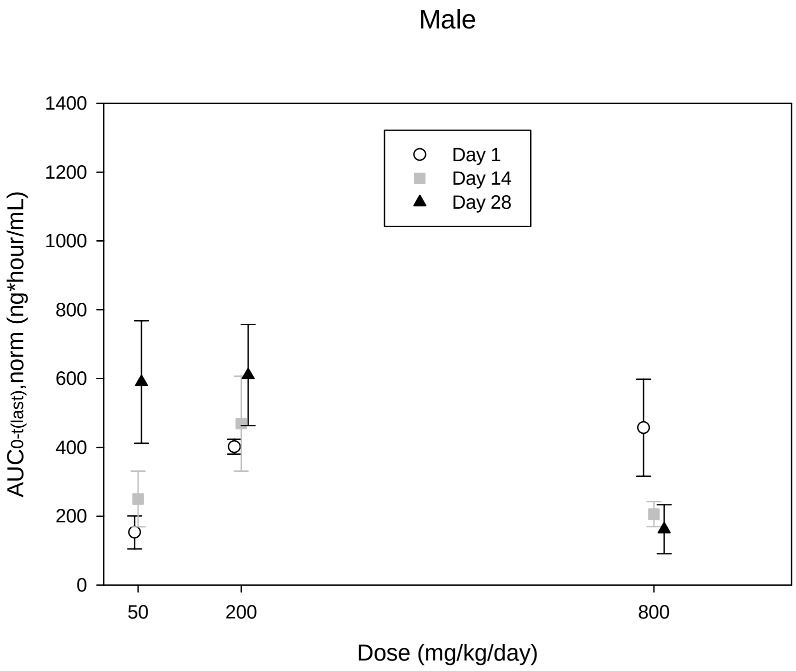

Figure 19. Mean (±SD) normalized C_{max} (upper panel) and $AUC_{0-t(last)}$ (on Day 28 AUC within 24 hours, lower panel) of the sulfoxide derivative after single and repeated oral 50, 200 and 800 mg/kg/day of Fexinidazole in female Sprague Dawley rats.

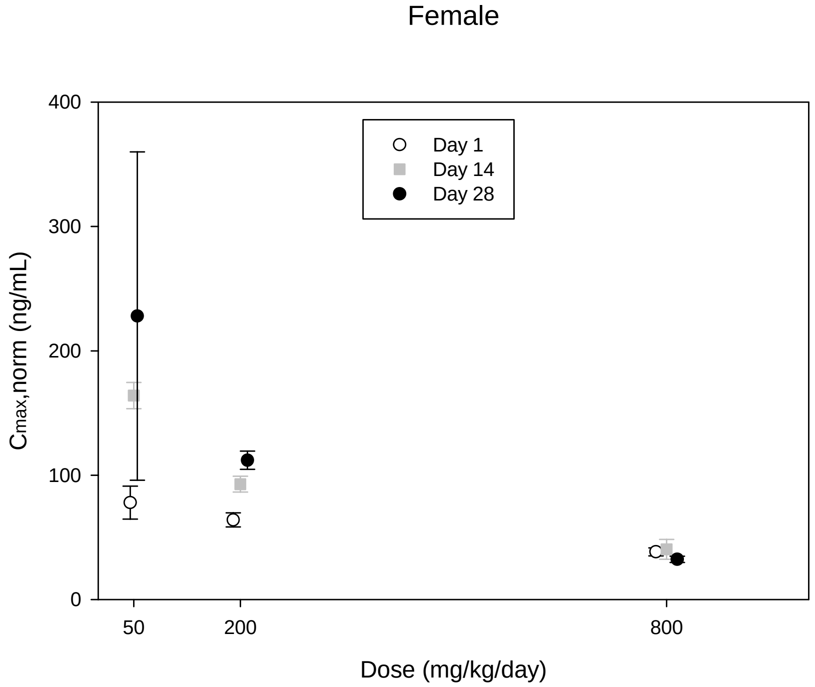

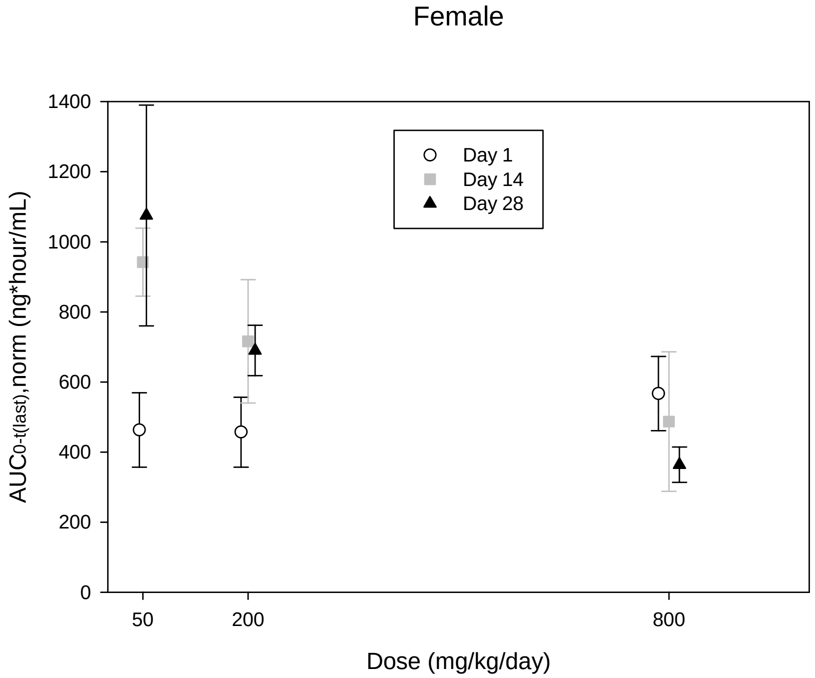

Figure 20. Mean (±SD) normalized C_{max} (upper panel) and $AUC_{0\text{-}t(last)}$ (on Day 28 AUC within 24 hours, lower panel) of the sulfone derivative after single and repeated oral 50, 200 and 800 mg/kg/day of Fexinidazole in male Sprague Dawley rats.

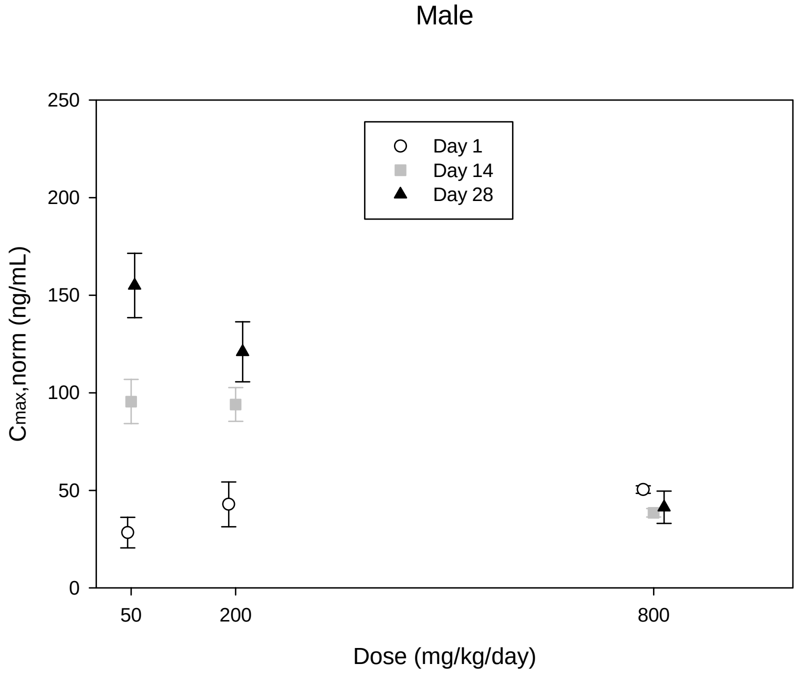

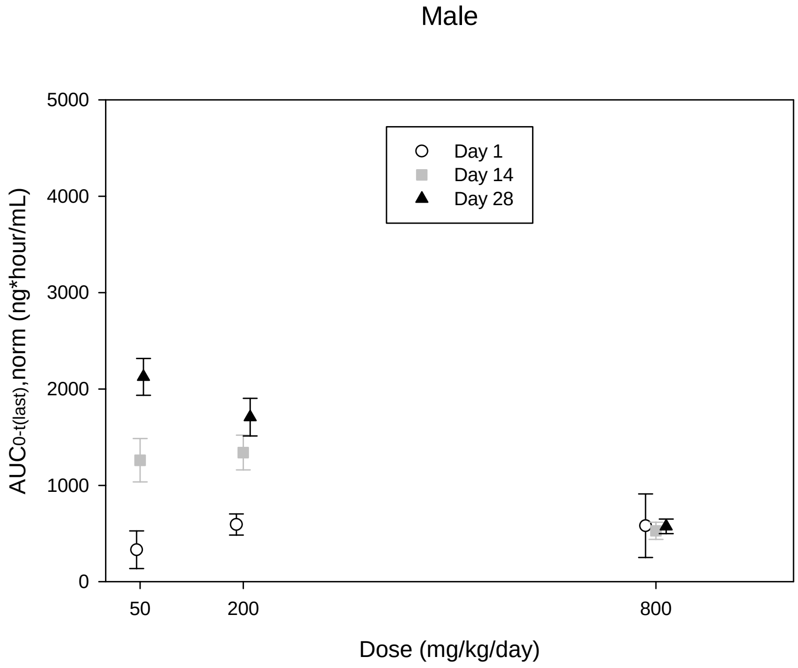

Figure 21. Mean (±SD) normalized C_{max} (upper panel) and $AUC_{0-t(last)}$ (on Day 28 AUC within 24 hours, lower panel) of the sulfone derivative after single and repeated oral 50, 200 and 800 mg/kg/day of Fexinidazole in female Sprague Dawley rats.

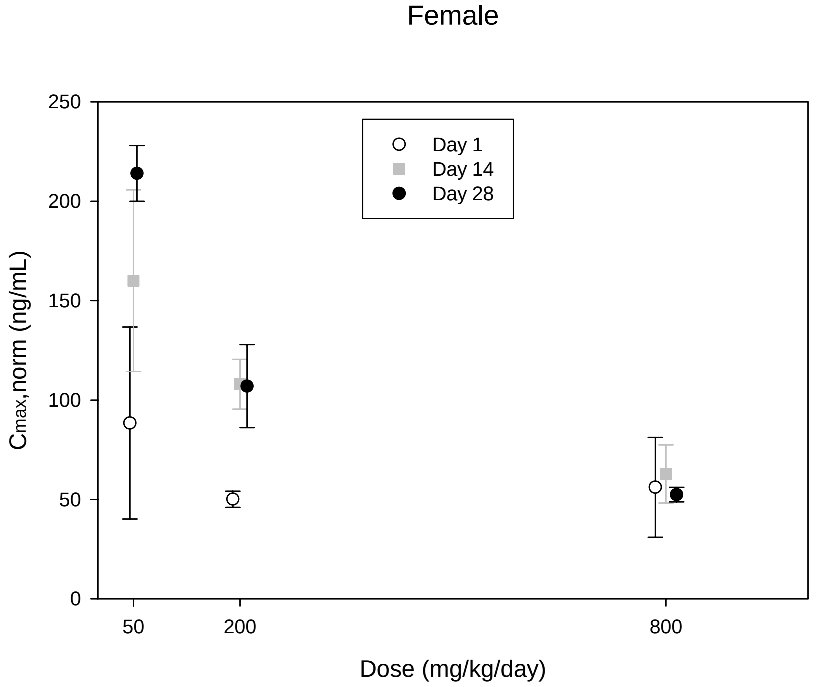

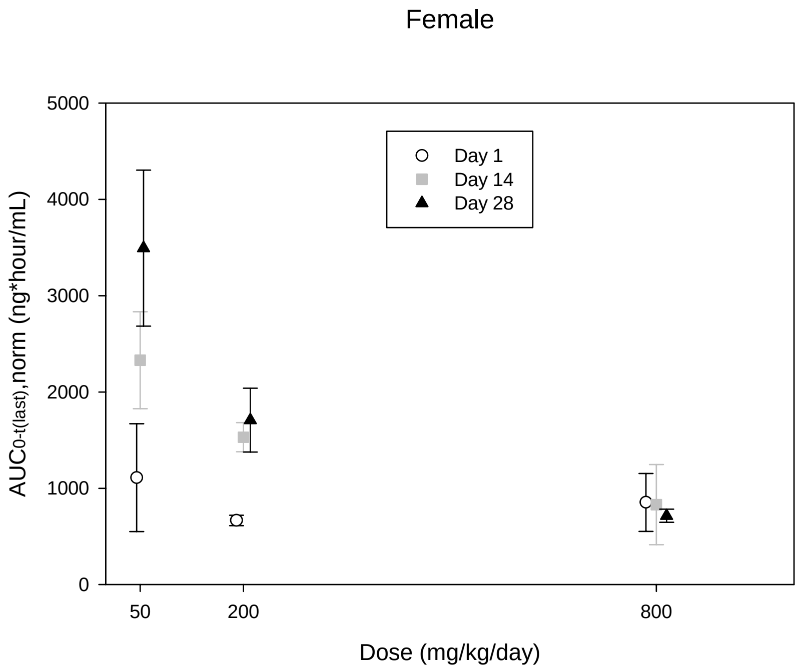

APPENDICES

Appendix 1. Individual plasma concentrations

Table 1A1. Individual plasma concentrations (ng/mL) of Fexinidazole and sulfoxide and sulfone metabolites after single and repeated oral administrations of the vehicle in male and female Sprague Dawley rats.

Day 1						
Fexinidazole						
Time (hour)	M			F		
	ID 2835	ID 2836	ID 2837	ID 2847	ID 2848	ID 2849
Pre-dose	<5	<5	<5	<5	<5	<5
2	<5	<5	<5	<5	<5	<5
Sulfoxide						
Pre-dose	<25	<25	<25	<25	<25	<25
2	<25	<25	<25	<25	<25	<25
Sulfone						
Pre-dose	<25	<25	<25	<25	<25	<25
2	<25	<25	<25	<25	<25	<25
Day 14						
Fexinidazole						
Time (hour)	M			F		
	ID 2835	ID 2836	ID 2837	ID 2847	ID 2848	ID 2849
Pre-dose	<5	<5	<5	<5	<5	<5
2	<5	<5	<5	<5	<5	<5
Sulfoxide						
Pre-dose	<25	<25	<25	<25	<25	<25
2	<25	<25	<25	<25	29.4	<25
Sulfone						
Pre-dose	<25	<25	<25	<25	<25	<25
2	<25	<25	<25	<25	32.7	<25
Day 28						
Fexinidazole						
Time (hour)	M			F		
	ID 2835	ID 2836	ID 2837	ID 2847	ID 2848	ID 2849
Pre-dose	<5	<5	<5	9.61	<5	<5
2	<5	<5	<5	<5	<5	<5
Sulfoxide						
Pre-dose	<25	<25	<25	<25	<25	<25
2	<25	<25	<25	<25	<25	<25
Sulfone						
Pre-dose	<25	<25	<25	<25	<25	<25
2	<25	<25	<25	<25	<25	<25

Table 2A1. Day 1 individual and mean (±SD, %CV) plasma concentrations (ng/mL) of Fexinidazole and sulfoxide and sulfone metabolites after oral 50 mg/kg/day of Fexinidazole in male Sprague Dawley rats.

Fexinidazole						
Time (hour)	ID 2838	ID 2839	ID 2840	Mean	SD	%CV
0	<5	<5	<5	N/A	N/A	N/A
0.5	25.7	22.3	47.7	31.9	13.8	43
1	35.5	24.3	56.9	38.9	16.6	43
2	35.5	22	53.1	36.9	15.6	42
4	23.2	13.4	33.7	23.4	10.2	44
8	10.8	<5	13.4	8.07	7.11	88
24	<5	<5	<5	N/A	N/A	N/A
Sulfoxide						
Time (hour)	ID 2838	ID 2839	ID 2840	Mean	SD	%CV
0	<25	<25	<25	N/A	N/A	N/A
0.5	884	946	1370	1070	265	25
1	1270	1120	1810	1400	363	26
2	1450	1060	1940	1480	441	30
4	1130	640	1330	1030	355	35
8	489	162	468	373	183	49
24	<25	<25	<25	N/A	N/A	N/A
Sulfone						
Time (hour)	ID 2838	ID 2839	ID 2840	Mean	SD	%CV
0	<25	<25	<25	N/A	N/A	N/A
0.5	119	161	167	149	26.2	18
1	308	331	421	353	59.7	17
2	611	664	847	707	124	18
4	1130	1000	1450	1190	232	20
8	1470	627	1780	1290	597	46
24	47.6	<25	62.4	36.7	32.6	89

Estimates of mean based on approximation that values below LLOQ are equal to zero.
N/A: not applicable.

Table 3A1. Day 1 individual and mean (±SD, %CV) plasma concentrations (ng/mL) of Fexinidazole and sulfoxide and sulfone metabolites after oral 50 mg/kg/day of Fexinidazole in female Sprague Dawley rats.

Fexinidazole						
Time (hour)	ID 2850	ID 2851	ID 2852	Mean	SD	%CV
Pre-dose	<5	<5	<5	N/A	N/A	N/A
0.5	134	168	73.2	125	48	38
1	182	146	77.6	135	53	39
2	149	144	91.5	128	31.9	25
4	135	90	95.6	107	24.5	23
8	45.1	28.7	50	41.3	11.2	27
24	<5	<5	<5	N/A	N/A	N/A
Sulfoxide						
Time (hour)	ID 2850	ID 2851	ID 2852	Mean	SD	%CV
Pre-dose	<25	<25	<25	N/A	N/A	N/A
0.5	2760	2670	1700	2380	588	25
1	4100	2730	2120	2980	1010	34
2	4200	3960	2580	3580	874	24
4	4520	3200	3200	3640	762	21
8	2420	1250	1530	1730	611	35
24	<25	<25	<25	N/A	N/A	N/A
Sulfone						
Time (hour)	ID 2850	ID 2851	ID 2852	Mean	SD	%CV
Pre-dose	<25	<25	<25	N/A	N/A	N/A
0.5	277	225	166	223	55.5	25
1	803	398	392	531	236	44
2	1750	1080	800	1210	488	40
4	3640	2080	1960	2560	937	37
8	7190	2830	3230	4420	2410	55
24	243	115	242	200	73.6	37
N/A: not applicable.						

Table 4A1. Day 14 individual and mean (±SD, %CV) plasma concentrations (ng/mL) of Fexinidazole and sulfoxide and sulfone metabolites after oral 50 mg/kg/day of Fexinidazole in male Sprague Dawley rats.

Fexinidazole						
Time (hour)	ID 2838	ID 2839	ID 2840	Mean	SD	%CV
Pre-dose	<5	<5	12.7	4.23*	7.33	173
0.5	34	14.6	50.7	33.1	18.1	55
1	67.1	31.6	65.8	54.8	20.1	37
2	70.9	42.2	59.8	57.6	14.5	25
4	79	32.3	65.6	59	24	41
8	15.4	6.17	12.4	11.3	4.71	42
24	<5	<5	<5	N/A	N/A	N/A
Sulfoxide						
Time (hour)	ID 2838	ID 2839	ID 2840	Mean	SD	%CV
Pre-dose	165	50.2	417	211	188	89
0.5	1390	759	1850	1330	548	41
1	2410	1400	2150	1990	524	26
2	2310	1830	2110	2080	241	12
4	2820	1080	1800	1900	874	46
8	413	221	285	306	97.8	32
24	<25	<25	36.8	12.3*	21.2	172
Sulfone						
Time (hour)	ID 2838	ID 2839	ID 2840	Mean	SD	%CV
Pre-dose	2440	1230	1630	1770	616	35
0.5	2100	1270	2050	1810	465	26
1	2750	1690	2290	2240	532	24
2	3290	2670	3160	3040	327	11
4	5110	4120	5090	4770	566	12
8	4690	3230	3760	3890	739	19
24	374	60.3	288	241	162	67

Estimates of mean based on approximation that values below LLOQ are equal to zero. For values marked *, more than half of the individual levels were below LLOQ; descriptive statistics was reported even if strongly biased.

N/A: not applicable.

Table 5A1. Day 14 individual and mean (±SD, %CV) plasma concentrations (ng/mL) of Fexinidazole and sulfoxide and sulfone metabolites after oral 50 mg/kg/day of Fexinidazole in female Sprague Dawley rats.

Fexinidazole						
Time (hour)	ID 2850	ID 2851	ID 2852	Mean	SD	%CV
Pre-dose	<5	<5	<5	N/A	N/A	N/A
0.5	263	395	152	270	122	45
1	288	278	201	256	47.6	19
2	292	220	213	242	43.7	18
4	162	130	143	145	16.1	11
8	106	82.2	94.4	94.2	11.9	13
24	<5	<5	<5	N/A	N/A	N/A
Sulfoxide						
Time (hour)	ID 2850	ID 2851	ID 2852	Mean	SD	%CV
Pre-dose	<25	<25	<25	N/A	N/A	N/A
0.5	6610	6870	6100	6530	392	6
1	7640	7450	7360	7480	143	2
2	8750	7720	8120	8200	519	6
4	7590	5630	5760	6330	1100	17
8	3850	2820	3790	3490	578	17
24	<25	<25	<25	N/A	N/A	N/A
Sulfone						
Time (hour)	ID 2850	ID 2851	ID 2852	Mean	SD	%CV
Pre-dose	266	252	475	331	125	38
0.5	987	817	1140	981	162	17
1	2050	1550	1940	1850	263	14
2	4350	3060	3930	3780	658	17
4	8310	4940	6680	6640	1690	26
8	10000	5500	8440	7980	2280	29
24	614	1650	1200	1150	519	45
N/A: not applicable.						

Table 6A1. Day 28 individual and mean (±SD, %CV) plasma concentrations (ng/mL) of Fexinidazole and sulfoxide and sulfone metabolites after oral 50 mg/kg/day of Fexinidazole in male Sprague Dawley rats.

Fexinidazole						
Time (hour)	ID 2838	ID 2839	ID 2840	Mean	SD	%CV
Pre-dose	22.8	7.43	13	14.4	7.78	54
1	87.9	51.9	97.6	79.1	24.1	31
2	124	80.8	123	109	24.7	23
4	148	105	145	133	24	18
8	64.9	63.4	40	56.1	14	25
24	<5	<5	7.99	2.66*	4.61	173
48	<5	<5	<5	N/A	N/A	N/A
72	<5	<5	<5	N/A	N/A	N/A
Sulfoxide						
Time (hour)	ID 2838	ID 2839	ID 2840	Mean	SD	%CV
Pre-dose	742	226	365	444	267	60
1	2790	2750	3010	2850	140	5
2	4170	3770	3890	3940	205	5
4	4820	3180	5000	4330	1000	23
8	1680	1930	1320	1640	307	19
24	<25	<25	261	87*	151	174
48	<25	<25	26.5	8.83*	15.3	173
72	<25	<25	<25	N/A	N/A	N/A
Sulfone						
Time (hour)	ID 2838	ID 2839	ID 2840	Mean	SD	%CV
Pre-dose	1240	750	851	947	259	27
1	1930	1900	2000	1940	51.3	3
2	3330	3530	3570	3480	129	4
4	6170	5700	8710	6860	1620	24
8	7260	7280	7700	7410	248	3
24	533	171	736	480	286	60
48	63.7	25.6	167	85.4	73.2	86
72	<25	<25	<25	N/A	N/A	N/A

Estimates of mean based on approximation that values below LLOQ are equal to zero. For values marked *, more than half of the individual levels were below LLOQ; descriptive statistics was reported even if strongly biased.

N/A: not applicable.

Table 7A1. Day 28 individual and mean (±SD, %CV) plasma concentrations (ng/mL) of Fexinidazole and sulfoxide and sulfone metabolites after oral 50 mg/kg/day of Fexinidazole in female Sprague Dawley rats.

Fexinidazole						
Time (hour)	ID 2850	ID 2851	ID 2852	Mean	SD	%CV
Pre-dose	<5	<5	52.2	17.4*	30.1	173
1	216	214	835	422	358	85
2	163	164	178	168	8.39	5
4	165	107	146	139	29.6	21
8	53	53.3	35.3	47.2	10.3	22
24	<5	41.3	<5	13.8*	23.8	173
48	<5	5.16	<5	1.72*	2.98	173
72	<5	<5	<5	N/A	N/A	N/A
Sulfoxide						
Time (hour)	ID 2850	ID 2851	ID 2852	Mean	SD	%CV
Pre-dose	47.3	<25	2510	852	1440	169
1	6050	8920	18900	11300	6740	60
2	6420	6490	7300	6740	489	7
4	6410	5120	5630	5720	650	11
8	2330	2140	1590	2020	384	19
24	<25	1910	<25	637*	1100	173
48	44.6	64.2	<25	36.3	32.9	91
72	<25	42.3	<25	14.1*	24.4	173
Sulfone						
Time (hour)	ID 2850	ID 2851	ID 2852	Mean	SD	%CV
Pre-dose	605	3640	5500	3250	2470	76
1	2170	7820	7530	5840	3180	55
2	3860	5270	8520	5880	2390	41
4	7890	6990	10000	8290	1550	19
8	10700	11400	9100	10400	1180	11
24	871	2850	1570	1760	1000	57
48	114	934	191	413	453	110
72	<25	1800	<25	600*	1040	173

Estimates of mean based on approximation that values below LLOQ are equal to zero. For values marked *, more than half of the individual levels were below LLOQ; descriptive statistics was reported even if strongly biased.

N/A: not applicable.

Table 8A1. Day 1 individual and mean (±SD, %CV) plasma concentrations (ng/mL) of Fexinidazole and sulfoxide and sulfone metabolites after oral 200 mg/kg/day of Fexinidazole in male Sprague Dawley rats.

Fexinidazole						
Time (hour)	ID 2841	ID 2842	ID 2843	Mean	SD	%CV
Pre-dose	<5	<5	<5	N/A	N/A	N/A
0.5	54.1	207	62.5	108	86	80
1	96.2	238	63	132	92.9	70
2	176	256	61.4	164	97.8	60
4	128	163	41	111	62.8	57
8	63.9	87	19.4	56.8	34.4	61
24	39.7	<5	<5	13.2*	22.9	174
Sulfoxide						
Time (hour)	ID 2841	ID 2842	ID 2843	Mean	SD	%CV
Pre-dose	<25	<25	<25	N/A	N/A	N/A
0.5	2410	5150	7460	5010	2530	51
1	4230	7120	10100	7150	2940	41
2	7950	9810	12200	9990	2130	21
4	7070	7240	11200	8500	2340	28
8	3270	3480	6610	4450	1870	42
24	1850	116	<25	655	1040	159
Sulfone						
Time (hour)	ID 2841	ID 2842	ID 2843	Mean	SD	%CV
Pre-dose	<25	<25	<25	N/A	N/A	N/A
0.5	303	543	796	547	247	45
1	830	1330	1760	1310	465	36
2	2770	3520	4280	3520	755	21
4	5370	5650	7690	6240	1270	20
8	6600	7950	11100	8550	2310	27
24	2340	404	228	991	1170	118

Estimates of mean based on approximation that values below LLOQ are equal to zero. For values marked *, more than half of the individual levels were below LLOQ; descriptive statistics was reported even if strongly biased.

N/A: not applicable.

Table 9A1. Day 1 individual and mean (±SD, %CV) plasma concentrations (ng/mL) of Fexinidazole and sulfoxide and sulfone metabolites after oral 200 mg/kg/day of Fexinidazole in female Sprague Dawley rats.

Fexinidazole						
Time (hour)	ID 2853	ID 2854	ID 2855	Mean	SD	%CV
Pre-dose	<5	<5	<5	N/A	N/A	N/A
0.5	761	415	412	529	201	38
1	732	446	376	518	189	37
2	830	502	408	580	222	38
4	454	569	283	435	144	33
8	156	259	142	186	63.9	34
24	<5	<5	<5	N/A	N/A	N/A
Sulfoxide						
Time (hour)	ID 2853	ID 2854	ID 2855	Mean	SD	%CV
Pre-dose	<25	<25	<25	N/A	N/A	N/A
0.5	8460	5070	6700	6740	1700	25
1	10900	6760	9680	9110	2130	23
2	13400	8910	11500	11300	2250	20
4	12400	13500	10600	12200	1460	12
8	6150	8310	5450	6640	1490	22
24	<25	<25	38.7	12.9*	22.3	173
Sulfone						
Time (hour)	ID 2853	ID 2854	ID 2855	Mean	SD	%CV
Pre-dose	<25	<25	<25	N/A	N/A	N/A
0.5	585	323	684	531	187	35
1	1440	861	1830	1380	488	35
2	3680	2200	4160	3350	1020	30
4	7090	6370	8470	7310	1070	15
8	9290	9880	10900	10000	815	8
24	564	820	414	599	205	34

Estimates of mean based on approximation that values below LLOQ are equal to zero. For values marked *, more than half of the individual levels were below LLOQ; descriptive statistics was reported even if strongly biased.

N/A: not applicable.

Table 10A1. Day 14 individual and mean (±SD, %CV) plasma concentrations (ng/mL) of Fexinidazole and sulfoxide and sulfone metabolites after oral 200 mg/kg/day of Fexinidazole in male Sprague Dawley rats.

Fexinidazole						
Time (hour)	ID 2841	ID 2842	ID 2843	Mean	SD	%CV
Pre-dose	65	12.5	<5	25.8	34.5	134
0.5	188	169	33.1	130	84.5	65
1	202	262	56.6	174	106	61
2	355	367	87.2	270	158	59
4	277	381	52.4	237	168	71
8	160	129	20.7	103	73.1	71
24	23.3	<5	<5	7.77*	13.5	174
Sulfoxide						
Time (hour)	ID 2841	ID 2842	ID 2843	Mean	SD	%CV
Pre-dose	2550	537	<25	1030	1340	130
0.5	7950	6190	6180	6770	1020	15
1	7490	10100	9220	8940	1330	15
2	12600	11600	11900	12000	513	4
4	11200	14300	12400	12600	1560	12
8	5710	4860	5030	5200	450	9
24	704	<25	<25	235*	406	173
Sulfone						
Time (hour)	ID 2841	ID 2842	ID 2843	Mean	SD	%CV
Pre-dose	3340	849	403	1530	1580	103
0.5	6010	2100	1980	3360	2290	68
1	5900	4320	4030	4750	1010	21
2	12500	7880	8080	9490	2610	28
4	19300	17900	16800	18000	1250	7
8	19700	19900	16100	18600	2140	12
24	2420	543	237	1070	1180	110

Estimates of mean based on approximation that values below LLOQ are equal to zero. For values marked *, more than half of the individual levels were below LLOQ; descriptive statistics was reported even if strongly biased.

Table 11A1. Day 14 individual and mean (±SD, %CV) plasma concentrations (ng/mL) of Fexinidazole and sulfoxide and sulfone metabolites after oral 200 mg/kg/day of Fexinidazole in female Sprague Dawley rats.

Fexinidazole						
Time (hour)	ID 2853	ID 2854	ID 2855	Mean	SD	%CV
Pre-dose	<5	<5	<5	N/A	N/A	N/A
0.5	428	746	380	518	199	38
1	656	532	419	536	119	22
2	891	658	683	744	128	17
4	548	498	597	548	49.5	9
8	275	399	246	307	81.3	27
24	<5	<5	<5	N/A	N/A	N/A
Sulfoxide						
Time (hour)	ID 2853	ID 2854	ID 2855	Mean	SD	%CV
Pre-dose	<25	<25	<25	N/A	N/A	N/A
0.5	11800	11300	10200	11100	819	7
1	17900	12700	12100	14200	3190	23
2	20000	14700	17400	17400	2650	15
4	18200	17600	18100	18000	321	2
8	11500	14500	8730	11600	2890	25
24	<25	<25	190	63.3*	110	174
Sulfone						
Time (hour)	ID 2853	ID 2854	ID 2855	Mean	SD	%CV
Pre-dose	891	729	1210	943	245	26
0.5	1910	1700	2440	2020	381	19
1	4020	3030	4410	3820	711	19
2	7710	5980	9280	7660	1650	22
4	13400	13200	16800	14500	2020	14
8	20700	19600	24300	21500	2460	11
24	1790	6740	3350	3960	2530	64

Estimates of mean based on approximation that values below LLOQ are equal to zero. For values marked *, more than half of the individual levels were below LLOQ; descriptive statistics was reported even if strongly biased.

N/A: not applicable.

Table 12A1. Day 28 individual and mean (±SD, %CV) plasma concentrations (ng/mL) of Fexinidazole and sulfoxide and sulfone metabolites after oral 200 mg/kg/day of Fexinidazole in male Sprague Dawley rats.

Fexinidazole						
Time (hour)	ID 2841	ID 2842	ID 2843	Mean	SD	%CV
Pre-dose	30.7	29.6	<5	20.1	17.4	87
1	311	239	77.5	209	120	57
2	248	261	100	203	89.4	44
4	261	353	99.1	238	129	54
8	196	293	31.2	173	132	76
24	82.7	<5	<5	27.6*	47.7	173
48	<5	18.8	<5	6.27*	10.9	174
72	<5	<5	<5	N/A	N/A	N/A
Sulfoxide						
Time (hour)	ID 2841	ID 2842	ID 2843	Mean	SD	%CV
Pre-dose	1120	170	253	514	526	102
1	9180	7840	13100	10000	2730	27
2	9300	9360	13200	10600	2230	21
4	8570	11500	14100	11400	2770	24
8	6230	8420	6480	7040	1200	17
24	3190	61.1	<25	1080	1820	169
48	43.5	656	<25	233	367	158
72	<25	<25	<25	N/A	N/A	N/A
Sulfone						
Time (hour)	ID 2841	ID 2842	ID 2843	Mean	SD	%CV
Pre-dose	3690	1090	2080	2290	1310	57
1	8680	3990	7650	6770	2460	36
2	11900	6620	11900	10100	3050	30
4	17900	14500	23300	18600	4440	24
8	22200	22700	27700	24200	3040	13
24	5050	2060	686	2600	2230	86
48	572	985	96.1	551	445	81
72	63.2	170	<25	77.7	85.9	111

Estimates of mean based on approximation that values below LLOQ are equal to zero. For values marked *, more than half of the individual levels were below LLOQ; descriptive statistics was reported even if strongly biased.

N/A: not applicable.

Table 13A1. Day 28 individual and mean (±SD, %CV) plasma concentrations (ng/mL) of Fexinidazole and sulfoxide and sulfone metabolites after oral 200 mg/kg/day of Fexinidazole in female Sprague Dawley rats.

Fexinidazole						
Time (hour)	ID 2853	ID 2854	ID 2855	Mean	SD	%CV
Pre-dose	<5	<5	<5	N/A	N/A	N/A
1	886	465	803	718	223	31
2	943	962	390	765	325	43
4	675	575	279	510	206	40
8	352	262	138	251	107	43
24	<5	<5	64.8	21.6*	37.4	173
48	<5	<5	12.7	4.23*	7.33	173
72	<5	<5	<5	N/A	N/A	N/A
Sulfoxide						
Time (hour)	ID 2853	ID 2854	ID 2855	Mean	SD	%CV
Pre-dose	<25	<25	34.1	11.4*	19.7	173
1	17300	13500	24000	18300	5320	29
2	21200	21800	13000	18700	4920	26
4	21100	18300	9910	16400	5820	36
8	14100	10700	5400	10100	4380	43
24	<25	<25	2960	987*	1710	173
48	<25	<25	604	201*	349	174
72	<25	<25	<25	N/A	N/A	N/A
Sulfone						
Time (hour)	ID 2853	ID 2854	ID 2855	Mean	SD	%CV
Pre-dose	4120	1420	2690	2740	1350	49
1	4240	7110	17400	9580	6920	72
2	11300	9130	9520	9980	1160	12
4	18400	13700	14300	15500	2560	17
8	26200	19700	18400	21400	4180	20
24	8010	1680	10800	6830	4670	68
48	236	87.5	2730	1020	1480	145
72	39.2	<25	232	90.4	124	137

Estimates of mean based on approximation that values below LLOQ are equal to zero. For values marked *, more than half of the individual levels were below LLOQ; descriptive statistics was reported even if strongly biased.

N/A: not applicable.

Table 14A1. Day 1 individual and mean (±SD, %CV) plasma concentrations (ng/mL) of Fexinidazole and sulfoxide and sulfone metabolites after oral 800 mg/kg/day of Fexinidazole in male Sprague Dawley rats.

Fexinidazole						
Time (hour)	ID 2844	ID 2845	ID 2846	Mean	SD	%CV
Pre-dose	<5	<5	<5	N/A	N/A	N/A
0.5	393	529	2760	1230	1330	108
1	603	632	2320	1190	983	83
2	805	868	1980	1220	661	54
4	710	635	1410	918	427	47
8	452	430	1070	651	363	56
24	33.4	63.6	(1)	48.5	21.4	44
Sulfoxide						
Time (hour)	ID 2844	ID 2845	ID 2846	Mean	SD	%CV
Pre-dose	<25	<25	<25	N/A	N/A	N/A
0.5	11400	12100	15600	13000	2250	17
1	19900	15000	19700	18200	2770	15
2	25900	24300	34700	28300	5600	20
4	36500	27200	32500	32100	4670	15
8	27900	23600	34800	28800	5650	20
24	1750	2880		2320	799	34
Sulfone						
Time (hour)	ID 2844	ID 2845	ID 2846	Mean	SD	%CV
Pre-dose	<25	<25	<25	N/A	N/A	N/A
0.5	1320	1520	1670	1500	176	12
1	4330	3440	3970	3910	448	12
2	9980	9800	10400	10100	308	3
4	23600	20800	20600	21700	1680	8
8	39500	42100	39300	40300	1560	4
24	16000	15000		15500	707	5

(1) Not available since the animal died
N/A: not applicable.

Table 15A1. Day 1 individual and mean (±SD, %CV) plasma concentrations (ng/mL) of Fexinidazole and sulfoxide and sulfone metabolites after oral 800 mg/kg/day of Fexinidazole in female Sprague Dawley rats.

Fexinidazole						
Time (hour)	ID 2856	ID 2857	ID 2858	Mean	SD	%CV
Pre-dose	<5	<5	<5	N/A	N/A	N/A
0.5	1670	810	1100	1190	438	37
1	1330	1030	1470	1280	225	18
2	1690	1290	1140	1370	284	21
4	1250	1280	1200	1240	40.4	3
8	807	844	444	698	221	32
24	65.4	412	31.4	170	211	124
Sulfoxide						
Time (hour)	ID 2856	ID 2857	ID 2858	Mean	SD	%CV
Pre-dose	<25	<25	<25	N/A	N/A	N/A
0.5	12100	10900	16100	13000	2720	21
1	16300	16200	22100	18200	3380	19
2	23000	22500	23100	22900	321	1
4	28200	30300	33400	30600	2620	9
8	24700	28400	22800	25300	2850	11
24	2980	15600	1690	6760	7690	114
Sulfone						
Time (hour)	ID 2856	ID 2857	ID 2858	Mean	SD	%CV
Pre-dose	<25	<25	<25	N/A	N/A	N/A
0.5	1130	1060	1490	1230	231	19
1	2670	2870	4070	3200	757	24
2	7420	7490	9660	8190	1270	16
4	14800	18700	22200	18600	3700	20
8	29400	35000	37500	34000	4150	12
24	23800	67600	10600	34000	29800	88
N/A: not applicable.						

Table 16A1. Day 14 individual and mean (±SD, %CV) plasma concentrations (ng/mL) of Fexinidazole and sulfoxide and sulfone metabolites after oral 800 mg/kg/day of Fexinidazole in male Sprague Dawley rats.

Fexinidazole						
Time (hour)	ID 2844	ID 2845	ID 2846	Mean	SD	%CV
Pre-dose	<5	<5	8.15	2.72*	4.71	173
0.5	249	231	167	216	43.1	20
1	310	296	198	268	61	23
2	584	551	352	496	126	25
4	678	496	467	547	114	21
8	319	146	201	222	88.4	40
24	53.8	15.6	5.28	24.9	25.6	103
Sulfoxide						
Time (hour)	ID 2844	ID 2845	ID 2846	Mean	SD	%CV
Pre-dose	53.4	<25	293	115	156	136
0.5	7970	8210	7720	7970	245	3
1	9530	11600	8900	10000	1410	14
2	16200	19400	13900	16500	2760	17
4	17800	14900	16000	16200	1460	9
8	9240	4590	8200	7340	2440	33
24	1710	515	152	792	815	103
Sulfone						
Time (hour)	ID 2844	ID 2845	ID 2846	Mean	SD	%CV
Pre-dose	869	394	4080	1780	2010	113
0.5	3570	3560	5650	4260	1200	28
1	5900	7500	7580	6990	948	14
2	14600	18700	15300	16200	2190	14
4	29400	29300	30500	29700	666	2
8	32700	21600	28800	27700	5630	20
24	6140	1580	1430	3050	2680	88

Estimates of mean based on approximation that values below LLOQ are equal to zero. For values marked *, more than half of the individual levels were below LLOQ; descriptive statistics was reported even if strongly biased.

N/A: not applicable.

Table 17A1. Day 14 individual and mean (±SD, %CV) plasma concentrations (ng/mL) of Fexinidazole and sulfoxide and sulfone metabolites after oral 800 mg/kg/day of Fexinidazole in female Sprague Dawley rats.

Fexinidazole						
Time (hour)	ID 2856	ID 2857	ID 2858	Mean	SD	%CV
Pre-dose	6.94	<5	100	35.6	55.8	157
0.5	624	371	2740	1250	1300	104
1	545	541	2110	1070	905	85
2	573	890	2260	1240	897	72
4	607	852	1960	1140	721	63
8	508	1070	1250	943	387	41
24	112	142	(1)	127	21.2	17
Sulfoxide						
Time (hour)	ID 2856	ID 2857	ID 2858	Mean	SD	%CV
Pre-dose	304	40	6180	2170	3470	160
0.5	20000	14800	21200	18700	3400	18
1	22600	22200	26300	23700	2260	10
2	25100	34000	37600	32200	6430	20
4	22700	33800	37500	31300	7700	25
8	18400	32900	21800	24400	7580	31
24	5970	6770		6370	566	9
Sulfone						
Time (hour)	ID 2856	ID 2857	ID 2858	Mean	SD	%CV
Pre-dose	4940	3900	15600	8150	6480	80
0.5	8180	5150	16900	10100	6100	60
1	12300	9150	19900	13800	5530	40
2	21200	20400	33300	25000	7230	29
4	30200	38700	49600	39500	9720	25
8	38500	61800	50400	50200	11700	23
24	19000	26800		22900	5520	24

(1) Not available since the animal died.

Table 18A1. Day 28 individual and mean (±SD, %CV) plasma concentrations (ng/mL) of Fexinidazole and sulfoxide and sulfone metabolites after oral 800 mg/kg/day of Fexinidazole in male Sprague Dawley rats.

Fexinidazole						
Time (hour)	ID 2844	ID 2845	ID 2846	Mean	SD	%CV
Pre-dose	7.32	39.2	31.3	25.9	16.6	64
1	287	448	413	383	84.7	22
2	670	403	400	491	155	32
4	489	307	451	416	96	23
8	620	304	268	397	194	49
24	<5	<5	<5	N/A	N/A	N/A
48	85.7	10.1	24.4	40.1	40.2	100
72	<5	<5	<5	N/A	N/A	N/A
Sulfoxide						
Time (hour)	ID 2844	ID 2845	ID 2846	Mean	SD	%CV
Pre-dose	168	1600	1260	1010	748	74
1	447	14200	17100	10600	8900	84
2	18100	13100	17400	16200	2710	17
4	12800	10300	16000	13000	2860	22
8	12800	10300	8480	10500	2170	21
24	37.9	<25	<25	12.6*	21.9	174
48	<25	310	887	399	450	113
72	<25	106	33.7	46.6	54.2	116
Sulfone						
Time (hour)	ID 2844	ID 2845	ID 2846	Mean	SD	%CV
Pre-dose	3090	5260	9970	6110	3520	58
1	5490	13600	20600	13200	7560	57
2	20200	19200	25400	21600	3330	15
4	30100	27800	40500	32800	6770	21
8	30700	27500	33100	30400	2810	9
24	1060	576	1230	955	339	36
48	133	1210	3130	1490	1520	102
72	49.4	374	184	202	163	81

Estimates of mean based on approximation that values below LLOQ are equal to zero. For values marked *, more than half of the individual levels were below LLOQ; descriptive statistics was reported even if strongly biased.

N/A: not applicable.

Table 19A1. Day 28 individual and mean (±SD, %CV) plasma concentrations (ng/mL) of Fexinidazole and sulfoxide and sulfone metabolites after oral 800 mg/kg/day of Fexinidazole in female Sprague Dawley rats.

Fexinidazole					
Time (hour)	ID 2856	ID 2857	Mean	SD	%CV
Pre-dose	74.3	43.2	58.8	22	37
1	210	812	511	426	83
2	685	704	695	13.4	2
4	580	805	693	159	23
8	307	441	374	94.8	25
24	36.5	22.9	29.7	9.62	32
48	<5	27.5	13.8	19.4	141
72	<5	<5	N/A	N/A	N/A
Sulfoxide					
Time (hour)	ID 2856	ID 2857	Mean	SD	%CV
Pre-dose	5240	559	2900	3310	114
1	7020	26100	16600	13500	81
2	24400	22200	23300	1560	7
4	23900	27300	25600	2400	9
8	12900	16800	14900	2760	19
24	1960	1360	1660	424	26
48	291	1320	806	728	90
72	<25	185	92.5	131	142
Sulfone					
Time (hour)	ID 2856	ID 2857	Mean	SD	%CV
Pre-dose	13200	4010	8610	6500	76
1	4550	11900	8230	5200	63
2	26100	15800	21000	7280	35
4	44000	30700	37400	9400	25
8	30200	39800	35000	6790	19
24	6030	10300	8170	3020	37
48	1550	3250	2400	1200	50
72	28.6	1810	919	1260	137

Estimates of mean based on approximation that values below LLOQ are equal to zero.

N/A: not applicable.

Appendix 2. In-Study Bioanalytical Validation Data

Calibration data

Table 1A2. Analytical Performance: Back-Calculated Concentrations (ng/mL) of Fexinidazole Calibration Standard in Rat Plasma for Study Protocol 0504-2007.

Assay Date	Analytical Run Number	STD.1 5.00 ng/mL	STD.2 10.0 ng/mL	STD.3 50.0 ng/mL	STD.4 100 ng/mL	STD.5 250 ng/mL	STD.6 500 ng/mL	STD.7 900 ng/mL	STD.8 1000 ng/mL
31-Mar-2008	1	4.82	9.5	47.4	101	231	479	845	1020
		5.32	9.84	52.8	113	261	452	*1070	1100
01-Apr-2008	2	5.08	9.56	47.7	93.2	253	496	880	979
		4.97	10.4	47.2	103	250	533	1030	952
01-Apr-2008	3	4.65	11	46.9	98.7	239	494	884	878
		5.1	10.1	52	92.9	236	546	1030	1050
02-Apr-2008	4	4.92	11	49.7	91.7	251	470	933	979
		4.76	10.5	47.9	96.3	265	537	889	1000
02-Apr-2008	5	4.83	9.8	50.3	89.9	244	484	783	955
		5.26	9.76	52.9	103	256	511	1030	1070
03-Apr-2008	6	4.9	9.86	47.1	96	270	484	952	1020
		5.14	10.2	46.7	100	266	477	*1070	*1200
03-Apr-2008	7	5.18	10	51.2	98.3	250	500	948	1030
		4.83	*13.1	51.3	89.9	245	467	976	986
04-Apr-2008	8	4.73	10.7	49.1	97.6	242	459	966	973
		4.91	10.8	51.5	90	259	475	944	1060
04-Apr-2008	9	4.37	9.46	47.5	92.6	249	462	874	995
		5.72	10.2	52.8	104	257	484	949	1110
Mean		4.97	10.2	49.6	97.3	251	489	932	1010
SD		0.298	0.505	2.31	6.11	10.7	27.1	69.8	58.3
%CV		6	5	4.7	6.3	4.3	5.5	7.5	5.8
%Bias		-0.6	2	-0.8	-2.7	0.4	-2.2	3.6	1
n		18	17	18	18	18	18	16	17

*Accuracy more than 15%; excluded from regression analysis.

Table 2A2. Analytical Performance: Back-Calculated Concentrations (ng/mL) of Sulfone Calibration Standard in Rat Plasma for Study Protocol 0504-2007.									
**Assay Date**	**Analytical Run Number**	**STD.1 25.0 ng/mL**	**STD.2 50.0 ng/mL**	**STD.3 250 ng/mL**	**STD.4 500 ng/mL**	**STD.5 2500 ng/mL**	**STD.6 5000 ng/mL**	**STD.7 22500 ng/mL**	**STD.8 25000 ng/mL**
31-Mar-2008	1	24.4	48.6	256	549	2450	5080	*16900	21400
		25.1	51.2	280	*592	2760	4690	21000	23600
01-Apr-2008	2	24.1	44.9	227	458	2480	4920	*16100	*19400
		27.1	*61.1	263	571	2810	*6200	23000	22700
01-Apr-2008	3	25.8	48.3	237	514	2460	5240	*17700	*18500
		23.6	53.4	278	484	2590	*5910	21100	23200
02-Apr-2008	4	26.5	49.2	234	492	2640	4950	*18000	*20000
		24.2	49	248	485	2660	5670	*16700	22200
02-Apr-2008	5	20.5	50	252	457	2430	4770	*15200	*19900
		29.2	*95.5	278	524	2680	5510	20600	22800
03-Apr-2008	6	22.3	47	232	532	2610	4790	*17600	*20600
		28.5	50.3	236	533	2640	4920	21800	25200
03-Apr-2008	7	26.6	50.1	274	514	2650	5270	21100	21600
		23.1	*130	*324	508	2750	5180	21300	21900
04-Apr-2008	8	22.7	52.8	257	516	2450	4710	19900	23700
		24.7	56	276	503	2740	5310	19200	24700
04-Apr-2008	9	23.7	48.5	253	485	2600	4750	*17400	*20400
		26.6	49.4	267	539	2630	4880	*18400	22100
Mean		24.9	49.9	256	510	2610	5040	21000	22900
SD		2.23	2.69	18	30.8	117	296	1080	1200
%CV		9	5.4	7	6	4.5	5.9	5.1	5.2
%Bias		-0.4	-0.2	2.4	2	4.4	0.8	-6.7	-8.4
n		18	15	17	17	18	16	9	12
*Accuracy more than 15%; excluded from regression analysis.									

Table 3A2. Analytical Performance: Back-Calculated Concentrations (ng/mL) of Sulfoxide Calibration Standard in Rat Plasma for Study Protocol 0504-2007.									
**Assay Date**	**Analytical Run Number**	**STD.1 25.0 ng/mL**	**STD.2 50.0 ng/mL**	**STD.3 250 ng/mL**	**STD.4 500 ng/mL**	**STD.5 2500 ng/mL**	**STD.6 5000 ng/mL**	**STD.7 22500 ng/mL**	**STD.8 25000 ng/mL**
31-Mar-2008	1	24.1	47	248	554	2450	5010	19700	22400
		26.9	47.6	272	*600	2820	4720	24100	24600
01-Apr-2008	2	23.5	*39.4	216	448	2460	4880	*18500	*20100
		25.7	54.8	243	549	2680	*5870	25700	23200
01-Apr-2008	3	26.3	47.8	233	499	2500	5270	20600	*19800
		25	46.6	272	479	2580	*6040	24500	24500
02-Apr-2008	4	28	47.4	238	511	2830	5140	21800	22200
		22.5	50.4	258	500	2810	*6050	20200	24400
02-Apr-2008	5	21.8	45.4	246	455	2490	4730	*17700	*20900
		29.3	*94.6	262	510	2660	5450	23500	23500
03-Apr-2008	6	22.8	*41.4	223	514	2700	4830	20700	21900
		28.4	46.5	229	526	2730	4960	25600	26800
03-Apr-2008	7	27.6	46.8	260	493	2620	5100	23600	22100
		23.1	*122	*306	488	2640	5030	23900	22600
04-Apr-2008	8	23.4	44.6	241	499	2380	4610	22700	24900
		27.3	52.4	268	497	2710	5200	22100	25500
04-Apr-2008	9	23.7	44.9	254	486	2690	4930	20800	22300
		27.5	49.2	266	548	2710	5060	22100	24300
Mean		25.4	48	249	503	2640	4990	22600	23700
SD		2.34	2.88	17.2	29.7	134	224	1900	1460
%CV		9.2	6	6.9	5.9	5.1	4.5	8.4	6.2
%Bias		1.6	-4	-0.4	0.6	5.6	-0.2	0.4	-5.2
n		18	14	17	17	18	15	16	15
*Accuracy more than 15%; excluded from regression analysis.									

Calibration curves parameters

Table 4A2. Calibration Curve Parameters for Fexinidazole Calibration Standards in Rat Plasma for Study Protocol 0504-2007.

Run Date	Curve Number	Slope	Intercept	R^2	LLOQ ng/mL	ULOQ ng/mL	Regression Footnote(s)
31-Mar-2008	1	0.00460	0.00102	0.9939	5	1000	1
01-Apr-2008	2	0.00482	0.00105	0.9961	5	1000	1
01-Apr-2008	3	0.00503	-0.00059	0.993	5	1000	1
02-Apr-2008	4	0.00508	0.00008	0.9966	5	1000	1
02-Apr-2008	5	0.00480	0.00090	0.9941	5	1000	1
03-Apr-2008	6	0.00472	0.00209	0.997	5	1000	1
03-Apr-2008	7	0.00500	0.00180	0.9974	5	1000	1
04-Apr-2008	8	0.00556	0.00035	0.9956	5	1000	1
04-Apr-2008	9	0.00439	0.00155	0.9934	5	1000	1
Mean		0.00489	0.00092	0.9952			
SD		0.00033	0.00086	0.0017			
%CV		6.8	93.6	0.2			
n		9	9	9			

Regression Footnote(s):
1) Resp. = Slope * Conc. + Intercept

Table 5A2. Calibration Curve Parameters for Sulfone Calibration Standards in Rat Plasma for Study Protocol 0504-2007.							
**Run Date**	**Curve Number**	**Slope**	**Intercept**	**R^2**	**LLOQ ng/mL**	**ULOQ ng/mL**	**Regression Footnote(s)**
31-Mar-2008	1	0.00423	0.00869	0.9929	25	25000	1
01-Apr-2008	2	0.00457	0.00539	0.9905	25	25000	1
01-Apr-2008	3	0.00529	0.01259	0.9954	25	25000	1
02-Apr-2008	4	0.00595	0.00952	0.9945	25	25000	1
02-Apr-2008	5	0.00520	0.00990	0.9878	25	25000	1
03-Apr-2008	6	0.00552	-0.00197	0.9939	25	25000	1
03-Apr-2008	7	0.00554	0.02695	0.9927	25	25000	1
04-Apr-2008	8	0.00751	0.00694	0.992	25	25000	1
04-Apr-2008	9	0.00449	0.00089	0.9953	25	25000	1
Mean		0.00537	0.00877	0.9928			
SD		0.00098	0.00821	0.0025			
%CV		18.3	93.6	0.3			
n		9	9	9			

Regression Footnote(s):
1) Resp. = Slope * Conc. + Intercept

**Table 6A2. Calibration Curve Parameters for Sulfoxide Calibration Standards in Rat Plasma for Study Protocol 0504-2007.**							
**Run Date**	**Curve Number**	**Slope**	**Intercept**	**R^2**	**LLOQ ng/mL**	**ULOQ ng/mL**	**Regression Footnote(s)**
31-Mar-2008	1	0.00432	0.01739	0.9923	25	25000	1
01-Apr-2008	2	0.00471	0.02234	0.9902	25	25000	1
01-Apr-2008	3	0.00524	0.02069	0.9955	25	25000	1
02-Apr-2008	4	0.00568	0.02901	0.9918	25	25000	1
02-Apr-2008	5	0.00519	0.02312	0.9911	25	25000	1
03-Apr-2008	6	0.00540	0.01665	0.99	25	25000	1
03-Apr-2008	7	0.00568	0.02696	0.9946	25	25000	1
04-Apr-2008	8	0.00764	0.01832	0.9956	25	25000	1
04-Apr-2008	9	0.00431	0.01868	0.994	25	25000	1
Mean		0.00535	0.02146	0.9928			
SD		0.00100	0.00431	0.0022			
%CV		18.8	20.1	0.2			
n		9	9	9			
Regression Footnote(s):<br>1) Resp. = Slope * Conc. + Intercept							

Individual and summary QC data

Table 7A2. Analytical Performance of Fexinidazole Quality Control Samples in Rat Plasma for Study Protocol 0504-2007.

Run Date	Curve Number	QC1 15.0 ng/mL	QC2 75.0 ng/mL	QC3 800 ng/mL	QC3 Dilution=2 800 ng/mL	QC4 Dilution=5 2000 ng/mL
31-Mar-2008	1	16.4	83.9	863		
		16.9	~86.8	903		
01-Apr-2008	2	16.8	82.2	828		
		~18.9	~90.8	903		
01-Apr-2008	3	14.7	80.9	889		
		16.5	~86.4	846		
02-Apr-2008	4	*36.4	79.7	823		
		16.7	84.5	847		
02-Apr-2008	5	16.7	85.7	803	814	
		17	~88.6	818	829	
					881	
03-Apr-2008	6	15.9	82.7	905		2140
		17.5	76.3	890		2180
						2240
03-Apr-2008	7	16.1	77.2	824		2010
		16.8	78.4	847		2000
						1860
04-Apr-2008	8	16.6	81.1	890		1970
		~18.6	~88.5	848		2240
						2150
04-Apr-2008	9	14.5	70.5	754		2170
		16.4	81	840		2100
						2270
Mean		16.6	82.5	851	841	2110
SD		1.1	5.05	40.6	35.2	126
%CV		6.6	6.1	4.8	4.2	6
%Bias		10.7	10	6.4	5.1	5.5
n		17	18	18	3	12

*Reason Deactivated: Error during sample preparation
~ > 15%Theoretical

**Table 8A2. Analytical Performance of Sulfone Quality Control Samples in Rat Plasma for Study Protocol 0504-2007.**						
**Run Date**	**Curve Number**	**QC1 75.0 ng/mL**	**QC2 750 ng/mL**	**QC3 20000 ng/mL**	**QC3 Dilution=2 20000 ng/mL**	**QC4 Dilution=5 50000 ng/mL**
31-Mar-2008	1	81.4	856	20000		
		79.1	~875	20900		
01-Apr-2008	2	76.6	811	18300		
		86.1	~999	~23800		
01-Apr-2008	3	70.3	842	20600		
		86.4	~886	20500		
02-Apr-2008	4	*775	817	19900		
		79.1	837	20100		
02-Apr-2008	5	79.2	852	21200	20700	
		84.7	~934	18900	21900	
					22800	
03-Apr-2008	6	74.1	822	19300		54700
		84.9	724	19900		53900
						55500
03-Apr-2008	7	73.1	791	22600		53400
		85.7	827	21700		52700
						48800
04-Apr-2008	8	81.9	815	21400		53600
		~91.2	~889	21200		55700
						54900
04-Apr-2008	9	79.4	703	17100		53500
		82.3	794	19000		53000
						56500
Mean		80.9	837	20400	21800	53900
SD		5.42	68.3	1570	1050	1980
%CV		6.7	8.2	7.7	4.8	3.7
%Bias		7.9	11.6	2	9	7.8
n		17	18	18	3	12
*Reason Deactivated: Error during sample preparation<br>~ > 15%Theoretical						

Table 9A2. Analytical Performance of Sulfoxide Quality Control Samples in Rat Plasma for Study Protocol 0504-2007.

Run Date	Curve Number	QC1 75.0 ng/mL	QC2 750 ng/mL	QC3 20000 ng/mL	QC3 Dilution=2 20000 ng/mL	QC4 Dilution=5 50000 ng/mL
31-Mar-2008	1	81.9	826	18900		
		80.4	834	20000		
01-Apr-2008	2	75.8	786	17500		
		86.1	~955	22100		
01-Apr-2008	3	72.3	826	19900		
		89.4	~881	19800		
02-Apr-2008	4	*972	821	20200		
		78.2	852	20200		
02-Apr-2008	5	79.2	824	20100	19800	
		86	~898	18300	20800	
					22000	
03-Apr-2008	6	74.2	812	19000		53400
		85.6	724	19500		51900
						54700
03-Apr-2008	7	72.9	756	20800		49300
		85.1	799	20400		48300
						44700
04-Apr-2008	8	83.8	788	19900		50700
		~90.4	~868	20100		51800
						51100
04-Apr-2008	9	80.5	713	17000		52900
		84.5	809	18800		51500
						55900
Mean		81.5	821	19600	20900	51400
SD		5.53	59	1200	1100	2970
%CV		6.8	7.2	6.1	5.3	5.8
%Bias		8.7	9.5	-2	4.5	2.8
n		17	18	18	3	12

*Reason Deactivated: Error during sample preparation
~ > 15%Theoretical

Appendix 3. Certificates of Analysis

[illegible]ASYNTH [illegible]NDUSTRIES — ORGASYNTH groupe

ANALYSIS CERTIFICATE

FEXINIDAZOLE

Manufact. date October-2007
Expiry date September-2008

DATE	October 15 , 2007
ANALYSIS N° CA	07-A-0495
SAMPLE N°	ECH:07285/23
WEIGHT (g)	10

DETERMINATIONS	RESULTS	SPECIFICATIONS
IDENTIFICATION	conforms	IR Spectrum complies
APPERANCE	powder	powder
COLOUR	yellow	yellow
LOSS ON DRYING (%)	0,1	<= 0,5
SULPHATED ASH (%)	0,0	<= 0,1
$HClO_4$ ASSAY (%)	98,5	98,5 à 101,5
RELATED SUBSTANCES - HPLC- Any known impurity (%)	nd	<= 0,15
RELATED SUBSTANCES - HPLC- Any other impurity (%)	aucune	<= 0,10
RELATED SUBSTANCES - HPLC - All impurities sum (%)	0,1	<= 0,5
RESIDUAL SOLVENTS - Acetone (ppm)	2887	<= 5000

CONFORMITY	YES / NO

Grasse, 15/10/2007
M.CONNAN
Quality Control Manager
nc

AFAQ ISO 9001 AFAQ N° 1997/7388

Siège social et usine : Chemin de la Madeleine - B.P. 45249 - 06131 GRASSE Cédex - FRANCE
Téléphone : 33 (0)4 93 70 01 32 - Télécopie : 33 (0)4 93 70 05 65 - E-mail : orgasynth-ind.contact@orgasynth.com - www.orgasynth.com
S.A.S au Capital Social de 1 525 000 € - SIREN : 326 171 378 - R.C.S. 326 171 378 B Grasse - TVA : FR 68 326 171 378 - APE 241 G

Universität Zürich-Irchel
Winterthurerstrasse 190
CH-8057 Zürich
Tel. + 41 (0)44 635 42 80
Fax + 41 (0)44 635 68 12

Analytisches Laboratorium
Organisch-chemisches Institut
Tel. + 41 (0)44 635 42 91
Fax + 41 (0)44 635 68 49
info@lpf.unizh.ch

Certificate of Analysis

Produktname

Chemical Name: 1-Methyl-2-(4-methylsulfinyl-phenoxymethyl)-5-nitroimidazol
(Fexinidazol-sulfoxid)

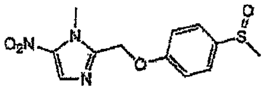

Molecular formula:	$C_{12}H_{13}N_3O_4S$
Molecular weight:	295.31
Project-Code:	PU 83 IIII
Manufacturer:	Gruppe Prof. Dr. J. S. Siegel, PU<br>University of Zurich, Switzerland
Manufacturing Date:	11.2006
Analytical Analysis Order:	1106-I-0170
Test date:	06.11.06
Retest date:	n/a
History of CoA:	06.11.06

Tests			Results
1.	Appearance	[visual]	colourless powder
2.	1H-NMR / MS	[CDCl3]	confirms structure
3.	FT-IR (ATR)	[cm-1]	for information only
4.	HPLC Purity (LPF123G3.M)	[Area-%]	99.34
4.1	Main Impurity		0.59
4.2	Sum of Impurities		0.62
5.	Water (KFT)	[%]	0.04
6.	Chlorides (T, 0.005 N $AgNO_3$)	[%]	na
7.	Residual Solvents (GC-Headspace)	[%]	
7.1	Ethanol		0.02
7.2	Ether		not detected

All results described above (except NMR) were obtained on equipment qualified according to GMP procedures in use at the Analytical Laboratory of the LPF (AnLab-OCI).

For investigational use according to ICH Q7A, Chapter 19

Issued by: Peter Uebelhart
ubelhart@oci.unizh.ch
Tel: +41 (0)44 635 42 74

Date: 07.11.2006

Universität Zürich-Irchel
Winterthurerstrasse 190
CH-8057 Zürich
Tel. + 41 (0)44 635 42 80
Fax + 41 (0)44 635 68 12

Analytisches Laboratorium
Organisch-chemisches Institut
Tel. + 41 (0)44 635 42 91
Fax + 41 (0)44 635 68 49
info@lpf.unizh.ch

Certificate of Analysis

Produktname

Chemical Name: 1-Methyl-2-(4-methylsulfonyl-phenoxymethyl)-5-nitroimidazol
(Fexinidazol-sulfon)

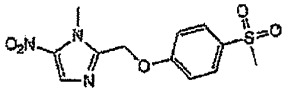

Molecular formula: $C_{12}H_{13}N_3O_5S$
Molecular weight: 311.31
Project-Code: PU 86
Manufacturer: Gruppe Prof. Dr. J. S. Siegel, PU
University of Zurich, Switzerland
Manufacturing Date: 10.2006
Analytical Analysis Order: 1106-I-0171
Test date: 07.11.06
Retest date: n/a
History of CoA: 07.11.06

Tests			Results
1.	Appearance	[visual]	slightly yellow powder
2.	1H-NMR / MS	[$CDCl_3$]	confirms structure
3.	FT-IR (ATR)	[cm-1]	for information only
4.	HPLC Purity (LPF123G3.M)	[Area-%]	99.92
4.1	Main Impurity		0.05
4.2	Sum of Impurities		0.07
5.	Water (KFT)	[%]	0.06
6.	Chlorides (T, 0.005 N $AgNO_3$)	[%]	na
7.	Residual Solvents (GC-Headspace)	[%]	
7.1	2-Propanol		0.07
7.2	EtOAc		0.02

All results described above (except NMR) were obtained on equipment qualified according to GMP procedures in use at the Analytical Laboratory of the LPF (AnLab-OCI).

For investigational use according to ICH Q7A, Chapter 19

Issued by: Peter Uebelhart
ubelhart@oci.unizh.ch
Tel: +41 (0)44 635 42 74

Date: 07.11.2006

Appendix 12 Pharmacy Documentation

Pharmacy Certification

STUDY NUMBER : **0504-2007**

TEST ITEM : **Fexinidazole**

NOTEBOOK NUMBER(S) : **19202/B**

DOCUMENTATION ENCLOSED IN THE NOTEBOOK:

A. Analysis certificate dated December 18, 2007 issued by Orgasynth Industries for Fexinidazole test item, raw material, Batch No. 3168-07-01/O
B. Material safety data sheet issued by Orgasynth Industries for Fexinidazole
C. Certificate of Analysis issued by Sigma-Aldrich for Tween® 80, raw material, Lot No. 1324202
D. Certificate of Analysis issued by Sigma-Aldrich for Methylcellulose 400 cP, raw material, Lot No. 105K0196
E. Certificate of Analysis issued by Sigma-Aldrich for Methylcellulose 400 cP, raw material, Lot No. 017K0087
F. Label's photocopy of Acqua per preparazioni iniettabili (Bieffe Medital S.p.A.), raw material, Lot No. 07G0201
G. Label's photocopy of Acqua per preparazioni iniettabili (Bieffe Medital S.p.A.), raw material, Lot No. 07K1503
H. Request cards for Fexinidazole test item

ANALYTICAL DOCUMENTATION:

Requests and Analytical Results issued by Accelera/ADMET/Preclinical Formulation and Accelera/DMPK&ART/Bioanalysis and Analytical Control

MATERIALS USED FOR THE STUDY:

1. Fexinidazole test item, raw material, Batch No. 3168-07-01/O
2. Tween® 80, raw material, Lot No. 1324202
3. Methylcellulose 400 cP, raw material, Lot No. 105K0074
4. Methylcellulose 400 cP, raw material, Lot No. 017K0087
5. Acqua per preparazioni iniettabili, raw material, Lot No. 07G0201
6. Acqua per preparazioni iniettabili, raw material, Lot No. 07K1503

PREPARATIONS:

Prepare suspension of Fexinidazole test item, raw material, Lot No. 3168-07-01/O in the vehicle (5% Tween® 80 in 0.5% Methylcellulose 400 cP solution) at the concentration of 5 mg/mL, 20 mg/mL and 80 mg/mL

Pharm. Cert. 0504-2007

CONCENTRATION CHECKS:

Fexinidazole suspensions:

		% of L.A.	Preparation date
80 mg/mL	Request No. 200800027 (Top)	100.23%	February 04, 2008
80 mg/mL	Request No. 200800028 (Middle)	97.84%	February 04, 2008
80 mg/mL	Request No. 200800029 (Bottom)	103.20%	February 04, 2008
20 mg/mL	Request No. 200800030 (Top)	97.48%	February 04, 2008
20 mg/mL	Request No. 200800031 (Middle)	99.01%	February 04, 2008
20 mg/mL	Request No. 200800032 (Bottom)	95.21%	February 04, 2008
5 mg/mL	Request No. 200800033 (Top)	100.83%	February 04, 2008
5 mg/mL	Request No. 200800034 (Middle)	101.22%	February 04, 2008
5 mg/mL	Request No. 200800035 (Bottom)	104.67%	February 04, 2008
80 mg/mL	Request No. 200800057 (Top)	98.45%	February 29, 2008
80 mg/mL	Request No. 200800058 (Middle)	96.50%	February 29, 2008
80 mg/mL	Request No. 200800059 (Bottom)	95.69%	February 29, 2008
20 mg/mL	Request No. 200800060 (Top)	99.95%	February 29, 2008
20 mg/mL	Request No. 200800061 (Middle)	98.18%	February 29, 2008
20 mg/mL	Request No. 200800062 (Bottom)	97.27%	February 29, 2008
5 mg/mL	Request No. 200800063 (Top)	98.72%	February 29, 2008
5 mg/mL	Request No. 200800064 (Middle)	107.40%	February 29, 2008
5 mg/mL	Request No. 200800065 (Bottom)	94.66%	February 29, 2008

STABILITY:

Fexinidazole test item:

Expire date October 2008 for Fexinidazole, test item, raw material, Lot No. 3168-07-01/O if stored at room temperature protected from light.

Fexinidazole suspension:

Stability data indicate that Fexinidazole suspensions in the vehicle (5% Tween® 80 in 0.5% Methylcellulose 400 cP solution) in the range 0.5-100 mg/mL are stable up to 7 days at room temperature and 14 days at +4°C (Nerviano MS 0293-2007-R)

Date: April 17, 2008

Appendix 13 Clinical Pathology Methods

HEMATOLOGY METHODS

PARAMETERS	CODE	UNIT	METHODS AND INSTRUMENTS
Red blood cells	RBC	n x 10^6/µL	Laser beam method. " ADVIA 120™ System" Bayer
Hemoglobin	HGB	g/dL	Colorimetric method as cyanmethemoglobin. "ADVIA 120™ System " Bayer
Hematocrit	HCT	%	Calculated: Wintrobe formulae “ADVIA 120™ System" Bayer
Mean corpuscular hemoglobin concentration	MCHC	g/dL	Calculated: Wintrobe formulae "ADVIA 120™ System" Bayer
Mean corpuscular volume	MCV	fL	Mean of RBC volume histogram x MCV calibration factor. "ADVIA 120™ System" Bayer
Mean corpuscular hemoglobin	MCH	pg	Calculated: Wintrobe formulae "ADVIA 120™ System" Bayer
Red cell distribution width	RDW	%	Calculated: Standard deviation of RBC volume histogram/MCV (fl) x 100. "ADVIA 120™ System" Bayer
Hemoglobin distribution width	HDW	g/dL	Calculated: Standard deviation of RBC HC histogram. "ADVIA 120™ System" Bayer
Reticulocytes	R	%	Laser beam method with colorimetric reaction for nucleic acids. "ADVIA 120™ System" Bayer
Reticulocytes absolutes	RAB	n x 10^9/L	Laser beam method with colorimetric reaction for nucleic acids. "ADVIA 120™ System" Bayer
Mean corpuscolar volume of reticulocytes	MCVr	fL	Mean of R volume histogram x MCV calibration factor. "ADVIA 120™ System" Bayer
Mean hemoglobin concentration of reticulocytes	CHCM	g/dL	Mean of R hemoglobin histogram "ADVIA 120™ System" Bayer
Cellular hemoglobin of reticulocytes	CHr	pg	Calculated: Wintrobe formulae "ADVIA 120™ System" Bayer
Platelets	PLT	n x 10^3/µL	Laser beam method. "ADVIA 120™ System" Bayer
Mean platelet volume	MPV	fL	Mean platelet volume histogram "ADVIA 120™ System" Bayer
Platelet distribution width	PDW	%	Standard deviation of platelet volume histogram/ MPV (fl) x 100 "ADVIA 120™ System" Bayer
Platelet hematocrit	PCT	%	Mean platelet volume x PLT. "ADVIA 120™ System" Bayer
White blood cells	WBC	n x 10^3/µL	Laser beam method with “Baso/Lobularity method” and Peroxidase reaction. "ADVIA 120™ System" Bayer

HEMATOLOGY METHODS (Cont.)

PARAMETERS	CODE	UNIT	METHODS AND INSTRUMENTS
WBC differential count:<br>Neutrophils<br>Lymphocytes<br>Eosinophils<br>Basophils<br>Monocytes<br>Large unstained cells	 N<br>LY<br>E<br>B<br>M<br>LU	%	Peroxidase and Basophil/Lobularity method "ADVIA 120™ System" Bayer
Band Neutrophils<br>Segmented Neutrophils	BANN<br>SEGN		Percentage of Band and Segmented Neutrophils manually calculated on 100 Neutrophils examined at microscopy
WBC differential count:<br>Neutrophils ABS<br>Lymphocytes ABS<br>Eosinophils ABS<br>Basophils ABS<br>Monocytes ABS<br>Large unstained cells ABS	 NAB<br>LYAB<br>EAB<br>BAB<br>MAB<br>LUAB	n x 10^3/µL	Peroxidase and Basophil/Lobularity method "ADVIA 120™ System" Bayer
Band Neutrophils ABS<br>Segmented Neutrophils ABS	BAAB<br>SEAB		Absolute number of Band and Segmented Neutrophils calculated on percentage of Band and Segmented Neutrophils and value of NAB

CLINICAL CHEMISTRY METHODS

PARAMETERS	CODE	UNIT	METHODS AND INSTRUMENTS
Urea	UREA	mg/dL	Urease - GLDH'': enzymatic UV test.<br>Kit: Horiba ABX adapted to the "Pentra 400" analyzer.
Creatinine	CREA	mg/dL	Colorimetric complex between the creatinine and the alkaline picrate (Jaffé).<br>Kit: Horiba ABX adapted to the "Pentra 400" analyzer.
Aspartate aminotransferase	AST	IU/L	UV-test according to IFCC modified method without pyridoxal phosphate.<br>Kit: Horiba ABX adapted to the "Pentra 400" analyzer.
Alanine aminotransferase	ALT	IU/L	UV-test according to IFCC modified method without pyridoxal phosphate.<br>Kit: Horiba ABX adapted to the "Pentra 400" analyzer.
Alkaline phosphatase	AP	IU/L	Kinetic photometric test, according to the IFCC.<br>Kit: Horiba ABX adapted to the "Pentra 400" analyzer.
G-Glutamyl transferase	GGT	IU/L	Kinetic photometric test according to Szasz modified.<br>Kit: Horiba ABX adapted to the "Pentra 400" analyzer.
Total bilirubin	T.BIL	mg/dL	Photometric test using 2,4-dichloroaniline.<br>Kit: Horiba ABX adapted to the "Pentra 400" analyzer.
Total proteins	TPRO	g/dL	Biuret reaction. End-point method.<br>Kit: Horiba ABX adapted to the "Pentra 400" analyzer.
Albumin	ALB.	g/dL	Colorimetric determination using bromocresol green.<br>Kit: Horiba ABX adapted to the "Pentra 400" analyzer.
Globulin	GLOB	g/dL	Calculated parameter (T.Protein-Albumin) by Xybion.
Glucose	GLUC	mg/dL	Enzymatic determination. (Trinder method).<br>Kit: Horiba ABX adapted to the "Pentra 400" analyzer.
Triglycerides	TG	mg/dL	Enzymatic determination<br>Kit: Horiba ABX adapted to the "Pentra 400" analyzer.
Total Cholesterol	TCHO	mg/dL	''CHOD-PAP'': enzymatic photometric test. (Trinder's reaction).<br>Kit: Horiba ABX adapted to the "Pentra 400" analyzer.
Calcium	Ca	mg/dL	Photometric test using ortho-cresolphtalein complexone (OPC).<br>Kit: Horiba ABX adapted to the "Pentra 400" analyzer.
Phosphorus	PHOS	mg/dL	UV method using phosphomolybdate<br>Kit: Horiba ABX adapted to the "Pentra 400" analyzer.
Sodium	Na	mEq/L	Direct potentiometry ion selective electrodes.<br>ISE module on the "Pentra 400" analyzer
Chloride	Cl	mEq/L	Direct potentiometry ion selective electrodes.<br>ISE module on the "Pentra 400" analyzer
Potassium	K	mEq/L	Direct potentiometry ion selective electrodes.<br>ISE module on the "Pentra 400" analyzer
Albumin/Globulin	A/G		Calculated parameter by Xybion

URINALYSIS METHODS

PARAMETERS	CODE	UNIT	METHODS AND INSTRUMENTS
Appearance			
Limpid	L		Turbidimetric method with Urysis 2400 (Roche).
Turbid	T		
Light Turbid	LT		
Colour:			
Yellow	Y		Photometric method with Urysis 2400 (Roche).
Amber	A		
Orange	O		
Greenish	G		
Reddish	R		
Brownish	B		
Colorless	W		
Other	O		
Volume	VOL	ml/16h	
Specific Gravity	S.G.		Refractometric method with Urysis 2400 (Roche).
pH	pH		
Proteins	PRO	Score 0-4	Semi-quantitative determination with Sticks Urysis 2400 Cassette (Roche).
Nitrites	NIT	" 0-1	
Glucose	GLU	" 0-4	
Ketones	KETO	" 0-3	
Urobilinogen	UBG	" 0-3	Photometric method with Urysis 2400 (Roche).
Bilirubin	BIL	" 0-2	
Hemoglobin/Red blood cells	ERY	" 0-3	
White blood cells	WBC	" 0-2	

URINALYSIS METHODS (Cont.)

The scoring system is as follows:

SCORE	PROTEINS mg/dL	NITRITES	GLUCOSE mg/dL	KETONES mg/dL	UROBIL. mg/dL	BILIR. mg/dL	WHITE B.CELLS WBC/μ L	HEMOGLOBIN ERYTHROC. RBC/μL
0	Absent	Absent	Absent	Up to 5	Up to 1	Up to 1	Up to 25	Up to 10
1	Up to 25	Present	Up to 50	Up to 15	Up to 4	Up to 3	Up to 100	Up to 50
2	Up to 75	…	Up to 100	Up to 50	Up to 8	Up to 6	Up to 500	Up to 150
3	Up to 150	…	Up to 300	Up to 150	Up to 12	…	…	Up to 250
4	Up to 500	…	Up to 1000	…	…	…	…	…
