# Supplementary material for: Fexinidazole – A New Oral Nitroimidazole Drug Candidate Entering Clinical Development for the Treatment of Sleeping Sickness
Source: PLoS Negl Trop Dis. 2010 Dec 21;4(12):e923. doi: 10.1371/journal.pntd.0000923 (PMC3006138; doi:10.1371/journal.pntd.0000923)
Supplement: Dataset S17 — (2.32 MB PDF) [file pntd.0000923.s018.pdf]

Fexinidazole: 28-day Oral Toxicity Study in the Dog

Product Name :	Fexinidazole
Study Number:	0505-2007
Study Director/Author:	
Sponsor Reference Study No.:	Not applicable
Status:	Final

SUMMARY

Methods

Fexinidaxole is a 5-nitroimidazole derivative biologically active against Trypanosoma parasites (*T.b. rhodesiense* and *T.b. brucei*).
The test article was given daily for twenty-eight days to five (control and high dose) or three (low and mid dose) beagle dogs/sex/dose at the doses of 0 (control group), 50, 200 and 800 mg/kg/day. The compound was administered orally, by gavage, as a suspension in 5% Tween 80 / 0.5% Methocel in a volume of 10 mL/kg/day. The control group received the vehicle alone (5% Tween 80 in 0.5% Methocel). At the end of the treatment period three animals/sex/group were sacrificed; the remaining animals of the control and high-dose groups were allowed a two-week recovery period.
Body weight was recorded weekly. Clinical signs and food consumption were recorded daily. Electrocardiographic, ophthalmoscopic and clinical pathology examinations were performed pretest and at the end of the treatment and recovery period.
A complete necropsy was performed on animals at the scheduled sacrifices (end of treatment and end of recovery); selected organs were weighed and collected. Histopathological examination was performed on all collected organs/tissues from control and high dose groups and additionally on bone marrow smears, sternum, mammary glands, ovaries and uterus from low and mid dose groups.
Systemic exposure to fexinidazole and of its sulfoxide and sulfone metabolites was evaluated on Days 1, 14 and 28 in the same animals used for the toxicological study.

Results

No mortality and no treatment-related clinical signs were observed during the study.
A slight to moderate reduction in food intake was observed in four females given 800 mg/kg/day throughout the treatment period. Food intake was normal after the end of the treatment. Concurrently, slight to moderate progressive body weight loss was seen in the same four females. Two males of the high dose group showed a progressive moderate body weight loss without changes in food consumption.
No treatment-related findings were noted at electrocardiographic and ophthalmoscopic examination.

A minimal to slight decrease in lymphocytes was observed mainly at the high dose in both sexes. No meaningful changes were observed at clinical pathology.

At necropsy, no drug-related changes were observed at gross pathology or in organ weights, either at the end of the treatment or after recovery.
At the end of the treatment period, moderately reduced cellularity of the bone marrow, severe involution of the thymus and slight to moderate atrophy of the adipose tissue in the sternal and femoral marrow spaces and in the fat deposits adjacent to the renal pelvis were

noted in a single high dose male. Minimally reduced cellularity was also noted in the bone marrow of one male treated with 200 mg/kg/day. These changes were considered as a consequence of the body weight loss.
At the end of the recovery period, increased severity of involution was noted in the thymus of both sexes compared with controls, secondary to body weight loss.

After single and repeated administrations at the three dose levels, no relevant gender difference in the systemic exposure to fexinidazole was observed. AUCs of fexinidazole increased less than expected assuming dose proportionality in the dose range investigated. No accumulation of fexinidazole was observed.
Fexinidazole was extensively metabolized to the sulfone and sulfoxide derivatives both after single and repeated administration. No accumulation of either metabolite was observed.

Mean ±SD systemic exposure to fexinidazole is reported in the following table:

		Male			Female		
Dose (mg/kg/ day)		Cmax (ng/mL)	tmax (hour)	AUC0-t(last) (ng·hour/mL)	Cmax (ng/mL)	tmax (hour)	AUC0-t(last) (ng·hour/mL)
50 (n=3)	D1	31.2±12.4	0.5±0	140±147	42.4±10.6	1±0.87	237±105
	D14	26.5±13	0.5±0	52.1±29.4	41.9±2.2	1.67±0.58	246±117
	D28	20.3±7.89	1±0	124±67	36.3±15.1	1±0	87.1±38.1
200 (n=3)	D1	54.9±10.8	1±0.87	419±61.4	84.1±36.7	1±0	454±119
	D14	78.1±23.5	1±0.87	452±41.1	77.7±50.8	2±0	443±217
	D28	57.9±12.5	1±0	395±15.8	73±12	1±0	377±83.5
800 (n=5)	D1	100±21.2	1.1±0.55	776±182	184±75.6	1.2±0.45	895±437
	D14	128±56.9	1±0	929±268	152±44	1.4±0.55	1170±309
	D28	86.2±38.5	1.2±0.4	736±141	101±47.1	1±0	956±378

Mean ±SD systemic exposure to the sulfone metabolite is reported in the following table:

		Male			Female		
Dose (mg/kg/ day)		Cmax (μg/mL)	tmax (hour)	AUC0-t(last) (μg·hour/mL)	Cmax (μg/mL)	tmax (hour)	AUC0-t(last) (μg·hour/mL)
50 (n=3)	D1	7.17±1.74	8±0	126±28.1	10±1.6	6.67±2.31	170±37.8
	D14	5.57±2.19	4±0	78±35.3	9.98±1.25	6.67±2.31	173±26.9
	D28	7.11±1.21	8±0	121±19.1	6.79±0.56	3.33±1.15	107±22.5
200 (n=3)	D1	17.2±1.69	13.3±9.24	338±50.8	18.1±3.42	12±10.6	358±85.7
	D14	22±5.59	5.33±2.31	387±49.5	21.5±2.42	5.33±2.31	381±61.2
	D28	14±3.31	4±4	258±48	15.4±2.66	11.7±10.1	277±29.9
800 (n=5)	D1	38.6±2.83	17.6±8.76	705±94.9	33.6±10.9	11.2±7.16	614±216
	D14	34.6±7.42	5.6±2.19	640±163	38.6±4.73	8±0	667±67.8
	D28	20.8±4.14	12.4±9.81	388±68.2 (1)	26±7.7	7.2±1.79	477±145 (1)
				601±29 (2)			693±351 (2)

(1) AUC0-24; (2) n=2

Mean ±SD systemic exposure to the sulfoxide metabolite is reported in the following table:

		Male			Female		
Dose (mg/kg/day)		Cmax (µg/mL)	tmax (hour)	AUC0-t(last) (µg·hour/mL)	Cmax (µg/mL)	tmax (hour)	AUC0-t(last) (µg·hour/mL)
50 (n=3)	D1	3.55±1.3	1±0	19.2±5.82	3.97±0.74	1.33±0.58	20±6.81
	D14	2.24±0.45	1±0	8.51±2.53	3.73±0.73	1.67±0.58	18.8±2.06
	D28	1.83±0.57	1.67±0.58	14.2±2.72	2.72±0.73	1±0	9.88±2.72
200 (n=3)	D1	7.55±1.03	1.67±0.58	50.7±8.61	8.7±3.95	0.83±0.29	52.1±20.6
	D14	8.96±2.53	2±0	56.1±9.96	9.02±3.69	2±0	57.2±24.3
	D28	5.76±1.17	1.33±0.58	42.2±6.46	5.43±0.34	1±0	33.8±1.65
800 (n=5)	D1	13.4±3.22	1.5±0.71	104±11.3	15.6±4.64	1.6±0.55	121±44.4
	D14	12.5±2.85	1.2±0.45	113±39.9	14.8±5.1	2±1.22	144±37.1
	D28	9.35±3.55	1.6±0.55	74.3±14.4(1)	9.48±3.38	1.4±0.55	89±45.2
				121±33(2)			

(1) AUC0-24; (2) n=2

Conclusions

Fexinidazole, given orally to beagle dogs at the doses of 50, 200 and 800 mg/kg/day for 28 consecutive days, was well tolerated.
Slight to moderate body weight loss and reduction in food intake was observed at the dose of 800 mg/kg/day during the treatment period.
A minimal to slight decrease in lymphocytes was seen at the high dose.
Fexinidazole was extensively metabolized to the sulfone and sulfoxide derivatives both after single and repeated administration.
In the conditions applied in the present study, the dose of 200 mg/kg/day can be considered as the NOEL.

TABLE OF CONTENTS

1. INTRODUCTION AND OBJECTIVES 10
2. STUDY SPONSOR 10
3. TEST FACILITY 10
4. REGULATORY REQUIREMENTS 10
5. SCHEDULE 10
6. MATERIALS AND METHODS 11
6.1. Test and Control Items 11
6.1.1. Test Item 11
6.1.2. Vehicle/Control Item 11
6.1.3. Test Formulation 11
6.1.4. Test Formulation Analyses 11
6.2. Test System 12
6.2.1. Allocation/Randomization 12
6.2.2. Identification 12
6.2.3. Environmental Conditions 12
6.3. Experimental Design 13
6.3.1. Dose Administration 13
6.3.2. Dose Justification 14
6.4. Clinical and physical examinations 14
6.5. Clinical Pathology 15
6.5.1. Hematology 15
6.5.2. Coagulation 15
6.5.3. Clinical Chemistry 15
6.5.4. Urine Analysis 16
6.6. Post mortem examinations 16
6.6.1. Unscheduled Deaths 16
6.6.2. Scheduled Necropsy, Tissue Collection, and Tissue Examination 16
6.6.3. Tissue Preparation 18
6.6.4. Peer review 18

6.7. Systemic Exposure 18
7. DATA ACQUISITION 19
8. STATISTICAL ANALYSIS 19
8.1. Variables 19
8.2. Methods 19
9. ARCHIVING 19
10. STUDY DEVIATIONS 20
11. STUDY PERSONNEL 20
12. RESULTS 20
12.1. Clinical and Physical Examination 20
12.1.1. Mortality 20
12.1.2. Clinical Signs 20
12.1.3. Body Weight 20
12.1.4. Food consumption 21
12.1.5. Electrocardiographic examination 21
12.1.6. Ophthalmoscopic examination 22
12.2. Clinical pathology 22
12.2.1. Hematology 22
12.2.2. Coagulation 22
12.2.3. Clinical chemistry 23
12.2.4. Urinalysis 23
12.3. Post-mortem examinations 23
12.3.1. Organ Weights 23
12.3.2. Gross Pathology 23
12.3.3. Histopathology 24
12.4. Systemic Exposure Evaluation 24
13. DISCUSSION 27
14. CONCLUSIONS 28
15. REFERENCES 28
16. FIGURES 30
16.1. Body weight 30

TABLES

Table 1 Clinical Signs
Table 2 Body Weights
Table 3 ECG Examinations
Table 4 Ophthalmoscopic Examination
Table 5 Hematology
Table 6 Coagulation
Table 7 Clinical Chemistry
Table 8 Urinalysis, Quantitative
Table 9 Urinalysis, Semi-quantitative
Table 10 Absolute Organ Weights
Table 11 Organ/Terminal Body Weight Ratios
Table 12 Gross Pathology
Table 13 Microscopic Pathology
Table 14 Histological changes in ovaries
Table 15 Stage of Estrus Evaluation

APPENDICES

Appendix 1 QA Statement
Appendix 2 Clinical Signs
Appendix 3 Body Weights
Appendix 4 ECG Examinations
Appendix 5 Ophthalmoscopic Examination
Appendix 6 Hematology
Appendix 7 Coagulation
Appendix 8 Clinical Chemistry
Appendix 9 Urinalysis
Appendix 10 Absolute Organ Weights
Appendix 11 Organ/Terminal Body Weight Ratios
Appendix 12 Gross And Microscopic Pathology
Appendix 13 Toxicokinetic Report
Appendix 14 Clinical Pathology Methods
Appendix 15 Pharmacy Documentation
Appendix 16 Protocol and Amendment

1. INTRODUCTION AND OBJECTIVES

The purpose of this study (0505-2007) was to determine the tolerability of and systemic exposure to fexinidazole when given orally for 28 consecutive days to beagle dogs, by gavage. Reversal of possible toxic effects was evaluated in control and high-dose groups over a two-week observation period.
Systemic exposure was assessed in the same animals used for toxicological evaluation.

Fexinidazole is a 5-nitroimidazole derivative, biologically active against Trypanosoma parasites (*T.b.rhodesiense* and *T.b. brucei*) and useful in the treatment of the Human African trypanosomiasis (HAT), known as sleeping sickness.

2. STUDY SPONSOR

DND*i* – Drugs for Neglected Diseases *Initiative*
1 Place St Gervais
CH – 1201 Geneva, Switzerland

3. TEST FACILITY

Accelera
.

4. REGULATORY REQUIREMENTS

This study was conducted in compliance with:

- Decreto Legislativo 2 Marzo 2007, No. 50;
- Organisation for Economic Co-operation and Development (OECD) Principles of Good Laboratory Practice (GLP) (as revised in 1997).

The methods employed in this study were those described in the "Standard Operating Procedures" of the laboratories involved.

5. SCHEDULE

Experimental Start Date (Randomization Date)	January 24, 2008	
	Males	Females
First Dose (Day 1)	January 29, 2008	January 30, 2008
Last Dose (Day 28)	February 25, 2008	February 26, 2008
Necropsy – Dosing Phase (Day 29-30)	February 26-27, 2008	February 27-28, 2008
Start of recovery (Day 29)	February 26, 2008	February 27, 2008
Necropsy – End of Recovery period (Day 43)	March 11, 2008	March 12, 2008
Experimental Completion Date	March 12, 2008	

6. MATERIALS AND METHODS

6.1. Test and Control Items

6.1.1. Test Item

Identification	Fexinidazole
Lot/Batch Number	3168-07-01/O
Purity and Expiry	100% October 2008
Storage Conditions	Room temperature, protected from light
Source and Manufacturer	Centipharm (formerly Orgasynth Industries)
Special Handling Precautions	According to MSDS (Material Safety Data Sheet)
The amount of the test article received and used at Accelera, Nerviano Medical Sciences was recorded according to Accelera, Nerviano Medical Sciences' standard procedures.	

6.1.2. Vehicle/Control Item

Identification	5% Tween 80 in 0.5% Methyl cellulose 400 cP (Methocel) suspension in water
Lot/Batch Number	Tween 80 1324202<br>Methyl cellulose 400 cP 125K0196
Expiry	Tween 80 February 2011<br>Methyl cellulose 400 cP January 2009
Storage Conditions	Room Temperature
Source and Manufacturer	Tween 80 Sigma-Aldrich<br>Methyl cellulose 400 cP Sigma-Aldrich
Method of Preparation	On file of ADMET/Preclinical Formulation

6.1.3. Test Formulation

Type of Formulation	5% Tween 80 in 0.5% Methyl cellulose 400 cP (Methocel) suspension
Method of Preparation	On file at Accelera/Experimental ADMET/Preclinical Formulation
Frequency of Preparation	The suspensions were prepared according to the stability data
Dose Concentrations	5, 20, 80 mg/mL
Storage Conditions	Room temperature
Source and Manufacturer	Accelera/Experimental ADMET/Preclinical Formulation

6.1.4. Test Formulation Analyses

6.1.4.1. Concentration/Homogeneity

Samples (top-middle-bottom, 5 mL each) of each dose suspension prepared for the first and the last week of treatment (Weeks 1 and 4) were collected under stirring for fexinidazole concentration and homogeneity checks; 10 mL were also taken from the vehicle. After collection, samples were directly transferred at room temperature to Accelera/DMPK & ART/Bioanalysis and Analytical Control for analysis. The analyses were performed using a validated HPLC-UV method [1].

6.1.4.2. Stability

Stability data indicated that fexinidazole/5% Tween 80 in 0.5% Methyl cellulose 400 cP (Methocel) suspension in the range 0.5-100 mg/mL is stable for 7 days at room temperature or for 14 days at +4°C [1].
All values were found to be within acceptable limits for the solutions.

6.2. Test System

Species/Strain or Breed, Source, Sex	Male and female dog/Beagle, Marshall, Europe
Justification of Species	The dog has been used extensively in safety studies and a large amount of biological data is available.
Age	About one year
Weight	6.83 to 9.87 kg
Acclimation	At least 20 days
Selection Criteria	Acceptable findings from pretest observations, body weight, physical examinations, some laboratory examinations
Pretest Animals	20/sex

6.2.1. Allocation/Randomization

Animals were randomly assigned to treatment groups based on the most recent pretest body weight. After excluding animals with unacceptable pretest findings, a computer program included in the Xybion Path/Tox system designed to achieve balance with respect to pretest body weights was used to exclude animals from both body weight extremes and randomize the remaining animals to treatment groups.

6.2.2. Identification

Animals were identified by individual tattoos and numbered collars (collars could be removed if required).

A color-coded cage card was affixed to each cage and indicated the study number and type, treatment start date, test article, animal number, sex and dose level.

Test Group	Color Code	Animal ID numbers	
		Males	Females
1	White	2516, 2518, 2520<br>Recovery.: 2527, 2533	2560, 2568, 2572<br>Recovery: 2575, 2577
2	Yellow	2514, 2521, 2529	2562, 2563, 2576
3	Green	2515, 2523, 2526	2561, 2570, 2567
4	Red	2517, 2519, 2525<br>Recovery: 2528, 2530	2564, 2565, 2569<br>Recovery: 2571, 2573

6.2.3. Environmental Conditions

Caging	Individual pens – Rooms 11, 12, 13, 18, 19, 20/C
Bedding	None

Temperature	18°C to 21°C
Air changes	15 +/- 5 air changes per hour
Humidity	40% to 70% relative humidity; monitored
Lighting	Approximate 12-hour light, 12-hour dark cycle (light from 6 a.m. to 6 p.m.).
Water	Municipal mains, available ad libitum via an auto-watering system
Diet	Altromin H (A. Rieper, Vandoies, Italy), about 300 g/day

Actual conditions were continuously monitored, recorded and records retained.
Release of each lot of feed by the manufacturers was based on analysis of composite samples of each lot, which had met specifications set by the manufacturers. Water was periodically analyzed for chemical and microbial impurities. No contaminants were identified in the food or water, that were deemed to interfere with the results or conclusions of this study.

All the above environmental conditions, as well as all the procedures adopted throughout the study for housing and handling the animals were in strict compliance with EEC and Italian Guidelines for Laboratory Animal Welfare.

6.3. Experimental Design

The study was performed according to the following experimental scheme:

Test Group	Dose (*tentative*) (mg/kg/day)	Number of Animals/Group			
		Main Study		Recovery	
		Males	Females	Males	Females
1	0 (vehicle)[a]	3	3	2	2
2	50	3	3	-	-
3	200	3	3	-	-
4	800	3	3	2	2
a: 5% Tween 80 in 0.5% Methyl cellulose 400 cP (Methocel) suspension					

Animals were treated orally, by gavage, daily for 28 consecutive days. At the end of the treatment period, all animals from groups 2 and 3 and the first three animals in numerical order from groups 1 and 4 (or otherwise chosen on the basis of the outcome of the study) were sacrificed; the remaining animals were allowed a two-week recovery period.

6.3.1. Dose Administration

Method and Route	Os, gavage
Duration	28 days
Frequency	Once daily, at approximately the same time
Volume	10 mL/kg/day; Individual dose volumes were calculated based on the most recent body weight recorded
Rate	Not applicable
Special Notes	Stir during administration.

6.3.2. Dose Justification

The doses were chosen in agreement with the Sponsor on the basis of the results of preliminary oral repeated dose study No. 0338-2007 in which a decrease in food consumption and mild body weight loss were observed in one out of two animals given daily escalating doses of the test article in Phase I of the study (200 mg/kg/day x 4 days +400x4 + 800x4) and in one out of two animals given 800 mg/kg/day for 7 days.

The oral route and the daily administration schedule are those envisaged for the clinical setting.

6.4. Clinical and physical examinations

Survival and Moribundity Observations	At least once a day during the pre-test and recovery periods; at least twice a day during the treatment period
Clinical Signs	Every day, at least one session/day
Body Weights	Once pretest, on Day 1, then weekly
Food Consumption	Daily, qualitative, by visual inspection. Only reduced intake was reported.
Electrocardiographic Evaluation	Pretest and at the end of the treatment period (Day 24) and the recovery period (Day 37 or 38). In addition ECG recording was repeated during the pretest period for animal 2568 to further check rhythm.<br><br>ECGs were recorded from fully conscious manually restrained animals at least from I, II, III, aVR, aVL, aVF and, whenever possible, from precordial leads V1 (rV2), V3 (V2) and V5 (V4).<br>Morphologic review for changes in waveform morphology and for the presence of arrhythmias and interval measurement were conducted on all recordings. The following parameters were measured or computed: heart rate, RR interval, P-R interval, QRS complex width, Q-T interval and Q-T interval corrected for heart rate using Fridericia's formula ($QT/RR^{1/3}$). In addition P wave width, P, Q, R, S and T wave heights and ST segment deviation were measured, and mean electrical axis computed.
Ophthalmoscopy	Pretest and at the end of the treatment (Day 24) and the recovery period (Day 37 or 38).<br><br>The examination was performed using an indirect ophthalmoscope and a slit lamp when necessary. Mydriasis was induced by instilling one drop of 0.5% tropicamide (Visumidriatic) solution into each eye. The following were examined: conjunctiva, cornea, sclera, anterior chamber, iris, lens, vitreous body and fundus.

6.5. Clinical Pathology

6.5.1. Hematology

Collection Intervals	Pretest and at the end of the treatment and recovery periods.
Number of Animals	All animals
Collection Site	Jugular vessels
Anesthesia	None
Fasting Requirements	Overnight (about 16 hours)
Unscheduled Samples	None
Target Blood Volume	0.5 mL
Anticoagulant	8% EDTA solution

Hematology Parameters Evaluated

White Blood Cells	Reticulocyte Count (absolute and percent)
Red Blood Cells	Mean Corpuscular Volume reticulocyte (MCVr)
Hemoglobin	Mean Corpuscular Hemoglobin reticulocyte (CHr)
Hematocrit	Mean Corpuscular Hemoglobin Concentration reticulocytes (CHCMr)
Mean Corpuscular Volume	Platelets
Mean Corpuscular Hemoglobin	Mean Platelet Volume
Mean Corpuscular Hemoglobin Concentration	Platelet Distruibution Width
Red Cell Distribution Width	Platelet Hematocrit
Hemoglobin Distribution Width	Differential White Blood Cells (absolute and percent)

6.5.2. Coagulation

Collection Intervals	Pretest and at the end of the treatment and recovery periods.
Number of Animals	All
Collection Site	Jugular vessels
Anesthesia	None
Fasting Requirements	Overnight (about 16 hours) for scheduled samplings
Unscheduled Sampling	None
Target Blood Volume	About 2.5 mL
Anticoagulant	3.8% sodium citrate solution

Coagulation Parameters Evaluated

Activated Partial Thromboplastin Time	Prothrombin Time
Activated Partial Thromboplastin Time Ratio	Prothrombin Time Ratio
Fibrinogen	

6.5.3. Clinical Chemistry

Collection Intervals	Pretest and at the end of the treatment and recovery periods.
Number of Animals	All animals
Collection Site	Jugular vessels
Anesthesia	None
Fasting Requirements	Overnight (about 16 hours)
Unscheduled Samples	None
Target Blood Volume	1 mL in tube for serum separation
Anticoagulant	None

Clinical Chemistry Parameters Evaluated

Urea	Creatinine
Alanine Aminotransferase	Aspartate Aminotransferase
Alkaline Phosphatase	Total Bilirubin
Albumin	Total Proteins
Glucose	Globulin
Gamma-glutamyl transferase	Total Cholesterol
Triglycerides	Calcium
Phosphorus	Albumin/Globulin Ratio (calculated)
Sodium	Potassium
Chloride	

6.5.4. Urine Analysis

Collection Intervals	Pretest and at the end of the treatment and recovery periods.
Number of Animals	All
Method of Collection	Metabolic cages
Sample Requirements	1% Thimerosal (4ml) in each bottle, before collection
Fasting Requirements	Overnight (about 16 hours) for scheduled samplings
Unscheduled Samplings	None

Urine Parameters Evaluated

Total Volume	
Macroscopic Appearance (Color, Appearance)	Ketone Bodies
pH	Urobilinogen
White Blood Cells	Bilirubin
Nitrites	Hemoglobin/Red Blood Cells
Proteins	Specific Gravity
Glucose	

6.6. Post mortem examinations

6.6.1. Unscheduled Deaths

No animal died during the study.

6.6.2. Scheduled Necropsy, Tissue Collection, and Tissue Examination

	End of treatment necropsy	End of recovery necropsy
Sacrifice Schedule	Days 29-30	Day 43
Number of Animals	The first three/group in numerical order	Remaining animals
Method of Euthanasia	Sodium thiopental anesthesia and exsanguination from the femoral vein	
Fasting Requirements	Overnight for scheduled sacrifices	
Terminal Body Weight	All animals	
Macroscopic Examination	All animals	

Organs/tissues from animals surviving to scheduled sacrifice were collected, weighed and examined microscopically (E) according to the following table. Paired organs were weighed together. Relative organ weights were calculated on terminal body weight.

Organ/Tissue	All animals		Examined (mg/kg/day) All animals			
	Collected	Weighed	0	50	200	800
Aorta	X		E			E
Adrenal glands (both)	X	X	E			E
Bone marrow smear	X		E	E (m)	E (m)	E
Brain	X [a]	X	E			E
Diaphragm	X		E			E
Epididymides (both)	X		E			E
Esophagus	X		E			E
Eyes, optic nerve (both)	X		E			E
Heart	X	X	E			E
Duodenum	X		E			E
Jejunum	X		E			E
Ileum	X		E			E
Cecum	X		E			E
Colon	X		E			E
Gallbladder	X		E			E
Femur head	X		E			E
Kidneys (both)	X	X	E			E
Lacrimal glands	X		E			E
Liver	X [a]	X	E			E
Lungs	*X*		E			E
Lymph node, mandibular	X		E			E
Lymph node, mesenteric	X		E			E
Mammary gland	X		E	E (f)	E (f)	E
Ovaries	X	X	E	E	E	E
Pancreas	X		E			E
Pituitary	X		E			E
Prostate	X	X	E			E
Salivary glands (mandibular, parotids)	X		E			E
Sciatic nerve	X		E			E
Skeletal muscle	X		E			E
Skin	X		E			E
Spinal cord (cervical)	X		E			E
Spleen	X	X	E			E
Sternum	X		E			E
Stomach	X		E			E
Testes (both)	X	X	E			E
Thymus	X	X	E			E
Thyroid glands (with parathyroid glands)[b]	X		E			E
Tongue	X		E			E
Trachea	X		E			E
Urinary bladder	X		E			E
Uterus	X		E	E	E	E
Vagina	X		E	E	E	E
Lesions	X		E	E	E	E

Organ/Tissue	All animals		Examined (mg/kg/day) All animals			
	Collected	Weighed	0	50	200	800

Histological examination was performed on all tissues/organs from dogs of the control and high dose group killed terminally and at the end of the recovery period. For organs/tissues showing treatment-related changes the examination was extended to the other dose-groups as necessary to establish a no-effect level.
a Samples of liver and brain were collected and frozen in liquid nitrogen and kept at -80°C as required by the Sponsor
b Parathyroid glands examined microscopically if included in the section of thyroid glands

(f) females only
(m) males only

Fixatives:
Eyes: Davidson's solution
Bone Marrow Smears: Methanol-ether
All Other Tissues: 10% neutral buffered formalin

6.6.3. Tissue Preparation

Histological sections of all tissues listed in Section 6.6.2 were trimmed, embedded, sectioned, and stained with hematoxylin and erythrosine. Bone marrow smears were prepared and stained with May Grünwald-Giemsa.

All organs/tissues were kept for any further test necessary.

6.6.4. Peer review

A pathology peer review was conducted by Anna Maria Giusti. A report was produced and filed with the study raw-data.

6.7. Systemic Exposure

The toxicokinetics of fexinidazole and its metabolites M1 (sulfoxide) and M2 (sulfone) were evaluated in accordance with the collection schedule and procedures tabulated below. The bioanalysis were conducted by DMPK & ART – Bioanalysis/Analytical Control, Nerviano.

Dose Levels (Groups)	All groups,
Collection Intervals	Days 1, 14 and 28
Collection Time Points	Days 1 and 14: Predose, 30 minutes, 1, 2, 4, 8 and 24h after dosing (all animals).<br>Day 28: Predose, 30 minutes, 1, 2, 4, 8, 24 (all animals), 48 and 72h after dosing (recovery animals)
Animals/Time Point	See above
Anesthesia	Not required
Collection Volume per Sample	About 1 mL of blood/point.
Collection Site	Peripheral veins (alternate sites may be used if necessary)
Sample Requirements	Blood was put in heparinized plastic tubes kept on a ice-water bath, then centrifuged (10 min, 1200g, +4°C). Two aliquots of about 200 μL each of plasma were stored in a freezer at –80°C until analysis.

After blood collection, frozen plasma specimens were transferred in plastic boxes to DMPK & ART, Nerviano Medical Sciences. The samples were loaded according to Watson 6.4.0.04 (Thermo Fisher Scientific Whaltham, MA, USA).

Plasma samples were analyzed for the quantitation of fexinidazole using a validated LC-MS-MS method by Bioanalysis/Analytical Control, DMPK & ART.
Toxicokinetic calculations were carried out by Pharmacokinetics, DMPK & ART.
The toxicokinetic report is appended to the present report as Appendix 13.
Details of the analytical method and of calculation methods are included in the toxicokinetic report.

7. DATA ACQUISITION

Clinical observations, body weights**,** food consumptions, ECG and opthalmoscopic examination data, organ weights, gross necropsy observations, histopathologic findings and dose administration documentation were entered directly into the Xybion Path/Tox System or were recorded on appropriate paper forms, and, if appropriate, later entered into the Xybion Path/Tox System. Clinical pathology data were processed according to Clinical Pathology Laboratories procedures.

The limits of a scheduled period were fixed by the “grace days” reported for scheduled determinations.

8. STATISTICAL ANALYSIS

8.1. Variables

Body weights, clinical chemistry, hematology, ECG data, absolute organ weights, urinalysis (volume and specific gravity only) were statistically analysed.

8.2. Methods

Dunnett’s test, included in a customized Xybion package, was used.

9. ARCHIVING

The original protocol, the protocol amendment, all raw data, supporting documents, and specimens produced at the Test Facility, and the final report with original signatures were filed in the Archives of Accelera, Nerviano Medical Sciences S.r.l., Nerviano (Italy) for the period of time agreed with the Sponsor (at least 3years) after which the Sponsor will be contacted for instructions regarding dispatch or disposal of the material.

A copy of the protocol, the report with original signatures, a reserve sample and all relevant original documentation of the test item were filed by the Sponsor.

10. STUDY DEVIATIONS

1) Report No. 0293-2007-R, mentioned in Paragraph 6.1.4.1 of the protocol. (Homogeneity and Concentration) concerning validation of the analysis method for quantification of fexinidazole, was finalized after start of the study.

2) Only plasma samples taken at 0 and 2h from control animals were analyzed for the presence of Fexinidazole and its metabolites, at variance with Amendment 1 (0505-2007-PA1).

11. STUDY PERSONNEL

12. RESULTS

12.1. Clinical and Physical Examination

12.1.1. Mortality

No mortality was observed during the study.

12.1.2. Clinical Signs

(Table 1, Appendix 2)

No treatment-related clinical signs were observed during the study.

The gastrointestinal disturbances observed (soft stools and/or diarrhea) were considered unrelated to the treatment since they were sporadic, present also during the pretest period and occurring with a similar incidence and severity in control and treated animals.

12.1.3. Body Weight

(Table 2 , Appendix 3, Figure 16.1)

Slight to moderate, progressive body weight loss was observed in one male given 200 mg/kg/day (No. 2523, -10% on Day 28 vs Day 1) and, in the high-dose group, in two males (Nos 2517 and 2530; -10 to –14%) and four females (all except No. 2569; -8 to -15%). A trend towards recovery was observed after the end of the treatment period in female No. 2571 only. Changes in body weight correlated with the decrease in food consumption (see below) in females only.

12.1.4. Food consumption

(Table 1, Appendix 2)

No significant changes in food intake were observed in treated males at any dose.
A slight to moderate reduction in food intake was repeatedly observed from Day 2 on, throughout the treatment period, in the same four females given 800 mg/kg/day that showed body weight loss. Female No. 2569 showed only sporadic episodes of slight reduction in food intake. The two females chosen for recovery showed normal food intake after the treatment was discontinued.

12.1.5. Electrocardiographic examination

(Table 3, Appendix 4)

No abnormalities in rhythm and morphology were recorded in any animal.

No meaningful abnormal values were observed in any measured or computed parameters including repolarization parameters, namely QT or QTc interval corrected for heart rate using the Fridericia's formula ($QT/RR^{1/3}$).

The changes observed when the various groups were compared with the control group were not considered to be toxicologically meaningful despite the statistical significance of some differencies.

The variations observed in comparison with the pretest values (see table below for mean variations of main parameters) showed no meaningful changes or changes similar to those observed in control animals.

Main ECG parameters - Differences in comparison with pretest – Mean values

		Males				Females			
		**control**	**grp2**	**grp3**	**grp4**	**control**	**grp2**	**grp3**	**grp4**
**Heart rate**	**pretest**	**93**	**82**	**81**	**95**	**95**	**116**	**93**	**102**
bpm	Day 24	2	20	16	5	4	-8	19	8
	Day 38	-10			-4	6			0
**P-R interval**	**pretest**	**87**	**89**	**97**	**86**	**88**	**83**	**92**	**87**
ms	Day 24	-6	-7	-7	0	-5	6	-5	-1
	Day 38	0			15	14			3

**QRS complex duration**	**pretest**	**47**	**46**	**55**	**44**	**46**	**51**	**49**	**50**
	Day 24	0	-1	-2	0	2	-3	4	3
ms	Day 38	4			7	5			-1
**Q-T interval**	**pretest**	**179**	**186**	**190**	**179**	**185**	**180**	**183**	**177**
ms	Day 24	10	1	6	18	1	4	9	15
	Day 38	20			24	9			27
**Q-Tc interval**	**pretest**	**208**	**204**	**209**	**207**	**214**	**224**	**210**	**211**
($QT/RR^{1/3}$)	Day 24	11	15	18	26	4	0	25	20
ms	Day 38	14			23	14			30

bpm = beats per minute; ms = milliseconds. Pretest data are reported as absolute values.

Other values or notes reported in the tables or appendices were deemed to be spontaneous because one or more of the following conditions apply: they were present at the pre-test examinations, without any dose and/or time relationship and, in our experience, are those commonly occurring under the experimental conditions or are well-known normal features of the dog heart rhythm such as respiratory sinus arrhythmia.

12.1.6. Ophthalmoscopic examination

(Table 4, Appendix 5)

No treatment-related changes were noted during the study.

The only alteration recorded (female dog No. 2575, small areas of corneal opacity in the right eye at the end of the treatment and recovery period) was unilateral and involved a control animal.

12.2. Clinical pathology

12.2.1. Hematology

(Table 5, Appendix 6)

No clear treatment-related changes were recorded during the study.
On Day 28 a minimal to slight (about 25%) variable decrease in lymphocytes was recorded in males dosed at 200 and 800 mg/kg/day. The same parameter showed a minimal (9 to 16%) variable decrease in all dosed females without dose-relationship. Recovery occurred on Day 42 in high-dose animals. In other groups recovery could not be ascertained.
Neutrophils were slightly (about 20%) decreased in males dosed at 200 mg/kg/day. Recovery could not be ascertained.

12.2.2. Coagulation

(Table 6, Appendix 7)

No treatment-related changes were recorded during the study.

12.2.3. Clinical chemistry

(Table 7, Appendix 8)

No clear treatment-related changes were recorded during the study.

On Day 28, a minimal (about 2-fold) increase in alkaline phosphatase was observed in high-dose male No. 2525. Recovery could not be ascertained.
A minimal (about 1.5-fold) increase in total cholesterol was recorded in high-dose female No. 2564 at the end of the treatment period. Recovery could not be ascertained.
In male No. 2528 given the high dose gamma-glutamyl transferase was minimally (about 1.4-fold) increased starting from Day 28 up to Day 42.

12.2.4. Urinalysis

(Tables 8 and 9, Appendix 9)

No treatment-related changes were recorded during the study.

12.3. Post-mortem examinations

12.3.1. Organ Weights

(Tables 10 and 11, Appendices 10 and 11)
End of treatment (Days 29-30)
The increases in mean absolute and relative weights of the ovaries observed in treated animals, without dose-relationship, are considered of doubtful toxicological significance and are further discussed in the histopathology section.

Other variations in mean weights, sometimes noted in treated animals, reflect the remarkable individual variability observed also in controls.

Final Phase Sacrifice (Day 43)
The changes observed in mean absolute and relative organ weights were not considered toxicologically significant due to the low number of animals available for evaluation and to individual variability.

12.3.2. Gross Pathology

(Table 12, Appendix 12)
End of treatment (Days 29-30)
Fairly good general condition, clear liquid content in the peritoneal cavity or small thymus were occasionally observed in treated animals as well as in controls.
A generalized increase in size of lymph nodes was noted in male No. 2523, receiving 200 mg/kg/day of the test compound.

Final Phase Sacrifice (Day 43)

Fairly good general condition was noted in one male treated with 800 mg/kg/day (No. 2530). Small prostate was noted in the same animal as well as in one control male (No. 2533).

12.3.3. Histopathology

(Table 13, Appendix 12)

End of treatment (Days 29-30)

Changes considered as indirectly related to treatment with the test compound were observed in the hematopoietic system.

Moderately reduced cellularity of the bone marrow and severe involution of the thymus were noted in a high dose male (No. 2517), accompanied by atrophy of the adipose tissue with grades ranging from slight to moderate in the sternal and femoral marrow spaces and in the fat deposits adjacent to the renal pelvis. Minimally reduced cellularity was also noted in the bone marrow of one male treated with 200 mg/kg/day (No. 2523). The changes observed in the bone marrow in both animals, and in the thymus and adipose tissue in the high-dose male were interpreted as a consequence of body weight loss.

Final Phase Sacrifice (Day 43)

No clear treatment related changes were noted in the hematopoietic system of high-dose animals at the end of the recovery period. The increased severity of involution noted in the thymus of both sexes compared with controls was considered related to body weight loss.

Additional changes of doubtful toxicological significance were noted as follows.

At the end of treatment period, corpora lutea were observed in all females at 200 and 800 mg/kg/day dose levels and in two out of three females treated with 50 mg/kg/day (Table 14). The situation of the stage of estrus of female animals is summarized in Table 15. The stage of estrus was assessed on the base of the comprehensive microscopic appearance of ovaries, uterus and vagina [5]. All three control animals sacrificed at the end of the treatment were immature. On the contrary, the majority of the treated animals sacrificed at the same timepoint were in diestrus, while one low dose was in estrus and one was in metestrus. The significance of this finding is discussed later (see 13.Discussion).

12.4. Systemic Exposure Evaluation

(Appendix 13)

Day 1

Mean ±SD systemic exposure to Fexinidazole is reported in the following table

Dose	Male			Female		
mg/kg	Cmax ng/mL	tmax hour	AUC0-t(last) ng·hour/mL	Cmax ng/mL	tmax hour	AUC0-t(last) ng·hour/mL
50 (n=3)	31.2±12.4	0.5±0	140±147	42.4±10.6	1±0.87	237±105
200 (n=3)	54.9±10.8	1±0.87	419±61.4	84.1±36.7	1±0	454±119
800 (n=5)	100±21.2	1.1±0.55	776±182	184±75.6	1.2±0.45	895±437

At each dose, no relevant gender difference was observed in terms of C_{max} and $AUC_{0-t(last)}$ values. The maximal plasma concentrations of fexinidazole were promptly achieved, on average at 1 hour post dosing. $AUC_{0-t(last)}$ values of fexinidazole increased with the dose

Mean ±SD systemic exposure to the sulfone metabolite is reported in the following table

Dose	Male			Female		
mg/kg	Cmax µg/mL	tmax Hour	AUC0-t(last) µg·hour/mL	Cmax µg/mL	tmax hour	AUC0-t(last) µg·hour/mL
50 (n=3)	7.17±1.74	8±0	126±28.1	10±1.6	6.67±2.31	170±37.8
200 (n=3)	17.2±1.69	13.3±9.24	338±50.8	18.1±3.42	12±10.6	358±85.7
800 (n=5)	38.6±2.83	17.6±8.76	705±94.9	33.6±10.9	11.2±7.16	614±216

At each dose, the levels of the metabolite were similar in males and females. T_{max} values of the metabolite were achieved later than for the parent compound. The systemic exposure to the metabolite increased with the dose.
The systemic exposure to the sulfone metabolite was definitely higher than that to the parent compound.

Mean ±SD systemic exposure to the sulfoxide metabolite is reported in the following table

Dose	Male			Female		
mg/kg	Cmax µg/mL	tmax hour	AUC0-t(last) µg·hour/mL	Cmax µg/mL	tmax hour	AUC0-t(last) µg·hour/mL
50 (n=3)	3.55±1.3	1±0	19.2±5.82	3.97±0.74	1.33±0.58	20±6.81
200 (n=3)	7.55±1.03	1.67±0.58	50.7±8.61	8.7±3.95	0.83±0.29	52.1±20.6
800 (n=5)	13.4±3.22	1.5±0.71	104±11.3	15.6±4.64	1.6±0.55	121±44.4

At each dose, the levels of the metabolite were similar in males and females. The maximal plasma concentrations of the sulfoxide metabolite were rapidly achieved, on average 1 - 2 hours post dosing.
The systemic exposure to the metabolite increased with the dose (Figures 26 - 27).
The systemic exposure to the metabolite was definitely higher than that to the parent compound.
<u>Repeated dosing</u>
Day 14 and Day 28 mean ±SD systemic exposure to fexinidazole is reported in the following table

Dose	Male			Female		
mg/kg/day	Cmax ng/mL	tmax hour	AUC0-t(last) ng·hour/mL	Cmax ng/mL	tmax hour	AUC0-t(last) ng·hour/mL
	Day 14					
50 (n=3)	26.5±13	0.5±0	52.1±29.4	41.9±2.2	1.67±0.58	246±117
200 (n=3)	78.1±23.5	1±0.87	452±41.1	77.7±50.8	2±0	443±217
800 (n=5)	128±56.9	1±0	929±268	152±44	1.4±0.55	1170±309
	Day 28					
50 (n=3)	20.3±7.89	1±0	124±67	36.3±15.1	1±0	87.1±38.1
200 (n=3)	57.9±12.5	1±0	395±15.8	73±12	1±0	377±83.5
800 (n=5)	86.2±38.5	1.2±0.4	736±141	101±47.1	1±0	956±378

At each dose, the levels of fexinidazole were similar in both genders after administration on Day 14 and Day 28. The maximal plasma concentrations of fexinidazole were achieved, on average, 1 - 2 hours post dosing. The systemic exposure to fexinidazole increased with the dose (Figures 22 - 23). C_{max} and $AUC_{0\text{-}t(last)}$ accumulation ratios were about 1.

Day 14 and Day 28 mean ±SD systemic exposure to the sulfone metabolite is reported in the following table

Dose	Male			Female		
mg/kg/day	C_{max} µg/mL	t_{max} hour	$AUC_{0\text{-}t(last)}$ µg·hour/mL	C_{max} µg/mL	t_{max} hour	$AUC_{0\text{-}t(last)}$ µg·hour/mL
	Day 14					
50 (n=3)	5.57±2.19	4±0	78±35.3	9.98±1.25	6.67±2.31	173±26.9
200 (n=3)	22±5.59	5.33±2.31	387±49.5	21.5±2.42	5.33±2.31	381±61.2
800 (n=5)	34.6±7.42	5.6±2.19	640±163	38.6±4.73	8±0	667±67.8
	Day 28					
50 (n=3)	7.11±1.21	8±0	121±19.1	6.79±0.56	3.33±1.15	107±22.5
200 (n=3)	14±3.31	4±4	258±48	15.4±2.66	11.7±10.1	277±29.9
800 (n=5)	20.8±4.14	12.4±9.81	388±68.2(1)<br>601±29(2)	26±7.7	7.2±1.79	477±145(1)<br>693±351(2)
(1) $AUC_{0\text{-}24}$; (2) n=2						

At each dose, the levels of the metabolite were similar in both genders. T_{max} values of the metabolite were achieved later than for the parent compound. On Day 28 after 800 mg/kg/day, mean ±SD apparent terminal half-lives were 6.7 ±0.7 and 7.8 ±0.7 (n=2) hours in males and females, respectively. After the three doses, systemic exposure to the metabolite increased with the dose (Figures 24 - 25). Accumulation ratios, in terms of both C_{max} and $AUC_{0\text{-}t(last)}$, were about 1.
The systemic exposure to the metabolite was remarkably higher than that to the parent compound.

Day 14 and Day 28 mean ±SD systemic exposure to the sulfoxide metabolite is reported in the following table

Dose	Male			Female		
mg/kg/day	C_{max} µg/mL	t_{max} hour	$AUC_{0\text{-}t(last)}$ µg·hour/mL	C_{max} µg/mL	t_{max} hour	$AUC_{0\text{-}t(last)}$ µg·hour/mL
	Day 14					
50 (n=3)	2.24±0.45	1±0	8.51±2.53	3.73±0.73	1.67±0.58	18.8±2.06
200 (n=3)	8.96±2.53	2±0	56.1±9.96	9.02±3.69	2±0	57.2±24.3
800 (n=5)	12.5±2.85	1.2±0.45	113±39.9	14.8±5.1	2±1.22	144±37.1
	Day 28					
50 (n=3)	1.83±0.57	1.67±0.58	14.2±2.72	2.72±0.73	1±0	9.88±2.72
200 (n=3)	5.76±1.17	1.33±0.58	42.2±6.46	5.43±0.34	1±0	33.8±1.65
800 (n=5)	9.35±3.55	1.6±0.55	74.3±14.4(1)<br>121±33(2)	9.48±3.38	1.4±0.55	89±45.2
(1) $AUC_{0\text{-}24}$; (2) n=2						

At each dose, the levels of the metabolite were similar in both genders. The maximal plasma concentrations of the sulfoxide metabolite were promptly achieved, on average, 1 - 2 hours

post dosing. On Day 28 after 800 mg/kg/day, mean ±SD apparent terminal half-lives were 9.4 ±5.4 (n=2) hours in males. After the three doses, the systemic exposure to the metabolite increased with the dose (Figures 26 - 27). Accumulation ratios, in terms of both C_{max} and $AUC_{0-t(last)}$, were about 1.
The systemic exposure to the metabolite was remarkably higher than that to the parent compound.

13. DISCUSSION

Fexinidazole was given daily for twenty-eight days to five (control and high dose) or three (low and mid dose) beagle dogs/sex/dose at the doses of 0 (control group), 50, 200 and 800 mg/kg/day. The compound was administered orally, by gavage, as a suspension in 5% Tween 80 / 0.5% Methocel in a volume of 10 mL/kg/day. The control group received the vehicle alone (5% Tween 80 in 0.5% Methocel).
No drug-related changes were observed at electrocardiographic and ophthalmoscopic examination.

No mortality and no drug-related clinical signs were observed.
Slight to moderate, progressive body weight loss was observed in a few males and in the majority of females given 800 mg/kg/day. A slight to moderate reduction in food intake was seen during the treatment period in the same females given 800 mg/kg/day that showed body weight loss, with normalization during the recovery period.
Similar effects on body weight and food consumption were observed in a previous repeated dose toxicity study in the dog with this same compound [2], [3].

A minimal to slight decrease in lymphocytes was observed mainly at the high dose in both sexes. Other changes observed at hematology and clinical chemistry were not dose-related or present only in single instances and thus considered of no toxicological relevance.
No clear cut treatment-related histological changes were identified in animals treated with the test compound when compared with controls.
The minimally to moderately reduced cellularity in the bone marrow observed in one male treated with 200 mg/kg/day and in one high dose male (No. 2517), and the severe involution of the thymus observed in the high dose male were considered as changes secondary to body weight loss, which was more prominent in this last male with respect to other males given the same dose. This interpretation would be further supported by the occurrence of atrophy of the adipose tissue, noted in the animal treated with the high dose, both in the marrow spaces of the sternum and femur [4] and in the fat deposits adjacent to the renal pelvis.

At the end of treatment period, corpora lutea were observed in all females at 200 and 800 mg/kg/day dose levels and in two out of three females treated with 50 mg/kg/day, while they were not present in the ovaries of control females, correlating with the increase in absolute and relative weight of the ovaries and with the differences in the stage of estrus noted in treated animals compared to controls.

The odd distribution of the stage of estrus within the groups, in animals with a quite homogeneous age at the time of necropsy (about 9.5 month), could suggest an effect of the test compound in terms of induction of estrus in treated animals, independently from the dose administered. Nevertheless, the two control animals sacrificed after recovery were both in diestrus and thus already cycling (i.e., mature) two weeks before, at the time of the end-of-treatment sacrifice, thus re-equilibrating the distribution of estrus stage with the treated groups at that time.
On the base of these considerations, the distribution observed in animals at the end of treatment is considered most likely due to the incidental occurrence of immature animals only in the control group and there is not a clear influence of the test compound on the stage of estrus of the animals in the study.

After single and repeated administrations at the three dose levels, no relevant gender difference in the systemic exposure to fexinidazole was observed. AUCs of fexinidazole increased less than expected assuming dose proportionality in the dose range investigated. No accumulation of fexinidazole was observed.
Fexinidazole was extensively metabolized to the sulfone and sulfoxide derivatives after both single and repeated administration. No accumulation of either metabolite was observed.

14. CONCLUSIONS

Fexinidazole, given orally to beagle dogs at the doses of 50, 200 and 800 mg/kg/day for 28 consecutive days, was well tolerated.
Slight to moderate body weight loss and reduction in food intake was observed at the dose of 800 mg/kg/day during the treatment period.
A minimal to slight decrease in lymphocytes was seen at the high dose.
Fexinidazole was extensively metabolized to the sulfone and sulfoxide derivatives both after single and repeated administration.
In the condition applied in the present study, the dose of 200 mg/kg/day can be considered as the NOEL.

15. REFERENCES

1. Fexinidazole: Validation of an Analytical Method for the Assay of Fexinidazole in 0.5% methocel containing 5% v/v of Tween 80 by HPLC-UV. Nerviano Medical Sciences, Study No. 0293-2007, report in progress.

2. Fexinidazole: Seven-Day Oral Toxicity Study in the Dog, (DRF - phased study) Nerviano Medical Sciences Study Report No. 0338-2007-R, March 18, 2008.

3. Tolerance testing of HOE 239 (= S 75 1239) after repeated oral administration to beagle dogs: 90-day tolerance study, Hoechst Document No. 1977.1239, February 4, 1977

4. Travlos GS (2006). Histopathology of Bone Marrow. Toxicol Pathol 34: 566-598.

5. Rehm S, Stanislaus DJ, Williams AM (2007). Estrous cycle-dependent histology and review of sex steroid receptor expression in dog reproductive tissues and mammary gland and associated hormone levels. Birth Defects Res B Dev Reprod Toxicol 80: 233-245.

16. FIGURES

16.1. Body weight

(during treatment period)

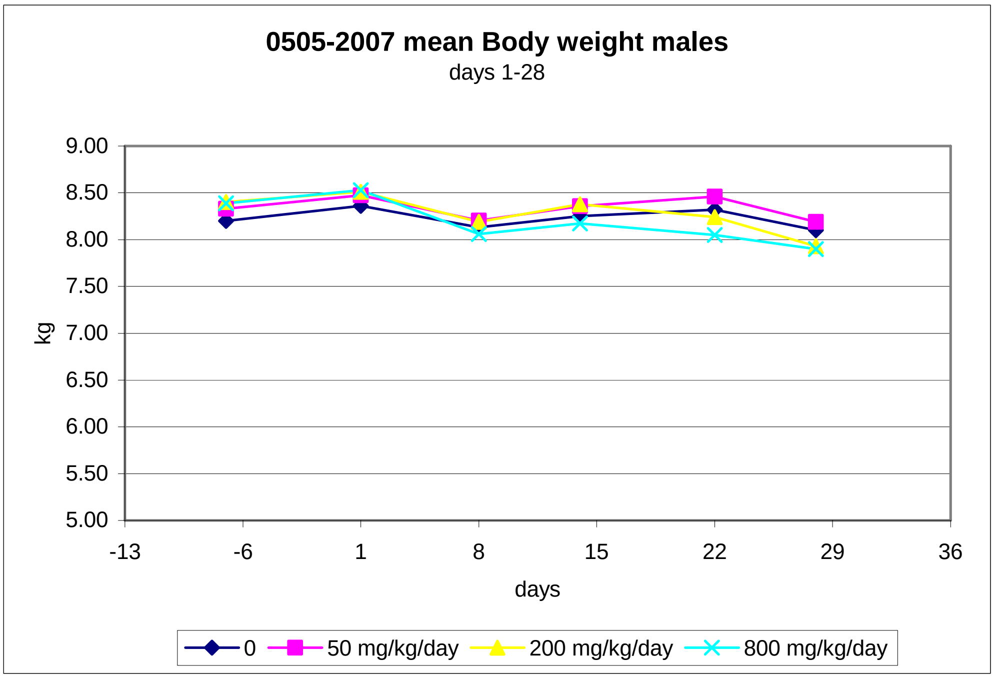

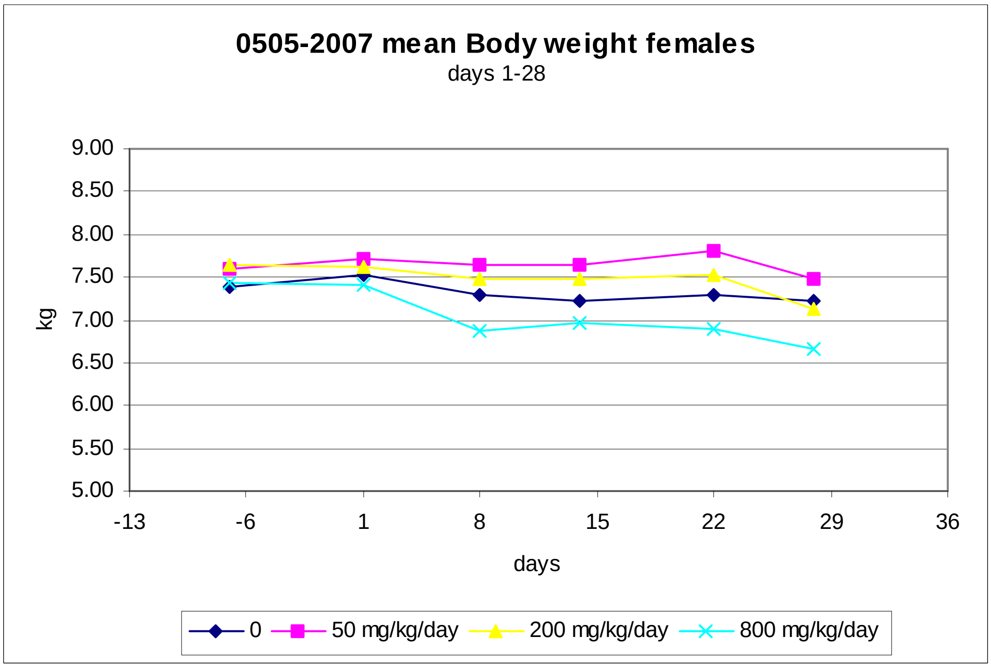

TABLES

Table 1 Clinical Signs

Table 1
Group Incidence of Clinical Signs

Fexinidazole | Study Number: 0505-2007

	Males							
Study Days -8--1 Group Number	1		2		3		4	
Number of Animals+	5		3		3		5	
	a	(b)	a	(b)	a	(b)	a	(b)
Normal								
NORMAL/NO SIGNIFICANT SIGNS	5	(6.6)	3	(7.3)	3	(6.7)	5	(7.4)
GASTRO-INTESTINAL FUNCTIONS								
DIARRHEA	1	(2.0)	0	(0.0)	0	(0.0)	0	(0.0)
SOFT STOOL	4	(1.3)	1	(2.0)	3	(1.3)	2	(1.5)

Group 1:Vehicle Group 2:50 mg/kg/day Group 3:200 mg/kg/day Group 4:800 mg/kg/day

Key: + = Number of animals alive at start of interval
a = Number of animals affected
(b) = Mean number of animal days that the group displayed the sign

Table 1
Group Incidence of Clinical Signs

Fexinidazole Study Number: 0505-2007

	Females							
Study Days -8--1 Group Number	1		2		3		4	
Number of Animals+	5		3		3		5	
	a	(b)	a	(b)	a	(b)	a	(b)
Normal								
NORMAL/NO SIGNIFICANT SIGNS	5	(7.8)	3	(7.3)	3	(6.7)	5	(7.6)
GASTRO-INTESTINAL FUNCTIONS								
SOFT STOOL	1	(1.0)	1	(2.0)	2	(2.0)	1	(1.0)
FOOD/WATER INTAKE								
REDUCED FOOD INTAKE	0	(0.0)	0	(0.0)	0	(0.0)	1	(1.0)

Group 1:Vehicle Group 2:50 mg/kg/day Group 3:200 mg/kg/day Group 4:800 mg/kg/day

Key: + = Number of animals alive at start of interval
a = Number of animals affected
(b) = Mean number of animal days that the group displayed the sign

Table 1
Group Incidence of Clinical Signs

Fexinidazole | Study Number: 0505-2007

Study Days 1-30		Males							
	Group Number	1		2		3		4	
	Number of Animals+	5		3		3		5	
		a	(b)	a	(b)	a	(b)	a	(b)
Normal									
NORMAL/NO SIGNIFICANT SIGNS		5	(29.6)	3	(29.0)	3	(29.3)	5	(29.6)
GASTRO-INTESTINAL FUNCTIONS									
DIARRHEA		3	(1.7)	1	(5.0)	0	(0.0)	4	(1.3)
SOFT STOOL		5	(6.4)	2	(6.5)	1	(3.0)	5	(4.0)
FOOD/WATER INTAKE									
REDUCED FOOD INTAKE		2	(1.0)	1	(6.0)	1	(3.0)	1	(1.0)

Group 1:Vehicle   Group 2:50 mg/kg/day   Group 3:200 mg/kg/day   Group 4:800 mg/kg/day

Key: + = Number of animals alive at start of interval
a = Number of animals affected
(b) = Mean number of animal days that the group displayed the sign

Table 1
Group Incidence of Clinical Signs

Fexinidazole | Study Number: 0505-2007

	Females							
Study Days 1-30 Group Number	1		2		3		4	
Number of Animals+	5		3		3		5	
	a	(b)	a	(b)	a	(b)	a	(b)
Normal								
NORMAL/NO SIGNIFICANT SIGNS	5	(29.6)	3	(29.3)	3	(29.3)	5	(29.4)
GASTRO-INTESTINAL FUNCTIONS								
DIARRHEA	3	(2.3)	0	(0.0)	1	(4.0)	2	(1.0)
EMESIS OF FOOD	0	(0.0)	0	(0.0)	0	(0.0)	1	(1.0)
SOFT STOOL	5	(2.6)	2	(2.0)	2	(5.0)	3	(1.0)
FOOD/WATER INTAKE								
REDUCED FOOD INTAKE	1	(1.0)	1	(1.0)	2	(2.5)	5	(12.8)

Group 1:Vehicle   Group 2:50 mg/kg/day   Group 3:200 mg/kg/day   Group 4:800 mg/kg/day

Key: + = Number of animals alive at start of interval
a = Number of animals affected
(b) = Mean number of animal days that the group displayed the sign

Table 1
Group Incidence of Clinical Signs

Fexinidazole Study Number: 0505-2007

	Males							
Study Days 31-43 Group Number	1		2		3		4	
Number of Animals+	5		3		3		5	
	a	(b)	a	(b)	a	(b)	a	(b)
Normal								
NORMAL/NO SIGNIFICANT SIGNS	2	(12.0)	0	(0.0)	0	(0.0)	2	(12.5)
GASTRO-INTESTINAL FUNCTIONS								
DIARRHEA	0	(0.0)	0	(0.0)	0	(0.0)	1	(1.0)
SOFT STOOL	2	(1.0)	0	(0.0)	0	(0.0)	0	(0.0)

Group 1:Vehicle Group 2:50 mg/kg/day Group 3:200 mg/kg/day Group 4:800 mg/kg/day

Key: + = Number of animals alive at start of interval
a = Number of animals affected
(b) = Mean number of animal days that the group displayed the sign

Table 1
Group Incidence of Clinical Signs

Fexinidazole Study Number: 0505-2007

	Females							
Study Days 31-43 Group Number	1		2		3		4	
Number of Animals+	5		3		3		5	
	a	(b)	a	(b)	a	(b)	a	(b)
Normal								
NORMAL/NO SIGNIFICANT SIGNS	2	(12.0)	0	(0.0)	0	(0.0)	2	(12.0)
GASTRO-INTESTINAL FUNCTIONS								
SOFT STOOL	2	(1.0)	0	(0.0)	0	(0.0)	2	(1.0)

Group 1:Vehicle Group 2:50 mg/kg/day Group 3:200 mg/kg/day Group 4:800 mg/kg/day

Key: + = Number of animals alive at start of interval
a = Number of animals affected
(b) = Mean number of animal days that the group displayed the sign

Table 2 Body Weights

Table 2
Body Weights (kg)

Fexinidazole Study Number: 0505-2007

Group Number	Dose Level	Study Day	-8"	1#	8	14	22	28	35	40
			M a l e s							
1	Vehicle	N	5	5	5	5	5	5	2	2
		Mean	8.20	8.36	8.14	8.25	8.32	8.10	7.58	7.71
		Sdev	0.444	0.436	0.410	0.423	0.472	0.411	0.240	0.153
2	50 mg/kg/day	N	3	3	3	3	3	3	0	0
		Mean	8.33	8.47	8.20	8.36	8.46	8.19	-	-
		Sdev	1.160	1.163	1.165	1.155	1.271	1.081	-	-
3	200 mg/kg/day	N	3	3	3	3	3	3	0	0
		Mean	8.40	8.51	8.19	8.37	8.24	7.93	-	-
		Sdev	0.691	0.482	0.642	0.479	0.531	0.351	-	-
4	800 mg/kg/day	N	5	5	5	5	5	5	2	2
		Mean	8.39	8.53	8.06	8.17	8.05	7.90	7.55	7.75
		Sdev	0.734	0.954	0.993	1.049	1.010	1.117	1.131	1.039

Note: " = Pretest phase; # = Test period

CONFIDENTIAL

Table 2
Body Weights (kg)

Fexinidazole | Study Number: 0505-2007

Group Number	Dose Level	Study Day	-8"	1#	8	14	22	28	35	40
			Females							
1	Vehicle	N	5	5	5	5	5	5	2	2
		Mean	7.37	7.52	7.29	7.22	7.29	7.23	8.06	8.21
		Sdev	0.471	0.592	0.621	0.737	0.783	1.020	0.445	0.426
2	50 mg/kg/day	N	3	3	3	3	3	3	0	0
		Mean	7.60	7.70	7.65	7.63	7.80	7.49	-	-
		Sdev	0.674	0.872	0.762	0.847	0.784	0.803	-	-
3	200 mg/kg/day	N	3	3	3	3	3	3	0	0
		Mean	7.65	7.63	7.48	7.48	7.52	7.13	-	-
		Sdev	0.960	0.807	0.808	0.862	0.770	0.809	-	-
4	800 mg/kg/day	N	5	5	5	5	5	5	2	2
		Mean	7.42	7.41	6.87	6.96	6.90	6.65	6.81	6.95
		Sdev	0.391	0.546	0.670	0.671	0.677	0.659	0.601	0.592

Note: " = Pretest phase; # = Test period

Table 3 ECG Examinations

Table 3
Incidence of ECG Rhythm and Morphology Findings

Fexinidazole Study Number: 0505-2007

Study Day	Group:	Males 1	2	3	4
-5					
ECG Notes	/No ECG abnormalities detected	1	0	0	2
	/Respiratory Sinus Arrhythmia	4	3	3	3
24					
ECG Notes	/No ECG abnormalities detected	1	3	1	2
	/Respiratory Sinus Arrhythmia	4	0	2	3
38					
ECG Notes	/No ECG abnormalities detected	0	0	0	1
	/Respiratory Sinus Arrhythmia	2	0	0	1

Group 1:Vehicle Group 2:50 mg/kg/day Group 3:200 mg/kg/day Group 4:800 mg/kg/day

Table 3

Incidence of ECG Rhythm and Morphology Findings

Fexinidazole | Study Number: 0505-2007

Study Day	Group:	Females 1	2	3	4
-5					
ECG Notes	/No ECG abnormalities detected	2	2	0	3
	/Respiratory Sinus Arrhythmia	3	1	3	2
24					
ECG Notes	/No ECG abnormalities detected	1	2	0	3
	/Respiratory Sinus Arrhythmia	4	1	3	2
38					
ECG Notes	/No ECG abnormalities detected	2	0	0	1
	/Respiratory Sinus Arrhythmia	0	0	0	1

Group 1:Vehicle   Group 2:50 mg/kg/day   Group 3:200 mg/kg/day   Group 4:800 mg/kg/day

Table 3
Day -5 ECG Examinations
Pretest phase

Session 1 (Scheduled)
Fexinidazole

Study Number: 0505-2007

		Males				Females			
Parameter	Group Number:	1	2	3	4	1	2	3	4
Heart rate	N	5	3	3	5	5	3	3	5
bpm	Mean	93.	82.	81.	95.	95.	116.	93.	102.
	Sdev	6.2	28.0	19.8	25.6	21.5	16.2	19.7	20.5
R-R interval	N	5	3	3	5	5	3	3	5
ms	Mean	642.	780.	769.	672.	664.	523.	658.	599.
	Sdev	41.8	221.6	184.0	204.8	176.5	70.9	124.6	102.4
P wave duration	N	5	3	3	5	5	3	3	5
ms	Mean	38.	37.	37.	39.	41.	37.	38.	36.+
	Sdev	1.8	1.5	4.4	3.0	3.4	2.1	1.7	2.3
P-R interval	N	5	3	3	5	5	3	3	5
ms	Mean	87.	89.	97.	86.	88.	83.	92.	87.
	Sdev	5.8	9.3	2.1	8.5	12.8	2.5	5.8	11.3
QRS complex duration	N	5	3	3	5	5	3	3	5
ms	Mean	47.	46.	55.	44.	46.	51.	49.	50.
	Sdev	11.7	2.6	9.0	1.3	7.0	7.2	5.5	10.6
Q-T interval	N	5	3	3	5	5	3	3	5
ms	Mean	179.	186.	190.	179.	185.	180.	183.	177.
	Sdev	1.8	11.5	13.6	6.8	6.5	1.2	10.1	6.4
Q-T interval (Fridericia)	N	5	3	3	5	5	3	3	5
ms	Mean	208.	204.	209.	207.	214.	224.	210.	211.
	Sdev	5.7	8.6	10.3	19.9	14.3	10.6	2.7	8.7

Group 1:Vehicle Group 2:50 mg/kg/day Group 3:200 mg/kg/day Group 4:800 mg/kg/day

Group means without footnotes were not statistically different from the control at p=0.05 or p=0.01.
\+ The group mean was significantly different from the control at p=0.05 with Dunnett's test

Table 3
Day -5 ECG Examinations
Pretest phase

Session 1 (Scheduled)
Fexinidazole

Study Number: 0505-2007

Parameter	Group Number:	Males 1	Males 2	Males 3	Males 4	Females 1	Females 2	Females 3	Females 4
Mean Electrical Axis	N	5	3	3	5	5	3	3	5
degree	Mean	45.	51.	59.	38.	46.	44.	42.	50.
	Sdev	60.1	12.2	12.2	11.7	32.1	10.0	28.4	24.8
P wave amplitude	N	5	3	3	5	5	3	3	5
mV	Mean	0.30	0.36	0.35	0.41	0.32	0.39	0.44	0.37
	Sdev	0.072	0.042	0.059	0.092	0.143	0.139	0.087	0.093
Q wave amplitude	N	5	3	3	5	5	3	3	5
mV	Mean	-0.37	-0.37	-0.50	-0.48	-0.39	-0.67	-0.69	-0.51
	Sdev	0.168	0.144	0.242	0.181	0.128	0.389	0.160	0.348
R wave amplitude	N	5	3	3	5	5	3	3	5
mV	Mean	1.88	2.99	3.01	2.17	2.45	2.31	2.71	3.00
	Sdev	0.839	0.483	0.449	0.483	0.348	0.829	0.578	1.378
S wave amplitude	N	5	3	3	5	5	3	3	5
mV	Mean	-0.19	-0.14	-0.10	-0.16	-0.31	-0.30	-0.23	-0.12
	Sdev	0.098	0.110	0.058	0.080	0.163	0.167	0.229	0.063
S-T segment deviation	N	5	3	3	5	5	3	3	5
mV	Mean	-0.07	-0.04	-0.10	-0.06	-0.03	-0.04	-0.05	-0.07
	Sdev	0.057	0.059	0.058	0.025	0.062	0.166	0.026	0.060
T wave amplitude	N	5	3	3	5	5	3	3	5
mV	Mean	0.20	0.10	0.17	0.15	0.20	-0.22	0.55	0.05
	Sdev	0.333	0.165	0.225	0.348	0.243	0.411	0.191	0.267

Group 1:Vehicle   Group 2:50 mg/kg/day   Group 3:200 mg/kg/day   Group 4:800 mg/kg/day

Group means without footnotes were not statistically different from the control at p=0.05 or p=0.01.

Table 3
Day 24 ECG Examinations
Test period

Session 1 (Scheduled)
Fexinidazole

Study Number: 0505-2007

Parameter	Group Number:	Males 1	Males 2	Males 3	Males 4	Females 1	Females 2	Females 3	Females 4
Heart rate	N	5	3	3	5	5	3	3	5
bpm	Mean	95.	102.	97.	100.	99.	108.	112.	110.
	Sdev	19.2	38.3	36.7	13.1	20.2	11.6	14.4	32.3
R-R interval	N	5	3	3	5	5	3	3	5
ms	Mean	646.	665.	669.	606.	629.	555.	543.	580.
	Sdev	117.3	316.0	213.1	82.6	143.3	63.9	75.9	169.7
P wave duration	N	5	3	3	5	5	3	3	5
ms	Mean	35.	34.	40.	37.	42.	35.+	40.	38.
	Sdev	3.2	2.3	4.2	3.8	3.5	1.5	1.0	4.2
P-R interval	N	5	3	3	5	5	3	3	5
ms	Mean	81.	82.	90.	86.	83.	89.	87.	86.
	Sdev	8.0	6.4	7.8	5.9	6.6	0.6	2.5	10.0
QRS complex duration	N	5	3	3	5	5	3	3	5
ms	Mean	47.	45.	53.	44.	48.	48.	53.	53.
	Sdev	5.9	3.8	7.6	1.9	5.6	6.1	3.5	7.9
Q-T interval	N	5	3	3	5	5	3	3	5
ms	Mean	189.	187.	196.	197.	186.	184.	192.	192.
	Sdev	9.9	14.0	8.5	8.1	13.5	10.1	7.6	16.5
Q-T interval (Fridericia)	N	5	3	3	5	5	3	3	5
ms	Mean	219.	219.	227.	233.	218.	224.	235.+	231.
	Sdev	5.7	15.4	19.2	12.0	6.7	5.8	8.9	9.7

Group 1:Vehicle   Group 2:50 mg/kg/day   Group 3:200 mg/kg/day   Group 4:800 mg/kg/day

Group means without footnotes were not statistically different from the control at p=0.05 or p=0.01.
\+ The group mean was significantly different from the control at p=0.05 with Dunnett’s test

Table 3
Day 24 ECG Examinations
Test period

Session 1 (Scheduled)
Fexinidazole
Study Number: 0505-2007

Parameter	Group Number:	Males 1	Males 2	Males 3	Males 4	Females 1	Females 2	Females 3	Females 4
Mean Electrical Axis	N	5	3	3	5	5	3	3	5
degree	Mean	52.	57.	61.	63.	53.	56.	50.	53.
	Sdev	43.2	6.7	2.1	23.1	36.3	9.2	19.1	23.7
P wave amplitude	N	5	3	3	5	5	3	3	5
mV	Mean	0.35	0.39	0.38	0.41	0.40	0.40	0.48	0.40
	Sdev	0.059	0.012	0.036	0.086	0.070	0.062	0.167	0.162
Q wave amplitude	N	5	3	3	5	5	3	3	5
mV	Mean	-0.32	-0.40	-0.63	-0.55	-0.42	-0.57	-0.68	-0.46
	Sdev	0.110	0.111	0.460	0.295	0.220	0.240	0.137	0.314
R wave amplitude	N	5	3	3	5	5	3	3	5
mV	Mean	2.05	3.17	3.24	2.25	2.62	2.62	3.05	2.88
	Sdev	0.904	0.706	0.299	0.406	0.443	0.691	0.813	1.182
S wave amplitude	N	5	3	3	5	5	3	3	5
mV	Mean	-0.16	-0.13	-0.16	-0.23	-0.26	-0.38	-0.26	-0.12
	Sdev	0.104	0.026	0.142	0.130	0.236	0.302	0.254	0.076
S-T segment deviation	N	5	3	3	5	5	3	3	5
mV	Mean	-0.05	-0.08	-0.10	-0.07	-0.02	-0.02	-0.08	-0.06
	Sdev	0.028	0.084	0.066	0.077	0.054	0.038	0.025	0.072
T wave amplitude	N	5	3	3	5	5	3	3	5
mV	Mean	0.29	-0.20	0.02	-0.14	0.40	0.03	0.46	-0.02+
	Sdev	0.286	0.168	0.335	0.222	0.209	0.313	0.135	0.218

Group 1:Vehicle   Group 2:50 mg/kg/day   Group 3:200 mg/kg/day   Group 4:800 mg/kg/day

Group means without footnotes were not statistically different from the control at p=0.05 or p=0.01.
\+ The group mean was significantly different from the control at p=0.05 with Dunnett’s test

Table 3
Day 38 ECG Examinations
Test period

Session 1 (Scheduled)
Fexinidazole

Study Number: 0505-2007

Parameter	Group Number:	Males 1	Males 2	Males 3	Males 4	Females 1	Females 2	Females 3	Females 4
Heart rate	N	2	0	0	2	2	0	0	2
bpm	Mean	83.	-	-	91.	101.	-	-	102.
	Sdev	15.6	-	-	35.4	35.4	-	-	28.3
R-R interval	N	2	0	0	2	2	0	0	2
ms	Mean	734.	-	-	708.	629.	-	-	609.
	Sdev	137.9	-	-	273.7	219.9	-	-	169.0
P wave duration	N	2	0	0	2	2	0	0	2
ms	Mean	39.	-	-	42.	41.	-	-	40.
	Sdev	4.9	-	-	3.5	4.9	-	-	2.8
P-R interval	N	2	0	0	2	2	0	0	2
ms	Mean	87.	-	-	101.	102.	-	-	90.
	Sdev	9.2	-	-	2.8	19.8	-	-	2.1
QRS complex duration	N	2	0	0	2	2	0	0	2
ms	Mean	51.	-	-	51.	51.	-	-	49.
	Sdev	3.5	-	-	11.3	5.7	-	-	7.8
Q-T interval	N	2	0	0	2	2	0	0	2
ms	Mean	199.	-	-	203.	194.	-	-	204.
	Sdev	7.1	-	-	14.8	10.6	-	-	10.6
Q-T interval (Fridericia)	N	2	0	0	2	2	0	0	2
ms	Mean	222.	-	-	230.	228.	-	-	241.
	Sdev	21.7	-	-	13.2	14.4	-	-	9.8

Group 1:Vehicle Group 2:50 mg/kg/day Group 3:200 mg/kg/day Group 4:800 mg/kg/day

Group means without footnotes were not statistically different from the control at p=0.05 or p=0.01.

Table 3
Day 38 ECG Examinations
Test period

Session 1 (Scheduled)
Fexinidazole

Study Number: 0505-2007

Parameter	Group Number:	Males 1	Males 2	Males 3	Males 4	Females 1	Females 2	Females 3	Females 4
Mean Electrical Axis	N	2	0	0	2	2	0	0	2
degree	Mean	19.	-	-	84.	57.	-	-	70.
	Sdev	53.7	-	-	7.8	22.6	-	-	6.4
P wave amplitude	N	2	0	0	2	2	0	0	2
mV	Mean	0.30	-	-	0.39	0.39	-	-	0.36
	Sdev	0.057	-	-	0.078	0.127	-	-	0.141
Q wave amplitude	N	2	0	0	2	2	0	0	2
mV	Mean	-0.26	-	-	-0.34	-0.39	-	-	-0.46
	Sdev	0.141	-	-	0.184	0.014	-	-	0.269
R wave amplitude	N	2	0	0	2	2	0	0	2
mV	Mean	1.61	-	-	2.23	2.64	-	-	2.98
	Sdev	0.997	-	-	0.332	0.403	-	-	1.301
S wave amplitude	N	2	0	0	2	2	0	0	2
mV	Mean	-0.30	-	-	-0.17	-0.36	-	-	-0.09
	Sdev	0.049	-	-	0.014	0.283	-	-	0.042
S-T segment deviation	N	2	0	0	2	2	0	0	2
mV	Mean	-0.03	-	-	-0.07	0.05	-	-	0.00
	Sdev	0.064	-	-	0.014	0.064	-	-	0.071
T wave amplitude	N	2	0	0	2	2	0	0	2
mV	Mean	0.22	-	-	0.27	-0.04	-	-	0.18
	Sdev	0.141	-	-	0.269	0.573	-	-	0.071

Group 1:Vehicle   Group 2:50 mg/kg/day   Group 3:200 mg/kg/day   Group 4:800 mg/kg/day

Group means without footnotes were not statistically different from the control at p=0.05 or p=0.01.

Table 4 Ophthalmoscopic Examination

Table 4
Incidence of Ophthalmoscopic Findings

Fexinidazole | Study Number: 0505-2007

Study Day	Group:	Males 1	2	3	4
-5					
NORMAL	/Normal (No Abnormal Findings)	5	3	3	5
24					
NORMAL	/Normal (No Abnormal Findings)	5	3	3	5
38					
NORMAL	/Normal (No Abnormal Findings)	2	0	0	2

Group 1:Vehicle   Group 2:50 mg/kg/day   Group 3:200 mg/kg/day   Group 4:800 mg/kg/day

Table 4

Incidence of Ophthalmoscopic Findings

Fexinidazole | Study Number: 0505-2007

Study Day	Group:	Females 1	2	3	4
-5					
NORMAL	/Normal (No Abnormal Findings)	5	3	3	5
24					
NORMAL	/Normal (No Abnormal Findings)	4	3	3	5
CORNEA	/opacity area/s, monolateral	1	0	0	0
38					
NORMAL	/Normal (No Abnormal Findings)	1	0	0	2
CORNEA	/opacity area/s, monolateral	1	0	0	0

Group 1:Vehicle   Group 2:50 mg/kg/day   Group 3:200 mg/kg/day   Group 4:800 mg/kg/day

Table 5 Hematology

Table 5
Day -7 Hematology Data
Pretest phase

Session 1 (Scheduled)
Fexinidazole

Study Number: 0505-2007

Parameter	Group Number:	Males 1	Males 2	Males 3	Males 4	Females 1	Females 2	Females 3	Females 4
RED BLOOD CELLS	N	5	3	3	5	5	3	3	5
10^6/mcL	Mean	6.55	7.08	6.74	6.57	6.54	7.46*	6.77	6.81
	Sdev	0.342	0.498	0.662	0.484	0.298	0.427	0.162	0.403
HEMOGLOBIN	N	5	3	3	5	5	3	3	5
g/dL	Mean	14.9	16.2	15.3	15.0	14.9	17.2*	15.4	15.5
	Sdev	0.86	1.10	1.35	0.96	0.79	0.85	0.65	0.73
HEMATOCRIT	N	5	3	3	5	5	3	3	5
%	Mean	42.4	46.0	43.8	42.8	43.9	50.5*	45.2	46.1
	Sdev	2.45	2.97	4.01	2.84	2.10	2.25	1.69	2.44
MEAN CORPUSCULAR VOLUME	N	5	3	3	5	5	3	3	5
fL	Mean	64.8	65.0	65.0	65.2	67.1	67.8	66.8	67.6
	Sdev	1.07	1.42	0.52	0.89	0.49	0.86	0.96	1.36
MEAN CORPUSCULAR HEMOGLOBIN	N	5	3	3	5	5	3	3	5
pg	Mean	22.8	22.8	22.7	22.9	22.7	23.1	22.6	22.8
	Sdev	0.62	0.70	0.20	0.36	0.23	0.20	0.55	0.67
MEAN CORPUSCULAR HGB CONC.	N	5	3	3	5	5	3	3	5
g/dL	Mean	35.2	35.1	34.9	35.1	33.8	34.1	33.9	33.7
	Sdev	0.44	0.35	0.15	0.29	0.37	0.17	0.40	0.35
RED CELL DISTRIBUTION WIDTH	N	5	3	3	5	5	3	3	5
%	Mean	13.2	13.1	13.1	13.1	11.2	11.4	11.4	11.2
	Sdev	0.53	0.23	0.26	0.26	0.48	0.36	0.15	0.31

Group 1:Vehicle Group 2:50 mg/kg/day Group 3:200 mg/kg/day Group 4:800 mg/kg/day

Group means without footnotes were not statistically different from the control at p=0.05 or p=0.01.
* The group mean was significantly different from the control at p=0.01 with Dunnett’s test of significance

Table 5
Day -7 Hematology Data
Pretest phase

Session 1 (Scheduled)
Fexinidazole

Study Number: 0505-2007

Parameter	Group Number:	Males 1	Males 2	Males 3	Males 4	Females 1	Females 2	Females 3	Females 4
HEMOGLOBIN DISTRIB. WIDTH	N	5	3	3	5	5	3	3	5
g/dL	Mean	1.59	1.65	1.59	1.62	1.70	1.71	1.67	1.68
	Sdev	0.048	0.040	0.085	0.088	0.044	0.021	0.047	0.041
RETICULOCYTES	N	5	3	3	5	5	3	3	5
%	Mean	0.7	1.1	0.7	0.6	0.5	0.8	0.5	0.5
	Sdev	0.27	0.21	0.17	0.26	0.13	0.25	0.14	0.21
RETICULOCYTES ABS	N	5	3	3	5	5	3	3	5
10^9/L	Mean	48.2	76.1	46.2	42.3	30.3	61.5+	33.7	37.1
	Sdev	18.30	10.24	15.60	19.39	7.54	20.22	9.01	15.87
MEAN CORPUSCOLAR VOL. RETIC.	N	5	3	3	5	5	3	3	5
fL	Mean	85.4	87.4	86.0	85.4	85.7	88.0	84.8	85.3
	Sdev	2.57	2.45	3.33	2.68	1.44	3.67	2.87	3.31
MEAN HEMOGLOBIN CONC. RETIC.	N	5	3	3	5	5	3	3	5
g/dL	Mean	30.5	30.0	30.4	30.8	29.0	28.5	28.7	29.1
	Sdev	0.78	0.95	0.59	0.63	0.62	0.83	0.26	0.71
CELLULAR HEMOGLOBIN RETIC.	N	5	3	3	5	5	3	3	5
pg	Mean	25.9	26.2	26.0	26.2	24.6	25.0	24.3	24.5
	Sdev	0.41	0.76	0.92	0.56	0.38	0.35	0.65	0.75
PLATELETS	N	5	3	3	5	5	3	3	5
10^3/mcL	Mean	332.	229.	322.	300.	341.	321.	348.	294.
	Sdev	100.0	53.6	85.0	88.3	82.3	89.4	69.6	30.1

Group 1:Vehicle   Group 2:50 mg/kg/day   Group 3:200 mg/kg/day   Group 4:800 mg/kg/day

Group means without footnotes were not statistically different from the control at p=0.05 or p=0.01.
\+ The group mean was significantly different from the control at p=0.05 with Dunnett’s test of significance

Table 5
Day -7 Hematology Data
Pretest phase

Session 1 (Scheduled)
Fexinidazole

Study Number: 0505-2007

Parameter	Group Number:	Males 1	Males 2	Males 3	Males 4	Females 1	Females 2	Females 3	Females 4
MEAN PLATELET VOLUME	N	5	3	3	5	5	3	3	5
fL	Mean	11.1	11.8	11.4	11.0	9.5	10.5	9.4	9.6
	Sdev	0.91	0.66	1.97	1.62	1.12	1.46	0.99	0.53
PLATELET DISTRIBUTION WIDTH	N	5	3	3	5	5	3	3	5
%	Mean	62.4	63.7	60.7	62.7	56.6	59.6	56.9	54.3
	Sdev	2.51	4.32	4.91	3.24	6.62	1.83	1.92	3.79
PLATELET HEMATOCRIT	N	5	3	3	5	5	3	3	5
%	Mean	0.37	0.27	0.36	0.33	0.32	0.33	0.32	0.28
	Sdev	0.110	0.056	0.040	0.093	0.083	0.066	0.074	0.016
WHITE BLOOD CELLS	N	5	3	3	5	5	3	3	5
10^3/mcL	Mean	8.93	8.50	10.34	8.30	10.50	10.82	9.58	8.07+
	Sdev	1.218	1.792	1.990	1.632	1.667	1.275	1.092	0.493
NEUTROPHILS ABS	N	5	3	3	5	5	3	3	5
10^3/mcL	Mean	5.01	4.74	6.31	4.70	6.12	6.53	5.57	4.59
	Sdev	0.909	1.311	1.067	1.317	1.129	1.184	2.026	0.517
LYMPHOCYTES ABS	N	5	3	3	5	5	3	3	5
10^3/mcL	Mean	3.07	2.89	3.02	2.77	3.47	3.12	2.92	2.63
	Sdev	0.370	0.480	0.608	0.525	0.655	0.152	0.870	0.263
MONOCYTES ABS	N	5	3	3	5	5	3	3	5
10^3/mcL	Mean	0.43	0.41	0.64	0.42	0.50	0.53	0.42	0.31
	Sdev	0.105	0.137	0.361	0.095	0.148	0.129	0.078	0.102

Group 1:Vehicle　Group 2:50 mg/kg/day　Group 3:200 mg/kg/day　Group 4:800 mg/kg/day

Group means without footnotes were not statistically different from the control at p=0.05 or p=0.01.
\+ The group mean was significantly different from the control at p=0.05 with Dunnett’s test of significance

Table 5
Day -7 Hematology Data
Pretest phase

Session 1 (Scheduled)
Fexinidazole

Study Number: 0505-2007

Parameter	Group Number:	Males 1	Males 2	Males 3	Males 4	Females 1	Females 2	Females 3	Females 4
EOSINOPHILS ABS	N	5	3	3	5	5	3	3	5
10^3/mcL	Mean	0.28	0.26	0.21	0.28	0.21	0.43	0.46	0.39
	Sdev	0.162	0.139	0.044	0.219	0.131	0.140	0.483	0.357
BASOPHILS ABS	N	5	3	3	5	5	3	3	5
10^3/mcL	Mean	0.08	0.14	0.11	0.10	0.14	0.15	0.15	0.10
	Sdev	0.037	0.042	0.023	0.029	0.031	0.031	0.091	0.021
LARGE UNSTAINED CELLS ABS	N	5	3	3	5	5	3	3	5
10^3/mcL	Mean	0.04	0.06	0.06	0.04	0.07	0.05	0.05	0.04
	Sdev	0.016	0.015	0.021	0.021	0.011	0.010	0.040	0.013
NEUTROPHILS %	N	5	3	3	5	5	3	3	5
%	Mean	55.9	55.2	61.2	56.2	58.2	60.1	57.2	56.9
	Sdev	4.69	5.03	2.36	7.00	5.12	3.70	13.89	5.46
LYMPHOCITES %	N	5	3	3	5	5	3	3	5
%	Mean	34.7	34.5	29.3	33.6	33.1	29.1	31.2	32.7
	Sdev	4.10	5.57	2.67	4.85	4.58	2.45	11.31	3.55
MONOCYTES %	N	5	3	3	5	5	3	3	5
%	Mean	4.9	4.8	5.8	5.1	4.7	4.9	4.4	3.8
	Sdev	0.86	1.04	2.46	1.22	0.72	0.98	0.35	1.30
EOSINOPHILS %	N	5	3	3	5	5	3	3	5
%	Mean	3.2	2.9	2.1	3.3	2.0	4.0	5.1	4.8
	Sdev	1.69	1.10	0.53	2.59	1.06	1.66	5.72	4.15

Group 1:Vehicle   Group 2:50 mg/kg/day   Group 3:200 mg/kg/day   Group 4:800 mg/kg/day

Group means without footnotes were not statistically different from the control at p=0.05 or p=0.01.

Table 5
Day -7 Hematology Data
Pretest phase

Session 1 (Scheduled)
Fexinidazole

Study Number: 0505-2007

Parameter	Group Number:	Males 1	2	3	4	Females 1	2	3	4
BASOPHILS %	N	5	3	3	5	5	3	3	5
%	Mean	0.9	1.8	1.1	1.2	1.4	1.4	1.6	1.3
	Sdev	0.40	0.96	0.15	0.33	0.32	0.26	0.98	0.30
LARGE UNSTAINED CELLS %	N	5	3	3	5	5	3	3	5
%	Mean	0.5	0.7	0.5	0.5	0.6	0.5	0.6	0.5
	Sdev	0.18	0.36	0.12	0.25	0.15	0.12	0.46	0.16

Group 1:Vehicle   Group 2:50 mg/kg/day   Group 3:200 mg/kg/day   Group 4:800 mg/kg/day

Group means without footnotes were not statistically different from the control at p=0.05 or p=0.01.

Table 5
Day 28 Hematology Data
Test period

Session 1 (Scheduled)
Fexinidazole
Study Number: 0505-2007

Parameter	Group Number:	Males 1	Males 2	Males 3	Males 4	Females 1	Females 2	Females 3	Females 4
RED BLOOD CELLS	N	5	3	3	5	5	3	3	5
10^6/mcL	Mean	6.09	7.10	6.16	6.06	6.37	6.97	6.27	6.80
	Sdev	0.466	0.492	0.717	0.449	0.611	0.293	0.323	0.496
HEMOGLOBIN	N	5	3	3	5	5	3	3	5
g/dL	Mean	13.3	15.5	13.4	13.3	13.9	15.5	13.7	14.9
	Sdev	0.89	1.31	1.59	1.00	1.41	0.17	0.91	1.12
HEMATOCRIT	N	5	3	3	5	5	3	3	5
%	Mean	40.1	46.6	40.5	39.7	42.6	46.5	41.2	45.0
	Sdev	2.98	4.05	4.63	2.74	4.19	1.32	2.46	3.50
MEAN CORPUSCULAR VOLUME	N	5	3	3	5	5	3	3	5
fL	Mean	65.8	65.6	65.8	65.6	66.8	66.8	65.6	66.1
	Sdev	1.21	1.39	0.68	0.99	0.64	1.23	0.82	1.20
MEAN CORPUSCULAR HEMOGLOBIN	N	5	3	3	5	5	3	3	5
pg	Mean	21.9	21.9	21.8	22.0	21.8	22.3	21.8	21.9
	Sdev	0.61	0.36	0.49	0.15	0.46	0.71	0.59	0.54
MEAN CORPUSCULAR HGB CONC.	N	5	3	3	5	5	3	3	5
g/dL	Mean	33.3	33.4	33.1	33.5	32.6	33.3	33.2	33.1
	Sdev	0.45	0.81	0.32	0.47	0.59	0.61	0.44	0.29
RED CELL DISTRIBUTION WIDTH	N	5	3	3	5	5	3	3	5
%	Mean	12.2	11.6	12.0	11.9	11.7	12.4	11.4	11.4
	Sdev	0.24	0.53	0.40	0.39	0.39	0.49	0.45	0.23

Group 1:Vehicle   Group 2:50 mg/kg/day   Group 3:200 mg/kg/day   Group 4:800 mg/kg/day

Group means without footnotes were not statistically different from the control at p=0.05 or p=0.01.

Table 5
Day 28 Hematology Data
Test period

Session 1 (Scheduled)
Fexinidazole
Study Number: 0505-2007

Parameter	Group Number:	Males 1	Males 2	Males 3	Males 4	Females 1	Females 2	Females 3	Females 4
HEMOGLOBIN DISTRIB. WIDTH	N	5	3	3	5	5	3	3	5
g/dL	Mean	1.81	1.75	1.80	1.81	1.75	1.89	1.72	1.79
	Sdev	0.063	0.075	0.055	0.071	0.085	0.103	0.026	0.047
RETICULOCYTES	N	5	3	3	5	5	3	3	5
%	Mean	1.0	0.7	0.5	0.6	0.6	1.3	0.4	0.5
	Sdev	0.35	0.29	0.11	0.24	0.41	0.64	0.15	0.24
RETICULOCYTES ABS	N	5	3	3	5	5	3	3	5
10^9/L	Mean	60.5	47.8	30.9	33.9	38.7	86.8+	23.8	32.4
	Sdev	21.72	21.01	6.64	15.33	21.27	44.03	8.20	18.19
MEAN CORPUSCOLAR VOL. RETIC.	N	5	3	3	5	5	3	3	5
fL	Mean	89.7	89.5	87.3	87.8	87.7	89.8	86.6	88.3
	Sdev	2.47	2.91	2.64	2.59	1.48	2.60	4.00	2.38
MEAN HEMOGLOBIN CONC. RETIC.	N	5	3	3	5	5	3	3	5
g/dL	Mean	27.5	28.0	27.8	28.3	27.8	27.8	27.7	27.9
	Sdev	0.54	0.92	0.15	0.43	0.32	0.76	0.17	0.43
CELLULAR HEMOGLOBIN RETIC.	N	5	3	3	5	5	3	3	5
pg	Mean	24.5	24.9	24.2	24.7	24.3	24.8	23.9	24.5
	Sdev	0.55	0.40	0.90	0.62	0.44	0.31	1.25	0.65
PLATELETS	N	5	3	3	5	5	3	3	5
10^3/mcL	Mean	370.	242.	384.	273.	424.	387.	363.	291.+
	Sdev	107.6	13.4	190.0	63.2	48.0	17.2	127.2	35.8

Group 1:Vehicle   Group 2:50 mg/kg/day   Group 3:200 mg/kg/day   Group 4:800 mg/kg/day

Group means without footnotes were not statistically different from the control at p=0.05 or p=0.01.
\+ The group mean was significantly different from the control at p=0.05 with Dunnett's test of significance

Table 5
Day 28 Hematology Data
Test period

Session 1 (Scheduled)
Fexinidazole
Study Number: 0505-2007

Parameter	Group Number:	Males 1	Males 2	Males 3	Males 4	Females 1	Females 2	Females 3	Females 4
MEAN PLATELET VOLUME	N	5	3	3	5	5	3	3	5
fL	Mean	10.6	10.9	10.4	10.9	9.1	9.5	10.1	10.3+
	Sdev	0.79	0.93	1.10	1.46	0.60	0.85	0.40	0.66
PLATELET DISTRIBUTION WIDTH	N	5	3	3	5	5	3	3	5
%	Mean	58.7	61.5	58.3	64.4+	50.2	55.2	59.1+	57.4+
	Sdev	3.36	3.09	0.44	3.86	4.25	4.01	3.77	1.44
PLATELET HEMATOCRIT	N	5	3	3	5	5	3	3	5
%	Mean	0.39	0.26	0.38	0.30	0.39	0.37	0.36	0.30
	Sdev	0.117	0.035	0.145	0.071	0.062	0.015	0.115	0.029
WHITE BLOOD CELLS	N	5	3	3	5	5	3	3	5
10^3/mcL	Mean	8.95	7.72	7.78	7.84	11.79	11.06	9.99	7.88+
	Sdev	1.865	1.203	0.972	1.625	2.592	1.892	1.036	0.746
NEUTROPHILS ABS	N	5	3	3	5	5	3	3	5
10^3/mcL	Mean	5.51	4.36	5.07	5.19	7.26	7.34	6.57	4.97+
	Sdev	1.555	0.794	1.117	1.559	1.680	1.758	0.826	0.763
LYMPHOCYTES ABS	N	5	3	3	5	5	3	3	5
10^3/mcL	Mean	2.75	2.81	2.19	2.09+	3.57	2.62	2.65	2.22*
	Sdev	0.126	0.400	0.263	0.545	0.712	0.060	0.609	0.375
MONOCYTES ABS	N	5	3	3	5	5	3	3	5
10^3/mcL	Mean	0.44	0.23	0.25	0.32	0.60	0.55	0.49	0.30+
	Sdev	0.277	0.046	0.045	0.090	0.212	0.105	0.123	0.049

Group 1:Vehicle   Group 2:50 mg/kg/day   Group 3:200 mg/kg/day   Group 4:800 mg/kg/day

Group means without footnotes were not statistically different from the control at p=0.05 or p=0.01.
+ The group mean was significantly different from the control at p=0.05 with Dunnett's test of significance
* The group mean was significantly different from the control at p=0.01 with Dunnett's test of significance

Table 5
Day 28 Hematology Data
Test period

Session 1 (Scheduled)
Fexinidazole
Study Number: 0505-2007

Parameter	Group Number:	Males 1	Males 2	Males 3	Males 4	Females 1	Females 2	Females 3	Females 4
EOSINOPHILS ABS	N	5	3	3	5	5	3	3	5
10^3/mcL	Mean	0.16	0.20	0.19	0.15	0.22	0.44	0.16	0.29
	Sdev	0.023	0.058	0.055	0.187	0.196	0.436	0.095	0.263
BASOPHILS ABS	N	5	3	3	5	5	3	3	5
10^3/mcL	Mean	0.04	0.07*	0.05	0.04	0.06	0.07	0.07	0.04
	Sdev	0.013	0.015	0.006	0.013	0.021	0.015	0.031	0.007
LARGE UNSTAINED CELLS ABS	N	5	3	3	5	5	3	3	5
10^3/mcL	Mean	0.04	0.05	0.05	0.04	0.08	0.05	0.06	0.06
	Sdev	0.013	0.006	0.010	0.023	0.021	0.010	0.044	0.024
NEUTROPHILS %	N	5	3	3	5	5	3	3	5
%	Mean	60.8	56.3	64.6	65.5	61.4	65.9	65.8	62.9
	Sdev	5.33	2.87	6.99	10.31	3.86	5.40	5.77	5.60
LYMPHOCITES %	N	5	3	3	5	5	3	3	5
%	Mean	31.8	36.6	28.5	27.2	30.6	24.1	26.5	28.2
	Sdev	6.05	2.54	5.98	7.70	3.54	4.03	5.00	4.28
MONOCYTES %	N	5	3	3	5	5	3	3	5
%	Mean	4.7	3.0	3.2	4.2	5.1	5.0	4.8	3.8+
	Sdev	1.91	0.40	0.81	0.98	0.88	0.25	0.72	0.50
EOSINOPHILS %	N	5	3	3	5	5	3	3	5
%	Mean	1.8	2.5	2.3	2.1	1.8	4.0	1.7	3.8
	Sdev	0.42	0.40	0.51	2.91	1.21	3.72	1.16	3.62

Group 1:Vehicle Group 2:50 mg/kg/day Group 3:200 mg/kg/day Group 4:800 mg/kg/day

Group means without footnotes were not statistically different from the control at p=0.05 or p=0.01.
+ The group mean was significantly different from the control at p=0.05 with Dunnett's test of significance
* The group mean was significantly different from the control at p=0.01 with Dunnett's test of significance

Table 5
Day 28 Hematology Data
Test period

Session 1 (Scheduled)
Fexinidazole

Study Number: 0505-2007

Parameter	Group Number:	Males 1	Males 2	Males 3	Males 4	Females 1	Females 2	Females 3	Females 4
BASOPHILS %	N	5	3	3	5	5	3	3	5
%	Mean	0.4	0.9*	0.6	0.5	0.5	0.6	0.6	0.5
	Sdev	0.10	0.32	0.15	0.20	0.23	0.10	0.32	0.11
LARGE UNSTAINED CELLS %	N	5	3	3	5	5	3	3	5
%	Mean	0.5	0.7	0.6	0.6	0.6	0.5	0.6	0.7
	Sdev	0.04	0.17	0.21	0.38	0.15	0.06	0.46	0.34

Group 1:Vehicle Group 2:50 mg/kg/day Group 3:200 mg/kg/day Group 4:800 mg/kg/day

Group means without footnotes were not statistically different from the control at p=0.05 or p=0.01.
* The group mean was significantly different from the control at p=0.01 with Dunnett’s test of significance

Table 5
Day 42 Hematology Data
Test period

Session 1 (Scheduled)
Fexinidazole
Study Number: 0505-2007

Parameter	Group Number:	Males 1	Males 2	Males 3	Males 4	Females 1	Females 2	Females 3	Females 4
RED BLOOD CELLS	N	2	0	0	2	2	0	0	2
10^6/mcL	Mean	5.95	-	-	6.50	6.80	-	-	6.57
	Sdev	0.092	-	-	0.537	0.085	-	-	0.042
HEMOGLOBIN	N	2	0	0	2	2	0	0	2
g/dL	Mean	13.1	-	-	14.4	15.3	-	-	14.5
	Sdev	0.71	-	-	1.13	0.42	-	-	0.35
HEMATOCRIT	N	2	0	0	2	2	0	0	2
%	Mean	40.0	-	-	43.8	45.2	-	-	43.2
	Sdev	0.57	-	-	3.11	1.34	-	-	1.77
MEAN CORPUSCULAR VOLUME	N	2	0	0	2	2	0	0	2
fL	Mean	67.3	-	-	67.4	66.4	-	-	65.6
	Sdev	0.07	-	-	0.71	1.06	-	-	2.40
MEAN CORPUSCULAR HEMOGLOBIN	N	2	0	0	2	2	0	0	2
pg	Mean	22.1	-	-	22.1	22.5	-	-	22.0
	Sdev	0.78	-	-	0.00	0.28	-	-	0.42
MEAN CORPUSCULAR HGB CONC.	N	2	0	0	2	2	0	0	2
g/dL	Mean	32.8	-	-	32.8	33.9	-	-	33.6
	Sdev	1.27	-	-	0.28	0.14	-	-	0.49
RED CELL DISTRIBUTION WIDTH	N	2	0	0	2	2	0	0	2
%	Mean	12.8	-	-	12.8	11.4	-	-	12.0
	Sdev	0.07	-	-	0.42	0.57	-	-	0.00

Group 1:Vehicle   Group 2:50 mg/kg/day   Group 3:200 mg/kg/day   Group 4:800 mg/kg/day

Group means without footnotes were not statistically different from the control at p=0.05 or p=0.01.

CONFIDENTIAL

Table 5
Day 42 Hematology Data
Test period

Session 1 (Scheduled)
Fexinidazole

Study Number: 0505-2007

Parameter	Group Number:	Males 1	Males 2	Males 3	Males 4	Females 1	Females 2	Females 3	Females 4
HEMOGLOBIN DISTRIB. WIDTH	N	2	0	0	2	2	0	0	2
g/dL	Mean	1.78	-	-	1.97	1.65	-	-	1.75
	Sdev	0.021	-	-	0.085	0.092	-	-	0.049
RETICULOCYTES	N	2	0	0	2	2	0	0	2
%	Mean	1.0	-	-	1.2	0.4	-	-	0.7
	Sdev	0.64	-	-	0.29	0.21	-	-	0.23
RETICULOCYTES ABS	N	2	0	0	2	2	0	0	2
10^9/L	Mean	59.5	-	-	77.7	24.4	-	-	43.3
	Sdev	37.69	-	-	12.45	14.42	-	-	14.42
MEAN CORPUSCOLAR VOL. RETIC.	N	2	0	0	2	2	0	0	2
fL	Mean	91.4	-	-	88.3	85.5	-	-	88.1
	Sdev	3.04	-	-	0.21	1.98	-	-	3.25
MEAN HEMOGLOBIN CONC. RETIC.	N	2	0	0	2	2	0	0	2
g/dL	Mean	27.7	-	-	27.9	29.2	-	-	28.7
	Sdev	0.28	-	-	0.28	0.35	-	-	0.42
CELLULAR HEMOGLOBIN RETIC.	N	2	0	0	2	2	0	0	2
pg	Mean	25.3	-	-	24.6	24.8	-	-	25.2
	Sdev	0.64	-	-	0.21	0.28	-	-	1.27
PLATELETS	N	2	0	0	2	2	0	0	2
10^3/mcL	Mean	421.	-	-	336.	381.	-	-	334.
	Sdev	26.9	-	-	113.8	108.9	-	-	2.1

Group 1:Vehicle   Group 2:50 mg/kg/day   Group 3:200 mg/kg/day   Group 4:800 mg/kg/day

Group means without footnotes were not statistically different from the control at p=0.05 or p=0.01.

Table 5
Day 42 Hematology Data
Test period

Session 1 (Scheduled)
Fexinidazole
Study Number: 0505-2007

Parameter	Group Number:	Males 1	Males 2	Males 3	Males 4	Females 1	Females 2	Females 3	Females 4
MEAN PLATELET VOLUME	N	2	0	0	2	2	0	0	2
fL	Mean	10.0	-	-	9.7	9.0	-	-	9.0
	Sdev	1.20	-	-	1.70	0.07	-	-	0.07
PLATELET DISTRIBUTION WIDTH	N	2	0	0	2	2	0	0	2
%	Mean	55.8	-	-	59.8	50.9	-	-	55.0
	Sdev	2.19	-	-	1.13	1.13	-	-	0.99
PLATELET HEMATOCRIT	N	2	0	0	2	2	0	0	2
%	Mean	0.42	-	-	0.34	0.34	-	-	0.30
	Sdev	0.021	-	-	0.170	0.099	-	-	0.000
WHITE BLOOD CELLS	N	2	0	0	2	2	0	0	2
10^3/mcL	Mean	8.72	-	-	6.63	9.51	-	-	7.61
	Sdev	1.103	-	-	1.393	2.220	-	-	0.898
NEUTROPHILS ABS	N	2	0	0	2	2	0	0	2
10^3/mcL	Mean	5.12	-	-	3.60	4.87	-	-	4.59
	Sdev	0.990	-	-	1.287	1.513	-	-	0.403
LYMPHOCYTES ABS	N	2	0	0	2	2	0	0	2
10^3/mcL	Mean	2.98	-	-	2.58	3.47	-	-	2.44
	Sdev	0.014	-	-	0.163	0.219	-	-	0.410
MONOCYTES ABS	N	2	0	0	2	2	0	0	2
10^3/mcL	Mean	0.42	-	-	0.28	0.60	-	-	0.29
	Sdev	0.049	-	-	0.021	0.134	-	-	0.014

Group 1:Vehicle   Group 2:50 mg/kg/day   Group 3:200 mg/kg/day   Group 4:800 mg/kg/day

Group means without footnotes were not statistically different from the control at p=0.05 or p=0.01.

Table 5
Day 42 Hematology Data
Test period

Session 1 (Scheduled)
Fexinidazole

Study Number: 0505-2007

Parameter	Group Number:	Males 1	Males 2	Males 3	Males 4	Females 1	Females 2	Females 3	Females 4
EOSINOPHILS ABS	N	2	0	0	2	2	0	0	2
10^3/mcL	Mean	0.15	-	-	0.08	0.44	-	-	0.17
	Sdev	0.113	-	-	0.021	0.318	-	-	0.042
BASOPHILS ABS	N	2	0	0	2	2	0	0	2
10^3/mcL	Mean	0.03	-	-	0.06	0.08	-	-	0.07
	Sdev	0.021	-	-	0.014	0.014	-	-	0.007
LARGE UNSTAINED CELLS ABS	N	2	0	0	2	2	0	0	2
10^3/mcL	Mean	0.04	-	-	0.04	0.07	-	-	0.06
	Sdev	0.014	-	-	0.007	0.028	-	-	0.014
NEUTROPHILS %	N	2	0	0	2	2	0	0	2
%	Mean	58.4	-	-	53.5	50.7	-	-	60.4
	Sdev	3.96	-	-	8.20	4.10	-	-	1.84
LYMPHOCITES %	N	2	0	0	2	2	0	0	2
%	Mean	34.5	-	-	39.5	37.2	-	-	32.0
	Sdev	4.45	-	-	5.87	6.43	-	-	1.70
MONOCYTES %	N	2	0	0	2	2	0	0	2
%	Mean	4.7	-	-	4.4	6.3	-	-	3.8*
	Sdev	0.00	-	-	1.20	0.00	-	-	0.28
EOSINOPHILS %	N	2	0	0	2	2	0	0	2
%	Mean	1.6	-	-	1.2	4.3	-	-	2.3
	Sdev	1.13	-	-	0.57	2.40	-	-	0.21

Group 1:Vehicle   Group 2:50 mg/kg/day   Group 3:200 mg/kg/day   Group 4:800 mg/kg/day

Group means without footnotes were not statistically different from the control at p=0.05 or p=0.01.
* The group mean was significantly different from the control at p=0.01 with Dunnett’s test of significance

Table 5
Day 42 Hematology Data
Test period

Session 1 (Scheduled)
Fexinidazole

Study Number: 0505-2007

Parameter	Group Number:	Males 1	Males 2	Males 3	Males 4	Females 1	Females 2	Females 3	Females 4
BASOPHILS %	N	2	0	0	2	2	0	0	2
%	Mean	0.3	-	-	1.0	0.9	-	-	0.9
	Sdev	0.28	-	-	0.35	0.07	-	-	0.07
LARGE UNSTAINED CELLS %	N	2	0	0	2	2	0	0	2
%	Mean	0.5	-	-	0.6	0.8	-	-	0.8
	Sdev	0.28	-	-	0.28	0.07	-	-	0.07

Group 1:Vehicle   Group 2:50 mg/kg/day   Group 3:200 mg/kg/day   Group 4:800 mg/kg/day

Group means without footnotes were not statistically different from the control at p=0.05 or p=0.01.

Table 6 Coagulation

Table 6
Day -7 Hematology Data
Pretest phase

Session 1 (Scheduled)
Fexinidazole

Study Number: 0505-2007

Parameter	Group Number:	Males 1	Males 2	Males 3	Males 4	Females 1	Females 2	Females 3	Females 4
PROTHROMBIN TIME	N	5	3	3	5	5	3	3	5
sec	Mean	7.2	7.6	7.2	7.4	7.8	7.7	7.2	7.8
	Sdev	0.46	0.36	0.31	0.46	0.99	0.99	0.15	0.75
PROTHROMBIN TIME RATIO	N	5	3	3	5	5	3	3	5
ratio	Mean	1.0	1.0	1.0	1.0	1.1	1.1	1.0	1.1
	Sdev	0.06	0.05	0.04	0.06	0.14	0.14	0.02	0.10
ACT. PAR. THROMB. TIME	N	5	3	3	5	5	3	3	5
sec	Mean	12.5	12.8	12.7	12.6	24.2	12.6	12.8	12.9
	Sdev	0.23	0.31	0.18	0.37	25.44	0.31	0.24	0.47
ACT.PAR.THROMB.TIME RATIO	N	5	3	3	5	5	3	3	5
ratio	Mean	1.0	1.0	1.0	1.0	1.9	1.0	1.0	1.0
	Sdev	0.02	0.03	0.01	0.03	2.01	0.02	0.02	0.04
FIBRINOGEN	N	5	3	3	5	5	3	3	5
mg/dL	Mean	209.	171.	196.	209.	183.	188.	215.	166.
	Sdev	17.3	14.8	45.2	29.2	22.0	50.2	43.0	23.5

Group 1:Vehicle Group 2:50 mg/kg/day Group 3:200 mg/kg/day Group 4:800 mg/kg/day

Group means without footnotes were not statistically different from the control at p=0.05 or p=0.01.

Table 6
Day 28 Hematology Data
Test period

Session 1 (Scheduled)
Fexinidazole

Study Number: 0505-2007

Parameter	Group Number:	Males 1	Males 2	Males 3	Males 4	Females 1	Females 2	Females 3	Females 4
PROTHROMBIN TIME	N	5	3	3	5	5	3	3	5
sec	Mean	7.2	8.0	7.4	7.7	7.4	7.4	7.2	7.6
	Sdev	0.29	0.34	0.87	0.56	0.34	0.49	0.31	0.52
PROTHROMBIN TIME RATIO	N	5	3	3	5	5	3	3	5
ratio	Mean	1.0	1.1	1.0	1.1	1.0	1.0	1.0	1.0
	Sdev	0.04	0.05	0.12	0.08	0.05	0.07	0.04	0.07
ACT. PAR. THROMB. TIME	N	5	3	3	5	5	3	3	5
sec	Mean	12.4	13.1+	12.7	12.6	12.9	12.5	12.5	12.7
	Sdev	0.19	0.40	0.29	0.32	0.52	0.10	0.06	0.10
ACT.PAR.THROMB.TIME RATIO	N	5	3	3	5	5	3	3	5
ratio	Mean	1.0	1.0+	1.0	1.0	1.0	1.0	1.0	1.0
	Sdev	0.01	0.03	0.03	0.03	0.04	0.01	0.01	0.01
FIBRINOGEN	N	5	3	3	5	5	3	3	5
mg/dL	Mean	220.	164.+	216.	173.+	188.	194.	213.	184.
	Sdev	14.3	9.3	57.0	17.9	26.8	15.3	60.8	25.9

Group 1:Vehicle Group 2:50 mg/kg/day Group 3:200 mg/kg/day Group 4:800 mg/kg/day

Group means without footnotes were not statistically different from the control at p=0.05 or p=0.01.
\+ The group mean was significantly different from the control at p=0.05 with Dunnett’s test of significance

Table 6
Day 42 Hematology Data
Test period

Session 1 (Scheduled)
Fexinidazole
Study Number: 0505-2007

Parameter	Group Number:	Males 1	Males 2	Males 3	Males 4	Females 1	Females 2	Females 3	Females 4
PROTHROMBIN TIME	N	2	0	0	2	2	0	0	2
sec	Mean	7.8	-	-	7.9	7.5	-	-	7.6
	Sdev	0.74	-	-	0.05	0.17	-	-	0.59
PROTHROMBIN TIME RATIO	N	2	0	0	2	2	0	0	2
ratio	Mean	1.1	-	-	1.1	1.0	-	-	1.0
	Sdev	0.10	-	-	0.01	0.03	-	-	0.08
ACT. PAR. THROMB. TIME	N	2	0	0	2	2	0	0	2
sec	Mean	12.6	-	-	12.4	12.4	-	-	12.3
	Sdev	0.28	-	-	0.28	0.06	-	-	0.24
ACT.PAR.THROMB.TIME RATIO	N	2	0	0	2	2	0	0	2
ratio	Mean	1.0	-	-	1.0	1.0	-	-	1.0
	Sdev	0.02	-	-	0.03	0.00	-	-	0.02
FIBRINOGEN	N	2	0	0	2	2	0	0	2
mg/dL	Mean	215.	-	-	190.	167.	-	-	188.
	Sdev	11.0	-	-	35.6	0.4	-	-	19.6

Group 1:Vehicle   Group 2:50 mg/kg/day   Group 3:200 mg/kg/day   Group 4:800 mg/kg/day

Group means without footnotes were not statistically different from the control at p=0.05 or p=0.01.

Table 7 Clinical Chemistry

Table 7
Day -7 Clinical Chemistry Data
Pretest phase

Session 1 (Scheduled)
Fexinidazole

Study Number: 0505-2007

Parameter	Group Number:	Males 1	Males 2	Males 3	Males 4	Females 1	Females 2	Females 3	Females 4
UREA	N	5	3	3	5	5	3	3	5
mg/dL	Mean	30.	21.+	23.	24.	26.	31.	28.	31.
	Sdev	5.4	2.6	1.7	3.1	2.8	5.6	4.7	8.6
CREATININE	N	5	3	3	5	5	3	3	5
mg/dL	Mean	0.91	0.87	0.86	0.87	0.82	0.84	0.77	0.89
	Sdev	0.067	0.065	0.042	0.049	0.113	0.047	0.035	0.084
ASPARTATE AMINO TRANSFERASE	N	5	3	3	5	5	3	3	5
IU/L	Mean	39.	33.	35.	38.	41.	37.	36.	34.
	Sdev	8.4	5.5	10.5	8.0	7.8	4.6	7.0	5.1
ALANINE AMINO TRANSFERASE	N	5	3	3	5	5	3	3	5
IU/L	Mean	33.	30.	31.	32.	29.	29.	23.	30.
	Sdev	8.5	0.6	8.1	6.1	2.5	9.0	2.1	7.0
ALKALINE PHOSPHATASE	N	5	3	3	5	5	3	3	5
IU/L	Mean	78.	78.	78.	73.	57.	70.	68.	62.
	Sdev	23.0	10.1	23.5	16.8	16.7	13.6	14.8	21.0
GAMMA GLUTAMYL TRANSFERASE	N	5	3	3	5	5	3	3	5
IU/L	Mean	6.	8.	8.	7.	8.	10.	7.	8.
	Sdev	1.1	1.5	3.0	1.8	1.9	1.5	0.6	0.4
TOTAL BILIRUBIN	N	5	3	3	5	5	3	3	5
mg/dL	Mean	0.10	0.12	0.15	0.09	0.14	0.14	0.12	0.16
	Sdev	0.020	0.023	0.078	0.005	0.060	0.061	0.006	0.041

Group 1:Vehicle   Group 2:50 mg/kg/day   Group 3:200 mg/kg/day   Group 4:800 mg/kg/day

Group means without footnotes were not statistically different from the control at p=0.05 or p=0.01.
\+ The group mean was significantly different from the control at p=0.05 with Dunnett's test of significance

Table 7
Day -7 Clinical Chemistry Data
Pretest phase

Session 1 (Scheduled)
Fexinidazole
Study Number: 0505-2007

Parameter	Group Number:	Males 1	2	3	4	Females 1	2	3	4
TOTAL PROTEIN	N	5	3	3	5	5	3	3	5
g/dL	Mean	5.9	5.8	6.1	6.2	5.8	5.8	5.9	5.9
	Sdev	0.22	0.25	0.00	0.30	0.24	0.00	0.21	0.21
ALBUMIN	N	5	3	3	5	5	3	3	5
g/dL	Mean	2.80	2.95	2.84	2.95	2.87	2.88	2.92	2.90
	Sdev	0.152	0.104	0.254	0.125	0.175	0.268	0.156	0.127
GLOBULIN	N	5	3	3	5	5	3	3	5
g/dL	Mean	3.1	2.9	3.3	3.2	2.9	2.9	3.0	3.0
	Sdev	0.23	0.17	0.25	0.20	0.30	0.27	0.16	0.30
GLUCOSE	N	5	3	3	5	5	3	3	5
mg/dL	Mean	87.	91.	92.	94.	96.	88.	98.	100.
	Sdev	1.9	5.0	3.0	9.4	14.6	19.6	11.9	6.3
TRIGLYCERIDES	N	5	3	3	5	5	3	3	5
mg/dL	Mean	18.	22.	18.	14.	14.	30.+	22.	21.
	Sdev	5.8	13.6	4.0	5.2	3.7	10.6	5.6	7.3
TOTAL CHOLESTEROL	N	5	3	3	5	5	3	3	5
mg/dL	Mean	124.	125.	122.	117.	112.	136.	131.+	135.
	Sdev	20.9	25.2	12.5	20.8	10.6	29.6	2.3	21.8
CALCIUM	N	5	3	3	5	5	3	3	5
mg/dL	Mean	10.8	10.6	10.8	10.7	10.4	10.7	10.6	10.9+
	Sdev	0.45	0.40	0.67	0.36	0.22	0.00	0.32	0.23

Group 1:Vehicle   Group 2:50 mg/kg/day   Group 3:200 mg/kg/day   Group 4:800 mg/kg/day

Group means without footnotes were not statistically different from the control at p=0.05 or p=0.01.
\+ The group mean was significantly different from the control at p=0.05 with Dunnett’s test of significance

Table 7
Day -7 Clinical Chemistry Data
Pretest phase

Session 1 (Scheduled)
Fexinidazole

Study Number: 0505-2007

Parameter	Group Number:	Males 1	Males 2	Males 3	Males 4	Females 1	Females 2	Females 3	Females 4
PHOSPHOROUS	N	5	3	3	5	5	3	3	5
mg/dL	Mean	5.0	4.8	4.2+	4.7	4.4	4.9	4.6	4.5
	Sdev	0.37	0.32	0.38	0.42	0.59	0.15	0.17	0.84
ALBUMIN/GLOBULIN	N	5	3	3	5	5	3	3	5
	Mean	0.9	1.0	0.9	0.9	1.0	1.0	1.0	1.0
	Sdev	0.09	0.04	0.15	0.04	0.14	0.18	0.08	0.14
SODIUM	N	5	3	3	5	5	3	3	5
mEq/L	Mean	141.2	142.3	141.7	141.4	141.8	142.7	142.0	143.0
	Sdev	1.30	0.58	2.31	0.89	1.79	1.15	2.00	1.00
POTASSIUM	N	5	3	3	5	5	3	3	5
mEq/L	Mean	4.7	4.7	4.6	4.6	4.3	4.7	4.4	4.4
	Sdev	0.18	0.25	0.08	0.14	0.20	0.10	0.27	0.16
CHLORIDE	N	5	3	3	5	5	3	3	5
mEq/L	Mean	109.2	109.0	110.0	109.8	108.0	106.0	108.0	106.4
	Sdev	1.48	1.00	3.46	2.17	1.58	1.00	1.00	3.05

Group 1:Vehicle Group 2:50 mg/kg/day Group 3:200 mg/kg/day Group 4:800 mg/kg/day

Group means without footnotes were not statistically different from the control at p=0.05 or p=0.01.
\+ The group mean was significantly different from the control at p=0.05 with Dunnett’s test of significance

Table 7
Day 28 Clinical Chemistry Data
Test period

Session 1 (Scheduled)
Fexinidazole
Study Number: 0505-2007

Parameter	Group Number:	Males 1	Males 2	Males 3	Males 4	Females 1	Females 2	Females 3	Females 4
UREA	N	5	3	3	5	5	3	3	5
mg/dL	Mean	30.	24.+	27.	33.	23.	27.	27.	32.
	Sdev	1.5	1.7	6.2	6.7	1.9	4.6	4.0	11.1
CREATININE	N	5	3	3	5	5	3	3	5
mg/dL	Mean	0.90	0.87	0.85	0.93	0.89	0.89	0.84	0.93
	Sdev	0.058	0.015	0.104	0.107	0.070	0.012	0.035	0.079
ASPARTATE AMINO TRANSFERASE	N	5	3	3	5	5	3	3	5
IU/L	Mean	37.	33.	31.	31.	35.	34.	40.	29.
	Sdev	6.6	2.0	7.5	6.4	6.2	4.6	6.5	4.3
ALANINE AMINO TRANSFERASE	N	5	3	3	5	5	3	3	5
IU/L	Mean	41.	34.	30.	36.	35.	34.	29.	39.
	Sdev	12.8	7.2	2.5	9.0	1.9	8.1	6.0	17.4
ALKALINE PHOSPHATASE	N	5	3	3	5	5	3	3	5
IU/L	Mean	68.	76.	82.	100.	65.	75.	78.	74.
	Sdev	8.7	12.1	19.2	33.8	17.2	17.0	18.0	17.6
GAMMA GLUTAMYL TRANSFERASE	N	5	3	3	5	5	3	3	5
IU/L	Mean	4.	5.	6.	8.*	4.	6.	7.+	7.*
	Sdev	1.8	0.6	0.6	2.5	1.0	1.5	1.0	1.1
TOTAL BILIRUBIN	N	5	3	3	5	5	3	3	5
mg/dL	Mean	0.25	0.25	0.24	0.27	0.13	0.14	0.11	0.18
	Sdev	0.034	0.036	0.021	0.051	0.053	0.064	0.006	0.088

Group 1:Vehicle Group 2:50 mg/kg/day Group 3:200 mg/kg/day Group 4:800 mg/kg/day

Group means without footnotes were not statistically different from the control at p=0.05 or p=0.01.
+ The group mean was significantly different from the control at p=0.05 with Dunnett's test of significance
* The group mean was significantly different from the control at p=0.01 with Dunnett's test of significance

Table 7
Day 28 Clinical Chemistry Data
Test period

Session 1 (Scheduled)
Fexinidazole
Study Number: 0505-2007

Parameter	Group Number:	Males 1	Males 2	Males 3	Males 4	Females 1	Females 2	Females 3	Females 4
TOTAL PROTEIN	N	5	3	3	5	5	3	3	5
g/dL	Mean	5.4	5.4	5.3	5.4	5.6	5.5	5.4	5.6
	Sdev	0.28	0.17	0.31	0.15	0.25	0.26	0.12	0.34
ALBUMIN	N	5	3	3	5	5	3	3	5
g/dL	Mean	2.74	2.95	2.75	2.88	2.85	2.89	2.86	2.96
	Sdev	0.205	0.095	0.361	0.157	0.187	0.193	0.125	0.124
GLOBULIN	N	5	3	3	5	5	3	3	5
g/dL	Mean	2.6	2.5	2.6	2.5	2.7	2.6	2.6	2.7
	Sdev	0.13	0.11	0.21	0.10	0.32	0.08	0.02	0.29
GLUCOSE	N	5	3	3	5	5	3	3	5
mg/dL	Mean	92.	95.	99.	93.	92.	92.	99.	106.
	Sdev	6.1	2.6	7.0	8.5	10.0	18.0	13.1	7.6
TRIGLYCERIDES	N	5	3	3	5	5	3	3	5
mg/dL	Mean	23.	26.	27.	28.	21.	36.	39.+	33.
	Sdev	5.4	11.4	4.0	4.8	2.9	13.9	4.4	8.6
TOTAL CHOLESTEROL	N	5	3	3	5	5	3	3	5
mg/dL	Mean	117.	126.	141.	155.	111.	141.	143.	180.+
	Sdev	18.2	12.5	12.4	33.0	18.5	40.8	33.2	40.5
CALCIUM	N	5	3	3	5	5	3	3	5
mg/dL	Mean	10.3	10.3	10.3	10.2	10.3	10.7	10.3	10.9
	Sdev	0.57	0.10	0.71	0.48	0.53	0.38	0.46	0.38

Group 1:Vehicle Group 2:50 mg/kg/day Group 3:200 mg/kg/day Group 4:800 mg/kg/day

Group means without footnotes were not statistically different from the control at p=0.05 or p=0.01.
\+ The group mean was significantly different from the control at p=0.05 with Dunnett’s test of significance

Table 7
Day 28 Clinical Chemistry Data
Test period

Session 1 (Scheduled)
Fexinidazole
Study Number: 0505-2007

Parameter	Group Number:	Males 1	Males 2	Males 3	Males 4	Females 1	Females 2	Females 3	Females 4
PHOSPHOROUS	N	5	3	3	5	5	3	3	5
mg/dL	Mean	4.3	4.4	3.9	4.3	4.0	4.3	3.9	3.9
	Sdev	0.42	0.06	0.60	0.56	0.41	0.15	0.80	0.32
ALBUMIN/GLOBULIN	N	5	3	3	5	5	3	3	5
	Mean	1.0	1.2	1.1	1.2	1.1	1.1	1.1	1.1
	Sdev	0.08	0.05	0.21	0.09	0.18	0.05	0.05	0.12
SODIUM	N	5	3	3	5	5	3	3	5
mEq/L	Mean	141.2	142.3	142.3	142.2	142.0	142.3	143.0	144.2+
	Sdev	0.45	0.58	1.53	1.30	0.71	1.15	1.00	1.64
POTASSIUM	N	5	3	3	5	5	3	3	5
mEq/L	Mean	4.5	4.3	4.4	4.4	4.5	4.3	4.4	4.3
	Sdev	0.12	0.16	0.21	0.19	0.24	0.14	0.27	0.18
CHLORIDE	N	5	3	3	5	5	3	3	5
mEq/L	Mean	110.2	110.3	111.3	111.6	110.6	109.7	111.7	109.8
	Sdev	2.59	0.58	4.04	2.19	2.07	1.15	1.53	3.90

Group 1:Vehicle   Group 2:50 mg/kg/day   Group 3:200 mg/kg/day   Group 4:800 mg/kg/day

Group means without footnotes were not statistically different from the control at p=0.05 or p=0.01.
\+ The group mean was significantly different from the control at p=0.05 with Dunnett’s test of significance

Table 7
Day 42 Clinical Chemistry Data
Test period

Session 1 (Scheduled)
Fexinidazole
Study Number: 0505-2007

Parameter	Group Number:	Males 1	Males 2	Males 3	Males 4	Females 1	Females 2	Females 3	Females 4
UREA	N	2	0	0	2	2	0	0	2
mg/dL	Mean	37.	-	-	25.	26.	-	-	28.
	Sdev	7.1	-	-	0.7	4.9	-	-	4.2
CREATININE	N	2	0	0	2	2	0	0	2
mg/dL	Mean	0.88	-	-	0.77	0.96	-	-	0.91
	Sdev	0.042	-	-	0.057	0.035	-	-	0.049
ASPARTATE AMINO TRANSFERASE	N	2	0	0	2	2	0	0	2
IU/L	Mean	38.	-	-	40.	36.	-	-	24.+
	Sdev	9.2	-	-	5.7	3.5	-	-	2.1
ALANINE AMINO TRANSFERASE	N	2	0	0	2	2	0	0	2
IU/L	Mean	32.	-	-	43.	31.	-	-	34.
	Sdev	12.7	-	-	21.9	7.1	-	-	6.4
ALKALINE PHOSPHATASE	N	2	0	0	2	2	0	0	2
IU/L	Mean	59.	-	-	71.	80.	-	-	69.
	Sdev	3.5	-	-	10.6	37.5	-	-	9.2
GAMMA GLUTAMYL TRANSFERASE	N	2	0	0	2	2	0	0	2
IU/L	Mean	7.	-	-	10.	7.	-	-	8.
	Sdev	2.1	-	-	4.2	0.7	-	-	0.7
TOTAL BILIRUBIN	N	2	0	0	2	2	0	0	2
mg/dL	Mean	0.08	-	-	0.08	0.08	-	-	0.08
	Sdev	0.007	-	-	0.014	0.007	-	-	0.014

Group 1:Vehicle   Group 2:50 mg/kg/day   Group 3:200 mg/kg/day   Group 4:800 mg/kg/day

Group means without footnotes were not statistically different from the control at p=0.05 or p=0.01.
+ The group mean was significantly different from the control at p=0.05 with Dunnett's test of significance

Table 7
Day 42 Clinical Chemistry Data
Test period

Session 1 (Scheduled)
Fexinidazole
Study Number: 0505-2007

Parameter	Group Number:	Males 1	Males 2	Males 3	Males 4	Females 1	Females 2	Females 3	Females 4
TOTAL PROTEIN	N	2	0	0	2	2	0	0	2
g/dL	Mean	5.7	-	-	6.2+	5.9	-	-	5.8
	Sdev	0.14	-	-	0.07	0.21	-	-	0.07
ALBUMIN	N	2	0	0	2	2	0	0	2
g/dL	Mean	2.56	-	-	2.92*	2.89	-	-	2.92
	Sdev	0.014	-	-	0.035	0.028	-	-	0.021
GLOBULIN	N	2	0	0	2	2	0	0	2
g/dL	Mean	3.1	-	-	3.2	3.0	-	-	2.8
	Sdev	0.13	-	-	0.04	0.18	-	-	0.05
GLUCOSE	N	2	0	0	2	2	0	0	2
mg/dL	Mean	99.	-	-	91.	97.	-	-	105.
	Sdev	8.5	-	-	12.7	5.7	-	-	14.8
TRIGLYCERIDES	N	2	0	0	2	2	0	0	2
mg/dL	Mean	29.	-	-	23.	33.	-	-	29.
	Sdev	6.4	-	-	6.4	7.8	-	-	12.0
TOTAL CHOLESTEROL	N	2	0	0	2	2	0	0	2
mg/dL	Mean	117.	-	-	116.	123.	-	-	159.
	Sdev	44.5	-	-	35.4	17.0	-	-	40.3
CALCIUM	N	2	0	0	2	2	0	0	2
mg/dL	Mean	10.3	-	-	10.7	11.0	-	-	10.9
	Sdev	0.99	-	-	0.28	0.21	-	-	0.35

Group 1:Vehicle Group 2:50 mg/kg/day Group 3:200 mg/kg/day Group 4:800 mg/kg/day

Group means without footnotes were not statistically different from the control at p=0.05 or p=0.01.
+ The group mean was significantly different from the control at p=0.05 with Dunnett's test of significance
* The group mean was significantly different from the control at p=0.01 with Dunnett's test of significance

Table 7
Day 42 Clinical Chemistry Data
Test period

Session 1 (Scheduled)
Fexinidazole

Study Number: 0505-2007

Parameter	Group Number:	Males 1	Males 2	Males 3	Males 4	Females 1	Females 2	Females 3	Females 4
PHOSPHOROUS	N	2	0	0	2	2	0	0	2
mg/dL	Mean	4.8	-	-	4.4	4.7	-	-	4.5
	Sdev	0.35	-	-	0.49	0.07	-	-	0.21
ALBUMIN/GLOBULIN	N	2	0	0	2	2	0	0	2
	Mean	0.8	-	-	0.9+	1.0	-	-	1.0
	Sdev	0.03	-	-	0.00	0.05	-	-	0.01
SODIUM	N	2	0	0	2	2	0	0	2
mEq/L	Mean	141.5	-	-	142.0	141.0	-	-	140.0
	Sdev	0.71	-	-	0.00	1.41	-	-	0.00
POTASSIUM	N	2	0	0	2	2	0	0	2
mEq/L	Mean	4.5	-	-	4.5	4.4	-	-	4.5
	Sdev	0.11	-	-	0.17	0.12	-	-	0.03
CHLORIDE	N	2	0	0	2	2	0	0	2
mEq/L	Mean	111.5	-	-	108.5	111.0	-	-	109.5
	Sdev	3.54	-	-	0.71	1.41	-	-	2.12

Group 1:Vehicle   Group 2:50 mg/kg/day   Group 3:200 mg/kg/day   Group 4:800 mg/kg/day

Group means without footnotes were not statistically different from the control at p=0.05 or p=0.01.
\+ The group mean was significantly different from the control at p=0.05 with Dunnett’s test of significance

Table 8 Urinalysis Quantitative

Table 8
Day -7 Urine Data
Pretest phase

Session 1 (Scheduled)
Fexinidazole

Study Number: 0505-2007

Parameter	Group Number:	Males 1	Males 2	Males 3	Males 4	Females 1	Females 2	Females 3	Females 4
URINARY VOLUME	N	5	3	3	5	5	3	3	5
mL	Mean	242.8	163.7	192.7	414.0	196.2	146.3	164.7	179.6
	Sdev	71.83	88.27	174.46	258.63	110.68	97.30	108.85	112.61
PH	N	5	3	3	5	5	3	3	5
UNITS	Mean	7.2	7.5	7.0	7.0	6.9	7.0	6.8	7.1
	Sdev	0.45	0.87	2.00	0.00	0.22	0.00	0.29	0.55
SPECIFIC GRAVITY	N	5	3	3	5	5	3	3	5
	Mean	1.017	1.021	1.020	1.023	1.027	1.019	1.023	1.021
	Sdev	0.0016	0.0064	0.0136	0.0118	0.0084	0.0038	0.0125	0.0067

Group 1:Vehicle Group 2:50 mg/kg/day Group 3:200 mg/kg/day Group 4:800 mg/kg/day

Group means without footnotes were not statistically different from the control at p=0.05 or p=0.01.

Table 8
Day 28 Urine Data
Test period

Session 1 (Scheduled)
Fexinidazole
Study Number: 0505-2007

Parameter	Group Number:	Males 1	Males 2	Males 3	Males 4	Females 1	Females 2	Females 3	Females 4
URINARY VOLUME	N	5	3	3	5	5	3	3	5
mL	Mean	148.6	224.7	309.0	355.6	216.2	165.0	167.3	190.8
	Sdev	96.44	291.43	126.53	170.55	155.53	16.46	91.59	115.38
PH	N	5	3	3	5	5	3	3	5
UNITS	Mean	7.0	7.0	6.7*	7.0	7.6	7.0	7.7	7.1
	Sdev	0.00	0.00	0.29	0.00	0.89	0.00	0.58	0.55
SPECIFIC GRAVITY	N	5	3	3	5	5	3	3	5
	Mean	1.023	1.026	1.020	1.019	1.028	1.015	1.026	1.021
	Sdev	0.0049	0.0095	0.0035	0.0037	0.0073	0.0104	0.0074	0.0081

Group 1:Vehicle   Group 2:50 mg/kg/day   Group 3:200 mg/kg/day   Group 4:800 mg/kg/day

Group means without footnotes were not statistically different from the control at p=0.05 or p=0.01.
* The group mean was significantly different from the control at p=0.01 with Dunnett's test of significance

Table 8
Day 42 Urine Data
Test period

Session 1 (Scheduled)
Fexinidazole

Study Number: 0505-2007

Parameter	Group Number:	Males 1	Males 2	Males 3	Males 4	Females 1	Females 2	Females 3	Females 4
URINARY VOLUME	N	2	0	0	2	2	0	0	2
mL	Mean	416.0	-	-	130.0	196.0	-	-	236.5
	Sdev	313.96	-	-	25.46	62.23	-	-	125.16
PH	N	2	0	0	2	2	0	0	2
UNITS	Mean	6.8	-	-	7.5	8.5	-	-	8.0
	Sdev	0.35	-	-	0.71	0.71	-	-	1.41
SPECIFIC GRAVITY	N	2	0	0	2	2	0	0	2
	Mean	1.020	-	-	1.031	1.026	-	-	1.020
	Sdev	0.0078	-	-	0.0078	0.0021	-	-	0.0064

Group 1:Vehicle   Group 2:50 mg/kg/day   Group 3:200 mg/kg/day   Group 4:800 mg/kg/day

Group means without footnotes were not statistically different from the control at p=0.05 or p=0.01.

Table 9 Urinalysis Semi-quantitative

Table 9
Day -7 Urine Data
Pretest phase

Session 1 (Scheduled)
Fexinidazole

Study Number: 0505-2007

Parameter	Group Number:	Males 1	Males 2	Males 3	Males 4	Females 1	Females 2	Females 3	Females 4
WHITE BLOOD CELLS	N	5	3	3	5	5	3	3	5
SCORE	Mean	1.	1.	1.	1.	0.	0.	0.	0.
	Sdev	0.7	1.0	1.2	0.9	0.0	0.0	0.0	0.4
NITRITES	N	5	3	3	5	5	3	3	5
SCORE	Mean	0.	0.	0.	0.	0.	0.	0.	0.
	Sdev	0.4	0.0	0.0	0.4	0.0	0.0	0.0	0.5
PROTEINS	N	5	3	3	5	5	3	3	5
SCORE	Mean	0.	0.	0.	0.	0.	0.	0.	1.
	Sdev	0.4	0.6	0.6	0.5	0.0	0.0	0.0	0.9
GLUCOSE	N	5	3	3	5	5	3	3	5
SCORE	Mean	1.	0.	1.	0.	0.	0.	0.	1.
	Sdev	1.3	0.0	1.2	0.0	0.0	0.0	0.0	1.8
KETONE BODIES	N	5	3	3	5	5	3	3	5
SCORE	Mean	0.	0.	0.	0.	0.	0.	0.	0.
	Sdev	0.0	0.0	0.0	0.0	0.0	0.0	0.0	0.4
UROBILINOGEN	N	5	3	3	5	5	3	3	5
SCORE	Mean	0.	0.	0.	0.	0.	0.	0.	0.
	Sdev	0.0	0.0	0.0	0.0	0.0	0.0	0.0	0.0
BILIRUBIN	N	5	3	3	5	5	3	3	5
SCORE	Mean	0.	0.	0.	0.	0.	0.	0.	0.
	Sdev	0.0	0.0	0.0	0.0	0.0	0.0	0.0	0.0
HEMOGLOBIN/RED BLOOD CE/+	N	5	3	3	5	5	3	3	5
SCORE	Mean	1.	0.	1.	1.	0.	0.	0.	1.
	Sdev	0.9	0.6	0.6	0.5	0.0	0.0	0.0	1.4

Group 1:Vehicle   Group 2:50 mg/kg/day   Group 3:200 mg/kg/day   Group 4:800 mg/kg/day

Table 9
Day 28 Urine Data
Test period

Session 1 (Scheduled)
Fexinidazole
Study Number: 0505-2007

Parameter	Group Number:	Males 1	Males 2	Males 3	Males 4	Females 1	Females 2	Females 3	Females 4
WHITE BLOOD CELLS	N	5	3	3	5	5	3	3	5
SCORE	Mean	2.	2.	1.	2.	0.	1.	1.	0.
	Sdev	0.5	0.0	1.0	0.4	0.5	1.2	1.2	0.9
NITRITES	N	5	3	3	5	5	3	3	5
SCORE	Mean	0.	0.	0.	0.	0.	0.	1.	0.
	Sdev	0.5	0.6	0.6	0.4	0.0	0.6	0.6	0.5
PROTEINS	N	5	3	3	5	5	3	3	5
SCORE	Mean	0.	1.	0.	0.	0.	0.	0.	1.
	Sdev	0.4	0.6	0.6	0.4	0.4	0.6	0.6	0.9
GLUCOSE	N	5	3	3	5	5	3	3	5
SCORE	Mean	0.	0.	0.	0.	0.	0.	0.	1.
	Sdev	0.0	0.0	0.0	0.0	0.0	0.0	0.0	1.3
KETONE BODIES	N	5	3	3	5	5	3	3	5
SCORE	Mean	0.	0.	0.	0.	0.	0.	0.	0.
	Sdev	0.0	0.0	0.0	0.0	0.0	0.0	0.0	0.4
UROBILINOGEN	N	5	3	3	5	5	3	3	5
SCORE	Mean	0.	0.	0.	0.	0.	0.	0.	0.
	Sdev	0.0	0.0	0.0	0.0	0.0	0.0	0.0	0.0
BILIRUBIN	N	5	3	3	5	5	3	3	5
SCORE	Mean	0.	0.	0.	0.	0.	0.	0.	0.
	Sdev	0.0	0.0	0.0	0.0	0.0	0.0	0.0	0.0
HEMOGLOBIN/RED BLOOD CE/+	N	5	3	3	5	5	3	3	5
SCORE	Mean	1.	0.	1.	0.	0.	1.	0.	1.
	Sdev	0.8	0.6	0.6	0.4	0.4	1.2	0.6	1.1

Group 1:Vehicle Group 2:50 mg/kg/day Group 3:200 mg/kg/day Group 4:800 mg/kg/day

Table 9
Day 42 Urine Data
Test period

Session 1 (Scheduled)
Fexinidazole

Study Number: 0505-2007

Parameter	Group Number:	Males 1	Males 2	Males 3	Males 4	Females 1	Females 2	Females 3	Females 4
WHITE BLOOD CELLS	N	2	0	0	2	2	0	0	2
SCORE	Mean	2.	-	-	1.	2.	-	-	0.
	Sdev	0.0	-	-	1.4	0.7	-	-	0.0
NITRITES	N	2	0	0	2	2	0	0	2
SCORE	Mean	0.	-	-	1.	0.	-	-	0.
	Sdev	0.0	-	-	0.7	0.0	-	-	0.0
PROTEINS	N	2	0	0	2	2	0	0	2
SCORE	Mean	0.	-	-	1.	0.	-	-	0.
	Sdev	0.0	-	-	0.7	0.0	-	-	0.0
GLUCOSE	N	2	0	0	2	2	0	0	2
SCORE	Mean	0.	-	-	0.	0.	-	-	0.
	Sdev	0.0	-	-	0.0	0.0	-	-	0.0
KETONE BODIES	N	2	0	0	2	2	0	0	2
SCORE	Mean	0.	-	-	1.	0.	-	-	0.
	Sdev	0.0	-	-	0.7	0.0	-	-	0.0
UROBILINOGEN	N	2	0	0	2	2	0	0	2
SCORE	Mean	0.	-	-	0.	0.	-	-	0.
	Sdev	0.0	-	-	0.0	0.0	-	-	0.0
BILIRUBIN	N	2	0	0	2	2	0	0	2
SCORE	Mean	0.	-	-	0.	0.	-	-	0.
	Sdev	0.0	-	-	0.0	0.0	-	-	0.0
HEMOGLOBIN/RED BLOOD CE/+	N	2	0	0	2	2	0	0	2
SCORE	Mean	1.	-	-	1.	1.	-	-	0.
	Sdev	0.7	-	-	1.4	1.4	-	-	0.0

Group 1:Vehicle   Group 2:50 mg/kg/day   Group 3:200 mg/kg/day   Group 4:800 mg/kg/day

Table 10 Absolute Organ Weights

Table 10
Absolute Organ Weights (g)
Test period
Days 29 Interim Sacrifice

Fexinidazole | Study Number: 0505-2007

Group Number	Dose Level		Terminal Body Wt(kg)	SPLEEN	THYMUS	KIDNEYS	LIVER	ADRENALS
					Males			
1	Vehicle							
		N	3	3	3	3	3	3
		Mean	8.47	21.83	5.063	39.29	284.38	1.260
		Sdev	0.366	3.340	3.2756	4.728	40.574	0.1323
2	50 mg/kg/day							
		N	3	3	3	3	3	3
		Mean	8.21	23.94	3.000	41.86	254.01	1.233
		Sdev	1.077	3.770	0.9299	6.169	11.157	0.0757
3	200 mg/kg/day							
		N	3	3	3	3	3	3
		Mean	8.10	18.64	1.483	36.33	280.82	1.420
		Sdev	0.504	5.496	0.2954	0.820	40.392	0.3874
4	800 mg/kg/day							
		N	3	3	3	3	3	3
		Mean	7.97	19.22	2.487	38.62	320.11	1.460
		Sdev	1.249	7.269	0.8406	3.819	25.000	0.1442

Note: Data collected using grace days.

CONFIDENTIAL

Table 10
Absolute Organ Weights (g)
Test period
Days 29 Interim Sacrifice

Fexinidazole | Study Number: 0505-2007

Group Number	Dose Level		Terminal Body Wt(kg)	HEART	BRAIN	TESTES	PROSTATE
				Males			
1	Vehicle						
		N	3	3	3	3	3
		Mean	8.47	62.57	75.61	12.26	3.44
		Sdev	0.366	3.057	10.097	1.036	0.990
2	50 mg/kg/day						
		N	3	3	3	3	3
		Mean	8.21	67.97	70.33	12.93	4.66
		Sdev	1.077	5.713	3.451	2.840	0.814
3	200 mg/kg/day						
		N	3	3	3	3	3
		Mean	8.10	63.46	72.85	10.94	3.58
		Sdev	0.504	5.660	4.945	4.583	1.230
4	800 mg/kg/day						
		N	3	3	3	3	3
		Mean	7.97	60.41	66.60	11.12	3.57
		Sdev	1.249	1.059	11.516	5.596	1.981

Note: Data collected using grace days.

Table 10
Absolute Organ Weights (g)
Test period
Days 29 Interim Sacrifice

Fexinidazole | Study Number: 0505-2007

Group Number	Dose Level		Terminal Body Wt(kg)	SPLEEN	THYMUS	KIDNEYS	LIVER
			Females				
1	Vehicle						
		N	3	3	3	3	3
		Mean	6.61	16.89	1.567	31.87	248.47
		Sdev	0.276	3.199	0.7504	1.010	12.579
2	50 mg/kg/day						
		N	3	3	3	3	3
		Mean	7.52	24.23	3.203	33.48	248.92
		Sdev	0.907	7.486	1.5864	2.945	46.166
3	200 mg/kg/day						
		N	3	3	3	3	3
		Mean	7.25	19.89	2.247	36.31	282.12
		Sdev	0.688	8.765	1.4275	2.933	30.857
4	800 mg/kg/day						
		N	3	3	3	3	3
		Mean	6.62	16.72	1.323	29.80	249.56
		Sdev	0.889	3.666	0.3320	2.819	29.715

Note: Data collected using grace days.

Table 10
Absolute Organ Weights (g)
Test period
Days 29 Interim Sacrifice

Fexinidazole

Study Number: 0505-2007

Group Number	Dose Level		Terminal Body Wt(kg)	ADRENALS	HEART	BRAIN	OVARIES
				Females			
1	Vehicle						
		N	3	3	3	3	3
		Mean	6.61	1.287	56.76	74.08	0.787
		Sdev	0.276	0.1365	1.687	2.305	0.1718
2	50 mg/kg/day						
		N	3	3	3	3	3
		Mean	7.52	1.140	59.45	69.02	1.894+
		Sdev	0.907	0.0964	2.395	4.286	0.2362
3	200 mg/kg/day						
		N	3	3	3	3	3
		Mean	7.25	1.290	60.32	75.30	1.667
		Sdev	0.688	0.2307	10.318	1.752	0.4140
4	800 mg/kg/day						
		N	3	3	3	3	3
		Mean	6.62	1.213	53.86	66.86	1.654
		Sdev	0.889	0.0839	8.825	8.293	0.7240

Note: Data collected using grace days.
+ The group mean was significantly different from the control at p=0.05.

Table 10
Absolute Organ Weights (g)
Test period
Days 43 Final Sacrifice

Fexinidazole | Study Number: 0505-2007

Group Number	Dose Level		Terminal Body Wt(kg)	SPLEEN	THYMUS	KIDNEYS	LIVER	ADRENALS
				**Males**				
1	Vehicle							
		N	2	2	2	2	2	2
		Mean	7.62	18.91	2.580	32.32	246.77	1.140
		Sdev	0.351	1.563	1.4849	1.520	10.769	0.0424
4	800 mg/kg/day							
		N	2	2	2	2	2	2
		Mean	7.60	17.27	1.380	39.86	245.12	1.110
		Sdev	1.323	4.313	0.3111	8.273	51.237	0.0849

Note: Data collected using grace days.

Table 10
Absolute Organ Weights (g)
Test period
Days 43 Final Sacrifice

Fexinidazole | Study Number: 0505-2007

Group Number	Dose Level		Terminal Body Wt(kg)	HEART	BRAIN	TESTES	PROSTATE
				Males			
1	Vehicle						
		N	2	2	2	2	2
		Mean	7.62	70.02	69.58	13.63	2.38
		Sdev	0.351	2.616	6.152	1.266	1.796
4	800 mg/kg/day						
		N	2	2	2	2	2
		Mean	7.60	67.03	69.37	10.22	2.08
		Sdev	1.323	6.145	8.429	0.198	1.817

Note: Data collected using grace days.

Table 10
Absolute Organ Weights (g)
Test period
Days 43 Final Sacrifice

Fexinidazole Study Number: 0505-2007

Group Number	Dose Level		Terminal Body Wt(kg)	SPLEEN	THYMUS	KIDNEYS	LIVER
				Females			
1	Vehicle						
		N	2	2	2	2	2
		Mean	8.33	25.41	3.045	35.25	270.76
		Sdev	0.310	4.830	0.4313	0.247	7.905
4	800 mg/kg/day						
		N	2	2	2	2	2
		Mean	7.07	20.57	2.035	31.80*	263.59
		Sdev	0.348	0.148	0.7566	0.205	11.003

Note: Data collected using grace days.
* The group mean was significantly different from the control at p=0.01.

Table 10
Absolute Organ Weights (g)
Test period
Days 43 Final Sacrifice

Fexinidazole | Study Number: 0505-2007

Group Number	Dose Level		Terminal Body Wt(kg)	ADRENALS	HEART	BRAIN	OVARIES
			**F e m a l e s**				
1	Vehicle						
		N	2	2	2	2	2
		Mean	8.33	1.035	63.75	68.02	1.981
		Sdev	0.310	0.2333	4.822	4.773	0.4171
4	800 mg/kg/day						
		N	2	2	2	2	2
		Mean	7.07	1.150	51.97	68.59	1.583
		Sdev	0.348	0.0283	7.333	0.417	0.0591

Note: Data collected using grace days.

Table 11 Organ/Terminal Body Weight Ratios

Table 11
Relative Organ Weights (% Body Weight)
Test period
Days 29 Interim Sacrifice

Fexinidazole | Study Number: 0505-2007

Group Number	Dose Level		Terminal Body Wt(kg)	SPLEEN	THYMUS	KIDNEYS	LIVER	ADRENALS
				**M a l e s**				
1	Vehicle							
		N	3	3	3	3	3	3
		Mean	8.47	0.26	0.060	0.46	3.36	0.015
		Sdev	0.366	0.028	0.0390	0.039	0.511	0.0011
2	50 mg/kg/day							
		N	3	3	3	3	3	3
		Mean	8.21	0.29	0.037	0.51	3.12	0.015
		Sdev	1.077	0.056	0.0127	0.013	0.324	0.0025
3	200 mg/kg/day							
		N	3	3	3	3	3	3
		Mean	8.10	0.23	0.018	0.45	3.47	0.017
		Sdev	0.504	0.060	0.0033	0.031	0.483	0.0045
4	800 mg/kg/day							
		N	3	3	3	3	3	3
		Mean	7.97	0.24	0.031	0.49	4.07	0.019
		Sdev	1.249	0.079	0.0066	0.027	0.642	0.0043

Note: Data collected using grace days.

CONFIDENTIAL

Table 11
Relative Organ Weights (% Body Weight)
Test period
Days 29 Interim Sacrifice

Fexinidazole | Study Number: 0505-2007

Group Number	Dose Level		Terminal Body Wt(kg)	HEART	BRAIN	TESTES	PROSTATE
				Males			
1	Vehicle						
		N	3	3	3	3	3
		Mean	8.47	0.74	0.89	0.14	0.04
		Sdev	0.366	0.012	0.088	0.007	0.011
2	50 mg/kg/day						
		N	3	3	3	3	3
		Mean	8.21	0.83	0.87	0.16	0.06
		Sdev	1.077	0.051	0.139	0.021	0.009
3	200 mg/kg/day						
		N	3	3	3	3	3
		Mean	8.10	0.78	0.90	0.14	0.04
		Sdev	0.504	0.021	0.063	0.062	0.018
4	800 mg/kg/day						
		N	3	3	3	3	3
		Mean	7.97	0.77	0.85	0.13	0.04
		Sdev	1.249	0.112	0.183	0.054	0.020

Note: Data collected using grace days.

Table 11
Relative Organ Weights (% Body Weight)
Test period
Days 29 Interim Sacrifice

Fexinidazole | Study Number: 0505-2007

Group Number	Dose Level		Terminal Body Wt(kg)	SPLEEN	THYMUS	KIDNEYS	LIVER
			**Females**				
1	Vehicle						
		N	3	3	3	3	3
		Mean	6.61	0.25	0.024	0.48	3.77
		Sdev	0.276	0.039	0.0120	0.020	0.347
2	50 mg/kg/day						
		N	3	3	3	3	3
		Mean	7.52	0.32	0.041	0.45	3.29
		Sdev	0.907	0.065	0.0159	0.021	0.222
3	200 mg/kg/day						
		N	3	3	3	3	3
		Mean	7.25	0.27	0.031	0.50	3.90
		Sdev	0.688	0.092	0.0199	0.020	0.331
4	800 mg/kg/day						
		N	3	3	3	3	3
		Mean	6.62	0.25	0.020	0.45	3.79
		Sdev	0.889	0.028	0.0046	0.034	0.374

Note: Data collected using grace days.

Table 11
Relative Organ Weights (% Body Weight)
Test period
Days 29 Interim Sacrifice

Fexinidazole | Study Number: 0505-2007

Group Number	Dose Level		Terminal Body Wt(kg)	ADRENALS	HEART	BRAIN	OVARIES
				**Females**			
1	Vehicle						
		N	3	3	3	3	3
		Mean	6.61	0.019	0.86	1.12	0.012
		Sdev	0.276	0.0021	0.059	0.042	0.0028
2	50 mg/kg/day						
		N	3	3	3	3	3
		Mean	7.52	0.015	0.80	0.93	0.025
		Sdev	0.907	0.0027	0.078	0.144	0.0015
3	200 mg/kg/day						
		N	3	3	3	3	3
		Mean	7.25	0.018	0.83	1.04	0.023
		Sdev	0.688	0.0043	0.070	0.100	0.0035
4	800 mg/kg/day						
		N	3	3	3	3	3
		Mean	6.62	0.019	0.81	1.01	0.024
		Sdev	0.889	0.0032	0.038	0.024	0.0072

Note: Data collected using grace days.

Table 11
Relative Organ Weights (% Body Weight)
Test period
Days 43 Final Sacrifice

Fexinidazole   Study Number: 0505-2007

Group Number	Dose Level		Terminal Body Wt(kg)	SPLEEN	THYMUS	KIDNEYS	LIVER	ADRENALS
					Males			
1	Vehicle							
		N	2	2	2	2	2	2
		Mean	7.62	0.25	0.033	0.42	3.24	0.015
		Sdev	0.351	0.009	0.0179	0.000	0.008	0.0012
4	800 mg/kg/day							
		N	2	2	2	2	2	2
		Mean	7.60	0.23	0.018	0.52	3.22	0.015
		Sdev	1.323	0.017	0.0009	0.018	0.114	0.0037

Note: Data collected using grace days.

Table 11
Relative Organ Weights (% Body Weight)
Test period
Days 43 Final Sacrifice

Fexinidazole | Study Number: 0505-2007

Group Number	Dose Level		Terminal Body Wt(kg)	HEART	BRAIN	TESTES	PROSTATE
				Males			
1	Vehicle						
		N	2	2	2	2	2
		Mean	7.62	0.92	0.92	0.18	0.03
		Sdev	0.351	0.008	0.123	0.025	0.022
4	800 mg/kg/day						
		N	2	2	2	2	2
		Mean	7.60	0.89	0.92	0.14	0.03
		Sdev	1.323	0.074	0.049	0.021	0.019

Note: Data collected using grace days.

Table 11
Relative Organ Weights (% Body Weight)
Test period
Days 43 Final Sacrifice

Fexinidazole

Study Number: 0505-2007

Group Number	Dose Level		Terminal Body Wt(kg)	SPLEEN	THYMUS	KIDNEYS	LIVER
			**F e m a l e s**				
1	Vehicle						
		N	2	2	2	2	2
		Mean	8.33	0.30	0.037	0.42	3.25
		Sdev	0.310	0.047	0.0065	0.019	0.026
4	800 mg/kg/day						
		N	2	2	2	2	2
		Mean	7.07	0.29	0.029	0.45	3.73
		Sdev	0.348	0.016	0.0093	0.019	0.028

Note: Data collected using grace days.

Table 11
Relative Organ Weights (% Body Weight)
Test period
Days 43 Final Sacrifice

Fexinidazole | Study Number: 0505-2007

Group Number	Dose Level		Terminal Body Wt (kg)	ADRENALS	HEART	BRAIN	OVARIES
			**F e m a l e s**				
1	Vehicle						
		N	2	2	2	2	2
		Mean	8.33	0.012	0.77	0.82	0.024
		Sdev	0.310	0.0023	0.087	0.088	0.0059
4	800 mg/kg/day						
		N	2	2	2	2	2
		Mean	7.07	0.016	0.73	0.97	0.022
		Sdev	0.348	0.0004	0.068	0.042	0.0003

Note: Data collected using grace days.

Table 12 Gross Pathology

Table 12
Incidence Summary for Gross Necropsy Observations
Test period
Days 29 Interim Sacrifice

Fexinidazole | Study Number: 0505-2007

	Males				Females			
Group Number:	1	2	3	4	1	2	3	4
Number in Group:	3	3	3	3	3	3	3	3
GENER. CONDITION								
GOOD	3	3	2	2	2	3	2	3
FAIRLY GOOD	0	0	1	1	1	0	1	0
LYMPH NODES								
ENLARGED	0	0	1	0	0	0	0	0
PERITONEAL CAV.								
CLEAR LIQUID CONTENT	0	0	1	0	2	0	1	0
PROSTATE								
SMALL	0	0	0	1	0	0	0	0
TESTES								
SMALL, BILATERALLY	0	0	1	1	0	0	0	0
FLACCID	0	0	1	1	0	0	0	0
THYMUS								
SMALL	0	0	0	0	1	0	1	0

Note: The necropsy was conducted over multiple days.
Group 1:Vehicle Group 2:50 mg/kg/day Group 3:200 mg/kg/day Group 4:800 mg/kg/day

Table 12
Incidence Summary for Gross Necropsy Observations
Test period
Days 43 Final Sacrifice

Fexinidazole Study Number: 0505-2007

	Males				Females			
Group Number:	1	2	3	4	1	2	3	4
Number in Group:	2	0	0	2	2	0	0	2
GENER. CONDITION								
GOOD	2	0	0	1	2	0	0	2
FAIRLY GOOD	0	0	0	1	0	0	0	0
PROSTATE								
SMALL	1	0	0	1	0	0	0	0

Note: The necropsy was conducted over multiple days.
Group 1:Vehicle Group 2:50 mg/kg/day Group 3:200 mg/kg/day Group 4:800 mg/kg/day

Table 13 Microscopic Pathology

Table 13
Expanded Incidence Summary for Microscopic Observations
Test period
Days 29 Interim Sacrifice

Fexinidazole | Study Number: 0505-2007

CONTROLS FROM GROUP(S): 1	ANIMAL SEX:	Males				Females			
	DOSAGE GROUP:	1	2	3	4	1	2	3	4
TISSUES WITH DIAGNOSES	NO. IN GROUP:	3	3	3	3	3	3	3	3
ADRENALS	NUMBER EXAMINED:	3	0	0	3	3	0	0	3
AORTA-THORACIC	NUMBER EXAMINED:	3	0	0	3	3	0	0	3
ECTOPIC THYROID									
	Nad>	3	0	0	2	3	0	0	2
	Present>	0	0	0	1	0	0	0	1
BONE MARROW	NUMBER EXAMINED:	3	3	3	3	3	0	0	3
REDUCED CELLULARITY									
	Nad>	3	3	2	2	3	0	0	3
	Minimal>	0	0	1	0	0	0	0	0
	Moderate>	0	0	0	1	0	0	0	0
ATROPHY OF ADIPOSE TISSUE IN STERNAL AND FEMORAL MARROW									
	Nad>	3	3	3	2	3	0	0	3
	Moderate>	0	0	0	1	0	0	0	0
BRAIN	NUMBER EXAMINED:	3	0	0	3	3	0	0	3
CECUM	NUMBER EXAMINED:	3	0	0	3	3	0	0	3
COLON	NUMBER EXAMINED:	3	0	0	3	3	0	0	3
CRYPT DILATATION WITH/WITHOUT LUMENAL NECROTIC DEBRIS									
	Nad>	1	0	0	3	3	0	0	3
	Minimal>	2	0	0	0	0	0	0	0
DIAPHRAGM	NUMBER EXAMINED:	3	0	0	3	3	0	0	3
DUODENUM	NUMBER EXAMINED:	3	0	0	3	3	0	0	3
ECTOPIC PANCREATIC TISSUE IN SUBMUCOSA									
	Nad>	3	0	0	3	3	0	0	3

Nad = No abnormalities detected NOS = Not otherwise specified
Group 1:Vehicle Group 2:50 mg/kg/day Group 3:200 mg/kg/day Group 4:800 mg/kg/day

Table 13
Expanded Incidence Summary for Microscopic Observations
Test period
Days 29 Interim Sacrifice

Fexinidazole — Study Number: 0505-2007

CONTROLS FROM GROUP(S): 1	ANIMAL SEX:	Males				Females			
	DOSAGE GROUP:	1	2	3	4	1	2	3	4
T I S S U E S W I T H D I A G N O S E S	NO. IN GROUP:	3	3	3	3	3	3	3	3
DUODENUM (Continued)	.NUMBER EXAMINED:	3	0	0	3	3	0	0	3
CRYPT DILATATION WITH/WITHOUT LUMENAL NECROTIC DEBRIS									
	Nad>	2	0	0	3	3	0	0	1
	Minimal>	0	0	0	0	0	0	0	2
	Slight>	1	0	0	0	0	0	0	0
EPIDIDYMIDES	.NUMBER EXAMINED:	3	0	0	3	0	0	0	0
IMMATURE									
	Nad>	3	0	0	2	0	0	0	0
	Present>	0	0	0	1	0	0	0	0
LUMENAL GERM CELLS/DEBRIS									
	Nad>	3	0	0	2	0	0	0	0
	Minimal>	0	0	0	1	0	0	0	0
ESOPHAGUS	.NUMBER EXAMINED:	3	0	0	3	3	0	0	3
ACUTE INFLAMMATION									
	Nad>	3	0	0	2	3	0	0	3
	Minimal>	0	0	0	1	0	0	0	0
CHRONIC INFLAMMATION									
	Nad>	3	0	0	3	2	0	0	2
	Minimal>	0	0	0	0	1	0	0	1
EYES	.NUMBER EXAMINED:	3	0	0	3	3	0	0	3
FEMUR HEAD	.NUMBER EXAMINED:	3	0	0	3	3	0	0	3
GALL BLADDER	.NUMBER EXAMINED:	3	0	0	3	3	0	0	3
LYMPHOCYTIC INFILTRATION									
	Nad>	1	0	0	0	0	0	0	0
	Minimal>	2	0	0	2	2	0	0	2
	Slight>	0	0	0	1	1	0	0	1
HEART	.NUMBER EXAMINED:	3	0	0	3	3	0	0	3

Nad = No abnormalities detected NOS = Not otherwise specified
Group 1:Vehicle Group 2:50 mg/kg/day Group 3:200 mg/kg/day Group 4:800 mg/kg/day

Table 13
Expanded Incidence Summary for Microscopic Observations
Test period
Days 29 Interim Sacrifice

Fexinidazole | Study Number: 0505-2007

CONTROLS FROM GROUP(S): 1	ANIMAL SEX:	Males				Females			
	DOSAGE GROUP:	1	2	3	4	1	2	3	4
TISSUES WITH DIAGNOSES	NO. IN GROUP:	3	3	3	3	3	3	3	3
HEART (Continued)	NUMBER EXAMINED:	3	0	0	3	3	0	0	3
ACUTE INFLAMMATION									
	Nad>	3	0	0	2	3	0	0	3
	Minimal>	0	0	0	1	0	0	0	0
MESOTHELIAL HYPERPLASIA, ATRIAL									
	Nad>	1	0	0	3	2	0	0	2
	Minimal>	1	0	0	0	0	0	0	0
	Slight>	1	0	0	0	1	0	0	1
ARTERIAL MEDIAL HYPERTROPHY									
	Nad>	3	0	0	3	3	0	0	2
	Minimal>	0	0	0	0	0	0	0	1
ILEUM	NUMBER EXAMINED:	3	0	0	3	3	0	0	3
JEJUNUM	NUMBER EXAMINED:	3	0	0	3	3	0	0	3
KIDNEYS	NUMBER EXAMINED:	3	0	0	3	3	0	0	3
CHRONIC INFLAMMATION									
	Nad>	3	0	0	3	2	0	0	3
	Minimal>	0	0	0	0	1	0	0	0
PAPILLARY MINERALIZATION									
	Nad>	0	0	0	1	0	0	0	1
	Minimal>	3	0	0	2	3	0	0	2
CORTICAL TUBULAR DILATION									
	Nad>	3	0	0	3	3	0	0	3
CORTICAL FIBROSIS									
	Nad>	3	0	0	3	3	0	0	3
CORTICAL TUBULAR REGENERATIVE BASOPHILIA									
	Nad>	3	0	0	3	3	0	0	3
ATROPHY OF ADJACENT ADIPOSE TISSUE									
	Nad>	3	0	0	2	3	0	0	3
	Slight>	0	0	0	1	0	0	0	0
LACRIMAL GLANDS	NUMBER EXAMINED:	3	0	0	3	3	0	0	3

Nad = No abnormalities detected NOS = Not otherwise specified
Group 1:Vehicle Group 2:50 mg/kg/day Group 3:200 mg/kg/day Group 4:800 mg/kg/day

Table 13
Expanded Incidence Summary for Microscopic Observations
Test period
Days 29 Interim Sacrifice

Fexinidazole — Study Number: 0505-2007

CONTROLS FROM GROUP(S): 1	ANIMAL SEX:	Males				Females			
	DOSAGE GROUP:	1	2	3	4	1	2	3	4
TISSUES WITH DIAGNOSES	NO. IN GROUP:	3	3	3	3	3	3	3	3
LACRIMAL GLANDS (Continued)	NUMBER EXAMINED:	3	0	0	3	3	0	0	3
ONLY ONE GLAND AVAILABLE FOR EXAMINATION									
	Nad>	2	0	0	2	2	0	0	2
	Present>	1	0	0	1	1	0	0	1
LIVER	NUMBER EXAMINED:	3	0	0	3	3	0	0	3
CHRONIC INFLAMMATION									
	Nad>	1	0	0	2	3	0	0	0
	Minimal>	2	0	0	1	0	0	0	3
GLYCOGEN DEPLETION									
	Nad>	3	0	0	2	3	0	0	3
	Slight>	0	0	0	1	0	0	0	0
HEPATOCELLULAR VACUOLATION									
	Nad>	3	0	0	3	2	0	0	3
	Slight>	0	0	0	0	1	0	0	0
EXTRAMEDULLARY HEMATOPOIESIS									
	Nad>	2	0	0	3	2	0	0	3
	Minimal>	1	0	0	0	1	0	0	0
MANDIBULAR L.N.	NUMBER EXAMINED:	3	0	1	3	3	0	0	3
SINUS ERYTHROCYTES/ERYTHROPHAGOCYTOSIS									
	Nad>	3	0	1	3	2	0	0	3
	Minimal>	0	0	0	0	1	0	0	0
MESENTERIC L.N.	NUMBER EXAMINED:	3	0	1	3	3	0	0	3
SINUS ERYTHROCYTES/ERYTHROPHAGOCYTOSIS									
	Nad>	1	0	1	2	0	0	0	1
	Minimal>	1	0	0	1	3	0	0	2
	Slight>	1	0	0	0	0	0	0	0
LUNG	NUMBER EXAMINED:	3	0	0	3	3	0	0	3

Nad = No abnormalities detected   NOS = Not otherwise specified
Group 1:Vehicle   Group 2:50 mg/kg/day   Group 3:200 mg/kg/day   Group 4:800 mg/kg/day

Table 13
Expanded Incidence Summary for Microscopic Observations
Test period
Days 29 Interim Sacrifice

Fexinidazole | Study Number: 0505-2007

CONTROLS FROM GROUP(S): 1	ANIMAL SEX:	Males				Females			
	DOSAGE GROUP:	1	2	3	4	1	2	3	4
TISSUES WITH DIAGNOSES	NO. IN GROUP:	3	3	3	3	3	3	3	3
LUNG (Continued)	NUMBER EXAMINED:	3	0	0	3	3	0	0	3
ALVEOLAR HEMORRHAGE									
	Nad>	3	0	0	3	3	0	0	3
ACUTE INFLAMMATION									
	Nad>	3	0	0	3	1	0	0	2
	Minimal>	0	0	0	0	2	0	0	1
CHRONIC INFLAMMATION									
	Nad>	2	0	0	3	2	0	0	3
	Minimal>	0	0	0	0	1	0	0	0
	Slight>	1	0	0	0	0	0	0	0
ALVEOLAR MACROPHAGE INFILTRATION									
	Nad>	1	0	0	1	0	0	0	0
	Minimal>	2	0	0	2	3	0	0	2
	Slight>	0	0	0	0	0	0	0	1
BRONCHOPNEUMONIA									
	Nad>	3	0	0	3	3	0	0	2
	Slight>	0	0	0	0	0	0	0	1
CAPILLARY ANGIOMATOSIS									
	Nad>	3	0	0	3	3	0	0	3
MAMMARY GLAND	NUMBER EXAMINED:	3	0	0	3	3	3	3	3
NO MAMMARY TISSUE IN THE SECTION									
	Nad>	0	0	0	0	0	3	3	3
	Present>	3	0	0	3	3	0	0	0
IMMATURE									
	Nad>	3	0	0	3	3	2	3	3
	Present>	0	0	0	0	0	1	0	0
EDEMA									
	Nad>	3	0	0	3	3	1	0	1
	Minimal>	0	0	0	0	0	1	1	0
	Slight>	0	0	0	0	0	1	1	2
	Moderate>	0	0	0	0	0	0	1	0

Nad = No abnormalities detected NOS = Not otherwise specified
Group 1:Vehicle Group 2:50 mg/kg/day Group 3:200 mg/kg/day Group 4:800 mg/kg/day

Table 13
Expanded Incidence Summary for Microscopic Observations
Test period
Days 29 Interim Sacrifice

Fexinidazole | Study Number: 0505-2007

CONTROLS FROM GROUP(S): 1	ANIMAL SEX:	Males				Females			
	DOSAGE GROUP:	1	2	3	4	1	2	3	4
T I S S U E S W I T H D I A G N O S E S	NO. IN GROUP:	3	3	3	3	3	3	3	3
MAMMARY GLAND (Continued)	NUMBER EXAMINED:	3	0	0	3	3	3	3	3
STROMAL PROLIFERATION									
	Nad>	3	0	0	3	3	1	0	1
	Minimal>	0	0	0	0	0	1	0	0
	Slight>	0	0	0	0	0	0	2	2
	Moderate>	0	0	0	0	0	1	1	0
DUCTAL-ALVEOLAR HYPERPLASIA									
	Nad>	3	0	0	3	3	1	0	1
	Minimal>	0	0	0	0	0	1	1	1
	Slight>	0	0	0	0	0	1	1	1
	Moderate>	0	0	0	0	0	0	1	0
LOBULAR HYPERPLASIA									
	Nad>	3	0	0	3	3	3	3	2
	Slight>	0	0	0	0	0	0	0	1
SECRETORY ACTIVITY									
	Nad>	3	0	0	3	3	3	3	2
	Slight>	0	0	0	0	0	0	0	1
SKELETAL MUSCLE	NUMBER EXAMINED:	3	0	0	3	3	0	0	3
CHRONIC INFLAMMATION									
	Nad>	3	0	0	3	3	0	0	3
SCIATIC NERVE	NUMBER EXAMINED:	3	0	0	3	3	0	0	3
OPTIC NERVES	NUMBER EXAMINED:	3	0	0	3	3	0	0	3
OVARIES	NUMBER EXAMINED:	0	0	0	0	3	3	3	3
IMMATURE									
	Nad>	0	0	0	0	0	3	3	3
	Present>	0	0	0	0	3	0	0	0
CORPORA LUTEA									
	Nad>	0	0	0	0	3	1	0	0
	Present>	0	0	0	0	0	2	3	3

Nad = No abnormalities detected   NOS = Not otherwise specified
Group 1:Vehicle   Group 2:50 mg/kg/day   Group 3:200 mg/kg/day   Group 4:800 mg/kg/day

Table 13
Expanded Incidence Summary for Microscopic Observations
Test period
Days 29 Interim Sacrifice

Fexinidazole | Study Number: 0505-2007

CONTROLS FROM GROUP(S): 1	ANIMAL SEX:	Males				Females			
	DOSAGE GROUP:	1	2	3	4	1	2	3	4
TISSUES WITH DIAGNOSES	NO. IN GROUP:	3	3	3	3	3	3	3	3
OVARIES (Continued)	NUMBER EXAMINED:	0	0	0	0	3	3	3	3
CYSTS									
	Nad>	0	0	0	0	3	3	3	3
PANCREAS	NUMBER EXAMINED:	3	0	0	3	3	0	0	3
ACINAR DEGRANULATION									
	Nad>	3	0	0	2	3	0	0	3
	Minimal>	0	0	0	1	0	0	0	0
ACINAR APOPTOSIS									
	Nad>	3	0	0	3	3	0	0	3
PITUITARY	NUMBER EXAMINED:	3	0	0	3	3	0	0	3
CYSTS, PARS DISTALIS									
	Nad>	1	0	0	3	2	0	0	3
	Minimal>	0	0	0	0	1	0	0	0
	Slight>	2	0	0	0	0	0	0	0
CRANIOPHARINGEAL CYSTS									
	Nad>	3	0	0	3	3	0	0	3
PROSTATE	NUMBER EXAMINED:	3	0	0	3	0	0	0	0
IMMATURE									
	Nad>	3	0	0	2	0	0	0	0
	Present>	0	0	0	1	0	0	0	0
PARATHYROIDS	NUMBER EXAMINED:	3	0	0	3	3	0	0	3
ONLY ONE PARATHYROID AVAILABLE FOR EXAMINATION									
	Nad>	1	0	0	2	0	0	0	0
	Present>	2	0	0	1	3	0	0	3
CYSTS									
	Nad>	3	0	0	2	2	0	0	2
	Minimal>	0	0	0	1	1	0	0	1
SPINAL CORD-CERV.	NUMBER EXAMINED:	3	0	0	3	3	0	0	3
STAGE OF ESTRUS	NUMBER EXAMINED:	0	0	0	0	3	3	3	3

Nad = No abnormalities detected   NOS = Not otherwise specified
Group 1:Vehicle   Group 2:50 mg/kg/day   Group 3:200 mg/kg/day   Group 4:800 mg/kg/day

Table 13
Expanded Incidence Summary for Microscopic Observations
Test period
Days 29 Interim Sacrifice

Fexinidazole Study Number: 0505-2007

CONTROLS FROM GROUP(S): 1	ANIMAL SEX:	Males				Females			
	DOSAGE GROUP:	1	2	3	4	1	2	3	4
TISSUES WITH DIAGNOSES	NO. IN GROUP:	3	3	3	3	3	3	3	3
STAGE OF ESTRUS (Continued)	NUMBER EXAMINED:	0	0	0	0	3	3	3	3
IMMATURE									
	Nad>	0	0	0	0	0	3	3	3
	Present>	0	0	0	0	3	0	0	0
PROESTRUS									
	Nad>	0	0	0	0	3	3	3	3
ESTRUS									
	Nad>	0	0	0	0	3	2	3	3
	Present>	0	0	0	0	0	1	0	0
METESTRUS									
	Nad>	0	0	0	0	3	3	3	2
	Present>	0	0	0	0	0	0	0	1
DIESTRUS									
	Nad>	0	0	0	0	3	1	0	1
	Present>	0	0	0	0	0	2	3	2
ANESTRUS									
	Nad>	0	0	0	0	3	3	3	3
MANDIBULAR S.G.	NUMBER EXAMINED:	3	0	0	3	3	0	0	3
LYMPHOCYTIC INFILTRATION									
	Nad>	3	0	0	3	2	0	0	2
	Minimal>	0	0	0	0	1	0	0	0
	Slight>	0	0	0	0	0	0	0	1
PAROTIDS	NUMBER EXAMINED:	3	0	0	3	3	0	0	3
LYMPHOCYTIC INFILTRATION									
	Nad>	3	0	0	3	3	0	0	2
	Minimal>	0	0	0	0	0	0	0	1
ACINAR ATROPHY									
	Nad>	2	0	0	1	1	0	0	3
	Minimal>	1	0	0	2	2	0	0	0
SKIN	NUMBER EXAMINED:	3	0	0	3	3	0	0	3

Nad = No abnormalities detected NOS = Not otherwise specified
Group 1:Vehicle Group 2:50 mg/kg/day Group 3:200 mg/kg/day Group 4:800 mg/kg/day

Table 13
Expanded Incidence Summary for Microscopic Observations
Test period
Days 29 Interim Sacrifice

Fexinidazole — Study Number: 0505-2007

CONTROLS FROM GROUP(S): 1	ANIMAL SEX:	Males				Females			
	DOSAGE GROUP:	1	2	3	4	1	2	3	4
TISSUES WITH DIAGNOSES	NO. IN GROUP:	3	3	3	3	3	3	3	3
SKIN (Continued)	NUMBER EXAMINED:	3	0	0	3	3	0	0	3
CHRONIC INFLAMMATION									
	Nad>	2	0	0	3	3	0	0	3
	Minimal>	1	0	0	0	0	0	0	0
SPLEEN	NUMBER EXAMINED:	3	0	0	3	3	0	0	3
EXTRAMEDULLARY HEMATOPOIESIS									
	Nad>	3	0	0	3	3	0	0	3
STOMACH	NUMBER EXAMINED:	3	0	0	3	3	0	0	3
ACUTE INFLAMMATION									
	Nad>	3	0	0	3	3	0	0	3
GASTRITIS									
	Nad>	3	0	0	3	3	0	0	3
STERNUM	NUMBER EXAMINED:	3	0	0	3	3	0	0	3
TESTES	NUMBER EXAMINED:	3	0	1	3	0	0	0	0
IMMATURE									
	Nad>	3	0	0	2	0	0	0	0
	Present>	0	0	1	1	0	0	0	0
SEGMENTAL HYPOPLASIA									
	Nad>	2	0	1	2	0	0	0	0
	Minimal>	0	0	0	1	0	0	0	0
	Slight>	1	0	0	0	0	0	0	0
THYROIDS	NUMBER EXAMINED:	3	0	0	3	3	0	0	3
CYSTIC FOLLICLES									
	Nad>	3	0	0	3	3	0	0	3
THYMUS	NUMBER EXAMINED:	3	0	0	2	3	0	0	3

Nad = No abnormalities detected NOS = Not otherwise specified
Group 1:Vehicle Group 2:50 mg/kg/day Group 3:200 mg/kg/day Group 4:800 mg/kg/day

Table 13
Expanded Incidence Summary for Microscopic Observations
Test period
Days 29 Interim Sacrifice

Fexinidazole — Study Number: 0505-2007

CONTROLS FROM GROUP(S): 1	ANIMAL SEX:	Males				Females			
	DOSAGE GROUP:	1	2	3	4	1	2	3	4
T I S S U E S W I T H D I A G N O S E S	NO. IN GROUP:	3	3	3	3	3	3	3	3
THYMUS (Continued)	NUMBER EXAMINED:	3	0	0	2	3	0	0	3
CYSTS									
	Nad>	2	0	0	1	2	0	0	2
	Minimal>	1	0	0	0	1	0	0	0
	Slight>	0	0	0	1	0	0	0	1
INVOLUTION									
	Nad>	1	0	0	0	0	0	0	0
	Slight>	2	0	0	1	0	0	0	0
	Moderate>	0	0	0	0	2	0	0	0
	Marked>	0	0	0	0	1	0	0	3
	Severe>	0	0	0	1	0	0	0	0
TONGUE	NUMBER EXAMINED:	3	0	0	3	3	0	0	3
ACUTE INFLAMMATION									
	Nad>	3	0	0	2	3	0	0	3
	Minimal>	0	0	0	1	0	0	0	0
CHRONIC INFLAMMATION									
	Nad>	3	0	0	3	2	0	0	3
	Minimal>	0	0	0	0	1	0	0	0
TRACHEA	NUMBER EXAMINED:	3	0	0	3	3	0	0	3
ACUTE INFLAMMATION									
	Nad>	3	0	0	3	2	0	0	3
	Minimal>	0	0	0	0	1	0	0	0
URINARY BLADDER	NUMBER EXAMINED:	3	0	0	3	3	0	0	2
MINERALIZATION IN MUSCULARIS/SUBSEROSA									
	Nad>	2	0	0	3	3	0	0	2
	Slight>	1	0	0	0	0	0	0	0
UTERUS	NUMBER EXAMINED:	0	0	0	0	3	3	3	3
IMMATURE									
	Nad>	0	0	0	0	1	3	3	3
	Present>	0	0	0	0	2	0	0	0

Nad = No abnormalities detected NOS = Not otherwise specified
Group 1:Vehicle Group 2:50 mg/kg/day Group 3:200 mg/kg/day Group 4:800 mg/kg/day

Table 13
Expanded Incidence Summary for Microscopic Observations
Test period
Days 29 Interim Sacrifice

Fexinidazole | Study Number: 0505-2007

CONTROLS FROM GROUP(S): 1	ANIMAL SEX:	Males				Females			
	DOSAGE GROUP:	1	2	3	4	1	2	3	4
TISSUES WITH DIAGNOSES	NO. IN GROUP:	3	3	3	3	3	3	3	3
UTERUS (Continued)	NUMBER EXAMINED:	0	0	0	0	3	3	3	3
ENDOMETRIAL GLAND HYPERTROPHY/HYPERPLASIA									
	Nad>	0	0	0	0	3	0	1	1
	Minimal>	0	0	0	0	0	2	0	0
	Slight>	0	0	0	0	0	0	1	1
	Moderate>	0	0	0	0	0	1	1	1
EOSINOPHILIC SECRETORY MATERIAL IN GLANDULAR LUMEN									
	Nad>	0	0	0	0	3	2	1	2
	Minimal>	0	0	0	0	0	1	2	0
	Slight>	0	0	0	0	0	0	0	1
VAGINA	NUMBER EXAMINED:	0	0	0	0	3	3	1	3

Nad = No abnormalities detected NOS = Not otherwise specified
Group 1:Vehicle Group 2:50 mg/kg/day Group 3:200 mg/kg/day Group 4:800 mg/kg/day

Table 13
Expanded Incidence Summary for Microscopic Observations
Test period
Days 43 Final Sacrifice

Fexinidazole | Study Number: 0505-2007

CONTROLS FROM GROUP(S): 1	ANIMAL SEX:	Males				Females			
	DOSAGE GROUP:	1	2	3	4	1	2	3	4
TISSUES WITH DIAGNOSES	NO. IN GROUP:	2	0	0	2	2	0	0	2
ADRENALS	NUMBER EXAMINED:	2	0	0	2	2	0	0	2
AORTA-THORACIC	NUMBER EXAMINED:	2	0	0	2	2	0	0	2
ECTOPIC THYROID									
	Nad>	2	0	0	2	2	0	0	2
BONE MARROW	NUMBER EXAMINED:	2	0	0	2	2	0	0	2
REDUCED CELLULARITY									
	Nad>	2	0	0	2	2	0	0	2
ATROPHY OF ADIPOSE TISSUE IN STERNAL AND FEMORAL MARROW									
	Nad>	2	0	0	2	2	0	0	2
BRAIN	NUMBER EXAMINED:	2	0	0	2	2	0	0	2
CECUM	NUMBER EXAMINED:	2	0	0	2	2	0	0	2
COLON	NUMBER EXAMINED:	2	0	0	2	2	0	0	2
CRYPT DILATATION WITH/WITHOUT LUMENAL NECROTIC DEBRIS									
	Nad>	2	0	0	2	2	0	0	2
DIAPHRAGM	NUMBER EXAMINED:	2	0	0	2	2	0	0	2
DUODENUM	NUMBER EXAMINED:	2	0	0	2	2	0	0	2
ECTOPIC PANCREATIC TISSUE IN SUBMUCOSA									
	Nad>	2	0	0	2	2	0	0	1
	Present>	0	0	0	0	0	0	0	1
CRYPT DILATATION WITH/WITHOUT LUMENAL NECROTIC DEBRIS									
	Nad>	1	0	0	1	2	0	0	2
	Minimal>	1	0	0	1	0	0	0	0
EPIDIDYMIDES	NUMBER EXAMINED:	2	0	0	2	0	0	0	0
IMMATURE									
	Nad>	2	0	0	2	0	0	0	0

Nad = No abnormalities detected NOS = Not otherwise specified
Group 1:Vehicle Group 2:50 mg/kg/day Group 3:200 mg/kg/day Group 4:800 mg/kg/day

Table 13
Expanded Incidence Summary for Microscopic Observations
Test period
Days 43 Final Sacrifice

Fexinidazole — Study Number: 0505-2007

CONTROLS FROM GROUP(S): 1	ANIMAL SEX:	Males				Females			
	DOSAGE GROUP:	1	2	3	4	1	2	3	4
TISSUES WITH DIAGNOSES	NO. IN GROUP:	2	0	0	2	2	0	0	2
EPIDIDYMIDES (Continued)	NUMBER EXAMINED:	2	0	0	2	0	0	0	0
LUMENAL GERM CELLS/DEBRIS									
	Nad>	2	0	0	2	0	0	0	0
ESOPHAGUS	NUMBER EXAMINED:	2	0	0	2	2	0	0	2
ACUTE INFLAMMATION									
	Nad>	2	0	0	2	2	0	0	2
CHRONIC INFLAMMATION									
	Nad>	2	0	0	2	2	0	0	2
EYES	NUMBER EXAMINED:	2	0	0	2	2	0	0	2
FEMUR HEAD	NUMBER EXAMINED:	2	0	0	2	2	0	0	2
GALL BLADDER	NUMBER EXAMINED:	2	0	0	2	2	0	0	2
LYMPHOCYTIC INFILTRATION									
	Nad>	0	0	0	0	0	0	0	1
	Minimal>	2	0	0	2	1	0	0	1
	Slight>	0	0	0	0	1	0	0	0
HEART	NUMBER EXAMINED:	2	0	0	2	2	0	0	2
ACUTE INFLAMMATION									
	Nad>	2	0	0	2	2	0	0	2
MESOTHELIAL HYPERPLASIA, ATRIAL									
	Nad>	1	0	0	2	2	0	0	1
	Minimal>	1	0	0	0	0	0	0	1
ARTERIAL MEDIAL HYPERTROPHY									
	Nad>	2	0	0	2	2	0	0	2
ILEUM	NUMBER EXAMINED:	2	0	0	2	2	0	0	2
JEJUNUM	NUMBER EXAMINED:	2	0	0	2	2	0	0	2
KIDNEYS	NUMBER EXAMINED:	2	0	0	2	2	0	0	2

Nad = No abnormalities detected NOS = Not otherwise specified
Group 1:Vehicle Group 2:50 mg/kg/day Group 3:200 mg/kg/day Group 4:800 mg/kg/day

Table 13
Expanded Incidence Summary for Microscopic Observations
Test period
Days 43 Final Sacrifice

Fexinidazole Study Number: 0505-2007

CONTROLS FROM GROUP(S): 1	ANIMAL SEX:	Males				Females			
	DOSAGE GROUP:	1	2	3	4	1	2	3	4
T I S S U E S W I T H D I A G N O S E S	NO. IN GROUP:	2	0	0	2	2	0	0	2
KIDNEYS (Continued)	NUMBER EXAMINED:	2	0	0	2	2	0	0	2
CHRONIC INFLAMMATION									
	Nad>	1	0	0	2	1	0	0	1
	Minimal>	0	0	0	0	1	0	0	1
	Slight>	1	0	0	0	0	0	0	0
PAPILLARY MINERALIZATION									
	Nad>	0	0	0	1	0	0	0	0
	Minimal>	2	0	0	1	2	0	0	2
CORTICAL TUBULAR DILATION									
	Nad>	1	0	0	2	1	0	0	2
	Minimal>	0	0	0	0	1	0	0	0
	Moderate>	1	0	0	0	0	0	0	0
CORTICAL FIBROSIS									
	Nad>	2	0	0	2	1	0	0	2
	Minimal>	0	0	0	0	1	0	0	0
CORTICAL TUBULAR REGENERATIVE BASOPHILIA									
	Nad>	0	0	0	1	1	0	0	2
	Minimal>	1	0	0	1	0	0	0	0
	Slight>	1	0	0	0	1	0	0	0
ATROPHY OF ADJACENT ADIPOSE TISSUE									
	Nad>	2	0	0	2	2	0	0	2
LACRIMAL GLANDS	NUMBER EXAMINED:	2	0	0	2	2	0	0	2
ONLY ONE GLAND AVAILABLE FOR EXAMINATION									
	Nad>	2	0	0	2	2	0	0	2
LIVER	NUMBER EXAMINED:	2	0	0	2	2	0	0	2
CHRONIC INFLAMMATION									
	Nad>	2	0	0	2	2	0	0	2
GLYCOGEN DEPLETION									
	Nad>	2	0	0	2	2	0	0	2

Nad = No abnormalities detected NOS = Not otherwise specified
Group 1:Vehicle Group 2:50 mg/kg/day Group 3:200 mg/kg/day Group 4:800 mg/kg/day

Table 13
Expanded Incidence Summary for Microscopic Observations
Test period
Days 43 Final Sacrifice

Fexinidazole | Study Number: 0505-2007

CONTROLS FROM GROUP(S): 1	ANIMAL SEX:	Males				Females			
	DOSAGE GROUP:	1	2	3	4	1	2	3	4
TISSUES WITH DIAGNOSES	NO. IN GROUP:	2	0	0	2	2	0	0	2
LIVER (Continued)	NUMBER EXAMINED:	2	0	0	2	2	0	0	2
HEPATOCELLULAR VACUOLATION									
	Nad>	2	0	0	2	2	0	0	2
EXTRAMEDULLARY HEMATOPOIESIS									
	Nad>	0	0	0	2	1	0	0	2
	Minimal>	2	0	0	0	1	0	0	0
MANDIBULAR L.N.	NUMBER EXAMINED:	2	0	0	2	2	0	0	2
SINUS ERYTHROCYTES/ERYTHROPHAGOCYTOSIS									
	Nad>	1	0	0	2	2	0	0	2
	Minimal>	1	0	0	0	0	0	0	0
MESENTERIC L.N.	NUMBER EXAMINED:	2	0	0	2	2	0	0	2
SINUS ERYTHROCYTES/ERYTHROPHAGOCYTOSIS									
	Nad>	0	0	0	1	0	0	0	0
	Minimal>	1	0	0	1	2	0	0	2
	Slight>	1	0	0	0	0	0	0	0
LUNG	NUMBER EXAMINED:	2	0	0	2	2	0	0	2
ALVEOLAR HEMORRHAGE									
	Nad>	1	0	0	2	1	0	0	2
	Minimal>	0	0	0	0	1	0	0	0
	Slight>	1	0	0	0	0	0	0	0
ACUTE INFLAMMATION									
	Nad>	2	0	0	2	2	0	0	2
CHRONIC INFLAMMATION									
	Nad>	2	0	0	2	1	0	0	2
	Minimal>	0	0	0	0	1	0	0	0
ALVEOLAR MACROPHAGE INFILTRATION									
	Nad>	1	0	0	1	0	0	0	1
	Minimal>	1	0	0	1	1	0	0	1
	Slight>	0	0	0	0	1	0	0	0

Nad = No abnormalities detected NOS = Not otherwise specified
Group 1:Vehicle Group 2:50 mg/kg/day Group 3:200 mg/kg/day Group 4:800 mg/kg/day

Table 13
Expanded Incidence Summary for Microscopic Observations
Test period
Days 43 Final Sacrifice

Fexinidazole | Study Number: 0505-2007

CONTROLS FROM GROUP(S): 1	ANIMAL SEX:	Males				Females			
	DOSAGE GROUP:	1	2	3	4	1	2	3	4
TISSUES WITH DIAGNOSES	NO. IN GROUP:	2	0	0	2	2	0	0	2
LUNG (Continued)	NUMBER EXAMINED:	2	0	0	2	2	0	0	2
BRONCHOPNEUMONIA									
	Nad>	2	0	0	2	2	0	0	2
CAPILLARY ANGIOMATOSIS									
	Nad>	1	0	0	2	2	0	0	2
	Slight>	1	0	0	0	0	0	0	0
MAMMARY GLAND	NUMBER EXAMINED:	2	0	0	2	2	0	0	2
NO MAMMARY TISSUE IN THE SECTION									
	Nad>	0	0	0	0	2	0	0	2
	Present>	2	0	0	2	0	0	0	0
IMMATURE									
	Nad>	2	0	0	2	2	0	0	2
EDEMA									
	Nad>	2	0	0	2	0	0	0	0
	Minimal>	0	0	0	0	1	0	0	0
	Slight>	0	0	0	0	1	0	0	1
	Moderate>	0	0	0	0	0	0	0	1
STROMAL PROLIFERATION									
	Nad>	2	0	0	2	0	0	0	0
	Minimal>	0	0	0	0	1	0	0	0
	Moderate>	0	0	0	0	1	0	0	2
DUCTAL-ALVEOLAR HYPERPLASIA									
	Nad>	2	0	0	2	0	0	0	0
	Minimal>	0	0	0	0	1	0	0	0
	Moderate>	0	0	0	0	1	0	0	2
LOBULAR HYPERPLASIA									
	Nad>	2	0	0	2	2	0	0	2
SECRETORY ACTIVITY									
	Nad>	2	0	0	2	2	0	0	2
SKELETAL MUSCLE	NUMBER EXAMINED:	2	0	0	2	2	0	0	2

Nad = No abnormalities detected NOS = Not otherwise specified
Group 1:Vehicle Group 2:50 mg/kg/day Group 3:200 mg/kg/day Group 4:800 mg/kg/day

Table 13
Expanded Incidence Summary for Microscopic Observations
Test period
Days 43 Final Sacrifice

Fexinidazole | Study Number: 0505-2007

CONTROLS FROM GROUP(S): 1	ANIMAL SEX:	Males				Females			
	DOSAGE GROUP:	1	2	3	4	1	2	3	4
T I S S U E S W I T H D I A G N O S E S	NO. IN GROUP:	2	0	0	2	2	0	0	2
SKELETAL MUSCLE (Continued)	NUMBER EXAMINED:	2	0	0	2	2	0	0	2
CHRONIC INFLAMMATION									
	Nad>	2	0	0	2	2	0	0	1
	Minimal>	0	0	0	0	0	0	0	1
SCIATIC NERVE	NUMBER EXAMINED:	2	0	0	2	2	0	0	2
OPTIC NERVES	NUMBER EXAMINED:	2	0	0	2	2	0	0	2
OVARIES	NUMBER EXAMINED:	0	0	0	0	2	0	0	2
IMMATURE									
	Nad>	0	0	0	0	2	0	0	2
CORPORA LUTEA									
	Present>	0	0	0	0	2	0	0	2
CYSTS									
	Nad>	0	0	0	0	2	0	0	1
	Slight>	0	0	0	0	0	0	0	1
PANCREAS	NUMBER EXAMINED:	2	0	0	2	2	0	0	2
ACINAR DEGRANULATION									
	Nad>	2	0	0	2	2	0	0	2
ACINAR APOPTOSIS									
	Nad>	2	0	0	2	1	0	0	1
	Minimal>	0	0	0	0	1	0	0	1
PITUITARY	NUMBER EXAMINED:	2	0	0	2	2	0	0	2
CYSTS, PARS DISTALIS									
	Nad>	2	0	0	1	1	0	0	2
	Slight>	0	0	0	1	1	0	0	0
CRANIOPHARINGEAL CYSTS									
	Nad>	2	0	0	2	2	0	0	1
	Moderate>	0	0	0	0	0	0	0	1
PROSTATE	NUMBER EXAMINED:	2	0	0	2	0	0	0	0

Nad = No abnormalities detected   NOS = Not otherwise specified
Group 1:Vehicle   Group 2:50 mg/kg/day   Group 3:200 mg/kg/day   Group 4:800 mg/kg/day

Table 13
Expanded Incidence Summary for Microscopic Observations
Test period
Days 43 Final Sacrifice

Fexinidazole — Study Number: 0505-2007

CONTROLS FROM GROUP(S): 1	ANIMAL SEX:	Males				Females			
	DOSAGE GROUP:	1	2	3	4	1	2	3	4
T I S S U E S W I T H D I A G N O S E S	NO. IN GROUP:	2	0	0	2	2	0	0	2
PROSTATE (Continued)	NUMBER EXAMINED:	2	0	0	2	0	0	0	0
IMMATURE									
	Nad>	1	0	0	1	0	0	0	0
	Present>	1	0	0	1	0	0	0	0
PARATHYROIDS	NUMBER EXAMINED:	2	0	0	2	2	0	0	2
ONLY ONE PARATHYROID AVAILABLE FOR EXAMINATION									
	Nad>	1	0	0	1	0	0	0	0
	Present>	1	0	0	1	2	0	0	2
CYSTS									
	Nad>	2	0	0	1	2	0	0	2
	Minimal>	0	0	0	1	0	0	0	0
SPINAL CORD-CERV.	NUMBER EXAMINED:	2	0	0	2	2	0	0	2
STAGE OF ESTRUS	NUMBER EXAMINED:	0	0	0	0	2	0	0	2
IMMATURE									
	Nad>	0	0	0	0	2	0	0	2
PROESTRUS									
	Nad>	0	0	0	0	2	0	0	2
ESTRUS									
	Nad>	0	0	0	0	2	0	0	2
METESTRUS									
	Nad>	0	0	0	0	2	0	0	2
DIESTRUS									
	Present>	0	0	0	0	2	0	0	2
ANESTRUS									
	Nad>	0	0	0	0	2	0	0	2
MANDIBULAR S.G.	NUMBER EXAMINED:	2	0	0	2	2	0	0	2

Nad = No abnormalities detected NOS = Not otherwise specified
Group 1:Vehicle Group 2:50 mg/kg/day Group 3:200 mg/kg/day Group 4:800 mg/kg/day

Table 13
Expanded Incidence Summary for Microscopic Observations
Test period
Days 43 Final Sacrifice

Fexinidazole — Study Number: 0505-2007

CONTROLS FROM GROUP(S): 1	ANIMAL SEX:	Males				Females			
	DOSAGE GROUP:	1	2	3	4	1	2	3	4
TISSUES WITH DIAGNOSES	NO. IN GROUP:	2	0	0	2	2	0	0	2
MANDIBULAR S.G. (Continued)	NUMBER EXAMINED:	2	0	0	2	2	0	0	2
LYMPHOCYTIC INFILTRATION									
	Nad>	1	0	0	2	1	0	0	1
	Minimal>	1	0	0	0	1	0	0	0
	Slight>	0	0	0	0	0	0	0	1
PAROTIDS	NUMBER EXAMINED:	2	0	0	2	2	0	0	2
LYMPHOCYTIC INFILTRATION									
	Nad>	2	0	0	2	2	0	0	2
ACINAR ATROPHY									
	Nad>	0	0	0	1	1	0	0	1
	Minimal>	2	0	0	0	1	0	0	1
	Slight>	0	0	0	1	0	0	0	0
SKIN	NUMBER EXAMINED:	2	0	0	2	2	0	0	2
CHRONIC INFLAMMATION									
	Nad>	2	0	0	1	2	0	0	2
	Minimal>	0	0	0	1	0	0	0	0
SPLEEN	NUMBER EXAMINED:	2	0	0	2	2	0	0	2
EXTRAMEDULLARY HEMATOPOIESIS									
	Nad>	2	0	0	2	1	0	0	1
	Minimal>	0	0	0	0	1	0	0	1
STOMACH	NUMBER EXAMINED:	2	0	0	2	2	0	0	2
ACUTE INFLAMMATION									
	Nad>	2	0	0	2	2	0	0	0
	Minimal>	0	0	0	0	0	0	0	2
GASTRITIS									
	Nad>	2	0	0	2	0	0	0	2
	Minimal>	0	0	0	0	1	0	0	0
	Slight>	0	0	0	0	1	0	0	0
STERNUM	NUMBER EXAMINED:	2	0	0	2	2	0	0	2

Nad = No abnormalities detected NOS = Not otherwise specified
Group 1:Vehicle Group 2:50 mg/kg/day Group 3:200 mg/kg/day Group 4:800 mg/kg/day

Table 13
Expanded Incidence Summary for Microscopic Observations
Test period
Days 43 Final Sacrifice

Fexinidazole — Study Number: 0505-2007

CONTROLS FROM GROUP(S): 1	ANIMAL SEX:	Males				Females			
	DOSAGE GROUP:	1	2	3	4	1	2	3	4
TISSUES WITH DIAGNOSES	NO. IN GROUP:	2	0	0	2	2	0	0	2
TESTES	NUMBER EXAMINED:	2	0	0	2	0	0	0	0
IMMATURE									
	Nad>	2	0	0	2	0	0	0	0
SEGMENTAL HYPOPLASIA									
	Nad>	2	0	0	2	0	0	0	0
THYROIDS	NUMBER EXAMINED:	2	0	0	2	2	0	0	2
CYSTIC FOLLICLES									
	Nad>	1	0	0	1	2	0	0	2
	Slight>	1	0	0	1	0	0	0	0
THYMUS	NUMBER EXAMINED:	2	0	0	2	2	0	0	2
CYSTS									
	Nad>	1	0	0	1	2	0	0	1
	Minimal>	1	0	0	1	0	0	0	1
INVOLUTION									
	Minimal>	0	0	0	0	2	0	0	1
	Slight>	1	0	0	0	0	0	0	0
	Moderate>	1	0	0	0	0	0	0	1
	Marked>	0	0	0	2	0	0	0	0
TONGUE	NUMBER EXAMINED:	2	0	0	2	2	0	0	2
ACUTE INFLAMMATION									
	Nad>	2	0	0	2	2	0	0	2
CHRONIC INFLAMMATION									
	Nad>	2	0	0	2	2	0	0	2
TRACHEA	NUMBER EXAMINED:	2	0	0	2	2	0	0	2
ACUTE INFLAMMATION									
	Nad>	2	0	0	2	2	0	0	2
URINARY BLADDER	NUMBER EXAMINED:	2	0	0	2	2	0	0	2

Nad = No abnormalities detected   NOS = Not otherwise specified
Group 1:Vehicle   Group 2:50 mg/kg/day   Group 3:200 mg/kg/day   Group 4:800 mg/kg/day

Table 13
Expanded Incidence Summary for Microscopic Observations
Test period
Days 43 Final Sacrifice

Fexinidazole | Study Number: 0505-2007

CONTROLS FROM GROUP(S): 1	ANIMAL SEX:	Males				Females			
	DOSAGE GROUP:	1	2	3	4	1	2	3	4
T I S S U E S W I T H D I A G N O S E S	NO. IN GROUP:	2	0	0	2	2	0	0	2
URINARY BLADDER (Continued)	NUMBER EXAMINED:	2	0	0	2	2	0	0	2
MINERALIZATION IN MUSCULARIS/SUBSEROSA									
	Nad>	2	0	0	2	2	0	0	2
UTERUS	NUMBER EXAMINED:	0	0	0	0	2	0	0	2
IMMATURE									
	Nad>	0	0	0	0	2	0	0	2
ENDOMETRIAL GLAND HYPERTROPHY/HYPERPLASIA									
	Minimal>	0	0	0	0	0	0	0	2
	Slight>	0	0	0	0	2	0	0	0
EOSINOPHILIC SECRETORY MATERIAL IN GLANDULAR LUMEN									
	Nad>	0	0	0	0	2	0	0	0
	Minimal>	0	0	0	0	0	0	0	2
VAGINA	NUMBER EXAMINED:	0	0	0	0	2	0	0	2

Nad = No abnormalities detected   NOS = Not otherwise specified
Group 1:Vehicle   Group 2:50 mg/kg/day   Group 3:200 mg/kg/day   Group 4:800 mg/kg/day

Table 14 Histological changes in ovaries

Table 14
Histological Changes in Ovaries
Test period

Fexinidazole | Study No. 0505-2007

**Dates of necropsy**	**End of treatment**<br>**Recovery**	27-28 february 2008<br>12 march 2008										
**END OF TREATMENT – Day 29**												
Test group	1			2			3			4		
Dose (mg/kg/day)	0	0	0	50	50	50	200	200	200	800	800	800
Female No.	2560	2568	2572	2562	2563	2576	2561	2570	2567	2564	2565	2569
IMMATURE	P	P	P	-	-	-	-	-	-	-	-	-
CORPORA LUTEA	-	-	-	P	-	P	P	P	P	P	P	P

**RECOVERY – Day 43**				
Test group	1		4	
Dose (mg/kg/day)	0	0	800	800
Female No.	2575	2577	2571	2573
IMMATURE	-	-	-	-
CORPORA LUTEA	P	P	P	P

**Non gradable findings**
- = finding absent<br>P = present

Table 15 Stage of Estrus Evaluation

Table 15
Stage of Estrus Evaluation
Test period

Fexinidazole | Study No. 0505-2007

**Dates of necropsy**	**End of treatment** 27-28 february 2008<br>**Recovery** 12 march 2008											
**END OF TREATMENT**	27-28 february 2008											
Test group	1			2			3			4		
Dose (mg/kg/day)	0	0	0	50	50	50	200	200	200	800	800	800
Female No.	2560	2568	2572	2562	2563	2576	2561	2570	2567	2564	2565	2569
**Date of birth**	03-5-07	09-5-07	11-5-07	05-5-07	05-5-07	16-5-07	05-5-07	11-5-07	06-5-07	05-5-07	05-5-07	11-5-07
IMMATURE	P	P	P	-	-	-	-	-	-	-	-	-
PROESTRUS	-	-	-	-	-	-	-	-	-	-	-	-
ESTRUS	-	-	-	-	P	-	-	-	-	-	-	-
METESTRUS	-	-	-	-	-	-	-	-	-	-	-	P
DIESTRUS	-	-	-	P	-	P	P	P	P	P	P	-
ANESTRUS	-	-	-	-	-	-	-	-	-	-	-	-

**RECOVERY**	12 march 2008			
Test group	1		4	
Dose (mg/kg/day)	0	0	800	800
Female No.	2575	2577	2571	2573
**Date of birth**	16-5-07	17-5-07	11-5-07	13-5-07
**Date of birth**				
IMMATURE	-	-	-	-
PROESTRUS	-	-	-	-
ESTRUS	-	-	-	-
METESTRUS	-	-	-	-
DIESTRUS	P	P	P	P
ANESTRUS	-	-	-	-

Length of estrus stages:
Proestrus + Estrus + Metestrus = 18 days
Diestrus = 2-3 month
Anestrus = 3-5 month

APPENDICES

Appendix 1 QA Statement

Appendix 2 Clinical Signs

Appendix 2
Individual Animal Clinical Signs

Fexinidazole | Study Number: 0505-2007

M a l e s

Animal Number	Dose	Clinical Signs	Days Present	Study Day(s) Noted
2516	Vehicle	NORMAL/NO SIGNIFICANT SIGNS	36	Pretest phase -8--2
				Test period 1-29
		DIARRHEA, MODERATE	1	Test period 9
		SOFT STOOL, SLIGHT	4	Test period 2,8,22-23
		SOFT STOOL, MODERATE	5	Pretest phase -1
				Test period 10,13-14,18
2518	Vehicle	NORMAL/NO SIGNIFICANT SIGNS	34	Pretest phase -8--6,-3--2
				Test period 1-29
		DIARRHEA, SLIGHT	1	Test period 6
		DIARRHEA, MODERATE	2	Pretest phase -4,-1
		DIARRHEA, MARKED	2	Test period 14-15
		SOFT STOOL, SLIGHT	6	Pretest phase -5
				Test period 2-3,8,12-13
		SOFT STOOL, MODERATE	3	Test period 7,11,16
		REDUCED FOOD INTAKE, SLIGHT	1	Test period 12
2520	Vehicle	NORMAL/NO SIGNIFICANT SIGNS	38	Pretest phase -8--1
				Test period 1-30
		SOFT STOOL, SLIGHT	5	Test period 10-13,16
		SOFT STOOL, MODERATE	3	Test period 3-4,21
2527	Vehicle	NORMAL/NO SIGNIFICANT SIGNS	48	Pretest phase -8--5,-2--1
				Test period 1-36,38-43
		SOFT STOOL, SLIGHT	2	Test period 22,37
		SOFT STOOL, MODERATE	2	Pretest phase -4--3
		REDUCED FOOD INTAKE, SLIGHT	1	Test period 4
2533	Vehicle	NORMAL/NO SIGNIFICANT SIGNS	49	Pretest phase -8--4,-2--1
				Test period 1-34,36-43
		DIARRHEA, SLIGHT	1	Test period 6
		SOFT STOOL, SLIGHT	5	Test period 2-3,10,17,28

Appendix 2
Individual Animal Clinical Signs

Fexinidazole

Study Number: 0505-2007

M a l e s

Animal Number	Dose	Clinical Signs	Days Present	Study Day(s) Noted
2533	Vehicle	SOFT STOOL, MODERATE	4	Pretest phase -3<br>Test period 9,15,35
2514	50 mg/kg/day	NORMAL/NO SIGNIFICANT SIGNS	37	Pretest phase -8--1<br>Test period 1-29
		SOFT STOOL, SLIGHT	1	Test period 12
2521	50 mg/kg/day	NORMAL/NO SIGNIFICANT SIGNS	37	Pretest phase -8--1<br>Test period 1-29
		REDUCED FOOD INTAKE, SLIGHT	6	Test period 2,4-6,8,10
2529	50 mg/kg/day	NORMAL/NO SIGNIFICANT SIGNS	35	Pretest phase -8--4,-2<br>Test period 1-29
		DIARRHEA, SLIGHT	2	Test period 22-23
		DIARRHEA, MODERATE	2	Test period 15,30
		DIARRHEA, MARKED	1	Test period 29
		SOFT STOOL, SLIGHT	11	Pretest phase -3,-1<br>Test period 2-3,7-8,10,13,16-17,26
		SOFT STOOL, MODERATE	3	Test period 11,14,21
2515	200 mg/kg/day	NORMAL/NO SIGNIFICANT SIGNS	36	Pretest phase -8,-6--1<br>Test period 1-29
		SOFT STOOL, MODERATE	1	Pretest phase -7
2523	200 mg/kg/day	NORMAL/NO SIGNIFICANT SIGNS	36	Pretest phase -8--4,-2--1<br>Test period 1-29
		SOFT STOOL, SLIGHT	1	Pretest phase -3
		SOFT STOOL, MODERATE	3	Test period 11,14-15
2526	200 mg/kg/day	NORMAL/NO SIGNIFICANT SIGNS	36	Pretest phase -8,-6--2<br>Test period 1-30
		SOFT STOOL, MODERATE	2	Pretest phase -7,-1

Appendix 2
Individual Animal Clinical Signs

Fexinidazole Study Number: 0505-2007

M a l e s

Animal Number	Dose	Clinical Signs	Days Present	Study Day(s) Noted
2526	200 mg/kg/day	REDUCED FOOD INTAKE, SLIGHT	2	Test period 5,15
		REDUCED FOOD INTAKE, MODERATE	1	Test period 16
2517	800 mg/kg/day	NORMAL/NO SIGNIFICANT SIGNS	37	Pretest phase -8--1<br>Test period 1-29
		DIARRHEA, SLIGHT	1	Test period 22
		SOFT STOOL, SLIGHT	3	Test period 2-4
		SOFT STOOL, MODERATE	3	Test period 7-8,12
2519	800 mg/kg/day	NORMAL/NO SIGNIFICANT SIGNS	35	Pretest phase -8--6,-3--1<br>Test period 1-29
		SOFT STOOL, SLIGHT	3	Test period 2,8,22
		SOFT STOOL, MODERATE	3	Pretest phase -5--4<br>Test period 16
		REDUCED FOOD INTAKE, MODERATE	1	Test period 5
2525	800 mg/kg/day	NORMAL/NO SIGNIFICANT SIGNS	38	Pretest phase -8--1<br>Test period 1-30
		DIARRHEA, MODERATE	1	Test period 5
		SOFT STOOL, SLIGHT	3	Test period 3,8,12
2528	800 mg/kg/day	NORMAL/NO SIGNIFICANT SIGNS	51	Pretest phase -8--1<br>Test period 1-43
		DIARRHEA, MODERATE	1	Test period 4
		SOFT STOOL, SLIGHT	3	Test period 5-6,28
		SOFT STOOL, MODERATE	1	Test period 13
2530	800 mg/kg/day	NORMAL/NO SIGNIFICANT SIGNS	49	Pretest phase -8--4,-2--1<br>Test period 1-41,43
		DIARRHEA, SLIGHT	1	Test period 6
		DIARRHEA, MODERATE	2	Test period 9,42
		SOFT STOOL, SLIGHT	2	Test period 5,22

Appendix 2
Individual Animal Clinical Signs

Fexinidazole — Study Number: 0505-2007

Males

Animal Number	Dose	Clinical Signs	Days Present	Study Day(s) Noted
2530	800 mg/kg/day	SOFT STOOL, MODERATE	2	Pretest phase -3<br>Test period 16

F e m a l e

Animal Number	Dose	Clinical Signs	Days Present	Study Day(s) Noted
2560	Vehicle	NORMAL/NO SIGNIFICANT SIGNS	35	Pretest phase -8--6,-4--1
				Test period 1-28
		DIARRHEA, SLIGHT	2	Test period 5,14
		DIARRHEA, MARKED	1	Test period 28
		SOFT STOOL, SLIGHT	2	Test period 19,22
		SOFT STOOL, MODERATE	6	Pretest phase -5
				Test period 4,11,13,15,29
2568	Vehicle	NORMAL/NO SIGNIFICANT SIGNS	38	Pretest phase -8--1
				Test period 1-30
		SOFT STOOL, SLIGHT	2	Test period 14,21
		REDUCED FOOD INTAKE, SLIGHT	1	Test period 4
2572	Vehicle	NORMAL/NO SIGNIFICANT SIGNS	38	Pretest phase -8--1
				Test period 1-30
		DIARRHEA, SLIGHT	1	Test period 5
		SOFT STOOL, SLIGHT	1	Test period 11
2575	Vehicle	NORMAL/NO SIGNIFICANT SIGNS	50	Pretest phase -8--1
				Test period 1-36,38-43
		SOFT STOOL, MODERATE	2	Test period 8,37
2577	Vehicle	NORMAL/NO SIGNIFICANT SIGNS	50	Pretest phase -8--1
				Test period 1-35,37-43
		DIARRHEA, SLIGHT	2	Test period 10,17
		DIARRHEA, MODERATE	1	Test period 16
		SOFT STOOL, SLIGHT	2	Test period 3,36
		SOFT STOOL, MODERATE	1	Test period 8
2562	50 mg/kg/day	NORMAL/NO SIGNIFICANT SIGNS	37	Pretest phase -8--1
				Test period 1-29
		SOFT STOOL, SLIGHT	2	Test period 15,21

Appendix 2
Individual Animal Clinical Signs

Fexinidazole Study Number: 0505-2007

F e m a l e

Animal Number	Dose	Clinical Signs	Days Present	Study Day(s) Noted
2563	50 mg/kg/day	NORMAL/NO SIGNIFICANT SIGNS	35	Pretest phase -8--4,-1<br>Test period 2-30
		SOFT STOOL, SLIGHT	3	Pretest phase -3--2<br>Test period 16
		SOFT STOOL, MODERATE, 2-4h AFTER DOSING	1	Test period 1
2576	50 mg/kg/day	NORMAL/NO SIGNIFICANT SIGNS	38	Pretest phase -8--1<br>Test period 1-30
		REDUCED FOOD INTAKE, SLIGHT	1	Test period 4
2561	200 mg/kg/day	NORMAL/NO SIGNIFICANT SIGNS	36	Pretest phase -8--5,-3--1<br>Test period 1-29
		SOFT STOOL, SLIGHT	1	Pretest phase -4
		REDUCED FOOD INTAKE, SLIGHT	1	Test period 28
2570	200 mg/kg/day	NORMAL/NO SIGNIFICANT SIGNS	38	Pretest phase -8--1<br>Test period 1-30
		DIARRHEA, SLIGHT	4	Test period 5,12,25-26
		SOFT STOOL, SLIGHT	2	Test period 14,21
		SOFT STOOL, MODERATE	3	Test period 8,15,23
2567	200 mg/kg/day	NORMAL/NO SIGNIFICANT SIGNS	34	Pretest phase -8--5,-3<br>Test period 1-25,27-30
		SOFT STOOL, SLIGHT	5	Pretest phase -4,-2--1<br>Test period 14-15
		SOFT STOOL, MODERATE	3	Test period 6,25-26
		REDUCED FOOD INTAKE, SLIGHT	3	Test period 4,8,20
		REDUCED FOOD INTAKE, MODERATE	1	Test period 16
2564	800 mg/kg/day	NORMAL/NO SIGNIFICANT SIGNS	37	Pretest phase -8--1<br>Test period 1-29
		SOFT STOOL, SLIGHT	1	Test period 16

Appendix 2
Individual Animal Clinical Signs

Fexinidazole Study Number: 0505-2007

F e m a l e

Animal Number	Dose	Clinical Signs	Days Present	Study Day(s) Noted
2564	800 mg/kg/day	REDUCED FOOD INTAKE, SLIGHT	2	Test period 8,19
		REDUCED FOOD INTAKE, MODERATE	9	Test period 2-4,6-7,11,14-15,18
		REDUCED FOOD INTAKE, MARKED	1	Test period 5
2565	800 mg/kg/day	NORMAL/NO SIGNIFICANT SIGNS	37	Pretest phase -8--2<br>Test period 1-30
		DIARRHEA, MARKED	1	Test period 28
		SOFT STOOL, SLIGHT	1	Pretest phase -1
		REDUCED FOOD INTAKE, SLIGHT	7	Test period 8,13-16,19-20
		REDUCED FOOD INTAKE, MODERATE	8	Test period 2-7,11,18
2569	800 mg/kg/day	NORMAL/NO SIGNIFICANT SIGNS	38	Pretest phase -8--1<br>Test period 1-30
		REDUCED FOOD INTAKE, SLIGHT	2	Test period 22,28
2571	800 mg/kg/day	NORMAL/NO SIGNIFICANT SIGNS	50	Pretest phase -8--1<br>Test period 1-30,32-43
		SOFT STOOL, SLIGHT	2	Test period 21,31
		REDUCED FOOD INTAKE, SLIGHT	8	Test period 2,8,10-11,14-15,19,23
		REDUCED FOOD INTAKE, MODERATE	6	Test period 3-5,7,18,28
		REDUCED FOOD INTAKE, MARKED	1	Test period 6
2573	800 mg/kg/day	NORMAL/NO SIGNIFICANT SIGNS	47	Pretest phase -8--5,-3--1<br>Test period 1-28,32-43
		DIARRHEA, SLIGHT	1	Test period 29
		EMESIS OF FOOD, MODERATE	1	Test period 18
		SOFT STOOL, MODERATE	2	Test period 30-31
		REDUCED FOOD INTAKE, SLIGHT	16	Pretest phase -4<br>Test period 3,6,9-10,13-17,19,22<br>23,25-26,28
		REDUCED FOOD INTAKE, MODERATE	5	Test period 2,4-5,8,11

Appendix 3 Body Weights

Appendix 3
Body Weights (kg)

Fexinidazole Study Number: 0505-2007

Dose Level	Animal Number	Group/ Subgroup	Study Day -8"	1#	8	14	22	28	35	40
			M a l e s							
Vehicle										
	2516	1/1	8.19	8.45	8.36	8.48	8.63	8.30	Dead	
	2518	1/1	8.90	9.04	8.74	8.87	8.94	8.59	Dead	
	2520	1/1	7.94	8.08	8.01	8.11	8.29	8.11	Dead	
	2527	1/1	7.72	7.90	7.77	7.82	7.90	8.02	7.75	7.82
	2533	1/1	8.26	8.34	7.79	7.98	7.84	7.48	7.41	7.60
		N	5	5	5	5	5	5	2	2
		Mean	8.20	8.36	8.14	8.25	8.32	8.10	7.58	7.71
		Sdev	0.444	0.436	0.410	0.423	0.472	0.411	0.240	0.153
50 mg/kg/day										
	2514	2/1	9.61	9.78	9.54	9.68	9.92	9.43	Dead	
	2521	2/1	8.03	8.08	7.67	7.85	7.80	7.50	Dead	
	2529	2/1	7.35	7.56	7.40	7.54	7.65	7.63	Dead	
		N	3	3	3	3	3	3	0	0
		Mean	8.33	8.47	8.20	8.36	8.46	8.19	-	-
		Sdev	1.160	1.163	1.165	1.155	1.271	1.081	-	-
200 mg/kg/day										
	2515	3/1	8.93	8.87	8.76	8.84	8.73	8.33	Dead	
	2523	3/1	8.66	8.70	8.32	8.41	8.32	7.79	Dead	
	2526	3/1	7.62	7.96	7.49	7.88	7.67	7.67	Dead	
		N	3	3	3	3	3	3	0	0
		Mean	8.40	8.51	8.19	8.37	8.24	7.93	-	-
		Sdev	0.691	0.482	0.642	0.479	0.531	0.351	-	-
800 mg/kg/day										
	2517	4/1	8.00	7.85	7.36	7.31	7.12	6.76	Dead	
	2519	4/1	8.18	8.27	7.81	7.97	8.01	8.07	Dead	
	2525	4/1	9.40	9.87	9.48	9.70	9.44	9.25	Dead	
	2528	4/1	8.84	9.11	8.62	8.70	8.62	8.65	8.35	8.49
	2530	4/1	7.53	7.54	7.03	7.18	7.08	6.77	6.75	7.02
		N	5	5	5	5	5	5	2	2
		Mean	8.39	8.53	8.06	8.17	8.05	7.90	7.55	7.75
		Sdev	0.734	0.954	0.993	1.049	1.010	1.117	1.131	1.039

Note: " = Pretest phase; # = Test period

Appendix 3
Body Weights (kg)

Fexinidazole Study Number: 0505-2007

Dose Level	Animal Number	Group/ Subgroup	Study Day -8"	1#	8	14	22	28	35	40
			Females							
Vehicle										
	2560	1/1	6.85	6.83	6.72	6.46	6.50	6.27	Dead	
	2568	1/1	7.22	7.38	7.11	6.98	7.09	6.92	Dead	
	2572	1/1	7.07	7.23	6.76	6.75	6.70	6.44	Dead	
	2575	1/1	7.82	7.76	7.75	7.64	7.79	7.84	7.74	7.91
	2577	1/1	7.92	8.39	8.12	8.29	8.38	8.70	8.37	8.51
		N	5	5	5	5	5	5	2	2
		Mean	7.37	7.52	7.29	7.22	7.29	7.23	8.06	8.21
		Sdev	0.471	0.592	0.621	0.737	0.783	1.020	0.445	0.426
50 mg/kg/day										
	2562	2/1	6.96	6.91	6.96	6.84	7.00	6.65	Dead	
	2563	2/1	7.55	7.56	7.53	7.53	7.82	7.56	Dead	
	2576	2/1	8.30	8.64	8.47	8.53	8.57	8.26	Dead	
		N	3	3	3	3	3	3	0	0
		Mean	7.60	7.70	7.65	7.63	7.80	7.49	-	-
		Sdev	0.674	0.872	0.762	0.847	0.784	0.803	-	-
200 mg/kg/day										
	2561	3/1	7.32	7.41	7.31	7.23	7.53	7.03	Dead	
	2570	3/1	6.90	6.95	6.77	6.76	6.75	6.38	Dead	
	2567	3/1	8.73	8.52	8.36	8.43	8.29	7.99	Dead	
		N	3	3	3	3	3	3	0	0
		Mean	7.65	7.63	7.48	7.48	7.52	7.13	-	-
		Sdev	0.960	0.807	0.808	0.862	0.770	0.809	-	-
800 mg/kg/day										
	2564	4/1	7.53	7.57	6.96	6.58	6.51	6.43	Dead	
	2565	4/1	7.17	6.83	6.15	6.53	6.39	6.01	Dead	
	2569	4/1	7.87	7.96	7.81	8.01	7.96	7.70	Dead	
	2571	4/1	7.66	7.86	7.13	7.27	7.21	6.84	7.23	7.37
	2573	4/1	6.90	6.85	6.31	6.43	6.45	6.27	6.38	6.54
		N	5	5	5	5	5	5	2	2
		Mean	7.42	7.41	6.87	6.96	6.90	6.65	6.81	6.95
		Sdev	0.391	0.546	0.670	0.671	0.677	0.659	0.601	0.592

Note: " = Pretest phase; # = Test period

Appendix 4 ECG Examinations

Appendix 4
Individual Animal ECG Rhythm and Morphology Findings

Fexinidazole Study Number: 0505-2007

M a l e s

Animal Number	Dose	Clinical Signs	Study Day(s) Noted
2516	Vehicle	Respiratory Sinus Arrhythmia	Pretest phase -5<br>Test period 24
2518	Vehicle	Respiratory Sinus Arrhythmia	Pretest phase -5<br>Test period 24
2520	Vehicle	Respiratory Sinus Arrhythmia	Pretest phase -5<br>Test period 24
2527	Vehicle	Respiratory Sinus Arrhythmia	Pretest phase -5<br>Test period 24,38
2533	Vehicle	No ECG abnormalities detected	Pretest phase -5<br>Test period 24
		Respiratory Sinus Arrhythmia	Test period 38
2514	50mg/kg/day	No ECG abnormalities detected	Test period 24
		Respiratory Sinus Arrhythmia	Pretest phase -5
2521	50mg/kg/day	No ECG abnormalities detected	Test period 24
		Respiratory Sinus Arrhythmia	Pretest phase -5
2529	50mg/kg/day	No ECG abnormalities detected	Test period 24
		Respiratory Sinus Arrhythmia	Pretest phase -5
2515	200mg/kg/day	Respiratory Sinus Arrhythmia	Pretest phase -5<br>Test period 24
2523	200mg/kg/day	Respiratory Sinus Arrhythmia	Pretest phase -5<br>Test period 24
2526	200mg/kg/day	No ECG abnormalities detected	Test period 24

Appendix 4
Individual Animal ECG Rhythm and Morphology Findings

Fexinidazole — Study Number: 0505-2007

Males

Animal Number	Dose	Clinical Signs	Study Day(s) Noted
2526	200mg/kg/day	Respiratory Sinus Arrhythmia	Pretest phase -5
2517	800mg/kg/day	No ECG abnormalities detected<br>Respiratory Sinus Arrhythmia	Pretest phase -5<br>Test period 24
2519	800mg/kg/day	Respiratory Sinus Arrhythmia	Pretest phase -5<br>Test period 24
2525	800mg/kg/day	Respiratory Sinus Arrhythmia	Pretest phase -5<br>Test period 24
2528	800mg/kg/day	No ECG abnormalities detected	Pretest phase -5<br>Test period 24,38
2530	800mg/kg/day	No ECG abnormalities detected<br>Respiratory Sinus Arrhythmia	Test period 24<br>Pretest phase -5<br>Test period 38

Appendix 4
Individual Animal ECG Rhythm and Morphology Findings

Fexinidazole — Study Number: 0505-2007

F e m a l e

Animal Number	Dose	Clinical Signs	Study Day(s) Noted
2560	Vehicle	Respiratory Sinus Arrhythmia	Pretest phase -6<br>Test period 24
2568	Vehicle	Respiratory Sinus Arrhythmia	Pretest phase -6<br>Test period 24
2572	Vehicle	No ECG abnormalities detected<br>Respiratory Sinus Arrhythmia	Pretest phase -6<br>Test period 24
2575	Vehicle	No ECG abnormalities detected	Pretest phase -6<br>Test period 24,37
2577	Vehicle	No ECG abnormalities detected<br>Respiratory Sinus Arrhythmia	Test period 37<br>Pretest phase -6<br>Test period 24
2562	50mg/kg/day	No ECG abnormalities detected<br>Respiratory Sinus Arrhythmia	Pretest phase -6<br>Test period 24
2563	50mg/kg/day	No ECG abnormalities detected<br>Respiratory Sinus Arrhythmia	Test period 24<br>Pretest phase -6
2576	50mg/kg/day	No ECG abnormalities detected	Pretest phase -6<br>Test period 24
2561	200mg/kg/day	Respiratory Sinus Arrhythmia	Pretest phase -6<br>Test period 24
2570	200mg/kg/day	Respiratory Sinus Arrhythmia	Pretest phase -6<br>Test period 24
2567	200mg/kg/day	Respiratory Sinus Arrhythmia	Pretest phase -6

Appendix 4
Individual Animal ECG Rhythm and Morphology Findings

Fexinidazole Study Number: 0505-2007

F e m a l e

Animal Number	Dose	Clinical Signs	Study Day(s) Noted
2567	200mg/kg/day	Respiratory Sinus Arrhythmia	Test period 24
2564	800mg/kg/day	Respiratory Sinus Arrhythmia	Pretest phase -6<br>Test period 24
2565	800mg/kg/day	No ECG abnormalities detected	Pretest phase -6<br>Test period 24
2569	800mg/kg/day	No ECG abnormalities detected	Pretest phase -6<br>Test period 24
2571	800mg/kg/day	No ECG abnormalities detected	Pretest phase -6<br>Test period 24,37
2573	800mg/kg/day	Respiratory Sinus Arrhythmia	Pretest phase -6<br>Test period 24,37

Appendix 4
ECG Examinations Data

Fexinidazole | Study Number: 0505-2007

M a l e s		Animal No.			
Dosage		Day:	-5"	24#	38
mg/kg : 0.		Session:	S 1	S 1	S 1
HR	bpm	2516	97.	89.	
		2518	94.	103.	
		2520	87.	80.	
		2527	101.	125.	94.
		2533	87.	79.	72.
		Mean	93.	95.	83.
		SD	6.2	19.2	15.6
		N	5	5	2
RR	ms	2516	613.	673.	
		2518	634.	579.	
		2520	688.	749.	
		2527	594.	478.	636.
		2533	683.	751.	831.
		Mean	642.	646.	734.
		SD	41.8	117.3	137.9
		N	5	5	2
Pdur	ms	2516	39.	33.	
		2518	37.	33.	
		2520	37.	38.	
		2527	37.	31.	35.
		2533	41.	38.	42.
		Mean	38.	35.	39.
		SD	1.8	3.2	4.9
		N	5	5	2
PR	ms	2516	83.	84.	
		2518	88.	85.	
		2520	87.	74.	
		2527	81.	72.	80.
		2533	96.	91.	93.
		Mean	87.	81.	87.
		SD	5.8	8.0	9.2
		N	5	5	2

Note: " = Pretest phase; # = Test period

M a l e s	Animal No.			
Dosage	Day:	-5"	24#	38
mg/kg : 0.	Session:	S 1	S 1	S 1
QRS ms	2516	68.	56.	
	2518	43.	40.	
	2520	42.	47.	
	2527	43.	45.	48.
	2533	40.	45.	53.
	Mean	47.	47.	51.
	SD	11.7	5.9	3.5
	N	5	5	2
QT ms	2516	180.	197.	
	2518	178.	179.	
	2520	177.	196.	
	2527	181.	177.	204.
	2533	181.	195.	194.
	Mean	179.	189.	199.
	SD	1.8	9.9	7.1
	N	5	5	2
QTcF ms	2516	212.	225.	
	2518	207.	214.	
	2520	200.	216.	
	2527	215.	226.	237.
	2533	205.	214.	206.
	Mean	208.	219.	222.
	SD	5.7	5.7	21.7
	N	5	5	2
MEA degree	2516	34.	39.	
	2518	-21.	1.	
	2520	26.	54.	
	2527	44.	44.	57.
	2533	143.	120.	-19.
	Mean	45.	52.	19.
	SD	60.1	43.2	53.7
	N	5	5	2

Note: " = Pretest phase; # = Test period

M a l e s	Animal No.			
Dosage	Day:	-5"	24#	38
mg/kg : 0.	Session:	S 1	S 1	S 1
Pamp mV	2516	0.32	0.38	
	2518	0.32	0.40	
	2520	0.22	0.37	
	2527	0.24	0.25	0.26
	2533	0.40	0.34	0.34
	Mean	0.30	0.35	0.30
	SD	0.072	0.059	0.057
	N	5	5	2
Qamp mV	2516	-0.15	-0.20	
	2518	-0.54	-0.46	
	2520	-0.53	-0.41	
	2527	-0.33	-0.28	-0.36
	2533	-0.28	-0.25	-0.16
	Mean	-0.37	-0.32	-0.26
	SD	0.168	0.110	0.141
	N	5	5	2
Ramp mV	2516	2.68	3.13	
	2518	1.37	1.38	
	2520	2.46	2.73	
	2527	2.22	2.06	2.31
	2533	0.67	0.96	0.90
	Mean	1.88	2.05	1.61
	SD	0.839	0.904	0.997
	N	5	5	2
Samp mV	2516	-0.11	-0.06	
	2518	-0.26	-0.13	
	2520	-0.07	-0.09	
	2527	-0.21	-0.20	-0.26
	2533	-0.30	-0.32	-0.33
	Mean	-0.19	-0.16	-0.30
	SD	0.098	0.104	0.049
	N	5	5	2

Note: " = Pretest phase; # = Test period

M a l e s		Animal No.			
Dosage		Day:	-5"	24#	38
mg/kg : 0.		Session:	S 1	S 1	S 1
STd mV		2516	-0.10	-0.08	
		2518	-0.07	-0.01	
		2520	-0.04	-0.04	
		2527	-0.15	-0.07	-0.07
		2533	0.00	-0.04	0.02
	Mean		-0.07	-0.05	-0.03
	SD		0.057	0.028	0.064
	N		5	5	2
Tamp mV		2516	-0.16	-0.15	
		2518	0.68	0.62	
		2520	-0.07	0.24	
		2527	0.27	0.44	0.12
		2533	0.28	0.28	0.32
	Mean		0.20	0.29	0.22
	SD		0.333	0.286	0.141
	N		5	5	2

Note: " = Pretest phase; # = Test period

F e m a l e s	Animal No.	-5"	24#	38
Dosage	Day:	-5"	24#	38
mg/kg : 0.	Session:	S 1	S 1	S 1
HR bpm	2560	63.	70.	
	2568	94.	123.	
	2572	116.	106.	
	2575	87.	88.	76.
	2577	113.	106.	126.
	Mean	95.	99.	101.
	SD	21.5	20.2	35.4
	N	5	5	2
RR ms	2560	952.	853.	
	2568	634.	484.	
	2572	515.	565.	
	2575	688.	679.	784.
	2577	530.	563.	473.
	Mean	664.	629.	629.
	SD	176.5	143.3	219.9
	N	5	5	2
Pdur ms	2560	47.	47.	
	2568	41.	39.	
	2572	40.	44.	
	2575	38.	41.	44.
	2577	40.	39.	37.
	Mean	41.	42.	41.
	SD	3.4	3.5	4.9
	N	5	5	2
PR ms	2560	79.	79.	
	2568	75.	77.	
	2572	87.	82.	
	2575	108.	94.	116.
	2577	89.	83.	88.
	Mean	88.	83.	102.
	SD	12.8	6.6	19.8
	N	5	5	2

Note: " = Pretest phase; # = Test period

Females		Animal No.			
Dosage		Day:	-5"	24#	38
mg/kg : 0.		Session:	S 1	S 1	S 1
QRS	ms	2560	42.	44.	
		2568	40.	43.	
		2572	45.	46.	
		2575	45.	49.	47.
		2577	58.	57.	55.
		Mean	46.	48.	51.
		SD	7.0	5.6	5.7
		N	5	5	2
QT	ms	2560	189.	206.	
		2568	182.	169.	
		2572	175.	181.	
		2575	191.	185.	201.
		2577	188.	189.	186.
		Mean	185.	186.	194.
		SD	6.5	13.5	10.6
		N	5	5	2
QTcF	ms	2560	192.	217.	
		2568	212.	215.	
		2572	218.	219.	
		2575	216.	210.	218.
		2577	232.	228.	238.
		Mean	214.	218.	228.
		SD	14.3	6.7	14.4
		N	5	5	2
MEA	degree	2560	101.	114.	
		2568	41.	53.	
		2572	34.	18.	
		2575	37.	44.	41.
		2577	17.	37.	73.
		Mean	46.	53.	57.
		SD	32.1	36.3	22.6
		N	5	5	2

Note: " = Pretest phase; # = Test period

F e m a l e s	Animal No.			
Dosage	Day:	-5"	24#	38
mg/kg : 0.	Session:	S 1	S 1	S 1
Pamp mV	2560	0.36	0.39	
	2568	0.44	0.44	
	2572	0.33	0.50	
	2575	0.08	0.32	0.30
	2577	0.41	0.36	0.48
	Mean	0.32	0.40	0.39
	SD	0.143	0.070	0.127
	N	5	5	2
Qamp mV	2560	-0.57	-0.81	
	2568	-0.22	-0.29	
	2572	-0.37	-0.30	
	2575	-0.34	-0.35	-0.40
	2577	-0.43	-0.35	-0.38
	Mean	-0.39	-0.42	-0.39
	SD	0.128	0.220	0.014
	N	5	5	2
Ramp mV	2560	2.22	2.27	
	2568	2.37	2.64	
	2572	2.99	2.82	
	2575	2.56	3.23	2.92
	2577	2.10	2.12	2.35
	Mean	2.45	2.62	2.64
	SD	0.348	0.443	0.403
	N	5	5	2
Samp mV	2560	-0.19	-0.17	
	2568	-0.19	-0.16	
	2572	-0.20	-0.61	
	2575	-0.48	0.00	-0.16
	2577	-0.50	-0.38	-0.56
	Mean	-0.31	-0.26	-0.36
	SD	0.163	0.236	0.283
	N	5	5	2

Note: " = Pretest phase; # = Test period

F e m a l e s	Animal No.			
Dosage	Day:	-5"	24#	38
mg/kg : 0.	Session:	S 1	S 1	S 1
STd mV	2560	-0.05	-0.05	
	2568	0.00	-0.06	
	2572	-0.09	-0.05	
	2575	-0.08	-0.03	0.00
	2577	0.06	0.07	0.09
	Mean	-0.03	-0.02	0.05
	SD	0.062	0.054	0.064
	N	5	5	2
Tamp mV	2560	0.33	0.46	
	2568	0.53	0.61	
	2572	0.19	0.45	
	2575	0.04	0.44	0.36
	2577	-0.09	0.05	-0.45
	Mean	0.20	0.40	-0.04
	SD	0.243	0.209	0.573
	N	5	5	2

Note: " = Pretest phase; # = Test period

M a l e s		Animal No.			
Dosage		Day:	-5"	24#	38
mg/kg : 50.		Session:	S 1	S 1	S 1
HR	bpm	2514	67.	120.	
		2521	64.	58.	
		2529	114.	128.	
		Mean	82.	102.	-
		SD	28.0	38.3	-
		N	3	3	0
RR	ms	2514	885.	499.	
		2521	929.	1029.	
		2529	525.	466.	
		Mean	780.	665.	-
		SD	221.6	316.0	-
		N	3	3	0
Pdur	ms	2514	37.	37.	
		2521	38.	33.	
		2529	35.	33.	
		Mean	37.	34.	-
		SD	1.5	2.3	-
		N	3	3	0
PR	ms	2514	99.	85.	
		2521	81.	75.	
		2529	86.	87.	
		Mean	89.	82.	-
		SD	9.3	6.4	-
		N	3	3	0
QRS	ms	2514	49.	47.	
		2521	45.	48.	
		2529	44.	41.	
		Mean	46.	45.	-
		SD	2.6	3.8	-
		N	3	3	0
QT	ms	2514	193.	181.	
		2521	193.	203.	
		2529	173.	177.	

Note: " = Pretest phase; # = Test period

M a l e s	Animal No.			
Dosage	Day:	-5"	24#	38
mg/kg : 50.	Session:	S 1	S 1	S 1
	Mean	186.	187.	-
	SD	11.5	14.0	-
	N	3	3	0
QTcF ms	2514	201.	228.	
	2521	198.	201.	
	2529	214.	228.	
	Mean	204.	219.	-
	SD	8.6	15.4	-
	N	3	3	0
MEA degree	2514	54.	59.	
	2521	62.	63.	
	2529	38.	50.	
	Mean	51.	57.	-
	SD	12.2	6.7	-
	N	3	3	0
Pamp mV	2514	0.35	0.40	
	2521	0.33	0.40	
	2529	0.41	0.38	
	Mean	0.36	0.39	-
	SD	0.042	0.012	-
	N	3	3	0
Qamp mV	2514	-0.25	-0.30	
	2521	-0.33	-0.39	
	2529	-0.53	-0.52	
	Mean	-0.37	-0.40	-
	SD	0.144	0.111	-
	N	3	3	0
Ramp mV	2514	2.84	3.00	
	2521	3.53	3.95	
	2529	2.60	2.57	
	Mean	2.99	3.17	-
	SD	0.483	0.706	-
	N	3	3	0

Note: " = Pretest phase; # = Test period

Males Dosage mg/kg : 50.	Animal No. Day: Session:	-5" S 1	24# S 1	38 S 1
Samp mV	2514	-0.05	-0.10	
	2521	-0.26	-0.15	
	2529	-0.10	-0.14	
	Mean	-0.14	-0.13	-
	SD	0.110	0.026	-
	N	3	3	0
STd mV	2514	0.00	0.02	
	2521	-0.02	-0.13	
	2529	-0.11	-0.12	
	Mean	-0.04	-0.08	-
	SD	0.059	0.084	-
	N	3	3	0
Tamp mV	2514	0.26	-0.05	
	2521	-0.07	-0.16	
	2529	0.10	-0.38	
	Mean	0.10	-0.20	-
	SD	0.165	0.168	-
	N	3	3	0

Note: " = Pretest phase; # = Test period

Females Animal No. Dosage mg/kg : 50.		Day: / Session:	-5" S 1	24# S 1	38 S 1
HR	bpm	2562	113.	95.	
		2563	101.	114.	
		2576	133.	116.	
		Mean	116.	108.	-
		SD	16.2	11.6	-
		N	3	3	0
RR	ms	2562	531.	629.	
		2563	589.	522.	
		2576	448.	515.	
		Mean	523.	555.	-
		SD	70.9	63.9	-
		N	3	3	0
Pdur	ms	2562	35.	35.	
		2563	39.	37.	
		2576	36.	34.	
		Mean	37.	35.	-
		SD	2.1	1.5	-
		N	3	3	0
PR	ms	2562	83.	89.	
		2563	85.	88.	
		2576	80.	89.	
		Mean	83.	89.	-
		SD	2.5	0.6	-
		N	3	3	0
QRS	ms	2562	49.	49.	
		2563	45.	53.	
		2576	59.	41.	
		Mean	51.	48.	-
		SD	7.2	6.1	-
		N	3	3	0
QT	ms	2562	179.	195.	
		2563	181.	175.	
		2576	181.	182.	

Note: " = Pretest phase; # = Test period

F e m a l e s Animal No.				
Dosage	Day:	-5"	24#	38
mg/kg : 50.	Session:	S 1	S 1	S 1
	Mean	180.	184.	-
	SD	1.2	10.1	-
	N	3	3	0
QTcF ms	2562	221.	227.	
	2563	216.	217.	
	2576	236.	227.	
	Mean	224.	224.	-
	SD	10.6	5.8	-
	N	3	3	0
MEA degree	2562	55.	45.	
	2563	40.	61.	
	2576	36.	61.	
	Mean	44.	56.	-
	SD	10.0	9.2	-
	N	3	3	0
Pamp mV	2562	0.30	0.35	
	2563	0.32	0.38	
	2576	0.55	0.47	
	Mean	0.39	0.40	-
	SD	0.139	0.062	-
	N	3	3	0
Qamp mV	2562	-1.10	-0.83	
	2563	-0.58	-0.51	
	2576	-0.34	-0.36	
	Mean	-0.67	-0.57	-
	SD	0.389	0.240	-
	N	3	3	0
Ramp mV	2562	3.21	2.76	
	2563	1.58	1.87	
	2576	2.13	3.23	
	Mean	2.31	2.62	-
	SD	0.829	0.691	-
	N	3	3	0

Note: " = Pretest phase; # = Test period

Females Animal No.		Day:	-5"	24#	38
Dosage mg/kg : 50.		Session:	S 1	S 1	S 1
Samp mV		2562	-0.15	-0.03	
		2563	-0.27	-0.58	
		2576	-0.48	-0.52	
	Mean		-0.30	-0.38	-
	SD		0.167	0.302	-
	N		3	3	0
STd mV		2562	-0.19	-0.05	
		2563	-0.06	0.02	
		2576	0.14	-0.04	
	Mean		-0.04	-0.02	-
	SD		0.166	0.038	-
	N		3	3	0
Tamp mV		2562	-0.58	-0.30	
		2563	-0.30	0.32	
		2576	0.23	0.08	
	Mean		-0.22	0.03	-
	SD		0.411	0.313	-
	N		3	3	0

Note: " = Pretest phase; # = Test period

Fexinidazole Study Number: 0505-2007

M a l e s		Animal No.			
Dosage		Day:	-5"	24#	38
mg/kg : 200.		Session:	S 1	S 1	S 1
HR	bpm	2515	63.	71.	
		2523	77.	81.	
		2526	102.	139.	
		Mean	81.	97.	-
		SD	19.8	36.7	-
		N	3	3	0
RR	ms	2515	951.	839.	
		2523	773.	738.	
		2526	583.	430.	
		Mean	769.	669.	-
		SD	184.0	213.1	-
		N	3	3	0
Pdur	ms	2515	35.	35.	
		2523	42.	41.	
		2526	34.	43.	
		Mean	37.	40.	-
		SD	4.4	4.2	-
		N	3	3	0
PR	ms	2515	95.	88.	
		2523	98.	84.	
		2526	99.	99.	
		Mean	97.	90.	-
		SD	2.1	7.8	-
		N	3	3	0
QRS	ms	2515	60.	51.	
		2523	61.	61.	
		2526	45.	46.	
		Mean	55.	53.	-
		SD	9.0	7.6	-
		N	3	3	0
QT	ms	2515	195.	197.	
		2523	201.	204.	
		2526	175.	187.	

Note: " = Pretest phase; # = Test period

M a l e s	Animal No.			
Dosage	Day:	-5"	24#	38
mg/kg : 200.	Session:	S 1	S 1	S 1
	Mean	190.	196.	-
	SD	13.6	8.5	-
	N	3	3	0
QTcF ms	2515	198.	209.	
	2523	219.	226.	
	2526	209.	247.	
	Mean	209.	227.	-
	SD	10.3	19.2	-
	N	3	3	0
MEA degree	2515	46.	59.	
	2523	70.	63.	
	2526	62.	62.	
	Mean	59.	61.	-
	SD	12.2	2.1	-
	N	3	3	0
Pamp mV	2515	0.37	0.34	
	2523	0.39	0.41	
	2526	0.28	0.39	
	Mean	0.35	0.38	-
	SD	0.059	0.036	-
	N	3	3	0
Qamp mV	2515	-0.76	-0.55	
	2523	-0.47	-1.12	
	2526	-0.28	-0.21	
	Mean	-0.50	-0.63	-
	SD	0.242	0.460	-
	N	3	3	0
Ramp mV	2515	3.42	3.58	
	2523	2.53	3.10	
	2526	3.08	3.03	
	Mean	3.01	3.24	-
	SD	0.449	0.299	-
	N	3	3	0

Note: " = Pretest phase; # = Test period

M a l e s Dosage mg/kg : 200.	Animal No. / Day: / Session:	-5" S 1	24# S 1	38 S 1
Samp mV	2515	-0.07	-0.08	
	2523	-0.07	-0.07	
	2526	-0.17	-0.32	
	Mean	-0.10	-0.16	-
	SD	0.058	0.142	-
	N	3	3	0
STd mV	2515	-0.07	-0.09	
	2523	-0.07	-0.04	
	2526	-0.17	-0.17	
	Mean	-0.10	-0.10	-
	SD	0.058	0.066	-
	N	3	3	0
Tamp mV	2515	0.22	-0.13	
	2523	0.36	0.40	
	2526	-0.08	-0.22	
	Mean	0.17	0.02	-
	SD	0.225	0.335	-
	N	3	3	0

Note: " = Pretest phase; # = Test period

F e m a l e s		Animal No.			
Dosage		Day:	-5"	24#	38
mg/kg : 200.		Session:	S 1	S 1	S 1
HR	bpm	2561	83.	95.	
		2570	116.	120.	
		2567	81.	120.	
		Mean	93.	112.	-
		SD	19.7	14.4	-
		N	3	3	0
RR	ms	2561	719.	631.	
		2570	515.	499.	
		2567	741.	500.	
		Mean	658.	543.	-
		SD	124.6	75.9	-
		N	3	3	0
Pdur	ms	2561	37.	39.	
		2570	40.	41.	
		2567	37.	40.	
		Mean	38.	40.	-
		SD	1.7	1.0	-
		N	3	3	0
PR	ms	2561	85.	84.	
		2570	95.	87.	
		2567	95.	89.	
		Mean	92.	87.	-
		SD	5.8	2.5	-
		N	3	3	0
QRS	ms	2561	46.	49.	
		2570	45.	53.	
		2567	55.	56.	
		Mean	49.	53.	-
		SD	5.5	3.5	-
		N	3	3	0
QT	ms	2561	189.	197.	
		2570	171.	183.	
		2567	188.	195.	

Note: " = Pretest phase; # = Test period

F e m a l e s Animal No.				
Dosage Day:		-5"	24#	38
mg/kg : 200. Session:		S 1	S 1	S 1
	Mean	183.	192.	-
	SD	10.1	7.6	-
	N	3	3	0
QTcF ms	2561	211.	229.	
	2570	213.	230.	
	2567	208.	245.	
	Mean	210.	235.	-
	SD	2.7	8.9	-
	N	3	3	0
MEA degree	2561	63.	60.	
	2570	54.	62.	
	2567	10.	28.	
	Mean	42.	50.	-
	SD	28.4	19.1	-
	N	3	3	0
Pamp mV	2561	0.39	0.30	
	2570	0.54	0.63	
	2567	0.39	0.51	
	Mean	0.44	0.48	-
	SD	0.087	0.167	-
	N	3	3	0
Qamp mV	2561	-0.85	-0.71	
	2570	-0.70	-0.80	
	2567	-0.53	-0.53	
	Mean	-0.69	-0.68	-
	SD	0.160	0.137	-
	N	3	3	0
Ramp mV	2561	2.92	2.72	
	2570	3.16	3.98	
	2567	2.06	2.46	
	Mean	2.71	3.05	-
	SD	0.578	0.813	-
	N	3	3	0

Note: " = Pretest phase; # = Test period

F e m a l e s	Animal No.			
Dosage	Day:	-5"	24#	38
mg/kg : 200.	Session:	S 1	S 1	S 1
Samp mV	2561	-0.18	-0.18	
	2570	-0.03	-0.05	
	2567	-0.48	-0.54	
	Mean	-0.23	-0.26	-
	SD	0.229	0.254	-
	N	3	3	0
STd mV	2561	-0.08	-0.08	
	2570	-0.04	-0.06	
	2567	-0.03	-0.11	
	Mean	-0.05	-0.08	-
	SD	0.026	0.025	-
	N	3	3	0
Tamp mV	2561	0.34	0.32	
	2570	0.61	0.47	
	2567	0.71	0.59	
	Mean	0.55	0.46	-
	SD	0.191	0.135	-
	N	3	3	0

Note: " = Pretest phase; # = Test period

M a l e s		Animal No.			
Dosage		Day:	-5"	24#	38
mg/kg : 800.		Session:	S 1	S 1	S 1
HR	bpm	2517	126.	88.	
		2519	80.	85.	
		2525	60.	104.	
		2528	108.	116.	116.
		2530	101.	107.	66.
		Mean	95.	100.	91.
		SD	25.6	13.1	35.4
		N	5	5	2
RR	ms	2517	474.	680.	
		2519	749.	706.	
		2525	991.	575.	
		2528	551.	514.	514.
		2530	593.	557.	901.
		Mean	672.	606.	708.
		SD	204.8	82.6	273.7
		N	5	5	2
Pdur	ms	2517	42.	39.	
		2519	37.	34.	
		2525	36.	35.	
		2528	43.	43.	44.
		2530	39.	35.	39.
		Mean	39.	37.	42.
		SD	3.0	3.8	3.5
		N	5	5	2
PR	ms	2517	81.	90.	
		2519	75.	77.	
		2525	90.	83.	
		2528	89.	90.	99.
		2530	97.	90.	103.
		Mean	86.	86.	101.
		SD	8.5	5.9	2.8
		N	5	5	2

Note: " = Pretest phase; # = Test period

M a l e s	Animal No.			
Dosage	Day:	-5"	24#	38
mg/kg : 800.	Session:	S 1	S 1	S 1
QRS ms	2517	44.	44.	
	2519	43.	46.	
	2525	43.	41.	
	2528	45.	44.	43.
	2530	46.	45.	59.
	Mean	44.	44.	51.
	SD	1.3	1.9	11.3
	N	5	5	2
QT ms	2517	175.	204.	
	2519	179.	201.	
	2525	181.	185.	
	2528	189.	203.	192.
	2530	171.	193.	213.
	Mean	179.	197.	203.
	SD	6.8	8.1	14.8
	N	5	5	2
QTcF ms	2517	224.	232.	
	2519	197.	225.	
	2525	182.	222.	
	2528	230.	253.	239.
	2530	203.	234.	220.
	Mean	207.	233.	230.
	SD	19.9	12.0	13.2
	N	5	5	2
MEA degree	2517	30.	41.	
	2519	34.	57.	
	2525	58.	47.	
	2528	40.	71.	78.
	2530	30.	99.	89.
	Mean	38.	63.	84.
	SD	11.7	23.1	7.8
	N	5	5	2

Note: " = Pretest phase; # = Test period

Fexinidazole Study Number: 0505-2007

M a l e s Dosage mg/kg : 800.	Animal No. Day: Session:	-5" S 1	24# S 1	38 S 1
Pamp mV	2517	0.53	0.54	
	2519	0.46	0.45	
	2525	0.36	0.38	
	2528	0.42	0.33	0.44
	2530	0.29	0.35	0.33
	Mean	0.41	0.41	0.39
	SD	0.092	0.086	0.078
	N	5	5	2
Qamp mV	2517	-0.24	-0.16	
	2519	-0.47	-0.44	
	2525	-0.73	-0.64	
	2528	-0.41	-0.55	-0.21
	2530	-0.56	-0.97	-0.47
	Mean	-0.48	-0.55	-0.34
	SD	0.181	0.295	0.184
	N	5	5	2
Ramp mV	2517	1.97	1.97	
	2519	2.41	2.64	
	2525	2.86	2.75	
	2528	2.02	1.95	2.46
	2530	1.59	1.95	1.99
	Mean	2.17	2.25	2.23
	SD	0.483	0.406	0.332
	N	5	5	2
Samp mV	2517	-0.30	-0.43	
	2519	-0.15	-0.27	
	2525	-0.13	-0.09	
	2528	-0.12	-0.18	-0.18
	2530	-0.10	-0.17	-0.16
	Mean	-0.16	-0.23	-0.17
	SD	0.080	0.130	0.014
	N	5	5	2

Note: " = Pretest phase; # = Test period

M a l e s		Animal No.			
Dosage		Day:	-5"	24#	38
mg/kg : 800.		Session:	S 1	S 1	S 1
STd mV		2517	-0.05	0.00	
		2519	-0.04	-0.08	
		2525	-0.04	0.00	
		2528	-0.10	-0.18	-0.06
		2530	-0.07	-0.11	-0.08
	Mean		-0.06	-0.07	-0.07
	SD		0.025	0.077	0.014
	N		5	5	2
Tamp mV		2517	0.29	0.20	
		2519	0.34	-0.27	
		2525	-0.47	-0.09	
		2528	0.33	-0.13	0.46
		2530	0.26	-0.39	0.08
	Mean		0.15	-0.14	0.27
	SD		0.348	0.222	0.269
	N		5	5	2

Note: " = Pretest phase; # = Test period

F e m a l e s	Animal No.			
Dosage	Day:	-5"	24#	38
mg/kg : 800.	Session:	S 1	S 1	S 1
HR bpm	2564	89.	107.	
	2565	87.	84.	
	2569	108.	151.	
	2571	136.	134.	122.
	2573	92.	75.	82.
	Mean	102.	110.	102.
	SD	20.5	32.3	28.3
	N	5	5	2
RR ms	2564	667.	558.	
	2565	687.	706.	
	2569	552.	395.	
	2571	441.	446.	489.
	2573	648.	796.	728.
	Mean	599.	580.	609.
	SD	102.4	169.7	169.0
	N	5	5	2
Pdur ms	2564	33.	39.	
	2565	37.	36.	
	2569	35.	45.	
	2571	35.	35.	38.
	2573	39.	35.	42.
	Mean	36.	38.	40.
	SD	2.3	4.2	2.8
	N	5	5	2
PR ms	2564	105.	103.	
	2565	89.	83.	
	2569	77.	85.	
	2571	88.	79.	91.
	2573	78.	79.	88.
	Mean	87.	86.	90.
	SD	11.3	10.0	2.1
	N	5	5	2

Note: " = Pretest phase; # = Test period

Females		Animal No.	-5"	24#	38
Dosage		Day:	-5"	24#	38
mg/kg : 800.		Session:	S 1	S 1	S 1
QRS	ms	2564	49.	49.	
		2565	39.	44.	
		2569	59.	59.	
		2571	62.	63.	54.
		2573	40.	49.	43.
		Mean	50.	53.	49.
		SD	10.6	7.9	7.8
		N	5	5	2
QT	ms	2564	180.	191.	
		2565	177.	196.	
		2569	182.	168.	
		2571	166.	189.	196.
		2573	180.	214.	211.
		Mean	177.	192.	204.
		SD	6.4	16.5	10.6
		N	5	5	2
QTcF	ms	2564	206.	232.	
		2565	200.	220.	
		2569	221.	228.	
		2571	217.	247.	248.
		2573	208.	231.	234.
		Mean	211.	231.	241.
		SD	8.7	9.7	9.8
		N	5	5	2
MEA	degree	2564	56.	57.	
		2565	7.	12.	
		2569	59.	64.	
		2571	57.	63.	65.
		2573	71.	71.	74.
		Mean	50.	53.	70.
		SD	24.8	23.7	6.4
		N	5	5	2

Note: " = Pretest phase; # = Test period

F e m a l e s Animal No.				
Dosage Day:		-5"	24#	38
mg/kg : 800. Session:		S 1	S 1	S 1
Pamp mV	2564	0.30	0.20	
	2565	0.32	0.46	
	2569	0.50	0.64	
	2571	0.43	0.37	0.46
	2573	0.29	0.35	0.26
	Mean	0.37	0.40	0.36
	SD	0.093	0.162	0.141
	N	5	5	2
Qamp mV	2564	-0.20	-0.06	
	2565	-0.38	-0.48	
	2569	-0.76	-0.60	
	2571	-0.99	-0.89	-0.65
	2573	-0.23	-0.29	-0.27
	Mean	-0.51	-0.46	-0.46
	SD	0.348	0.314	0.269
	N	5	5	2
Ramp mV	2564	2.63	2.23	
	2565	1.52	1.62	
	2569	4.36	3.98	
	2571	4.53	4.30	3.90
	2573	1.96	2.29	2.06
	Mean	3.00	2.88	2.98
	SD	1.378	1.182	1.301
	N	5	5	2
Samp mV	2564	-0.13	0.00	
	2565	-0.17	-0.19	
	2569	-0.05	-0.18	
	2571	-0.19	-0.11	-0.06
	2573	-0.06	-0.13	-0.12
	Mean	-0.12	-0.12	-0.09
	SD	0.063	0.076	0.042
	N	5	5	2

Note: " = Pretest phase; # = Test period

F e m a l e s	Animal No.			
Dosage	Day:	-5"	24#	38
mg/kg : 800.	Session:	S 1	S 1	S 1
STd mV	2564	-0.13	0.06	
	2565	-0.13	-0.11	
	2569	-0.04	-0.12	
	2571	-0.06	-0.07	-0.05
	2573	0.01	-0.06	0.05
	Mean	-0.07	-0.06	0.00
	SD	0.060	0.072	0.071
	N	5	5	2
Tamp mV	2564	-0.35	0.11	
	2565	0.32	-0.07	
	2569	-0.04	-0.29	
	2571	0.26	0.27	0.23
	2573	0.04	-0.14	0.13
	Mean	0.05	-0.02	0.18
	SD	0.267	0.218	0.071
	N	5	5	2

Note: " = Pretest phase; # = Test period

Appendix 5 Ophthalmoscopic Examination

Appendix 5
Individual Animal Ophthalmoscopic Observations

Fexinidazole Study Number: 0505-2007

Males

Animal Number	Dose	Clinical Signs	Study Day(s) Noted
2516	Vehicle	NORMAL	Pretest phase -5<br>Test period 24
2518	Vehicle	NORMAL	Pretest phase -5<br>Test period 24
2520	Vehicle	NORMAL	Pretest phase -5<br>Test period 24
2527	Vehicle	NORMAL	Pretest phase -5<br>Test period 24,38
2533	Vehicle	NORMAL	Pretest phase -5<br>Test period 24,38
2514	50mg/kg/day	NORMAL	Pretest phase -5<br>Test period 24
2521	50mg/kg/day	NORMAL	Pretest phase -5<br>Test period 24
2529	50mg/kg/day	NORMAL	Pretest phase -5<br>Test period 24
2515	200mg/kg/day	NORMAL	Pretest phase -5<br>Test period 24
2523	200mg/kg/day	NORMAL	Pretest phase -5<br>Test period 24
2526	200mg/kg/day	NORMAL	Pretest phase -5<br>Test period 24

Appendix 5
Individual Animal Ophthalmoscopic Observations

Fexinidazole Study Number: 0505-2007

Males

Animal Number	Dose	Clinical Signs	Study Day(s) Noted
2517	800mg/kg/day	NORMAL	Pretest phase -5<br>Test period 24
2519	800mg/kg/day	NORMAL	Pretest phase -5<br>Test period 24
2525	800mg/kg/day	NORMAL	Pretest phase -5<br>Test period 24
2528	800mg/kg/day	NORMAL	Pretest phase -5<br>Test period 24,38
2530	800mg/kg/day	NORMAL	Pretest phase -5<br>Test period 24,38

Appendix 5
Individual Animal Ophthalmoscopic Observations

Fexinidazole | Study Number: 0505-2007

F e m a l e

Animal Number	Dose	Clinical Signs	Study Day(s) Noted
2560	Vehicle	NORMAL	Pretest phase -6<br>Test period 24
2568	Vehicle	NORMAL	Pretest phase -6<br>Test period 24
2572	Vehicle	NORMAL	Pretest phase -6<br>Test period 24
2575	Vehicle	NORMAL<br>opacity area/s, monolateral	Pretest phase -6<br>Test period 24,37
2577	Vehicle	NORMAL	Pretest phase -6<br>Test period 24,37
2562	50mg/kg/day	NORMAL	Pretest phase -6<br>Test period 24
2563	50mg/kg/day	NORMAL	Pretest phase -6<br>Test period 24
2576	50mg/kg/day	NORMAL	Pretest phase -6<br>Test period 24
2561	200mg/kg/day	NORMAL	Pretest phase -6<br>Test period 24
2570	200mg/kg/day	NORMAL	Pretest phase -6<br>Test period 24
2567	200mg/kg/day	NORMAL	Pretest phase -6<br>Test period 24

Appendix 5
Individual Animal Ophthalmoscopic Observations

Fexinidazole Study Number: 0505-2007

F e m a l e

Animal Number	Dose	Clinical Signs	Study Day(s) Noted
2564	800mg/kg/day	NORMAL	Pretest phase -6<br>Test period 24
2565	800mg/kg/day	NORMAL	Pretest phase -6<br>Test period 24
2569	800mg/kg/day	NORMAL	Pretest phase -6<br>Test period 24
2571	800mg/kg/day	NORMAL	Pretest phase -6<br>Test period 24,37
2573	800mg/kg/day	NORMAL	Pretest phase -6<br>Test period 24,37

Appendix 6 Hematology

Appendix 6
Day -7 Hematology Data
Pretest phase

Session 1 (Scheduled)
Fexinidazole
Study Number: 0505-2007

Males

Dose Level	Animal Number	Group/ Subgroup	Study Day	RBC 10^6/mcL	HGB g/dL	HCT %	MCV fL	MCH pg	MCHC g/dL	RDW %	HDW g/dL
Vehicle											
	2516	1/1	-7	6.94	15.7	45.1	65.0	22.6	34.7	12.8	1.58
	2518	1/1	-7	6.55	15.5	43.4	66.2	23.6	35.7	13.1	1.54
	2520	1/1	-7	6.38	14.0	40.3	63.2	21.9	34.7	13.0	1.60
	2527	1/1	-7	6.79	15.5	43.9	64.6	22.8	35.3	14.1	1.67
	2533	1/1	-7	6.07	14.0	39.4	64.9	23.0	35.4	12.9	1.58
			N	5	5	5	5	5	5	5	5
			Mean	6.55	14.9	42.4	64.8	22.8	35.2	13.2	1.59
			Sdev	0.342	0.86	2.45	1.07	0.62	0.44	0.53	0.048
50 mg/kg/day											
	2514	2/1	-7	6.51	15.1	42.9	65.9	23.1	35.1	13.0	1.69
	2521	2/1	-7	7.41	17.3	48.8	65.8	23.3	35.4	13.4	1.66
	2529	2/1	-7	7.33	16.1	46.4	63.4	22.0	34.7	13.0	1.61
			N	3	3	3	3	3	3	3	3
			Mean	7.08	16.2	46.0	65.0	22.8	35.1	13.1	1.65
			Sdev	0.498	1.10	2.97	1.42	0.70	0.35	0.23	0.040
200 mg/kg/day											
	2515	3/1	-7	7.30	16.4	47.0	64.4	22.5	34.9	13.2	1.68
	2523	3/1	-7	6.01	13.8	39.3	65.3	22.9	35.1	12.8	1.59
	2526	3/1	-7	6.91	15.7	45.1	65.3	22.7	34.8	13.3	1.51
			N	3	3	3	3	3	3	3	3
			Mean	6.74	15.3	43.8	65.0	22.7	34.9	13.1	1.59
			Sdev	0.662	1.35	4.01	0.52	0.20	0.15	0.26	0.085
800 mg/kg/day											
	2517	4/1	-7	6.32	14.6	41.0	64.9	23.0	35.5	13.2	1.74
	2519	4/1	-7	6.96	15.8	45.1	64.8	22.8	35.2	12.7	1.52
	2525	4/1	-7	7.06	16.1	46.1	65.4	22.9	35.0	13.0	1.66
	2528	4/1	-7	6.65	14.9	42.8	64.4	22.4	34.7	13.4	1.55
	2530	4/1	-7	5.88	13.7	39.2	66.7	23.4	35.0	13.0	1.63
			N	5	5	5	5	5	5	5	5
			Mean	6.57	15.0	42.8	65.2	22.9	35.1	13.1	1.62
			Sdev	0.484	0.96	2.84	0.89	0.36	0.29	0.26	0.088

RBC - RED BLOOD CELLS
HGB - HEMOGLOBIN
HCT - HEMATOCRIT
MCV - MEAN CORPUSCULAR VOLUME
MCH - MEAN CORPUSCULAR HEMOGLOBIN
MCHC - MEAN CORPUSCULAR HGB CONC.
RDW - RED CELL DISTRIBUTION WIDTH
HDW - HEMOGLOBIN DISTRIB. WIDTH

Appendix 6
Day -7 Hematology Data
Pretest phase

Session 1 (Scheduled)
Fexinidazole

Study Number: 0505-2007

M a l e s

Dose Level	Animal Number	Group/ Subgroup	Study Day	R %	RAB 10^9/L	MCVr fL	CHCM g/dL	CHr pg	PLT 10^3/mcL	MPV fL	PDW %
Vehicle											
	2516	1/1	-7	1.1	74.2	86.3	30.0	25.7	213.	12.0	61.1
	2518	1/1	-7	0.6	38.5	86.3	30.8	26.5	244.	10.6	58.9
	2520	1/1	-7	0.9	54.6	84.8	30.3	25.6	450.	10.9	65.4
	2527	1/1	-7	0.7	48.5	88.3	29.7	26.2	387.	12.0	63.8
	2533	1/1	-7	0.4	25.2	81.4	31.7	25.6	366.	9.9	63.0
			N	5	5	5	5	5	5	5	5
			Mean	0.7	48.2	85.4	30.5	25.9	332.	11.1	62.4
			Sdev	0.27	18.30	2.57	0.78	0.41	100.0	0.91	2.51
50 mg/kg/day											
	2514	2/1	-7	1.3	87.8	89.8	29.0	26.0	205.	12.5	58.7
	2521	2/1	-7	0.9	68.9	87.6	30.9	27.0	191.	11.7	66.5
	2529	2/1	-7	1.0	71.5	84.9	30.1	25.5	290.	11.2	65.8
			N	3	3	3	3	3	3	3	3
			Mean	1.1	76.1	87.4	30.0	26.2	229.	11.8	63.7
			Sdev	0.21	10.24	2.45	0.95	0.76	53.6	0.66	4.32
200 mg/kg/day											
	2515	3/1	-7	0.9	64.0	87.2	29.7	25.8	246.	12.7	62.5
	2523	3/1	-7	0.6	34.9	82.2	30.8	25.2	414.	9.1	55.1
	2526	3/1	-7	0.6	39.7	88.5	30.6	27.0	307.	12.3	64.4
			N	3	3	3	3	3	3	3	3
			Mean	0.7	46.2	86.0	30.4	26.0	322.	11.4	60.7
			Sdev	0.17	15.60	3.33	0.59	0.92	85.0	1.97	4.91
800 mg/kg/day											
	2517	4/1	-7	0.9	56.2	84.4	30.7	25.9	409.	9.5	58.2
	2519	4/1	-7	0.5	36.3	82.5	30.8	25.4	212.	10.9	66.1
	2525	4/1	-7	0.9	66.0	87.6	30.6	26.7	239.	13.3	65.0
	2528	4/1	-7	0.6	36.6	88.8	30.2	26.7	260.	9.5	63.4
	2530	4/1	-7	0.3	16.2	83.7	31.9	26.4	379.	11.8	60.6
			N	5	5	5	5	5	5	5	5
			Mean	0.6	42.3	85.4	30.8	26.2	300.	11.0	62.7
			Sdev	0.26	19.39	2.68	0.63	0.56	88.3	1.62	3.24

R - RETICULOCYTES
RAB - RETICULOCYTES ABS
MCVr - MEAN CORPUSCOLAR VOL. RETIC.
CHCM - MEAN HEMOGLOBIN CONC. RETIC.
CHr - CELLULAR HEMOGLOBIN RETIC.
PLT - PLATELETS
MPV - MEAN PLATELET VOLUME
PDW - PLATELET DISTRIBUTION WIDTH

Appendix 6
Day -7 Hematology Data
Pretest phase

Session 1 (Scheduled)
Fexinidazole

Study Number: 0505-2007

Males

Dose Level	Animal Number	Group/ Subgroup	Study Day	PCT %	WBC 10^3/mcL	NAB 10^3/mcL	LYAB 10^3/mcL	MAB 10^3/mcL	EAB 10^3/mcL	BAB 10^3/mcL
Vehicle										
	2516	1/1	-7	0.26	9.51	5.14	3.36	0.36	0.55	0.07
	2518	1/1	-7	0.26	8.84	5.47	2.57	0.43	0.15	0.14
	2520	1/1	-7	0.49	6.92	3.42	2.81	0.35	0.26	0.05
	2527	1/1	-7	0.47	10.14	5.65	3.44	0.61	0.29	0.09
	2533	1/1	-7	0.36	9.22	5.38	3.17	0.42	0.16	0.05
			N	5	5	5	5	5	5	5
			Mean	0.37	8.93	5.01	3.07	0.43	0.28	0.08
			Sdev	0.110	1.218	0.909	0.370	0.105	0.162	0.037
50 mg/kg/day										
	2514	2/1	-7	0.26	9.85	5.49	3.44	0.39	0.38	0.11
	2521	2/1	-7	0.22	9.19	5.51	2.64	0.56	0.30	0.13
	2529	2/1	-7	0.33	6.47	3.23	2.58	0.29	0.11	0.19
			N	3	3	3	3	3	3	3
			Mean	0.27	8.50	4.74	2.89	0.41	0.26	0.14
			Sdev	0.056	1.792	1.311	0.480	0.137	0.139	0.042
200 mg/kg/day										
	2515	3/1	-7	0.31	8.35	5.14	2.62	0.26	0.19	0.10
	2523	3/1	-7	0.38	10.34	6.56	2.72	0.67	0.26	0.10
	2526	3/1	-7	0.38	12.33	7.23	3.72	0.98	0.18	0.14
			N	3	3	3	3	3	3	3
			Mean	0.36	10.34	6.31	3.02	0.64	0.21	0.11
			Sdev	0.040	1.990	1.067	0.608	0.361	0.044	0.023
800 mg/kg/day										
	2517	4/1	-7	0.39	6.48	3.87	2.07	0.37	0.07	0.07
	2519	4/1	-7	0.23	7.99	4.06	2.64	0.54	0.58	0.14
	2525	4/1	-7	0.32	10.19	6.82	2.75	0.35	0.18	0.08
	2528	4/1	-7	0.25	9.78	5.12	3.54	0.51	0.45	0.11
	2530	4/1	-7	0.45	7.08	3.62	2.83	0.34	0.14	0.08
			N	5	5	5	5	5	5	5
			Mean	0.33	8.30	4.70	2.77	0.42	0.28	0.10
			Sdev	0.093	1.632	1.317	0.525	0.095	0.219	0.029

PCT - PLATELET HEMATOCRIT
WBC - WHITE BLOOD CELLS
NAB - NEUTROPHILS ABS
LYAB - LYMPHOCYTES ABS
MAB - MONOCYTES ABS
EAB - EOSINOPHILS ABS
BAB - BASOPHILS ABS

Appendix 6
Day -7 Hematology Data
Session 1 (Scheduled) Pretest phase
Fexinidazole Study Number: 0505-2007

Males

Dose Level	Animal Number	Group/ Subgroup	Study Day	LUAB 10^3/mcL	N %	LY %	M %	E %	B %	LU %
Vehicle										
	2516	1/1	-7	0.03	54.0	35.3	3.7	5.8	0.7	0.4
	2518	1/1	-7	0.07	61.9	29.1	4.9	1.7	1.6	0.8
	2520	1/1	-7	0.03	49.4	40.6	5.0	3.8	0.8	0.4
	2527	1/1	-7	0.04	55.8	34.0	6.1	2.9	0.9	0.4
	2533	1/1	-7	0.04	58.4	34.3	4.6	1.8	0.6	0.4
			N	5	5	5	5	5	5	5
			Mean	0.04	55.9	34.7	4.9	3.2	0.9	0.5
			Sdev	0.016	4.69	4.10	0.86	1.69	0.40	0.18
50 mg/kg/day										
	2514	2/1	-7	0.04	55.8	35.0	4.0	3.8	1.1	0.4
	2521	2/1	-7	0.06	59.9	28.7	6.0	3.3	1.4	0.6
	2529	2/1	-7	0.07	49.9	39.8	4.5	1.7	2.9	1.1
			N	3	3	3	3	3	3	3
			Mean	0.06	55.2	34.5	4.8	2.9	1.8	0.7
			Sdev	0.015	5.03	5.57	1.04	1.10	0.96	0.36
200 mg/kg/day										
	2515	3/1	-7	0.05	61.5	31.4	3.1	2.3	1.2	0.6
	2523	3/1	-7	0.04	63.4	26.3	6.4	2.5	0.9	0.4
	2526	3/1	-7	0.08	58.7	30.2	7.9	1.5	1.1	0.6
			N	3	3	3	3	3	3	3
			Mean	0.06	61.2	29.3	5.8	2.1	1.1	0.5
			Sdev	0.021	2.36	2.67	2.46	0.53	0.15	0.12
800 mg/kg/day										
	2517	4/1	-7	0.02	59.8	32.0	5.7	1.1	1.1	0.4
	2519	4/1	-7	0.04	50.8	33.0	6.7	7.3	1.7	0.5
	2525	4/1	-7	0.02	66.9	27.0	3.4	1.7	0.8	0.2
	2528	4/1	-7	0.05	52.4	36.2	5.2	4.6	1.1	0.5
	2530	4/1	-7	0.07	51.2	40.0	4.7	2.0	1.1	0.9
			N	5	5	5	5	5	5	5
			Mean	0.04	56.2	33.6	5.1	3.3	1.2	0.5
			Sdev	0.021	7.00	4.85	1.22	2.59	0.33	0.25

LUAB - LARGE UNSTAINED CELLS ABS
N - NEUTROPHILS %
LY - LYMPHOCITES %
M - MONOCYTES %
E - EOSINOPHILS %
B - BASOPHILS %
LU - LARGE UNSTAINED CELLS %

Appendix 6
Day -7 Hematology Data
Pretest phase

Session 1 (Scheduled)
Fexinidazole

Study Number: 0505-2007

Females

Dose Level	Animal Number	Group/ Subgroup	Study Day	RBC 10^6/mcL	HGB g/dL	HCT %	MCV fL	MCH pg	MCHC g/dL	RDW %	HDW g/dL
Vehicle											
	2560	1/1	-7	6.45	14.8	43.3	67.1	22.9	34.1	11.0	1.65
	2568	1/1	-7	6.54	14.7	44.2	67.5	22.5	33.2	11.1	1.65
	2572	1/1	-7	6.16	13.9	41.4	67.1	22.6	33.7	11.9	1.72
	2575	1/1	-7	6.99	16.1	47.2	67.5	23.0	34.1	10.6	1.71
	2577	1/1	-7	6.57	14.8	43.6	66.3	22.5	33.9	11.4	1.75
			N	5	5	5	5	5	5	5	5
			Mean	6.54	14.9	43.9	67.1	22.7	33.8	11.2	1.70
			Sdev	0.298	0.79	2.10	0.49	0.23	0.37	0.48	0.044
50 mg/kg/day											
	2562	2/1	-7	7.94	18.2	53.1	66.9	22.9	34.3	11.7	1.73
	2563	2/1	-7	7.13	16.6	48.9	68.6	23.3	34.0	11.5	1.69
	2576	2/1	-7	7.30	16.9	49.6	68.0	23.1	34.0	11.0	1.70
			N	3	3	3	3	3	3	3	3
			Mean	7.46	17.2	50.5	67.8	23.1	34.1	11.4	1.71
			Sdev	0.427	0.85	2.25	0.86	0.20	0.17	0.36	0.021
200 mg/kg/day											
	2561	3/1	-7	6.96	16.0	47.1	67.7	23.0	34.0	11.6	1.63
	2570	3/1	-7	6.67	14.7	43.8	65.8	22.0	33.5	11.4	1.72
	2567	3/1	-7	6.69	15.4	44.8	67.0	22.9	34.3	11.3	1.65
			N	3	3	3	3	3	3	3	3
			Mean	6.77	15.4	45.2	66.8	22.6	33.9	11.4	1.67
			Sdev	0.162	0.65	1.69	0.96	0.55	0.40	0.15	0.047
800 mg/kg/day											
	2564	4/1	-7	6.45	15.0	44.1	68.4	23.3	34.0	10.7	1.63
	2565	4/1	-7	7.13	16.6	49.1	68.9	23.2	33.7	11.5	1.67
	2569	4/1	-7	7.20	15.9	47.8	66.3	22.1	33.3	11.0	1.73
	2571	4/1	-7	6.97	15.4	46.0	66.0	22.0	33.4	11.3	1.71
	2573	4/1	-7	6.32	14.8	43.3	68.5	23.3	34.1	11.3	1.65
			N	5	5	5	5	5	5	5	5
			Mean	6.81	15.5	46.1	67.6	22.8	33.7	11.2	1.68
			Sdev	0.403	0.73	2.44	1.36	0.67	0.35	0.31	0.041

RBC - RED BLOOD CELLS
HGB - HEMOGLOBIN
HCT - HEMATOCRIT
MCV - MEAN CORPUSCULAR VOLUME
MCH - MEAN CORPUSCULAR HEMOGLOBIN
MCHC - MEAN CORPUSCULAR HGB CONC.
RDW - RED CELL DISTRIBUTION WIDTH
HDW - HEMOGLOBIN DISTRIB. WIDTH

Appendix 6
Day -7 Hematology Data
Pretest phase

Session 1 (Scheduled)
Fexinidazole

Study Number: 0505-2007

Females

Dose Level	Animal Number	Group/ Subgroup	Study Day	R %	RAB 10^9/L	MCVr fL	CHCM g/dL	CHr pg	PLT 10^3/mcL	MPV fL	PDW %
Vehicle											
	2560	1/1	-7	0.6	39.1	84.1	29.0	24.2	317.	11.4	66.5
	2568	1/1	-7	0.5	30.1	87.6	28.5	24.8	301.	8.6	49.6
	2572	1/1	-7	0.6	34.9	86.7	28.2	24.3	467.	9.3	55.7
	2575	1/1	-7	0.4	28.5	85.0	29.7	25.1	368.	8.8	52.0
	2577	1/1	-7	0.3	19.1	84.9	29.4	24.8	250.	9.2	59.1
			N	5	5	5	5	5	5	5	5
			Mean	0.5	30.3	85.7	29.0	24.6	341.	9.5	56.6
			Sdev	0.13	7.54	1.44	0.62	0.38	82.3	1.12	6.62
50 mg/kg/day											
	2562	2/1	-7	1.0	76.6	87.2	28.8	25.0	261.	10.0	58.2
	2563	2/1	-7	1.0	69.3	92.0	27.6	25.3	279.	12.1	61.7
	2576	2/1	-7	0.5	38.5	84.8	29.2	24.6	424.	9.3	59.0
			N	3	3	3	3	3	3	3	3
			Mean	0.8	61.5	88.0	28.5	25.0	321.	10.5	59.6
			Sdev	0.25	20.22	3.67	0.83	0.35	89.4	1.46	1.83
200 mg/kg/day											
	2561	3/1	-7	0.5	34.1	85.2	28.6	24.3	365.	10.5	58.6
	2570	3/1	-7	0.6	42.5	81.8	29.0	23.6	407.	8.7	54.8
	2567	3/1	-7	0.4	24.5	87.5	28.5	24.9	271.	8.9	57.2
			N	3	3	3	3	3	3	3	3
			Mean	0.5	33.7	84.8	28.7	24.3	348.	9.4	56.9
			Sdev	0.14	9.01	2.87	0.26	0.65	69.6	0.99	1.92
800 mg/kg/day											
	2564	4/1	-7	0.3	18.2	87.0	29.2	25.1	287.	9.5	52.9
	2565	4/1	-7	0.7	50.5	90.2	28.3	25.4	281.	10.0	52.8
	2569	4/1	-7	0.7	47.8	82.1	29.0	23.6	324.	9.0	50.8
	2571	4/1	-7	0.7	47.7	84.1	28.7	24.0	324.	9.3	54.3
	2573	4/1	-7	0.3	21.5	83.0	30.2	24.6	254.	10.3	60.7
			N	5	5	5	5	5	5	5	5
			Mean	0.5	37.1	85.3	29.1	24.5	294.	9.6	54.3
			Sdev	0.21	15.87	3.31	0.71	0.75	30.1	0.53	3.79

R - RETICULOCYTES
RAB - RETICULOCYTES ABS
MCVr - MEAN CORPUSCOLAR VOL. RETIC.
CHCM - MEAN HEMOGLOBIN CONC. RETIC.
CHr - CELLULAR HEMOGLOBIN RETIC.
PLT - PLATELETS
MPV - MEAN PLATELET VOLUME
PDW - PLATELET DISTRIBUTION WIDTH

Appendix 6
Day -7 Hematology Data
Pretest phase

Session 1 (Scheduled)
Fexinidazole

Study Number: 0505-2007

Females

Dose Level	Animal Number	Group/ Subgroup	Study Day	PCT %	WBC 10^3/mcL	NAB 10^3/mcL	LYAB 10^3/mcL	MAB 10^3/mcL	EAB 10^3/mcL	BAB 10^3/mcL
Vehicle										
	2560	1/1	-7	0.36	12.60	7.58	4.01	0.70	0.09	0.15
	2568	1/1	-7	0.26	10.13	5.71	3.64	0.42	0.21	0.09
	2572	1/1	-7	0.44	9.83	6.50	2.53	0.38	0.18	0.16
	2575	1/1	-7	0.32	11.65	6.30	4.07	0.62	0.43	0.17
	2577	1/1	-7	0.23	8.31	4.50	3.08	0.39	0.14	0.14
			N	5	5	5	5	5	5	5
			Mean	0.32	10.50	6.12	3.47	0.50	0.21	0.14
			Sdev	0.083	1.667	1.129	0.655	0.148	0.131	0.031
50 mg/kg/day										
	2562	2/1	-7	0.26	12.29	7.89	3.26	0.64	0.29	0.16
	2563	2/1	-7	0.34	10.02	5.71	3.15	0.57	0.42	0.12
	2576	2/1	-7	0.39	10.15	6.00	2.96	0.39	0.57	0.18
			N	3	3	3	3	3	3	3
			Mean	0.33	10.82	6.53	3.12	0.53	0.43	0.15
			Sdev	0.066	1.275	1.184	0.152	0.129	0.140	0.031
200 mg/kg/day										
	2561	3/1	-7	0.38	9.18	4.50	3.81	0.37	0.15	0.25
	2570	3/1	-7	0.35	10.82	7.91	2.07	0.51	0.22	0.08
	2567	3/1	-7	0.24	8.75	4.31	2.89	0.38	1.02	0.11
			N	3	3	3	3	3	3	3
			Mean	0.32	9.58	5.57	2.92	0.42	0.46	0.15
			Sdev	0.074	1.092	2.026	0.870	0.078	0.483	0.091
800 mg/kg/day										
	2564	4/1	-7	0.27	7.83	3.77	2.90	0.48	0.50	0.12
	2565	4/1	-7	0.28	8.55	4.81	2.36	0.30	0.97	0.07
	2569	4/1	-7	0.29	8.65	5.12	2.88	0.30	0.19	0.12
	2571	4/1	-7	0.30	7.70	4.44	2.65	0.26	0.23	0.10
	2573	4/1	-7	0.26	7.61	4.80	2.37	0.21	0.08	0.11
			N	5	5	5	5	5	5	5
			Mean	0.28	8.07	4.59	2.63	0.31	0.39	0.10
			Sdev	0.016	0.493	0.517	0.263	0.102	0.357	0.021

PCT - PLATELET HEMATOCRIT
WBC - WHITE BLOOD CELLS
NAB - NEUTROPHILS ABS
LYAB - LYMPHOCYTES ABS
MAB - MONOCYTES ABS
EAB - EOSINOPHILS ABS
BAB - BASOPHILS ABS

Appendix 6
Day -7 Hematology Data
Pretest phase

Session 1 (Scheduled)
Fexinidazole
Study Number: 0505-2007

Females

Dose Level	Animal Number	Group/ Subgroup	Study Day	LUAB 10^3/mcL	N %	LY %	M %	E %	B %	LU %
Vehicle										
	2560	1/1	-7	0.06	60.2	31.9	5.5	0.8	1.2	0.5
	2568	1/1	-7	0.05	56.4	36.0	4.2	2.1	0.9	0.5
	2572	1/1	-7	0.08	66.2	25.7	3.8	1.8	1.6	0.8
	2575	1/1	-7	0.07	54.1	34.9	5.3	3.7	1.4	0.6
	2577	1/1	-7	0.07	54.1	37.1	4.6	1.7	1.7	0.8
			N	5	5	5	5	5	5	5
			Mean	0.07	58.2	33.1	4.7	2.0	1.4	0.6
			Sdev	0.011	5.12	4.58	0.72	1.06	0.32	0.15
50 mg/kg/day										
	2562	2/1	-7	0.05	64.2	26.6	5.2	2.3	1.3	0.4
	2563	2/1	-7	0.04	57.0	31.5	5.7	4.2	1.2	0.4
	2576	2/1	-7	0.06	59.1	29.2	3.8	5.6	1.7	0.6
			N	3	3	3	3	3	3	3
			Mean	0.05	60.1	29.1	4.9	4.0	1.4	0.5
			Sdev	0.010	3.70	2.45	0.98	1.66	0.26	0.12
200 mg/kg/day										
	2561	3/1	-7	0.10	49.0	41.5	4.0	1.6	2.7	1.1
	2570	3/1	-7	0.03	73.2	19.1	4.7	2.0	0.8	0.3
	2567	3/1	-7	0.03	49.3	33.0	4.4	11.7	1.3	0.3
			N	3	3	3	3	3	3	3
			Mean	0.05	57.2	31.2	4.4	5.1	1.6	0.6
			Sdev	0.040	13.89	11.31	0.35	5.72	0.98	0.46
800 mg/kg/day										
	2564	4/1	-7	0.05	48.2	37.0	6.1	6.4	1.6	0.7
	2565	4/1	-7	0.03	56.3	27.6	3.5	11.3	0.8	0.4
	2569	4/1	-7	0.05	59.2	33.3	3.4	2.1	1.4	0.6
	2571	4/1	-7	0.02	57.7	34.4	3.3	3.0	1.3	0.3
	2573	4/1	-7	0.04	63.0	31.1	2.8	1.1	1.4	0.6
			N	5	5	5	5	5	5	5
			Mean	0.04	56.9	32.7	3.8	4.8	1.3	0.5
			Sdev	0.013	5.46	3.55	1.30	4.15	0.30	0.16

LUAB - LARGE UNSTAINED CELLS ABS
N - NEUTROPHILS %
LY - LYMPHOCITES %
M - MONOCYTES %
E - EOSINOPHILS %
B - BASOPHILS %
LU - LARGE UNSTAINED CELLS %

Appendix 6
Day 28 Hematology Data
Test period

Session 1 (Scheduled)
Fexinidazole

Study Number: 0505-2007

M a l e s

Dose Level	Animal Number	Group/ Subgroup	Study Day	RBC 10^6/mcL	HGB g/dL	HCT %	MCV fL	MCH pg	MCHC g/dL	RDW %	HDW g/dL
Vehicle											
	2516	1/1	28	6.46	14.1	43.0	66.6	21.9	32.9	12.1	1.76
	2518	1/1	28	6.11	13.8	41.1	67.2	22.6	33.6	12.5	1.91
	2520	1/1	28	6.62	13.9	42.4	64.0	21.0	32.9	11.9	1.80
	2527	1/1	28	5.77	12.8	37.9	65.7	22.3	33.9	12.4	1.75
	2533	1/1	28	5.50	12.0	36.1	65.6	21.7	33.1	12.3	1.81
			N	5	5	5	5	5	5	5	5
			Mean	6.09	13.3	40.1	65.8	21.9	33.3	12.2	1.81
			Sdev	0.466	0.89	2.98	1.21	0.61	0.45	0.24	0.063
50 mg/kg/day											
	2514	2/1	28	7.00	15.1	46.5	66.4	21.6	32.5	12.0	1.82
	2521	2/1	28	7.63	17.0	50.7	66.4	22.3	33.5	11.8	1.75
	2529	2/1	28	6.66	14.5	42.6	64.0	21.8	34.1	11.0	1.67
			N	3	3	3	3	3	3	3	3
			Mean	7.10	15.5	46.6	65.6	21.9	33.4	11.6	1.75
			Sdev	0.492	1.31	4.05	1.39	0.36	0.81	0.53	0.075
200 mg/kg/day											
	2515	3/1	28	6.81	14.6	44.4	65.3	21.5	33.0	12.0	1.80
	2523	3/1	28	5.39	11.6	35.4	65.6	21.6	32.9	11.6	1.86
	2526	3/1	28	6.27	14.0	41.8	66.6	22.4	33.5	12.4	1.75
			N	3	3	3	3	3	3	3	3
			Mean	6.16	13.4	40.5	65.8	21.8	33.1	12.0	1.80
			Sdev	0.717	1.59	4.63	0.68	0.49	0.32	0.40	0.055
800 mg/kg/day											
	2517	4/1	28	5.72	12.6	37.2	65.1	22.0	33.7	11.4	1.82
	2519	4/1	28	6.41	14.0	41.1	64.1	21.8	34.1	11.5	1.76
	2525	4/1	28	6.64	14.7	43.7	65.8	22.2	33.8	12.3	1.83
	2528	4/1	28	5.95	13.0	39.4	66.2	21.9	33.1	12.0	1.74
	2530	4/1	28	5.59	12.3	37.3	66.6	22.0	33.0	12.1	1.92
			N	5	5	5	5	5	5	5	5
			Mean	6.06	13.3	39.7	65.6	22.0	33.5	11.9	1.81
			Sdev	0.449	1.00	2.74	0.99	0.15	0.47	0.39	0.071

RBC - RED BLOOD CELLS
HGB - HEMOGLOBIN
HCT - HEMATOCRIT
MCV - MEAN CORPUSCULAR VOLUME
MCH - MEAN CORPUSCULAR HEMOGLOBIN
MCHC - MEAN CORPUSCULAR HGB CONC.
RDW - RED CELL DISTRIBUTION WIDTH
HDW - HEMOGLOBIN DISTRIB. WIDTH

Appendix 6
Day 28 Hematology Data
Test period

Session 1 (Scheduled)
Fexinidazole
Study Number: 0505-2007

Males

Dose Level	Animal Number	Group/ Subgroup	Study Day	R %	RAB 10^9/L	MCVr fL	CHCM g/dL	CHr pg	PLT 10^3/mcL	MPV fL	PDW %
Vehicle											
	2516	1/1	28	1.3	82.6	90.9	26.8	24.3	221.	11.5	57.7
	2518	1/1	28	1.2	73.0	91.2	27.9	25.3	302.	10.3	58.2
	2520	1/1	28	0.8	52.0	87.1	27.6	23.9	493.	11.2	64.5
	2527	1/1	28	0.5	27.1	92.4	27.0	24.9	418.	10.7	57.1
	2533	1/1	28	1.2	67.9	87.1	28.0	24.3	415.	9.5	55.9
			N	5	5	5	5	5	5	5	5
			Mean	1.0	60.5	89.7	27.5	24.5	370.	10.6	58.7
			Sdev	0.35	21.72	2.47	0.54	0.55	107.6	0.79	3.36
50 mg/kg/day											
	2514	2/1	28	1.0	66.4	92.8	27.0	25.0	252.	12.0	58.9
	2521	2/1	28	0.7	51.9	88.2	28.8	25.3	227.	10.3	60.6
	2529	2/1	28	0.4	25.0	87.4	28.2	24.5	248.	10.5	64.9
			N	3	3	3	3	3	3	3	3
			Mean	0.7	47.8	89.5	28.0	24.9	242.	10.9	61.5
			Sdev	0.29	21.01	2.91	0.92	0.40	13.4	0.93	3.09
200 mg/kg/day											
	2515	3/1	28	0.5	36.8	86.0	27.8	23.7	263.	11.1	58.1
	2523	3/1	28	0.6	32.1	85.5	27.7	23.6	603.	9.1	58.0
	2526	3/1	28	0.4	23.7	90.3	28.0	25.2	286.	10.9	58.8
			N	3	3	3	3	3	3	3	3
			Mean	0.5	30.9	87.3	27.8	24.2	384.	10.4	58.3
			Sdev	0.11	6.64	2.64	0.15	0.90	190.0	1.10	0.44
800 mg/kg/day											
	2517	4/1	28	0.4	22.5	83.6	28.5	23.7	374.	9.8	58.6
	2519	4/1	28	0.3	19.9	87.8	28.9	25.2	271.	11.0	64.5
	2525	4/1	28	0.8	50.1	90.6	28.0	25.2	254.	12.8	67.8
	2528	4/1	28	0.9	51.0	89.0	27.9	24.8	200.	9.2	68.0
	2530	4/1	28	0.5	26.2	87.9	28.0	24.5	265.	11.8	63.2
			N	5	5	5	5	5	5	5	5
			Mean	0.6	33.9	87.8	28.3	24.7	273.	10.9	64.4
			Sdev	0.24	15.33	2.59	0.43	0.62	63.2	1.46	3.86

R - RETICULOCYTES
RAB - RETICULOCYTES ABS
MCVr - MEAN CORPUSCOLAR VOL. RETIC.
CHCM - MEAN HEMOGLOBIN CONC. RETIC.
CHr - CELLULAR HEMOGLOBIN RETIC.
PLT - PLATELETS
MPV - MEAN PLATELET VOLUME
PDW - PLATELET DISTRIBUTION WIDTH

Appendix 6
Day 28 Hematology Data
Test period

Session 1 (Scheduled)
Fexinidazole
Study Number: 0505-2007

M a l e s

Dose Level	Animal Number	Group/ Subgroup	Study Day	PCT %	WBC 10^3/mcL	NAB 10^3/mcL	LYAB 10^3/mcL	MAB 10^3/mcL	EAB 10^3/mcL	BAB 10^3/mcL
Vehicle										
	2516	1/1	28	0.25	9.38	5.93	2.91	0.28	0.15	0.05
	2518	1/1	28	0.31	9.08	5.82	2.63	0.37	0.17	0.05
	2520	1/1	28	0.55	6.49	3.41	2.62	0.27	0.13	0.02
	2527	1/1	28	0.45	11.61	7.62	2.82	0.93	0.15	0.04
	2533	1/1	28	0.39	8.19	4.78	2.79	0.36	0.19	0.03
			N	5	5	5	5	5	5	5
			Mean	0.39	8.95	5.51	2.75	0.44	0.16	0.04
			Sdev	0.117	1.865	1.555	0.126	0.277	0.023	0.013
50 mg/kg/day										
	2514	2/1	28	0.30	8.79	4.91	3.26	0.28	0.23	0.06
	2521	2/1	28	0.23	7.96	4.72	2.69	0.20	0.23	0.07
	2529	2/1	28	0.26	6.42	3.45	2.49	0.20	0.13	0.09
			N	3	3	3	3	3	3	3
			Mean	0.26	7.72	4.36	2.81	0.23	0.20	0.07
			Sdev	0.035	1.203	0.794	0.400	0.046	0.058	0.015
200 mg/kg/day										
	2515	3/1	28	0.29	8.49	6.14	1.89	0.20	0.19	0.05
	2523	3/1	28	0.55	8.17	5.15	2.39	0.29	0.24	0.04
	2526	3/1	28	0.31	6.67	3.91	2.28	0.25	0.13	0.05
			N	3	3	3	3	3	3	3
			Mean	0.38	7.78	5.07	2.19	0.25	0.19	0.05
			Sdev	0.145	0.972	1.117	0.263	0.045	0.055	0.006
800 mg/kg/day										
	2517	4/1	28	0.37	6.83	5.32	1.13	0.30	0.03	0.02
	2519	4/1	28	0.30	6.52	3.43	2.22	0.31	0.48	0.05
	2525	4/1	28	0.33	9.91	7.18	2.31	0.26	0.10	0.03
	2528	4/1	28	0.18	9.28	6.14	2.47	0.48	0.11	0.04
	2530	4/1	28	0.31	6.64	3.87	2.33	0.27	0.04	0.05
			N	5	5	5	5	5	5	5
			Mean	0.30	7.84	5.19	2.09	0.32	0.15	0.04
			Sdev	0.071	1.625	1.559	0.545	0.090	0.187	0.013

PCT - PLATELET HEMATOCRIT
WBC - WHITE BLOOD CELLS
NAB - NEUTROPHILS ABS
LYAB - LYMPHOCYTES ABS
MAB - MONOCYTES ABS
EAB - EOSINOPHILS ABS
BAB - BASOPHILS ABS

Appendix 6
Day 28 Hematology Data
Test period

Session 1 (Scheduled)
Fexinidazole

Study Number: 0505-2007

M a l e s

Dose Level	Animal Number	Group/ Subgroup	Study Day	LUAB 10^3/mcL	N %	LY %	M %	E %	B %	LU %
Vehicle										
	2516	1/1	28	0.05	63.3	31.0	3.0	1.6	0.5	0.5
	2518	1/1	28	0.05	64.1	28.9	4.1	1.9	0.5	0.5
	2520	1/1	28	0.03	52.6	40.5	4.1	2.0	0.3	0.5
	2527	1/1	28	0.06	65.6	24.3	8.0	1.3	0.3	0.5
	2533	1/1	28	0.03	58.3	34.1	4.4	2.4	0.4	0.4
			N	5	5	5	5	5	5	5
			Mean	0.04	60.8	31.8	4.7	1.8	0.4	0.5
			Sdev	0.013	5.33	6.05	1.91	0.42	0.10	0.04
50 mg/kg/day										
	2514	2/1	28	0.05	55.9	37.1	3.2	2.6	0.7	0.6
	2521	2/1	28	0.05	59.4	33.8	2.5	2.9	0.8	0.6
	2529	2/1	28	0.06	53.7	38.8	3.2	2.1	1.3	0.9
			N	3	3	3	3	3	3	3
			Mean	0.05	56.3	36.6	3.0	2.5	0.9	0.7
			Sdev	0.006	2.87	2.54	0.40	0.40	0.32	0.17
200 mg/kg/day										
	2515	3/1	28	0.04	72.3	22.2	2.3	2.2	0.6	0.4
	2523	3/1	28	0.06	63.0	29.2	3.6	2.9	0.5	0.7
	2526	3/1	28	0.05	58.6	34.1	3.8	1.9	0.8	0.8
			N	3	3	3	3	3	3	3
			Mean	0.05	64.6	28.5	3.2	2.3	0.6	0.6
			Sdev	0.010	6.99	5.98	0.81	0.51	0.15	0.21
800 mg/kg/day										
	2517	4/1	28	0.02	78.0	16.6	4.3	0.5	0.3	0.3
	2519	4/1	28	0.04	52.5	34.1	4.7	7.3	0.7	0.6
	2525	4/1	28	0.03	72.4	23.4	2.6	1.0	0.3	0.3
	2528	4/1	28	0.04	66.1	26.6	5.2	1.2	0.4	0.4
	2530	4/1	28	0.08	58.3	35.1	4.1	0.6	0.7	1.2
			N	5	5	5	5	5	5	5
			Mean	0.04	65.5	27.2	4.2	2.1	0.5	0.6
			Sdev	0.023	10.31	7.70	0.98	2.91	0.20	0.38

LUAB - LARGE UNSTAINED CELLS ABS   N - NEUTROPHILS %   LY - LYMPHOCITES %
M - MONOCYTES %   E - EOSINOPHILS %   B - BASOPHILS %
LU - LARGE UNSTAINED CELLS %

Appendix 6
Day 28 Hematology Data
Test period

Session 1 (Scheduled)
Fexinidazole

Study Number: 0505-2007

Females

Dose Level	Animal Number	Group/ Subgroup	Study Day	RBC 10^6/mcL	HGB g/dL	HCT %	MCV fL	MCH pg	MCHC g/dL	RDW %	HDW g/dL
Vehicle											
	2560	1/1	28	5.57	12.3	37.2	66.8	22.0	33.0	11.7	1.75
	2568	1/1	28	6.57	13.9	44.0	67.0	21.2	31.7	11.7	1.76
	2572	1/1	28	5.91	12.7	39.2	66.2	21.4	32.3	12.1	1.74
	2575	1/1	28	6.82	15.2	46.2	67.8	22.3	32.9	11.1	1.64
	2577	1/1	28	7.00	15.4	46.4	66.3	22.0	33.1	12.0	1.88
			N	5	5	5	5	5	5	5	5
			Mean	6.37	13.9	42.6	66.8	21.8	32.6	11.7	1.75
			Sdev	0.611	1.41	4.19	0.64	0.46	0.59	0.39	0.085
50 mg/kg/day											
	2562	2/1	28	7.29	15.7	47.7	65.4	21.5	32.9	12.2	1.86
	2563	2/1	28	6.89	15.4	46.8	67.8	22.4	33.0	13.0	2.00
	2576	2/1	28	6.72	15.4	45.1	67.1	22.9	34.0	12.1	1.80
			N	3	3	3	3	3	3	3	3
			Mean	6.97	15.5	46.5	66.8	22.3	33.3	12.4	1.89
			Sdev	0.293	0.17	1.32	1.23	0.71	0.61	0.49	0.103
200 mg/kg/day											
	2561	3/1	28	6.01	13.3	39.6	65.8	22.0	33.5	11.8	1.70
	2570	3/1	28	6.16	13.0	39.9	64.7	21.1	32.7	10.9	1.75
	2567	3/1	28	6.63	14.7	44.0	66.3	22.2	33.4	11.4	1.71
			N	3	3	3	3	3	3	3	3
			Mean	6.27	13.7	41.2	65.6	21.8	33.2	11.4	1.72
			Sdev	0.323	0.91	2.46	0.82	0.59	0.44	0.45	0.026
800 mg/kg/day											
	2564	4/1	28	6.04	13.5	40.3	66.7	22.4	33.6	11.2	1.77
	2565	4/1	28	7.32	16.3	49.2	67.2	22.2	33.1	11.8	1.86
	2569	4/1	28	7.11	15.3	46.5	65.4	21.6	33.0	11.5	1.79
	2571	4/1	28	6.63	14.0	42.7	64.4	21.1	32.8	11.4	1.79
	2573	4/1	28	6.91	15.3	46.3	67.0	22.2	33.1	11.3	1.73
			N	5	5	5	5	5	5	5	5
			Mean	6.80	14.9	45.0	66.1	21.9	33.1	11.4	1.79
			Sdev	0.496	1.12	3.50	1.20	0.54	0.29	0.23	0.047

RBC - RED BLOOD CELLS
HGB - HEMOGLOBIN
HCT - HEMATOCRIT
MCV - MEAN CORPUSCULAR VOLUME
MCH - MEAN CORPUSCULAR HEMOGLOBIN
MCHC - MEAN CORPUSCULAR HGB CONC.
RDW - RED CELL DISTRIBUTION WIDTH
HDW - HEMOGLOBIN DISTRIB. WIDTH

Appendix 6
Day 28 Hematology Data
Session 1 (Scheduled)
Test period
Fexinidazole
Study Number: 0505-2007

Females

Dose Level	Animal Number	Group/ Subgroup	Study Day	R %	RAB 10^9/L	MCVr fL	CHCM g/dL	CHr pg	PLT 10^3/mcL	MPV fL	PDW %
Vehicle											
	2560	1/1	28	1.3	73.6	85.8	28.0	24.0	446.	10.0	57.4
	2568	1/1	28	0.6	41.6	88.8	27.8	24.6	358.	8.4	47.3
	2572	1/1	28	0.5	29.0	89.4	27.6	24.6	461.	9.4	50.3
	2575	1/1	28	0.5	31.8	88.0	28.2	24.7	466.	8.9	48.7
	2577	1/1	28	0.3	17.7	86.7	27.4	23.7	389.	9.0	47.1
			N	5	5	5	5	5	5	5	5
			Mean	0.6	38.7	87.7	27.8	24.3	424.	9.1	50.2
			Sdev	0.41	21.27	1.48	0.32	0.44	48.0	0.60	4.25
50 mg/kg/day											
	2562	2/1	28	0.9	65.4	87.1	28.3	24.5	381.	9.6	59.7
	2563	2/1	28	2.0	137.4	92.3	26.9	24.7	373.	10.3	53.7
	2576	2/1	28	0.9	57.5	89.9	28.1	25.1	406.	8.6	52.1
			N	3	3	3	3	3	3	3	3
			Mean	1.3	86.8	89.8	27.8	24.8	387.	9.5	55.2
			Sdev	0.64	44.03	2.60	0.76	0.31	17.2	0.85	4.01
200 mg/kg/day											
	2561	3/1	28	0.4	23.9	90.5	27.9	25.2	355.	10.2	59.6
	2570	3/1	28	0.5	31.9	82.5	27.6	22.7	494.	9.7	55.1
	2567	3/1	28	0.2	15.5	86.7	27.6	23.8	240.	10.5	62.6
			N	3	3	3	3	3	3	3	3
			Mean	0.4	23.8	86.6	27.7	23.9	363.	10.1	59.1
			Sdev	0.15	8.20	4.00	0.17	1.25	127.2	0.40	3.77
800 mg/kg/day											
	2564	4/1	28	0.5	29.4	88.8	27.6	24.5	303.	10.2	55.3
	2565	4/1	28	0.9	62.4	91.0	27.3	24.7	239.	11.3	57.4
	2569	4/1	28	0.5	32.1	85.8	28.1	24.1	338.	10.1	56.7
	2571	4/1	28	0.4	24.4	85.8	27.9	23.8	283.	9.5	58.5
	2573	4/1	28	0.2	13.7	89.9	28.4	25.5	290.	10.5	58.9
			N	5	5	5	5	5	5	5	5
			Mean	0.5	32.4	88.3	27.9	24.5	291.	10.3	57.4
			Sdev	0.24	18.19	2.38	0.43	0.65	35.8	0.66	1.44

R - RETICULOCYTES
RAB - RETICULOCYTES ABS
MCVr - MEAN CORPUSCOLAR VOL. RETIC.
CHCM - MEAN HEMOGLOBIN CONC. RETIC.
CHr - CELLULAR HEMOGLOBIN RETIC.
PLT - PLATELETS
MPV - MEAN PLATELET VOLUME
PDW - PLATELET DISTRIBUTION WIDTH

Appendix 6
Day 28 Hematology Data
Test period

Session 1 (Scheduled)
Fexinidazole

Study Number: 0505-2007

Females

Dose Level	Animal Number	Group/ Subgroup	Study Day	PCT %	WBC 10^3/mcL	NAB 10^3/mcL	LYAB 10^3/mcL	MAB 10^3/mcL	EAB 10^3/mcL	BAB 10^3/mcL
Vehicle										
	2560	1/1	28	0.45	13.22	8.04	4.35	0.56	0.12	0.07
	2568	1/1	28	0.30	11.94	7.67	3.45	0.56	0.16	0.04
	2572	1/1	28	0.43	10.45	6.86	2.84	0.46	0.10	0.09
	2575	1/1	28	0.41	15.06	9.10	4.27	0.97	0.57	0.06
	2577	1/1	28	0.35	8.28	4.62	2.95	0.46	0.16	0.04
			N	5	5	5	5	5	5	5
			Mean	0.39	11.79	7.26	3.57	0.60	0.22	0.06
			Sdev	0.062	2.592	1.680	0.712	0.212	0.196	0.021
50 mg/kg/day										
	2562	2/1	28	0.37	8.98	5.64	2.56	0.45	0.24	0.05
	2563	2/1	28	0.38	12.68	9.15	2.61	0.66	0.14	0.07
	2576	2/1	28	0.35	11.52	7.22	2.68	0.54	0.94	0.08
			N	3	3	3	3	3	3	3
			Mean	0.37	11.06	7.34	2.62	0.55	0.44	0.07
			Sdev	0.015	1.892	1.758	0.060	0.105	0.436	0.015
200 mg/kg/day										
	2561	3/1	28	0.36	11.00	7.51	2.73	0.59	0.07	0.06
	2570	3/1	28	0.48	8.93	6.24	2.01	0.35	0.26	0.04
	2567	3/1	28	0.25	10.05	5.96	3.22	0.52	0.15	0.10
			N	3	3	3	3	3	3	3
			Mean	0.36	9.99	6.57	2.65	0.49	0.16	0.07
			Sdev	0.115	1.036	0.826	0.609	0.123	0.095	0.031
800 mg/kg/day										
	2564	4/1	28	0.31	7.64	4.06	2.52	0.33	0.59	0.04
	2565	4/1	28	0.27	7.32	4.82	1.59	0.26	0.57	0.04
	2569	4/1	28	0.34	9.19	6.18	2.46	0.36	0.09	0.04
	2571	4/1	28	0.27	7.64	4.87	2.35	0.24	0.12	0.03
	2573	4/1	28	0.30	7.60	4.90	2.18	0.31	0.09	0.05
			N	5	5	5	5	5	5	5
			Mean	0.30	7.88	4.97	2.22	0.30	0.29	0.04
			Sdev	0.029	0.746	0.763	0.375	0.049	0.263	0.007

PCT - PLATELET HEMATOCRIT
WBC - WHITE BLOOD CELLS
NAB - NEUTROPHILS ABS
LYAB - LYMPHOCYTES ABS
MAB - MONOCYTES ABS
EAB - EOSINOPHILS ABS
BAB - BASOPHILS ABS

Appendix 6
Day 28 Hematology Data
Test period

Session 1 (Scheduled)
Fexinidazole

Study Number: 0505-2007

Females

Dose Level	Animal Number	Group/ Subgroup	Study Day	LUAB 10^3/mcL	N %	LY %	M %	E %	B %	LU %
Vehicle										
	2560	1/1	28	0.08	60.8	32.9	4.3	0.9	0.5	0.6
	2568	1/1	28	0.06	64.3	28.9	4.7	1.3	0.3	0.5
	2572	1/1	28	0.10	65.7	27.1	4.4	0.9	0.9	0.9
	2575	1/1	28	0.09	60.4	28.4	6.4	3.8	0.4	0.6
	2577	1/1	28	0.05	55.8	35.6	5.5	1.9	0.5	0.6
			N	5	5	5	5	5	5	5
			Mean	0.08	61.4	30.6	5.1	1.8	0.5	0.6
			Sdev	0.021	3.86	3.54	0.88	1.21	0.23	0.15
50 mg/kg/day										
	2562	2/1	28	0.04	62.8	28.5	5.0	2.7	0.6	0.5
	2563	2/1	28	0.05	72.1	20.6	5.2	1.1	0.5	0.4
	2576	2/1	28	0.06	62.7	23.2	4.7	8.2	0.7	0.5
			N	3	3	3	3	3	3	3
			Mean	0.05	65.9	24.1	5.0	4.0	0.6	0.5
			Sdev	0.010	5.40	4.03	0.25	3.72	0.10	0.06
200 mg/kg/day										
	2561	3/1	28	0.04	68.3	24.9	5.3	0.6	0.5	0.3
	2570	3/1	28	0.03	69.9	22.5	4.0	2.9	0.4	0.3
	2567	3/1	28	0.11	59.2	32.1	5.2	1.5	1.0	1.1
			N	3	3	3	3	3	3	3
			Mean	0.06	65.8	26.5	4.8	1.7	0.6	0.6
			Sdev	0.044	5.77	5.00	0.72	1.16	0.32	0.46
800 mg/kg/day										
	2564	4/1	28	0.09	53.2	33.0	4.4	7.8	0.5	1.2
	2565	4/1	28	0.04	65.8	21.8	3.5	7.8	0.6	0.5
	2569	4/1	28	0.06	67.3	26.7	3.9	0.9	0.5	0.6
	2571	4/1	28	0.03	63.8	30.8	3.1	1.5	0.4	0.4
	2573	4/1	28	0.07	64.5	28.7	4.0	1.2	0.7	1.0
			N	5	5	5	5	5	5	5
			Mean	0.06	62.9	28.2	3.8	3.8	0.5	0.7
			Sdev	0.024	5.60	4.28	0.50	3.62	0.11	0.34

LUAB - LARGE UNSTAINED CELLS ABS
N - NEUTROPHILS %
LY - LYMPHOCITES %
M - MONOCYTES %
E - EOSINOPHILS %
B - BASOPHILS %
LU - LARGE UNSTAINED CELLS %

Appendix 6
Day 42 Hematology Data
Test period

Session 1 (Scheduled)
Fexinidazole

Study Number: 0505-2007

Males

Dose Level	Animal Number	Group/ Subgroup	Study Day	RBC 10^6/mcL	HGB g/dL	HCT %	MCV fL	MCH pg	MCHC g/dL	RDW %	HDW g/dL
Vehicle											
	2527	1/1	42	6.01	13.6	40.4	67.2	22.6	33.7	12.8	1.76
	2533	1/1	42	5.88	12.6	39.6	67.3	21.5	31.9	12.7	1.79
			N	2	2	2	2	2	2	2	2
			Mean	5.95	13.1	40.0	67.3	22.1	32.8	12.8	1.78
			Sdev	0.092	0.71	0.57	0.07	0.78	1.27	0.07	0.021
800 mg/kg/day											
	2528	4/1	42	6.88	15.2	46.0	66.9	22.1	33.0	12.5	1.91
	2530	4/1	42	6.12	13.6	41.6	67.9	22.1	32.6	13.1	2.03
			N	2	2	2	2	2	2	2	2
			Mean	6.50	14.4	43.8	67.4	22.1	32.8	12.8	1.97
			Sdev	0.537	1.13	3.11	0.71	0.00	0.28	0.42	0.085

RBC - RED BLOOD CELLS
HGB - HEMOGLOBIN
HCT - HEMATOCRIT
MCV - MEAN CORPUSCULAR VOLUME
MCH - MEAN CORPUSCULAR HEMOGLOBIN
MCHC - MEAN CORPUSCULAR HGB CONC.
RDW - RED CELL DISTRIBUTION WIDTH
HDW - HEMOGLOBIN DISTRIB. WIDTH

CONFIDENTIAL

Appendix 6
Day 42 Hematology Data
Test period

Session 1 (Scheduled)
Fexinidazole

Study Number: 0505-2007

Males

Dose Level	Animal Number	Group/ Subgroup	Study Day	R %	RAB 10^9/L	MCVr fL	CHCM g/dL	CHr pg	PLT 10^3/mcL	MPV fL	PDW %
Vehicle											
	2527	1/1	42	0.6	32.8	93.5	27.5	25.7	402.	10.8	57.3
	2533	1/1	42	1.5	86.1	89.2	27.9	24.8	440.	9.1	54.2
			N	2	2	2	2	2	2	2	2
			Mean	1.0	59.5	91.4	27.7	25.3	421.	10.0	55.8
			Sdev	0.64	37.69	3.04	0.28	0.64	26.9	1.20	2.19
800 mg/kg/day											
	2528	4/1	42	1.0	68.9	88.1	27.7	24.4	255.	8.5	59.0
	2530	4/1	42	1.4	86.5	88.4	28.1	24.7	416.	10.9	60.6
			N	2	2	2	2	2	2	2	2
			Mean	1.2	77.7	88.3	27.9	24.6	336.	9.7	59.8
			Sdev	0.29	12.45	0.21	0.28	0.21	113.8	1.70	1.13

R - RETICULOCYTES
RAB - RETICULOCYTES ABS
MCVr - MEAN CORPUSCOLAR VOL. RETIC.
CHCM - MEAN HEMOGLOBIN CONC. RETIC.
CHr - CELLULAR HEMOGLOBIN RETIC.
PLT - PLATELETS
MPV - MEAN PLATELET VOLUME
PDW - PLATELET DISTRIBUTION WIDTH

Appendix 6
Day 42 Hematology Data
Test period

Session 1 (Scheduled)
Fexinidazole

Study Number: 0505-2007

Males

Dose Level	Animal Number	Group/ Subgroup	Study Day	PCT %	WBC 10^3/mcL	NAB 10^3/mcL	LYAB 10^3/mcL	MAB 10^3/mcL	EAB 10^3/mcL	BAB 10^3/mcL
Vehicle										
	2527	1/1	42	0.43	9.50	5.82	2.97	0.45	0.23	0.01
	2533	1/1	42	0.40	7.94	4.42	2.99	0.38	0.07	0.04
			N	2	2	2	2	2	2	2
			Mean	0.42	8.72	5.12	2.98	0.42	0.15	0.03
			Sdev	0.021	1.103	0.990	0.014	0.049	0.113	0.021
800 mg/kg/day										
	2528	4/1	42	0.22	5.64	2.69	2.46	0.29	0.09	0.07
	2530	4/1	42	0.46	7.61	4.51	2.69	0.26	0.06	0.05
			N	2	2	2	2	2	2	2
			Mean	0.34	6.63	3.60	2.58	0.28	0.08	0.06
			Sdev	0.170	1.393	1.287	0.163	0.021	0.021	0.014

PCT - PLATELET HEMATOCRIT
WBC - WHITE BLOOD CELLS
NAB - NEUTROPHILS ABS
LYAB - LYMPHOCYTES ABS
MAB - MONOCYTES ABS
EAB - EOSINOPHILS ABS
BAB - BASOPHILS ABS

Appendix 6
Day 42 Hematology Data
Test period

Session 1 (Scheduled)
Fexinidazole

Study Number: 0505-2007

Males

Dose Level	Animal Number	Group/ Subgroup	Study Day	LUAB 10^3/mcL	N %	LY %	M %	E %	B %	LU %
Vehicle										
	2527	1/1	42	0.03	61.2	31.3	4.7	2.4	0.1	0.3
	2533	1/1	42	0.05	55.6	37.6	4.7	0.8	0.5	0.7
			N	2	2	2	2	2	2	2
			Mean	0.04	58.4	34.5	4.7	1.6	0.3	0.5
			Sdev	0.014	3.96	4.45	0.00	1.13	0.28	0.28
800 mg/kg/day										
	2528	4/1	42	0.04	47.7	43.6	5.2	1.6	1.2	0.8
	2530	4/1	42	0.03	59.3	35.3	3.5	0.8	0.7	0.4
			N	2	2	2	2	2	2	2
			Mean	0.04	53.5	39.5	4.4	1.2	1.0	0.6
			Sdev	0.007	8.20	5.87	1.20	0.57	0.35	0.28

LUAB - LARGE UNSTAINED CELLS ABS
N - NEUTROPHILS %
LY - LYMPHOCITES %
M - MONOCYTES %
E - EOSINOPHILS %
B - BASOPHILS %
LU - LARGE UNSTAINED CELLS %

Appendix 6
Day 42 Hematology Data
Test period

Session 1 (Scheduled)
Fexinidazole
Study Number: 0505-2007

Females

Dose Level	Animal Number	Group/ Subgroup	Study Day	RBC 10^6/mcL	HGB g/dL	HCT %	MCV fL	MCH pg	MCHC g/dL	RDW %	HDW g/dL
Vehicle											
	2575	1/1	42	6.86	15.6	46.1	67.1	22.7	33.8	11.0	1.58
	2577	1/1	42	6.74	15.0	44.2	65.6	22.3	34.0	11.8	1.71
			N	2	2	2	2	2	2	2	2
			Mean	6.80	15.3	45.2	66.4	22.5	33.9	11.4	1.65
			Sdev	0.085	0.42	1.34	1.06	0.28	0.14	0.57	0.092
800 mg/kg/day											
	2571	4/1	42	6.54	14.2	41.9	63.9	21.7	33.9	12.0	1.78
	2573	4/1	42	6.60	14.7	44.4	67.3	22.3	33.2	12.0	1.71
			N	2	2	2	2	2	2	2	2
			Mean	6.57	14.5	43.2	65.6	22.0	33.6	12.0	1.75
			Sdev	0.042	0.35	1.77	2.40	0.42	0.49	0.00	0.049

RBC - RED BLOOD CELLS
HGB - HEMOGLOBIN
HCT - HEMATOCRIT
MCV - MEAN CORPUSCULAR VOLUME
MCH - MEAN CORPUSCULAR HEMOGLOBIN
MCHC - MEAN CORPUSCULAR HGB CONC.
RDW - RED CELL DISTRIBUTION WIDTH
HDW - HEMOGLOBIN DISTRIB. WIDTH

Appendix 6
Day 42 Hematology Data
Test period

Session 1 (Scheduled)
Fexinidazole

Study Number: 0505-2007

Females

Dose Level	Animal Number	Group/ Subgroup	Study Day	R %	RAB 10^9/L	MCVr fL	CHCM g/dL	CHr pg	PLT 10^3/mcL	MPV fL	PDW %
Vehicle											
	2575	1/1	42	0.5	34.6	84.1	29.4	24.6	458.	9.0	51.7
	2577	1/1	42	0.2	14.2	86.9	28.9	25.0	304.	8.9	50.1
			N	2	2	2	2	2	2	2	2
			Mean	0.4	24.4	85.5	29.2	24.8	381.	9.0	50.9
			Sdev	0.21	14.42	1.98	0.35	0.28	108.9	0.07	1.13
800 mg/kg/day											
	2571	4/1	42	0.8	53.5	85.8	28.4	24.3	335.	8.9	54.3
	2573	4/1	42	0.5	33.1	90.4	29.0	26.1	332.	9.0	55.7
			N	2	2	2	2	2	2	2	2
			Mean	0.7	43.3	88.1	28.7	25.2	334.	9.0	55.0
			Sdev	0.23	14.42	3.25	0.42	1.27	2.1	0.07	0.99

R - RETICULOCYTES
RAB - RETICULOCYTES ABS
MCVr - MEAN CORPUSCOLAR VOL. RETIC.
CHCM - MEAN HEMOGLOBIN CONC. RETIC.
CHr - CELLULAR HEMOGLOBIN RETIC.
PLT - PLATELETS
MPV - MEAN PLATELET VOLUME
PDW - PLATELET DISTRIBUTION WIDTH

Appendix 6
Day 42 Hematology Data
Test period

Session 1 (Scheduled)
Fexinidazole
Study Number: 0505-2007

Females

Dose Level	Animal Number	Group/ Subgroup	Study Day	PCT %	WBC 10^3/mcL	NAB 10^3/mcL	LYAB 10^3/mcL	MAB 10^3/mcL	EAB 10^3/mcL	BAB 10^3/mcL
Vehicle										
	2575	1/1	42	0.41	11.08	5.94	3.62	0.69	0.66	0.09
	2577	1/1	42	0.27	7.94	3.80	3.31	0.50	0.21	0.07
			N	2	2	2	2	2	2	2
			Mean	0.34	9.51	4.87	3.47	0.60	0.44	0.08
			Sdev	0.099	2.220	1.513	0.219	0.134	0.318	0.014
800 mg/kg/day										
	2571	4/1	42	0.30	6.97	4.30	2.15	0.28	0.14	0.06
	2573	4/1	42	0.30	8.24	4.87	2.73	0.30	0.20	0.07
			N	2	2	2	2	2	2	2
			Mean	0.30	7.61	4.59	2.44	0.29	0.17	0.07
			Sdev	0.000	0.898	0.403	0.410	0.014	0.042	0.007

PCT - PLATELET HEMATOCRIT
WBC - WHITE BLOOD CELLS
NAB - NEUTROPHILS ABS
LYAB - LYMPHOCYTES ABS
MAB - MONOCYTES ABS
EAB - EOSINOPHILS ABS
BAB - BASOPHILS ABS

Appendix 6
Day 42 Hematology Data
Test period

Session 1 (Scheduled)
Fexinidazole

Study Number: 0505-2007

Females

Dose Level	Animal Number	Group/ Subgroup	Study Day	LUAB 10^3/mcL	N %	LY %	M %	E %	B %	LU %
Vehicle										
	2575	1/1	42	0.09	53.6	32.6	6.3	6.0	0.8	0.8
	2577	1/1	42	0.05	47.8	41.7	6.3	2.6	0.9	0.7
			N	2	2	2	2	2	2	2
			Mean	0.07	50.7	37.2	6.3	4.3	0.9	0.8
			Sdev	0.028	4.10	6.43	0.00	2.40	0.07	0.07
800 mg/kg/day										
	2571	4/1	42	0.05	61.7	30.8	4.0	2.1	0.8	0.7
	2573	4/1	42	0.07	59.1	33.2	3.6	2.4	0.9	0.8
			N	2	2	2	2	2	2	2
			Mean	0.06	60.4	32.0	3.8	2.3	0.9	0.8
			Sdev	0.014	1.84	1.70	0.28	0.21	0.07	0.07

LUAB - LARGE UNSTAINED CELLS ABS
N - NEUTROPHILS %
LY - LYMPHOCITES %
M - MONOCYTES %
E - EOSINOPHILS %
B - BASOPHILS %
LU - LARGE UNSTAINED CELLS %

Appendix 7 Coagulation

Appendix 7
Day -7 Hematology Data
Pretest phase

Session 1 (Scheduled)
Fexinidazole

Study Number: 0505-2007

M a l e s

Dose Level	Animal Number	Group/ Subgroup	Study Day	PT sec	PTr ratio	PTT sec	PTTr ratio	FIB mg/dL
Vehicle								
	2516	1/1	-7	7.3	1.0	12.7	1.0	213.
	2518	1/1	-7	6.9	1.0	12.4	1.0	222.
	2520	1/1	-7	6.9	1.0	12.4	1.0	216.
	2527	1/1	-7	7.0	1.0	12.7	1.0	215.
	2533	1/1	-7	8.0	1.1	12.2	1.0	179.
			N	5	5	5	5	5
			Mean	7.2	1.0	12.5	1.0	209.
			Sdev	0.46	0.06	0.23	0.02	17.3
50 mg/kg/day								
	2514	2/1	-7	7.7	1.1	12.4	1.0	164.
	2521	2/1	-7	7.2	1.0	12.9	1.0	188.
	2529	2/1	-7	8.0	1.1	13.0	1.0	161.
			N	3	3	3	3	3
			Mean	7.6	1.0	12.8	1.0	171.
			Sdev	0.36	0.05	0.31	0.03	14.8
200 mg/kg/day								
	2515	3/1	-7	7.4	1.0	12.7	1.0	176.
	2523	3/1	-7	6.9	0.9	12.6	1.0	248.
	2526	3/1	-7	7.5	1.0	12.9	1.0	165.
			N	3	3	3	3	3
			Mean	7.2	1.0	12.7	1.0	196.
			Sdev	0.31	0.04	0.18	0.01	45.2
800 mg/kg/day								
	2517	4/1	-7	7.4	1.0	13.1	1.0	205.
	2519	4/1	-7	6.8	0.9	12.2	1.0	250.
	2525	4/1	-7	8.0	1.1	12.5	1.0	175.
	2528	4/1	-7	7.1	1.0	13.0	1.0	226.
	2530	4/1	-7	7.5	1.0	12.4	1.0	191.
			N	5	5	5	5	5
			Mean	7.4	1.0	12.6	1.0	209.
			Sdev	0.46	0.06	0.37	0.03	29.2

PT - PROTHROMBIN TIME PTr - PROTHROMBIN TIME RATIO PTT - ACT. PAR. THROMB. TIME
PTTr - ACT.PAR.THROMB.TIME RATIO FIB - FIBRINOGEN

Appendix 7
Day -7 Hematology Data
Pretest phase

Session 1 (Scheduled)
Fexinidazole

Study Number: 0505-2007

Females

Dose Level	Animal Number	Group/ Subgroup	Study Day	PT sec	PTr ratio	PTT sec	PTTr ratio	FIB mg/dL
Vehicle								
	2560	1/1	-7	7.0	1.0	12.9	1.0	217.
	2568	1/1	-7	9.5	1.3	69.7	5.5	163.
	2572	1/1	-7	7.1	1.0	12.8	1.0	189.
	2575	1/1	-7	7.6	1.0	12.6	1.0	179.
	2577	1/1	-7	7.8	1.1	12.8	1.0	166.
			N	5	5	5	5	5
			Mean	7.8	1.1	24.2	1.9	183.
			Sdev	0.99	0.14	25.44	2.01	22.0
50 mg/kg/day								
	2562	2/1	-7	8.7	1.2	12.9	1.0	166.
	2563	2/1	-7	6.8	0.9	12.4	1.0	246.
	2576	2/1	-7	7.5	1.0	12.5	1.0	154.
			N	3	3	3	3	3
			Mean	7.7	1.1	12.6	1.0	188.
			Sdev	0.99	0.14	0.31	0.02	50.2
200 mg/kg/day								
	2561	3/1	-7	7.0	1.0	13.0	1.0	264.
	2570	3/1	-7	7.2	1.0	12.6	1.0	199.
	2567	3/1	-7	7.3	1.0	12.9	1.0	182.
			N	3	3	3	3	3
			Mean	7.2	1.0	12.8	1.0	215.
			Sdev	0.15	0.02	0.24	0.02	43.0
800 mg/kg/day								
	2564	4/1	-7	7.3	1.0	12.3	1.0	172.
	2565	4/1	-7	7.1	1.0	12.7	1.0	202.
	2569	4/1	-7	8.0	1.1	13.0	1.0	164.
	2571	4/1	-7	7.4	1.0	13.1	1.0	150.
	2573	4/1	-7	8.9	1.2	13.6	1.1	141.
			N	5	5	5	5	5
			Mean	7.8	1.1	12.9	1.0	166.
			Sdev	0.75	0.10	0.47	0.04	23.5

PT - PROTHROMBIN TIME
PTr - PROTHROMBIN TIME RATIO
PTT - ACT. PAR. THROMB. TIME
PTTr - ACT.PAR.THROMB.TIME RATIO
FIB - FIBRINOGEN

Appendix 7
Day 28 Hematology Data
Test period

Session 1 (Scheduled)
Fexinidazole

Study Number: 0505-2007

Males

Dose Level	Animal Number	Group/ Subgroup	Study Day	PT sec	PTr ratio	PTT sec	PTTr ratio	FIB mg/dL
Vehicle								
	2516	1/1	28	7.4	1.0	12.4	1.0	197.
	2518	1/1	28	7.0	1.0	12.2	1.0	224.
	2520	1/1	28	6.9	1.0	12.5	1.0	227.
	2527	1/1	28	7.2	1.0	12.7	1.0	216.
	2533	1/1	28	7.7	1.1	12.4	1.0	235.
			N	5	5	5	5	5
			Mean	7.2	1.0	12.4	1.0	220.
			Sdev	0.29	0.04	0.19	0.01	14.3
50 mg/kg/day								
	2514	2/1	28	8.0	1.1	12.6	1.0	174.
	2521	2/1	28	7.7	1.1	13.2	1.0	161.
	2529	2/1	28	8.4	1.2	13.4	1.1	156.
			N	3	3	3	3	3
			Mean	8.0	1.1	13.1	1.0	164.
			Sdev	0.34	0.05	0.40	0.03	9.3
200 mg/kg/day								
	2515	3/1	28	7.0	1.0	12.4	1.0	262.
	2523	3/1	28	6.9	1.0	12.6	1.0	235.
	2526	3/1	28	8.4	1.2	13.0	1.0	152.
			N	3	3	3	3	3
			Mean	7.4	1.0	12.7	1.0	216.
			Sdev	0.87	0.12	0.29	0.03	57.0
800 mg/kg/day								
	2517	4/1	28	7.5	1.0	13.0	1.0	175.
	2519	4/1	28	7.4	1.0	12.4	1.0	170.
	2525	4/1	28	8.6	1.2	13.0	1.0	162.
	2528	4/1	28	7.2	1.0	12.3	1.0	203.
	2530	4/1	28	8.0	1.1	12.4	1.0	158.
			N	5	5	5	5	5
			Mean	7.7	1.1	12.6	1.0	173.
			Sdev	0.56	0.08	0.32	0.03	17.9

PT - PROTHROMBIN TIME
PTr - PROTHROMBIN TIME RATIO
PTT - ACT. PAR. THROMB. TIME
PTTr - ACT.PAR.THROMB.TIME RATIO
FIB - FIBRINOGEN

Appendix 7
Day 28 Hematology Data
Test period

Session 1 (Scheduled)
Fexinidazole

Study Number: 0505-2007

Females

Dose Level	Animal Number	Group/ Subgroup	Study Day	PT sec	PTr ratio	PTT sec	PTTr ratio	FIB mg/dL
Vehicle								
	2560	1/1	28	7.2	1.0	13.0	1.0	209.
	2568	1/1	28	7.8	1.1	12.5	1.0	157.
	2572	1/1	28	7.2	1.0	13.1	1.0	210.
	2575	1/1	28	7.2	1.0	12.4	1.0	202.
	2577	1/1	28	7.8	1.1	13.7	1.1	160.
			N	5	5	5	5	5
			Mean	7.4	1.0	12.9	1.0	188.
			Sdev	0.34	0.05	0.52	0.04	26.8
50 mg/kg/day								
	2562	2/1	28	8.0	1.1	12.4	1.0	176.
	2563	2/1	28	7.0	1.0	12.6	1.0	204.
	2576	2/1	28	7.3	1.0	12.5	1.0	202.
			N	3	3	3	3	3
			Mean	7.4	1.0	12.5	1.0	194.
			Sdev	0.49	0.07	0.10	0.01	15.3
200 mg/kg/day								
	2561	3/1	28	7.5	1.0	12.5	1.0	175.
	2570	3/1	28	7.3	1.0	12.5	1.0	181.
	2567	3/1	28	6.9	0.9	12.4	1.0	283.
			N	3	3	3	3	3
			Mean	7.2	1.0	12.5	1.0	213.
			Sdev	0.31	0.04	0.06	0.01	60.8
800 mg/kg/day								
	2564	4/1	28	7.3	1.0	12.7	1.0	206.
	2565	4/1	28	7.1	1.0	12.9	1.0	218.
	2569	4/1	28	8.4	1.2	12.6	1.0	162.
	2571	4/1	28	7.4	1.0	12.8	1.0	170.
	2573	4/1	28	7.8	1.1	12.6	1.0	165.
			N	5	5	5	5	5
			Mean	7.6	1.0	12.7	1.0	184.
			Sdev	0.52	0.07	0.10	0.01	25.9

PT - PROTHROMBIN TIME
PTr - PROTHROMBIN TIME RATIO
PTT - ACT. PAR. THROMB. TIME
PTTr - ACT.PAR.THROMB.TIME RATIO
FIB - FIBRINOGEN

Appendix 7
Day 42 Hematology Data
Test period

Session 1 (Scheduled)
Fexinidazole

Study Number: 0505-2007

Males

Dose Level	Animal Number	Group/ Subgroup	Study Day	PT sec	PTr ratio	PTT sec	PTTr ratio	FIB mg/dL
Vehicle								
	2527	1/1	42	7.3	1.0	12.8	1.0	223.
	2533	1/1	42	8.3	1.1	12.4	1.0	207.
			N	2	2	2	2	2
			Mean	7.8	1.1	12.6	1.0	215.
			Sdev	0.74	0.10	0.28	0.02	11.0
800 mg/kg/day								
	2528	4/1	42	7.9	1.1	12.6	1.0	165.
	2530	4/1	42	8.0	1.1	12.2	1.0	215.
			N	2	2	2	2	2
			Mean	7.9	1.1	12.4	1.0	190.
			Sdev	0.05	0.01	0.28	0.03	35.6

PT - PROTHROMBIN TIME
PTr - PROTHROMBIN TIME RATIO
PTT - ACT. PAR. THROMB. TIME
PTTr - ACT.PAR.THROMB.TIME RATIO
FIB - FIBRINOGEN

Appendix 7
Day 42 Hematology Data
Test period

Session 1 (Scheduled)
Fexinidazole

Study Number: 0505-2007

Females

Dose Level	Animal Number	Group/ Subgroup	Study Day	PT sec	PTr ratio	PTT sec	PTTr ratio	FIB mg/dL
Vehicle								
	2575	1/1	42	7.4	1.0	12.5	1.0	167.
	2577	1/1	42	7.6	1.1	12.4	1.0	167.
			N	2	2	2	2	2
			Mean	7.5	1.0	12.4	1.0	167.
			Sdev	0.17	0.03	0.06	0.00	0.4
800 mg/kg/day								
	2571	4/1	42	7.1	1.0	12.2	1.0	201.
	2573	4/1	42	8.0	1.1	12.5	1.0	174.
			N	2	2	2	2	2
			Mean	7.6	1.0	12.3	1.0	188.
			Sdev	0.59	0.08	0.24	0.02	19.6

PT - PROTHROMBIN TIME
PTr - PROTHROMBIN TIME RATIO
PTT - ACT. PAR. THROMB. TIME
PTTr - ACT.PAR.THROMB.TIME RATIO
FIB - FIBRINOGEN

Appendix 8 Clinical Chemistry

Appendix 8
Day -7 Clinical Chemistry Data
Pretest phase

Session 1 (Scheduled)
Fexinidazole
Study Number: 0505-2007

Males

Dose Level	Animal Number	Group/ Subgroup	Study Day	UREA mg/dL	CREA mg/dL	AST IU/L	ALT IU/L	AP IU/L	GGT IU/L	TBIL mg/dL
Vehicle										
	2516	1/1	-7	24.	0.86	35.	39.	84.	6.	0.09
	2518	1/1	-7	26.	0.92	38.	39.	114.	5.	0.13
	2520	1/1	-7	29.	0.83	28.	21.	55.	6.	0.08
	2527	1/1	-7	38.	0.94	44.	26.	72.	7.	0.09
	2533	1/1	-7	31.	1.00	50.	38.	63.	8.	0.11
			N	5	5	5	5	5	5	5
			Mean	30.	0.91	39.	33.	78.	6.	0.10
			Sdev	5.4	0.067	8.4	8.5	23.0	1.1	0.020
50 mg/kg/day										
	2514	2/1	-7	18.	0.94	28.	31.	79.	8.	0.09
	2521	2/1	-7	23.	0.81	33.	30.	67.	7.	0.13
	2529	2/1	-7	22.	0.87	39.	30.	87.	10.	0.13
			N	3	3	3	3	3	3	3
			Mean	21.	0.87	33.	30.	78.	8.	0.12
			Sdev	2.6	0.065	5.5	0.6	10.1	1.5	0.023
200 mg/kg/day										
	2515	3/1	-7	22.	0.91	34.	36.	89.	11.	0.10
	2523	3/1	-7	25.	0.85	46.	36.	51.	8.	0.24
	2526	3/1	-7	22.	0.83	25.	22.	94.	5.	0.11
			N	3	3	3	3	3	3	3
			Mean	23.	0.86	35.	31.	78.	8.	0.15
			Sdev	1.7	0.042	10.5	8.1	23.5	3.0	0.078
800 mg/kg/day										
	2517	4/1	-7	26.	0.80	30.	36.	67.	9.	0.09
	2519	4/1	-7	23.	0.92	33.	24.	93.	6.	0.10
	2525	4/1	-7	23.	0.90	34.	26.	84.	8.	0.09
	2528	4/1	-7	28.	0.85	45.	35.	74.	9.	0.10
	2530	4/1	-7	20.	0.90	48.	37.	49.	5.	0.09
			N	5	5	5	5	5	5	5
			Mean	24.	0.87	38.	32.	73.	7.	0.09
			Sdev	3.1	0.049	8.0	6.1	16.8	1.8	0.005

UREA - UREA
CREA - CREATININE
AST - ASPARTATE AMINO TRANSFERASE
ALT - ALANINE AMINO TRANSFERASE
AP - ALKALINE PHOSPHATASE
GGT - GAMMA GLUTAMYL TRANSFERASE
TBIL - TOTAL BILIRUBIN

Appendix 8
Day -7 Clinical Chemistry Data
Pretest phase

Session 1 (Scheduled)
Fexinidazole
Study Number: 0505-2007

Males

Dose Level	Animal Number	Group/ Subgroup	Study Day	TPRO g/dL	ALB g/dL	GLOB g/dL	GLUC mg/dL	TG mg/dL	TCHO mg/dL
Vehicle									
	2516	1/1	-7	5.6	2.74	2.9	85.	19.	112.
	2518	1/1	-7	6.1	3.03	3.1	90.	17.	146.
	2520	1/1	-7	6.0	2.85	3.2	88.	9.	101.
	2527	1/1	-7	6.1	2.62	3.5	86.	23.	147.
	2533	1/1	-7	5.8	2.77	3.0	88.	23.	116.
			N	5	5	5	5	5	5
			Mean	5.9	2.80	3.1	87.	18.	124.
			Sdev	0.22	0.152	0.23	1.9	5.8	20.9
50 mg/kg/day									
	2514	2/1	-7	5.6	2.90	2.7	90.	8.	98.
	2521	2/1	-7	5.8	2.88	2.9	86.	35.	148.
	2529	2/1	-7	6.1	3.07	3.0	96.	24.	128.
			N	3	3	3	3	3	3
			Mean	5.8	2.95	2.9	91.	22.	125.
			Sdev	0.25	0.104	0.17	5.0	13.6	25.2
200 mg/kg/day									
	2515	3/1	-7	6.1	3.12	3.0	89.	17.	134.
	2523	3/1	-7	6.1	2.62	3.5	95.	22.	109.
	2526	3/1	-7	6.1	2.79	3.3	92.	14.	123.
			N	3	3	3	3	3	3
			Mean	6.1	2.84	3.3	92.	18.	122.
			Sdev	0.00	0.254	0.25	3.0	4.0	12.5
800 mg/kg/day									
	2517	4/1	-7	6.4	3.10	3.3	88.	8.	141.
	2519	4/1	-7	6.5	2.99	3.5	90.	12.	108.
	2525	4/1	-7	6.2	3.00	3.2	93.	20.	137.
	2528	4/1	-7	5.8	2.79	3.0	90.	18.	105.
	2530	4/1	-7	5.9	2.85	3.1	111.	10.	94.
			N	5	5	5	5	5	5
			Mean	6.2	2.95	3.2	94.	14.	117.
			Sdev	0.30	0.125	0.20	9.4	5.2	20.8

TPRO - TOTAL PROTEIN
ALB - ALBUMIN
GLOB - GLOBULIN
GLUC - GLUCOSE
TG - TRIGLYCERIDES
TCHO - TOTAL CHOLESTEROL

Appendix 8
Day -7 Clinical Chemistry Data
Pretest phase

Session 1 (Scheduled)
Fexinidazole

Study Number: 0505-2007

M a l e s

Dose Level	Animal Number	Group/ Subgroup	Study Day	CA mg/dL	PHOS mg/dL	AG	NA mEq/L	K mEq/L	CL mEq/L
Vehicle									
	2516	1/1	-7	10.6	5.5	1.0	140.0	5.0	109.0
	2518	1/1	-7	10.9	4.7	1.0	142.0	4.5	110.0
	2520	1/1	-7	11.0	4.6	0.9	140.0	4.6	109.0
	2527	1/1	-7	11.4	5.2	0.8	141.0	4.6	107.0
	2533	1/1	-7	10.2	5.0	0.9	143.0	4.6	111.0
			N	5	5	5	5	5	5
			Mean	10.8	5.0	0.9	141.2	4.7	109.2
			Sdev	0.45	0.37	0.09	1.30	0.18	1.48
50 mg/kg/day									
	2514	2/1	-7	11.0	4.9	1.1	143.0	4.8	110.0
	2521	2/1	-7	10.7	5.0	1.0	142.0	4.4	108.0
	2529	2/1	-7	10.2	4.4	1.0	142.0	4.8	109.0
			N	3	3	3	3	3	3
			Mean	10.6	4.8	1.0	142.3	4.7	109.0
			Sdev	0.40	0.32	0.04	0.58	0.25	1.00
200 mg/kg/day									
	2515	3/1	-7	11.4	4.5	1.0	143.0	4.6	108.0
	2523	3/1	-7	10.1	3.8	0.8	143.0	4.7	114.0
	2526	3/1	-7	11.0	4.4	0.8	139.0	4.6	108.0
			N	3	3	3	3	3	3
			Mean	10.8	4.2	0.9	141.7	4.6	110.0
			Sdev	0.67	0.38	0.15	2.31	0.08	3.46
800 mg/kg/day									
	2517	4/1	-7	10.9	4.2	0.9	142.0	4.8	108.0
	2519	4/1	-7	11.0	5.2	0.9	142.0	4.5	109.0
	2525	4/1	-7	11.1	5.1	0.9	140.0	4.6	108.0
	2528	4/1	-7	10.4	4.7	0.9	141.0	4.7	111.0
	2530	4/1	-7	10.3	4.5	0.9	142.0	4.5	113.0
			N	5	5	5	5	5	5
			Mean	10.7	4.7	0.9	141.4	4.6	109.8
			Sdev	0.36	0.42	0.04	0.89	0.14	2.17

CA - CALCIUM
PHOS - PHOSPHOROUS
AG - ALBUMIN/GLOBULIN
NA - SODIUM
K - POTASSIUM
CL - CHLORIDE

Appendix 8
Day -7 Clinical Chemistry Data
Pretest phase

Session 1 (Scheduled)
Fexinidazole

Study Number: 0505-2007

Females

Dose Level	Animal Number	Group/ Subgroup	Study Day	UREA mg/dL	CREA mg/dL	AST IU/L	ALT IU/L	AP IU/L	GGT IU/L	TBIL mg/dL
Vehicle										
	2560	1/1	-7	26.	0.78	39.	28.	46.	11.	0.10
	2568	1/1	-7	29.	0.79	54.	31.	53.	6.	0.25
	2572	1/1	-7	24.	0.81	39.	25.	51.	8.	0.12
	2575	1/1	-7	29.	0.71	37.	28.	87.	9.	0.13
	2577	1/1	-7	23.	1.01	34.	31.	50.	7.	0.12
			N	5	5	5	5	5	5	5
			Mean	26.	0.82	41.	29.	57.	8.	0.14
			Sdev	2.8	0.113	7.8	2.5	16.7	1.9	0.060
50 mg/kg/day										
	2562	2/1	-7	30.	0.79	38.	30.	77.	11.	0.21
	2563	2/1	-7	26.	0.86	32.	20.	54.	10.	0.11
	2576	2/1	-7	37.	0.88	41.	38.	78.	8.	0.10
			N	3	3	3	3	3	3	3
			Mean	31.	0.84	37.	29.	70.	10.	0.14
			Sdev	5.6	0.047	4.6	9.0	13.6	1.5	0.061
200 mg/kg/day										
	2561	3/1	-7	32.	0.73	28.	21.	52.	7.	0.12
	2570	3/1	-7	30.	0.79	41.	25.	72.	7.	0.12
	2567	3/1	-7	23.	0.79	39.	24.	81.	8.	0.13
			N	3	3	3	3	3	3	3
			Mean	28.	0.77	36.	23.	68.	7.	0.12
			Sdev	4.7	0.035	7.0	2.1	14.8	0.6	0.006
800 mg/kg/day										
	2564	4/1	-7	28.	0.92	38.	28.	47.	8.	0.13
	2565	4/1	-7	46.	0.84	29.	23.	51.	8.	0.21
	2569	4/1	-7	30.	1.02	39.	36.	74.	7.	0.12
	2571	4/1	-7	28.	0.88	35.	39.	94.	8.	0.15
	2573	4/1	-7	24.	0.80	28.	25.	46.	8.	0.20
			N	5	5	5	5	5	5	5
			Mean	31.	0.89	34.	30.	62.	8.	0.16
			Sdev	8.6	0.084	5.1	7.0	21.0	0.4	0.041

UREA - UREA
CREA - CREATININE
AST - ASPARTATE AMINO TRANSFERASE
ALT - ALANINE AMINO TRANSFERASE
AP - ALKALINE PHOSPHATASE
GGT - GAMMA GLUTAMYL TRANSFERASE
TBIL - TOTAL BILIRUBIN

Appendix 8
Day -7 Clinical Chemistry Data
Pretest phase

Session 1 (Scheduled)
Fexinidazole
Study Number: 0505-2007

Females

Dose Level	Animal Number	Group/ Subgroup	Study Day	TPRO g/dL	ALB g/dL	GLOB g/dL	GLUC mg/dL	TG mg/dL	TCHO mg/dL
Vehicle									
	2560	1/1	-7	5.8	2.57	3.2	79.	16.	109.
	2568	1/1	-7	5.9	3.03	2.9	96.	11.	108.
	2572	1/1	-7	5.6	2.95	2.7	104.	14.	111.
	2575	1/1	-7	6.1	2.89	3.2	86.	19.	130.
	2577	1/1	-7	5.5	2.89	2.6	116.	10.	102.
			N	5	5	5	5	5	5
			Mean	5.8	2.87	2.9	96.	14.	112.
			Sdev	0.24	0.175	0.30	14.6	3.7	10.6
50 mg/kg/day									
	2562	2/1	-7	5.8	3.09	2.7	66.	34.	116.
	2563	2/1	-7	5.8	2.58	3.2	104.	38.	170.
	2576	2/1	-7	5.8	2.98	2.8	93.	18.	122.
			N	3	3	3	3	3	3
			Mean	5.8	2.88	2.9	88.	30.	136.
			Sdev	0.00	0.268	0.27	19.6	10.6	29.6
200 mg/kg/day									
	2561	3/1	-7	6.1	2.90	3.2	90.	28.	128.
	2570	3/1	-7	6.0	3.08	2.9	112.	17.	132.
	2567	3/1	-7	5.7	2.77	2.9	93.	21.	132.
			N	3	3	3	3	3	3
			Mean	5.9	2.92	3.0	98.	22.	131.
			Sdev	0.21	0.156	0.16	11.9	5.6	2.3
800 mg/kg/day									
	2564	4/1	-7	5.9	2.74	3.2	106.	18.	148.
	2565	4/1	-7	6.1	2.79	3.3	99.	20.	156.
	2569	4/1	-7	5.6	2.99	2.6	108.	12.	104.
	2571	4/1	-7	5.7	3.01	2.7	95.	32.	145.
	2573	4/1	-7	6.0	2.98	3.0	94.	22.	120.
			N	5	5	5	5	5	5
			Mean	5.9	2.90	3.0	100.	21.	135.
			Sdev	0.21	0.127	0.30	6.3	7.3	21.8

TPRO - TOTAL PROTEIN
ALB - ALBUMIN
GLOB - GLOBULIN
GLUC - GLUCOSE
TG - TRIGLYCERIDES
TCHO - TOTAL CHOLESTEROL

Appendix 8
Day -7 Clinical Chemistry Data
Pretest phase

Session 1 (Scheduled)
Fexinidazole
Study Number: 0505-2007

Females

Dose Level	Animal Number	Group/ Subgroup	Study Day	CA mg/dL	PHOS mg/dL	AG	NA mEq/L	K mEq/L	CL mEq/L
Vehicle									
	2560	1/1	-7	10.1	4.2	0.8	140.0	4.5	110.0
	2568	1/1	-7	10.7	4.9	1.1	140.0	4.2	109.0
	2572	1/1	-7	10.4	3.6	1.1	143.0	4.3	108.0
	2575	1/1	-7	10.5	5.1	0.9	142.0	4.6	106.0
	2577	1/1	-7	10.5	4.4	1.1	144.0	4.1	107.0
			N	5	5	5	5	5	5
			Mean	10.4	4.4	1.0	141.8	4.3	108.0
			Sdev	0.22	0.59	0.14	1.79	0.20	1.58
50 mg/kg/day									
	2562	2/1	-7	10.7	5.0	1.1	142.0	4.7	106.0
	2563	2/1	-7	10.7	4.9	0.8	142.0	4.7	105.0
	2576	2/1	-7	10.7	4.7	1.1	144.0	4.6	107.0
			N	3	3	3	3	3	3
			Mean	10.7	4.9	1.0	142.7	4.7	106.0
			Sdev	0.00	0.15	0.18	1.15	0.10	1.00
200 mg/kg/day									
	2561	3/1	-7	10.7	4.7	0.9	142.0	4.7	108.0
	2570	3/1	-7	10.8	4.7	1.1	144.0	4.3	109.0
	2567	3/1	-7	10.2	4.4	0.9	140.0	4.1	107.0
			N	3	3	3	3	3	3
			Mean	10.6	4.6	1.0	142.0	4.4	108.0
			Sdev	0.32	0.17	0.08	2.00	0.27	1.00
800 mg/kg/day									
	2564	4/1	-7	10.9	5.5	0.9	143.0	4.4	107.0
	2565	4/1	-7	11.3	5.1	0.8	144.0	4.1	102.0
	2569	4/1	-7	10.7	3.6	1.1	142.0	4.4	110.0
	2571	4/1	-7	10.8	3.7	1.1	144.0	4.6	108.0
	2573	4/1	-7	10.8	4.7	1.0	142.0	4.4	105.0
			N	5	5	5	5	5	5
			Mean	10.9	4.5	1.0	143.0	4.4	106.4
			Sdev	0.23	0.84	0.14	1.00	0.16	3.05

CA - CALCIUM
PHOS - PHOSPHOROUS
AG - ALBUMIN/GLOBULIN
NA - SODIUM
K - POTASSIUM
CL - CHLORIDE

Appendix 8
Day 28 Clinical Chemistry Data
Test period

Session 1 (Scheduled)
Fexinidazole
Study Number: 0505-2007

Males

Dose Level	Animal Number	Group/ Subgroup	Study Day	UREA mg/dL	CREA mg/dL	AST IU/L	ALT IU/L	AP IU/L	GGT IU/L	TBIL mg/dL
Vehicle										
	2516	1/1	28	28.	0.90	33.	50.	72.	3.	0.30
	2518	1/1	28	31.	0.81	46.	57.	80.	6.	0.24
	2520	1/1	28	30.	0.95	29.	26.	57.	2.	0.27
	2527	1/1	28	28.	0.88	37.	32.	66.	2.	0.21
	2533	1/1	28	31.	0.95	41.	38.	64.	5.	0.24
			N	5	5	5	5	5	5	5
			Mean	30.	0.90	37.	41.	68.	4.	0.25
			Sdev	1.5	0.058	6.6	12.8	8.7	1.8	0.034
50 mg/kg/day										
	2514	2/1	28	23.	0.87	35.	39.	83.	6.	0.21
	2521	2/1	28	26.	0.89	31.	38.	62.	5.	0.28
	2529	2/1	28	23.	0.86	33.	26.	83.	5.	0.26
			N	3	3	3	3	3	3	3
			Mean	24.	0.87	33.	34.	76.	5.	0.25
			Sdev	1.7	0.015	2.0	7.2	12.1	0.6	0.036
200 mg/kg/day										
	2515	3/1	28	25.	0.97	31.	32.	99.	5.	0.23
	2523	3/1	28	34.	0.78	39.	30.	61.	6.	0.22
	2526	3/1	28	22.	0.80	24.	27.	85.	6.	0.26
			N	3	3	3	3	3	3	3
			Mean	27.	0.85	31.	30.	82.	6.	0.24
			Sdev	6.2	0.104	7.5	2.5	19.2	0.6	0.021
800 mg/kg/day										
	2517	4/1	28	43.	0.78	31.	49.	87.	9.	0.32
	2519	4/1	28	34.	0.94	23.	25.	116.	6.	0.20
	2525	4/1	28	32.	1.03	30.	35.	151.	7.	0.28
	2528	4/1	28	29.	1.03	31.	32.	80.	12.	0.25
	2530	4/1	28	25.	0.87	41.	40.	66.	6.	0.32
			N	5	5	5	5	5	5	5
			Mean	33.	0.93	31.	36.	100.	8.	0.27
			Sdev	6.7	0.107	6.4	9.0	33.8	2.5	0.051

UREA - UREA
CREA - CREATININE
AST - ASPARTATE AMINO TRANSFERASE
ALT - ALANINE AMINO TRANSFERASE
AP - ALKALINE PHOSPHATASE
GGT - GAMMA GLUTAMYL TRANSFERASE
TBIL - TOTAL BILIRUBIN

Appendix 8
Day 28 Clinical Chemistry Data
Test period

Session 1 (Scheduled)
Fexinidazole
Study Number: 0505-2007

Males

Dose Level	Animal Number	Group/ Subgroup	Study Day	TPRO g/dL	ALB g/dL	GLOB g/dL	GLUC mg/dL	TG mg/dL	TCHO mg/dL
Vehicle									
	2516	1/1	28	5.3	2.82	2.5	97.	21.	112.
	2518	1/1	28	5.6	2.92	2.7	90.	28.	132.
	2520	1/1	28	5.7	2.92	2.8	85.	16.	110.
	2527	1/1	28	5.3	2.57	2.7	99.	22.	137.
	2533	1/1	28	5.0	2.48	2.5	87.	29.	92.
			N	5	5	5	5	5	5
			Mean	5.4	2.74	2.6	92.	23.	117.
			Sdev	0.28	0.205	0.13	6.1	5.4	18.2
50 mg/kg/day									
	2514	2/1	28	5.5	3.05	2.5	98.	21.	113.
	2521	2/1	28	5.5	2.94	2.6	94.	39.	138.
	2529	2/1	28	5.2	2.86	2.3	93.	18.	127.
			N	3	3	3	3	3	3
			Mean	5.4	2.95	2.5	95.	26.	126.
			Sdev	0.17	0.095	0.11	2.6	11.4	12.5
200 mg/kg/day									
	2515	3/1	28	5.4	3.05	2.4	92.	31.	155.
	2523	3/1	28	5.0	2.35	2.7	98.	23.	133.
	2526	3/1	28	5.6	2.85	2.8	106.	26.	134.
			N	3	3	3	3	3	3
			Mean	5.3	2.75	2.6	99.	27.	141.
			Sdev	0.31	0.361	0.21	7.0	4.0	12.4
800 mg/kg/day									
	2517	4/1	28	5.2	2.87	2.3	88.	25.	169.
	2519	4/1	28	5.4	2.79	2.6	86.	22.	125.
	2525	4/1	28	5.6	3.15	2.5	89.	28.	172.
	2528	4/1	28	5.3	2.76	2.5	95.	35.	193.
	2530	4/1	28	5.3	2.82	2.5	107.	28.	116.
			N	5	5	5	5	5	5
			Mean	5.4	2.88	2.5	93.	28.	155.
			Sdev	0.15	0.157	0.10	8.5	4.8	33.0

TPRO - TOTAL PROTEIN
ALB - ALBUMIN
GLOB - GLOBULIN
GLUC - GLUCOSE
TG - TRIGLYCERIDES
TCHO - TOTAL CHOLESTEROL

Appendix 8
Day 28 Clinical Chemistry Data
Test period

Session 1 (Scheduled)
Fexinidazole

Study Number: 0505-2007

M a l e s

Dose Level	Animal Number	Group/ Subgroup	Study Day	CA mg/dL	PHOS mg/dL	AG	NA mEq/L	K mEq/L	CL mEq/L
Vehicle									
	2516	1/1	28	10.5	4.6	1.1	142.0	4.6	112.0
	2518	1/1	28	10.5	4.9	1.1	141.0	4.6	111.0
	2520	1/1	28	10.7	3.9	1.1	141.0	4.5	108.0
	2527	1/1	28	10.5	4.3	0.9	141.0	4.3	107.0
	2533	1/1	28	9.3	4.0	1.0	141.0	4.6	113.0
			N	5	5	5	5	5	5
			Mean	10.3	4.3	1.0	141.2	4.5	110.2
			Sdev	0.57	0.42	0.08	0.45	0.12	2.59
50 mg/kg/day									
	2514	2/1	28	10.4	4.3	1.2	143.0	4.5	111.0
	2521	2/1	28	10.3	4.4	1.1	142.0	4.4	110.0
	2529	2/1	28	10.2	4.4	1.2	142.0	4.2	110.0
			N	3	3	3	3	3	3
			Mean	10.3	4.4	1.2	142.3	4.3	110.3
			Sdev	0.10	0.06	0.05	0.58	0.16	0.58
200 mg/kg/day									
	2515	3/1	28	10.4	3.9	1.3	144.0	4.4	112.0
	2523	3/1	28	9.5	3.3	0.9	142.0	4.6	115.0
	2526	3/1	28	10.9	4.5	1.0	141.0	4.2	107.0
			N	3	3	3	3	3	3
			Mean	10.3	3.9	1.1	142.3	4.4	111.3
			Sdev	0.71	0.60	0.21	1.53	0.21	4.04
800 mg/kg/day									
	2517	4/1	28	9.4	3.9	1.2	141.0	4.5	111.0
	2519	4/1	28	10.4	4.2	1.1	143.0	4.1	109.0
	2525	4/1	28	10.7	5.2	1.3	144.0	4.6	111.0
	2528	4/1	28	10.2	4.4	1.1	142.0	4.3	115.0
	2530	4/1	28	10.1	3.8	1.1	141.0	4.5	112.0
			N	5	5	5	5	5	5
			Mean	10.2	4.3	1.2	142.2	4.4	111.6
			Sdev	0.48	0.56	0.09	1.30	0.19	2.19

CA - CALCIUM
PHOS - PHOSPHOROUS
AG - ALBUMIN/GLOBULIN
NA - SODIUM
K - POTASSIUM
CL - CHLORIDE

Appendix 8
Day 28 Clinical Chemistry Data
Test period

Session 1 (Scheduled)
Fexinidazole

Study Number: 0505-2007

Females

Dose Level	Animal Number	Group/ Subgroup	Study Day	UREA mg/dL	CREA mg/dL	AST IU/L	ALT IU/L	AP IU/L	GGT IU/L	TBIL mg/dL
Vehicle										
	2560	1/1	28	21.	0.79	45.	36.	48.	3.	0.22
	2568	1/1	28	26.	0.91	34.	38.	55.	5.	0.12
	2572	1/1	28	23.	0.86	29.	35.	66.	4.	0.09
	2575	1/1	28	22.	0.91	31.	33.	93.	5.	0.10
	2577	1/1	28	22.	0.98	35.	34.	62.	3.	0.11
			N	5	5	5	5	5	5	5
			Mean	23.	0.89	35.	35.	65.	4.	0.13
			Sdev	1.9	0.070	6.2	1.9	17.2	1.0	0.053
50 mg/kg/day										
	2562	2/1	28	31.	0.88	30.	38.	92.	6.	0.10
	2563	2/1	28	22.	0.88	33.	25.	58.	4.	0.10
	2576	2/1	28	28.	0.90	39.	40.	76.	7.	0.21
			N	3	3	3	3	3	3	3
			Mean	27.	0.89	34.	34.	75.	6.	0.14
			Sdev	4.6	0.012	4.6	8.1	17.0	1.5	0.064
200 mg/kg/day										
	2561	3/1	28	31.	0.84	47.	30.	60.	8.	0.11
	2570	3/1	28	26.	0.81	34.	35.	77.	6.	0.11
	2567	3/1	28	23.	0.88	40.	23.	96.	7.	0.10
			N	3	3	3	3	3	3	3
			Mean	27.	0.84	40.	29.	78.	7.	0.11
			Sdev	4.0	0.035	6.5	6.0	18.0	1.0	0.006
800 mg/kg/day										
	2564	4/1	28	23.	0.83	31.	26.	66.	8.	0.12
	2565	4/1	28	51.	0.92	25.	28.	58.	7.	0.32
	2569	4/1	28	30.	1.03	33.	57.	101.	9.	0.20
	2571	4/1	28	29.	0.98	31.	59.	81.	7.	0.12
	2573	4/1	28	26.	0.88	23.	25.	62.	6.	0.12
			N	5	5	5	5	5	5	5
			Mean	32.	0.93	29.	39.	74.	7.	0.18
			Sdev	11.1	0.079	4.3	17.4	17.6	1.1	0.088

UREA - UREA
CREA - CREATININE
AST - ASPARTATE AMINO TRANSFERASE
ALT - ALANINE AMINO TRANSFERASE
AP - ALKALINE PHOSPHATASE
GGT - GAMMA GLUTAMYL TRANSFERASE
TBIL - TOTAL BILIRUBIN

Appendix 8
Day 28 Clinical Chemistry Data
Test period

Session 1 (Scheduled)
Fexinidazole
Study Number: 0505-2007

Females

Dose Level	Animal Number	Group/ Subgroup	Study Day	TPRO g/dL	ALB g/dL	GLOB g/dL	GLUC mg/dL	TG mg/dL	TCHO mg/dL
Vehicle									
	2560	1/1	28	5.6	2.52	3.1	87.	20.	97.
	2568	1/1	28	5.3	2.94	2.4	79.	23.	93.
	2572	1/1	28	5.3	2.91	2.4	101.	17.	113.
	2575	1/1	28	5.8	2.87	2.9	91.	23.	140.
	2577	1/1	28	5.8	2.99	2.8	103.	24.	111.
			N	5	5	5	5	5	5
			Mean	5.6	2.85	2.7	92.	21.	111.
			Sdev	0.25	0.187	0.32	10.0	2.9	18.5
50 mg/kg/day									
	2562	2/1	28	5.6	3.00	2.6	75.	43.	107.
	2563	2/1	28	5.2	2.67	2.5	111.	45.	186.
	2576	2/1	28	5.7	3.01	2.7	91.	20.	129.
			N	3	3	3	3	3	3
			Mean	5.5	2.89	2.6	92.	36.	141.
			Sdev	0.26	0.193	0.08	18.0	13.9	40.8
200 mg/kg/day									
	2561	3/1	28	5.5	2.92	2.6	108.	37.	181.
	2570	3/1	28	5.5	2.95	2.6	105.	44.	128.
	2567	3/1	28	5.3	2.72	2.6	84.	36.	120.
			N	3	3	3	3	3	3
			Mean	5.4	2.86	2.6	99.	39.	143.
			Sdev	0.12	0.125	0.02	13.1	4.4	33.2
800 mg/kg/day									
	2564	4/1	28	5.4	2.74	2.7	106.	25.	232.
	2565	4/1	28	5.9	3.01	2.9	104.	38.	200.
	2569	4/1	28	5.4	3.00	2.4	118.	45.	122.
	2571	4/1	28	5.4	2.99	2.4	105.	34.	177.
	2573	4/1	28	6.1	3.05	3.1	97.	25.	171.
			N	5	5	5	5	5	5
			Mean	5.6	2.96	2.7	106.	33.	180.
			Sdev	0.34	0.124	0.29	7.6	8.6	40.5

TPRO - TOTAL PROTEIN
ALB - ALBUMIN
GLOB - GLOBULIN
GLUC - GLUCOSE
TG - TRIGLYCERIDES
TCHO - TOTAL CHOLESTEROL

Appendix 8
Day 28 Clinical Chemistry Data
Test period

Session 1 (Scheduled)
Fexinidazole
Study Number: 0505-2007

Females

Dose Level	Animal Number	Group/ Subgroup	Study Day	CA mg/dL	PHOS mg/dL	AG	NA mEq/L	K mEq/L	CL mEq/L
Vehicle									
	2560	1/1	28	9.6	3.8	0.8	141.0	4.6	113.0
	2568	1/1	28	10.6	4.6	1.2	142.0	4.8	109.0
	2572	1/1	28	10.1	3.5	1.2	142.0	4.3	112.0
	2575	1/1	28	10.3	4.0	1.0	142.0	4.4	111.0
	2577	1/1	28	11.0	4.1	1.1	143.0	4.2	108.0
			N	5	5	5	5	5	5
			Mean	10.3	4.0	1.1	142.0	4.5	110.6
			Sdev	0.53	0.41	0.18	0.71	0.24	2.07
50 mg/kg/day									
	2562	2/1	28	10.5	4.3	1.2	141.0	4.4	109.0
	2563	2/1	28	10.4	4.2	1.1	143.0	4.2	109.0
	2576	2/1	28	11.1	4.5	1.1	143.0	4.4	111.0
			N	3	3	3	3	3	3
			Mean	10.7	4.3	1.1	142.3	4.3	109.7
			Sdev	0.38	0.15	0.05	1.15	0.14	1.15
200 mg/kg/day									
	2561	3/1	28	10.8	3.1	1.1	144.0	4.2	112.0
	2570	3/1	28	9.9	4.7	1.2	143.0	4.7	113.0
	2567	3/1	28	10.2	3.9	1.1	142.0	4.2	110.0
			N	3	3	3	3	3	3
			Mean	10.3	3.9	1.1	143.0	4.4	111.7
			Sdev	0.46	0.80	0.05	1.00	0.27	1.53
800 mg/kg/day									
	2564	4/1	28	10.6	3.9	1.0	145.0	4.2	112.0
	2565	4/1	28	11.5	4.4	1.0	146.0	4.0	104.0
	2569	4/1	28	10.6	3.7	1.3	145.0	4.4	114.0
	2571	4/1	28	10.9	3.6	1.2	142.0	4.3	111.0
	2573	4/1	28	10.7	4.1	1.0	143.0	4.4	108.0
			N	5	5	5	5	5	5
			Mean	10.9	3.9	1.1	144.2	4.3	109.8
			Sdev	0.38	0.32	0.12	1.64	0.18	3.90

CA - CALCIUM
PHOS - PHOSPHOROUS
AG - ALBUMIN/GLOBULIN
NA - SODIUM
K - POTASSIUM
CL - CHLORIDE

Appendix 8
Day 42 Clinical Chemistry Data
Test period

Session 1 (Scheduled)
Fexinidazole

Study Number: 0505-2007

Males

Dose Level	Animal Number	Group/ Subgroup	Study Day	UREA mg/dL	CREA mg/dL	AST IU/L	ALT IU/L	AP IU/L	GGT IU/L	TBIL mg/dL
Vehicle										
	2527	1/1	42	42.	0.91	31.	23.	56.	5.	0.07
	2533	1/1	42	32.	0.85	44.	41.	61.	8.	0.08
			N	2	2	2	2	2	2	2
			Mean	37.	0.88	38.	32.	59.	7.	0.08
			Sdev	7.1	0.042	9.2	12.7	3.5	2.1	0.007
800 mg/kg/day										
	2528	4/1	42	24.	0.81	36.	58.	78.	13.	0.09
	2530	4/1	42	25.	0.73	44.	27.	63.	7.	0.07
			N	2	2	2	2	2	2	2
			Mean	25.	0.77	40.	43.	71.	10.	0.08
			Sdev	0.7	0.057	5.7	21.9	10.6	4.2	0.014

UREA - UREA
CREA - CREATININE
AST - ASPARTATE AMINO TRANSFERASE
ALT - ALANINE AMINO TRANSFERASE
AP - ALKALINE PHOSPHATASE
GGT - GAMMA GLUTAMYL TRANSFERASE
TBIL - TOTAL BILIRUBIN

Appendix 8
Day 42 Clinical Chemistry Data
Test period

Session 1 (Scheduled)
Fexinidazole

Study Number: 0505-2007

Males

Dose Level	Animal Number	Group/ Subgroup	Study Day	TPRO g/dL	ALB g/dL	GLOB g/dL	GLUC mg/dL	TG mg/dL	TCHO mg/dL
Vehicle									
	2527	1/1	42	5.8	2.57	3.2	105.	33.	148.
	2533	1/1	42	5.6	2.55	3.1	93.	24.	85.
			N	2	2	2	2	2	2
			Mean	5.7	2.56	3.1	99.	29.	117.
			Sdev	0.14	0.014	0.13	8.5	6.4	44.5
800 mg/kg/day									
	2528	4/1	42	6.1	2.89	3.2	82.	27.	141.
	2530	4/1	42	6.2	2.94	3.3	100.	18.	91.
			N	2	2	2	2	2	2
			Mean	6.2	2.92	3.2	91.	23.	116.
			Sdev	0.07	0.035	0.04	12.7	6.4	35.4

TPRO - TOTAL PROTEIN
ALB - ALBUMIN
GLOB - GLOBULIN
GLUC - GLUCOSE
TG - TRIGLYCERIDES
TCHO - TOTAL CHOLESTEROL

Appendix 8
Day 42 Clinical Chemistry Data
Test period

Session 1 (Scheduled)
Fexinidazole
Study Number: 0505-2007

Males

Dose Level	Animal Number	Group/ Subgroup	Study Day	CA mg/dL	PHOS mg/dL	AG	NA mEq/L	K mEq/L	CL mEq/L
Vehicle									
	2527	1/1	42	11.0	5.0	0.8	142.0	4.4	109.0
	2533	1/1	42	9.6	4.5	0.8	141.0	4.6	114.0
			N	2	2	2	2	2	2
			Mean	10.3	4.8	0.8	141.5	4.5	111.5
			Sdev	0.99	0.35	0.03	0.71	0.11	3.54
800 mg/kg/day									
	2528	4/1	42	10.9	4.0	0.9	142.0	4.6	108.0
	2530	4/1	42	10.5	4.7	0.9	142.0	4.3	109.0
			N	2	2	2	2	2	2
			Mean	10.7	4.4	0.9	142.0	4.5	108.5
			Sdev	0.28	0.49	0.00	0.00	0.17	0.71

CA - CALCIUM
PHOS - PHOSPHOROUS
AG - ALBUMIN/GLOBULIN
NA - SODIUM
K - POTASSIUM
CL - CHLORIDE

Appendix 8
Day 42 Clinical Chemistry Data
Test period

Session 1 (Scheduled)
Fexinidazole

Study Number: 0505-2007

Females

Dose Level	Animal Number	Group/ Subgroup	Study Day	UREA mg/dL	CREA mg/dL	AST IU/L	ALT IU/L	AP IU/L	GGT IU/L	TBIL mg/dL
Vehicle										
	2575	1/1	42	22.	0.93	33.	26.	106.	7.	0.07
	2577	1/1	42	29.	0.98	38.	36.	53.	6.	0.08
			N	2	2	2	2	2	2	2
			Mean	26.	0.96	36.	31.	80.	7.	0.08
			Sdev	4.9	0.035	3.5	7.1	37.5	0.7	0.007
800 mg/kg/day										
	2571	4/1	42	31.	0.94	22.	38.	75.	8.	0.07
	2573	4/1	42	25.	0.87	25.	29.	62.	7.	0.09
			N	2	2	2	2	2	2	2
			Mean	28.	0.91	24.	34.	69.	8.	0.08
			Sdev	4.2	0.049	2.1	6.4	9.2	0.7	0.014

UREA - UREA
CREA - CREATININE
AST - ASPARTATE AMINO TRANSFERASE
ALT - ALANINE AMINO TRANSFERASE
AP - ALKALINE PHOSPHATASE
GGT - GAMMA GLUTAMYL TRANSFERASE
TBIL - TOTAL BILIRUBIN

Appendix 8
Day 42 Clinical Chemistry Data
Test period

Session 1 (Scheduled)
Fexinidazole

Study Number: 0505-2007

Females

Dose Level	Animal Number	Group/ Subgroup	Study Day	TPRO g/dL	ALB g/dL	GLOB g/dL	GLUC mg/dL	TG mg/dL	TCHO mg/dL
Vehicle									
	2575	1/1	42	6.0	2.91	3.1	93.	27.	135.
	2577	1/1	42	5.7	2.87	2.8	101.	38.	111.
			N	2	2	2	2	2	2
			Mean	5.9	2.89	3.0	97.	33.	123.
			Sdev	0.21	0.028	0.18	5.7	7.8	17.0
800 mg/kg/day									
	2571	4/1	42	5.8	2.93	2.9	115.	37.	187.
	2573	4/1	42	5.7	2.90	2.8	94.	20.	130.
			N	2	2	2	2	2	2
			Mean	5.8	2.92	2.8	105.	29.	159.
			Sdev	0.07	0.021	0.05	14.8	12.0	40.3

TPRO - TOTAL PROTEIN
ALB - ALBUMIN
GLOB - GLOBULIN
GLUC - GLUCOSE
TG - TRIGLYCERIDES
TCHO - TOTAL CHOLESTEROL

Appendix 8
Day 42 Clinical Chemistry Data
Test period

Session 1 (Scheduled)
Fexinidazole
Study Number: 0505-2007

Females

Dose Level	Animal Number	Group/ Subgroup	Study Day	CA mg/dL	PHOS mg/dL	AG	NA mEq/L	K mEq/L	CL mEq/L
Vehicle									
	2575	1/1	42	10.8	4.6	0.9	140.0	4.5	112.0
	2577	1/1	42	11.1	4.7	1.0	142.0	4.3	110.0
			N	2	2	2	2	2	2
			Mean	11.0	4.7	1.0	141.0	4.4	111.0
			Sdev	0.21	0.07	0.05	1.41	0.12	1.41
800 mg/kg/day									
	2571	4/1	42	11.1	4.3	1.0	140.0	4.5	111.0
	2573	4/1	42	10.6	4.6	1.0	140.0	4.6	108.0
			N	2	2	2	2	2	2
			Mean	10.9	4.5	1.0	140.0	4.5	109.5
			Sdev	0.35	0.21	0.01	0.00	0.03	2.12

CA - CALCIUM
PHOS - PHOSPHOROUS
AG - ALBUMIN/GLOBULIN
NA - SODIUM
K - POTASSIUM
CL - CHLORIDE

Appendix 9 Urinalysis

Appendix 9
Day -7 Urine Data
Pretest phase

Session 1 (Scheduled)
Fexinidazole

Study Number: 0505-2007

M a l e s

Dose Level	Animal Number	Group/ Subgroup	Study Day	VOL mL	PH UNITS	WBC SCORE	NIT SCORE	PRO SCORE	GLU SCORE	KET SCORE
Vehicle										
	2516	1/1	-7	246.0	7.0	1.	0.	0.	0.	0.
	2518	1/1	-7	364.0	7.0	2.	0.	0.	0.	0.
	2520	1/1	-7	192.0	8.0	1.	0.	0.	0.	0.
	2527	1/1	-7	188.0	7.0	1.	0.	0.	0.	0.
	2533	1/1	-7	224.0	7.0	0.	1.	1.	3.	0.
			N	5	5	5	5	5	5	5
			Mean	242.8	7.2	1.	0.	0.	1.	0.
			Sdev	71.83	0.45	0.7	0.4	0.4	1.3	0.0
50 mg/kg/day										
	2514	2/1	-7	240.0	8.0	0.	0.	0.	0.	0.
	2521	2/1	-7	67.0	8.0	1.	0.	0.	0.	0.
	2529	2/1	-7	184.0	6.5	2.	0.	1.	0.	0.
			N	3	3	3	3	3	3	3
			Mean	163.7	7.5	1.	0.	0.	0.	0.
			Sdev	88.27	0.87	1.0	0.0	0.6	0.0	0.0
200 mg/kg/day										
	2515	3/1	-7	98.0	9.0	2.	0.	1.	0.	0.
	2523	3/1	-7	394.0	7.0	2.	0.	0.	0.	0.
	2526	3/1	-7	86.0	5.0	0.	0.	0.	2.	0.
			N	3	3	3	3	3	3	3
			Mean	192.7	7.0	1.	0.	0.	1.	0.
			Sdev	174.46	2.00	1.2	0.0	0.6	1.2	0.0
800 mg/kg/day										
	2517	4/1	-7	690.0	7.0	0.	0.	0.	0.	0.
	2519	4/1	-7	191.0	7.0	1.	1.	1.	0.	0.
	2525	4/1	-7	383.0	7.0	0.	0.	0.	0.	0.
	2528	4/1	-7	138.0	7.0	0.	0.	1.	0.	0.
	2530	4/1	-7	668.0	7.0	2.	0.	0.	0.	0.
			N	5	5	5	5	5	5	5
			Mean	414.0	7.0	1.	0.	0.	0.	0.
			Sdev	258.63	0.00	0.9	0.4	0.5	0.0	0.0

VOL - URINARY VOLUME
PH - PH
WBC - WHITE BLOOD CELLS
NIT - NITRITES
PRO - PROTEINS
GLU - GLUCOSE
KET - KETONE BODIES

Appendix 9
Day -7 Urine Data
Pretest phase

Session 1 (Scheduled)
Fexinidazole

Study Number: 0505-2007

M a l e s

Dose Level	Animal Number	Group/ Subgroup	Study Day	UBG SCORE	BIL SCORE	RBC SCORE	SG	COL	APP
Vehicle									
	2516	1/1	-7	0.	0.	0.	1.017	A	L
	2518	1/1	-7	0.	0.	1.	1.018	Y	T
	2520	1/1	-7	0.	0.	0.	1.014	Y	L
	2527	1/1	-7	0.	0.	0.	1.018	Y	L
	2533	1/1	-7	0.	0.	2.	1.017	A	LT
			N	5	5	5	5	0	0
			Mean	0.	0.	1.	1.017	-	-
			Sdev	0.0	0.0	0.9	0.0016	-	-
50 mg/kg/day									
	2514	2/1	-7	0.	0.	0.	1.026	A	L
	2521	2/1	-7	0.	0.	0.	1.014	W	L
	2529	2/1	-7	0.	0.	1.	1.024	A	L
			N	3	3	3	3	0	0
			Mean	0.	0.	0.	1.021	-	-
			Sdev	0.0	0.0	0.6	0.0064	-	-
200 mg/kg/day									
	2515	3/1	-7	0.	0.	0.	1.034	B	LT
	2523	3/1	-7	0.	0.	1.	1.018	Y	L
	2526	3/1	-7	0.	0.	1.	1.007	W	L
			N	3	3	3	3	0	0
			Mean	0.	0.	1.	1.020	-	-
			Sdev	0.0	0.0	0.6	0.0136	-	-
800 mg/kg/day									
	2517	4/1	-7	0.	0.	0.	1.011	Y	L
	2519	4/1	-7	0.	0.	1.	1.029	A	T
	2525	4/1	-7	0.	0.	0.	1.020	A	L
	2528	4/1	-7	0.	0.	1.	1.040	B	T
	2530	4/1	-7	0.	0.	1.	1.014	Y	T
			N	5	5	5	5	0	0
			Mean	0.	0.	1.	1.023	-	-
			Sdev	0.0	0.0	0.5	0.0118	-	-

UBG - UROBILINOGEN
BIL - BILIRUBIN
RBC - HEMOGLOBIN/RED BLOOD CELLS
SG - SPECIFIC GRAVITY
COL - COLOUR
APP - APPEARANCE

Appendix 9
Day -7 Urine Data
Pretest phase

Session 1 (Scheduled)
Fexinidazole

Study Number: 0505-2007

Females

Dose Level	Animal Number	Group/ Subgroup	Study Day	VOL mL	PH UNITS	WBC SCORE	NIT SCORE	PRO SCORE	GLU SCORE	KET SCORE
Vehicle										
	2560	1/1	-7	121.0	7.0	0.	0.	0.	0.	0.
	2568	1/1	-7	113.0	7.0	0.	0.	0.	0.	0.
	2572	1/1	-7	381.0	7.0	0.	0.	0.	0.	0.
	2575	1/1	-7	152.0	6.5	0.	0.	0.	0.	0.
	2577	1/1	-7	214.0	7.0	0.	0.	0.	0.	0.
			N	5	5	5	5	5	5	5
			Mean	196.2	6.9	0.	0.	0.	0.	0.
			Sdev	110.68	0.22	0.0	0.0	0.0	0.0	0.0
50 mg/kg/day										
	2562	2/1	-7	34.0	7.0	0.	0.	0.	0.	0.
	2563	2/1	-7	201.0	7.0	0.	0.	0.	0.	0.
	2576	2/1	-7	204.0	7.0	0.	0.	0.	0.	0.
			N	3	3	3	3	3	3	3
			Mean	146.3	7.0	0.	0.	0.	0.	0.
			Sdev	97.30	0.00	0.0	0.0	0.0	0.0	0.0
200 mg/kg/day										
	2561	3/1	-7	82.0	7.0	0.	0.	0.	0.	0.
	2570	3/1	-7	288.0	7.0	0.	0.	0.	0.	0.
	2567	3/1	-7	124.0	6.5	0.	0.	0.	0.	0.
			N	3	3	3	3	3	3	3
			Mean	164.7	6.8	0.	0.	0.	0.	0.
			Sdev	108.85	0.29	0.0	0.0	0.0	0.0	0.0
800 mg/kg/day										
	2564	4/1	-7	159.0	8.0	0.	0.	0.	0.	0.
	2565	4/1	-7	50.0	6.5	0.	1.	2.	4.	1.
	2569	4/1	-7	275.0	7.0	1.	1.	1.	0.	0.
	2571	4/1	-7	314.0	7.0	0.	0.	0.	0.	0.
	2573	4/1	-7	100.0	7.0	0.	0.	0.	0.	0.
			N	5	5	5	5	5	5	5
			Mean	179.6	7.1	0.	0.	1.	1.	0.
			Sdev	112.61	0.55	0.4	0.5	0.9	1.8	0.4

VOL - URINARY VOLUME
PH - PH
WBC - WHITE BLOOD CELLS
NIT - NITRITES
PRO - PROTEINS
GLU - GLUCOSE
KET - KETONE BODIES

Appendix 9
Day -7 Urine Data
Pretest phase

Session 1 (Scheduled)
Fexinidazole

Study Number: 0505-2007

Females

Dose Level	Animal Number	Group/ Subgroup	Study Day	UBG SCORE	BIL SCORE	RBC SCORE	SG	COL	APP
Vehicle									
	2560	1/1	-7	0.	0.	0.	1.034	A	LT
	2568	1/1	-7	0.	0.	0.	1.035	A	LT
	2572	1/1	-7	0.	0.	0.	1.016	Y	L
	2575	1/1	-7	0.	0.	0.	1.028	Y	L
	2577	1/1	-7	0.	0.	0.	1.020	Y	L
			N	5	5	5	5	0	0
			Mean	0.	0.	0.	1.027	-	-
			Sdev	0.0	0.0	0.0	0.0084	-	-
50 mg/kg/day									
	2562	2/1	-7	0.	0.	0.	1.021	Y	L
	2563	2/1	-7	0.	0.	0.	1.015	Y	L
	2576	2/1	-7	0.	0.	0.	1.022	Y	L
			N	3	3	3	3	0	0
			Mean	0.	0.	0.	1.019	-	-
			Sdev	0.0	0.0	0.0	0.0038	-	-
200 mg/kg/day									
	2561	3/1	-7	0.	0.	0.	1.011	W	L
	2570	3/1	-7	0.	0.	0.	1.022	A	L
	2567	3/1	-7	0.	0.	0.	1.036	A	LT
			N	3	3	3	3	0	0
			Mean	0.	0.	0.	1.023	-	-
			Sdev	0.0	0.0	0.0	0.0125	-	-
800 mg/kg/day									
	2564	4/1	-7	0.	0.	0.	1.020	Y	L
	2565	4/1	-7	0.	0.	3.	1.026	A	T
	2569	4/1	-7	0.	0.	2.	1.019	Y	T
	2571	4/1	-7	0.	0.	0.	1.011	Y	L
	2573	4/1	-7	0.	0.	0.	1.028	Y	LT
			N	5	5	5	5	0	0
			Mean	0.	0.	1.	1.021	-	-
			Sdev	0.0	0.0	1.4	0.0067	-	-

UBG - UROBILINOGEN
SG - SPECIFIC GRAVITY
BIL - BILIRUBIN
COL - COLOUR
RBC - HEMOGLOBIN/RED BLOOD CELLS
APP - APPEARANCE

Appendix 9
Day 28 Urine Data
Test period

Session 1 (Scheduled)
Fexinidazole
Study Number: 0505-2007

Males

Dose Level	Animal Number	Group/ Subgroup	Study Day	VOL mL	PH UNITS	WBC SCORE	NIT SCORE	PRO SCORE	GLU SCORE	KET SCORE
Vehicle										
	2516	1/1	28	212.0	7.0	2.	0.	0.	0.	0.
	2518	1/1	28	144.0	7.0	2.	1.	0.	0.	0.
	2520	1/1	28	52.0	7.0	2.	0.	0.	0.	0.
	2527	1/1	28	275.0	7.0	1.	0.	0.	0.	0.
	2533	1/1	28	60.0	7.0	1.	1.	1.	0.	0.
			N	5	5	5	5	5	5	5
			Mean	148.6	7.0	2.	0.	0.	0.	0.
			Sdev	96.44	0.00	0.5	0.5	0.4	0.0	0.0
50 mg/kg/day										
	2514	2/1	28	558.0	7.0	2.	0.	0.	0.	0.
	2521	2/1	28	18.0	7.0	2.	1.	1.	0.	0.
	2529	2/1	28	98.0	7.0	2.	0.	1.	0.	0.
			N	3	3	3	3	3	3	3
			Mean	224.7	7.0	2.	0.	1.	0.	0.
			Sdev	291.43	0.00	0.0	0.6	0.6	0.0	0.0
200 mg/kg/day										
	2515	3/1	28	221.0	6.5	2.	1.	1.	0.	0.
	2523	3/1	28	454.0	6.5	1.	0.	0.	0.	0.
	2526	3/1	28	252.0	7.0	0.	0.	0.	0.	0.
			N	3	3	3	3	3	3	3
			Mean	309.0	6.7	1.	0.	0.	0.	0.
			Sdev	126.53	0.29	1.0	0.6	0.6	0.0	0.0
800 mg/kg/day										
	2517	4/1	28	197.0	7.0	1.	0.	0.	0.	0.
	2519	4/1	28	149.0	7.0	2.	0.	0.	0.	0.
	2525	4/1	28	478.0	7.0	2.	0.	0.	0.	0.
	2528	4/1	28	432.0	7.0	2.	1.	1.	0.	0.
	2530	4/1	28	522.0	7.0	2.	0.	0.	0.	0.
			N	5	5	5	5	5	5	5
			Mean	355.6	7.0	2.	0.	0.	0.	0.
			Sdev	170.55	0.00	0.4	0.4	0.4	0.0	0.0

VOL - URINARY VOLUME
PH - PH
WBC - WHITE BLOOD CELLS
NIT - NITRITES
PRO - PROTEINS
GLU - GLUCOSE
KET - KETONE BODIES

Appendix 9
Day 28 Urine Data
Test period

Session 1 (Scheduled)
Fexinidazole

Study Number: 0505-2007

Males

Dose Level	Animal Number	Group/ Subgroup	Study Day	UBG SCORE	BIL SCORE	RBC SCORE	SG	COL	APP
Vehicle									
	2516	1/1	28	0.	0.	0.	1.018	A	L
	2518	1/1	28	0.	0.	1.	1.026	Y	T
	2520	1/1	28	0.	0.	1.	1.018	A	L
	2527	1/1	28	0.	0.	0.	1.022	Y	L
	2533	1/1	28	0.	0.	2.	1.029	A	T
			N	5	5	5	5	0	0
			Mean	0.	0.	1.	1.023	-	-
			Sdev	0.0	0.0	0.8	0.0049	-	-
50 mg/kg/day									
	2514	2/1	28	0.	0.	1.	1.019	A	T
	2521	2/1	28	0.	0.	0.	1.023	B	LT
	2529	2/1	28	0.	0.	0.	1.037	B	LT
			N	3	3	3	3	0	0
			Mean	0.	0.	0.	1.026	-	-
			Sdev	0.0	0.0	0.6	0.0095	-	-
200 mg/kg/day									
	2515	3/1	28	0.	0.	1.	1.024	A	T
	2523	3/1	28	0.	0.	1.	1.017	Y	L
	2526	3/1	28	0.	0.	0.	1.020	A	L
			N	3	3	3	3	0	0
			Mean	0.	0.	1.	1.020	-	-
			Sdev	0.0	0.0	0.6	0.0035	-	-
800 mg/kg/day									
	2517	4/1	28	0.	0.	1.	1.017	Y	LT
	2519	4/1	28	0.	0.	0.	1.023	Y	L
	2525	4/1	28	0.	0.	0.	1.018	A	L
	2528	4/1	28	0.	0.	0.	1.022	A	T
	2530	4/1	28	0.	0.	0.	1.014	Y	L
			N	5	5	5	5	0	0
			Mean	0.	0.	0.	1.019	-	-
			Sdev	0.0	0.0	0.4	0.0037	-	-

UBG - UROBILINOGEN
BIL - BILIRUBIN
RBC - HEMOGLOBIN/RED BLOOD CELLS
SG - SPECIFIC GRAVITY
COL - COLOUR
APP - APPEARANCE

Appendix 9
Day 28 Urine Data
Test period

Session 1 (Scheduled)
Fexinidazole
Study Number: 0505-2007

F e m a l e s

Dose Level	Animal Number	Group/ Subgroup	Study Day	VOL mL	PH UNITS	WBC SCORE	NIT SCORE	PRO SCORE	GLU SCORE	KET SCORE
Vehicle										
	2560	1/1	28	90.0	9.0	1.	0.	1.	0.	0.
	2568	1/1	28	98.0	7.0	1.	0.	0.	0.	0.
	2572	1/1	28	474.0	7.0	0.	0.	0.	0.	0.
	2575	1/1	28	222.0	7.0	0.	0.	0.	0.	0.
	2577	1/1	28	197.0	8.0	0.	0.	0.	0.	0.
			N	5	5	5	5	5	5	5
			Mean	216.2	7.6	0.	0.	0.	0.	0.
			Sdev	155.53	0.89	0.5	0.0	0.4	0.0	0.0
50 mg/kg/day										
	2562	2/1	28	174.0	7.0	0.	0.	0.	0.	0.
	2563	2/1	28	146.0	7.0	0.	0.	0.	0.	0.
	2576	2/1	28	175.0	7.0	2.	1.	1.	0.	0.
			N	3	3	3	3	3	3	3
			Mean	165.0	7.0	1.	0.	0.	0.	0.
			Sdev	16.46	0.00	1.2	0.6	0.6	0.0	0.0
200 mg/kg/day										
	2561	3/1	28	138.0	7.0	0.	1.	0.	0.	0.
	2570	3/1	28	94.0	8.0	2.	1.	1.	0.	0.
	2567	3/1	28	270.0	8.0	0.	0.	0.	0.	0.
			N	3	3	3	3	3	3	3
			Mean	167.3	7.7	1.	1.	0.	0.	0.
			Sdev	91.59	0.58	1.2	0.6	0.6	0.0	0.0
800 mg/kg/day										
	2564	4/1	28	290.0	7.0	0.	0.	0.	0.	0.
	2565	4/1	28	16.0	6.5	0.	1.	2.	3.	1.
	2569	4/1	28	233.0	7.0	2.	1.	1.	0.	0.
	2571	4/1	28	280.0	7.0	0.	0.	0.	0.	0.
	2573	4/1	28	135.0	8.0	0.	0.	0.	0.	0.
			N	5	5	5	5	5	5	5
			Mean	190.8	7.1	0.	0.	1.	1.	0.
			Sdev	115.38	0.55	0.9	0.5	0.9	1.3	0.4

VOL - URINARY VOLUME
PH - PH
WBC - WHITE BLOOD CELLS
NIT - NITRITES
PRO - PROTEINS
GLU - GLUCOSE
KET - KETONE BODIES

Appendix 9
Day 28 Urine Data
Test period

Session 1 (Scheduled)
Fexinidazole

Study Number: 0505-2007

F e m a l e s

Dose Level	Animal Number	Group/ Subgroup	Study Day	UBG SCORE	BIL SCORE	RBC SCORE	SG	COL	APP
Vehicle									
	2560	1/1	28	0.	0.	1.	1.035	A	T
	2568	1/1	28	0.	0.	0.	1.022	Y	L
	2572	1/1	28	0.	0.	0.	1.019	Y	LT
	2575	1/1	28	0.	0.	0.	1.035	A	LT
	2577	1/1	28	0.	0.	0.	1.028	Y	L
			N	5	5	5	5	0	0
			Mean	0.	0.	0.	1.028	-	-
			Sdev	0.0	0.0	0.4	0.0073	-	-
50 mg/kg/day									
	2562	2/1	28	0.	0.	0.	1.010	W	L
	2563	2/1	28	0.	0.	2.	1.008	W	L
	2576	2/1	28	0.	0.	2.	1.027	A	T
			N	3	3	3	3	0	0
			Mean	0.	0.	1.	1.015	-	-
			Sdev	0.0	0.0	1.2	0.0104	-	-
200 mg/kg/day									
	2561	3/1	28	0.	0.	0.	1.032	A	L
	2570	3/1	28	0.	0.	1.	1.029	A	T
	2567	3/1	28	0.	0.	0.	1.018	Y	L
			N	3	3	3	3	0	0
			Mean	0.	0.	0.	1.026	-	-
			Sdev	0.0	0.0	0.6	0.0074	-	-
800 mg/kg/day									
	2564	4/1	28	0.	0.	0.	1.015	Y	L
	2565	4/1	28	0.	0.	2.	1.035	Y	T
	2569	4/1	28	0.	0.	2.	1.019	A	T
	2571	4/1	28	0.	0.	0.	1.016	Y	L
	2573	4/1	28	0.	0.	0.	1.022	Y	L
			N	5	5	5	5	0	0
			Mean	0.	0.	1.	1.021	-	-
			Sdev	0.0	0.0	1.1	0.0081	-	-

UBG - UROBILINOGEN
SG - SPECIFIC GRAVITY
BIL - BILIRUBIN
COL - COLOUR
RBC - HEMOGLOBIN/RED BLOOD CELLS
APP - APPEARANCE

Appendix 9
Day 42 Urine Data
Test period

Session 1 (Scheduled)
Fexinidazole

Study Number: 0505-2007

Males

Dose Level	Animal Number	Group/ Subgroup	Study Day	VOL mL	PH UNITS	WBC SCORE	NIT SCORE	PRO SCORE	GLU SCORE	KET SCORE
Vehicle										
	2527	1/1	42	194.0	6.5	2.	0.	0.	0.	0.
	2533	1/1	42	638.0	7.0	2.	0.	0.	0.	0.
			N	2	2	2	2	2	2	2
			Mean	416.0	6.8	2.	0.	0.	0.	0.
			Sdev	313.96	0.35	0.0	0.0	0.0	0.0	0.0
800 mg/kg/day										
	2528	4/1	42	148.0	7.0	0.	0.	0.	0.	0.
	2530	4/1	42	112.0	8.0	2.	1.	1.	0.	1.
			N	2	2	2	2	2	2	2
			Mean	130.0	7.5	1.	1.	1.	0.	1.
			Sdev	25.46	0.71	1.4	0.7	0.7	0.0	0.7

VOL - URINARY VOLUME
PH - PH
WBC - WHITE BLOOD CELLS
NIT - NITRITES
PRO - PROTEINS
GLU - GLUCOSE
KET - KETONE BODIES

Appendix 9
Day 42 Urine Data
Test period

Session 1 (Scheduled)
Fexinidazole

Study Number: 0505-2007

Males

Dose Level	Animal Number	Group/ Subgroup	Study Day	UBG SCORE	BIL SCORE	RBC SCORE	SG	COL	APP
Vehicle									
	2527	1/1	42	0.	0.	0.	1.025	Y	L
	2533	1/1	42	0.	0.	1.	1.014	Y	T
			N	2	2	2	2	0	0
			Mean	0.	0.	1.	1.020	-	-
			Sdev	0.0	0.0	0.7	0.0078	-	-
800 mg/kg/day									
	2528	4/1	42	0.	0.	0.	1.036	A	LT
	2530	4/1	42	0.	0.	2.	1.025	A	T
			N	2	2	2	2	0	0
			Mean	0.	0.	1.	1.031	-	-
			Sdev	0.0	0.0	1.4	0.0078	-	-

UBG - UROBILINOGEN
BIL - BILIRUBIN
RBC - HEMOGLOBIN/RED BLOOD CELLS
SG - SPECIFIC GRAVITY
COL - COLOUR
APP - APPEARANCE

Appendix 9
Day 42 Urine Data
Test period

Session 1 (Scheduled)
Fexinidazole

Study Number: 0505-2007

Females

Dose Level	Animal Number	Group/ Subgroup	Study Day	VOL mL	PH UNITS	WBC SCORE	NIT SCORE	PRO SCORE	GLU SCORE	KET SCORE
Vehicle										
	2575	1/1	42	152.0	9.0	1.	0.	0.	0.	0.
	2577	1/1	42	240.0	8.0	2.	0.	0.	0.	0.
			N	2	2	2	2	2	2	2
			Mean	196.0	8.5	2.	0.	0.	0.	0.
			Sdev	62.23	0.71	0.7	0.0	0.0	0.0	0.0
800 mg/kg/day										
	2571	4/1	42	325.0	7.0	0.	0.	0.	0.	0.
	2573	4/1	42	148.0	9.0	0.	0.	0.	0.	0.
			N	2	2	2	2	2	2	2
			Mean	236.5	8.0	0.	0.	0.	0.	0.
			Sdev	125.16	1.41	0.0	0.0	0.0	0.0	0.0

VOL - URINARY VOLUME
PH - PH
WBC - WHITE BLOOD CELLS
NIT - NITRITES
PRO - PROTEINS
GLU - GLUCOSE
KET - KETONE BODIES

Appendix 9
Day 42 Urine Data
Test period

Session 1 (Scheduled)
Fexinidazole

Study Number: 0505-2007

Females

Dose Level	Animal Number	Group/ Subgroup	Study Day	UBG SCORE	BIL SCORE	RBC SCORE	SG	COL	APP
Vehicle									
	2575	1/1	42	0.	0.	2.	1.027	A	T
	2577	1/1	42	0.	0.	0.	1.024	Y	L
			N	2	2	2	2	0	0
			Mean	0.	0.	1.	1.026	-	-
			Sdev	0.0	0.0	1.4	0.0021	-	-
800 mg/kg/day									
	2571	4/1	42	0.	0.	0.	1.015	Y	L
	2573	4/1	42	0.	0.	0.	1.024	Y	L
			N	2	2	2	2	0	0
			Mean	0.	0.	0.	1.020	-	-
			Sdev	0.0	0.0	0.0	0.0064	-	-

UBG - UROBILINOGEN
BIL - BILIRUBIN
RBC - HEMOGLOBIN/RED BLOOD CELLS
SG - SPECIFIC GRAVITY
COL - COLOUR
APP - APPEARANCE

Appendix 10 Absolute Organ Weights

Appendix 10
Absolute Organ Weights (g)
Test period
Days 29 Interim Sacrifice

Fexinidazole | Study Number: 0505-2007

Dose Level	Animal Number	Group/ Subgroup		Terminal Body Wt(kg)	SPLEEN	THYMUS	KIDNEYS	LIVER	ADRENALS
				M a l e s					
Vehicle									
	2516	1/1		8.41	21.81	8.690	40.50	238.06	1.160
	2518	1/1		8.86	25.18	4.180	43.29	301.45	1.410
	2520	1/1		8.14	18.50	2.320	34.07	313.63	1.210
			N	3	3	3	3	3	3
			Mean	8.47	21.83	5.063	39.29	284.38	1.260
			Sdev	0.366	3.340	3.2756	4.728	40.574	0.1323
50 mg/kg/day									
	2514	2/1		9.45	24.80	3.110	48.91	262.00	1.180
	2521	2/1		7.58	27.20	3.870	37.44	258.76	1.320
	2529	2/1		7.59	19.81	2.020	39.24	241.26	1.200
			N	3	3	3	3	3	3
			Mean	8.21	23.94	3.000	41.86	254.01	1.233
			Sdev	1.077	3.770	0.9299	6.169	11.157	0.0757
200 mg/kg/day									
	2515	3/1		8.60	24.19	1.770	35.71	269.07	1.370
	2523	3/1		8.10	13.20	1.180	37.26	325.78	1.830
	2526	3/1		7.60	18.52	1.500	36.02	247.60	1.060
			N	3	3	3	3	3	3
			Mean	8.10	18.64	1.483	36.33	280.82	1.420
			Sdev	0.504	5.496	0.2954	0.820	40.392	0.3874
800 mg/kg/day									
	2517	4/1		6.87	10.92	1.580	35.25	330.48	1.620
	2519	4/1		7.71	24.45	2.640	37.85	291.59	1.340
	2525	4/1		9.33	22.29	3.240	42.77	338.25	1.420
			N	3	3	3	3	3	3
			Mean	7.97	19.22	2.487	38.62	320.11	1.460
			Sdev	1.249	7.269	0.8406	3.819	25.000	0.1442

Note: Data collected using grace days.

Appendix 10
Absolute Organ Weights (g)
Test period
Days 29 Interim Sacrifice

Fexinidazole Study Number: 0505-2007

Dose Level	Animal Number	Group/ Subgroup	Terminal Body Wt (kg)	HEART	BRAIN	TESTES	PROSTATE
			Males				
Vehicle							
	2516	1/1	8.41	63.18	67.65	12.63	4.44
	2518	1/1	8.86	65.27	86.97	13.06	3.42
	2520	1/1	8.14	59.25	72.22	11.09	2.46
		N	3	3	3	3	3
		Mean	8.47	62.57	75.61	12.26	3.44
		Sdev	0.366	3.057	10.097	1.036	0.990
50 mg/kg/day							
	2514	2/1	9.45	74.02	67.20	15.74	5.19
	2521	2/1	7.58	62.67	69.75	12.99	3.72
	2529	2/1	7.59	67.21	74.03	10.06	5.06
		N	3	3	3	3	3
		Mean	8.21	67.97	70.33	12.93	4.66
		Sdev	1.077	5.713	3.451	2.840	0.814
200 mg/kg/day							
	2515	3/1	8.60	69.10	72.18	11.97	3.33
	2523	3/1	8.10	63.49	78.10	5.93	2.50
	2526	3/1	7.60	57.78	68.28	14.92	4.92
		N	3	3	3	3	3
		Mean	8.10	63.46	72.85	10.94	3.58
		Sdev	0.504	5.660	4.945	4.583	1.230
800 mg/kg/day							
	2517	4/1	6.87	60.61	72.09	5.06	1.41
	2519	4/1	7.71	59.27	53.37	12.22	4.01
	2525	4/1	9.33	61.36	74.35	16.09	5.30
		N	3	3	3	3	3
		Mean	7.97	60.41	66.60	11.12	3.57
		Sdev	1.249	1.059	11.516	5.596	1.981

Note: Data collected using grace days.

Appendix 10
Absolute Organ Weights (g)
Test period
Days 29 Interim Sacrifice

Fexinidazole | Study Number: 0505-2007

Dose Level	Animal Number	Group/ Subgroup	Terminal Body Wt(kg)	SPLEEN	THYMUS	KIDNEYS	LIVER
			Females				
Vehicle							
	2560	1/1	6.35	15.28	2.260	32.03	261.69
	2568	1/1	6.90	20.57	1.670	32.79	236.65
	2572	1/1	6.60	14.81	0.770	30.79	247.06
		N	3	3	3	3	3
		Mean	6.61	16.89	1.567	31.87	248.47
		Sdev	0.276	3.199	0.7504	1.010	12.579
50 mg/kg/day							
	2562	2/1	6.57	19.48	1.960	30.90	200.63
	2563	2/1	7.61	20.35	2.660	32.86	253.51
	2576	2/1	8.38	32.86	4.990	36.69	292.62
		N	3	3	3	3	3
		Mean	7.52	24.23	3.203	33.48	248.92
		Sdev	0.907	7.486	1.5864	2.945	46.166
200 mg/kg/day							
	2561	3/1	7.10	16.59	3.720	34.40	252.23
	2570	3/1	6.65	13.26	0.870	34.85	280.27
	2567	3/1	8.00	29.83	2.150	39.69	313.86
		N	3	3	3	3	3
		Mean	7.25	19.89	2.247	36.31	282.12
		Sdev	0.688	8.765	1.4275	2.933	30.857
800 mg/kg/day							
	2564	4/1	6.10	16.09	0.940	26.90	216.61
	2565	4/1	6.11	13.41	1.510	29.96	257.76
	2569	4/1	7.65	20.66	1.520	32.53	274.32
		N	3	3	3	3	3
		Mean	6.62	16.72	1.323	29.80	249.56
		Sdev	0.889	3.666	0.3320	2.819	29.715

Note: Data collected using grace days.

Appendix 10
Absolute Organ Weights (g)
Test period
Days 29 Interim Sacrifice

Fexinidazole Study Number: 0505-2007

Dose Level	Animal Number	Group/ Subgroup	Terminal Body Wt(kg)	ADRENALS	HEART	BRAIN	OVARIES
			Females				
Vehicle							
	2560	1/1	6.35	1.350	58.70	71.76	0.917
	2568	1/1	6.90	1.380	55.94	74.11	0.852
	2572	1/1	6.60	1.130	55.64	76.37	0.592
		N	3	3	3	3	3
		Mean	6.61	1.287	56.76	74.08	0.787
		Sdev	0.276	0.1365	1.687	2.305	0.1718
50 mg/kg/day							
	2562	2/1	6.57	1.210	56.94	71.89	1.725
	2563	2/1	7.61	1.030	61.71	64.09	1.793
	2576	2/1	8.38	1.180	59.70	71.07	2.164
		N	3	3	3	3	3
		Mean	7.52	1.140	59.45	69.02	1.894
		Sdev	0.907	0.0964	2.395	4.286	0.2362
200 mg/kg/day							
	2561	3/1	7.10	1.510	54.28	77.32	1.458
	2570	3/1	6.65	1.310	54.44	74.16	1.400
	2567	3/1	8.00	1.050	72.23	74.43	2.144
		N	3	3	3	3	3
		Mean	7.25	1.290	60.32	75.30	1.667
		Sdev	0.688	0.2307	10.318	1.752	0.4140
800 mg/kg/day							
	2564	4/1	6.10	1.170	50.79	63.38	1.127
	2565	4/1	6.11	1.310	46.98	60.88	1.355
	2569	4/1	7.65	1.160	63.81	76.33	2.479
		N	3	3	3	3	3
		Mean	6.62	1.213	53.86	66.86	1.654
		Sdev	0.889	0.0839	8.825	8.293	0.7240

Note: Data collected using grace days.

Appendix 10
Absolute Organ Weights (g)
Test period
Days 43 Final Sacrifice

Fexinidazole — Study Number: 0505-2007

Dose Level	Animal Number	Group/ Subgroup	Terminal Body Wt(kg)	SPLEEN	THYMUS	KIDNEYS	LIVER	ADRENALS
			Males					
Vehicle								
	2527	1/1	7.87	20.01	3.630	33.39	254.38	1.110
	2533	1/1	7.38	17.80	1.530	31.24	239.15	1.170
		N	2	2	2	2	2	2
		Mean	7.62	18.91	2.580	32.32	246.77	1.140
		Sdev	0.351	1.563	1.4849	1.520	10.769	0.0424
800 mg/kg/day								
	2528	4/1	8.53	20.32	1.600	45.71	281.35	1.050
	2530	4/1	6.66	14.22	1.160	34.01	208.89	1.170
		N	2	2	2	2	2	2
		Mean	7.60	17.27	1.380	39.86	245.12	1.110
		Sdev	1.323	4.313	0.3111	8.273	51.237	0.0849

Note: Data collected using grace days.

Appendix 10
Absolute Organ Weights (g)
Test period
Days 43 Final Sacrifice

Fexinidazole — Study Number: 0505-2007

Dose Level	Animal Number	Group/ Subgroup	Terminal Body Wt(kg)	HEART	BRAIN	TESTES	PROSTATE
			Males				
Vehicle							
	2527	1/1	7.87	71.87	65.23	12.73	3.65
	2533	1/1	7.38	68.17	73.93	14.52	1.11
		N	2	2	2	2	2
		Mean	7.62	70.02	69.58	13.63	2.38
		Sdev	0.351	2.616	6.152	1.266	1.796
800 mg/kg/day							
	2528	4/1	8.53	71.37	75.33	10.36	3.36
	2530	4/1	6.66	62.68	63.41	10.08	0.79
		N	2	2	2	2	2
		Mean	7.60	67.03	69.37	10.22	2.08
		Sdev	1.323	6.145	8.429	0.198	1.817

Note: Data collected using grace days.

Appendix 10
Absolute Organ Weights (g)
Test period
Days 43 Final Sacrifice

Fexinidazole | Study Number: 0505-2007

Dose Level	Animal Number	Group/ Subgroup	Terminal Body Wt(kg)	SPLEEN	THYMUS	KIDNEYS	LIVER
			Females				
Vehicle							
	2575	1/1	8.11	21.99	3.350	35.42	265.17
	2577	1/1	8.55	28.82	2.740	35.07	276.35
		N	2	2	2	2	2
		Mean	8.33	25.41	3.045	35.25	270.76
		Sdev	0.310	4.830	0.4313	0.247	7.905
800 mg/kg/day							
	2571	4/1	7.32	20.46	2.570	31.94	271.37
	2573	4/1	6.83	20.67	1.500	31.65	255.81
		N	2	2	2	2	2
		Mean	7.07	20.57	2.035	31.80	263.59
		Sdev	0.348	0.148	0.7566	0.205	11.003

Note: Data collected using grace days.

CONFIDENTIAL

Appendix 10
Absolute Organ Weights (g)
Test period
Days 43 Final Sacrifice

Fexinidazole | Study Number: 0505-2007

Dose Level	Animal Number	Group/ Subgroup	Terminal Body Wt(kg)	ADRENALS	HEART	BRAIN	OVARIES
			Females				
Vehicle							
	2575	1/1	8.11	0.870	67.16	71.39	2.276
	2577	1/1	8.55	1.200	60.34	64.64	1.686
		N	2	2	2	2	2
		Mean	8.33	1.035	63.75	68.02	1.981
		Sdev	0.310	0.2333	4.822	4.773	0.4171
800 mg/kg/day							
	2571	4/1	7.32	1.170	57.15	68.88	1.625
	2573	4/1	6.83	1.130	46.78	68.29	1.542
		N	2	2	2	2	2
		Mean	7.07	1.150	51.97	68.59	1.583
		Sdev	0.348	0.0283	7.333	0.417	0.0591

Note: Data collected using grace days.

Appendix 11 Organ/Terminal Body Weight Ratios

Appendix 11
Relative Organ Weights (% Body Weight)
Test period
Days 29 Interim Sacrifice

Fexinidazole | Study Number: 0505-2007

Dose Level	Animal Number	Group/ Subgroup	Terminal Body Wt(kg)	SPLEEN	THYMUS	KIDNEYS	LIVER	ADRENALS
				Males				
Vehicle								
	2516	1/1	8.41	0.26	0.103	0.48	2.83	0.014
	2518	1/1	8.86	0.28	0.047	0.49	3.40	0.016
	2520	1/1	8.14	0.23	0.029	0.42	3.85	0.015
		N	3	3	3	3	3	3
		Mean	8.47	0.26	0.060	0.46	3.36	0.015
		Sdev	0.366	0.028	0.0390	0.039	0.511	0.0011
50 mg/kg/day								
	2514	2/1	9.45	0.26	0.033	0.52	2.77	0.012
	2521	2/1	7.58	0.36	0.051	0.49	3.41	0.017
	2529	2/1	7.59	0.26	0.027	0.52	3.18	0.016
		N	3	3	3	3	3	3
		Mean	8.21	0.29	0.037	0.51	3.12	0.015
		Sdev	1.077	0.056	0.0127	0.013	0.324	0.0025
200 mg/kg/day								
	2515	3/1	8.60	0.28	0.021	0.42	3.13	0.016
	2523	3/1	8.10	0.16	0.015	0.46	4.02	0.023
	2526	3/1	7.60	0.24	0.020	0.47	3.26	0.014
		N	3	3	3	3	3	3
		Mean	8.10	0.23	0.018	0.45	3.47	0.017
		Sdev	0.504	0.060	0.0033	0.031	0.483	0.0045
800 mg/kg/day								
	2517	4/1	6.87	0.16	0.023	0.51	4.81	0.024
	2519	4/1	7.71	0.32	0.034	0.49	3.78	0.017
	2525	4/1	9.33	0.24	0.035	0.46	3.63	0.015
		N	3	3	3	3	3	3
		Mean	7.97	0.24	0.031	0.49	4.07	0.019
		Sdev	1.249	0.079	0.0066	0.027	0.642	0.0043

Note: Data collected using grace days.

Appendix 11
Relative Organ Weights (% Body Weight)
Test period
Days 29 Interim Sacrifice

Fexinidazole | Study Number: 0505-2007

Dose Level	Animal Number	Group/ Subgroup	Terminal Body Wt(kg)	HEART	BRAIN	TESTES	PROSTATE
Males							
Vehicle							
	2516	1/1	8.41	0.75	0.80	0.15	0.05
	2518	1/1	8.86	0.74	0.98	0.15	0.04
	2520	1/1	8.14	0.73	0.89	0.14	0.03
		N	3	3	3	3	3
		Mean	8.47	0.74	0.89	0.14	0.04
		Sdev	0.366	0.012	0.088	0.007	0.011
50 mg/kg/day							
	2514	2/1	9.45	0.78	0.71	0.17	0.05
	2521	2/1	7.58	0.83	0.92	0.17	0.05
	2529	2/1	7.59	0.89	0.98	0.13	0.07
		N	3	3	3	3	3
		Mean	8.21	0.83	0.87	0.16	0.06
		Sdev	1.077	0.051	0.139	0.021	0.009
200 mg/kg/day							
	2515	3/1	8.60	0.80	0.84	0.14	0.04
	2523	3/1	8.10	0.78	0.96	0.07	0.03
	2526	3/1	7.60	0.76	0.90	0.20	0.06
		N	3	3	3	3	3
		Mean	8.10	0.78	0.90	0.14	0.04
		Sdev	0.504	0.021	0.063	0.062	0.018
800 mg/kg/day							
	2517	4/1	6.87	0.88	1.05	0.07	0.02
	2519	4/1	7.71	0.77	0.69	0.16	0.05
	2525	4/1	9.33	0.66	0.80	0.17	0.06
		N	3	3	3	3	3
		Mean	7.97	0.77	0.85	0.13	0.04
		Sdev	1.249	0.112	0.183	0.054	0.020

Note: Data collected using grace days.

Appendix 11
Relative Organ Weights (% Body Weight)
Test period
Days 29 Interim Sacrifice

Fexinidazole | Study Number: 0505-2007

Dose Level	Animal Number	Group/ Subgroup	Terminal Body Wt(kg)	SPLEEN	THYMUS	KIDNEYS	LIVER
Females							
Vehicle							
	2560	1/1	6.35	0.24	0.036	0.50	4.12
	2568	1/1	6.90	0.30	0.024	0.48	3.43
	2572	1/1	6.60	0.22	0.012	0.47	3.74
		N	3	3	3	3	3
		Mean	6.61	0.25	0.024	0.48	3.77
		Sdev	0.276	0.039	0.0120	0.020	0.347
50 mg/kg/day							
	2562	2/1	6.57	0.30	0.030	0.47	3.05
	2563	2/1	7.61	0.27	0.035	0.43	3.33
	2576	2/1	8.38	0.39	0.060	0.44	3.49
		N	3	3	3	3	3
		Mean	7.52	0.32	0.041	0.45	3.29
		Sdev	0.907	0.065	0.0159	0.021	0.222
200 mg/kg/day							
	2561	3/1	7.10	0.23	0.052	0.48	3.55
	2570	3/1	6.65	0.20	0.013	0.52	4.21
	2567	3/1	8.00	0.37	0.027	0.50	3.92
		N	3	3	3	3	3
		Mean	7.25	0.27	0.031	0.50	3.90
		Sdev	0.688	0.092	0.0199	0.020	0.331
800 mg/kg/day							
	2564	4/1	6.10	0.26	0.015	0.44	3.55
	2565	4/1	6.11	0.22	0.025	0.49	4.22
	2569	4/1	7.65	0.27	0.020	0.43	3.59
		N	3	3	3	3	3
		Mean	6.62	0.25	0.020	0.45	3.79
		Sdev	0.889	0.028	0.0046	0.034	0.374

Note: Data collected using grace days.

Appendix 11
Relative Organ Weights (% Body Weight)
Test period
Days 29 Interim Sacrifice

Fexinidazole | Study Number: 0505-2007

Dose Level	Animal Number	Group/ Subgroup	Terminal Body Wt(kg)	ADRENALS	HEART	BRAIN	OVARIES
Females							
Vehicle							
	2560	1/1	6.35	0.021	0.92	1.13	0.014
	2568	1/1	6.90	0.020	0.81	1.07	0.012
	2572	1/1	6.60	0.017	0.84	1.16	0.009
		N	3	3	3	3	3
		Mean	6.61	0.019	0.86	1.12	0.012
		Sdev	0.276	0.0021	0.059	0.042	0.0028
50 mg/kg/day							
	2562	2/1	6.57	0.018	0.87	1.09	0.026
	2563	2/1	7.61	0.014	0.81	0.84	0.024
	2576	2/1	8.38	0.014	0.71	0.85	0.026
		N	3	3	3	3	3
		Mean	7.52	0.015	0.80	0.93	0.025
		Sdev	0.907	0.0027	0.078	0.144	0.0015
200 mg/kg/day							
	2561	3/1	7.10	0.021	0.76	1.09	0.021
	2570	3/1	6.65	0.020	0.82	1.12	0.021
	2567	3/1	8.00	0.013	0.90	0.93	0.027
		N	3	3	3	3	3
		Mean	7.25	0.018	0.83	1.04	0.023
		Sdev	0.688	0.0043	0.070	0.100	0.0035
800 mg/kg/day							
	2564	4/1	6.10	0.019	0.83	1.04	0.018
	2565	4/1	6.11	0.021	0.77	1.00	0.022
	2569	4/1	7.65	0.015	0.83	1.00	0.032
		N	3	3	3	3	3
		Mean	6.62	0.019	0.81	1.01	0.024
		Sdev	0.889	0.0032	0.038	0.024	0.0072

Note: Data collected using grace days.

Appendix 11
Relative Organ Weights (% Body Weight)
Test period
Days 43 Final Sacrifice

Fexinidazole | Study Number: 0505-2007

Dose Level	Animal Number	Group/ Subgroup	Terminal Body Wt(kg)	SPLEEN	THYMUS	KIDNEYS	LIVER	ADRENALS
				Males				
Vehicle								
	2527	1/1	7.87	0.25	0.046	0.42	3.23	0.014
	2533	1/1	7.38	0.24	0.021	0.42	3.24	0.016
		N	2	2	2	2	2	2
		Mean	7.62	0.25	0.033	0.42	3.24	0.015
		Sdev	0.351	0.009	0.0179	0.000	0.008	0.0012
800 mg/kg/day								
	2528	4/1	8.53	0.24	0.019	0.54	3.30	0.012
	2530	4/1	6.66	0.21	0.017	0.51	3.14	0.018
		N	2	2	2	2	2	2
		Mean	7.60	0.23	0.018	0.52	3.22	0.015
		Sdev	1.323	0.017	0.0009	0.018	0.114	0.0037

Note: Data collected using grace days.

CONFIDENTIAL

Appendix 11
Relative Organ Weights (% Body Weight)
Test period
Days 43 Final Sacrifice

Fexinidazole | Study Number: 0505-2007

Dose Level	Animal Number	Group/ Subgroup	Terminal Body Wt(kg)	HEART	BRAIN	TESTES	PROSTATE
			M a l e s				
Vehicle							
	2527	1/1	7.87	0.91	0.83	0.16	0.05
	2533	1/1	7.38	0.92	1.00	0.20	0.02
		N	2	2	2	2	2
		Mean	7.62	0.92	0.92	0.18	0.03
		Sdev	0.351	0.008	0.123	0.025	0.022
800 mg/kg/day							
	2528	4/1	8.53	0.84	0.88	0.12	0.04
	2530	4/1	6.66	0.94	0.95	0.15	0.01
		N	2	2	2	2	2
		Mean	7.60	0.89	0.92	0.14	0.03
		Sdev	1.323	0.074	0.049	0.021	0.019

Note: Data collected using grace days.

Appendix 11
Relative Organ Weights (% Body Weight)
Test period
Days 43 Final Sacrifice

Fexinidazole | Study Number: 0505-2007

Dose Level	Animal Number	Group/ Subgroup	Terminal Body Wt(kg)	SPLEEN	THYMUS	KIDNEYS	LIVER
			**F e m a l e s**				
Vehicle							
	2575	1/1	8.11	0.27	0.041	0.44	3.27
	2577	1/1	8.55	0.34	0.032	0.41	3.23
		N	2	2	2	2	2
		Mean	8.33	0.30	0.037	0.42	3.25
		Sdev	0.310	0.047	0.0065	0.019	0.026
800 mg/kg/day							
	2571	4/1	7.32	0.28	0.035	0.44	3.71
	2573	4/1	6.83	0.30	0.022	0.46	3.75
		N	2	2	2	2	2
		Mean	7.07	0.29	0.029	0.45	3.73
		Sdev	0.348	0.016	0.0093	0.019	0.028

Note: Data collected using grace days.

Appendix 11
Relative Organ Weights (% Body Weight)
Test period
Days 43 Final Sacrifice

Fexinidazole | Study Number: 0505-2007

Dose Level	Animal Number	Group/ Subgroup	Terminal Body Wt(kg)	ADRENALS	HEART	BRAIN	OVARIES
			Females				
Vehicle							
	2575	1/1	8.11	0.011	0.83	0.88	0.028
	2577	1/1	8.55	0.014	0.71	0.76	0.020
		N	2	2	2	2	2
		Mean	8.33	0.012	0.77	0.82	0.024
		Sdev	0.310	0.0023	0.087	0.088	0.0059
800 mg/kg/day							
	2571	4/1	7.32	0.016	0.78	0.94	0.022
	2573	4/1	6.83	0.017	0.69	1.00	0.023
		N	2	2	2	2	2
		Mean	7.07	0.016	0.73	0.97	0.022
		Sdev	0.348	0.0004	0.068	0.042	0.0003

Note: Data collected using grace days.

Appendix 12 Gross And Microscopic Pathology

Appendix 12
Individual Animal Microscopic vs. Gross Observations

Fexinidazole Study Number: 0505-2007

ANIMAL: 2516 SEX: Male GROUP: 1 DOSE LEVEL: Vehicle
DAY OF DEATH: 29 Test period STATUS: Scheduled phase sacrifice # 1 TERMINAL BODY WEIGHT (kg) : 8.41

Tissue	Gross observations/comments	Correlated Microscopic Observations
COLON	No gross observations on tissue	CRYPT DILATATION WITH/WITHOUT LUMENAL NECROTIC DEBRIS, Minimal, Focal.
DUODENUM	No gross observations on tissue	CRYPT DILATATION WITH/WITHOUT LUMENAL NECROTIC DEBRIS, Slight, Multifocal.
GALL BLADDER	No gross observations on tissue	LYMPHOCYTIC INFILTRATION, Minimal, Multifocal.
GENER. CONDITION . . .	GOOD	No micropathology observations on tissue.
HEART	No gross observations on tissue	MESOTHELIAL HYPERPLASIA, ATRIAL, Minimal, Focal, Unilateral left..
KIDNEYS	No gross observations on tissue	PAPILLARY MINERALIZATION, Minimal, Multifocal.
LIVER	No gross observations on tissue	CHRONIC INFLAMMATION, Minimal, Multifocal.
		EXTRAMEDULLARY HEMATOPOIESIS, Minimal, Multifocal.
MESENTERIC L.N.	No gross observations on tissue	SINUS ERYTHROCYTES/ERYTHROPHAGOCYTOSIS, Minimal.
MAMMARY GLAND	No gross observations on tissue	NO MAMMARY TISSUE IN THE SECTION, Present.

Appendix 12
Individual Animal Microscopic vs. Gross Observations

Fexinidazole Study Number: 0505-2007

ANIMAL: 2516 SEX: Male GROUP: 1 DOSE LEVEL: Vehicle
DAY OF DEATH: 29 Test period STATUS: Scheduled phase sacrifice # 1 TERMINAL BODY WEIGHT (kg) : 8.41

Tissue	Gross observations/comments	Correlated Microscopic Observations
PITUITARY	No gross observations on tissue	CYSTS, PARS DISTALIS, Slight, Focal.
PARATHYROIDS	No gross observations on tissue	ONLY ONE PARATHYROID AVAILABLE FOR EXAMINATION, Present.
PAROTIDS	No gross observations on tissue	ACINAR ATROPHY, Minimal, Focal, Unilateral..
TESTES	No gross observations on tissue	SEGMENTAL HYPOPLASIA, Slight, Multifocal, Unilateral..

The following tissues have no gross observations and were not examined microscopically:
No tissue to list.

The following tissues have no gross observations and were marked as unremarkable microscopically:

ADRENALS	AORTA-THORACIC	BONE MARROW	BRAIN	CECUM
DIAPHRAGM	EPIDIDYMIDES	ESOPHAGUS	EYES	FEMUR HEAD
ILEUM	JEJUNUM	LACRIMAL GLANDS	MANDIBULAR L.N.	LUNG
SKELETAL MUSCLE	SCIATIC NERVE	OPTIC NERVES	PANCREAS	PROSTATE
SPINAL CORD-CERV	MANDIBULAR S.G.	SKIN	SPLEEN	STOMACH
STERNUM	THYROIDS	THYMUS	TONGUE	TRACHEA
URINARY BLADDER				

Appendix 12
Individual Animal Microscopic vs. Gross Observations

Fexinidazole — Study Number: 0505-2007

ANIMAL: 2518 — SEX: Male — GROUP: 1 — DOSE LEVEL: Vehicle
DAY OF DEATH: 29 Test period — STATUS: Scheduled phase sacrifice # 1 — TERMINAL BODY WEIGHT (kg) : 8.86

Tissue	Gross observations/comments	Correlated Microscopic Observations
COLON	No gross observations on tissue	CRYPT DILATATION WITH/WITHOUT LUMENAL NECROTIC DEBRIS, Minimal, Focal.
GENER. CONDITION . . .	GOOD	No micropathology observations on tissue.
HEART	No gross observations on tissue	MESOTHELIAL HYPERPLASIA, ATRIAL, Slight, Focal, Unilateral left..
KIDNEYS	No gross observations on tissue	PAPILLARY MINERALIZATION, Minimal, Multifocal.
LACRIMAL GLANDS	No gross observations on tissue	ONLY ONE GLAND AVAILABLE FOR EXAMINATION, Present.
LUNG	No gross observations on tissue	CHRONIC INFLAMMATION, Slight, Multifocal.<br>ALVEOLAR MACROPHAGE INFILTRATION, Minimal, Multifocal.
MAMMARY GLAND	No gross observations on tissue	NO MAMMARY TISSUE IN THE SECTION, Present.
PARATHYROIDS	No gross observations on tissue	ONLY ONE PARATHYROID AVAILABLE FOR EXAMINATION, Present.
THYMUS	No gross observations on tissue	CYSTS, Minimal, Focal.<br>INVOLUTION, Slight.

ANIMAL: 2518 SEX: Male GROUP: 1 DOSE LEVEL: Vehicle
DAY OF DEATH: 29 Test period STATUS: Scheduled phase sacrifice # 1 TERMINAL BODY WEIGHT (kg) : 8.86

Tissue	Gross observations/comments	Correlated Microscopic Observations
URINARY BLADDER	No gross observations on tissue	MINERALIZATION IN MUSCULARIS/SUBSEROSA, Slight, Focal. / associated with small vessels and smooth muscle tissue

The following tissues have no gross observations and were not examined microscopically:
No tissue to list.

The following tissues have no gross observations and were marked as unremarkable microscopically:

ADRENALS	AORTA-THORACIC	BONE MARROW	BRAIN	CECUM
DIAPHRAGM	DUODENUM	EPIDIDYMIDES	ESOPHAGUS	EYES
FEMUR HEAD	GALL BLADDER	ILEUM	JEJUNUM	LIVER
MANDIBULAR L.N.	MESENTERIC L.N.	SKELETAL MUSCLE	SCIATIC NERVE	OPTIC NERVES
PANCREAS	PITUITARY	PROSTATE	SPINAL CORD-CERV	MANDIBULAR S.G.
PAROTIDS	SKIN	SPLEEN	STOMACH	STERNUM
TESTES	THYROIDS	TONGUE	TRACHEA	

Appendix 12
Individual Animal Microscopic vs. Gross Observations

Fexinidazole — Study Number: 0505-2007

ANIMAL: 2520 | SEX: Male | GROUP: 1 | DOSE LEVEL: Vehicle
DAY OF DEATH: 30 Test period | STATUS: Scheduled phase sacrifice # 1 | TERMINAL BODY WEIGHT (kg) : 8.14

Tissue	Gross observations/comments	Correlated Microscopic Observations
GALL BLADDER	No gross observations on tissue	LYMPHOCYTIC INFILTRATION, Minimal, Multifocal.
GENER. CONDITION	GOOD	No micropathology observations on tissue.
KIDNEYS	No gross observations on tissue	PAPILLARY MINERALIZATION, Minimal, Multifocal.
LIVER	No gross observations on tissue	CHRONIC INFLAMMATION, Minimal, Multifocal.
MESENTERIC L.N.	No gross observations on tissue	SINUS ERYTHROCYTES/ERYTHROPHAGOCYTOSIS, Slight.
LUNG	No gross observations on tissue	ALVEOLAR MACROPHAGE INFILTRATION, Minimal, Multifocal.
MAMMARY GLAND	No gross observations on tissue	NO MAMMARY TISSUE IN THE SECTION, Present.
PITUITARY	No gross observations on tissue	CYSTS, PARS DISTALIS, Slight, Multifocal.
SKIN	No gross observations on tissue	CHRONIC INFLAMMATION, Minimal, Multifocal. / in the dermis perifollicular
THYMUS	No gross observations on tissue	INVOLUTION, Slight.

Appendix 12
Individual Animal Microscopic vs. Gross Observations

Fexinidazole Study Number: 0505-2007

ANIMAL: 2520 SEX: Male GROUP: 1 DOSE LEVEL: Vehicle
DAY OF DEATH: 30 Test period STATUS: Scheduled phase sacrifice # 1 TERMINAL BODY WEIGHT (kg) : 8.14

Tissue	Gross observations/comments	Correlated Microscopic Observations

The following tissues have no gross observations and were not examined microscopically:
No tissue to list.

The following tissues have no gross observations and were marked as unremarkable microscopically:

ADRENALS	AORTA-THORACIC	BONE MARROW	BRAIN	CECUM
COLON	DIAPHRAGM	DUODENUM	EPIDIDYMIDES	ESOPHAGUS
EYES	FEMUR HEAD	HEART	ILEUM	JEJUNUM
LACRIMAL GLANDS	MANDIBULAR L.N.	SKELETAL MUSCLE	SCIATIC NERVE	OPTIC NERVES
PANCREAS	PROSTATE	PARATHYROIDS	SPINAL CORD-CERV	MANDIBULAR S.G.
PAROTIDS	SPLEEN	STOMACH	STERNUM	TESTES
THYROIDS	TONGUE	TRACHEA	URINARY BLADDER	

ANIMAL: 2527   SEX: Male   GROUP: 1   DOSE LEVEL: Vehicle
DAY OF DEATH: 43 Test period   STATUS: Final phase sacrifice   TERMINAL BODY WEIGHT (kg) : 7.87

Tissue	Gross observations/comments	Correlated Microscopic Observations
DUODENUM	No gross observations on tissue	CRYPT DILATATION WITH/WITHOUT LUMENAL NECROTIC DEBRIS, Minimal, Focal.
GALL BLADDER	No gross observations on tissue	LYMPHOCYTIC INFILTRATION, Minimal, Multifocal.
GENER. CONDITION . . .	GOOD	No micropathology observations on tissue.
HEART	No gross observations on tissue	MESOTHELIAL HYPERPLASIA, ATRIAL, Minimal, Multifocal, Unilateral left..
KIDNEYS	No gross observations on tissue	CHRONIC INFLAMMATION, Slight, Multifocal.
		PAPILLARY MINERALIZATION, Minimal, Multifocal.
		CORTICAL TUBULAR DILATION, Moderate, Multifocal.
		CORTICAL TUBULAR REGENERATIVE BASOPHILIA, Slight, Multifocal.
LIVER	No gross observations on tissue	EXTRAMEDULLARY HEMATOPOIESIS, Minimal, Multifocal.
MESENTERIC L.N.	No gross observations on tissue	SINUS ERYTHROCYTES/ERYTHROPHAGOCYTOSIS, Slight.

Appendix 12
Individual Animal Microscopic vs. Gross Observations

Fexinidazole | Study Number: 0505-2007

ANIMAL: 2527 | SEX: Male | GROUP: 1 | DOSE LEVEL: Vehicle
DAY OF DEATH: 43 Test period | STATUS: Final phase sacrifice | TERMINAL BODY WEIGHT (kg) : 7.87

Tissue	Gross observations/comments	Correlated Microscopic Observations
LUNG	No gross observations on tissue	ALVEOLAR MACROPHAGE INFILTRATION, Minimal, Multifocal.
MAMMARY GLAND	No gross observations on tissue	NO MAMMARY TISSUE IN THE SECTION, Present.
PARATHYROIDS	No gross observations on tissue	ONLY ONE PARATHYROID AVAILABLE FOR EXAMINATION, Present.
MANDIBULAR S.G.	No gross observations on tissue	LYMPHOCYTIC INFILTRATION, Minimal, Focal, Unilateral..
PAROTIDS	No gross observations on tissue	ACINAR ATROPHY, Minimal, Focal.
THYROIDS	No gross observations on tissue	CYSTIC FOLLICLES, Slight, Focal, Unilateral..
THYMUS	No gross observations on tissue	CYSTS, Minimal, Focal.
		INVOLUTION, Slight.

The following tissues have no gross observations and were not examined microscopically:
No tissue to list.

The following tissues have no gross observations and were marked as unremarkable microscopically:
ADRENALS AORTA-THORACIC BONE MARROW BRAIN CECUM

Appendix 12
Individual Animal Microscopic vs. Gross Observations

Fexinidazole Study Number: 0505-2007

ANIMAL: 2527 SEX: Male GROUP: 1 DOSE LEVEL: Vehicle
DAY OF DEATH: 43 Test period STATUS: Final phase sacrifice TERMINAL BODY WEIGHT (kg) : 7.87

Tissue	Gross observations/comments	Correlated Microscopic Observations

The following tissues have no gross observations and were marked as unremarkable microscopically:

COLON	DIAPHRAGM	EPIDIDYMIDES	ESOPHAGUS	EYES
FEMUR HEAD	ILEUM	JEJUNUM	LACRIMAL GLANDS	MANDIBULAR L.N.
SKELETAL MUSCLE	SCIATIC NERVE	OPTIC NERVES	PANCREAS	PITUITARY
PROSTATE	SPINAL CORD-CERV	SKIN	SPLEEN	STOMACH
STERNUM	TESTES	TONGUE	TRACHEA	URINARY BLADDER

Appendix 12
Individual Animal Microscopic vs. Gross Observations

Fexinidazole | Study Number: 0505-2007

ANIMAL: 2533 | SEX: Male | GROUP: 1 | DOSE LEVEL: Vehicle
DAY OF DEATH: 43 Test period | STATUS: Final phase sacrifice | TERMINAL BODY WEIGHT (kg) : 7.38

Tissue	Gross observations/comments	Correlated Microscopic Observations
GALL BLADDER	No gross observations on tissue	LYMPHOCYTIC INFILTRATION, Minimal, Multifocal.
GENER. CONDITION . . .	GOOD	No micropathology observations on tissue.
KIDNEYS	No gross observations on tissue	PAPILLARY MINERALIZATION, Minimal, Multifocal.<br>CORTICAL TUBULAR REGENERATIVE BASOPHILIA, Minimal, Focal, Unilateral left..
LIVER	No gross observations on tissue	EXTRAMEDULLARY HEMATOPOIESIS, Minimal, Multifocal.
MANDIBULAR L.N.	No gross observations on tissue	SINUS ERYTHROCYTES/ERYTHROPHAGOCYTOSIS, Minimal.
MESENTERIC L.N.	No gross observations on tissue	SINUS ERYTHROCYTES/ERYTHROPHAGOCYTOSIS, Minimal.
LUNG	No gross observations on tissue	ALVEOLAR HEMORRHAGE, Slight, Multifocal.<br>CAPILLARY ANGIOMATOSIS, Slight, Multifocal.
MAMMARY GLAND	No gross observations on tissue	NO MAMMARY TISSUE IN THE SECTION, Present.
PROSTATE	SMALL	Examined 1 correlation found:<br>IMMATURE, Present.

Appendix 12
Individual Animal Microscopic vs. Gross Observations

Fexinidazole | Study Number: 0505-2007

ANIMAL: 2533 | SEX: Male | GROUP: 1 | DOSE LEVEL: Vehicle
DAY OF DEATH: 43 Test period | STATUS: Final phase sacrifice | TERMINAL BODY WEIGHT (kg) : 7.38

Tissue	Gross observations/comments	Correlated Microscopic Observations
PAROTIDS	. . . . No gross observations on tissue	ACINAR ATROPHY, Minimal, Focal, Unilateral..
THYMUS	. . . . No gross observations on tissue	INVOLUTION, Moderate.

The following tissues have no gross observations and were not examined microscopically:
No tissue to list.

The following tissues have no gross observations and were marked as unremarkable microscopically:

ADRENALS	AORTA-THORACIC	BONE MARROW	BRAIN	CECUM
COLON	DIAPHRAGM	DUODENUM	EPIDIDYMIDES	ESOPHAGUS
EYES	FEMUR HEAD	HEART	ILEUM	JEJUNUM
LACRIMAL GLANDS	SKELETAL MUSCLE	SCIATIC NERVE	OPTIC NERVES	PANCREAS
PITUITARY	PARATHYROIDS	SPINAL CORD-CERV	MANDIBULAR S.G.	SKIN
SPLEEN	STOMACH	STERNUM	TESTES	THYROIDS
TONGUE	TRACHEA	URINARY BLADDER		

Appendix 12
Individual Animal Microscopic vs. Gross Observations

Fexinidazole | Study Number: 0505-2007

ANIMAL: 2514 | SEX: Male | GROUP: 2 | DOSE LEVEL: 50 mg/kg/day
DAY OF DEATH: 29 Test period | STATUS: Scheduled phase sacrifice # 1 | TERMINAL BODY WEIGHT (kg) : 9.45

Tissue	Gross observations/comments	Correlated Microscopic Observations
GENER. CONDITION . . .	GOOD	No micropathology observations on tissue.

The following tissues have no gross observations and were not examined microscopically:

ADRENALS	AORTA-THORACIC	BRAIN	CECUM	COLON
DIAPHRAGM	DUODENUM	EPIDIDYMIDES	ESOPHAGUS	EYES
FEMUR HEAD	GALL BLADDER	HEART	ILEUM	JEJUNUM
KIDNEYS	LACRIMAL GLANDS	LIVER	MANDIBULAR L.N.	MESENTERIC L.N.
LUNG	MAMMARY GLAND	SKELETAL MUSCLE	SCIATIC NERVE	OPTIC NERVES
PANCREAS	PITUITARY	PROSTATE	PARATHYROIDS	SPINAL CORD-CERV
MANDIBULAR S.G.	PAROTIDS	SKIN	SPLEEN	STOMACH
STERNUM	TESTES	THYROIDS	THYMUS	TONGUE
TRACHEA	URINARY BLADDER			

The following tissues have no gross observations and were marked as unremarkable microscopically:

BONE MARROW

Appendix 12
Individual Animal Microscopic vs. Gross Observations

Fexinidazole Study Number: 0505-2007

ANIMAL: 2521 SEX: Male GROUP: 2 DOSE LEVEL: 50 mg/kg/day
DAY OF DEATH: 29 Test period STATUS: Scheduled phase sacrifice # 1 TERMINAL BODY WEIGHT (kg) : 7.58

Tissue	Gross observations/comments	Correlated Microscopic Observations
GENER. CONDITION . . .	GOOD	No micropathology observations on tissue.

The following tissues have no gross observations and were not examined microscopically:

ADRENALS	AORTA-THORACIC	BRAIN	CECUM	COLON
DIAPHRAGM	DUODENUM	EPIDIDYMIDES	ESOPHAGUS	EYES
FEMUR HEAD	GALL BLADDER	HEART	ILEUM	JEJUNUM
KIDNEYS	LACRIMAL GLANDS	LIVER	MANDIBULAR L.N.	MESENTERIC L.N.
LUNG	MAMMARY GLAND	SKELETAL MUSCLE	SCIATIC NERVE	OPTIC NERVES
PANCREAS	PITUITARY	PROSTATE	PARATHYROIDS	SPINAL CORD-CERV
MANDIBULAR S.G.	PAROTIDS	SKIN	SPLEEN	STOMACH
STERNUM	TESTES	THYROIDS	THYMUS	TONGUE
TRACHEA	URINARY BLADDER			

The following tissues have no gross observations and were marked as unremarkable microscopically:

BONE MARROW

Appendix 12
Individual Animal Microscopic vs. Gross Observations

Fexinidazole — Study Number: 0505-2007

ANIMAL: 2529 — SEX: Male — GROUP: 2 — DOSE LEVEL: 50 mg/kg/day
DAY OF DEATH: 30 Test period — STATUS: Scheduled phase sacrifice # 1 — TERMINAL BODY WEIGHT (kg) : 7.59

Tissue	Gross observations/comments	Correlated Microscopic Observations
GENER. CONDITION . . .	GOOD	No micropathology observations on tissue.

The following tissues have no gross observations and were not examined microscopically:

ADRENALS	AORTA-THORACIC	BRAIN	CECUM	COLON
DIAPHRAGM	DUODENUM	EPIDIDYMIDES	ESOPHAGUS	EYES
FEMUR HEAD	GALL BLADDER	HEART	ILEUM	JEJUNUM
KIDNEYS	LACRIMAL GLANDS	LIVER	MANDIBULAR L.N.	MESENTERIC L.N.
LUNG	MAMMARY GLAND	SKELETAL MUSCLE	SCIATIC NERVE	OPTIC NERVES
PANCREAS	PITUITARY	PROSTATE	PARATHYROIDS	SPINAL CORD-CERV
MANDIBULAR S.G.	PAROTIDS	SKIN	SPLEEN	STOMACH
STERNUM	TESTES	THYROIDS	THYMUS	TONGUE
TRACHEA	URINARY BLADDER			

The following tissues have no gross observations and were marked as unremarkable microscopically:

BONE MARROW

Appendix 12
Individual Animal Microscopic vs. Gross Observations

Fexinidazole Study Number: 0505-2007

ANIMAL: 2515 SEX: Male GROUP: 3 DOSE LEVEL: 200 mg/kg/day
DAY OF DEATH: 29 Test period STATUS: Scheduled phase sacrifice # 1 TERMINAL BODY WEIGHT (kg) : 8.60

Tissue	Gross observations/comments	Correlated Microscopic Observations
GENER. CONDITION . . .	GOOD	No micropathology observations on tissue.

The following tissues have no gross observations and were not examined microscopically:

ADRENALS	AORTA-THORACIC	BRAIN	CECUM	COLON
DIAPHRAGM	DUODENUM	EPIDIDYMIDES	ESOPHAGUS	EYES
FEMUR HEAD	GALL BLADDER	HEART	ILEUM	JEJUNUM
KIDNEYS	LACRIMAL GLANDS	LIVER	MANDIBULAR L.N.	MESENTERIC L.N.
LUNG	MAMMARY GLAND	SKELETAL MUSCLE	SCIATIC NERVE	OPTIC NERVES
PANCREAS	PITUITARY	PROSTATE	PARATHYROIDS	SPINAL CORD-CERV
MANDIBULAR S.G.	PAROTIDS	SKIN	SPLEEN	STOMACH
STERNUM	TESTES	THYROIDS	THYMUS	TONGUE
TRACHEA	URINARY BLADDER			

The following tissues have no gross observations and were marked as unremarkable microscopically:

BONE MARROW

Appendix 12
Individual Animal Microscopic vs. Gross Observations

Fexinidazole — Study Number: 0505-2007

ANIMAL: 2523 — SEX: Male — GROUP: 3 — DOSE LEVEL: 200 mg/kg/day
DAY OF DEATH: 29 Test period — STATUS: Scheduled phase sacrifice # 1 — TERMINAL BODY WEIGHT (kg) : 8.10

Tissue	Gross observations/comments	Correlated Microscopic Observations
BONE MARROW	No gross observations on tissue	REDUCED CELLULARITY, Minimal.
GENER. CONDITION . . .	FAIRLY GOOD	No micropathology observations on tissue.
LYMPH NODES	ENLARGED/ all lymph nodes.	No micropathology observations on tissue.
PERITONEAL CAV.	CLEAR LIQUID CONTENT	No micropathology observations on tissue.
TESTES	SMALL, BILATERALLY	Examined 1 correlation found: IMMATURE, Present.
	FLACCID	Examined 1 correlation found: IMMATURE, Present.

The following tissues have no gross observations and were not examined microscopically:

ADRENALS	AORTA-THORACIC	BRAIN	CECUM	COLON
DIAPHRAGM	DUODENUM	EPIDIDYMIDES	ESOPHAGUS	EYES
FEMUR HEAD	GALL BLADDER	HEART	ILEUM	JEJUNUM
KIDNEYS	LACRIMAL GLANDS	LIVER	LUNG	MAMMARY GLAND
SKELETAL MUSCLE	SCIATIC NERVE	OPTIC NERVES	PANCREAS	PITUITARY
PROSTATE	PARATHYROIDS	SPINAL CORD-CERV	MANDIBULAR S.G.	PAROTIDS
SKIN	SPLEEN	STOMACH	STERNUM	THYROIDS
THYMUS	TONGUE	TRACHEA	URINARY BLADDER	

The following tissues have no gross observations and were marked as unremarkable microscopically:

MANDIBULAR L.N. MESENTERIC L.N.

Appendix 12
Individual Animal Microscopic vs. Gross Observations

Fexinidazole | Study Number: 0505-2007

ANIMAL: 2526 | SEX: Male | GROUP: 3 | DOSE LEVEL: 200 mg/kg/day
DAY OF DEATH: 30 Test period | STATUS: Scheduled phase sacrifice # 1 | TERMINAL BODY WEIGHT (kg) : 7.60

Tissue	Gross observations/comments	Correlated Microscopic Observations
GENER. CONDITION . . .	GOOD	No micropathology observations on tissue.

The following tissues have no gross observations and were not examined microscopically:

ADRENALS	AORTA-THORACIC	BRAIN	CECUM	COLON
DIAPHRAGM	DUODENUM	EPIDIDYMIDES	ESOPHAGUS	EYES
FEMUR HEAD	GALL BLADDER	HEART	ILEUM	JEJUNUM
KIDNEYS	LACRIMAL GLANDS	LIVER	MANDIBULAR L.N.	MESENTERIC L.N.
LUNG	MAMMARY GLAND	SKELETAL MUSCLE	SCIATIC NERVE	OPTIC NERVES
PANCREAS	PITUITARY	PROSTATE	PARATHYROIDS	SPINAL CORD-CERV
MANDIBULAR S.G.	PAROTIDS	SKIN	SPLEEN	STOMACH
STERNUM	TESTES	THYROIDS	THYMUS	TONGUE
TRACHEA	URINARY BLADDER			

The following tissues have no gross observations and were marked as unremarkable microscopically:
BONE MARROW

Appendix 12
Individual Animal Microscopic vs. Gross Observations

Fexinidazole　　　　Study Number: 0505-2007

ANIMAL: 2517　SEX: Male　GROUP: 4　DOSE LEVEL: 800 mg/kg/day
DAY OF DEATH: 29 Test period　STATUS: Scheduled phase sacrifice # 1　TERMINAL BODY WEIGHT (kg) : 6.87

Tissue	Gross observations/comments	Correlated Microscopic Observations
BONE MARROW	No gross observations on tissue	REDUCED CELLULARITY, Moderate.<br>ATROPHY OF ADIPOSE TISSUE IN STERNAL AND FEMORAL MARROW, Moderate, Diffuse.
EPIDIDYMIDES	No gross observations on tissue	IMMATURE, Present.<br>LUMENAL GERM CELLS/DEBRIS, Minimal.
ESOPHAGUS	No gross observations on tissue	ACUTE INFLAMMATION, Minimal, Multifocal. / associated with submucosal glands
GALL BLADDER	No gross observations on tissue	LYMPHOCYTIC INFILTRATION, Minimal, Multifocal.
GENER. CONDITION . . .	FAIRLY GOOD	No micropathology observations on tissue.
KIDNEYS	No gross observations on tissue	PAPILLARY MINERALIZATION, Minimal, Multifocal, Unilateral left..<br>ATROPHY OF ADJACENT ADIPOSE TISSUE, Slight, Diffuse.
LIVER	No gross observations on tissue	GLYCOGEN DEPLETION, Slight.
MAMMARY GLAND	No gross observations on tissue	NO MAMMARY TISSUE IN THE SECTION, Present.

Appendix 12
Individual Animal Microscopic vs. Gross Observations

Fexinidazole   Study Number: 0505-2007

ANIMAL: 2517   SEX: Male   GROUP: 4   DOSE LEVEL: 800 mg/kg/day
DAY OF DEATH: 29 Test period   STATUS: Scheduled phase sacrifice # 1   TERMINAL BODY WEIGHT (kg) : 6.87

Tissue	Gross observations/comments	Correlated Microscopic Observations
PROSTATE	SMALL	Examined 1 correlation found: IMMATURE, Present.
PAROTIDS	No gross observations on tissue	ACINAR ATROPHY, Minimal, Focal, Unilateral..
TESTES	SMALL, BILATERALLY	Examined 1 correlation found: IMMATURE, Present.
	FLACCID	Examined 1 correlation found: IMMATURE, Present.
THYMUS	No gross observations on tissue	CYSTS, Slight, Multifocal.
		INVOLUTION, Severe.

The following tissues have no gross observations and were not examined microscopically:
No tissue to list.

The following tissues have no gross observations and were marked as unremarkable microscopically:

ADRENALS	AORTA-THORACIC	BRAIN	CECUM	COLON
DIAPHRAGM	DUODENUM	EYES	FEMUR HEAD	HEART
ILEUM	JEJUNUM	LACRIMAL GLANDS	MANDIBULAR L.N.	MESENTERIC L.N.
LUNG	SKELETAL MUSCLE	SCIATIC NERVE	OPTIC NERVES	PANCREAS
PITUITARY	PARATHYROIDS	SPINAL CORD-CERV	MANDIBULAR S.G.	SKIN
SPLEEN	STOMACH	STERNUM	THYROIDS	TONGUE
TRACHEA	URINARY BLADDER			

Fexinidazole Study Number: 0505-2007

ANIMAL: 2519 SEX: Male GROUP: 4 DOSE LEVEL: 800 mg/kg/day
DAY OF DEATH: 29 Test period STATUS: Scheduled phase sacrifice # 1 TERMINAL BODY WEIGHT (kg) : 7.71

Tissue	Gross observations/comments	Correlated Microscopic Observations
AORTA-THORACIC	No gross observations on tissue	ECTOPIC THYROID, Present.
GALL BLADDER	No gross observations on tissue	LYMPHOCYTIC INFILTRATION, Minimal, Multifocal.
GENER. CONDITION . . .	GOOD	No micropathology observations on tissue.
KIDNEYS	No gross observations on tissue	PAPILLARY MINERALIZATION, Minimal, Multifocal.
LACRIMAL GLANDS	No gross observations on tissue	ONLY ONE GLAND AVAILABLE FOR EXAMINATION, Present.
LIVER	No gross observations on tissue	CHRONIC INFLAMMATION, Minimal, Multifocal.
LUNG	No gross observations on tissue	ALVEOLAR MACROPHAGE INFILTRATION, Minimal, Multifocal.
MAMMARY GLAND	No gross observations on tissue	NO MAMMARY TISSUE IN THE SECTION, Present.
PANCREAS	No gross observations on tissue	ACINAR DEGRANULATION, Minimal, Multifocal.
PARATHYROIDS	No gross observations on tissue	CYSTS, Minimal, Multifocal, Unilateral..
TESTES	No gross observations on tissue	SEGMENTAL HYPOPLASIA, Minimal, Focal, Unilateral..

Appendix 12
Individual Animal Microscopic vs. Gross Observations

Fexinidazole Study Number: 0505-2007

ANIMAL: 2519 SEX: Male GROUP: 4 DOSE LEVEL: 800 mg/kg/day
DAY OF DEATH: 29 Test period STATUS: Scheduled phase sacrifice # 1 TERMINAL BODY WEIGHT (kg) : 7.71

Tissue	Gross observations/comments	Correlated Microscopic Observations
THYMUS	No gross observations on tissue	Tissue is missing.<br><br>only adipose tissue detectable in the samples evaluated
TONGUE	No gross observations on tissue	ACUTE INFLAMMATION, Minimal, Focal.

The following tissues have no gross observations and were not examined microscopically:
No tissue to list.

The following tissues have no gross observations and were marked as unremarkable microscopically:

ADRENALS	BONE MARROW	BRAIN	CECUM	COLON
DIAPHRAGM	DUODENUM	EPIDIDYMIDES	ESOPHAGUS	EYES
FEMUR HEAD	HEART	ILEUM	JEJUNUM	MANDIBULAR L.N.
MESENTERIC L.N.	SKELETAL MUSCLE	SCIATIC NERVE	OPTIC NERVES	PITUITARY
PROSTATE	SPINAL CORD-CERV	MANDIBULAR S.G.	PAROTIDS	SKIN
SPLEEN	STOMACH	STERNUM	THYROIDS	TRACHEA
URINARY BLADDER				

Appendix 12
Individual Animal Microscopic vs. Gross Observations

Fexinidazole Study Number: 0505-2007

ANIMAL: 2525 SEX: Male GROUP: 4 DOSE LEVEL: 800 mg/kg/day
DAY OF DEATH: 30 Test period STATUS: Scheduled phase sacrifice # 1 TERMINAL BODY WEIGHT (kg) : 9.33

Tissue	Gross observations/comments	Correlated Microscopic Observations
GALL BLADDER	No gross observations on tissue	LYMPHOCYTIC INFILTRATION, Slight, Multifocal.
GENER. CONDITION . . .	GOOD	No micropathology observations on tissue.
HEART	No gross observations on tissue	ACUTE INFLAMMATION, Minimal, Focal. / in the septum
MESENTERIC L.N.	No gross observations on tissue	SINUS ERYTHROCYTES/ERYTHROPHAGOCYTOSIS, Minimal.
LUNG	No gross observations on tissue	ALVEOLAR MACROPHAGE INFILTRATION, Minimal, Multifocal.
MAMMARY GLAND	No gross observations on tissue	NO MAMMARY TISSUE IN THE SECTION, Present.
PARATHYROIDS	No gross observations on tissue	ONLY ONE PARATHYROID AVAILABLE FOR EXAMINATION, Present.
PAROTIDS	No gross observations on tissue	ACINAR ATROPHY, Minimal, Focal, Unilateral..
THYMUS	No gross observations on tissue	INVOLUTION, Slight.

The following tissues have no gross observations and were not examined microscopically:
No tissue to list.

Appendix 12
Individual Animal Microscopic vs. Gross Observations

Fexinidazole   Study Number: 0505-2007

ANIMAL: 2525   SEX: Male   GROUP: 4   DOSE LEVEL: 800 mg/kg/day
DAY OF DEATH: 30 Test period   STATUS: Scheduled phase sacrifice # 1   TERMINAL BODY WEIGHT (kg) : 9.33

Tissue	Gross observations/comments	Correlated Microscopic Observations

The following tissues have no gross observations and were marked as unremarkable microscopically:

ADRENALS	AORTA-THORACIC	BONE MARROW	BRAIN	CECUM
COLON	DIAPHRAGM	DUODENUM	EPIDIDYMIDES	ESOPHAGUS
EYES	FEMUR HEAD	ILEUM	JEJUNUM	KIDNEYS
LACRIMAL GLANDS	LIVER	MANDIBULAR L.N.	SKELETAL MUSCLE	SCIATIC NERVE
OPTIC NERVES	PANCREAS	PITUITARY	PROSTATE	SPINAL CORD-CERV
MANDIBULAR S.G.	SKIN	SPLEEN	STOMACH	STERNUM
TESTES	THYROIDS	TONGUE	TRACHEA	URINARY BLADDER

Fexinidazole Study Number: 0505-2007

ANIMAL: 2528 SEX: Male GROUP: 4 DOSE LEVEL: 800 mg/kg/day
DAY OF DEATH: 43 Test period STATUS: Final phase sacrifice TERMINAL BODY WEIGHT (kg) : 8.53

Tissue	Gross observations/comments	Correlated Microscopic Observations
DUODENUM	No gross observations on tissue	CRYPT DILATATION WITH/WITHOUT LUMENAL NECROTIC DEBRIS, Minimal, Focal.
GALL BLADDER	No gross observations on tissue	LYMPHOCYTIC INFILTRATION, Minimal, Multifocal.
GENER. CONDITION . . .	GOOD	No micropathology observations on tissue.
KIDNEYS	No gross observations on tissue	PAPILLARY MINERALIZATION, Minimal, Multifocal.
MESENTERIC L.N.	No gross observations on tissue	SINUS ERYTHROCYTES/ERYTHROPHAGOCYTOSIS, Minimal.
LUNG	No gross observations on tissue	ALVEOLAR MACROPHAGE INFILTRATION, Minimal, Multifocal.
MAMMARY GLAND	No gross observations on tissue	NO MAMMARY TISSUE IN THE SECTION, Present.
PITUITARY	No gross observations on tissue	CYSTS, PARS DISTALIS, Slight, Focal.
PARATHYROIDS	No gross observations on tissue	CYSTS, Minimal, Focal, Unilateral..
SKIN	No gross observations on tissue	CHRONIC INFLAMMATION, Minimal, Focal, Granulomatous. / periadnexal in the dermis

Appendix 12
Individual Animal Microscopic vs. Gross Observations

Fexinidazole Study Number: 0505-2007

ANIMAL: 2528 SEX: Male GROUP: 4 DOSE LEVEL: 800 mg/kg/day
DAY OF DEATH: 43 Test period STATUS: Final phase sacrifice TERMINAL BODY WEIGHT (kg) : 8.53

Tissue	Gross observations/comments	Correlated Microscopic Observations
THYROIDS	. . . . No gross observations on tissue	CYSTIC FOLLICLES, Slight, Focal, Unilateral..
THYMUS	. . . . No gross observations on tissue	INVOLUTION, Marked.

The following tissues have no gross observations and were not examined microscopically:
No tissue to list.

The following tissues have no gross observations and were marked as unremarkable microscopically:

ADRENALS	AORTA-THORACIC	BONE MARROW	BRAIN	CECUM
COLON	DIAPHRAGM	EPIDIDYMIDES	ESOPHAGUS	EYES
FEMUR HEAD	HEART	ILEUM	JEJUNUM	LACRIMAL GLANDS
LIVER	MANDIBULAR L.N.	SKELETAL MUSCLE	SCIATIC NERVE	OPTIC NERVES
PANCREAS	PROSTATE	SPINAL CORD-CERV	MANDIBULAR S.G.	PAROTIDS
SPLEEN	STOMACH	STERNUM	TESTES	TONGUE
TRACHEA	URINARY BLADDER			

Appendix 12
Individual Animal Microscopic vs. Gross Observations

Fexinidazole | Study Number: 0505-2007

ANIMAL: 2530 | SEX: Male | GROUP: 4 | DOSE LEVEL: 800 mg/kg/day
DAY OF DEATH: 43 Test period | STATUS: Final phase sacrifice | TERMINAL BODY WEIGHT (kg) : 6.66

Tissue	Gross observations/comments	Correlated Microscopic Observations
GALL BLADDER	No gross observations on tissue	LYMPHOCYTIC INFILTRATION, Minimal, Focal.
GENER. CONDITION . . .	FAIRLY GOOD	No micropathology observations on tissue.
KIDNEYS	No gross observations on tissue	CORTICAL TUBULAR REGENERATIVE BASOPHILIA, Minimal, Focal, Unilateral right..
MAMMARY GLAND	No gross observations on tissue	NO MAMMARY TISSUE IN THE SECTION, Present.
PROSTATE	SMALL	Examined 1 correlation found: IMMATURE, Present.
PARATHYROIDS	No gross observations on tissue	ONLY ONE PARATHYROID AVAILABLE FOR EXAMINATION, Present.
PAROTIDS	No gross observations on tissue	ACINAR ATROPHY, Slight, Focal, Unilateral..
THYMUS	No gross observations on tissue	CYSTS, Minimal, Multifocal.<br>INVOLUTION, Marked.

The following tissues have no gross observations and were not examined microscopically:
No tissue to list.

The following tissues have no gross observations and were marked as unremarkable microscopically:
ADRENALS AORTA-THORACIC BONE MARROW BRAIN CECUM

Appendix 12
Individual Animal Microscopic vs. Gross Observations

Fexinidazole — Study Number: 0505-2007

ANIMAL: 2530 | SEX: Male | GROUP: 4 | DOSE LEVEL: 800 mg/kg/day
DAY OF DEATH: 43 Test period | STATUS: Final phase sacrifice | TERMINAL BODY WEIGHT (kg) : 6.66

Tissue	Gross observations/comments	Correlated Microscopic Observations

The following tissues have no gross observations and were marked as unremarkable microscopically:

COLON	DIAPHRAGM	DUODENUM	EPIDIDYMIDES	ESOPHAGUS
EYES	FEMUR HEAD	HEART	ILEUM	JEJUNUM
LACRIMAL GLANDS	LIVER	MANDIBULAR L.N.	MESENTERIC L.N.	LUNG
SKELETAL MUSCLE	SCIATIC NERVE	OPTIC NERVES	PANCREAS	PITUITARY
SPINAL CORD-CERV	MANDIBULAR S.G.	SKIN	SPLEEN	STOMACH
STERNUM	TESTES	THYROIDS	TONGUE	TRACHEA
URINARY BLADDER				

Appendix 12
Individual Animal Microscopic vs. Gross Observations

Fexinidazole — Study Number: 0505-2007

ANIMAL: 2560 | SEX: Female | GROUP: 1 | DOSE LEVEL: Vehicle
DAY OF DEATH: 29 Test period | STATUS: Scheduled phase sacrifice # 1 | TERMINAL BODY WEIGHT (kg) : 6.35

Tissue	Gross observations/comments	Correlated Microscopic Observations
GALL BLADDER	No gross observations on tissue	LYMPHOCYTIC INFILTRATION, Minimal, Multifocal.
GENER. CONDITION . . .	GOOD	No micropathology observations on tissue.
HEART	No gross observations on tissue	MESOTHELIAL HYPERPLASIA, ATRIAL, Slight, Multifocal, Unilateral left..
KIDNEYS	No gross observations on tissue	CHRONIC INFLAMMATION, Minimal, Focal, Unilateral right..
		PAPILLARY MINERALIZATION, Minimal, Multifocal.
LIVER	No gross observations on tissue	HEPATOCELLULAR VACUOLATION, Slight, Multifocal.
MANDIBULAR L.N.	No gross observations on tissue	SINUS ERYTHROCYTES/ERYTHROPHAGOCYTOSIS, Minimal, Unilateral..
MESENTERIC L.N.	No gross observations on tissue	SINUS ERYTHROCYTES/ERYTHROPHAGOCYTOSIS, Minimal.
LUNG	No gross observations on tissue	ACUTE INFLAMMATION, Minimal, Multifocal.
		CHRONIC INFLAMMATION, Minimal, Multifocal.
		ALVEOLAR MACROPHAGE INFILTRATION, Minimal, Multifocal.

Appendix 12
Individual Animal Microscopic vs. Gross Observations

Fexinidazole Study Number: 0505-2007

ANIMAL: 2560 SEX: Female GROUP: 1 DOSE LEVEL: Vehicle
DAY OF DEATH: 29 Test period STATUS: Scheduled phase sacrifice # 1 TERMINAL BODY WEIGHT (kg) : 6.35

Tissue	Gross observations/comments	Correlated Microscopic Observations
MAMMARY GLAND	No gross observations on tissue	NO MAMMARY TISSUE IN THE SECTION, Present.
OVARIES	No gross observations on tissue	IMMATURE, Present.
PERITONEAL CAV.	CLEAR LIQUID CONTENT	No micropathology observations on tissue.
PARATHYROIDS	No gross observations on tissue	ONLY ONE PARATHYROID AVAILABLE FOR EXAMINATION, Present.
STAGE OF ESTRUS	No gross observations on tissue	IMMATURE, Present.
MANDIBULAR S.G.	No gross observations on tissue	LYMPHOCYTIC INFILTRATION, Minimal, Focal, Unilateral..
THYMUS	No gross observations on tissue	INVOLUTION, Moderate.
TONGUE	No gross observations on tissue	CHRONIC INFLAMMATION, Minimal, Multifocal.
UTERUS	No gross observations on tissue	IMMATURE, Present.

The following tissues have no gross observations and were not examined microscopically:
No tissue to list.

The following tissues have no gross observations and were marked as unremarkable microscopically:
ADRENALS AORTA-THORACIC BONE MARROW BRAIN CECUM

Appendix 12
Individual Animal Microscopic vs. Gross Observations

Fexinidazole — Study Number: 0505-2007

ANIMAL: 2560 SEX: Female GROUP: 1 DOSE LEVEL: Vehicle
DAY OF DEATH: 29 Test period STATUS: Scheduled phase sacrifice # 1 TERMINAL BODY WEIGHT (kg) : 6.35

Tissue	Gross observations/comments	Correlated Microscopic Observations

The following tissues have no gross observations and were marked as unremarkable microscopically:

COLON	DIAPHRAGM	DUODENUM	ESOPHAGUS	EYES
FEMUR HEAD	ILEUM	JEJUNUM	LACRIMAL GLANDS	SKELETAL MUSCLE
SCIATIC NERVE	OPTIC NERVES	PANCREAS	PITUITARY	SPINAL CORD-CERV
PAROTIDS	SKIN	SPLEEN	STOMACH	STERNUM
THYROIDS	TRACHEA	URINARY BLADDER	VAGINA	

Fexinidazole Study Number: 0505-2007

ANIMAL: 2568 SEX: Female GROUP: 1 DOSE LEVEL: Vehicle
DAY OF DEATH: 30 Test period STATUS: Scheduled phase sacrifice # 1 TERMINAL BODY WEIGHT (kg) : 6.90

Tissue	Gross observations/comments	Correlated Microscopic Observations
ESOPHAGUS	No gross observations on tissue	CHRONIC INFLAMMATION, Minimal, Multifocal. / in submucosa
GALL BLADDER	No gross observations on tissue	LYMPHOCYTIC INFILTRATION, Slight, Multifocal.
GENER. CONDITION . . .	GOOD	No micropathology observations on tissue.
KIDNEYS	No gross observations on tissue	PAPILLARY MINERALIZATION, Minimal, Multifocal.
LIVER	No gross observations on tissue	EXTRAMEDULLARY HEMATOPOIESIS, Minimal, Multifocal.
MESENTERIC L.N.	No gross observations on tissue	SINUS ERYTHROCYTES/ERYTHROPHAGOCYTOSIS, Minimal.
LUNG	No gross observations on tissue	ALVEOLAR MACROPHAGE INFILTRATION, Minimal, Multifocal.
MAMMARY GLAND	No gross observations on tissue	NO MAMMARY TISSUE IN THE SECTION, Present.
OVARIES	No gross observations on tissue	IMMATURE, Present.
PITUITARY	No gross observations on tissue	CYSTS, PARS DISTALIS, Minimal, Focal.

Appendix 12
Individual Animal Microscopic vs. Gross Observations

Fexinidazole | Study Number: 0505-2007

ANIMAL: 2568 | SEX: Female | GROUP: 1 | DOSE LEVEL: Vehicle
DAY OF DEATH: 30 Test period | STATUS: Scheduled phase sacrifice # 1 | TERMINAL BODY WEIGHT (kg) : 6.90

Tissue	Gross observations/comments	Correlated Microscopic Observations
PARATHYROIDS	No gross observations on tissue	ONLY ONE PARATHYROID AVAILABLE FOR EXAMINATION, Present.<br><br>CYSTS, Minimal, Multifocal.
STAGE OF ESTRUS	No gross observations on tissue	IMMATURE, Present.
PAROTIDS	No gross observations on tissue	ACINAR ATROPHY, Minimal, Focal, Unilateral..
THYMUS	No gross observations on tissue	INVOLUTION, Marked.

The following tissues have no gross observations and were not examined microscopically:
No tissue to list.

The following tissues have no gross observations and were marked as unremarkable microscopically:

ADRENALS	AORTA-THORACIC	BONE MARROW	BRAIN	CECUM
COLON	DIAPHRAGM	DUODENUM	EYES	FEMUR HEAD
HEART	ILEUM	JEJUNUM	LACRIMAL GLANDS	MANDIBULAR L.N.
SKELETAL MUSCLE	SCIATIC NERVE	OPTIC NERVES	PANCREAS	SPINAL CORD-CERV
MANDIBULAR S.G.	SKIN	SPLEEN	STOMACH	STERNUM
THYROIDS	TONGUE	TRACHEA	URINARY BLADDER	UTERUS
VAGINA				

Appendix 12
Individual Animal Microscopic vs. Gross Observations

Fexinidazole | Study Number: 0505-2007

ANIMAL: 2572 | SEX: Female | GROUP: 1 | DOSE LEVEL: Vehicle
DAY OF DEATH: 30 Test period | STATUS: Scheduled phase sacrifice # 1 | TERMINAL BODY WEIGHT (kg) : 6.60

Tissue	Gross observations/comments	Correlated Microscopic Observations
GALL BLADDER	No gross observations on tissue	LYMPHOCYTIC INFILTRATION, Minimal, Focal.
GENER. CONDITION . . .	FAIRLY GOOD	No micropathology observations on tissue.
KIDNEYS	No gross observations on tissue	PAPILLARY MINERALIZATION, Minimal, Multifocal.
LACRIMAL GLANDS	No gross observations on tissue	ONLY ONE GLAND AVAILABLE FOR EXAMINATION, Present.
MESENTERIC L.N.	No gross observations on tissue	SINUS ERYTHROCYTES/ERYTHROPHAGOCYTOSIS, Minimal.
LUNG	No gross observations on tissue	ACUTE INFLAMMATION, Minimal, Multifocal.
		ALVEOLAR MACROPHAGE INFILTRATION, Minimal, Multifocal.
MAMMARY GLAND	No gross observations on tissue	NO MAMMARY TISSUE IN THE SECTION, Present.
OVARIES	No gross observations on tissue	IMMATURE, Present.
PERITONEAL CAV.	CLEAR LIQUID CONTENT	No micropathology observations on tissue.
PARATHYROIDS	No gross observations on tissue	ONLY ONE PARATHYROID AVAILABLE FOR EXAMINATION, Present.

Fexinidazole Study Number: 0505-2007

ANIMAL: 2572 SEX: Female GROUP: 1 DOSE LEVEL: Vehicle
DAY OF DEATH: 30 Test period STATUS: Scheduled phase sacrifice # 1 TERMINAL BODY WEIGHT (kg) : 6.60

Tissue	Gross observations/comments	Correlated Microscopic Observations
STAGE OF ESTRUS	No gross observations on tissue	IMMATURE, Present.
PAROTIDS	No gross observations on tissue	ACINAR ATROPHY, Minimal, Focal, Unilateral..
THYMUS	SMALL	Examined 1 correlation found: INVOLUTION, Moderate.
		CYSTS, Minimal, Focal.
TRACHEA	No gross observations on tissue	ACUTE INFLAMMATION, Minimal, Multifocal.
UTERUS	No gross observations on tissue	IMMATURE, Present.

The following tissues have no gross observations and were not examined microscopically:
No tissue to list.

The following tissues have no gross observations and were marked as unremarkable microscopically:

ADRENALS	AORTA-THORACIC	BONE MARROW	BRAIN	CECUM
COLON	DIAPHRAGM	DUODENUM	ESOPHAGUS	EYES
FEMUR HEAD	HEART	ILEUM	JEJUNUM	LIVER
MANDIBULAR L.N.	SKELETAL MUSCLE	SCIATIC NERVE	OPTIC NERVES	PANCREAS
PITUITARY	SPINAL CORD-CERV	MANDIBULAR S.G.	SKIN	SPLEEN
STOMACH	STERNUM	THYROIDS	TONGUE	URINARY BLADDER
VAGINA				

Appendix 12
Individual Animal Microscopic vs. Gross Observations

Fexinidazole — Study Number: 0505-2007

ANIMAL: 2575 | SEX: Female | GROUP: 1 | DOSE LEVEL: Vehicle
DAY OF DEATH: 43 Test period | STATUS: Final phase sacrifice | TERMINAL BODY WEIGHT (kg) : 8.11

Tissue	Gross observations/comments	Correlated Microscopic Observations
GALL BLADDER	No gross observations on tissue	LYMPHOCYTIC INFILTRATION, Slight, Multifocal.
GENER. CONDITION	GOOD	No micropathology observations on tissue.
KIDNEYS	No gross observations on tissue	CHRONIC INFLAMMATION, Minimal, Focal, Unilateral right..
		PAPILLARY MINERALIZATION, Minimal, Multifocal.
LIVER	No gross observations on tissue	EXTRAMEDULLARY HEMATOPOIESIS, Minimal, Multifocal.
MESENTERIC L.N.	No gross observations on tissue	SINUS ERYTHROCYTES/ERYTHROPHAGOCYTOSIS, Minimal.
LUNG	No gross observations on tissue	CHRONIC INFLAMMATION, Minimal, Multifocal.
		ALVEOLAR MACROPHAGE INFILTRATION, Slight, Multifocal.
MAMMARY GLAND	No gross observations on tissue	EDEMA, Minimal.
		STROMAL PROLIFERATION, Minimal.
		DUCTAL-ALVEOLAR HYPERPLASIA, Minimal.

Appendix 12
Individual Animal Microscopic vs. Gross Observations

Fexinidazole | Study Number: 0505-2007

ANIMAL: 2575 | SEX: Female | GROUP: 1 | DOSE LEVEL: Vehicle
DAY OF DEATH: 43 Test period | STATUS: Final phase sacrifice | TERMINAL BODY WEIGHT (kg) : 8.11

Tissue	Gross observations/comments	Correlated Microscopic Observations
OVARIES	No gross observations on tissue	CORPORA LUTEA, Present.
PARATHYROIDS	No gross observations on tissue	ONLY ONE PARATHYROID AVAILABLE FOR EXAMINATION, Present.
STAGE OF ESTRUS	No gross observations on tissue	DIESTRUS, Present.
MANDIBULAR S.G.	No gross observations on tissue	LYMPHOCYTIC INFILTRATION, Minimal, Focal, Unilateral..
PAROTIDS	No gross observations on tissue	ACINAR ATROPHY, Minimal, Multifocal, Unilateral..
SPLEEN	No gross observations on tissue	EXTRAMEDULLARY HEMATOPOIESIS, Minimal.
STOMACH	No gross observations on tissue	GASTRITIS, Slight, Multifocal, Chronic active. / in the pyloric region
THYMUS	No gross observations on tissue	INVOLUTION, Minimal.
UTERUS	No gross observations on tissue	ENDOMETRIAL GLAND HYPERTROPHY/HYPERPLASIA, Slight, Diffuse.

The following tissues have no gross observations and were not examined microscopically:
No tissue to list.

Appendix 12
Individual Animal Microscopic vs. Gross Observations

Fexinidazole Study Number: 0505-2007

ANIMAL: 2575 SEX: Female GROUP: 1 DOSE LEVEL: Vehicle
DAY OF DEATH: 43 Test period STATUS: Final phase sacrifice TERMINAL BODY WEIGHT (kg) : 8.11

Tissue	Gross observations/comments	Correlated Microscopic Observations

The following tissues have no gross observations and were marked as unremarkable microscopically:

ADRENALS	AORTA-THORACIC	BONE MARROW	BRAIN	CECUM
COLON	DIAPHRAGM	DUODENUM	ESOPHAGUS	EYES
FEMUR HEAD	HEART	ILEUM	JEJUNUM	LACRIMAL GLANDS
MANDIBULAR L.N.	SKELETAL MUSCLE	SCIATIC NERVE	OPTIC NERVES	PANCREAS
PITUITARY	SPINAL CORD-CERV	SKIN	STERNUM	THYROIDS
TONGUE	TRACHEA	URINARY BLADDER	VAGINA	

Appendix 12
Individual Animal Microscopic vs. Gross Observations

Fexinidazole — Study Number: 0505-2007

ANIMAL: 2577 | SEX: Female | GROUP: 1 | DOSE LEVEL: Vehicle
DAY OF DEATH: 43 Test period | STATUS: Final phase sacrifice | TERMINAL BODY WEIGHT (kg) : 8.55

Tissue	Gross observations/comments	Correlated Microscopic Observations
GALL BLADDER	No gross observations on tissue	LYMPHOCYTIC INFILTRATION, Minimal, Multifocal.
GENER. CONDITION . . .	GOOD	No micropathology observations on tissue.
KIDNEYS	No gross observations on tissue	PAPILLARY MINERALIZATION, Minimal, Multifocal.
		CORTICAL TUBULAR DILATION, Minimal, Multifocal.
		CORTICAL FIBROSIS, Minimal, Multifocal.
		CORTICAL TUBULAR REGENERATIVE BASOPHILIA, Slight, Multifocal.
MESENTERIC L.N.	No gross observations on tissue	SINUS ERYTHROCYTES/ERYTHROPHAGOCYTOSIS, Minimal.
LUNG	No gross observations on tissue	ALVEOLAR HEMORRHAGE, Minimal, Multifocal.
		ALVEOLAR MACROPHAGE INFILTRATION, Minimal, Multifocal.
MAMMARY GLAND	No gross observations on tissue	EDEMA, Slight.
		STROMAL PROLIFERATION, Moderate.
		DUCTAL-ALVEOLAR HYPERPLASIA, Moderate.

Appendix 12
Individual Animal Microscopic vs. Gross Observations

Fexinidazole — Study Number: 0505-2007

ANIMAL: 2577 SEX: Female GROUP: 1 DOSE LEVEL: Vehicle
DAY OF DEATH: 43 Test period STATUS: Final phase sacrifice TERMINAL BODY WEIGHT (kg) : 8.55

Tissue	Gross observations/comments	Correlated Microscopic Observations
OVARIES	No gross observations on tissue	CORPORA LUTEA, Present.
PANCREAS	No gross observations on tissue	ACINAR APOPTOSIS, Minimal, Multifocal.
PITUITARY	No gross observations on tissue	CYSTS, PARS DISTALIS, Slight, Multifocal.
PARATHYROIDS	No gross observations on tissue	ONLY ONE PARATHYROID AVAILABLE FOR EXAMINATION, Present.
STAGE OF ESTRUS	No gross observations on tissue	DIESTRUS, Present.
STOMACH	No gross observations on tissue	GASTRITIS, Minimal, Multifocal, Chronic active. / in the pyloric region
THYMUS	No gross observations on tissue	INVOLUTION, Minimal.
UTERUS	No gross observations on tissue	ENDOMETRIAL GLAND HYPERTROPHY/HYPERPLASIA, Slight, Diffuse.

The following tissues have no gross observations and were not examined microscopically:
No tissue to list.

The following tissues have no gross observations and were marked as unremarkable microscopically:
ADRENALS AORTA-THORACIC BONE MARROW BRAIN CECUM

ANIMAL: 2577 SEX: Female GROUP: 1 DOSE LEVEL: Vehicle
DAY OF DEATH: 43 Test period STATUS: Final phase sacrifice TERMINAL BODY WEIGHT (kg) : 8.55

Tissue	Gross observations/comments	Correlated Microscopic Observations

The following tissues have no gross observations and were marked as unremarkable microscopically:

COLON	DIAPHRAGM	DUODENUM	ESOPHAGUS	EYES
FEMUR HEAD	HEART	ILEUM	JEJUNUM	LACRIMAL GLANDS
LIVER	MANDIBULAR L.N.	SKELETAL MUSCLE	SCIATIC NERVE	OPTIC NERVES
SPINAL CORD-CERV	MANDIBULAR S.G.	PAROTIDS	SKIN	SPLEEN
STERNUM	THYROIDS	TONGUE	TRACHEA	URINARY BLADDER
VAGINA				

Appendix 12
Individual Animal Microscopic vs. Gross Observations

Fexinidazole | Study Number: 0505-2007

ANIMAL: 2562 | SEX: Female | GROUP: 2 | DOSE LEVEL: 50 mg/kg/day
DAY OF DEATH: 29 Test period | STATUS: Scheduled phase sacrifice # 1 | TERMINAL BODY WEIGHT (kg) : 6.57

Tissue	Gross observations/comments	Correlated Microscopic Observations
GENER. CONDITION . . .	GOOD	No micropathology observations on tissue.
MAMMARY GLAND	No gross observations on tissue	EDEMA, Slight.
		STROMAL PROLIFERATION, Moderate.
		DUCTAL-ALVEOLAR HYPERPLASIA, Slight.
OVARIES	No gross observations on tissue	CORPORA LUTEA, Present.
STAGE OF ESTRUS	No gross observations on tissue	DIESTRUS, Present.
UTERUS	No gross observations on tissue	ENDOMETRIAL GLAND HYPERTROPHY/HYPERPLASIA, Minimal.
		EOSINOPHILIC SECRETORY MATERIAL IN GLANDULAR LUMEN, Minimal.

The following tissues have no gross observations and were not examined microscopically:

ADRENALS	AORTA-THORACIC	BONE MARROW	BRAIN	CECUM
COLON	DIAPHRAGM	DUODENUM	ESOPHAGUS	EYES
FEMUR HEAD	GALL BLADDER	HEART	ILEUM	JEJUNUM
KIDNEYS	LACRIMAL GLANDS	LIVER	MANDIBULAR L.N.	MESENTERIC L.N.
LUNG	SKELETAL MUSCLE	SCIATIC NERVE	OPTIC NERVES	PANCREAS
PITUITARY	PARATHYROIDS	SPINAL CORD-CERV	MANDIBULAR S.G.	PAROTIDS
SKIN	SPLEEN	STOMACH	STERNUM	THYROIDS
THYMUS	TONGUE	TRACHEA	URINARY BLADDER	

The following tissues have no gross observations and were marked as unremarkable microscopically:
VAGINA

Appendix 12
Individual Animal Microscopic vs. Gross Observations

Fexinidazole Study Number: 0505-2007

ANIMAL: 2563 SEX: Female GROUP: 2 DOSE LEVEL: 50 mg/kg/day
DAY OF DEATH: 30 Test period STATUS: Scheduled phase sacrifice # 1 TERMINAL BODY WEIGHT (kg) : 7.61

Tissue	Gross observations/comments	Correlated Microscopic Observations
GENER. CONDITION . . .	GOOD	No micropathology observations on tissue.
MAMMARY GLAND	No gross observations on tissue	IMMATURE, Present.
STAGE OF ESTRUS	No gross observations on tissue	ESTRUS, Present.
UTERUS	No gross observations on tissue	ENDOMETRIAL GLAND HYPERTROPHY/HYPERPLASIA, Minimal.

The following tissues have no gross observations and were not examined microscopically:

ADRENALS	AORTA-THORACIC	BONE MARROW	BRAIN	CECUM
COLON	DIAPHRAGM	DUODENUM	ESOPHAGUS	EYES
FEMUR HEAD	GALL BLADDER	HEART	ILEUM	JEJUNUM
KIDNEYS	LACRIMAL GLANDS	LIVER	MANDIBULAR L.N.	MESENTERIC L.N.
LUNG	SKELETAL MUSCLE	SCIATIC NERVE	OPTIC NERVES	PANCREAS
PITUITARY	PARATHYROIDS	SPINAL CORD-CERV	MANDIBULAR S.G.	PAROTIDS
SKIN	SPLEEN	STOMACH	STERNUM	THYROIDS
THYMUS	TONGUE	TRACHEA	URINARY BLADDER	

The following tissues have no gross observations and were marked as unremarkable microscopically:

OVARIES VAGINA

Appendix 12
Individual Animal Microscopic vs. Gross Observations

Fexinidazole   Study Number: 0505-2007

ANIMAL: 2576   SEX: Female   GROUP: 2   DOSE LEVEL: 50 mg/kg/day
DAY OF DEATH: 30 Test period   STATUS: Scheduled phase sacrifice # 1   TERMINAL BODY WEIGHT (kg) : 8.38

Tissue	Gross observations/comments	Correlated Microscopic Observations
GENER. CONDITION . . .	GOOD	No micropathology observations on tissue.
MAMMARY GLAND	No gross observations on tissue	EDEMA, Minimal.<br>STROMAL PROLIFERATION, Minimal.<br>DUCTAL-ALVEOLAR HYPERPLASIA, Minimal.
OVARIES	No gross observations on tissue	CORPORA LUTEA, Present.
STAGE OF ESTRUS	No gross observations on tissue	DIESTRUS, Present.
UTERUS	No gross observations on tissue	ENDOMETRIAL GLAND HYPERTROPHY/HYPERPLASIA, Moderate.

The following tissues have no gross observations and were not examined microscopically:

ADRENALS	AORTA-THORACIC	BONE MARROW	BRAIN	CECUM
COLON	DIAPHRAGM	DUODENUM	ESOPHAGUS	EYES
FEMUR HEAD	GALL BLADDER	HEART	ILEUM	JEJUNUM
KIDNEYS	LACRIMAL GLANDS	LIVER	MANDIBULAR L.N.	MESENTERIC L.N.
LUNG	SKELETAL MUSCLE	SCIATIC NERVE	OPTIC NERVES	PANCREAS
PITUITARY	PARATHYROIDS	SPINAL CORD-CERV	MANDIBULAR S.G.	PAROTIDS
SKIN	SPLEEN	STOMACH	STERNUM	THYROIDS
THYMUS	TONGUE	TRACHEA	URINARY BLADDER	

The following tissues have no gross observations and were marked as unremarkable microscopically:
VAGINA

Appendix 12
Individual Animal Microscopic vs. Gross Observations

Fexinidazole Study Number: 0505-2007

ANIMAL: 2561 SEX: Female GROUP: 3 DOSE LEVEL: 200 mg/kg/day
DAY OF DEATH: 29 Test period STATUS: Scheduled phase sacrifice # 1 TERMINAL BODY WEIGHT (kg) : 7.10

Tissue	Gross observations/comments	Correlated Microscopic Observations
GENER. CONDITION . . .	GOOD	No micropathology observations on tissue.
MAMMARY GLAND	No gross observations on tissue	EDEMA, Minimal.
		STROMAL PROLIFERATION, Slight.
		DUCTAL-ALVEOLAR HYPERPLASIA, Moderate.
OVARIES	No gross observations on tissue	CORPORA LUTEA, Present.
STAGE OF ESTRUS	No gross observations on tissue	DIESTRUS, Present.
UTERUS	No gross observations on tissue	EOSINOPHILIC SECRETORY MATERIAL IN GLANDULAR LUMEN, Minimal.

The following tissues have no gross observations and were not examined microscopically:

ADRENALS	AORTA-THORACIC	BONE MARROW	BRAIN	CECUM
COLON	DIAPHRAGM	DUODENUM	ESOPHAGUS	EYES
FEMUR HEAD	GALL BLADDER	HEART	ILEUM	JEJUNUM
KIDNEYS	LACRIMAL GLANDS	LIVER	MANDIBULAR L.N.	MESENTERIC L.N.
LUNG	SKELETAL MUSCLE	SCIATIC NERVE	OPTIC NERVES	PANCREAS
PITUITARY	PARATHYROIDS	SPINAL CORD-CERV	MANDIBULAR S.G.	PAROTIDS
SKIN	SPLEEN	STOMACH	STERNUM	THYROIDS
THYMUS	TONGUE	TRACHEA	URINARY BLADDER	

The following tissues have no gross observations and were marked as unremarkable microscopically:
VAGINA

Appendix 12
Individual Animal Microscopic vs. Gross Observations

Fexinidazole | Study Number: 0505-2007

ANIMAL: 2570 | SEX: Female | GROUP: 3 | DOSE LEVEL: 200 mg/kg/day
DAY OF DEATH: 30 Test period | STATUS: Scheduled phase sacrifice # 1 | TERMINAL BODY WEIGHT (kg) : 6.65

Tissue	Gross observations/comments	Correlated Microscopic Observations
GENER. CONDITION . . .	FAIRLY GOOD	No micropathology observations on tissue.
MAMMARY GLAND	No gross observations on tissue	EDEMA, Moderate.<br>STROMAL PROLIFERATION, Moderate.<br>DUCTAL-ALVEOLAR HYPERPLASIA, Slight.
OVARIES	No gross observations on tissue	CORPORA LUTEA, Present.
PERITONEAL CAV.	CLEAR LIQUID CONTENT	No micropathology observations on tissue.
STAGE OF ESTRUS	No gross observations on tissue	DIESTRUS, Present.
THYMUS	SMALL	Tissue is missing.<br>only adipose tissue detectable in the samples evaluated
UTERUS	No gross observations on tissue	ENDOMETRIAL GLAND HYPERTROPHY/HYPERPLASIA, Slight.<br>EOSINOPHILIC SECRETORY MATERIAL IN GLANDULAR LUMEN, Minimal.

Appendix 12
Individual Animal Microscopic vs. Gross Observations

Fexinidazole Study Number: 0505-2007

ANIMAL: 2570 SEX: Female GROUP: 3 DOSE LEVEL: 200 mg/kg/day
DAY OF DEATH: 30 Test period STATUS: Scheduled phase sacrifice # 1 TERMINAL BODY WEIGHT (kg) : 6.65

Tissue	Gross observations/comments	Correlated Microscopic Observations

The following tissues have no gross observations and were not examined microscopically:

ADRENALS	AORTA-THORACIC	BONE MARROW	BRAIN	CECUM
COLON	DIAPHRAGM	DUODENUM	ESOPHAGUS	EYES
FEMUR HEAD	GALL BLADDER	HEART	ILEUM	JEJUNUM
KIDNEYS	LACRIMAL GLANDS	LIVER	MANDIBULAR L.N.	MESENTERIC L.N.
LUNG	SKELETAL MUSCLE	SCIATIC NERVE	OPTIC NERVES	PANCREAS
PITUITARY	PARATHYROIDS	SPINAL CORD-CERV	MANDIBULAR S.G.	PAROTIDS
SKIN	SPLEEN	STOMACH	STERNUM	THYROIDS
TONGUE	TRACHEA	URINARY BLADDER	VAGINA	

The following tissues have no gross observations and were marked as unremarkable microscopically:
No tissue to list.

Appendix 12
Individual Animal Microscopic vs. Gross Observations

Fexinidazole Study Number: 0505-2007

ANIMAL: 2567 SEX: Female GROUP: 3 DOSE LEVEL: 200 mg/kg/day
DAY OF DEATH: 30 Test period STATUS: Scheduled phase sacrifice # 1 TERMINAL BODY WEIGHT (kg) : 8.00

Tissue	Gross observations/comments	Correlated Microscopic Observations
GENER. CONDITION . . .	GOOD	No micropathology observations on tissue.
MAMMARY GLAND	No gross observations on tissue	EDEMA, Slight.<br>STROMAL PROLIFERATION, Slight.<br>DUCTAL-ALVEOLAR HYPERPLASIA, Minimal.
OVARIES	No gross observations on tissue	CORPORA LUTEA, Present.
STAGE OF ESTRUS	No gross observations on tissue	DIESTRUS, Present.
UTERUS	No gross observations on tissue	ENDOMETRIAL GLAND HYPERTROPHY/HYPERPLASIA, Moderate.

The following tissues have no gross observations and were not examined microscopically:

ADRENALS	AORTA-THORACIC	BONE MARROW	BRAIN	CECUM
COLON	DIAPHRAGM	DUODENUM	ESOPHAGUS	EYES
FEMUR HEAD	GALL BLADDER	HEART	ILEUM	JEJUNUM
KIDNEYS	LACRIMAL GLANDS	LIVER	MANDIBULAR L.N.	MESENTERIC L.N.
LUNG	SKELETAL MUSCLE	SCIATIC NERVE	OPTIC NERVES	PANCREAS
PITUITARY	PARATHYROIDS	SPINAL CORD-CERV	MANDIBULAR S.G.	PAROTIDS
SKIN	SPLEEN	STOMACH	STERNUM	THYROIDS
THYMUS	TONGUE	TRACHEA	URINARY BLADDER	VAGINA

The following tissues have no gross observations and were marked as unremarkable microscopically:
No tissue to list.

Appendix 12
Individual Animal Microscopic vs. Gross Observations

Fexinidazole   Study Number: 0505-2007

ANIMAL: 2564   SEX: Female   GROUP: 4   DOSE LEVEL: 800 mg/kg/day
DAY OF DEATH: 29 Test period   STATUS: Scheduled phase sacrifice # 1   TERMINAL BODY WEIGHT (kg) : 6.10

Tissue	Gross observations/comments	Correlated Microscopic Observations
AORTA-THORACIC	No gross observations on tissue	ECTOPIC THYROID, Present.
ESOPHAGUS	No gross observations on tissue	CHRONIC INFLAMMATION, Minimal, Focal. / associated with submucosal glands
GALL BLADDER	No gross observations on tissue	LYMPHOCYTIC INFILTRATION, Minimal, Multifocal.
GENER. CONDITION . . .	GOOD	No micropathology observations on tissue.
KIDNEYS	No gross observations on tissue	PAPILLARY MINERALIZATION, Minimal, Multifocal.
LIVER	No gross observations on tissue	CHRONIC INFLAMMATION, Minimal, Multifocal.
MESENTERIC L.N.	No gross observations on tissue	SINUS ERYTHROCYTES/ERYTHROPHAGOCYTOSIS, Minimal.
LUNG	No gross observations on tissue	ACUTE INFLAMMATION, Minimal, Focal.<br>ALVEOLAR MACROPHAGE INFILTRATION, Minimal, Multifocal.
MAMMARY GLAND	No gross observations on tissue	LOBULAR HYPERPLASIA, Slight.<br>SECRETORY ACTIVITY, Slight.

Appendix 12
Individual Animal Microscopic vs. Gross Observations

Fexinidazole | Study Number: 0505-2007

ANIMAL: 2564 | SEX: Female | GROUP: 4 | DOSE LEVEL: 800 mg/kg/day
DAY OF DEATH: 29 Test period | STATUS: Scheduled phase sacrifice # 1 | TERMINAL BODY WEIGHT (kg) : 6.10

Tissue	Gross observations/comments	Correlated Microscopic Observations
OVARIES	No gross observations on tissue	CORPORA LUTEA, Present.
PARATHYROIDS	No gross observations on tissue	ONLY ONE PARATHYROID AVAILABLE FOR EXAMINATION, Present.
STAGE OF ESTRUS	No gross observations on tissue	DIESTRUS, Present.
MANDIBULAR S.G.	No gross observations on tissue	LYMPHOCYTIC INFILTRATION, Slight, Focal, Unilateral..
THYMUS	No gross observations on tissue	INVOLUTION, Marked.
UTERUS	No gross observations on tissue	EOSINOPHILIC SECRETORY MATERIAL IN GLANDULAR LUMEN, Slight, Diffuse.

The following tissues have no gross observations and were not examined microscopically:
No tissue to list.

The following tissues have no gross observations and were marked as unremarkable microscopically:

ADRENALS	BONE MARROW	BRAIN	CECUM	COLON
DIAPHRAGM	DUODENUM	EYES	FEMUR HEAD	HEART
ILEUM	JEJUNUM	LACRIMAL GLANDS	MANDIBULAR L.N.	SKELETAL MUSCLE
SCIATIC NERVE	OPTIC NERVES	PANCREAS	PITUITARY	SPINAL CORD-CERV
PAROTIDS	SKIN	SPLEEN	STOMACH	STERNUM
THYROIDS	TONGUE	TRACHEA	URINARY BLADDER	VAGINA

Appendix 12
Individual Animal Microscopic vs. Gross Observations

Fexinidazole Study Number: 0505-2007

ANIMAL: 2565 SEX: Female GROUP: 4 DOSE LEVEL: 800 mg/kg/day
DAY OF DEATH: 30 Test period STATUS: Scheduled phase sacrifice # 1 TERMINAL BODY WEIGHT (kg) : 6.11

Tissue	Gross observations/comments	Correlated Microscopic Observations
DUODENUM	No gross observations on tissue	CRYPT DILATATION WITH/WITHOUT LUMENAL NECROTIC DEBRIS, Minimal, Focal.
GALL BLADDER	No gross observations on tissue	LYMPHOCYTIC INFILTRATION, Minimal, Multifocal.
GENER. CONDITION . . .	GOOD	No micropathology observations on tissue.
HEART	No gross observations on tissue	MESOTHELIAL HYPERPLASIA, ATRIAL, Slight, Multifocal, Unilateral right..
KIDNEYS	No gross observations on tissue	PAPILLARY MINERALIZATION, Minimal, Multifocal.
LIVER	No gross observations on tissue	CHRONIC INFLAMMATION, Minimal, Multifocal.
LUNG	No gross observations on tissue	ALVEOLAR MACROPHAGE INFILTRATION, Slight, Multifocal.
		BRONCHOPNEUMONIA, Slight, Multifocal, Subacute.
MAMMARY GLAND	No gross observations on tissue	EDEMA, Slight.
		STROMAL PROLIFERATION, Slight.
		DUCTAL-ALVEOLAR HYPERPLASIA, Slight.

Appendix 12
Individual Animal Microscopic vs. Gross Observations

Fexinidazole   Study Number: 0505-2007

ANIMAL: 2565   SEX: Female   GROUP: 4   DOSE LEVEL: 800 mg/kg/day
DAY OF DEATH: 30 Test period   STATUS: Scheduled phase sacrifice # 1   TERMINAL BODY WEIGHT (kg) : 6.11

Tissue	Gross observations/comments	Correlated Microscopic Observations
OVARIES	No gross observations on tissue	CORPORA LUTEA, Present.
PARATHYROIDS	No gross observations on tissue	ONLY ONE PARATHYROID AVAILABLE FOR EXAMINATION, Present.<br>CYSTS, Minimal, Focal. / in adjacent tissue
STAGE OF ESTRUS	No gross observations on tissue	DIESTRUS, Present.
PAROTIDS	No gross observations on tissue	LYMPHOCYTIC INFILTRATION, Minimal, Multifocal.
THYMUS	No gross observations on tissue	INVOLUTION, Marked.
UTERUS	No gross observations on tissue	ENDOMETRIAL GLAND HYPERTROPHY/HYPERPLASIA, Moderate, Diffuse.

The following tissues have no gross observations and were not examined microscopically:
URINARY BLADDER

The following tissues have no gross observations and were marked as unremarkable microscopically:

ADRENALS	AORTA-THORACIC	BONE MARROW	BRAIN	CECUM
COLON	DIAPHRAGM	ESOPHAGUS	EYES	FEMUR HEAD
ILEUM	JEJUNUM	LACRIMAL GLANDS	MANDIBULAR L.N.	MESENTERIC L.N.
SKELETAL MUSCLE	SCIATIC NERVE	OPTIC NERVES	PANCREAS	PITUITARY
SPINAL CORD-CERV	MANDIBULAR S.G.	SKIN	SPLEEN	STOMACH
STERNUM	THYROIDS	TONGUE	TRACHEA	VAGINA

ANIMAL: 2569 SEX: Female GROUP: 4 DOSE LEVEL: 800 mg/kg/day
DAY OF DEATH: 30 Test period STATUS: Scheduled phase sacrifice # 1 TERMINAL BODY WEIGHT (kg) : 7.65

Tissue	Gross observations/comments	Correlated Microscopic Observations
DUODENUM	No gross observations on tissue	CRYPT DILATATION WITH/WITHOUT LUMENAL NECROTIC DEBRIS, Minimal, Focal.
GALL BLADDER	No gross observations on tissue	LYMPHOCYTIC INFILTRATION, Slight, Multifocal.
GENER. CONDITION . . .	GOOD	No micropathology observations on tissue.
HEART	No gross observations on tissue	ARTERIAL MEDIAL HYPERTROPHY, Minimal, Focal, Unilateral right.. / in the atrium
LACRIMAL GLANDS	No gross observations on tissue	ONLY ONE GLAND AVAILABLE FOR EXAMINATION, Present.
LIVER	No gross observations on tissue	CHRONIC INFLAMMATION, Minimal, Multifocal.
MESENTERIC L.N.	No gross observations on tissue	SINUS ERYTHROCYTES/ERYTHROPHAGOCYTOSIS, Minimal.
LUNG	No gross observations on tissue	ALVEOLAR MACROPHAGE INFILTRATION, Minimal, Multifocal.
MAMMARY GLAND	No gross observations on tissue	EDEMA, Slight.
		STROMAL PROLIFERATION, Slight.
		DUCTAL-ALVEOLAR HYPERPLASIA, Minimal.

Fexinidazole Study Number: 0505-2007

ANIMAL: 2569 SEX: Female GROUP: 4 DOSE LEVEL: 800 mg/kg/day
DAY OF DEATH: 30 Test period STATUS: Scheduled phase sacrifice # 1 TERMINAL BODY WEIGHT (kg) : 7.65

Tissue	Gross observations/comments	Correlated Microscopic Observations
OVARIES	No gross observations on tissue	CORPORA LUTEA, Present.
PARATHYROIDS	No gross observations on tissue	ONLY ONE PARATHYROID AVAILABLE FOR EXAMINATION, Present.
STAGE OF ESTRUS	No gross observations on tissue	METESTRUS, Present.
THYMUS	No gross observations on tissue	CYSTS, Slight, Multifocal.
		INVOLUTION, Marked.
UTERUS	No gross observations on tissue	ENDOMETRIAL GLAND HYPERTROPHY/HYPERPLASIA, Slight, Diffuse.

The following tissues have no gross observations and were not examined microscopically:
No tissue to list.

The following tissues have no gross observations and were marked as unremarkable microscopically:

ADRENALS	AORTA-THORACIC	BONE MARROW	BRAIN	CECUM
COLON	DIAPHRAGM	ESOPHAGUS	EYES	FEMUR HEAD
ILEUM	JEJUNUM	KIDNEYS	MANDIBULAR L.N.	SKELETAL MUSCLE
SCIATIC NERVE	OPTIC NERVES	PANCREAS	PITUITARY	SPINAL CORD-CERV
MANDIBULAR S.G.	PAROTIDS	SKIN	SPLEEN	STOMACH
STERNUM	THYROIDS	TONGUE	TRACHEA	URINARY BLADDER
VAGINA				

Appendix 12
Individual Animal Microscopic vs. Gross Observations

Fexinidazole | Study Number: 0505-2007

ANIMAL: 2571 | SEX: Female | GROUP: 4 | DOSE LEVEL: 800 mg/kg/day
DAY OF DEATH: 43 Test period | STATUS: Final phase sacrifice | TERMINAL BODY WEIGHT (kg) : 7.32

Tissue	Gross observations/comments	Correlated Microscopic Observations
GENER. CONDITION . . .	GOOD	No micropathology observations on tissue.
HEART	No gross observations on tissue	MESOTHELIAL HYPERPLASIA, ATRIAL, Minimal, Focal, Unilateral left..
KIDNEYS	No gross observations on tissue	PAPILLARY MINERALIZATION, Minimal, Multifocal.
MESENTERIC L.N.	No gross observations on tissue	SINUS ERYTHROCYTES/ERYTHROPHAGOCYTOSIS, Minimal.
MAMMARY GLAND	No gross observations on tissue	EDEMA, Slight.
		STROMAL PROLIFERATION, Moderate.
		DUCTAL-ALVEOLAR HYPERPLASIA, Moderate.
SKELETAL MUSCLE	No gross observations on tissue	CHRONIC INFLAMMATION, Minimal, Focal.
OVARIES	No gross observations on tissue	CORPORA LUTEA, Present.
PANCREAS	No gross observations on tissue	ACINAR APOPTOSIS, Minimal, Multifocal.
PARATHYROIDS	No gross observations on tissue	ONLY ONE PARATHYROID AVAILABLE FOR EXAMINATION, Present.

Appendix 12
Individual Animal Microscopic vs. Gross Observations

Fexinidazole

Study Number: 0505-2007

ANIMAL: 2571 SEX: Female GROUP: 4 DOSE LEVEL: 800 mg/kg/day
DAY OF DEATH: 43 Test period STATUS: Final phase sacrifice TERMINAL BODY WEIGHT (kg) : 7.32

Tissue	Gross observations/comments	Correlated Microscopic Observations
STAGE OF ESTRUS	No gross observations on tissue	DIESTRUS, Present.
MANDIBULAR S.G.	No gross observations on tissue	LYMPHOCYTIC INFILTRATION, Slight, Focal, Unilateral..
SPLEEN	No gross observations on tissue	EXTRAMEDULLARY HEMATOPOIESIS, Minimal.
STOMACH	No gross observations on tissue	ACUTE INFLAMMATION, Minimal, Multifocal. / in the pyloric region
THYMUS	No gross observations on tissue	INVOLUTION, Minimal.
UTERUS	No gross observations on tissue	ENDOMETRIAL GLAND HYPERTROPHY/HYPERPLASIA, Minimal, Diffuse.<br>EOSINOPHILIC SECRETORY MATERIAL IN GLANDULAR LUMEN, Minimal, Multifocal.

The following tissues have no gross observations and were not examined microscopically:
No tissue to list.

The following tissues have no gross observations and were marked as unremarkable microscopically:

ADRENALS	AORTA-THORACIC	BONE MARROW	BRAIN	CECUM
COLON	DIAPHRAGM	DUODENUM	ESOPHAGUS	EYES
FEMUR HEAD	GALL BLADDER	ILEUM	JEJUNUM	LACRIMAL GLANDS
LIVER	MANDIBULAR L.N.	LUNG	SCIATIC NERVE	OPTIC NERVES
PITUITARY	SPINAL CORD-CERV	PAROTIDS	SKIN	STERNUM
THYROIDS	TONGUE	TRACHEA	URINARY BLADDER	VAGINA

ANIMAL: 2573 SEX: Female GROUP: 4 DOSE LEVEL: 800 mg/kg/day
DAY OF DEATH: 43 Test period STATUS: Final phase sacrifice TERMINAL BODY WEIGHT (kg) : 6.83

Tissue	Gross observations/comments	Correlated Microscopic Observations
DUODENUM	No gross observations on tissue	ECTOPIC PANCREATIC TISSUE IN SUBMUCOSA, Present.
GALL BLADDER	No gross observations on tissue	LYMPHOCYTIC INFILTRATION, Minimal, Multifocal.
GENER. CONDITION . . .	GOOD	No micropathology observations on tissue.
KIDNEYS	No gross observations on tissue	CHRONIC INFLAMMATION, Minimal, Focal, Unilateral left..
		PAPILLARY MINERALIZATION, Minimal, Multifocal.
MESENTERIC L.N.	No gross observations on tissue	SINUS ERYTHROCYTES/ERYTHROPHAGOCYTOSIS, Minimal.
LUNG	No gross observations on tissue	ALVEOLAR MACROPHAGE INFILTRATION, Minimal, Multifocal.
MAMMARY GLAND	No gross observations on tissue	EDEMA, Moderate.
		STROMAL PROLIFERATION, Moderate.
		DUCTAL-ALVEOLAR HYPERPLASIA, Moderate.
OVARIES	No gross observations on tissue	CORPORA LUTEA, Present.
		CYSTS, Slight, Focal, Unilateral..

Appendix 12
Individual Animal Microscopic vs. Gross Observations

Fexinidazole Study Number: 0505-2007

ANIMAL: 2573 SEX: Female GROUP: 4 DOSE LEVEL: 800 mg/kg/day
DAY OF DEATH: 43 Test period STATUS: Final phase sacrifice TERMINAL BODY WEIGHT (kg) : 6.83

Tissue	Gross observations/comments	Correlated Microscopic Observations
PITUITARY	No gross observations on tissue	CRANIOPHARINGEAL CYSTS, Moderate, Focal.
PARATHYROIDS	No gross observations on tissue	ONLY ONE PARATHYROID AVAILABLE FOR EXAMINATION, Present.
STAGE OF ESTRUS	No gross observations on tissue	DIESTRUS, Present.
PAROTIDS	No gross observations on tissue	ACINAR ATROPHY, Minimal, Focal.
STOMACH	No gross observations on tissue	ACUTE INFLAMMATION, Minimal, Multifocal. / in the pyloric region
THYMUS	No gross observations on tissue	CYSTS, Minimal, Multifocal.
		INVOLUTION, Moderate.
UTERUS	No gross observations on tissue	ENDOMETRIAL GLAND HYPERTROPHY/HYPERPLASIA, Minimal, Diffuse.
		EOSINOPHILIC SECRETORY MATERIAL IN GLANDULAR LUMEN, Minimal, Multifocal.

The following tissues have no gross observations and were not examined microscopically:
No tissue to list.

The following tissues have no gross observations and were marked as unremarkable microscopically:
ADRENALS AORTA-THORACIC BONE MARROW BRAIN CECUM

Appendix 12
Individual Animal Microscopic vs. Gross Observations

Fexinidazole — Study Number: 0505-2007

ANIMAL: 2573 SEX: Female GROUP: 4 DOSE LEVEL: 800 mg/kg/day
DAY OF DEATH: 43 Test period STATUS: Final phase sacrifice TERMINAL BODY WEIGHT (kg) : 6.83

Tissue	Gross observations/comments	Correlated Microscopic Observations

The following tissues have no gross observations and were marked as unremarkable microscopically:

COLON	DIAPHRAGM	ESOPHAGUS	EYES	FEMUR HEAD
HEART	ILEUM	JEJUNUM	LACRIMAL GLANDS	LIVER
MANDIBULAR L.N.	SKELETAL MUSCLE	SCIATIC NERVE	OPTIC NERVES	PANCREAS
SPINAL CORD-CERV	MANDIBULAR S.G.	SKIN	SPLEEN	STERNUM
THYROIDS	TONGUE	TRACHEA	URINARY BLADDER	VAGINA

Appendix 13 Toxicokinetic Report

TOXICOKINETIC REPORT FOR THE STUDY

Fexinidazole: 28-day Oral Toxicity Study in the Dog

Nerviano Medical Sciences Study Number: 0505-2007

Study Director:

Status Final

TABLE OF CONTENTS

1. INTRODUCTION AND OBJECTIVE 3
2. STUDY SPONSOR 3
3. TEST FACILITY 3
4. REGULATORY REQUIREMENTS 3
5. ABBREVIATIONS 3
6. METHODS 4
  6.1. Study Design 4
  6.2. Sample Information 4
  6.3. Bioanalytical Method 5
  6.4. Pharmacokinetic Calculations 5
7. RESULTS 5
  7.1. Tables and Figures 5
  7.2. Pharmacokinetic Results 6
8. CONCLUSIONS 9
9. CONTRIBUTORS 9
10. ARCHIVING 9
11. REFERENCES 9
TABLES AND FIGURES 10
APPENDICES
  Appendix 1. Individual plasma concentrations 59
  Appendix 2. In-Study Bioanalytical Validation Data 81

1. INTRODUCTION AND OBJECTIVE

Fexinidazole is a 5-nitroimidazole derivate, biologically active against Trypanosoma parasites (*T.b.rhodesiense* and *T.b. brucei*) and useful in the treatment of the Human African trypanosomiasis (HAT), known as sleeping sickness.

As part of a GLP toxicity study, the toxicokinetics of Fexinidazole and its sulfone and sulfoxide metabolites were evaluated after the first and repeated oral administrations of Fexinidazole to male and female Beagle dogs.

2. STUDY SPONSOR

DND*i* – Drugs for Neglected Diseases *Initiative*
1 Place St Gervais
CH - 1201 Geneva, Switzerland.

3. TEST FACILITY

Accelera

4. REGULATORY REQUIREMENTS

This study was conducted in compliance with

- Decreto Legislativo 2 Marzo 2007, No. 50;

- Organization for Economic Co-operation and Development (OECD) Principles of Good Laboratory Practice (GLP) (as revised in 1997).

The methods employed in this study were those described in the "Standard Operating Procedures" of the laboratories involved.

5. ABBREVIATIONS

The following abbreviations are used in this document:

AUC_{0-24}	Area under the plasma concentration *vs.* time curve up to 24 hours post dosing
$AUC_{0-t(last)}$	Area under the plasma concentration *vs.* time curve up to finite time
C_{max}	Maximal plasma concentration
CV	Coefficient of variation of the mean
F	Female
ID	Animal identification code
LC	Liquid chromatography

LLOQ	Lower limit of quantification
M	Male
MS	Mass-spectrometry
QC	Quality control sample
R^2	Correlation coefficient
R_A	Accumulation ratio
SD	Standard deviation of the mean
STD	Standard sample
$t_{1/2,z}$	Terminal half-life
t_{max}	Time to peak plasma concentration
ULOQ	Upper limit of quantification

6. METHODS

6.1. Study Design

The study was conducted according to the study protocol and related amendment [1,2]. Fexinidazole was orally given by gavage once a day for 28 days to male and female Beagle dogs according to the following scheme

Dose (mg/kg/day)	Volume (mL/kg/day)	Dog ID
0	10	M: 2516, 2518, 2520, 2527, 2533; F: 2560, 2568, 2572, 2575, 2577
50	10	M: 2514, 2521, 2529; F: 2562, 2563, 2576
200	10	M: 2515, 2523, 2526; F: 2561, 2567, 2570
800	10	M: 2517, 2519, 2525, 2528, 2530; F: 2564, 2565, 2569, 2571, 2573

Fexinidazole was suspended with 5% Tween 80 in 0.5% Methyl cellulose 400 cP.

6.2. Sample Information

Blood samples (about 1 mL/sampling time) were withdrawn from peripheral veins and put in heparinized plastic tubes kept on a ice-water bath, then centrifuged for 10 min at 1200g at +4°C and two aliquots of about 200 µL of plasma were stored in a freezer at -80°C until analysis. Blood was taken on Days 1, 14 from three dogs/gender/dose at pre-dose and 0.5, 1, 2, 4, 8 and 24 hours post dosing; on Day 28, blood was taken from three dogs/gender after 50 and 200 mg/kg/day at pre-dose and 0.5, 1, 2, 4, 8 and 24 hours post dosing and from five dogs/gender after 800 mg/kg/day at pre-dose and 1, 2, 4, 8, 24, 48 (recovery animals, [n=2]) and 72 (recovery animals, [n=2]) hours post dosing. After the administration of the vehicle, the samples were taken from five dogs/gender at all timepoints.

6.3. Bioanalytical Method

Dog plasma concentrations of Fexinidazole and its sulfoxide (M1) and sulfone (M2) metabolites were determined by a validated LC/MS/MS method [3,4]. The calibration range for the assay was 5-1000 ng/mL for Fexinidazole and 25-25000 ng/mL for M1 and M2. Study samples containing analytes concentrations above the upper limit of quantification were diluted with blank matrix prior to analysis.

6.4. Pharmacokinetic Calculations

Pharmacokinetic calculations were performed using a non-compartmental approach with the aid of Watson package (v. 6.4.0.04, Thermo Fisher Scientific, Waltham, MA, USA) and Excel spreadsheet (Microsoft Inc., Seattle, USA).

In the calculations, the undetectable concentrations between detectable ones were ignored.

After each dose level, C_{max} and t_{max} of Fexinidazole and sulfone and sulfoxide metabolites were read from raw data as the coordinates of the highest measured concentration. The area under plasma concentration *vs.* time curve up to finite time, $AUC_{0-t(last)}$, was determined for each compound by the linear trapezoidal rule up to the last detectable concentration. On Day 28, since the blood sampling was performed up to 72 hours post dosing, in addition to $AUC_{0-t(last)}$, AUC_{0-24} of both metabolites were calculated. The AUC_{0-24} was calculated in order to compare AUC values after Day 28 to those after Day 1 and Day 14.

On Day 28, in the two animals underwent recovery, the half-life of the terminal phase, $t_{1/2,z}$, was determined by linear regression analysis of the natural-log concentration *vs.* time curve, where $t_{1/2,z} = \ln(2)/\text{slope}$ of the regression line.

After each dose, C_{max} and AUC values of each compound were also normalized to 1 mg/kg/day dose level.

Fexinidazole and metabolites accumulation ratios, based on C_{max} and $AUC_{0-t(last)}$ (AUC_{0-24} for Day 28 sulfone and male sulfoxide data) were calculated as the ratio between the parameters obtained on Day 14 and Day 28 to the corresponding one on Day 1.

Descriptive statistics (mean ± SD, %CV) were reported for plasma concentrations and pharmacokinetic parameters of each compound sorted by dose, gender and day of administration.

Plasma concentrations and pharmacokinetic parameters of Fexinidazole and both metabolites were reported to three significant figures.

7. RESULTS

7.1. Tables and Figures

Mean C_{max}, t_{max} and $AUC_{0-t(last)}$ parameters of each compound are reported in Tables 1 - 3, whilst individual and mean parameters of each compound are reported in Tables 4 - 57. Individual and mean (±SD, CV%) plasma concentrations of Fexinidazole and metabolites

are reported in Tables 1A1 - 21A1 of Appendix 1. Individual plasma concentrations of Fexinidazole and both metabolites are plot in Figures 1 - 15, whilst the mean concentrations are plot in Figures 16 - 21. Mean normalized C_{max} and $AUC_{0-t(last)}$ values of Fexinidazole and metabolites vs. dose are plot in Figures 22 - 27.

In-study bioanalytical validation data are reported in Appendix 2. All analytical batches met acceptance criteria as described in PCD-M-BPK-001-01 SOP [5]. Bioanalytical data are stored in Watson LIMS (v. 6.4.0.04, Thermo Fisher Scientific, Waltham, MA, USA) under Project ID: 348-Fexinidazole and Study ID 0505-2007. Certificates of analysis are reported in Appendix 3.

7.2. Pharmacokinetic Results

Control plasma samples were analysed only at pre-dose and 2 hours post dosing.
No detectable concentration of each compound was measured in the control samples.

Day 1
Mean ±SD systemic exposure to Fexinidazole is reported in the following table

Dose	Male			Female		
mg/kg	C_{max}	t_{max}	$AUC_{0-t(last)}$	C_{max}	t_{max}	$AUC_{0-t(last)}$
	ng/mL	hour	ng·hour/mL	ng/mL	hour	ng·hour/mL
50 (n=3)	31.2±12.4	0.5±0	140±147	42.4±10.6	1±0.87	237±105
200 (n=3)	54.9±10.8	1±0.87	419±61.4	84.1±36.7	1±0	454±119
800 (n=5)	100±21.2	1.1±0.55	776±182	184±75.6	1.2±0.45	895±437

At each dose, no relevant gender difference was observed in terms of both C_{max} and $AUC_{0-t(last)}$ values. The maximal plasma concentrations of Fexinidazole were promptly achieved, on average at 1 hour post dosing. $AUC_{0-t(last)}$ values of Fexinidazole increased with the dose (Figures 22 - 23).

Mean ±SD systemic exposure to the sulfone metabolite is reported in the following table

Dose	Male			Female		
mg/kg	C_{max}	t_{max}	$AUC_{0-t(last)}$	C_{max}	t_{max}	$AUC_{0-t(last)}$
	µg/mL	Hour	µg·hour/mL	µg/mL	hour	µg·hour/mL
50 (n=3)	7.17±1.74	8±0	126±28.1	10±1.6	6.67±2.31	170±37.8
200 (n=3)	17.2±1.69	13.3±9.24	338±50.8	18.1±3.42	12±10.6	358±85.7
800 (n=5)	38.6±2.83	17.6±8.76	705±94.9	33.6±10.9	11.2±7.16	614±216

At each dose, the levels of the metabolite were similar in males and females. T_{max} values of the metabolite were achieved later than the corresponding ones of the parent compound. The systemic exposure to the metabolite increased with the dose (Figures 24 - 25).
The systemic exposure to the sulfone metabolite was definitely higher than that to the parent compound.

Mean ±SD systemic exposure to the sulfoxide metabolite is reported in the following table

Dose	Male			Female		
mg/kg	C_{max}	t_{max}	$AUC_{0-t(last)}$	C_{max}	t_{max}	$AUC_{0-t(last)}$
	µg/mL	hour	µg·hour/mL	µg/mL	hour	µg·hour/mL
50 (n=3)	3.55±1.3	1±0	19.2±5.82	3.97±0.74	1.33±0.58	20±6.81
200 (n=3)	7.55±1.03	1.67±0.58	50.7±8.61	8.7±3.95	0.83±0.29	52.1±20.6
800 (n=5)	13.4±3.22	1.5±0.71	104±11.3	15.6±4.64	1.6±0.55	121±44.4

At each dose, the levels of the metabolite were similar in males and females. The maximal plasma concentrations of the sulfoxide metabolite were rapidly achieved, on average 1 - 2 hours post dosing.
The systemic exposure to the metabolite increased with the dose (Figures 26 - 27).
The systemic exposure to the metabolite was definitely higher than that to the parent compound.

<u>Repeated dosing</u>
Day 14 and Day 28 mean ±SD systemic exposure to Fexinidazole is reported in the following table

Dose	Male			Female		
mg/kg/day	C_{max}	t_{max}	$AUC_{0-t(last)}$	C_{max}	t_{max}	$AUC_{0-t(last)}$
	ng/mL	hour	ng·hour/mL	ng/mL	hour	ng·hour/mL
	Day 14					
50 (n=3)	26.5±13	0.5±0	52.1±29.4	41.9±2.2	1.67±0.58	246±117
200 (n=3)	78.1±23.5	1±0.87	452±41.1	77.7±50.8	2±0	443±217
800 (n=5)	128±56.9	1±0	929±268	152±44	1.4±0.55	1170±309
	Day 28					
50 (n=3)	20.3±7.89	1±0	124±67	36.3±15.1	1±0	87.1±38.1
200 (n=3)	57.9±12.5	1±0	395±15.8	73±12	1±0	377±83.5
800 (n=5)	86.2±38.5	1.2±0.4	736±141	101±47.1	1±0	956±378

At each dose, the levels of Fexinidazole were similar in both genders after Day 14 and Day 28 administrations. The maximal plasma concentrations of Fexinidazole were achieved, on average, 1 - 2 hours post dosing. The systemic exposure to Fexinidazole increased with the dose (Figures 22 - 23). C_{max} and $AUC_{0-t(last)}$ accumulation ratios were about 1.

Day 14 and Day 28 mean ±SD systemic exposure to the sulfone metabolite is reported in the following table

Dose	Male			Female		
mg/kg/day	C_{max}	t_{max}	$AUC_{0-t(last)}$	C_{max}	t_{max}	$AUC_{0-t(last)}$
	µg/mL	hour	µg·hour/mL	µg/mL	hour	µg·hour/mL
	Day 14					
50 (n=3)	5.57±2.19	4±0	78±35.3	9.98±1.25	6.67±2.31	173±26.9
200 (n=3)	22±5.59	5.33±2.31	387±49.5	21.5±2.42	5.33±2.31	381±61.2

800 (n=5)	34.6±7.42	5.6±2.19	640±163	38.6±4.73	8±0	667±67.8
	Day 28					
50 (n=3)	7.11±1.21	8±0	121±19.1	6.79±0.56	3.33±1.15	107±22.5
200 (n=3)	14±3.31	4±4	258±48	15.4±2.66	11.7±10.1	277±29.9
800 (n=5)	20.8±4.14	12.4±9.81	388±68.2[1]<br>601±29[2]	26±7.7	7.2±1.79	477±145[1]<br>693±351[2]
[1] AUC_{0-24}; [2] n=2						

At each dose, the levels of the metabolite were similar in both genders. T_{max} values of the metabolite were achieved later than the corresponding ones of the parent compound. On Day 28 after 800 mg/kg/day, mean ±SD apparent terminal half-lives were 6.7 ±0.7 and 7.8 ±0.7 (n=2) hours in males and females, respectively. After the three doses, systemic exposure to the metabolite increased with the dose (Figures 24 - 25). Accumulation ratios, in terms of both C_{max} and $AUC_{0-t(last)}$, were about 1.
The systemic exposure to the metabolite was remarkably higher than that to the parent compound.

Day 14 and Day 28 mean ±SD systemic exposure to the sulfoxide metabolite is reported in the following table

Dose	Male			Female		
mg/kg/day	C_{max}	t_{max}	$AUC_{0-t(last)}$	C_{max}	t_{max}	$AUC_{0-t(last)}$
	μg/mL	hour	μg·hour/mL	μg/mL	hour	μg·hour/mL
	Day 14					
50 (n=3)	2.24±0.45	1±0	8.51±2.53	3.73±0.73	1.67±0.58	18.8±2.06
200 (n=3)	8.96±2.53	2±0	56.1±9.96	9.02±3.69	2±0	57.2±24.3
800 (n=5)	12.5±2.85	1.2±0.45	113±39.9	14.8±5.1	2±1.22	144±37.1
	Day 28					
50 (n=3)	1.83±0.57	1.67±0.58	14.2±2.72	2.72±0.73	1±0	9.88±2.72
200 (n=3)	5.76±1.17	1.33±0.58	42.2±6.46	5.43±0.34	1±0	33.8±1.65
800 (n=5)	9.35±3.55	1.6±0.55	74.3±14.4[1]<br>121±33[2]	9.48±3.38	1.4±0.55	89±45.2
[1] AUC_{0-24}; [2] n=2						

At each dose, the levels of the metabolite were similar in both genders. The maximal plasma concentrations of the sulfoxide metabolite were promptly achieved, on average, 1 - 2 hours post dosing. On Day 28 after 800 mg/kg/day, mean ±SD apparent terminal half-lives were 9.4 ±5.4 (n=2) hours in males. After the three doses, the systemic exposure to the metabolite increased with the dose (Figures 26 - 27). Accumulation ratios, in terms of both C_{max} and $AUC_{0-t(last)}$, were about 1.
The systemic exposure to the metabolite was remarkably higher than that to the parent compound.

8. CONCLUSIONS

After the first and repeated administrations of the three dose levels, no relevant gender difference in the systemic exposure to Fexinidazole was observed. AUCs of Fexinidazole increased less than expected assuming dose proportionality in the dose range investigated. No accumulation of Fexinidazole was observed.
Fexinidazole was extensively metabolized to the sulfone and sulfoxide derivatives. No accumulation of both metabolites was observed.

9. CONTRIBUTORS

10. ARCHIVING

Raw data and pharmacokinetic analysis produced at the Test Facility were filed in the Archives of Accelera, Nerviano Medical Sciences S.r.l., Nerviano (Italy) for the period of time agreed with the Sponsor (at least 3 years) after which the Sponsor will be contacted for instructions regarding dispatch or disposal of the material.

A copy of the protocol, the report with original signatures, a reserve sample and all relevant original documentation of the test item were filed by the Sponsor.

11. REFERENCES

1. Fexinidazole: 28-day Oral Toxicity Study in the Dog. Nerviano Medical Sciences Study Protocol 0505-2007-P, January 23, 2008.
2. Amendment no. 1 to Fexinidazole: 28-day Oral Toxicity Study in the Dog. February 21, 2008.
3. Analytical Procedure for the Determination of Fexinidazole and its Metabolites Fexinidazole Sulphoxide (M1) and Fexinidazole Sulphone (M2) in Dog Plasma by LC-MS-MS Following Plasma Protein Precipitation. Analytical Procedure Number: NMS/FEXINIDAZOLE/02.0. NervianoMS Reference Number: 0291-2007-AP. February 27, 2008.
4. Validation of an Analytical Method for the Determination of Fexinidazole and its Metabolites M1 and M2 in Dog Plasma by LC-MS-MS. Document Number: 0291-2007-R.
5. SOP: PCD-M-BPK-001-01: “Bioanalytical Run Acceptance”. 13 December 2006.

TABLES AND FIGURES

Table 1. Mean (±SD, n) systemic exposure parameters of Fexinidazole after single (Day 1) and repeated (Days 14 and 28) oral administrations of Fexinidazole to male and female Beagle dogs.

Gender	Day	Dose mg/kg/day	C_{max} ng/mL			t_{max} hour			$AUC_{0-t(last)}$ ng·hour/mL		
			Mean	SD	n	Mean	SD	n	Mean	SD	n
M	1	50	31.2	12.4	3	0.5	0	3	140	147	3
		200	54.9	10.8	3	1	0.866	3	419	61.4	3
		800	100	21.2	5	1.1	0.548	5	776	182	5
	14	50	26.5	13.0	3	0.5	0	3	52.1	29.4	3
		200	78.1	23.5	3	1	0.866	3	452	41.1	3
		800	128	56.9	5	1	0	5	929	268	5
	28	50	20.3	7.89	3	1	0	3	124	67	3
		200	57.9	12.5	3	1	0	3	395	15.8	3
		800	86.2	38.5	5	1.2	0.447	5	736	141	5
F	1	50	42.4	10.6	3	1	0.866	3	237	105	3
		200	84.1	36.7	3	1	0	3	454	119	3
		800	184	75.6	5	1.2	0.447	5	895	437	5
	14	50	41.9	2.20	3	1.67	0.577	3	246	117	3
		200	77.7	50.8	3	2	0	3	443	217	3
		800	152	44.0	5	1.4	0.548	5	1170	309	5
	28	50	36.3	15.1	3	1	0	3	87.1	38.1	3
		200	73	12	3	1	0	3	377	83.5	3
		800	101	47.1	5	1	0	5	956	378	5
M + F	1	50	36.8	12.0	6	0.75	0.612	6	188	126	6
		200	69.5	29.0	6	1	0.548	6	437	86.5	6
		800	142	68.3	10	1.15	0.474	10	836	322	10
	14	50	34.2	11.8	6	1.08	0.736	6	149	131	6
		200	77.9	35.4	6	1.5	0.775	6	447	140	6
		800	140	49.6	10	1.2	0.422	10	1050	301	10
	28	50	28.3	13.9	6	1	0	6	106	52.8	6
		200	65.4	13.7	6	1	0	6	386	54.7	6
		800	93.5	41.3	10	1.1	0.316	10	846	293	10

Table 2. Mean (±SD, n) systemic exposure parameters of the sulfone metabolite after single (Day 1) and repeated (Days 14 and 28) oral administrations of Fexinidazole to male and female Beagle dogs.

Gender	Day	Dose mg/kg/day	C_{max} µg/mL			t_{max} hour			$AUC_{0-t(last)}$ µg·hour/mL		
			Mean	SD	n	Mean	SD	n	Mean	SD	n
M	1	50	7.17	1.74	3	8	0	3	126	28.1	3
		200	17.2	1.69	3	13.3	9.24	3	338	50.8	3
		800	38.6	2.83	5	17.6	8.76	5	705	94.9	5
	14	50	5.57	2.19	3	4	0	3	78.0	35.3	3
		200	22.0	5.59	3	5.33	2.31	3	387	49.5	3
		800	34.6	7.42	5	5.6	2.19	5	640	163	5
	28	50	7.11	1.21	3	8	0	3	121	19.1	3
		200	14	3.31	3	4	4	3	258	48	3
		800	20.8	4.14	5	12.4	9.81	5	388*	68.2	5
F	1	50	10.0	1.60	3	6.67	2.31	3	170	37.8	3
		200	18.1	3.42	3	12.0	10.6	3	358	85.7	3
		800	33.6	10.9	5	11.2	7.16	5	614	216	5
	14	50	9.98	1.25	3	6.67	2.31	3	173	26.9	3
		200	21.5	2.42	3	5.33	2.31	3	381	61.2	3
		800	38.6	4.73	5	8	0	5	667	67.8	5
		50	6.79	0.555	3	3.33	1.15	3	107	22.5	3
	28	200	15.4	2.66	3	11.7	10.1	3	277	29.9	3
		800	26	7.7	5	7.2	1.79	5	477*	145	5
M + F	1	50	8.59	2.15	6	7.33	1.63	6	148	38.3	6
		200	17.6	2.46	6	12.7	8.91	6	348	63.9	6
		800	36.1	7.94	10	14.4	8.26	10	660	164	10
	14	50	7.78	2.89	6	5.33	2.07	6	126	59.1	6
		200	21.8	3.86	6	5.33	2.07	6	384	49.9	6
		800	36.6	6.24	10	6.80	1.93	10	653	118	10
		50	6.95	0.862	6	5.67	2.66	6	114	20.1	6
	28	200	14.7	2.79	6	7.86	8.08	6	267	37.2	6
		800	23.4	6.43	10	9.8	7.19	10	432*	117	10

* AUC_{0-24}

Table 3. Mean (±SD, n) systemic exposure parameters of the sulfoxide metabolite after single (Day 1) and repeated (Days 14 and 28) oral administrations of Fexinidazole to male and female Beagle dogs.

Gender	Day	Dose mg/kg/day	C_{max} µg/mL			t_{max} hour			$AUC_{0-t(last)}$ µg·hour/mL		
			Mean	SD	n	Mean	SD	n	Mean	SD	n
M	1	50	3.55	1.3	3	1	0	3	19.2	5.82	3
		200	7.55	1.03	3	1.67	0.577	3	50.7	8.61	3
		800	13.4	3.22	5	1.50	0.707	5	104	11.3	5
	14	50	2.24	0.454	3	1	0	3	8.51	2.53	3
		200	8.96	2.53	3	2	0	3	56.1	9.96	3
		800	12.5	2.85	5	1.2	0.447	5	113	39.9	5
	28	50	1.83	0.567	3	1.67	0.577	3	14.2	2.72	3
		200	5.76	1.17	3	1.33	0.577	3	42.2	6.46	3
		800	9.35	3.55	5	1.6	0.548	5	74.3*	14.4	5
F	1	50	3.97	0.738	3	1.33	0.577	3	20	6.81	3
		200	8.70	3.95	3	0.833	0.289	3	52.1	20.6	3
		800	15.6	4.64	5	1.60	0.548	5	121	44.4	5
	14	50	3.73	0.725	3	1.67	0.577	3	18.8	2.06	3
		200	9.02	3.69	3	2.00	0.00	3	57.2	24.3	3
		800	14.8	5.10	5	2.00	1.22	5	144	37.1	5
	28	50	2.72	0.729	3	1	0	3	9.88	2.72	3
		200	5.43	0.34	3	1	0	3	33.8	1.65	3
		800	9.48	3.38	5	1.4	0.548	5	89	45.2	5
M + F	1	50	3.76	0.975	6	1.17	0.408	6	19.6	5.68	6
		200	8.12	2.66	6	1.25	0.612	6	51.4	14.1	6
		800	14.5	3.95	10	1.55	0.599	10	112	31.8	10
	14	50	2.99	0.976	6	1.33	0.516	6	13.6	5.99	6
		200	8.99	2.83	6	2	0	6	56.7	16.6	6
		800	13.6	4.09	10	1.60	0.966	10	129	39.8	10
	28	50	2.27	0.76	6	1.33	0.516	6	12.1	3.41	6
		200	5.6	0.792	6	1.17	0.408	6	38	6.26	6
		800	9.41	3.27	10	1.5	0.527	10	81.7*	32.6	10

* AUC_{0-24}

Table 4. Day 1 individual and mean (±SD, %CV) pharmacokinetic parameters of Fexinidazole after oral 50 mg/kg/day of Fexinidazole in male Beagle dogs.

Parameter	Units	ID 2514	ID 2521	ID 2529	Mean	SD	%CV
C_{max}	ng/mL	45.5	23.1	24.9	31.2	12.4	39.9
t_{max}	hour	0.5	0.5	0.5	0.5	0	0
AUC Interval	hour	(0-24)	(0-4)	(0-4)			
$AUC_{0-t(last)}$	ng·hour/mL	309	64.1	45.7	140	147	105.3
C_{max}/Dose	ng/mL	0.910	0.462	0.498	0.623	0.249	39.9
$AUC_{0-t(last)}$/Dose	ng·hour/mL	6.18	1.28	0.914	2.79	2.94	105.3

Table 5. Day 14 individual and mean (±SD, %CV) pharmacokinetic parameters of Fexinidazole after oral 50 mg/kg/day of Fexinidazole in male Beagle dogs.

Parameter	Units	ID 2514	ID 2521	ID 2529	Mean	SD	%CV
C_{max}	ng/mL	41.4	21.1	17.1	26.5	13	49.1
t_{max}	hour	0.5	0.5	0.5	0.5	0	0
AUC Interval	hour	(0-4)	(0-4)	(0-2)			
$AUC_{0-t(last)}$	ng·hour/mL	83.2	48.3	24.8	52.1	29.4	56.4
C_{max}/Dose	ng/mL	0.828	0.422	0.342	0.531	0.261	49.1
$AUC_{0-t(last)}$/Dose	ng·hour/mL	1.66	0.967	0.496	1.04	0.586	56.2
$R_{A,Cmax}$		0.91	0.913	0.687	0.84	0.13	15.5
$R_{A,AUC0-t(last)}$		0.269	0.754	0.543	0.522	0.243	46.5

Table 6. Day 28 individual and mean (±SD, %CV) pharmacokinetic parameters of Fexinidazole after oral 50 mg/kg/day of Fexinidazole in male Beagle dogs.

Parameter	Units	ID 2514	ID 2521	ID 2529	Mean	SD	%CV
C_{max}	ng/mL	29.3	17	14.6	20.3	7.89	38.8
t_{max}	hour	1	1	1	1	0	0
AUC Interval	hour	(0-8)	(0-24) (1)	(0-8)			
$AUC_{0-t(last)}$	ng·hour/mL	104	199	69.6	124	67	54
C_{max}/Dose	ng/mL	0.586	0.34	0.292	0.406	0.158	38.8
$AUC_{0-t(last)}$/Dose	ng·hour/mL	2.08	3.97	1.39	2.48	1.34	53.9
$R_{A,Cmax}$		0.644	0.736	0.586	0.655	0.0754	0.644
$R_{A,AUC0-t(last)}$		0.337	3.1	1.52	1.65	1.39	0.337
(1) Nominal time							

Table 7. Day 1 individual and mean (±SD, %CV) pharmacokinetic parameters of Fexinidazole after oral 50 mg/kg/day of Fexinidazole in female Beagle dogs.

Parameter	Units	ID 2562	ID 2563	ID 2576	Mean	SD	%CV
C_{max}	ng/mL	38.8	54.3	34.1	42.4	10.6	24.9
t_{max}	hour	2	0.5	0.5	1.00	0.87	86.6
AUC Interval	hour	(0-24)	(0-4)	(0-24)			
$AUC_{0-t(last)}$	ng·hour/mL	317	118	275	237	105	44.3
C_{max}/Dose	ng/mL	0.776	1.09	0.682	0.849	0.214	25.2
$AUC_{0-t(last)}$/Dose	ng·hour/mL	6.35	2.37	5.50	4.74	2.10	44.2

Table 8. Day 14 individual and mean (±SD, %CV) pharmacokinetic parameters of Fexinidazole after oral 50 mg/kg/day of Fexinidazole in female Beagle dogs.

Parameter	Units	ID 2562	ID 2563	ID 2576	Mean	SD	%CV
C_{max}	ng/mL	44.4	40.8	40.4	41.9	2.2	5.3
t_{max}	hour	1	2	2	1.67	0.577	34.6
AUC Interval	hour	(0-24)	(0-4)	(0-24)			
$AUC_{0-t(last)}$	ng·hour/mL	275	118	346	246	117	47.4
C_{max}/Dose	ng/mL	0.888	0.816	0.808	0.837	0.0441	5.3
$AUC_{0-t(last)}$/Dose	ng·hour/mL	5.51	2.37	6.93	4.94	2.33	47.3
R_A,C_{max}		1.14	0.751	1.18	1.03	0.239	23.3
R_A,$AUC_{0-t(last)}$		0.868	1	1.26	1.04	0.199	19.1

Table 9. Day 28 individual and mean (±SD, %CV) pharmacokinetic parameters of Fexinidazole after oral 50 mg/kg/day of Fexinidazole in female Beagle dogs.

Parameter	Units	ID 2562	ID 2563	ID 2576	Mean	SD	%CV
C_{max}	ng/mL	23.5	53	32.4	36.3	15.1	41.7
t_{max}	hour	1	1	1	1	0	0
AUC Interval	hour	(0-4)	(0-4)	(0-4)			
$AUC_{0-t(last)}$	ng·hour/mL	62.9	131	67.5	87.1	38.1	43.7
C_{max}/Dose	ng/mL	0.470	1.06	0.648	0.726	0.303	41.7
$AUC_{0-t(last)}$/Dose	ng·hour/mL	1.26	2.63	1.35	1.75	0.766	43.9
R_A,C_{max}		0.606	0.976	0.95	0.844	0.207	24.5
R_A,$AUC_{0-t(last)}$		0.198	1.11	0.245	0.518	0.513	99.1

Table 10. Day 1 individual and mean (±SD, %CV) pharmacokinetic parameters of Fexinidazole after oral 200 mg/kg/day of Fexinidazole in male Beagle dogs.

Parameter	Units	ID 2515	ID 2523	ID 2526	Mean	SD	%CV
C_{max}	ng/mL	46.4	67	51.3	54.9	10.8	19.6
t_{max}	hour	0.5	2	0.5	1	0.866	86.6
AUC Interval	hour	(0-24)	(0-24)	(0-24)			
$AUC_{0-t(last)}$	ng·hour/mL	467	350	441	419	61.4	14.7
C_{max}/Dose	ng/mL	0.232	0.335	0.257	0.275	0.0537	19.6
$AUC_{0-t(last)}$/Dose	ng·hour/mL	2.33	1.75	2.21	2.10	0.306	14.6

Table 11. Day 14 individual and mean (±SD, %CV) pharmacokinetic parameters of Fexinidazole after oral 200 mg/kg/day of Fexinidazole in male Beagle dogs.

Parameter	Units	ID 2515	ID 2523	ID 2526	Mean	SD	%CV
C_{max}	ng/mL	52.6	82.9	98.8	78.1	23.5	30.1
t_{max}	hour	0.5	2	0.5	1	0.866	86.6
AUC Interval	hour	(0-24)	(0-24)	(0-24)			
$AUC_{0-t(last)}$	ng·hour/mL	491	456	409	452	41.1	9.1
C_{max}/Dose	ng/mL	0.263	0.415	0.494	0.391	0.117	30.1
$AUC_{0-t(last)}$/Dose	ng·hour/mL	2.45	2.28	2.05	2.26	0.201	8.9
R_{A},C_{max}		1.13	1.24	1.93	1.43	0.431	30.1
R_{A},$AUC_{0-t(last)}$		1.05	1.3	0.927	1.09	0.191	17.5

Table 12. Day 28 individual and mean (±SD, %CV) pharmacokinetic parameters of Fexinidazole after oral 200 mg/kg/day of Fexinidazole in male Beagle dogs.

Parameter	Units	ID 2515	ID 2523	ID 2526	Mean	SD	%CV
C_{max}	ng/mL	71.2	56.1	46.3	57.9	12.5	21.7
t_{max}	hour	1	1	1	1	0	0
AUC Interval (1)	hour	(0-24)	(0-24)	(0-24)			
$AUC_{0-t(last)}$	ng·hour/mL	399	378	409	395	15.8	4
C_{max}/Dose	ng/mL	0.356	0.281	0.232	0.290	0.0625	21.6
$AUC_{0-t(last)}$/Dose	ng·hour/mL	1.99	1.89	2.04	1.97	0.0764	3.9
R_{A},C_{max}		1.53	0.837	0.903	1.09	0.385	35.3
R_{A},$AUC_{0-t(last)}$		0.854	1.08	0.927	0.954	0.115	12.1
(1) Nominal time							

Table 13. Day 1 individual and mean (±SD, %CV) pharmacokinetic parameters of Fexinidazole after oral 200 mg/kg/day of Fexinidazole in female Beagle Dogs.

Parameter	Units	ID 2561	ID 2567	ID 2570	Mean	SD	%CV
C_{max}	ng/mL	57.4	126	69	84.1	36.7	43.6
t_{max}	hour	1	1	1	1	0	0
AUC Interval	hour	(0-24)	(0-24)	(0-24)			
$AUC_{0-t(last)}$	ng·hour/mL	357	586	418	454	119	26.1
C_{max}/Dose	ng/mL	0.287	0.630	0.345	0.421	0.184	43.6
$AUC_{0-t(last)}$/Dose	ng·hour/mL	1.79	2.93	2.09	2.27	0.591	26

Table 14. Day 14 individual and mean (±SD, %CV) pharmacokinetic parameters of Fexinidazole after oral 200 mg/kg/day of Fexinidazole in female Beagle dogs.

Parameter	Units	ID 2561	ID 2567	ID 2570	Mean	SD	%CV
C_{max}	ng/mL	42.9	136	54.2	77.7	50.8	65.4
t_{max}	hour	2	2	2	2	0	0
AUC Interval	hour	(0-8)	(0-8)	(0-24)			
$AUC_{0-t(last)}$	ng·hour/mL	193	588	547	443	217	49.1
C_{max}/Dose	ng/mL	0.215	0.680	0.271	0.389	0.254	65.3
$AUC_{0-t(last)}$/Dose	ng·hour/mL	0.965	2.94	2.73	2.21	1.08	49
R_{A},C_{max}		0.747	1.08	0.786	0.871	0.182	20.9
R_{A},$AUC_{0-t(last)}$		0.541	1	1.31	0.951	0.387	40.7

Table 15. Day 28 individual and mean (±SD, %CV) pharmacokinetic parameters of Fexinidazole after oral 200 mg/kg/day of Fexinidazole in female Beagle dogs.

Parameter	Units	ID 2561	ID 2567	ID 2570	Mean	SD	%CV
C_{max}	ng/mL	84	74.7	60.2	73	12	16.4
t_{max}	hour	1	1	1	1	0	0
AUC Interval	hour	(0-8)	(0-24) [(1)]	(0-24) [(1)]			
$AUC_{0-t(last)}$	ng·hour/mL	286	450	395	377	83.5	22.1
C_{max}/Dose	ng/mL	0.420	0.374	0.301	0.365	0.060	16.4
$AUC_{0-t(last)}$/Dose	ng·hour/mL	1.43	2.25	1.98	1.89	0.418	22.1
R_{A},C_{max}		1.46	0.593	0.872	0.976	0.444	45.5
R_{A},$AUC_{0-t(last)}$		0.801	0.768	0.945	0.838	0.094	11.2
[(1)] Nominal time							

Table 16. Day 1 individual and mean (±SD, %CV) pharmacokinetic parameters of Fexinidazole after oral 800 mg/kg/day of Fexinidazole in male Beagle dogs.

Parameter	Units	Dog ID					Mean	SD	%CV
		2517	2519	2525	2528	2530			
C_{max}	ng/mL	132	108	80.8	98.8	81.5	100	21.2	21.2
t_{max}	hour	0.5	1	2	1	1	1.1	0.548	49.8
AUC Interval	hour	(0-24)	(0-24)	(0-24)	(0-24)	(0-24)			
$AUC_{0-t(last)}$	ng·hour/mL	630	623	811	1070	748	776	182	23.5
C_{max}/Dose	ng/mL	0.165	0.135	0.101	0.124	0.102	0.125	0.0265	21.1
$AUC_{0-t(last)}$/Dose	ng·hour/mL	0.787	0.779	1.01	1.34	0.935	0.97	0.229	23.6

Table 17. Day 14 individual and mean (±SD, %CV) pharmacokinetic parameters of Fexinidazole after oral 800 mg/kg/day of Fexinidazole in male Beagle dogs.

Parameter	Units	Dog ID					Mean	SD	%CV
		2517	2519	2525	2528	2530			
C_{max}	ng/mL	130	220	75.1	127	86.9	128	56.9	44.5
t_{max}	hour	1	1	1	1	1	1	0	0
AUC Interval	hour	(0-24)	(0-24)	(0-24)	(0-24)	(0-24)			
$AUC_{0-t(last)}$	ng·hour/mL	1120	663	611	1090	1160	929	268	28.9
C_{max}/Dose	ng/mL	0.163	0.275	0.0939	0.159	0.109	0.16	0.0711	44.4
$AUC_{0-t(last)}$/Dose	ng·hour/mL	1.4	0.829	0.763	1.36	1.44	1.16	0.333	28.7
R_A,C_{max}		0.985	2.04	0.929	1.29	1.07	1.26	0.455	36.1
R_A,$AUC_{0-t(last)}$		1.78	1.06	0.753	1.02	1.55	1.23	0.419	34

Table 18. Day 28 individual and mean (±SD, %CV) pharmacokinetic parameters of Fexinidazole after oral 800 mg/kg/day of Fexinidazole in male Beagle Dogs.

Parameter	Units	Dog ID					Mean	SD	%CV
		2517	2519	2525	2528	2530			
C_{max}	ng/mL	141	54.1	107	48.4	80.4	86.2	38.5	44.7
t_{max}	hour	1	1	1	2	1	1.2	0.447	37.3
AUC Interval (1)	hour	(0-24)	(0-24)	(0-24)	(0-24)	(0-24)			
$AUC_{0-t(last)}$	ng·hour/mL	938	824	673	605	638	736	141	19.1
C_{max}/Dose	ng/mL	0.176	0.0676	0.134	0.0605	0.101	0.108	0.0481	44.6
$AUC_{0-t(last)}$/Dose	ng·hour/mL	1.17	1.03	0.842	0.757	0.797	0.919	0.175	19
R_A,C_{max}		1.07	0.501	1.32	0.49	0.987	0.874	0.367	42
R_A,$AUC_{0-t(last)}$		1.49	1.32	0.83	0.565	0.853	1.01	0.381	37.7
(1) Nominal time									

Table 19. Day 1 individual and mean (±SD, %CV) pharmacokinetic parameters of Fexinidazole after oral 800 mg/kg/day of Fexinidazole in female Beagle dogs.

Parameter	Units	Dog ID					Mean	SD	%CV
		2564	2565	2569	2571	2573			
C_{max}	ng/mL	293	233	128	135	129	184	75.6	41.2
t_{max}	hour	1	2	1	1	1	1.2	0.447	37.3
AUC Interval	hour	(0-8)	(0-24)	(0-24)	(0-24)	(0-24)			
$AUC_{0-t(last)}$	ng·hour/mL	628	1580	1050	736	479	895	437	48.8
C_{max}/Dose	ng/mL	0.366	0.291	0.160	0.169	0.161	0.229	0.0943	41.1
$AUC_{0-t(last)}$/Dose	ng·hour/mL	0.785	1.97	1.31	0.920	0.599	1.12	0.544	48.7

Table 20. Day 14 individual and mean (±SD, %CV) pharmacokinetic parameters of Fexinidazole after oral 800 mg/kg/day of Fexinidazole in female Beagle dogs.

Parameter	Units	Dog ID					Mean	SD	%CV
		2564	2565	2569	2571	2573			
C_{max}	ng/mL	194	171	182	89.2	124	152	44	29
t_{max}	hour	1	2	2	1	1	1.4	0.548	39.1
AUC Interval	hour	(0-24)	(0-24)	(0-24)	(0-24)	(0-24)			
$AUC_{0-t(last)}$	ng·hour/mL	1270	1660	1020	869	1030	1170	309	26.4
C_{max}/Dose	ng/mL	0.243	0.214	0.228	0.112	0.155	0.19	0.0551	28.9
$AUC_{0-t(last)}$/Dose	ng·hour/mL	1.59	2.07	1.27	1.09	1.29	1.46	0.384	26.3
$R_{A,Cmax}$		0.662	0.734	1.42	0.661	0.961	0.888	0.323	36.4
$R_{A,AUC0-t(last)}$		2.02	1.05	0.971	1.18	2.15	1.48	0.565	38.3

Table 21. Day 28 individual and mean (±SD, %CV) pharmacokinetic parameters of Fexinidazole after oral 800 mg/kg/day of Fexinidazole in female Beagle dogs.

Parameter	Units	Dog ID					Mean	SD	%CV
		2564	2565	2569	2571	2573			
C_{max}	ng/mL	94.7	179	102	69.5	59	101	47.1	46.7
t_{max}	hour	1	1	1	1	1	1	0	0
AUC Interval (1)	hour	(0-24)	(0-24)	(0-24)	(0-24)	(0-24)			
$AUC_{0-t(last)}$	ng·hour/mL	684	1540	1140	696	720	956	378	39.6
C_{max}/Dose	ng/mL	0.118	0.224	0.128	0.0869	0.0738	0.126	0.059	46.8
$AUC_{0-t(last)}$/Dose	ng·hour/mL	0.854	1.93	1.42	0.869	0.900	1.19	0.474	39.7
$R_{A,Cmax}$		0.323	0.768	0.797	0.515	0.457	0.572	0.205	35.8
$R_{A,AUC0-t(last)}$		1.09	0.975	1.09	0.946	1.5	1.12	0.224	20

(1) Nominal time

Table 22. Day 1 individual and mean (±SD, %CV) pharmacokinetic parameters of the sulfone metabolite after oral 50 mg/kg/day of Fexinidazole in male Beagle dogs.

Parameter	Units	ID 2514	ID 2521	ID 2529	Mean	SD	%CV
C_{max}	μg/mL	9.17	6.33	6.02	7.17	1.74	24.2
t_{max}	hour	8	8	8	8	0	0
AUC Interval	hour	(0-24)	(0-24)	(0-24)			
$AUC_{0-t(last)}$	μg·hour/mL	158	107	112	126	28.1	22.4
C_{max}/Dose	μg/mL	0.183	0.127	0.12	0.143	0.035	24.1
$AUC_{0-t(last)}$/Dose	μg·hour/mL	3.16	2.13	2.25	2.51	0.563	22.4

Table 23. Day 14 individual and mean (±SD, %CV) pharmacokinetic parameters of the sulfone metabolite after oral 50 mg/kg/day of Fexinidazole in male Beagle dogs.

Parameter	Units	ID 2514	ID 2521	ID 2529	Mean	SD	%CV
C_{max}	μg/mL	8.09	4.44	4.18	5.57	2.19	39.3
t_{max}	hour	4	4	4	4	0	0
AUC Interval	hour	(0-24)	(0-24)	(0-24)			
$AUC_{0-t(last)}$	μg·hour/mL	117	68.9	48.1	78	35.3	45.3
C_{max}/Dose	μg/mL	0.162	0.0888	0.0836	0.111	0.0438	39.3
$AUC_{0-t(last)}$/Dose	μg·hour/mL	2.35	1.38	0.961	1.56	0.712	45.6
R_A,C_{max}		0.882	0.701	0.694	0.759	0.106	14
R_A,$AUC_{0-t(last)}$		0.741	0.644	0.429	0.605	0.159	26.3

Table 24. Day 28 individual and mean (±SD, %CV) pharmacokinetic parameters of the sulfone metabolite after oral 50 mg/kg/day of Fexinidazole in male Beagle dogs.

Parameter	Units	ID 2514	ID 2521	ID 2529	Mean	SD	%CV
C_{max}	μg/mL	8.43	6.87	6.04	7.11	1.21	17.1
t_{max}	hour	8	8	8	8	0	0
AUC Interval (1)	hour	(0-24)	(0-24)	(0-24)			
$AUC_{0-t(last)}$	μg·hour/mL	139	123	101	121	19.1	15.8
C_{max}/Dose	μg/mL	0.169	0.137	0.121	0.142	0.0244	17.2
$AUC_{0-t(last)}$/Dose	μg·hour/mL	2.77	2.45	2.02	2.41	0.376	15.6
R_A,C_{max}		0.919	1.09	1	1	0.083	8.3
R_A,$AUC_{0-t(last)}$		0.88	1.15	0.902	0.977	0.15	15.3
(1) Nominal time							

Table 25. Day 1 individual and mean (±SD, %CV) pharmacokinetic parameters of the sulfone metabolite after oral 50 mg/kg/day of Fexinidazole in female Beagle dogs.

Parameter	Units	ID 2562	ID 2563	ID 2576	Mean	SD	%CV
C_{max}	µg/mL	11.5	8.32	10.2	10	1.6	16
t_{max}	hour	8	4	8	6.67	2.31	34.6
AUC Interval	hour	(0-24)	(0-24)	(0-24)			
$AUC_{0-t(last)}$	µg·hour/mL	199	127	183	170	37.8	22.3
C_{max}/Dose	µg/mL	0.23	0.166	0.204	0.2	0.0322	16.1
$AUC_{0-t(last)}$/Dose	µg·hour/mL	3.97	2.54	3.66	3.39	0.752	22.2

Table 26. Day 14 individual and mean (±SD, %CV) pharmacokinetic parameters of the sulfone metabolite after oral 50 mg/kg/day of Fexinidazole in female Beagle dogs.

Parameter	Units	ID 2562	ID 2563	ID 2576	Mean	SD	%CV
C_{max}	µg/mL	10.6	8.54	10.8	9.98	1.25	12.5
t_{max}	hour	8	8	4	6.67	2.31	34.6
AUC Interval	hour	(0-24)	(0-24)	(0-24)			
$AUC_{0-t(last)}$	µg·hour/mL	204	157	158	173	26.9	15.5
C_{max}/Dose	µg/mL	0.212	0.171	0.216	0.2	0.0249	12.5
$AUC_{0-t(last)}$/Dose	µg·hour/mL	4.08	3.13	3.16	3.46	0.54	15.6
R_A,C_{max}		0.922	1.03	1.06	1	0.0717	7.1
R_A,$AUC_{0-t(last)}$		1.03	1.24	0.863	1.04	0.187	17.9

Table 27. Day 28 individual and mean (±SD, %CV) pharmacokinetic parameters of the sulfone metabolite after oral 50 mg/kg/day of Fexinidazole in female Beagle dogs.

Parameter	Units	ID 2562	ID 2563	ID 2576	Mean	SD	%CV
C_{max}	µg/mL	6.5	7.43	6.44	6.79	0.56	8.2
t_{max}	hour	4	4	2	3.33	1.15	34.6
AUC Interval [(1)]	hour	(0-24)	(0-24)	(0-24)			
$AUC_{0-t(last)}$	µg·hour/mL	83.3	128	110	107	22.5	21
C_{max}/Dose	µg/mL	0.13	0.149	0.129	0.136	0.0113	8.3
$AUC_{0-t(last)}$/Dose	µg·hour/mL	1.67	2.56	2.2	2.14	0.448	20.9
R_A,C_{max}		0.565	0.893	0.631	0.697	0.173	24.9
R_A,$AUC_{0-t(last)}$		0.419	1.01	0.601	0.676	0.302	44.6
[(1)] Nominal time							

Table 28. Day 1 individual and mean (±SD, %CV) pharmacokinetic parameters of the sulfone metabolite after oral 200 mg/kg/day of Fexinidazole in male Beagle dogs.

Parameter	Units	ID 2515	ID 2523	ID 2526	Mean	SD	%CV
C_{max}	µg/mL	16	16.4	19.1	17.2	1.69	9.8
t_{max}	hour	24	8	8	13.3	9.24	69.3
AUC Interval	hour	(0-24)	(0-24)	(0-24)			
$AUC_{0-t(last)}$	µg·hour/mL	295	325	394	338	50.8	15
C_{max}/Dose	µg/mL	0.08	0.082	0.0955	0.0858	0.0084	9.8
$AUC_{0-t(last)}$/Dose	µg·hour/mL	1.47	1.62	1.97	1.69	0.257	15.2

Table 29. Day 14 individual and mean (±SD, %CV) pharmacokinetic parameters of the sulfone metabolite after oral 200 mg/kg/day of Fexinidazole in male Beagle dogs.

Parameter	Units	ID 2515	ID 2523	ID 2526	Mean	SD	%CV
C_{max}	µg/mL	15.6	24.2	26.1	22	5.59	25.5
t_{max}	hour	8	4	4	5.33	2.31	43.3
AUC Interval	hour	(0-24)	(0-24)	(0-24)			
$AUC_{0-t(last)}$	µg·hour/mL	336	389	435	387	49.5	12.8
C_{max}/Dose	µg/mL	0.078	0.121	0.131	0.11	0.0282	25.6
$AUC_{0-t(last)}$/Dose	µg·hour/mL	1.68	1.95	2.17	1.93	0.245	12.7
R_{A},C_{max}		0.975	1.48	1.37	1.27	0.263	20.7
R_{A},$AUC_{0-t(last)}$		1.14	1.2	1.1	1.15	0.0469	4.1

Table 30. Day 28 individual and mean (±SD, %CV) pharmacokinetic parameters of the sulfone metabolite after oral 200 mg/kg/day of Fexinidazole in male Beagle dogs.

Parameter	Units	ID 2515	ID 2523	ID 2526	Mean	SD	%CV
C_{max}	µg/mL	13.3	11.1	17.6	14	3.31	23.6
t_{max}	hour	8	4	0	4	4	100
AUC Interval (1)	hour	(0-24)	(0-24)	(0-24)			
$AUC_{0-t(last)}$	µg·hour/mL	256	211	307	258	48	18.6
C_{max}/Dose	µg/mL	0.0665	0.0555	0.088	0.07	0.0165	23.6
$AUC_{0-t(last)}$/Dose	µg·hour/mL	1.28	1.06	1.53	1.29	0.235	18.2
R_{A},C_{max}		0.831	0.677	0.921	0.81	0.124	15.3
R_{A},$AUC_{0-t(last)}$		0.868	0.649	0.779	0.765	0.11	14.4
(1) Nominal time							

Table 31. Day 1 individual and mean (±SD, %CV) pharmacokinetic parameters of the sulfone metabolite after oral 200 mg/kg/day of Fexinidazole in female Beagle dogs.

Parameter	Units	ID 2561	ID 2567	ID 2570	Mean	SD	%CV
C_{max}	µg/mL	15.8	22	16.4	18.1	3.42	18.9
t_{max}	hour	24	8	4	12	10.6	88.2
AUC Interval	hour	(0-24)	(0-24)	(0-24)			
$AUC_{0-t(last)}$	µg·hour/mL	318	456	299	358	85.7	24
C_{max}/Dose	µg /mL	0.079	0.11	0.082	0.0903	0.0171	18.9
$AUC_{0-t(last)}$/Dose	µg·hour/mL	1.59	2.28	1.5	1.79	0.427	23.8

Table 32. Day 14 individual and mean (±SD, %CV) pharmacokinetic parameters of the sulfone metabolite after oral 200 mg/kg/day of Fexinidazole in female Beagle dogs.

Parameter	Units	ID 2561	ID 2567	ID 2570	Mean	SD	%CV
C_{max}	µg/mL	19.3	24.1	21.2	21.5	2.42	11.2
t_{max}	hour	4	8	4	5.33	2.31	43.3
AUC Interval	hour	(0-24)	(0-24)	(0-24)			
$AUC_{0-t(last)}$	µg·hour/mL	312	428	404	381	61.2	16.1
C_{max}/Dose	µg/mL	0.0965	0.121	0.106	0.108	0.012	11.5
$AUC_{0-t(last)}$/Dose	µg·hour/mL	1.56	2.14	2.02	1.91	0.306	16.1
R_{A},C_{max}		1.22	1.1	1.29	1.2	0.1	8.3
$R_{A},AUC_{0-t(last)}$		0.981	0.939	1.35	1.09	0.227	20.8

Table 33. Day 28 individual and mean (±SD, %CV) pharmacokinetic parameters of the sulfone metabolite after oral 200 mg/kg/day of Fexinidazole in female Beagle dogs.

Parameter	Units	ID 2561	ID 2567	ID 2570	Mean	SD	%CV
C_{max}	µg/mL	17.9	12.6	15.6	15.4	2.66	17.3
t_{max}	hour	8	4	23.18	11.7	10.1	86.3
AUC Interval (1)	hour	(0-24)	(0-24)	(0-24)			
$AUC_{0-t(last)}$	µg·hour/mL	309	250	271	277	29.9	10.8
C_{max}/Dose	µg/mL	0.0895	0.063	0.078	0.0768	0.0133	17.3
$AUC_{0-t(last)}$/Dose	µg·hour/mL	1.55	1.25	1.36	1.39	0.152	10.9
R_{A},C_{max}		1.13	0.573	0.951	0.886	0.286	32.3
$R_{A},AUC_{0-t(last)}$		0.972	0.548	0.906	0.809	0.228	28.2
(1) Nominal time							

Table 34. Day 1 individual and mean (±SD, %CV) pharmacokinetic parameters of the sulfone metabolite after oral 800 mg/kg/day of Fexinidazole in male Beagle dogs.

Parameter	Units	Dog ID					Mean	SD	%CV
		2517	2519	2525	2528	2530			
C_{max}	µg/mL	39.8	37.2	43	36.4	36.5	38.6	2.83	7.3
t_{max}	hour	24	8	24	24	8	17.6	8.76	49.8
AUC Interval	hour	(0-24)	(0-24)	(0-24)	(0-24)	(0-24)			
$AUC_{0-t(last)}$	µg·hour/mL	769	544	778	731	703	705	94.9	13.5
C_{max}/Dose	µg/mL	0.0498	0.0465	0.0538	0.0455	0.0456	0.048	0.0036	7.4
$AUC_{0-t(last)}$/Dose	µg·hour/mL	0.962	0.68	0.972	0.914	0.878	0.881	0.119	13.5

Table 35. Day 14 individual and mean (±SD, %CV) pharmacokinetic parameters of the sulfone metabolite after oral 800 mg/kg/day of Fexinidazole in male Beagle dogs.

Parameter	Units	Dog ID					Mean	SD	%CV
		2517	2519	2525	2528	2530			
C_{max}	µg/mL	33.8	25.3	29.8	42.5	41.5	34.6	7.42	21.5
t_{max}	hour	4	4	4	8	8	5.6	2.19	39.1
AUC Interval	hour	(0-24)	(0-24)	(0-24)	(0-24)	(0-24)			
$AUC_{0-t(last)}$	µg·hour/mL	675	431	514	807	771	640	163	25.4
C_{max}/Dose	µg/mL	0.0423	0.0316	0.0373	0.0531	0.0519	0.043	0.0093	21.4
$AUC_{0-t(last)}$/Dose	µg·hour/mL	0.844	0.539	0.643	1.01	0.964	0.8	0.204	25.4
R_A,C_{max}		0.849	0.68	0.693	1.17	1.14	0.905	0.235	26
R_A,$AUC_{0-t(last)}$		0.878	0.792	0.661	1.1	1.1	0.906	0.193	21.3

Table 36. Day 28 individual and mean (±SD, %CV) pharmacokinetic parameters of the sulfone metabolite after oral 800 mg/kg/day of Fexinidazole in male Beagle dogs.

Parameter	Units	Dog ID					Mean	SD	%CV
		2517	2519	2525	2528	2530			
C_{max}	µg/mL	21.4	16	20	27.3	19.3	20.8	4.14	19.9
t_{max}	hour	23	23	4	8	4	12.4	9.81	79.1
AUC Interval (1)	hour	(0-24)	(0-24)	(0-24)	(0-72)	(0-72)			
AUC_{0-24}	µg·hour/mL	448	282	405	441	362	388	68.2	17.6
$AUC_{0-t(last)}$	µg·hour/mL				580	621	601	29	4.8
C_{max}/Dose	µg/mL	0.0268	0.02	0.025	0.0341	0.0241	0.026	0.0052	19.9
AUC_{0-24}/Dose	µg·hour/mL	0.561	0.352	0.506	0.551	0.453	0.485	0.0857	17.7
$AUC_{0-t(last)}$/Dose	µg·hour/mL				0.725	0.776	0.751	0.0361	4.8
$t_{1/2,z}$	hour				6.2	7.16	6.68	0.679	10.2
R_A,C_{max}		0.538	0.43	0.465	0.75	0.529	0.542	0.124	22.9
R_A,AUC_{0-24}		0.583	0.518	0.521	0.603	0.515	0.548	0.0418	7.6

(1) Nominal time

Table 37. Day 1 individual and mean (±SD, %CV) pharmacokinetic parameters of the sulfone metabolite after oral 800 mg/kg/day of Fexinidazole in female Beagle dogs.

Parameter	Units	Dog ID					Mean	SD	%CV
		2564	2565	2569	2571	2573			
C_{max}	µg/mL	35	44.9	43.1	23.8	21.1	33.6	10.9	32.4
t_{max}	hour	8	8	8	24	8	11.2	7.16	63.9
AUC Interval	hour	(0-24)	(0-24)	(0-24)	(0-24)	(0-24)			
$AUC_{0-t(last)}$	µg·hour/mL	592	848	811	481	340	614	216	35.2
C_{max}/Dose	µg/mL	0.0438	0.0561	0.0539	0.0298	0.0264	0.042	0.0136	32.3
$AUC_{0-t(last)}$/Dose	µg·hour/mL	0.74	1.06	1.01	0.601	0.425	0.767	0.269	35.1

Table 38. Day 14 individual and mean (±SD, %CV) pharmacokinetic parameters of the sulfone metabolite after oral 800 mg/kg/day of Fexinidazole in female Beagle dogs.

Parameter	Units	ID					Mean	SD	%CV
		2564	2565	2569	2571	2573			
C_{max}	µg/mL	37.3	47	36.1	37.2	35.6	38.6	4.73	12.2
t_{max}	hour	8	8	8	8	8	8	0	0
AUC Interval	hour	(0-24)	(0-24)	(0-24	(0-24)	(0-24)			
$AUC_{0-t(last)}$	µg·hour/mL	696	766	641	586	645	667	67.8	10.2
C_{max}/Dose	µg/mL	0.0466	0.0588	0.0451	0.0465	0.0445	0.048	0.0059	12.3
$AUC_{0-t(last)}$/Dose	µg·hour/mL	0.87	0.957	0.802	0.733	0.806	0.834	0.0843	10.1
R_A,C_{max}		1.07	1.05	0.838	1.56	1.69	1.24	0.365	29.5
R_A,$AUC_{0-t(last)}$		1.18	0.903	0.79	1.22	1.9	1.2	0.431	36

Table 39. Day 28 individual and mean (±SD, %CV) pharmacokinetic parameters of the sulfone metabolite after oral 800 mg/kg/day of Fexinidazole in female Beagle dogs.

Parameter	Units	Dog ID					Mean	SD	%CV
		2564	2565	2569	2571	2573			
C_{max}	µg/mL	18.2	36.2	28.6	28.5	18.3	26	7.7	29.7
t_{max}	hour	4	8	8	8	8	7.2	1.79	24.8
AUC Interval (1)	hour	(0-24)	(0-24)	(0-24)	(0-72)	(0-72)			
AUC_{0-24}	µg·hour/mL	353	691	516	493	331	477	145	30.4
$AUC_{0-t(last)}$	µg·hour/mL				941	445	693	351	50.6
C_{max}/Dose	µg/mL	0.0228	0.0453	0.0358	0.0356	0.0229	0.033	0.0096	29.6
AUC_{0-24}/Dose	µg·hour/mL	0.442	0.863	0.646	0.616	0.414	0.596	0.181	30.4
$AUC_{0-t(last)}$/Dose	µg·hour/mL				1.18	0.556	0.868	0.441	50.8
$t_{1/2,z}$	hour				8.24	7.27	7.76	0.686	8.8
R_A,C_{max}		0.52	0.806	0.664	1.2	0.867	0.811	0.254	31.4
R_A,AUC_{0-24}		0.596	0.815	0.636	1.02	0.974	0.809	0.193	23.8

(1) Nominal time

Table 40. Day 1 individual and mean (±SD, %CV) pharmacokinetic parameters of the sulfoxide metabolite after oral 50 mg/kg/day of Fexinidazole in male Beagle dogs.

Parameter	Units	ID 2514	ID 2521	ID 2529	Mean	SD	%CV
C_{max}	µg/mL	5.04	2.65	2.95	3.55	1.3	36.7
t_{max}	hour	1	1	1	1	0	0
AUC Interval	hour	(0-24)	(0-24)	(0-24)			
$AUC_{0-t(last)}$	µg·hour/mL	25.7	17.6	14.4	19.2	5.82	30.3
C_{max}/Dose	µg/mL	0.101	0.053	0.059	0.071	0.0262	36.8
$AUC_{0-t(last)}$/Dose	µg·hour/mL	0.514	0.352	0.287	0.384	0.117	30.4

Table 41. Day 14 individual and mean (±SD, %CV) pharmacokinetic parameters of the sulfoxide metabolite after oral 50 mg/kg/day of Fexinidazole in male Beagle dogs.

Parameter	Units	ID 2514	ID 2521	ID 2529	Mean	SD	%CV
C_{max}	µg/mL	2.76	2.06	1.91	2.24	0.454	20.2
t_{max}	hour	1	1	1	1	0	0
AUC Interval	hour	(0-8)	(0-24)	(0-8)			
$AUC_{0-t(last)}$	µg·hour/mL	10.8	8.93	5.79	8.51	2.53	29.8
C_{max}/Dose	µg/mL	0.0552	0.0412	0.0382	0.0449	0.00907	20.2
$AUC_{0-t(last)}$/Dose	µg·hour/mL	0.217	0.179	0.116	0.171	0.051	29.9
R_{A},C_{max}		0.548	0.777	0.647	0.657	0.115	17.5
$R_{A},AUC_{0-t(last)}$		0.42	0.507	0.402	0.443	0.0563	12.7

Table 42. Day 28 individual and mean (±SD, %CV) pharmacokinetic parameters of the sulfoxide metabolite after oral 50 mg/kg/day of Fexinidazole in male Beagle dogs.

Parameter	Units	ID 2514	ID 2521	ID 2529	Mean	SD	%CV
C_{max}	µg/mL	2.48	1.44	1.57	1.83	0.567	31
t_{max}	hour	2	1	2	1.67	0.577	34.6
AUC Interval (1)	hour	(0-24)	(0-24)	(0-24)			
$AUC_{0-t(last)}$	µg·hour/mL	15.9	15.7	11.1	14.2	2.72	19.1
C_{max}/Dose	µg/mL	0.0496	0.0288	0.0314	0.0366	0.0113	31
$AUC_{0-t(last)}$/Dose	µg·hour/mL	0.318	0.314	0.221	0.284	0.0549	19.3
R_{A},C_{max}		0.492	0.543	0.532	0.523	0.027	5.2
$R_{A},AUC_{0-t(last)}$		0.619	0.892	0.771	0.761	0.137	18
(1) Nominal time							

Table 43. Day 1 individual and mean (±SD, %CV) pharmacokinetic parameters of the sulfoxide metabolite after oral 50 mg/kg/day of Fexinidazole in female Beagle dogs.

Parameter	Units	ID 2562	ID 2563	ID 2576	Mean	SD	%CV
C_{max}	µg/mL	3.6	4.82	3.49	3.97	0.738	18.6
t_{max}	hour	2	1	1	1.33	0.577	43.3
AUC Interval	hour	(0-24)	(0-8)	(0-24)			
$AUC_{0\text{-}t(last)}$	µg·hour/mL	26.2	12.7	21	20	6.81	34.1
C_{max}/Dose	µg/mL	0.072	0.0964	0.0698	0.0794	0.0148	18.6
$AUC_{0\text{-}t(last)}$/Dose	µg·hour/mL	0.524	0.255	0.42	0.4	0.136	33.9

Table 44. Day 14 individual and mean (±SD, %CV) pharmacokinetic parameters of the sulfoxide metabolite after oral 50 mg/kg/day of Fexinidazole in female Beagle dogs.

Parameter	Units	ID 2562	ID 2563	ID 2576	Mean	SD	%CV
C_{max}	µg/mL	3.12	3.53	4.53	3.73	0.725	19.5
t_{max}	hour	1	2	2	1.67	0.577	34.6
AUC Interval	hour	(0-24)	(0-24)	(0-8)			
$AUC_{0\text{-}t(last)}$	µg·hour/mL	21.1	17.2	18	18.8	2.06	11
C_{max}/Dose	µg/mL	0.0624	0.0706	0.0906	0.0745	0.0145	19.5
$AUC_{0\text{-}t(last)}$/Dose	µg·hour/mL	0.421	0.344	0.36	0.375	0.0406	10.8
R_{A},C_{max}		0.867	0.732	1.3	0.966	0.296	30.6
R_{A},$AUC_{0\text{-}t(last)}$		0.805	1.35	0.857	1.01	0.303	30.1

Table 45. Day 28 individual and mean (±SD, %CV) pharmacokinetic parameters of the sulfoxide metabolite after oral 50 mg/kg/day of Fexinidazole in female Beagle dogs.

Parameter	Units	ID 2562	ID 2563	ID 2576	Mean	SD	%CV
C_{max}	µg/mL	1.91	2.91	3.33	2.72	0.729	26.9
t_{max}	hour	1	1	1	1	0	0
AUC Interval (1)	hour	(0-24)	(0-24)	(0-24)			
$AUC_{0\text{-}t(last)}$	µg·hour/mL	6.93	12.3	10.4	9.88	2.72	27.6
C_{max}/Dose	µg/mL	0.0382	0.0582	0.0666	0.0543	0.0146	26.9
$AUC_{0\text{-}t(last)}$/Dose	µg·hour/mL	0.139	0.245	0.207	0.197	0.0537	27.3
R_{A},C_{max}		0.531	0.604	0.954	0.696	0.226	32.5
R_{A},$AUC_{0\text{-}t(last)}$		0.265	0.969	0.495	0.576	0.359	62.3
(1) Nominal time							

Table 46. Day 1 individual and mean (±SD, %CV) pharmacokinetic parameters of the sulfoxide metabolite after oral 200 mg/kg/day of Fexinidazole in male Beagle dogs.

Parameter	Units	ID 2515	ID 2523	ID 2526	Mean	SD	%CV
C_{max}	µg/mL	6.37	7.97	8.3	7.55	1.03	13.7
t_{max}	hour	1	2	2	1.67	0.58	34.6
AUC Interval	hour	(0-24)	(0-24)	(0-24			
$AUC_{0-t(last)}$	µg·hour/mL	44.5	47	60.5	50.7	8.61	17
C_{max}/Dose	µg/mL	0.0319	0.0399	0.0415	0.0378	0.0051	13.6
$AUC_{0-t(last)}$/Dose	µg·hour/mL	0.223	0.235	0.302	0.253	0.0426	16.8

Table 47. Day 14 individual and mean (±SD, %CV) pharmacokinetic parameters of the sulfoxide metabolite after oral 200 mg/kg/day of Fexinidazole in male Beagle dogs.

Parameter	Units	ID 2515	ID 2523	ID 2526	Mean	SD	%CV
C_{max}	µg/mL	6.97	11.8	8.1	8.96	2.53	28.2
t_{max}	hour	2	2	2	2	0	0
AUC Interval	hour	(0-24)	(0-24)	(0-24)			
$AUC_{0-t(last)}$	µg·hour/mL	57.4	65.4	45.6	56.1	9.96	17.7
C_{max}/Dose	µg/mL	0.0349	0.059	0.0405	0.0448	0.0126	28.2
$AUC_{0-t(last)}$/Dose	µg·hour/mL	0.287	0.327	0.228	0.281	0.0498	17.7
$R_{A,Cmax}$		1.09	1.48	0.976	1.18	0.264	22.3
$R_{A,AUC0-t(last)}$		1.29	1.39	0.754	1.15	0.343	29.9

Table 48. Day 28 individual and mean (±SD, %CV) pharmacokinetic parameters of the sulfoxide metabolite after oral 200 mg/kg/day of Fexinidazole in male Beagle dogs.

Parameter	Units	ID 2515	ID 2523	ID 2526	Mean	SD	%CV
C_{max}	µg/mL	6.96	5.7	4.62	5.76	1.17	20.3
t_{max}	hour	1	2	1	1.33	0.58	43.3
AUC Interval [(1)]	hour	(0-24)	(0-24)	(0-24)			
$AUC_{0-t(last)}$	µg·hour/mL	44.7	47.1	34.9	42.2	6.46	15.3
C_{max}/Dose	µg/mL	0.0348	0.0285	0.0231	0.0288	0.0059	20.3
$AUC_{0-t(last)}$/Dose	µg·hour/mL	0.224	0.235	0.174	0.211	0.0325	15.4
$R_{A,Cmax}$		1.09	0.715	0.557	0.788	0.275	34.9
$R_{A,AUC0-t(last)}$		1	1	0.577	0.861	0.246	28.6
[(1)] Nominal time							

Table 49. Day 1 individual and mean (±SD, %CV) pharmacokinetic parameters of the sulfoxide metabolite after oral 200 mg/kg/day of Fexinidazole in female Beagle dogs.

Parameter	Units	ID 2561	ID 2567	ID 2570	Mean	SD	%CV
C_{max}	µg/mL	4.92	12.8	8.38	8.7	3.95	45.4
t_{max}	hour	0.5	1	1	0.833	0.289	34.6
AUC Interval	hour	(0-24)	(0-24)	(0-24)			
$AUC_{0-t(last)}$	µg·hour/mL	39.4	75.8	41	52.1	20.6	39.5
C_{max}/Dose	µg/mL	0.0246	0.064	0.0419	0.0435	0.0197	45.4
$AUC_{0-t(last)}$/Dose	µg·hour/mL	0.197	0.379	0.205	0.26	0.103	39.5

Table 50. Day 14 individual and mean (±SD, %CV) pharmacokinetic parameters of the sulfoxide metabolite after oral 200 mg/kg/day of Fexinidazole in female Beagle dogs.

Parameter	Units	ID 2561	ID 2567	ID 2570	Mean	SD	%CV
C_{max}	µg/mL	5.32	12.7	9.03	9.02	3.69	40.9
t_{max}	hour	2	2	2	2	0	0
AUC Interval	hour	(0-24)	(0-24)	(0-24)			
$AUC_{0-t(last)}$	µg·hour/mL	31.7	80.1	59.8	57.2	24.3	42.5
C_{max}/Dose	µg/mL	0.0266	0.0635	0.0452	0.0451	0.0185	40.9
$AUC_{0-t(last)}$/Dose	µg·hour/mL	0.159	0.401	0.299	0.286	0.121	42.4
R_A,C_{max}		1.08	0.992	1.08	1.05	0.0504	4.8
R_A,$AUC_{0-t(last)}$		0.805	1.06	1.46	1.11	0.33	29.8

Table 51. Day 28 individual and mean (±SD, %CV) pharmacokinetic parameters of the sulfoxide metabolite after oral 200 mg/kg/day of Fexinidazole in female Beagle dogs.

Parameter	Units	ID 2561	ID 2567	ID 2570	Mean	SD	%CV
C_{max}	µg/mL	5.41	5.78	5.1	5.43	0.34	6.3
t_{max}	hour	1	1	1	1	0	0
AUC Interval (1)	hour	(0-24)	(0-24)	(0-24)			
$AUC_{0-t(last)}$	µg·hour/mL	31.9	34.6	34.9	33.8	1.65	4.9
C_{max}/Dose	µg/mL	0.0271	0.0289	0.0255	0.027	0.0017	6.3
$AUC_{0-t(last)}$/Dose	µg·hour/mL	0.16	0.173	0.174	0.169	0.00781	4.6
R_A,C_{max}		1.1	0.452	0.609	0.72	0.338	47
R_A,$AUC_{0-t(last)}$		0.81	0.456	0.851	0.706	0.217	30.7

(1) Nominal time

Table 52. Day 1 individual and mean (±SD, %CV) pharmacokinetic parameters of the sulfoxide metabolite after oral 800 mg/kg/day of Fexinidazole in male Beagle dogs.

Parameter	Units	Dog ID					Mean	SD	%CV
		2517	2519	2525	2528	2530			
C_{max}	µg/mL	17.5	15.2	9.39	11.1	13.7	13.4	3.22	24.1
t_{max}	hour	0.5	2	2	2	1	1.5	0.707	47.1
AUC Interval	hour	(0-24)	(0-24)	(0-24)	(0-24)	(0-24)			
$AUC_{0-t(last)}$	µg·hour/mL	89.7	95	105	114	115	104	11.3	10.9
C_{max}/Dose	µg/mL	0.0219	0.019	0.0117	0.0139	0.0171	0.017	0.00404	24.2
$AUC_{0-t(last)}$/Dose	µg·hour/mL	0.112	0.119	0.131	0.143	0.144	0.13	0.0142	11

Table 53. Day 14 individual and mean (±SD, %CV) pharmacokinetic parameters of the sulfoxide metabolite after oral 800 mg/kg/day of Fexinidazole in male Beagle dogs.

Parameter	Units	Dog ID					Mean	SD	%CV
		2517	2519	2525	2528	2530			
C_{max}	µg/mL	12.8	14.9	7.56	13.9	13.1	12.5	2.85	22.9
t_{max}	hour	1	1	1	1	2	1.2	0.4472	37.3
AUC Interval	hour	(0-24)	(0-24)	(0-24)	(0-24)	(0-24)			
$AUC_{0-t(last)}$	µg·hour/mL	116	77.5	69	160	144	113	39.9	35.2
C_{max}/Dose	µg/mL	0.016	0.0186	0.00945	0.0174	0.0164	0.016	0.00357	22.9
$AUC_{0-t(last)}$/Dose	µg·hour/mL	0.145	0.0969	0.0863	0.200	0.18	0.142	0.0499	35.2
R_A,C_{max}		0.731	0.98	0.805	1.25	0.956	0.945	0.201	21.2
R_A,$AUC_{0-t(last)}$		1.29	0.816	0.657	1.4	1.25	1.08	0.327	30.2

Table 54. Day 28 individual and mean (±SD, %CV) pharmacokinetic parameters of the sulfoxide metabolite after oral 800 mg/kg/day of Fexinidazole in male Beagle dogs.

Parameter	Units	Dog ID					Mean	SD	%CV
		2517	2519	2525	2528	2530			
C_{max}	µg/mL	15.1	6.19	8.2	7.04	10.2	9.35	3.55	38
t_{max}	hour	1	2	1	2	2	1.6	0.548	34.2
AUC Interval (1)	hour	(0-24)	(0-24)	(0-24)	(0-48)	(0-48)			
AUC_{0-24}	µg·hour/mL	87.3	59.8	57.6	84.1	82.8	74.3	14.4	19.3
$AUC_{0-t(last)}$	µg·hour/mL				97.3	144	121	33	27.4
C_{max}/Dose		0.0189	0.00774	0.0103	0.0088	0.0128	0.012	0.00445	38
AUC_{0-24}/Dose	µg·hour/mL	0.109	0.0747	0.072	0.105	0.104	0.0929	0.018	19.4
$AUC_{0-t(last)}$/Dose	µg·hour/mL				0.122	0.18	0.151	0.041	27.2
$t_{1/2,z}$	hour				5.5	13.2	9.35	5.44	58.2
R_A,C_{max}		0.863	0.407	0.873	0.634	0.745	0.704	0.193	27.3
R_A,AUC_{0-24}		0.973	0.629	0.549	0.738	0.72	0.722	0.16	22.1

(1) Nominal time

Table 55. Day 1 individual and mean (±SD, %CV) pharmacokinetic parameters of the sulfoxide metabolite after oral 800 mg/kg/day of Fexinidazole in female Beagle dogs.

Parameter	Units	Dog ID					Mean	SD	%CV
		2564	2565	2569	2571	2573			
C_{max}	µg/mL	18.4	21.7	13.8	14.7	9.51	15.6	4.64	29.7
t_{max}	hour	2	2	1	1	2	1.6	0.548	34.2
AUC Interval	hour	(0-24)	(0-24)	(0-24)	(0-24)	(0-24)			
$AUC_{0-t(last)}$	µg·hour/mL	135	173	105	136	54	121	44.4	36.8
C_{max}/Dose	µg/mL	0.023	0.0271	0.0173	0.0184	0.0119	0.02	0.00578	29.6
$AUC_{0-t(last)}$/Dose	µg·hour/mL	0.168	0.216	0.131	0.17	0.0675	0.151	0.0553	36.8

Table 56 Day 14 individual and mean (±SD, %CV) pharmacokinetic parameters of the sulfoxide metabolite after oral 800 mg/kg/day of Fexinidazole in female Beagle dogs.

Parameter	Units	Dog ID					Mean	SD	%CV
		2564	2565	2569	2571	2573			
C_{max}	µg/mL	21.7	14.1	18.2	10.5	9.73	14.8	5.1	34.3
t_{max}	hour	1	4	2	2	1	2	1.22	61.2
AUC Interval	hour	(0-24)	(0-24)	(0-24)	(0-24)	(0-24)			
$AUC_{0-t(last)}$	µg·hour/mL	178	190	126	113	113	144	37.1	25.8
C_{max}/Dose	µg/mL	0.0271	0.0176	0.0228	0.0131	0.0122	0.019	0.0064	34.3
$AUC_{0-t(last)}$/Dose	µg·hour/mL	0.223	0.238	0.157	0.141	0.141	0.18	0.0469	26
R_A,C_{max}		1.18	0.65	1.32	0.714	1.02	0.977	0.29	29.7
R_A,$AUC_{0-t(last)}$		1.32	1.1	1.2	0.831	2.09	1.31	0.474	36.2

Table 57. Day 28 individual and mean (±SD, %CV) pharmacokinetic parameters of the sulfoxide metabolite after oral 800 mg/kg/day of Fexinidazole in female Beagle dogs.

Parameter	Units	Dog ID					Mean	SD	%CV
		2564	2565	2569	2571	2573			
C_{max}	µg/mL	11.9	13.2	9.8	7.83	4.66	9.48	3.38	35.7
t_{max}	hour	1	2	1	2	1	1.4	0.548	39.1
AUC Interval (1)	hour	(0-24)	(0-24)	(0-24)	(0-24)	(0-24)			
$AUC_{0-t(last)}$	µg·hour/mL	66.3	163	73.6	96.7	45.4	89	45.2	50.8
C_{max}/Dose	µg/mL	0.0149	0.0165	0.0123	0.00979	0.00583	0.0119	0.00423	35.7
$AUC_{0-t(last)}$/Dose	µg·hour/mL	0.0829	0.203	0.092	0.121	0.0567	0.111	0.0563	50.6
R_A,C_{max}		0.647	0.608	0.71	0.533	0.49	0.598	0.088	14.7
R_A,$AUC_{0-t(last)}$		0.491	0.942	0.701	0.711	0.841	0.737	0.17	23
(1) Nominal time									

Figure 1. Day 1 individual plasma concentrations (ng/mL) of Fexinidazole and metabolites after oral 50 mg/kg/day dose of Fexinidazole in male (upper panel) and female (lower panel) Beagle dogs.

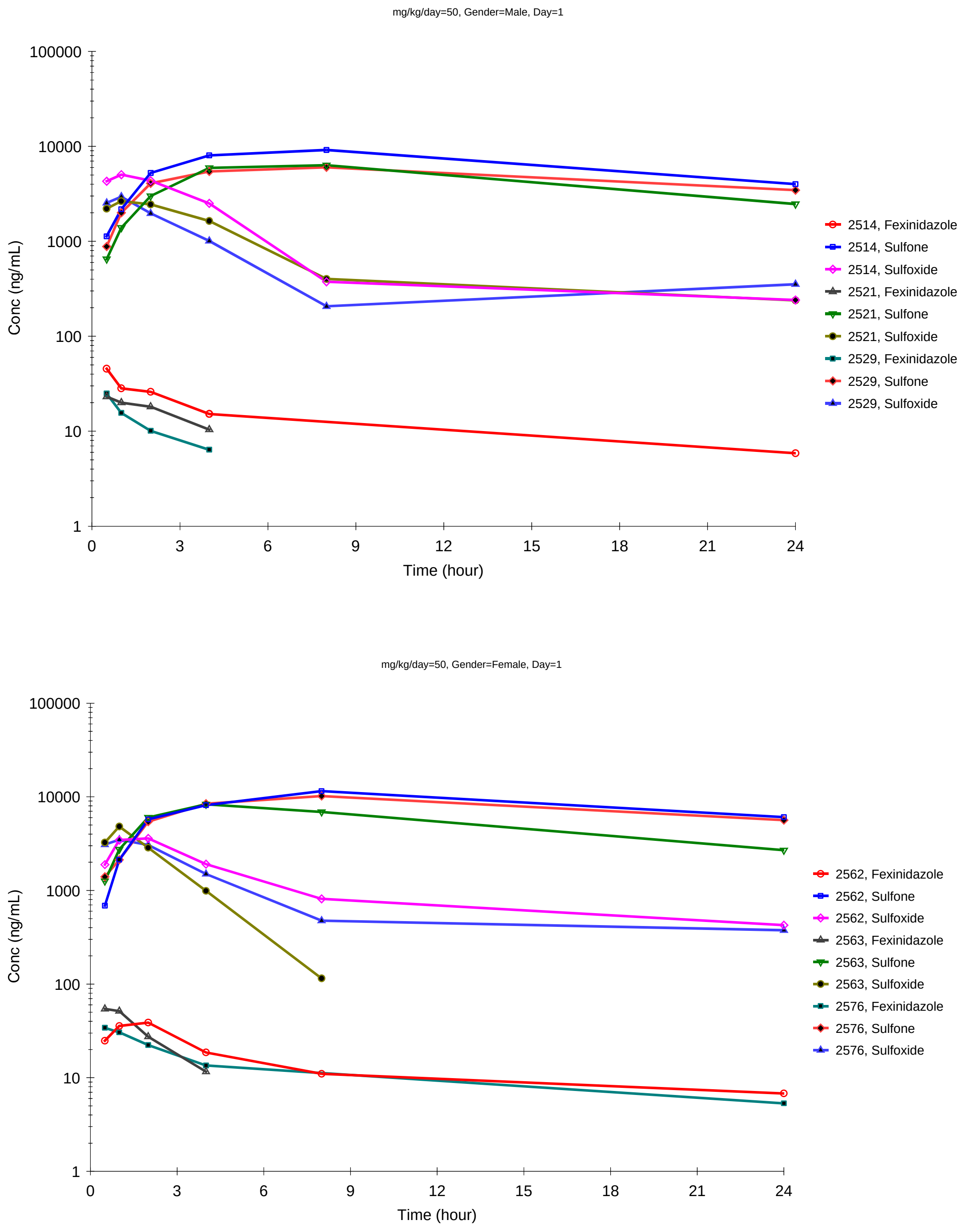

Figure 2. Day 14 individual plasma concentrations (ng/mL) of Fexinidazole and metabolites after oral 50 mg/kg/day dose of Fexinidazole in male (upper panel) and female (lower panel) Beagle dogs.

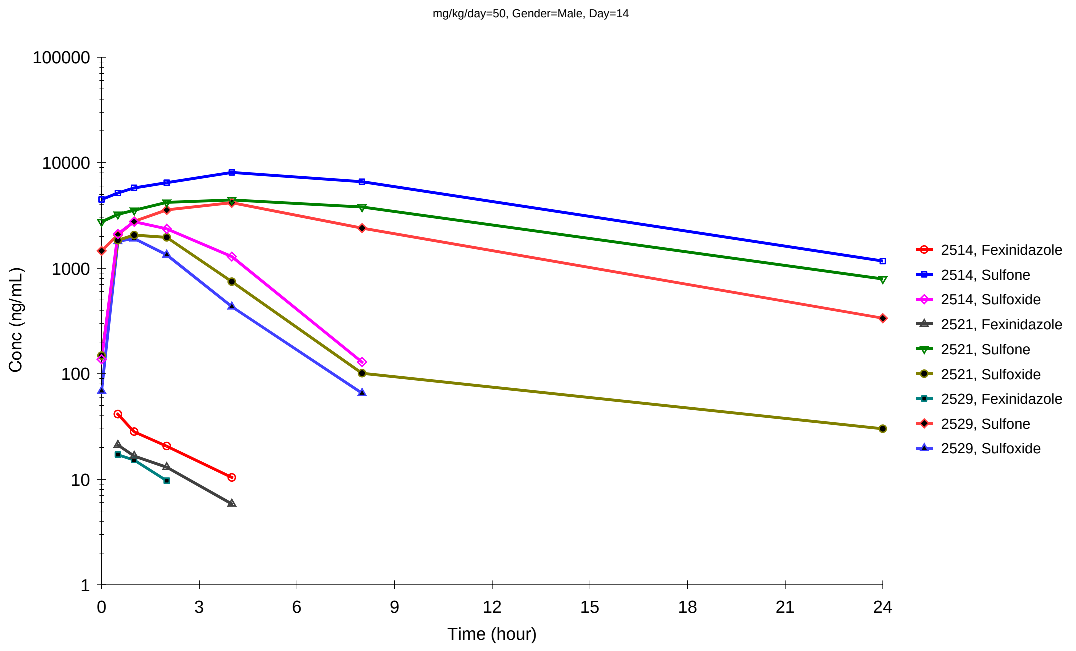

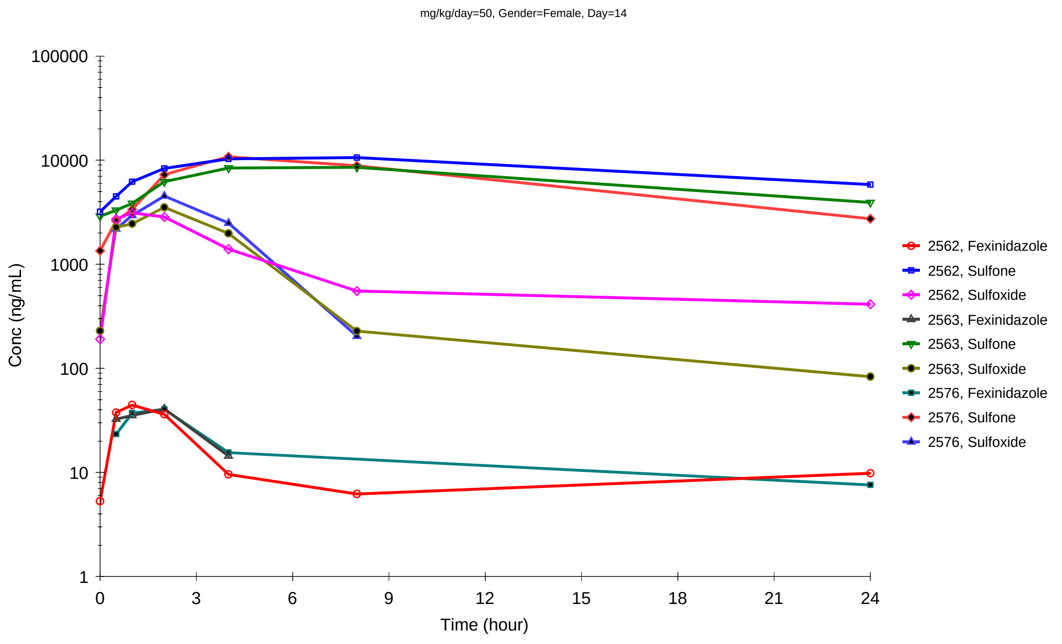

Figure 3. Day 28 individual plasma concentrations (ng/mL) of Fexinidazole and metabolites after oral 50 mg/kg/day dose of Fexinidazole in male (upper panel) and female (lower panel) Beagle dogs.

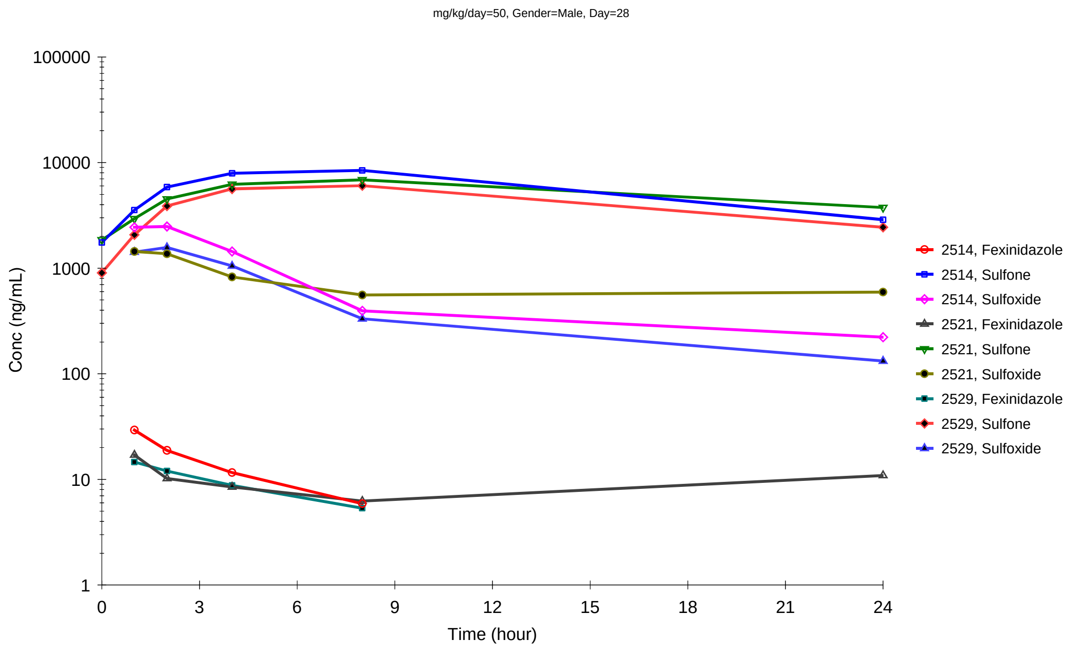

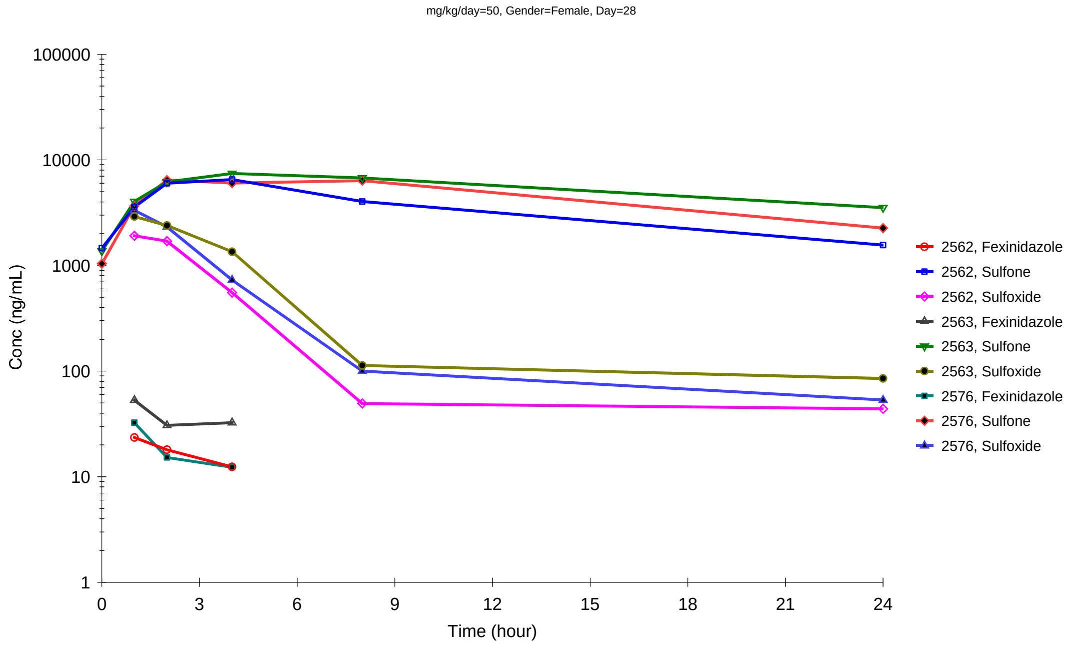

Figure 4. Day 1 individual plasma concentrations (ng/mL) of Fexinidazole and metabolites after oral 200 mg/kg/day dose of Fexinidazole in male (upper panel) and female (lower panel) Beagle dogs.

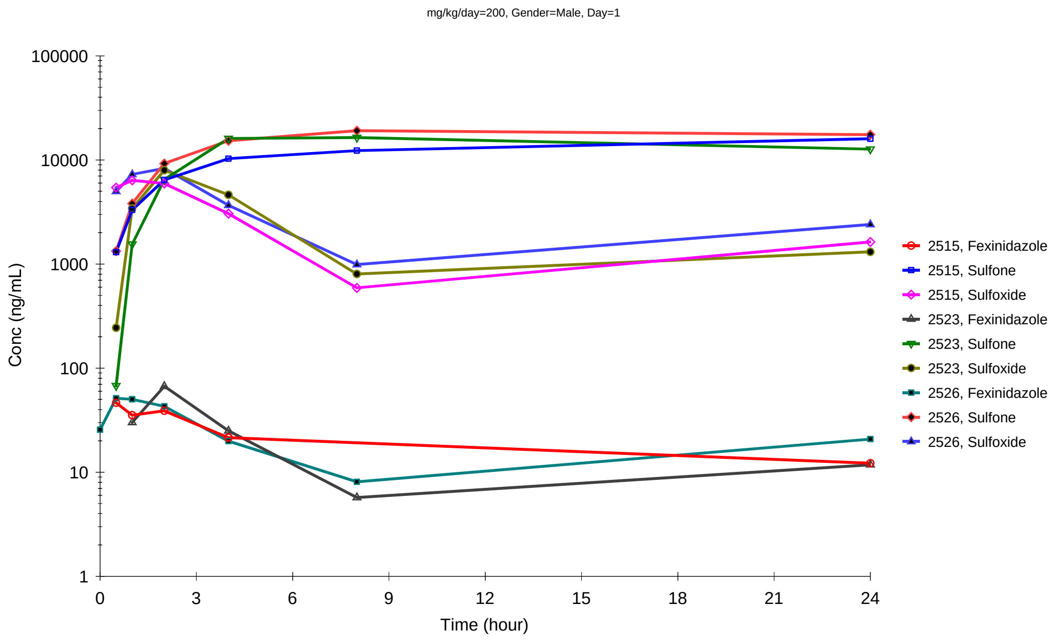

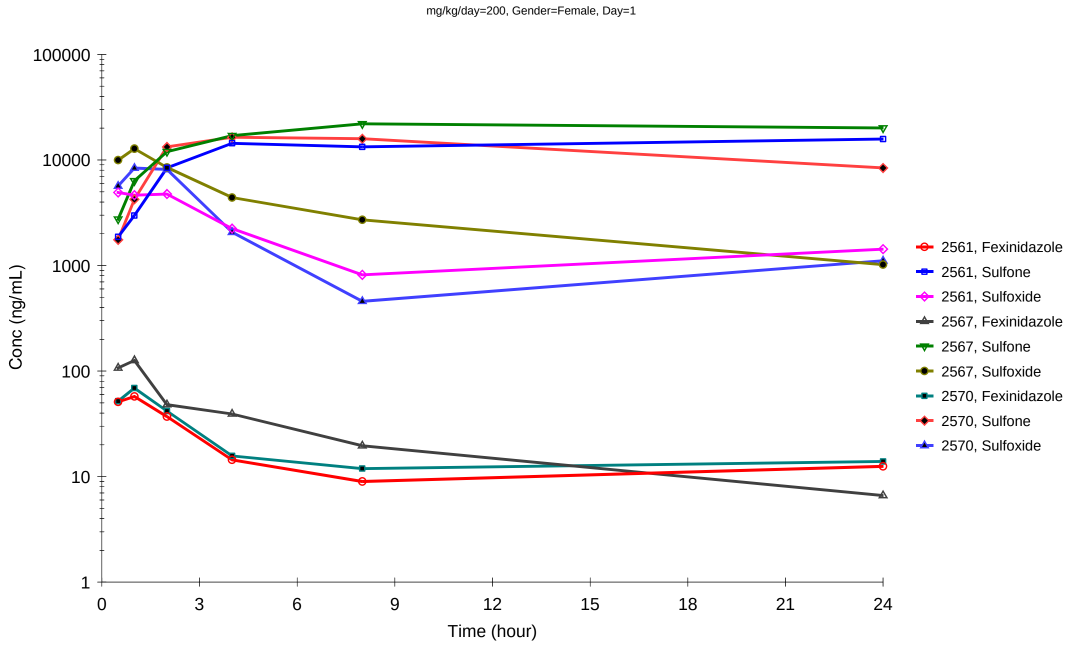

Figure 5. Day 14 individual plasma concentrations (ng/mL) of Fexinidazole and metabolites after oral 200 mg/kg/day dose of Fexinidazole in male (upper panel) and female (lower panel) Beagle dogs.

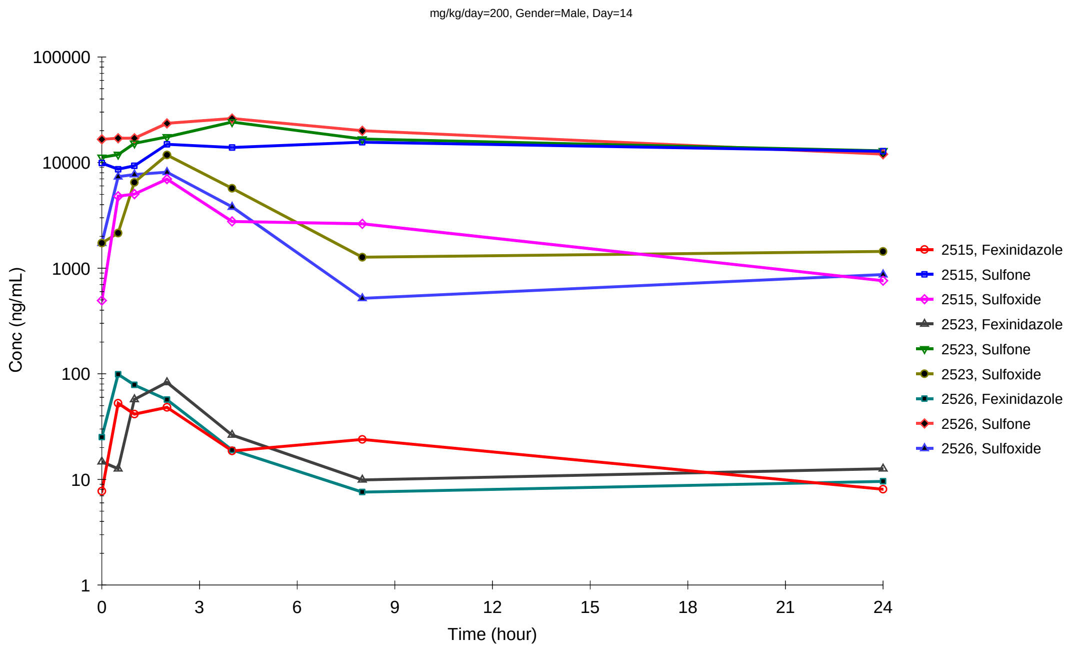

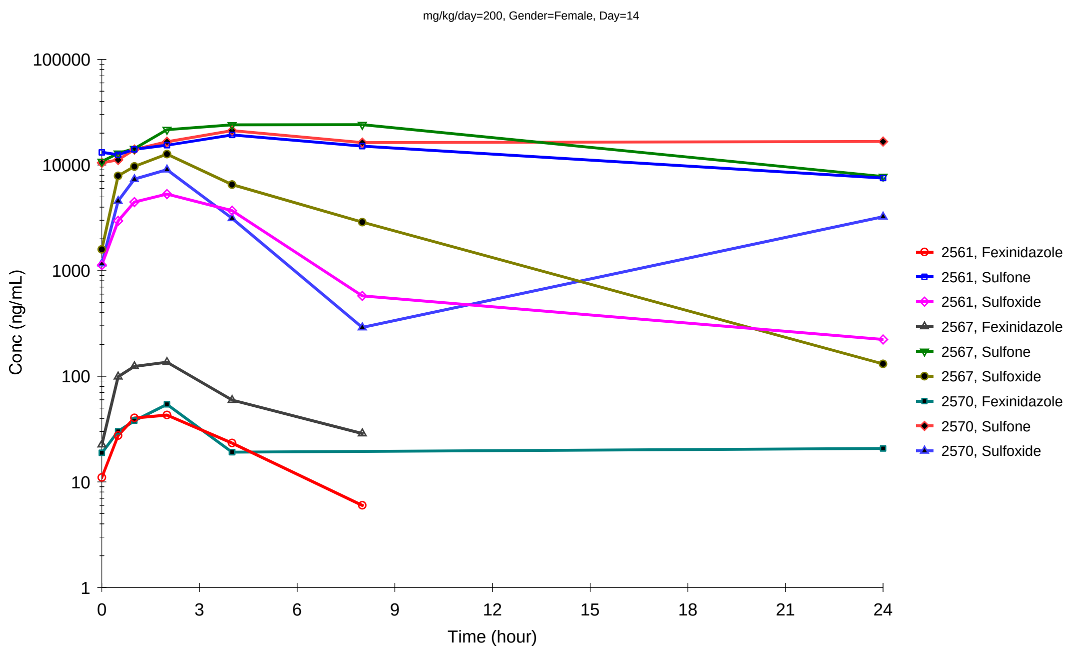

Figure 6. Day 28 individual plasma concentrations (ng/mL) of Fexinidazole and metabolites after oral 200 mg/kg/day dose of Fexinidazole in male (upper panel) and female (lower panel) Beagle dogs.

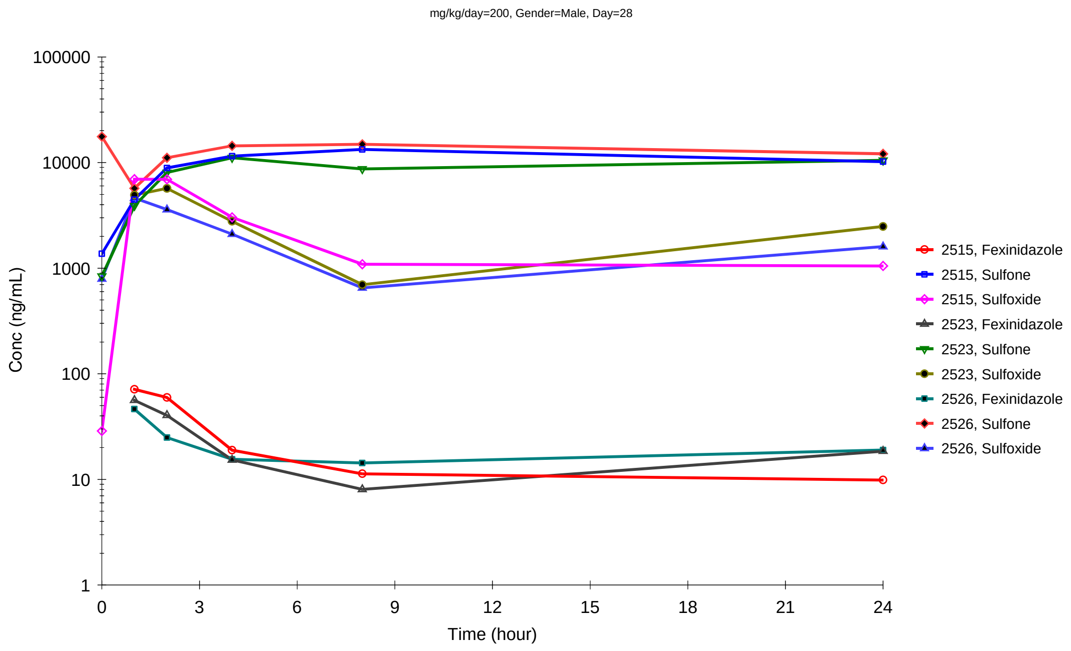

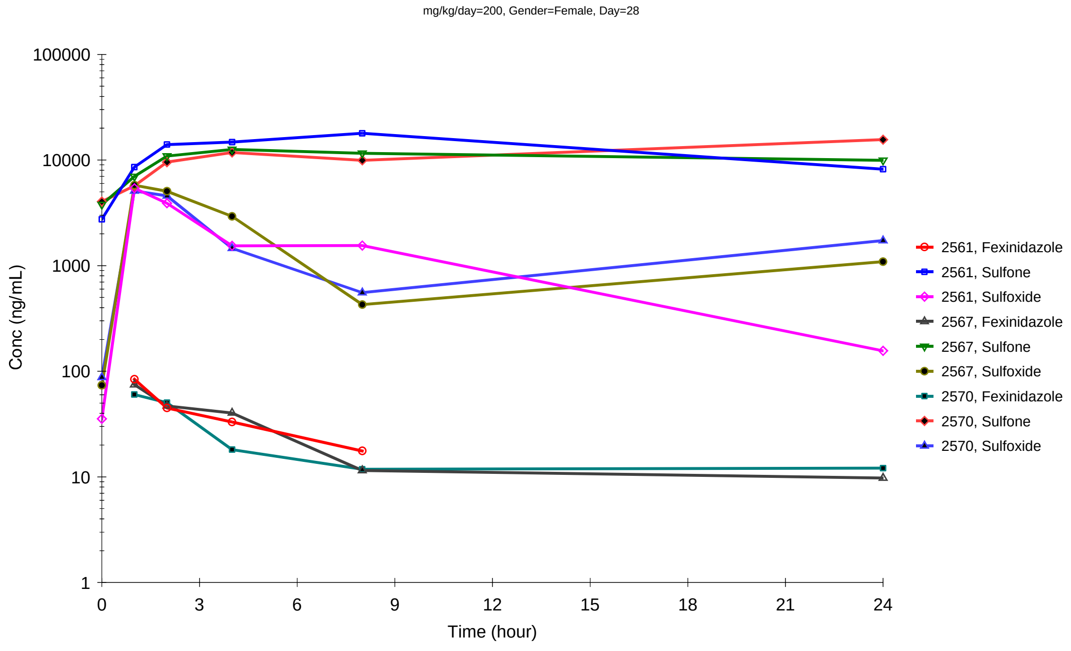

Figure 7. Day 1 individual plasma concentrations (ng/mL) of Fexinidazole after oral 800 mg/kg/day dose of Fexinidazole in male (upper panel) and female (lower panel) Beagle

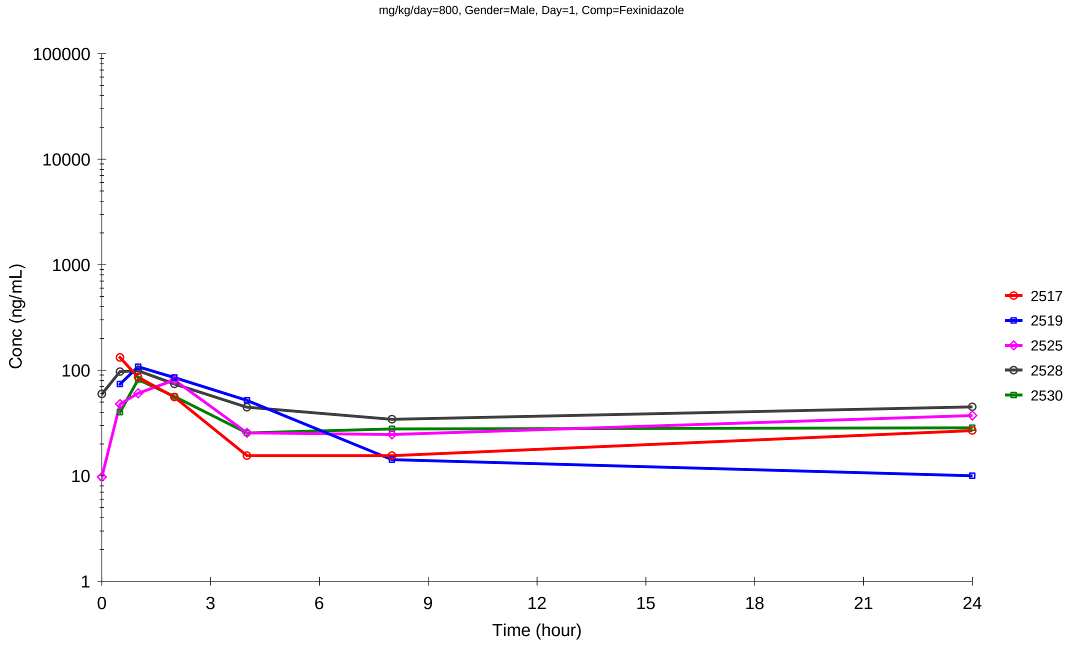

dogs.

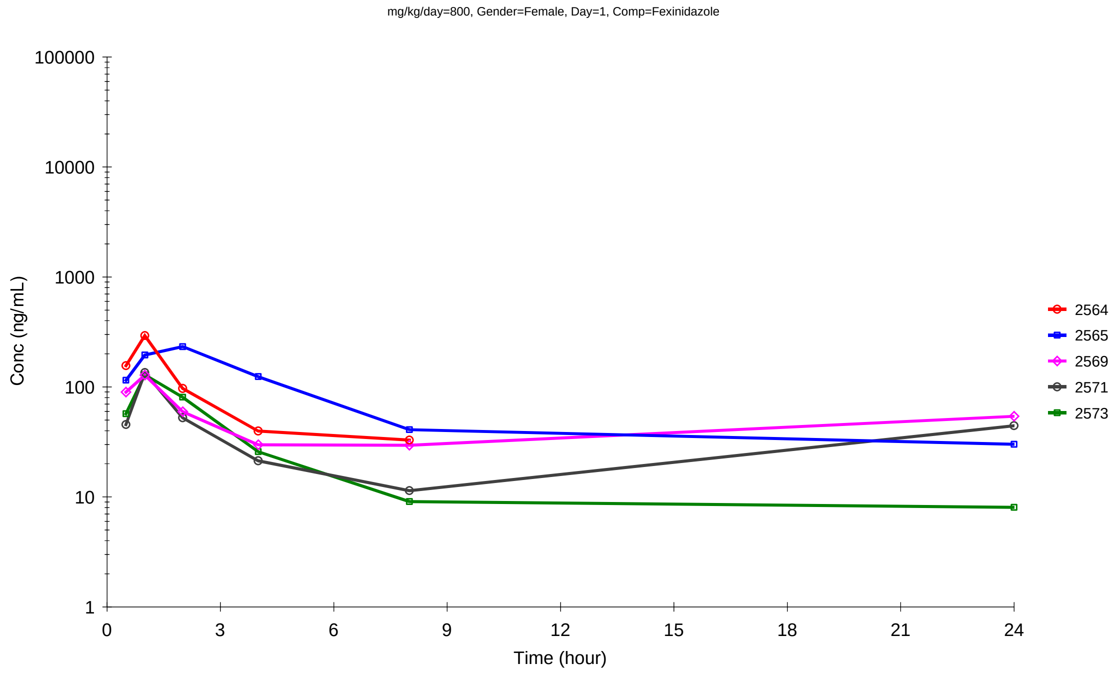

Figure 8. Day 1 individual plasma concentrations (ng/mL) of sulfone metabolite after oral 800 mg/kg/day dose of Fexinidazole in male (upper panel) and female (lower panel) Beagle dogs.

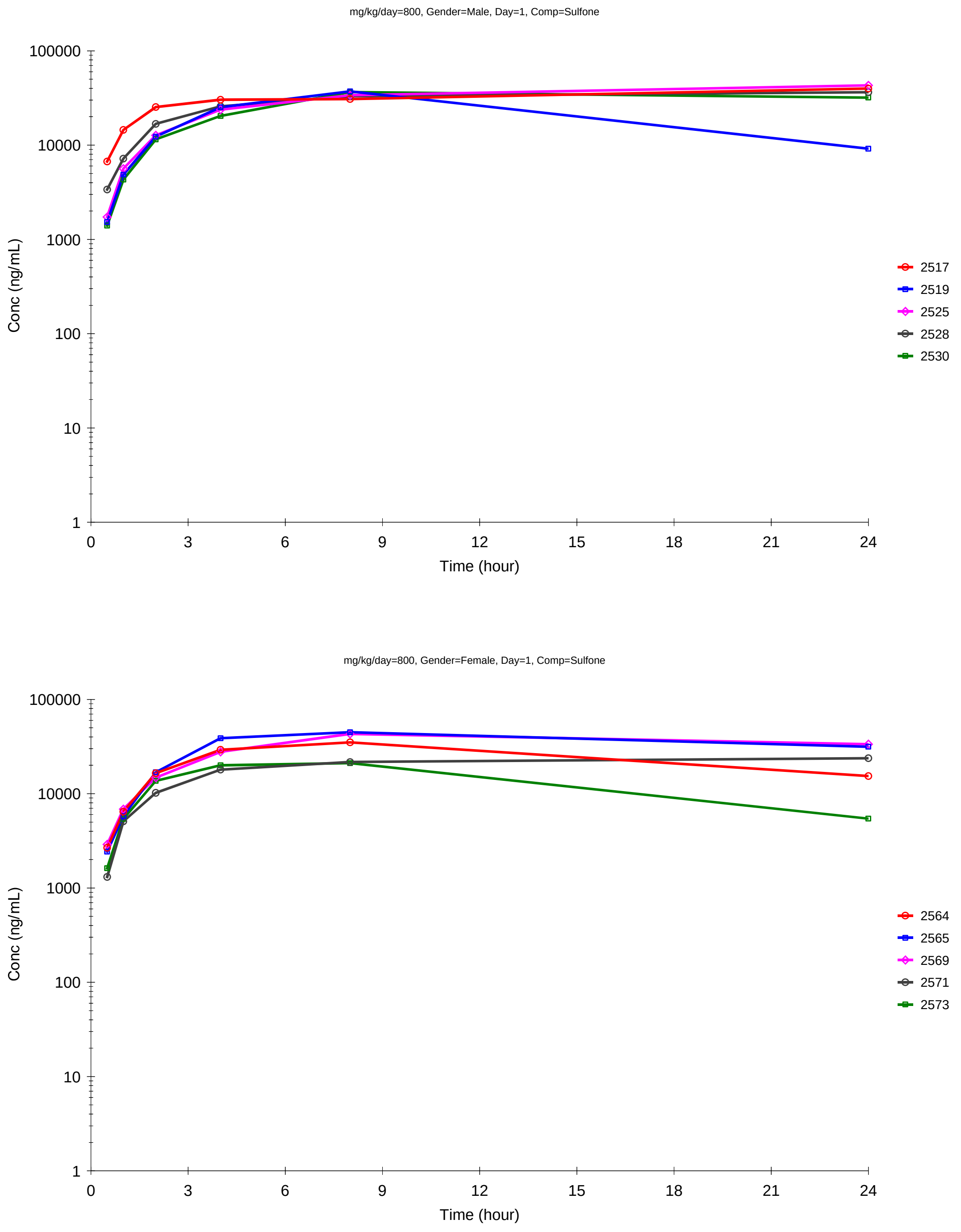

Figure 9. Day 1 individual plasma concentrations (ng/mL) of sulfoxide metabolite after oral 800 mg/kg/day dose of Fexinidazole in male (upper panel) and female (lower panel) Beagle dogs.

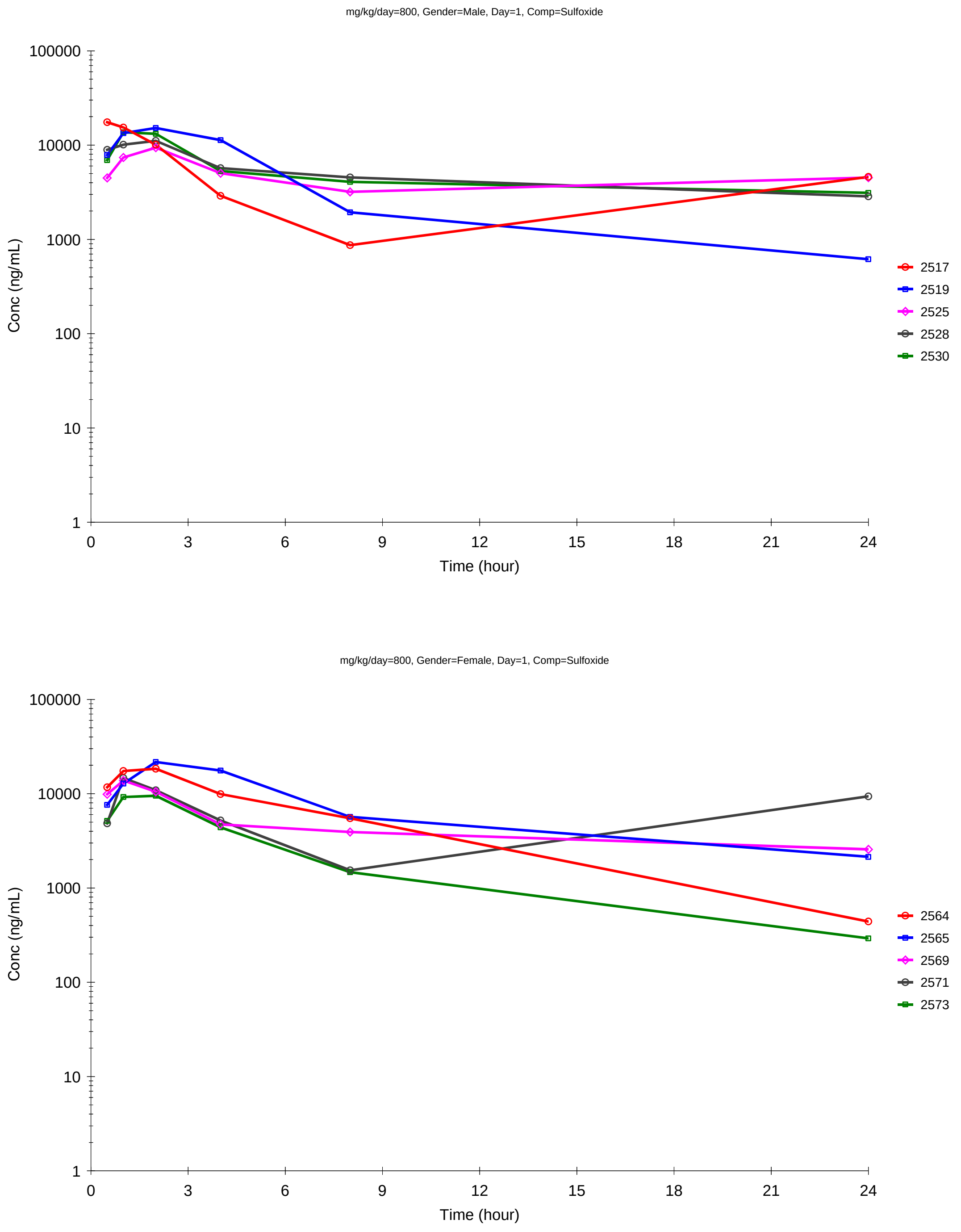

Figure 10. Day 14 individual plasma concentrations (ng/mL) of Fexinidazole after oral 800 mg/kg/day dose of Fexinidazole in male (upper panel) and female (lower panel) Beagle

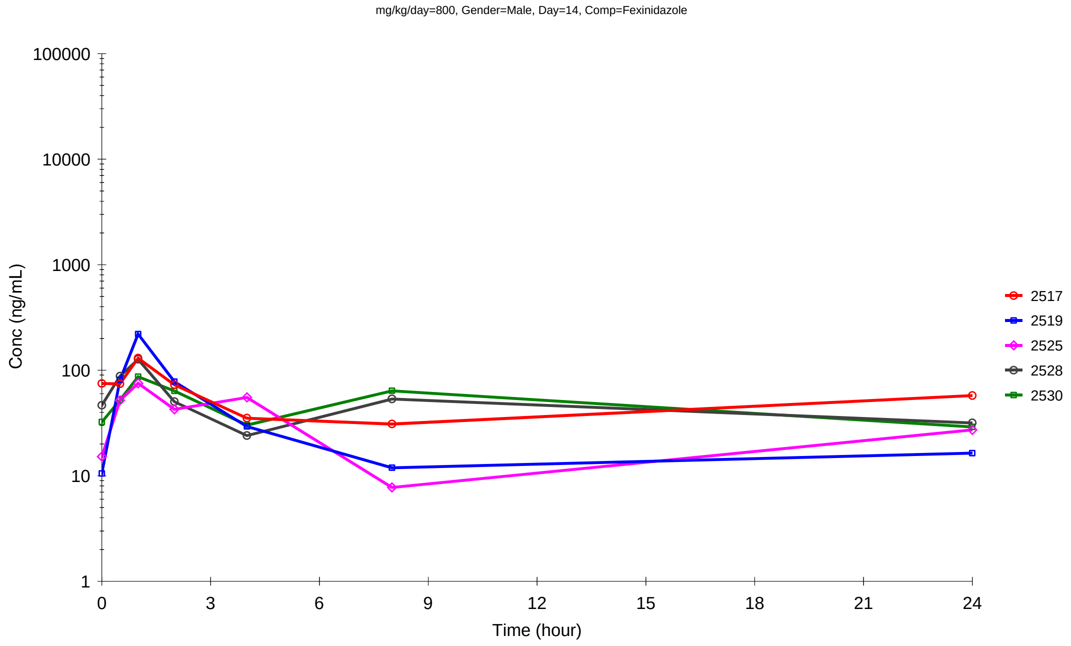

dogs.

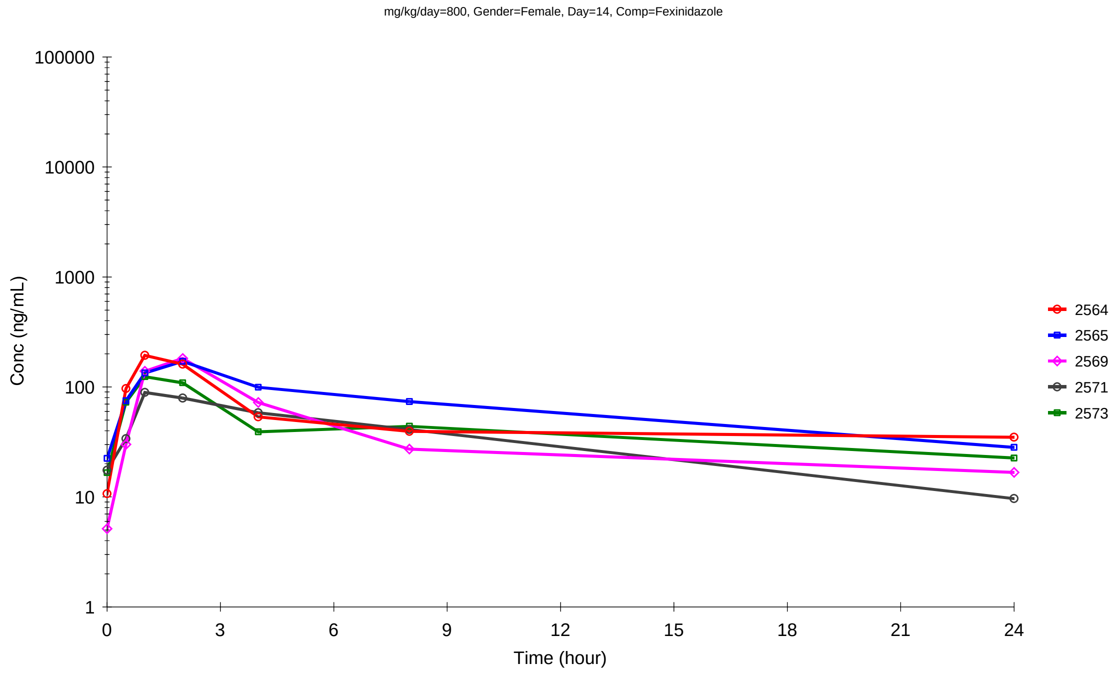

Figure 11. Day 14 individual plasma concentrations (ng/mL) of sulfone metabolite after oral 800 mg/kg/day dose of Fexinidazole in male (upper panel) and female (lower panel) Beagle dogs.

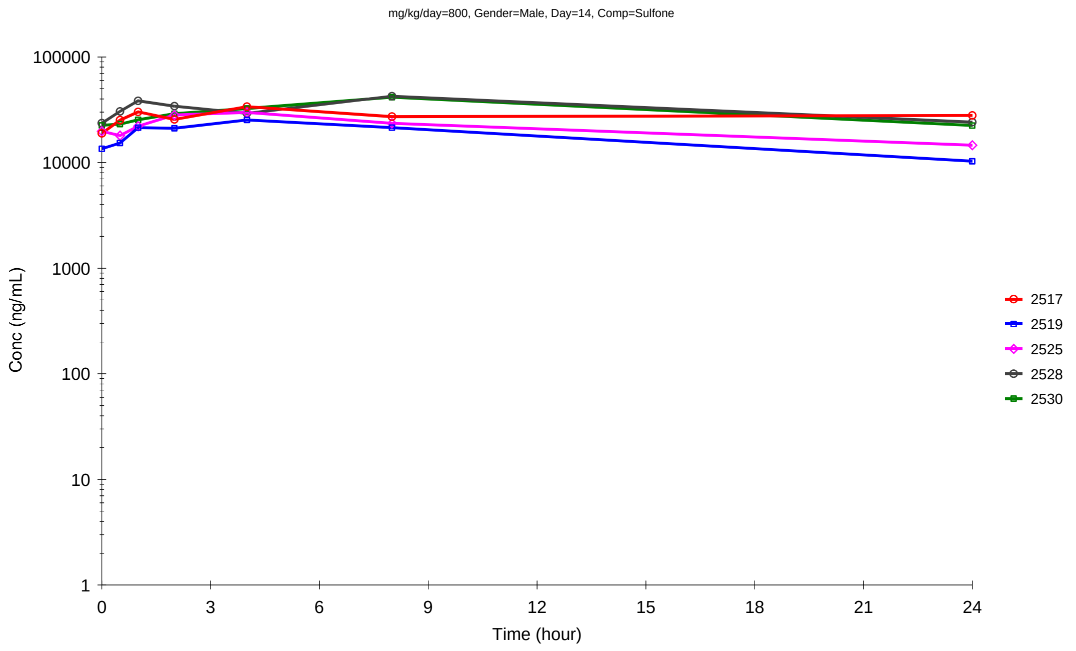

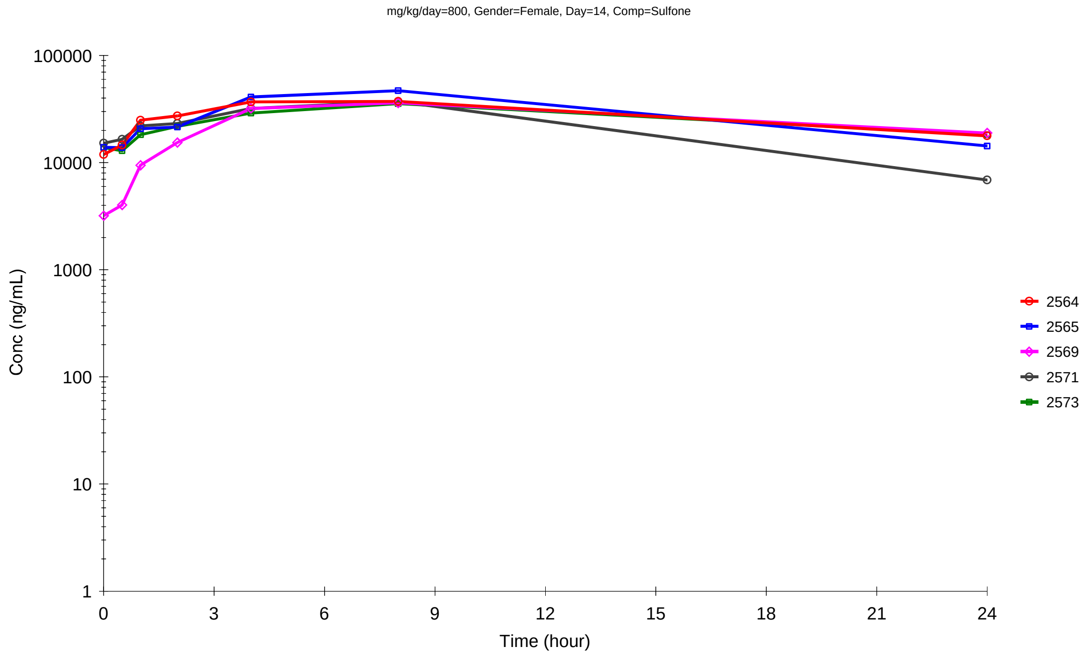

Figure 12. Day 14 individual plasma concentrations (ng/mL) of sulfoxide metabolite after oral 800 mg/kg/day dose of Fexinidazole in male (upper panel) and female (lower panel) Beagle dogs.

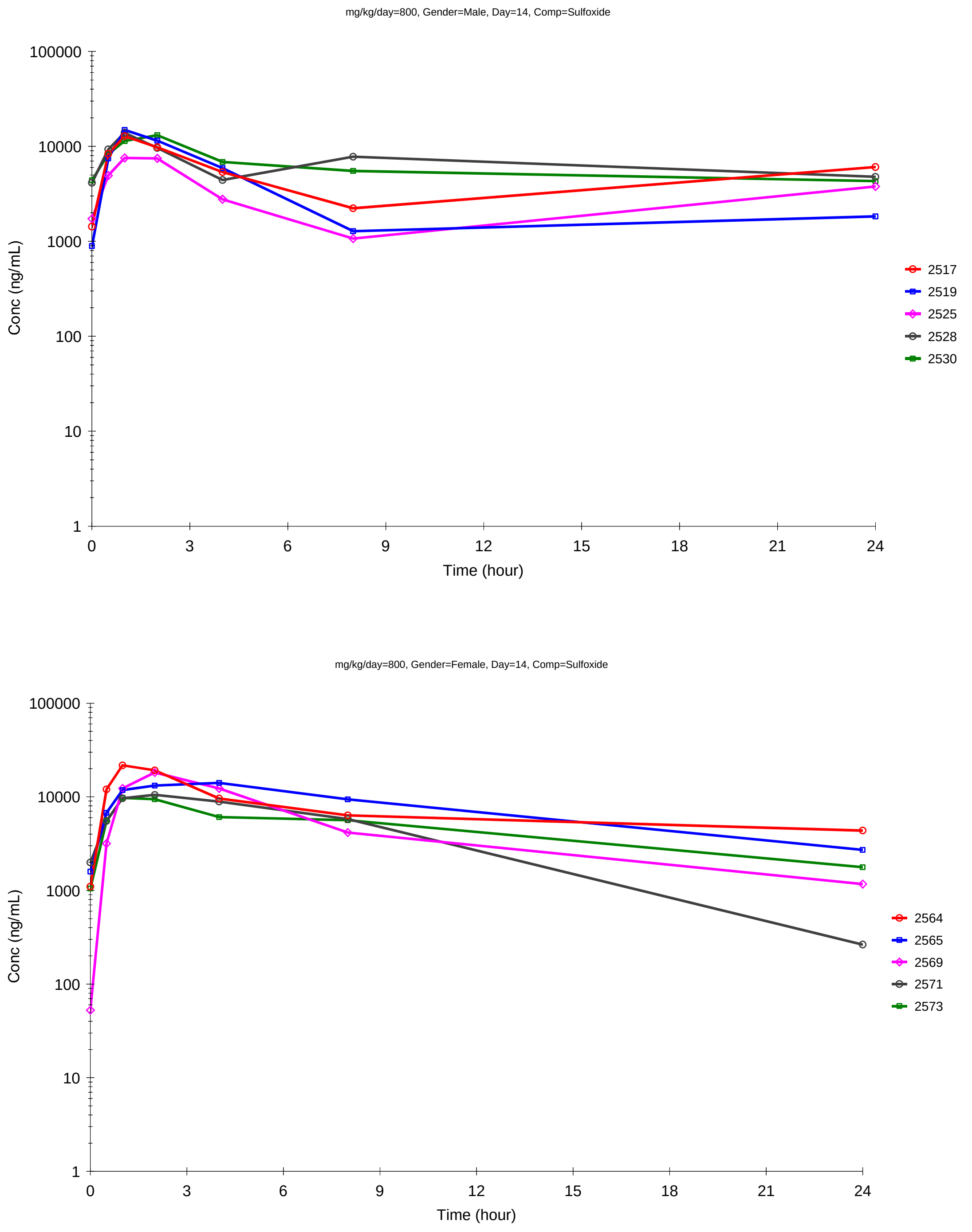

Figure 13. Day 28 individual plasma concentrations (ng/mL) of Fexinidazole after oral 800 mg/kg/day dose of Fexinidazole in male (upper panel) and female (lower panel) Beagle dogs.

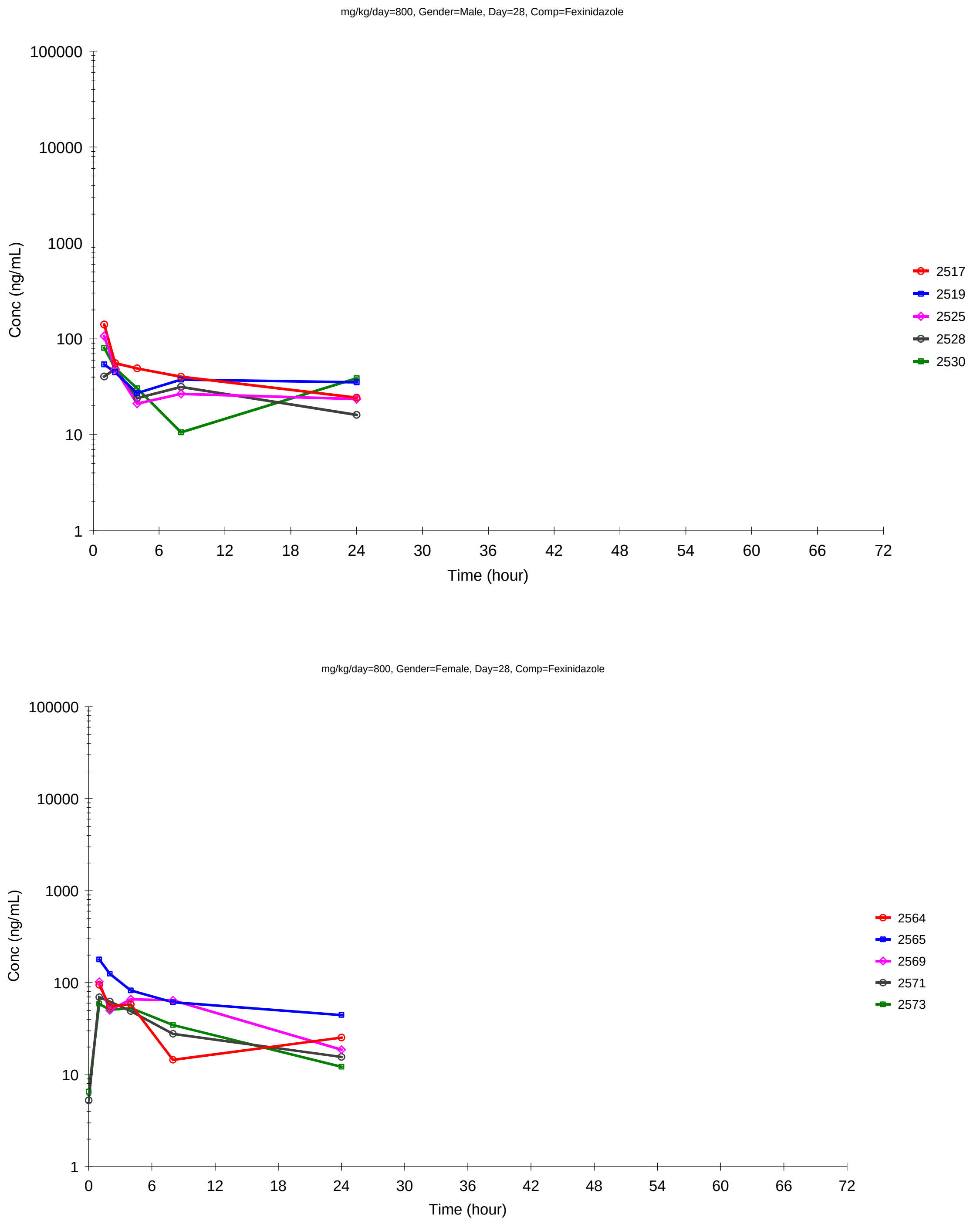

Figure 14. Day 28 individual plasma concentrations (ng/mL) of sulfone metabolite after oral 800 mg/kg/day dose of Fexinidazole in male (upper panel) and female (lower panel) Beagle dogs.

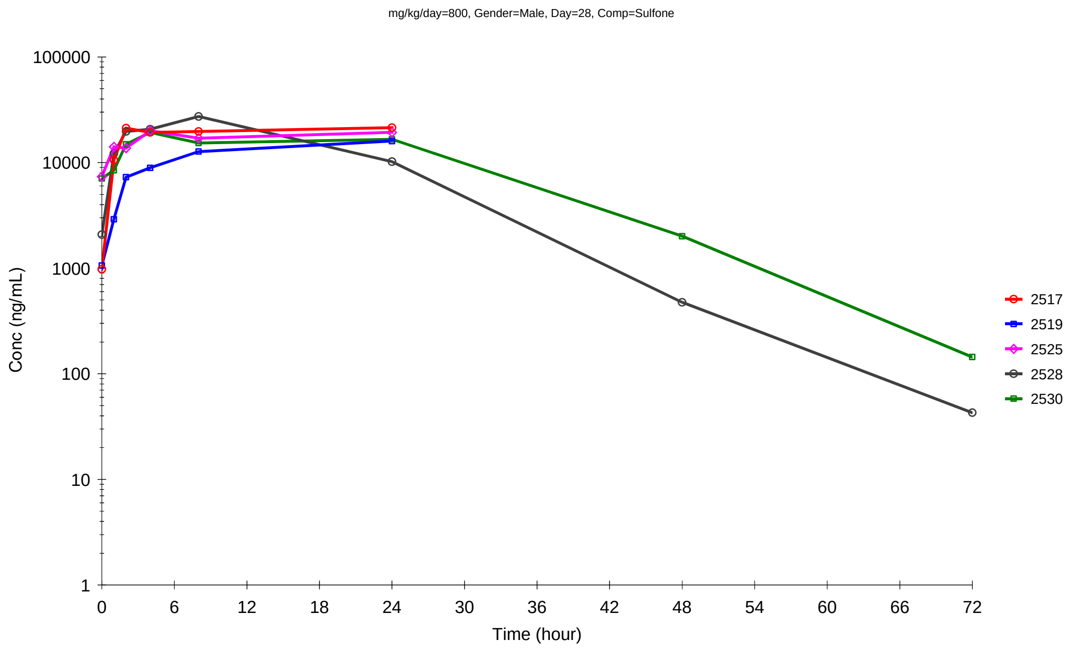

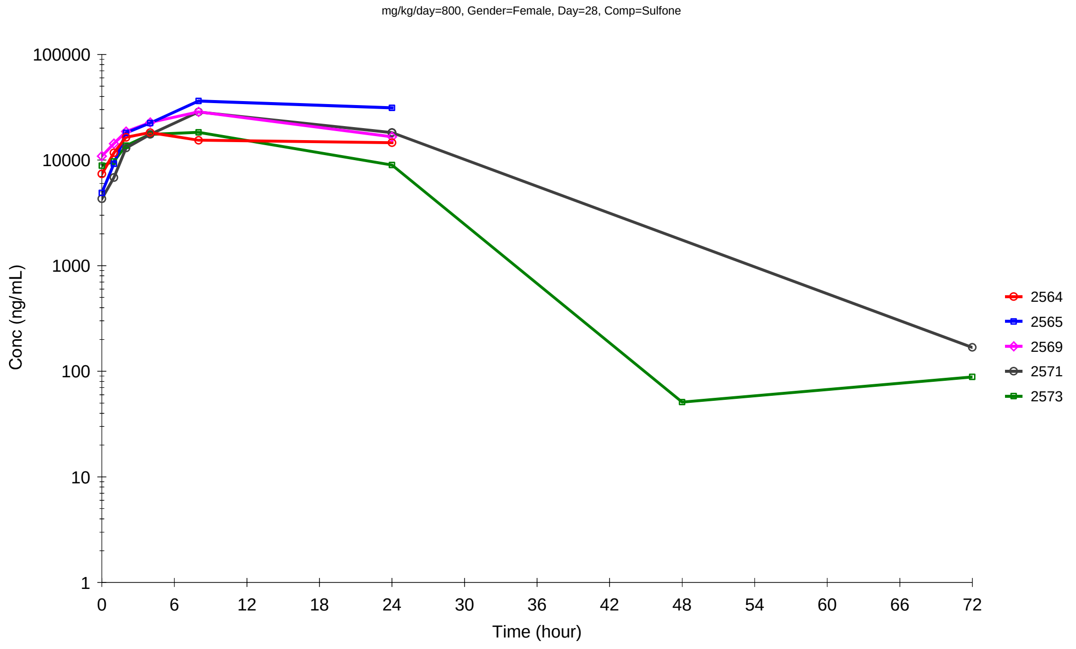

Figure 15. Day 28 individual plasma concentrations (ng/mL) of sulfoxide metabolite after oral 800 mg/kg/day dose of Fexinidazole in male (upper panel) and female (lower panel) Beagle dogs.

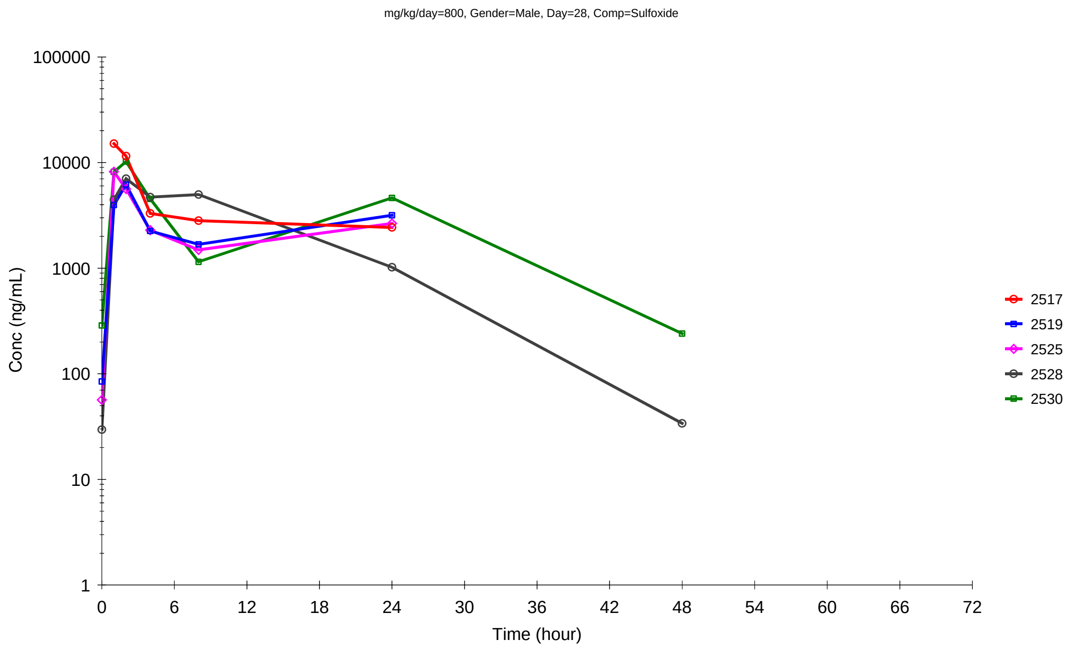

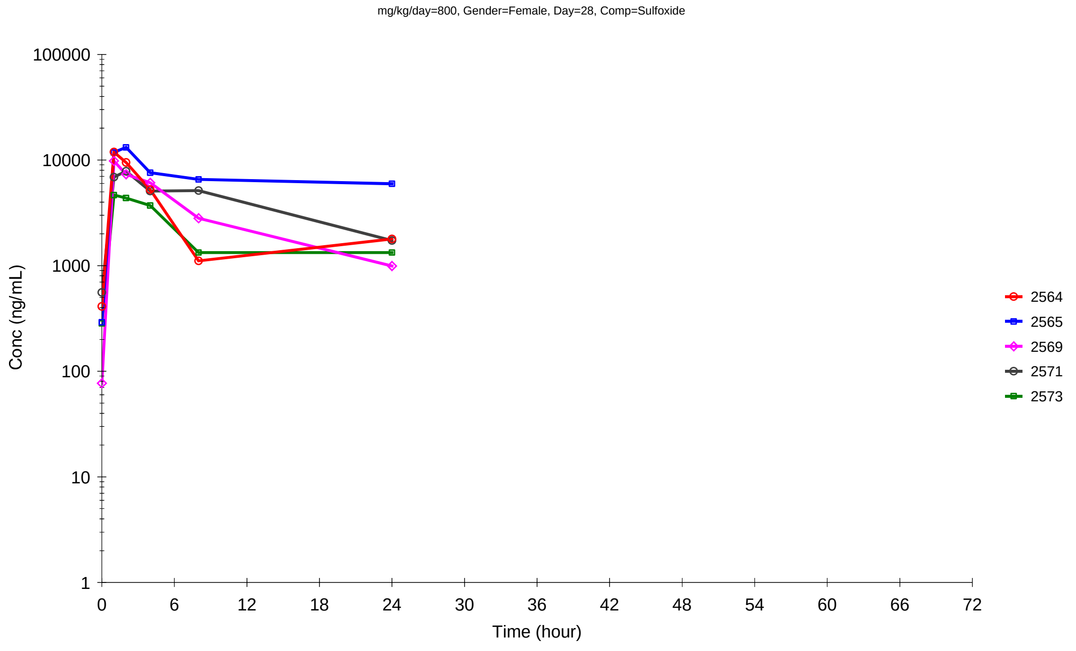

Figure 16. Day 1 mean (+SD) plasma concentrations (ng/mL) of Fexinidazole and metabolites after single and repeated oral 50, 200 and 800 mg/kg/day of Fexinidazole in male Beagle dogs.

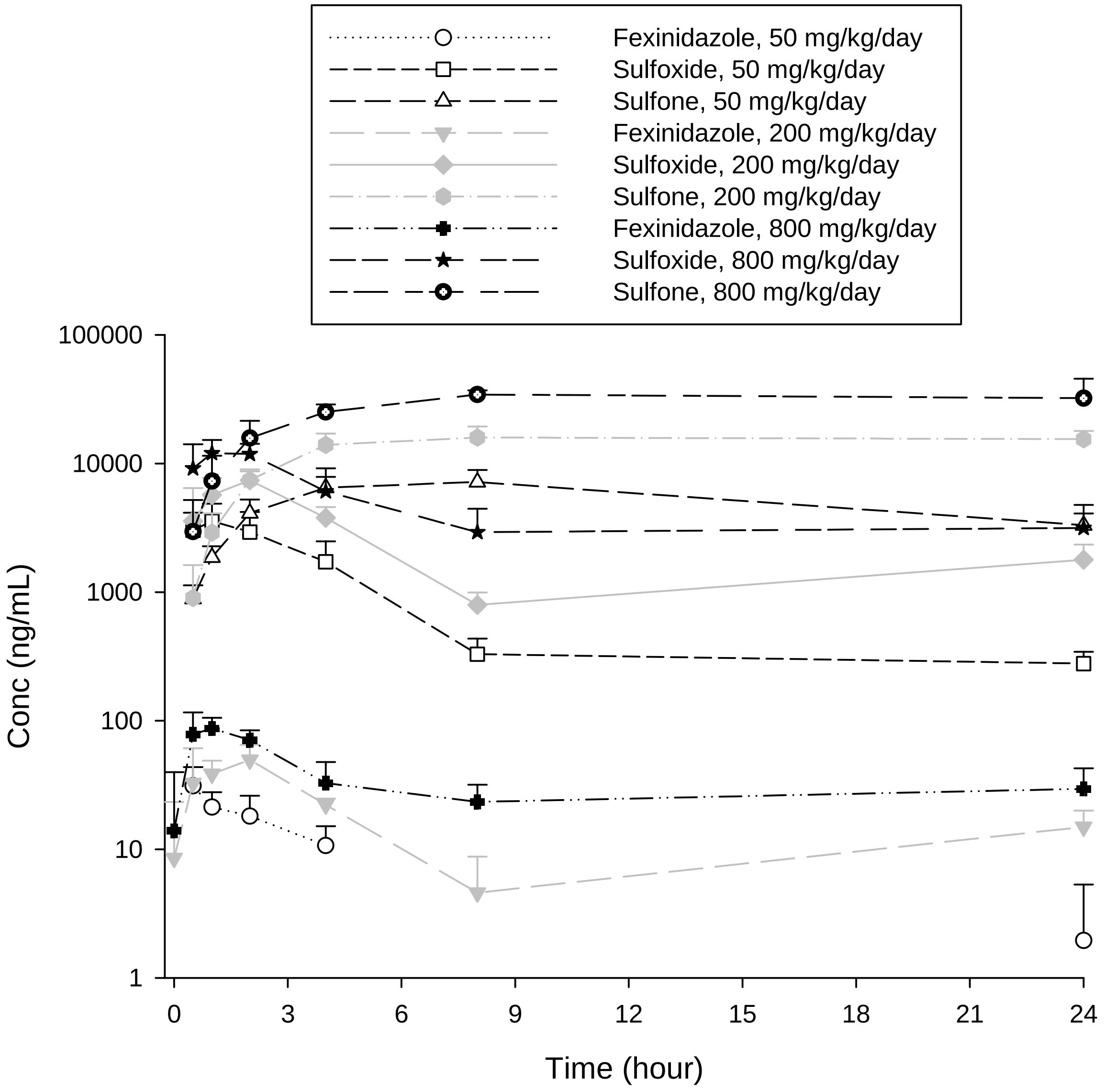

Figure 17. Day 1 mean (+SD) plasma concentrations (ng/mL) of Fexinidazole and metabolites after single and repeated oral 50, 200 and 800 mg/kg/day of Fexinidazole in female Beagle dogs.

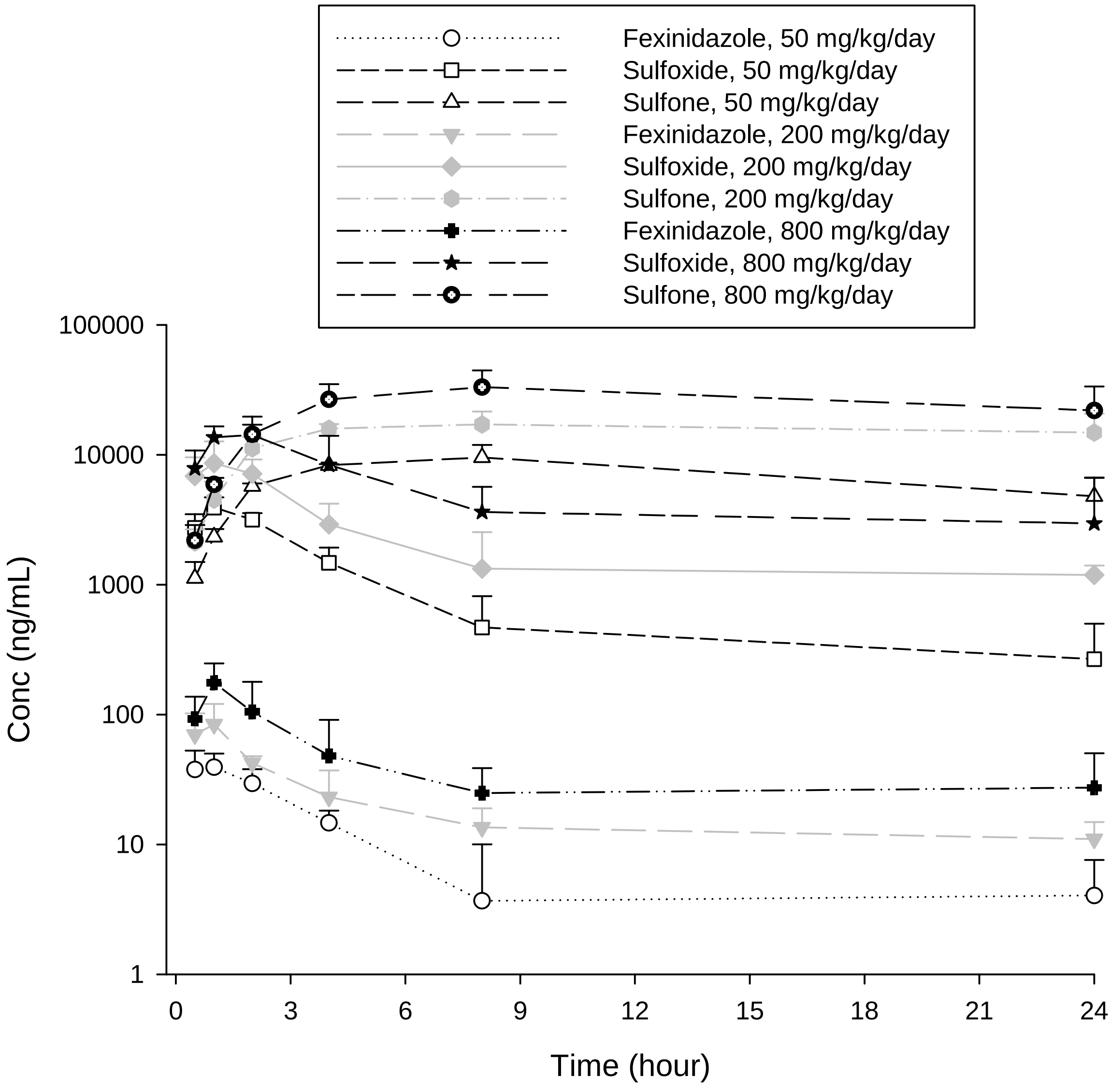

Figure 18. Day 14 mean (+SD) plasma concentrations (ng/mL) of Fexinidazole and metabolites after single and repeated oral 50, 200 and 800 mg/kg/day of Fexinidazole in male Beagle dogs.

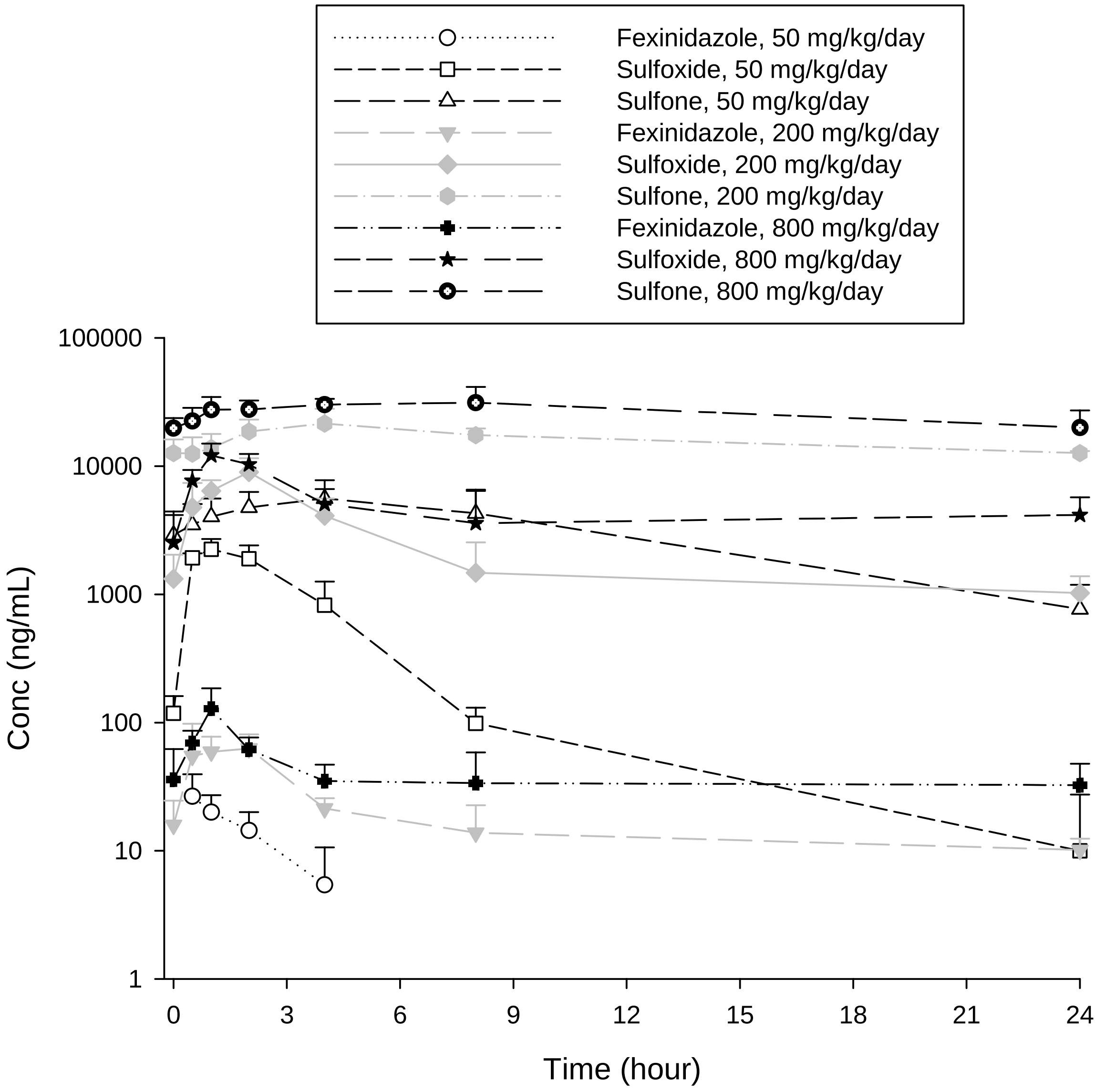

Figure 19. Day 14 mean (+SD) plasma concentrations (ng/mL) of Fexinidazole and metabolites after single and repeated oral 50, 200 and 800 mg/kg/day of Fexinidazole in female Beagle dogs.

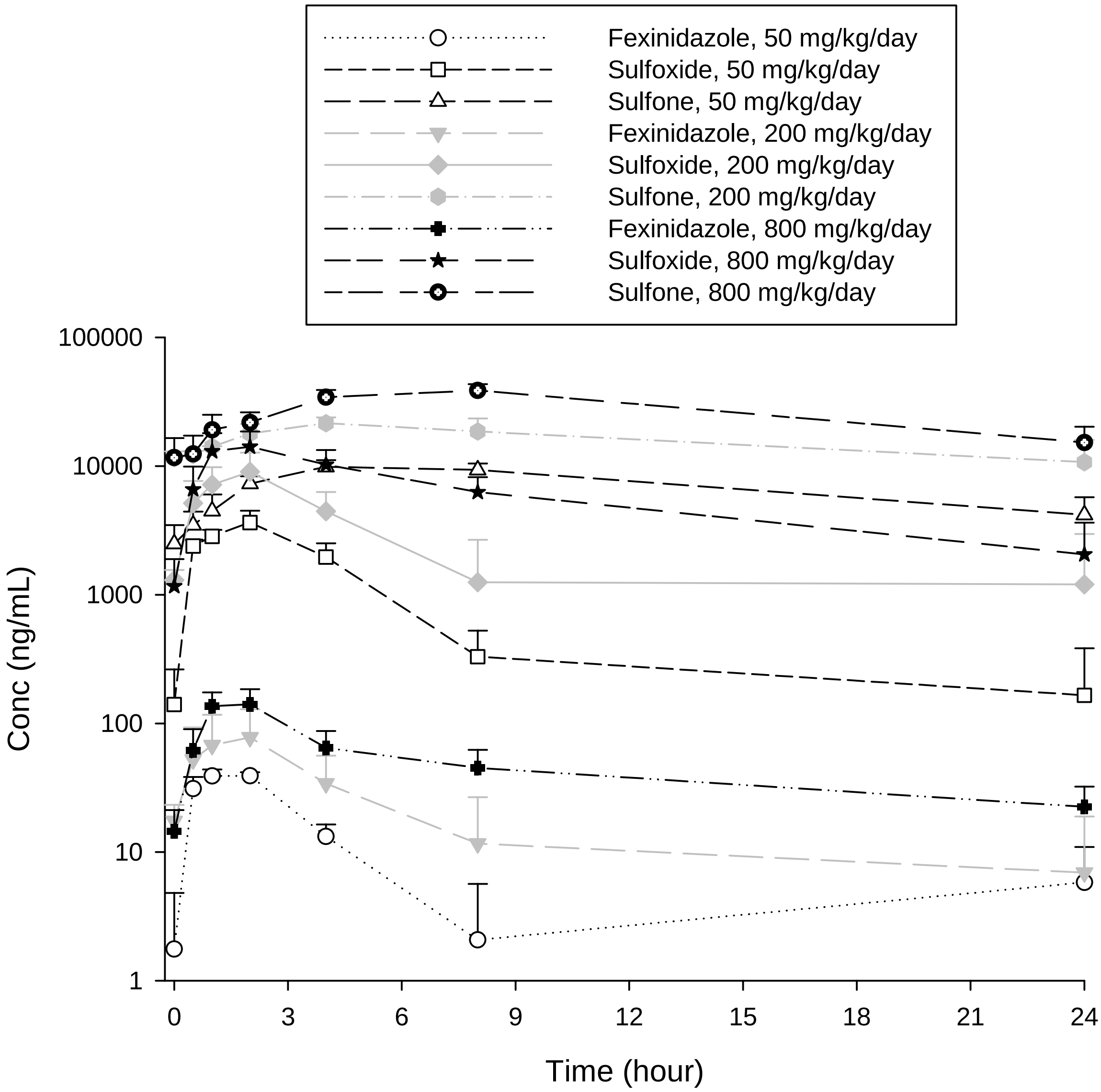

Figure 20. Day 28 mean (+SD) plasma concentrations (ng/mL) of Fexinidazole and metabolites after single and repeated oral 50, 200 and 800 mg/kg/day of Fexinidazole in male Beagle dogs.

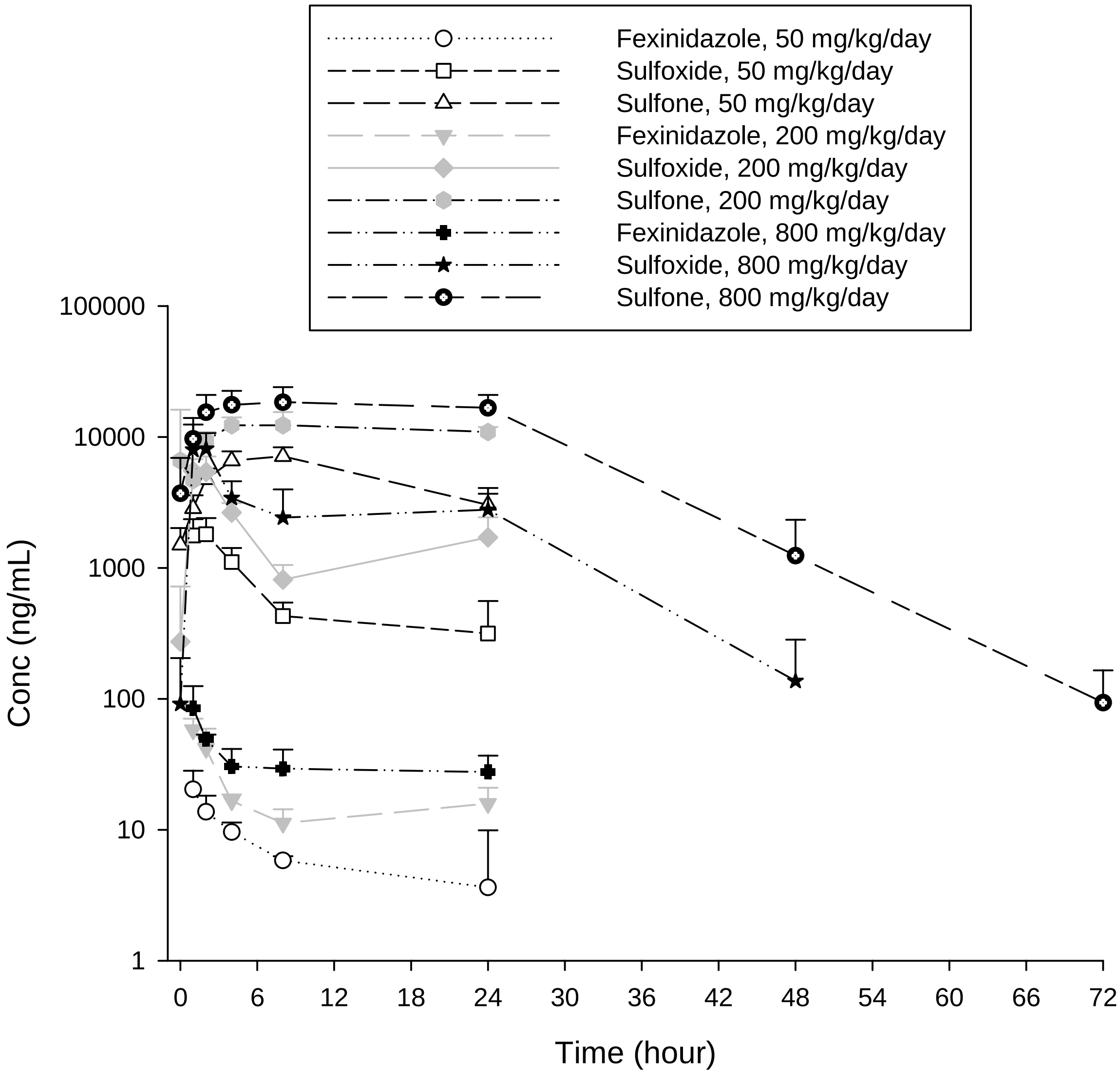

Figure 21. Day 28 mean (+SD) plasma concentrations (ng/mL) of Fexinidazole and metabolites after single and repeated oral 50, 200 and 800 mg/kg/day of Fexinidazole in female Beagle dogs.

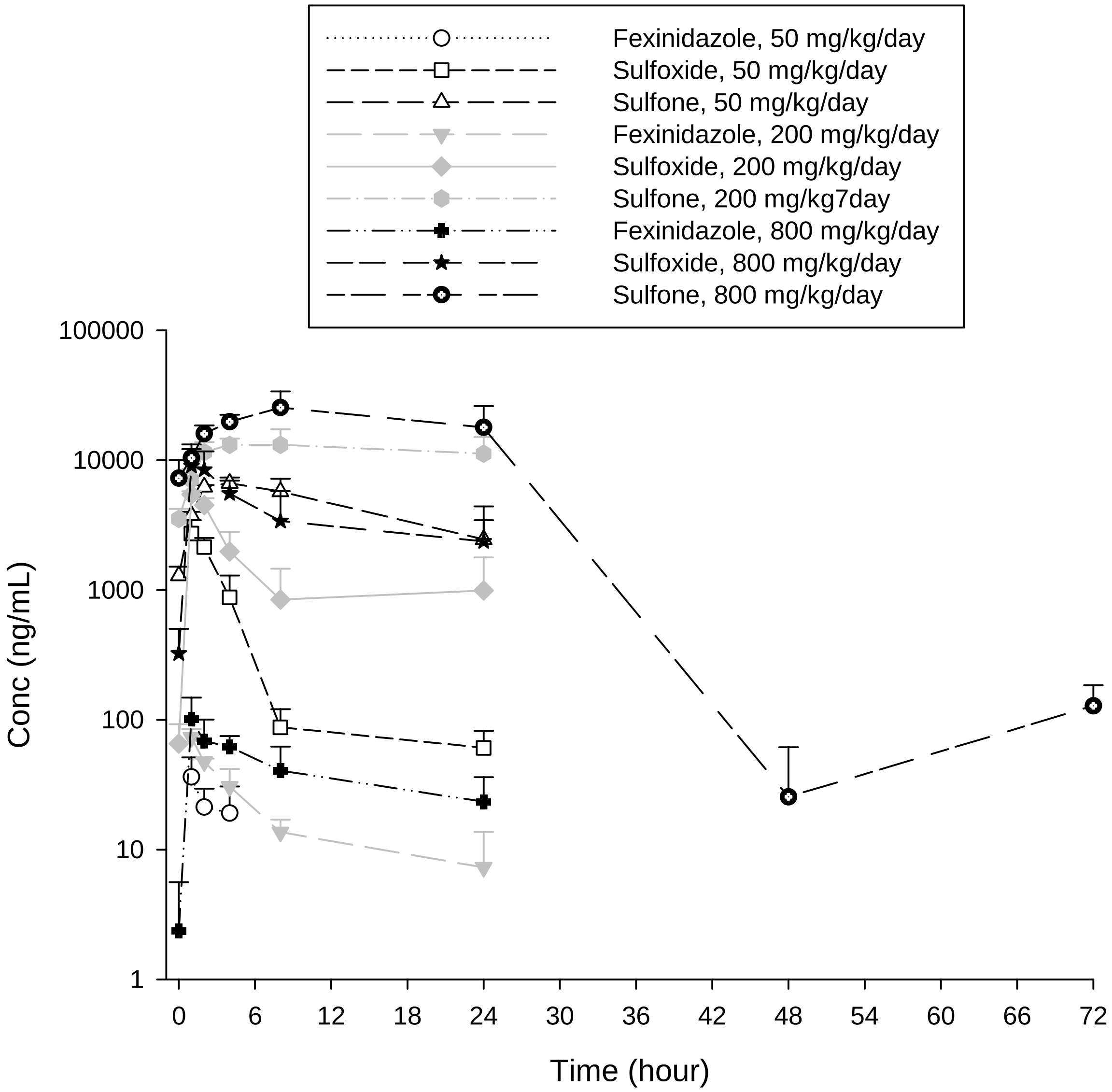

Figure 22. Mean (±SD) normalized C_{max} (upper panel) and $AUC_{0-t(last)}$ (lower panel) of Fexinidazole after single and repeated oral 50, 200 and 800 mg/kg/day of Fexinidazole in male Beagle dogs.

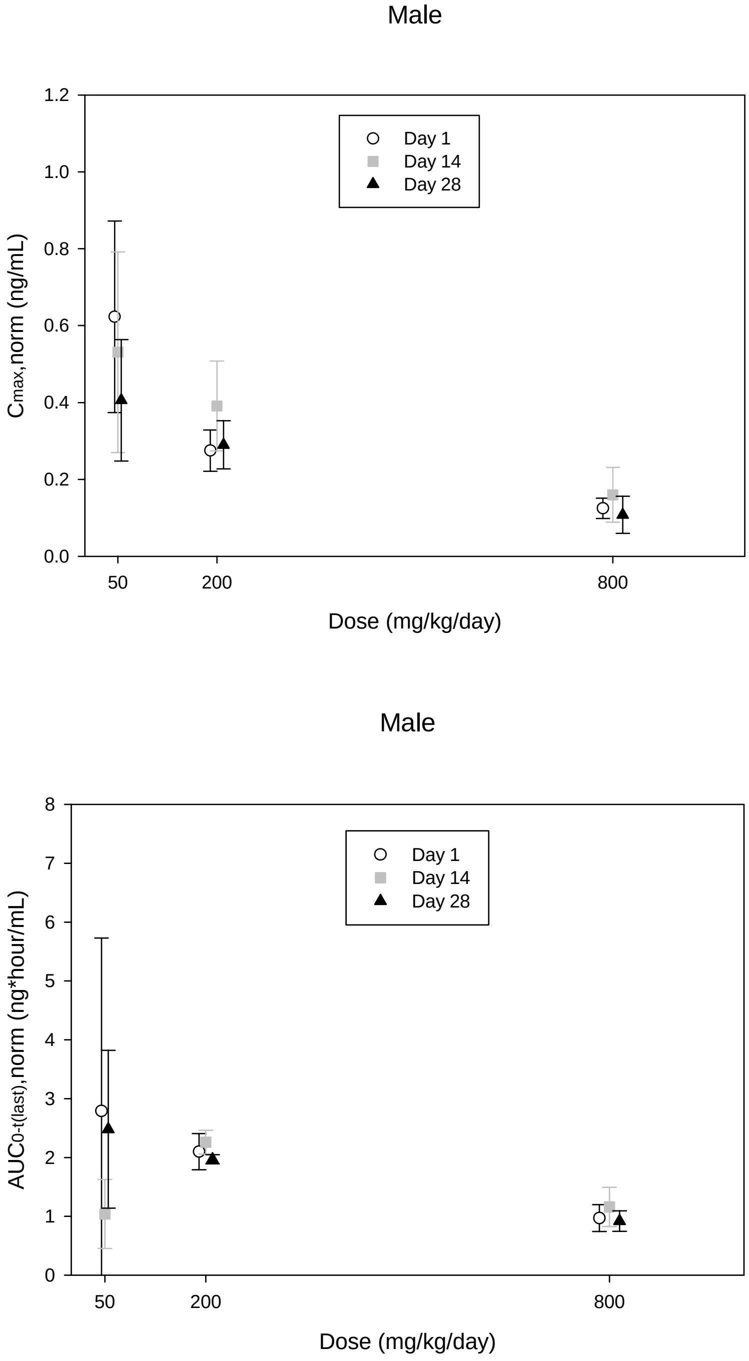

Figure 23. Mean (±SD) normalized C_{max} (upper panel) and $AUC_{0-t(last)}$ (lower panel) of Fexinidazole after single and repeated oral 50, 200 and 800 mg/kg/day of Fexinidazole in female Beagle dogs.

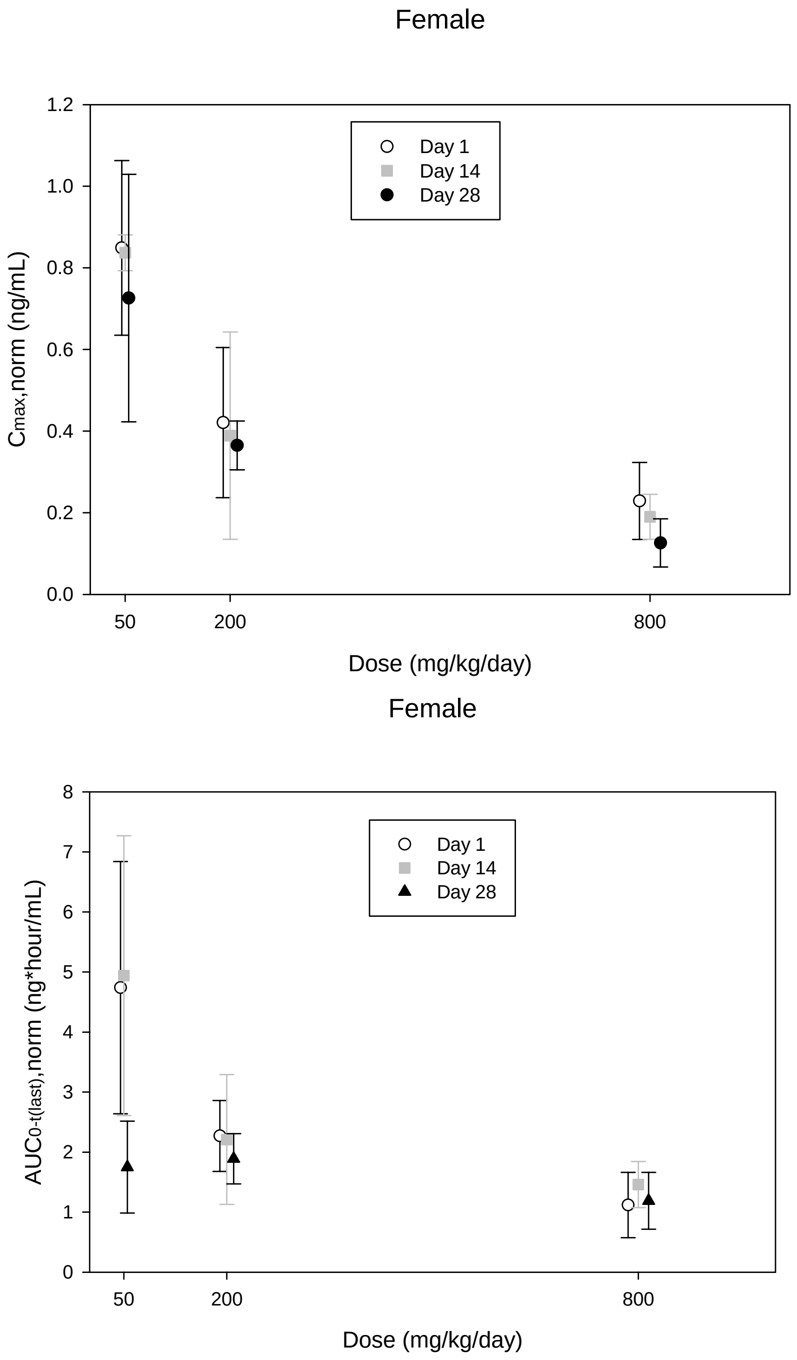

Figure 24. Mean (±SD) normalized C_{max} (upper panel) and $AUC_{0\text{-}t(last)}$ (lower panel) of sulfone derivative after single and repeated oral 50, 200 and 800 mg/kg/day of Fexinidazole in male Beagle dogs.

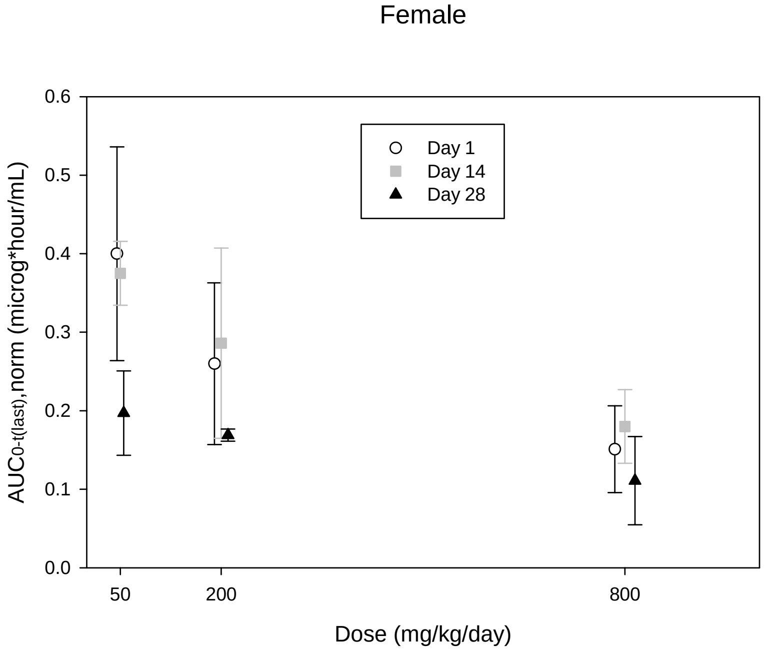

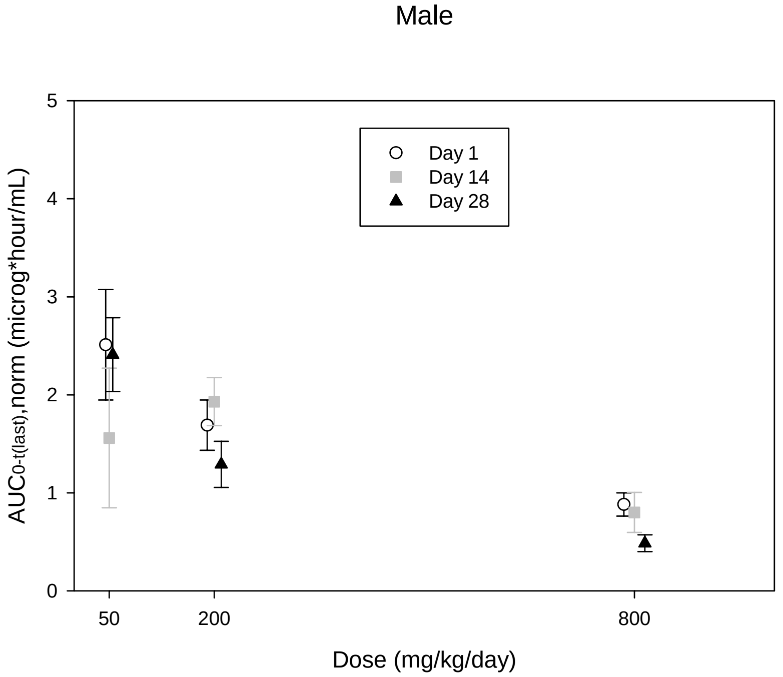

Figure 25. Mean (±SD) normalized C_{max} (upper panel) and $AUC_{0\text{-}t(last)}$ (lower panel) of sulfone derivative after single and repeated oral 50, 200 and 800 mg/kg/day of Fexinidazole in female Beagle dogs.

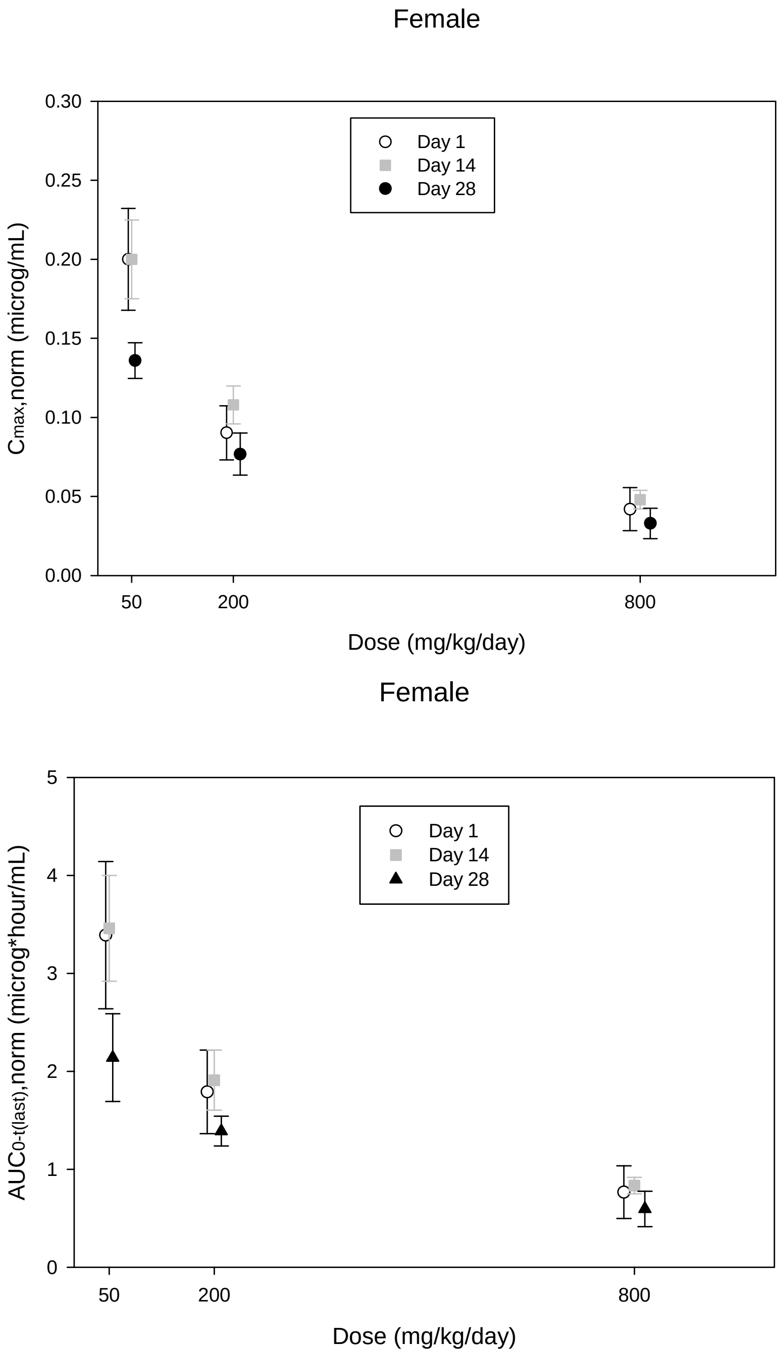

Figure 26. Mean (±SD) normalized C_{max} (upper panel) and $AUC_{0\text{-}t(last)}$ (lower panel) of sulfoxide derivative after single and repeated oral 50, 200 and 800 mg/kg/day of Fexinidazole in male Beagle dogs.

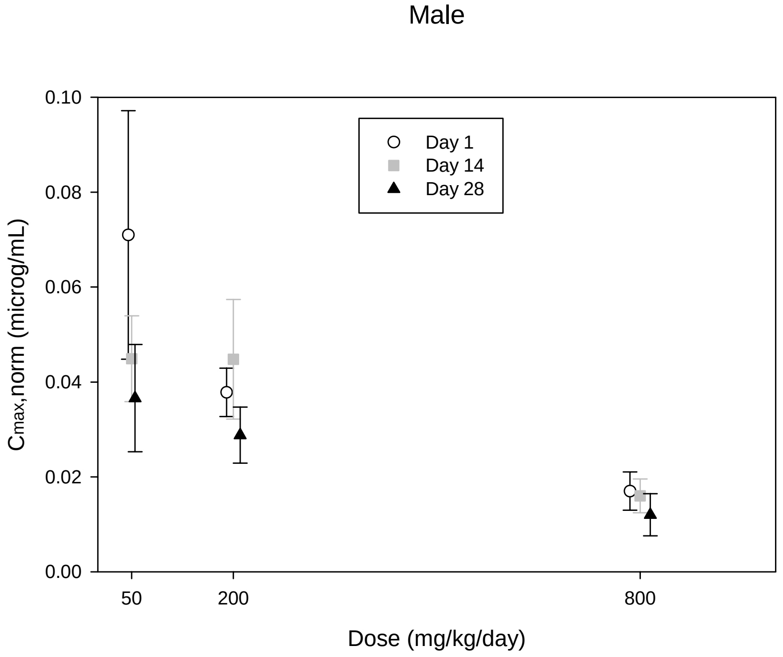

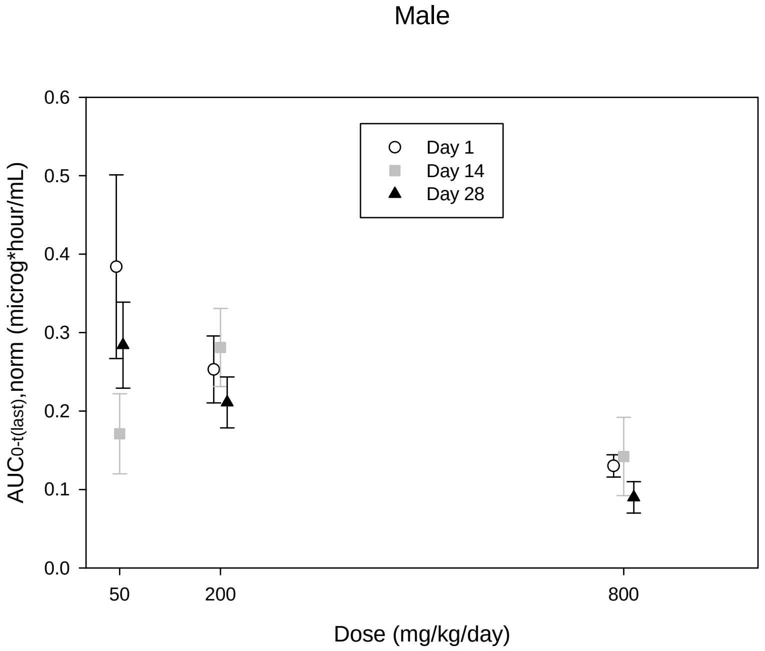

Figure 27. Mean (±SD) normalized C_{max} (upper panel) and $AUC_{0-t(last)}$ (lower panel) of sulfoxide derivative after single and repeated oral 50, 200 and 800 mg/kg/day of Fexinidazole in female Beagle dogs.

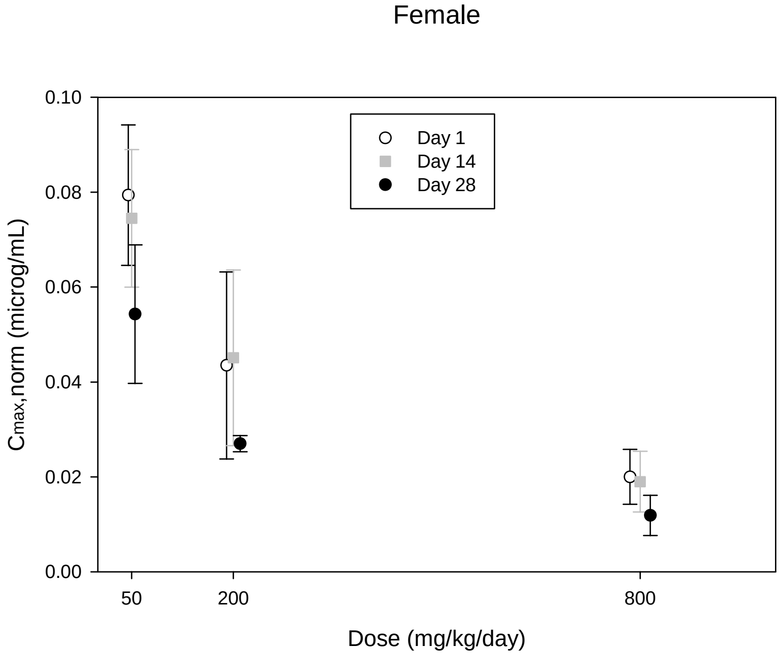

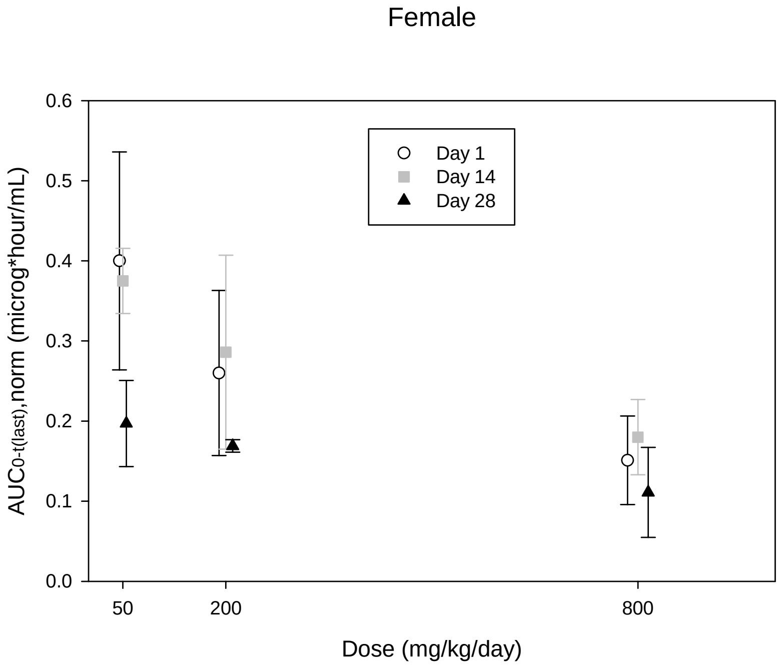

APPENDICES

Appendix 1. Individual plasma concentrations

Table 1A1. Individual plasma concentrations (ng/mL) of Fexinidazole after single (Day 1) and repeated (Day 14 and Day 28) oral administrations of the vehicle of Fexinidazole to male and female Beagle dogs.

Day 1										
Time	Dog ID									
(hour)	2516	2518	2520	2527	2533	2560	2568	2572	2575	2577
0	<5	<5	<5	<5	<5	<5	<5	<5	<5	<5
2	<5	<5	<5	<5	<5	<5	<5	<5	<5	<5

Day 14										
Time	Dog ID									
(hour)	2516	2518	2520	2527	2533	2560	2568	2572	2575	2577
0	<5	<5	<5	<5	<5	<5	<5	<5	<5	<5
2	<5	<5	<5	<5	<5	<5	<5	<5	<5	<5

Day 28										
Time	Dog ID									
(hour)	2516	2518	2520	2527	2533	2560	2568	2572	2575	2577
0	<5	<5	<5	<5	<5	<5	<5	<5	<5	<5
2	<5	<5	<5	<5	<5	<5	<5	<5	<5	<5

Table 2A1. Individual plasma concentrations (ng/mL) of sulfone metabolite after single (Day 1) and repeated (Day 14 and Day 28) oral administrations of the vehicle of Fexinidazole to male and female Beagle dogs.

Day 1										
Time	Dog ID									
(hour)	2516	2518	2520	2527	2533	2560	2568	2572	2575	2577
0	<25	<25	<25	<25	<25	<25	<25	<25	<25	<25
2	<25	<25	<25	<25	<25	<25	<25	<25	<25	<25

Day 14										
Time	Dog ID									
(hour)	2516	2518	2520	2527	2533	2560	2568	2572	2575	2577
0	<25	<25	<25	<25	<25	<25	<25	<25	<25	<25
2	<25	<25	<25	<25	<25	<25	<25	<25	<25	<25

Day 28										
Time	Dog ID									
(hour)	2516	2518	2520	2527	2533	2560	2568	2572	2575	2577
0	<25	<25	<25	<25	<25	<25	<25	<25	<25	<25
2	<25	<25	<25	<25	<25	<25	<25	<25	<25	<25

Table 3A1. Individual plasma concentrations (ng/mL) of sulfoxide metabolite after single (Day 1) and repeated (Day 14 and Day 28) oral administrations of the vehicle of Fexinidazole to male and female Beagle dogs.

Day 1										
Time	Dog ID									
(hour)	2516	2518	2520	2527	2533	2560	2568	2572	2575	2577
0	<25	<25	<25	<25	<25	<25	<25	<25	<25	<25
2	<25	<25	<25	<25	<25	<25	<25	<25	<25	<25

Day 14										
Time	Dog ID									
(hour)	2516	2518	2520	2527	2533	2560	2568	2572	2575	2577
0	<25	<25	<25	<25	<25	<25	<25	<25	<25	<25
2	<25	<25	<25	<25	<25	<25	<25	<25	<25	<25

Day 28										
Time	Dog ID									
(hour)	2516	2518	2520	2527	2533	2560	2568	2572	2575	2577
0	<25	<25	<25	<25	<25	<25	<25	<25	<25	<25
2	<25	<25	<25	<25	<25	<25	<25	<25	<25	<25

Table 4A1. Individual plasma concentrations (ng/mL) of Fexinidazole after single (Day 1) and repeated (Day 14 and Day 28) oral administrations of Fexinidazole at the dose of 50 mg/kg/day to male Beagle dogs.

Time (hour)	ID 2514	ID 2521	ID 2529	Mean	SD
	Day 1				
0	<5	<5	<5	N/A	N/A
0.5	45.5	23.1	24.9	31.2	12.4
1	28.3	20.0	15.6	21.3	6.45
2	26.0	18.1	10.1	18.1	7.95
4	15.2	10.4	6.38	10.7	4.42
8	<5	<5	<5	N/A	N/A
24	5.86	<5	<5	1.95*	3.38
	Day 14				
0	<5	<5	<5	N/A	N/A
0.5	41.4	21.1	17.1	26.5	13.0
1	28.2	16.6	15.2	20.0	7.14
2	20.6	13.0	9.70	14.4	5.59
4	10.4	5.84	<5	5.41	5.21
8	<5	<5	<5	N/A	N/A
24	<5	<5	<5	N/A	N/A
	Day 28				
0	<5	<5	<5	N/A	N/A
1	29.3	17.0	14.6	20.3	7.89
2	18.8	10.2	12.0	13.7	4.54
4	11.6	8.47	8.77	9.61	1.73
8	5.89	6.23	5.34	5.82	0.449
24	<5	10.9	<5	3.63*	6.29

Estimates of mean based on approximation that values below LLOQ are equal to zero. For values marked *, more than half of the individual levels were below LLOQ; descriptive statistics was reported even if strongly biased.

N/A: not applicable.

Table 5A1. Individual plasma concentrations (ng/mL) of Fexinidazole after single (Day 1) and repeated (Day 14 and Day 28) oral administrations of Fexinidazole at the dose of 50 mg/kg/day to female Beagle dogs.

Time (hour)	ID 2562	ID 2563	ID 2576	Mean	SD
	Day 1				
0	<5	<5	<5	N/A	N/A
0.5	24.8	54.3	34.1	37.7	15.1
1	35.7	51.4	30.5	39.2	10.9
2	38.8	27.4	22.3	29.5	8.45
4	18.6	11.6	13.5	14.6	3.62
8	11.0	<5	<5	3.67*	6.35
24	6.79	<5	5.32	4.04	3.57
	Day 14				
0	5.28	<5	<5	1.76*	3.05
0.5	37.6	32.5	23.3	31.1	7.25
1	44.4	35.3	37.2	39.0	4.80
2	36.1	40.8	40.4	39.1	2.61
4	9.57	14.4	15.5	13.2	3.15
8	6.20	<5	<5	2.07*	3.58
24	9.81	<5	7.58	5.80	5.14
	Day 28				
0	<5	<5	<5	N/A	N/A
1	23.5	53.0	32.4	36.3	15.1
2	18.0	30.6	15.2	21.3	8.20
4	12.4	32.5	12.3	19.1	11.6
8	<5	<5	<5	N/A	N/A
24	<5	<5	<5	N/A	N/A

Estimates of mean based on approximation that values below LLOQ are equal to zero. For values marked *, more than half of the individual levels were below LLOQ; descriptive statistics was reported even if strongly biased.

N/A: not applicable.

Table 6A1. Individual plasma concentrations (ng/mL) of Fexinidazole after single (Day 1) and repeated (Day 14 and Day 28) oral administrations of Fexinidazole at the dose of 200 mg/kg/day to male Beagle dogs.

Time (hour)	ID 2515	ID 2523	ID 2526	Mean	SD
	Day 1				
0	<5	<5	25.6[1]	N/A	N/A
0.5	46.4	<5	51.3	32.6	28.3
1	35.3	30.1	50.2	38.5	10.4
2	38.9	67.0	42.9	49.6	15.2
4	21.5	25.0	19.9	22.1	2.61
8	<5	5.71	8.09	4.60	4.16
24	12.2	11.8	20.8	14.9	5.08
	Day 14				
0	7.72	14.7	25.1	15.8	8.75
0.5	52.6	12.6	98.8	54.7	43.1
1	41.5	57.2	78.4	59.0	18.5
2	48.1	82.9	56.9	62.6	18.1
4	18.6	26.3	19.0	21.3	4.33
8	23.9	9.89	7.59	13.8	8.83
24	8.08	12.6	9.57	10.1	2.30
	Day 28				
0	<5	<5	<5	N/A	N/A
1	71.2	56.1	46.3	57.9	12.5
2	59.7	40.4	24.9	41.7	17.4
4	18.9	15.3	15.5	16.6	2.02
8	11.3	8.05	14.3	11.2	3.13
24	9.86	18.5	19.0	15.8	5.14

(1) Likely due to contamination.

Estimates of mean based on approximation that values below LLOQ are equal to zero.

N/A: not applicable.

Table 7A1. Individual plasma concentrations (ng/mL) of Fexinidazole after single (Day 1) and repeated (Day 14 and Day 28) oral administrations of Fexinidazole at the dose of 200 mg/kg/day to female Beagle dogs.

Time (hour)	ID 2561	ID 2570	ID 2567	Mean	SD
	Day 1				
0	<5	<5	<5	N/A	N/A
0.5	51.0	51.9	107	70.0	32.1
1	57.4	69.0	126	84.1	36.7
2	37.0	41.8	48.0	42.3	5.51
4	14.4	15.7	39.2	23.1	14.0
8	8.98	11.9	19.6	13.5	5.49
24	12.5	13.9	6.61	11.0	3.87
	Day 14				
0	11.0	18.8	22.5	17.4	5.87
0.5	27.4	30.1	99.0	52.2	40.6
1	40.3	37.9	124	67.4	49.0
2	42.9	54.2	136	77.7	50.8
4	23.3	19.1	59.5	34.0	22.2
8	5.99	<5	28.7	11.6	15.1
24	<5	20.7	<5	6.90*	12.0
	Day 28				
0	<5	<5	<5	N/A	N/A
1	84.0	60.2	74.7	73.0	12.0
2	44.9	50.4	47.0	47.4	2.78
4	33.1	18.1	40.3	30.5	11.3
8	17.6	11.8	11.5	13.6	3.44
24	<5	12.1	9.75	7.28	6.42

Estimates of mean based on approximation that values below LLOQ are equal to zero. For values marked *, more than half of the individual levels were below LLOQ; descriptive statistics was reported even if strongly biased.

N/A: not applicable.

Table 8A1. Individual plasma concentrations (ng/mL) of Fexinidazole after single (Day 1) and repeated (Day 14 and Day 28) oral administrations of Fexinidazole at the dose of 800 mg/kg/day to male Beagle dogs.

Time (hour)	ID 2517	ID 2519	ID 2525	ID 2528	ID 2530	Mean	SD
	Day 1						
0	<5	<5	9.75	59.5	<5	13.9*	25.9
0.5	132	73.8	47.8	97.0	40.0	78.1	37.6
1	86.0	108	60.4	98.8	81.5	86.9	18.2
2	55.6	85.2	80.8	74.1	56.4	70.4	13.7
4	15.5	51.8	25.5	44.6	25.5	32.6	15.0
8	15.5	14.2	24.6	34.3	27.8	23.3	8.46
24	26.8	9.99	37.2	44.9	28.5	29.5	13.1
	Day 14						
0	74.9	10.5	15.2	46.6	32.3	35.9	26.1
0.5	74.3	80.9	51.9	87.8	52.1	69.4	16.6
1	130	220	75.1	127	86.9	128	56.9
2	72.8	78.0	42.6	50.3	63.7	61.5	14.9
4	35.1	29.3	55.2	24.0	30.2	34.8	12.1
8	30.9	11.9	7.75	53.4	63.8	33.6	24.8
24	57.5	16.4	27.2	31.7	29.0	32.4	15.2
	Day 28						
0	<5	<5	<5	<5	<5	N/A	N/A
1	141	54.1	107	40.5	80.4	84.6	40.5
2	55.7	44.8	48.3	48.4	49.5	49.3	3.97
4	49.2	27.2	21.1	24.1	30.5	30.4	11.1
8	40.3	37.6	26.7	31.5	10.6	29.3	11.7
24	24.4	35.2	23.6	16.1	38.8	27.6	9.24
48				<5	<5	N/A	N/A
72				<5	<5	N/A	N/A

Estimates of mean based on approximation that values below LLOQ are equal to zero. For values marked *, more than half of the individual levels were below LLOQ; descriptive statistics was reported even if strongly biased.

N/A: not applicable.

Table 9A1. Individual plasma concentrations (ng/mL) of Fexinidazole after single (Day 1) and repeated (Day 14 and Day 28) oral administrations of Fexinidazole at the dose of 800 mg/kg/day to female Beagle dogs.

Time (hour)	ID 2564	ID 2565	ID 2569	ID 2571	ID 2573	Mean	SD
	Day 1						
0	<5	<5	<5	<5	<5	N/A	N/A
0.5	156	115	89.8	45.5	57.0	92.7	44.8
1	293	195	128	135	129	176	71.1
2	96.9	233	59.6	52.4	80.7	105	73.9
4	39.7	124	29.8	21.3	25.8	48.1	43.0
8	32.9	40.9	29.5	11.4	9.08	24.8	13.9
24	<5	30.1	54.1	44.4	8.04	27.3	23.1
	Day 14						
0	10.7	22.4	5.14	17.5	16.6	14.5	6.67
0.5	96.7	74.8	30.0	33.8	72.5	61.6	28.7
1	194	134	139	89.2	124	136	37.8
2	161	171	182	79.2	109	140	44.2
4	53.4	99.3	72.2	58.3	39.1	64.5	22.8
8	39.5	73.5	27.2	41.0	43.9	45.0	17.2
24	34.9	28.2	16.7	9.66	22.6	22.4	9.81
	Day 28						
0	<5	<5	<5	5.26	6.52	2.36*	3.26
1	94.7	179	102	69.5	59.0	101	47.1
2	55.2	125	50.1	62.4	50.7	68.7	31.9
4	58.2	82.3	66.0	49.0	53.0	61.7	13.2
8	14.5	61.3	64.4	27.8	34.7	40.5	21.6
24	25.3	44.5	18.7	15.6	12.2	23.3	12.8
48				<5	<5	N/A	N/A
72				<5	<5	N/A	N/A

Estimates of mean based on approximation that values below LLOQ are equal to zero. For values marked *, more than half of the individual levels were below LLOQ; descriptive statistics was reported even if strongly biased.

N/A: not applicable.

Table 10A1. Individual plasma concentrations (µg/mL) of sulfone metabolite after single (Day 1) and repeated (Day 14 and Day 28) oral administrations of Fexinidazole at the dose of 50 mg/kg/day to male Beagle dogs.

Time (hour)	ID 2514	ID 2521	ID 2529	Mean	SD
	Day 1				
0	<0.025	<0.025	<0.025	N/A	N/A
0.5	1.13	0.650	0.882	0.887	0.240
1	2.18	1.39	2.00	1.86	0.414
2	5.25	3.00	4.08	4.11	1.13
4	8.04	5.93	5.45	6.47	1.38
8	9.17	6.33	6.02	7.17	1.74
24	4.00	2.47	3.46	3.31	0.776
	Day 14				
0	4.48	2.75	1.46	2.90	1.52
0.5	5.16	3.24	2.07	3.49	1.56
1	5.77	3.54	2.78	4.03	1.55
2	6.46	4.21	3.57	4.75	1.52
4	8.09	4.44	4.18	5.57	2.19
8	6.60	3.80	2.40	4.27	2.14
24	1.17	0.791	0.336	0.766	0.418
	Day 28				
0	1.75	1.85	0.904	1.50	0.520
1	3.55	2.94	2.07	2.85	0.744
2	5.85	4.52	3.88	4.75	1.00
4	7.91	6.22	5.63	6.59	1.18
8	8.43	6.87	6.04	7.11	1.21
24	2.88	3.75	2.44	3.02	0.667
N/A: not applicable.					

Table 11A1. Individual plasma concentrations (µg/mL) of sulfone metabolite after single (Day 1) and repeated (Day 14 and Day 28) oral administrations of Fexinidazole at the dose of 50 mg/kg/day to female Beagle dogs.

Time (hour)	ID 2562	ID 2563	ID 2576	Mean	SD
	Day 1				
0	<0.025	<0.025	<0.025	N/A	N/A
0.5	0.688	1.26	1.40	1.12	0.377
1	2.13	2.74	2.13	2.33	0.352
2	5.74	6.02	5.43	5.73	0.295
4	8.15	8.32	8.45	8.31	0.150
8	11.5	6.89	10.2	9.53	2.38
24	6.07	2.69	5.64	4.80	1.84
	Day 14				
0	3.20	2.89	1.35	2.48	0.991
0.5	4.49	3.31	2.61	3.47	0.950
1	6.22	3.85	3.37	4.48	1.53
2	8.34	6.22	7.27	7.28	1.06
4	10.3	8.43	10.8	9.84	1.25
8	10.6	8.54	8.86	9.33	1.11
24	5.84	3.93	2.74	4.17	1.56
	Day 28				
0	1.46	1.37	1.04	1.29	0.221
1	3.58	4.04	3.66	3.76	0.246
2	6.01	6.18	6.44	6.21	0.217
4	6.50	7.43	6.03	6.65	0.712
8	4.04	6.75	6.36	5.72	1.47
24	1.56	3.53	2.26	2.45	0.999
N/A: not applicable.					

Table 12A1. Individual plasma concentrations (µg/mL) of sulfone metabolite after single (Day 1) and repeated (Day 14 and Day 28) oral administrations of Fexinidazole at the dose of 200 mg/kg/day to male Beagle dogs.

Time (hour)	ID 2515	ID 2523	ID 2526	Mean	SD
Day 1					
0	<0.025	<0.025	<0.025	N/A	N/A
0.5	1.30	0.0676	1.33	0.899	0.720
1	3.32	1.55	3.82	2.90	1.19
2	6.43	6.49	9.25	7.39	1.61
4	10.3	16.1	15.3	13.9	3.14
8	12.3	16.4	19.1	15.9	3.42
24	16.0	12.7	17.5	15.4	2.46
Day 14					
0	9.88	11.2	16.6	12.6	3.56
0.5	8.65	11.9	17.0	12.5	4.21
1	9.32	15.2	17.0	13.8	4.02
2	14.9	17.5	23.5	18.6	4.41
4	13.9	24.2	26.1	21.4	6.56
8	15.6	16.7	20.0	17.4	2.29
24	12.8	12.9	12.0	12.6	0.493
Day 28					
0	1.37	0.840	17.6	6.60	9.53
1	4.47	3.90	5.71	4.69	0.925
2	8.89	8.06	11.1	9.35	1.57
4	11.5	11.1	14.4	12.3	1.80
8	13.3	8.70	14.9	12.3	3.22
24	10.2	10.5	12.1	10.9	1.02
N/A: not applicable.					

Table 13A1. Individual plasma concentrations (µg/mL) of sulfone metabolite after single (Day 1) and repeated (Day 14 and Day 28) oral administrations of Fexinidazole at the dose of 200 mg/kg/day to female Beagle dogs.

Time (hour)	ID 2561	ID 2570	ID 2567	Mean	SD
Day 1					
0	<0.025	<0.025	<0.025	N/A	N/A
0.5	1.87	1.75	2.75	2.12	0.546
1	2.98	4.24	6.34	4.52	1.70
2	8.41	13.3	12.0	11.2	2.53
4	14.4	16.4	17.0	15.9	1.36
8	13.3	15.9	22.0	17.1	4.47
24	15.8	8.40	20.1	14.8	5.92
Day 14					
0	13.2	10.5	10.8	11.5	1.48
0.5	12.5	11.2	12.9	12.2	0.889
1	14.1	14.0	14.3	14.1	0.153
2	15.4	16.6	21.6	17.9	3.29
4	19.3	21.2	24.0	21.5	2.36
8	15.1	16.3	24.1	18.5	4.89
24	7.52	16.7	7.79	10.7	5.22
Day 28					
0	2.75	4.04	3.79	3.53	0.684
1	8.58	5.69	7.00	7.09	1.45
2	14.0	9.56	10.9	11.5	2.28
4	14.8	11.8	12.6	13.1	1.55
8	17.9	9.94	11.6	13.1	4.20
24	8.18	15.6	9.94	11.2	3.88

N/A: not applicable.

Table 14A1. Individual plasma concentrations (µg/mL) of sulfone metabolite after single (Day 1) and repeated (Day 14 and Day 28) oral administrations of Fexinidazole at the dose of 800 mg/kg/day to male Beagle dogs.

Time (hour)	ID 2517	ID 2519	ID 2525	ID 2528	ID 2530	Mean	SD
Day 1							
0	<0.025	<0.025	<0.025	<0.025	<0.025	N/A	N/A
0.5	6.69	1.53	1.73	3.38	1.40	2.95	2.24
1	14.5	4.86	5.62	7.18	4.31	7.29	4.17
2	25.3	12.3	12.7	16.8	11.5	15.7	5.74
4	30.3	25.2	23.8	25.8	20.4	25.1	3.58
8	30.8	37.2	33.8	33.0	36.5	34.3	2.62
24	39.8	9.17	43.0	36.4	31.9	32.1	13.4
Day 14							
0	18.9	13.5	19.8	23.6	22.5	19.7	3.94
0.5	25.2	15.3	18.0	30.5	23.1	22.4	5.99
1	30.2	21.4	22.1	38.4	25.4	27.5	7.02
2	25.6	21.1	28.2	34.2	29.0	27.6	4.80
4	33.8	25.3	29.8	29.2	32.5	30.1	3.29
8	27.2	21.4	23.5	42.5	41.5	31.2	10.1
24	27.9	10.3	14.6	24.1	22.4	19.9	7.21
Day 28							
0	0.978	1.06	7.36	2.08	7.06	3.71	3.23
1	10.6	2.91	14.1	12.2	8.46	9.65	4.30
2	21.2	7.27	13.7	19.7	14.9	15.4	5.51
4	19.3	8.92	20.0	20.7	19.3	17.6	4.91
8	19.7	12.7	17.0	27.3	15.3	18.4	5.59
24	21.4	16.0	19.3	10.2	16.6	16.7	4.23
48				0.475	2.01	1.24	1.09
72				0.0428	0.144	0.0934	0.0716

N/A: not applicable.

Table 15A1. Individual plasma concentrations (μg/mL) of sulfone metabolite after single (Day 1) and repeated (Day 14 and Day 28) oral administrations of Fexinidazole at the dose of 800 mg/kg/day to female Beagle dogs.

Time (hour)	ID 2564	ID 2565	ID 2569	ID 2571	ID 2573	Mean	SD
Day 1							
0	<0.025	<0.025	<0.025	<0.025	<0.025	N/A	N/A
0.5	2.67	2.43	2.90	1.31	1.62	2.19	0.688
1	6.45	5.78	6.86	5.09	5.41	5.92	0.730
2	16.5	16.9	14.8	10.2	13.7	14.4	2.69
4	29.2	38.7	27.8	18.0	20.0	26.7	8.25
8	35.0	44.9	43.1	21.7	21.1	33.2	11.4
24	15.4	31.5	33.5	23.8	5.44	21.9	11.7
Day 14							
0	11.9	13.9	3.20	15.2	13.8	11.6	4.84
0.5	14.6	13.8	4.02	16.5	12.9	12.4	4.85
1	24.9	20.7	9.46	22.0	18.2	19.1	5.88
2	27.3	21.5	15.4	23.2	21.8	21.8	4.28
4	36.9	41.0	32.0	32.0	29.0	34.2	4.75
8	37.3	47.0	36.1	37.2	35.6	38.6	4.73
24	17.8	14.3	18.9	6.89	18.2	15.2	4.98
Day 28							
0	7.38	4.88	10.9	4.30	8.83	7.26	2.75
1	11.7	9.22	14.3	6.83	9.70	10.4	2.81
2	16.4	18.1	18.6	13.0	13.7	16.0	2.53
4	18.2	22.4	22.7	17.5	17.5	19.7	2.66
8	15.4	36.2	28.6	28.5	18.3	25.4	8.47
24	14.6	31.2	16.6	18.2	8.97	17.9	8.21
48				<0.025	0.0510	0.0255	0.0361
72				0.168	0.0883	0.128	0.0564

Estimates of mean based on approximation that values below LLOQ (0.025 μg/mL) are equal to zero.

N/A: not applicable.

Table 16A1. Individual plasma concentrations (µg/mL) of sulfoxide metabolite after single (Day 1) and repeated (Day 14 and Day 28) oral administrations of Fexinidazole at the dose of 50 mg/kg/day to male Beagle dogs.

Time (hour)	ID 2514	ID 2521	ID 2529	Mean	SD
Day 1					
0	<0.025	<0.025	<0.025	N/A	N/A
0.5	4.3	2.21	2.54	3.02	1.12
1	5.04	2.65	2.95	3.55	1.3
2	4.36	2.45	1.97	2.93	1.26
4	2.51	1.64	1.01	1.72	0.753
8	0.375	0.402	0.207	0.328	0.106
24	0.241	0.239	0.353	0.278	0.065
Day 14					
0	0.137	0.148	0.0686	0.118	0.043
0.5	2.11	1.84	1.8	1.92	0.169
1	2.76	2.06	1.91	2.24	0.454
2	2.36	1.96	1.34	1.89	0.514
4	1.29	0.745	0.432	0.822	0.434
8	0.129	0.101	0.0654	0.099	0.032
24	<0.025	0.0301	<0.025	0.01*	0.017
Day 28					
0	<0.025	<0.025	<0.025	N/A	N/A
1	2.44	1.44	1.42	1.77	0.58
2	2.48	1.37	1.57	1.81	0.59
4	1.44	0.826	1.05	1.11	0.31
8	0.395	0.557	0.332	0.428	0.12
24	0.222	0.593	0.132	0.316	0.24

Estimates of mean based on approximation that values below LLOQ (0.025 µg/mL) are equal to zero. For values marked *, more than half of the individual levels were below LLOQ; descriptive statistics was reported even if strongly biased.

N/A: not applicable.

Table 17A1. Individual plasma concentrations (μg/mL) of sulfoxide after single (Day 1) and repeated (Day 14 and Day 28) oral administrations of Fexinidazole at the dose of 50 mg/kg/day to female Beagle dogs.

Time (hour)	ID 2562	ID 2563	ID 2576	Mean	SD
Day 1					
0	<0.025	<0.025	<0.025	N/A	N/A
0.5	1.89	3.25	3.09	2.74	0.74
1	3.45	4.82	3.49	3.92	0.78
2	3.6	2.86	3.06	3.17	0.38
4	1.91	0.991	1.5	1.47	0.46
8	0.813	0.115	0.476	0.468	0.35
24	0.426	<0.025	0.376	0.267	0.23
Day 14					
0	0.191	0.229	<0.025	0.14	0.12
0.5	2.72	2.27	2.19	2.39	0.29
1	3.12	2.45	2.95	2.84	0.35
2	2.85	3.53	4.53	3.64	0.85
4	1.4	1.98	2.49	1.96	0.55
8	0.554	0.228	0.204	0.329	0.2
24	0.413	0.0831	<0.025	0.165	0.22
Day 28					
0	<0.025	<0.025	<0.025	N/A	N/A
1	1.91	2.91	3.33	2.72	0.729
2	1.7	2.39	2.32	2.14	0.38
4	0.554	1.35	0.728	0.877	0.418
8	0.0493	0.113	0.0999	0.087	0.034
24	0.0439	0.0851	0.0532	0.061	0.022

Estimates of mean based on approximation that values below LLOQ (0.025 μg/mL) are equal to zero.

N/A: not applicable.

Table 18A1. Individual plasma concentrations (μg/mL) of sulfoxide metabolite after single (Day 1) and repeated (Day 14 and Day 28) oral administrations of Fexinidazole at the dose of 200 mg/kg/day to male Beagle dogs.

Time (hour)	ID 2515	ID 2523	ID 2526	Mean	SD
Day 1					
0	<0.025	<0.025	<0.025	N/A	N/A
0.5	5.43	0.244	4.99	3.55	2.88
1	6.37	3.42	7.27	5.69	2.01
2	5.95	7.97	8.3	7.41	1.27
4	3.05	4.62	3.67	3.78	0.79
8	0.591	0.802	0.988	0.794	0.2
24	1.63	1.31	2.4	1.78	0.56
Day 14					
0	0.494	1.73	1.73	1.32	0.71
0.5	4.8	2.15	7.35	4.77	2.6
1	5.03	6.5	7.71	6.41	1.34
2	6.97	11.8	8.1	8.96	2.53
4	2.77	5.71	3.8	4.09	1.49
8	2.63	1.27	0.518	1.47	1.07
24	0.761	1.44	0.871	1.02	0.36
Day 28					
0	0.0287	<0.025	0.79	0.273	0.45
1	6.96	4.93	4.62	5.5	1.27
2	6.94	5.7	3.59	5.41	1.69
4	3.03	2.78	2.1	2.64	0.48
8	1.09	0.698	0.651	0.813	0.24
24	1.05	2.48	1.6	1.71	0.72

Estimates of mean based on approximation that values below LLOQ (0.025 μg/mL) are equal to zero.

N/A: not applicable.

Table 19A1. Individual plasma concentrations (µg/mL) of sulfoxide metabolite after single (Day 1) and repeated (Day 14 and Day 28) oral administrations of Fexinidazole at the dose of 200 mg/kg/day to female Beagle dogs.

Time (hour)	ID 2561	ID 2570	ID 2567	Mean	SD
Day 1					
0	<0.025	<0.025	<0.025	N/A	N/A
0.5	4.92	5.67	9.97	6.85	2.73
1	4.64	8.38	12.8	8.61	4.08
2	4.76	8.13	8.5	7.13	2.06
4	2.24	2.07	4.41	2.91	1.3
8	0.816	0.458	2.71	1.33	1.21
24	1.43	1.11	1.02	1.19	0.22
Day 14					
0	1.13	1.17	1.59	1.3	0.26
0.5	2.96	4.57	7.9	5.14	2.52
1	4.46	7.35	9.69	7.17	2.62
2	5.32	9.03	12.7	9.02	3.69
4	3.7	3.13	6.53	4.45	1.82
8	0.577	0.29	2.88	1.25	1.42
24	0.223	3.24	0.131	1.2	1.77
Day 28					
0	0.0354	0.0876	0.0735	0.066	0.027
1	5.41	5.1	5.78	5.43	0.34
2	3.9	4.58	5.06	4.51	0.583
4	1.54	1.46	2.93	1.98	0.827
8	1.55	0.555	0.428	0.844	0.614
24	0.156	1.73	1.09	0.992	0.792

N/A: not applicable.

Table 20A1. Individual plasma concentrations (µg/mL) of sulfoxide metabolite after single (Day 1) and repeated (Day 14 and Day 28) oral administrations of Fexinidazole at the dose of 800 mg/kg/day to male Beagle dogs.

Time (hour)	ID 2517	ID 2519	ID 2525	ID 2528	ID 2530	Mean	SD
Day 1							
0	<0.025	<0.025	<0.025	<0.025	<0.025	N/A	N/A
0.5	17.5	7.86	4.49	8.92	6.94	9.14	5
1	15.4	13.4	7.39	10.1	13.7	12	3.2
2	10.1	15.2	9.39	11.1	13.2	11.8	2.4
4	2.9	11.3	5.04	5.7	5.32	6.05	3.1
8	0.869	1.94	3.19	4.54	4.08	2.92	1.5
24	4.61	0.616	4.52	2.86	3.12	3.15	1.6
Day 14							
0	1.43	0.891	1.73	4.14	4.39	2.52	1.6
0.5	8.33	7.49	4.94	9.31	8.29	7.67	1.7
1	12.8	14.9	7.56	13.9	11.4	12.1	2.9
2	9.8	11.5	7.48	9.63	13.1	10.3	2.1
4	5.36	5.9	2.77	4.42	6.86	5.06	1.6
8	2.23	1.28	1.07	7.79	5.51	3.58	3
24	6.06	1.83	3.78	4.78	4.31	4.15	1.6
Day 28							
0	<25	0.0845	0.0566	0.0296	0.287	0.092	0.11
1	15.1	3.96	8.2	4.45	8.16	7.97	4.46
2	11.5	6.19	5.63	7.04	10.2	8.11	2.59
4	3.3	2.25	2.29	4.71	4.53	3.42	1.18
8	2.82	1.68	1.49	4.98	1.15	2.42	1.56
24	2.43	3.17	2.65	1.02	4.63	2.78	1.3
48				0.0339	0.24	0.137	0.15
72				<0.025	<0.025	N/A	N/A

Estimates of mean based on approximation that values below LLOQ (0.025 µg/mL) are equal to zero.

N/A: not applicable.

Table 21A1. Individual plasma concentrations (µg/mL) of sulfoxide metabolite after single (Day 1) and repeated (Day 14 and Day 28) oral administrations of Fexinidazole at the dose of 800 mg/kg/day to female Beagle dogs.

Time (hour)	ID 2564	ID 2565	ID 2569	ID 2571	ID 2573	Mean	SD
Day 1							
0	<0.025	<0.025	<0.025	<0.025	<0.025	N/A	N/A
0.5	11.7	7.61	9.88	4.85	5.15	7.84	2.97
1	17.4	12.8	13.8	14.7	9.22	13.6	2.98
2	18.4	21.7	10.5	10.9	9.51	14.2	5.49
4	9.91	17.6	4.72	5.20	4.40	8.37	5.63
8	5.49	5.67	3.92	1.54	1.47	3.62	2.05
24	0.441	2.14	2.57	9.37	0.292	2.96	3.72
Day 14							
0	1.10	1.59	0.0525	1.99	1.05	1.16	0.727
0.5	12.0	6.74	3.17	5.52	5.48	6.58	3.29
1	21.7	11.8	12.3	9.64	9.73	13.0	4.99
2	19.2	13.2	18.2	10.5	9.43	14.1	4.43
4	9.61	14.1	12.3	8.90	6.08	10.2	3.11
8	6.34	9.39	4.16	5.78	5.62	6.26	1.93
24	4.36	2.71	1.17	0.264	1.77	2.05	1.57
Day 28							
0	0.411	0.291	0.0769	0.558	0.284	0.324	0.178
1	11.9	11.8	9.80	6.89	4.66	9.01	3.17
2	9.49	13.2	7.32	7.83	4.37	8.44	3.24
4	5.24	7.58	6.09	5.08	3.71	5.54	1.42
8	1.11	6.56	2.81	5.14	1.33	3.39	2.39
24	1.79	5.96	0.992	1.73	1.33	2.36	2.04
48				<0.025	<0.025	N/A	N/A
72				<0.025	<0.025	N/A	N/A
N/A: not applicable.							

Appendix 2. In-Study Bioanalytical Validation Data

Calibration data

Table 1A2. Analytical Performance: Back-Calculated Concentrations (ng/mL) of Fexinidazole Calibration Standard in Dog Plasma for Study Protocol 0505-2007.

Assay Date	Analytical Run Number	STD.1 5.00 ng/mL	STD.2 10.0 ng/mL	STD.3 50.0 ng/mL	STD.4 100 ng/mL	STD.5 250 ng/mL	STD.6 500 ng/mL	STD.7 900 ng/mL	STD.8 1000 ng/mL
12-Mar-2008	1	4.68	10.9	*40.5	99.3	258	479	933	981
		4.94	10.9	44.6	97.7	245	464	970	1040
13-Mar-2008	2	5.45	9.34	50.7	93.6	238	495	897	1000
		4.47	10.8	53.9	102	254	492	868	1040
13-Mar-2008	3	4.5	9.69	46.6	92.5	272	495	869	1060
		5.77	9.4	50.3	97.5	257	499	950	1020
14-Mar-2008	4	4.98	10.4	46.7	89.7	250	480	896	998
		5.03	9.88	44.7	113	264	540	*1100	1030
21-Mar-2008	5	4.92	9.98	52.3	95.5	267	515	901	991
		5.2	9.73	45.8	90.4	250	472	930	1120
26-Mar-2008	6	5.33	10.9	49	94.1	244	524	872	1060
		4.2	11	53.9	96.8	234	509	816	1030
26-Mar-2008	7	5.01	*12.1	46.9	108	*201	497	1000	997
		5.12	9.69	45.4	97.2	256	515	813	1040
27-Mar-2008	8	5	10	44.8	103	257	554	1010	883
		4.76	11	55	87.4	258	457	900	951
27-Mar-2008	9	4.63	9.59	46.1	96.3	267	429	970	912
		5.48	10.2	48.7	97.8	267	515	956	1080
28-Mar-2008	10	4.77	10.6	48.3	101	253	491	963	1030
		5.08	10.1	50.9	90.9	255	508	948	877
29-Mar-2008	11	4.81	10.7	50.9	85.5	260	528	916	1150
		5.23	9.49	45.9	88.4	261	487	852	1070
Mean		4.97	10.2	48.6	96.3	256	498	916	1020
SD		0.365	0.581	3.29	6.63	9.72	28.2	55	67.9
%CV		7.3	5.7	6.8	6.9	3.8	5.7	6	6.7
%Bias		-0.6	2	-2.8	-3.7	2.4	-0.4	1.8	2
n		22	21	21	22	21	22	21	22

*Accuracy more than 15%; excluded from regression analysis.

Table 2A2. Analytical Performance: Back-Calculated Concentrations (ng/mL) of Sulfone Calibration Standard in Dog Plasma for Study Protocol 0505-2007.

Assay Date	Analytical Run Number	STD.1 25.0 ng/mL	STD.2 50.0 ng/mL	STD.3 250 ng/mL	STD.4 500 ng/mL	STD.5 2500 ng/mL	STD.6 5000 ng/mL	STD.7 22500 ng/mL	STD.8 25000 ng/mL
12-Mar-2008	1	28.5	*66.6	241	*616	*3130	5630	*27100	25900
		21.9	49	236	511	2630	4830	21300	23700
13-Mar-2008	2	27.1	52.1	266	519	2460	5360	19800	22200
		21.1	52.8	273	538	2690	5110	19700	22600
13-Mar-2008	3	25.1	51.5	237	478	2820	5000	20100	22500
		25.1	46.5	279	524	2810	5220	21400	23200
14-Mar-2008	4	23.2	56.5	236	469	2850	5010	21400	23600
		26	48.8	218	*668	2810	*6060	25000	22500
21-Mar-2008	5	24.9	50.2	267	484	2700	5280	19800	23600
		26.1	44.2	278	488	2610	4990	21000	25400
26-Mar-2008	6	23.7	56.7	269	493	2650	5290	19300	23100
		24.4	*59.3	*301	506	2490	5350	*18300	22400
26-Mar-2008	7	22.2	54.7	239	557	*1990	4970	20400	*20600
		26.1	52.4	240	506	2680	5130	*17600	22100
27-Mar-2008	8	22	50.2	232	539	2730	5640	22100	*19400
		26.6	55.8	*306	470	2730	4860	19500	21400
27-Mar-2008	9	23.5	49.5	236	492	2830	4680	21800	*19700
		26.6	*68.7	260	508	2830	5280	20200	22300
28-Mar-2008	10	25.2	51.2	252	541	2670	5040	20600	21300
		24.4	49.2	269	483	2680	5250	20500	*19300
29-Mar-2008	11	**14.9	*39.1	249	433	2780	5570	20100	25400
		25.9	46.9	248	495	2870	5310	20000	23600
Mean		24.7	51	251	502	2720	5180	20700	23200
SD		1.93	3.47	17.6	29.6	114	258	1310	1320
%CV		7.8	6.8	7	5.9	4.2	5	6.3	5.7
%Bias		-1.2	2	0.4	0.4	8.8	3.6	-8	-7.2
n		21	18	20	20	20	21	19	18

*Accuracy more than 15%; excluded from regression analysis.
**Accuracy more than 20%; excluded from regression analysis.

Table 3A2. Analytical Performance: Back-Calculated Concentrations (ng/mL) of Sulfoxide Calibration Standard in Dog Plasma for Study Protocol 0505-2007.									
**Assay Date**	**Analytical Run Number**	**STD.1 25.0 ng/mL**	**STD.2 50.0 ng/mL**	**STD.3 250 ng/mL**	**STD.4 500 ng/mL**	**STD.5 2500 ng/mL**	**STD.6 5000 ng/mL**	**STD.7 22500 ng/mL**	**STD.8 25000 ng/mL**
12-Mar-2008	1	29.7	*68.8	258	*630	*3130	5580	*27100	26200
		21.4	45.7	240	505	2590	4710	21300	23800
13-Mar-2008	2	26.7	54.4	266	523	2400	5230	20000	22200
		21	52.3	276	558	2620	5030	19800	22900
13-Mar-2008	3	24.4	52.3	224	487	2710	4800	20100	22700
		25.1	48.9	285	539	2750	5230	21800	23600
14-Mar-2008	4	23	57.3	241	483	2760	4850	21300	23800
		25.6	49.7	225	*681	2740	*6040	25200	22300
21-Mar-2008	5	25.5	49.9	265	484	2640	5190	19700	23900
		24.7	48.1	273	495	2590	4960	21300	25600
26-Mar-2008	6	22.8	56.7	261	492	2500	5000	*18800	22600
		25.3	*60.9	*321	552	2540	5480	19200	23500
26-Mar-2008	7	20.9	56.8	243	560	*1930	4870	20200	*20000
		26.6	53.2	240	518	2640	5080	*17500	21700
27-Mar-2008	8	21.7	50.1	237	555	2680	5610	21900	*19300
		27.1	54.6	*303	480	2710	4740	19900	21300
27-Mar-2008	9	22.4	49	235	516	2800	4680	21300	*19500
		27	52.7	269	512	2750	5210	20100	22000
28-Mar-2008	10	25.4	50.9	258	545	2590	4990	20500	21400
		23.6	51.5	267	492	2660	5210	20400	*19200
29-Mar-2008	11	22.6	49.2	263	456	2720	5500	19600	24600
		27.6	49.2	259	507	2790	5270	19500	23200
Mean		24.6	51.6	254	513	2660	5110	20700	23200
SD		2.39	3.15	17.4	30.3	103	283	1360	1360
%CV		9.7	6.1	6.9	5.9	3.9	5.5	6.6	5.9
%Bias		-1.6	3.2	1.6	2.6	6.4	2.2	-8	-7.2
n		22	20	20	20	20	21	19	18
*Accuracy more than 15%; excluded from regression analysis.									

Table 4A2. Calibration Curve Parameters for Fexinidazole Calibration Standards in Dog Plasma for Study Protocol 0505-2007.

Run Date	Curve Number	Slope	Intercept	R^2	LLOQ ng/mL	ULOQ ng/mL	Regression Footnote(s)
12-Mar-2008	1	0.00403	0.00314	0.9954	5	1000	1
13-Mar-2008	2	0.00415	0.00179	0.9959	5	1000	1
13-Mar-2008	3	0.00598	0.00208	0.9943	5	1000	1
14-Mar-2008	4	0.00552	0.00811	0.9946	5	1000	1
21-Mar-2008	5	0.00599	0.00416	0.9959	5	1000	1
26-Mar-2008	6	0.00536	0.00265	0.9931	5	1000	1
26-Mar-2008	7	0.00562	0.00168	0.9955	5	1000	1
27-Mar-2008	8	0.00515	-0.00008	0.9911	5	1000	1
27-Mar-2008	9	0.00545	-0.00013	0.993	5	1000	1
28-Mar-2008	10	0.00547	-0.00093	0.9965	5	1000	1
29-Mar-2008	11	0.00448	0.00000	0.9922	5	1000	1
Mean		0.00520	0.00204	0.9943			
SD		0.00068	0.00256	0.0017			
%CV		13.2	125.1	0.2			
n		11	11	11			

Regression Footnote(s):
1) Resp. = Slope * Conc. + Intercept

Table 5A2. Calibration Curve Parameters for Sulfone Calibration Standards in Dog Plasma for Study Protocol 0505-2007.							
**Run Date**	**Curve Number**	**Slope**	**Intercept**	**R^2**	**LLOQ ng/mL**	**ULOQ ng/mL**	**Regression Footnote(s)**
12-Mar-2008	1	0.00433	0.02697	0.9923	25	25000	1
13-Mar-2008	2	0.00425	0.00502	0.9898	25	25000	1
13-Mar-2008	3	0.00614	0.01536	0.9924	25	25000	1
14-Mar-2008	4	0.00543	0.04225	0.9887	25	25000	1
21-Mar-2008	5	0.00655	0.01189	0.9943	25	25000	1
26-Mar-2008	6	0.00485	0.00781	0.9922	25	25000	1
26-Mar-2008	7	0.00543	0.00947	0.992	25	25000	1
27-Mar-2008	8	0.00475	0.01223	0.988	25	25000	1
27-Mar-2008	9	0.00516	0.01442	0.9926	25	25000	1
28-Mar-2008	10	0.00512	0.00214	0.994	25	25000	1
29-Mar-2008	11	0.00398	0.04711	0.9912	25	25000	1
Mean		0.00509	0.01770	0.9916			
SD		0.00078	0.01484	0.002			
%CV		15.4	83.9	0.2			
n		11	11	11			

Regression Footnote(s):
1) Resp. = Slope * Conc. + Intercept

Table 6A2. Calibration Curve Parameters for Sulfoxide Calibration Standards in Dog Plasma for Study Protocol 0505-2007.							
**Run Date**	**Curve Number**	**Slope**	**Intercept**	**R^2**	**LLOQ ng/mL**	**ULOQ ng/mL**	**Regression Footnote(s)**
12-Mar-2008	1	0.00385	0.01864	0.9894	25	25000	1
13-Mar-2008	2	0.00376	0.01092	0.9895	25	25000	1
13-Mar-2008	3	0.00541	0.01822	0.9927	25	25000	1
14-Mar-2008	4	0.00492	0.04264	0.9905	25	25000	1
21-Mar-2008	5	0.00578	0.00405	0.9964	25	25000	1
26-Mar-2008	6	0.00446	0.01375	0.9915	25	25000	1
26-Mar-2008	7	0.00533	0.00604	0.988	25	25000	1
27-Mar-2008	8	0.00462	0.01543	0.9883	25	25000	1
27-Mar-2008	9	0.00507	0.01603	0.9916	25	25000	1
28-Mar-2008	10	0.00501	0.00873	0.994	25	25000	1
29-Mar-2008	11	0.00407	0.01632	0.991	25	25000	1
Mean		0.00475	0.01553	0.9912			
SD		0.00066	0.01022	0.0025			
%CV		13.9	65.8	0.3			
n		11	11	11			

Regression Footnote(s):
1) Resp. = Slope * Conc. + Intercept

Table 7A2. Analytical Performance of Fexinidazole Quality Control Samples in Dog Plasma for Study Protocol 0505-2007.							
**Run Date**	**Curve Number**	**QC1 15.0 ng/mL**	**QC2 75.0 ng/mL**	**QC3 800 ng/mL**	**QC3 800 ng/mL Dilution=2**	**QC4 2000 ng/mL Dilution=5**	**QC4 2000 ng/mL Dilution=10**
12-Mar-2008	1	15.5	83.5	853			
		15.5	81.5	798			
13-Mar-2008	2	14.7	75.9	780			
		15.6	82.4	816			
13-Mar-2008	3	15	83.3	904			
		15.3	~86.5	780			
14-Mar-2008	4	14.2	79.4	822	847		
		15.8	83.7	898	844		
					775		
21-Mar-2008	5	16	80.5	836	757		
		17.8	~89.6	701	912		
					~1120		
26-Mar-2008	6	16.8	71.7	831			2020
		16.7	79.2	784			2220
							2000
26-Mar-2008	7	13.7	77.5	739		1950	
		13.5	80.6	779		1980	
						2020	
27-Mar-2008	8	~36.4	72.6	845		2050	
		17	78.9	838		2170	
						2160	
27-Mar-2008	9	15	76.3	782		1890	
		16.1	80.6	810		2130	
						1900	
28-Mar-2008	10	14.5	74.6	760		2010	
		14.9	77.9	820		1940	
						1980	
29-Mar-2008	11	15.3	68.4	797		2100	
		15.4	80.5	854		2130	
						2220	
Mean		16.4	79.3	810	876	2040	2080
SD		4.59	4.88	47.7	132	104	122
%CV		28	6.2	5.9	15.1	5.1	5.9
%Bias		9.3	5.7	1.3	9.5	2	4
n		22	22	22	6	15	3
~ > 15% Theoretical							

Table 8A2. Analytical Performance of Sulfone Quality Control Samples in Dog Plasma for Study Protocol 0505-2007.

Run Date	Curve Number	QC1 75.0 ng/mL	QC2 750 ng/mL	QC3 20000 ng/mL	QC3 20000 ng/mL Dilution=2	QC4 50000 ng/mL Dilution=5	QC4 50000 ng/mL Dilution=10
12-Mar-2008	1	~91.4	~931	22200			
		77.2	788	18900			
13-Mar-2008	2	75.5	783	18100			
		82.8	831	18300			
13-Mar-2008	3	69.4	752	19700			
		79	~881	18000			
14-Mar-2008	4	65.6	853	18400	22700		
		84.6	~946	20100	21900		
					21000		
21-Mar-2008	5	78.8	777	19000	18800		
		85.7	~892	~16500	23000		
					~26900		
26-Mar-2008	6	83.5	709	19700			52000
		~92.7	799	17800			~57600
							53100
26-Mar-2008	7	67.7	740	~16000		47400	
		71	789	17400		46000	
						49700	
27-Mar-2008	8	~604	711	19000		52500	
		82.8	821	19100		52600	
						54200	
27-Mar-2008	9	73.4	734	~16900		46100	
		81.6	791	17500		50100	
						45700	
28-Mar-2008	10	72.4	743	~16400		48900	
		80.4	774	18000		46200	
						47600	
29-Mar-2008	11	~244	656	18500		52300	
		84.6	802	22400		56200	
						56000	
Mean		110	796	18500	22400	50100	54200
SD		116	72.2	1630	2680	3670	2970
%CV		105.5	9.1	8.8	12	7.3	5.5
%Bias		46.7	6.1	-7.5	12	0.2	8.4
n		22	22	22	6	15	3

~ > 15% Theoretical

Table 9A2. Analytical Performance of Sulfoxide Quality Control Samples in Dog Plasma for Study Protocol 0505-2007.

Run Date	Curve Number	QC1 75.0 ng/mL	QC2 750 ng/mL	QC3 20000 ng/mL	QC3 20000 ng/mL Dilution=2	QC4 50000 ng/mL Dilution=5	QC4 50000 ng/mL Dilution=10
12-Mar-2008	1	~92.0	~937	22400			
		83	780	18800			
13-Mar-2008	2	76	772	18500			
		84.1	827	18600			
13-Mar-2008	3	69	754	20100			
		81	~869	18200			
14-Mar-2008	4	65.9	852	18600	22700		
		88.8	~946	20200	21800		
		NA	NA	NA	21100		
21-Mar-2008	5	77.9	769	19100	18600		
		~90.5	844	~16700	23000		
					~27400		
26-Mar-2008	6	77.8	675	19400			50200
		~94.3	858	19100			~61000
							56000
26-Mar-2008	7	69.6	736	~15800		47200	
		69.9	797	17300		46200	
						49300	
27-Mar-2008	8	~613	710	19100		53000	
		84.3	804	19100		53700	
						55200	
27-Mar-2008	9	73.7	729	~16900		46300	
		79.2	788	17500		50000	
						46000	
28-Mar-2008	10	70.3	735	~16500		49300	
		76.4	773	18100		47400	
						47900	
29-Mar-2008	11	83.2	660	17700		51900	
		83.7	808	22100		55500	
						55200	
Mean		104	792	18600	22400	50300	55700
SD		114	73.7	1630	2900	3520	5400
%CV		109.6	9.3	8.8	12.9	7	9.7
%Bias		38.7	5.6	-7	12	0.6	11.4
n		22	22	22	6	15	3

~ > 15% Theoretical

Appendix 14 Clinical Pathology Methods

HEMATOLOGY METHODS

PARAMETERS	CODE	UNIT	METHODS AND INSTRUMENTS
Red blood cells	RBC	n x 10^6/µL	Laser beam method. " ADVIA 120™ System" Bayer
Hemoglobin	HGB	g/dL	Colorimetric method as cyanmethemoglobin. "ADVIA 120™ System " Bayer
Hematocrit	HCT	%	Calculated: Wintrobe formulae “ADVIA 120™ System" Bayer
Mean corpuscular hemoglobin concentration	MCHC	g/dL	Calculated: Wintrobe formulae "ADVIA 120™ System" Bayer
Mean corpuscular volume	MCV	fL	Mean of RBC volume histogram x MCV calibration factor. "ADVIA 120™ System" Bayer
Mean corpuscular hemoglobin	MCH	pg	Calculated: Wintrobe formulae "ADVIA 120™ System" Bayer
Red cell distribution width	RDW	%	Calculated: Standard deviation of RBC volume histogram/MCV (fl) x 100. "ADVIA 120™ System" Bayer
Hemoglobin distribution width	HDW	g/dL	Calculated: Standard deviation of RBC HC histogram. "ADVIA 120™ System" Bayer
Reticulocytes	R	%	Laser beam method with colorimetric reaction for nucleic acids. "ADVIA 120™ System" Bayer
Reticulocytes absolutes	RAB	n x 10^9/L	Laser beam method with colorimetric reaction for nucleic acids. "ADVIA 120™ System" Bayer
Mean corpuscolar volume of reticulocytes	MCVr	fL	Mean of R volume histogram x MCV calibration factor. "ADVIA 120™ System" Bayer
Mean hemoglobin concentration of reticulocytes	CHCM	g/dL	Mean of R hemoglobin histogram "ADVIA 120™ System" Bayer
Cellular hemoglobin of reticulocytes	CHr	pg	Calculated: Wintrobe formulae "ADVIA 120™ System" Bayer
Platelets	PLT	n x 10^3/µL	Laser beam method. "ADVIA 120™ System" Bayer
Mean platelet volume	MPV	fL	Mean platelet volume histogram "ADVIA 120™ System" Bayer
Platelet distribution width	PDW	%	Standard deviation of platelet volume histogram/ MPV (fl) x 100 "ADVIA 120™ System" Bayer
Platelet hematocrit	PCT	%	Mean platelet volume x PLT. "ADVIA 120™ System" Bayer
White blood cells	WBC	n x 10^3/µL	Laser beam method with “Baso/Lobularity method” and Peroxidase reaction. "ADVIA 120™ System" Bayer

HEMATOLOGY METHODS (Cont.)

PARAMETERS	CODE	UNIT	METHODS AND INSTRUMENTS
WBC differential count:<br>Neutrophils<br>Lymphocytes<br>Eosinophils<br>Basophils<br>Monocytes<br>Large unstained cells<br>Band Neutrophils<br>Segmented Neutrophils	 N<br>LY<br>E<br>B<br>M<br>LU<br>BANN<br>SEGN	%	Peroxidase and Basophil/Lobularity method "ADVIA 120™ System" Bayer<br>Percentage of Band and Segmented Neutrophils manually calculated on 100 Neutrophils examined at microscopy
WBC differential count:<br>Neutrophils ABS<br>Lymphocytes ABS<br>Eosinophils ABS<br>Basophils ABS<br>Monocytes ABS<br>Large unstained cells ABS<br>Band Neutrophils ABS<br>Segmented Neutrophils ABS	 NAB<br>LYAB<br>EAB<br>BAB<br>MAB<br>LUAB<br>BAAB<br>SEAB	n x 10^3/µL	Peroxidase and Basophil/Lobularity method "ADVIA 120™ System" Bayer<br>Absolute number of Band and Segmented Neutrophils calculated on percentage of Band and Segmented Neutrophils and value of NAB
Prothrombin Time	PT	Sec	Photometric method. Kit: Dade Behring. Coagulometer "BCS" Dade Behring.
Prothrombin Time Ratio	PTr		Calculated parameter
Fibrinogen	FIBR	mg/dL	Photometric method. Kit: Dade Behring. Coagulometer "BCS" Dade Behring.
Activated Partial Thromboplastin Time	PTT	Sec	Photometric method. Kit: Dade Behring. Coagulometer "BCS" Dade Behring.
Activated Partial Thromboplastin Time Ratio	PTTr		Calculated parameter

CLINICAL CHEMISTRY METHODS

PARAMETERS	CODE	UNIT	METHODS AND INSTRUMENTS
Urea	UREA	mg/dL	Urease - GLDH'': enzymatic UV test.<br>Kit: Horiba ABX adapted to the "Pentra 400" analyzer.
Creatinine	CREA	mg/dL	Colorimetric complex between the creatinine and the alkaline picrate (Jaffé).<br>Kit: Horiba ABX adapted to the "Pentra 400" analyzer.
Aspartate aminotransferase	AST	IU/L	UV-test according to IFCC modified method without pyridoxal phosphate.<br>Kit: Horiba ABX adapted to the "Pentra 400" analyzer.
Alanine aminotransferase	ALT	IU/L	UV-test according to IFCC modified method without pyridoxal phosphate.<br>Kit: Horiba ABX adapted to the "Pentra 400" analyzer.
Alkaline phosphatase	AP	IU/L	Kinetic photometric test, according to the IFCC.<br>Kit: Horiba ABX adapted to the "Pentra 400" analyzer.
G-Glutamyl transferase	GGT	IU/L	Kinetic photometric test according to Szasz modified.<br>Kit: Horiba ABX adapted to the "Pentra 400" analyzer.
Total bilirubin	T.BIL	mg/dL	Photometric test using 2,4-dichloroaniline.<br>Kit: Horiba ABX adapted to the "Pentra 400" analyzer.
Total proteins	TPRO	g/dL	Biuret reaction. End-point method.<br>Kit: Horiba ABX adapted to the "Pentra 400" analyzer.
Albumin	ALB.	g/dL	Colorimetric determination using bromocresol green.<br>Kit: Horiba ABX adapted to the "Pentra 400" analyzer.
Globulin	GLOB	g/dL	Calculated parameter (T.Protein-Albumin) by Xybion.
Glucose	GLUC	mg/dL	Enzymatic determination. (Trinder method).<br>Kit: Horiba ABX adapted to the "Pentra 400" analyzer.
Triglycerides	TG	mg/dL	Enzymatic determination<br>Kit: Horiba ABX adapted to the "Pentra 400" analyzer.
Total Cholesterol	TCHO	mg/dL	''CHOD-PAP'': enzymatic photometric test. (Trinder's reaction).<br>Kit: Horiba ABX adapted to the "Pentra 400" analyzer.
Calcium	Ca	mg/dL	Photometric test using ortho-cresolphtalein complexone (OPC).<br>Kit: Horiba ABX adapted to the "Pentra 400" analyzer.
Phosphorus	PHOS	mg/dL	UV method using phosphomolybdate<br>Kit: Horiba ABX adapted to the "Pentra 400" analyzer.
Sodium	Na	mEq/L	Direct potentiometry ion selective electrodes.<br>ISE module on the "Pentra 400" analyzer
Chloride	Cl	mEq/L	Direct potentiometry ion selective electrodes.<br>ISE module on the "Pentra 400" analyzer
Potassium	K	mEq/L	Direct potentiometry ion selective electrodes.<br>ISE module on the "Pentra 400" analyzer
Albumin/Globulin	A/G		Calculated parameter by Xybion

URINALYSIS METHODS

PARAMETERS	CODE	UNIT	METHODS AND INSTRUMENTS
Appearance			
Limpid	L		
Turbid	T		Turbidimetric method with Urysis 2400 (Roche).
Light Turbid	LT		
Colour:			
Yellow	Y		
Amber	A		
Orange	O		
Greenish	G		Photometric method with Urysis 2400 (Roche).
Reddish	R		
Brownish	B		
Colorless	W		
Other	O		
Volume	VOL	ml/16h	
Specific Gravity	S.G.		Refractometric method with Urysis 2400 (Roche).
pH	pH		
Proteins	PRO	Score 0-4	
Nitrites	NIT	" 0-1	Semi-quantitative determination with Sticks Urysis 2400 Cassette
Glucose	GLU	" 0-4	(Roche).
Ketones	KETO	" 0-3	
Urobilinogen	UBG	" 0-3	
Bilirubin	BIL	" 0-2	Photometric method with Urysis 2400 (Roche).
Hemoglobin/Red blood cells	ERY	" 0-3	
White blood cells	WBC	" 0-2	

The scoring system is as follows:

SCORE	PROTEINS mg/dL	NITRITES	GLUCOSE mg/dL	KETONES mg/dL	UROBIL. mg/dL	BILIR. mg/dL	WHITE B.CELLS WBC/µ L	HEMOGLOBIN ERYTHROC. RBC/µL
0	Absent	Absent	Absent	Up to 5	Up to 1	Up to 1	Up to 25	Up to 10
1	Up to 25	Present	Up to 50	Up to 15	Up to 4	Up to 3	Up to 100	Up to 50
2	Up to 75	…	Up to 100	Up to 50	Up to 8	Up to 6	Up to 500	Up to 150
3	Up to 150	…	Up to 300	Up to 150	Up to 12	…	…	Up to 250
4	Up to 500	…	Up to 1000	…	…	…	…	…

Appendix 15 Pharmacy Documentation

Pharmacy Certification

**STUDY NUMBER**	**:**	**0505-2007**
**TEST ITEM**	**:**	**Fexinidazole**
**NOTEBOOK NUMBER(S)**	**:**	**19201/B and 19094/D**

DOCUMENTATION ENCLOSED IN THE NOTEBOOK:

A. Analysis certificate dated December 18, 2007 issued by Orgasynth Industries for Fexinidazole test item, raw material, Batch No. 3168-07-01/O
B. Material safety data sheet issued by Orgasynth Industries for Fexinidazole
C. Certificate of Analysis issued by Sigma-Aldrich for Methylcellulose 400 cP, raw material, Lot No. 125K0196
D. Certificate of Analysis issued by Sigma-Aldrich for Tween® 80, raw material, Lot No. 1324202
E. Label’s photocopy of Acqua per preparazioni iniettabili (Bieffe Medital S.p.A.), raw material, Lot No. 07G0201
F. Label’s photocopy of Acqua per preparazioni iniettabili (Bieffe Medital S.p.A.), raw material, Lot No. 07K1503
G. Copy of request cards of test item

ANALYTICAL DOCUMENTATION:

Requests and Analytical Results issued by Accelera/ADMET/Preclinical Formulation and Accelera/DMPK&ART/Bioanalysis and Analytical Control

MATERIALS USED FOR THE STUDY:

1. Fexinidazole test item, raw material, Batch No. 3168-07-01/O
2. Methylcellulose 400 cP, raw material, Lot No. 125K0196
3. Tween® 80, raw material, Lot No. 1324202
4. Acqua per preparazioni iniettabili, raw material, Lot No. 07G0201
5. Acqua per preparazioni iniettabili, raw material, Lot No. 07K1503

PREPARATIONS:

Prepare suspension of Fexinidazole test item, raw material, Lot No. 3168-07-01/O in the vehicle (5% Tween® 80 in 0.5% Methylcellulose 400 cP solution) at the concentration of 5 mg/mL, 20 mg/mL and 80 mg/mL

Pharm. Cert. 0505-2007

CONCENTRATION CHECKS:

Fexinidazole suspensions:

		% of L.A.	Preparation date
80 mg/mL	Request No. 200800009 (Top)	95.92%	January 28, 2008
80 mg/mL	Request No. 200800010 (Middle)	97.38%	January 28, 2008
80 mg/mL	Request No. 200800011 (Bottom)	96.87%	January 28, 2008
20 mg/mL	Request No. 200800012 (Top)	104.34%	January 28, 2008
20 mg/mL	Request No. 200800013 (Middle)	99.17%	January 28, 2008
20 mg/mL	Request No. 200800014 (Bottom)	93.68%	January 28, 2008
5 mg/mL	Request No. 200800015 (Top)	95.89%	January 28, 2008
5 mg/mL	Request No. 200800016 (Middle)	107.31%	January 28, 2008
5 mg/mL	Request No. 200800017 (Bottom)	107.93%	January 28, 2008
80 mg/mL	Request No. 200800045 (Top)	97.94%	February 22, 2008
80 mg/mL	Request No. 200800046 (Middle)	105.62%	February 22, 2008
80 mg/mL	Request No. 200800047 (Bottom)	98.53%	February 22, 2008
20 mg/mL	Request No. 200800048 (Top)	95.76%	February 22, 2008
20 mg/mL	Request No. 200800049 (Middle)	98.19%	February 22, 2008
20 mg/mL	Request No. 200800050 (Bottom)	95.13%	February 22, 2008
5 mg/mL	Request No. 200800051 (Top)	108.04%	February 22, 2008
5 mg/mL	Request No. 200800052 (Middle)	105.43%	February 22, 2008
5 mg/mL	Request No. 200800053 (Bottom)	102.30%	February 22, 2008

S**TABILITY:**

Fexinidazole test item:

Expire date October 2008 for Fexinidazole, test item, raw material, Lot No. 3168-07-01/O if stored at room temperature protected from light.

Fexinidazole suspension:

Stability data indicate that Fexinidazole suspensions in the vehicle (5% Tween® 80 in 0.5% Methylcellulose 400 cP solution) in the range 0.5-100 mg/mL are stable up to 7 days at room temperature and 14 days at +4°C (Nerviano MS 0293-2007-R)

Date: May 20, 2008
