# Supplementary material for: Fexinidazole – A New Oral Nitroimidazole Drug Candidate Entering Clinical Development for the Treatment of Sleeping Sickness
Source: PLoS Negl Trop Dis. 2010 Dec 21;4(12):e923. doi: 10.1371/journal.pntd.0000923 (PMC3006138; doi:10.1371/journal.pntd.0000923)
Supplement: Dataset S18 — (0.22 MB PDF) [file pntd.0000923.s019.pdf]

Final Summary Report

Study Title	Fexinidazole Reverse mutation in four histidine-requiring strains of *Salmonella typhimurium*
Author	
Sponsor	Drugs for Neglected Diseases Initiative (DNDi)<br>1 place Saint Gervais<br>1201 Geneva<br>Switzerland
Study Monitor	
Test Facility	Covance Laboratories Ltd
Covance Study Number	2647/3
Covance Master Protocol Number	2647 MPR Ames (Version 1, January 2006)
Report Issued	February 2009
Page Number	1 of 18

COVANCE
THE DEVELOPMENT SERVICES COMPANY

**COMPOUND**	Fexinidazole
**SOLVENT**	Sterile anhydrous analytical grade dimethyl sulphoxide (DMSO)
**CONCENTRATION LEVELS**	0.8, 4, 20, 100, 350, 1000 and 5000 µg/plate.
**STRAINS TESTED**	*Salmonella typhimurium* TA98, TA98NR, TA100 and TA100NR
**METHODOLOGY**	All experimentation was performed according to the protocol, with treatments of all the tester strains being performed both in the absence and in the presence of a rat liver metabolic activation system (Aroclor 1254 induced S-9) at the concentrations specified above.
**RESULTS**	
**Precipitation**	Precipitation of the test article was observed at the highest two concentrations tested in the absence and presence of S-9.
**Toxicity**	Evidence of toxicity in the form of a slight thinning of the background bacterial lawn or a complete killing of the test bacteria was observed in strains TA98, TA98NR and TA100 following treatments at the highest two or three concentrations tested in the presence of S-9. A tailing off of the mutagenic response at 5000 µg/plate in strains TA98 and TA100NR in the absence of S-9 may also be indicative of toxicity.

MUTAGENICITY

Statistically significant, dose related increases in revertant numbers were observed following Fexinidazole treatments of all of the tester strains both in the absence and presence of S-9 (with the exception of strain TA98NR in the absence of S-9) when the data were analysed at the 1% level using Dunnett's test. Although significant increases in revertant numbers were observed following most treatments of strains TA98NR and TA100NR, the magnitude of these effects was substantially reduced in comparison to the respective parent strains. Treatments in the presence of S-9 increased mutagenicity and potency in all strains tested although this effect was more apparent in strains TA98NR and TA100NR. This may indicate that metabolites of different mutational specificity to the parent compound may be formed.

This screening study was considered to have provided evidence of Fexinidazole mutagenic activity in this assay system. This mutagenic activity can be attributed, at least in part, to bacterial nitroreductase enzymes.

Lowest detectable concentration

The lowest concentrations of Fexinidazole to give a statistically significant result when the data were analysed at the 1% level using Dunnett's test for each strain are as follows:

TA98 –S-9	350 µg/plate
TA98NR –S-9	Not applicable
TA98 +S-9	100 µg/plate
TA98NR+S-9	1000 µg/plate
TA100 –S-9	4 µg/plate
TA100NR –S-9	350 µg/plate
TA100 +S-9	4 µg/plate
TA100NR +S-9	100 µg/plate

CONCLUSION

It was concluded that Fexinidazole did induce mutation in *Salmonella typhimurium* strains TA98, TA100, and TA100NR in the absence and presence of S-9 and in strain TA98NR in the presence of S-9 only, when tested under the conditions employed in this screening study. These conditions included treatment at concentrations up to 5000 µg/plate, in the absence and presence of a rat liver metabolic activation system (S-9). This mutagenic activity was attributed, at least in part, to bacterial nitroreductase enzymes.

COMMENTS

Following treatments of strain TA100 at 1000 and 5000 µg/plate in the absence of S-9, the nature of the precipitation, along with the large number of colonies made it impossible to obtain accurate revertant counts from these plates. Therefore no counts are reported for these concentrations of TA100 in the absence of S-9 in the data tables.

MUTATION EXPERIMENT RESULTS

Fexinidazole: summary of mean revertant colonies (-S-9) - Experiment 1

Substance	Dose Level µg/plate	**TA98**	**TA98NR**	**TA100**	**TA100NR**
		Mean ± SD	Mean ± SD	Mean ± SD	Mean ± SD
DMSO	100 µl	24 ± 6	22 ± 6	90 ± 5	100 ± 7
Fexinidazole	0.8	29 ± 3	23 ± 3	106 ± 8	107 ± 20
	4	25 ± 4	19 ± 6	169 ± 30	111 ± 30
	20	26 ± 8	22 ± 6	364 ± 14	107 ± 23
	100	47 ± 13	19 ± 2	1339 ± 77	131 ± 26
	350	167 ± 8	23 ± 8	2250 ± 84	165 ± 12
	1000	158 ± 85 (Ppn+M)	18 ± 4 (Ppn+M)	- (Ppn + X)	162 ± 8 (Ppn+M)
	5000	98 ± 4 (Ppn+M)	19 ± 3 (Ppn+M)	- (Ppn + X)	116 ± 21 (Ppn+M)
Positive controls	Compound	NQO	NQO	NaN_3	NaN_3
	Dose Level	1 µg	1 µg	2 µg	2 µg
	Mean ± SD	494 ± 29	355 ± 23	1107 ± 129	885 ± 57
Controls confirming nitroreductase proficiency / deficiency	Compound	2NF	2NF	NFZ	NFZ
	Dose Level	5 µg	5 µg	2 µg	2 µg
	Mean ± SD	1095 ± 64	161 ± 14	859 ± 34	183 ± 17

SD Standard deviation
2NF 2-Nitrofluorene
NQO 4-Nitroquinoline 1-oxide
NaN_3 Sodium azide
NFZ Nitrofurazone
M : Plate counted manually
Ppn : Precipitation of test article observed
X : Too many colonies. Unable to determine accurate counts due to level and nature of precipitation.

Fexinidazole: summary of mean revertant colonies (+S-9) - Experiment 1

Substance	Dose Level µg/plate	TA98	TA98NR	TA100	TA100NR
		Mean ± SD	Mean ± SD	Mean ± SD	Mean ± SD
DMSO	100 µl	45 ± 4	19 ± 5	177 ± 6	154 ± 14
Fexinidazole	0.8	43 ± 10	18 ± 1	207 ± 3	124 ± 11
	4	66 ± 6	21 ± 8	403 ± 9	141 ± 5
	20	91 ± 16	25 ± 10	1392 ± 127	178 ± 20
	100	517 ± 77	25 ± 6	2586 ± 62	309 ± 11
	350	284 ± 140	27 ± 2	- (T)	549 ± 21
	1000	- (T+Ppn)	47 ± 4 (Ppn+M+S)	- (T+Ppn)	533 ± 8 (Ppn+M)
	5000	- (T+Ppn)	33 ± 10 (Ppn+M+S)	- (Ppn+T)	379 ± 45 (Ppn+M)
Positive controls	Compound	B[a]P	B[a]P	AAN	AAN
	Dose Level	10 µg	10 µg	5 µg	5 µg
	Mean ± SD	573 ± 69	214 ± 10	2573 ± 85	2290 ± 192

SD Standard deviation

B[a]P Benzo[a]pyrene
AAN 2-Aminoanthracene

S : Slight thinning of background bacterial lawn
Ppn : Precipitation of test article observed
T : Toxic, no revertant colonies
M : Plate counted manually

Fexinidazole Experiment 1

Table 1

Test strain: TA98 -S-9

Treatment (µg/plate)	Revertant numbers/plate				
Solvent	16	30	19	24	30
0.8	33	27	28		
4	23	23	30		
20	17	30	31		
100	36	61	43		
350	174	170	158		
1000	126 Ppn+M	94 Ppn+M	255 Ppn+M		
5000	98 Ppn+M	94 Ppn+M	101 Ppn+M		
Positive	1051	1065	1169		

Treatment (µg/plate)	Mean (of)	N	Fold Increase	Standard Deviation	Correlation Coefficient	Slope of best fit	Dunnett's t value
Solvent	23.80	5		6.34			
0.8	29.33	3	1.23	3.21	0.49 NS	6.92	0.64 NS
4	25.33	3	1.06	4.04	0.04 NS	0.13	0.20 NS
20	26.00	3	1.09	7.81	0.03 NS	0.02	0.24 NS
100	46.67	3	1.96	12.90	0.76 ***	0.21	2.18 NS
350	167.33	3	7.03^	8.33	0.98 ***	0.40	9.06 ***
1000	158.33	3	6.65	85.23	0.78 ***	0.15	8.34 ***
5000	97.67	3	4.10	3.51	0.32 NS	0.01	5.64 ***
Positive	1095.00	3	46.01	64.47			
	M Statistic = 6.301						

Key to significance:

* $p \leq 0.05$ ** $p \leq 0.01$ *** $p \leq 0.005$ NS not significant

Key to postfixes:

Ppn : Precipitation of test article observed
M : Plate counted manually

^ : Maximum increase above control

Fexinidazole Experiment 1

Table 2

Test strain: TA98 +S-9

Treatment (µg/plate)	Revertant numbers/plate				
Solvent	42	50	50	44	41
0.8	54	42	34		
4	68	71	59		
20	105	74	94		
100	433	534	584		
350	332	394	127		
1000	T+Ppn	T+Ppn	T+Ppn		
5000	T+Ppn	T+Ppn	T+Ppn		
Positive	651	546	522		

Treatment (µg/plate)	Mean (of)	N	Fold Increase	Standard Deviation	Correlation Coefficient		Slope of best fit	Dunnett's t value	
Solvent	45.40	5		4.34					
0.8	43.33	3	0.95	10.07	0.17	NS	-2.58	-0.13	NS
4	66.00	3	1.45	6.24	0.82	***	5.45	1.00	NS
20	91.00	3	2.00	15.72	0.88	***	2.29	2.00	NS
100	517.00	3	11.39^	76.92	0.98	***	4.74	11.49	***
350	284.33	3	6.26	139.74	0.53	**	0.77	6.99	***
Positive	573.00	3	12.62	68.61					
	M Statistic = 12.370								

Key to significance:

* p ≤ 0.05 ** p ≤ 0.01 *** p ≤ 0.005 NS not significant

Key to postfixes:

Ppn : Precipitation of test article observed
T : Toxic, no revertant colonies

^ : Maximum increase above control

Fexinidazole Experiment 1

Table 3

Test strain: TA98NR -S-9

Treatment (µg/plate)	Revertant numbers/plate				
Solvent	24	20	23	12	29
0.8	25	24	19		
4	21	12	23		
20	28	20	17		
100	21	18	17		
350	22	31	16		
1000	13 Ppn+M	21 Ppn+M	20 Ppn+M		
5000	19 Ppn+M	16 Ppn+M	22 Ppn+M		
Positive	147	174	163		

Treatment (µg/plate)	Mean (of)	N	Fold Increase	Standard Deviation	Correlation Coefficient	Slope of best fit	Dunnett's t value
Solvent	21.60	5		6.27			
0.8	22.67	3	1.05	3.21	0.11 NS	1.33	0.35 NS
4	18.67	3	0.86	5.86	0.27 NS	-0.81	-0.75 NS
20	21.67	3	1.00	5.69	0.00 NS	0.00	0.06 NS
100	18.67	3	0.86	2.08	0.21 NS	-0.03	-0.67 NS
350	23.00	3	1.06^	7.55	0.11 NS	0.00	0.35 NS
1000	18.00	3	0.83	4.36	0.17 NS	-0.00	-0.89 NS
5000	19.00	3	0.88	3.00	0.14 NS	-0.00	-0.59 NS
Positive	161.33	3	7.47	13.58			
	M Statistic = 1.296						

Key to significance:

* p ≤ 0.05 ** p ≤ 0.01 *** p ≤ 0.005 NS not significant

Key to postfixes:

Ppn : Precipitation of test article observed
M : Plate counted manually

^ : Maximum increase above control

Fexinidazole Experiment 1

Table 4

Test strain: TA98NR +S-9

Treatment (µg/plate)	Revertant numbers/plate							
Solvent	25		20		22		17	13
0.8	19		18		17			
4	28		12		23			
20	16		35		23			
100	32		21		23			
350	29		25		28			
1000	43	Ppn+M+S	47	Ppn+M+S	50	Ppn+M+S		
5000	34	Ppn+M+S	43	Ppn+M+S	23	Ppn+M+S		
Positive	204		214		223			

Treatment (µg/plate)	Mean (of)	N	Fold Increase	Standard Deviation	Correlation Coefficient		Slope of best fit	Dunnett's t value	
Solvent	19.40	5		4.62					
0.8	18.00	3	0.93	1.00	0.20	NS	-1.75	-0.29	NS
4	21.00	3	1.08	8.19	0.18	NS	0.48	0.30	NS
20	24.67	3	1.27	9.61	0.38	NS	0.29	1.13	NS
100	25.33	3	1.31	5.86	0.36	NS	0.06	1.37	NS
350	27.33	3	1.41	2.08	0.44	*	0.02	1.83	NS
1000	46.67	3	2.41^	3.51	0.86	***	0.03	5.29	***
5000	33.33	3	1.72	10.02	0.40	*	0.00	2.91	*
Positive	213.67	3	11.01	9.50					
	M Statistic = 1.553								

Key to significance:

* p ≤ 0.05 ** p ≤ 0.01 *** p ≤ 0.005 NS not significant

Key to postfixes:

S : Slight thinning of background bacterial lawn
Ppn : Precipitation of test article observed
M : Plate counted manually

^ : Maximum increase above control

Fexinidazole Experiment 1

Table 5

Test strain: TA100 -S-9

Treatment (µg/plate)	Revertant numbers/plate				
Solvent	92	91	92	95	82
0.8	112	97	110		
4	204	150	154		
20	370	374	348		
100	1428	1295	1295		
350	2312	2283	2155		
1000	X+Ppn	X+Ppn	X+Ppn		
5000	X+Ppn	X+Ppn	X+Ppn		
Positive	1149	962	1209		

Treatment (µg/plate)	Mean (of)	N	Fold Increase	Standard Deviation	Correlation Coefficient		Slope of best fit	Dunnett's t value	
Solvent	90.40	5		4.93					
0.8	106.33	3	1.18	8.14	0.82	**	19.92	1.54	NS
4	169.33	3	1.87	30.09	0.92	***	19.73	6.65	***
20	364.00	3	4.03	14.00	0.99	***	13.49	18.33	***
100	1339.33	3	14.82	76.79	1.00	***	12.40	51.86	***
350	2250.00	3	24.89^	83.54	0.96	***	6.23	72.62	***
Positive	1106.67	3	12.24	128.83					
	M Statistic = 2.079								

Key to significance:

* p ≤ 0.05   ** p ≤ 0.01   *** p ≤ 0.005   NS not significant

Key to postfixes:

Ppn : Precipitation of test article observed
X : Too many colonies. Unable to determine accurate counts due to level and nature of precipitation.

^ : Maximum increase above control

Fexinidazole Experiment 1

Table 6

Test strain: TA100 +S-9

Treatment (µg/plate)	Revertant numbers/plate				
Solvent	170	179	186	173	175
0.8	203	209	209		
4	396	401	413		
20	1535	1351	1291		
100	2515	2626	2617		
350	T	T	T		
1000	T+Ppn	T+Ppn	T+Ppn		
5000	Ppn+T	Ppn+T	Ppn+T		
Positive	2476	2633	2611		

Treatment (µg/plate)	Mean (of)	N	Fold Increase	Standard Deviation	Correlation Coefficient	Slope of best fit	Dunnett's t value
Solvent	176.60	5		6.19			
0.8	207.00	3	1.17	3.46	0.95 ***	38.00	2.00 NS
4	403.33	3	2.28	8.74	1.00 ***	57.42	12.38 ***
20	1392.33	3	7.88	127.14	0.99 ***	61.15	43.72 ***
100	2586.00	3	14.64^	61.65	0.95 ***	23.56	68.43 ***
Positive	2573.33	3	14.57	85.01			
	M Statistic = 2.294						

Key to significance:

* p ≤ 0.05 ** p ≤ 0.01 *** p ≤ 0.005 NS not significant

Key to postfixes:

Ppn : Precipitation of test article observed
T : Toxic, no revertant colonies

^ : Maximum increase above control

Fexinidazole Experiment 1

Table 7

Test strain: TA100NR -S-9

Treatment (µg/plate)	Revertant numbers/plate				
Solvent	108	91	100	95	104
0.8	129	89	103		
4	145	103	86		
20	123	118	81		
100	160	111	121		
350	173	172	151		
1000	154 Ppn+M	163 Ppn+M	169 Ppn+M		
5000	128 Ppn+M	92 Ppn+M	127 Ppn+M		
Positive	943	883	830		

Treatment (µg/plate)	Mean (of)	N	Fold Increase	Standard Deviation	Correlation Coefficient	Slope of best fit	Dunnett's t value
Solvent	99.60	5		6.80			
0.8	107.00	3	1.07	20.30	0.30 NS	9.25	0.52 NS
4	111.33	3	1.12	30.37	0.27 NS	2.68	0.79 NS
20	107.33	3	1.08	22.94	0.10 NS	0.23	0.53 NS
100	130.67	3	1.31	25.89	0.48 *	0.27	2.20 NS
350	165.33	3	1.66^	12.42	0.78 ***	0.18	4.46 ***
1000	162.00	3	1.63	7.55	0.70 ***	0.06	4.26 ***
5000	115.67	3	1.16	20.50	0.05 NS	0.00	1.16 NS
Positive	885.33	3	8.89	56.54			
	M Statistic = 3.113						

Key to significance:

* $p \leq 0.05$ ** $p \leq 0.01$ *** $p \leq 0.005$ NS not significant

Key to postfixes:

Ppn : Precipitation of test article observed
M : Plate counted manually

^ : Maximum increase above control

Fexinidazole Experiment 1

Table 8

Test strain: TA100NR +S-9

Treatment (µg/plate)	Revertant numbers/plate				
Solvent	145	169	147	170	141
0.8	120	136	116		
4	145	142	135		
20	155	191	188		
100	300	321	305		
350	567	526	553		
1000	524 Ppn+M	536 Ppn+M	540 Ppn+M		
5000	340 Ppn+M	428 Ppn+M	368 Ppn+M		
Positive	2502	2127	2242		

Treatment (µg/plate)	Mean (of)	N	Fold Increase	Standard Deviation	Correlation Coefficient	Slope of best fit	Dunnett's t value
Solvent	154.40	5		13.96			
0.8	124.00	3	0.80	10.58	0.80 NS	-38.00	-3.00 NS
4	140.67	3	0.91	5.13	0.22 NS	-2.07	-1.30 NS
20	178.00	3	1.15	19.97	0.63 **	1.76	2.12 NS
100	308.67	3	2.00	10.97	0.97 ***	1.68	11.99 ***
350	548.67	3	3.55^	20.84	0.99 ***	1.17	25.62 ***
1000	533.33	3	3.45	8.33	0.83 ***	0.42	24.86 ***
5000	378.67	3	2.45	44.96	0.37 *	0.04	16.35 ***
Positive	2290.33	3	14.83	192.12			
	M Statistic = 1.389						

Key to significance:

* $p \leq 0.05$ ** $p \leq 0.01$ *** $p \leq 0.005$ NS not significant

Key to postfixes:

Ppn : Precipitation of test article observed
M : Plate counted manually

^ : Maximum increase above control

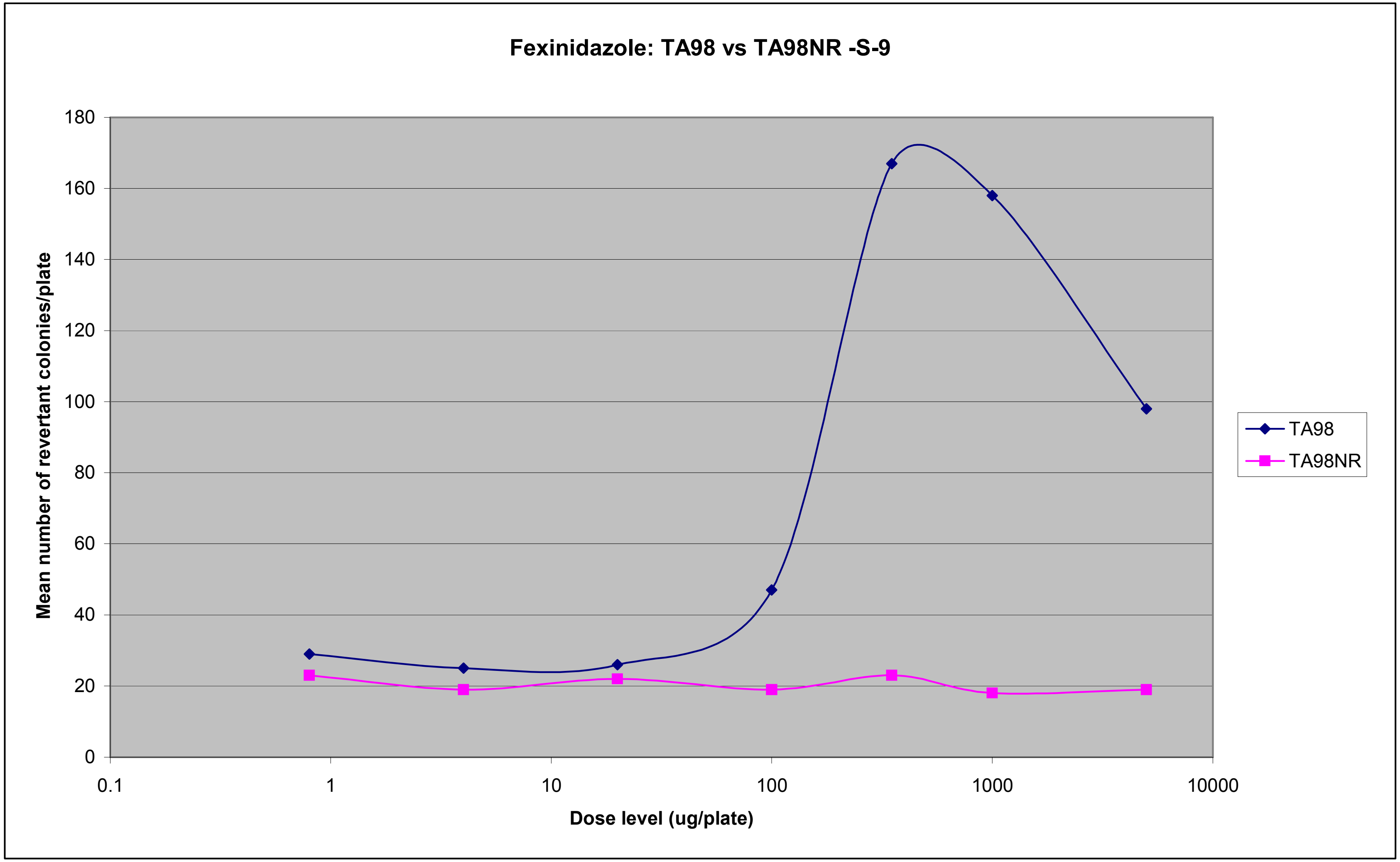
Fexinidazole: TA98 vs TA98NR -S-9
Mean number of revertant colonies/plate
180
160
140
120
100
80
60
40
20
0
0.1
1
10
100
1000
10000
Dose level (ug/plate)
TA98
TA98NR

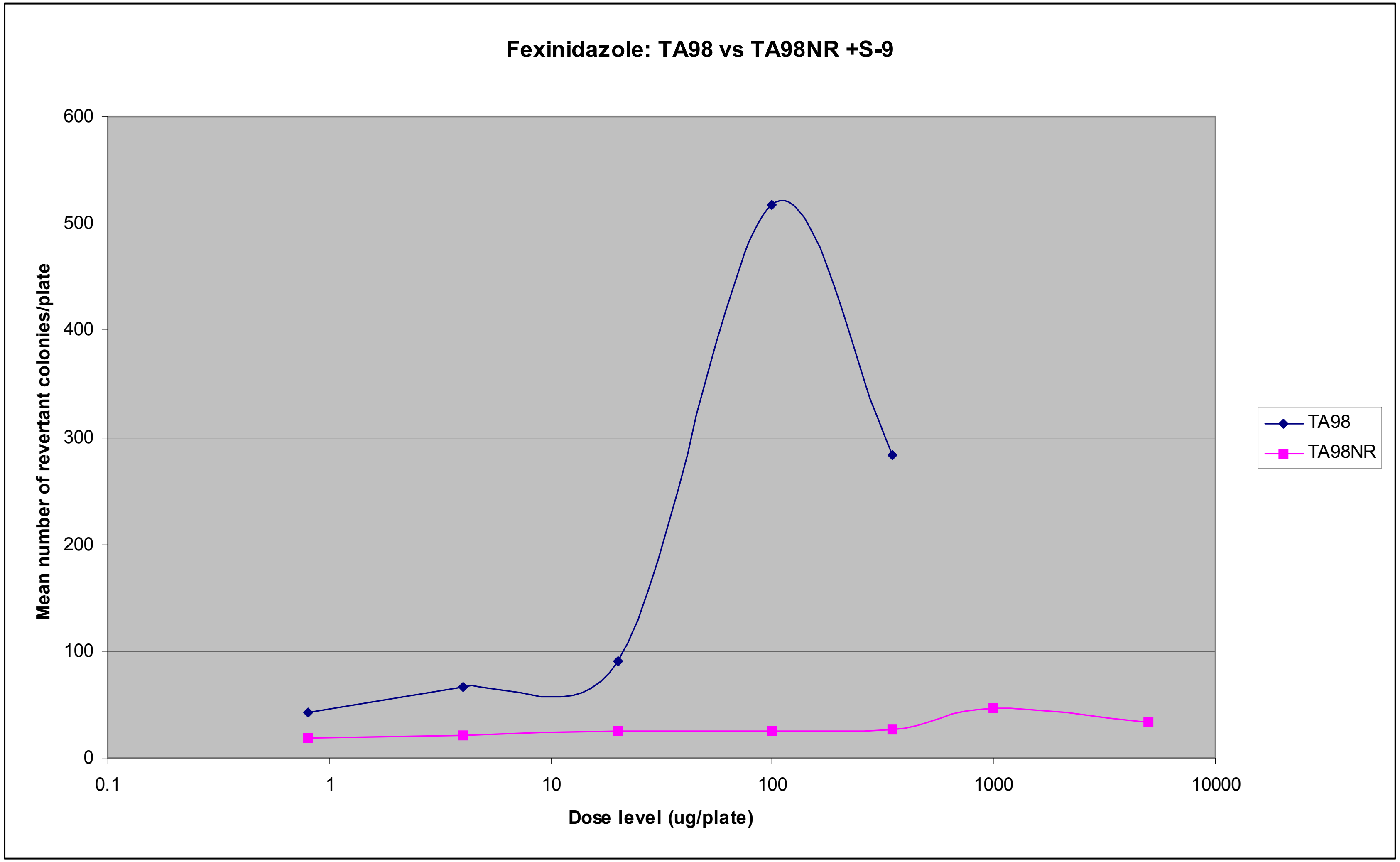
Fexinidazole: TA98 vs TA98NR +S-9
Mean number of revertant colonies/plate
600
500
400
300
200
100
0
0.1
1
10
100
1000
10000
Dose level (ug/plate)
TA98
TA98NR

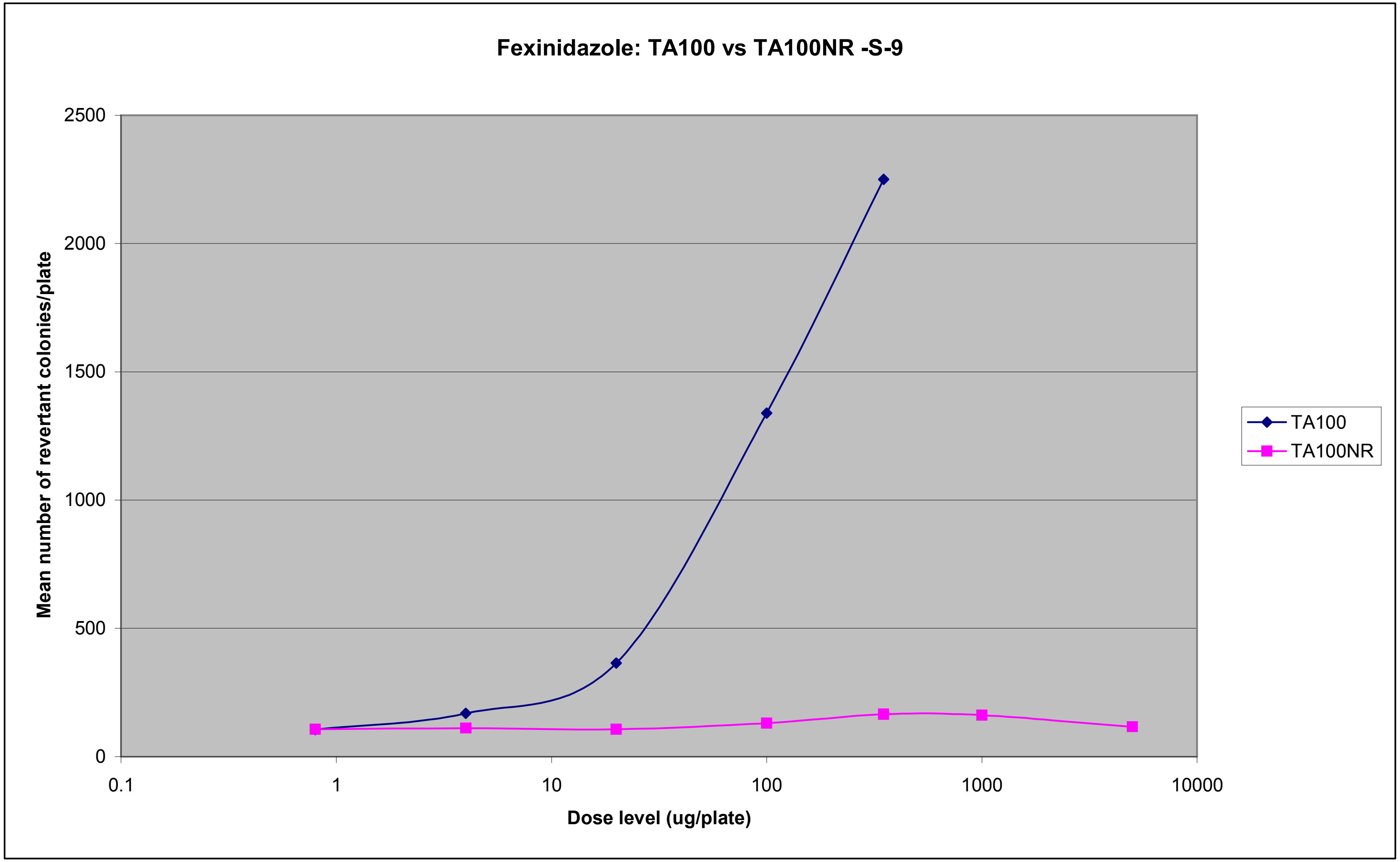
Fexinidazole: TA100 vs TA100NR -S-9
Mean number of revertant colonies/plate
2500
2000
1500
1000
500
0
0.1
1
10
100
1000
10000
Dose level (ug/plate)
TA100
TA100NR

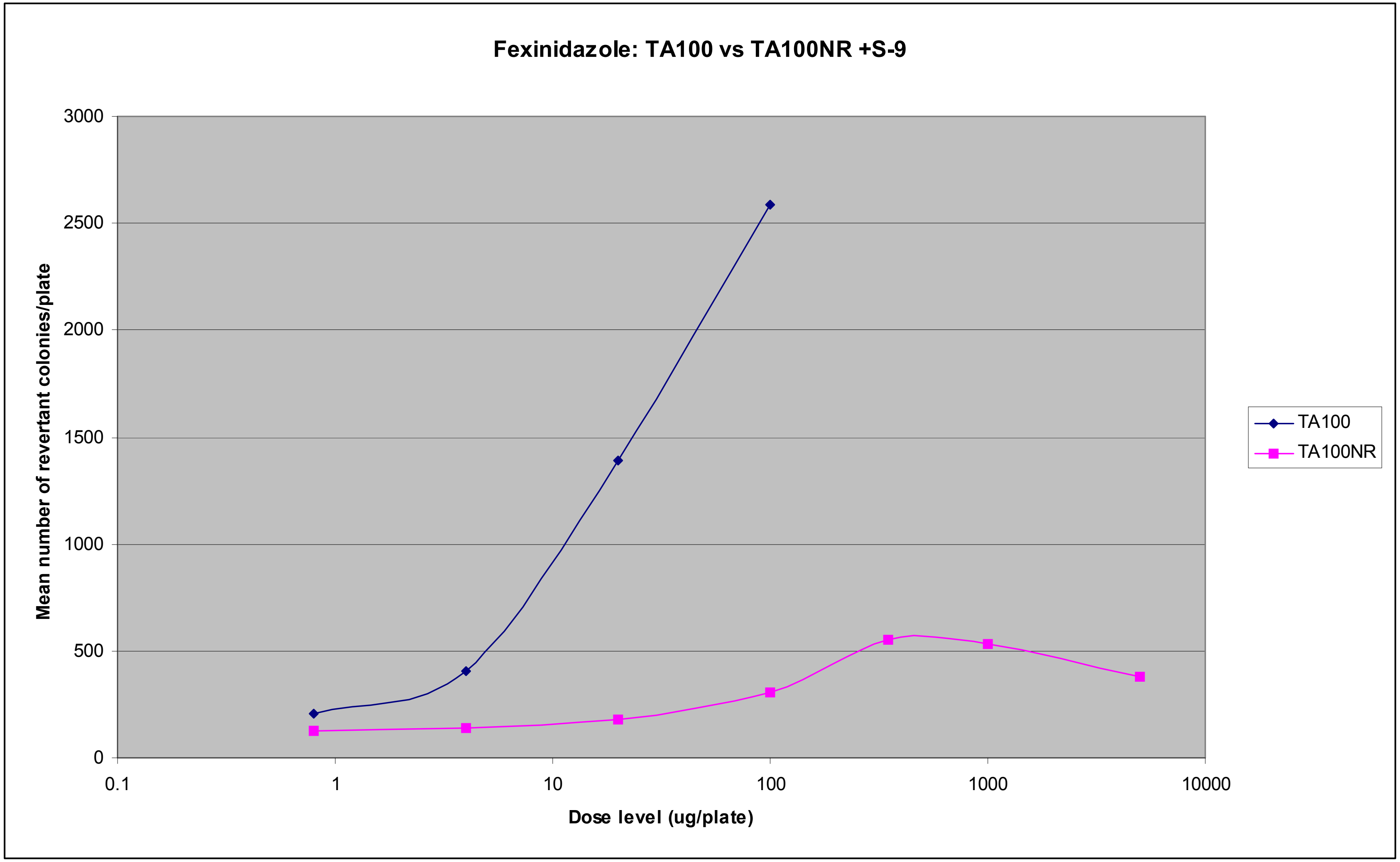
Fexinidazole: TA100 vs TA100NR +S-9
Mean number of revertant colonies/plate
3000
2500
2000
1500
1000
500
0
0.1
1
10
100
1000
10000
Dose level (ug/plate)
TA100
TA100NR
