# Supplementary material for: Fexinidazole – A New Oral Nitroimidazole Drug Candidate Entering Clinical Development for the Treatment of Sleeping Sickness
Source: PLoS Negl Trop Dis. 2010 Dec 21;4(12):e923. doi: 10.1371/journal.pntd.0000923 (PMC3006138; doi:10.1371/journal.pntd.0000923)
Supplement: Dataset S19 — (0.41 MB PDF) [file pntd.0000923.s020.pdf]

Final Report

Study Title	Induction of micronuclei in cultured human peripheral blood lymphocytes
Test Article	Fexinidazole
Author	
Sponsor	Drugs for Neglected Diseases Institute (DNDi)<br>1 place Saint Gervais<br>1201 Geneva<br>SWITZERLAND
Study Monitor	
Test Facility	Covance Laboratories Ltd
Covance Study Number	2647/22
Report Issued	June 2008
Page Number	1 of 41

COVANCE
THE DEVELOPMENT SERVICES COMPANY

CONTENTS

STUDY DIRECTOR AUTHENTICATION AND GLP COMPLIANCE STATEMENT 2

QUALITY ASSURANCE STATEMENT 3

RESPONSIBLE AND REVIEWING SCIENTISTS' STATEMENTS 4

RESPONSIBLE PERSONNEL 5

ARCHIVE STATEMENT 6

CONTENTS 7

SUMMARY 8

INTRODUCTION 11

MATERIALS 13
- Test article 13
- Analysis of achieved concentration 14
- Controls 14
- Metabolic activation system 14
- Blood cultures 15

METHODS 16
- Harvesting 17
- Slide preparation 17
- Selection of concentrations for micronucleus analysis 17
- Slide analysis 19
- Analysis of results 20

RESULTS 22
- Micronucleus analysis 24
- Analysis of achieved concentration 25

CONCLUSION 26

REFERENCES 27

APPENDICES 30
- Appendix 1 Binucleate cells with micronuclei 30
- Appendix 2 Statistical analysis of test article data 34
- Appendix 3 Historical vehicle control ranges for the human peripheral blood lymphocyte micronucleus assay 36
- Appendix 4 Quality control statement for S-9 38
- Appendix 5 Manufacturer's certificate of analysis 39
- Appendix 6 Analytical determination of achieved concentrations 40
- Appendix 7 Minor deviations from protocol 41

SUMMARY

Fexinidazole was tested in an *in vitro* micronucleus assay using duplicate human lymphocyte cultures prepared from the pooled blood of two female donors in two independent experiments. Treatments covering a broad range of concentrations, separated by narrow intervals, were performed both in the absence and presence of metabolic activation (S-9) from Aroclor 1254 induced animals. The test article was formulated in sterile anhydrous analytical grade dimethyl sulphoxide (DMSO) and the highest concentration used, 220.0 µg/mL, was selected in conjunction with the Study Monitor, based on a preliminary study (Covance Study Number 2647/4).

Treatments were conducted (as detailed in the following summary tables) either 24 or 48 hours following mitogen stimulation by Phytohaemagglutinin (PHA). The test article concentrations for micronucleus analysis were selected by evaluating the effect of Fexinidazole on the replication index (RI). In each experiment, micronuclei were analysed at three concentrations and a summary of the micronucleus data is presented in the following tables (Table 1 and Table 2):

Table 1: Experiment 1 (24 hour PHA) – Results summary

Treatment	Concentration (µg/mL)	Cytotoxicity (%)	Mean MNBN cell frequency (%)	Historical Control Range (%) #	Statistical significance
20+28 hour -S-9	Vehicle [a]	-	0.75	0.2-1.7	-
	20.00	19	0.50		NS
	40.00	0	0.40		NS
	80.00	32♦	0.70		NS
	*NQO, 5.00	ND	10.80		$p \leq 0.001$
	*Vinblastine, 0.08	ND	3.20		$p \leq 0.001$
3+45 hour +S-9	Vehicle [a]	-	0.40	0.2-1.4	-
	80.00	27	0.50		NS
	140.0	43	0.55		NS
	180.0	47♦	0.75		NS
	*CPA, 6.25	ND	3.60		$p \leq 0.001$

♦ The highest concentrations selected were the maximum practicable concentrations limited by precipitation observed at the end of the treatment period
[a] Vehicle control was DMSO
* Positive control
#95th percentile calculated range
NS = not significant
ND = not determined

Table 2: Experiment 2 (48 hour PHA) – Results summary

Treatment	Concentration (µg/mL)	Cytotoxicity (%)	Mean MNBN cell frequency (%)	Historical Control Range (%) #	Statistical significance
20+28 hour -S-9	Vehicle [a]	-	1.05	0.0-1.0	-
	40.00	4	1.10		NS
	80.00	23	0.60		NS
	100.0	29♦	0.85		NS
	*NQO, 5.00	ND	8.95		$p \leq 0.001$
	*Vinblastine, 0.06	ND	5.60		$p \leq 0.001$
3+45 hour +S-9	Vehicle [a]	-	0.90	0.0-0.9	-
	80.00	2	0.60		NS
	140.0	13	1.20		NS
	180.0	8♦	0.35		NS
	*CPA, 6.25	ND	12.85		$p \leq 0.001$

♦ The highest concentrations selected were the maximum practicable concentrations limited by precipitation observed at the end of the treatment period
[a] Vehicle control was DMSO
* Positive control
#95th percentile calculated range
NS = not significant
ND = not determined

Appropriate negative (vehicle) control cultures were included in the test system in both experiments under each treatment condition. The proportion of micronucleated binucleate cells (MNBN) in these cultures fell within or very close to current historical vehicle control (normal) ranges. 4-Nitroquinoline 1-oxide (NQO) and Vinblastine (VIN) were employed as clastogenic and aneugenic positive control chemicals respectively in the absence of rat liver S-9. Cyclophosphamide (CPA) was employed as a clastogenic positive control chemical in the presence of rat liver S-9. Cells receiving these were sampled in each experiment at 48 hours after the start of treatment; all compounds induced statistically significant increases in the proportion of cells with micronuclei.

Treatment of cells with Fexinidazole in the absence and presence of metabolic activation (S-9) in both experiments resulted in frequencies of MNBN which were similar to and not significantly ($p \leq 0.05$) different from those observed in concurrent vehicle controls for all concentrations analysed. The MNBN cell frequency of Fexinidazole treated cultures generally fell within the 95th percentile of the normal range, with the exception of single cultures in Experiment 2 at the lowest concentration analysed (40.00 µg/mL) in the absence of S-9 and at the intermediate concentration (140.0 µg/mL) in the presence of S-9. As the effect was observed only in single cultures in one experiment, there was no evidence of reproducibility within

or between experiments and the study plan criteria for a positive result were not fulfilled. These observations were therefore not considered biologically relevant.

Samples of the highest Fexinidazole concentration (22.00 mg/mL) were analysed to determine achieved concentration (Experiment 2 only). The achieved concentration was higher than the nominal 22.00 mg/mL (115, 115, 114% of nominal for the three runs performed). As these values exceed the 100+/-10% acceptance criteria, the reserve sample was analysed. The values for the reserve sample were 88 and 88% of nominal (there was insufficient sample for a third run). However, as the achieved concentrations were only marginally outside the +/-10% range on each occasion and the highest concentration for analysis was limited by precipitation observed at the end of the treatment period, these values were considered acceptable and it is considered that this had no impact upon the validity of the study in any way.

It is concluded that Fexinidazole did not induce micronuclei in cultured human peripheral blood lymphocytes when tested under two different experimental conditions up to the maximum practicable concentration (limited by solubility in culture medium), in both the absence and presence of S-9.

INTRODUCTION

Chromosome defects are recognised as the basis of a number of human genetic diseases [1]. There is a large database on the use of chromosomal assays for screening purposes ([2], [3], [4]). The use of human peripheral blood lymphocytes is recommended because the cells are only used in short-term culture and maintain a stable karyotype [5]. Experiments with these cells can also be performed in conjunction with a rat liver metabolising system (S-9) since, for short incubation periods, no toxicity is induced by the liver homogenate itself.

An alternative to measuring structural aberrations in mitotic cells is to measure micronuclei. These are produced from whole chromosomes or acentric fragments that are unable to attach to the spindle at mitosis and appear during the next interphase as small darkly staining bodies adjacent to the main daughter nucleus. Cytochalasin B, if added to cultures, inhibits cytokinesis (cell division) but not karyokinesis (nuclear division) resulting in the formation of binucleate cells [6]. If micronuclei are counted in binucleate cells, then a measurement of micronucleus induction resulting from cell division can be obtained.

In-vitro micronucleus assays in human peripheral blood lymphocytes have been used extensively at Covance and historical vehicle control data have been accumulated for male and female donors.

In the first instance, cells were exposed to the test article for 20 hours in the absence of S-9 (from rats induced with Aroclor) and for 3 hours in the presence of S-9. The test chemical was added 24 hours following culture initiation and cells were harvested at 72 hours.

Clear positive responses in Experiment 1 were to be discussed with the Sponsor and may not have necessitated further testing in Experiment 2. Negative or equivocal results in Experiment 1 were to be followed by further testing in a second experiment. In this study, a second experiment was performed in which cells were treated at 48 hours following culture initiation and harvested at 96 hours. Again, treatment in the absence of S-9 was for 20 hours and in the presence of S-9 for 3 hours.

The objective of this study was to evaluate the clastogenic and aneugenic potential of Fexinidazole by examining its effects on the frequency of micronuclei in cultured human peripheral blood lymphocytes treated in the absence and presence of S-9.

The test methodology is based on the recommendations of the International Workshop on Genotoxicity Testing [7], draft OECD guideline [8] and accepted scientific/regulatory principles described in current guidelines for clastogenicity testing *in vitro* ([3], [4], [9], [10], [11], [12], [13], [14], [15], [16]).

This study was performed according to the protocol and one amendment with the exception of the minor deviations detailed in Appendix 7, none of which in any way prejudiced the validity of the study.

The study was initiated on 22 January 2008. Experimental work started on 22 January 2008 and was completed on 25 February 2008. The study completion date is considered to be the date the Study Director signs the final report.

MATERIALS

Test article

Fexinidazole, batch number 3168-07-01/O (see minor deviations from protocol, Appendix 7), was a yellow powder. It was received on 21 January 2008 and stored at room temperature in the dark. Purity was stated as 100.2% and the expiry date was given as October 2008. The Manufacturer's certificate of analysis, provided by the Sponsor, is presented in Appendix 5. The test article information and certificate of analysis provided by the Sponsor are considered an adequate description of the characterisation, purity and stability of the test article. Determinations of stability and characteristics of the test article were the responsibility of the Sponsor.

Preliminary solubility data (taken from a previous study, CLE Study Number 2647/4) indicated that Fexinidazole was soluble in sterile anhydrous analytical grade dimethyl sulphoxide (DMSO) at concentrations up to approximately 276.2 mg/mL. The solubility limit in culture medium was in the range of 172.6 to 345.3 µg/mL, as indicated by precipitation at the higher concentration which persisted for at least 20 hours after test article addition. The highest concentration used in this study, 220.0 µg/mL, was selected in conjunction with the Study Monitor, based on Covance Study Number 2647/4.

Test article stock solutions were prepared by formulating Fexinidazole under yellow light conditions in DMSO, with the aid of vortex mixing and warming at 37°C, to give the maximum concentrations as specified in Table 3. Subsequent dilutions were made using DMSO. The test article solutions were protected from light and used within approximately 4 hours of initial formulation as follows:

Table 3: Fexinidazole Concentration Ranges Tested

Experiment	Treatment	Concentration range (mg/mL)			Final concentration range (µg/mL)		
1 and 2	20+28, -S-9, 24 hour PHA	2.000	to	10.00	20.00	to	100.0
	3+45, +S-9, 24 hour PHA	8.000	to	22.00	80.00	to	220.0

Analysis of achieved concentration

Duplicate samples of the highest test article formulation (22.00 mg/mL) and vehicle control taken from Experiment 2 were retained for analysis of achieved concentration. Samples were analysed as soon as possible on the day of formulation by Covance Laboratories Ltd. The analytical methods used and results obtained are presented in Appendix 6.

Controls

Sterile DMSO was added to cultures designated as negative controls as described in the methods section of this report.

Table 4: Positive Controls

Chemical**	Stock concentration (mg/mL)*	Final concentration (µg/mL)	S-9
4-Nitroquinoline 1-oxide	0.250	2.50	-
(NQO)	0.500	5.00	-
Cyclophosphamide	0.625	6.25	+
(CPA)	1.25	12.5	+
Vinblastine	0.006	0.06	-
(VIN)	0.008	0.08	-

* The positive control chemicals, CPA and NQO were dissolved in DMSO, frozen (-80°C nominal) and thawed immediately prior to use. Vinblastine (VIN) was dissolved in purified water immediately prior to use.

** Obtained from Sigma-Aldrich Chemical Co, Poole, UK.

Metabolic activation system

The mammalian liver post-mitochondrial fraction (S-9) used for metabolic activation was obtained from Molecular Toxicology Incorporated, USA where it is prepared from male Sprague Dawley rats induced with Aroclor 1254. The batches of MolTox™ S-9 were stored frozen in aliquots at -80°C nominal prior to use. Each batch was checked by the manufacturer for sterility, protein content, ability to convert known promutagens to bacterial mutagens and cytochrome P-450-catalyzed enzyme activities (alkoxyresorufin-O-dealkylase activities). The quality control statement, relating to the batch of S-9 preparation used, is included in Appendix 4 of this report.

The S-9 mix was prepared in the following way:

Glucose-6-phosphate (180 mg/mL), β-Nicotinamide adenine dinucleotide phosphate (NADP) (25 mg/mL), Potassium chloride (KCl) (150 mM) and rat liver S-9 were mixed in the ratio 1:1:1:2. For all cultures treated in the presence of S-9, an aliquot of

the mix was added to each cell culture to achieve the required final concentration of test article in a total of 10 mL. The final concentration of the liver homogenate in the test system was 2%.

Cultures treated in the absence of S-9 received an equivalent volume of KCl (150 mM).

Blood cultures

Blood from two healthy, non-smoking female volunteers was used for each experiment in this study:

Table 5: Blood Cultures

Experiment	Donor Sex	Donor Age (years)	Donor Identity
1	Female	26, 28	7619, 9679
2	Female	25, 35	7069, 7811

No volunteer was suspected of any virus infection or exposed to high levels of radiation or hazardous chemicals. The measured cell cycle time of the donors used at Covance falls within the range 13 +/- 1.5 hours. For each experiment, an appropriate volume of whole blood was drawn from the peripheral circulation into heparinised tubes within two days of to culture initiation. Blood was stored refrigerated and pooled using equal volumes from each donor prior to use.

Whole blood cultures were established in sterile disposable centrifuge tubes by placing 0.4 mL of pooled heparinised blood into 9.0 mL HEPES-buffered RPMI medium containing 20% (v/v) heat inactivated foetal calf serum and 50 µg/mL gentamycin, so that the final volume following addition of S-9 mix/KCl and the test article in its chosen vehicle is 10 mL. The mitogen Phytohaemagglutinin (PHA, reagent grade) was included in the culture medium at a concentration of approximately 2% of culture to stimulate the lymphocytes to divide. Blood cultures were incubated at 37°C±1°C and rocked continuously.

For treatments conducted as part of Experiment 1, blood cultures were incubated in the presence of PHA for approximately 24 hours prior to treatment.

For treatments conducted as part of Experiment 2, blood cultures were incubated in the presence of PHA for approximately 48 hours prior to treatment.

METHODS

The test system was suitably labelled (using a colour-coded procedure) to clearly identify the study number, experiment number, treatment time, test article concentration (if applicable), positive and negative controls.

S-9 mix or KCl (0.5 mL per culture) was added appropriately. Cultures were treated with the test article or controls (0.1 mL per culture) as indicated in the following table. The final culture volume was 10 mL. Cultures were incubated at 37°C ± 1°C for the designated exposure time.

This scheme is illustrated as follows:

Table 6: Treatment Scheme

Treatment	S-9	Number of cultures Experiment 1		Experiment 2	
		3+45*	20+28*	3+45*	20+28*
Negative	-		4		4
control	+	4		4	
Test article	-		2		2
	+	2		2	
Positive	-		2#		2#
controls	+	2		2	

* Hours treatment + hours recovery
\# Two cultures for each positive control.

For removal of the test chemical, cells were pelleted (approximately 300 *g*, 10 minutes), washed twice with sterile saline (pre-warmed to approximately 37°C), and resuspended in fresh pre-warmed medium containing foetal calf serum and gentamycin (see minor deviations from protocol, Appendix 7). At the appropriate times, Cytochalasin B, formulated in DMSO, was either added directly (0.1 mL/culture) or added to post wash-off culture medium to give a final concentration of 6 µg/mL per culture.

Table 7: Summary of treatment conditions

Duration of treatment (hours)	S-9	Hours after culture initiation			
		Addition of test chemical	Removal of test chemical	Addition of Cytochalasin B	Harvest time
20	-	24	44	45*	72
20	-	48	68	69*	96
3	+	24	27	45*	72
3	+	48	51	69*	96

* Approximate times

Changes in osmolality of more than 50 mOsm/kg and fluctuations in pH of more than one unit may be responsible for an increase in chromosome aberrations ([17], [18]). Osmolality and pH measurements on post-treatment incubation medium were taken in Experiment 1.

Harvesting

At the defined sampling time, cultures were centrifuged at approximately 300 *g* for 10 minutes, the supernatant removed and discarded and cells resuspended in 4 mL (hypotonic) 0.075 M KCl at 37°C for 4 minutes to allow cell swelling to occur. Cells were then fixed by dropping the KCl suspension into fresh, cold methanol/glacial acetic acid (3:1, v/v). The fixative was changed by centrifugation (approximately 300 *g*, 10 minutes) and resuspension. This procedure was repeated as necessary (centrifuging at approximately 1250 *g*, 2-3 minutes) until the cell pellets were clean.

Slide preparation

Lymphocytes were kept in fixative at 1-10°C before slides were prepared but slides were not made on the day of harvest to ensure cells were adequately fixed. Cells were centrifuged and resuspended in a minimal amount of fresh fixative (if required) to give a milky suspension. Several drops of suspension were gently spread onto multiple clean, dry microscope slides. After the slides had dried the cells were stained for 5 minutes in filtered 4% (v/v) Giemsa in pH 6.8 buffer. The slides were rinsed, dried and mounted with coverslips.

Selection of concentrations for micronucleus analysis

Slides were examined, uncoded, for proportions of mono-, bi- and multinucleate cells to a minimum of 500 cells per culture.

The Replication Index (RI), which indicates the relative number of nuclei compared to controls, was determined using the formulae below:

$$RI = \frac{\textit{number binucleate cells} + 2(\textit{number multinucleate cells})}{\textit{total number of cells in treated cultures}}$$

Relative RI (expressed in terms of percentage) for each treated culture was calculated as follows:

$$\textit{Relative RI (\%)} = \frac{\textit{RI of treated cultures}}{\textit{RI of vehicle controls}} \quad x100$$

Cytotoxicity (%) is expressed as (100 – Relative RI).

A selection of random fields was observed from enough treatments to determine whether chemically induced cell cycle delay or cytotoxicity has occurred.

The highest concentration for micronucleus analysis was to be one at which at least 60% (approximately) reduction in RI had occurred or should be the highest concentration tested.

In this study, the highest concentrations selected for analysis were the maximum practicable concentrations based on precipitation observed at the end of the treatment period. Slides from precipitating cultures were checked to confirm that the presence of precipitate did not preclude analysis. Slides from the highest selected concentration and two lower concentrations were taken for microscopic analysis.

The rationale for the limit of approximately 60% cytotoxicity is based on limited data (discussed by the IWGTP [7]) which show that in some cases (i.e. some aneugens), a very steep toxicity curve is observed and very closely spaced concentrations in the range of 50-60% toxicity need to be evaluated. Data from certain validation experiments have demonstrated that the lowest observed effective dose (LOEDs) for the aneugens diethylstilbestrol and vincristine showed a relative cell count (RCC) of 42% and 43% respectively; this corresponding to a toxicity of approximately 60%. These compounds might not have been found to be micronucleus inducers if they had not been tested up to the 60% toxicity level.

For each treatment regime, two vehicle control cultures were analysed for micronuclei. Positive control concentrations, which gave satisfactory responses in terms of quality and quantity of binucleated cells and numbers of micronuclei, were analysed.

Slide analysis

Slides from the CPA, NQO and Vinblastine positive control treatments were checked to ensure that the system had operated satisfactorily. All slides for analysis were coded, using randomly generated letters, by an individual not connected with the scoring of the slides. Labels with only the study number, experiment number, the sex of the donor and the code were used to cover treatment details on the slides.

One thousand binucleate cells from each culture (2000 per concentration) were analysed for micronuclei. The number of cells containing micronuclei and the number of micronuclei per cell on each slide was noted. Observations were recorded on raw data sheets. The microscope stage co-ordinates of the first six micronucleated cells were recorded.

Binucleate cells were only included in the analysis if all of the following criteria were met:

1) The cytoplasm remained essentially intact, and

2) The daughter nuclei were of approximately equal size.

A micronucleus was only recorded if it met the following criteria:

1) The micronucleus has the same staining characteristics and a similar morphology to the main nuclei, and

2) Any micronucleus present was separate in the cytoplasm or only just touching a main nucleus, and

3) micronuclei were smooth edged and smaller than approximately one third the diameter of the main nuclei.

Slide analysis was performed by competent analysts trained in the applicable Covance Laboratories Harrogate (CLEH) standard operating procedures. The analysts were physically located remote from the CLEH facility, but were subject to CLEH management and GLP control systems (including QA inspection). All slides and raw data generated by the remote analysts were returned to CLEH for archiving on completion of analysis.

Analysis of results

Treatment of data

After completion of scoring and decoding of slides, the numbers of binucleate cells with micronuclei (MNBN cells) in each culture were obtained.

The proportions of MNBN cells in each replicate were used to establish acceptable heterogeneity between replicates by means of a binomial dispersion test [19].

The proportion of MNBN cells for each treatment condition were compared with the proportion in negative controls by using Fisher's exact test [19]. Probability values of $p \leq 0.05$ were accepted as significant. Additionally, the number of micronuclei per binucleate cell were obtained and recorded.

Acceptance criteria

The assay was to be considered valid if the following criteria were met:

1. The binomial dispersion test demonstrated acceptable heterogeneity (in terms of MNBN cell frequency) between replicate cultures, particularly where no positive responses were seen

2. The frequency of MNBN cells in negative controls fell within the normal ranges

3. The positive control chemicals induced statistically significant increases in the proportion of MNBN cells

4. A minimum of 50% of cells had gone through at least one cell division (as measured by binucleate + multinucleate cell counts) in negative control cultures at the time of harvest.

Evaluation criteria

For valid data, the test article was considered to induce clastogenic and/or aneugenic events if:

1. A statistically significant increase in the frequency of MNBN cells at one or more concentrations was observed

2. An incidence of MNBN cells at such a concentration that exceeded the normal range in both replicates was observed

3. A concentration-related increase in the proportion of MNBN cells was observed.

The test article was considered as positive in this assay if all of the above criteria were met.

The test article was considered as negative in this assay if none of the above criteria were met.

Results which only partially satisfied the above criteria were dealt with on a case-by-case basis. Evidence of a concentration-related effect was considered useful but not essential in the evaluation of a positive result [20]. Biological relevance was taken into account, for example consistency of response within and between concentrations and (where applicable) between experiments, or effects occurring only at high or very toxic concentrations.

RESULTS

The results of the RI determinations from Experiment 1 were as follows:

Table 8: Data for 20+28 hour treatments -S-9, 24 hour PHA, female donors

Dose (µg/mL)	Replicate	Mono	Bi	Multi	Total Number of Cells	RI	Cytotoxicity (%)
Vehicle	A	204	218	78	500	0.75	-
	B	227	187	86	500	0.72	
	C	204	224	72	500	0.74	
	D	160	221	119	500	0.92	
**20.00**	**A**	**262**	**168**	**70**	**500**	**0.62**	**19#**
	**B**	**244**	**189**	**67**	**500**	**0.65**	
**40.00**	**A**	**140**	**280**	**80**	**500**	**0.88**	**0#**
	**B**	**146**	**199**	**155**	**500**	**1.02**	
**80.00**	**A**	**288**	**194**	**18**	**500**	**0.46**	**32#PEH♦**
	**B**	**248**	**207**	**45**	**500**	**0.59**	
100.0	A	269	188	43	500	0.55	34PEH
	B	286	184	30	500	0.49	

P Indicates precipitation observed at the beginning of treatment
E Indicates precipitation observed at the end of the treatment incubation period
H Indicates precipitation observed at harvest

# = Highlighted concentrations were selected for analysis

♦ The highest concentration selected was the maximum practicable concentration limited by precipitation observed at the end of the treatment period

Table 9: Data for 3+45 hour treatments +S-9, 24 hour PHA female donors

Dose (µg/mL)	Replicate	Mono	Bi	Multi	Total Number of Cells	RI	Cytotoxicity (%)
Vehicle	A	254	166	80	500	0.65	-
	B	250	173	77	500	0.65	
	C	177	218	105	500	0.86	
	D	180	217	103	500	0.85	
**80.00**	**A**	**282**	**172**	**46**	**500**	**0.53**	**27#P**
	**B**	**270**	**175**	**55**	**500**	**0.57**	
**140.0**	**A**	**322**	**137**	**41**	**500**	**0.44**	**43#P**
	**B**	**332**	**129**	**39**	**500**	**0.41**	
**180.0**	**A**	**342**	**138**	**20**	**500**	**0.36**	**47#PEH♦**
	**B**	**307**	**169**	**24**	**500**	**0.43**	
220.0	A	309	172	19	500	0.42	48PEH
	B	332	153	15	500	0.37	

P Indicates precipitation observed at the beginning of treatment
E Indicates precipitation observed at the end of the treatment incubation period
H Indicates precipitation observed at harvest

# = Highlighted concentrations were selected for analysis

♦ The highest concentration selected was the maximum practicable concentration limited by precipitation observed at the end of the treatment period

The results of the RI determinations from Experiment 2 were as follows:

Table 10: Data for 20+28 hour treatments -S-9, 48 hour PHA, female donors

Dose (µg/mL)	Replicate	Mono	Bi	Multi	Total Number of Cells	RI	Cytotoxicity (%)
Vehicle	A	102	281	117	500	1.03	-
	B	97	293	110	500	1.03	
	C	104	263	133	500	1.06	
	D	106	270	124	500	1.04	
20.00	A	98	240	162	500	1.13	0
	B	90	241	169	500	1.16	
**40.00**	**A**	**129**	**252**	**119**	**500**	**0.98**	**4#**
	**B**	**113**	**266**	**121**	**500**	**1.02**	
**80.00**	**A**	**174**	**264**	**62**	**500**	**0.78**	**23#P**
	**B**	**160**	**271**	**69**	**500**	**0.82**	
**100.0**	**A**	**178**	**283**	**39**	**500**	**0.72**	**29#PEH♦**
	**B**	**165**	**291**	**44**	**500**	**0.76**	

P Indicates precipitation observed at the beginning of treatment
E Indicates precipitation observed at the end of the treatment incubation period
H Indicates precipitation observed at harvest

# = Highlighted concentrations were selected for analysis

♦ The highest concentration selected was the maximum practicable concentration limited by precipitation observed at the end of the treatment period

Table 11: Data for 3+45 hour treatments +S-9, 48 hour PHA, female donors

Dose (µg/mL)	Replicate	Mono	Bi	Multi	Total Number of Cells	RI	Cytotoxicity (%)
Vehicle	A	102	222	176	500	1.15	-
	B	100	226	174	500	1.15	
	C	94	244	162	500	1.14	
	D	82	229	189	500	1.21	
**80.00**	**A**	**106**	**215**	**179**	**500**	**1.15**	**2#P**
	**B**	**101**	**231**	**168**	**500**	**1.13**	
**140.0**	**A**	**141**	**219**	**140**	**500**	**1.00**	**13#PEH♦**
	**B**	**128**	**231**	**141**	**500**	**1.03**	
**180.0**	**A**	**111**	**227**	**162**	**500**	**1.10**	**8#PEH♦**
	**B**	**121**	**239**	**140**	**500**	**1.04**	
220.0	A	189	210	101	500	0.82	25PEH
	B	162	221	117	500	0.91	

P Indicates precipitation observed at the beginning of treatment
E Indicates precipitation observed at the end of the treatment incubation period
H Indicates precipitation observed at harvest

Highlighted concentrations were selected for analysis

♦ The highest concentration selected was the maximum practicable concentration limited by precipitation observed at the end of the treatment period. Although the two highest concentrations selected for analysis exhibited precipitation at the end of the treatment period, the slides were checked and it was confirmed that the presence of precipitation did not preclude analysis

No significant changes in osmolality or pH were observed at the highest concentrations tested as compared to the concurrent vehicle controls when measured in Experiment 1 (individual data not reported).

Micronucleus analysis

Raw data

The raw data for the observations on the test article plus positive and negative controls are retained by Covance Laboratories Limited. A summary of the number of cells containing micronuclei is given in Appendix 1.

Validity of study

The data in Appendix 1, Appendix 2 and Appendix 3 indicate that:

1) The binomial dispersion test generally demonstrated acceptable heterogeneity (in terms of MNBN cell frequency) between replicate cultures (Appendix 2), with the exception of Experiment 1 in the absence of S-9, where sporadic differences between replicate cultures were observed. As all MNBN frequencies fell within the normal range, this was not considered to affect the integrity of the study in any way

2) The frequency of MNBN cells in the majority of vehicle controls fell within the normal range. The MNBN cell frequency of single vehicle control cultures in the absence and presence of S-9 in Experiment 2 marginally exceeded the normal range (Appendix 3). However, as these increases were small and the majority of test article treated cultures exhibited MNBN cell values similar or lower than observed in the concurrent vehicle controls, there was clearly no test article related effect. As such, the vehicle control data were accepted as valid

3) The positive control chemicals induced statistically significant increases in the proportion of MNBN cells (Appendix 1)

4) A minimum of 50% of cells had gone through at least one cell division (as measured by binucleate + multinucleate cell counts) in negative control cultures at the time of harvest.

Analysis of data

Treatment of cells with Fexinidazole in the absence and presence of metabolic activation (S-9) in both experiments resulted in frequencies of MNBN which were

similar to and not significantly ($p \leq 0.05$) different from those observed in concurrent vehicle controls for all concentrations analysed (Appendix 1 and Appendix 2). The MNBN cell frequency of Fexinidazole treated cultures generally fell within the 95th percentile of the normal range, with the exception of single cultures in Experiment 2 at the lowest concentration analysed (40.00 µg/mL) in the absence of S-9 and at the intermediate concentration (140.0 µg/mL) in the presence of S-9 (Appendix 3). As the effect was observed only in single cultures in one experiment, there was no evidence of reproducibility within or between experiments and the study plan criteria for a positive result were not fulfilled. These observations were therefore not considered biologically relevant.

Analysis of achieved concentration

Samples of the highest Fexinidazole concentration (22.00 mg/mL) were analysed to determine achieved concentration (Experiment 2 only). The results are presented in Appendix 6. The achieved concentration was higher than the nominal 22.00 mg/mL (115, 115, 114% of nominal for the three runs performed). As these values exceeded the 100+/-10% acceptance criteria, the reserve sample was analysed. The values for the reserve sample were 88 and 88% of nominal (there was insufficient sample for a third run). However, as the achieved concentrations were only marginally outside the +/-10% range on each occasion and the highest concentration for analysis is limited by precipitation observed at the end of the treatment period, these values were considered acceptable and it is considered that this had no impact upon the validity of the study in any way.

CONCLUSION

It is concluded that Fexinidazole did not induce micronuclei in cultured human peripheral blood lymphocytes when tested under two different experimental conditions at concentrations up to the maximum practicable concentration (limited by solubility in culture medium), in both the absence and presence of S-9.

REFERENCES

1 Mitelman F (1991) "Catalogue of Chromosome Aberrations in Cancer, 4th ed". New York: Wiley-Liss

2 Preston R J, Au W, Bender M A, Brewen J G, Carrano A V, Heddle J A, McFee A F, Wolff S and Wassom J S (1981) Mammalian *in vivo* and *in vitro* cytogenetic assays. A report of the U.S. EPA's Gene-Tox Program. Mutation Research 87, 143-188

3 Fenech M (1998) Important variables that influence base-line micronucleus frequency in cytokinesis-blocked lymphocytes – a biomarker for DNA damage in human populations. Mutation Research 404, 155-165

4 Fenech M, Bonassi S, Turner J, Lando C, Ceppi M, Chang W P, Holland N, Kirsch-Volders M, Zeiger E, Bigatti M P, Bolognesi C, Cao J, De Luca G, Di Giorgio M, Ferguson L R, Fucic A, Lima O G, Hadjidekova VV, Hrelia P, Jaworska A, Joksic G, Krishnaja A P, Lee T K, Martelli A, McKay M J, Migliore L, Mirkova E, Muller W U, Odagiri Y, Orsiere T, Scarfi M R, Silva M J, Sofuni T, Suralles J, Trenta G, Vorobtsova I, Vral A and Zijno A (2003) HUman MicroNucleus project. Intra- and inter-laboratory variation in the scoring of micronuclei and nucleoplasmic bridges in binucleated human lymphocytes. Results of an international slide-scoring exercise by the HUMN project. Mutation Research 534, 45-64

5 Evans H J and O'Riordan M L (1975) Human lymphocytes for analysis of chromosome aberrations in mutagen tests. Mutation Research 31, 135-148

6 Fenech M and Morley A A (1985) Measurement of micronuclei in human lymphocytes. Mutation Research 147, 29-36

7 Kirsch-Volders M, Sofuni T, Aardema M, Albertini S, Eastmond D, Fenech M, Ishidate M, Kirchener S, Lorge E, Morita T, Norppa H, Surralles J, Vanhauwaert A, Wakata A (2003) Report from the *in vitro* micronucleus assay working group. Mutation Research 540, 153-163

8 OECD (2004) 'Genetic Toxicology: OECD Guideline for the testing of chemicals. Draft proposal for a new Guideline 487: *In vitro* micronucleus test

9 Rosefort C, Fauth E and Zankl H (2004) Micronuclei induced by aneugens and clastogens in mononucleate and binucleate cells using the cytokinesis block assay. Mutagenesis 19, 277-284

10 Elhajouji A, Cunha M, Kirsch-Volders M (1998) Spindle poisons can induce polyploidy by mitotic slippage and micronucleate mononucleates in the cytokinesis-block assay. Mutagenesis 13, 193-198

11 Migliore L and Nieri M (1991) Evaluation of twelve potential aneuploidogenic chemicals by the *in vitro* human lymphocyte micronucleus assay. Toxicology *In Vitro* 5, 325-336

12 Galloway S M, Aardema M J, Ishidate M, Ivett J L, Kirkland D J, Morita T, Mosesso P, Sofuni T (1994) Report from working group on *in vitro* tests for chromosomal aberrations. In: Sheila M. Galloway (Ed), Report of the International Workshop on Standardisation of Genotoxicity Test Procedures. Mutation Research 312, 241-261

13 Aardema M S, Albertini S, Arni P, Henderson L M, Kirsch-Volders M, Mackay J M, Sarriff A M, Stringer D A and Taalman R D F (1998) Aneuploidy: a report of an ECETOC task force. Mutation Research 410, 3-79

14 Miller B, Potter-Locher F, Seelbach A, Stopper H, Utesch D and Madle S (1998) Evaluation of the *in vitro* micronucleus test as an alternative to the *in vitro* chromosomal aberration assay: position of the GUM working group on the *in vitro* micronucleus test. Mutation Research 410, 81-116

15 European Agency for the Evaluation of Medicinal Products (1995) ICH Topic S 2 A. Genotoxicity: Guidance on Specific Aspects of Genotoxicity Tests for Pharmaceuticals. ICH Harmonised Tripartite Guideline

16 Fenech M, Holland N, Chang W P, Zeiger E, Bonassi S (1999) The HUman MicroNucleus project: an international collaborative study on the use of the micronucleus technique for measuring DNA damage in humans. Mutation Research 428, 271-283

17 Scott D, Galloway S M, Marshall R R, Ishidate M, Brusick D, Ashby J and Myhr B C (1991) Genotoxicity under extreme culture conditions. A report from ICPEMC Task Group 9. Mutation Research 257, 147-204

18 Brusick D (1986) Genotoxic effects in cultured mammalian cells produced by low pH treatment conditions and increased ion concentrations. Environ Mutagenesis 8, 879-886

19 Richardson C, Williams D A, Allen J A, Amphlett G, Chanter D O and Phillips B (1989) Analysis of data from *in vitro* cytogenetic assays. In "Statistical Evaluation of Mutagenicity Test Data", (UKEMS Guidelines Sub-committee Report, Part III), Ed D J Kirkland, Cambridge University Press, pp 141-154

20 Scott D, Dean B J, Danford N D Kirkland D J (1990) Metaphase chromosome aberration assays *in vitro*. Basic Mutagenicity Tests; UKEMS recommended procedures. Ed. Kirkland D J.

APPENDICES

Appendix 1
Binucleate cells with micronuclei

Table 12: Fexinidazole, 20+28 hour treatments in the absence of S-9
Experiment 1 - 24 hour PHA, female donors

Treatment (µg/mL)	Replicate	Total BN Cells Scored	Total MNBN Cells Scored	Frequency of MNBN Cells/ Cells Scored (%)	Significance §
Vehicle	A	1000	10	1.00	
	B	1000	5	0.50	
	Total	2000	15	0.75	-
20.00	A	1000	2	0.20	
	B	1000	8	0.80	
	Total	2000	10	0.50	NS
40.00	A	1000	3	0.30	
	B	1000	5	0.50	
	Total	2000	8	0.40	NS
80.00	A	1000	10	1.00	
	B	1000	4	0.40	
	Total	2000	14	0.70	NS
NQO, 5.00	A	1000	105	**10.50#**	
	B	1000	111	**11.10#**	
	Total	2000	216	10.80	$p \leq 0.001$
VIN, 0.08	A	1000	37	**3.70#**	
	B	1000	27	**2.70#**	
	Total	2000	64	3.20	$p \leq 0.001$

§ Statistical significance (Appendix 2)
NS = not significant
# = Numbers highlighted exceed historical negative control range (Appendix 3)

Table 13: Fexinidazole, 3+45 hour treatments in the presence of S-9 Experiment 1- 24 hour PHA, female donors

Treatment (µg/mL)	Replicate	Total BN Cells Scored	Total MNBN Cells Scored	Frequency of MNBN Cells/ Cells Scored (%)	Significance §
Vehicle	A	1000	5	0.50	
	B	1000	3	0.30	
	Total	2000	8	0.40	-
80.00	A	1000	7	0.70	
	B	1000	3	0.30	
	Total	2000	10	0.50	NS
140.0	A	1000	8	0.80	
	B	1000	3	0.30	
	Total	2000	11	0.55	NS
180.0	A	1000	11	1.10	
	B	1000	4	0.40	
	Total	2000	15	0.75	NS
CPA, 6.25	A	1000	35	**3.50#**	
	B	1000	37	**3.70#**	
	Total	2000	72	3.60	$p \leq 0.001$

§ Statistical significance (Appendix 2)
NS = not significant
[#] = Numbers highlighted exceed historical negative control range (Appendix 3)

**Table 14: Fexinidazole, 20+28 hour treatments in the absence of S-9
Experiment 2- 48 hour PHA, female donors**

Treatment (µg/mL)	Replicate	Total BN Cells Scored	Total MNBN Cells Scored	Frequency of MNBN Cells/ Cells Scored (%)	Significance §
Vehicle	A	1000	9	0.90	
	B	1000	12	**1.20#**	
	Total	2000	21	1.05	-
40.00	A	1000	12	**1.20#**	
	B	1000	10	1.00	
	Total	2000	22	1.10	NS
80.00	A	1000	4	0.4	
	B	1000	8	0.80	
	Total	2000	12	0.60	NS
100.0	A	1000	9	0.90	
	B	1000	8	0.80	
	Total	2000	17	0.85	NS
NQO, 5.00	A	1000	107	**10.70#**	
	B	1000	72	**7.20#**	
	Total	2000	179	8.95	$p \leq 0.001$
VIN, 0.06	A	1000	67	**6.70#**	
	B	1000	45	**4.50#**	
	Total	2000	112	5.60	$p \leq 0.001$

§ Statistical significance (Appendix 2)
NS = not significant
[#] = Numbers highlighted exceed historical negative control range (Appendix 3)

Table 15: Fexinidazole, 3+45 hour treatments in the presence of S-9 Experiment 2- 48 hour PHA, female donors

Treatment (µg/mL)	Replicate	Total BN Cells Scored	Total MNBN Cells Scored	Frequency of MNBN Cells/ Cells Scored (%)	Significance §
Vehicle	A	1000	8	0.80	
	B	1000	10	**1.00#**	
	Total	2000	18	0.90	-
80.00	A	1000	9	0.90	
	B	1000	3	0.30	
	Total	2000	12	0.60	NS
140.0	A	1000	16	**1.60#**	
	B	1000	8	0.80	
	Total	2000	24	1.20	NS
180.0	A	1000	4	0.40	
	B	1000	3	0.30	
	Total	2000	7	0.35	NS
CPA, 6.25	A	1000	88	**8.80#**	
	B	1000	169	**16.90#**	
	Total	2000	257	12.85	$p \leq 0.001$

§ Statistical significance (Appendix 2)
NS = not significant
# = Numbers highlighted exceed historical negative control range (Appendix 3)

Appendix 2
Statistical analysis of test article data

Table 16: Fexinidazole, 20+28 hour treatments in the absence of S-9
Experiment 1- 24 hour PHA, female donors

Binomial Dispersion Test $\chi^2 = 8.39$	DF: 4
Significance: $p \leq 0.05$	

Treatment (µg/mL)	Total BN Cells	BN Cells with micronuclei	Proportion	Fisher's exact test	Significance
Vehicle	2000	15	0.008	-	-
20.00	2000	10	0.005	0.837	NS
40.00	2000	8	0.004	0.925	NS
80.00	2000	14	0.007	0.572	NS
NQO, 5.00	2000	216	0.108	0.000	$p \leq 0.001$
VIN, 0.08	2000	64	0.032	0.000	$p \leq 0.001$

Table 17: Fexinidazole, 3+45 hour treatments in the presence of S-9
Experiment 1- 24 hour PHA, female donors

Binomial Dispersion Test $\chi^2 = 7.69$	DF: 4
Significance: NS	

Treatment (µg/mL)	Total BN Cells	BN Cells with micronuclei	Proportion	Fisher's exact test	Significance
Vehicle	2000	8	0.004	-	-
80.00	2000	10	0.005	0.323	NS
140.0	2000	11	0.006	0.251	NS
180.0	2000	15	0.008	0.075	NS
CPA, 6.25	2000	72	0.036	0.000	$p \leq 0.001$

NS = not significant DF = degrees of freedom BN = binucleate

Table 18: Fexinidazole, 20+28 hour treatment in the absence of S-9
Experiment 2- 48 hour PHA, female donors

Binomial Dispersion Test $\chi^2 = 2.02$ DF: 4
Significance: NS

Treatment (µg/mL)	Total BN Cells	BN Cells with micronuclei	Proportion	Fisher's exact test	Significance
Vehicle	2000	21	0.011	-	-
40.00	2000	22	0.011	0.440	NS
80.00	2000	12	0.006	0.940	NS
100.0	2000	17	0.009	0.740	NS
NQO, 5.00	2000	179	0.090	0.000	$p \leq 0.001$
VIN, 0.06	2000	112	0.056	0.000	$p \leq 0.001$

Table 19: Fexinidazole, 3+45 hour treatment in the presence of S-9
Experiment 2- 48 hour PHA, female donors

Binomial Dispersion Test $\chi^2 = 6.08$ DF:4
Significance: NS

Treatment (µg/mL)	Total BN Cells	BN Cells with micronuclei	Proportion	Fisher's exact test	Significance
Vehicle	2000	18	0.009	-	-
80.00	2000	12	0.006	0.860	NS
140.0	2000	24	0.012	0.179	NS
180.0	2000	7	0.004	0.986	NS
CPA, 6.25	2000	257	0.129	0.000	$p \leq 0.001$

NS = not significant DF = degrees of freedom BN = binucleate

Appendix 3
Historical vehicle control ranges for the human peripheral blood lymphocyte micronucleus assay

Table 20: 24 hour PHA

		Micronucleated binucleates observed in 1000 binucleates scored	
		Male donors	Female donors
-S9	Number of cultures	74	86
	Median	4.00	9.00
	Mean	4.55	9.26
	SD	3.03	3.97
	Observed range	0 – 16	2 – 19
	**95% reference range**	**0 – 12**	**2 –17**
+S9	Number of cultures	72	62
	Median	3.00	7.00
	Mean	3.93	6.92
	SD	2.86	3.37
	Observed range	0 – 12	1 – 17
	**95% reference range**	**0 – 12**	**2 – 14**

Reference ranges are calculated from percentiles of the observed distributions.

Calculated in August 2006 by CLEH Statistics, for studies started between January 2004 and May 2006.

Table 21: 48 hour PHA

		Micronucleated binucleates observed in 1000 binucleates scored	
		Male donors	Female donors
-S9	Number of cultures	82	70
	Median	4.00	4.00
	Mean	3.65	4.26
	SD	2.43	2.56
	Observed range	0 – 13	0 – 10
	**95% reference range**	**0 – 10**	**0 – 10**
+S9	Number of cultures	70	60
	Median	3.00	4.50
	Mean	3.60	4.50
	SD	2.19	2.70
	Observed range	0 – 11	0 – 13
	**95% reference range**	**0 – 10**	**0 – 9**

Reference ranges are calculated from percentiles of the observed distributions.

Calculated in August 2006 by CLEH Statistics, for studies started between January 2004 and May 2006.

Appendix 4
Quality control statement for S-9

MOLTOX POST MITOCHONDRIAL SUPERNATANT (S-9) QUALITY CONTROL & PRODUCTION CERTIFICATE

LOT NO.: 2159
PART NO.: 11-101
VOLUME: 5ml
SPECIES: Rat
STRAIN: Sprague Dawley
SEX: Male
TISSUE: Liver
PREPARATION DATE: June 27, 2007
EXPIRATION DATE: June 27, 2009
BUFFER: 0.154 M KCl
INDUCING AGENT(s): Aroclor 1254 (Monsanto KL615), 500 mg/kg i.p.
REFERENCE: Maron, D & Ames, B, *Mutat Res* **113**:173, 1983
STORAGE: At or below -70°C

BIOCHEMISTRY:

- PROTEIN

37.8 mg/ml

Assayed according to the method of Lowry et al., *JBC* **193**:265, 1951 using bovine serum albumin as the standard.

- ALKOXYRESORUFIN-0-DEALKYLASE ACTIVITIES

Activity	P450	Fold - Induction
EROD	IA1, IA2	61.0
PROD	2B1	31.7
BROD	2B1	12.0
MROD	1A2	306.6

Assays for ethoxyresorufin-0-deethylase (EROD), pentoxy-, benzyl- and methoxyresorufin-0-dealkylases (PROD, BROD, & MROD) were conducted using a modification of the methods of Burke, et al., *Biochem Pharm* **34**:3337, 1985. Fold-inductions were calculated as the ratio of the sample vs. uninduced specific activities (SA's). Control SA's (pmoles/min/mg protein) were 25.3, 14.3, 42.5, & 6.4 for EROD, PROD, BROD and MROD, respectively.

BIOASSAY:

- TEST FOR THE PRESENCE OF ADVENTITIOUS AGENTS

Samples of S-9 were assayed for the presence of contaminating microflora by plating 1.0 ml volumes on Nutrient Agar and Minimal Glucose (Vogel-Bonner E, supplemented with 0.05 mM L-histidine and D-biotin) media. Triplicate plates were read after 40 - 48 h incubation at 35 ± 2°C. The tested samples met acceptance criteria.

- PROMUTAGEN ACTIVATION

No. His+ Revertants	
TA98	TA1535
272	1914

The ability of the sample to activate ethidium (EtBr) EtBr/CPA/and cyclophosphamide (CPA) to intermediates mutagenic to TA98 and TA1535, respectively, was determined according to Lesca, et al., *Mutation Res* **129**:299, 1984. Data were expressed as revertants per μg EtBr or per mg CPA.

Dilutions of the sample S9, ranging from 0.2 - 10% in S9 mix, were tested for their ability to activate benzo(a)pyrene (BP) and 2-aminoanthracene (2-AA) to intermediates mutagenic to TA100. Assays were conducted using duplicate plates as described by Maron & Ames, (*Mutat Res* **113**:173, 1983).

μl S9 per plate/number *his*$^+$ revertants per plate

Promutagen	0	1	5	10	20	50
BP (5 μg)	101	288	454	525	933	1186
2-AA (2.5 μg)	137	547	2529	2643	2529	1582

MOLECULAR TOXICOLOGY, INC.
157 Industrial Park Dr.
Boone, NC 28607
(828) 264-9099

Approved: Kristie Evans 7.9.07

Appendix 5
Manufacturer's certificate of analysis

CENTIPHARM
Active for your synthesis

ANALYSIS CERTIFICATE

FEXINIDAZOLE

Manufact. date November-2007
Expiry date October-2008

DATE	January 15, 2008
ANALYSIS N° CA	07327/01
BATCH CENTIPHARM	3168-07-01/O
SAMPLE N°	ECH:08015/51
WEIGHT (g)	70

DETERMINATIONS	RESULTS	SPECIFICATIONS
IDENTIFICATION	IR Spectrum complies	IR Spectrum complies
APPEARANCE	powder	powder
COLOUR	yellow	yellow
LOSS ON DRYING (%)	0,0	<= 0,5
SULPHATED ASH (%)	0,0	<= 0,1
$HClO_4$ ASSAY (%)	100,2	98,5 à 101,5
RELATED SUBSTANCES - HPLC- Any known impurity (%)	< 0,05	<= 0,15
RELATED SUBSTANCES - HPLC- Any other impurity (%)	0,08	<= 0,10
RELATED SUBSTANCES - HPLC - All impurities sum (%)	0,1	<= 0,5
RESIDUAL SOLVENTS -GC- Acetone (ppm)	740	<= 5000
RESIDUAL SOLVENTS -GC- Methanol (ppm)	20	<= 3000
RESIDUAL SOLVENTS -GC- Toluene (ppm)	4	<= 890

CONFORMITY	YES / NO

Grasse, 16/01/2008
M.CONNAN
Quality Control Manager

AXYNTIS
CENTIPHARM
Chemin de la Madeleine - B.P. 45249 - F-06131 GRASSE CEDEX - Tél. : 33 (0)4 93 70 01 32 - Fax : 33 (0)4 93 70 05 65 - http://www.axyntis.com - contact@axyntis.com
S.A.S. au capital de 1 525 000 € - R.C.S. GRASSE 326 171 378 - APE 241 G - TVA FR 68 326 171 378

Appendix 6
Analytical determination of achieved concentrations

SUMMARY

The test article, Fexindazole, was formulated in DMSO by Genetic and Molecular Toxicology for treatment during the study. Formulations prepared on 6 February 2008 were received 6 and 7 February 2008 and were analysed to verify achieved concentration.

The analytical procedure was validated in study 2647/23.

PROCEDURES

Achieved concentration
Formulations received on 6 and 7 February 2008 were analysed in duplicate (apart from the controls where only a single analysis was performed).

Analytical procedure
The written analytical procedure Covance 2647/023-01F was used to determine achieved concentration.

Data collection and processing
The data was collected and processed using an Empower2 data capture system, version Build number 2154.

RESULTS

6 February 2008: Experiment 2

Concentration mg/mL	Results as % nominal		
22	115	115	114*

* third sample analysed due to high results

7 February 2008: Experiment 2 (Reserve)

Concentration mg/mL	Results as % nominal	
22	88	88

further analysis not possible due to insufficient sample volume

Test article was not detected in the control sample.

Appendix 7
Minor deviations from protocol

Protocol section	Subject	Deviation
Methods	Main experiments	In Experiment 1, during the medium change procedure, the cells were pelleted at approximately 1250 *g* for 2.5 minutes following the second saline wash in error. This deviation did not have any negative impact upon the results of this study.
Study specific information	Test article	The batch number in the Protocol contained a typographical error. The Protocol stated that batch number 3168-07-01/0 was to be used. The correct batch number of Fexinidazole to be used in this study was 3168-07-01/O.
Materials, Methods	Controls, osmolality and pH	Reference is made in the Protocol to a Range-Finder Experiment. Following consultation with the Study Monitor prior to the study start, it was not deemed necessary to perform a Range-Finder Experiment.
