# Supplementary material for: Fexinidazole – A New Oral Nitroimidazole Drug Candidate Entering Clinical Development for the Treatment of Sleeping Sickness
Source: PLoS Negl Trop Dis. 2010 Dec 21;4(12):e923. doi: 10.1371/journal.pntd.0000923 (PMC3006138; doi:10.1371/journal.pntd.0000923)
Supplement: Dataset S20 — (0.30 MB PDF) [file pntd.0000923.s021.pdf]

Final Report

Study Title	Induction of micronuclei in the bone marrow of treated mice
Test Article	Fexinidazole
Author	
Sponsor	Drugs for Neglected Diseases Initiative (DNDi)<br>1 place Saint Gervais<br>1201 Geneva<br>SWITZERLAND
Study Monitor	
Test Facility	Covance Laboratories Ltd
Responsible Scientist and Analytical Test Site, bioanalysis	Accelera/Pre-clinical Development<br>Nerviano Medical Sciences
Covance Study Number	2647/23
Report Issued	June 2008
Page Number	1 of 58

COVANCE
THE DEVELOPMENT SERVICES COMPANY

CONTENTS

STUDY DIRECTOR AUTHENTICATION AND GLP COMPLIANCE STATEMENT 2

QUALITY ASSURANCE STATEMENT 3

RESPONSIBLE AND REVIEWING SCIENTISTS' STATEMENTS 4

RESPONSIBLE PERSONNEL 5

ARCHIVE STATEMENT 6

CONTENTS 7

SUMMARY 9

OBJECTIVE 12

INTRODUCTION 12

EXPERIMENTAL DESIGN 13
- Regulatory test guidelines 13
- Justification for selection of the test system 13
- Test article administration 13
- Dose selection 13
- Dose levels 14
- Dose volumes 14

TEST AND CONTROL ARTICLES 15
- Test article 15
- Controls 15

TEST ARTICLE FORMULATION 16
- Preparation 16
- Stability and homogeneity 16
- Formulations analysis 17

TEST SYSTEM 18
- Species, strain and supplier 18
- Specification 18
- Environment 18
- Environmental enrichment 18
- Diet, water and bedding 19

METHODS 20
- Pre-experimental procedures 20
- Bioanalysis 20
- Experimental observations 21
- Bone marrow sampling and slide preparation 22
- Slide analysis 23
- Analysis of results 23

RESULTS 26
- Micronucleus Experiment 26
- Formulations analysis 28

Bioanalysis 28

CONCLUSION 29

REFERENCES 30

APPENDICES 32
Appendix 1 Body weights and dosages 32
Appendix 2 Summary of micronucleus data 33
Appendix 3 Micronucleus data 35
Appendix 4 Historical vehicle control data ranges 38
Appendix 5 Certificate of analysis 39
Appendix 6 Formulations analysis 40
Appendix 7 Bioanalysis 44
Appendix 8 Minor deviation from protocol 58

SUMMARY

Fexinidazole was tested for its ability to induce micronuclei in the polychromatic erythrocytes (PCE) of the bone marrow of treated mice.

From data provided by the Sponsor, a dose of 2000 mg/kg, given twice 24 hours apart, of Fexinidazole (a recommended maximum dose for *in vivo* assays, in accordance with current regulatory guidelines) was well tolerated. Therefore, the Micronucleus Experiment was performed using a maximum intended dose of 2000 mg/kg/day, with two lower intended doses of 500 and 1000 mg/kg/day. Bone marrow samples were taken 24 hours after the seconds dose.

Groups of six male mice were treated twice with the vehicle Methocel 0.5% (w/v) with 5% (v/v) Tween 80 or Fexinidazole (at the doses stated above) via oral gavage (dose volume of 10 mL/kg). A group of six male mice were treated once with the required positive control at a dose volume of 10 mL/kg on the second day of dosing. The positive control used was cyclophosphamide (CPA, 40 mg/kg in saline).

All doses were well tolerated and no clinical signs were observed. Bone marrow smears were prepared from sacrificed animals approximately 24 hours following the final administration.

In addition to the micronucleus animals, groups of satellite animals were dosed with vehicle or Fexinidazole (highest dose). Plasma was isolated from these animals and used to assess systemic exposure to Fexinidazole and its metabolites.

Results of formulation analysis confirmed that the mean achieved concentration for all doses were either within or marginally outside the required 100±10% of the nominal test article concentrations for both day 1 and day 2. Homogeneity analysis for day 1 indicated that test article formulations were not considered homogenous (coefficient of variation (CV); exceeded 6%). For day 2, test article formulations were either within or marginally outside the required CV of ≤ 6%. Formulations were however continually stirred prior to and throughout dosing, with all mice receiving the required (nominal) dose over the two days of dosing.

Negative (vehicle) control mice exhibited group mean frequencies of polychromatic erythrocytes (PCE) to normochromatic erythrocytes (NCE); ratio expressed as %PCE within historical (vehicle) control ranges. Individual frequencies of micronucleated PCE (MN PCE) were consistent with historical vehicle control distribution data.

Positive control animals exhibited increased numbers of MN PCE such that the frequency in the positive control group was significantly greater than in concurrent controls with all animals exhibiting marked increases in numbers of MN PCE.

The assay system was therefore considered valid.

Mice treated with Fexinidazole at all doses exhibited group mean %PCE that were similar to the values for the vehicle control group and which were comparable with historical control data, thus indicating no evidence of test article related bone marrow toxicity.

Mice treated with Fexinidazole resulted in a weak, but statistically significant increase in group mean MN PCE at the maximum dose compared to the concurrent vehicle control. Whilst a weak, but significant linear trend was observed, this was in the absence of a dose related response. Following additional scoring of spare slides, these initial increases were poorly replicated, with all dose groups similar to those seen in the concurrent vehicle control.

Consequently, combining the two data sets resulted in both a weak linear trend and a weak, but statistically significant increase in group mean MN PCE frequency in the maximum dose group only. This increase in MN PCE frequency was not considered biologically important, with initial increases in MN PCE frequency being poorly replicated following additional scoring. In addition, individual MN PCE frequencies for all treated animals from both the initial and additional analyses were consistent with the laboratory's historical control distribution data. Overall, these data indicated a lack of evidence for test article related effect.

Analysis of plasma samples confirmed that animals were systemically exposed to Fexinidazole and its sulfoxide and sulphone metabolites.

It is concluded that Fexinidazole did not induce micronuclei in the polychromatic erythrocytes of the bone marrow of male mice treated up to a nominal dose of 2000 mg/kg/day (a recommended maximum dose for the *in vivo* micronucleus assay according to current regulatory guidelines) under the assay conditions employed.

Whilst small, but statistically significant increases in micronuclei was observed in the high dose group, following initial slide analysis, following additional slide analysis and combing of the data sets this initial increase was small and non-reproducible. There was also no evidence of a dose-related effect, or bone marrow toxicity. As such, these small increases were considered chance events not related to test article effect.

OBJECTIVE

The objective of this study was to evaluate the potential of Fexinidazole to induce micronuclei in the polychromatic erythrocyte (PCE) of the bone marrow of treated mice.

INTRODUCTION

Chromosome defects are recognised as being the basis of a number of human genetic diseases [1]. Chemicals can be tested *in vitro* for their ability to produce chromosome aberrations in cells with which they have direct contact in a static system. Chemicals may react differently *in vivo*, however, where metabolic systems other than liver cytochrome P448/P450 operate, and where the dynamic processes of absorption, metabolism and excretion are involved. Chemicals should therefore also be tested for chromosome damaging activity *in vivo*.

In the bone marrow micronucleus test ([2], [3], [4]) erythroblasts undergoing their last chromosome replication are the target cell. Chromosome fragments or whole chromosomes, which are unable to attach to the spindle, are left behind as micronuclei when the main nucleus is extruded in the production of the PCE. Micronuclei can be clearly seen in these cells and are more easily counted than structural chromosome aberrations in metaphase cells. They may also be formed from intact chromosomes, therefore agents which affect spindle formation or function (that is, result in aneuploidy) can be detected.

This study was performed according to the protocol and two amendments with the exception of the minor deviations detailed in Appendix 8, none of which prejudiced the validity of the study.

The study was initiated on 21 January 2008. Experimental work started on 13 January 2008 and was completed on 5 March 2008. The study completion date is considered to be the date the Study Director signs the final report.

EXPERIMENTAL DESIGN

Regulatory test guidelines

The test methodology used in this study was in accordance with current literature ([5], [6]) and the protocol was designed to meet the known requirements of the OECD Guideline 474, 1997 [7] and the ICH Tripartite Harmonised Guideline on Genotoxicity: Specific Aspects of Regulatory Tests, 1995 [8].

Justification for selection of the test system

The rodent bone marrow micronucleus test in mice is recommended by various regulatory authorities as an appropriate test to determine the genotoxic potential of a compound *in vivo*. The mouse was selected because there is a large volume of background data in this species.

Test article administration

All treatments were given via oral gavage as this is the intended route of human exposure. Animals were not fasted prior to dose administration.

The test article was given as two administrations, 24 hours apart and animals were sampled 24 hours after the final administration, thus enabling examination of cells exposed to the test article over a period of 24 to 48 hours prior to sampling.

This has been shown to be of sufficient duration for the expression of any genotoxic potential ([9], [10], [11], [12]).

Dose selection

The Sponsor provided the following information on the *in vivo* toxicity of Fexinidazole.

Oral doses below 10000 mg/kg were not lethal or markedly toxic. At the maximum dose for this experiment, 2000 mg/kg, some piloerection had been noted in previous studies at this dose and occasionally at lower doses.

Based on the toxicity data provided, the Micronucleus Experiment was conducted at a maximum intended dose of 2000 mg/kg/day, the maximum dose in accordance with current guidelines ([7], [13]). Two further intended doses of 500 and 1000 mg/kg/day (equivalent to 25% and 50% of the maximum dose) were also tested.

Dose levels

The following dose levels were tested in this study:

Table 1: Dose Levels - Micronucleus Experiment

Group Number	Treatment	Dose volume (mL/kg)	Dose (mg/kg/day)	No. of animals	Sample time (hours after final administration)
1	Vehicle control[a]	10	0	6M	24
2	Fexinidazole	10	500[b]	6M	24
3	Fexinidazole	10	1000[b]	6M	24
4	Fexinidazole	10	2000[b]	6M	24
5	Positive control[c]	10	40	6M	24

M Male
a Methocel 0.5% (w/v) with 5% (v/v) Tween 80
b Mice were treated with Fexindazole at intended dose levels stated in the table. Formulation analysis however confirmed that for day 1, whilst the achieved dose was within the specified limits, all formulations on day 1 were considered not to be homogenous. For day 2, all concentrations were either marginally below the specified limits or within for both achieved concentration and homogeneity. All doses from this point have therefore been expressed as nominal dose levels administered, as confirmed by formulation analysis (Appendix 6)
c Cyclophosphamide (CPA) administered once only on day 2

Dose volumes

A dose volume of 10 mL/kg was used. Individual dose volumes were based on individual body weight.

TEST AND CONTROL ARTICLES

Test article

Fexinidazole, batch number 3168-07-01/O was a yellow powder. It was received on 21 January 2008 and stored at room temperature in the dark. Purity was stated as 100.2% and expiry date was given as October 2008. The certificate of analysis, provided by the Sponsor is given in Appendix 5. The test article information and certificate of analysis provided by the Sponsor are considered an adequate description of the characterisation, purity and stability of the test article. Determinations of stability and characteristics of the test article were the responsibility of the Sponsor.

Controls

The negative (vehicle) control group consisted of animals dosed with sterile Methocel 0.5% (w/v) with 5% (v/v) Tween 80 using the same dosing regime and dose volume used for the test article treated animals.

Untreated controls were not required as this vehicle has been tested previously in this laboratory.

The positive control, Cyclophosphamide (CPA, Sigma-Aldrich Chemical Co, Poole, UK) was freshly prepared in saline and administered once only via oral gavage 24 hours prior to necropsy as tabulated below:

Table 2: Positive Controls

Dose volume (mL/kg)	Concentration of CPA solution (mg/mL)	Dose of CPA administered (mg/kg)
10.0	4.00	40.0

TEST ARTICLE FORMULATION

Preparation

Dosing preparations were freshly prepared prior to each dosing occasion by formulating Fexinidazole in Methocel 0.5% (w/v) with 5% (v/v) Tween 80 to give the concentrations specified in the table below.

The test article was weighed into suitable containers and transferred to a mortar and pestle. The container was rinsed using a small volume of vehicle, which was then added to the test article to form a smooth paste. The mixture was transferred to the formulation bottle and the mortar and pestle rinsed with the vehicle, which was subsequently added (together with any remaining vehicle) to the formulation bottle to achieve the final volume. Formulations were then mixed using a Silverson mixer until visibly homogenous.

The following concentrations of Fexinidazole were used during this study:

Table 3: Fexinidazole Concentrations Tested

Experiment	Concentration of dosing preparation (mg/mL)[a]	Dose administered (mg/kg/day)[b]
Micronucleus Experiment	50	500
	100	1000
	200	2000

a All dosing preparations expressed as nominal concentrations
b All treatment doses expressed as nominal dose administered

Stability and homogeneity

No specific storage details were provided by the Sponsor. The Sponsor stated that following dose preparation formulations should be refrigerated (2-8°C) and used within 2 hours of preparation. However, following dose preparation, doses were not refrigerated as stated above, but stored at room temperature and used immediately, with all dosing taking place approximately 2 hours following preparation.

To ensure homogeneity, dose bottles were stirred continuously (on a magnetic stirrer) immediately before and throughout dosing.

Formulations analysis

Duplicate samples of test article formulations (all test article concentrations) and vehicle control from the Micronucleus Experiment were retained for analysis of achieved concentration and homogeneity (refer to minor deviations Appendix 8). Samples taken immediately after preparation from the top, middle and bottom of the same bottle as was used for dosing.

Samples were not stored, and were analysed immediately post-sampling by Covance Laboratories Ltd. The analytical methods used and results obtained are presented in Appendix 6.

Samples were analysed by HPLC and the method used for the analysis was the current version of Covance 2647/23-01F, validated according to Standard Operating Procedures under this study.

TEST SYSTEM

Species, strain and supplier

A sufficient number of out-bred young adult male Crl:CD-1 (ICR) mice were obtained from Charles River (UK) Ltd, Margate, UK.

Specification

Animal specification was as follows:

Table 4: Animal Specification

	Micronucleus Experiment
Number of animals used in study	36M[a]
Weight range on first day of assay (g)	30-35
Approximate age on first day of dosing (weeks)	7

M Male
a Includes 6 satellite animals for bioanalysis

Environment

The animals were routinely kept in the following environment except for short periods of time where experimental procedures dictated otherwise. The animals were housed in a room air-conditioned to provide a minimum of 15 air changes/hour. The temperature and relative humidity ranges were 19 to 25°C and 40 to 70%, respectively. Fluorescent lighting was controlled automatically to give a cycle of 12 hours light (0600 to 1800) and 12 hours dark. The study room was used to house animals allocated to other studies.

The animals were housed in groups of up to three in cages that conformed with the 'Code of practice for the housing and care of animals used in scientific procedures' (Home Office, London, 1989).

Environmental enrichment

In order to enrich both the environment and the welfare of the animals, they were provided with wooden Aspen chew blocks.

Diet, water and bedding

Throughout the study the animals had access *ad libitum* to SQC Rat and Mouse Maintenance Diet No 1, Expanded (Special Diets Services Ltd. Witham). Each batch of diet was analysed for specific constituents and contaminants.

Mains water was provided *ad libitum* via water bottles. The water is periodically analysed for specific contaminants.

Bedding was provided on a weekly basis to each cage by use of clean Aspen wood chips (Datesand Ltd, Manchester). The bedding was analysed for specific contaminants.

No contaminants were expected to be present in diet, water or bedding at levels that might interfere with achieving the objective of the study. Results of analyses performed on diet, water, bedding and environmental enrichment are held centrally at Covance Laboratories Ltd.

METHODS

Pre-experimental procedures

Acclimatisation and health procedures

All animals were given a clinical inspection for ill health on arrival. They were acclimatised for 8 days and a veterinary inspection was performed before the start of dosing to ensure their suitability for study.

Allocation to treatment group

Animals were randomised to groups of six animals using a system of random numbers.

Checks were made prior to dosing on the first day of treatment to ensure individual group weights differed from the mean group weight by no more than 5%.

The allocation of animals to groups is detailed in Appendix 1.

Identification of the test system

The animals were individually identified by uniquely numbered ear-tag. Cages were appropriately identified (using a colour-coded procedure) with study information including study number, study type, start date, number and sex of animals, together with a description of the dose level and proposed time of necropsy.

In-life experimental procedures

Animals were dosed in groups as described in the section entitled "Dose levels".

Individual body weights and dosages administered are shown in Appendix 1.

Bioanalysis

Groups of male satellite animals were dosed with vehicle or Fexinidazole (highest dose only). Animals were dosed by the same route, dose level and at the same dosing frequency as that described for the micronucleus animals. Plasma was isolated from these animals and used to assess systemic exposure to the test article.

Animals were dosed and bled as follows:

Table 5: Bioanalysis

Group	Number of animals	Dose volume (mL/kg)	Dose (mg/kg/day)	Sample time: 1 hour after final administration
Vehicle	3M	10	0	√
Fexinidazole	3M	10	2000[a]	√

M Male
a Treatment doses expressed as nominal dose administered

Approximately 1 mL of whole blood was taken via cardiac puncture (under terminal isoflourane anaesthesia) approximately 1 hour after final administration.

Blood was collected at room temperature into tubes containing lithium heparin and mixed thoroughly before placing on ice until centrifugation (2300 *g*, 4°C, 10 minutes). Plasma was separated into appropriately labelled polypropylene tubes and stored frozen at –20°C nominal until dispatch on dry ice to the Test Site.

The analytical methods used and results obtained are presented in Appendix 7.

Experimental observations

Routine health status check

All animals were examined at the beginning and the end of the working day to ensure that they were in good health and displayed no signs of overt toxicity.

Post dosing observations

All animals were observed daily for signs of ill health or overt toxicity. An individual record was maintained of the clinical condition of all Micronucleus animals dosed in the study.

In the Micronucleus Experiment post-dosing observations were performed immediately after each dose administration, at least twice in the 4 hours following each dose and prior to the second administration. Observations were also recorded at least once on the day of bone marrow sampling.

Satellite animals were for blood sampling purposes only, no specific clinical observations were recorded for these animals.

Body weights

Individual body weights were recorded on each day of dosing and the day of bone marrow sampling (Micronucleus animals). The body weights of satellite animals were recorded on each day of dosing (data not reported).

Bone marrow sampling and slide preparation

Test article and vehicle-treated mice were sampled in groups, 24 hours after the second administration; CPA-treated mice were sampled 24 hours after the single dose. Mice were killed by an overdose of sodium pentobarbitone, given via intraperitoneal injection and subsequently ensured by cervical dislocation, in the same sequence used for dosing.

Both femurs from each animal were exposed, removed, cleaned of adherent tissue and the ends removed from the shanks. Using a syringe and needle, bone marrows were flushed from the marrow cavity with 1 mL foetal bovine serum into appropriately labelled centrifuge tubes.

A further 1 mL of foetal bovine serum was added to the tubes, which were then centrifuged at 200*g* for approximately five minutes; the serum was aspirated to leave one or two drops and the cell pellet. The pellet was mixed into this small volume of serum in each tube by using a Pasteur pipette, and from each tube one drop of suspension was placed on the end of each slide labelled with the appropriate study number, sampling time, sex, date of preparation and animal number (details where appropriate). The latter served as the code so analysis could be conducted "blind". A smear was made from the drop by drawing the end of a clean slide along the labelled slide.

Slides were allowed to air-dry and then fixed for 5 minutes in absolute methanol before being stained according to the modification of Gollapudi and Kamra [14]. Slides were stained immediately. Staining was performed on the same day as slide preparation (with the exception of the additional slides that were analysed. In this instance, an additional fix was performed as detailed above). One slide from each set was taken (any remaining slides kept in reserve). Prior to staining any stored slides were fixed again for 5 minutes in absolute methanol. After rinsing several times in distilled water, slides were stained for 10 minutes in filtered Giemsa stain diluted in distilled water (1:4 (v/v)). Slides were rinsed and allowed to dry before clearing in xylene for 3 minutes. When dry, slides were mounted with coverslips and stored at room temperature prior to analysis.

Slide analysis

Slides from the CPA-treated mice were initially checked to ensure the system was operating satisfactorily. The slides from all groups were arranged in numerical order by sex and sampling time and analysed by an individual not connected with the dosing phase of the study.

Initially the relative proportions of polychromatic erythrocytes (PCE), seen as pale blue or blue/grey enucleate cells, and normochromatic erythrocytes (NCE), seen as yellow/orange-stained enucleate cells, were determined until a total of at least 1000 cells where possible (PCE plus NCE) had been analysed. Counting continued until at least 2000 PCE per animal where possible had been examined. All PCE containing micronuclei observed during these two phases of counting were recorded.

A further 2000 PCE per animal were examined from reserve slides for all dose groups to clarify the initial result obtained.

Slide analysis was performed by a competent analyst trained in the applicable Covance Laboratories Harrogate (CLEH) standard operating procedures. The analyst was physically located remote from the CLEH facility, but was subject to CLEH management and GLP control systems (including QA inspection). All slides and raw data generated by the remote analyst were returned to CLEH for archiving on completion of analysis.

Analysis of results

Treatment of data

After completion of microscopic analysis and decoding of the data the following were calculated:

1. % PCE for each animal and the mean for each group. The group mean % PCE values were examined to see if there was any decrease in groups of treated animals that could be taken as evidence of bone marrow toxicity

2. Frequency of MN PCE (i.e. MN per 2000 PCE) and % MN PCE for each animal and the group mean % MN PCE (± standard deviation).

The numbers of MN PCE in vehicle control animals were compared with the laboratory's historical control data to determine whether the assay was acceptable. For

each group, inter-individual variation in the numbers of MN PCE was estimated by means of a heterogeneity chi-square test [15].

The numbers of micronucleated PCE in each treated group were compared with the numbers in vehicle control groups by using a 2 x 2 contingency table to determine chi-square [15]. Probability values of $p \leq 0.05$ were accepted as significant. A further statistical test (for linear trend) was used to evaluate possible dose-response relationships.

Acceptance criteria

The assay was considered valid if all the following criteria were met:

1. The incidence and distribution of MN PCE in vehicle control groups were consistent with the laboratory's historical vehicle control data, and

2. At least five animals out of each group were available for analysis, and

3. The positive control chemical (CPA) induced a statistically significant increase in the frequency of MN PCE.

Acceptance under any other criteria are discussed in the results section.

Evaluation criteria

For valid data, the test article was considered to induce clastogenic / aneugenic damage if:

1. A statistically significant increase in the frequency of MN PCE occurred at one or more dose levels

2. The incidence and distribution of MN PCE in individual animals at such a point exceeded the laboratory's historical vehicle control data

3. A dose-response trend in the proportion of MN PCE was observed (where more than two dose levels were analysed).

The test article was considered as positive in this assay if all of the above criteria were met.

The test article was considered as negative in this assay if none of the above criteria were met.

Results which only partially satisfied the above criteria were dealt with on a case-by-case basis. Evidence of a dose-related effect was considered useful but not essential in the evaluation of a positive result [16]. Biological relevance was taken into account, for example consistency of response within and between dose levels.

RESULTS

From data provided by the Sponsor, the Micronucleus Experiment was performed using a maximum intended dose of 2000 mg/kg/day (the maximum recommended dose in accordance with current guidelines), two lower intended doses of 500 and 1000 mg/kg/day were also used.

Micronucleus Experiment

Raw data

1. The total numbers of PCE, NCE, and MN PCE counted for each animal, together with calculated %PCE and % MN PCE values appear in Appendix 3.

2. The data from Appendix 3 are summarised as group means in Appendix 2. Appendix 4 shows the current historical vehicle control data from micronucleus experiments in this laboratory.

Validity of study

The acceptance criteria for the micronucleus test are listed in the acceptance criteria section. The data in Appendix 2, Appendix 3 and Appendix 4 confirm that:

1. The incidence and distribution of micronucleated PCE in the vehicle control group were consistent with the laboratory's historical vehicle control data, and

2. At least five animals out of each group were available for analysis, and

3. The positive control chemical (CPA) induced a statistically significant increase in the frequency of micronucleated PCE (Appendix 2).

The assay data were therefore considered valid.

Clinical signs

No clinical signs of toxicity were observed in any animal following treatments with vehicle, Fexinidazole (at 500, 1000 or 2000 mg/kg/day, nominal) or the positive control (CPA).

Mean weight change from Day 1 to Day 3 for the vehicle treated animal group was -2%, with the low, intermediate and high dose treated animals showing mean group weight gains of +1%, +4% and +4% respectively.

Analysis of micronucleus data

Groups of mice treated with Fexinidazole exhibited %PCE values that were similar to vehicle controls (Appendix 2 and Appendix 4) and which fell within acceptable ranges (Appendix 4). There was no evidence of any test article-induced toxicity to the bone marrow (as would usually be indicated by a notable decrease in %PCE values compared to the vehicle control group or dose dependant decrease).

Initial 2000 PCE scored

The group mean frequency of MN PCE for mice treated with Fexinidazole in the maximum dose group (2000 mg/kg/day, nominal) produced a weak, but statistically significant increase ($p \leq 0.05$) in group mean MN PCE compared to the concurrent vehicle control. This increase was also accompanied by a statistically significant linear trend ($p \leq 0.01$). Three animals in the maximum dose group exhibited 5 or greater MN PCE/2000 PCE scored, compared to the vehicle control group which had a maximum of 4 MN PCE/2000 PCE scored from a single animal. Due to this abnormal distribution of MN PCE observed in the maximum dose group compared to the concurrent vehicle control, a further 2000 PCE/animal were analysed from reserve slides to aid in clarifying the data.

Additional 2000 PCE scored

The group mean frequencies of MN PCE for animals treated with Fexinidazole at all dose levels were similar to and not statistically significant from the concurrent vehicle control group. It should be noted the initial increases in individual MN frequencies observed at the maximum dose were not reproducible with additional scoring. Initially animals 43, 48 and 70 exhibited MN frequencies of 6, 5 and 5 respectively, following additional scoring MN frequencies of 0, 5 and 3 respectively were obtained.

Combined data

Whilst both a weak, linear trend ($p \leq 0.05$) and a weak, but statistically significant increase ($p \leq 0.05$) in group mean MN PCE frequency was apparent in the high dose; it is important to highlight that individual frequencies of MN PCE for all treated animals from both the initial and additional analyses were consistent with the laboratory's historical control distribution data.

In view of these observations, these initial increases in individual MN PCE observed were considered chance events, which were poorly replicated following additional

scoring. Overall, these data indicate a lack of evidence for test article related effect (Appendix 2, Appendix 3 and Appendix 4).

Formulations analysis

Results of formulation analyses (Appendix 6) demonstrated that whilst the mean achieved concentrations for all doses prepared on day 1 were within the required 100±10% of the nominal test article concentrations, the results of homogeneity analysis showed that all doses exceeded the required coefficient of variation (CV) by more than 6%. As such all doses on day 1 were not considered homogenous.

Results from day 2 confirmed that the achieved concentration for all doses prepared were either within or marginally outside the required 100 ±10% of the nominal test article concentrations. Homogeneity analysis also confirmed that all doses were either within or marginally outside the required CV of ≤ 6%.

Whilst the homogeneity analysis on day 1 suggested that there were distinct differences between the top, middle and bottom within each dose, all doses were continuously stirred prior to and throughout the dosing period. This should have negated the differences observed within the analyses, with all mice receiving the required (nominal) dose over the two days of dosing.

No test article was detected in the vehicle samples.

Bioanalysis

The results of bioanalysis are presented in Appendix 7. These results confirm that animals dosed with Fexinidazole were systemically exposed to the parent compound and both major metabolites after 1 hour. The majority of the parent compound had been metabolised to the sulfoxide and the sulphone metabolites.

There was no test article contamination in vehicle samples.

CONCLUSION

It is concluded that Fexinidazole did not induce micronuclei in the polychromatic erythrocytes of the bone marrow of male mice treated up to a nominal dose of 2000 mg/kg/day (a recommended maximum dose for the *in vivo* micronucleus assay according to current regulatory guidelines) under the assay conditions employed.

Whilst small, but statistically significant increases in micronuclei was observed in the high dose group, following initial slide analysis, following additional slide analysis and combing of the data sets this initial increase was small and non-reproducible. There was also no evidence of a dose-related effect, or bone marrow toxicity. As such, these small increases were considered chance events not related to test article effect.

REFERENCES

1 Mitelman F (1991) "Catalogue of Chromosome Aberrations in Cancer, 4th edition". New York: Wiley-Liss

2 Schmid W (1976) The micronucleus test for cytogenetic analysis. In "Chemical Mutagens. Principles and Methods for Their Detection." Vol 4 A Hollaender Ed. Plenum Press, New York-London. pp 31-53

3 Heddle J A (1973) A rapid *in vivo* test for chromosomal damage. Mutation Research 18, 187-190

4 Salamone M F, Heddle J A, Stuart E and Katz M (1980) Towards an improved micronucleus test - studies on three model agents, mitomycin C, cyclophosphamide and dimethylbenzanthracene. Mutation Research 74, 347-356

5 Hayashi M, Tice RR, MacGregor JT, Anderson D, Blakey DH, Kirsh-Volders M, Olerson FB, Pacchierotti F, Romagna F, Shimada H, Sutou S and Vannier B. (1994) *In vivo* rodent erythrocyte micronucleus assay. In: SM Galloway (Ed), Report of the International Workshop on Standardisation of Genotoxicity Test Procedures. Mutation Research, 312, 293-304

6 Richold M, Chandley A, Ashby J, Gatehouse D G, Bootman J and Henderson L. (1990) *In vivo* cytogenetics assays, in: DJ Kirkland (Ed.), Basic Mutagenicity Test. UKEMS Recommended Procedures. Cambridge University Press, Cambridge, UK, pp. 115-141

7 OECD (1997) “Genetic Toxicology: Mammalian Erythrocyte Micronucleus Test”. In OECD Guidelines for the testing of chemicals. OECD Paris, Test Guideline 474

8 European Agency for the Evaluation of Medicinal Products. (1995) ICH Topic S 2 A. “Genotoxicity: Guidance on Specific Aspects of Genotoxicity Tests for Pharmaceuticals”. ICH Harmonised Tripartite Guideline

9 Purchase I F H and Ray V (1981) Summary Report on the Performance of In vivo Assays. In "Evaluation of Short-Term Tests for Carcinogens",

F J de Serres and J Ashby Eds. Elsevier/North Holland, New York-Amsterdam-Oxford, pp 86-95

10 Heddle J A, Stuart E and Salamone M F (1984) The bone marrow micronucleus test. In "Handbook of Mutagenicity Test Procedures", B J Kilbey et al Eds. Elsevier Science Publishers, Amsterdam - New York, Oxford, pp 441-457

11 Ashby J, Tinwell H, Gulati D and Heddle J A (1990) Overview of the study in relation to protocol design for the rodent bone-marrow micronucleus assay. Mutation Research 234, 223-248

12 Ashby J and Mirkova E (1987) Re-evaluation of the need for multiple sampling times in the mouse bone marrow micronucleus assay: results for DMBA. Environmental Mutagenesis 10, 297-305

13 Fielder R J, Allen J A, Boobis A R, Botham P A, Doe J, Esdaile D J, Gatehouse D G, Hodson-Walker G, Morton D B, Kirkland D J and Richold M (1993) Report of the BTS/UKEMS Working Group on Dose Setting in *in vivo* Mutagenicity Assays. Human & Experimental Toxicology 12, 189-198

14 Gollapudi B and Kamra O P (1979) Application of a simple Giemsa-staining method in the micronucleus test. Mutation Research 64, 45-46

15 Lovell D P, Anderson D, Albanese R, Amphlett G E, Clare G, Ferguson R, Richold M, Papworth D G and Savage J R K (1989) Statistical analysis of *in vivo* cytogenetic assays. In "Statistical Evaluation of Mutagenicity Test Data" (UKEMS sub-committee on guidelines for mutagenicity testing. Report. Part III). Ed D J Kirkland. Cambridge University Press, pp 184-232

16 Scott D, Dean B. J, Danford N. D. Kirkland D .J. Metaphase chromosome aberration assays *in vitro*. Basic Mutagenicity Tests; UKEMS recommended procedures. Ed. Kirkland D. J. 1990.

APPENDICES

Appendix 1
Body weights and dosages

Table 6: Fexinidazole: Body weights and dosages: Micronucleus Experiment

Treatment (mg/kg/day)	Group	Animal number	Day 1 Body weight (g)	Day 1 Dose given (mL)	Day 2 Body weight (g)	Day 2 Dose given (mL)	Day 3 Body weight (g)
Vehicle control	1	47	33	0.33	33	0.33	32
		49	30	0.30	29	0.29	29
		51	31	0.31	30	0.30	30
		63	34	0.34	34	0.34	34
		56	31	0.31	31	0.31	31
		55	31	0.31	31	0.31	31
Group mean weight (g) ± SD			32 ± 1.5	Group weight change (Day 1 to 3)			-2%
500[a]	2	68	33	0.33	33	0.33	33
		66	34	0.34	35	0.35	34
		45	30	0.30	31	0.31	30
		41	34	0.34	33	0.33	32
		67	30	0.30	32	0.32	32
		58	31	0.31	32	0.32	32
Group mean weight (g) ± SD			32 ± 1.9	Group weight change (Day 1 to 3)			+1%
1000[a]	3	65	34	0.34	35	0.35	35
		62	35	0.35	35	0.35	35
		50	32	0.32	33	0.33	33
		53	32	0.32	33	0.33	33
		69	34	0.34	34	0.34	35
		54	32	0.32	33	0.33	35
Group mean weight (g) ± SD			33 ± 1.3	Group weight change (Day 1 to 3)			+4%
2000[a]	4	48	30	0.30	30	0.30	30
		43	33	0.33	35	0.35	34
		60	34	0.34	36	0.36	36
		70	31	0.31	33	0.33	33
		59	32	0.32	33	0.33	33
		57	33	0.33	34	0.34	34
Group mean weight (g) ± SD			32 ± 1.5	Group weight change (Day 1 to 3)			+4%
Positive control, CPA 40+	5	46	34	-	35	0.35	34
		64	31	-	31	0.31	30
		52	30	-	30	0.30	30
		42	33	-	34	0.34	34
		44	34	-	34	0.34	34
		61	34	-	35	0.35	34
Group mean weight (g) ± SD			33 ± 1.8	Group weight change (Day 1 to 3)			0%

a All treatment doses expressed as nominal dose administered
\+ Administered as a single dose
SD Standard Deviation

Appendix 2
Summary of micronucleus data

Table 7: Fexinidazole: Summary of Micronucleus Data

Treatment group (mg/kg/day)	Kill Time (hours)	Sex	% PCE	Group mean % micronucleated PCE per treatment group (±sd)
Vehicle Control	24H	M	43.27	0.09 ± 0.07
500[a]	24H	M	44.62	0.15 ± 0.09
1000[a]	24H	M	43.27	0.13 ± 0.09
2000[a]	24H	M	43.33	0.22 ± 0.06
CPA, 40+	24H	M	46.95	1.93 ± 0.78

a	All treatment doses expressed as nominal dose administered
+	Administered as a single dose
SD	Standard deviation

Table 8: Fexinidazole: Statistical Analysis of Micronucleus Data – Initial 2000 PCE scored

Treatment (mg/kg/day)	PCE Scored	MN PCE observed	% MN PCE	Standard Deviation	Heterogeneity		2 x 2 Contingency	
					X^2	Significance	X^2	Significance
Vehicle	12000	11	0.09	0.07	5.91	NS		
500[a]	12000	18	0.15	0.09	5.34	NS	1.24	NS
1000[a]	12000	15	0.13	0.09	6.21	NS	0.35	NS
2000[a]	12000	26	0.22	0.06	1.70	NS	5.31	$p \leq 0.05$
CPA, 40+	12000	231	1.93	0.78			200.20	$p \leq 0.001$

Linear Trend: $z = 2.386$ $p \leq 0.01$
a	All treatment doses expressed as nominal dose administered
NS	Not significant
MN	Micronucleated
+	Administered as a single dose
SD	Standard deviation

Table 9: Fexinidazole: Statistical Analysis of Micronucleus Data – Additional 2000 PCE scored

Treatment (mg/kg/day)	PCE Scored	MN PCE observed	% MN PCE	Standard Deviation	Heterogeneity		2 x 2 Contingency	
					X^2	Significance	X^2	Significance
Vehicle	12000	13	0.11	0.07	5.01	NS		
500[a]	12000	15	0.13	0.07	3.80	NS	0.04	NS
1000[a]	12000	11	0.09	0.07	4.82	NS	0.04	NS
2000[a]	12000	17	0.14	0.10	6.66	NS	0.30	NS
CPA, 40+	12000	241	2.01	1.09			205.04	$p \leq 0.001$

Linear Trend: z = 0.633 NS
a All treatment doses expressed as nominal dose administered
NS Not significant
MN Micronucleated
\+ Administered as a single dose
SD Standard deviation

Table 10: Fexinidazole: Statistical Analysis of Micronucleus Data – Combined data

Treatment (mg/kg/day)	PCE Scored	MN PCE observed	% MN PCE	Standard Deviation	Heterogeneity		2 x 2 Contingency	
					X^2	Significance	X^2	Significance
Vehicle	24000	24	0.10	0.04	4.00	NS		
500[a]	24000	33	0.14	0.07	7.56	NS	1.12	NS
1000[a]	24000	26	0.11	0.06	5.85	NS	0.02	NS
2000[a]	24000	43	0.18	0.05	2.91	NS	4.84	$p \leq 0.05$
CPA, 40+	24000	472	1.97	0.92			407.05	$p \leq 0.001$

Linear Trend: z = 2.200 $p \leq 0.05$
a All treatment doses expressed as nominal dose administered
NS Not significant
MN Micronucleated
\+ Administered as a single dose
SD Standard deviation

Appendix 3
Micronucleus data

Table 11: Fexinidazole: Individual animal micronucleus frequencies - Initial 2000 PCE scored

Treatment (mg/kg/day)	Animal Number	PCE Count	NCE Count	% PCE	Total PCE Count	MN PCE	% MN PCE
Vehicle control	47	442	558	44.20	2000	1	0.05
	49	458	542	45.80	2000	1	0.05
	51	441	559	44.10	2000	4	0.20
	63	414	586	41.40	2000	0	0.00
	56	419	581	41.90	2000	3	0.15
	55	422	578	42.20	2000	2	0.10
500[a]	68	458	542	45.80	2000	4	0.20
	66	434	566	43.40	2000	3	0.15
	45	398	602	39.80	2000	4	0.20
	41	478	522	47.80	2000	0	0.00
	67	470	530	47.00	2000	2	0.10
	58	439	561	43.90	2000	5	0.25
1000[a]	65	379	621	37.90	2000	3	0.15
	62	447	553	44.70	2000	3	0.15
	50	423	577	42.30	2000	5	0.25
	53	420	580	42.00	2000	0	0.00
	69	441	559	44.10	2000	3	0.15
	54	486	514	48.60	2000	1	0.05
2000[a]	48	429	571	42.90	2000	5	0.25
	43	444	556	44.40	2000	6	0.30
	60	415	585	41.50	2000	3	0.15
	70	408	592	40.80	2000	5	0.25
	59	424	576	42.40	2000	4	0.20
	57	480	520	48.00	2000	3	0.15
CPA, 40+	46	476	524	47.60	2000	22	1.10
	64	466	534	46.60	2000	24	1.20
	52	474	526	47.40	2000	35	1.75
	42	456	544	45.60	2000	50	2.50
	44	489	511	48.90	2000	63	3.15
	61	456	544	45.60	2000	37	1.85

a All treatment doses expressed as nominal dose administered
\+ Administered as a single dose

Table 12: Fexinidazole: Individual animal micronucleus frequencies – Additional 2000 PCE scored

Treatment (mg/kg/day)	Animal Number	PCE Count	NCE Count	% PCE	Total PCE Count	MN PCE	% MN PCE
Vehicle control	47	442	558	44.20	2000	1	0.05
	49	458	542	45.80	2000	3	0.15
	51	441	559	44.10	2000	2	0.10
	63	414	586	41.40	2000	4	0.20
	56	419	581	41.90	2000	3	0.15
	55	422	578	42.20	2000	0	0.00
500[a]	68	458	542	45.80	2000	5	0.25
	66	434	566	43.40	2000	2	0.10
	45	398	602	39.80	2000	2	0.10
	41	478	522	47.80	2000	1	0.05
	67	470	530	47.00	2000	2	0.10
	58	439	561	43.90	2000	3	0.15
1000[a]	65	379	621	37.90	2000	0	0.00
	62	447	553	44.70	2000	1	0.05
	50	423	577	42.30	2000	3	0.15
	53	420	580	42.00	2000	3	0.15
	69	441	559	44.10	2000	3	0.15
	54	486	514	48.60	2000	1	0.05
2000[a]	48	429	571	42.90	2000	5	0.25
	43	444	556	44.40	2000	0	0.00
	60	415	585	41.50	2000	1	0.05
	70	408	592	40.80	2000	3	0.15
	59	424	576	42.40	2000	4	0.20
	57	480	520	48.00	2000	4	0.20
CPA, 40+	46	476	524	47.60	2000	26	1.30
	64	466	534	46.60	2000	30	1.50
	52	474	526	47.40	2000	24	1.20
	42	456	544	45.60	2000	53	2.65
	44	489	511	48.90	2000	79	3.95
	61	456	544	45.60	2000	29	1.45

a All treatment doses expressed as nominal dose administered
+ Administered as a single dose
PCE and NCE counts were determined from the initial analysis

Table 13: Fexinidazole: Individual animal micronucleus frequencies – Combined data

Treatment (mg/kg/day)	Animal Number	PCE Count	NCE Count	% PCE	Total PCE Count	MN PCE	% MN PCE
Vehicle control	47	442	558	44.20	4000	2	0.05
	49	458	542	45.80	4000	4	0.10
	51	441	559	44.10	4000	6	0.15
	63	414	586	41.40	4000	4	0.10
	56	419	581	41.90	4000	6	0.15
	55	422	578	42.20	4000	2	0.05
500[a]	68	458	542	45.80	4000	9	0.23
	66	434	566	43.40	4000	5	0.13
	45	398	602	39.80	4000	6	0.15
	41	478	522	47.80	4000	1	0.03
	67	470	530	47.00	4000	4	0.10
	58	439	561	43.90	4000	8	0.20
1000[a]	65	379	621	37.90	4000	3	0.08
	62	447	553	44.70	4000	4	0.10
	50	423	577	42.30	4000	8	0.20
	53	420	580	42.00	4000	3	0.08
	69	441	559	44.10	4000	6	0.15
	54	486	514	48.60	4000	2	0.05
2000[a]	48	429	571	42.90	4000	10	0.25
	43	444	556	44.40	4000	6	0.15
	60	415	585	41.50	4000	4	0.10
	70	408	592	40.80	4000	8	0.20
	59	424	576	42.40	4000	8	0.20
	57	480	520	48.00	4000	7	0.18
CPA, 40+	46	476	524	47.60	4000	48	1.20
	64	466	534	46.60	4000	54	1.35
	52	474	526	47.40	4000	59	1.48
	42	456	544	45.60	4000	103	2.58
	44	489	511	48.90	4000	142	3.55
	61	456	544	45.60	4000	66	1.65

a All treatment doses expressed as nominal dose administered
\+ Administered as a single dose

Appendix 4
Historical vehicle control data ranges

Table 14: Historical vehicle control range

Control Type		Individual PCE %	Individual frequency of micronucleated PCE per 2000 (%)	Animals (%) with 0,1 (or more) micronuclei (for 2000 PCE scored)						
				0	1	2	3	4	5	6+
Vehicle	Mean	44	1.48 (0.07%)	27.3	28.0	25.9	11.5	4.6	1.8	1.0
	SD	8.2								
	Median	44								
	Observed range	24-70								
	95% confidence interval for group mean of:									
	4 values	35-53								
	5 values	36-52								
	6 values	37-51								
	7 values	37-51								
	8 values	38-50								
	9 values	38-50								
	10 values	38-50								
Positive	Mean	41	26[(1)] (1.30%)							
	SD	6.1	16.3							
	Median	42	24							
	Observed range	28-53	3-84							
	95% confidence interval for group mean of:									
	4 values	35-48	13-44[(1)]							
	5 values	35-47	14-42[(1)]							
	6 values	36-47	15-41[(1)]							
	7 values	36-46	16-40[(1)]							
	8 values	36-46	16-39[(1)]							
	9 values	37-46	17-38[(1)]							
	10 values	37-45	17-37[(1)]							

[(1)] Calculated from square root transformed data

Calculated in February 2008 by CLEH Statistics from studies started between June 2004 and September 2007. Vehicle control statistics based on 286 animals from 35 studies; positive control statistics based on 80 animals from 12 studies.

Appendix 5
Certificate of analysis

CENTIPHARM
Active for your synthesis

ANALYSIS CERTIFICATE

FEXINIDAZOLE

Manufact. date November-2007
Expiry date October-2008

DATE	January 15, 2008
ANALYSIS N° CA	07327/01
BATCH CENTIPHARM	3168-07-01/O
SAMPLE N°	ECH:08015/51
WEIGHT (g)	70

DETERMINATIONS	RESULTS	SPECIFICATIONS
IDENTIFICATION	IR Spectrum complies	IR Spectrum complies
APPEARANCE	powder	powder
COLOUR	yellow	yellow
LOSS ON DRYING (%)	0,0	<= 0,5
SULPHATED ASH (%)	0,0	<= 0,1
$HClO_4$ ASSAY (%)	100,2	98,5 à 101,5
RELATED SUBSTANCES - HPLC- Any known impurity (%)	< 0,05	<= 0,15
RELATED SUBSTANCES - HPLC- Any other impurity (%)	0,08	<= 0,10
RELATED SUBSTANCES - HPLC - All impurities sum (%)	0,1	<= 0,5
RESIDUAL SOLVENTS -GC- Acetone (ppm)	740	<= 5000
RESIDUAL SOLVENTS -GC- Methanol (ppm)	20	<= 3000
RESIDUAL SOLVENTS -GC- Toluene (ppm)	4	<= 890

CONFORMITY	YES / NO

Grasse, 16/01/2008
M.CONNAN
Quality Control Manager

AXYNTIS
Active for your synthesis

CENTIPHARM

Chemin de la Madeleine - B.P. 45249 - F-06131 GRASSE CEDEX - Tél. : 33 (0)4 93 70 01 32 - Fax : 33 (0)4 93 70 05 65 - http://www.axyntis.com - contact@axyntis.com
S.A.S. au capital de 1 525 000 € - R.C.S. GRASSE 326 171 378 - APE 241 G - TVA FR 68 326 171 378

Appendix 6
Formulations analysis

SUMMARY

The test article Fexindazole was formulated in 0.5% methyl-cellulose in 5% Tween 80 by Central Dispensary for dosing during the study.

Formulations received on the 6 and 7 February 2008 were analysed to verify achieved concentration.

The analytical procedure was validated in this study. The validation also included analysis of DMSO as this was used as a vehicle on other studies using the same analytical procedure.

PROCEDURES

Validation of analytical method

To determine the validity of an analytical method it is necessary to:

- verify linearity of response
- determine the precision of the analytical method
- determine the accuracy of the analytical method
- confirm specificity

Linearity of response

A 'calibration set' of solutions of increasing test article concentration was submitted to HPLC.

The linearity was validated over the approximate range 16.8 to 101 µg/mL. This was the working calibration range.

A calibration graph was constructed for the standard solutions.

Precision and accuracy

The precision and accuracy of the analytical procedure over the approximate concentration range 16.7 to 100 µg/mL (correction factor not applied) was determined as follows:

- Sets of standards at concentrations 16.70 and 100.2 µg/mL were prepared from a stock solution (each set contained six standards).

- Another set was prepared at 50.11 µg/mL with the addition of 1 mL of DMSO made to 10 mL.

- A further set was prepared at 50.11 µg/mL with the addition of 1 mL of 0.5% methyl-cellulose in 5% Tween 80 made to 10 mL.

- Blank solutions was also prepared in a similar manner.

- A six point calibration line was constructed from a different stock solution from the one above.

- All solutions were submitted to HPLC.

- The concentration of test chemical sets was calculated from their HPLC responses using the calibration line.

- The mean and standard deviations of these observations were used to determine the relative precision (coefficient of variation) and accuracy of the assay.

$$relative\ precision\,\% = \frac{standard\ deviation}{mean}\ x\,100$$

$$accuracy\,\% = \frac{mean\ calculated\ test\ article\ concentration}{theoretical\ test\ article\ concentration}\ x\,100$$

Homogeneity and Achieved concentration

Samples (1 mL) from the top, middle and bottom were removed in duplicate from each of the formulations. These were analysed for test article concentration to determine homogeneity and achieved concentration (apart from the controls where only a single (1 mL) analysis was performed).

Analytical procedure

The written analytical procedure Covance 2647/023-01F was used to determine achieved concentration.

Data collection and processing

The data was collected and processed using an Empower 2 data capture system version, Build number 2154.

RESULTS

Validation

Linearity of response

A set of standard solutions was submitted to HPLC. The concentration/response curve was straight with an intercept approaching zero. The correlation coefficient (r) was 1.0000. A minimum value of 0.9950 is considered acceptable (r = 1.00 for a perfect fit to the line).

Linearity of response was therefore considered to be acceptable.

Precision

The precision over the calibration range was determined:

	Nominal Concentration (µg/mL)			
	16.70	50.11*	50.11#	100.2
Precision %	0.77	2.78	0.51	0.50
Accuracy %	99	99	99	101

* contains DMSO
\# contains 0.5% methyl-cellulose in 5% Tween 80

Precision is normally expected to be less than 5% for this type of test and accuracy is regarded as acceptable if it is between 95% and 105%. The results were within these limits. The method was therefore accepted in terms of precision and accuracy.

There was no significant detector response from control solution thereby confirming specificity of the method.

Homogeneity and Achieved Concentration:

Day 1: 6 February 2008:

Conc (mg/mL)	Results as % nominal concentration						Mean (%)	CV (%)
	Top		Middle		Bottom			
50	117	114	96	83	59	72	90	25.65
100	121	108	103	83	71	62	91	25.15
200	96	78	170	102	73	51	95	43.10

Day 2: 7 February 2008:

Conc (mg/mL)	Results as % nominal concentration						Mean (%)	CV (%)
	Top		Middle		Bottom			
50	91	80	95	100	96	97	93	7.60
100	82	82	86	83	82	80	83	2.39
200	82	85	93	89	82	91	87	5.39

Test article was not present in the control samples.

Appendix 8
Minor deviation from protocol

Protocol section	Subject	Deviation
Test Article Formulation	Stability analysis	In error, the protocol stated that stability analysis would be conducted. No stability analysis was conducted, with all formulations being dosed approximately 2 hours following preparation. This is considered to have had no impact on the validity of the data.
Blood Sampling Requirements	Volume of plasma	It can be confirmed that 12 plasma samples were taken, with 6 samples sent to the test site, and the remaining 6 retained here. However, the required volume of plasma (2 aliquots 150 uL/animal) sampled was not recorded. This is considered to have had no impact on the validity of the data as bioanalysis has been completed.
