# Supplementary material for: Fexinidazole – A New Oral Nitroimidazole Drug Candidate Entering Clinical Development for the Treatment of Sleeping Sickness
Source: PLoS Negl Trop Dis. 2010 Dec 21;4(12):e923. doi: 10.1371/journal.pntd.0000923 (PMC3006138; doi:10.1371/journal.pntd.0000923)
Supplement: Dataset 21 — (0.40 MB PDF) [file pntd.0000923.s022.pdf]

Final Report

Study Title	Measurement of unscheduled DNA synthesis in rat liver using an *in vivo/in vitro* procedure
Test Article	Fexinidazole
Author	
Sponsor	Drugs for Neglected Diseases Initiative (DNDi)<br>1 place Saint Gervais<br>1201 Geneva<br>SWITZERLAND
Study Monitor	
Test Facility	Covance Laboratories Ltd
Responsible Scientist and Analytical Test Site, bioanalysis	Accelera/Pre-clinical Development
Covance Study Number	2647/24
Test Site Ref. Number	0065-2008
Report Issued	August 2008
Page Number	1 of 60

CONTENTS

STUDY DIRECTOR AUTHENTICATION AND GLP COMPLIANCE STATEMENT 2

QUALITY ASSURANCE STATEMENT 3

RESPONSIBLE AND REVIEWING SCIENTISTS' STATEMENTS 4

RESPONSIBLE PERSONNEL 5

ARCHIVE STATEMENT 6

CONTENTS 7

SUMMARY 9

OBJECTIVE 11

INTRODUCTION 11

EXPERIMENTAL DESIGN 13
- Regulatory test guidelines 13
- Justification for selection of the test system 13
- Test article administration 13
- Dose selection 13
- Dose levels 14
- Dose volumes 14

TEST AND CONTROL ARTICLES 15
- Test article 15
- Controls 15

TEST ARTICLE FORMULATION 16
- Preparation 16
- Stability and homogeneity 16
- Formulations analysis 16

TEST SYSTEM 18
- Species, strain and supplier 18
- Specification 18
- Environment 18
- Environmental enrichment 19
- Diet, water and bedding 19

METHODS 20
- Pre-experimental procedures 20
- In-life experimental procedures 20
- Bioanalysis 21
- Experimental observations 21
- Liver perfusion and preparation of hepatocyte cultures 22
- Cell attachment 23
- Radiolabelling of hepatocyte cultures 23
- Cell fixation 23
- Autoradiography 24
- Grain counting 24
- Analysis of results 25

RESULTS 27
Selection of doses for UDS Experiments 27
UDS Experiments 27
Formulations analysis 28
Bioanalysis 29

CONCLUSION 30

REFERENCES 31

APPENDICES 32
Appendix 1 Body weights and dosages 32
Appendix 2 Tables of results 34
Appendix 3 Historical vehicle control data ranges for: UDS Assay 42
Appendix 4 Certificate of analysis 43
Appendix 5 Formulations Analysis 44
Appendix 6 Bioanalysis 46
Appendix 7 Minor deviations from protocol 60

SUMMARY

Fexinidazole was tested for its potential to induce unscheduled DNA synthesis (UDS) in the hepatocytes of treated rats.

From data generated by Hoechst (the originators of this compound), Fexinidazole is not lethal after single oral doses up to 10 g/kg to Wistar rats. Repeat dosing of 200 mg/kg and 800 mg/kg of three months duration to Wistar rats resulted in a haemolytic anaemia, testicular atrophy and vacuolar degeneration of hepatocytes tending to necrosis.

Based on the toxicity data provided 2000 mg/kg (the highest recommended dose level) was considered an appropriate maximum dose in this study.

As no gender differences in toxicity have been previously identified this study was conducted in male animals only.

In the UDS Experiments groups of four male rats were treated once with the vehicle Methocel (0.5% w/v) with Tween 80 (5% v/v) or Fexinidazole (at 500.0, 1000 and 2000 mg/kg) via oral gavage, at a dose volume of 10 mL/kg. A group of four male rats were treated once via oral gavage with the required positive control at a dose volume of 10 mL/kg. The positive controls used were 75 mg/kg 2-acetamidofluorene (2-AAF) suspended in corn oil (12-14 hour time point) and 10 mg/kg dimethylnitrosamine (DMN) dissolved in purified water (2-4 hour time point).

No clinical signs were observed in the UDS Experiments. Animals were sacrificed approximately 12-14 hours or 2-4 hours following dose administration.

In addition to the UDS animals, groups of satellite animals were dosed with vehicle and 2000 mg/kg Fexinidazole. Plasma was isolated from these animals and used to assess systemic exposure to Fexinidazole.

Negative (vehicle) control animals gave a group mean NNG value of $\leq$ 0.6 (less than the upper limit of the historical control range) with $\leq$ 0.7% cells in repair. Group mean NNG values were increased by 2-AAF and DMN treatment to $\geq$ 8.9 and more than 50% cells found to be in repair.

In this study the vehicle control NNG value was consistent with both published and historical control data, and the system was shown to be sensitive to two known DNA

damaging agents requiring metabolism for their action. The assay was therefore accepted as valid.

Treatment with 500, 1000 or 2000 mg/kg Fexinidazole did not produce a group mean NNG value greater than 0.5 (within vehicle historical control range) nor were more than 1% cells found in repair for any dose.

Results of formulation analyses demonstrated achieved concentrations within or above 100 ± 10% of the nominal test article concentrations and were therefore considered acceptable.

Analysis of plasma samples confirmed that animals were systemically exposed to Fexinidazole and its sulphoxide and sulphone metabolites.

When treated once via oral gavage with Fexinidazole at doses up to 2000 mg/kg (the maximum recommended dose based on current guidelines), male Sprague Dawley rats showed no induction of UDS in hepatocytes isolated *ex vivo* approximately 12-14 or 2-4 hours after dosing. It is concluded that Fexinidazole had no genotoxic activity detectable in this test system under the experimental conditions employed.

OBJECTIVE

The objective of this study was to evaluate the potential of Fexinidazole to induce unscheduled DNA synthesis (UDS) in the hepatocytes of treated rats.

INTRODUCTION

Many organic chemical carcinogens are thought to act by interaction with macromolecules within the cell, particularly DNA [1]. This interaction results from the metabolism of the compound *in vivo* by, for example, liver mono-oxygenases, which produces reactive electrophilic species. Such molecules will interact with nucleophilic sites within the cell to give covalently bound products [2]. Since animals and man are being continuously exposed to low levels of DNA damaging agents the cell has developed DNA repair systems in order to maintain the integrity of the genome. Cancer is thought to be a somatic mutational event arising either directly or indirectly from DNA damage. Agents which damage DNA therefore have the potential of being carcinogenic.

Examination of the cell for DNA repair can provide a means of studying carcinogen-DNA adducts indirectly and thus provides a useful *in vivo* test. This is the principle behind the *in vivo/in vitro* UDS assay.

Rats are treated with the test article and at time intervals thereafter the rats are humanely killed and their livers dissociated with collagenase and removed. The resulting hepatocytes are exposed to [^{3}H] thymidine which is incorporated into the DNA if UDS is occurring. Normal S-phase synthesis is rare in hepatocytes and can readily be distinguished from UDS autoradiographically. Incorporation is followed by autoradiography of the hepatocytes and grain counting. The technique described here was developed by Mirsalis and Butterworth [3], modified by Ashby *et al* [4] and is detailed by Kennelly *et al* [5]. The advantage of this model is that it is an *in vivo* assay which more accurately represents the pharmacokinetics and pharmacodynamics of the compound than *in vitro* systems.

If a test article is rapidly eliminated from the body, it would be expected that the peak of any induction of UDS would occur over a relatively short time period, as with dimethylnitrosamine (DMN) [3]. Therefore since some test agents may give negative responses at 12 to 14 hours in one experiment, a second experiment was conducted using a 2 to 4 hour sampling time.

This study was performed according to the protocol and two amendments with the exception of the minor deviations detailed in Appendix 7, none of which prejudiced the validity of the study.

The study was initiated on 24 January 2008. Experimental work started on 6 February 2008 and was completed on 10 April 2008. The study completion date is considered to be the date the Study Director signs the final report.

EXPERIMENTAL DESIGN

Regulatory test guidelines

The test methodology used in this study was in accordance with current literature ([1], [2], [3], [4], [5]) and the protocol was designed to meet the known requirements of the OECD Guideline 486, 1997 [6] and the ICH Tripartite Harmonised Guideline on Genotoxicity: Specific Aspects of Regulatory Tests, 1995 [7].

Justification for selection of the test system

The rodent UDS test in the rat is recommended by various regulatory authorities as an appropriate test to determine the genotoxic potential of a compound *in vivo*. The rat has been selected because there is a large volume of background data in this species.

Test article administration

All treatments were given via oral gavage as this is the intended route of human exposure. Animals were not fasted prior to dose administration.

The test article was given as a single administration and animals sampled 12-14 hours and 2-4 hours after administration. This has been shown to be of sufficient duration for the expression of any genotoxic potential ([3], [4], [5]).

Dose selection

The Sponsor provided the following information on the *in vivo* toxicity of Fexinidazole.

From data generated by Hoechst (the originators of this compound), fexinidazole is not lethal after single oral doses up to 10 g/kg to Wistar rats. Repeat dosing of 200 mg/kg and 800 mg/kg of three months duration to Wistar rats resulted in a haemolytic anaemia, testicular atrophy and vacuolar degeneration of hepatocytes tending to necrosis. This is possibly due to induction of enzymes involved in metabolism of this compound.

Based on the toxicity data provided 2000 mg/kg (the highest recommended dose) was considered an appropriate maximum dose according to current recommendations [6], [7]. The UDS test was therefore conducted at a maximum dose of 2000 mg/kg. Lower

doses of 1000 and 500 mg/kg (equivalent to 50 and 25% of the maximum dose respectively) were also tested.

As no gender differences in toxicity have been previously identified this study was conducted in male animals only [6].

Dose levels

The following dose levels were tested in this study:

Table 1: Dose Levels - UDS Experiments

Group Number	Treatment	Dose volume (mL/kg)	Dose (mg/kg)	No. of animals	Sample time (hours after administration)
1	Vehicle control [a]	10	0	4M	12-14
2	Fexinidazole	10	500.0	4M	12-14
3	Fexinidazole	10	1000	4M	12-14
4	Fexinidazole	10	2000	4M	12-14
5	Positive control [b]	10	75	4M	12-14
6	Vehicle control [a]	10	0	4M	2-4
7	Fexinidazole	10	500.0	4M	2-4
8	Fexinidazole	10	1000	4M	2-4
9	Fexinidazole	10	2000	4M	2-4
10	Positive control [c]	10	10	4M	2-4

M Male
a Methocel (0.5% w/v) with Tween 80 (5% v/v)
b 2-acetamidofluorene (2-AAF)
c Dimethylnitrosamine (DMN)

Dose volumes

A dose volume of 10 mL/kg was used. Individual dose volumes were based on individual body weight.

TEST AND CONTROL ARTICLES

Test article

Fexinidazole, batch number 3168-07-01/O was a yellow powder. It was received on 21 January 2008 and stored at room temperature (10-30°C) in the dark. Purity was stated as 100.2% and expiry date was given as October 2008. The certificate of analysis, provided by the Sponsor, is given in Appendix 4. The test article information and certificate of analysis provided by the Sponsor are considered an adequate description of the characterisation, purity and stability of the test article. Determinations of stability and characteristics of the test article were the responsibility of the Sponsor.

Controls

The negative (vehicle) control group consisted of animals dosed with sterile Methocel (0.5% w/v) with Tween 80 (5% v/v) using the same dosing regime and dose volume used for the test article treated animals.

Untreated controls were not required as this vehicle has been tested previously in this laboratory.

The positive control for the 2 to 4 hour time point, 1.0 mg/mL dimethylnitrosamine (DMN; Sigma Chemical Co, Poole, UK) was freshly prepared in purified water and dosed once via oral gavage 2 to 4 hours before sacrifice.

The positive control for the 12 to 14 hour time point, 7.5 mg/mL 2-acetamidofluorene (2-AAF; Sigma Chemical Co, Poole, UK) was freshly prepared in corn oil and dosed once via oral gavage 12 to 14 hours before sacrifice.

The positive control compounds were freshly prepared before use and administered at a dose volume of 10 mL/kg.

Table 2: Positive Controls

Positive control	Dose volume (mL/kg)	Concentration of solution (mg/mL)	Dose administered (mg/kg)
DMN	10	1.0	10
2-AAF	10	7.5	75

TEST ARTICLE FORMULATION

Preparation

Dosing preparations were freshly prepared prior to each dosing occasion by formulating Fexinidazole in Methocel (0.5% w/v) with Tween 80 (5% v/v) to give the concentrations specified in the table below.

The test article was weighed into suitable containers and transferred to a mortar and pestle. The container was rinsed using a small volume of vehicle, which was then added to the test article to form a smooth paste. The mixture was transferred to the formulation bottle and the mortar and pestle rinsed with the vehicle, which was subsequently added (together with any remaining vehicle) to the formulation bottle to achieve the final volume. Formulations were then mixed using a Silverson until visibly homogenous.

The following concentrations of Fexinidazole were used during this study:

Table 3: Fexinidazole Concentrations Tested

Experiment	Concentration of dosing preparation (mg/mL)	Dose administered (mg/kg)
UDS Experiments	50.00	500.0
	100.0	1000
	200.0	2000

Stability and homogeneity

The formulations were stored refrigerated (1-10°C), prior to dosing or formulation analysis and used within 2 hours of preparation.

To ensure homogeneity, dose bottles were stirred continuously (on a magnetic stirrer) immediately before and throughout dosing.

Formulations analysis

Samples of test article formulations (all test article concentrations) and vehicle control from the UDS Experiments were retained for analysis of achieved concentration and homogeneity (refer to Appendix 7). Samples were taken from the same bottle as was used for dosing.

Samples were stored refrigerated at nominal 4°C prior to analysis by Covance Laboratories Ltd. The analytical methods used and results obtained are presented in Appendix 5.

TEST SYSTEM

Species, strain and supplier

A sufficient number of out-bred young adult male Han Wistar Crl:WI (Han) (satellites) and Sprague Dawley Crl:CD® (SD) (main study) rats were obtained from Charles River (UK) Ltd, Margate, UK (refer to Appendix 7).

Specification

Animal specification was as follows:

Table 4: Animal Specification

	UDS Experiments
Number of animals used in study	46M[a]
Weight range on first day of assay (g)	190-300
Approximate age on first day of dosing (weeks)	7-9

M Male
F Female
[a] Includes 6 satellite animals for bioanalysis

Environment

The animals were routinely kept in the following environment except for short periods of time where experimental procedures dictated otherwise. The animals were housed in a room air-conditioned to provide a minimum of 15 air changes/hour. The temperature and relative humidity ranges were 19 to 25°C and 38 to 70%, respectively (see minor deviations from protocol, Appendix 7). Fluorescent lighting was controlled automatically to give a cycle of 12 hours light (0600 to 1800) and 12 hours dark. The study room was used to house animals allocated to other studies.

The animals were housed in groups of up to four, in cages that conformed with the 'Code of practice for the housing and care of animals used in scientific procedures' (Home Office, London, 1989).

Environmental enrichment

In order to enrich both the environment and the welfare of the animals, they were provided with wooden Aspen chew blocks.

Diet, water and bedding

Throughout the study the animals had access *ad libitum* to SQC Rat and Mouse Maintenance Diet No 1, Expanded (Special Diets Services Ltd. Witham). Each batch of diet was analysed for specific constituents and contaminants.

Mains water was provided *ad libitum* via water bottles. The water is periodically analysed for specific contaminants.

Bedding was provided on a weekly basis to each cage by use of clean Aspen wood chips (Datesand Ltd, Manchester). The bedding was analysed for specific contaminants.

No contaminants were expected to be present in diet, water or bedding at levels that might interfere with achieving the objective of the study. Results of analyses performed on diet, water, bedding and environmental enrichment are held centrally at Covance Laboratories Ltd.

METHODS

Pre-experimental procedures

Acclimatisation and health procedures

All animals were given a clinical inspection for ill health on arrival. They were acclimatised for at least 8 days (UDS Experiments) and a veterinary inspection was performed before the start of dosing to ensure their suitability for study.

Allocation to treatment group

Satellite animals were allocated to groups of three animals but were not randomised.

UDS animals were randomised to groups of four animals using a system of random numbers.

Checks were made prior to dosing on the first day of treatment to ensure individual group weights differed from the mean group weight by no more than 5%.

The allocation of animals to groups is detailed in Appendix 1.

Identification of the test system

The animals were individually identified by uniquely numbered ear-tag. Cages were appropriately identified (using a colour-coded procedure) with study information including study number, study type, start date, number and sex of animals, together with a description of the dose level and proposed time of necropsy.

In-life experimental procedures

UDS Experiments

No gender differences in toxicity have been previously identified. Animals were dosed in groups as described in the section entitled "dose levels".

Individual body weights and dosages administered are shown in Appendix 1.

Bioanalysis

Groups of male satellite animals were dosed with vehicle or Fexinidazole (highest dose). Animals were dosed by the same route, dose level and at the same dosing frequency as that described for the UDS animals. Plasma was isolated from these animals and used to assess systemic exposure to the test article.

Animals were dosed and bled as follows:

Table 5: Bioanalysis

Group	Number of animals	Dose (mg/kg)	Sample time (hours after final administration)
			1
Vehicle	3M	0	√
Fexinidazole	3M	2000	√

M Male

Approximately 1 mL of whole blood was taken via cardiac puncture under terminal isoflourane anaesthesia approximately 1 hour after final administration.

Blood was collected at room temperature into tubes containing lithium heparin and mixed thoroughly before placing on ice until centrifugation (2300 *g*, 4°C, 10 minutes). Plasma was separated into appropriately labelled polypropylene tubes and stored frozen at –20°C nominal until dispatch on dry ice to the Test Site.

The analytical methods used and results obtained are presented in Appendix 6.

Experimental observations

Routine health status check

All animals were examined at the beginning and the end of the working day to ensure that they were in good health and displayed no signs of overt toxicity.

Post dosing observations

All animals were observed daily for signs of ill health or overt toxicity. An individual record was maintained of the clinical condition of all UDS animals dosed in the study.

In the UDS Experiments post-dosing observations were performed immediately after dose administration, at least once in the 2 hours following dose administration. Observations were also recorded at least once on each day of liver perfusion.

Satellite animals were for blood sampling purposes only, no specific clinical observations were recorded for these animals.

Body weights

Individual body weights were recorded on each day of liver perfusion (UDS animals). The body weights of satellite animals were recorded on each day of dosing (data not reported).

Liver perfusion and preparation of hepatocyte cultures

Test article, vehicle and positive control treated rats were taken in groups of four, either 2 to 4 or 12 to 14 hours after dosing as appropriate. Hepatocytes were required from only three animals per group; initially hepatocytes were isolated from the first three animals numerically, according to the randomised ear-tag number. The remaining animal in each group was held in reserve and perfused in the event of technical problems or low hepatocyte culture viability in another animal. Any animal not required for perfusion was humanely destroyed.

Individual animals were anaesthetised with isofluorane and maintained under deep anaesthesia to prevent any likelihood of recovery. The liver was surgically exposed, the hepatic portal vein and superior *vena cava* cannulated with suitable cannulars and the liver perfused with suitable buffers. Approximately 400 mL of calcium free Buffer 1 (150 mM NaCl, 3.76 mM $NaHCO_3$, 4.84 mM Na_2HPO_4, 4.97 mM KCl, 1.24 mM KH_2PO_4, 0.62 mM $MgSO_4$, 0.62 mM $MgCl_2$, 10 μg/mL Phenol red indicator) was pumped at a flow rate of approximately 40 mL/min to wash the liver free of blood. The liver was then perfused with Buffer 2 (142 mM NaCl, 24 mM $NaHCO_3$, 4.37 mM KCl, 1.24 mM KH_2PO_4, 0.62 mM $MgSO_4$, 0.62 mM $MgCl_2$, 10 μg/mL Phenol red indicator) also at a flow rate of 40 mL/min for approximately 5 minutes. Both buffers were gassed with 5% CO_2 in air (v/v) prior to use and Buffer 2 throughout perfusion.

Calcium and collagenase (approximately 50 mg of collagenase dissolved in 1 mL of 769 mM $CaCl_2$ and 10 mL of Buffer 2) was added to the reservoir and after one to two minutes the waste line was placed in the Buffer 2 reservoir so that the perfusate recirculates and the flow rate was reduced to 20 mL/min. When the reticular pattern

of the liver had begun to break up and the liver became 'spongy', the perfusion was stopped.

The liver was cut free into a suitable container with Buffer 2. The liver was transferred to a sterile dish, cut open and the hepatocytes carefully teased out. The resulting hepatocyte suspension was gently filtered through 150 µm nylon mesh with Williams E medium-Complete (WE-C) to a volume of approximately 100 mL. Of this suspension, approximately 50 mL was taken and centrifuged at approximately 40 x 'g' for two to three minutes. The resultant pellet was resuspended in WE-C. The centrifugation and resuspension procedure was repeated at least twice and the pellet resuspended finally in approximately 20 mL WE-C. A sample (0.5 mL) of this suspension was taken, diluted with an equal volume of 0.4% (w/v) trypan blue in phosphate buffered saline (PBS) and the proportion of viable cells (those with unstained nuclei) determined using an haemocytometer.

At least three hepatocyte cultures per treatment group were selected (based on the viability assessments described above) for UDS assessment. These cell suspensions were individually diluted in WE-C to provide approximately 1.5×10^5 viable cells/mL.

Cell attachment

Three mL of hepatocyte suspension was added to each well of a six-well multiplate containing 25 mm round plastic coverslips and incubated at 37°C ± 1°C in a 5% CO_2 in air (v/v) atmosphere for at least 90 minutes to allow cells to attach.

Radiolabelling of hepatocyte cultures

Medium was removed from the cells and the monolayers washed with 2 mL Williams E medium-Incomplete (WE-I) which was then replaced with 2 mL WE-I containing 10 µCi/mL [^{3}H] thymidine. After approximately 4 hours incubation at 37°C ± 1°C in a 5% CO_2 in air (v/v) atmosphere, the medium was removed and the cells washed with three changes of WE-I containing 0.25 mM thymidine. Cultures were then incubated overnight with 3 mL of the same medium.

Cell fixation

To prepare for autoradiography, coverslips were washed with 2 mL phosphate buffered saline (PBS) and the cells fixed with three changes of 2 mL glacial acetic acid:ethanol (1:3 v/v). The coverslips were then washed a minimum of four times

with purified water, allowed to dry and mounted cell side up onto labelled microscope slides with DPX.

Autoradiography

Three of the six slides from each animal were coated in Kodak NTB liquid emulsion using a dipping technique. Each slide was dipped individually into the molten emulsion, ensuring that no air-bubbles were generated. The slides were incubated in a light-tight box at room temperature for approximately 4 hours to let the emulsion dry. The slides were then packed in light-tight boxes containing desiccant, sealed with tape and refrigerated for 5 days. At the end of this time, the emulsion was developed in Kodak D19 developer and fixed using Ilford Hypam fixer. The cell nuclei and cytoplasm were then stained with Meyers haemalum/eosin Y. Slides were then dehydrated in ethanol, cleared in xylene and mounted with coverslips for microscopic examination. The spare, duplicate sets of slides were not required.

Grain counting

Grain counting was performed using a microscope with a video camera connected to an image analysis system (Perceptive Instruments) and a computer programmed for automatic data capture.

Each slide was examined to ensure that the culture was viable. A patch of cells was selected as a starting point and cells were scored in a regular fashion by bringing new cells into the field of view, moving only in one axis. If the desired number of cells had not been scored before coming to the edge of the slide, the stage was moved one or two fields on the other axis and counting resumed. The circular field was centred over the nucleus of a suitable cell and the grains counted. The field of view was moved and counts obtained for three separate adjacent areas of cytoplasm. Nuclear and mean cytoplasmic grain counts were then recorded, and the net grains/nucleus (NNG) determined. 100 cells were analysed per animal (50 from each of two slides), using two of the three slides in each case.

The following criteria were used for analysis of slides:

1. Only cells with normal morphology were scored

2. Isolated nuclei with no surrounding cytoplasm were not scored

3. Cells without nuclear and/or cytoplasmic graining were not scored

4. Cells with unusual staining artefacts were not scored

5. Heavily labelled cells in S-phase were not scored

6. All other normal cells, 100 per animal were scored

7. All slides were analysed blind (coded).

All slides and raw data have been retained at Covance Laboratories Limited for archiving in accordance with the archive statement in this report.

Analysis of results

Treatment of data

The following were calculated for each slide, animal and dose point:

1. The population average NNG and standard deviation (SD)

2. The percent of cells responding or in repair (NNG ≥ 5)

3. The population average cytoplasmic and nuclear grain count.

Acceptance criteria

The assay was considered valid if all the following criteria were met:

1. Negative control animals had a group mean NNG value that did not exceed the upper limit of the historical range

2. The positive control treatments had group mean NNG values of not less than five NNG counts with 50% or more cells having NNG counts of five or greater.

Acceptance under any other criteria are discussed in the results section.

Evaluation criteria

For valid data, the test article was considered to induce UDS if:

1. The group mean NNG values were greater than 0 NNG and 20% or more of cells responded (mean NNG values ≥ 5)

2. A marked increase above vehicle control levels was seen in both NNG and the percentage of cells in repair.

The test article was considered as positive in this assay if all of the above criteria were met.

The test article was considered as negative in this assay if none of the above criteria were met.

Cytoplasmic and nuclear grain count values as well as the concurrent negative control data was considered in relation to the overall NNG values of cultures from treated animals.

Results which only partially satisfied the above criteria were dealt with on a case-by-case basis. Biological relevance was taken into account, for example consistency of response within and between dose levels.

RESULTS

Selection of doses for UDS Experiments

Based on the toxicity data provided from the Sponsor, 2000 mg/kg (highest recommended dose) was considered an appropriate maximum dose for this study (6,7).

As no gender differences in toxicity have been previously identified this study was conducted in male animals only (6).

UDS Experiments

Raw data

The group mean, individual animal and individual slide NNG data and the mean nuclear and cytoplasmic grain counts appear in Appendix 2.

Validity of study

The acceptance criteria for the UDS assay are described in the acceptance criteria section. The data in Appendix 2 confirm that:

1. The group mean net grain count for vehicle-treated animals was less than the upper limit of the historical control range (0.6 and 0.2 for 12-14 and 2-4 hour time points respectively) with ≤ 0.7% cells in repair

2. The positive control chemicals 2-AAF and DMN induced increases in group mean net grain count of five or more (9.8 and 8.9 respectively), and 50% or more of cells (89% and 77.4% respectively) in repair. This result showed that the test system was sensitive to two known DNA damaging agents requiring metabolism for their action and that the experiment was valid.

For the 12-14 hour experiment some slide replicates were unscorable and therefore 100 cells were scored from one slide. Fifty cells were scored and immediately following this another fifty cells were scored thus no cells were scored more than once.

The assay data were therefore considered valid.

Clinical signs

No clinical signs of toxicity were observed in any animal following treatments with vehicle, Fexinidazole (at 500.0, 1000 or 2000 mg/kg) or the positive controls (DMN or 2-AAF).

No notable effect of treatment on bodyweights was observed.

Analysis of data

Treatment with Fexinidazole at doses up to 2000 mg/kg yielded NNG values within the historical vehicle control range, producing group mean NNG values over the two time points in the range 0.5 to -0.4. No more than 1% cells were seen in repair at any dose of Fexinidazole.

The data obtained in this study indicate that treatment of male rats dosed once via oral gavage with 500.0, 1000 or 2000 mg/kg Fexinidazole did not result in increased UDS in hepatocytes isolated approximately 12-14 or 2-4 hours after dosing.

Formulations analysis

Results of formulation analyses for the 12-14 hour sample time demonstrated achieved concentrations within 100±10% of the nominal test article concentrations and were therefore considered acceptable (Appendix 5). Results of formulations analysis for the 2-4 hour sample time demonstrated achieved concentrations slightly higher than 100±10%. As the maximum test concentration was exceeded and an acceptable range of concentrations were tested, these data are considered to be acceptable.

The test article formulations were considered homogenous for the main study experiments. For the formulation prepared at 50 mg/mL in the 12-14 hour experiment the %CV was greater than 6, however, only two values were slightly outside the range 100±10% nominal, and the achieved concentration was acceptable. Therefore data are accepted as valid.

No test article was detected in the vehicle samples.

Results of formulations analysis for the animals used for bioanalysis were below 100±10% nominal, and %CV was greater than 6. However, systemic exposure of fexinidazole was confirmed at these concentrations and therefore data are considered acceptable.

Bioanalysis

The results of bioanalysis are presented in Appendix 6. These results confirm that animals dosed at 2000 mg/kg were systemically exposed to Fexinidazole and its sulphoxide and sulphone metabolites.

There was no test article contamination in vehicle samples.

CONCLUSION

When treated once via oral gavage with Fexinidazole at doses up to 2000 mg/kg (the maximum recommended dose based on current guidelines) male Sprague Dawley rats showed no induction of UDS in hepatocytes isolated *ex vivo* approximately 12-14 or 2-4 hours after dosing. It is concluded that Fexinidazole had no genotoxic activity detectable in this test system under the experimental conditions employed.

REFERENCES

1 Miller E C and Miller J A (1981) Searches for ultimate chemical carcinogens and their reactions with cellular macromolecules. Cancer, 47, 2327-2345

2 Garner R C (1979) Carcinogen prediction in the laboratory: a personal view, in "Long-term hazards from environmental chemicals. A Royal Society Discussion". pp 121-124. Published by The Royal Society, London

3 Mirsalis J and Butterworth B E (1980) Detection of unscheduled DNA synthesis in hepatocytes isolated from rats treated with genotoxic agents: An *in vivo/in vitro* assay for potential carcinogens and mutagens. Carcinogenesis 1, 621-625

4 Ashby J, Lefevre PA, Burlinson B and Penman MG (1985) An assessment of the *in vivo* rat hepatocyte DNA repair assay. Mutation Research 156 1-18

5 Kennelly J C, Waters R, Ashby J, Lefevre P A, Burlinson B, Benford D J, Dean S W and Mitchell I de G (1993) *In vivo* rat liver UDS assay. Supplementary Mutagenicity Tests UKEMS Recommended Procedures. Eds D J Kirkland and M Fox, Cambridge University Press, pp 52-77

6 OECD Test Guideline 486 (1997) “Unscheduled DNA Synthesis (UDS) Test with Mammalian Liver Cells *In Vivo*”. In OECD Guidelines for the testing of chemicals, OECD Paris

7 European Agency for the Evaluation of Medicinal Products. (1995). ICH Topic S 2 A. “Genotoxicity: Guidance on Specific Aspects of Genotoxicity Tests for Pharmaceuticals”. ICH Harmonised Tripartite Guideline.

APPENDICES

Appendix 1
Body weights and dosages

Table 6: Fexinidazole: Body weights and dosages: UDS Experiment, 12-14 hour sample time

Treatment (mg/kg)	Group	Animal number	Body weight (g)†	Dose given (mL)	Body weight (g)††	Cell viab. %
Vehicle control	1	2	266	2.7	270	65
		7	248	2.5	251	67
		19	276	2.8	285	77
		20	277	2.8	288	-
	Mean weight (g) ± SD		267 ± 13.5			
500.0	2	4	260	2.6	267	69
		8	262	2.6	272	55
		11	282	2.8	288	69
		15	300	3.0	302	-
	Mean weight (g) ± SD		276 ± 18.8			
1000	3	6	284	2.8	289	74
		9	247	2.5	253	81
		10	265	2.7	266	77
		16	279	2.8	280	-
	Mean weight (g) ± SD		269 ± 16.6			
2000	4	3	263	2.6	267	70
		12	272	2.7	273	77
		13	260	2.6	265	b
		14	289	2.9	297	64
	Mean weight (g) ± SD		271 ± 13.0			
Positive control, 2-AAF, 75	5	1	296	3.0	299	54
		5	254	2.5	260	58
		17	247	2.5	246	55
		18	264	2.6	264	-
	Mean weight (g) ± SD		265 ± 21.7			

Dose volume = 10 mL/kg
† Animals weighed within 24 hours prior to dosing
†† Animals weighed prior to sacrifice
Cell viab. % = percentage of viable cells as determined by trypan blue exclusion
b = Low cell viability, culture not processed
- = Animal not required

Table 7: Fexinidazole: Body weights and dosages: UDS Experiment, 2-4 hour sample time

Treatment (mg/kg)	Group	Animal number	Body weight (g)†	Dose given (mL)	Cell viab. %
Vehicle control	6	21	218	2.2	82
		22	239	2.4	75
		24	219	2.2	69
		34	229	2.3	-
		Mean weight (g) ± SD	226 ± 9.8		
500.0	7	26	203	2.0	67
		28	222	2.2	56
		30	240	2.4	54
		35	211	2.1	-
		Mean weight (g) ± SD	219 ± 16.0		
1000	8	25	212	2.1	76
		27	206	2.1	70
		31	215	2.2	70
		37	240	2.4	-
		Mean weight (g) ± SD	218 ± 15.0		
2000	9	29	222	2.2	60
		33	190	1.9	73
		36	216	2.2	73
		40	224	2.2	-
		Mean weight (g) ± SD	213 ± 15.7		
Positive control, DMN, 10	10	23	215	2.2	61
		32	233	2.3	73
		38	219	2.2	82
		39	214	2.1	-
		Mean weight (g) ± SD	220 ± 8.8		

Dose volume = 10 mL/kg
† Animals weighed within 24 hours prior to dosing
Cell viab. % = percentage of viable cells as determined by trypan blue exclusion
- = Animal not required

Appendix 2
Tables of results

Table 8 Fexinidazole: Group mean net grain count values, 12-14 hour sample time

Dose (mg/kg)	Compound	Net grain count (NNG)		Percent of cells in repair (NNG ≥5)		Net grain count of cells in repair	
		mean	SD	mean	SD	mean	SD
0	Methocel	0.6	0.0	0.7	0.6	5.7	0.5
500	Fexinidazole	0.4	0.1	-	-	-	-
1000	Fexinidazole	0.5	0.3	1.0	0.0	6.8	2.0
2000	Fexinidazole	0.2	0.3	0.3	0.6	5.7	-
75	2-AAF	9.8	0.8	89.0	7.2	10.6	0.3

Table 9 Fexinidazole: Group mean net grain count values, 2-4 hour sample time

Dose (mg/kg)	Compound	Net grain count (NNG)		Percent of cells in repair (NNG ≥5)		Net grain count of cells in repair	
		mean	SD	mean	SD	mean	SD
0	Methocel	0.2	0.1	-	-	-	-
500	Fexinidazole	0.0	0.3	-	-	-	-
1000	Fexinidazole	-0.1	0.1	-	-	-	-
2000	Fexinidazole	-0.4	0.2	-	-	-	-
10	DMN	8.9	0.4	77.4	2.4	11.0	0.3

Table 10 Fexinidazole: Individual animal net grain count values, 12-14 hour sample time

Compound (mg/kg)	Animal number	Net grain count (NNG)		% cells in repair (NNG ≥5)	Net grain count of cells in repair		No. of cells scored
		mean	SD		mean	SD	
Methocel 0	2	0.62	0.08	1.00	5.33	-	100
	7	0.56	0.01	1.00	6.00	-	100
	19	0.56	0.19	0.00	-	-	100
Fexinidazole 500	4	0.49	0.22	0.00	-	-	100
	8	0.37	0.04	0.00	-	-	100
	11	0.28	0.30	0.00	-	-	100
Fexinidazole 1000	6	0.18	0.12	1.00	5.00	-	100
	9	0.52	0.18	1.00	9.00	-	100
	10	0.81	0.28	1.00	6.33	-	100
Fexinidazole 2000	3	0.37	0.36	0.00	-	-	100
	12	0.44	0.01	1.00	5.67	-	100
	14	-0.09	0.26	0.00	-	-	100
2-AAF 75	1	10.69	0.49	97.00	10.92	0.45	100
	5	9.26	1.07	83.00	10.63	0.36	100
	17	9.34	0.49	87.00	10.29	0.89	100

Table 11 Fexinidazole: Individual animal net grain count values, 2-4 hour sample time

Compound (mg/kg)	Animal number	Net grain count (NNG)		% cells in repair (NNG ≥5)	Net grain count of cells in repair		No. of cells scored
		mean	SD		mean	SD	
Methocel 0	21	0.12	0.17	0.00	-	-	100
	22	0.31	0.05	0.00	-	-	100
	24	0.07	0.08	0.00	-	-	100
Fexinidazole 500	26	0.38	0.16	0.00	-	-	100
	28	0.06	0.16	0.00	-	-	100
	30	-0.30	0.04	0.00	-	-	100
Fexinidazole 1000	25	-0.07	0.05	0.00	-	-	100
	27	0.02	0.06	0.00	-	-	100
	31	-0.12	0.16	0.00	-	-	100
Fexinidazole 2000	29	-0.59	0.36	0.00	-	-	100
	33	-0.21	0.35	0.00	-	-	100
	36	-0.29	0.09	0.00	-	-	100
DMN 10	23	8.50	0.27	75.27	10.60	0.25	101
	32	8.97	0.69	77.00	11.18	0.01	100
	38	9.21	1.44	80.00	11.10	0.60	100

Table 12 Fexinidazole: Individual slide net grain count values, 12-14 hour sample time

Compound (mg/kg)	Animal number	Slide number	Net grain count (NNG)		% cells in repair (NNG ≥5)	Net grain count of cells in repair		No. of cells scored
			mean	SD		mean	SD	
Methocel 0	2	2	0.6	1.1	2.0	5.3	-	50
		3	0.7	1.3	0.0	-	-	50
	7	2	0.6	1.3	2.0	6.0	-	50
		3	0.6	1.3	0.0	-	-	50
	19	1	0.4	1.0	0.0	-	-	50
		2	0.7	1.2	0.0	-	-	50
Fexinidazole 500	4	2	0.3	0.9	0.0	-	-	50
		3	0.6	1.3	0.0	-	-	50
	8#	2	0.3	1.1	0.0	-	-	50
		2	0.4	1.3	0.0	-	-	50
	11#	2	0.1	1.3	0.0	-	-	50
		2	0.5	1.0	0.0	-	-	50
Fexinidazole 1000	6	2	0.3	1.3	0.0	-	-	50
		3	0.1	1.6	2.0	5.0	-	50
	9#	2	0.7	1.0	0.0	-	-	50
		2	0.4	1.8	2.0	9.0	-	50
	10#	2	1.0	1.4	2.0	6.3	-	50
		2	0.6	1.2	0.0	-	-	50
Fexinidazole 2000	3	2	0.6	1.1	0.0	-	-	50
		3	0.1	1.1	0.0	-	-	50
	12#	2	0.4	1.5	0.0	-	-	50
		2	0.5	1.4	2.0	5.7	-	50
	14#	2	-0.3	1.2	0.0	-	-	50
		2	0.1	1.2	0.0	-	-	50
2-AAF 75	1	2	10.3	3.0	96.0	10.6	2.8	50
		3	11.0	4.0	98.0	11.2	3.8	50
	5	2	8.5	5.2	78.0	10.4	4.1	50
		3	10.0	5.0	88.0	10.9	4.7	50
	17	1	9.7	4.8	84.0	10.9	4.2	50
		2	9.0	3.9	90.0	9.7	3.4	50

Where no scoreable cells were available on slides 1 and 3, 100 cells were scored from slide 2

Table 13 Fexinidazole: Individual slide net grain count values, 2-4 hour sample time

Compound (mg/kg)	Animal number	Slide number	Net grain count (NNG)		% cells in repair (NNG ≥5)	Net grain count of cells in repair		No. of cells scored
			mean	SD		mean	SD	
Methocel 0	21	1	0.2	0.4	0.0	-	-	50
		3	0.0	1.1	0.0	-	-	50
	22	1	0.3	0.8	0.0	-	-	50
		2	0.3	0.9	0.0	-	-	50
	24	1	0.0	0.9	0.0	-	-	50
		3	0.1	0.8	0.0	-	-	50
Fexinidazole 500	26	1	0.3	0.8	0.0	-	-	50
		2	0.5	0.8	0.0	-	-	50
	28	1	0.2	0.9	0.0	-	-	50
		2	-0.0	0.7	0.0	-	-	50
	30	1	-0.3	1.1	0.0	-	-	50
		2	-0.3	1.2	0.0	-	-	50
Fexinidazole 1000	25	1	-0.0	0.9	0.0	-	-	50
		2	-0.1	0.8	0.0	-	-	50
	27	1	0.1	0.9	0.0	-	-	50
		2	-0.0	1.0	0.0	-	-	50
	31	1	0.0	1.0	0.0	-	-	50
		2	-0.2	1.5	0.0	-	-	50
Fexinidazole 2000	29	1	-0.3	1.2	0.0	-	-	50
		2	-0.8	1.1	0.0	-	-	50
	33	1	-0.5	1.3	0.0	-	-	50
		3	0.0	1.4	0.0	-	-	50
	36	1	-0.4	1.1	0.0	-	-	50
		2	-0.2	1.1	0.0	-	-	50
DMN 10	23	1	8.3	5.3	72.5	10.8	3.9	51
		2	8.7	4.7	78.0	10.4	3.6	50
	32	1	9.5	5.3	82.0	11.2	4.1	50
		2	8.5	5.6	72.0	11.2	3.9	50
	38	1	10.2	5.9	86.0	11.5	5.3	50
		2	8.2	5.5	74.0	10.7	3.9	50

Table 14 Fexinidazole: Cytoplasmic and nuclear grain count values, 12-14 hour sample time

Compound (mg/kg)	Animal number	Slide number	Nuclear grain count (N)		Cytoplasmic grain count (C)		Net nuclear grain count (N-C)		No. of cells scored
			mean	SD	mean	SD	mean	SD	
Methocel 0	2	2	1.84	1.11	1.27	0.67	0.57	1.15	50
		3	2.10	1.22	1.43	0.65	0.67	1.27	50
	Total		1.97	0.18	1.35	0.11	0.62	0.08	100
	7	2	2.00	1.23	1.43	0.55	0.57	1.34	50
		3	2.10	1.07	1.55	0.62	0.55	1.28	50
	Total		2.05	0.07	1.49	0.08	0.56	0.01	100
	19	1	1.78	0.86	1.35	0.53	0.43	1.01	50
		2	2.32	1.11	1.62	0.63	0.70	1.23	50
	Total		2.05	0.38	1.49	0.19	0.56	0.19	100
Total			2.02	0.05	1.44	0.08	0.58	0.03	300
Fexinidazole 500	4	2	1.72	0.86	1.39	0.51	0.33	0.90	50
		3	2.22	1.25	1.58	0.76	0.64	1.29	50
	Total		1.97	0.35	1.48	0.14	0.49	0.22	100
	8	1	1.78	0.95	1.44	0.65	0.34	1.11	50
		2	1.96	1.05	1.57	0.79	0.39	1.27	50
	Total		1.87	0.13	1.50	0.09	0.37	0.04	100
	11	1	2.06	1.19	1.99	0.64	0.07	1.29	50
		2	2.16	0.96	1.67	0.54	0.49	1.00	50
	Total		2.11	0.07	1.83	0.23	0.28	0.30	100
Total			1.98	0.12	1.61	0.20	0.38	0.11	300
Fexinidazole 1000	6	2	1.88	1.10	1.61	0.66	0.27	1.29	50
		3	2.14	1.31	2.05	0.85	0.09	1.56	50
	Total		2.01	0.18	1.83	0.31	0.18	0.12	100
	9	1	1.90	0.91	1.25	0.44	0.65	1.03	50
		2	1.98	1.67	1.59	0.80	0.39	1.77	50
	Total		1.94	0.06	1.42	0.24	0.52	0.18	100
	10	1	2.52	1.36	1.51	0.59	1.01	1.41	50
		2	1.88	0.94	1.27	0.60	0.61	1.16	50
	Total		2.20	0.45	1.39	0.17	0.81	0.28	100
Total			2.05	0.13	1.55	0.25	0.50	0.32	300
Fexinidazole 2000	3	2	1.92	0.88	1.30	0.67	0.62	1.09	50
		3	1.76	0.96	1.65	0.58	0.11	1.06	50
	Total		1.84	0.11	1.47	0.25	0.37	0.36	100
	12	1	2.30	1.16	1.87	0.81	0.43	1.54	50
		2	1.94	1.19	1.49	0.64	0.45	1.35	50
	Total		2.12	0.25	1.68	0.27	0.44	0.01	100
	14	1	2.38	1.28	2.65	0.92	-0.27	1.22	50
		2	2.50	1.18	2.41	0.96	0.09	1.24	50
	Total		2.44	0.08	2.53	0.17	-0.09	0.26	100
Total			2.13	0.30	1.89	0.56	0.24	0.29	300

Table continued overleaf

Compound (mg/kg)	Animal number	Slide number	Nuclear grain count (N)		Cytoplasmic grain count (C)		Net nuclear grain count (N-C)		No. of cells scored
			mean	SD	mean	SD	mean	SD	
2-AAF 75	1	2	12.72	3.14	2.38	0.93	10.34	3.03	50
		3	13.70	4.49	2.67	1.23	11.03	4.03	50
	Total		13.21	0.69	2.52	0.20	10.69	0.49	100
	5	2	11.58	5.10	3.07	1.09	8.51	5.20	50
		3	13.36	5.01	3.35	1.07	10.01	4.99	50
	Total		12.47	1.26	3.21	0.19	9.26	1.07	100
	17	1	11.64	4.72	1.95	0.89	9.69	4.77	50
		2	11.22	3.74	2.23	0.87	8.99	3.86	50
	Total		11.43	0.30	2.09	0.20	9.34	0.49	100
Total			12.37	0.89	2.61	0.56	9.76	0.80	300

Table 15 Fexinidazole: Cytoplasmic and nuclear grain count values, 2-4 hour sample time

Compound (mg/kg)	Animal number	Slide number	Nuclear grain count (N)		Cytoplasmic grain count (C)		Net nuclear grain count (N-C)		No. of cells scored
			mean	SD	mean	SD	mean	SD	
Methocel 0	21	1	1.10	0.30	0.86	0.35	0.24	0.36	50
		3	2.28	1.26	2.28	1.18	0.00	1.12	50
	Total		1.69	0.83	1.57	1.00	0.12	0.17	100
	22	1	1.36	0.69	1.02	0.44	0.34	0.81	50
		2	1.38	0.73	1.11	0.62	0.27	0.88	50
	Total		1.37	0.01	1.06	0.06	0.31	0.05	100
	24	1	1.36	0.60	1.35	0.65	0.01	0.86	50
		3	1.52	0.71	1.40	0.53	0.12	0.81	50
	Total		1.44	0.11	1.37	0.04	0.07	0.08	100
Total			1.50	0.17	1.34	0.26	0.16	0.13	300
Fexinidazole 500	26	1	1.42	0.73	1.15	0.55	0.27	0.80	50
		2	1.44	0.64	0.95	0.52	0.49	0.79	50
	Total		1.43	0.01	1.05	0.14	0.38	0.16	100
	28	1	1.44	0.58	1.27	0.66	0.17	0.88	50
		2	1.38	0.70	1.43	0.69	-0.05	0.72	50
	Total		1.41	0.04	1.35	0.11	0.06	0.16	100
	30	1	1.74	1.07	2.07	1.20	-0.33	1.12	50
		2	1.90	0.97	2.17	1.02	-0.27	1.24	50
	Total		1.82	0.11	2.12	0.08	-0.30	0.04	100
Total			1.55	0.23	1.50	0.55	0.05	0.34	300
Fexinidazole 1000	25	1	1.52	0.89	1.55	0.80	-0.03	0.88	50
		2	1.28	0.54	1.39	0.60	-0.11	0.84	50
	Total		1.40	0.17	1.47	0.12	-0.07	0.05	100
	27	1	1.52	0.65	1.45	0.81	0.07	0.87	50
		2	1.66	0.85	1.68	0.79	-0.02	0.96	50
	Total		1.59	0.10	1.57	0.16	0.02	0.06	100
	31	1	1.52	0.89	1.52	0.72	0.00	1.01	50
		2	2.42	1.50	2.65	1.25	-0.23	1.51	50
	Total		1.97	0.64	2.09	0.80	-0.12	0.16	100
Total			1.65	0.29	1.71	0.33	-0.05	0.07	300
Fexinidazole 2000	29	1	1.80	1.09	2.14	0.98	-0.34	1.19	50
		2	1.74	0.72	2.59	0.98	-0.85	1.13	50
	Total		1.77	0.04	2.36	0.32	-0.59	0.36	100
	33	1	2.28	1.47	2.74	1.52	-0.46	1.30	50
		3	2.36	1.27	2.32	0.95	0.04	1.36	50
	Total		2.32	0.06	2.53	0.30	-0.21	0.35	100
	36	1	1.48	0.86	1.83	0.87	-0.35	1.09	50
		2	1.98	1.25	2.21	1.08	-0.23	1.13	50
	Total		1.73	0.35	2.02	0.26	-0.29	0.09	100
Total			1.94	0.33	2.30	0.26	-0.36	0.20	300

Table continued overleaf

Compound (mg/kg)	Animal number	Slide number	Nuclear grain count (N)		Cytoplasmic grain count (C)		Net nuclear grain count (N-C)		No. of cells scored
			mean	SD	mean	SD	mean	SD	
DMN 10	23	1	9.39	5.42	1.08	0.64	8.31	5.32	51
		2	10.10	4.75	1.41	0.63	8.69	4.66	50
	Total		9.75	0.50	1.25	0.23	8.50	0.27	101
	32	1	10.28	5.16	0.82	0.47	9.46	5.32	50
		2	10.74	5.36	2.25	1.32	8.49	5.58	50
	Total		10.51	0.33	1.54	1.01	8.97	0.69	100
	38	1	11.98	5.86	1.75	0.98	10.23	5.95	50
		2	9.78	5.14	1.59	0.92	8.19	5.46	50
	Total		10.88	1.56	1.67	0.12	9.21	1.44	100
Total			10.38	0.58	1.48	0.22	8.89	0.36	301

Appendix 3
Historical vehicle control data ranges for: UDS Assay

Table 16: Historical vehicle control range: Male Sprague Dawley rats

Control Type		Net grain count (NNG)	% cells in repair (NNG ≥ 5)
Negative / vehicle	Number of studies	13	13
	Number of animals	93	93
	Median	-0.56	0.0
	Mean	-0.59	0.5
	SD	0.95	1.3
	**Observed range**	**-4.99 to 1.05**	**0.0 to 9.0**
	**95% reference range**	**-2.36 to 0.86**	**0.0 to 4.0**
DMN	Number of studies	8	8
	Number of animals	23	23
	Median	14.60	99.0
	Mean	16.95	93.2
	SD	6.52	12.4
	**Observed range**	**7.26 to 30.04**	**57.0 to 100.0**
	**95% reference range**	**7.26 to 30.04**	**57.0 to 100.0**
2-AAF	Number of studies	13	13
	Number of animals	42	42
	Median	11.01	96.0
	Mean	12.33	91.7
	SD	5.82	11.7
	**Observed range**	**5.33 to 31.26**	**51.0 to 100.0**
	**95% reference range**	**6.57 to 31.06**	**67.0 to 100.0**

Reference ranges are calculated from percentiles of the observed distributions.
Calculated in October 2006 by CLEH Statistics, from audited report data of studies started between February 2003 and December 2005

Appendix 4
Certificate of analysis

XCENTIPHARM
Active for your synthesis

ANALYSIS CERTIFICATE

FEXINIDAZOLE

Manufact. date November-2007
Expiry date October-2008

DATE	January 15, 2008
ANALYSIS N° CA	07327/01
BATCH CENTIPHARM	3168-07-01/O
SAMPLE N°	ECH:08015/51
WEIGHT (g)	70

DETERMINATIONS	RESULTS	SPECIFICATIONS
IDENTIFICATION	IR Spectrum complies	IR Spectrum complies
APPEARANCE	powder	powder
COLOUR	yellow	yellow
LOSS ON DRYING (%)	0,0	<= 0,5
SULPHATED ASH (%)	0,0	<= 0,1
$HClO_4$ ASSAY (%)	100,2	98,5 à 101,5
RELATED SUBSTANCES - HPLC- Any known impurity (%)	< 0,05	<= 0,15
RELATED SUBSTANCES - HPLC- Any other impurity (%)	0,08	<= 0,10
RELATED SUBSTANCES - HPLC - All impurities sum (%)	0,1	<= 0,5
RESIDUAL SOLVENTS -GC- Acetone (ppm)	740	<= 5000
RESIDUAL SOLVENTS -GC- Methanol (ppm)	20	<= 3000
RESIDUAL SOLVENTS -GC- Toluene (ppm)	4	<= 890

CONFORMITY	YES / NO

Grasse, 16/01/2008
M.CONNAN
Quality Control Manager

AXYNTIS
CENTIPHARM
Chemin de la Madeleine - B.P. 45249 - F-06131 GRASSE CEDEX - Tél. : 33 (0)4 93 70 01 32 - Fax : 33 (0)4 93 70 05 65 - http://www.axyntis.com - contact@axyntis.com
S.A.S. au capital de 1 525 000 € - R.C.S. GRASSE 326 171 378 - APE 241 G - TVA FR 68 326 171 378

Appendix 5
Formulations Analysis

SUMMARY

Test article Fexinidazole, was formulated in 0.5% methyl cellulose and 5% tween by Central Dispensary for dosing during the study.

Formulations received 6, 13 and 14 February 2008 were analysed to determine homogeneity and achieved concentration.

Test article was not detected in the control samples.

The analytical procedure was validated in the study 2647/23.

PROCEDURES

Homogeneity and Achieved Concentration

Samples were removed in duplicate from the top, middle and bottom of each formulation, except for the control samples where a single analysis was performed. These were analysed for test article concentration to determine homogeneity and achieved concentration.

Analytical procedure

The analytical procedure Covance 2647/023-01F was used to determine achieved concentration.

Data collection and processing

The data was collected and processed using an Empower2, version Build number 2154, data capture system.

RESULTS

Homogeneity and Achieved Concentration

2-4 hour sample time (6 February 2008)

Conc. mg/mL	Results as % nominal concentration Top		Middle		Bottom		Mean (%)	CV (%)
50	113	113	111	116	106	112	112	2.96
100	115	114	115	115	114	116	115	0.66
200	102	114	111	113	112	112	111	3.95

12-14 hour sample time (13 February 2008)

Conc. mg/mL	Results as % nominal concentration Top		Middle		Bottom		Mean (%)	CV (%)
50	97	112	87	108	102	100	101	8.67
100	110	111	101	102	101	103	105	4.39
200	104	105	104	106	103	103	104	1.12

Bioanalysis (14 February 2008)

Conc. mg/mL	Results as % nominal concentration Top		Middle		Bottom		Mean (%)	CV (%)
200	76	78	82	79	113	93	87	16.31

Formulations were to be considered homogeneous if the coefficient of variation (CV) of the results is ≤ 6.0%. In addition all the homogeneity results should be within ± 10% of the mean. The target range for preparation of liquid formulations is 90 to 110% of nominal.

Test Article was not found in control samples.

Appendix 7
Minor deviations from protocol

Protocol section	Subject	Deviation
Test system	Humidity of holding rooms	During the main Experiment the humidity in the holding room fell to 38% on one occasion. This lasted for less than 24 hours and is considered to have had no adverse impact on the integrity of the study.
Materials	Animals	The protocol states that out-bred Han Wistar rats were to be used in the UDS experiment, however Sprague Dawley rats were used in error. Both strains are equally acceptable for use in genetic toxicology<br>Toxicokinetic data confirmed systemic exposure of Fexinidazole and its metabolites in Sprague Dawley rats and therefore this is considered to have had no adverse impact on the integrity of the study.
Materials	Formulations analysis	The protocol states that stability of the test article will be assessed. This is incorrect, no analysis of stability was required.
Methods	Autoradiography	The protocol states that K2 liquid emulsion will be used and slides refrigerated for 14 days, which is incorrect.<br>Kodak NTB liquid emulsion was used and slides were refrigerated for 5 days, as following in house trials this emulsion was found to give better quality slides for scoring. This did not impact on study validity.
