# Supplementary material for: Fexinidazole – A New Oral Nitroimidazole Drug Candidate Entering Clinical Development for the Treatment of Sleeping Sickness
Source: PLoS Negl Trop Dis. 2010 Dec 21;4(12):e923. doi: 10.1371/journal.pntd.0000923 (PMC3006138; doi:10.1371/journal.pntd.0000923)
Supplement: Text S1 — Non-clinical studies list (0.03 MB DOC) [file pntd.0000923.s023.doc]

Box:

Nonclinical studies carried out with fexinidazole to meet the regulatory requirements for testing in humans.

| Nonclinical Studies | Guidance documents | GLP required |
| --- | --- | --- |
| Efficacy in *in vitro* models (drug potency with respect to trypanosome killing, mechanism of action etc.) | ICH M3(R2) Guideline: Guidance on Nonclinical Safety studies for the Conduct of Human Clinical Trials and marketing authorisation for Pharmaceuticals; June 2009. Section 2 | No |
| Efficacy in animal models (drug potency via different routes of administration in acute and chronic mouse models of HAT) | ICH M3(R2) Guideline: Section 2 | No |
| *In vitro* ligand binding and enzyme interactions to assess potential adverse effects | ICH S7A Guideline: Safety Pharmacology Studies for Human Pharmaceuticals. Section 2.2 General Considerations | No |
| Key Safety pharmacology in vivo: Cardiac, respiratory and CNS models | ICH S7A Guideline: Section 2.7: Safety Pharmacology Core battery | Yes |
| Additional cardiac safety relating to QT interval if warranted (*in vitro* and *in vivo*) | ICH S7B Guideline: The nonclinical Evaluation of the Potential for delayed Ventricular Repolarization (QT Interval prolongation) by Human Pharmaceuticals | Yes |
| Nonclinical assessment of ADME and metabolite profiles | ICH M3(R2) Guideline: Section 3 | No |
| Toxicokinetic studies in two species (one non-rodent) to reflect the expected duration of treatment in man | ICH M3(R2) Guideline: Sections 3-5  ICH S3A Guideline: Toxicokinetics: A Guidance for Assessing Systemic Exposure in Toxicology Studies. Section 4 | Yes |
| *In vitro* and *in vivo* genetic toxicology as related to the genotoxic potential of the drug and/or drug class | ICH M3(R2) Guideline: Section 9  ICH S3A Guideline: Section 4.4  ICH S2B Guideline: Genotoxicity: A standard battery for genotoxicity testing of Pharmaceuticals | Yes |
| Reproductive toxicity studies (ongoing for fexinidazole; did not need to be completed before phaseI) | ICH M3(R2) Guideline: Section 11  ICH S3A Guideline: Section 4.6  ICH S5(R2) Guideline | Yes |
